# Radical chain monoalkylation of pyridines†

**DOI:** 10.1039/d1sc02748d

**Published:** 2021-11-03

**Authors:** Samuel Rieder, Camilo Meléndez, Fabrice Dénès, Harish Jangra, Kleni Mulliri, Hendrik Zipse, Philippe Renaud

**Affiliations:** Department of Chemistry, Biochemistry and Pharmaceutical Sciences, University of Bern Freiestrasse 3 CH-3012 Bern Switzerland philippe.renaud@unibe.ch; Department of Chemistry, LMU München Butenandtstrasse 5-13 81377 München Germany zipse@cup.uni-muenchen.de

## Abstract

The monoalkylation of *N*-methoxypyridinium salts with alkyl radicals generated from alkenes (*via* hydroboration with catecholborane), alkyl iodides (*via* iodine atom transfer) and xanthates is reported. The reaction proceeds under neutral conditions since no acid is needed to activate the heterocycle and no external oxidant is required. A rate constant for the addition of a primary radical to *N*-methoxylepidinium >10^7^ M^−1^ s^−1^ was experimentally determined. This rate constant is more than one order of magnitude larger than the one measured for the addition of primary alkyl radicals to protonated lepidine demonstrating the remarkable reactivity of methoxypyridinium salts towards radicals. The reaction has been used for the preparation of unique pyridinylated terpenoids and was extended to a three-component carbopyridinylation of electron-rich alkenes including enol esters, enol ethers and enamides.

## Abstract 2

*N*-Methoxypyridinium salts are exceptionally reactive radical traps that can be used in efficient radical chain reactions with organoboranes.

## Graphical abstract

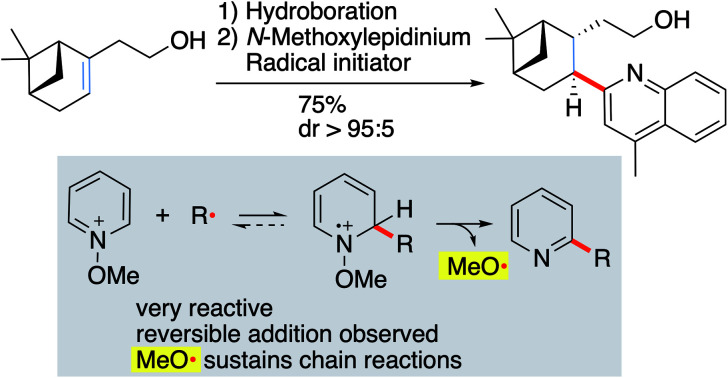

## Introduction

**Scheme 1:**
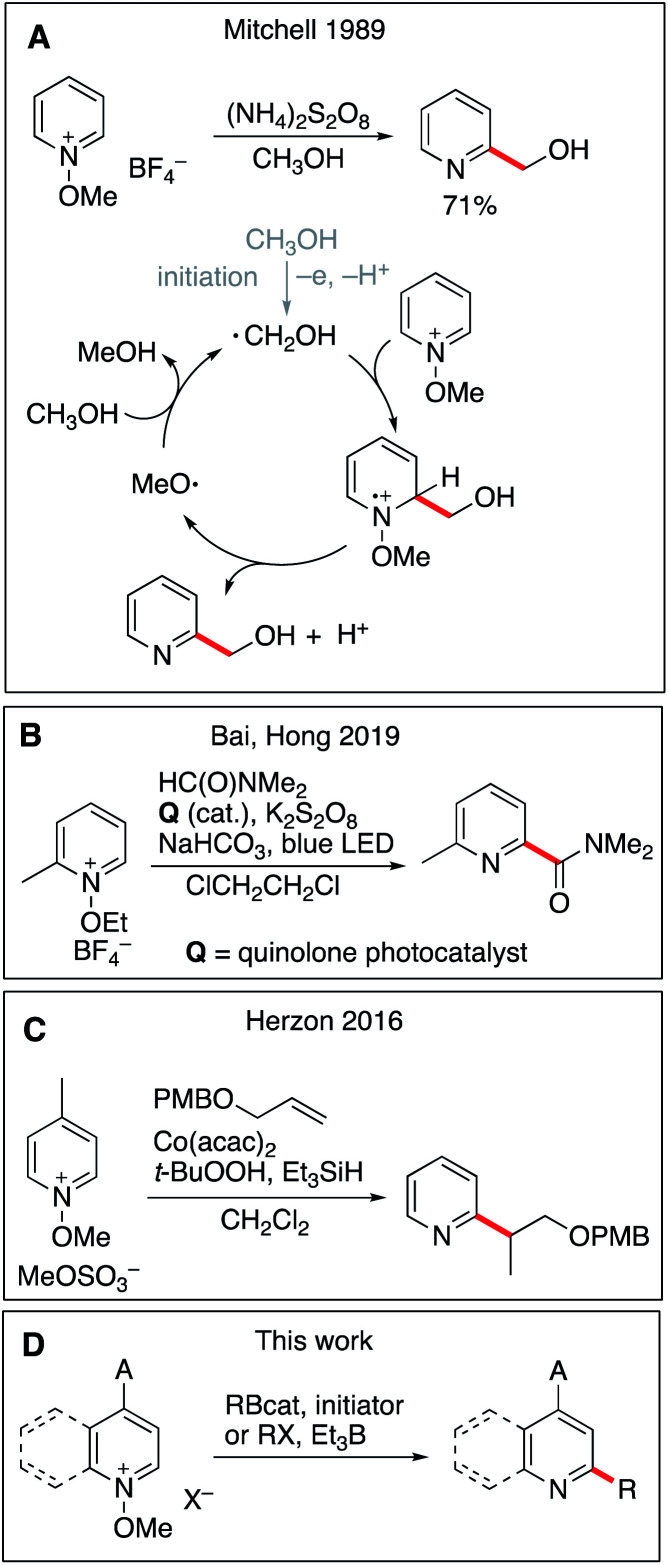
Radical addition to *N*-alkoxypyridinium salts. Previous leading work in the field (A–C) and this work (D).

Aromatic heterocyclic compounds, especially nitrogen-containing rings, are core elements of vitamins, amino acids, nucleic acids, and alkaloids and have thus attracted the attention of the synthetic community and the pharmaceutical industry for many years.^1^ A recent study has shown that 59% of U.S. FDA approved small-molecule drugs contain nitrogen heterocycles.^2^ Their late-stage functionalization is of great interest to modify and tune their pharmaceutical properties. For instance, introduction of carbon substituents under mild conditions has great potential and remains a privileged goal.^3^ Homolytic aromatic substitution (S_H_Ar) is a long-known reaction and early examples date back to over a century ago.^4,5^ The Minisci reaction, which involves the addition of a nucleophilic carbon-centered alkyl or acyl radical onto a protonated heteroaromatic compound, is of particular importance due to its broad scope.^6,7^ The classical method uses alkyl and acyl radicals generated by hydrogen atom abstraction with persulfate and a silver(i) salt.^6,8^ Over the years, many variations of this transformation have been reported with different sources of alkyl radicals such as alkyl trifluoroborates,^9,10^ boronic acids,^11,12^ alcohols,^13^ zinc sulfinates,^14,15^*N*-(acyloxy)-phthalimides,^16^ and simple alkanes and ethers^17–20^ to name some of the leading work. The efficacy of the Minisci reaction is highlighted in a recent review of Proctor and Phipps.^21^ All the above-mentioned examples involve a rearomatization process *via* single-electron oxidation using either a stoichiometric oxidant or photoredox catalysis. Non-acidic activation of pyridine derivatives has also been reported. Recently, Baran and co-workers have reported that easily prepared *N*-alkylpyridinium salts can be used to direct regioselective functionalization at C4.^22^ The use of pyridine-*N*-oxides,^23,24^*N*-iminopyridine ylides,^18^ and *N*-methoxypyridinium salts^25–29^ has been examined. These substrates are particularly interesting since the aromatization step does not require any external oxidant and the reaction affords simple pyridines that are less prone to further alkylation. In pioneer work, Mitchell and co-workers have developed a Minisci-type procedure for the hydroxymethylation of pyridines involving *N*-methoxypyridinium derivatives (Scheme 1A).^25^ Three different mechanisms were proposed for the rearomatization step, among them the one involving the fragmentation of a methoxyl radical that sustains a chain process *via* hydrogen atom transfer from methanol being the most plausible. The role of the oxidant (ammonium persulfate) used in a substoichiometric amount is to initiate the reaction. The scope of this reaction was limited to methanol and ethanol (one example) used as the solvent, but this study nicely demonstrated the usefulness of methoxypyridinium salts to perform monoalkylation of pyridine derivatives. This work was extended by Baik, Hong and co-workers who developed a site selective photocatalyzed functionalization of *N*-methoxypyridinium salts with phosphinoyl and carbamoyl radicals (Scheme 1B).^30,31^ Recently, related processes involving radical generation *via* hydrogen atom transfer were reported by Shen *et al.*,^32^ Lakhdar and co-workers^33^ and by Alfonzo and Hande.^34^ These examples extended the scope of Mitchell's strategy, and the desired alkyl radicals were generated by hydrogen atom transfer to the highly reactive alkoxyl radical released in the rearomatization process. Chemo- and regioselective control of this approach is very challenging and is usually limited to precursors presenting electron-rich weak C–H bonds positioned alpha to a heteroatom (*O*, *N*, *S*). The lack of selectivity between aliphatic C–H bonds presenting similar bond dissociation energies is usually circumvented by the choice of non-substituted cycloalkanes, used in excess to prevent polyalkylation. Herzon and co-workers extended considerably the scope of the Mitchell variant to the use of secondary and tertiary alkyl radicals generated from alkenes under cobalt-mediated hydrogen atom transfer conditions, and to a broad range of heterocycles such as pyridine, imidazole and pyridazine (Scheme 1C).^28,29^ This reaction requires a stoichiometric amount of cobalt and it was proposed that aromatization took place *via* reduction of the intermediate radical cation by a Co(ii) species followed by methanol elimination. Interestingly, the reaction could be extended to a borono-Minisci reaction under oxidative conditions (potassium persulfate and silver(i)). In a related manner, three-component coupling reactions involving *N*-methoxypyridinium salts were developed by Baik, Hong and co-workers^35^ using a Mn(iii)/Ag(i) oxidizing system and by Nagib and co-workers using an iridium based photocatalyst.^36,37^

Based on these precedents, we hypothesized that the methoxyl radical proposed in Mitchell's mechanism could be used to sustain a radical chain process involving organoboranes. We report here a general method for a radical mediated monoalkylation of pyridines and related heterocycles using easily available *B*-alkylcatecholboranes (RBcat), alkyl iodides, and xanthates under radical chain reaction conditions without the need for any stoichiometric oxidant (Scheme 1D).

## Results and discussion

### (a) Reaction with organoboranes generated *via* hydroboration of alkenes

#### Optimization

We initially examined the alkylation of *N*-methoxy-4-phenylpyridinium tetrafluoroborate (1a·BF_4_) with 2-cyclohexylbenzo-[*d*][1,3,2]dioxaborole (CyBcat) as a model system.^38^ Methoxypyridinium salt 1a·BF_4_ was prepared by oxidation of 4-phenylpyridine with *m*-CPBA followed by methylation with Meerwein's salt (Me_3_OBF_4_).^39^ CyBcat was prepared *in situ* by hydroboration of cyclohexene with catecholborane (catBH) and *N*,*N*-dimethylacetamide (DMA) as a catalyst.^40^ The reactions were performed with di-*tert*-butyl-hyponitrite (DTBHN)^41^ as a radical initiator. Optimization experiments are summarized in Table 1.

**Table 1:** Optimizing the conditions for the alkylation of *N*-methoxypyridinium 1a with CyBcat

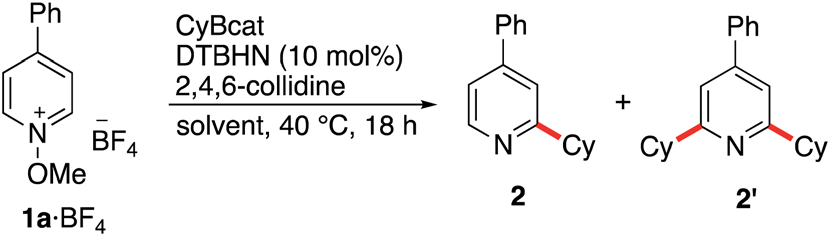					
Entry^a^	Solvent	CyBcat (equiv.)	2,4,6-Collidine (equiv.)	Yield^b^	
				2	2′
1	CH_2_Cl_2_	3.0	—	36%^c^	14%^c^
2	CH_2_Cl_2_	3.0	5	46%	—
3	CH_2_Cl_2_	1.5	—	55%	5%
4	CH_2_Cl_2_	1.5	3	54%	8%
5	EtOAc	1.5	—	45%^c^	17%
6	C_6_H_6_	1.5	—	10%	—
**7**	**DCE**	**1.5**	**—**	**63%**	**5%**
**8**	**DCE**	**1.5**	**3**	**63%** c	**—**
**9**	**DCE**	**1.5**	**3**	**63%** d	**—**
10	DCE	1.5	—	55%^e^	4%
11	DCE	1.5	—	28%^f^	2%

a Reagents and conditions (entries 3 and 5–7: 1a·BF_4_ (1 mmol), alkene (1.5 equiv.), catecholborane (3.0 equiv.), DMA (0.32 mol%), DTBHN (10 mol%), 40 °C, 18 h).

b Yields are determined by GC unless otherwise stated.

c Isolated yield.

d Using 1a·PF_6_.

e Air as an initiator instead of DTBHN.

f No added initiator.

In dichloromethane and in the absence of any additive, the desired mono-alkylated product 2 was obtained in 36% yield along with 14% of the disubstituted product 2′. The formation of the bisalkylated product was attributed to acidic activation of pyridine 2 by protons released during the rearomatization process (see the mechanism in Scheme 4). The bis-alkylation could be suppressed by adding 2,4,6-trimethylpyridine (*sym*-collidine, 5 equiv.) as a base (Table 1, entry 2). Slightly higher yields were obtained by using a smaller excess (1.5 equivalents) of CyBcat with or without *sym*-collidine (Table 1, entries 3 and 4). Different solvents were tested next. Ethyl acetate (EtOAc) and benzene proved to be less efficient than dichloromethane (Table 1, entries 5 and 6) due to limited solubility of the methoxypyridinium 1a·BF_4_ in these solvents. The use of 1,2-dichloroethane (DCE) allows 1a·BF_4_ to be fully solubilized at 40 °C and the reaction afforded 2 in 63% yield, together with 5% of the dialkylated pyridine 2′ (Table 1, entry 7). When the reaction was run in DCE with 2,4,6-collidine as an additive (3 equiv.), the reaction exclusively afforded 2 in 63% isolated yield (Table 1, entry 8). Changing the counter-anion from tetrafluoroborate (BF_4_^−^) of 1a to hexafluorophosphate (PF_6_^−^) increased the solubility of the substrate but it had no influence on the outcome of the reaction (Table 1, compare entries 8 and 9). The reaction was also run using air initiation (open system) leading to a slight decrease of the yield (55%, Table 1, entry 10). In the absence of any added initiator, traces of oxygen proved to be sufficient to trigger the formation of 2 albeit in lower yield (28%, Table 1, entry 11) suggesting that an efficient chain process is taking place.

Since pyridine derivatives are challenging to purify, the reaction was further optimized with lepidine (Table 2). Reaction of the *N*-methoxylepidinium 1b·BF_4_ substrate furnished the alkylated lepidine 3 in 93% yield (GC analysis) (Table 2, entry 1). In this reaction, the use of 2,4,6-collidine was not necessary since no di- or polyalkylation was observed and the yield remains identical in the absence of collidine (Table 2, entry 2). An experiment with air initiation (open reaction vessel) gave the product in 95% yield (Table 2, entry 3). To guarantee an optimal reproducibility, all reactions were performed with DTBHN, air initiation being more influenced by the exact experimental setup. The nature of the alkoxy group (methoxy *vs.* ethoxy) was tested then (Table 2, entry 4) and did not affect the outcome of the reaction. Product isolation is often problematic with pyridine derivatives. Best results were obtained by filtration of the crude mixture through basic aluminum oxide to remove residual acid impurities and catechol byproducts followed by column chromatography on silica gel. With this method, the isolated yield closely matched the yield determined by GC analysis (Table 2, entry 5).

**Table 2:** Final optimization of the reaction with *N*-methoxylepidinium 1b·BF4

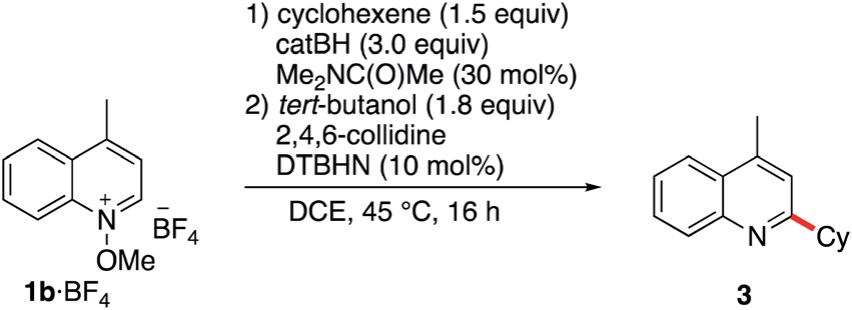			
Entry	Initiator	Collidine	Yield 3
1	DTBHN	3 equiv.	93% (GC)
2	DTBHN	—	93% (GC)
3	Air	—	95% (GC)
4	DTBHN	—	92% (GC)^a^
5	DTBHN	—	85% (isolated)

a Using the ethoxylepidinium instead of the methoxylepidinium salt.

#### Reaction scope

**Scheme 2:**
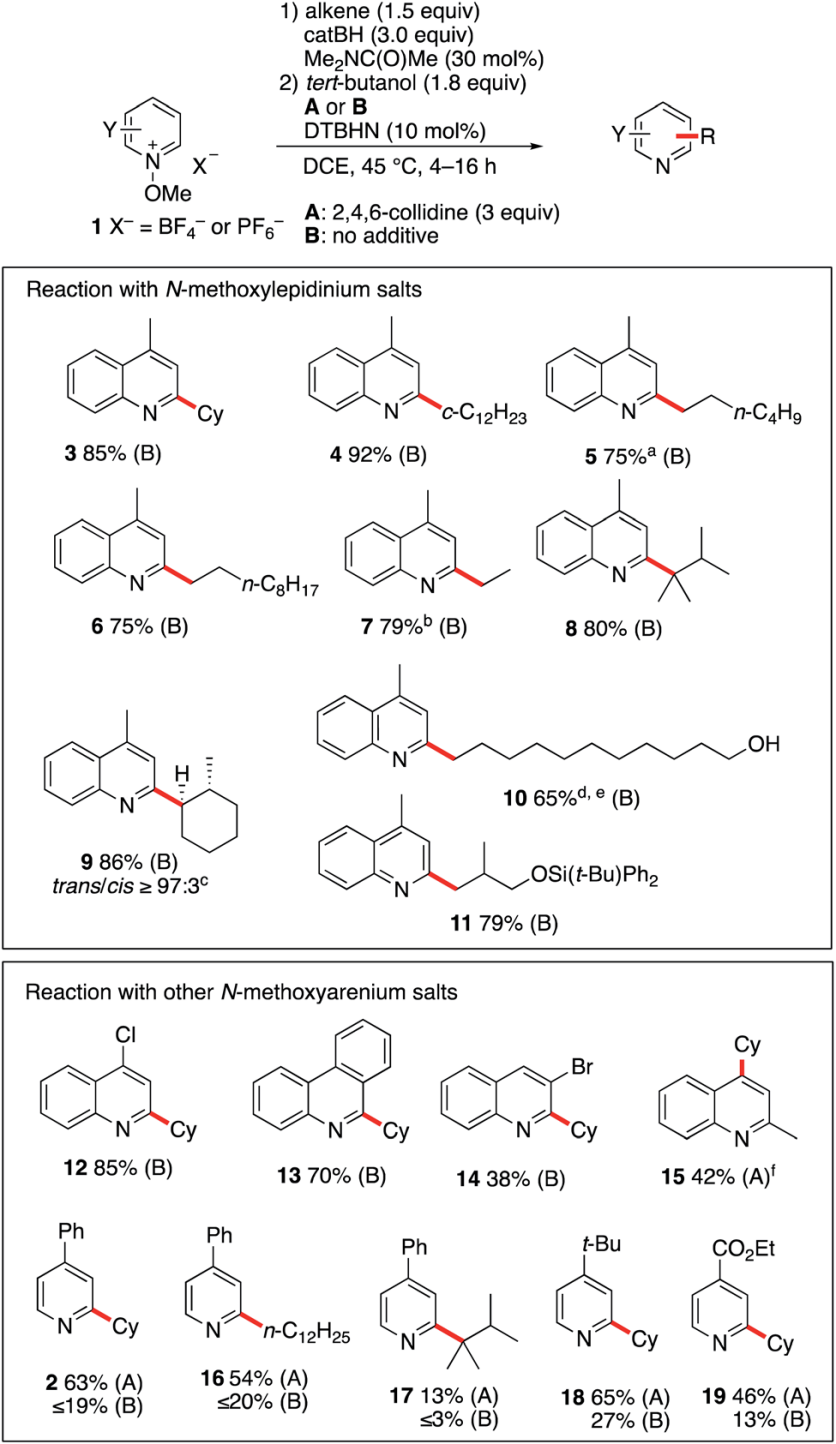
Reaction of *N*-methoxypyridinium salts 1 with a radical generated from *B*-alkylcatecholboranes. Yields refer to isolated products. (a) contains 11% of the branched 2-hexyl isomer. (b) Reaction run with Et_3_B (1.5 equiv.). (c) Diastereoselectivity determined by GC-analysis, only the major diastereoisomer is depicted. (d) Reaction run with catBH (4.5 equiv.). (e) The branched isomer (9%) was also isolated. (f) Using *N*-ethoxylepidinium tetrafluoroborate.

The optimized reaction conditions were tested with a series of quinolines and pyridines and several radical precursors. Results are summarized in Scheme 2. All reactions involving methoxyquinolinium ions were run without 2,4,6-collidine. Secondary cyclic radicals generated from cyclohexene and cyclododecene reacted with *N*-methoxylepidinium 1b to give products 3 and 4 in high yields. Terminal alkenes such as 1-hexene and 1-octene gave the alkylated products 5 and 6 in good yields. Since the hydroboration is not fully regioselective, small amounts of the branched isomers are also observed (6–11%). Performing the reaction with commercially available triethylborane as a source of ethyl radicals furnished product 7 in 79% yield. Even the tertiary alkyl radical generated from tetramethylethylene reacts efficiently with 1b to afford 8 in 80% yield. The diastereoselectivity of the process was investigated with 1-methylcyclohexene. The *trans* isomer of 9 was formed with an excellent stereocontrol (*trans*/*cis* ≥ 97 : 3). The reaction was also found to be efficient with alkenes containing a free hydroxy group. Starting from undec-10-en-1-ol, the linear hydroxyalkylated lepidine 10 was obtained in 65% yield. In this case too, a small amount (9%) of the branched isomer was also isolated. The silylether 11 was obtained in 79% yield from silylated 2-methallyl alcohol. Interestingly, in this first screening with 1b, products such as 9–11 are particularly easily accessible from alkenes with good to excellent regioselectivity control that cannot easily be matched using other Minisci type processes. A second series of experiments was performed with different *N*-methoxyheteroarenium salts. *N*-Methoxy-4-chloroquinolinium 1c and *N*-methoxyphenanthridin-5-ium 1d afforded upon reaction with cyclohexene the monoalkylated products 12 and 13 in 85% and 70% yield, respectively. Reaction of cyclohexene with *N*-methoxy-3-bromoquinolinium 1e gave product 14 in modest yield (38%). *N*-Methoxyquinaldinium 1f was not alkylated under the same reaction conditions. However, in the presence of 2,4,5-collidine (conditions A) the 4-cyclohexyquinaldine 15 was obtained in 38% yield together with a significant amount of quinaldine. A marginally higher yield was obtained with the *N*-ethoxyquinaldinium salt 1f′ (42%). A possible pathways leading to the formation of quinaldine involves deprotonation at the methyl position followed by a homolytic fragmentation of the N–OMe bond according to the work of Shen *et al.* liberating a methoxyl radical and a benzylic radical that can abstract a hydrogen from the reaction mixture.^32^ Reactions with substituted *N*-methoxypyridinium salts were also investigated. For these substrates, the use of 2,4,6-collidine had a very positive effect on the outcome of the reactions (compare conditions A with 2,4,6-collidine and B without a base). Reaction of 1a with cyclohexene and 1-dodecene under conditions A afforded 2 and 16 in 63% and 54% yields. Reaction with tetramethylethylene gave 17 in much lower yield. The 4-*tert*-butyl- and 4-ethoxycarbonyl-1-methoxypyridinium salts 1g and 1h were both monoalkylated with cyclohexene to afford 18 and 19 in 65% and 46% yields, respectively.

**Scheme 3:**
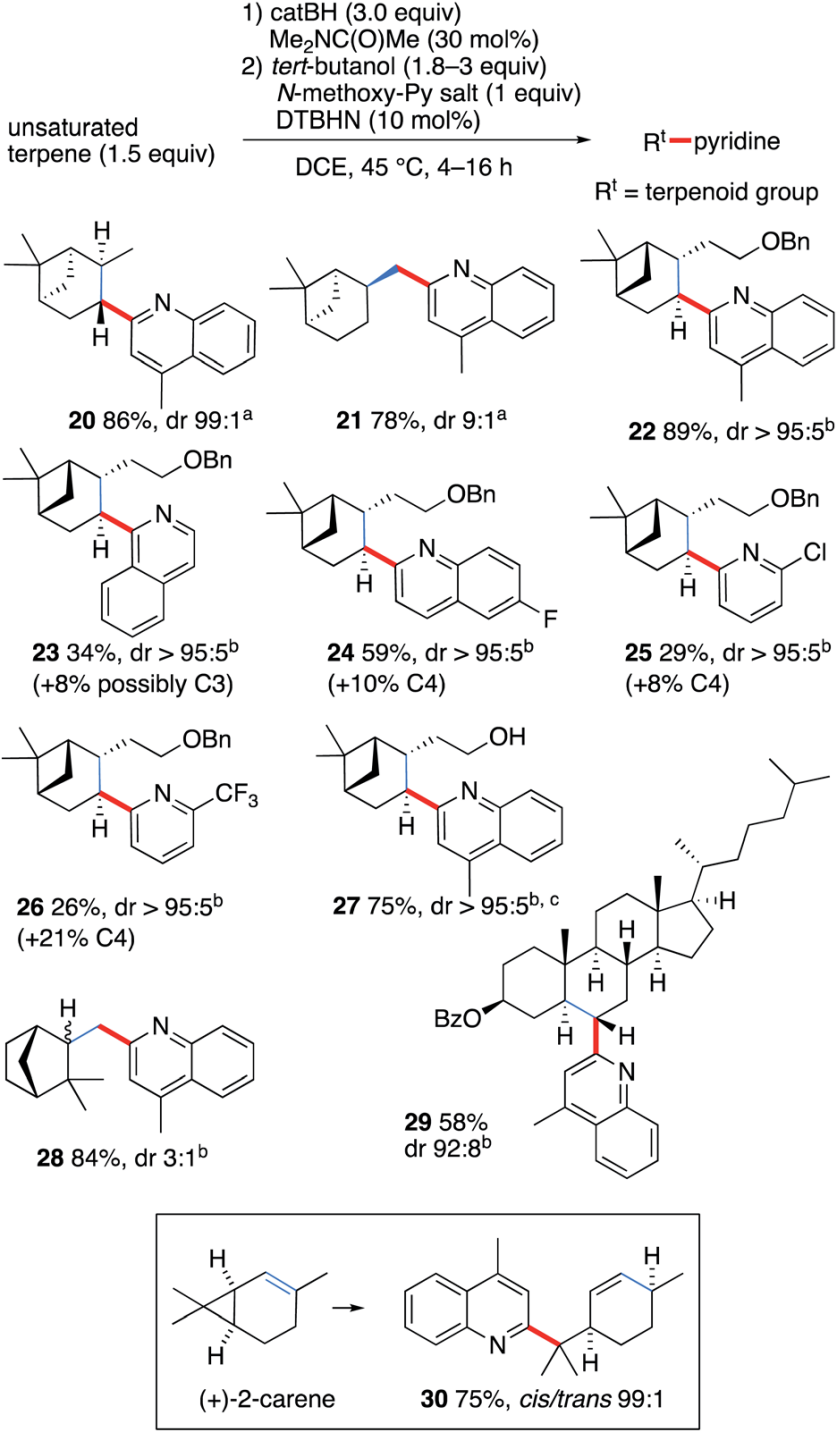
Pyridinylation of terpenoids. Radical probe experiment with (+)-2-carene. The position of the initial double bond is indicated in blue. Yields refer to isolated products. (a) Diastereoselectivity determined by GC-analysis. (b) Diastereoselectivity determined by ^1^H NMR. (c) Reaction run with catBH (4.5 equiv.).

In order to demonstrate further the utility of the hydroboration approach, the pyridinylation of a series of terpenoid natural products was investigated (Scheme 3). The reaction of α- and β-pinene provided the alkylated lepidines 20 and 21 in high yields and excellent stereocontrol.^42^ The reaction of *O*-benzyl-nopol was investigated next. It afforded the arylated quinoline and pyridines 22–26 in moderate to high yields. Satisfactory regiocontrol was observed, excepted for the 2-trifluromethylated pyridine 26 that was obtained as a nearly 1 : 1 mixture of C(2) and C(4) regioisomers. Non-protected nopol afforded the lepidinated product 27 in an excellent 75% yield. The reaction of camphene with 1b afforded the desired product 28 in 84% yield as a 3 : 1 mixture of diastereomers. Cholesteryl benzoate was converted to 29 in 58% yield with satisfactory stereocontrol (dr 92 : 8). Finally, the radical nature of the reaction was unambiguously demonstrated by the cyclopropane ring opening process observed when (+)-2-carene^43,44^ was used as a radical precursor. Reaction with 1b afforded the substituted lepidine 30 in 76% yield and high stereoselectivity (Scheme 3).

#### Mechanism

**Scheme 4:**
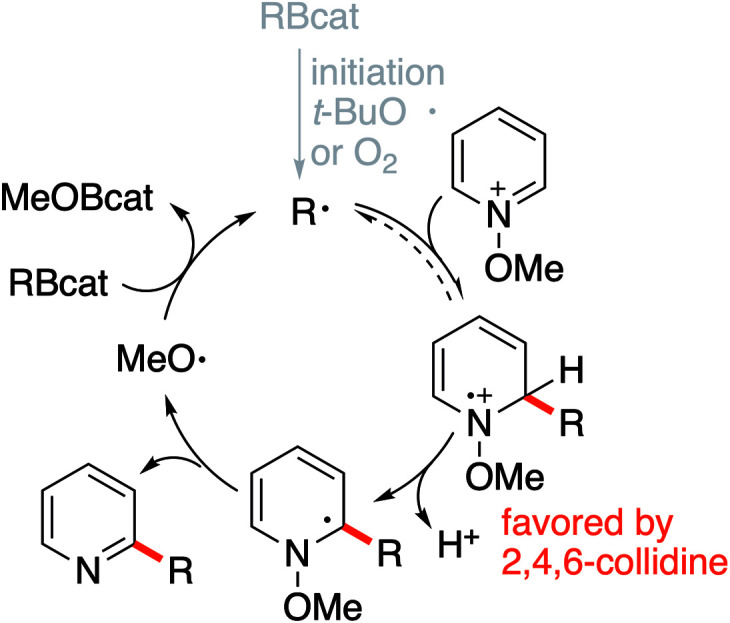
Proposed chain mechanism for the alkylation of *N*-methoxypyridinium salts.

The proposed mechanism for this reaction is depicted in Scheme 4. The chain is initiated by the thermal decomposition of DTBHN resulting in a *tert*-butoxyl radical that reacts with RBcat to produce the initial alkyl radical.^38,45,46^ Similarly, oxygen can initiate the reaction by reacting with the RBcat. Addition of the radical to the *N*-methoxypyridinium affords a radical cation intermediate. Depending on the nature of the alkyl radical and of the trap, this step may be a reversible process (see results involving alkyl iodides in Scheme 8). Rearomatization takes place by loss of a proton followed by rapid elimination of a methoxyl radical that propagates the chain reaction.^47^ The rearomatization presumably involves the formation of an intermediate α-amino radical. Similar intermediates are produced during the photoinduced demethoxylation of *N*-methoxypyridinium salts.^48–50^ 2,4,6-Collidine is expected to facilitate the rearomatization step and revoke the possible reversibility of the radical addition step. A related effect of a base has been recently reported by Shirakawa.^51,52^ This effect may explain why the more stabilized tertiary alkyl radicals react better in the presence of the base with the methoxypyridinium derivative (see formation of 18 in Scheme 2). Collidine is also expected to inhibit the acid catalyzed substitution process leading to dialkylation that can take place if the medium becomes too acidic.

The formation of MeO–Bcat was confirmed by analysis of the crude product before purification. Although 1.5 equivalents of *t*-BuOBcat are formed during the treatment of excess catBH with *tert*-butanol, the presence of nearly one equivalent of MeOBcat can be detected by ^11^B NMR. Indeed, the two borate esters give distinct signals at +22.4 ppm (*t*-BuBcat) and +23.5 ppm (MeOBcat).^53^

### (b) Reactions with alkyl iodides

#### Design and scope of the reaction

**Scheme 5:**
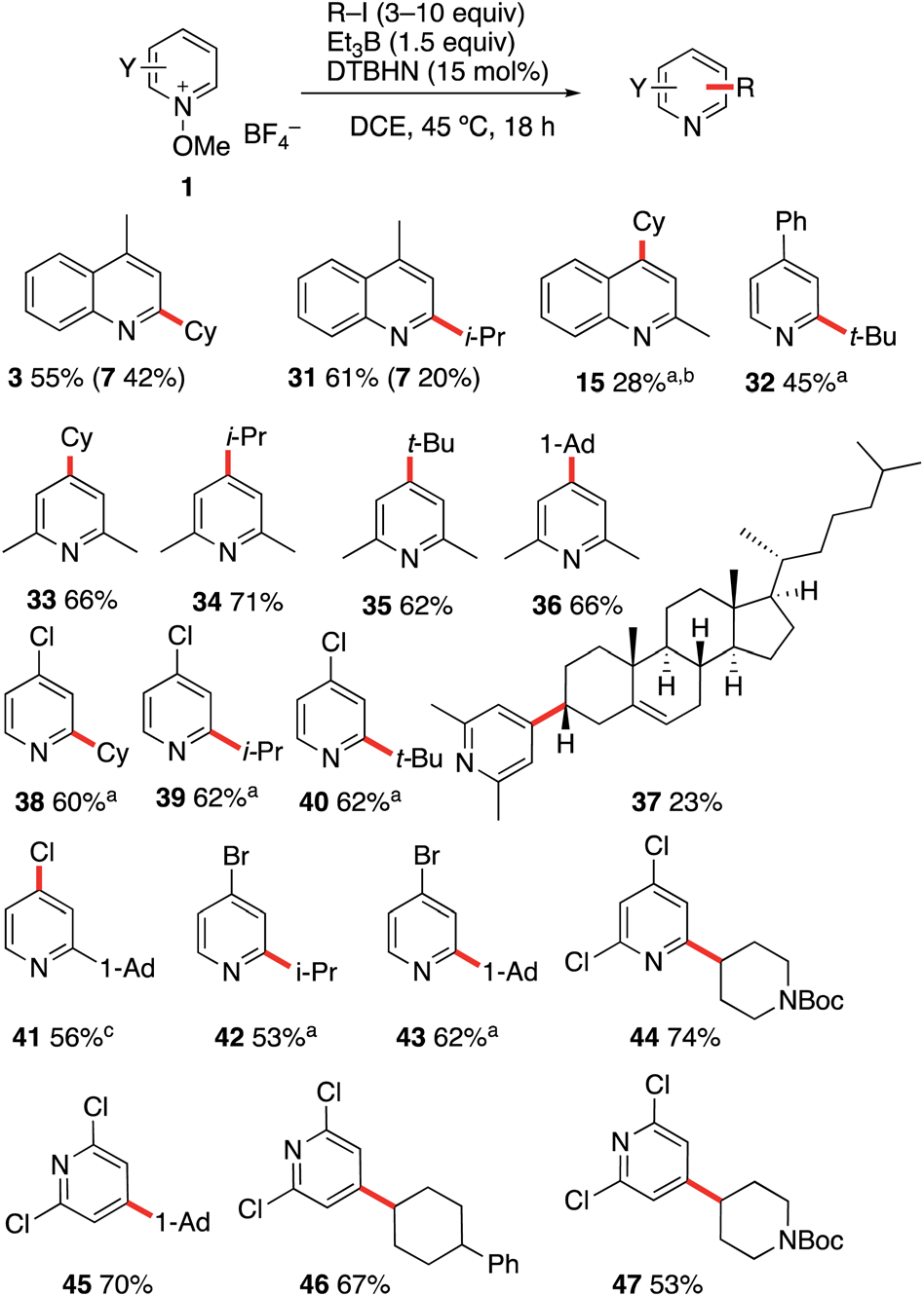
Alkylation of quinolines and pyridines with alkyl iodides. (a) Using 2,4,6-collidine (3 equiv.) as a base. (b) Using 20 equiv. of CyI. (c) Using K_2_CO_3_ (3 equiv.) as a base.

In order to extend the scope of the reaction to radicals generated from alkyl iodides, the reaction was examined in the presence of triethylborane as a chain transfer agent (Scheme 5). Starting from 1b and in the absence of alkyl iodides, the ethylated product 7 was obtained in 79% isolated yield (92% based on GC analysis). In the presence of cyclohexyl iodide (10 equiv.), a mixture of the cyclohexylated product 3 (55%) and ethylated 7 (42%) was obtained. The reaction with isopropyl iodide provided 31 (61%) together with 7 (20%). This result was expected since the iodine atom transfer process between a primary alkyl radical is faster with isopropyl iodide than with cyclohexyl iodide.^54^ Reaction of the methoxyquinaldinium 1f with 20 equivalents of cyclohexyl iodide and in the presence of triethylborane afforded the cyclohexylated quinaldine 15 in 28% yield. In this case, the ethylated product was only formed in trace amounts. The more efficient iodine atom transfer process indicates that the quinaldinium salt 1f is less reactive than the corresponding lepidinium derivative 1b towards alkyl radicals (see the Calculations below). The reaction was further investigated with *N*-methoxy-4-phenylpyridinium 1a and *N*-methoxy-2,6-lutidinium 1i. The products 32–36 were obtained in moderate to good yields using 6 equivalents of the starting iodides. The reaction with cholesteryl iodide (3 equivalents) afforded the desired lutidine 37 in 23% yield with an excellent level of stereocontrol. Similar yields were obtained in reactions involving the 4-chloro and 4-bromo-*N*-methoxypyridinium salts 1j and 1k with secondary and tertiary radicals (38–43). Highly electrophilic pyridinium salts such as 2,4- and 2,6-dichloro derivatives 1l and 1m afforded the corresponding products 44–47 in good yields. Among the latter, the adducts 44 and 47 are particularly attractive as two different heterocyclic structures are merged in a single step.

### (c) Reaction with xanthates

**Scheme 6:**
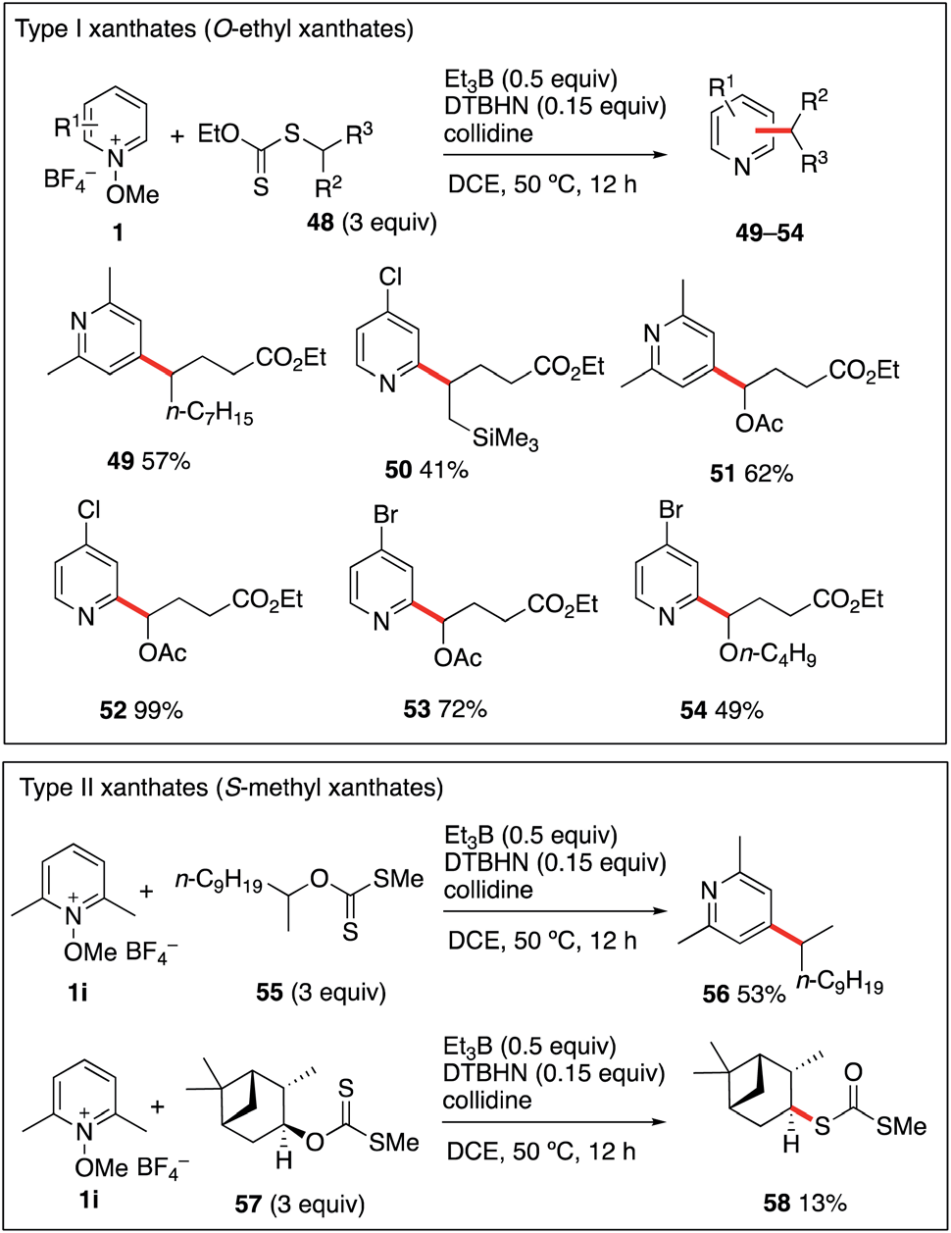
Alkylation of pyridines with xanthates.

The generation of more functionalized radicals such α-oxygenated and β-silylated radicals from iodide radical precursors cannot be performed due to the instability of the required iodides. This issue could be efficiently circumvented by using the more stable *O*-ethyl xanthate radical precursors (type I).^55–58^ Theses xanthates 48a–d were prepared by adding ethyl 2-((ethoxycarbonothioyl)thio)acetate to 1-nonene, allyltrimethylsilane, vinyl acetate, and vinyl butyl ether. Reaction of these three radical precursors with different *N*-methoxypyridinium salts 1i–k have been examined and results are reported in Scheme 6. The products 49–54 were isolated in moderate to excellent yields and the reaction tolerates the presence of a trimethylsilyl group at position 2 as well as 1-acetoxy and 1-butoxy groups. Extension of the reaction to *S*-methyl xanthates (type II), the well-established intermediates of the Barton–McCombie deoxygenation process,^59^ was examined next. The simple secondary *S*-methyl xanthate 55 was engaged in a reaction with *N*-methoxy-2,6-lutidinium 1i. Satisfyingly, it provided the desired 4-alkylated lutidine 56 in moderate 53% yield. Extending the reaction to the *S*-methyl xanthate 57 derived from isopinocampheol and 1i provided the dithiocarbonate rearrangement product 58 in 13% yield as the only identified product.^60,61^ This result is not surprising since xanthates of type II are good radical traps, and, in contrast to the type I xanthates, their reaction with the liberated alkyl radical is not a degenerative reaction liberating the original xanthate, but it leads to the formation of radical inactive dithiocarbonates such as 58.^55,62^ Finally, reaction of type II xanthate derived for cholesterol with *N*-methoxylepidinium 1b was attempted. In this case however, the only isolated product was 7 indicating that 1b was reacting much faster with the ethyl radical than the type II xanthate (see the ESI† for details). The results summarized in Scheme 6 indicate that type I xanthates are of synthetic interest for the alkylation of pyridines under Et_3_B-mediated conditions, while type II xanthates have a much more limited scope.

### (d) One-pot three-component alkylation of *N*-methoxy-pyridinium salts

**Scheme 7:**
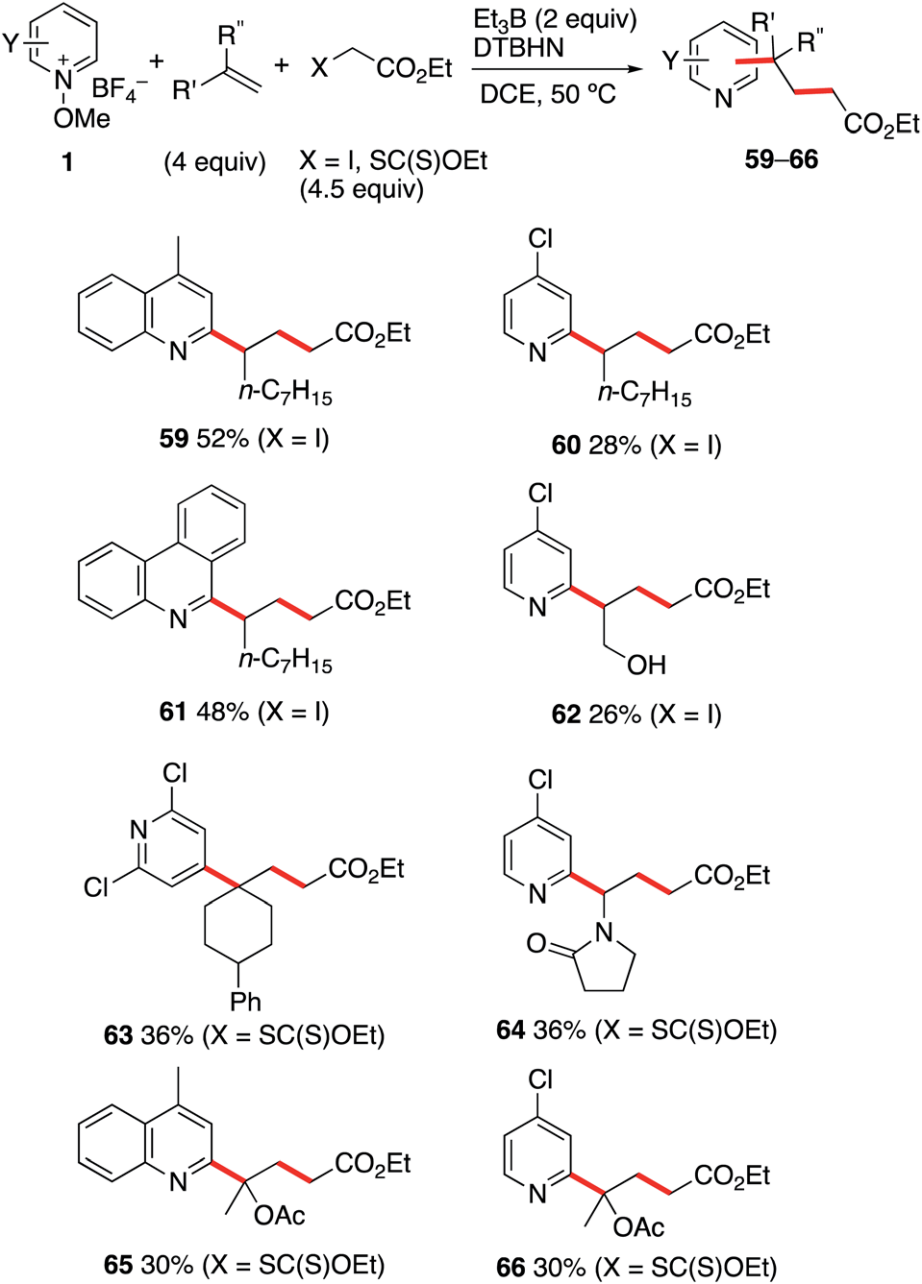
Carbopyridinylation of alkenes (three-component coupling process).

The reaction involving iodides and xanthates described above opens the possibility to develop a three-component coupling process involving a radical precursor, an alkene, and an *N*-methoxypyridinium salt. This approach is expected to complement the related photoredox catalyzed approaches involving *N*-methoxypyridinium salts,^35–37^ lepidinium and quinaldinium trifluoroacetate,^63^ as well as quinoxalin-2(1*H*)-ones.^64^ As the xanthates 48a–d are prepared by radical mediated xanthate transfer addition to the corresponding alkenes,^55–58^ all products presented in Scheme 6 result formally from a two-step carbopyridinylation reaction. Since electrophilic radicals are not expected to react with the *N*-methoxypyridinium salts 1, a one-pot process appears feasible. Gratifyingly, the Et_3_B-mediated three-component coupling process involving *N*-methoxypyridinium salts 1, electron-rich alkenes and ethyl α-iodo- or α-((ethoxycarbonothioyl)thio)acetate afforded the desired products in moderate yields (Scheme 7). Reaction of 1-nonene with ethyl iodoacetate and the *N*-methoxypyridinium salts 1b, 1j and 1d led to the formation of 59–61 in 28–52% yield. Interestingly, even the non-protected allyl alcohol could be used in this process giving 62 in 26% yield. For 2,2-disubstituted alkenes such as 1-phenyl-1-methylenecyclohexene, the best results for the formation of 63 were obtained with the xanthate radical precursor. Similar results were obtained with *n*-vinylpyrrolidinone and 2-acetoxypropene. In these last examples, the one-pot three-component approach is the only way to perform the transformation since isolation of the intermediate tertiary xanthate proved to be impossible. Moderate yields for the formation of 64–66 were obtained when the reactions were performed under these conditions.

### (e) Mechanistic investigations

#### Kinetic study

**Fig. 1:**
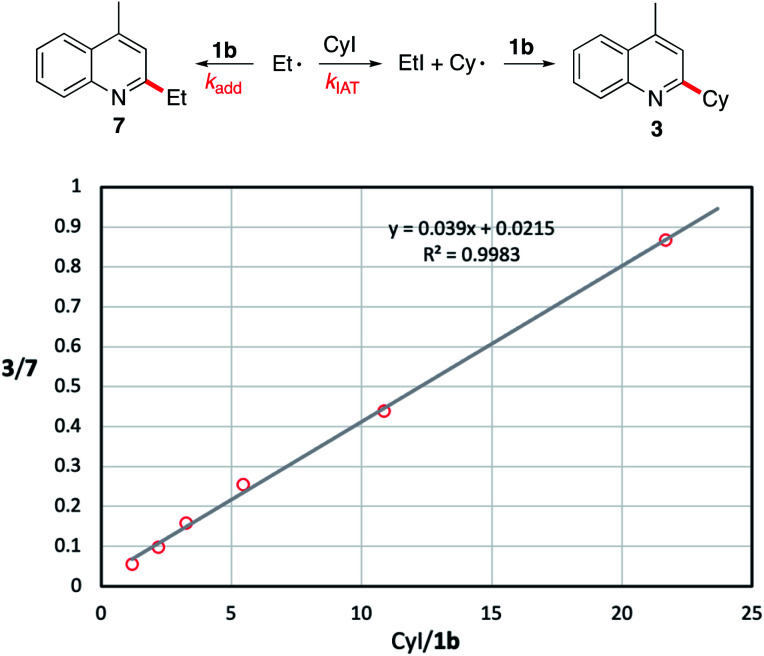
Estimation of the rate constant *k*_add_ for the addition of the ethyl radical to 1b at 50 °C. Reported *k*_IAT_ = 5.4 ± 0.9 ×10^5^ M^−1^ s^−1^ at 50 °C.^54^*k*_add_ = *k*_IAT_/slope. Using the experimental slope of 0.039, *k*_add_ (50 °C) = 1.4 ± 1 × 10^7^ M^−1^ s^−1^.

During our study with iodides (see point b), the formation of the ethylated product 7 was observed together with the cyclohexylated product 3 when the *N*-methoxylepidinium 1b was treated with triethylborane and cyclohexyl iodide (10 equivalents). Since the rate of iodine atom transfer between cyclohexyl iodide and a primary alkyl radical (the *n*-octyl radical) (*k*_IAT_ = 5.4 ± 0.9 ×10^5^ M^−1^ s^−1^ at 50 °C)^54^ has been reported, it is possible to estimate the rate of addition *k*_add_ to *N*-methoxylepidinium by running a competition experiment. For this purpose, mixtures of 1b and various amounts of cyclohexyl iodide were treated with Et_3_B under DTBHN initiation. The reaction was stopped at low conversion to ensure quasi-steady-state conditions. Plotting the ratio of 3/7 relative to 1b/CyI gave a straight line (*R*^2^ = 0.9983) whose slope can be used to estimate *k*_add_ (slope = *k*_IAT_/*k*_add_). Results are summarized in Fig. 1. From this study, a rate constant of 1.4 ± 1 × 10^7^ M^−1^ s^−1^ (50 °C) was obtained for the addition of the ethyl radical to *N*-methoxylepidinium 1b. This is more than one order of magnitude larger than the rate constant measured for addition of the *n*-butyl radical to protonated lepidine (*k*_add_ = 4.8 × 10^5^ M^−1^ s^−1^ (50 °C))^65^ confirming the remarkable reactivity of *N*-methoxypyridinium salts towards alkyl radicals. Since the literature rate constant for the addition of primary alkyl radicals to protonated lepidine was measured under very different reaction conditions, *i.e.* by generating the radicals by reduction of valeroyl peroxide with Cu(OAc) in an acetic acid/water mixture in the presence of CuCl_2_ as a competitive radical trap, we decided to check if this value was also valid under our reaction conditions. For this purpose, two competition experiments were run with 10 and 20 equivalents of cyclohexyl iodide relative to lepidinium trifluoroacetate under reaction conditions close to the ones use above with *N*-methoxylepidinium 1b (50 °C in DCE, see the ESI† for details). The measured ratio of 3/7 is in good agreement with the rate constant measured by Citterio, Minisci and co-workers, confirming the significant difference of reactivity of *N*-methoxylepidinium tetrafluoroborate 1b and lepidinium trifluoroacetate. To illustrate further the very high reactivity of 1b towards radicals, the reported rate constant for the addition of primary alkyl radicals to methyl acrylate (*k*_add_ = ≤5 × 10^5^ M^−1^ s^−1^ at 0–20 °C),^66,67^ one of the most commonly used radical traps, is considerably smaller than the one we report here for the methoxylepidinium salt 1b. Interestingly, in the case of methyl acrylate too, a good match of kinetic data between reactions run under very different conditions (dichloromethane^67^*vs.* acetic acid/acetonitrile/copper salts^66^) was already reported by Giese and co-workers.

#### Reversibility of the radical addition

**Scheme 8:**
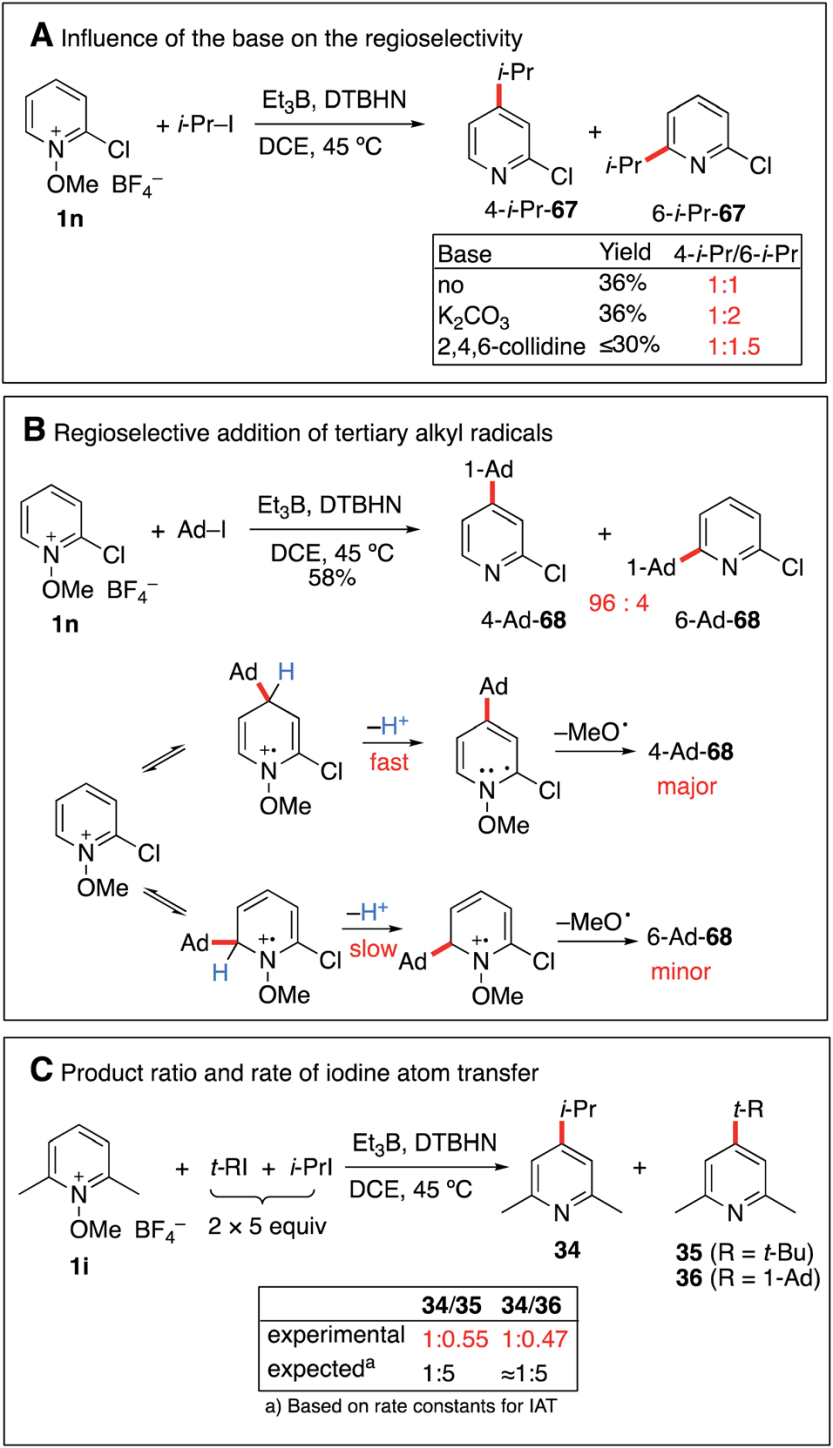
Regioselectivity and possible reversibility of the radical addition.

The reaction of 2-chloro-*N*-methoxypyridinium salt 1n with isopropyl iodide afforded 67 in 36% yield as a nearly 1 : 1 mixture of 4- and 6-isopropyl regioisomers (Scheme 8A). By running the reaction in the presence of K_2_CO_3_, a similar yield was obtained for 67 but the formation of the 6-isopropyl-2-chloropyridine became the major process. A similar trend, albeit less pronounced was observed when 2,4,6-collidine was used as a base. This influence of the base on the regioselectivity is attributed to a faster deprotonation of the intermediate radical cation that favors formation of the 6-isopropyl addition product.

**Fig. 2:**
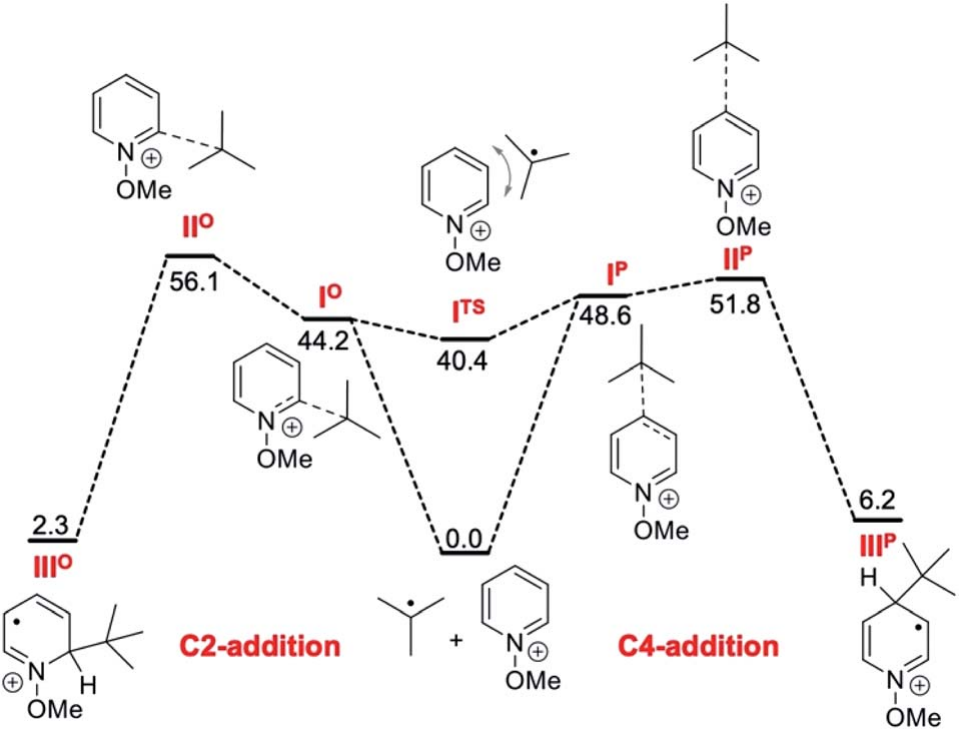
Reaction free energy profile (kJ mol^−1^) in dichloromethane for the reaction of the *tert*-butyl radical with the *N*-methoxypyridinium cation calculated at the SMD(DCM)/DLPNO-CCSD(T)/cc-pVTZ//M06-2X/def2-TZVP level of theory. Single point solvation energies are calculated at the SMD(DCM)/UB3LYP/6-31+G(d)//M06-2X/def2-TZVP level of theory.

Interestingly, the same reaction with 1-iodoadamantane afforded 68 in 58% yield as a 94 : 6 mixture of 4-Ad/6-Ad (Scheme 8B). Similar regioselectivities for tertiary alkyl radicals were already observed by Herzon and co-workers.^29^ The impressive difference of regioselectivity between a secondary and a tertiary radical may not strictly reflect the different rates of addition but it may also be a consequence of the possible reversibility of the reaction. Indeed, addition of tertiary radicals is expected to be fast and reversible (see the Calculations below) (Fig. 2) and the observed product ratio may be governed by the difference of deprotonation rates, the 4-adamantyl adduct being more difficult to deprotonate due to steric reasons (see Scheme 8B).

A further indication of reversibility was observed when the alkylation of *N*-methoxy-2,6-dimethylpyridine 1i was run with an equimolar excess of a *tert*-butyl or adamantyl iodide and isopropyl iodide (Scheme 8C). In both cases, the major product was the 4-isopropylpyridine 34 despite the fact that iodine atom abstractions involving *tert*-butyl iodide (*k*_IAT_ = 3 × 10^6^ M^−1^ s^−1^ at 50 °C)^54^ and presumably also adamantyl iodide are faster than the one involving isopropyl iodide (5.6 × 10^5^ M^−1^ s^−1^ at 50 °C).^54^ This result is best explained by the higher reversibility of the addition of the more stable *tert*-butyl radical compared to the isopropyl radical.

#### Calculations

In order to gain more insight into the mechanism and factors that influence the regioselectivity, calculations were performed. First, the reaction of the *tert*-butyl radical with the 1-methoxypyridinium cation was studied in the gas phase at the M06-2X^68^/def2-TZVP^69^ level of theory. This is similar to earlier studies where this level of theory has been used for single point calculations.^70^ The initially obtained gas phase (U)M06-2X/def2-TZVP results were subsequently refined by DLPNO-CCSD(T)^71,72^/cc-pVTZ^73^ single point calculations to obtain more reliable gas phase reaction profiles. Addition of solvation free energies calculated through single point calculations at the SMD(DCM)^74^/B3LYP^75,76^/6-31+G(d)^77,78^ level and standard state corrections to the 1 mol L^−1^ standard state then yielded the final free energies in solution that are depicted in Fig. 2. The reactants first meet to form two loosely bound, but structurally well-defined reactant complexes I*^p^* and I*^o^*, whose interconversion through transition state I^TS^ occurs with minimal energetic effort. Addition at C4 has a somewhat lower barrier as compared to addition at C2 (+51.8 *vs.* +56.1 kJ mol^−1^). Interestingly, both addition reactions are actually endergonic, the C2 addition product III*^o^* being slightly more stable (Δ*G*_298_ = +2.3 kJ mol^−1^) as compared to the C4 addition product III*^p^* (Δ*G*_298_ = +6.2 kJ mol^−1^). The formation of both products is thus likely to be at least partly reversible under a variety of reaction conditions, and the final product distribution may thus depend on the rate of the following deprotonation step. As expected, reaction barriers and reaction energies are significantly less favorable for formation of the C3 adduct II*^m^* (not depicted in Fig. 2, see the ESI† for details) to a degree that the formation of C3 addition products may not play any role in practical experiments. We note in passing that transition states for the direct migratory interconversion of adducts III*^o^*, III*^p^*, and III*^m^* could not be located (see the ESI† for details involving proton and alkyl shift pathways).

**Fig. 3:**
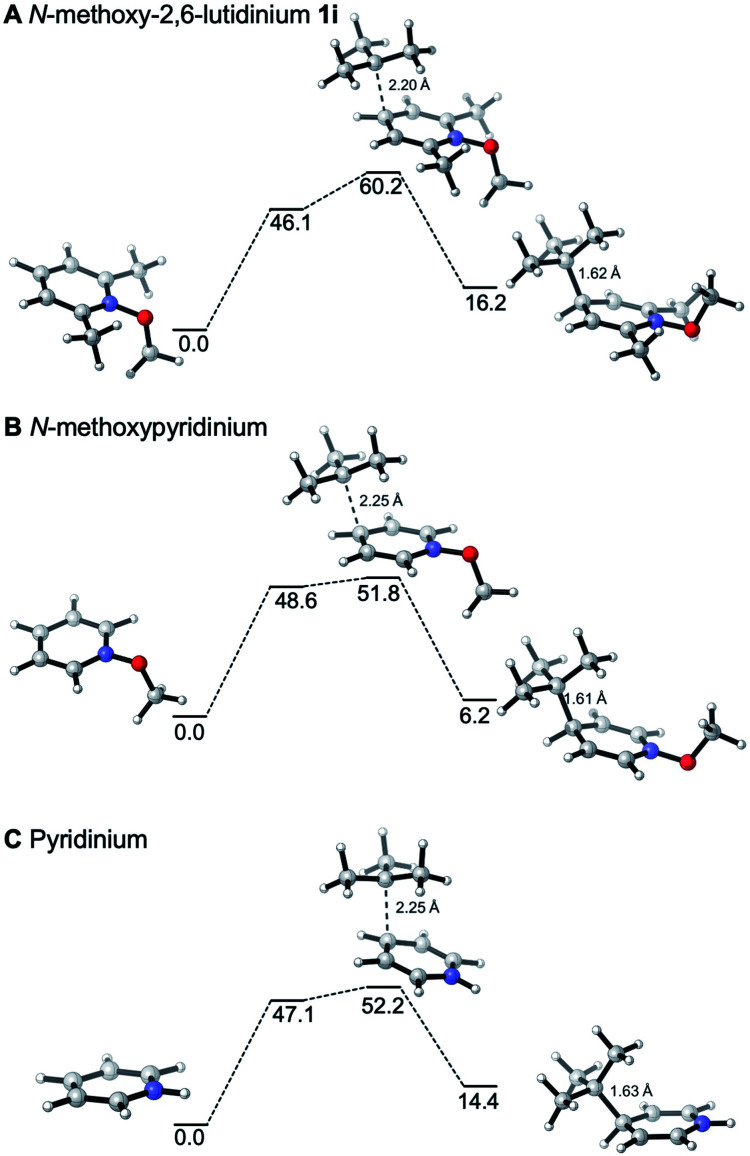
Reaction free energy profiles (kJ mol^−1^) for the addition of the *tert*-butyl radical to the *N*-methoxy-2,6-lutidinium salt (A), *N*-methoxypyridine (B) and protonated pyridine (C) at the SMD(DCM)/DLPNO-CCSD(T)/cc-pVTZ//M06-2X/def2-TZVP level of theory. Single point solvation energies are calculated at the SMD(DCM)/UB3LYP/6-31+G(d)//M06-2X/def2-TZVP level of theory. Energies are given in kJ mol^−1^.

**Fig. 4:**
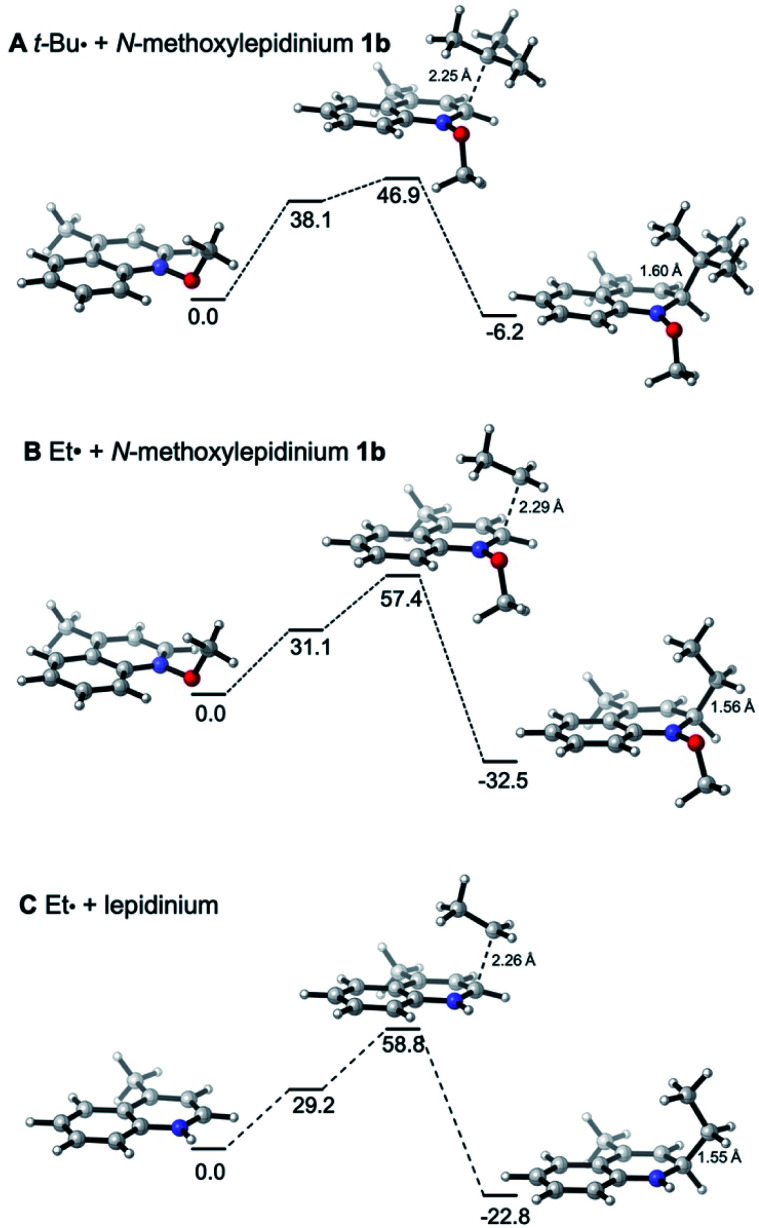
Reaction free energy profiles (Δ*G*_sol_, kJ mol^−1^) for the addition of the (A) *tert*-butyl and (B) ethyl radicals to *N*-methoxylepidinium and of the (C) ethyl radical to protonated lepidine calculated at the SMD(DCM)/DLPNO-CCSD(T)/cc-pVTZ//M06-2X/def2-TZVP level of theory. Single point solvation energies are calculated at the SMD(DCM)/UB3LYP/6-31+G(d)//M06-2X/def2-TZVP level of theory.

The reactivities of various *N*-methoxypyridinium systems towards *tert*-butyl radical addition were investigated next by comparing the addition energy for different systems such as *N*-methoxypyridinium (C4-addition, Δ*G*^‡^ = +51.8 kJ mol^−1^, Δ*G* = +6.2 kJ mol^−1^), *N*-methoxy-2,6-lutidinium 1i (C4-addition, Δ*G*^‡^ = +60.2 kJ mol^−1^, Δ*G* = +16.2 kJ mol^−1^), *N*-methoxyquinaldinium 1f (C4-addition, Δ*G*^‡^ = +47.2 kJ mol^−1^, Δ*G* = −3.4 kJ mol^−1^, see ESI†) and *N*-methoxylepidinium 1b (C2-addition, Δ*G*^‡^ = +46.9 kJ mol^−1^, Δ*G* = −6.2 kJ mol^−1^) as well as protonated pyridine (C4-addition, Δ*G*^‡^ = +52.2 kJ mol^−1^, Δ*G* = +14.4 kJ mol^−1^). For the more reactive quinolinium salts 1f and 1b, the radical addition reactions are slightly exergonic (Δ*G* = −3.4 and −6.2 kJ mol^−1^, respectively). In the case of the less reactive *N*-methoxy-2,6-lutidinium 1i, the reaction is strikingly endergonic (Δ*G* = +16.2 kJ mol^−1^) and therefore reversibility is expected to occur (Fig. 3A) supporting the experimental results presented in Scheme 8C. The energy profiles of the addition of the *tert*-butyl radical to the methoxypyridinium salt (Fig. 3B) and the protonated pyridine (Fig. 3C) have similar activation energies, but differ noticeably in their thermochemistry, the addition to the protonated pyridine being 8 kJ mol^−1^ more endergonic. A similar trend was found for the addition of the ethyl radical to *N*-methoxylepidinium and protonated lepidine (see Fig. 4B and C). The difference of the addition rates observed for these two systems may result from stronger interactions of the protonated species with the counterions, an effect that was neglected in our calculations.

In Fig. 4, the energy profiles for the addition of the *tert*-butyl and ethyl radicals to *N*-methoxylepidinium 1b (Fig. 4A and B) as well as the addition of the ethyl radical to the protonated lepidinium (Fig. 4C) are depicted. As anticipated based on the nucleophilic character of the radicals, the energy barrier for the addition of the ethyl radical (Δ*G*^‡^ = +57.4 kJ mol^−1^) is higher than the one calculated for the *tert*-butyl radical (Δ*G*^‡^ = +46.9 kJ mol^−1^). Also anticipated, the addition of the ethyl radical is more exothermic (Δ*G* = −32.5 kJ mol^−1^) than the addition of the *tert*-butyl radical (Δ*G* = −6.2 kJ mol^−1^). These results support the hypothesis made in our kinetic study that the addition of the ethyl radical to *N*-methoxylepidinium is irreversible under our reaction conditions. Finally, the addition of the ethyl radical to the protonated lepidine indicates, in agreement with the pyridine system discussed above (Fig. 3B and C), that the activation energies for the addition step are very similar but the thermochemistry is less favorable by about 10 kJ mol^−1^ for the protonated lepidine relative to the *N*-methoxylepidinium 1b.

The outcome of our calculations fits well with literature reports. Minisci and co-workers have reported experimental evidence for the reversibility of the addition of stabilized radicals such as benzyl,^79^*tert*-butyl^80^ and α-oxygenated^81^ radicals to protonated pyridines. This reversibility was recently invoked by Phipps and co-workers to rationalize the enantioselective Minisci reactions involving stabilized α-aminoalkyl radicals.^82^ Our calculations support these findings showing that the addition of a *tert*-butyl radical to protonated pyridine is an endergonic process. *N*-Methoxypyridinium salts behave similarly but the reaction is less endergonic and even slightly exergonic with quinoline derivatives. To get a full picture of the factors that govern the regioselectivity, the energy barrier for the fast subsequent deprotonation step should be calculated. However, we did not perform these calculations because of their arbitrary character due to the strong dependence on the solvation model used.

## Conclusion

An efficient and experimentally simple method for monoalkylation of pyridine derivatives and related compounds has been developed. The transformation is achieved by reaction of *N*-methoxypyridinium salts, easily prepared by alkylation of *N*-oxides with trimethyloxonium tetrafluoroborate (Meerwein's salt). The generality of the process is demonstrated by using radicals generated either from alkenes *via* a hydroboration process, from alkyl iodides and from xanthates. In terms of regioselectivity control, the hydroboration approach complements nicely the cobalt-mediated pyridinylation developed by Herzon. The high selectivity observed for the formation of monoalkylated products is best explained by the exceptional reactivity of the *N*-methoxypyridinium salts towards radicals. Indeed, these pyridine salts were found to react faster with radicals than the corresponding protonated pyridines. All these reactions rely on an efficient chain reaction involving the fragmentation of a weak N–OMe bond leading to rearomatization and generation of a methoxyl radical that sustains the chain process by reaction with an organoboron species. Based on the strong favorable polar effects, a three-component coupling process leading to the carbopyridinylation of electron-rich alkenes could also be performed. This work is expected to find applications in natural product synthesis and this aspect of the chemistry is currently being further investigated.

## Data availability

The datasets supporting this article have been uploaded as part of the ESI.†

## Author contributions

P. R. secured funding for the project and wrote the initial research proposal. P. R., S. R., C. M. and F. D. conceptualized the work and interpreted the results. S. R., C. M., K. M. and F. D. conceived and performed the experiments. H. J. and H. Z. performed the calculations. S. R. wrote the initial draft, P. R., C. R. and F. D. prepared the final version and all the authors discussed the results and commented on the manuscript.

## Conflicts of interest

There are no conflicts to declare.

## Supplementary Material

SC-012-D1SC02748D-s001

SC-012-D1SC02748D-s002
