# Supplementary material for: Radical chain monoalkylation of pyridines
Source: Chem Sci. 2021 Nov 3;12(46):15362–73. doi: 10.1039/d1sc02748d (PMC8635225; doi:10.1039/d1sc02748d)
Supplement: SC-012-D1SC02748D-s001 [file SC-012-D1SC02748D-s001.pdf]

Electronic Supplementary Material (ESI) for Chemical Science.
This journal is © The Royal Society of Chemistry 2021

Supporting Information

Radical Chain Monoalkylation of Pyridines

Samuel Rieder, Camilo Meléndez, Fabrice Dénès, Harish Jangra, Kleni Mulliri, Hendrik Zipse* and Philippe Renaud*

Theoretical methods

All geometry optimizations have been performed in the gas phase using the M06-2X hybrid functional[2] and the def2-TZVP basis set.[3] This is closely similar to earlier studies,[1] where this level of theory has been used for single point calculations in geometries optimized at B3LYP[5]/6-31+G(d) level.[6,7,8] This latter level of theory was subsequently used for the calculation of solvation free energies in dichloromethane with the SMD continuum solvation model following a single point strategy. Finally, gas phase energies were recalculated at the DLPNO-CCSD(T)/cc-pVTZ level.[4,10] All free energy values in solution include a correction of +7.91 kJ/mol (= 0.0030128 Hartree) for a standard state of 1 mol/l.

References

[1] F. Rammal, D. Gao, S. Boujnah, A. A.Hussein, J. Laleveé, A.-C. Gaumont, F. Morlet-Savary, S. Lakhdar, *ACS Catal.* **2020**, *10*, 13710 − 13717.
[2] Y. Zhao, D.G. Truhlar, *Theor. Chem. Acc.* **2008**, *120*, 215 – 241.
[3] F. Weigend, R. Ahlrichs, *Phys. Chem. Chem. Phys.* **2005**, *7*, 3297 - 3305.
[4] T. H. Dunning, Jr., *J. Chem. Phys.* **1989**, *90*, 1007.
[5] A. D. Becke, *J. Chem. Phys.* 1993, **98**, 5648 - 5652.
[6] W. J. Hehre, R. Ditchfield, J. A. Pople, *J. Chem. Phys.* **1972**, *56*, 2257.
[7] P. C. Hariharan, J. A. Pople, *Theor. Chim. Acta.* **1973**, *28*, 213.
[8] T. Clark, J. Chandrasekhar, G. W. Spitznagel, P. v. R. Schleyer, *J. Comp. Chem.* **1983**, *4*, 294.
[9] A. V. Marenich, C. J. Cramer, and D. G. Truhlar, *J. Phys. Chem. B* **2009**, *113*, 6378 - 6396.
[10] (a) F. Neese, E. F. Valeev, *J. Chem. Theory Comput.* **2011,** *7,* 33. (b) M. Saitow, U. Becker, C. Riplinger, E. F. Valeev, F. Neese, *J. Chem. Phys.* **2016**, *146*, 164105. (c) A. Altun, F. Neese, G. Bistoni, *Beilstein J. Org. Chem.* **2018**, *14,* 919.
[11] H. Fischer, L. Radom, *Angew. Chem. Int. Ed.* **2001**, *40*, 1340 - 1371.

The reaction of *tert*-butyl radical (**1**) with 1-methoxypyridinium cation (**2**) was studied first in the gas phase at the M06-2X/def2-TZVP level of theory. Reaction and activation free energies for this reaction are shown in Table S1, and we will concentrate on the calculated free energy values in the following. The reactants first meet to form two loosely bound, but structurally well-defined reactant complexes **3p** and **3o**, whose interconversion through transition state **3TS** faces a barrier of only 10 kJ mol^{-1}.

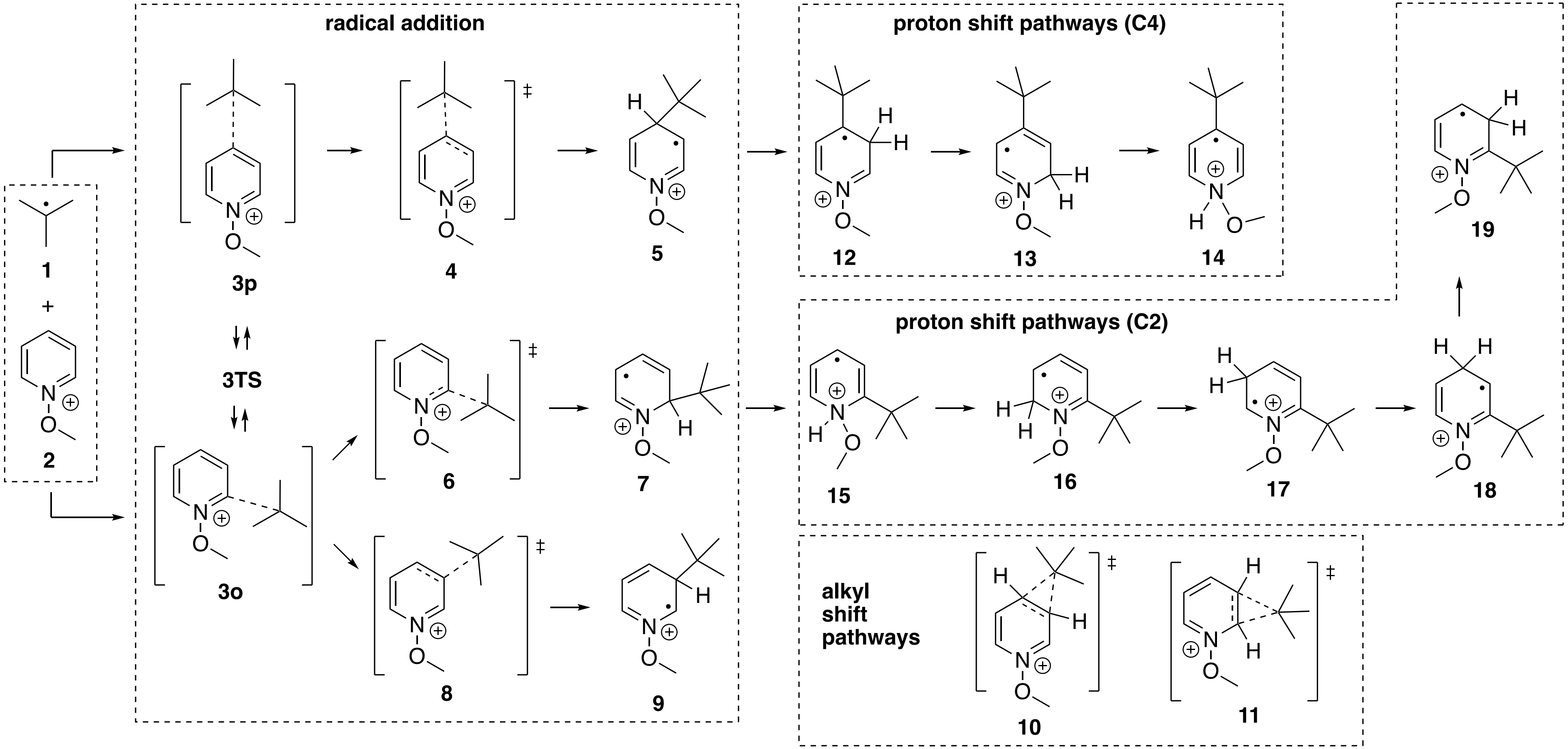

Figure S1. Reaction of *tert*-butyl radical (**1**) with 1-methoxypyridinium cation (**2**).

From reactant complex **3p** the reaction pathway for C4 addition proceeds with a barrier of only 6 kJ mol^{-1} to yield adduct **5** with an overall reaction free energy (relative to reactants) of ΔG(**5**) = -9.6 kJ mol^{-1}. This implies that the reverse reaction faces a barrier of (only) 26.8 kJ mol^{-1}. The competing addition at C2 of the pyridinium ring starts from reactant complex **3o** and proceeds with a barrier of approx. 12 kJ mol^{-1} through transition state **6** to C2 adduct **7** with a reaction free energy of ΔG(**7**) = -11.1 kJ mol^{-1}. Barriers and reaction energies for the C2 and C4 addition pathways are thus quite similar, and the final product distribution obtained may depend to some extent on the rates of the follow-up deprotonation reactions of the primary adducts **5** and **7**. Reaction barriers and reaction energies are significantly less favorable for formation of the C3 adduct **9** to a degree that the formation of C3 addition products may not play any role in practical experiments. We note in passing that transition states such as **10** or **11** for the direct migratory interconversion of adducts **5**, **7**, and **9** could not be located. The deprotonation of primary addition products **5**, **7**, and **9** through *intermolecular* proton transfer with an external (added) base competes, in principle, with the *intramolecular* proton migration from the addition site to the pyridine nitrogen atom, followed by methoxy radical elimination. In order to assess the energetic requirements of this latter pathway, the stabilities of the relevant tautomeric forms have been studied for the C4 and the C2 addition pathways. For the C4 addition pathway the relative free energies of intermediates (relative to reactants) are -9.6 kJ mol^{-1} (**5**), +1.3 kJ mol^{-1} (**12**), -15.9 kJ mol^{-1} (**13**), and +55.5 kJ mol^{-1} (**14**). The tautomer **14** required for the subsequent methoxy radical elimination step is thus quite unfavorable energetically. In addition, the reaction barriers for (unassisted) unimolecular 1,2-proton migration reactions between these tautomeric forms are quite unfavorable. An analogous analysis of intermediates along the C2 addition pathway yields reaction free energies of -11.1 kJ mol^{-1} (**7**) and +51.3 kJ mol^{-1} (**15**). The low stability of this latter tautomeric form thus represents a substantial hurdle for the methoxy radical elimination step. Other tautomeric forms such as **16** (-14.9 kJ mol^{-1}), **17** (+22.9 kJ mol^{-1}), **18** (-2.2 kJ mol^{-1}), and **19** (+10.3 kJ mol^{-1}) also exist, but are not necessarily intermediates along the overall addition/elimination pathway. All taken together these results indicate that the proton and alkyl shift pathways shown in Figures S1 may not add to the understanding of the radical addition reactions of 1-metjoxypyridinium ion 2. All further theoretical studies therefore concentrate on the actual addition steps.

Table S1. Relative energies (in kJ mol^{-1}) for the systems shown in Fig. S1.

system	E_{tot} (U)M06-2X/ def2-TZVP	H_{298} (U)M06-2X/ def2-TZVP	G_{298} (U)M06-2X/ def2-TZVP
**1 + 2**	0.0	0.0	0.0
**3p**	-41.3	-38.8	+11.4
**3TS (TS)**	-29.3	-26.2	+21.5
**3o**	-41.2	-35.6	+11.7
**addition to the C4 position**			
**3p**	-41.3	-38.8	+11.4
**4 (TS)**	-40.4	-36.8	+17.2
**5**	-73.5	-67.9	-9.6
**TS5_12 (TS)**	+76.3	+71.1	+131.5
**12**	-66.1	-58.1	+1.3

**TS12_13 (TS)**	+81.5	+76.5	+137.6
**13**	-84.3	-74.4	-15.9
**TS13_14 (TS)**	+178.2	+174.0	+233.0
**14**	-15.2	-4.2	+55.5
**addition to the C2 position**			
**3o**	-41.2	-35.6	+11.7
**6 (TS)**	-38.4	-34.6	+23.4
**7**	-86.6	-75.0	-11.1
**TS7_15 (TS)**	+176.1	+172.5	+234.2
**15**	-23.8	-12.2	+51.3
**16**	-88.2	-77.1	-14.9
**17**	-49.4	-40.5	+22.9
**18**	-74.3	-65.9	-2.2
**19**	-63.7	-54.0	+10.5
**addition to C3 the position**			
**3o**	-41.2	-35.6	+11.7
**8 (TS)**	-14.4	-10.7	+46.7
**9**	-57.0	-48.9	+10.8

The initially obtained gas phase (U)M06-2X/def2-TZVP results were subsequently refined by DLPNO-CCSD(T)/cc-pVTZ single point calculations to obtain more reliable gas phase reaction profiles. Addition of solvation free energies calculated through single point calculations at the SMD(DCM)/B3LYP/6-31+G(d) level and standard state corrections to the 1 mol/l standard state then yield the final free energies in solution. These are shown in Table S2 and in Figures S2 - S4 in a graphical format. Concentrating on the free energies in DCM solution shown in Figure S4, we note that addition at C4 has a somewhat lower barrier as compared to addition to C2 (+51.8 vs. +56.1 kJ/mol). Both addition reactions are actually endergonic, the C2 addition product **7** being slightly more stable (ΔG_{298} = +2.3 kJ/mol) as compared to the C4 addition product **5** at ΔG_{298} = +6.2 kJ/mol. The formation of both products is thus likely to be reversible under a variety of reaction conditions, and the final product distribution may thus depend on the rates of one of the following steps. Comparing these results with those obtained in the gas phase (shown in Figure S3), we note that both the barriers as well as the reaction energies have a very substantial solvation free energy component. This is due to the fact that 1-methoxypyridinium ion **1** is better solvated as compared to any other point along the reaction pathway.

Table S2. Energies of reaction of *tert*-butyl radical (**1**) with 1-methoxypyridinium cation (**2**) calculated at different levels of theory.

**M06-2X/def2-TZVP (gas phase)**												
	**ΔE**				**ΔH**				**ΔG**			
	**1 + 2**	**RC**	**TS**	**P**	**1 + 2**	**RC**	**TS**	**P**	**1 + 2**	**RC**	**TS**	**P**
**TS3**	0.0	-41.3	-29.3	-41.2	0.0	-38.8	-26.2	-35.6	0.0	11.4	21.5	11.7
**TS4 (C4)**	0.0	-41.3	-40.4	-73.5	0.0	-38.8	-36.8	-67.9	0.0	11.4	17.2	-9.6
**TS6 (C2)**	0.0	-41.2	-38.4	-86.6	0.0	-35.6	-34.6	-75.0	0.0	11.7	23.4	-11.1
**TS8 (C3)**	0.0	-41.2	-14.4	-57.0	0.0	-35.6	-10.7	-48.9	0.0	11.7	46.7	10.8
**DLPNO-CCSD(T)/cc-pVTZ//M06-2X/def2-TZVP (gas phase)**												
	**ΔE**				**ΔH**				**ΔG**			
	**1 + 2**	**RC**	**TS**	**P**	**1 + 2**	**RC**	**TS**	**P**	**1 + 2**	**RC**	**TS**	**P**
**TS3**	0.0	-25.5	-21.6	-28.8	0.0	-22.9	-18.3	-23.0	0.0	27.3	29.4	24.2
**TS4 (C4)**	0.0	-25.5	-27.9	-73.4	0.0	-22.9	-24.4	-67.7	0.0	27.3	29.6	-9.3
**TS6 (C2)**	0.0	-28.8	-28.2	-86.1	0.0	-23.0	-24.2	-74.4	0.0	24.2	33.8	-12.7
**TS8**	0.0	-28.8	-11.1	-61.0	0.0	-23.0	-7.3	-51.9	0.0	24.2	50.2	7.9
**SMD(DCM)/DLPNO-CCSD(T)/cc-pVTZ//M06-2X/def2-TZVP (DCM solution)** Single point solvation energies are calculated at the SMD(DCM)/UB3LYP/6-31+G(d)//M06-2X/def2-TZVP level of theory												
	**ΔE**				**ΔH**				**ΔG**			
	**1 + 2**	**RC**	**TS**	**P**	**1 + 2**	**RC**	**TS**	**P**	**1 + 2**	**RC**	**TS**	**P**
**TS3**	0.0	-6.0	-10.6	-8.8	0.0	-1.6	-7.3	-3.0	0.0	48.6	40.4	44.2
**TS4 (C4)**	0.0	-6.0	-7.4	-57.8	0.0	-1.6	-3.7	-52.0	0.0	48.6	51.8	6.2
**TS6 (C2)**	0.0	-8.8	-5.9	-70.1	0.0	-3.0	-1.9	-58.3	0.0	44.2	56.1	2.3
**TS8**	0.0	-8.8	8.8	-47.0	0.0	-3.0	12.6	-38.8	0.0	44.2	70.0	20.9

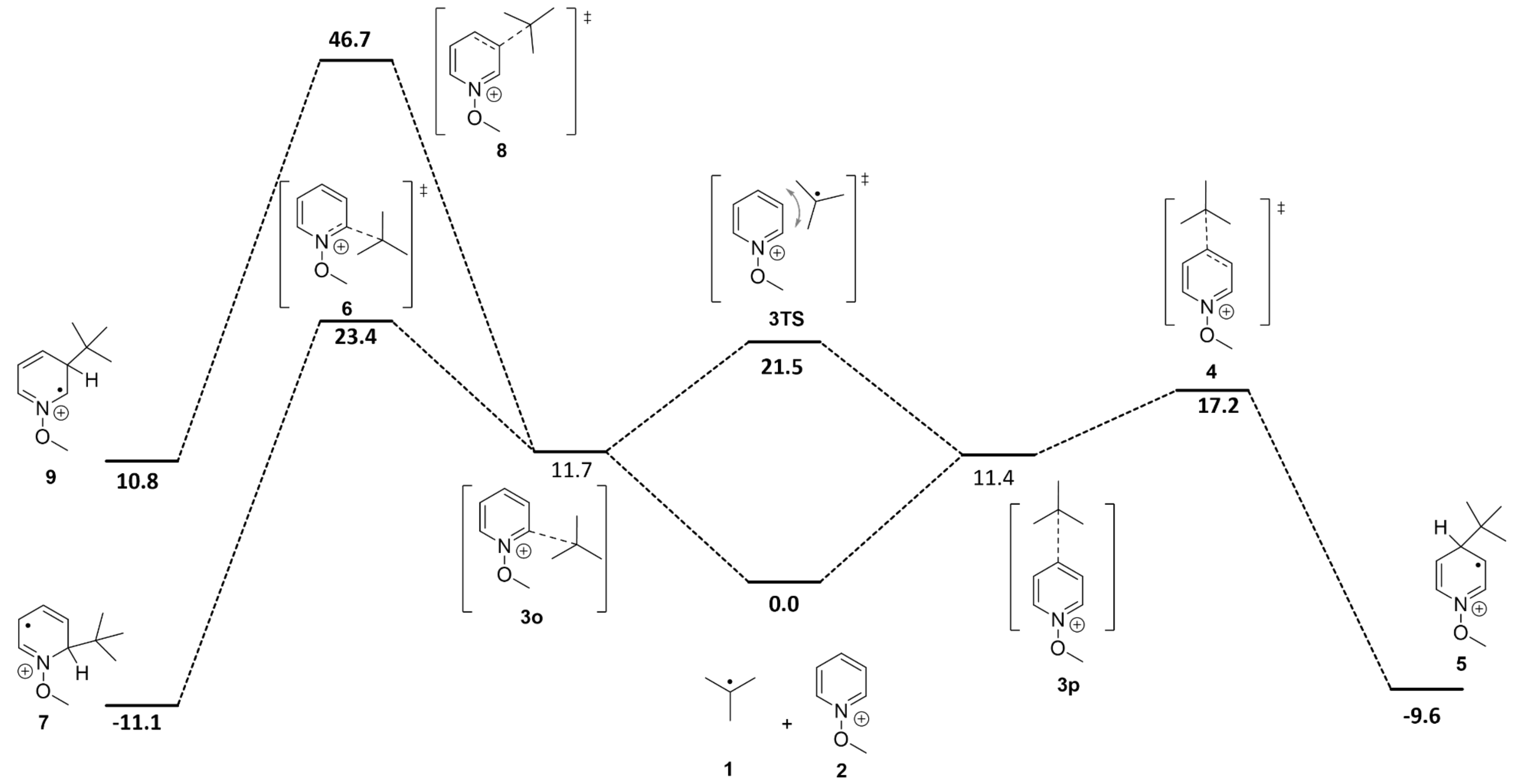

Figure S2. Gas phase reaction free energy profile for the reaction of *tert*-butyl radical (**1**) with 1-methoxypyridinium cation (**2**) calculated at the M06-2X/def2-TZVP level of theory.

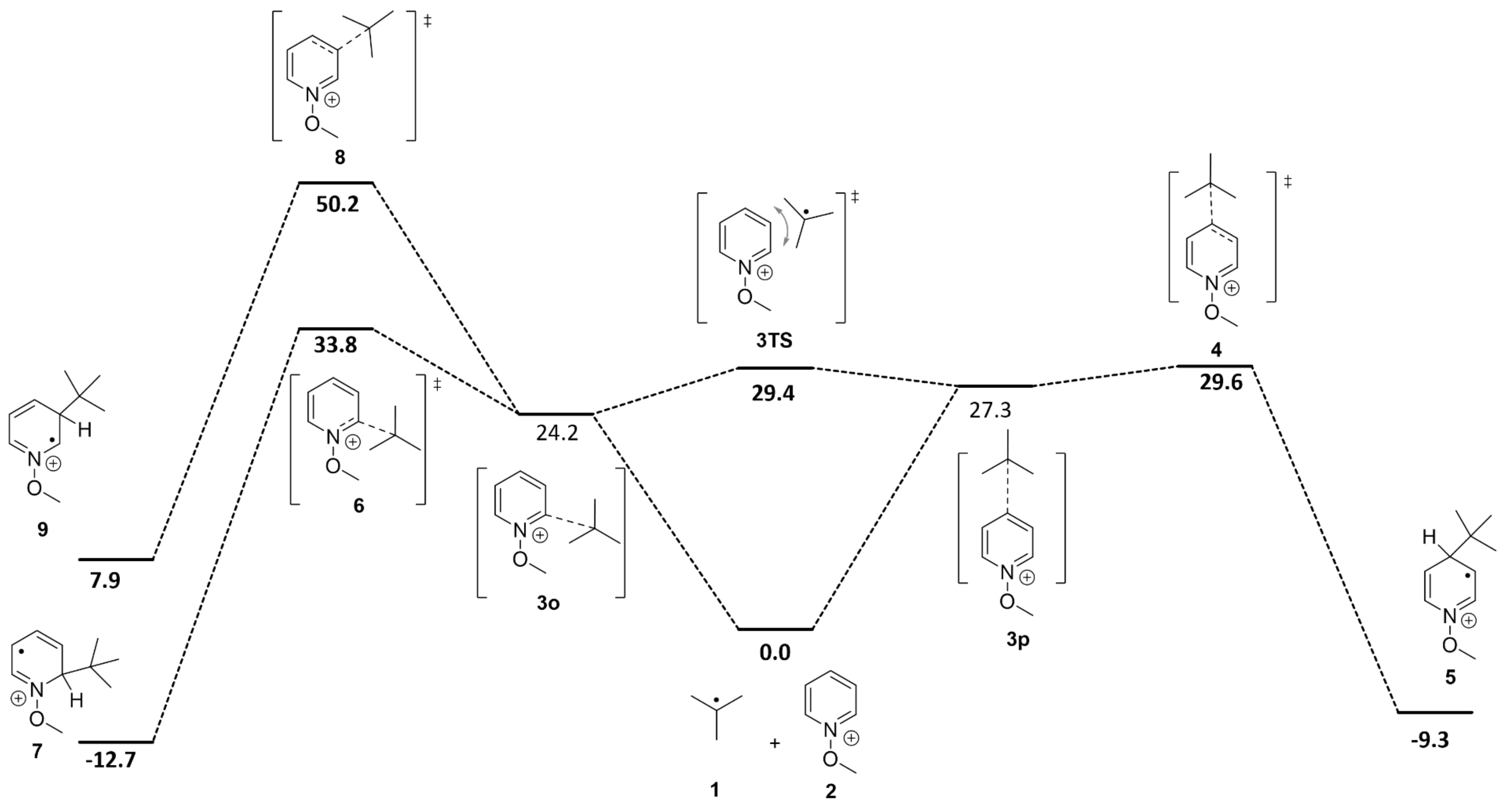

Figure S3. Gas phase reaction free energy profile for the reaction of *tert*-butyl radical (**1**) with 1-methoxypyridinium cation (**2**) calculated at the DLPNO-CCSD(T)/cc-pVTZ//M06-2X/def2-TZVP level of theory.

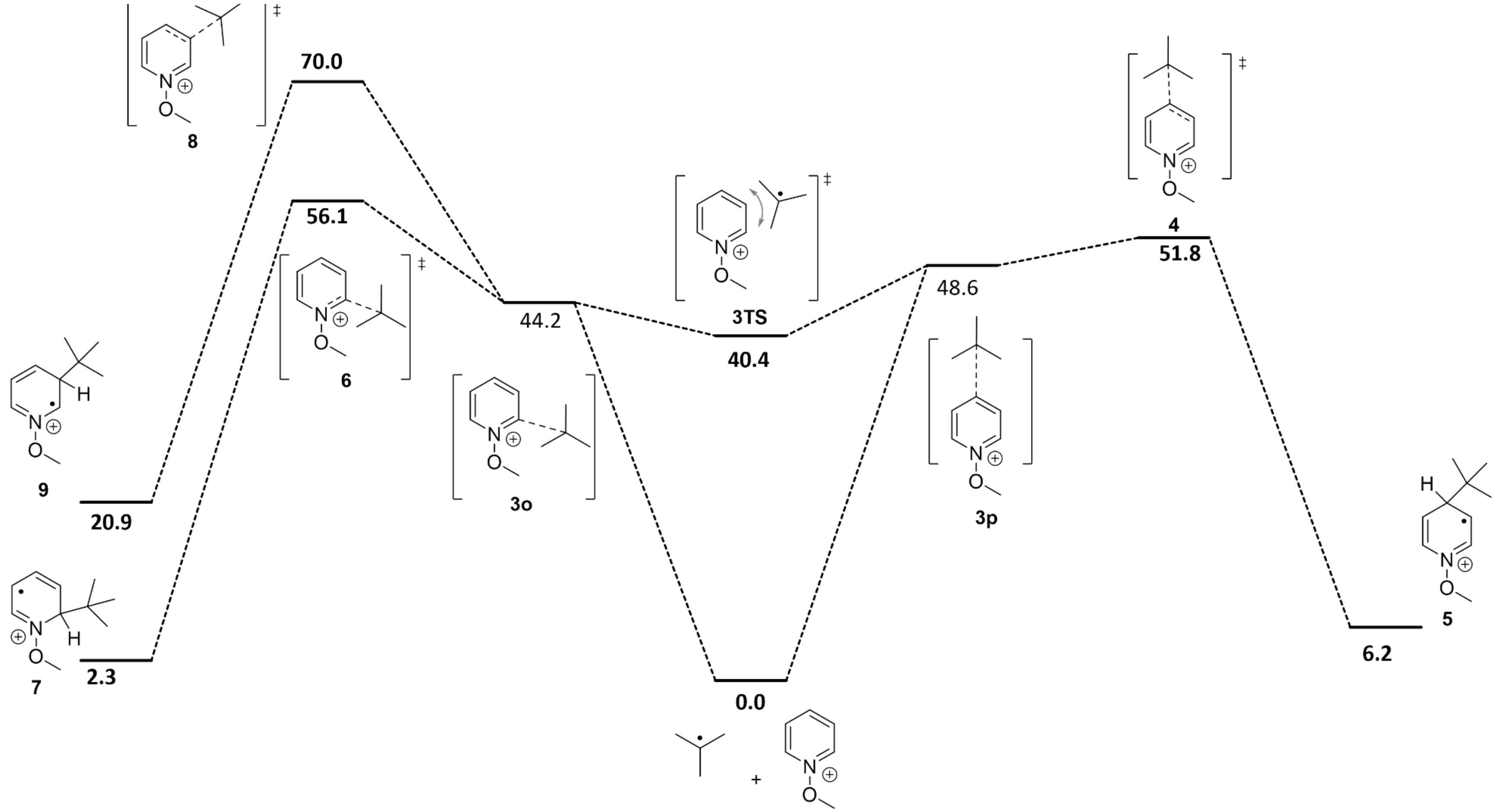

Figure S4. Reaction free energy profile in dichloromethane (DCM) for the reaction of *tert*-butyl radical (**1**) with 1-methoxypyridinium cation (**2**) calculated at the SMD(DCM)/DLPNO-CCSD(T)/cc-pVTZ//M06-2X/def2-TZVP level of theory. Single point solvation energies are calculated at the SMD(DCM)/UB3LYP/6-31+G(d)//M06-2X/def2-TZVP level of theory.

In order to compare the reactivities of various N-methoxypyridinium systems towards tBu radical addition, we compare here the barriers for addition to the C4 position in systems **2**, **20**, **30**, and **40** (Figure S5). For comparison, we also include **50** as a regioisomer of **30**, where the methyl substituent is attached to the C4 position (and radical attack therefore directed towards C2). Concentrating on the results obtained in dichloromethane solution (Figure S8) we see that the C4 addition barrier is largest for the 2,6-dimethyl-N-methoxypyridinium cation **20** ($\Delta G^{\ddagger}$ = +60.2 kJ/mol) followed by the N-methoxypyridinium system **2** or pyridinium cation **40** (both with $\Delta G^{\ddagger}$ = +52 kJ/mol), and finally the 2-methyl-N-methoxyquinolinium cation **30** ($\Delta G^{\ddagger}$ = +45.4 kJ/mol). C2-Addition to 4-methyl-N-methoxyquinolinium cation **50** faces an almost identical barrier ($\Delta G^{\ddagger}$ = +46.9 kJ/mol, Figure S12) as compared to the C4-addition to **30**, which implies that the radical addition barriers reflect (to some extend) the intrinsic electronic properties of the quinolinium system itself. Even for these latter two systems it is interesting to note that the radical addition reactions are essentially thermoneutral, which implies that the relative reactivities found experimentally will depend on the addition barriers as well as the rates of the subsequent deprotonation steps.

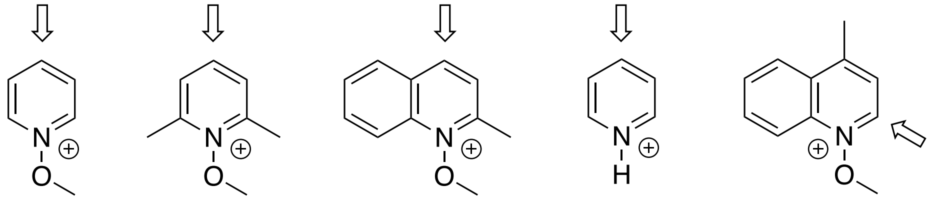

2 **20** **30** **40** **50**

Figure S5. Pyridinium systems **2**, **20**, **30**, **40** and **50**. Reaction parameters for the addition of *tert*-butyl radical **1** to the designated C4 positions have been calculated and collected in the following tables.

Table S3. Energies for the addition of *tert*-butyl radical (**1**) to the C4 positions of pyridinium systems **2**, **20**, **30**, **40**, and **50** calculated at different levels of theory.

**M06-2X/def2-TZVP (gas phase)**												
	**ΔE**				**ΔH**				**ΔG**			
	**Ref**	**RC**	**TS**	**P**	**Ref**	**RC**	**TS**	**P**	**Ref**	**RC**	**TS**	**P**
**2**	0.0	-41.3	-40.4	-73.5	0.0	-38.8	-36.8	-67.9	0.0	11.4	17.2	-9.6
**20**	0.0	-34.7	-30.4	-62.7	0.0	-29.9	-26.7	-55.2	0.0	19.4	33.5	5.4
**30**	0.0	-40.7	-40.1	-85.8	0.0	-34.5	-36.1	-75.5	0.0	16.4	19.3	-13.0
**40**	0.0	-45.9	-45.2	-70.4	0.0	-41.2	-44.3	-63.7	0.0	4.6	11.4	-5.3
**50**	0.0	-41.6	-39.7	-85.4	0.0	-36.1	-35.4	-74.5	0.0	13.3	22.3	-15.1
**DLPNO-CCSD(T)/cc-pVTZ//M06-2X/def2-TZVP (gas phase)**												

	ΔE				ΔH				ΔG			
	**Ref**	**RC**	**TS**	**P**	**Ref**	**RC**	**TS**	**P**	**Ref**	**RC**	**TS**	**P**
**2**	0.0	-25.5	-27.9	-73.4	0.0	-22.9	-24.4	-67.7	0.0	27.3	29.6	-9.3
**20**	0.0	-21.7	-19.7	-63.2	0.0	-17.2	-16.4	-55.7	0.0	32.4	43.2	4.9
**30**	0.0	-23.0	-26.3	-87.0	0.0	-16.9	-23.4	-76.7	0.0	33.3	31.2	-14.1
**40**	0.0	-30.5	-33.0	-70.2	0.0	-26.0	-32.3	-63.7	0.0	19.8	23.8	-5.2
**50**	0.0	-28.7	-29.6	-89.7	0.0	-23.2	-25.3	-78.9	0.0	26.2	32.4	-19.5
**SMD(DCM)/ M06-2X/def2-TZVP//M06-2X/def2-TZVP (DCM solution)** Single point solvation energies are calculated at the SMD(DCM)/UB3LYP/6-31+G(d)//M06-2X/def2-TZVP level of theory												
	**ΔE**				**ΔH**				**ΔG**			
	**Ref**	**RC**	**TS**	**P**	**Ref**	**RC**	**TS**	**P**	**Ref**	**RC**	**TS**	**P**
**2**	0.0	-22.5	-20.0	-58.1	0.0	-17.5	-16.3	-52.3	0.0	32.6	39.4	5.9
**20**	0.0	-20.9	-14.2	-51.4	0.0	-16.1	-10.5	-43.9	0.0	33.1	49.7	16.7
**30**	0.0	-28.3	-27.7	-75.1	0.0	-22.1	-23.7	-64.8	0.0	29.7	32.9	-2.2
**40**	0.0	-19.1	-16.8	-50.6	0.0	-13.9	-16.0	-44.0	0.0	31.9	39.8	14.4
**50**	0.0	-27.5	-25.2	-72.1	0.0	-22.0	-20.9	-61.3	0.0	26.9	36.8	-1.9
**SMD(DCM)/DLPNO-CCSD(T)/cc-pVTZ//M06-2X/def2-TZVP (DCM solution)** Single point solvation energies are calculated at the SMD(DCM)/UB3LYP/6-31+G(d)//M06-2X/def2-TZVP level of theory												
	**ΔE**				**ΔH**				**ΔG**			
	**Ref**	**RC**	**TS**	**P**	**Ref**	**RC**	**TS**	**P**	**Ref**	**RC**	**TS**	**P**
**2**	0.0	-6.0	-7.4	-57.8	0.0	-1.6	-3.7	-52.0	0.0	48.6	51.8	6.2
**20**	0.0	-7.9	-3.5	-51.9	0.0	-3.1	0.2	-44.4	0.0	46.1	60.2	16.2
**30**	0.0	-9.2	-14.0	-76.3	0.0	-3.1	-10.0	-66.0	0.0	47.2	45.4	-3.4
**40**	0.0	-3.4	-4.7	-50.6	0.0	1.3	-4.0	-44.0	0.0	47.1	52.2	14.4
**50**	0.0	-14.6	-15.1	-76.5	0.0	-9.1	-10.8	-65.7	0.0	38.1	46.9	-6.2

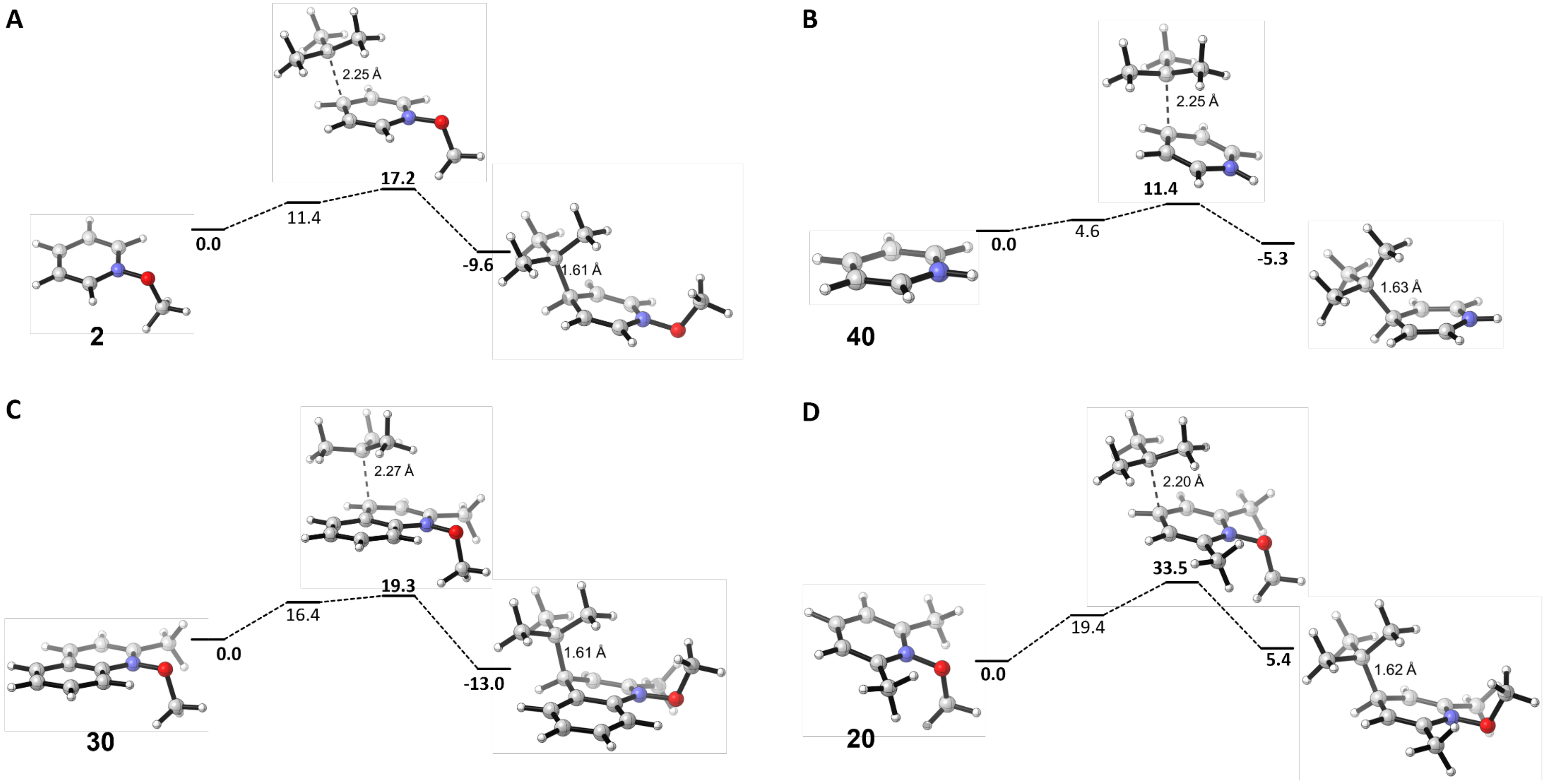

Figure S6. Gas phase reaction free energy profile for the addition of *tert*-butyl radical (**1**) to the C4 positions in pyridinium systems **2**, **20**, **30**, and **40** calculated at the M06-2X/def2-TZVP level of theory.

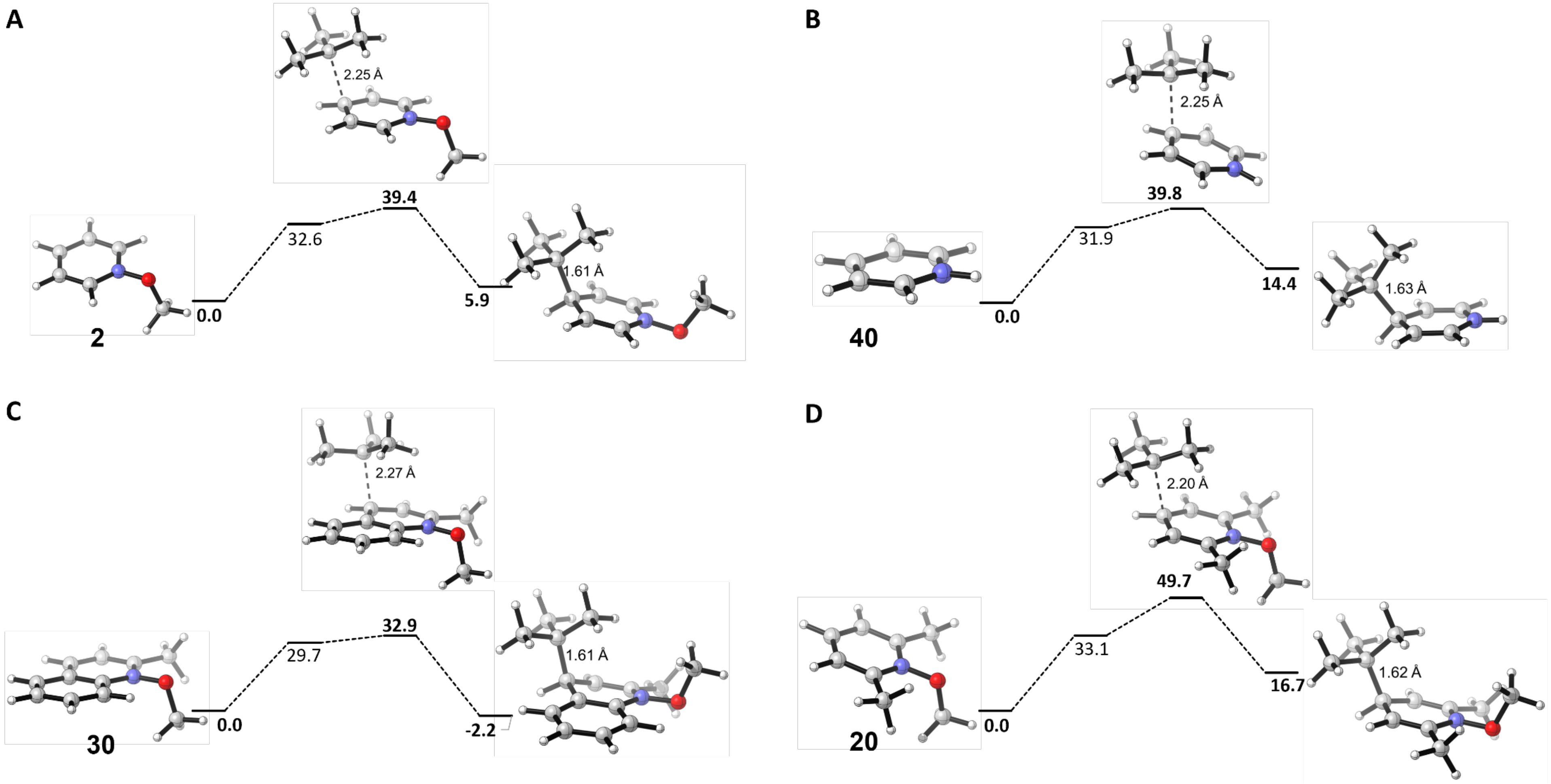

Figure S7. Reaction free energy profile for the addition of *tert*-butyl radical (**1**) to the C4 positions in pyridinium systems **2**, **20**, **30**, and **40** calculated at the SMD(DCM)/ M06-2X/def2-TZVP level of theory. Single point solvation energies are calculated at the SMD(DCM)/UB3LYP/6-31+G(d)//M06-2X/def2-TZVP level of theory.

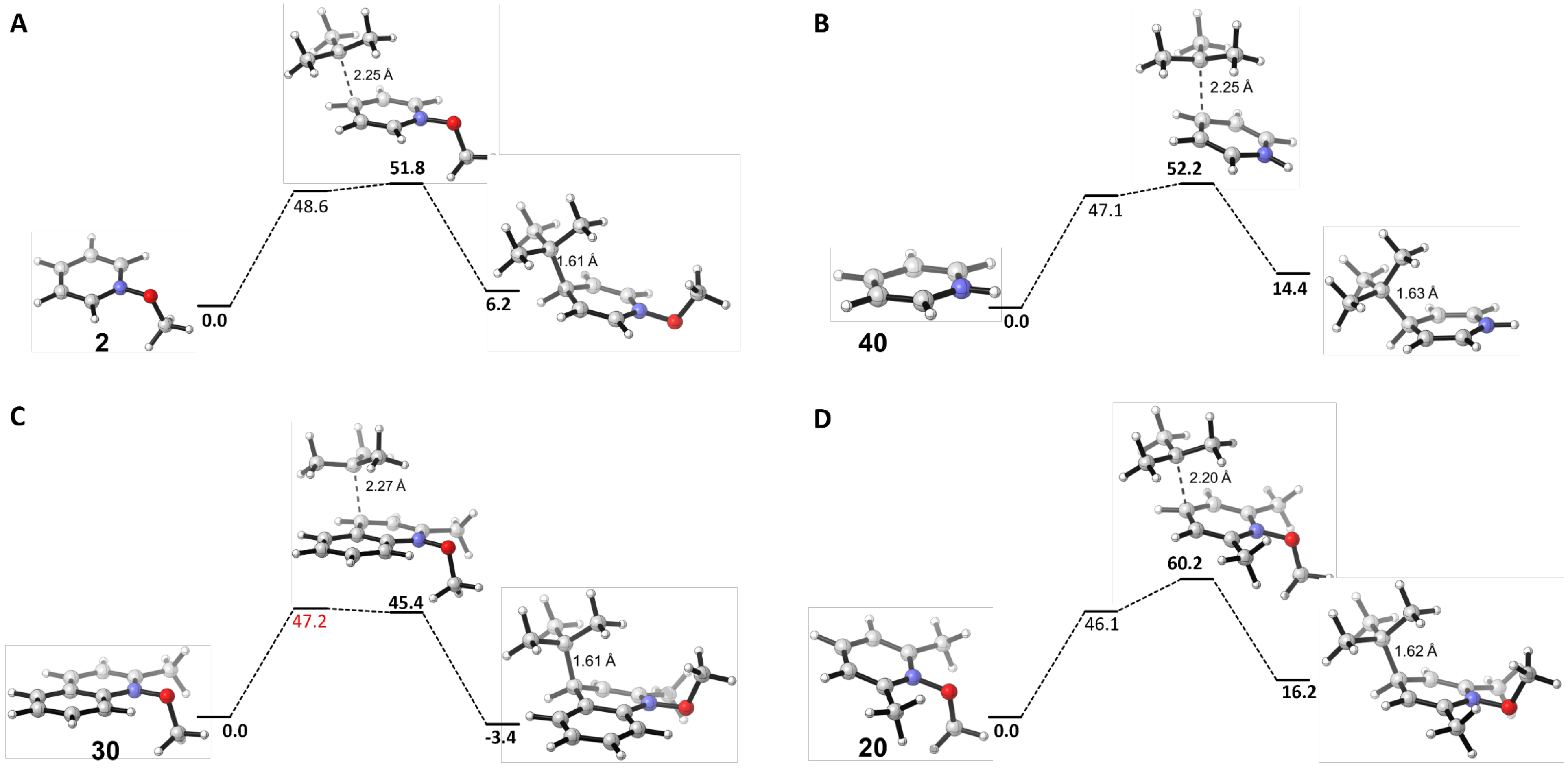

Figure S8. Reaction free energy profile for the addition of *tert*-butyl radical (**1**) to the C4 positions in pyridinium systems **2**, **20**, **30**, and **40** calculated at the SMD(DCM)/DLPNO-CCSD(T)/cc-pVTZ//M06-2X/def2-TZVP level of theory. Single point solvation energies are calculated at the SMD(DCM)/UB3LYP/6-31+G(d)//M06-2X/def2-TZVP level of theory.

Moving from tBu radical **1** to ethyl radical **100** leads to changes in reaction barriers as well as reaction energies. The discussion will again focus on the results obtained for DCM solution as described in Table S4 and Fig. S12. The lower stability of ethyl radical **100** as compared to tBu radical **1** leads to a notably higher reaction energy in the addition to **50** (ΔG_{sol}(**100**) = -32.5 kJ/mol vs. ΔG_{sol}(**1**) = -6.2 kJ/mol). That the reaction barrier for ethyl radical addition is nevertheless higher at $\Delta G^{\ddagger}_{sol}$(**100**) = +57.4 kJ/mol as compared to tBu radical addition at $\Delta G^{\ddagger}_{sol}$(**1**) = +46.9 kJ/mol may then be a reflection of polar effects in favour of the more nucleophilic tBu radical.[11] The addition of ethyl radical **100** to lepidinium cation **60** faces almost the same barrier as compared to reaction of the methoxylepidinium cation **50**, but shows, in comparison, a somewhat reduced reaction energy.

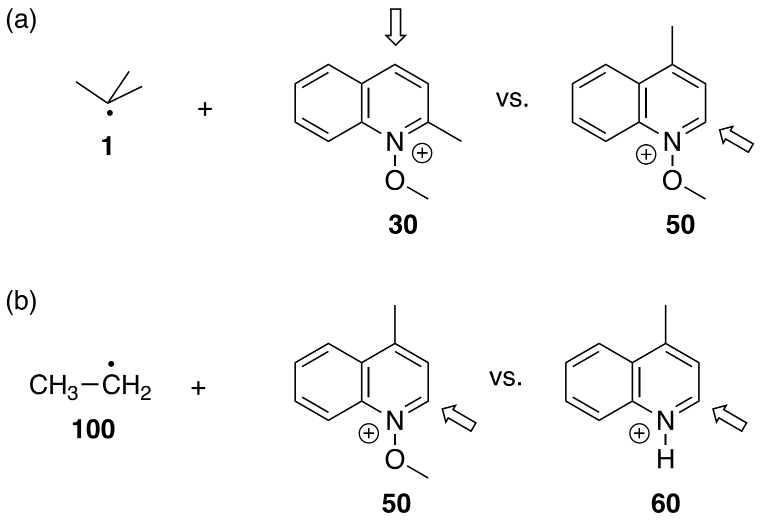

Figure S9. Addition of (a) *tert*-butyl radical **1** to the C4 position in **30** vs. the C2 position in **50**; and (b) ethyl radical **100** to the C2 positions in **50** and **60**.

Table S4. Energies for the addition of (a) *tert*-butyl radical **1** to the C4 position in **30** vs. the C2 position in **50**; and (b) ethyl radical **100** to the C2 positions in **50** and **60**.

**M06-2X/def2-TZVP (gas phase)**												
	**ΔE**				**ΔH**				**ΔG**			
	**Ref**	**RC**	**TS**	**P**	**Ref**	**RC**	**TS**	**P**	**Ref**	**RC**	**TS**	**P**
**30 + 1**	0.0	-40.7	-40.1	-85.8	0.0	-34.5	-36.1	-75.5	0.0	16.4	19.3	-13.0
**50 + 1**	0.0	-41.6	-39.7	-85.4	0.0	-36.1	-35.4	-74.5	0.0	13.3	22.3	-15.1
**50 + 100**	0.0	-30.6	-19.4	-106.4	0.0	-23.3	-13.2	-91.9	0.0	16.4	39.7	-38.5
**60 + 100**	0.0	-23.9	-13.1	-97.2	0.0	-17.6	-9.0	-83.9	0.0	17.3	41.6	-30.1

**DLPNO-CCSD(T)/cc-pVTZ//M06-2X/def2-TZVP (gas phase)**												
	**ΔE**				**ΔH**				**ΔG**			
	**Ref**	**RC**	**TS**	**P**	**Ref**	**RC**	**TS**	**P**	**Ref**	**RC**	**TS**	**P**
**30 + 1**	0.0	-23.0	-26.3	-87.0	0.0	-16.9	-23.4	-76.7	0.0	33.3	31.2	-14.1
**50 + 1**	0.0	-28.7	-29.6	-89.7	0.0	-23.2	-25.3	-78.9	0.0	26.2	32.4	-19.5
**50 + 100**	0.0	-15.6	-7.0	-103.8	0.0	-9.9	-0.8	-89.3	0.0	27.4	51.9	-35.9
**60 + 100**	0.0	-14.1	-0.9	-94.9	0.0	-7.8	3.2	-81.6	0.0	25.9	53.7	-27.8
**SMD(DCM)/M06-2X/def2-TZVP//M06-2X/def2-TZVP (DCM solution)** Single point solvation energies are calculated at the SMD(DCM)/UB3LYP/6-31+G(d)//M06-2X/def2-TZVP level of theory												
	**ΔE**				**ΔH**				**ΔG**			
	**Ref**	**RC**	**TS**	**P**	**Ref**	**RC**	**TS**	**P**	**Ref**	**RC**	**TS**	**P**
**30 + 1**	0.0	-28.3	-27.7	-75.1	0.0	-22.1	-23.7	-64.8	0.0	29.7	32.9	-2.2
**50 + 1**	0.0	-27.5	-25.2	-72.1	0.0	-22.0	-20.9	-61.3	0.0	26.9	36.8	-1.9
**50 + 100**	0.0	-29.0	-13.7	-102.9	0.0	-21.7	-7.6	-88.4	0.0	20.1	45.4	-35.1
**60 + 100**	0.0	-20.5	-7.0	-92.2	0.0	-14.2	-2.9	-79.0	0.0	20.6	47.5	-25.1
**SMD(DCM)/DLPNO-CCSD(T)/cc-pVTZ//M06-2X/def2-TZVP (DCM solution)** Single point solvation energies are calculated at the SMD(DCM)/UB3LYP/6-31+G(d)//M06-2X/def2-TZVP level of theory												
	**ΔE**				**ΔH**				**ΔG**			
	**Ref**	**RC**	**TS**	**P**	**Ref**	**RC**	**TS**	**P**	**Ref**	**RC**	**TS**	**P**
**30 + 1**	0.0	-9.2	-14.0	-76.3	0.0	-3.1	-10.0	-66.0	0.0	47.2	45.4	-3.4
**50 + 1**	0.0	-14.6	-15.1	-76.5	0.0	-9.1	-10.8	-65.7	0.0	38.1	46.9	-6.2
**50 + 100**	0.0	-13.4	-1.3	-100.3	0.0	-6.2	4.9	-85.8	0.0	31.1	57.4	-32.5
**60 + 100**	0.0	-10.7	5.1	-89.9	0.0	-4.4	9.2	-76.7	0.0	29.2	58.8	-22.8

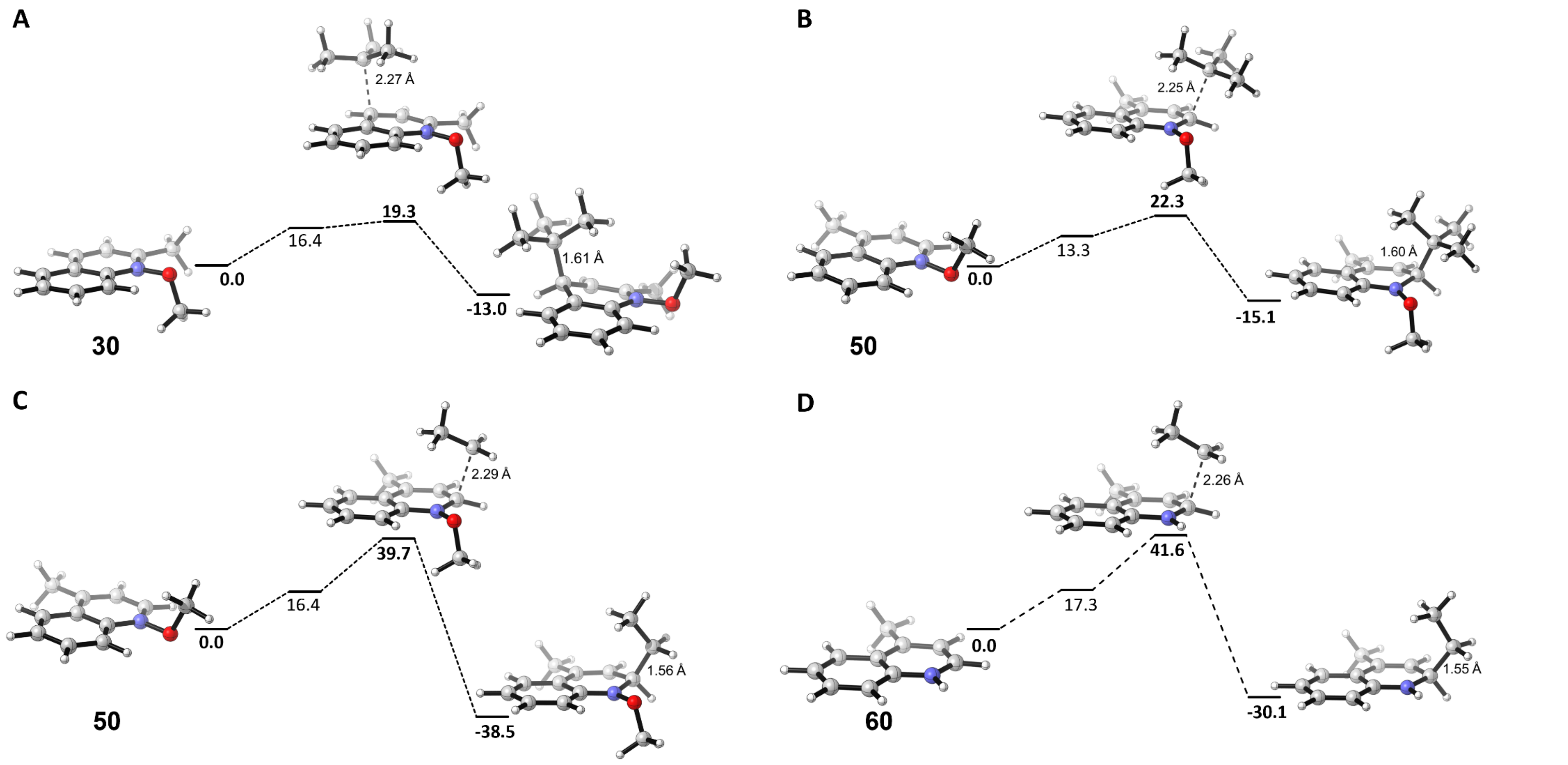

Figure S10. Gas phase reaction free energy profile (ΔG_{298}, kJ/mol) for the addition of *tert*-butyl (**1**, **A** & **B**) and ethyl (**100**, **C** & **D**) radical to pyridinium systems **30**, **50**, and **60** calculated at the M06-2X/def2-TZVP level of theory.

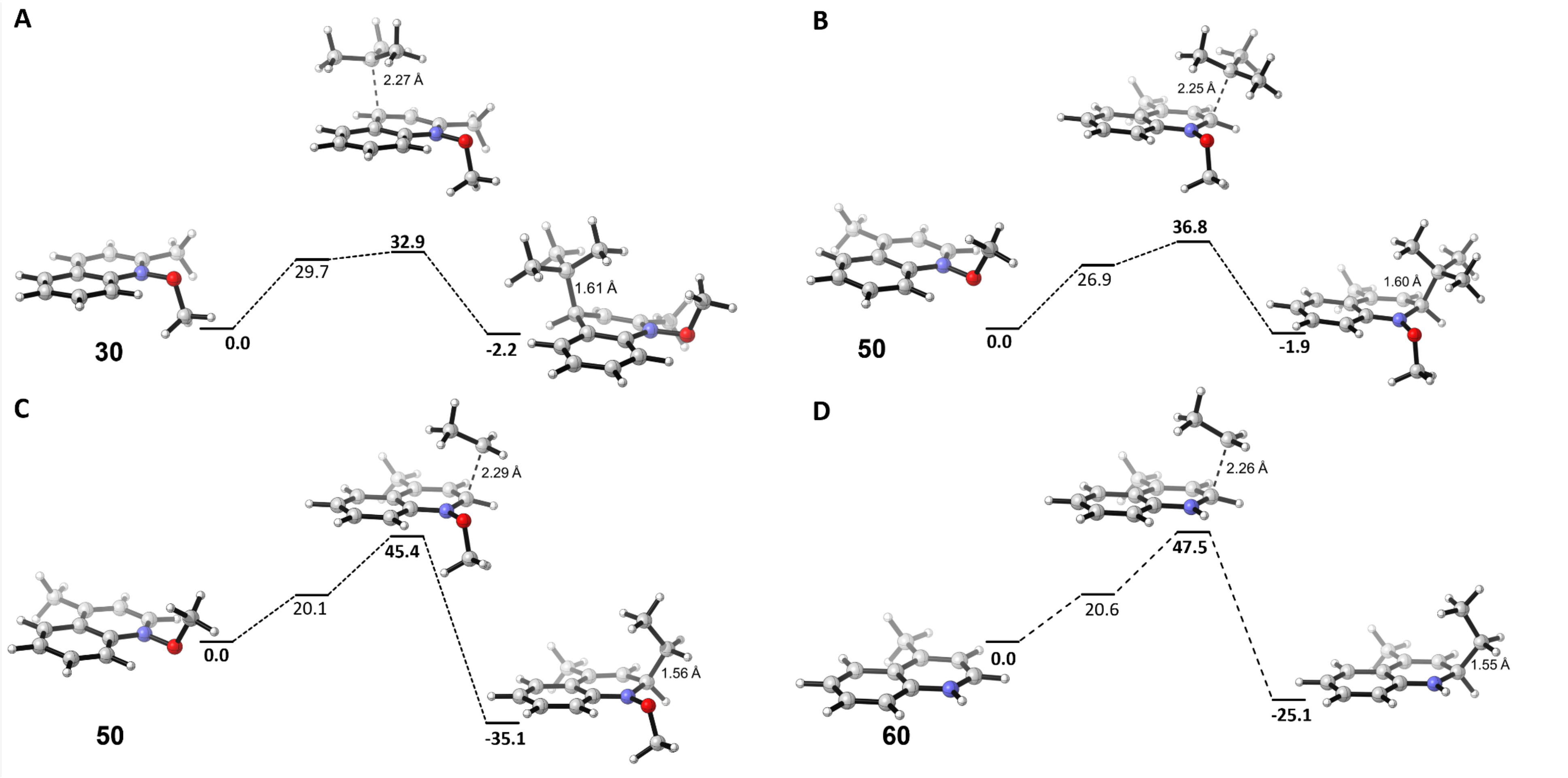

Figure S11. Reaction free energy profile (ΔG_{sol}, kJ/mol) for the addition of *tert*-butyl (**1**, **A** & **B**) and ethyl (**100**, **C** & **D**) radical to pyridinium systems **30**, **50**, and **60** calculated at the SMD(DCM)/M06-2X/def2-TZVP level of theory. Single point solvation energies are calculated at the SMD(DCM)/UB3LYP/6-31+G(d)//M06-2X/def2-TZVP level of theory.

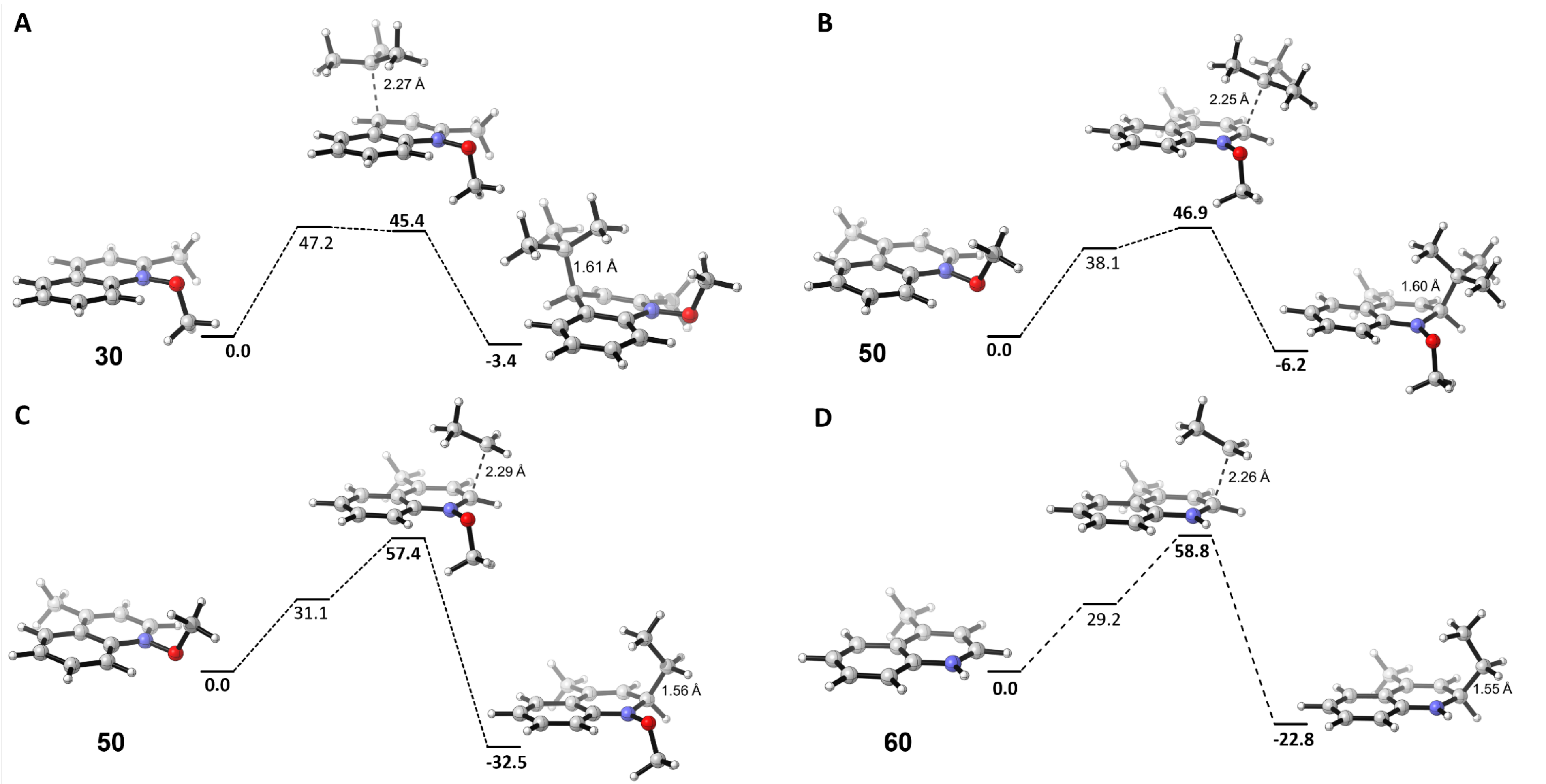

Figure S12. Reaction free energy profile (ΔG_{sol}, kJ/mol) for the addition of *tert*-butyl (**1**, **A** & **B**) and ethyl (**100**, **C** & **D**) radical to pyridinium systems **30**, **50**, and **60** calculated at the SMD(DCM)/DLPNO-CCSD(T)/cc-pVTZ//M06-2X/def2-TZVP level of theory. Single point solvation energies are calculated at the SMD(DCM)/UB3LYP/6-31+G(d)//M06-2X/def2-TZVP level of theory.

Table S5. Energies for the systems shown in Fig. S1.

**system**	E_{tot} **(U)M06-2X/ def2-TZVP**	H_{298} **(U)M06-2X/ def2-TZVP**	G_{298} **(U)M06-2X/ def2-TZVP**	**q(tBu,Mull)**	**SD(tBu,Mull)**
**1**					
r1_1	**-157.7593473**	-157.634902	**-157.671651**		
mepy_004 (C_{3v})	-157.7593439	**-157.635016**	-157.671001	+0.00	1.00
**2**					
mepy_001 (Cs)	**-363.0983257**	-362.955434	-362.994563		
r2_1	-363.0982671	**-362.955621**	**-362.995128**		
mepy_002	-363.0958445	-362.953864 imag -40 cm^{-1}	-362.990642		
**3p**					
mepy_009	**-520.8734071**	-520.604277	**-520.662438**	+0.25	0.71
ts_r1r2_3_ircf_o	-520.8734071	-520.604277	-520.662442		
ts_r1r2_2_ircf_o	-520.8733733	-520.604030	-520.660953		
ts_r1r2_4_ircr_o	-520.8733617	**-520.605407**	-520.660883		
**3TS**					
mepy_016	**-520.8688479**	**-520.600610** imag -36 cm^{-1}	-520.658571		
mepy_025	-520.8688472	-520.600609 imag -36 cm^{-1}	**-520.658588**		
**3o**					
mepy_015	-520.8733678	-520.604187	-520.662334	+0.19	0.81
**4**					
mepy_018	**-520.8730454**	-520.604578 imag -201 cm^{-1}	-520.659661		
ts_r1r2_3	-520.8730454	-520.604580	-520.659662		
ts_r1r2_4	-520.8729737	**-520.604655**	**-520.660236**		
ts_r1r2_2	-520.8728307	-520.604442	-520.659068		
**5**					
ts_r1r2_2_ircr_o	**-520.8856630**	-520.615330	-520.669871		
mepy_032	-520.8856629	**-520.615439**	**-520.670438**		

ts_r1r2_4_ircf_o	-520.8855016	-520.615041	-520.668589		
ts_r1r2_3_ircr_o	-520.8854127	-520.615330	-520.669871		
mepy_006	-520.8854127	-520.615330	-520.669872		
mepy_007	-520.8849925	-520.614300	-520.668019		
**6**					
mepy_012	-520.8723173	-520.603823<br>imag -256 cm^{-1}	-520.657879		
**7**					
mepy_010	**-520.8906408**	**-520.619198**	-520.670457		
mepy_011	-520.8890645	-520.617749	**-520.670997**		
**8**					
mepy_022	-520.8631483	-520.594720<br>imag -380 cm^{-1}	-520.648979		
**9**					
mepy_019	**-520.8793675**	-520.608933	-520.661907		
mepy_035	-520.8793581	**-520.609250**	**-520.662657**		
**proton transfer pathways from adduct 5**					
**TS5_12**					
mepy_029	-520.8285999	**-520.563550**<br>im: -1597 cm^{-1}	-520.616341		
mepy_024	-520.8283017	-520.563538<br>im: -1626 cm^{-1}	**-520.616701**		
**12**					
mepy_029r	-520.8829582	**-520.612779**	**-520.666297**		
mepy_024f	-520.8828627	-520.612767	-520.666264		
**TS12_13**					
mepy_033	-520.8266284	-520.561495<br>im: -1609 cm^{-1}	-520.614358		
**13**					
mepy_020	-520.8897724	-520.618974	-520.672837		
**TS13_14**					
mepy_034	-520.7898070	-520.524380	-520.578038		

		im: -2234 cm^{-1}			
**14**					
mepy_030	-520.8634611	**-520.592217**	**-520.645623**		
mepy_027	-520.8595017	-520.588360	-520.642172		
**proton transfer pathways from adduct 7**					
**TS7_15**					
mepy_036	-520.7906180	-520.524955<br>im: -2163 cm^{-1}	-520.577589		
**15**					
mepy_031	-520.8667255	**-520.595296**	**-520.647243**		
mepy_028	-520.8620233	-520.591189	-520.643965		
**16**					
mepy_023	-520.8912716	-520.620004	-520.672456		
**17**					
mepy_038	-520.8764808	-520.606052	-520.658073		
**18**					
mepy_021	-520.8859861	-520.615723	-520.667598		
**19**					
mepy_039	-520.8819489	-520.611205	-520.662791		

Table S6. QM data (in Hartree) calculated at M06-2X/def2-TZVP level. Single point solvation energies (in kcal/mol) are calculated at SMD(DCM)/UB3LYP/6-31+G(d)//M06-2X/def2-TZVP level. "Type" refers to R=Reactant, RC=Reactant Complex, TS=Transition State, PC=Product Complex, and P=Product.

			**M06-2X/def2-TZVP**										
**Parent**	**Type**	**FileName**	**Δ*E*$_{tot}$**	**HOMO**	**LUMO**	**Low Frequency**			**corr. ZPE**	**corr. δH**	**corr. δG**	**corr. qh-δG**	**ΔG$_{solv}$ (kcal/mol)**
Reaction of *tert*-butyl radical (**1**) with 1-methoxypyridinium cation (**2**)													
**1**	R	r1_1	-157.7593473	-0.22344	0.06847	-17	0	0	0.117137	0.124445	0.087696	0.087695	-2.94
		mepy_004	-157.7593439	-0.22347	0.06847	-19	-16	-3	0.116945	0.124328	0.088343	0.088344	-2.94
**2**	R	r2_1	-363.0982671	-0.52939	-0.21726	-31	-8	0	0.134708	0.142646	0.103139	0.103442	-54.50
		mepy_001	-363.0983257	-0.52938	-0.21724	0	0	0	0.135085	0.142891	0.103763	0.104014	-54.45
**3**	TS	mepy_025	-520.8688474	-0.33793	-0.20575	-36	-11	0	0.253185	0.268235	0.210257	0.213146	-52.93
	TS	mepy_016	-520.8688479	-0.33793	-0.20576	-36	-11	0	0.253190	0.268238	0.210277	0.213152	-52.93
**4**	RC	mepy_009	-520.8734071	-0.35480	-0.18241	-22	-11	0	0.253701	0.269131	0.210969	0.213518	-50.47
	RC	ts_r1r2_3_ircf_o	-520.8734071	-0.35481	-0.18241	-22	-11	0	0.253700	0.269130	0.210965	0.213518	-50.46
	RC	ts_r1r2_4_ircr_o	-520.8733617	-0.35469	-0.18257	-33	-18	0	0.253273	0.267955	0.212479	0.213571	-50.45
	RC	ts_r1r2_2_ircf_o	-520.8733733	-0.35744	-0.18469	0	0	0	0.254150	0.269343	0.212420	0.214194	-51.03
	TS	ts_r1r2_4	-520.8729737	-0.37030	-0.16161	-208	-31	-15	0.253897	0.268319	0.212738	0.215076	-50.24
	TS	ts_r1r2_3	-520.8730454	-0.37018	-0.16185	-200	-17	-6	0.254143	0.268465	0.213383	0.215439	-50.35
	TS	ts_r1r2_2	-520.8728307	-0.37344	-0.16221	-221	-16	-9	0.254161	0.268389	0.213763	0.215637	-50.78
	PC	ts_r1r2_2_ircr_o	-520.8856630	-0.43414	-0.16066	-23	0	0	0.256236	0.270224	0.215221	0.218446	-51.83
	PC	ts_r1r2_3_ircr_o	-520.8854127	-0.43364	-0.16115	-30	-11	0	0.256057	0.270083	0.215542	0.218151	-51.69
	PC	p_r1r2_1	-520.8856629	-0.43415	-0.16067	-42	-24	0	0.255932	0.269147	0.216354	0.218836	-51.80
	PC	ts_r1r2_4_ircf_o	-520.8855016	-0.43342	-0.16111	-20	0	0	0.256616	0.270461	0.216913	0.218788	-51.78
	PC	mepy_007	-520.8849925	-0.42505	-0.15621	-11	0	0	0.256925	0.270692	0.216973	0.219370	-51.58
**6**	RC	mepy_015	-520.8733678	-0.35096	-0.19287	-13	-12	0	0.253670	0.269181	0.211034	0.213168	-50.77

	RC	mepy_016f	-520.8733677	-0.35096	-0.19287	-13	-12	0	0.253670	0.269181	0.211035	0.213168	-50.77
	TS	mepy_012	-520.8723172	-0.37049	-0.17644	-258	-15	0	0.254371	0.268494	0.214438	0.215841	-50.21
	PC	mepy_011	-520.8890645	-0.41955	-0.17524	-21	0	0	0.257670	0.271315	0.218067	0.220250	-51.96
	PC	mepy_010	-520.8906408	-0.42561	-0.18012	-21	0	0	0.258117	0.271443	0.220184	0.220670	-51.71
**8**	TS	mepy_022	-520.8631483	-0.36046	-0.18814	-380	-16	-5	0.254270	0.268428	0.214167	0.215670	-50.80
	PC	mepy_035	-520.8793581	-0.40743	-0.19595	-8	0	0	0.256225	0.270108	0.216701	0.218107	-52.44
	PC	mepy_019	-520.8793675	-0.40672	-0.19470	-15	0	0	0.256671	0.270435	0.217460	0.218688	-52.18
Reaction of *tert*-butyl radical (**1**) to the **C4** positions of pyridinium systems **20**, **30**, **40**													
**20**	R	r20_1	-441.7371107	-0.49030	-0.19834	-22	0	0	0.190691	0.201806	0.154865	0.155904	-51.56
**30**	R	r30_1	-556.0631232	-0.45167	-0.21054	-8	0	0	0.210134	0.222023	0.173864	0.173863	-50.99
**40**	R	r40_1	-248.6300054	-0.53448	-0.22477	-14	0	0	0.103646	0.108921	0.076178	0.076178	-59.03
**20 + 1**	RC	ts_r1r20_1_ircf_o	-599.5096620	-0.34344	-0.17748	-14	0	0	0.309271	0.327964	0.263153	0.265640	-49.32
	RC	ts_r1r20_3_ircr_o	-599.5091122	-0.33924	-0.17747	-10	0	0	0.309975	0.327673	0.265749	0.267204	-48.98
	TS	ts_r1r20_3	-599.5069115	-0.36350	-0.14290	-287	-17	0	0.310278	0.327585	0.265757	0.268685	-48.54
	TS	ts_r1r20_1	-599.5080186	-0.36522	-0.14561	-279	-12	-6	0.310216	0.327524	0.266877	0.268333	-48.74
	TS	ts_r1r20_4	-599.5069116	-0.36350	-0.14289	-287	-20	0	0.310253	0.326641	0.268626	0.269371	-48.54
	P	ts_r1r20_1_ircr_o	-599.5203444	-0.41899	-0.13965	-18	-8	0	0.311964	0.328988	0.268495	0.270864	-49.90
	P	ts_r1r20_3_ircf_o	-599.5190867	-0.41893	-0.14216	-20	0	0	0.312012	0.329165	0.268593	0.270468	-49.70
**30 + 1**	RC	ts_r1r30_2_ircr_o	-713.8376896	-0.34219	-0.18657	-16	0	0	0.329426	0.348665	0.283022	0.285181	-48.73
	RC	ts_r1r30_1_ircf_o	-713.8379834	-0.34607	-0.18614	-13	-8	0	0.329567	0.348727	0.283665	0.285419	-49.07
	TS	ts_r1r30_2	-713.8369102	-0.35836	-0.16425	-238	-23	-17	0.328864	0.347338	0.283358	0.285496	-48.63
	TS	ts_r1r30_1	-713.8377267	-0.35830	-0.16892	-172	-19	0	0.329599	0.347871	0.284652	0.286641	-49.09
	P	ts_r1r30_1_ircr_o	-713.8551585	-0.41336	-0.13841	-13	-2	0	0.332613	0.350277	0.289314	0.290285	-49.47
	P	ts_r1r30_2_ircf_o	-713.8524937	-0.41093	-0.14076	-23	-15	0	0.332317	0.350017	0.288666	0.290110	-49.42
**40 + 1**	RC	ts_r1r40_4_ircr_o	-406.4067580	-0.36365	-0.18140	-37	-21	0	0.222196	0.234956	0.183023	0.185076	-53.54

	RC	ts_r1r40_2_ircr_o	-406.4068303	-0.36267	-0.18290	-5	0	0	0.222889	0.235415	0.184914	0.186057	-53.67
	TS	ts_r1r40_4	-406.4065663	-0.37618	-0.16422	-179	-32	-16	0.222549	0.234284	0.185432	0.186771	-53.30
	TS	ts_r1r40_2	-406.4065022	-0.37711	-0.16304	-198	-36	-23	0.222693	0.233515	0.187625	0.187740	-53.31
	P	ts_r1r40_2_ircf_o	-406.4161211	-0.44061	-0.16367	-33	-1	0	0.224512	0.235758	0.188630	0.189696	-55.37
	P	ts_r1r40_4_ircf_o	-406.4161662	-0.44038	-0.16358	-18	0	0	0.224797	0.236023	0.188915	0.190007	-55.35
Reaction of *tert*-butyl radical (**1**) and ethyl radical (**100**) to the C2 positions of pyridinium systems **50** and **60**													
**100**	R	r100_1	-79.1345877	-0.25636	0.08722	-99	-48	0	0.059389	0.064379	0.035033	0.035126	-1.24
**50**	R	r50_1	-556.0606283	-0.45177	-0.21227	-11	-8	0	0.210049	0.221961	0.173629	0.173747	-51.10
**60**	R	r60_1	-441.5934611	-0.45688	-0.21589	-26	0	0	0.178463	0.187762	0.145415	0.145415	-55.13
**50 + 1**	RC	ts_r1r50_2_ircr_o	-713.8358208	-0.34423	-0.18700	-20	-11	0	0.328921	0.348377	0.282231	0.284317	-48.77
	RC	ts_r1r50_1_ircr_o	-713.8324836	-0.34320	-0.20126	-17	-10	0	0.329438	0.349083	0.281041	0.284842	-50.25
	TS	ts_r1r50_2	-713.8350855	-0.35996	-0.16957	-224	-18	0	0.329661	0.347903	0.284930	0.286693	-48.68
	P	p_r1r50_2	-713.8524881	-0.40697	-0.16157	-12	-5	0	0.332532	0.350411	0.288085	0.290339	-48.98
	P	p_r1r50_1	-713.8433546	-0.40705	-0.16281	-16	0	0	0.333621	0.351107	0.290671	0.291828	-49.63
**50 + 100**	RC	ts_r100r50_3_ircr_o	-635.2068690	-0.38370	-0.20794	-6	0	0	0.272181	0.289100	0.228354	0.230140	-50.06
	RC	ts_r100r50_2_ircr_o	-635.2054857	-0.38350	-0.20755	-18	-11	0	0.272297	0.289271	0.227937	0.230102	-50.14
	RC	ts_r100r50_4_ircr_o	-635.2053406	-0.37178	-0.20317	-18	-8	-5	0.271229	0.288528	0.225286	0.228806	-49.57
	RC	ts_r100r50_7_ircr_o	-635.2053404	-0.37178	-0.20317	-19	-10	-5	0.271204	0.288521	0.225041	0.228762	-49.57
	RC	ts_r100r50_1_ircr_o	-635.2050113	-0.38139	-0.20466	-7	0	0	0.271483	0.288720	0.226605	0.228881	-50.11
	RC	ts_r100r50_5_ircr_o	-635.2037309	-0.37104	-0.20329	-5	0	0	0.272004	0.289210	0.225946	0.229527	-49.90
	TS	ts_r100r50_4	-635.2026032	-0.38045	-0.18055	-267	-5	0	0.273072	0.288687	0.231183	0.232326	-49.09
	TS	ts_r100r50_5	-635.2011124	-0.38044	-0.18116	-252	-8	0	0.272620	0.288474	0.229898	0.231408	-49.15
	TS	ts_r100r50_7	-635.2005458	-0.38018	-0.17935	-267	-14	-4	0.272517	0.288324	0.230211	0.231386	-49.16
	TS	ts_r100r50_1	-635.1981401	-0.38673	-0.17671	-366	-15	-5	0.272954	0.288432	0.231725	0.232460	-50.27
	TS	ts_r100r50_3	-635.1961246	-0.38789	-0.17669	-393	-14	-7	0.272962	0.288659	0.230244	0.232193	-50.37
	TS	ts_r100r50_2	-635.1954737	-0.38658	-0.17449	-407	-17	-11	0.272940	0.288564	0.230943	0.232267	-50.45
	P	ts_r100r50_4_ircf_o	-635.2357229	-0.41133	-0.16563	-17	-13	0	0.276528	0.291863	0.234499	0.236391	-49.63

	P	ts_r100r50_5_ircf_o	-635.2343811	-0.41103	-0.16512	-14	0	0	0.276441	0.291808	0.234888	0.235943	-49.66
	P	ts_r100r50_1_ircf_o	-635.2331943	-0.41254	-0.16677	-6	0	0	0.277064	0.292033	0.236563	0.237351	-50.05
	P	ts_r100r50_7_ircf_o	-635.2326328	-0.41175	-0.16450	-18	-14	0	0.276276	0.291593	0.234662	0.235980	-49.76
	P	ts_r100r50_3_ircf_o	-635.2316375	-0.41209	-0.16680	-14	-8	0	0.276838	0.292060	0.235566	0.236605	-49.64
	P	ts_r100r50_2_ircf_o	-635.2292015	-0.41257	-0.16628	-18	-14	0	0.276864	0.291983	0.235936	0.236973	-50.04
	P	p_r100r50_8	-635.2224751	-0.41540	-0.15931	-7	0	0	0.277087	0.292089	0.236184	0.237331	-49.92
**60 + 100**	RC	ts_r100r60_3_ircr_o	-520.7371425	-0.37855	-0.20661	-6	0	0	0.239970	0.254536	0.198103	0.200645	-53.66
	RC	ts_r100r60_1_ircr_o	-520.7361787	-0.37669	-0.20654	-17	-12	-4	0.239587	0.254402	0.195175	0.200013	-53.70
	TS	ts_r100r60_3	-520.7330303	-0.38655	-0.18070	-317	-22	-11	0.240553	0.253703	0.201272	0.202671	-53.03
	TS	ts_r100r60_2	-520.7321922	-0.38795	-0.17968	-322	-13	-7	0.240798	0.254009	0.200725	0.202881	-53.24
	TS	ts_r100r60_1	-520.7318833	-0.38678	-0.18130	-307	-10	0	0.240852	0.254038	0.201514	0.202755	-53.18
	P	ts_r100r60_3_ircf_o	-520.7650694	-0.41516	-0.16969	-16	-12	0	0.244414	0.257192	0.206013	0.206957	-53.29
	P	ts_r100r60_2_ircf_o	-520.7647785	-0.41433	-0.16887	-14	0	0	0.245012	0.257619	0.206930	0.207619	-53.11
	P	ts_r100r60_1_ircf_o	-520.7642865	-0.41445	-0.16938	-11	-7	0	0.244671	0.257375	0.206436	0.207187	-53.17

Table S7. QM data calculated at DLPNO-CCSD(T) level with cc-pVTZ and, in part, cc-pVQZ basis sets and extrapolated to CBS limit. RefE = Reference Energy, FCE = Final Correlation Energy, FSPE = Final Single Point Energy.

			DLPNO-CCSD(T)/cc-pVTZ			DLPNO-CCSD(T)/cc-pVQZ			DLPNO-CCSD(T)/CBS
**Parent**	**Type**	**FileName**	**RefE**	**FCE**	**FSPE**	**RefE**	**FCE**	**FSPE**	
**1**	R	r1_1	-156.7260801	-0.76396	-157.4900366	-156.7360924	-0.80059	-157.5366863	-157.5657849
		mepy_004	-156.7260840	-0.76395	-157.4900337	-156.7360962	-0.80059	-157.5366847	-157.5657841
**2**	R	r2_1	-361.0173628	-1.47802	-362.4953872	-361.0406112	-1.55918	-362.5997888	-362.6645658
		mepy_001	-361.0174061	-1.47797	-362.4953779	-361.0406565	-1.55912	-362.5997784	-362.6645538
**3**	TS	mepy_025	-517.7388093	-2.25484	-519.9936444	-517.7718743	-2.37250	-520.1443759	-520.2381041
	TS	mepy_016	-517.7388062	-2.25484	-519.9936440	-517.7724887	-2.37267	-520.1451552	-520.2391848
**4**	RC	mepy_009	-517.7243139	-2.27083	-519.9951411	-517.7573366	-2.38881	-520.1461487	-520.2400909
	RC	ts_r1r2_3_ircf_o	-517.7243100	-2.27083	-519.9951403	-517.7573328	-2.38882	-520.1461501	-520.2400937
	RC	ts_r1r2_4_ircr_o	-517.7243915	-2.27074	-519.9951324	-517.7574139	-2.38873	-520.1461411	-520.2400840
	RC	ts_r1r2_2_ircf_o	-517.7242272	-2.27068	-519.9949100	-517.7572242	-2.38868	-520.1459006	-520.2398412
	TS	ts_r1r2_4	-517.7112274	-2.28483	-519.9960533	-517.7442116	-2.40289	-520.1471005	-520.2410866
	TS	ts_r1r2_3	-517.7112774	-2.28476	-519.9960369	-517.7442611	-2.40282	-520.1470831	-520.2410686
	TS	ts_r1r2_2	-517.7100123	-2.28583	-519.9958461	-517.7429671	-2.40393	-520.1468961	-520.2408963
	PC	ts_r1r2_2_ircr_o	-517.7260646	-2.28729	-520.0133510	-517.7590450	-2.40521	-520.1642589	-520.2581475
	PC	ts_r1r2_3_ircr_o	-517.7263080	-2.28686	-520.0131675	-517.7593041	-2.40477	-520.1640749	-520.2579566
	PC	p_r1r2_1	-517.7260769	-2.28730	-520.0133812	-517.7590571	-2.40521	-520.1642660	-520.2581382
	PC	ts_r1r2_4_ircf_o	-517.7261863	-2.28697	-520.0131540	-517.7591823	-2.40488	-520.1640590	-520.2579390
	PC	mepy_007	-517.7233315	-2.28936	-520.0126879	-517.7564922	-2.40718	-520.1636745	-520.2575450
**6**	RC	mepy_015	-517.7312598	-2.26514	-519.9963958	-517.7640954	-2.38312	-520.1472165	-520.2411023
	RC	mepy_016f	-517.7312646	-2.26513	-519.9963954	-517.7641006	-2.38312	-520.1472169	-520.2411032
	TS	mepy_012	-517.7092936	-2.28687	-519.9961609	-	-	-	-
	PC	mepy_011	-517.7242273	-2.29325	-520.0174786	-	-	-	-

	PC	mepy_010	-517.7301465	-2.28808	-520.0182258	-	-	-	-
**8**	TS	mepy_022	-517.6961698	-2.29347	-519.9896398	-517.7290850	-2.41145	-520.1405330	-520.2344378
	PC	mepy_035	-517.7228849	-2.28541	-520.0082934	-517.7558896	-2.40333	-520.1592225	-520.2531161
	PC	mepy_019	-517.7239260	-2.28474	-520.0086626	-517.7569527	-2.40265	-520.1596043	-520.2534977
**20**	R	r20_1	-439.1316334	-1.86658	-440.9982169	-439.1595777	-1.96632	-441.1259011	-441.2053246
**30**	R	r30_1	-552.7804022	-2.32597	-555.1063692	-552.8147559	-2.45017	-555.2649255	-555.3636950
**40**	R	r40_1	-247.1538780	-1.04441	-248.1982881	-247.1692319	-1.09868	-248.2679086	-248.3111666
**20 + 1**	RC	ts_r1r20_1_ircf_o	-595.8450486	-2.65146	-598.4965106	-595.8827301	-2.78786	-598.6705943	-598.7790512
	RC	ts_r1r20_3_ircr_o	-595.8464650	-2.64988	-598.4963478	-595.8841728	-2.78632	-598.6704895	-598.7789769
	TS	ts_r1r20_3	-595.8205943	-2.67440	-598.4949921	-595.8582234	-2.81092	-598.6691417	-598.7776669
	TS	ts_r1r20_1	-595.8218927	-2.67385	-598.4957441	-595.8594929	-2.81038	-598.6698768	-598.7784020
	TS	ts_r1r20_4	-595.8205952	-2.67440	-598.4949939	-595.8582244	-2.81092	-598.6691435	-598.7776688
	P	ts_r1r20_1_ircr_o	-595.8330695	-2.67927	-598.5123352	-	-	-	-
	P	ts_r1r20_3_ircf_o	-595.8329999	-2.67832	-598.5113196	-595.8706661	-2.81478	-598.6854480	-598.7939430
**30 + 1**	RC	ts_r1r30_2_ircr_o	-709.4895005	-3.11567	-712.6051704	-	-	-	-
	RC	ts_r1r30_1_ircf_o	-709.4884182	-3.11612	-712.6045365	-	-	-	-
	TS	ts_r1r30_2	-709.4609209	-3.14540	-712.6063205	-	-	-	-
	TS	ts_r1r30_1	-709.4639194	-3.14249	-712.6064137	-	-	-	-
	P	ts_r1r30_1_ircr_o	-709.4848908	-3.14465	-712.6295455	-	-	-	-
	P	ts_r1r30_2_ircf_o	-709.4817028	-3.14568	-712.6273808	-	-	-	-
**40 + 1**	RC	ts_r1r40_4_ircr_o	-403.8615837	-1.83836	-405.6999426	-403.8868227	-1.92947	-405.8162968	-405.8887653
	RC	ts_r1r40_2_ircr_o	-403.8622046	-1.83763	-405.6998371	-403.8874371	-1.92872	-405.8161614	-405.8886113
	TS	ts_r1r40_4	-403.8535393	-1.84728	-405.7008158	-403.8787989	-1.93846	-405.8172599	-405.8897840
	TS	ts_r1r40_2	-403.8530384	-1.84786	-405.7008947	-403.8783006	-1.93905	-405.8173501	-405.8898813
	P	ts_r1r40_2_ircf_o	-403.8596289	-1.85545	-405.7150790	-	-	-	-

	P	ts_r1r40_4_ircf_o	-403.8595244	-1.85553	-405.7150585	-403.8846692	-1.94680	-405.8314734	-405.9040239
**100**	R	r100_1	-78.6231095	-0.37918	-79.0022858	-	-	-	-
**50**	R	r50_1	-552.7783528	-2.32557	-555.1039180	-	-	-	-
**60**	R	r60_1	-438.9180753	-1.88960	-440.8076771	-	-	-	-
**50 + 1**	RC	ts_r1r50_2_ircr_o	-709.4921732	-3.11270	-712.6048774	-	-	-	-
	RC	ts_r1r50_1_ircr_o	-709.4993345	-3.10285	-712.6021812	-	-	-	-
	TS	ts_r1r50_2	-709.4651555	-3.14006	-712.6052124	-	-	-	-
	P	p_r1r50_2	-709.4841361	-3.14400	-712.6281351	-	-	-	-
	P	p_r1r50_1	-709.4704281	-3.14752	-712.6179486	-	-	-	-
**50 + 100**	RC	ts_r100r50_3_ircr_o	-631.3979217	-2.71400	-634.1119255	-	-	-	-
	RC	ts_r100r50_2_ircr_o	-631.3982545	-2.71283	-634.1110879	-	-	-	-
	RC	ts_r100r50_4_ircr_o	-631.3986695	-2.71347	-634.1121425	-	-	-	-
	RC	ts_r100r50_7_ircr_o	-631.3986690	-2.71347	-634.1121423	-	-	-	-
	RC	ts_r100r50_1_ircr_o	-631.3976909	-2.71362	-634.1113104	-	-	-	-
	RC	ts_r100r50_5_ircr_o	-631.4007370	-2.71031	-634.1110434	-	-	-	-
	TS	ts_r100r50_4	-631.3659997	-2.74286	-634.1088567	-	-	-	-
	TS	ts_r100r50_5	-631.3688008	-2.73886	-634.1076587	-	-	-	-
	TS	ts_r100r50_7	-631.3792180	-2.72756	-634.1067748	-	-	-	-
	TS	ts_r100r50_1	-631.3692749	-2.73458	-634.1038537	-	-	-	-
	TS	ts_r100r50_3	-631.3690236	-2.73308	-634.1020988	-	-	-	-
	TS	ts_r100r50_2	-631.3685436	-2.73293	-634.1014726	-	-	-	-
	P	ts_r100r50_4_ircf_o	-631.3976217	-2.74810	-634.1457235	-	-	-	-
	P	ts_r100r50_5_ircf_o	-631.3979209	-2.74648	-634.1444005	-	-	-	-
	P	ts_r100r50_1_ircf_o	-631.3920694	-2.75013	-634.1421956	-	-	-	-
	P	ts_r100r50_7_ircf_o	-631.3954006	-2.74737	-634.1427674	-	-	-	-
	P	ts_r100r50_3_ircf_o	-631.3925490	-2.74847	-634.1410222	-	-	-	-
	P	ts_r100r50_2_ircf_o	-631.3891766	-2.74925	-634.1384287	-	-	-	-

	P	p_r100r50_8	-631.3809460	-2.75141	-634.1323590	-	-	-	-
**60 + 100**	RC	ts_r100r60_3_ircr_o	-517.5387143	-2.27662	-519.8153300	-	-	-	-
	RC	ts_r100r60_1_ircr_o	-517.5410568	-2.27376	-519.8148127	-	-	-	-
	TS	ts_r100r60_3	-517.5157515	-2.29457	-519.8103203	-	-	-	-
	TS	ts_r100r60_2	-517.5172236	-2.29257	-519.8097950	-	-	-	-
	TS	ts_r100r60_1	-517.5173123	-2.29198	-519.8092889	-	-	-	-
	P	ts_r100r60_3_ircf_o	-517.5365642	-2.30954	-519.8461021	-	-	-	-
	P	ts_r100r60_2_ircf_o	-517.5374263	-2.30838	-519.8458037	-	-	-	-
	P	ts_r100r60_1_ircf_o	-517.5370675	-2.30828	-519.8453437	-	-	-	-
