# Supplementary material for: Radical chain monoalkylation of pyridines
Source: Chem Sci. 2021 Nov 3;12(46):15362–73. doi: 10.1039/d1sc02748d (PMC8635225; doi:10.1039/d1sc02748d)

Electronic Supplementary Material (ESI) for Chemical Science.
This journal is © The Royal Society of Chemistry 2021

Supporting Information

Radical Chain Monoalkylation of Pyridines

Samuel Rieder, Camilo Meléndez, Fabrice Dénès, Harish Jangra, Kleni Mulliri, Hendrik Zipse* and Philippe Renaud*

Contents

1 *General information* 7

1.1 Techniques and materials 7

1.2 Instrumentation 7

2 *Preparation of starting materials* 8

2.1 Alkyl Iodides 8

(3S,8S,9S,10R,13R,14S,17R)-3-Iodo-10,13-dimethyl-17-((R)-6-methylheptan-2-yl)-2,3,4,7,8,9,10,11,12,13,14,15,16,17-tetradecahydro-1*H*-cyclopenta[a]phenanthrene[4] 8

(4-Iodocyclohexyl)benzene[5] 9

tert-Butyl 4-iodopiperidine-1-carboxylate[4] 10

2.2 Xanthates 10

General procedure A, GPA (Synthesis of Xanthates) 10

Ethyl 4-((ethoxycarbonothioyl)thio)undecanoate (48a) 11

Ethyl 4-((ethoxycarbonothioyl)thio)-5-(trimethylsilyl)pentanoate (48b) 11

Ethyl 4-acetoxy-4-((ethoxycarbonothioyl)thio)butanoate (48c) 11

Ethyl 4-butoxy-4-((ethoxycarbonothioyl)thio)butanoate (48d) 12

S-Methyl *O*-(undecan-2-yl) carbonodithioate (55) 12

S-Methyl *O*-((1*S*,2*S*,3*S*,5*R*)-2,6,6-trimethylbicyclo[3.1.1]heptan-3-yl) carbonodithioate (57) 12

2.3 Alkenes 13

(4-Methylenecyclohexyl)benzene 13

2-(2-(Benzyloxy)ethyl)-6,6-dimethylbicyclo[3.1.1]hept-2-ene 13

2.4 *N*-Methoxypyridinium salts 14

General procedure 1 (GP1): synthesis of pyridinium- and quinolinium-*N*-oxides with H_2O_2.[8] 14

General procedure 2 (GP2): synthesis of pyridinium- and quinolinium-*N*-oxides with *m*-CPBA 14

4-Methylquinoline 1-oxide 14

2-Methyl-quinoline 1-oxide 15

4-(*tert*-Butyl)pyridine 1-oxide 15

4-(Ethoxycarbonyl)pyridine 1-oxide 15

General procedure 3 (GP3): methylation of pyridinium- and quinolinium-N-oxides with trimethyloxonium tetrafluoroborate 15

General procedure 4 (GP4): preparation of *N*-methoxypyridinium·PF6 salts 15

General procedure 4bis (GP4bis): Direct synthesis of 2,4- and 2,6-dichloro pyridinium-*N*-OMe·BF_4 salts. 16

1-Methoxy-4-phenylpyridin-1-ium tetrafluoroborate (1a·BF_4) 16

1-Methoxy-4-phenylpyridin-1-ium hexafluorophosphate (1a·PF_6) 16

1-Methoxy-4-methylquinolin-1-ium tetrafluoroborate (1b) 17
1-Methoxy-4-chloroquinolin-1-ium tetrafluoroborate (1c) 17
5-Methoxyphenanthridin-5-ium tetrafluoroborate (1d) 17
3-Bromo-1-methoxyquinolin-1-ium (1e) 17
1-Methoxy-2-methylquinolin-1-ium tetrafluoroborate (1f) 18
1-Methoxy-4-tert-butylpyridinium hexafluorophosphate (1g) 18
4-(Ethoxycarbonyl)-1-methoxypyridin-1-ium hexafluorophosphate (1h) 18
1-Methoxy-2,6-dimethylpyridin-1-ium tetrafluoroborate (1i) 18
4-Chloro-1-methoxypyridin-1-ium tetrafluoroborate (1j) 19
4-Bromo-1-methoxypyridin-1-ium tetrafluoroborate (1k) 19
2,6-Dichloro-1-methoxypyridin-1-ium tetrafluoroborate (1l) 19
2,4-Dichloro-1-methoxypyridin-1-ium tetrafluoroborate (1m) 19
2-Chloro-1-methoxypyridin-1-ium tetrafluoroborate (1n) 19
1-Methoxy-2-(trifluoromethyl)pyridin-1-ium tetrafluoroborate (1o) 20
6-Fluoro-1-methoxyquinolin-1-ium tetrafluoroborate (1p) 20
2-Methoxyisoquinolin-2-ium tetrafluoroborate (1q) 21

3 Alkylation of N-methoxypyridinium 21

3.1 Alkylation with organoboranes 21

General procedure 5 (GP5): alkylation with *B*-alkylcatecholboranes 21
2-Cyclohexyl-4-phenylpyridine (2) 21
2-Cyclohexyl-4-methylquinoline (3) 22
2-cyclododecyl-4-methylquinoline (4) 22
2-*n*-Hexyl-4-methylquinoline (5) 22
2-*n*-Decyl-4-methylquinoline (6) 23
2-Ethyl-4-methylquinoline (7) 23
2-(2,3-Dimethylbutan-2-yl)-4-methylquinoline (8) 24
trans-4-Methyl-2-(2-methylcyclohexyl)quinoline (9) 24
11-(4-Methylquinolin-2-yl)undecan-1-ol (10) 25
2-(3-((*tert*-Butyldiphenylsilyl)oxy)-2-methylpropyl)-4-methylquinoline (11) 25
4-Chloro-2-cyclohexylquinoline (12) 26
6-Cyclohexylphenanthridine (13) 26
3-Bromo-2-cyclohexylquinoline (14) 26
4-Cyclohexyl-2-methylquinoline (15) 27
2-Dodecyl-4-phenylpyridine (16) 27
2-(2,3-dimethylbutan-2-yl)-4-phenylpyridine (17) 27
2-Cyclohexyl-4-*tert*-butyl-pyridine (18) 28
Ethyl 2-cyclohexylisonicotinate (19) 28

4-Methyl-2-((1R,2R,3R,5S)-2,6,6-trimethylbicyclo[3.1.1]heptan-3-yl) (20) 28

2-(((1*S*,2*S*,5*S*)-6,6-Dimethylbicyclo[3.1.1]heptan-2-yl)methyl)-4-methylquinoline (21) 29

2-(2-(2-(Benzyloxy)ethyl)-6,6-dimethylbicyclo[3.1.1]heptan-3-yl)-4-methylquinoline (22) 29

1-(2-(2-(benzyloxy)ethyl)-6,6-dimethylbicyclo[3.1.1]heptan-3-yl)isoquinoline (23).... 30

2-(2-(2-(Benzyloxy)ethyl)-6,6-dimethylbicyclo[3.1.1]heptan-3-yl)-6-fluoroquinoline (24) 31

2-(2-(2-(Benzyloxy)ethyl)-6,6-dimethylbicyclo[3.1.1]heptan-3-yl)-6-chloropyridine (25) 32

2-(2-(2-(Benzyloxy)ethyl)-6,6-dimethylbicyclo[3.1.1]heptan-3-yl)-6-(trifluoromethyl)pyridine (26) and 4-(2-(2-(benzyloxy)ethyl)-6,6-dimethylbicyclo[3.1.1]heptan-3-yl)-2-(trifluoromethyl)pyridine (C_4-26) 33

2-(6,6-Dimethyl-3-(4-methylquinolin-2-yl)bicyclo[3.1.1]heptan-2-yl)ethan-1-ol (27) 34

2-(((1S,2R,4R)-3,3-dimethylbicyclo[2.2.1]heptan-2-yl)methyl)-4-methylquinoline (28).... 34

(3S,5R,6S,8S,9S,10R,13R,14S,17R)-10,13-Dimethyl-17-((R)-6-methylheptan-2-yl)-6-(4-methylquinolin-2-yl)hexadecahydro-1*H*-cyclopenta[a]phenanthren-3-yl benzoate (29).... 35

4-Methyl-2-[1-methyl-1-[(1R,4S)-4-methylcyclohex-2-en-1-yl]ethyl]quinoline (30) 36

3.2 Alkylation with alkyl iodides 36

General procedure 6 (GP-6): alkylation of *N*-methoxypyridinium salts with alkyl iodides 36

2-Cyclohexyl-4-methylquinoline (3) 37

4-Cyclohexyl-2-methylquinoline (15) 37

2-Isopropyl-4-methylquinoline (31) 37

2-(*tert*-Butyl)-4-phenylpyridine (32).... 37

4-Cyclohexyl-2,6-dimethylpyridine (33) 38

4-Isopropyl-2,6-dimethylpyridine (34) 38

4-(*tert*-Butyl)-2,6-dimethylpyridine (35) 38

4-Adamantyl-2,6-dimethylpyridine (36).... 38

4-((3*R*,8*S*,9*S*,10*R*,13*R*,14*S*,17*R*)-10,13-Dimethyl-17-((*R*)-6-methylheptan-2-yl)-2,3,4,7,8,9,10,11,12,13,14,15,16,17-tetradecahydro-1H-cyclopenta[a]phenanthren-3-yl)-2,6-dimethylpyridine (37).... 39

4-Chloro-2-cyclohexylpyridine (38) 39

4-Chloro-2-isopropylpyridine (39).... 39

4-Chloro-2-isopropylpyridine (40).... 40

4-Chloro-2-adamantylpyridine (41).... 40

4-Bromo-2-isopropylpyridine (42) 40

4-Bromo-2-adamantylpyridine (43) 40

tert-Butyl 6-(2,4-dichloropyridin-4-yl)piperidine-1-carboxylate (44) 41

2,6-Dichloro-4-adamantylpyrindine (45).... 41

2,6-Dichloro-4-(4-phenylcyclohexyl)pyridine (46).... 41

tert-Butyl 4-(2,6-dichloropyridin-4-yl)piperidine-1-carboxylate (47) 41

3.3 Alkylation with xanthates 42

General procedure 7, GP7 (alkylation of *N*-methoxypyridinium salts with xanthates) 42
Ethyl 4-(2,6-dimethylpyridin-4-yl)undecanoate (49) 42
Ethyl 4-(4-chloropyridin-2-yl)-5-(trimethylsilyl)pentanoate (50) 42
Ethyl 4-acetoxy-4-(2,6-dimethylpyridin-4-yl)butanoate (51) 43
Ethyl 4-acetoxy-4-(4-chloropyridin-2-yl)butanoate (52) 43
Ethyl 4-acetoxy-4-(4-bromopyridin-2-yl)butanoate (53) 43
Ethyl 4-(4-bromopyridin-2-yl)-4-butoxybutanoate (54) 43
2,6-Dimethyl-4-(undecan-2-yl)pyridine (56) 44
S-Methyl *S*-((1*S*,2*S*,3*S*,5*R*)-2,6,6-trimethylbicyclo[3.1.1]heptan-3-yl) carbonodithioate (58): 44
Attempted addition of cholesteryl *O*-ethyl xanthate to 1b·BF4 45

3.4 One-pot three-component alkylation of *N*-methoxypyridinium salts 45

General procedure 8, GP8 (three-component alkylation of *N*-methoxypyridinium salts) 45
Ethyl 4-(4-methylquinolin-2-yl)undecanoate (59) 46
Ethyl 4-(4-chloropyridin-2-yl)undecanoate (60) 46
Ethyl 4-(phenanthridin-6-yl)undecanoate (61) 46
Ethyl 4-(4-chloropyridin-2-yl)-5-hydroxypentanoate (62) 47
Ethyl 3-(1-(2,6-dichloropyridin-4-yl)-4-phenylcyclohexyl) propanoate (63) 47
Ethyl 4-(4-chloropyridin-2-yl)-4-(2-oxopyrrolidin-1-yl) butanoate (64) 47
Ethyl 4-acetoxy-4-(4-methylquinolin-2-yl)pentanoate (65) 48
Ethyl 4-acetoxy-4-(4-chloropyridin-2-yl)pentanoate (66) 48

4 Mechanistic investigations (kinetic study and competition experiments) 48

4.1 Kinetic study (determination of the the rate of addition k_{add} to *N*-methoxylepidinium) ..48

General 48
Experimental procedure for the kinetic experiment 49
Table for rate constant determination 49
Graphical representation 50
Rate constant calculation 50

4.2 Verification of the validity of the reported rate constant for the addition of primary alkyl radicals to protonated lepidine 51

Experimental procedure 51

4.3 Influence of the base on the regioselectivity 52

2-Chloro-4- and 6--isopropylpyridine (67) 52

4.4 Regioselective addition of *tert*-alkyl radicals 52

4-(1-Adamantyl)-2-chloro-pyridine (68) 52

4.5 Competitive addition of *sec*- and *tert*-alkyl radicals 53

General 53
Experimental procedure 53
Conclusion 53

5 References 54

6 ^{1}H and ^{13}C NMR spectra 55

1 General information

1.1 Techniques and materials

Glassware. Otherwise stated, all the glassware used to perform the reactions was oven-dried at 180 °C or flame-dried under high vacuum, assembled hot and allowed to cool down to room temperature under a stream of argon. Except for processes initiated by oxygen (Et_3B), all reactions were carried out under argon atmosphere.

Materials. Catecholborane used for hydroboration is commercially available (Sigma Aldrich) and was distilled under argon atmosphere prior to use. Commercial triethylborane (neat) was used as a solution in benzene (1.15 M, considering additive volumes) or hexane (1 M, considering additive volumes). The solutions were prepared using degassed solvents (freeze/thaw method) and the addition carried out by inserting the needle directly into the solvent. Unless otherwise stated, the alkyl iodides and alkenes used were commercially available.

Solvents. 1,2-Dichloroethane (DCE) and dichloromethane (DCM) used as solvents in the reactions were dried by filtration through a column of dry alumina under a positive pressure of argon and kept stored in the presence of molecular sieves (4 Å). In certain cases, DCE was dried by distillation from CaH_2 under inert atmosphere. Ethanol absolute (Merck) employed for recrystallization of *N*-methoxypyridinium salts was used without prior distillation. Solvents employed for extraction (DCM, Et_2O, AcOEt) were of technical grade and were distilled prior to their use.

Purification: The corresponding reaction crudes were purified by flash column chromatography using silica gel 60 Å (230–400 mesh particle size). All solvents were of technical grade and distilled before use. The reactions involving the use of triethylborane or *B*-alkylcatecholboranes were passed through a pad of neutral aluminum oxide (40–160 µm) prior to purification to remove boron and polar residues.

Reaction monitoring. Thin layer chromatography (TLC) was performed on 0.25 mm silica gel plates 60 (glass supported) with fluorescent indicator UV 254 using UV (254 nm) visualization or stained with a solution of potassium permanganate [$KMnO_4$ (3 g), K_2CO_3 (20 g) and NaOH 5% (3 mL) in H_2O (300 mL)] or cerium ammonium molybdate (($(NH_4)Mo_7O_{24}$ (5 g) + $Ce(SO_4)_2$ (0.2 g) in H_2O (100 mL, 5% H_2SO_4)) and subsequent heating.

1.2 Instrumentation

^1H and ^{13}C NMR. Spectra were recorded on a Bruker Avance 300 (^1H: 300.13 MHz, ^{13}C: 75.48 MHz, 11B: 96.30 MHz). Some ^1H and ^{13}C NMR were recorded on a Bruker Avance II 400 spectrometer (1H: 400.13 MHz, 13C: 101 MHz). Chemical shifts (δ) were reported in parts per million (ppm) with the residual solvent peak as an internal standard ($CHCl_3$: δ = 7.26 ppm, C_6H_6: δ = 7.16 ppm, CH_3CN: δ =

1.94 ppm for ^{1}H NMR and $CDCl_3$: δ = 77.16 ppm, C_6D6: δ = 128.06 ppm, CD_3CN: δ = 1.32 ppm for ^{13}C NMR spectra).

High-resolution mass spectrometry (HRMS). Spectra were recorded on an Applied Biosystems Sciex QSTAR Pulsar (hybrid quadrupole time-of-flight mass spectrometer) using positive electron spray. Infrared spectra were recorded neat on a *Jasco* FT-IR-460 Plus spectrometer equipped with a Specac MKII Golden Gate Single Reflection Diamond ATR System or a *Jasco* FT/IR-4700 equipped with a *Jasco* ATR Pro One.

Gas Chromatography analysis. Analyses were carried out on a *Thermo Quest* instrument Trace GC Ultra fitted with a *Macherey-Nagel* Optima delta-3-0.25 µm capillary column (20 m, 0.25 mm) or delta-3-0.25 µm capillary column (30 m, 0.25 mm); carrier gas: He 1.2 mL/min; injector: 220 °C split mode; detector: FID 280 °C, H_2 35 mL/min, air 350 mL/min. GC-MS analyses were performed on a *Thermo Quest* instrument *Trace GC* coupled with *Finnigan* Trace MS (quadrupole mass analyzer using electron impact 70 eV) fitted with a *Macherey-Nagel* Optima delta-3-0.25 µm capillary column (20 m, 0.25 mm) or delta-3-0.25 µm capillary column (l = 30 m, i.d. = 0.25 mm); carrier gas: He 1.4 mL/min; injector: 220 °C split mode.

IR Analysis. Infrared spectra were recorded neat on a *Jasco* FT-IR-460 Plus spectrometer equipped with a Specac MKII Golden Gate Single Reflection Diamond ATR System or a *Jasco* FT/IR-4700 equipped with a *Jasco* ATR Pro One.

2 Preparation of starting materials

Ethyl ethyl iodoacetate[1] and ethyl 2-((ethoxycarbonothioyl)thio)acetate[2] used for multicomponent reactions were prepared according to known methods. Similarly, di-tert-butylhyponitrite (DTBHN) used as initiator for the radical reactions was synthetized according to our previous reports.[3]

2.1 Alkyl Iodides

(3S,8S,9S,10R,13R,14S,17R)-3-Iodo-10,13-dimethyl-17-((R)-6-methylheptan-2-yl)-2,3,4,7,8,9,10,11,12,13,14,15,16,17-tetradecahydro-1*H*-cyclopenta[a]phenanthrene[4]

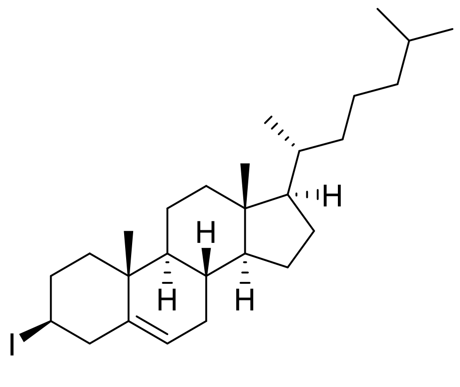

To a solution of imidazole (1.62 g, 23.7 mmol) and PPh_3 (6.62 g, 25.2 mmol) in CH_2Cl_2 (15 mL), I_2 (6.02 g, 23.7 mmol) was added at 0 °C. The mixture was stirred for 30 min at 0 °C. Cholesterol (6.11 g, 15.0 mmol (95% pure)) in CH_2Cl_2 (25 mL) was added to the reaction mixture dropwise. The reaction mixture was allowed to warm up to rt slowly and stirred overnight. The reaction was followed by TLC and treated with water (100 mL). The aqueous layer was extracted with

pentane (3 × 40 mL). The combined organic layers were washed with brine, dried over Na_2SO_4, filtered and concentrated under reduced pressure. The crude mixture was purified by flash chromatography on silica gel (pentane). The solvents were removed under reduced pressure. Recrystallization from acetone afforded the desired product (6.07 g, yield 82%) as clear needles. ^{1}H NMR (400 MHz, $CDCl_3$) δ 5.47 – 5.19 (m, 1H), 4.04 (tt, J = 12.5, 4.4 Hz, 1H), 3.01 – 2.86 (m, 1H), 2.67 (ddd, J = 13.7, 4.4, 2.1 Hz, 1H), 2.35 – 2.13 (m, 2H), 2.08 – 1.90 (m, 2H), 1.89 – 1.77 (m, 1H), 1.73 (dt, J = 13.4, 3.4 Hz, 1H), 1.64 – 0.94 (m, 23H), 0.91 (d, J = 6.5 Hz, 3H), 0.86 (dd, J = 6.6, 1.8 Hz, 6H), 0.67 (s, 3H). ^{13}C NMR (101 MHz, CDC 3) δ 142.9, 121.8, 56.9, 56.3, 50.6, 46.6, 42.5, 42.1, 39.9, 39.7, 36.8, 36.6, 36.4, 35.9, 31.9, 31.8, 30.6, 28.4, 28.2, 24.4, 24.0, 23.0, 22.7, 21.0, 19.4, 18.90, 12.0. Spectral data in accordance with literature.[4]

(4-Iodocyclohexyl)benzene[5]

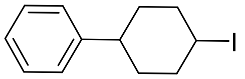

4-Phenylcyclohexanone (3.485 g, 20 mmol) was dissolved in MeOH (50 mL) and $NaBH_4$ (0.76 g, 20 mmol) was added to the reaction mixture portion wise. After 1 h, additional $NaBH_4$ (0.38 g, 10 mmol) was added. After full conversion of the starting material, saturated aqueous solution of NH_4Cl (50 mL) was added dropwise and the mixture was stirred for additional 15 min. The volatiles were then removed under reduced pressure. The aqueous layer was extracted with Et_2O (3 × 70 mL). The combined organic layers were washed with brine, dried over Na_2SO_4 and concentrated. The crude mixture was purified by flash chromatography (cyclohexane/EtOAc 7:3) to afford 4-phenylcyclohexanol (3.24 g, 92%) as a mixture of diastereoisomers. ^{1}H NMR (400 MHz, $CDCl_3$) δ 7.33 – 7.13 (m, 5H), 4.17 – 4.02 (m, minor, 0.2H), 3.69 (ddt, J = 14.9, 10.5, 4.3 Hz, major, 0.8H), 2.51 (tt, J = 11.9, 9.2, 5.8 Hz, 1H), 2.22– 2.03 (m, 2H), 2.01 – 1.83 (m, 2H), 1.79 – 1.64 (m, 1H), 1.63 – 1.24 (m, 5H). Spectral data in accordance with literature.[5]

To a solution of imidazole (1.97 g, 28.9 mmol) and PPh_3 (8.1g, 30.75 mmol) in CH_2Cl_2 (30 mL) was added iodine (0.75 g, 2.9 mmol) at 0 °C and the resulting mixture was stirred for 30 minutes at the same temperature. The alcohol obtained in the previous step (3.23 g, 18.3 mmol) in CH_2Cl_2 (15 mL) was added dropwise to the solution followed with additional iodine (6.6 g, 2.8 mmol) at 0 °C. The resulting brown solution was stirred overnight and allowed to warm up to rt slowly. After completion of the reaction, water (100 mL) was added to the mixture and it was extracted with CH_2Cl_2 (3 × 100 mL). The combined organic layers were washed with $Na_2S_2O_3$ (aq. sat. sol.), brine, dried over Na_2SO_4 and concentrated under reduced pressure. The crude mixture was purified by flash chromatography (pentane) to give the desired alkyl iodide (3.21 g, yield 61%) as a mixture of diastereoisomers. White powder. ^{1}H NMR (400 MHz, $CDCl_3$) δ 7.3 – 7.2 (m, 5H), 7.2 – 7.1 (m, 1H), 4.9 – 4.8 (m, 0.8H), 4.1 (tt, J = 12.3, 4.1 Hz, 0.2H), 2.6 – 2.4 (m, 1H), 2.2 – 1.9 (m, 4H), 1.8 – 1.4 (m, 5H). Spectral data in accordance with literature.[5]

***tert*-Butyl 4-iodopiperidine-1-carboxylate**[4]

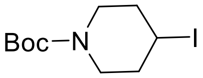

To a solution of sodium hydroxide (1.80 g, 45 mmol) in water (38 mL) was added *tert*-butanol (38 mL, 45 mmol), piperidin-4-ol (3.03 g, 30 mmol) and Boc_2O (7.93 mL, 34 mmol). The reaction mixture was stirred at rt overnight and was then diluted with 0.2 M HCl and extracted with EtOAc (3 × 100 mL). The combined organic layers were washed with brine, dried over Na_2SO_4, filtered and concentrated under reduced pressure. The crude product was purified by flash chromatography (heptane/EtOAc 1:1) to give *tert*-butyl 4-hydroxypiperidine-1-carboxylate (5.89 g, 98% yield). White solid. ^{1}H NMR (400 MHz, CDC_3) δ 4.01 – 3.55 (m, 3H), 3.20 – 2.75 (m, 2H), 1.96 – 1.61 (m, 2H), 1.52 – 1.31 (m, 11H). ^{13}C NMR (101 MHz, CDC_3) δ 154.9, 79.66, 67.5, 41.4 (br., 2C), 34.2 (2C), 28.5 (3C). Spectral data in accordance with literature.[4]

I_2 (14.54 g, 57 mmol) was added to a solution of PPh_3 (15.02 g, 57 mmol) and imidazole (3.90 g, 57 mmol) in dry CH_2Cl_2 (100 mL) under argon atmosphere at 0 °C. The reaction was stirred at 0 °C during 30 min. The previously prepared *N*-Boc protected alcohol (9.75 g, 38 mmol) in CH_2Cl_2 (40 mL) was added dropwise to the heterogeneous solution at 0 °C. The reaction was stirred overnight and was allowed to warm to rt slowly (the cold bath was not removed). Water (20 mL) was added to the reaction mixture and was stirred vigorously. The aqueous layer was extracted with CH_2Cl_2 (3 × 100 mL). The combined organic layer was washed with $Na_2S_2O_6$ (aq. sat. sol.) and brine, dried over $MgSO_4$, filtered and concentrated under reduced pressure to give a white solid. The crude mixture was then purified by flash chromatography on silica gel (cyclohexane/EtOAc 99:1). A fraction with a pure compound was dried under high vaccum and the rest was recrystallized from EtOH/H_2O (seeded with the pure fraction) to give the desired alkyl iodide (5.53 g, 62% yield). White solid. ^{1}H NMR (400 MHz, CDC_3) δ 4.44 (quint, J = 5.9 Hz, 1H), 3.58 (app. dt, J = 13.6, 5.1 Hz, 2H), 3.28 (app. dt, J = 13.6, 5.9 Hz, 2H), 2.02 (app q, J = 5.7 Hz, 4H), 1.45 (s, 9H). ^{13}C NMR (101 MHz, CDC_3) δ 154.8, 80.0, 44.0 (br.), 37.5 (2C), 28.6 (3C), 27.9 (2C). Spectral data in accordance with literature.[4]

2.2 Xanthates

General procedure A, GPA (Synthesis of Xanthates)

A mixture of ethyl 2-((ethoxycarbonothioyl)thio)acetate (1,7 g,8 mmol) and the corresponding olefin (16 mmol) in ethyl acetate (24 mL) was refluxed under Ar for 15 minutes. Lauroyl peroxide (DLP) (480 mg, 16 mmol) was added every 1 h until full conversion of the starting xanthate. The mixture was cooled down to rt and concentrated under reduced pressure. The crude product was purified by column chromatography using heptane/EtOAc mixtures.

Ethyl 4-((ethoxycarbonothioyl)thio)undecanoate (48a)

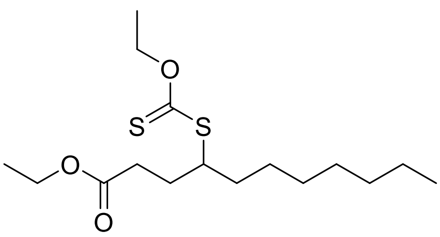

$C_{16}H_{30}O_3S_2$
MW = 334.56

Prepared according to **GPA** from 2-((ethoxycarbonothioyl)thio) acetate (3 g, 14.4 mmol) and 1-nonene (5 mL, 28.8 mmol). FC (Pentane/Et_2O 50:1) gave the desired xanthate (1.97 g, 41%). Clear oil. ^1H NMR (300 MHz, $CDCl_3$) δ 4.62 (q, *J* = 7.1 Hz, 2H), 4.11 (q, *J* = 7.1 Hz, 2H), 3.74 (dtd, *J* = 8.6, 6.8, 4.9 Hz, 1H), 2.43 (ddd, *J* = 8.1, 6.9, 1.3 Hz, 2H), 2.17 – 1.99 (m, 1H), 1.89 (dtd, *J* = 14.4, 8.3, 7.0 Hz, 1H), 1.71 – 1.58 (m, 2H), 1.40 (t, *J* = 7.1 Hz, 3H), 1.35 – 1.16 (m, 13H), 0.93 – 0.81 (m, 3H). ^{13}C NMR (75 MHz, $CDCl_3$) δ 214.57, 173.15, 69.90, 60.56, 50.93, 34.42, 31.87, 31.73, 29.57, 29.48, 29.22, 26.90, 22.73, 14.33, 14.18, 13.89.). IR (cm^{-1}): 2970 (s), 2863 (m) 1740 (s), 1470 (w) 1241 (m), 1046 (m).

Ethyl 4-((ethoxycarbonothioyl)thio)-5-(trimethylsilyl)pentanoate (48b)

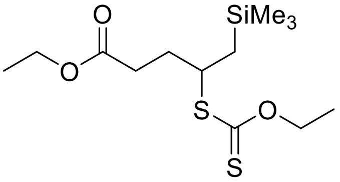

$C_{13}H_{26}O_3S_2Si$
MW = 322.55

Prepared according to **GPA** from 2-((ethoxycarbonothioyl)thio)acetate (833 mg, 4 mmol) and allyl(trimethyl)silane (914 mg, 8 mmol). FC (heptane/AcOEt 30:1) afforded the desired xanthate. Colorless oil (1.3 g, 89%). ^1H NMR (300 MHz, $CDCl_3$) δ 4.62 (qd, *J* = 7.1, 1.4 Hz, 2H), 4.11 (q, *J* = 7.1 Hz, 2H), 3.88 (tdd, *J* = 8.2, 7.1, 4.6 Hz, 1H), 2.42 (t, *J* = 7.6 Hz, 2H), 2.11 (dtd, *J* = 14.4, 7.9, 4.6 Hz, 1H), 1.90 (ddd, *J* = 14.9, 8.1, 6.9 Hz, 1H), 1.40 (t, *J* = 7.1 Hz, 3H), 1.23 (t, *J* = 7.1 Hz, 3H), 1.17 – 0.91 (m, 2H), 0.06 (s, 8H). ^{13}C NMR (75 MHz, $CDCl_3$) δ 214.73, 173.51, 70.12, 60.95, 48.55, 32.67, 32.01, 23.73, 14.74, 14.32, -0.25. Spectral data in accordance with literature.[1]

Ethyl 4-acetoxy-4-((ethoxycarbonothioyl)thio)butanoate (48c)

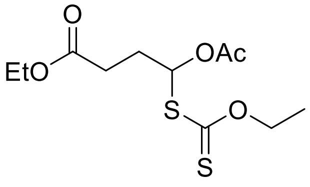

$C_{11}H_{18}O_5S_2$
MW = 294.38

Prepared according to **GPA** from 2-((ethoxycarbonothioyl)thio)acetate (4 g, 19.2 mmol) and isopropenyl acetate (3.5 mL, 38.4 mmol). FC (heptane/AcOEt 10:1) gave the desired xanthate (3.00 g, 53%). Clear-yellow oil. ^1H NMR (300 MHz, $CDCl_3$) δ 6.60 (t, *J* = 6.3 Hz, 1H), 4.57 (dtt, *J* = 10.5, 6.9, 3.5 Hz, 2H), 4.08 (q, *J* = 7.1 Hz, 2H), 2.40 (d, 2H), 2.28-2.16 (m, 2H), 2.01 (s, 3H), 1.35 (t, *J* = 6.0 Hz, 3H), 1.20 (t, *J* = 6.0 Hz, 3H). ^{13}C NMR (75 MHz, $CDCl_3$) δ 210.4, 172.1, 169.4, 79.1, 70.4, 60.9, 30.3, 29.6, 20.0, 14.8, 13.3. Spectral data in accordance with literature.[6]

Ethyl 4-butoxy-4-((ethoxycarbonothioyl)thio)butanoate (48d)

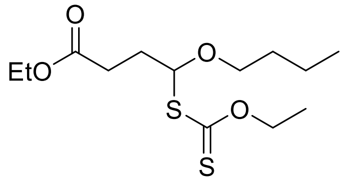

$C_{13}H_{24}O_4S_2$
MW = 308.45

Prepared according to **GPA** from 2-((ethoxycarbonothioyl)thio)acetate (833 mg, 4 mmol) nd 1-(vinyloxy)butane (800 mg, 8 mmol). FC (heptane/AcOEt, 10:1). Colorless oil (862 mg, 70%). ^{1}H NMR (300 MHz, $CDCl_3$) δ 5.56 (t, *J* = 6.3 Hz, 1H), 4.65 (q, *J* = 7.2 Hz, 2H), 4.14 (q, *J* = 7.2 Hz, 2H), 3.80 – 3.34 (m, 4H), 2.54 – 2.47 (m, 2H), 2.35 – 2.21 (m, 2H), 1.59 – 1.48 (m, 2H), 1.42 (t, *J* = 7.1 Hz, 3H), 1.39 – 1.29 (m, 2H), 1.26 (t, *J* = 7.1 Hz, 3H), 0.90 (t, *J* = 7.3 Hz, 3H). ^{13}C NMR (75 MHz, $CDCl_3$) δ 213.91, 172.90, 91.62, 69.87, 69.63, 60.65, 30.76, 19.41, 14.37, 13.89. HRMS: m/z calculated for $C_{13}H_{24}O_4NaS_2^+$ $[M+Na]^+$: 331.1013, found: 331.1008.

***S*-Methyl *O*-(undecan-2-yl) carbonodithioate (55)**

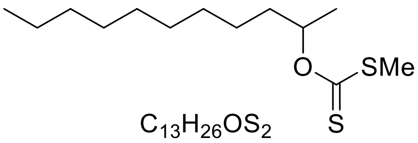

$C_{13}H_{26}OS_2$
MW = 262.47

To a suspension of sodium hydride (0.96 g, 24 mmol, 55–65% in mineral oil), prewashed with pentane, in dry DMF (20 mL) was carefully added dropwise undecan-2-ol (3.44 g, 20 mmol) at rt. The reaction mixture was stirred at rt for 30 min (gas evolution ceased) and carbon disulfide (1.85 mL, 24 mmol) was then added at room temperature, resulting in a very exothermic reaction. The reaction mixture was stirred for additional 12h and MeI (1.5 mL, 24 mmol) was added at 0 °C. The reaction mixture was stirred for 10h, with the temperature progressively reaching rt, before it was quenched with an aqueous saturated solution of NH_4Cl (50 mL). Et_2O (20 mL) was added, the phases were separated, and the aqueous layer was extracted with Et_2O (2 x 20 mL). The combined organic phase was washed with brine (5 x 50 mL), dried over Na_2SO_4, and the solvent removed under reduced pressure. The crude product was purified by flash column chromatography over silica gel (pentane/Et_2O 100:0 to 90:10) to give the titled compound (4.35 g, 82%) as a yellow oil (contamination with small amounts of an unidentified impurity). ^{1}H NMR (300 MHz, $CDCl_3$) δ 5.65 (hx, *J* = 6.3 Hz, 1H), 2.49 (s, 3H), 1.85–1.71 (m, 1H), 1.62–1.52 (m, 1H), 1.43–1.16 (m, 14H), 1.31 (d, *J* = 6.3 Hz, 3H), 0.84 (m, 3H). ^{13}C NMR (75 MHz, $CDCl_3$) δ 215.0 (C=S), 81.0 (CH), 35.5 (CH_2), 31.8 (CH_2), 29.5 (CH_2), 29.5 (CH_2), 29.4 (CH_2), 29.3 (CH_2), 25.2 (CH_2), 22.6 (CH_2), 19.2 (CH_3), 18.7 (CH_3), 14.0 (CH_3). FT–IR (cm^{-1}, neat): 2924, 2852, 1455, 1374, 1218, 1110, 1042, 959, 819, 722. HRMS (ESI): m/z calculated for $C_{13}H_{27}OS_2$ $[M+H]^+$: 263.1498, found: 263.1499.

***S*-Methyl *O*-((1*S*,2*S*,3*S*,5*R*)-2,6,6-trimethylbicyclo[3.1.1]heptan-3-yl) carbonodithioate (57)**

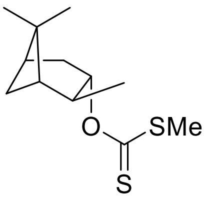

$C_{12}H_{20}OS_2$
MW = 244.41

To a suspension of sodium hydride (0.96 g, 24 mmol, 55–65% in mineral oil), prewashed with pentane, in dry DMF (20 mL) was carefully added dropwise isopinocampheol (3.09 g, 20 mmol) at rt. The reaction mixture was stirred at rt for 30 min (gas evolution ceased) and carbon disulfide (1.85 mL, 24 mmol) was then added at room temperature, resulting in a very exothermic reaction. The reaction mixture was stirred for additional 12 h and MeI (1.5 mL, 24 mmol) was added at 0

°C. The reaction mixture was stirred for 10h, with the temperature progressively reaching rt, before it was quenched with an aqueous saturated solution of NH_4Cl (50 mL). Et_2O (20 mL) was added, the phases separated, and the aqueous layer was extracted with Et_2O (2 x 20 mL). The combined organic phase was washed with brine (5 x 50 mL), dried over Na_2SO_4, and the solvent removed under reduced pressure. The crude product was purified by flash column chromatography over silica gel (pentane/Et_2O 100:0 to 90:10) to give the titled compound (4.35 g, 89%) as a yellow solid (contamination with small amounts of an unidentified impurity). ^{1}H NMR (400 MHz, $CDCl_3$) δ 5.82 (ddd, *J* = 9.2, 5.0, 4.1 Hz, 1H), 2.70 (ddt, *J* = 14.8, 9.3, 2.8 Hz, 1H), 2.55 (s, 3H), 2.43–2.31 (m, 2H), 1.96 (tt, *J* = 5.9, 3.0 Hz, 1H), 1.87 (td, *J* = 5.8, 2.3 Hz, 1H), 1.74 (ddd, *J* = 14.6, 4.1, 2.9 Hz, 1H), 1.24 (s, 3H), 1.14 (d, *J* = 7.5 Hz, 3H), 1.13 (d, *J* = 10.0 Hz, 1H), 0.97 (s, 3H). ^{13}C NMR (101 MHz, $CDCl_3$) δ 215.5 (C=S), 84.6 (CH), 47.6 (CH), 43.4 (CH), 41.3 (CH), 38.4 (Cq), 35.3 (CH_2), 33.4 (CH_2), 27.5 (CH_3), 23.9 (CH_3), 20.6 (CH_3), 19.1 (S-CH_3). $[\alpha]_D^{20}$ –29 (*c* 1.0, $CHCl_3$). Yellow solid. M.p. 61–62 ºC. FT–IR (cm^{-1}, neat): 2987, 2955, 2926, 2880, 2865, 1468, 1452, 1416, 1387, 1367, 1330, 1317, 1279, 1264, 1228, 1201, 1183, 1147, 1060, 1040, 963, 911, 857, 819, 727, 669, 617. HRMS (ESI): m/z calculated for $C_{12}H_{21}OS_2$ $[M+H]^+$: 245.1028, found: 245.1029.

2.3 Alkenes

(4-Methylenecyclohexyl)benzene[4]

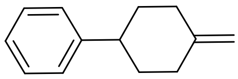

To a solution of *t*-BuOK (5.05 g, 45 mmol) in dry Et_2O (60 mL) was added Ph_3PCH_2Br (16.08 g, 45 mmol) under Ar at rt. The solution turned to yellow and was stirred 30 min. 4-Phenylcyclohexanone (5.23 g, 30 mmol) in dry Et_2O (20 mL) was then canulated into the reaction mixture and refluxed for 1.5 h. The reaction mixture was then diluted into Et_2O and washed with water. The organic layer was then dried over $MgSO_4$, filtered, and concentrated under reduced pressure to afford a yellow oil. The crude mixture was then purified by flash chromatography on silica gel (heptane/EtOAc 95:5) to give the desired olefin (5.16 g, quant. yield) as a colorless oil. ^{1}H NMR (300 MHz, $CDCl_3$) *δ* 7.38 – 7.17 (m, 5H), 4.71 (t, *J*= 1.7 Hz, 2H), 2.70 (tt, *J* = 12.2, 3.4 Hz, 1H), 2.54 – 2.31 (m, 2H), 2.32 – 2.14 (m, 2H), 2.08 – 1.94 (m, 2H), 1.58 (app. qd, *J* = 12.7, 3.7 Hz, 2H). ^{13}C NMR (75 MHz, $CDCl_3$) *δ* 149.0, 147.0, 128.5 (2C), 127.0 (2C), 126.1, 107.5, 44.3, 35.7 (2C), 35.3 (2C). Spectral data in accordance with literature.[4]

2-(2-(Benzyloxy)ethyl)-6,6-dimethylbicyclo[3.1.1]hept-2-ene

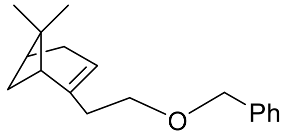

$C_{18}H_{24}O$
MW = 256.39

To a suspension of NaH (1.04 g, 24 mmol, 55–60 wt% dispersion in mineral oil) in dry DMF (40 mL) was added carefully (1*R*)-(−)-Nopol (3.33 g, 20 mmol) at 0 °C. The cold bath was removed and the reaction mixture was stirred at rt for 45 min and benzyl bromide (2.85 mL, 24 mmol) was added dropwise. The reaction mixture was stirred at rt for 12h. The reaction mixture was quenched with aqueous 1M HCl (40 mL). Et_2O (60 mL) was added and the biphasic mixture was

transferred into a separatory funnel and the phases were separated. The aqueous layer was extracted with Et_2O (3 x 40 mL), the combined organic layer was washed with aqueous $NaHCO_3$ (2 x 50 mL), and brine (6 x 50 mL). The organic layer was then dried over $MgSO_4$, filtered, and concentrated under reduced pressure to afford a yellow oil. The crude mixture was then purified by flash chromatography on silica gel (heptane/EtOAc 95:5) to give the desired olefin (4.26 g, 83%) as a colorless oil. ^{1}H NMR (300 MHz, $CDCl_3$) δ 7.38–7.24 (m, 5H), 5.29–5.26 (m, 1H), 4.50 (s, 2H), 3.49 (t, J = 7.1 Hz, 2H), 2.38–2.14 (m, 5H), 2.09–2.02 (m, 2H), 1.26 (s, 3H), 1.16 (d, J = 8.5 Hz, 1H), 0.83 (s, 3H). ^{13}C NMR (75 MHz, $CDCl_3$) δ 145.3 (Cq), 138.7 (Cq), 128.4 ($2CH_{Ar}$), 127.7 ($2CH_{Ar}$), 127.5 (CH), 118.0 (CH), 72.9 (CH_2), 69.0 (CH_2), 45.9, 40.9, 38.1 (Cq), 37.3 (CH_2), 31.8 (CH_2), 31.5 (CH_2), 26.4, 21.3. Spectral data in accordance with literature data.[7]

2.4 *N*-Methoxypyridinium salts

General procedure 1 (GP1): synthesis of pyridinium- and quinolinium-*N*-oxides with H_2O_2.[8]
To a stirred solution of the pyridine derivative (10 mmol) in glacial acetic acid (10 mL) was added hydrogen peroxide (3.4 mL, 30 wt% in H_2O, 30 mmol) and the reaction mixture was stirred at 70 ºC for 1–3 days. The crude solution was cooled down to rt, neutralized with Na_2CO_3 (sat.) and extracted with chloroform (3 × 20 mL). The combined organic layers were washed with brine and dried over Na_2SO_4. The volatiles were removed under reduced pressure to give the crude *N*-oxide.

General procedure 2 (GP2): synthesis of pyridinium- and quinolinium-*N*-oxides with *m*-CPBA
To a solution of pyridine derivative (20 mmol) in $CHCl_3$ (65 mL, stab. with EtOH) was added *m*-CPBA (<77%, 4.9 g, 22 mmol) in one portion. The resulting solution was stirred at rt for 13 h. To the crude yellow solution was added aqueous Na_2CO_3 (sat.) until a pH of ~10 was reached. The biphasic mixture was transferred into a separatory funnel and the aqueous layer was extracted with chloroform (3 × 20 mL). The combined organic layers were washed with brine, dried over Na_2SO_4 and the volatiles were removed under reduced pressure to give the crude *N*-oxide.

4-Methylquinoline 1-oxide

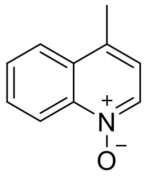

$C_{10}H_9NO$
MW = 159.19

Prepared according to **GP2** from 4-methylquinoline (3.8 mL, 28.1 mmol). The crude product (4.3 g, 97%) was pure enough to be used without further purification for the next step. Pale yellow solid. ^{1}H NMR (300 MHz, $CDCl_3$): δ 8.81 (dd, J = 8.5, 0.9 Hz, 1H), 8.44 (d, J = 6.2 Hz, 1H), 7.98 (dd, J = 8.5, 0.9 Hz, 1H), 7.73 (ddABq, $\Delta\delta_{AB}$ = 0.9, J_{AB} = 8.5 Hz, J_A = J_B = 8.5, 1.4 Hz, 2H), 7.12 (dd, J = 6.2, 0.5 Hz, 1H), 2.66 (br s, 3H). ^{13}C NMR (75 MHz, $CDCl_3$): δ 141.1 (Cq), 135.1 (CH), 134.7 (Cq), 130.2 (CH), 130.0 (Cq), 128.6 (CH), 124.8 (CH), 121.5 (CH), 120.5 (CH), 18.5 (CH_3). HRMS: m/z calculated for $C_{10}H_{10}NO^+$ $[M+H]^+$: 160.0757, found: 160.0754. Spectral data are in accordance with literature.[9]

2-Methyl-quinoline 1-oxide

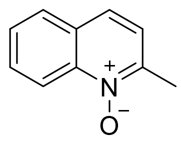

$C_{10}H_9NO$
MW = 159.19

Prepared according to **GP2** from 2-methyl-quinoline (98%, 2.8 mL, 20.3 mmol). The crude product (3.2 g, 99%) was pure enough to be used without further purification for the next step. Orange oil. ^{1}H NMR (300 MHz, $CDCl_3$): δ 8.78 (d, J = 8.7 Hz, 1H), 7.83 (dd, J = 8.1, 1.3 Hz, 1H), 7.75 (ddd, J = 8.7, 7.0, 1.3 Hz, 1H), 7.65 (d, J = 8.5 Hz, 1H), 7.59 (ddd, J = 8.1, 7.0, 1.3 Hz, 1H), 7.31 (d, J = 8.5 Hz, 1H), 2.72 (s, 3H). ^{13}C NMR (101 MHz, $CDCl_3$): δ 146.0 (Cq), 141.8 (Cq), 130.5 (CH), 129.4 (Cq), 128.1 (CH), 127.9 (CH), 125.3 (CH), 123.1 (CH), 119.7 (CH), 18.9 (CH_3). Spectral data are in accordance with literature.[10]

4-(*tert*-Butyl)pyridine 1-oxide

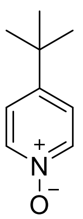

$C_9H_{13}NO$
MW = 151.1

Prepared according to **GP1** from 4-*tert*-butylpyridine (3.0 mL, 20.1 mmol). The crude product (3.06 g, 99%) was pure enough to be used without further purification for the next step. Slightly yellow amorphous solid. ^{1}H NMR (300 MHz, $CDCl_3$): δ 8.15 – 8.09 (m, 2H), 7.27 – 7.20 (m, 2H), 1.30 (s, 9H). ^{13}C NMR (101 MHz, $CDCl_3$): δ 151.0 (Cq), 138.7 (2C, CH), 123.2 (2C, CH), 34.7 (Cq), 30.6 (3C, CH_3). Spectral data are in accordance with literature.[8]

4-(Ethoxycarbonyl)pyridine 1-oxide

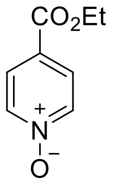

$C_8H_9NO_3$
MW = 167.06

Prepared according to **GP1** from ethyl isonicotinate (3.0 mL, 20.0 mmol). The crude product (3.3 g, 99%) was pure enough to be used without further purification for the next step. White amorphous solid ^{1}H NMR (300 MHz, $CDCl_3$): δ 8.23 – 8.17 (m, 2H), 7.89 – 7.83 (m, 2H), 4.37 (q, J = 7.1 Hz, 2H), 1.38 (t, J = 7.1 Hz, 3H). ^{13}C NMR (75 MHz, $CDCl_3$): δ 163.4 (Cq), 139.5 (2C, CH), 126.9 (Cq), 126.5 (2C, CH), 62.1 (CH_2), 14.3 (CH_3). Spectral data are in accordance with literature.[8]

General procedure 3 (GP3): methylation of pyridinium- and quinolinium-N-oxides with trimethyloxonium tetrafluoroborate

To a solution of the crude *N*-oxide (10 mmol) in DCM (20 mL) was added trimethyloxonium tetrafluoroborate (97%, 1,8 12 mmol) and the resulting mixture was stirred for 1–2 h. MeOH (≥99.8%, 1 mL) was added and, after stirring for another 20 min, the volatiles were removed under reduced pressure. The obtained solid was recrystallized from absolute ethanol to give the BF_4^- salt.

General procedure 4 (GP4): preparation of *N*-methoxypyridinium·PF6 salts

The crude *N*-methoxypyridinium·BF_4 salt obtained from **GP3** (20 mmol) was dissolved in water (200 mL) and a solution of potassium hexafluorophosphate in water (44mL, 22 mmol) was added. A white precipitate was formed immediately. The heterogeneous solution was stirred for 20 min, cooled down to 0 °C. The precipitate was isolated by filtration, washed with water, and air-dried for 4 h. The dry solid was then washed with DCM to remove the product from residual KPF_6. The volatiles were

removed under reduced pressure and the obtained solid was recrystallized from abs. EtOH to furnish the *N*-OMe·PF_6 salt.

General procedure 4bis (GP4bis): Direct synthesis of 2,4- and 2,6-dichloro pyridinium-*N*-OMe·BF_4 salts

To a stirred solution of the pyridine derivative (7 mmol) in TFA (10 mL) was added an aqu. sol. of hydrogen peroxide (30%, 2.4 mL,21 mmol) and the reaction mixture was stirred at 100 ºC for 5 h. The solution was cooled down to rt, poured into water and stirred for additional 15 min. The formed solid residue was filtered off. The filtrate was treated with a solid Na_2HCO_3 until a pH~10 was reached and the solution was extracted with DCM (2 × 20 mL). The combined organic layers were washed with brine and dried over Na_2SO_4. The solvent was removed under reduced pressure to provide the crude pyridine *N*-oxide as a solid residue. After trituration with hot Et_2O, the solid was isolated by filtration, dried *in vacuo* for 4 h, and transferred into a reaction flask. Dry DCM (20 mL) was added and the resulting solution was cooled down to 0 ºC (ice bath). Trimethyloxonium tetrafluoroborate (97%, 2.4 g, 7.7 mmol) was added and the mixture was stirred for 3 h. After this time, MeOH (99%, 2 mL) was added and the solution was stirred for additional 20 minutes. The volatiles were removed under reduced pressure to yield the crude *N*-methoxypyridinium salt which was further purified by recrystallization from EtOH or DCM (low temperature).

1-Methoxy-4-phenylpyridin-1-ium tetrafluoroborate (1a·BF_4)

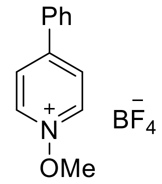

$C_{12}H_{12}BF_4N$
MW = 257.19

Prepared according to **GP3** from 4-phenylpyridine-N-oxide (98%, 3.45 g, 19.7 mmol). Recrystallization from abs. EtOH gave a solid (5.3 g, 98%). Colorless needles. ^1H NMR (300 MHz, CD_3CN): δ 8.95 – 8.93 (m, 2H), 8.35 – 8.31 (m, 2H), 7.94 – 7.90 (m, 2H), 7.71 – 7.61 (m, 3H), 4.41 (s, 3H). ^{13}C NMR (75 MHz, CD_3CN): δ 157.3 (Cq), 141.5 (2C, CH), 134.6 (Cq), 133.5 (CH), 130.9 (2C, CH), 129.3 (2C, CH), 127.3 (2C, CH), 70.9 (CH_3). Spectral data are in accordance with literature.[11]

1-Methoxy-4-phenylpyridin-1-ium hexafluorophosphate (1a·PF_6)

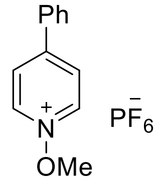

$C_{12}H_{12}F_6NOP$
MW = 331.2

Prepared according to **GP4** from 1-Methoxy-4-phenylpyridin-1-ium tetrafluoroborate (1a·BF_4) (98%, 3.5 g, 20.0 mmol). Recrystallization from abs. EtOH gave colorless needles. (2.52 g, 76%). ^1H NMR (300 MHz, CD_3CN): δ 8.96 – 8.89 (m, 2H), 8.35 – 8.29 (m, 2H), 7.94 – 7.88 (m, 2H), 7.72 – 7.60 (m, 3H), 4.41 (s, 3H). ^{13}C NMR (75 MHz, CD_3CN): δ 157.3 (Cq), 141.5 (2C, CH), 134.5 (Cq), 133.5 (CH), 130.9 (2C, CH), 129.3 (2C, CH), 127.2 (2C, CH), 70.9 (CH_3). Spectral data are in accordance with literature.[12]

1-Methoxy-4-methylquinolin-1-ium tetrafluoroborate (1b)

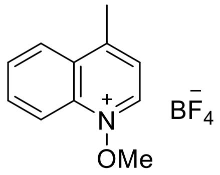

$C_{11}H_{12}BF_4NO$
MW = 261.09

Prepared according to **GP3** from 4-methyl-quinoline (98%, 3.8 mL, 28.1 mmol). Recrystallization from absolute EtOH gave the product (4.86 g, 85%). Colorless needles. ^{1}H NMR (300 MHz, CD_3CN): δ 9.21 (d, J = 6.6 Hz, 1H), 8.48 (m, 2H), 8.28 (ddd, J = 8.7, 7.1, 1.3 Hz, 1H), 8.07 (ddd, J = 8.7, 7.1, 1.3 Hz, 1H), 7.92 (dd, J = 6.6, 0.9 Hz, 1H), 4.46 (s, 3H), 3.02 (d, J = 0.9 Hz, 3H). ^{13}C NMR (75 MHz, CD_3CN): δ 160.2 (Cq), 143.4 (CH), 137.4 (CH), 136.5 (Cq), 131.8 (CH), 131.3 (Cq), 128.0 (CH), 123.7 (CH), 117.6 (CH), 70.7 (CH_3), 20.4 (CH_3). HRMS: m/z calculated for $C_{11}H_{12}NO^+$ $[M]^+$: 174.0913, found: 174.0909. Spectral data are in accordance with literature.[13]

1-Methoxy-4-chloroquinolin-1-ium tetrafluoroborate (1c)

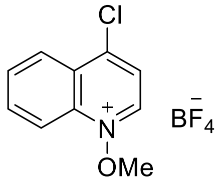

$C_{10}H_9BClF_4NO$
MW = 281.44

Prepared according to **GP3** from 4-chloroquinoline (99%, 1.64 g, 10.0 mmol, 1.0). Recrystallization from DCM gave a soldid (1.82 g, 63%). White powder. ^{1}H NMR (300 MHz, CD_3CN): δ 9.37 (d, J = 6.9 Hz, 1H), 8.63 (dd, J = 8.7, 0.5 Hz, 1H), 8.53 (d, J = 8.7 Hz, 1H), 8.42 – 8.34 (m, 1H), 8.22 (d, J = 6.9 Hz, 1H), 8.18 (ddd, J = 8.2, 4.0, 0.5 Hz, 1H), 4.51 (s, 3H). ^{13}C NMR (75 MHz, CD_3CN): δ 153.8 (Cq), 144.5 (CH), 138.8 (CH), 138.0 (Cq), 133.3 (CH), 130.1 (Cq), 127.7 (CH), 123.7 (CH), 118.1 (CH), 71.3 (CH_3). HRMS (ESI): m/z calculated for $C_{10}H_9ClNO^+$ $[M]^+$: 194.0367, found: 194.0366.

5-Methoxyphenanthridin-5-ium tetrafluoroborate (1d)

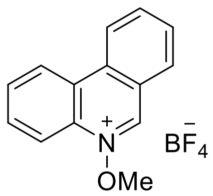

$C_{14}H_{12}BF_4NO$
MW = 297.09

Prepared according to **GP3** from phenantridine (98%, 1.83 g, 10.0 mmol**).** Recrystallization from abs. EtOH gave an off-white solid (2.43 g, 81%). ^{1}H NMR (300 MHz, CD_3CN): δ 10.10 (s, 1H), 9.08 – 8.96 (m, 2H), 8.56 (dd, J = 7.1, 2.3 Hz, 2H), 8.41 (ddd, J = 8.5, 4.3, 1.2 Hz, 1H), 8.25 – 8.08 (m, 3H), 4.59 (s, 3H). ^{13}C NMR (75 MHz, CD_3CN): δ 149.2 (CH), 139.4 (CH), 135.1 (Cq), 133.9 (CH), 133.6 (CH), 132.7 (Cq), 132.6 (CH), 131.9 (CH), 128.1 (Cq), 125.6 (CH), 124.5 (Cq), 124.2 (CH), 118.3 (CH), 70.7 (CH_3). HRMS (ESI): m/z calculated for $C_{14}H_{12}NO^+$ $[M]^+$: 210.0913, found: 210.0911. Spectral data are in accordance with the literature.[14]

3-Bromo-1-methoxyquinolin-1-ium (1e)

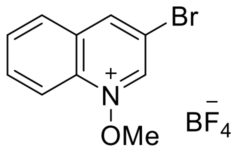

$C_{10}H_9BBrF_4NO$
MW = 324.99

Prepared according to **GP3** from 3-bromoquinoline (98%, 1.38 mL, 10.0 mmol). Recrystallization from abs. EtOH gave a solid (2.51 g, 77%). White powder. ^{1}H NMR (300 MHz, CD_3CN): δ 9.64 (d, J = 0.9 Hz, 1H), 9.32 (dd, J = 2.0, 0.9 Hz, 1H), 8.47 (ddd, J = 8.5, 2.0, 0.9 Hz, 1H), 8.38 – 8.29 (m, 2H), 8.10 (ddd, J = 8.5, 7.2, 0.9 Hz, 1H), 4.54 (s, 3H). ^{13}C NMR (75 MHz, CD_3CN): δ 149.4 (CH), 146.0 (CH), 138.3 (CH), 136.4 (Cq), 133.3 (CH), 132.3 (Cq), 130.4 (CH), 117.4 (CH), 115.1 (Cq), 71.6 (CH_3). HRMS (ESI): m/z calculated for $C_{10}H_9BrNO^+$ $[M]^+$: 237.9862, found: 237.9865; HRMS (ESI): m/z

calculated for $C_{10}H_9{}^{81}BrNO^+$ $[M]^+$: 239.9842, found: 237.9842 (the mass detected for the residue $C_{10}H_9BrNO^+$ correlates with the isotope ^{81}Br). Spectral data are in accordance with literature.[13]

1-Methoxy-2-methylquinolin-1-ium tetrafluoroborate (1f)

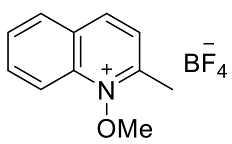

$C_{11}H_{12}BF_4NO$
MW = 261.09

Prepared according to **GP3** from 2-methyl-quinoline (98%, 2.8 mL, 20.3 mmol). Recrystallization from abs. EtOH gave a solid (5.6 g, 87%). Colorless needles. ^{1}H NMR (300 MHz, CD_3CN): δ 8.91 (d, J = 8.7 Hz, 1H), 8.41 (dd, J = 8.4, 1.0 Hz, 1H), 8.36 (dd, J = 8.6, 1.0 Hz, 1H), 8.26 (ddd, J = 8.7, 7.1, 1.0 Hz, 1H), 8.01 (ddd, J = 8.7, 7.1, 1.0 Hz, 1H), 7.92 (d, J = 8.6 Hz, 1H), 4.37 (s, 3H), 3.06 (s, 3H). ^{13}C NMR (75 MHz, CD_3CN): δ 158.6 (Cq), 146.2 (CH), 137.7 (Cq), 137.4 (CH), 131.3 (CH), 131.2 (CH), 130.8 (Cq), 125.6 (CH), 117.2 (CH), 68.7 (CH_3), 18.9 (CH_3). HRMS (ESI): m/z calculated for $C_{11}H_{12}NO^+$ $[M]^+$: 174.0908, found: 174.0913.

1-Methoxy-4-tert-butylpyridinium hexafluorophosphate (1g)

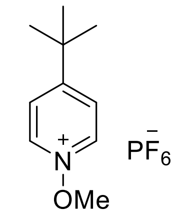

$C_{10}H_{16}F_6NOP$
MW = 311.2

Prepared according to **GP4** from 4-*tert*-butylpyridine-*N*-oxide (99%, 3.0 mL, 20.1 mmol)**.** Recrystallized from abs. EtOH gave a white powder (4.13 g, 65%). ^{1}H NMR (300 MHz, CD_3CN): δ 8.83 – 8.77 (m, 2H), 8.09 – 8.04 (m, 2H), 4.35 (s, 3H), 1.40 (s, 9H). ^{13}C NMR (75 MHz, CD_3CN): δ 172.1 (Cq), 140.7 (2C, CH), 127.6 (2C, CH), 70.8 (Cq), 37.5 (Cq), 30.1 (3C, CH_3). HRMS (ESI): m/z calculated for $C_{10}H_{16}NO^+$ $[M]^+$: 166.1226, found: 166.1223.

4-(Ethoxycarbonyl)-1-methoxypyridin-1-ium hexafluorophosphate (1h)

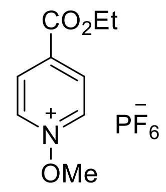

$C_9H_{12}F_6NOP$
MW = 327.16

Prepared according to **GP4** from ethyl isonicotinate (99%, 3.0 mL, 20.0 mmol, (99%, 3.0 mL, 20.1 mmol)**.** Recrystallized from abs. EtOH gave a white powder (3.74 g, 57%). ^{1}H NMR (300 MHz, CD_3CN): 9.12 – 9.05 (m, 2H), 8.55 – 8.48 (m, 2H), 4.48 (q, J = 7.1 Hz, 2H), 4.45 (s, 3H), 1.41 (t, J = 7.1 Hz, 3H). ^{13}C NMR (75 MHz, CD_3CN): δ 172.1 (Cq), 140.7 (2C, CH), 127.6 (2C, CH), 70.8 (Cq), 37.5 (Cq), 30.1 (3C, CH_3).

1-Methoxy-2,6-dimethylpyridin-1-ium tetrafluoroborate (1i)

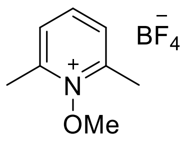

$C_8H_{12}BF_4NO$
MW = 255.09

Prepared according to **GP3** from 2,6-dimethylpyridine (910 mg, 8.5 mmol). Recrystallization from abs. EtOH afforded colorless crystals (786 mg, 74%).. ^{1}H NMR (300 MHz, DMSO-d_6): δ 8.36 (t, J = 7.9 Hz, 1H), 7.96 (d, J = 7.9 Hz, 2H), 4.29 (s, 3H), 2.83 (s, 6H). ^{13}C NMR (75 MHz, DMSO d6): δ 153.35, 143.92, 128.02, 66.95, 16.99. ^{11}B NMR (96 MHz, DMSO-d_6): δ -1.32. Spectral data are in accordance with literature.[15]

4-Chloro-1-methoxypyridin-1-ium tetrafluoroborate (1j)

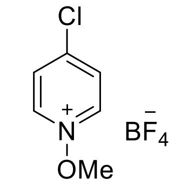

$C_6H_7ClBF_4NO$
MW = 231.02

Prepared according to **GP3** from 4-chloropyridine (908 mg, 8 mmol). Recrystallization from DCM (low temperature) afforded a yellowish solid (4.13 g, 65%). ^{1}H NMR (300 MHz, DMSO-*d6*): δ 9.49 (d, J = 6.8 Hz, 2H), 8.47 (d, J = 6.8 Hz, 2H), 4.41 (s, 3H). (s, 3H), 2.83 (s, 6H). ^{13}C NMR (75 MHz, DMSO d6): δ 151.43, 142.29, 129.47, 69.76. Spectral data are in accordance with literature.[11]

4-Bromo-1-methoxypyridin-1-ium tetrafluoroborate (1k)

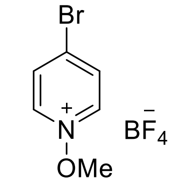

$C_6H_7BrBF_4NO$
MW = 274.97

Prepared according to **GP3** from 4-bromopyridine hydrochloride (3.2 g, 21,3 mmol, 1.0). Recrystallization from DCM (low temperature) afforded a white solid (3.5 g, 37%). M.p. = 78–80 ºC). **^{1}**H NMR (300 MHz, DMSO): 9.37 ppm (d, J = 9 Hz, 2H), 8.59 ppm (d, J = 6 Hz, 2H), 4.41 ppm (s, 3H). ^{13}C NMR (75 MHz, DMSO): 142.68, 142.14, 132.96, 70.12. IR (cm^{-1}): 3113.51, 1605.45, 11473.35, 1444.42 ,1015.34, 830.687. HRMS (ESI): m/z calculated for $C_6H_8BrNO^+$ $[M]^+$: 187.9700; found 187.9706.

2,6-Dichloro-1-methoxypyridin-1-ium tetrafluoroborate (1l)

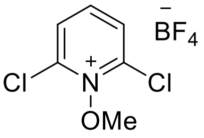

$C_6H_6BCl_2F_4NO$
MW = 285.62

Prepared from 2,6-dichloropyridine (1.0 g, 7.0 mmol) according to **GP4bis**. Recrystallization from DCM (low temperature) afforded colorless crystals. (1.26 g, 70%). ^{1}H NMR (300 MHz, CD_3CN): δ 8.29 (AX_{2q}, $\Delta\delta_{AX}$ = 0.27, $J_A = J_X$ = 8.4 Hz, 3H), 4.42 (s, 3H). ^{13}C NMR (75 MHz, CD_3CN): δ 148.81 (2C, Cq), 147.9 (CH), 130.6 (2C, CH), 69.8 (CH_3). HRMS (ESI): m/z calculated for $C_6H_6Cl_2NO^+$ $[M]^+$: 177.9821, found: 177.9817. Spectral data in accordance with literature.[15]

2,4-Dichloro-1-methoxypyridin-1-ium tetrafluoroborate (1m)

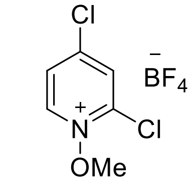

$C_6H_6BCl_2F_4NO$
MW = 285.62

Prepared from 2,4-dichloropyridine (843 mg, 5.7 mmol) according to **GP4bis**. Recrystallized from DCM (low temperature) afforded colorless crystals (966 mg, 66%). ^{1}H NMR (300 MHz DMSO- *d₆*): δ 9.72 (dd, J = 7.6, 1.9 Hz, 1H), 8.66 (dd, J = 6.2, 1.9 Hz, 1H), 8.53 (dd, J = 6.2, 1.9 Hz, 1H), 4.95 (s, 3H). ^{13}C NMR (75 MHz, DMSO- d_6): 152.15, 145.64, 144.11, 130.59, 127.98, 69.77. HRMS (ESI): m/z calculated for $C_6H_6Cl_2NO^+$ $[M]^+$: 177.9821, found: 177.9815.

2-Chloro-1-methoxypyridin-1-ium tetrafluoroborate (1n)

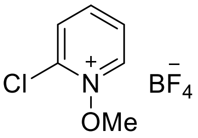

$C_6H_7ClBF_4NO$
MW = 231.02

Prepared according to **GP3** from 2,6-dimethylpyridine (910 mg, 8.5 mmol). Recrystallization from abs. EtOH afforded colorless crystals (786 mg, 74%).. ^{1}H NMR (300 MHz, DMSO-d_6): 9.64 (dd, J = 6.6, 1.5 Hz, 1H), 8.68 – 8.55 (m, 1H), 8.50 (dd, J = 8.3, 1.8 Hz, 1H), 8.21 (ddd, J = 7.5, 6.6, 1.9 Hz, 1H), 4.43 (s, 3H).^{13}C NMR (75 MHz, DMSO d6): δ 146.32, 144.44, 143.59, 130.83, 127.70, 69.62. HRMS (ESI): m/z calculated for $C_6H_8ClNO^+$ $[M]^+$: 144.0209, found 144.0211.

1-Methoxy-2-(trifluoromethyl)pyridin-1-ium tetrafluoroborate (1o)

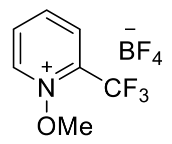

$C_7H_7BF_7NO^-$
MW = 264.94

To a solution of 2-(trifluomethyl)pyridine (2 g, 13.6 mmol) in $CHCl_3$ (50 mL, stab. with EtOH) was added *m*-CPBA (<77%, 3.65 g, 15.32 mmol) in one portion. The resulting solution was stirred at rt for 13 h. To the crude yellow solution was added aqueous Na_2CO_3 (sat.) until a pH of ~10 was reached. The biphasic mixture was transferred into a separatory funnel and the aqueous layer was extracted with chloroform (3 × 40 mL). The combined organic layers were washed with brine, dried over Na_2SO_4 and the volatiles were removed under reduced pressure to give the crude *N*-oxide. To a solution of the crude *N*-oxide (0.9 g5.5 mmol) in DCM (50 mL) was added trimethyloxonium tetrafluoroborate (1 g, 6.6 mmol) and the resulting mixture was stirred for 12 h. MeOH (1 mL) was added and, after stirring for another 15 min, the volatiles were removed under reduced pressure. The obtained solid was recrystallized from absolute ethanol to give 6-fluoro-1-methoxyquinolin-1-ium tetrafluoroborate (1.20 g, 82%). ^{1}H NMR (400 MHz, DMSO): δ 10.00 (dd, J = 6.5, 1.2 Hz, 1H), 8.89–8.84 (m, 1H), 8.76 (dd, J = 8.0, 1.9 Hz, 1H), 8.63 (ddd, J = 8.2, 6.5, 1.9 Hz, 1H), 4.58 (s, 3H). ^{13}C NMR (101 MHz, DMSO): δ 146.7 (CH_{Ar}), 144.3 (CH_{Ar}), 136.74 (q, J = 37.8 Hz, Cq), 133.2, 128.51 (q, J = 4.0 Hz, CH_{Ar}), 118.08 (q, J = 274.8 Hz, Cq), 70.8 (CH_3). ^{19}F NMR (282 MHz, DMSO): δ –63.3, –148.2 ($^{10}BF_4^-$), –148.3 ($^{11}BF_4^-$).

6-Fluoro-1-methoxyquinolin-1-ium tetrafluoroborate (1p)

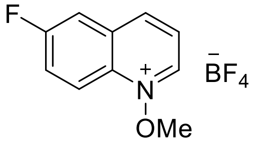

$C_{10}H_9BF_5NO$
MW = 264.99

To a solution of 6-fluoroisoquinoline (1 g, 6.79 mmol) in $CHCl_3$ (40 mL, stab. with EtOH) was added *m*-CPBA (<77%, 1.82 g, 8.15 mmol) in one portion. The resulting solution was stirred at rt for 13 h. To the crude yellow solution was added aqueous Na_2CO_3 (sat.) until a pH of ~10 was reached. The biphasic mixture was transferred into a separatory funnel and the aqueous layer was extracted with chloroform (3 × 30 mL). The combined organic layers were washed with brine, dried over Na_2SO_4 and the volatiles were removed under reduced pressure to give the crude *N*-oxide. To a solution of the crude *N*-oxide (1.11 g, 6.79 mmol) in DCM (50 mL) was added trimethyloxonium tetrafluoroborate (1.2 g, 8.15 mmol) and the resulting mixture was stirred for 12 h. MeOH (1 mL) was added and, after stirring for another 15 min, the volatiles were removed under reduced pressure. The obtained solid was recrystallized from absolute ethanol to give 6-fluoro-1-methoxyquinolin-1-ium tetrafluoroborate (1.20 g, 66%). Colorless needles. ^{1}H NMR (400 MHz, DMSO): δ 10.00 (dd, J = 6.2, 1.2 Hz, 1H), 9.23 (dd, J = 8.5, 1.0 Hz, 1H), 8.68 (dd, J = 9.6, 4.6 Hz, 1H), 8.43 (dd, J = 8.6, 2.8 Hz, 2H), 8.32–8.26 (m, 2H), 4.56 (s, 3H). ^{13}C NMR (101 MHz, DMSO): δ 161.45 (d, $^1J_{C\text{–}F}$ = 252.6 Hz, Cq), 145.54 (d, J = 5.3 Hz, CH_{Ar}), 144.00 (d, J = 2.3 Hz, CH_{Ar}), 133.1 (Cq), 132.10 (d, J = 11.5 Hz, Cq), 126.30 (d, $^2J_{C\text{–}F}$ = 27.0 Hz, CH_{Ar}), 123.53 (CH_{Ar}), 120.02 (d, J = 9.8 Hz, CH_{Ar}), 113.72 (d, $^2J_{C\text{–}F}$ = 23.7 Hz, CH_{Ar}), 69.92 (CH_3). ^{19}F NMR (376 MHz, DMSO): δ –106.77 (td, $^3J_{H\text{–}F}$ = 8.6, $^4J_{H\text{–}F}$ = 4.6 Hz), –148.2 ($^{10}BF_4^-$), –148.3 ($^{11}BF_4^-$).

2-Methoxyisoquinolin-2-ium tetrafluoroborate (1q)

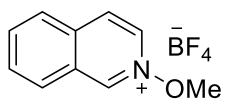

$C_{10}H_{10}BF_4NO$
MW = 247.00

Prepared according to **GP3** from isoquinoline 2-oxide (1g, 6,8 mmol). Recrystallization from abs. EtOH gave the desired product (675 mg, 68%). Colorless needles. (Full characterization found in *J. Am. Chem. Soc.* 2002, 124, 51, 15225–15238. ^{1}H NMR (400 MHz, DMSO): δ 10.49 (d, J = 2.2 Hz, 1H), 9.15 (dd, J = 7.2, 2.2 Hz, 1H), 8.71 (d, J = 7.2 Hz, 1H), 8.51 (dt, J = 8.4, 1.0, 1.0 Hz, 1H), 8.41 (dd, J = 8.4, 1.0 Hz, 1H), 8.29 (ddd, J = 8.3, 7.0, 1.2 Hz, 1H), 8.13 (ddd, J = 8.2, 7.0, 1.2 Hz, 1H), 4.52 (s, 3H). ^{13}C NMR (101 MHz, DMSO): δ 144.9, 136.8, 136.3, 131.6, 131.6, 130.3, 127.5, 127.5, 127.4, 69.1 (CH_3).

3 Alkylation of *N*-methoxypyridinium

3.1 Alkylation with organoboranes

General procedure 5 (GP5): alkylation with *B*-alkylcatecholboranes

To a stirred solution of alkene (1.4 mmol) and *N,N*-dimethylacetamide (28 μL, 0.3 mmol) in DCE (2.8 mL) was added dropwise catecholborane (0.32 mL, 3.0 mmol)) at 0 °C under argon. After complete addition, the solution was allowed to slowly reach rt and then stirred for 15 h. The next day, more DCE (1 mL) was added and the excess catecholborane was deactivated by adding dropwise *tert*-butanol (0.11 mL, 1.2 mmol) at 0 °C. Then, the solution was allowed to warm up to rt and then stirred for 1 h. Afterwards, the *N*-methoxyheteoarenium salt (1.0 mmol) was added in one portion followed by a DTBHN (3 mg, 1 mol%). In some cases, sym-collidine (0.4 mL, 3.0 mmol) was added. Three more portions of DTBHN (3 mg, 1mol% each) were added every hour and the resulting solution was stirred at 45 °C for additional 15h. The crude solution was filtered over a pad of basic aluminum oxide (h = 2 cm, Ø = 4 cm) and using EtOAc (50 mL) as eluent. The volatiles were removed under reduced pressure and the residue was purified by FC (solid deposition, EtOAc/heptanes) to afford the alkylated pyridine.

2-Cyclohexyl-4-phenylpyridine (2)

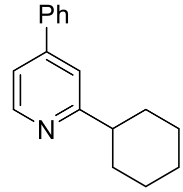

$C_{17}H_{19}N$
MW = 237.35

Prepared according to **GP5** from 1-methoxy-4-phenylpyridinium hexafluorophosphate (331 mg, 1.0 mmol) and cyclohexene (0.15 mL, 1.5 mmol) with sym-collidine, 0.4 mL, 3.0 mmol). FC, 12 g silica, solid deposition, (heptane/EtOAc 80:20) afforded **2** (149 mg, 63%). Colorless oil

^{1}H NMR (300 MHz, $CDCl_3$): δ 8.57 (d, J = 4.8 Hz, 1H), 7.67 – 7.60 (m, 2H), 7.53 – 7.39 (m, 3H), 7.39 – 7.34 (m, 1H), 7.31 (dd, J = 5.2, 1.7 Hz, 1H), 2.77 (tt, J = 11.8, 3.1 Hz, 1H), 2.08 – 1.95 (m, 2H), 1.95 – 1.82 (m, 2H), 1.82 – 1.71 (m, 1H), 1.60 (qd, J = 12.3, 3.1 Hz, 2H), 1.52 – 1.22 (m, 3H). ^{13}C NMR (75 MHz, $CDCl_3$): δ 166.9 (Cq), 149.5 (Cq), 149.2 (CH), 138.8 (Cq), 129.2 (2C, CH), 129.1 (CH), 127.2 (2C, CH), 119.5 (CH), 119.4 (CH), 46.6 (CH), 33.1 (2C, CH2), 26.7 (2C, CH2), 26.2 (CH2). IR (cm^{-1}): 3083 (w), 3059 (w), 3023 (w), 2922 (s), 2849 (s), 1594 (s).

HRMS (ESI): m/z calculated for $C_{17}H_{20}N^+$ $[M+H]^+$: 238.1590, found: 238.1583. Spectral data in accordance with literature.[16]

2-Cyclohexyl-4-methylquinoline (3)

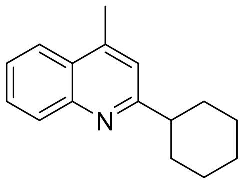

$C_{16}H_{19}N$
MW = 225.15

Prepared according to **GP5** from 1-methoxy-4-methylquinolinium tetrafluoroborate (263 mg, 1.0 mmol) and cyclohexene (0.15 mL, 1.5 mmol). FC (EtOAc/heptanes 5:95) afforded **3** (210 mg, 93%). Colorless oil. ^{1}H NMR (300 MHz, $CDCl_3$): δ 8.08 – 8.02 (m, 1H), 7.94 (dd, J = 8.3, 1.3 Hz, 1H), 7.66 (ddd, J = 8.3, 6.9, 1.3 Hz, 1H), 7.49 (ddd, J = 8.3, 6.9, 1.3 Hz, 1H), 7.16 (d, J = 0.7 Hz, 1H), 2.87 (tt, J = 11.9, 3.4 Hz, 1H), 2.68 (d, J = 0.7 Hz, 3H), 2.07 – 1.96 (m, 2H), 1.96 – 1.73 (m, 3H), 1.71 – 1.55 (m, 2H), 1.55 – 1.29 (m, 3H). ^{13}C NMR (75 MHz, $CDCl_3$): δ 166.6 (Cq), 147.8 (Cq), 144.3 (Cq), 129.6 (CH), 129.0 (CH), 127.2 (Cq), 125.5 (CH), 123.7 (CH), 120.4 (CH), 47.7 (CH), 33.0 (2C, CH2), 26.7 (2C, CH2), 26.3 (CH2), 18.9 (CH3). IR (cm^{-1}): 3060 (w), 3034 (w), 2920 (s), 2848 (s), 1601 (s), 1558 (s). HRMS (ESI): m/z calculated for $C_{16}H_{20}N^+$ $[M+H]^+$: 226.1590, found: 226.1591. Spectral data in accordance with literature.[17]

2-cyclododecyl-4-methylquinoline (4)

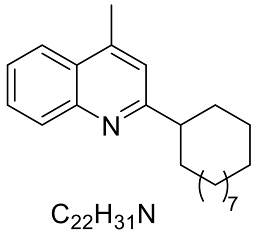

$C_{22}H_{31}N$
MW = 309.50

Prepared according to **GP5** from1-methoxy-4-methylquinolinium tetrafluoroborate (263 mg, 1.0 mmol), cyclododecene (97%, 0.31 mL, 1.5 mmol) FC (15 g silica, solid deposition, 3% EA/heptanes isocratic) afforded **4** (286 mg, 92%) Colorless amorphous solid. ^{1}H NMR (300 MHz, $CDCl_3$): δ 8.06 (d, J = 8.3 Hz, 1H), 7.94 (dd, J = 8.3, 1.6 Hz, 1H), 7.66 (ddd, J = 8.3, 6.9, 1.6 Hz, 1H), 7.48 (ddd, J = 8.3, 6.9, 1.6 Hz, 1H), 7.13 (d, J = 1.0 Hz, 1H), 3.18 – 3.03 (m, 1H), 2.68 (*d*, J = 1.0 Hz, 3H), 1.98 – 1.84 (m, 2H), 1.80 – 1.66 (m, 2H), 1.65 – 1.24 (m, 18H). ^{13}C NMR (75 MHz, $CDCl_3$): δ 166.8 (Cq), 147.9 (Cq), 143.8 (Cq), 129.8 (CH), 128.9 (CH), 127.1 (Cq), 125.4 (CH), 123.6 (CH), 121.6 (CH), 43.3 (CH), 30.3 (2C, CH2), 24.1 (2C, CH2), 24.0 (2C, CH2), 23.9 (2C, CH2), 23.5 (CH2), 23.1 (2C, CH2), 18.9 (CH3). IR (cm^{-1}): 3059 (w), 3034 (w), 2940 (m), 2925 (m), 2898 (m), 2858 (m). HRMS (ESI): m/z calculated for $C_{22}H_{32}N^+$ $[M+H]^+$: 310.2529, found: 310.2531.

2-*n*-Hexyl-4-methylquinoline (5)

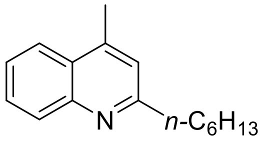

$C_{16}H_{21}N$
MW = 227.35

Prepared according to **GP5** from 1-methoxy-4-methylquinoline tetrafluoroborate (263 mg, 1.0 mmol) and 1-hexene (0.18 mL, 1.5 mmol, 1.5). FC (heptanes/EtOAc 95:5) afforded **5** accompanied by the branched isomer 2-(hexan-2-yl)-4-methylquinoline (170 mg, 75% combined yield, 1-hexyl/2-hexyl 6:1) together with the 2-hexyl isomer. Colorless liquid. ^{1}H NMR (300 MHz, $CDCl_3$): δ 8.04 (br d, J = 8.3 Hz, 1H), 7.94 (dd, J = 8.3, 1.5 Hz, 1H), 7.66 (ddd, J = 8.3, 6.9, 1.5 Hz, 1H), 7.49 (ddd, J = 8.3, 6.9, 1.5 Hz, 1H), 7.13 (br s, 1H), 2.95 – 2.88 (m, 2H), 2.67 (br s, 3H), 1.87 – 1.73 (m, 2H), 1.48 – 1.25 (m, 6H), 0.95 – 0.81 (m, 3H). Characteristic ^{1}H NMR signals for the minor

(branched) product: δ 8.06 (d, *J* = 8.6 Hz, 1H), 3.09 – 2.96 (m, 1H). ^{13}C NMR (75 MHz, $CDCl_3$): δ 162.8 (Cq), 147.8 (Cq), 144.2 (Cq), 129.4 (CH), 129.0 (CH), 126.8 (Cq), 125.4 (CH), 123.6 (CH), 122.1 (CH), 39.4 (CH_2), 31.8 (CH_2), 30.1 (CH2), 29.4 (CH_2), 22.7 (CH_2), 18.7 (CH_3), 14.2 (CH_3). Characteristic ^{13}C NMR signals for the minor (branched) product: δ 129.60 (CH), 128.9 (CH), 120.2 (CH), 43.0 (CH), 36.9 (CH_2), 30.0 (CH_2), 22.9 (CH_2), 20.9 (CH_3), 18.9 (CH_3). HRMS (ESI): m/z calculated for $C_{16}H_{22}N^+$ $[M+H]^+$: 228.1747, found: 228.1743.

2-*n*-Decyl-4-methylquinoline (6)

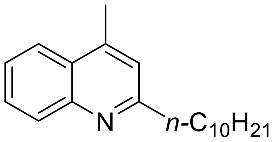

$C_{20}H_{29}N$
MW = 283.46

Prepared according to **GP5** from 1-methoxy-4-methylquinolinium tetrafluoroborate (261 mg, 1.0 mmol, 1.0) and 1-decene (0.27 mL, 1.5 mmol, 1.5). FC (heptanes/EtOAc 90:10) afforded **6** (207 mg, 73%) containing 6% of the regioisomer. Colourless oil. ^{1}H NMR (300 MHz, $CDCl_3$): δ 8.05 (dd, *J* = 8.4, 1.5 Hz, 1H), 7.92 (dd, *J* = 8.4, 0.8 Hz, 1H), 7.65 (ddd, *J* = 8.4, 6.9, 1.5 Hz, 1H), 7.47 (ddd, *J* = 8.4, 6.9, 1.3 Hz, 1H), 7.12 (d, *J* = 0.8 Hz, 1H), 2.97 – 2.86 (m, 2H), 2.65 (d, *J* = 0.7 Hz, 3H), 1.87 – 1.72 (m, 2H), 1.48 – 1.15 (m, 14H), 0.87 (t, *J* = 6.7 Hz, 3H). ^{13}C NMR (75 MHz, $CDCl_3$): δ 162.9 (Cq), 147.8 (Cq), 144.2 (Cq), 129.4 (CH), 129.1 (CH), 126.9 (Cq), 125.5 (CH), 123.7 (CH), 122.1 (CH), 39.4 (CH_2), 32.0 (CH_2), 30.2 (CH_2), 29.7 (4C, CH2), 29.4 (CH2), 22.8 (CH2), 18.8 (CH3), 14.2 (CH3). IR (cm^{-1}): 3061 (w), 3031 (w), 2952 (w), 2921 (s), 2852 (s), 1603 (s). HRMS (ESI): m/z calculated for $C_{20}H_{30}N^+$ $[M+H]^+$: 284.2373, found: 284.2363.

2-Ethyl-4-methylquinoline (7)

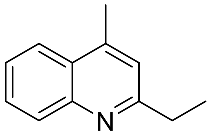

$C_{12}H_{13}N$
MW = 171.1

To a stirred solution of 1-methoxy-4-methylquinolinium tetrafluoroborate (261 mg, 1.0 mmol) in DCE (10 mL) was added a 2 M solution of BEt_3 in benzene (0.7 mL, 1.4 mmol) in one portion and DTBHN (3 mg, 1mol%). Three more portions of DTBHN (3 mg, 1mol% each) were added every hour. After further stirring for 15 hours, the crude solution was filtered over a pad of basic aluminum oxide (h = 2 cm, Ø = 4 cm) with EtOAc as eluent. The volatiles were removed under reduced pressure to give a crude oil. FC (heptanes/EtOAc 85:15) afforded **7** (135 mg, 79%). Light-yellow liquid. ^{1}H NMR (300 MHz, C_6D_6): δ 8.35 (dd, *J* = 8.4, 0.6 Hz, 1H), 7.63 (dd, *J* = 8.4, 1.0 Hz, 1H), 7.41 (ddd, *J* = 8.4, 6.9, 1.4 Hz, 1H), 7.22 (ddd, *J* = 8.4, 6.9, 1.4 Hz, 1H), 6.71 (d, *J* = 0.6 Hz, 1H), 2.88 (q, *J* = 7.6 Hz, 2H), 2.19 (s, 3H), 1.38 (t, *J* = 7.6 Hz, 3H). ^{13}C NMR (101 MHz, C_6D_6): δ 163.5 (Cq), 148.8 (Cq), 143.6 (Cq), 130.5 (CH), 129.0 (CH), 127.2 (Cq), 125.4 (CH), 123.7 (CH), 121.8 (CH), 32.3 (CH_2), 18.3 (CH_3), 13.8 (CH_3). Spectral data in accordance with literature.[18]

2-(2,3-Dimethylbutan-2-yl)-4-methylquinoline (8)

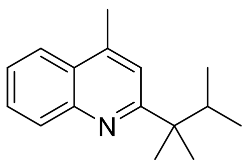

$C_{16}H_{21}N$
MW = 227.17

Prepared according to **GP5** from 1-methoxy-4-methylquinoline tetrafluoroborate (261 mg, 1.0 mmol) and 2,3-dimethylbut-2-ene (97% 0.18 mL, 1.5 mmol). FC (heptanes/EtOAc 98:2) afforded **8** (183 mg, 80%). Colorless oil. ^{1}H NMR (300 MHz, $CDCl_3$): δ 8.06 (br d, J = 8.3 Hz, 1H), 7.94 (dd, J = 8.3, 1.4 Hz, 1H), 7.65 (ddd, J = 8.3, 7.0, 1.4 Hz, 1H), 7.49 (ddd, J = 8.3, 7.0, 1.4 Hz, 1H), 7.30 (br s, 1H), 2.69 (d, J = 0.4 Hz, 3H), 2.29 (hept, J = 6.9 Hz, 1H), 1.38 (s, 6H), 0.81 (d, J = 6.9 Hz, 6H). ^{13}C NMR (75 MHz, $CDCl_3$): δ 169.0 (Cq), 147.4 (Cq), 143.2 (Cq), 130.2 (CH), 128.7 (CH), 126.6 (Cq), 125.4 (CH), 123.5 (CH), 119.7 (CH), 44.0 (Cq), 37.2 (CH), 23.8 (2C, CH_3), 19.1 (CH_3), 18.1 (2C, CH_3). IR (cm^{-1}): 3061 (w), 3036 (w), 2961 (s), 2935 (m), 2872 (m), 1600 (s). HRMS (ESI): m/z calculated for $C_{16}H_{2}2N^+$ [M+H]$^+$: 228.1747, found: 228.1745. Spectral data are in accordance with literature.[19]

***trans*-4-Methyl-2-(2-methylcyclohexyl)quinoline (9)**

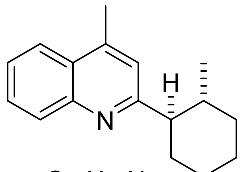

$C_{16}H_{21}N$
MW = 227.17

Prepared according to **GP5** from 1-methoxy-4-methylquinoline tetrafluoroborate (261 mg, 1.0 mmol) and 1-methylcyclohexene (0.17 mL, 1.5 mmol). FC (heptanes/EtOAc 97:3) afforded **9** (201 mg, 86%, *trans/cis* > 97:3). Colorless liquid. ^{1}H NMR (300 MHz, C_6D_6): δ 8.35 (dd, J = 8.4, 0.7 Hz, 1H), 7.65 (dd, J = 8.4, 1.4 Hz, 1H), 7.40 (ddd, J = 8.4, 6.9, 1.4 Hz, 1H), 7.22 (ddd, J = 8.4, 6.9, 1.4 Hz, 1H), 6.85 (d, J = 0.7 Hz, 1H), 2.53 (ddd, J = 11.7, 10.5, 3.8 Hz, 1H), 2.24 (d, J = 0.7 Hz, 3H), 2.21 – 2.06 (m, 1H), 2.02 – 1.69 (m, 5H), 1.51 – 1.25 (m, 2H), 1.19 – 1.02 (m, 1H), 0.83 (d, J = 6.5 Hz, 3H). ^{13}C NMR (75 MHz, C_6D_6): δ 166.0 (Cq), 148.9 (Cq), 143.6 (Cq), 130.6 (CH), 129.0 (Cq), 127.5 (CH), 125.4 (CH), 123.8 (CH), 121.9 (CH), 55.9 (CH), 36.8 (CH), 35.9 (CH_2), 34.3 (CH_2), 27.1 (CH_2), 27.0 (CH_2), 21.2 (CH_3), 18.5 (CH_3). IR (cm^{-1}): 3060 (w), 3033 (w), 2920 (s), 2850 (m), 1603 (s), 1559 (m). HRMS (ESI): m/z calculated for $C_{17}H_{22}N^+$ [M+H]$^+$: 240.1747; found: 240.1747.

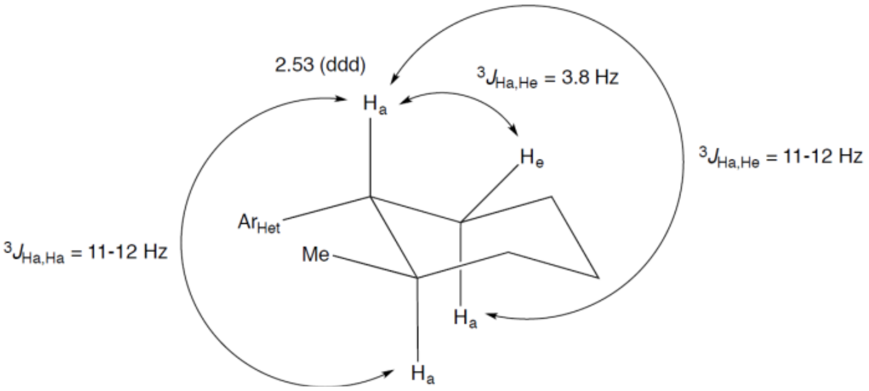

11-(4-Methylquinolin-2-yl)undecan-1-ol (10)

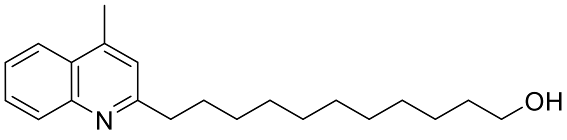

$C_{21}H_{31}NO$
MW = 313.49

Prepared according to **GP5** from 1-methoxy-4-methylquinoline tetrafluoroborate (263 mg, 1.0 mmol, 1.0 equiv) and 1-undecenol (258 mg, 1.5 mmol, 1.5 equiv), but using catecholborane (0.48 mL, 4.5 mmol, 4.5 equiv) and *t*-BuOH (3 mmol, 3 equiv). FC, 40 g silica, solid deposition, (heptanes/EtOAc 90:10 to 40:60) afforded **10** (204 mg, 65%) as a white solid, as well as its regioisomer 10-(4-methylquinolin-2-yl)undecan-1-ol (28 mg, 9%, contaminated with small amounts of the major isomer).

11-(4-Methylquinolin-2-yl)undecan-1-ol (major regioisomer, 65%). ^{1}H NMR (400 MHz, $CDCl_3$) δ 8.04 (dd, J = 8.5, 1.2 Hz, 1H), 7.92 (dd, J = 8.2, 1.4 Hz, 1H), 7.65 (ddd, J = 8.4, 6.8, 1.4 Hz, 1H), 7.48 (ddd, J = 8.2, 6.9, 1.3 Hz, 1H), 7.12 (bs, 1H), 3.62 (t, J = 6.7 Hz, 2H), 2.92–2.88 (m, 2H), 2.65 (d, J = 0.9 Hz, 3H), 2.18 (bs, 1H, OH), 1.82–1.74 (m, 2H), 1.58–1.51 (m, 2H), 1.44–1.19 (m, 14H). ^{13}C NMR (101 MHz, $CDCl_3$) δ 162.9 (Cq), 147.7 (Cq), 144.4 (Cq), 129.3 (CH_{Ar}), 129.1 (CH_{Ar}), 126.9 (Cq), 125.5 (CH_{Ar}), 123.7 (CH_{Ar}), 122.2 (CH_{Ar}), 62.9 (CH_2), 39.3 (CH_2), 32.9 (CH_2), 30.2 (CH_2), 29.68 (CH_2), 29.65 (CH_2), 29.59 (CH_2), 29.57 (CH_2), 29.50 (CH_2), 25.9 (CH_2), 18.8 (CH_3). White solid. M.p. 93–94 ºC. FT–IR (cm^{-1}, neat): 3264, 2913, 2841, 1708, 1600, 1562, 1506, 1470, 1463, 1445, 1410, 1375, 1363, 1330, 1314, 1272, 1186, 1174, 1157, 1111, 1098, 1064, 1025, 995, 965, 950, 931, 882, 864, 754, 717, 689, 676. HRMS (ESI): m/z calculated for $C_{21}H_{31}NO$ $[M+H]^+$: 314.2478, found: 314.2476.

10-(4-Methylquinolin-2-yl)undecan-1-ol (minor regioisomer, ca. 9%). ^{1}H NMR (400 MHz, $CDCl_3$) δ 8.06 (dd, J = 8.4, 1.4 Hz, 1H), 7.94 (dd, J = 8.2, 1.5 Hz, 1H), 7.66 (ddd, J = 8.4, 6.9, 1.5 Hz, 1H), 7.49 (ddd, J = 8.3, 6.9, 1.3 Hz, 1H), 7.13 (bs, 1H), 3.61 (t, J = 6.7 Hz, 2H), 3.03 (sext, J = 7.1 Hz, 1H), 2.68 (d, J = 0.9 Hz, 3H), 1.84–1.71 (m, 1H), 1.69–1.60 (m, 1H), 1.56–1.48 (m, 2H), 1.37–1.12 (m, 12H), 1.34 (d, J = 6.9 Hz, 3H). HRMS (ESI): m/z calculated for $C_{21}H_{31}NO$ $[M+H]^+$: 314.2478, found: 314.2469.

2-(3-((*tert*-Butyldiphenylsilyl)oxy)-2-methylpropyl)-4-methylquinoline (11)

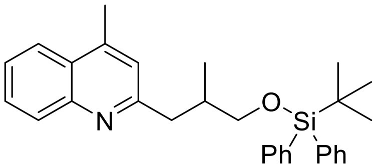

$C_{30}H_{35}NOSi$
MW = 453.70

Prepared according to **GP5** from 1-methoxy-4-methylquinolinium tetrafluoroborate (263 mg, 1.0 mmol, 1.0 equiv) and *tert*-butyl((2-methylallyl)oxy)diphenylsilane (466 mg, 1.5 mmol, 1.5 equiv). FC (heptanes/EtOAc 100:0 to 80:20) afforded **11** (360 mg, 79%). Colorless oil. ^{1}H NMR (300 MHz, $CDCl_3$): δ 8.06 (dd, J = 8.4, 1.2 Hz, 1H), 7.96 (dd, J = 8.4, 1.4 Hz, 1H), 7.70–7.66 (m, 5H), 7.51 (ddd, J = 8.3, 6.9, 1.3 Hz, 1H), 7.44–7.33 (m, 6H), 7.13 (d, J = 1.1 Hz, 1H), 3.63 (d, J = 5.6 Hz, 1H), 3.17 (dd, J = 13.3, 6.4 Hz, 1H), 2.78 (dd, J = 13.3, 8.3 Hz, 1H), 2.66 (d, J = 0.9 Hz, 3H), 2.35 (ddt, J = 12.1, 7.7, 6.1 Hz, 1H), 1.10 (s, 9H), 1.02 (d, J = 6.8 Hz, 3H). ^{13}C NMR (75 MHz, $CDCl_3$): δ 161.7 (Cq), 147.9 (Cq), 143.9 (Cq), 135.75 ($2CH_{Ar}$), 135.73 ($2CH_{Ar}$), 134.1 (Cq), 134.0 (Cq), 129.62 ($2CH_{Ar}$), 129.58

(CH_{Ar}), 129.0 (CH_{Ar}), 127.7 ($4CH_{Ar}$), 126.9 (Cq), 125.5 (CH_{Ar}), 123.7 (CH_{Ar}), 122.9 (CH_{Ar}), 68.5 (CH_2), 42.8 (CH_2), 36.9 (CH), 27.0 ($3CH_3$), 19.5 (Cq), 18.8 (CH_3), 17.0 (CH_3). FT–IR (cm^{-1}, neat): 2936, 2862, 2849, 1706, 1598, 1465, 1447, 1373, 1326, 1312, 1273, 1173, 1110, 1066, 1025, 999, 949, 936, 879, 862, 758, 714. HRMS (ESI): m/z calculated for $C_{30}H_{35}NOSi$ $[M+H]^+$: 454.2561, found: 454.2527.

4-Chloro-2-cyclohexylquinoline (12)

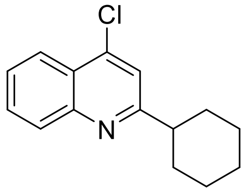

$C_{16}H_{19}N$
MW = 222.15

Prepared according to **GP5** from 1-methoxy-4-chloroquinolinium tetrafluoroborate (281 mg, 1.0 mmol) and cyclohexene (0.15 mL, 1.5 mmol). FC (heptanes/EtOAc 98:2) afforded **12** (211 mg, 85%). Colorless oil. ^1H NMR (300 MHz, $CDCl_3$): δ 8.17 (dd, J = 8.4, 0.6 Hz, 1H), 8.05 (dd, J = 8.5, 0.6 Hz, 1H), 7.72 (ddd, J = 8.4, 6.9, 1.3 Hz, 1H), 7.56 (ddd, J = 8.4, 6.9, 1.3 Hz, 1H), 7.42 (s, 1H), 2.89 (tt, J = 11.9, 3.4 Hz, 1H), 2.08 – 1.97 (m, 2H), 1.95 – 1.84 (m, 2H), 1.84 – 1.73 (m, 1H), 1.69 – 1.54 (m, 2H), 1.54 – 1.24 (m, 3H). ^{13}C NMR (75 MHz, $CDCl_3$): δ 166.9 (Cq), 148.8 (Cq), 142.7 (Cq), 130.2 (CH), 129.4 (Cq), 126.7 (CH), 125.2 (Cq), 124.0 (CH), 119.89 (CH), 47.5 (CH), 32.8 (2C, CH_2), 26.5 (2C, CH_2), 26.1 (CH_2). HRMS (ESI): m/z calculated for $C_{15}H_{17}NCl^+$ $[M+H]^+$: 246.1044, found: 246.1042. Spectral data in accordance with the literature.[20]

6-Cyclohexylphenanthridine (13)

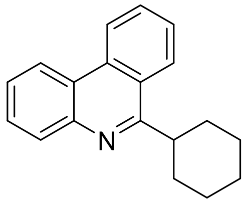

$C_{19}H_{19}N$
MW = 261.15

Prepared according to **GP5** from 5-methoxyphenanthridin-5-ium tetrafluoroborate (299 mg, 1.0 mmol) and cyclohexene (0.15 mL, 1.5 mmol). FC (heptanes/EtOAc 99.5:0.5 to 98:2) afforded **13** (183 mg, 70%). Colorless oil. ^1H NMR (300 MHz, $CDCl_3$): δ 8.65 (br d, J = 8.2 Hz, 1H), 8.54 (dd, J = 8.2, 1.3 Hz, 1H), 8.32 (br d, J = 8.2 Hz, 1H), 8.15 (dd, J = 8.2, 1.0 Hz, 1H), 7.81 (ddd, J = 8.3, 7.2, 1.3 Hz, 1H), 7.70 (tdd, J = 8.2, 7.2, 1.3, 2H), 7.60 (ddd, J = 8.2, 7.2, 1.3 Hz, 1H), 3.62 (tt, J = 11.1, 3.3 Hz, 1H), 2.16 – 1.79 (m, 7H), 1.71 – 1.35 (m, 3H). ^{13}C NMR (75 MHz, $CDCl_3$): δ 165.4 (Cq), 144.0 (Cq), 133.1 (Cq), 130.1 (CH), 130.0 (CH), 128.5 (CH), 127.2 (CH), 126.2 (Cq), 125.7 (CH), 124.9 (CH), 123.5 (Cq), 122.7 (CH), 121.9 (CH), 42.1 (CH), 32.4 (2C, CH_2), 27.0 (2C, CH_2), 26.5 (CH_2). HRMS (ESI): m/z calculated for $C_{19}H_{20}N^+$ $[M+H]^+$: 262.1590, found: 262.1589. Spectral data in accordance with literature.[21]

3-Bromo-2-cyclohexylquinoline (14)

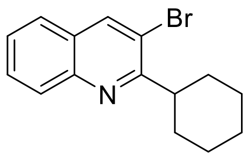

$C_{15}H_{16}BrN$
MW = 289.05

Prepared according to **GP5** from 1-methoxy-3-bromoquinolinium tetrafluoroborate (326 mg, 1.0 mmol) and cyclohexene (0.15 mL, 1.5 mmol). FC (heptanes/EtOAc 98:2) afforded **14** (110 mg, 38%). Light-orange solid. ^1H NMR (300 MHz, $CDCl_3$): δ 8.30 (d, J = 1.1 Hz, 1H), 8.07 – 7.99 (m, 1H), 7.73 – 7.63 (m, 2H), 7.48 (ddd, J = 8.1, 6.9, 1.1 Hz, 1H), 3.35 (tt, J = 11.7, 3.4 Hz, 1H), 2.04 – 1.20 (m, 10H). ^{13}C NMR (75 MHz, $CDCl_3$): δ 163.6 (CH), 146.8 (Cq), 138.8 (CH), 129.5 (CH), 129.3 (CH), 128.0 (Cq), 126.6 (CH), 126.5 (CH), 118.9 (Cq), 44.7 (CH3), 31.7 (2C, CH2), 26.7 (2C, CH2), 26.2 (CH2). HRMS (ESI): m/z calculated for $C_{15}H_{17}N_7{}^{79}Br^+$ [M+H]+: 290.0539,

found: 290.0536 HRMS (ESI): m/z calculated for $C_{15}H_{17}N^{81}Br^{+}$ $[M+H]^{+}$: 292.0518, found: 292.0514. Spectral data in accordance with literature.[22]

4-Cyclohexyl-2-methylquinoline (15)

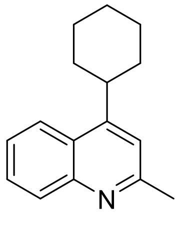

$C_{15}H_{16}N$
MW = 210.13

Prepared according to **GP5** from 1-ethoxy-4-methylquinolinium tetrafluoroborate (275 mg, 1.0 mmol) and cyclohexene (1.5 mL, 1.5 mmol) with sym-collidine, 0.4 mL, 3.0 mmol) . FC (heptanes/EtOAc 80:20) afforded **15** (95 mg, 42%). Cololress oil. ^{1}H NMR (300 MHz, $CDCl_3$): δ 8.07 – 7.99 (m, 2H), 7.64 (ddd, J = 8.4, 6.9, 1.4 Hz, 1H), 7.48 (ddd, J = 8.4, 6.9, 1.4 Hz, 1H), 7.16 (s, 1H), 3.29 (tt, J = 11.7, 3.2 Hz, 1H), 2.72 (s, 3H), 2.08 – 1.80 (m, 5H), 1.64 – 1.44 (m, 4H), 1.42 – 1.26 (m, 1H). ^{13}C NMR (75 MHz, $CDCl_3$): δ 158.9 (Cq), 153.4 (Cq), 148.2 (Cq), 129.6 (CH), 128.8 (CH), 125.3 (CH), 125.2 (Cq), 122.9 (CH), 118.4 (CH), 38.9 (CH), 33.6 (2C, CH_2), 27.0 (2C, CH2), 26.40 (CH_2), 25.6 (CH_3). Spectral data in accordance with literature.[23]

2-Dodecyl-4-phenylpyridine (16)

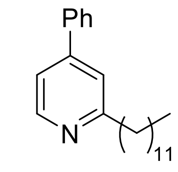

$C_{23}H_{33}N$
MW = 323.52

Prepared according to **GP5** from 1-methoxy-4-phenylpyridinium hexafluorophosphate (331 mg, 1.0 mmol) and 1-dodecene (94%, 0.33 mL, 1.4 mmol**)** with sym-collidine, 0.4 mL, 3.0 mmol). FC (pentane to pentane/Et_2O 70:30) afforded **16** (173 mg, 54%). Colourless oil. ^{1}H NMR (300 MHz, $CDCl_3$): δ 8.62 – 8.54 (m, 1H), 7.66 – 7.60 (m, 2H), 7.52 – 7.39 (m, 3H), 7.37 – 7.28 (m, 2H), 2.97 – 2.75 (m, 2H), 1.85 – 1.71 (m, 2H), 1.46 – 1.15 (m, 18H), 0.88 (t, J = 6.7 Hz, 3H). ^{13}C NMR (75 MHz, $CDCl_3$): δ 163.2 (Cq), 149.8 (CH), 148.7 (Cq), 138.72 (Cq), 129.1 (2C, CH), 129.0 (CH), 127.1 (2C, CH), 120.7 (CH), 119.1 (CH), 38.7 (CH2), 32.0 (CH_2), 30.1 (CH_2), 29.8 (CH_2), 29.8 (CH_2), 29.7 (2C, CH_2), 29.6 (CH_2), 29.6 (CH_2), 29.5 (CH_2), 22.8 (CH_2), 14.2 (CH_3). HRMS (ESI): m/z calculated for $C_{23}H_{34}N^{+}$ $[M+H]^{+}$: 324.2686, found: 324.2686.

2-(2,3-dimethylbutan-2-yl)-4-phenylpyridine (17)

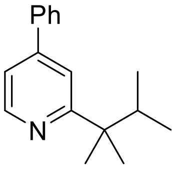

$C_{17}H_{21}N$
MW = 239.17

Prepared according to **GP5** from 1-methoxy-4-phenylpyridinium tetrafluorophosphate (278 mg, 1.0 mmol) and 2,3-dimethylbutene (98%, 0.17 mL, 1.5 mmol) with sym-collidine, 0.4 mL, 3.0 mmol). FC (heptanes/EtOAc 80:20) afforded 17 (30 mg, 13%). Colorless liquid. ^{1}H NMR (300 MHz, $CDCl_3$): δ 8.62 (br d, J = 5.1 Hz, 1H), 7.68 – 7.59 (m, 2H), 7.53 – 7.38 (m, 4H), 7.30 (dd, J = 5.1, 1.7 Hz, 1H), 2.25 (hept, J = 6.9 Hz, 1H), 1.34 (s, 6H), 0.80 (d, J = 6.9 Hz, 6H). ^{13}C NMR (75 MHz, $CDCl_3$): δ 169.7 (Cq), 149.0 (CH), 148.4 (Cq), 139.3 (Cq), 129.1 (2C, CH), 128.9 (CH), 127.3 (2C, CH), 118.8 (CH), 118.5 (CH), 43.5 (Cq), 37.3 (CH), 24.0 (2C, CH_3), 18.0 (2C, CH_3). HRMS (ESI): calculated for $C_{17}H_{22}N^{+}$ $[M+H]^{+}$: 240.1747, found: 240.1744.

2-Cyclohexyl-4-*tert*-butyl-pyridine (18)

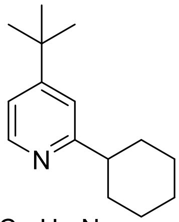

$C_{15}H_{23}N$
MW = 217.18

Prepared according to **GP5** from 1-methoxy-4-*tert*-butyl-pyridinium hexafluorophosphate (311 mg, 1.0 mmol) and cyclohexene (0.15 mL, 1.5 mmol) with sym-collidine, 0.4 mL, 3.0 mmol). FC (heptanes/EtOAc 80:20) afforded **18** (161 mg, 65%). Colorless liquid. ^{1}H NMR (300 MHz, $CDCl_3$): δ 8.42 (d, J = 5.3 Hz, 1H), 7.10 (d, J = 2.0 Hz, 1H), 7.07 (dd, J = 5.3, 2.0 Hz, 1H), 2.67 (tt, J = 11.8, 3.4 Hz, 1H), 2.00 – 1.68 (m, 5H), 1.65 – 1.21 (m, 5H), 1.29 (s, 9H). ^{13}C NMR (75 MHz, $CDCl_3$): δ 166.4 (Cq), 160.5 (Cq), 148.9 (CH), 118.4 (CH), 118.1 (CH), 46.9 (CH), 34.8 (Cq), 33.2 (2C, CH2), 30.7 (3C, CH_3), 26.8 (2C, CH_2), 26.2 (CH_2). Spectral data in accordance with literature.[24]

Ethyl 2-cyclohexylisonicotinate (19)

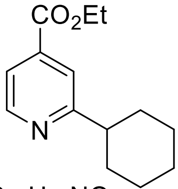

$C_{14}H_{19}NO_2$
MW = 233.14

Prepared according to **GP5** from 4-(ethoxycarbonyl)-1-methoxypyridin-1-ium hexafluorophosphate (337 mg, 1.0 mmol) and cyclohexene (0.15 mL, 1.5 mmol) with sym-collidine, 0.4 mL, 3.0 mmol). FC (heptanes/EtOAc 90:10) afforded **19** (107 mg, 43%). Colorless oil. ^{1}H NMR (300 MHz, $CDCl_3$): δ 8.65 (br d, J = 5.0 Hz, 1H), 7.70 (br s, 1H), 7.63 (dd, J = 5.1, 1.7 Hz, 1H), 4.40 (q, J = 7.2 Hz, 2H), 2.78 (tt, J = 11.9, 3.5 Hz, 1H), 2.02 – 1.91 (m, 2H), 1.91 – 1.81 (m, 2H), 1.80 – 1.69 (m, 1H), 1.64 – 1.19 (m, 5H), 1.40 (t, J = 7.2 Hz, 3H). ^{13}C NMR (75 MHz, $CDCl_3$): δ 167.8 (Cq), 165.7 (Cq), 149.9 (CH), 138.1 (Cq), 120.4 (CH), 120.3 (CH), 61.7 (CH_2), 46.7 (CH_3), 32.9 (2C, CH_2), 26.6 (2C, CH_2), 26.1 (CH_2), 14.3 (CH_3). Spectral data in accordance with literature.[25]

4-Methyl-2-((1R,2R,3R,5S)-2,6,6-trimethylbicyclo[3.1.1]heptan-3-yl) (20)

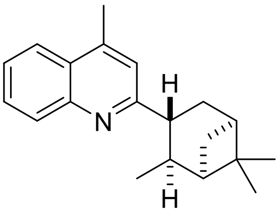

$C_{20}H_{25}N$
MW = 279.43

Prepared according to **GP5** from 1-methoxy-4-methylquinolinium tetrafluoroborate (263 mg, 1.0 mmol) and (+)-α-pinene (≥99%, 0.23 mL, 1.5 mmol). FC (heptanes/EtOAc 95:5) afforded **20** (239 mg, 87%, dr 99:1) Colorless oil. ^{1}H NMR (300 MHz, $CDCl_3$): δ 8.10 – 8.03 (m, 1H), 7.95 (dd, J = 8.3, 1.3 Hz, 1H), 7.67 (ddd, J = 8.3, 6.9, 1.3 Hz, 1H), 7.50 (ddd, J = 8.3, 6.9, 1.3 Hz, 1H), 7.30 (d, J = 0.4 Hz, 1H), 3.39 (app dt, J = 10.4, 7.5 Hz, 1H), 2.70 (d, J = 0.8 Hz, 3H), 2.57 – 2.33 (m, 3H), 2.13 – 2.02 (m, 2H), 1.97 – 1.90 (m, 1H), 1.36 (d, J = 9.6 Hz, 1H), 1.30 (s, 3H), 1.21 (s, 3H), 1.08 (d, J = 7.2 Hz, 3H). ^{13}C NMR (75 MHz, $CDCl_3$): δ 168.1 (Cq), 147.6 (Cq), 144.3 (Cq), 129.8 (CH), 129.0 (CH), 126.9 (Cq), 125.5 (CH), 123.6 (CH), 121.6 (CH), 48.1 (CH), 47.3 (CH), 43.7 (CH), 41.9 (CH), 39.4 (Cq), 35.4 (CH_2), 34.4 (CH_2), 28.6 (CH_3), 23.0 (CH_3), 21.5 (CH_3), 19.0 (CH_3). IR (cm^{-1}): 3062 (w), 3033 (w), 2948 (m), 2898 (m), 2868 (m), 1601 (s). HRMS (ESI): m/z calculated for $C_{20}H_{26}N^+$ $[M+H]^+$: 280.2060, found: 280.2050. Spectral and physical data in accordance with literature reported from *ent*-**20**.[17]

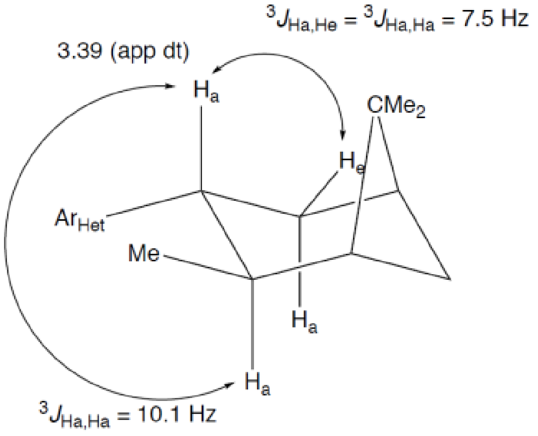

2-(((1*S*,2*S*,5*S*)-6,6-Dimethylbicyclo[3.1.1]heptan-2-yl)methyl)-4-methylquinoline (21)

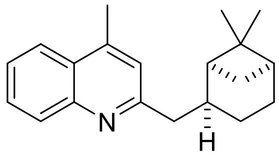

$C_{20}H_{25}N$
MW = 279.43

Prepared according to **GP5** from 1-methoxy-4-methylquinolinium tetrafluoroborate (261 mg, 1.0 mmol) and (–)-*β*-pinene (99%, 0.22 mL, 1.5 mmol). FC (heptanes/EtOAc 95:5) afforded **21** (222 mg, 78%, dr 9:1 determined by GC-MS). Colorless oil ^{1}H NMR (300 MHz, $CDCl_3$): *δ* 7.93 (d, *J* = 8.3 Hz, 1H), 7.83 (dd, *J* = 8.3, 1.2 Hz, 1H), 7.55 (ddd, *J* = 8.3, 6.9, 1.2 Hz, 1H), 7.38 (ddd, *J* = 8.3, 6.9, 1.2 Hz, 1H), 6.99 (s, 1H), 2.91 (s, 1H), 2.88 (s, 1H), 2.63 – 2.50 (m, 1H), 2.56 (d, *J* = 0.6 Hz, 3H), 2.26 – 2.16 (m, 1H), 1.92 – 1.67 (m, 5H), 1.60 – 1.48 (m, 1H), 1.10 (s, 6H), 0.81 (d, *J* = 9.6 Hz, 1H). ^{13}C NMR (75 MHz, $CDCl_3$): *δ* 162.2 (Cq), 148.0 (Cq), 143.8 (Cq), 129.6 (CH), 129.0 (CH), 126.9 (Cq), 125.5 (CH), 123.7 (CH), 123.0 (CH), 46.6 (CH_2), 46.2 (CH), 42.1 (CH), 41.6 (CH), 39.0 (Cq), 33.9 (CH_2), 28.3 (CH_3), 26.6 (CH_2), 23.7 (CH_3), 22.1 (CH_2), 18.8 (CH_3). HRMS (ESI): m/z calculated for $C_{20}H_{26}N^+$ [M+H]$^+$: 280.2060, found: 280.2060.

2-(2-(2-(Benzyloxy)ethyl)-6,6-dimethylbicyclo[3.1.1]heptan-3-yl)-4-methylquinoline (22)

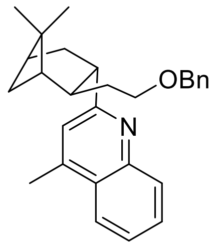

$C_{28}H_{33}NO$
MW = 399.58

Prepared according to **GP5** from 1-methoxy-4-methylquinoline tetrafluoroborate (263 mg, 1.0 mmol, 1.0 equiv) and 2-(2-(benzyloxy)ethyl)-6,6-dimethylbicyclo[3.1.1]hept-2-ene (385 mg, 1.5 mmol, 1.5 equiv). FC, 40 g silica, solid deposition, (heptanes/EtOAc 95:5 to 85:15) afforded **22** (354 mg, 89%, dr > 95:5) as a yellow oil. ^{1}H NMR (400 MHz, C_6D_6): *δ* 8.32 (dd, *J* = 8.4, 2.0 Hz, 1H), 7.64 (dd, *J* = 8.3, 1.4 Hz, 1H), 7.42 (ddd, *J* = 8.3, 6.8, 1.4 Hz, 1H), 7.23 (ddd, *J* = 8.2, 6.9, 1.3 Hz, 1H), 7.10–7.00 (m, 5H), 6.99 (d, *J* = 1.1 Hz, 1H), 4.04 (d, *J* = 11.9 Hz, 1H), 3.96 (d, *J* = 11.9 Hz, 1H), 3.52 (dt, *J* = 10.5, 7.1, 1H), 3.38–3.29 (m, 2H), 3.04 (qd, J = 7.5, 2.0 Hz, 1H), 2.55–2.45 (m, 2H), 2.21 (d, *J* = 1.0 Hz, 1H), 2.18 (ddd, *J* = 13.6, 6.7, 2.5 Hz, 1H), 2.11 (ddd, *J* = 6.5, 5.2, 1.9 Hz, 1H), 2.03 (dq, *J* = 13.3, 6.6 Hz, 1H), 2.00–1.97 (m, 1H), 1.94 (ddt, *J* = 13.7, 7.9, 6.8 Hz, 1H), 1.71 (d, *J* = 9.4 Hz, 1H), 1.26 (s, 3H), 1.17 (s, 3H). ^{13}C NMR (101 MHz, C_6D_6): *δ* 168.4 (Cq), 148.8 (Cq), 143.9 (Cq), 139.4 (Cq), 130.5 (CH_{Ar}), 129.0 (CH_{Ar}), 128.3 ($2CH_{Ar}$), 127.5 ($2CH_{Ar}$), 127.3 (CH_{Ar}), 127.2 (Cq), 125.4 (CH_{Ar}), 123.8 (CH_{Ar}), 122.5 (CH_{Ar}), 72.7 (CH_2), 69.3 (CH_2),

46.1 (CH), 44.5 (CH), 42.1 (CH), 39.3 (CH), 37.3 (CH_2), 36.2 (CH_2), 33.5 (CH_2), 28.4 (CH_3), 23.2 (CH_3), 18.5 (CH_3). $[\alpha]_D^{20}$ –2.8 (*c* 1.1, $CHCl_3$). FT–IR (cm^{-1}, neat): 2929, 2860, 2845, 1708, 1599, 1558, 1506, 1465, 1449, 1410, 1373, 1363, 1326, 1311, 1273, 1172, 1157, 1147, 1109, 1097, 1067, 1024, 999, 949, 934, 859, 761, 716, 675. HRMS (ESI): m/z calculated for $C_{28}H_{33}NO$ $[M+H]^+$: 400.2635, found: 400.2575.

1-(2-(2-(benzyloxy)ethyl)-6,6-dimethylbicyclo[3.1.1]heptan-3-yl)isoquinoline (23)

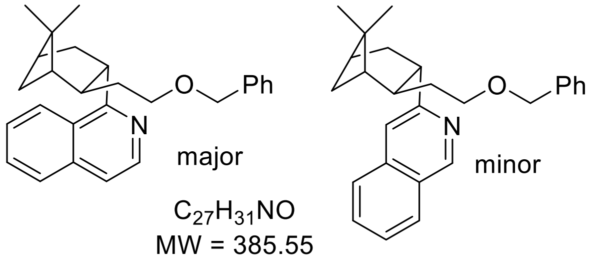

Prepared according to **GP5** from 2-methoxyisoquinolin-2-ium tetrafluoroborate (247 mg, 1.0 mmol, 1.0 equiv) and 2-(2-(benzyloxy)ethyl)-6,6-dimethylbicyclo[3.1.1]hept-2-ene (385 mg, 1.5 mmol, 1.5 equiv). FC (heptanes/EtOAc 95:5 to 30:70) afforded **23** (130 mg, 34%, dr > 95:5) and a second product tentatively assigned as **C_3-23** (30 mg, 8%, dr > 95:5). Colorless oils.

1-(2-(2-(Benzyloxy)ethyl)-6,6-dimethylbicyclo[3.1.1]heptan-3-yl)isoquinoline (**23**, major regioisomer, 34%). ^1H NMR (400 MHz, $CDCl_3$): δ 8.55 (d, *J* = 5.6 Hz, 1H), 8.26 (d, *J* = 8.4 Hz, 1H), 7.82 (dd, *J* = 8.2, 1.4 Hz, 1H), 7.66 (ddd, *J* = 8.1, 6.8, 1.2 Hz, 1H), 7.59 (ddd, *J* = 8.3, 6.8, 1.4 Hz, 1H), 7.48 (dd, *J* = 5.6, 0.9 Hz, 1H), 7.25–7.18 (m, 3H), 7.13–7.05 (m, 2H), 4.22 (dt, *J* = 10.7, 6.8, 6.8 Hz, 1H), 4.12 (d, *J* = 11.7 Hz, 1H), 4.07 (d, *J* = 11.8 Hz, 1H), 3.29–3.16 (m, 3H), 2.63–2.55 (m, 1H), 2.43 (dtd, J = 9.6, 6.3, 6.2, 2.1 Hz, 1H), 2.13 (ddd, J = 6.6, 5.0, 2.0 Hz, 1H), 2.02 (tt, *J* = 5.8, 5.8, 3.0, 3.0 Hz, 1H), 1.93–1.75 (m, 3H), 1.56 (d, *J* = 9.5 Hz, 1H), 1.32 (s, 3H), 1.31 (s, 3H). ^{13}C NMR (101 MHz, $CDCl_3$): δ 167.4 (Cq), 142.3 (Cq), 138.5 (Cq), 136.6 (Cq), 129.7 (CH_{Ar}), 128.3 ($2CH_{Ar}$), 127.7 ($2CH_{Ar}$), 127.6 (CH_{Ar}), 127.4 (CH_{Ar}), 127.3 (Cq), 127.1 (CH_{Ar}), 125.1 (CH_{Ar}), 118.7 (CH_{Ar}), 72.8 (CH_2), 69.5 (CH_2), 46.1 (CH), 42.7 (CH), 41.9 (CH), 39.1 (Cq), 39.0 (CH), 37.2 (CH_2), 36.5 (CH_2), 32.5 (CH_2), 28.2 (CH_3), 23.5 (CH_3). $[\alpha]_D^{20}$ +16 (*c* 0.9, $CHCl_3$). FT–IR (cm^{-1}, neat): 3051, 3028, 2993, 2913, 2853, 1621, 1585, 1560, 1496, 1471, 1453, 1380, 1358, 1323, 1296, 1261, 1218, 1203, 1144, 1095, 1028, 1014, 907, 867, 821, 798, 736, 697, 677. HRMS (ESI): m/z calculated for $C_{27}H_{31}NO$ $[M+H]^+$: 386.2478, found: 386.2471.

Minor regioisomer, 8% (tentatively **C3-23**). ^1H NMR (400 MHz, $CDCl_3$): δ 8.24 (dd, *J* = 8.1, 1.5 Hz, 1H), 8.18 (d, *J* = 8.4 Hz, 1H), 8.10 (s, 1H), 7.64–7.55 (m, 2H), 7.21–7.14 (m, 3H), 7.10–7.05 (m, 2H), 4.20–4.08 (m, 3H), 3.26–3.14 (m, 2H), 3.06 (q, *J* = 8.4, 7.5 Hz, 1H), 2.53–2.46 (m, 1H), 2.38–2.30 (m, 1H), 2.09–2.04 (m, 1H), 2.00–1.96 (m, 1H), 1.89–1.79 (m, 2H), 1.72 (ddt, *J* = 13.3, 8.3, 6.4 Hz, 1H), 1.52 (d, *J* = 9.6 Hz, 1H), 1.28 (s, 3H), 1.27 (s, 3H). ^{13}C NMR (101 MHz, $CDCl_3$): δ 157.8 (Cq), 147.3 (Cq), 138.4 (Cq), 129.0 (CH_{Ar}), 128.3 ($2CH_{Ar}$), 128.1 (Cq), 127.8 ($2CH_{Ar}$), 127.6 (CH_{Ar}), 127.4 (CH_{Ar}), 125.3 (CH_{Ar}), 124.9 (CH_{Ar}), 122.4 (CH_{Ar}), 72.8 (CH_2), 69.5 (CH_2), 46.0 (CH), 42.9 (CH), 42.0 (CH), 39.3 (Cq), 38.8 (CH), 36.8 (CH_2), 36.5 (CH_2), 33.2 (CH_2), 28.5 (CH_3), 23.5 (CH_3).

2-(2-(2-(Benzyloxy)ethyl)-6,6-dimethylbicyclo[3.1.1]heptan-3-yl)-6-fluoroquinoline (24)

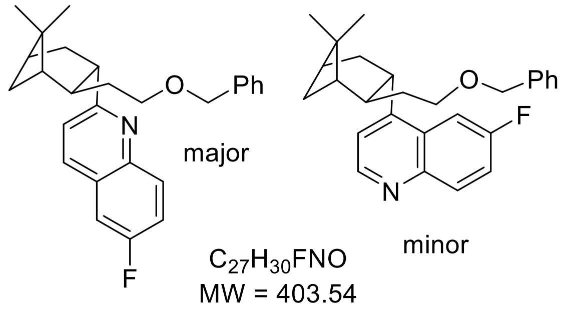

Prepared according to **GP5** from 6-fluoro-1-methoxyquinolin-1-ium tetrafluoroborate (265 mg, 1.0 mmol, 1.0 equiv) and 2-(2-(benzyloxy)ethyl)-6,6-dimethylbicyclo[3.1.1]hept-2-ene (385 mg, 1.5 mmol, 1.5 equiv). FC (heptanes/EtOAc 95:5 to 10:90) afforded **24** (338 mg, 59%, dr > 95:5) and **C$_4$-24** (39 mg, ca. 10%, dr > 95:5, contaminated with two unidentified, fluorinated impurities). Colorless oil.

2-(2-(2-(Benzyloxy)ethyl)-6,6-dimethylbicyclo[3.1.1]heptan-3-yl)-6-fluoroquinoline (**24**, major regioisomer, 59%). ^{1}H NMR (400 MHz, $CDCl_3$): δ 8.05 (dd, J = 9.3, 5.3 Hz, 1H), 8.00 (d, J = 8.6 Hz, 1H), 7.48 (d, J = 9.3 Hz, 1H), 7.44 (dt, J = 6.4, 2.8 Hz, 1H), 7.37 (dd, J = 8.9, 2.8 Hz, 1H), 7.21–7.16 (m, 3H), 7.06–6.99 (m, 2H), 4.14 (d, J = 11.8 Hz, 1H), 4.07 (d, J = 11.8 Hz, 1H), 3.51 (dt, J = 10.7, 7.3 Hz, 1H), 3.40–3.31 (m, 2H), 2.65–2.48 (m, 3H), 2.09–2.04 (m, 2H), 1.98 (ddd, J = 13.5, 7.0, 2.1 Hz, 1H), 1.94–1.81 (m, 2H), 1.38 (d, J = 9.7 Hz, 1H), 1.30 (s, 3H), 1.21 (s, 3H). ^{13}C NMR (101 MHz, $CDCl_3$): δ 168.00 (d, $^6J_{C\text{–}F}$ = 2.7 Hz, Cq), 160.1 (d, $^1J_{C\text{–}F}$ = 246.5 Hz, Cq), 144.8 (Cq), 138.5 (Cq), 135.88 (d, $^3J_{C\text{–}F}$ = 5.3 Hz, CH_{Ar}), 131.5 (d, J = 9.1 Hz, CH_{Ar}), 128.2 ($2CH_{Ar}$), 127.5 ($2CH_{Ar}$), 127.4 (CH_{Ar}), 127.2 (d, $^3J_{C\text{–}F}$ = 9.7 Hz, Cq), 121.8 (CH_{Ar}) 119.3 (d, $^2J_{C\text{–}F}$ = 25.5 Hz, CH_{Ar}), 110.5 (d, $^2J_{C\text{–}F}$ = 21.5 Hz, CH_{Ar}), 72.6 (CH_2), 68.8 (CH_2), 46.1 (CH), 45.7 (CH), 44.6 (CH), 41.8 (CH), 39.2 (Cq), 36.5 (CH_2), 36.1 (CH_2), 33.7 (CH_2), 28.4 (CH_3), 23.1 (CH_3). $[\alpha]_D^{20}$ –3 (c 1.0, $CHCl_3$). FT–IR (cm^{-1}, neat): 3066, 3032, 2990, 2913, 2857, 1625, 1606, 1562, 1500, 1475, 1451, 1383, 1363, 1232, 1215, 1140, 1096, 1026, 962, 889, 864, 830, 814, 733, 695, 670, 626, 612. HRMS (ESI): m/z calculated for: $C_{27}H_{30}FNO$ $[M+H]^+$ 404.2384, found: 404.2357.

4-(2-(2-(Benzyloxy)ethyl)-6,6-dimethylbicyclo[3.1.1]heptan-3-yl)-6-fluoroquinoline (**C$_4$-24**, minor regioisomer, ca. 10%, *contaminated with unidentified fluorinated impurities*). ^{1}H NMR (400 MHz, $CDCl_3$): δ 8.73 (d, J = 4.7 Hz, 1H), 8.03 (dd, J = 9.2, 5.7 Hz, 1H), 7.64 (dd, J = 10.8, 2.8 Hz, 1H), 7.41 (d, J = 4.9 Hz, 1H), 7.38 (ddd, J = 9.2, 7.8, 2.7 Hz, 1H), 7.18–7.07 (m, 3H), 7.00–6.94 (m, 2H), 3.98–3.90 (m, 2H), 3.76 (dt, J = 10.5, 7.6 Hz, 1H), 3.13 (dt, J = 9.5, 6.5 Hz, 1H), 3.04 (dt, J = 9.4, 6.6 Hz, 1H), 2.59–2.52 (m, 2H), 2.43 (dtd, J = 9.9, 6.2, 2.1 Hz, 1H), 2.04 (ddd, J = 6.6, 5.2, 1.9 Hz, 1H), 1.97–1.93 (m, 1H), 1.73 (dq, J = 13.8, 6.7 Hz, 1H), 1.61 (dq, J = 13.5, 6.5, Hz, 1H), 1.57 (ddd, J = 13.7, 7.4, 2.4 Hz, 1H), 1.21 (s, 3H), 1.18 (s, 3H), 1.09 (d, J = 9.7 Hz, 1H).^{13}C NMR (101 MHz, $CDCl_3$): δ 160.8 (d, $^1J_{C\text{–}F}$= 246.7 Hz, Cq), 155.6 (d, $^3J_{C\text{–}F}$ = 5.8 Hz, Cq), 149.96 (d, $^3J_{C\text{–}F}$ = 2.6 Hz, CH_{Ar}), 145.7 (Cq), 138.3 (Cq), 133.0 (CH_{Ar}), 132.9 (CH_{Ar}), 128.3 ($2CH_{Ar}$), 127.6 ($2CH_{Ar}$), 127.5 (CH_{Ar}), 119.4 (CH_{Ar}), 119.27 (d, $^3J_{C\text{–}F}$ = 25.7 Hz, CH_{Ar}), 106.98 (d, $^3J_{C\text{–}F}$ = 22.7 Hz, CH_{Ar}), 72.8 (CH_2), 68.5 (CH_2), 46.0 (CH), 44.9 (CH), 41.9 (CH), 39.1 (Cq), 38.1 (CH_2), 37.0 (CH_2), 36.7 (CH), 34.1 (CH_2), 28.3 (CH_3), 23.5 (CH_3). $C_{27}H_{30}FNO$ $[M+H]^+$ 404.2384, found: 404.2369.

2-(2-(2-(Benzyloxy)ethyl)-6,6-dimethylbicyclo[3.1.1]heptan-3-yl)-6-chloropyridine (25)

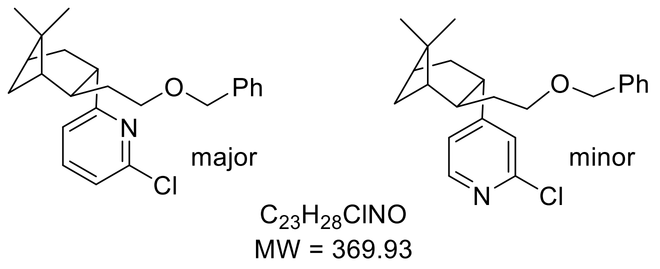

Prepared according to **GP5** from 1-methoxy-4-methylquinolinium tetrafluoroborate (263 mg, 1.0 mmol, 1.0 equiv) and 2-(2-(benzyloxy)ethyl)-6,6-dimethylbicyclo[3.1.1]hept-2-ene (385 mg, 1.5 mmol, 1.5 equiv). FC (heptanes/EtOAc 95:5 to 85:15) afforded **25** (109 mg, 29%, dr > 95:5) and **C_4-25** (33 mg, 8%, dr > 95:5). Colorless oils.

2-(2-(2-(Benzyloxy)ethyl)-6,6-dimethylbicyclo[3.1.1]heptan-3-yl)-6-chloropyridine (**25**, major regioisomer, 29%). ^{1}H NMR (400 MHz, $CDCl_3$): δ 7.41 (t, J = 7.7 Hz, 1H), 7.24–7.13 (m, 3H), 7.11–7.09 (m, 2H), 7.06 (d, J = 7.6 Hz, 1H), 7.00 (d, J = 7.8 Hz, 1H), 4.16 (s, 2H), 3.26 (t, J = 6.8 Hz, 1H), 3.15 (dt, J = 10.4, 7.1 Hz, 1H), 2.39–2.31 (m, 3H), 1.92–1.90 (m, 2H), 1.82 (ddd, J = 13.2, 6.8, 1.9 Hz, 1H), 1.72 (q, J = 7.0 Hz, 1H), 1.25 (d, J = 9.7 Hz, 1H), 1.17 (s, 3H), 1.04 (s, 3H). ^{13}C NMR (101 MHz, $CDCl_3$): δ 169.6 (Cq), 150.8 (Cq), 138.9 (CH_{Ar}), 138.6 (Cq), 128.3 (2CH_{Ar}), 127.6 (2CH_{Ar}), 127.5 (CH_{Ar}), 121.2 (CH_{Ar}), 120.9 (CH_{Ar}), 72.8 (CH_2), 68.9 (CH_2), 45.3 (CH), 45.1 (CH), 44.9 (CH), 41.6 (CH), 39.0 (Cq), 36.3 (CH_2), 36.1 (CH_2), 33.4 (CH_2), 28.3 (CH_3), 23.0 (CH_3). $[\alpha]_D^{20}$ +25 (c 1.2, $CHCl_3$). FT–IR (cm^{-1}, neat): 2929, 2860, 2845, 1708, 1599, 1580, 1556, 1508, 1494, 1469, 1447, 1432, 1411, 1381, 1364, 1329, 1310, 1273, 1173, 1159, 1134, 1109, 1096, 1069, 1026, 986, 952, 788, 760, 736, 716, 697,610. HRMS (ESI): m/z calculated for $C_{23}H_{28}ClNO$ $[M+H]^+$: 370.1932, found: 370.1909.

4-(2-(2-(Benzyloxy)ethyl)-6,6-dimethylbicyclo[3.1.1]heptan-3-yl)-2-chloropyridine (**C_4-25**, minor regioisomer, 8%). ^{1}H NMR (400 MHz, $CDCl_3$) δ 8.19 (d, J = 5.1 Hz, 1H), 7.26–7.15 (m, 4H), 7.12–7.09 (m, 2H), 7.07 (dd, J = 5.2, 1.6 Hz, 1H), 4.19 (s, 2H), 3.27–3.19 (m, 2H), 2.98 (dt, J = 10.4, 7.6 Hz, 1H), 2.46–2.40 (m, 1H), 2.37 (ddt, J = 13.4, 10.5, 2.2 Hz, 1H), 2.14 (tdd, J = 8.0, 6.2, 1.6 Hz, 1H), 1.97–1.93 (m, 2H), 1.74–1.60 (m, 3H), 1.19 (s, 3H), 1.05 (s, 3H), 0.99 (d, J = 9.9 Hz, 1H). ^{13}C NMR (101 MHz, $CDCl_3$) δ 162.1 (Cq), 151.8 (Cq), 149.7 (CH_{Ar}), 138.4 (Cq), 128.4 (2CH_{Ar}), 127.7 (2CH_{Ar}), 127.6 (CH_{Ar}), 124.0 (CH_{Ar}), 122.5 (CH_{Ar}), 72.9 (CH_2), 68.5 (CH_2), 45.9 (CH), 45.1 (CH), 43.0 (CH), 41.6 (CH), 39.0 (Cq), 37.6 (CH_2), 36.1 (CH_2), 34.4 (CH_2), 28.3 (CH_3), 23.1 (CH_3). $[\alpha]_D^{20}$ +14 (c 0.6, $CHCl_3$). FT–IR (cm^{-1}, neat): 2910, 2860, 1589, 1539, 1497, 1462, 1452, 1390, 1364, 1212, 1119, 1095, 1084, 1027, 987, 961, 940, 875, 841, 814, 733, 694, 666, 656, 616, 594. HRMS (ESI): m/z calculated for $C_{23}H_{28}ClNO$ $[M+H]^+$: 370.1932, found: 370.1917.

2-(2-(2-(Benzyloxy)ethyl)-6,6-dimethylbicyclo[3.1.1]heptan-3-yl)-6-(trifluoromethyl)pyridine (26) and 4-(2-(2-(benzyloxy)ethyl)-6,6-dimethylbicyclo[3.1.1]heptan-3-yl)-2-(trifluoromethyl)pyridine (C_4-26)

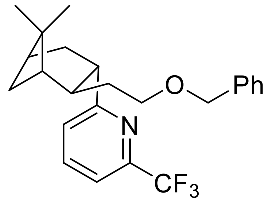

major

$C_{24}H_{28}F_3NO$
MW = 403.49

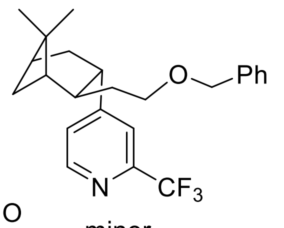

minor

Prepared according to **GP5** from 1-methoxy-2-(trifluoromethyl)pyridin-1-ium tetrafluoroborate (265 mg, 1.0 mmol, 1.0 equiv) and 2-(2-(benzyloxy)ethyl)-6,6-dimethylbicyclo[3.1.1]hept-2-ene (385 mg, 1.5 mmol, 1.5 equiv). FC (heptanes/EtOAc 95:5 to 85:15) afforded **26** (105 mg, 26%, dr > 95:5) and **C_4-26** (84 mg, 21%, dr > 95:5). Colorless oils.

2-(2-(2-(Benzyloxy)ethyl)-6,6-dimethylbicyclo[3.1.1]heptan-3-yl)-6-(trifluoromethyl)pyridine (**26**, major regioisomer, 26%). ^{1}H NMR (400 MHz, $CDCl_3$): δ 7.61 (t, J = 7.8 Hz, 1H), 7.35 (d, J = 7.7 Hz, 1H), 7.32 (d, J = 7.9 Hz, 1H), 7.21–7.13 (m, 3H), 7.07–7.04 (m, 2H), 4.16–4.09 (m, 2H), 3.29–3.21 (m, 1H), 3.25 (t, J = 6.9 Hz, 2H), 2.42–2.32 (m, 3H), 1.92 (d, J = 6.2 Hz, 2H), 1.85 (dd, J = 12.7, 6.7 Hz, 1H), 1.74 (q, J = 7.0 Hz, 2H), 1.30 (d, J = 9.6 Hz, 1H), 1.18 (s, 3H), 1.06 (s, 3H). ^{13}C NMR (101 MHz, $CDCl_3$): δ 169.4 (Cq), 147.76 (q, $^2J_{C-F}$ = 34.1 Hz, CH_{Ar}), 138.6 (Cq), 137.4 (CH_{Ar}), 128.3 ($2CH_{Ar}$), 127.6 ($2CH_{Ar}$), 127.5 (CH_{Ar}), 125.4 (CH_{Ar}), 121.83 (q, $^1J_{C-F}$ = 274.1 Hz, Cq), 117.38 (q, $^3J_{C-F}$ = 2.9 Hz, CH_{Ar}), 72.7 (CH_2), 68.9 (CH_2), 45.5 (CH), 45.1 (CH), 45.0 (CH), 41.6 (CH), 39.0 (Cq), 36.5 (CH_2), 36.1 (CH_2), 33.2 (CH_2), 28.2 (CH_3), 23.1 (CH_3). ^{19}F NMR (376 MHz, $CDCl_3$): δ –68.0. $[\alpha]_D^{20}$ +15 (*c* 0.9, $CHCl_3$). FT–IR (cm^{-1}, neat): 2983, 2910, 2868, 1596, 1576, 1494, 1459, 1431, 1384, 1365, 1340, 1311, 1181, 1134, 1109, 1097, 1025, 991, 833, 805, 747, 733, 696, 674, 648, 630. $C_{24}H_{28}F_3NO$ $[M+H]^+$: 404.2196, found: 404.2188.

4-(2-(2-(Benzyloxy)ethyl)-6,6-dimethylbicyclo[3.1.1]heptan-3-yl)-2-(trifluoromethyl)pyridine (**C_4-26**, minor regioisomer, 21%): ^{1}H NMR (400 MHz, $CDCl_3$): δ 8.52 (d, J = 5.0 Hz, 1H), 7.54 (d, J = 1.7 Hz, 1H), 7.33 (dd, J = 5.1, 1.7 Hz, 1H), 7.26–7.14 (m, 3H), 7.09–7.06 (m, 2H), 4.16 (s, 2H), 3.27–3.19 (m, 2H), 3.09 (dt, J = 10.4, 7.6 Hz, 1H), 2.48–2.36 (m, 2H), 2.17 (tdd, J = 8.0, 5.9, 1.6 Hz, 1H), 2.00–1.95 (m, 2H), 1.76–1.60 (m, 3H), 1.20 (s, 3H), 1.07 (s, 3H), 1.01 (d, J = 10.0 Hz, 1H). ^{13}C NMR (101 MHz, $CDCl_3$): δ 160.7 (Cq), 150.2 (CH_{Ar}), 148.5 (q, $^2J_{C-F}$ = 34.0 Hz, CH_{Ar}), 138.3 (Cq), 128.4 ($2CH_{Ar}$), 127.6 ($2CH_{Ar}$), 126.3 (CH_{Ar}), 121.9 (q, $^1J_{C-F}$ = 274.1 Hz, Cq), 120.4 (q, $^3J_{C-F}$ = 2.8 Hz, CH_{Ar}), 72.9 (CH_2), 68.4 (CH_2), 46.1 (CH), 45.1 (CH), 43.3 (CH), 41.6 (CH), 39.0 (Cq), 37.8 (CH_2), 36.1 (CH_2), 34.5 (CH_2), 28.3 (CH_3), 23.1 (CH_3). ^{19}F NMR (376 MHz, $CDCl_3$): δ –67.8. $[\alpha]_D^{20}$ +17 (*c* 0.7, $CHCl_3$). FT–IR (cm^{-1}, neat): 2990, 2913, 2857, 1602, 1496, 1477, 1455, 1429, 1387, 1365, 1328, 1278, 1200, 1179, 1136, 1113, 1082, 1027, 999, 959, 854, 817, 733, 698, 689, 664, 627, 614. HRMS (ESI): m/z calculated for $C_{24}H_{28}F_3NO$ $[M+H]^+$: 404.2196, found: 404.2155.

2-(6,6-Dimethyl-3-(4-methylquinolin-2-yl)bicyclo[3.1.1]heptan-2-yl)ethan-1-ol (27)

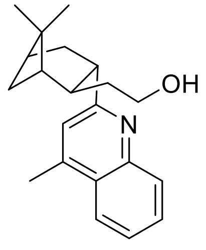

$C_{21}H_{27}NO$
MW = 309.45

Prepared according to **GP5** from 1-methoxy-4-methylquinoline tetrafluoroborate (263 mg, 1.0 mmol, 1.0 equiv) and (1*R*)-(−)-nopol (249 mg, 1.5 mmol, 1.5 equiv), but using catecholborane (0.48 mL, 4.5 mmol, 4.5 equiv). FC, 40 g silica, solid deposition, (heptanes/EtOAc 90:10 to 50:50) afforded **27** (230 mg, 74%, dr > 95:5) as a sticky oil, contaminated with traces amounts of lepidine (*ca.* 5%). ^{1}H NMR (400 MHz, $CDCl_3$): δ 8.02 (dd, J = 8.6, 1.3 Hz, 1H), 7.93 (dd, J = 8.4, 1.4 Hz, 1H), 7.66 (ddd, J = 8.4, 6.9, 1.5 Hz, 1H), 7.51 (ddd, J = 8.3, 6.9, 1.3 Hz, 1H), 7.29 (d, J = 1.1 Hz, 1H), 4.66 (bs, 1H, OH), 3.52 (dt, J = 10.9, 7.1, 7.1 Hz, 1H), 3.49–3.36 (m, 2H), 3.01 (dddd, J = 9.2, 7.5, 5.7, 2.0 Hz, 1H), 2.74–2.66 (m, 1H), 2.69 (s, 3H), 2.44 (dtd, J = 9.8, 6.2, 2.1 Hz, 1H), 2.08–1.97 (m, 3H), 1.86–1.73 (m, 2H), 1.28 (s, 3H), 1.17 (s, 3H), 1.09 (d, J = 9.8 Hz, 1H). ^{13}C NMR (101 MHz, $CDCl_3$): δ ^{13}C NMR (101 MHz, $CDCl_3$) δ 167.7 (Cq), 146.4 (Cq), 145.1 (Cq), 129.5 (CH_{Ar}), 129.1 (CH_{Ar}), 126.9 (Cq), 126.0 (CH_{Ar}), 123.6 (CH_{Ar}), 121.8 (CH_{Ar}), 60.6 (CH_2), 47.3 (CH), 42.9 (CH), 41.9 (CH), 41.6 (CH), 40.6 (CH_2), 39.1 (Cq), 36.2 (CH_2), 33.8 (CH_2), 28.1 (CH_3), 23.3 (CH_3), 19.0 (CH_3). $[\alpha]_D^{20}$ +53 (*c* 1.3, $CHCl_3$). FT–IR (cm^{-1}, neat): 3302, 2983, 2906, 2860, 1599, 1558, 1509, 1447, 1405, 1367, 1138, 1116, 1062, 1013, 757, 735. HRMS (ESI): m/z calculated for $C_{21}H_{27}NO$ $[M+H]^+$: 310.2165, found: 310.2135.

2-(((1S,2R,4R)-3,3-dimethylbicyclo[2.2.1]heptan-2-yl)methyl)-4-methylquinoline (28)

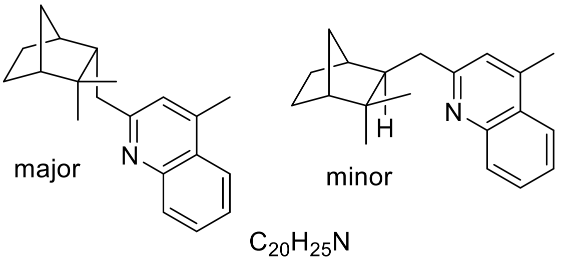

$C_{20}H_{25}N$
MW = 279.43

Prepared according to **GP5** from 1-methoxy-4-methylquinolinium tetrafluoroborate (263 mg, 1.0 mmol, 1.0 equiv) and camphen (204 mg, 1.5 mmol, 1.5 equiv). FC (heptanes/EtOAc 100:0 to 70:30) afforded **25** as an inseparable mixture of diasteoisomers (234 mg, 84%, dr = 75:25). Colorless oil. The relative configuration for both isomers was tentatively assigned on the basis of the known selectivity for the hydroboration of camphene with organoboranes (Brown, H. C.; Zweifel, G. *J. Am. Chem. Soc.* **1967**, *89*, 561).

Major isomer. ^{1}H NMR (400 MHz, $CDCl_3$): δ 8.04 (dt, J = 8.5, 0.9 Hz, 1H), 7.92 (dd, J = 8.2, 1.6 Hz, 1H), 7.65 (ddd, J = 8.4, 6.8, 1.5 Hz, 1H), 7.47 (ddd, J = 8.3, 6.9, 1.4 Hz, 1H), 7.10 (d, J = 1.1 Hz, 1H), 2.93 (dd, J = 13.7, 6.3 Hz, 1H), 2.87 (dd, J = 13.7, 10.3 Hz, 1H), 2.65 (d, J = 1.0 Hz, 3H), 2.08–2.03 (m, 1H), 1.94–1.91 (m, 1H), 1.79–1.76 (m, 1H), 1.71–1.65 (m, 2H), 1.64–1.58 (m, 1H), 1.31–1.25 (m, 2H), 1.13 (dt, J = 9.8, 1.7, 1.7 Hz, 1H), 1.03 (s, 3H), 0.93 (s, 3H). ^{13}C NMR (101 MHz, $CDCl_3$): δ 162.8 (Cq), 147.9 (Cq), 143.8 (Cq), 129.5 (CH_{Ar}), 129.0 (CH_{Ar}), 126.8 (Cq), 125.4 (CH_{Ar}), 123.7 (CH_{Ar}), 122.6 (CH_{Ar}), 50.8 (CH), 49.4 (CH), 41.3 (CH), 37.6 (Cq), 37.1 (CH_2), 36.4 (CH_2), 32.3 (CH_3), 24.9 (CH_2), 22.0 (CH_3), 20.6 (CH_2), 18.8 (CH_3).

Minor isomer (characteristic signals). ^{1}H NMR (300 MHz, $CDCl_3$): *δ* 8.01–8.04 (m, 1H), 7.94–7.92 (m, 1H), 7.67–7.63 (m, 1H), 7.50–7.46 (m, 1H), 7.12 (d, *J* = 1.1 Hz, 1H), 2.99 (dd, *J* = 13.6, 4.9 Hz, 1H), 2.69–2.61 (m, 1H), 2.65 (d, *J* = 0.9 Hz, 3H), 1.94–1.88 (m, 2H), 1.74 (dd, *J* = 4.0, 1.6 Hz, 1H), 1.72–1.63 (m, 2H), 1.42 (tdd, *J* = 11.8, 4.5, 3.3 Hz, 1H), 1.35–1.22 (m, 1H), 1.14–1.06 (m, 2H), 1.09 (s, 3H), 1.00 (s, 3H). ^{13}C NMR (101 MHz, $CDCl_3$): *δ* 163.0 (Cq), 147.8 (Cq), 143.9 (Cq), 129.5 (CH_{Ar}), 129.0 (CH_{Ar}), 126.8, 125.4, 123.7, 122.6, 54.3 (CH), 49.7 (CH), 42.2 (CH), 40.9 (Cq), 40.5 (CH_2), 35.8 (CH_2), 29.9 (CH_2), 28.0 (CH_3), 25.3 (CH_3), 24.3 (CH_2), 18.8 (CH_3).

Mixture of isomers. FT–IR (cm^{-1}, neat): 2927, 2866, 2849, 1707, 1599, 1560, 1508, 1467, 1447, 1375, 1362, 1325, 1313, 1274, 1174, 1112, 1069, 1024, 952, 760, 717. HRMS (ESI): m/z calculated for $C_{20}H_{25}N$ $[M+H]^+$: 280.2060, found: 280.2016.

(3S,5R,6S,8S,9S,10R,13R,14S,17R)-10,13-Dimethyl-17-((R)-6-methylheptan-2-yl)-6-(4-methylquinolin-2-yl)hexadecahydro-1*H*-cyclopenta[a]phenanthren-3-yl benzoate (29)

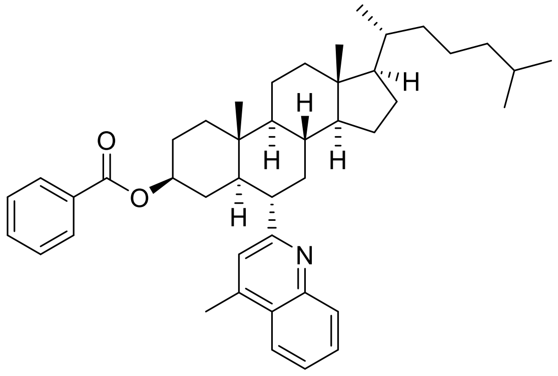

$C_{44}H_{59}NO_2$
MW = 633.96

Prepared according to **GP5** from 1-methoxy-4-methylquinolinium tetrafluoroborate (263 mg, 1.0 mmol, 1.0 equiv) and cholesteryl benzoate (736 mg, 1.5 mmol, 1.5 equiv). FC (heptanes/EtOAc 100:0 to 80:20) afforded **29** (370 mg, 58%, dr = 92:8). Colorless oil. Major isomer: ^{1}H NMR (400 MHz, $CDCl_3$): *δ* 8.05 (d, *J* = 8.4 Hz, 1H), 7.92–7.89 (m, 3H), 7.64 (ddd, *J* = 8.4, 6.8, 1.4 Hz, 1H), 7.51–7.38 (m, 2H), 7.32 (t, *J* = 7.6 Hz, 2H), 7.13 (s, 1H), 4.92 (tt, *J* = 11.0, 4.9 Hz, 1H), 2.95 (td, *J* = 12.0, 3.7 Hz, 1H), 2.68 (s, 3H), 2.06–1.98 (m, 2H), 1.94–1.85 (m, 2H), 1.83–1.73 (m, 2H), 1.71–0.88 (m, 23H), 1.08 (s, 3H), 0.94 (d, *J* = 6.5 Hz, 3H), 0.87 (d, *J* = 7.3 Hz, 3H), 0.87 (d, *J* = 6.6 Hz, 3H), 0.70 (s, 3H). ^{13}C NMR (101 MHz, $CDCl_3$): *δ* 166.0 (Cq), 165.1 (Cq), 147.8 (Cq), 144.6 (Cq), 132.6 (CH_{Ar}), 130.8 (Cq), 129.6 (CH_{Ar}), 129.5 ($2CH_{Ar}$), 129.1 (CH_{Ar}), 128.2 ($2CH_{Ar}$), 127.3 (Cq), 125.6 (CH_{Ar}), 123.7 (CH_{Ar}), 120.2 (CH_{Ar}), 74.2 (CH), 56.4 (CH), 56.3 (CH), 54.1 (CH), 48.2 (CH), 47.9 (CH), 42.7 (Cq), 40.1 (CH_2), 40.0 (CH_2), 39.6 (CH_2), 37.0 (CH_2), 36.3 (CH_2), 36.1 (Cq), 35.9 (CH), 35.3 (CH), 31.0 (CH_2), 28.4 (CH_2), 28.1 (CH), 27.6 (CH_2), 24.2 (CH_2), 24.0 (CH_2), 22.9 (CH_3), 22.7 (CH_3), 21.4 (CH_2), 19.1 (CH_3), 18.8 (CH_3), 13.4 (CH_3), 12.2 (CH_3). FT–IR (cm^{-1}, neat): 2941, 2865, 2851, 1705, 1599, 1558, 1508, 1468, 1448, 1375, 1324, 1312, 1271, 1174, 1112, 1069, 1025, 998, 949, 936, 866, 860, 759, 71. HRMS (ESI): m/z calculated for $C_{44}H_{59}NO_2$ $[M+H]^+$: 634.4619, found: 634.4588.

The relative configurations for the minor isomer could not be attributed with confidence. According to previous reports on the hydroboration of similar steroidal systems, it is very likely that the hydroboration step was not 100% stereoselective. However, other possibilities cannot be ruled out at this stage.[26]

The relative configurations for the major isomer were attributed on the basis of the known selectivity for the hydroboration of similar steroids with organoboranes,[27] together with the coupling constants for the H on the CH-4-methylquinoline, which are typical for an axial C–H having scalar ^{3}J coupling with two axial C-H:

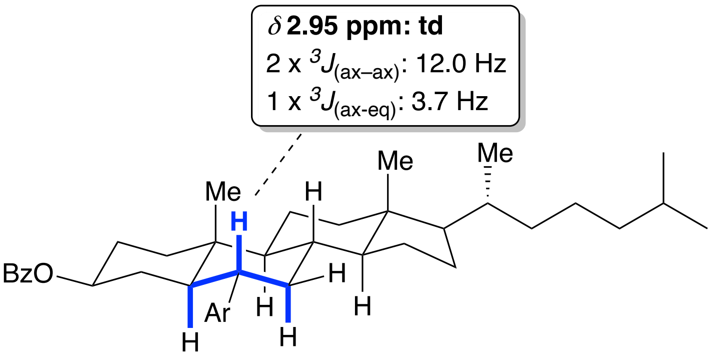

4-Methyl-2-[1-methyl-1-[(1R,4S)-4-methylcyclohex-2-en-1-yl]ethyl]quinoline (30)

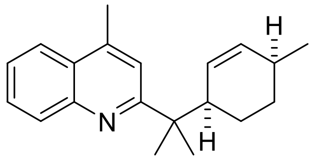

$C_{20}H_{25}N$
MW = 279.20

Prepared according to **GP5** from 1-methoxy-4-methylquinolinium tetrafluoroborate (263 mg, 1.0 mmol) and (+)-2-carene (>99%, 0.23 mL, 1.5 mmol). FC (heptanes/EtOAc 95:5) afforded **30** (214 mg, 75 %, dr 99:1). Colorless oil. ^{1}H NMR (300 MHz, $CDCl_3$): *δ* 8.07 (d, *J* = 8.3 Hz, 1H), 7.95 (dd, *J* = 8.3, 1.5 Hz, 1H), 7.66 (ddd, *J* = 8.3, 7.0, 1.5 Hz, 1H), 7.50 (ddd, *J* = 8.3, 7.0, 1.5 Hz, 1H), 7.32 (br s, 1H), 5.69 – 5.60 (m, 1H), 5.47 – 5.39 (m, 1H), 2.86 – 2.74 (m, 1H), 2.69 (d, *J* = 1.1 Hz, 3H), 2.23 – 2.08 (m, 1H), 1.78 – 1.56 (m, 2H), 1.43 (s, 3H), 1.48 – 1.33 (m, 2H), 1.39 (s, 3H), 0.96 (d, *J* = 7.0 Hz, 3H). ^{13}C NMR (75 MHz, $CDCl_3$): *δ* 168.3 (Cq), 147.4 (Cq), 143.4 (Cq), 134.4 (CH), 130.2 (CH), 128.7 (CH), 128.1 (CH), 126.6 (Cq), 125.5 (CH), 123.5 (CH), 119.8 (CH), 45.27 (CH), 43.7 (Cq), 29.4 (CH_2), 28.9 (CH), 24.5 (CH_3), 24.3 (CH_3), 21.1 (CH_3), 20.2 (CH_2), 19.1 (CH_3).

3.2 Alkylation with alkyl iodides

General procedure 6 (GP-6): alkylation of *N*-methoxypyridinium salts with alkyl iodides

To a solution of the *N*-methoxypyridinium salt (1 mmol) in DCE or DCM (20 mL) was added the alkyl iodide (6-10 mmol) under argon at rt. Et_3B (0.25 mL, 2 M in benzene, 0.5 mmol) was added while keeping the tip of the needle underneath the solvent surface, followed by DTBHN (18 mg, 0.05 mmol). In some cases, sym-collidine (0.4 mL, 3.0 mmol) was added and the mixture was stirred at 45–55 ºC. Two more portions of Et_3B (0.25 mL, 2 M in benzene, 0.5 mmol) and DTBHN (18 mg, 0.05 mmol) were added every 90 minutes. Alternatively, the Et_3B solution (1.75 mL, 2 M in benzene, 1.5 mmol) was added via syringe pump over 3 hours adding three portions of DTBHN every 90 minutes (18 mg, 0.05 mmol each). After further stirring for additional 3-12 h, the reaction mixture was cooled down to rt, filtered through a pad of neutral aluminum oxide using DCM as eluent, washed with water (2 × 20

mL) and brine. The organic layers were dried over Na_2SO_4, filtered, and concentrated under vacuum. The crude product was purified by column chromatography with heptane/EtOAc or pentane/Et_2O mixtures.

2-Cyclohexyl-4-methylquinoline (3)

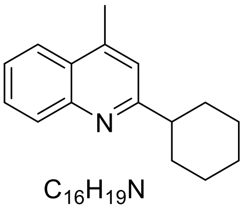

$C_{16}H_{19}N$
MW = 225.15

Prepared according to **GP6** (Slow addition of Et_3B) from 1-methoxy-4-methylquinolinium tetrafluoroborate (131 mg, 0.5 mmol) and cyclohexyl iodide (1.3 mL, 10.0 mmol) at 50 °C. Product **3** was formed in 55% yield according to GC analysis with hexadecane as internal standard. Spectral data are described in the previous section.

4-Cyclohexyl-2-methylquinoline (15)

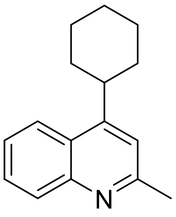

$C_{16}H_{19}N$
MW = 225.34

Prepared according to **GP6** (Slow addition of Et_3B) from 1-ethoxy-2-methyl-quinolinium tetrafluoroborate (138 mg, 0.5 mmol) and cyclohexyl iodide (1.3 mL, 10.0 mmol) with sym-collidine (0.2 mL, 1.5 mmol) at 50 °C. Product **15** was formed in 28% yield according to GC analysis with hexadecane as internal standard. Spectral data are described in the previous section.

2-Isopropyl-4-methylquinoline (31)

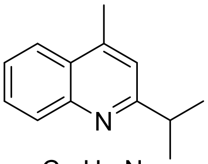

$C_{13}H_{15}N$
MW = 185.27

Prepared according to **GP6** (Slow addition of Et_3B) from 1-methoxy-4-methylquinolinium tetrafluoroborate (131 mg, 0.5 mmol, 1.0) and 2-iodopropane (0.53 mL, 5.3 mmol,) at 40 °C. FC heptanes/EtOAc 85:15) afforded **31** (51 mg, 61%). Colorless oil. ^1H NMR (300 MHz, C_6D_6): δ 8.35 (d, J = 8.4 Hz, 1H), 7.64 (dd, J = 8.3, 0.9 Hz, 1H), 7.40 (ddd, J = 8.3, 6.9, 1.4 Hz, 1H), 7.22 (ddd, J = 8.2, 6.9, 1.2 Hz, 1H), 6.83 (s, 1H), 3.16 (hept, J = 6.9 Hz, 1H), 2.21 (d, J = 0.8 Hz, 3H), 1.42 (d, J = 6.9 Hz, 6H). ^{13}C NMR (75 MHz, C_6D_6): δ 167.1 (Cq), 148.7 (Cq), 143.8 (Cq), 130.6 (CH), 129.0 (CH), 127.4 (Cq), 125.4 (CH), 123.7 (CH), 120.8 (CH), 37.3 (CH), 22.6 (2C, CH3), 18.4 (CH3). HRMS (ESI): m/z calculated for $C_{13}H_{16}N^+$ $[M+H]^+$: 186.1277, found: 186.1276. Spectral data in accordance with the literature.[17]

2-(*tert*-Butyl)-4-phenylpyridine (32)

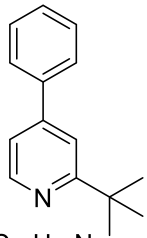

$C_{15}H_{17}N$
MW = 211.14

Prepared according to **GP6** from 1-methoxy-4-phenylpyridin-1-ium tetrafluoroborate (231 mg, 1 mmol) and 2-iodo-2-methylpropane (0.7 mL, 6 mmol) with sym-collidine (0.4 mL, 3 mmol). FC (heptanes/EtOAc 95:5) afforded **32** (80 mg, 45%). Colorless oil. ^1H NMR (300 MHz, CD_2Cl_2): δ 8.57 (dd, J = 5.1, 0.8 Hz, 1H), 7.68 – 7.63 (m, 2H), 7.56 (dd, J = 1.7, 0.8 Hz, 1H), 7.53 – 7.40 (m, 3H), 7.32 (dd, J = 5.1, 1.7 Hz, 1H), 1.40 (s, 9H). ^{13}C NMR (75 MHz, $CDCl_3$): 169.98, 149.17, 148.78, 139.27, 129.14, 128.86, 127.25, 119.02, 117.36, 37.65, 30.40. IR (cm^{-1})

2936.34, 1245.36, 1592.43, 1476.24, 719.856. HRMS: m/z calculated for $C_{16}H_{18}N^+$ $[M+H]^+$: 212.1438, found: 212.1434.

4-Cyclohexyl-2,6-dimethylpyridine (33)

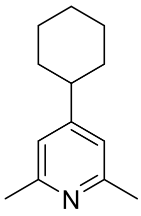

$C_{13}H_{19}N$
MW = 189.30

Prepared according to GP6 from 1-methoxy-2,6-dimethylpyridin-1-ium tetrafluoroborate (225 mg, 1 mmol) and cyclohexyl iodide (1.3 mL, 10 mmol). FC (pentane/Et_2O 10:1) afforded **33** (135.4 mg, 66%). Colorless oil. **^{1}H NMR (300 MHz, $CDCl_3$):** δ 6.97 (s, 2H), 2.74 (m, 1H), 2.61 (s, 3H), 1.90 – 1.84 (m, 4H), 1.63- 1.54 (m, 6H). **^{13}C NMR (75 MHz, $CDCl_3$):** δ 150.45, 142.43, 120.49, 45.25, 35.02, 25.93, 25.34. Spectral data in accordance with the literature.[28]

4-Isopropyl-2,6-dimethylpyridine (34)

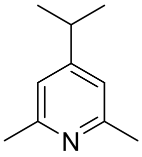

$C_{10}H_{15}N$
MW = 149.24

Prepared according to **GP6** from 1-methoxy-2,6-dimethylpyridin-1-ium tetrafluoroborate (225 mg, 1 mmol) and 2-iododpropane (0.6 mL, 6 mmol). FC (pentane/Et_2O 15:1) afforded **34** (106 mg, 71%). Yellow oil. ^{1}H NMR (300 MHz, $CDCl_3$): δ 6.88 (s, 2H), 2.82 (h, J = 6.9 Hz, 1H), 2.57 (s, 6H), 1.24 (d, J = 6.9 Hz, 6H). ^{13}C NMR (75 MHz, $CDCl_3$): δ 153.32, 143.90, 128.00, 66.95, 16.99. Spectral data in accordance with the literature.[24]

4-(*tert*-Butyl)-2,6-dimethylpyridine (35)

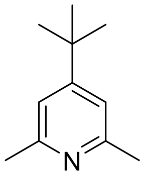

$C_{11}H_{17}N$
MW = 163.14

Prepared according to **GP6** from 1-methoxy-2,6-dimethylpyridin-1-ium tetrafluoroborate (225 mg, 1 mmol) and 2-iodo-2-methylpropane (1.2 mL, 10 mmol). FC (pentane/Et_2O 15:1) afforded **35** (103 mg, 62%). Colorless oil. ^{1}H NMR (300 MHz, $CDCl_3$): δ 6.83 (s, 2H), 2.61 (s, 6H), 1.13 (s, 9H). ^{13}C NMR (75 MHz, $CDCl_3$): δ 159.56, 156.62, 119.74, 35.05, 32.54, 25.33. Spectral data in accordance with literature.[24]

4-Adamantyl-2,6-dimethylpyridine (36)

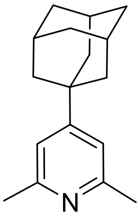

$C_{17}H_{23}N$
MW = 241.18

Prepared according to **GP6** from 1-methoxy-2,6-dimethylpyridin-1-ium tetrafluoroborate (225 mg, 1 mmol) and 1-iododadamantane (2.1 g, 8 mmol) . FC (heptanes/EtOAc 20:1) afforded **36** (164.13 mg, 66%). White solid. M.p. 95-97 ºC. ^{1}H NMR (300 MHz, $CDCl_3$): δ 6.92 (s, 2H), 2.51 (s, 6H), 2.09 (p, J = 3.1 Hz, 4H), 1.86 (d, J = 2.9 Hz, 6H), 1.79 – 1.72 (m, 6H). ^{13}C NMR (75 MHz, $CDCl_3$): 161.03, 157.30, 117.14, 42.53, 36.77, 28.80, 24.55. 52. IR (cm^{-1}) 1292.59 (m), 1245.36 (w), 1231.33 (m), 1027.15 (w), 630.002 (w). HRMS: m/z calculated for $C_{17}H_{24}N^+$ $[M+H]^+$: 242.1805, found: 242.1801.

4-((3*R*,8*S*,9*S*,10*R*,13*R*,14*S*,17*R*)-10,13-Dimethyl-17-((*R*)-6-methylheptan-2-yl)-2,3,4,7,8,9,10,11,12,13,14,15,16,17-tetradecahydro-1H-cyclopenta[a]phenanthren-3-yl)-2,6-dimethylpyridine (37)

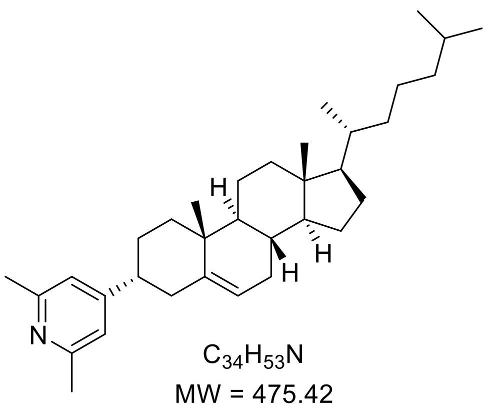

$C_{34}H_{53}N$
MW = 475.42

Prepared according to GP6 from 1-methoxy-2,6-dimethylpyridin-1-ium tetrafluoroborate (113 mg, 0.5 mmol). FC (heptanes/EtOAc 10:1) and (3*S*,8*S*,9*S*,10*R*,13*R*,14*S*,17*R*)-3-Iodo-10,13-dimethyl-17-((*R*)-6-methylheptan-2-yl)-2,3,4,7,8,9,10,11,12,13,14,15,16,17-tetradecahydro-1*H*cyclopenta[*a*]phenanthrene (0,74 mg) mg, 1.5 mmol) afforded **37** (54 mg, 23%) White solid. M.p. 131–133 ºC. ^{1}H NMR (300 MHz, $CDCl_3$): δ 6.81 (s, 2H), 5.34 (dd, J = 4.5, 2.7 Hz, 1H), 2.49 (s, 6H), 2.46 – 2.34 (m, 2H), 2.19 – 2.09 (m, 1H), 2.09 – 1.92 (m, 2H), 1.91 – 1.75 (m, 1H), 1.71 (td, J = 7.3, 5.8, 2.6 Hz, 2H), 1.63 – 1.40 (m, 6H), 1.40 – 0.95 (m, 16H), 0.96 – 0.82 (m, 12H), 0.69 (s, 4H).^{13}C NMR (75 MHz, $CDCl_3$): 157.55, 156.62, 142.28, 120.77, 119.01, 56.95, 56.32, 50.56, 45.22, 42.46, 39.95, 39.80, 39.71, 39.66, 37.04, 36.34, 35.94, 32.06, 32.01, 29.85, 29.37, 28.38, 28.15, 24.46, 24.43, 23.99, 22.96, 22.71, 21.09, 19.71, 18.88, 12.02. IR (cm^{-1}) 1292.04, 105.45, 1564.95, 1432.4, 1374.03. HRMS: m/z calculated for $C_{34}H_{54}N^{+}$ $[M+H]^{+}$: 476.4279, found: 476.4251.

4-Chloro-2-cyclohexylpyridine (38)

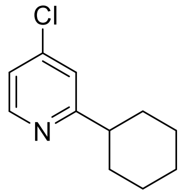

$C_{11}H_{14}ClN$
MW = 195.69

Prepared according to **GP6** from 4-chloro-1-methoxypyridin-1-ium tetrafluoroborate (200 mg, 1 mmol) and cyclohexyl iodide (1.3 mL, 10 mmol) with sym-collidine (0.4 mL, 3 mmol). FC (heptanes/EtOAc 15:1) afforded **38** (117.41 mg, 60%). Yellow oil. ^{1}H NMR (300 MHz, $CDCl_3$): δ 8.41 (dd, J = 5.5, 2.7 Hz, 1H), 7.17 (t, J = 2.5 Hz, 1H), 7.11 (td, J = 4.6, 3.9, 2.0 Hz, 1H), 2.69 (tq, J = 11.0, 3.5 Hz, 1H), 2.02 – 1.68 (m, 5H), 1.61 – 1.18 (m, 5H).^{13}C NMR (75 MHz, $CDCl_3$): 169.28, 149.90, 144.48, 121.67, 121.62, 46.46, 32.96, 26.57, 26.09. IR (cm^{-1}) 1243.04, 100.89, 1473.48, 1525.7. HRMS: m/z calculated for $C_{11}H_{15}NCl^{+}$ $[M+H]^{+}$: 196.6965, found: 196.6958.

4-Chloro-2-isopropylpyridine (39)

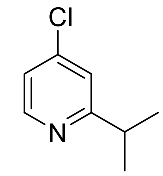

$C_8H_{10}ClN$
MW = 155.63

Prepared according to **GP6** from 4-chloro-1-methoxypyridin-1-ium tetrafluoroborate (200 mg, 1 mmol) and 2-iodo-2-methylpropane (0.6 mL, 6 mmol) with sym-collidine (0.4 mL, 3 mmol). FC (heptanes/EtOAc 15:1) afforded **39** (96.5 mg, 62%). Yellow oil. ^{1}H NMR (300 MHz, $CDCl_3$ δ 8.43 (dd, J = 5.4, 0.6 Hz, 1H), 7.18 (dt, J = 2.1, 0.5 Hz, 1H), 7.12 (dd, J = 5.3, 2.0 Hz, 1H), 3.05 (hept, J = 6.9 Hz, 1H), 1.30 (d, J = 6.9 Hz, 6H). ^{13}C NMR (75 MHz, $CDCl_3$):169.20, 150.06, 144.49, 121.70, 121.17, 36.42, 26.50. IR (cm^{-1}) 1257.04, 105.37, 156.68, 1512.5. HRMS: m/z calculated for $C_8H_{11}NCl^{+}$ $[M+H]^{+}$: 156.0567 found: 156.0561.

4-Chloro-2-isopropylpyridine (40)

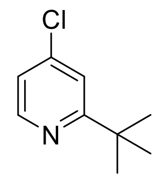

$C_9H_{12}ClN$
MW = 169.65

Prepared according to **GP6** from 4-chloro-1-methoxypyridin-1-ium tetrafluoroborate (231 mg, 1 mmol) and 2-iodo-2-methylpropane (1.2 mL, 10 mmol) with sym-collidine (0.4 mL, 3 mmol). FC (heptanes/EtOAc 15:1) afforded **40** (65 mg, 62%). Yellow oil. ^{1}H NMR (300 MHz, $CDCl_3$): δ 8.42 (d, J = 6.2 Hz, 1H), 7.33 (d, J = 2.1 Hz, 1H), 7.11 (dd, J = 6.2, 2.1 Hz, 1H), 1.36 (s, 9H). ^{13}C NMR (75 MHz, $CDCl_3$): 169.54, 149.71, 141.28, 124.23, 122.62, 35.43, 30.00. IR (cm^{-1}) 1263.79, 1227.73, 1272.60, 1000.15, 624.48. HRMS: m/z calculated for $C_9H_{13}ClN^+$ $[M+H]^+$: 170.0732, found: 170.0717.

4-Chloro-2-adamantylpyridine (41)

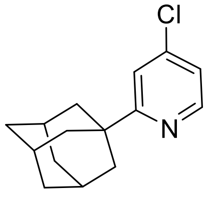

$C_{15}H_{18}ClN$
MW = 241.18

Prepared according to **GP6** from 4-chloro-1-methoxypyridin-1-ium tetrafluoroborate (231 mg, 1 mmol) and 1-iodoadamantane (2.1 g, 8 mmol) with K_2CO_3 (0.41g, 3 mmol). FC (pentane/Et_2O 9:1) afforded **41** (138.7 mg, 56%). Colorless oil. ^{1}H NMR (300 MHz, $CDCl_3$): δ 8.39 (d, J = 5.3 Hz, 1H), 7.19 (d, J = 1.6 Hz, 1H), 7.03 (dd, J = 5.2, 1.9 Hz, 1H), 2.05 (d, J = 4.8 Hz, 3H), 1.90 (d, J = 2.9 Hz, 6H), 1.71 (d, J = 3.3 Hz, 6H). ^{13}C NMR (75 MHz, $CDCl_3$): 171.09, 149.81, 144.42, 121.22, 119.79, 41.88, 39.32, 36.82, 28.82. HRMS: m/z calculated for $C_{15}H_{19}ClN^+$ $[M+H]^+$: 242.1195, found: 242.1184.

4-Bromo-2-isopropylpyridine (42)

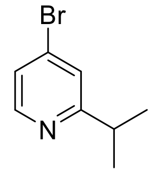

$C_8H_{10}BrN$
MW = 200.08

Prepared according to **GP6** from 4-bromo-1-methoxypyridin-1-ium tetrafluoroborate (258 mg, 1 mmol) and 2-iodo-2-methylpropane (0.6 mL, 6 mmol) with sym-collidine (0.4 mL, 3 mmol). FC (pentane/Et_2O 7:1) afforded **42** (175.5 mg, 53%). Yellow oil. ^{1}H NMR (300 MHz, $CDCl_3$): δ 7.94 (dd, J = 5.6, 0.8 Hz, 1H), 7.12 (dt, J = 2.3, 0.8 Hz, 1H), 6.95 (dd, J = 5.6, 2.0 Hz, 1H), 3.12 (hept, J = 6.7 Hz, 1H), 1.34 (d, J = 6.9 Hz, 6H). ^{13}C NMR (75 MHz, $CDCl_3$): 167.23, 151.36, 133.496, 125.94, 124.75, 121.27, 34.35, 22.43. IR (cm^{-1}) 1292.59, 1245.36, 1231.33, 1027.15, 630.002. HRMS: m/z calculated for $C_9H_{11}BrN^+$ $[M+H]^+$: 201.0833, found: 201.0837.

4-Bromo-2-adamantylpyridine (43)

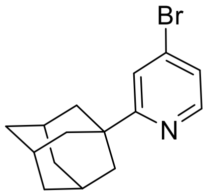

$C_{15}H_{18}BrN$
MW = 292.22

Prepared according to **GP6** from 4-bromo-1-methoxypyridin-1-ium tetrafluoroborate (258 mg, 1 mmol) and 1-iodoadamantane (2.1 g, 8 mmol) with sym-collidine (0.4 mL, 3 mmol). FC (pentane/Et_2O 5:1) afforded **43** (191 mg, 62%). Brown oil. ^{1}H NMR (300 MHz, $CDCl_3$): δ 8.40 (d, J = 5.5 Hz, 1H), 7.24 (d, J = 1.6 Hz, 1H), 7.08 (dd, J = 5.5, 1.6 Hz, 1H), 2.13 (d, J = 5.1 Hz, 3H), 1.95 (d, J = 2.9 Hz, 6H), 1.75 (d, J = 2.9 Hz, 6H). ^{13}C NMR (75 MHz, $CDCl_3$): 171.09, 149.81, 144.42, 121.22, 119.79, 41.88, 39.32, 36.82, 28.82. HRMS: m/z calculated for $C_{15}H_{19}BrN^+$ $[M+H]^+$: 293.2642, found: 293.2646.

***tert*-Butyl 6-(2,4-dichloropyridin-4-yl)piperidine-1-carboxylate (44)**

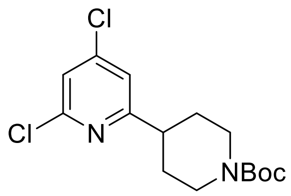

$C_{15}H_{20}Cl_2N_2O_2$
MW = 331.24

Prepared according to **GP6** from 2,4-dichloro-1-methoxypyridin-1-ium tetrafluoroborate (256 mg, 1 mmol) and *tert*-butyl 4-iodopiperidine-1-carboxylate (1,8 g, 6 mmol). FC (heptanes/EtOAc 4:1) afforded **44** (245 mg, 74%). White solid. m.p. (100–102 ºC). ^{1}H NMR (300 MHz, $CDCl_3$): δ 7.19 (d, J = 1.6 Hz, 1H), 7.07 (d, J = 1.6 Hz, 1H), 4.24 (d, J = 11.8 Hz, 2H), 2.80 (tt, J = 12.0, 3.7 Hz, 3H), 1.96–1.82 (m, 2H), 1.76–1.56 (m, 2H), 1.45 (s, 9H). ^{13}C NMR (75 MHz, $CDCl_3$): 166.64, 154.77, 151.60, 146.14, 122.10, 120.21, 44.24, 31.40, 28.58. HRMS: m/z calculated for $C_{15}H_{21}Cl_2N_2O_2^+$ [M+H]$^+$: 332.2240, found: 332.2249.

2,6-Dichloro-4-adamantylpyrindine (45)

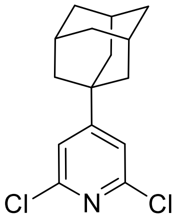

$C_{15}H_{17}Cl_2N$
MW = 282.21

Prepared according to **GP6** from 2,6-dichloro-1-methoxypyridin-1-ium tetrafluoroborate (256 mg, 1 mmol) and 1-iodoadamantane (2.1 g, 8 mmol). FC (heptane/EtOAc 20:1) afforded **45** (197 mg, 70%). Yellow oil. ^{1}H NMR (300 MHz, $CDCl_3$): δ 7.44 (s, 2H), 2.11 (d, J = 5.4 Hz, 4H), 1.98 (d, J = 2.7 Hz, 6H), 1.78 (d, J = 2.7 Hz 6H). ^{13}C NMR (75 MHz, $CDCl_3$): 168.56, 147.65, 122.45, 39.37, 34.28, 31.45, 26.29, 158.29, 118.34, 45.36, 32.45, 28.84, 25.41. 52 HRMS: m/z calculated for $C_{15}H_{18}Cl_2N^+$ [M+H]$^+$: 283.2134, found: 283.2128.

2,6-Dichloro-4-(4-phenylcyclohexyl)pyridine (46)

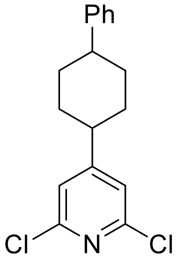

$C_{17}H_{17}Cl_2N$
MW= 306.23

Prepared according to **GP6** from 2,6-dichloro-1-methoxypyridin-1-ium tetrafluoroborate (256 mg, 1 mmol). FC (heptane/EtOAc 10:1–3:1) afforded **46** (205 mg, 67%) and (4-iodocyclohexyl)benzene (1,71 g, 6 mmol). Colorless oil. ^{1}H NMR (300 MHz, $CDCl_3$): δ 7.03-7.43 (m, 7H), 2.34 – 2.53, (m, 2H) 1.96 (ddq, J =6.7, 4.2, 2.8 4H), 1.30 – 1.68 (m, 4H). ^{13}C NMR (75 MHz, $CDCl_3$): 157.52, 147.17, 128.55, 126.93, 126.23, 119.26, 117.62, 44.03, 43.51, 34.34, 33.8169.61, 149.29, 121.34, 79.83, 46.32, 40.32, 29.36, 28.45. IR (cm^{-1}) 2847.87 (m), 1127.84, 1161.73, 1123.28. HRMS: m/z calculated for $C_{17}H_{18}Cl_2N^+$ [M+H]$^+$: 306.2156, found: 316.2150.

***tert*-Butyl 4-(2,6-dichloropyridin-4-yl)piperidine-1-carboxylate (47)**

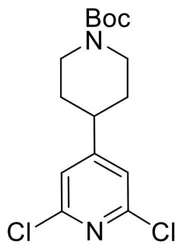

$C_{15}H_{20}Cl_2N_2O_2$
MW = 331.24

Prepared according to **GP6** from 2,6-dichloro-1-methoxypyridin-1-ium tetrafluoroborate (256 mg, 1 mmol) and tert-butyl 4-iodopiperidine-1-carboxylate (1,24 g, 4 mmol). FC (heptane/EtOAc 4:1) afforded **47** (175.5 mg, 53%). Colorless oil. ^{1}H NMR (300 MHz, $CDCl_3$):) δ 7.10 (s, 2H), 4.26 (d, J = 13.3 Hz, 2H), 2.78 (t, J = 12.8 Hz, 2H), 2.65 (tt, J = 12.2, 3.6 Hz, 1H), 1.88 – 1.76 (m, 2H), 1.66 – 1.54 (m, 2H), 1.47 (s, 9H). ^{13}C NMR (75 MHz, $CDCl_3$): δ 160.45, 154.72, 150.90, 121.59, 80.02, 41.98, 32.17, 28.57. IR (cm^{-1}) 2922.59, 1230.36, 1231.33, 1027.15, 630.002. HRMS: m/z calculated for $C_{15}H_{21}Cl_2N_2O_2^+$ [M+H]$^+$: 332.1940, found: 332.1945.

3.3 Alkylation with xanthates

General procedure 7, GP7 (alkylation of *N*-methoxypyridinium salts with xanthates)

The *N*-methoxypyridinium salt (1 mmol) and xanthate (3 mmol) were dissolved in DCE (15 mL) under argon at rt. Et_3B (0.45 mL, 1.15 M in benzene, 0.5 mmol) was added while keeping the tip of the needle underneath the solvent surface, followed by DTBHN (18 mg, 0.05 mmol). In some cases, sym-collidine (0.4 mL, 3.0 mmol) was added and the mixture was stirred at 50 ºC. Two more portions of Et_3B (0.45 mL, 1.15 M in benzene, 0.5 mmol) and DTBHN (18 mg, 0.05 mmol) were added every 60 minutes. After further stirring for additional 15 h, the reaction mixture was cooled down to rt, filtered through a pad of neutral aluminum oxide using ethyl acetate as eluent and concentrated under vacuum. The crude product was purified by column chromatography with heptane/EtOAc.

Ethyl 4-(2,6-dimethylpyridin-4-yl)undecanoate (49)

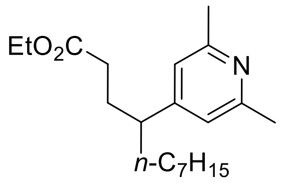

$C_{20}H_{33}NO_2$
MW = 319.49

Prepared according to **GP7** from 1-methoxy-2,6-dimethylpyridin-1-ium tetrafluoroborate (226 mg, 1 mmol). FC (pentane/TBME 10:1) afforded **49** (160 mg, 57%). Yellow oil. ^{1}H NMR (300 MHz, CDCl3) δ 6.72 (s, 2H), 4.07 (qd, *J* = 7.1, 0.9 Hz, 2H), 2.49 (s, 6H), 2.42 (tt, *J* = 9.8, 5.4 Hz, 1H), 2.10 (t, *J* = 7.8, 6.4 Hz, 2H), 2.06 – 1.89 (m, 1H), 1.86 – 1.70 (m, 1H), 1.65 – 1.50 (m, 2H), 1.33 – 1.04 (m, 15H), 0.85 (t, *J* = 6.7 Hz, 3H). ^{13}C NMR (75 MHz, $CDCl_3$) δ 173.53, 157.73, 154.96, 119.95, 60.44, 45.05, 36.18, 32.43, 31.92, 31.16, 29.65, 29.23, 27.53, 24.49, 22.74, 14.34, 14.20. IR (cm^{-1}): 2929.34, 2861.36, 1731.76, 1366.32, 1171.06, 803.689. HRMS (ESI): m/z calculated for $C_{20}H_{34}NO_2^+$ [M+H]$^+$: 320.1434 m/z found (320.1431).

Ethyl 4-(4-chloropyridin-2-yl)-5-(trimethylsilyl)pentanoate (50)

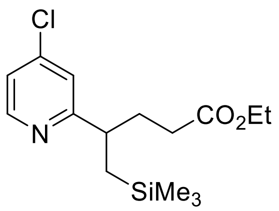

$C_{15}H_{24}ClNO_2Si$
MW = 313.90

Prepared according to **GP15** from 4-chloro-1-methoxypyridin-1-ium tetrafluoroborate (231 mg, 1 mmol). FC (pentane/TBME 15:1) afforded **50** (128 mg, 41%). Colorless oil. ^{1}H NMR (300 MHz, $CDCl_3$) δ 8.47 – 8.39 (m, 1H), 7.13 (d, *J* = 4.7 Hz, 2H), 4.08 (q, *J* = 7.1 Hz, 2H), 2.95 – 2.78 (m, 1H), 2.23 – 2.07 (m, 2H), 2.02 (ddd, *J* = 8.7, 6.6, 1.5 Hz, 2H), 1.22 (t, *J* = 7.2 Hz, 4H), 0.06 (d, *J* = 4.8 Hz, 2H), -0.16 (s, 9H).^{13}C NMR (75 MHz, $CDCl_3$) δ 173.47, 167.45, 150.38, 122.96, 121.96, 117.63, 60.44, 43.39, 34.04, 32.43, 23.82, 14.36, -1.04. IR (cm^{-1}): 2900.56, 2839.28, 1638.43, 1326.82, 707.528. HRMS (ESI): m/z calculated for $C_{15}H_{24}ClNO_2Si^+$ [M+H]$^+$: 314.1434 m/z found (314.1431).

Ethyl 4-acetoxy-4-(2,6-dimethylpyridin-4-yl)butanoate (51)

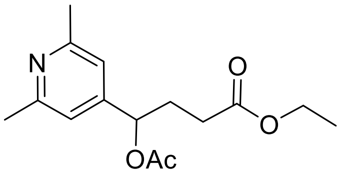

$C_{15}H_{21}NO_4$
MW = 279.15

Prepared according to **GP7** from 1-methoxy-2,6-dimethylpyridin-1-ium tetrafluoroborate (226 mg, 1 mmol). FC (heptaned/EtOAc 5:1) afforded **51** (173 mg, 62%). Yellow oil. ^{1}H NMR (300 MHz, $CDCl_3$) δ = 6.85 (s, 2H), 5.64 (t, *J* = 6.5 Hz, 1H), 4.09 (q, *J* = 7.1 Hz, 2H), 2.48 (s, 6H), 2.30 (dd, *J* = 8.9, 6.9 Hz, 2H), 2.17 – 2.02 (m, 5H), 1.22 (t, *J* = 7.1 Hz, 3H). ^{13}C NMR (75 MHz, $CDCl_3$) δ = 172.47, 170.01, 158.14, 149.33, 117.46, 73.63, 60.61, 30.99, 30.04, 21.00, 14.18. HRMS (ESI): m/z calculated for $C_{15}H_{22}NO_4^+$ $[M+H]^+$: 280.1556 m/z found (280.1550).

Ethyl 4-acetoxy-4-(4-chloropyridin-2-yl)butanoate (52)

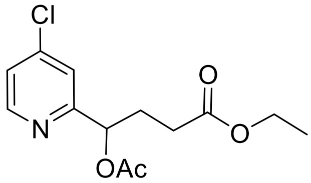

$C_{13}H_{16}ClNO_4$
MW = 282.21

Prepared according to **GP7** from 4-chloro-1-methoxypyridin-1-ium tetrafluoroborate (231 mg, 1.00 mmol). FC (heptanes/EtOAc 3:1) afforded **52** (280 mg, 99%). Colorless oil. ^{1}H NMR (300 MHz, $CDCl_3$) δ = 8.47 (dd, *J* = 5.3, 0.6 Hz, 1H), 7.31 (d, *J* = 2.0 Hz, 1H), 7.22 (dd, *J* = 5.3, 2.0 Hz, 1H), 5.80 (t, *J* = 6.2 Hz, 1H), 4.11 (q, *J* = 7.1 Hz, 2H), 2.41 – 2.32 (m, 2H), 2.32 – 2.22 (m, 2H), 2.14 (s, 3H), 1.24 (t, *J* = 7.2 Hz, 3H).^{13}C NMR (75 MHz, $CDCl_3$) δ = 172.28, 169.84, 150.06, 122.93, 121.06, 117.19, 74.69, 60.30, 29.79, 29.46, 20.74, 13.91. IR (cm^{-1}): 2961.64, 1730.8, 1225.06, 1166.72, 1022.09, 794.528, 707.747. HRMS (ESI): m/z calculated for $C_{13}H_{17}ClNO_4^+$ $[M+H]^+$: 283.0838, m/z found (286.0841).

Ethyl 4-acetoxy-4-(4-bromopyridin-2-yl)butanoate (53)

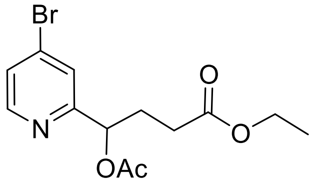

$C_{13}H_{16}BrNO_4$
MW = 329.03

Prepared according to **GP7** from 4-bromo-1-methoxypyridin-1-ium tetrafluoroborate (274.97 mg, 1 mmol), . FC (heptanes/EtOAc 5:1) afforded **54** (237 mg, 72%). Colorless oil. ^{1}H NMR (300 MHz, $CDCl_3$) δ = 8.39 (dd, *J* = 5.3, 0.6 Hz, 1H), 7.48 (d, *J* = 1.9 Hz, 1H), 7.38 (dd, *J* = 5.3, 1.9 Hz, 1H), 5.79 (t, *J* = 6.2 Hz, 1H), 4.11 (q, *J* = 7.1 Hz, 2H), 2.41 – 2.33 (m, 2H), 2.32 – 2.22 (m, 2H), 2.14 (s, 3H), 1.24 (t, *J* = 7.1 Hz, 3H). ^{13}C NMR (75 MHz, $CDCl_3$) δ = 172.56, 170.13, 160.42, 150.16, 133.58, 126.25, 124.38, 117.51, 74.88, 60.59, 30.08, 21.03, 14.20. IR (cm^{-1}): 2928.86, 1730.8, 1224.58, 1164.79, 1042.34, 690.391. HRMS (ESI): m/z calculated for $C_{13}H_{17}BrNO_4^+$ $[M+H]^+$: 330.0345, found: 330.0335.

Ethyl 4-(4-bromopyridin-2-yl)-4-butoxybutanoate (54)

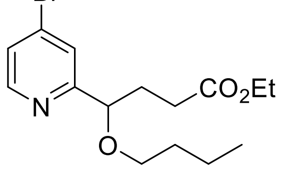

$C_{15}H_{22}BrNO_3$
MW = 344.25

Prepared according to **GP7** from 4-bromo-1-methoxypyridin-1-ium tetrafluoroborate (274.97 mg, 1 mmol). FC (heptane/EtOAc 10:1) afforded **54** (168 mg, 49%). Colorless oil. ^{1}H NMR (300 MHz, $CDCl_3$) δ = 8.43 (dd, *J* = 5.4, 0.6 Hz, 1H), 7.44 (d, *J* = 2.0 Hz, 1H), 7.20 (dd, *J* = 5.3, 2.1 Hz, 1H), 4.39 (dd, *J* = 7.8, 4.9 Hz, 1H), 4.10 (q, *J* = 7.2 Hz, 3H), 3.47 – 3.29 (m, 2H), 2.42 (t, *J* = 7.3 Hz, 2H), 2.19 – 1.94 (m, 2H), 1.65 – 1.48 (m, 4H), 1.48 – 1.31

(m, 3H), 1.24 (t, J = 7.1 Hz, 3H), 0.90 (t, J = 7.4 Hz, 6H). ^{13}C NMR (75 MHz, $CDCl_3$) δ = 173.37, 164.32, 149.92, 123.00, 121.00, 81.62, 69.80, 60.49, 32.05, 31.80, 30.51, 19.46, 14.36, 14.00. IR (cm^{-1}): 2934.61, 1683.6, 1238.39, 1141.78, 1032.76. HRMS (ESI): m/z calculated for $C_{15}H_{23}BrNO_4^+$ $[M+H]^+$: 344.0834 m/z found (344.0831).

2,6-Dimethyl-4-(undecan-2-yl)pyridine (56)

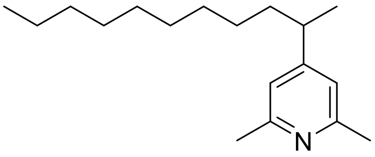

$C_{18}H_{31}N$
MW = 261.45

Prepared according to **GP7** from 1-methoxy-2,6-dimethylpyridin-1-ium tetrafluoroborate (113 mg, 0.5 mmol) and *S*-methyl *O*-(undecan-2-yl) carbonodithioate (**55a**) (393 mg, 1.5 mmol). FC (pentane/Et_2O 100:0 to 10:90) afforded **56** (70 mg, 53%). Colorless oil. ^{1}H NMR (400 MHz, $CDCl_3$): δ 6.75 (s, 2H), 2.55 (hx, J = 7.1 Hz, 1H), 2.48 (s, 6H), 1.56–1.46 (m, 2H), 1.31–1.06 (m, 14H), 1.18 (d, J = 6.9 Hz, 3H), 0.86 (t, J = 6.9 Hz, 3H). ^{13}C NMR (101 MHz, $CDCl_3$): δ 157.6 (2Cq), 157.6 (Cq), 119.2 ($2CH_{Ar}$), 39.5 (CH), 37.7 (CH_2), 32.0 (CH_2), 29.8 (CH_2), 29.7 (CH_2), 29.6 (CH_2), 29.4 (CH_2), 27.7 (CH_2), 24.6 ($2CH_3$), 22.8 (CH_2), 21.6 (CH_3), 14.2 (CH_3). FT–IR (cm^{-1}, neat): 2956, 2920, 2852, 1604, 1566, 1455, 1422, 1374, 1096, 1030, 996, 914, 858, 735. HRMS (ESI): m/z calculated for $C_{18}H_{32}N$ $[M+H]^+$: 262.2529, found: 262.2527.

***S*-Methyl *S*-((1*S*,2*S*,3*S*,5*R*)-2,6,6-trimethylbicyclo[3.1.1]heptan-3-yl) carbonodithioate (58):**

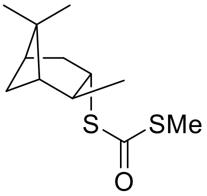

$C_{12}H_{20}OS_2$
MW = 244.41

Prepared according to **GP7** from 1-methoxy-2,6-dimethylpyridin-1-ium tetrafluoroborate (113 mg, 0.5 mmol) and *S*-methyl *O*-((1*S*,2*S*,3*S*,5*R*)-2,6,6-trimethylbicyclo[3.1.1]heptan-3-yl) carbonodithioate (**57**) (367 mg, 1.5 mmol). FC (pentane/Et_2O 100:0 to 10:90) afforded **58** (73 mg, 13%). Colorless oil. ^{1}H NMR (400 MHz, $CDCl_3$): δ 3.88 (ddd, J = 10.2, 8.0, 6.3 Hz, 1H), 2.69 (dddd, J = 13.7, 10.0, 3.3, 2.0 Hz, 1H), 2.41 (s, 3H), 2.39–2.34 (m, 1H), 2.01–1.92 (m, 3H), 1.84 (td, J = 5.8, 5.6, 2.1 Hz, 1H), 1.20 (s, 3H), 1.11 (d, J = 7.2 Hz, 3H), 1.07 (s, 3H), 0.93 (d, J = 10.0 Hz, 1H).^{13}C NMR (101 MHz, $CDCl_3$): δ ^{13}C NMR (101 MHz, $CDCl_3$) δ 190.5 (C=O), 48.2 (CH), 43.9 (CH), 42.9 (CH), 42.2 (CH), 38.7 (Cq), 38.3 (CH_2), 34.6 (CH_2), 28.0 (CH_3), 23.5 (CH_3), 20.9 (CH_3), 13.0 (S-CH_3). FT–IR (cm^{-1}, neat): 2902, 2873, 1636, 1467, 1453, 1387, 1370, 1308, 1146, 967, 862. HRMS (ESI): m/z calculated for $C_{18}H_{32}N$ $[M+H]^+$: 262.2529, found: 262.2527.

Attempted addition of cholesteryl *O*-ethyl xanthate to 1b·BF4

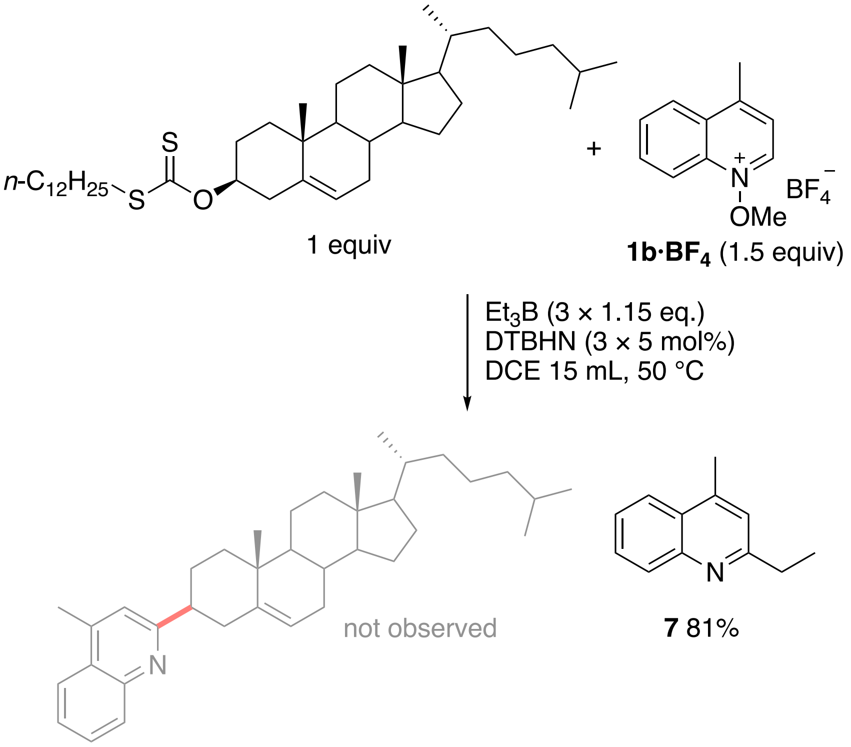

N-Methoxylepidinium tetrafluoroborate **1b·BF**$_4$ (391 mg, 1.5 mmol) and *O*-((3*S*,10*R*,13*R*)-10,13-dimethyl-17-((*R*)-6-methylheptan-2-yl)-2,3,4,7,8,9,10,11,12,13,14,15,16,17-tetradecahydro-1*H*-cyclopenta[a]phenanthren-3-yl) *S*-dodecyl carbonodithioate[29] (631 mg, 1 mmol) were dissolved in DCE (15 mL) under argon at rt. Et_3B (1.0 mL, 1.15 mmol, 1.15 M in benzene) was added to the solution, followed by DTBHN (8 mg, 0.05 mmol) and the mixture was heated to 50 ºC. Two more portions of Et_3B (1.0 mL, 1.15 mmol, 1.15 M in benzene) and DTBHN (8 mg, 0.05 mmol) were added every 60 minutes. After further stirring for additional 15 h, the reaction mixture was cooled down to rt, filtered through a pad of neutral aluminium oxide using ethyl acetate as eluent and concentrated under vacuum. Flash column chromatography over silica gel (heptanes/EtOAc 100:0 to 20:80) afforded 2-ethyl-4-methylquinoline **7** (207 mg, 81% based on **1b·BF**$_4$).

3.4 One-pot three-component alkylation of *N*-methoxypyridinium salts

General procedure 8, GP8 (three-component alkylation of *N*-methoxypyridinium salts)

The *N*-methoxypyridinium salt (1 mmol), alkene (4 mmol) and radical precursor (xanthate or iodide, 4.5 mmol) were dissolved in DCE (15-30 mL) under argon at rt. Et_3B (0.45 mL, 1.15 M in benzene, 0.5 mmol) was added while keeping the tip of the needle underneath the solvent surface, followed by DTBHN (18 mg, 0.05 mmol) and the mixture was stirred at 50 ºC. Three more portions of Et_3B (0.45 mL, 1.15 M in benzene, 0.5 mmol) and DTBHN (18 mg, 0.05 mmol) were added every 90 minutes. After further stirring for additional 15 h, the reaction mixture was cooled down to rt, filtered through a

pad of neutral aluminum oxide using ethyl acetate as eluent and concentrated under vacuum. The crude product was purified by column chromatography with heptane/EtOAc.

Ethyl 4-(4-methylquinolin-2-yl)undecanoate (59)

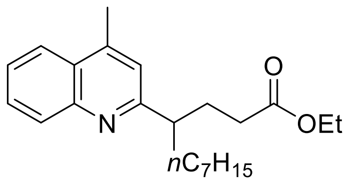

$C_{23}H_{33}NO_2$
MW = 355.25

Prepared according to **GP8** from 1-methoxy-4-methylquinolin-1-ium tetrafluoroborate (131 mg, 0.5 mmol), nonene (0.35 mL, 2 mmol) and ethyl iodoacetate (0.3 mL, 2.25 mmol). FC (heptanes/EtOAc 5:1) afforded **59** (92 mg, 52%). Yellow oil. ^{1}H NMR (300 MHz, $CDCl_3$) δ = 8.05 (dd, *J* = 8.5, 1.3 Hz, 1H), 7.95 (dd, *J* = 8.3, 1.5 Hz, 1H), 7.66 (ddd, *J* = 8.4, 6.8, 1.5 Hz, 1H), 7.50 (ddd, *J* = 8.2, 6.8, 1.3 Hz, 1H), 7.10 (d, *J* = 1.1 Hz, 1H), 4.04 (qd, *J* = 7.1, 1.6 Hz, 2H), 3.04 – 2.83 (m, 1H), 2.68 (d, *J* = 0.9 Hz, 3H), 2.31 – 2.17 (m, 1H), 2.17 – 2.04 (m, 3H), 1.76 (dddd, *J* = 23.3, 13.4, 9.0, 7.1 Hz, 2H), 1.28 – 1.13 (m, 13H), 0.88 – 0.77 (m, 3H).^{13}C NMR (75 MHz, $CDCl_3$) δ = 173.71, 164.61, 147.70, 144.29, 129.67, 128.93, 127.12, 125.60, 120.87, 117.49, 60.23, 48.13, 35.64, 32.52, 31.80, 30.46, 29.70, 29.14, 27.59, 22.62, 28.87, 14.18, 14.07. IR (cm^{-1}): 2924.52, 2853.65, 2342.12, 2360.44, 1733.21, 1179.74. HRMS (ESI): m/z calculated for $C_{22}H_{34}NO_2^+$ [M+H]$^+$: 356.2552, found: 356.2557.

Ethyl 4-(4-chloropyridin-2-yl)undecanoate (60)

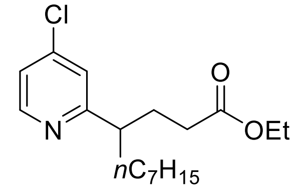

$C_{18}H_{28}ClNO_2$
MW = 325.88

Prepared according to **GP8** from 4-chloro-1-methoxypyridin-1-ium tetrafluoroborate (231 mg, 1.00 mmol), nonene (0.7 mL, 4 mmol) and ethyl iodoacetate (0.6 mL, 4.5 mmol). FC (heptanes/EtOAc 10:1) afforded **60** (91 mg, 29%). Yellow oil. ^{1}H NMR (300 MHz, $CDCl_3$) δ 8.45 (d, *J* = 5.3 Hz, 1H), 7.13 (dd, *J* = 5.3, 2.0 Hz, 1H), 7.10 (d, *J* = 1.8 Hz, 1H), 4.08 (q, *J* = 7.1 Hz, 2H), 2.71 (tt, *J* = 8.4, 6.0 Hz, 1H), 2.18 – 2.10 (m, 2H), 2.06 – 1.95 (m, 2H), 1.75 – 1.59 (m, 2H), 1.27 – 1.12 (m, 13H), 0.85 (t, *J* = 6.8 Hz, 3H). ^{13}C NMR (75 MHz, $CDCl_3$) δ 173.53, 166.35, 150.49, 144.30, 123.31, 121.88, 60.43, 47.32, 35.60, 32.42, 31.92, 30.47, 29.70, 29.26, 27.56, 22.76, 14.35, 14.2. IR (cm^{-1}): 2925.97, 2854.61, 1732.25, 1573.63, 1030.28. HRMS (ESI): m/z calculated for $C_{18}H_{29}NO_2Cl^+$ [M+H]$^+$: 325.0634, found: 326.0628

Ethyl 4-(phenanthridin-6-yl)undecanoate (61)

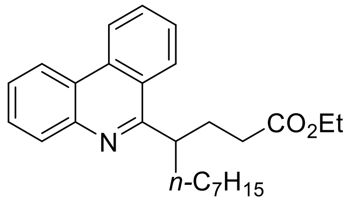

$C_{26}H_{33}NO_2$
MW = 391.56

Prepared according to **GP8** from 5-methoxyphenanthridin-5-ium tetrafluoroborate (1.00 mmol, 279 mg, 1.0 equiv), nonene (0.7 mL, 4 mmol) and ethyl iodoacetate (0.6 mL, 4.5 mmol). FC (pentane/TBME 15:1) afforded **61** (188 mg, 48%). Yellow oil^{1}H NMR (300 MHz, $CDCl_3$ δ 8.72 – 8.60 (m, 1H), 8.56 (dd, *J* = 8.1, 1.5 Hz, 1H), 8.32 (dd, *J* = 8.4, 1.3 Hz, 1H), 8.14 (dd, *J* = 8.1, 1.5 Hz, 1H), 7.83 (ddd, *J* = 8.3, 7.0, 1.3 Hz, 1H), 7.70 (dddd, *J* = 8.3, 7.3, 5.9, 1.3 Hz, 2H), 7.62 (ddd, *J* = 8.3, 7.0, 1.4 Hz, 1H), 4.04 (q, *J* = 7.2 Hz, 2H), 3.84 (p, *J* = 6.8 Hz, 1H), 2.50 (dddd, *J* = 11.6, 9.9, 8.6, 3.8 Hz, 1H), 2.39 – 1.98 (m, 4H), 1.93 – 1.56 (m, 2H), 1.46 – 1.06 (m, 13H), 0.89 – 0.79 (m, 3H). ^{13}C

NMR (75 MHz, $CDCl_3$) δ 174.04, 163.86, 143.96, 133.06, 130.14, 128.51, 127.33, 126.38, 126.07, 125.62, 123.37, 122.69, 121.95, 60.24, 35.43, 32.37, 31.93, 29.95, 29.52, 29.27, 27.80, 22.81, 22.74, 14.28, 14.19. IR (cm^{-1}): 2924.04, 2853.65, 1728.39, 1158.53, 909.272, 760.298. HRMS (ESI): m/z calculated for $C_{26}H_{34}O_2N^+$ $[M+H]^+$: 392.5680, found: 392.5684.

Ethyl 4-(4-chloropyridin-2-yl)-5-hydroxypentanoate (62)

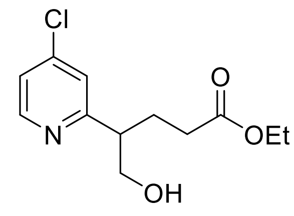

$C_{12}H_{16}ClNO_3$
MW = 257.71

Prepared according to **GP8** from 4-chloro-1-methoxypyridin-1-ium tetrafluoroborate (116 mg, 0.5 mmol), allyl alcohol (0.14 mL, 2 mmol) and ethyl iodoacetate (0.3 mL, 2.25 mmol). FC (heptanes/EtOAc 10:1) afforded **62** (67 mg, 26%). Yellow oil. ^{1}H NMR (300 MHz, $CDCl_3$) δ 8.35 (dd, *J* = 5.1, 1.0 Hz, 1H), 7.15 (s, 1H), 7.13 (d, *J* = 5.1 Hz, 1H), 4.05 (q, *J* = 6.9 Hz, 3H), 3.85 (dd, *J* = 13.8, 4.4 Hz, 2H), 2.83 (tdd, *J* = 7.5, 5.2, 3.4 Hz, 1H), 2.31 – 2.13 (m, 2H), 2.02 (dtd, *J* = 13.8, 7.0, 4.1 Hz, 2H), 1.18 (t, *J* = 6.9 Hz, 3H). ^{13}C NMR (75 MHz, $CDCl_3$) δ 173.36, 164.97, 150.08, 123.90, 122.48, 64.78, 60.61, 47.17, 32.01, 26.80, 14.34. IR (cm^{-1}): 2934.16, 2874.86, 2360.93, 1725.98, 1576.04, 1157.56, 1032.69. HRMS (ESI): m/z calculated for $C_{12}H_{17}NO_3Cl^+$ $[M+H]^+$: 258.0894, found: 258.0891.

Ethyl 3-(1-(2,6-dichloropyridin-4-yl)-4-phenylcyclohexyl) propanoate (63)

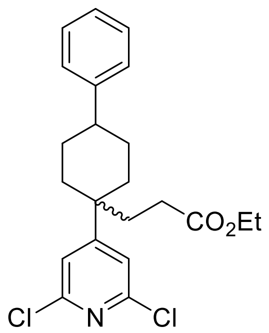

$C_{23}H_{27}Cl_2NO_2$
MW = 420.37

Prepared according to **GP8** from 2,6-dichloro-1-methoxypyridin-1-ium tetrafluoroborate (1 mmol, 285 mg,), (4-Methylenecyclohexyl)benzene (683 mg, 4 mmol) and 2-((ethoxycarbonothioyl)thio)acetate (937 mg, 4.5 mmol) FC (heptanes/EtOAc 5:1) afforded **63** (146 mg, 36%) as a 13:1 mixture of diastereomers. Yellow oil . ^{1}H NMR (300 MHz, $CDCl_3$) δ 7.31 – 7.21 (m, 2H), 7.20 – 7.09 (m, 5H), major 4.01 (q, *J* = 7.1 Hz, major, 2H), minor 4.09 (q, *J* = 7.1 Hz, minor, 0.16 H), 2.45 (tt, *J* = 10.7, 4.7 Hz, 1H), 2.17 – 2.06 (m, 2H), 2.08 – 1.96 (m, 2H), 1.93 – 1.84 (m, 2H), 1.82 – 1.73 (m, 3H), 1.61 (td, *J* = 13.6, 13.1, 5.4 Hz, 2H), 1.16 (t, *J* = 7.2 Hz, 3H). ^{13}C NMR (75 MHz, $CDCl_3$) δ 173.11, 164.48, 151.11, 146.11, 128.63, 126.90, 126.47, 120.74, 60.83, 43.56, 40.10, 35.12, 30.06, 29.15, 29.11, 14.28. IR (cm^{-1}): 2930.31, 2861.63, 2360.44, 1730.32, 1573.63, 1173.47, 700.998 HRMS (ESI): m/z calculated for $C_{23}H_{28}O_2NCl_2^+$ $[M+H]^+$: 421.1331, m/z found (421.1335).

Ethyl 4-(4-chloropyridin-2-yl)-4-(2-oxopyrrolidin-1-yl) butanoate (64)

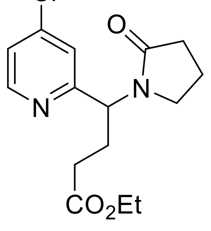

$C_{15}H_{19}ClN_2O_3$
MW = 310.78

Prepared according to **GP8** from 4-chloro-1-methoxypyridin-1-ium tetrafluoroborate (1.00 mmol, 231 mg), 1-vinylpyrrolidin-2-one (0.45 mL, 4 mmol) and 2-((ethoxycarbonothioyl)thio)acetate (937 mg, 4.5 mmol). FC (heptanes/EtOAc 5:1 to EtOAc) gave **64** (111 mg, 36%). Yellow oil. ^{1}H NMR (300 MHz, $CDCl_3$) δ 8.38 (d, *J* = 5.3 Hz, 1H), 7.25 (d, *J* = 2.0 Hz, 1H), 7.14 (dd, *J* = 5.3, 2.0 Hz, 1H), 5.21 (t, *J* = 2.4 Hz, 1H), 4.06 (q, *J* = 7.1 Hz, 2H), 3.47 – 3.10 (m, 2H), 2.53 – 2.15 (m, 6H), 1.92 (qd, *J* = 7.9, 6.0 Hz, 2H), 1.19 (t, *J* = 7.1 Hz, 3H). ^{13}C

NMR (75 MHz, $CDCl_3$) δ 175.51, 172.81, 159.87, 149.98, 145.18, 123.73, 123.32, 60.72, 54.96, 43.34, 31.28, 31.20, 25.11, 18.25, 14.30. IR (cm^{-1}): 2979.31, 2899.93, 1727.91, 1573.63, 1417.91, 1025.94. HRMS (ESI): m/z calculated for $C_{15}H_{20}ClO_2N_2$ $[M+H]^+$: 311.1149, found: 311.1157.

Ethyl 4-acetoxy-4-(4-methylquinolin-2-yl)pentanoate (65)

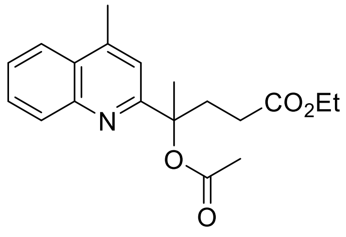

$C_{19}H_{23}NO_4$
MW = 329.40

Prepared according to **GP8** from 1-methoxy-4-methylquinolin-1-ium tetrafluoroborate (1.00 mmol, 261 mg), and 2-((ethoxycarbonothioyl)thio)acetate (937 mg, 4.5 mmol). FC (heptanes/EtOAc 5:1 to EtOAc) afforded **65** (99 mg, 30%). Yellow oil. ^{1}H NMR (300 MHz, $CDCl_3$) δ 8.04 (dt, J = 8.2, 1.0 Hz, 1H), 7.95 (dd, J = 8.4, 1.4 Hz, 1H), 7.67 (ddd, J = 8.4, 6.8, 1.5 Hz, 1H), 7.52 (ddd, J = 8.2, 6.9, 1.3 Hz, 1H), 4.05 (q, J = 7.1 Hz, 2H), 2.70 (d, J = 1.0 Hz, 3H), 2.62 – 2.33 (m, 4H), 2.14 (s, 3H), 1.96 (s, 3H), 1.20 (t, J = 7.1 Hz, 3H). ^{13}C NMR (75 MHz, $CDCl_3$) δ 173.75, 170.34, 162.52, 130.76, 129.59, 126.65, 124.07, 118.50, 60.98, 36.75, 29.78, 24.34, 22.65, 19.70, 14.72. HRMS (ESI): m/z calculated for $C_{19}H_{24}NO_4$ $[M+H]^+$: 330.1637, found 330.1632.

Ethyl 4-acetoxy-4-(4-chloropyridin-2-yl)pentanoate (66)

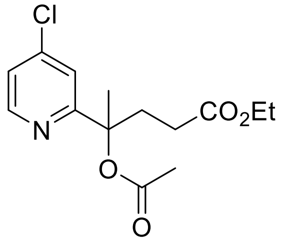

$C_{14}H_{18}ClNO_4$
MW = 299.75

Prepared according to **GP8** from 4-chloro-1-methoxypyridin-1-ium tetrafluoroborate (1 mmol, 231 mg), isopropenyl acetate (0.4 mL, 4 mmol) and 2-((ethoxycarbonothioyl)thio)acetate FC (heptanes/EtOAc 5:1 to EtOAc) afforded **66** (99 mg, 30%). Colorless oil. ^{1}H NMR (300 MHz, $CDCl_3$) δ 8.44 (dd, J = 5.3, 0.6 Hz, 1H), 7.33 (dd, J = 1.9, 0.6 Hz, 1H), 7.17 (dd, J = 5.3, 1.9 Hz, 1H), 4.07 (q, J = 7.1 Hz, 2H), 2.46 – 2.12 (m, 4H), 2.10 (s, 3H), 1.84 (s, 3H), 1.22 (t, J = 7.1 Hz, 3H). ^{13}C NMR (75 MHz, $CDCl_3$) δ 172.94, 169.64, 164.24, 149.92, 144.64, 122.55, 120.18, 83.60, 60.64, 35.91, 29.14, 24.09, 22.10, 14.30. HRMS (ESI): m/z calculated for $C_{14}H_{19}ClO_4N$ $[M+H]^+$: 300.0924, found: 300.0926.

4 Mechanistic investigations (kinetic study and competition experiments)

4.1 Kinetic study (determination of the the rate of addition k_{add} to *N*-methoxylepidinium)

General

Competition experiments were carried out from mixtures of **1b** and various amounts of cyclohexyl iodide using Et_3B under DTBHN initiation (Scheme 1, Table 1). The reaction was stopped at low conversion (<20% conversion) to ensure quasi steady state conditions.[30]

The following assumptions were made: 1) the iodine atom transfer process is irreversible, i.e. the iodine atom abstraction between a cyclohexyl radical and ethyl iodide is much slower than the reaction of the cyclohexyl radical with *N*-methoxylepidinium **1b**; 2) radical addition of ethyl and cyclohexyl radicals to **1b** are irreversible, this assumption is supported that the fact that reversibility was only observed for tertiary radicals with less reactive traps such as *N*-methoxy-2,6-dimethylpyridinium **1i**.

Experimental procedure for the kinetic experiment

To a stirred solution of 1-methoxy-4-methylquinolinium tetrafluoroborate (131 mg, 0.50 mmol, 1.00 equiv.) in 1,2-dichloroethane (dist. from CaH_2, 5 mL) was added cyclohexyl iodide (various amounts, see reagent table), a 2 M solution of triethylborane in benzene (0.25 mL, 0.50 mmol, 1.00 equiv.) *via* syringe pump (2 h) and a small crystal of DTBHN (3-5 mg). The reaction was carried out at 50 °C during 2.5 h and another small crystal of DTBHN was added once every hour (2x). After complete addition of the triethylborane (2 h), heating was removed and hexadecane (in DCE, 22.6 mg, 0.10 mmol, 0.20 equiv.) was added. After filtration of an aliquot over a pad of basic aluminum oxide the mixture was analyzed by GC.

Table for rate constant determination

CyI [mmol][1]	[**1b**] [mmol][1]	CyI/**1b**	**3**/**7**[2]
0.54	0.45	1.18	0.06
1.01	0.46	2.19	0.10
1.46	0.45	3.25	0.16
2.46	0.45	5.45	0.25
5.02	0.46	10.86	0.44
10.08	0.47	21.69	0.87

[1]) average value between the beginning and the end of the reaction (conversion ≤20%). 2) Determined by GC analysis using hexadecane as a standard.

Graphical representation

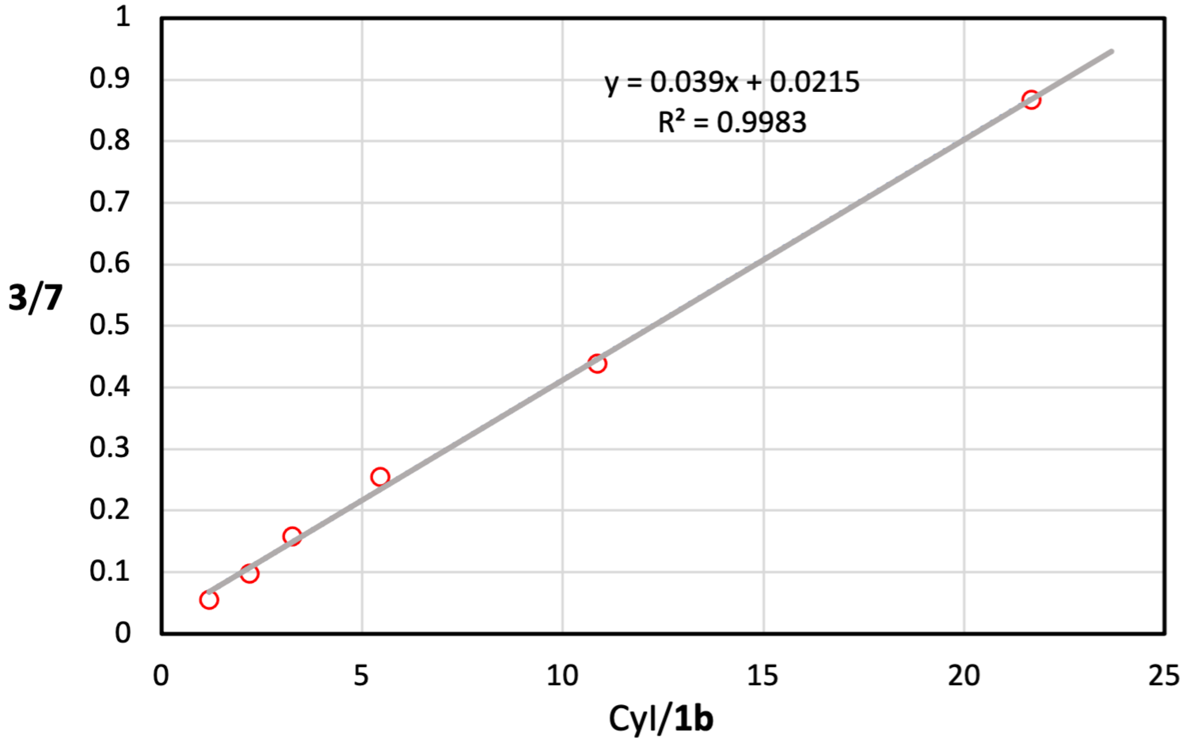

Rate constant calculation

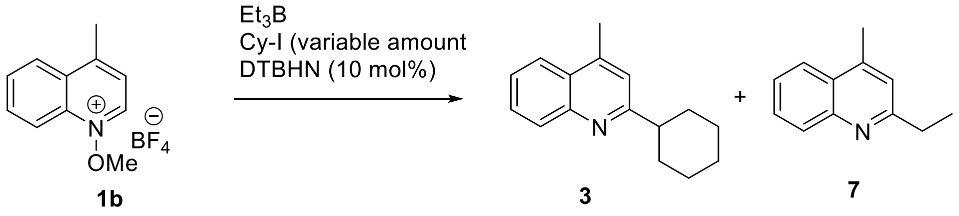

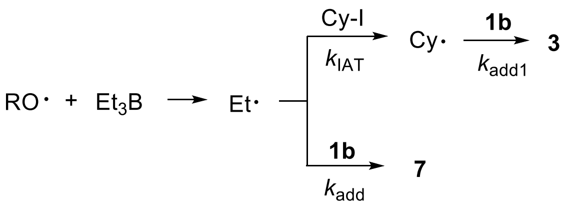

The rate constant for the iodine atom transfer to the *n*-octyl radical has been measured ($k_{\mathrm{IAT}} = 5.4 \pm 0.9 \times 10^5\ \mathrm{M^{-1}\ s^{-1}}$).[31]

$$\frac{\mathbf{3}}{\mathbf{7}} = \frac{k_{\mathrm{IAT}}}{k_{\mathrm{add}}}\frac{\mathrm{CyI}}{\mathbf{1b}}$$

From the slope of the graphic, one can deduced that $k_{\mathrm{add}} = k_{\mathrm{IAT}}/\mathrm{slope}$. Using the experimental slope of 0.039, $\mathrm{k_{add}}$ (50 °C) = $1.4 \pm 1 \times 10^7\ \mathrm{M^{-1}\ s^{-1}}$.

4.2 Verification of the validity of the reported rate constant for the addition of primary alkyl radicals to protonated lepidine

Experimental procedure

To a stirred solution of lepidine (72 mg, 0.50 mmol, 1.00 equiv.) in 1,2-dichloroethane (5 mL) were successively added trifluoroacetic acid (63 mg, 0.55 mmol, 1.1 equiv.), cyclohexyl iodide (various amounts, see reagent table), di-*tert*-butylperoxide (80 mg, 0.55 mmol, 1.1 equiv.), Et_3B (0.45 mL, 0.50 mmol, 1.00 equiv., 1.15M in benzene), a small crystal of DTBHN (5–7 mg) and *n*-hexadecane (30 µL, 22.6 mg, 0.10 mmol, 0.20 equiv.) as an internal standard. The reaction was then heated at 50 °C and stirred for 1h and another portion of DTBHN (5–7 mg) was added. The reaction mixture was stirred for an additional 1 h at 50 °C. An aliquot was collected (0.1–0.2 mL) and filtered over a pad of basic aluminum oxide (elution with EtOAc) and the mixture was analyzed by GC (10–15% conversion when the reaction was stopped after 2 h).

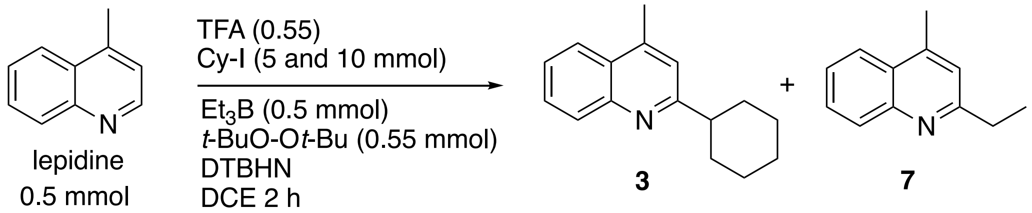

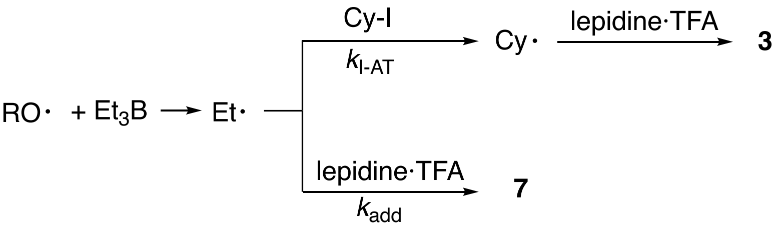

Expected ratio using reported absolute rate constants for the addition of *n*-octyl radical to protonated lepidine ($k_{Add} = 4.8 \times 10^5\ M^{-1}\ s^{-1}$ at 50 °C) and iodine atom transfer from secondary alkyl iodide to the ethyl radical ($k_{IAT} = 5.4 \times 10^5\ M^{-1}\ s^{-1}$ at 50 °C):

$$\frac{n(\mathbf{3})}{n(\mathbf{7})} = \frac{k_{IAT}}{k_{add}} \frac{n(CyI)}{n(lepidine \cdot TFA)}$$

For n(CyI)/n(lepidine·TFA) = 10:

Expected ratio: $\frac{n(3)}{n(7)} = 11$; found experimentally: $\frac{n(3)}{n(7)} = 25$

For CyI/**lepidine** = 20:

Expected ratio: $\frac{n(3)}{n(7)} = 23$; found experimentally: $\frac{n(3)}{n(7)} = 39$

Conclusion: The experimental ratio of cyclohexylated and ethylated lepidine (**3**/**7**) measured for the protonated lepidine at 50 °C in DCE are in surprisingly good agreement with the ratio expected based on the rate constants reported by Citterio, Minsici and co-workers[32] in under very different solvent and reaction conditions (*n*-butyl radical generated from valeroyl peroxide by treatment with Cu(OAc) in a mixture of water/acetic acid)

4.3 Influence of the base on the regioselectivity

2-Chloro-4- and 6--isopropylpyridine (67)

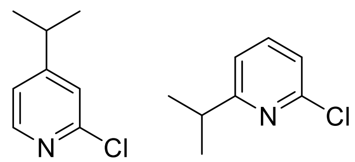

$C_8H_{10}ClN$
MW = 155.63

Prepared according to **GP6** from 2-chloro-1-methoxypyridin-1-ium tetrafluoroborate (231 mg, 1 mmol) and 2-iodo-2-methylpropane (0.6 mL, 6 mmol) with K_2CO_3 (0.41g, 3 mmol). FC (heptane/EtOAc 10:1–3:1) afforded **67** (4-substituted: 18 mg, 12% and 6-substituted: 37 mg, 24%).

The reaction in the absence of base afforded a 1:1 mixture of 4-substituted (28 mg, 18%) and 6-substituted **67** (28 mg, 18%). When 2,4,6-collidine (3 equiv) was used, the reaction was dirty and low yielding (yield ≤30%). Analysis of the crude product showed that the 4- and 6-substituted regioisomers were present at as a 1:1.5 mixture.

2-Chloro-4-isopropylpyridine. Colorless oil. ^{1}H NMR (300 MHz, $CDCl_3$): δ 8.27 (d, *J* = 5.2 Hz, 1H), 7.21 – 7.15 (m, 1H), 7.07 (dd, *J* = 5.1, 1.5 Hz, 1H), 2.88 (hept, *J* = 6.9 Hz, 1H), 1.25 (d, *J* = 6.9 Hz, 6H). ^{13}C NMR (75 MHz, $CDCl_3$): δ 161.00, 151.61, 149.52, 120.92, 33.54, 22.93. Spectral data in accordance with literature.[33]

2-Chloro-6-isopropylpyridine. Colorless oil. ^{1}H NMR (300 MHz, $CDCl_3$): δ 7.53 (t, *J* = 7.7 Hz, 1 H), 7.13 (d, *J* = 7.9 Hz, 1 H), 7.09 (d, *J* = 7.7 Hz, 1 H), 2.94 (sept, *J* = 6.9 Hz, 1 H), 1.27 (d, *J* = 6.9 Hz, 6 H), ppm; ^{13}C NMR (75 MHz, CDCl3): δ 22.5, 36.2, 118.8, 121.4, 138.9, 150.5, 168.5. Spectral data in accordance with literature.[34]

4.4 Regioselective addition of *tert*-alkyl radicals

4-(1-Adamantyl)-2-chloro-pyridine (68)

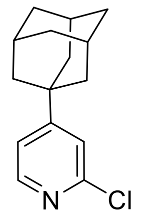

$C_{15}H_{18}ClN$
MW = 247.77

Prepared according to **GP6** from 2-chloro-1-methoxypyridin-1-ium tetrafluoroborate (231 mg, 1 mmol) and 1-iodoadamantane (2.1 g, 8 mmol). FC (heptanes/ EtOAc 10:1) afforded **39** (144 mg, 58%, regioselectivity = 96:4). Colorless oil. ^{1}H NMR (300 MHz, $CDCl_3$): 8.22 (d, *J* = 5.3 Hz, 1H), 7.20 (d, *J* = 1.7 Hz, 1H), 7.11 (dd, *J* = 5.3, 1.7 Hz, 1H), 2.05 (p, *J* = 3.1 Hz, 3H), 1.80 (d, *J* = 2.9 Hz, 6H), 1.76–1.62 (m, 6H). ^{13}C NMR (75 MHz, $CDCl_3$): δ 163.61, 151.98, 149.60, 121.20, 119.44, 42.40, 36.79, 36.56,

28.65. IR (cm^{-1}) 2900.41, 2357.55, 1585.68, 1376.44, 746.799. HRMS: m/z calculated for $C_{15}H_{19}ClN^+$ $[M+H]^+$: 248.1123, found: 248.1118.

4.5 Competitive addition of *sec*- and *tert*-alkyl radicals

General

Competition experiments for the alkylation of *N*-methoxy-2,5-dimethylpyridinium involving a secondary alkyl iodide (isopropyl iodide) and a tertiary alkyl iodide (*tert*-butyl iodide and 1-adamantyl iodide) were examined. Since with *N*-methoxy-2,5-dimethylpyridinium steric effects for the addition at position 4 is not expected to be strongly influenced by steric effects, product distribution was expected to be controlled by the rate of formation of the radicals. Based on the reported rate constants of iodine atom transfer from these iodides to the 1-octyl radical,[35] it was expected that the introduction of the tertiary alkyl group would predominate by a factor 5 for the *tert*-butyl radical if the radical addition to the pyridinium salt is irreversible.

Experimental procedure

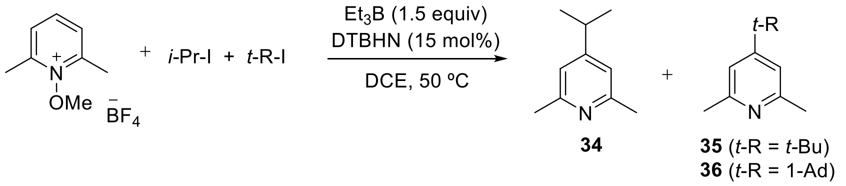

To a stirred solution of 1-methoxy-2,6-dimethylpyridinium tetrafluoroborate (112 mg, 0.50 mmol) in 1,2-dichloroethane (dist. from CaH_2, 8mL) were added isopropyl iodide (510 mg, 3.00 mmol) and *tert*-butyl iodide (552 mg, 3.00 mmol)), a 2 M solution of triethylborane in benzene (0.13 mL, 0.25 mmol, 0.5 equiv.) and DTBHN (7 mg). The reaction was heated at 50 °C during 90 min and two additional portions of triethylborane (0.13 mL, 2 M solution in benzene, 0.25 mmol) and DTBHN (7 mg) were added at 90 min intervals. The reaction mixture was then stirred for 12 h and dodecane (12.7 mg, 0.075 mmol in DCE) was added as an internal standard. The mixture sonicated for 5 min. After filtration of an aliquot over a pad of basic aluminum oxide, the filtrate was diluted with EtOAc and a 1.7:1 mixture of **34**/**35** was obtained (ratio determined by GC analysis).

The same procedure was repeated using 1-iodoadamantane (786 mg, 3.00 mmol) instead of *tert*-butyl iodide affording a 2.1:1 mixture of **34**/**36**.

Conclusion

The isopropyl substituted product was found to be the major addition product in both experiments. Since the tertiary alkyl radicals are expected to be formed preferentially, this could indicate that the addition of the radical to *N*-methoxypyridinium salt is a reversible process and the **34**/**35** and **34**/**6** product ratio is influenced by the higher reversibility of the addition of the more stable tertiary over secondary radicals.

5 References

1 A. Kaga, X. Wu, J. Y. J. Lim, H. Hayashi, Y. Lu, E. K. L. Yeow and S. Chiba, *Beilstein J. Org. Chem.*, 2018, **14**, 3047–3058.

2 A. Kapat, E. Nyfeler, G. T. Giuffredi and P. Renaud, *J. Am. Chem. Soc.*, 2009, **131**, 17746–17747.

3 N. D. C. Tappin, M. Gnägi-Lux and P. Renaud, *Chem. – Eur. J.*, 2018, **24**, 11498–11502.

4 V. Soulard, G. Villa, D. P. Vollmar and P. Renaud, *J. Am. Chem. Soc.*, 2018, **140**, 155–158.

5 L. Thomas, F. H. Lutter, M. S. Hofmayer, K. Karaghiosoff and P. Knochel, *Org. Lett.*, 2018, **20**, 2441–2444.

6 P. López-Mendoza, J. E. Díaz, A. E. Loaiza and L. D. Miranda, *Tetrahedron*, 2018, **74**, 5494–5502.

7 D. Meyer, H. Jangra, F. Walther, H. Zipse and P. Renaud, *Nat. Commun.*, 2018, **9**, 4888.

8 S. Duric and C. C. Tzschucke, *Org. Lett.*, 2011, **13**, 2310–2313.

9 L. Bering and A. P. Antonchick, *Org. Lett.*, 2015, **17**, 3134–3137.

10 R. Rubio-Presa, M. A. Fernández-Rodríguez, M. R. Pedrosa, F. J. Arnáiz and R. Sanz, *Adv. Synth. Catal.*, 2017, **359**, 1752–1757.

11 A.-L. Barthelemy, B. Tuccio, E. Magnier and G. Dagousset, *Angew. Chem. Int. Ed.*, 2018, **57**, 13790–13794.

12 D. Shukla, S. P. Adiga, W. G. Ahearn, J. P. Dinnocenzo and S. Farid, *J. Org. Chem.*, 2013, **78**, 1955–1964.

13 C. Bünz, D. Heber and U. Ravens, *Arch. Pharm. (Weinheim)*, 1993, **326**, 229–236.

14 E. D. Lorance, W. H. Kramer and I. R. Gould, *J. Am. Chem. Soc.*, 2002, **124**, 15225–15238.

15 M. A. AmrollahiBiyouki, R. A. J.Smith, J. J. Bedford and J. P. Leader, *Synth. Commun.*, 1998, **28**, 3817–3825.

16 L. Fang, L. Chen, J. Yu and L. Wang, *Eur. J. Org. Chem.*, 2015, **2015**, 1910–1914.

17 G. A. Molander, V. Colombel and V. A. Braz, *Org. Lett.*, 2011, **13**, 1852–1855.

18 L. Zhang and Z.-Q. Liu, *Org. Lett.*, 2017, **19**, 6594–6597.

19 E. Sato, Y. Ikeda and Y. Kanaoka, *Chem. Pharm. Bull. (Tokyo)*, 1990, **38**, 1205–1210.

20 G.-X. Li, C. A. Morales-Rivera, Y. Wang, F. Gao, G. He, P. Liu and G. Chen, *Chem. Sci.*, 2016, **7**, 6407–6412.

21 C. Tang, Y. Yuan and N. Jiao, *Org. Lett.*, 2015, **17**, 2206–2209.

22 A. P. Antonchick and L. Burgmann, *Angew. Chem.*, 2013, **125**, 3349–3353.

23 M. C. Quattrini, S. Fujii, K. Yamada, T. Fukuyama, D. Ravelli, M. Fagnoni and I. Ryu, *Chem. Commun.*, 2017, **53**, 2335–2338.

24 S. Paul and J. Guin, *Chem. – Eur. J.*, 2015, **21**, 17618–17622.

25 D. Chianelli, L. Testaferri, M. Tiecco and M. Tingoli, *Tetrahedron*, 1982, **38**, 657–663.

26 J. Ciciolil Hilario-Martínez, F. Murillo, J. García-Méndez, E. Dzib, J. Sandoval-Ramírez, M. Ángel Muñoz-Hernández, S. Bernès, L. Kürti, F. Duarte, G. Merino and M. A. Fernández-Herrera, *Chem. Sci.*, 2020, **11**, 12764–12768.

27 M. Nussim, Y. Mazur and F. Sondheimer, *J. Org. Chem.*, 1964, **29**, 1120–1131.

28 J. Dong, X. Lyu, Z. Wang, X. Wang, H. Song, Y. Liu and Q. Wang, *Chem. Sci.*, 2019, **10**, 976–982.

29 V. Soulard, G. Villa, D. P. Vollmar and P. Renaud, *J. Am. Chem. Soc.*, 2018, **140**, 155–158.

30 M. Newcomb, in *Encyclopedia of Radicals in Chemistry, Biology and Materials*, Wiley, 2012.

31 M. Newcomb, R. M. Sanchez and J. Kaplan, *J. Am. Chem. Soc.*, 1987, **109**, 1195–1199.

32 A. Citterio, F. Minisci, O. Porta and G. Sesana, *J. Am. Chem. Soc.*, 1977, **99**, 7960–7968.

33 Q. Chen, T. León and P. Knochel, *Angew. Chem. Int. Ed.*, 2014, **53**, 8746–8750.

34 L. Hintermann, T. T. Dang, A. Labonne, T. Kribber, L. Xiao and P. Naumov, *Chem. – Eur. J.*, 2009, **15**, 7167–7179.

35 M. Newcomb, R. M. Sanchez and J. Kaplan, *J. Am. Chem. Soc.*, 1987, **109**, 1195–1199.

6 ^{1}H and ^{13}C NMR spectra

2-(2-(Benzyloxy)ethyl)-6,6-dimethylbicyclo[3.1.1]hept-2-ene

^{1}H-NMR (300 MHz, $CDCl_3$)

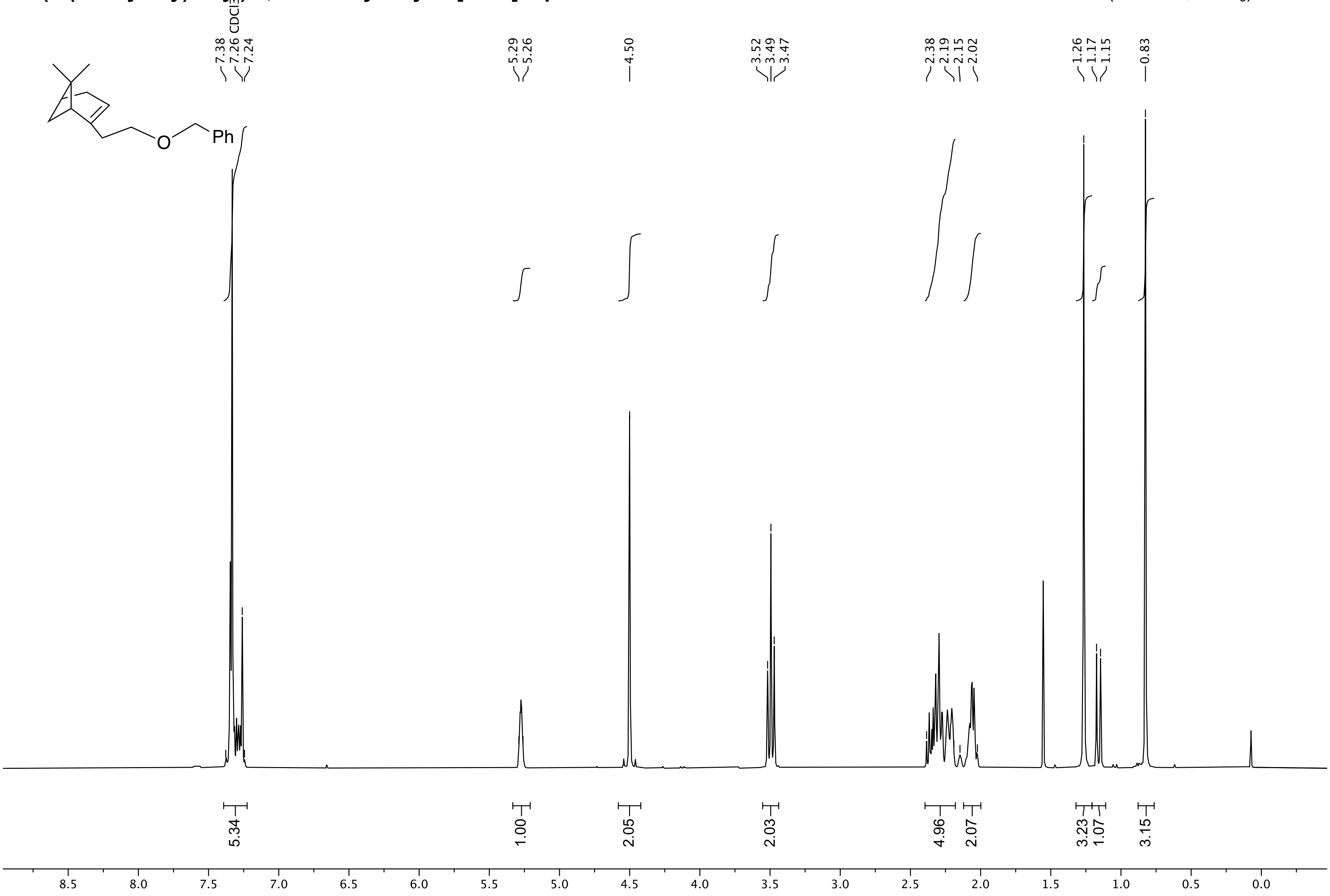

2-(2-(Benzyloxy)ethyl)-6,6-dimethylbicyclo[3.1.1]hept-2-ene

^{13}C-NMR (75 MHz, $CDCl_3$)

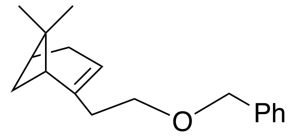

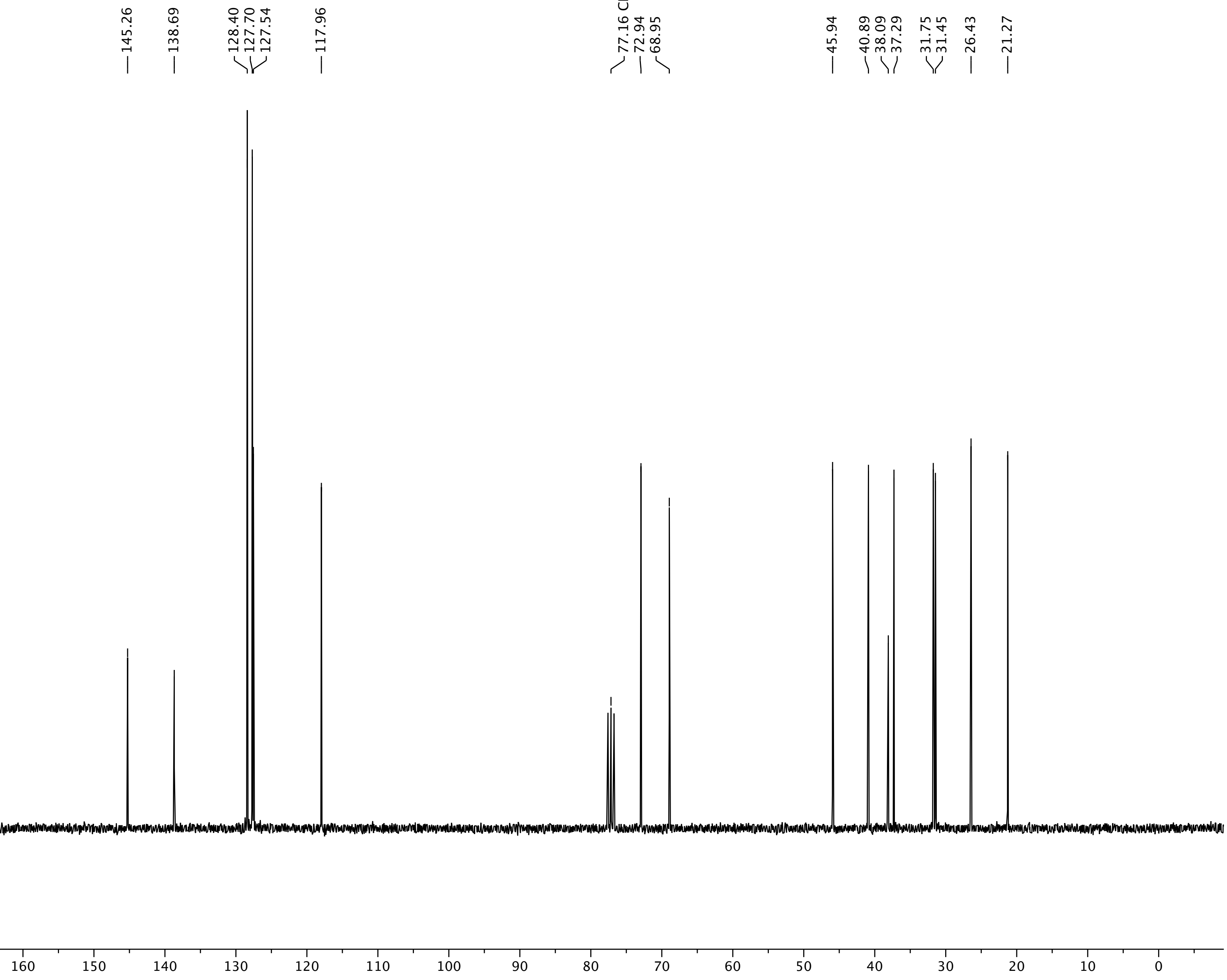

2-(2-(Benzyloxy)ethyl)-6,6-dimethylbicyclo[3.1.1]hept-2-ene

DEPT135 (75 MHz, $CDCl_3$)

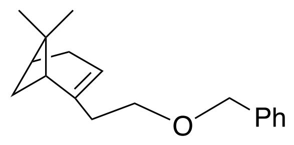

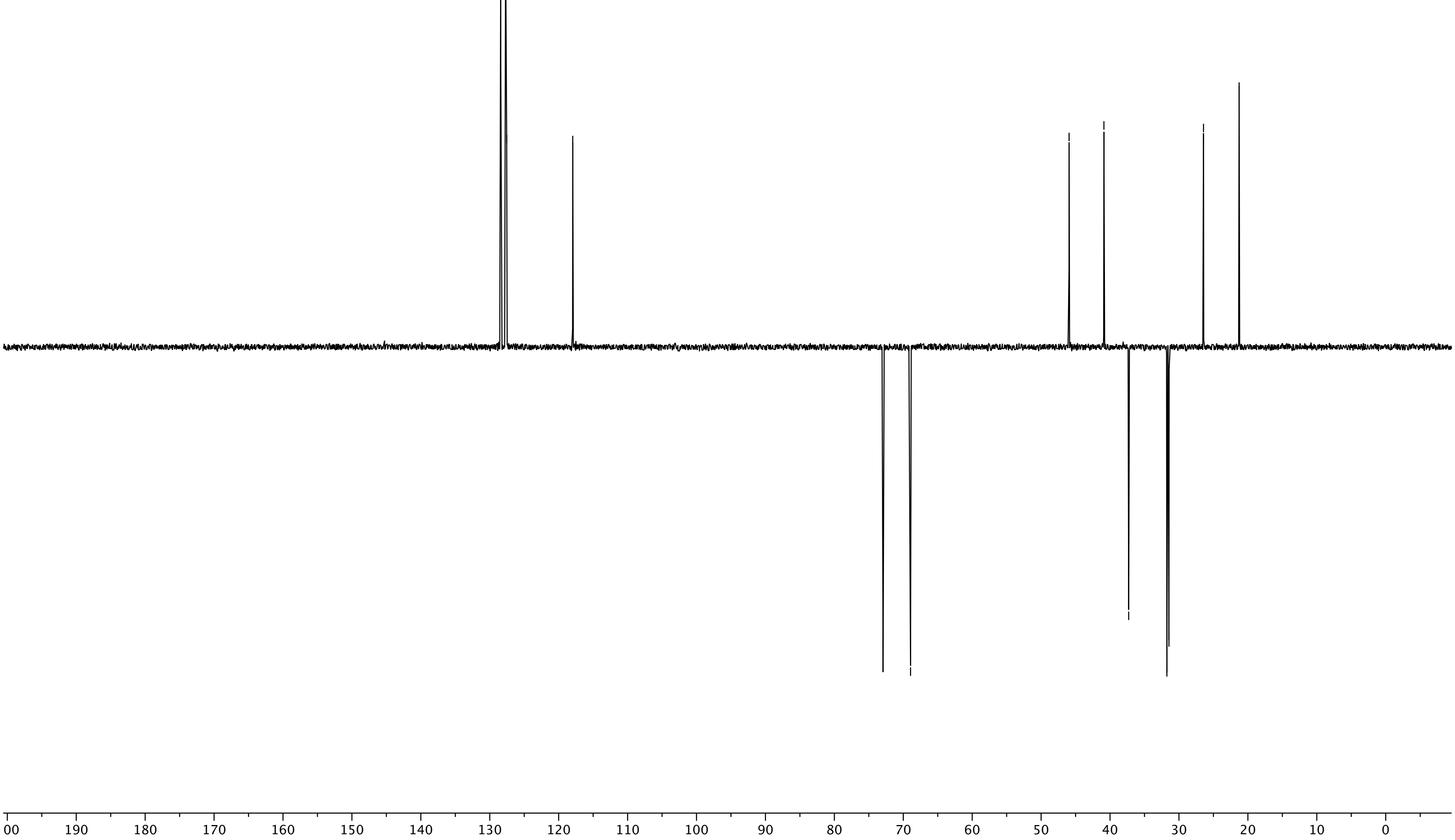

4-Methylquinoline 1-oxide

^1H-NMR, 300 MHz, CD_3CN

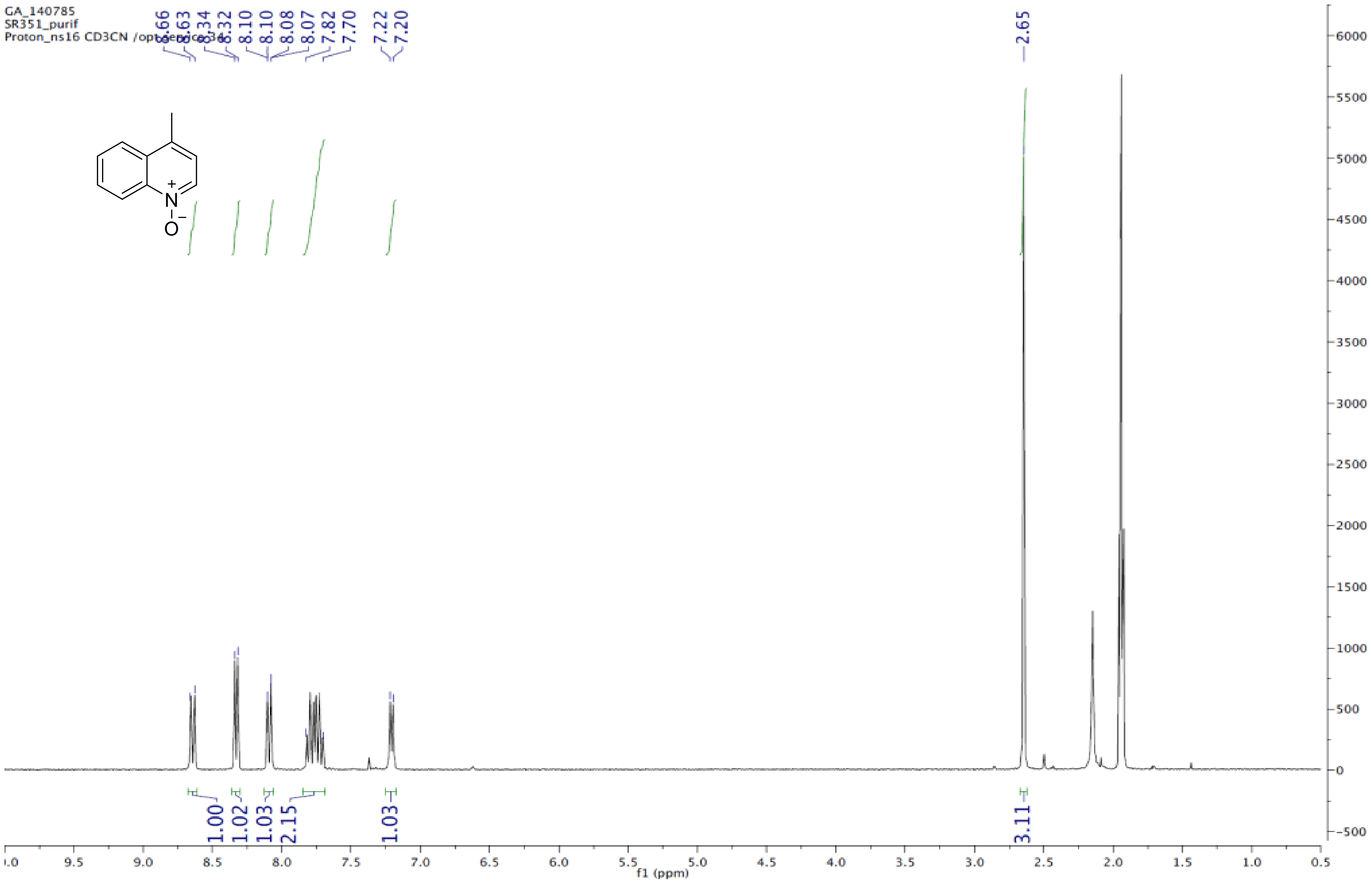

4-Methylquinoline 1-oxide

^{13}C-NMR, 300 MHz, CD_3CN

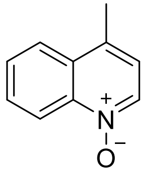

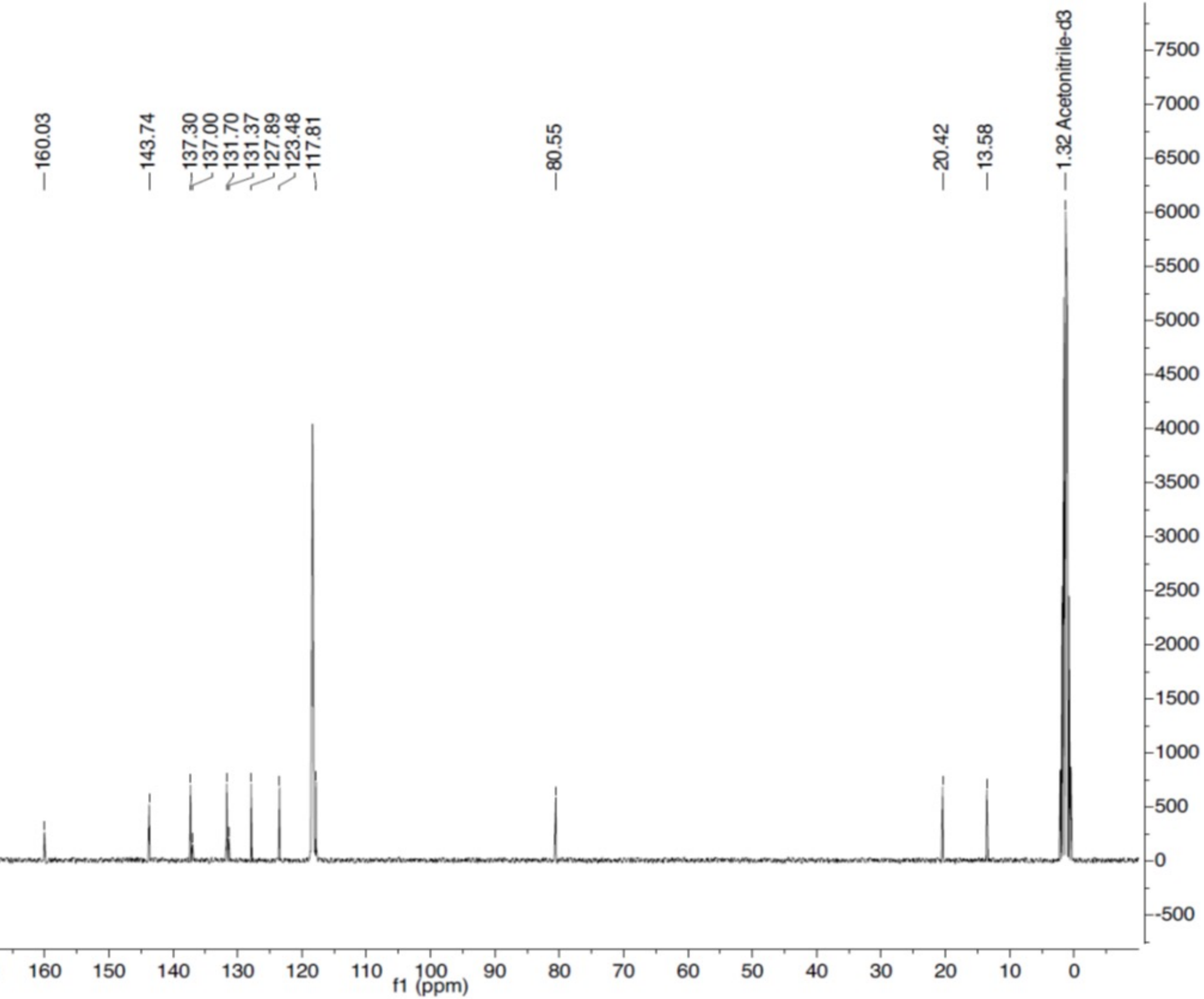

4-(*tert*-Butyl)pyridine 1-oxide

^{1}H-NMR, 75 MHz, CD_3CN

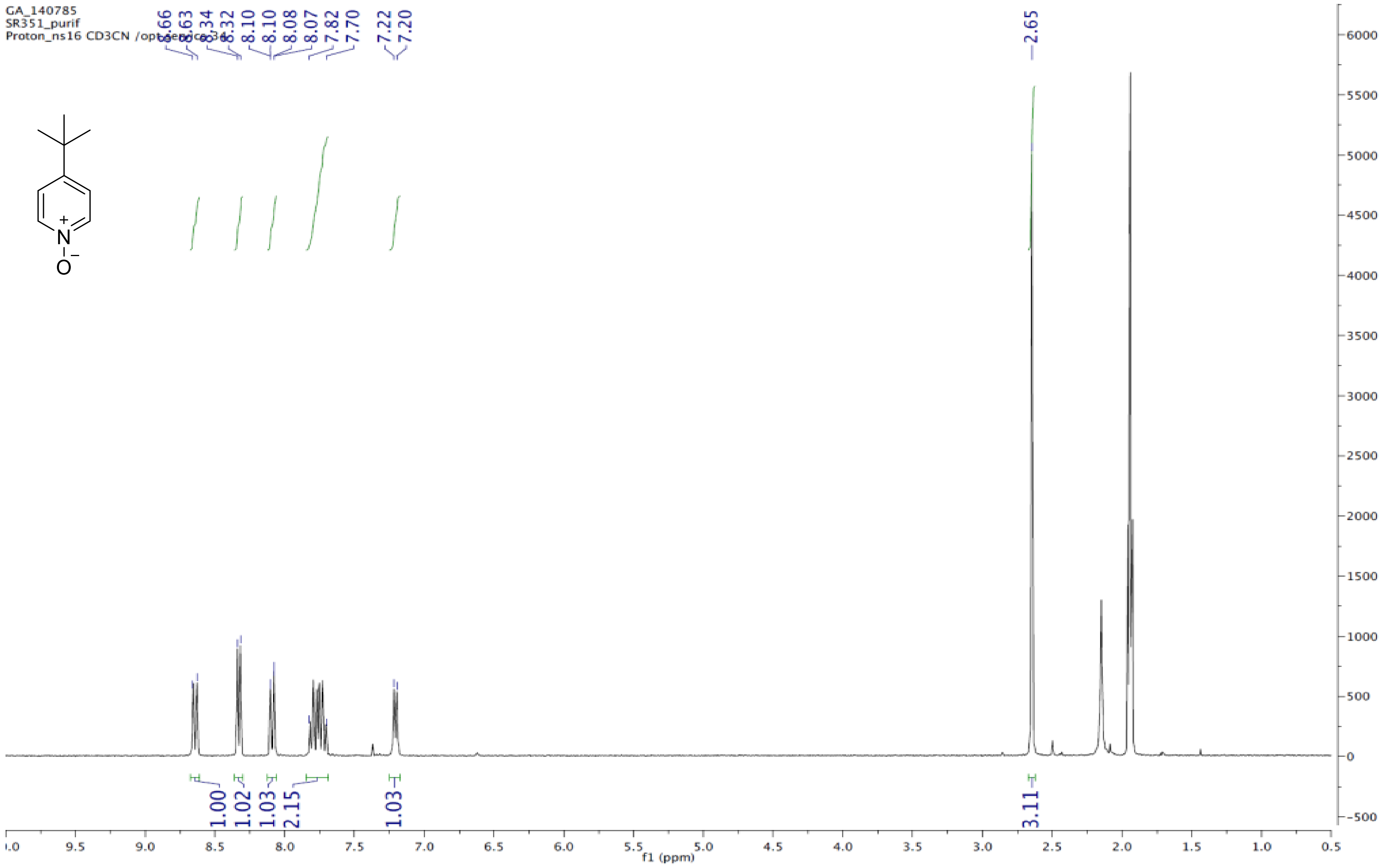

4-(*tert*-Butyl)pyridine 1-oxide

^{13}C-NMR, 75 MHz, $CDCl_3$

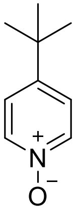

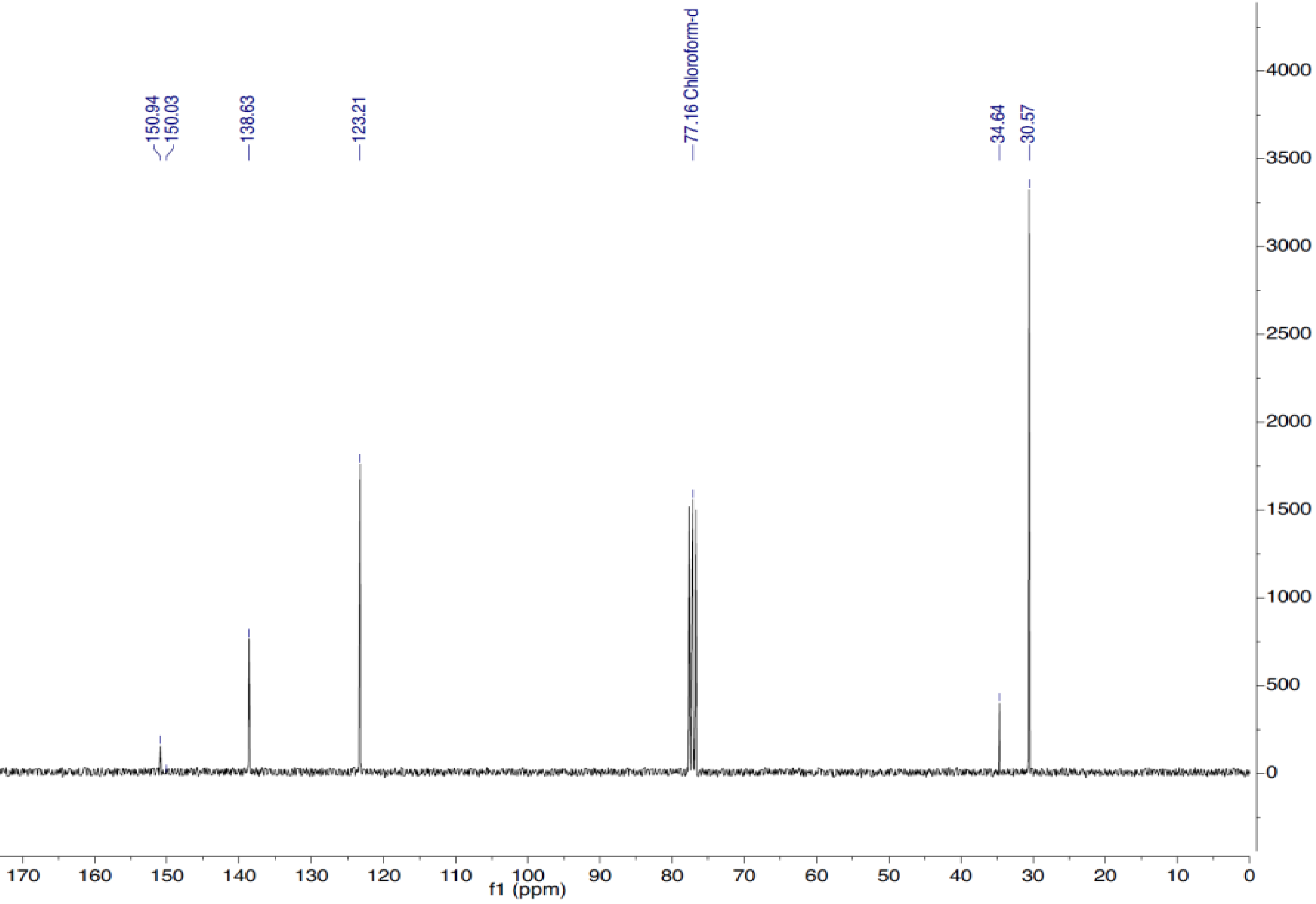

4-(Ethoxycarbonyl)pyridine 1-oxide

^{13}C-NMR, 75 MHz, $CDCl_3$

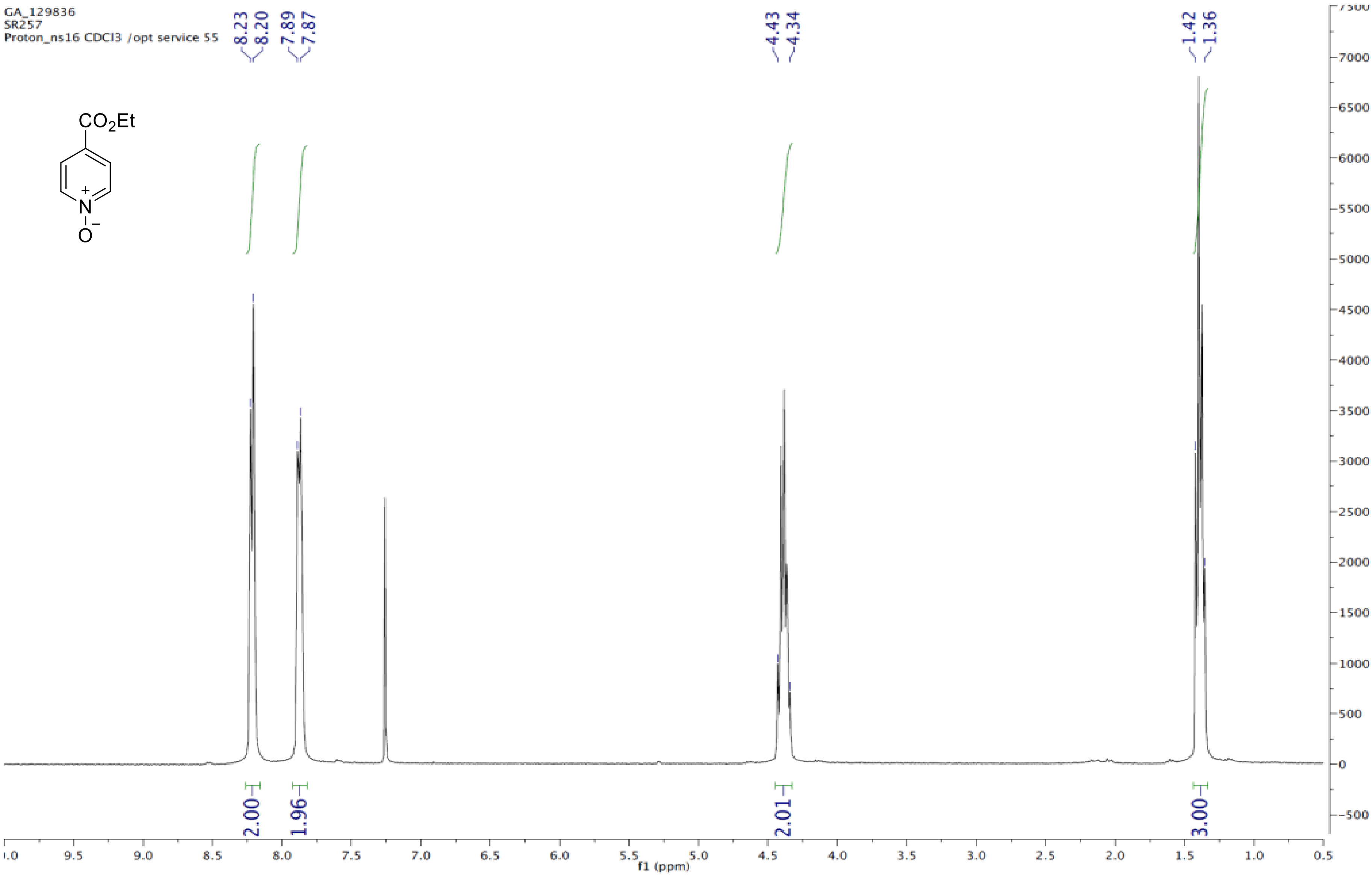

4-(Ethoxycarbonyl)pyridine 1-oxide

^{13}C-NMR, 75 MHz, $CDCl_3$

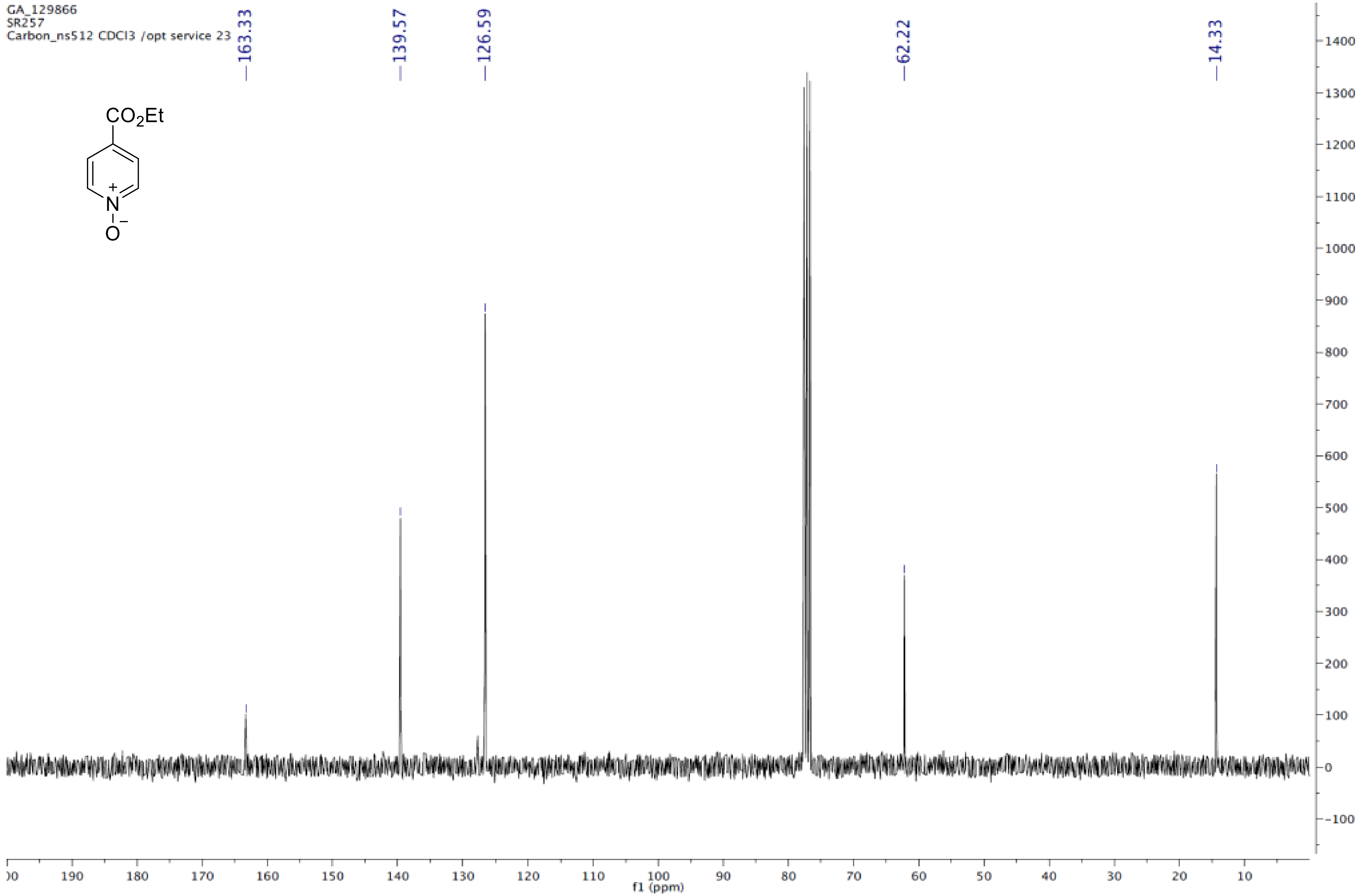

1-Methoxy-4-phenylpyridin-1-ium tetrafluoroborate (1a·BF_4)

^{1}H-NMR, 300 MHz, CD_3CN

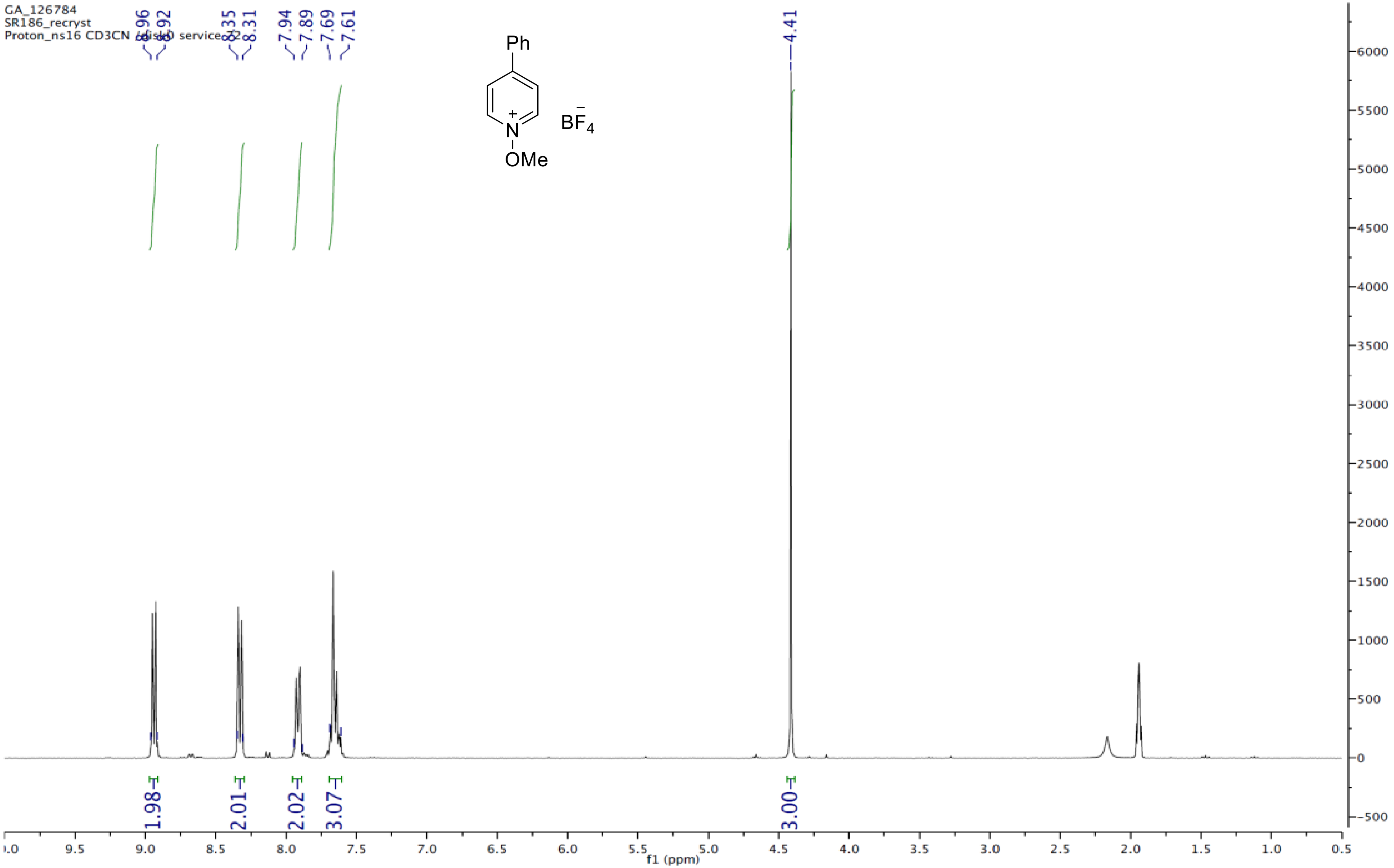

1-Methoxy-4-phenylpyridin-1-ium tetrafluoroborate (1a·BF_4)

^{13}C-NMR, 75 MHz, CD_3CN

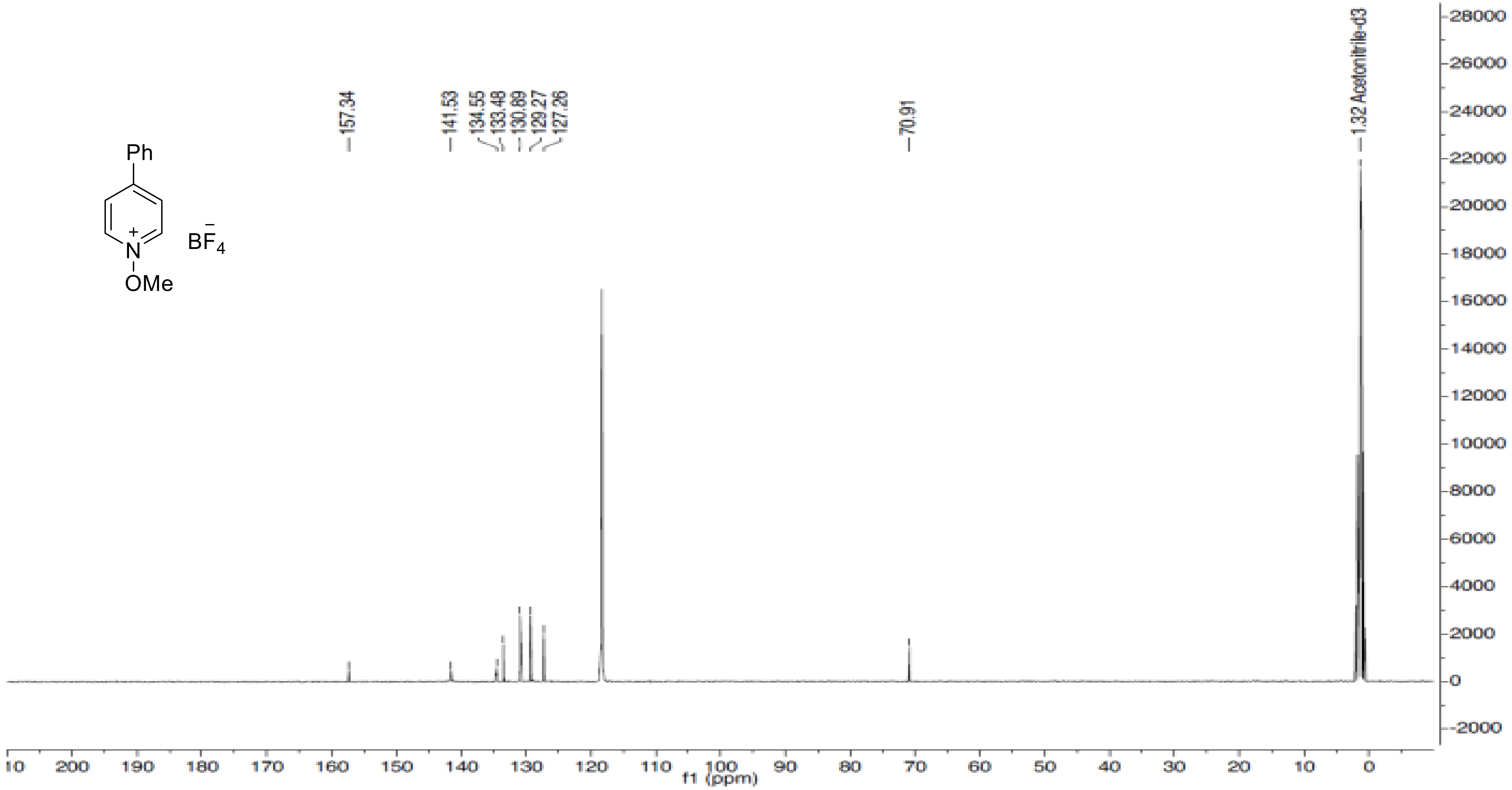

1-Methoxy-4-phenylpyridin-1-ium hexafluorophosphate (1a·PF_6)

^{1}H-NMR, 300 MHz, CD_3CN

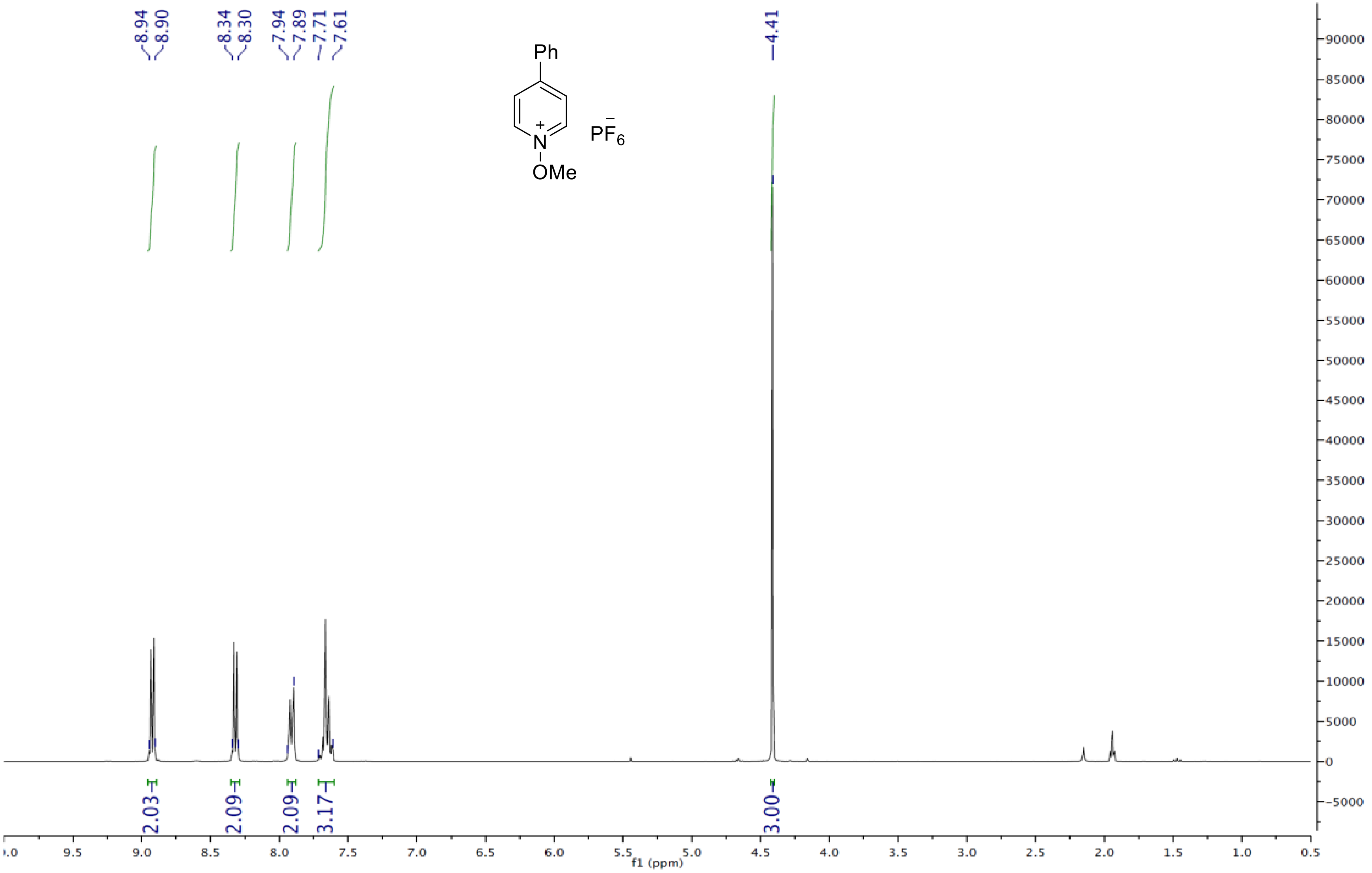

1-Methoxy-4-phenylpyridin-1-ium hexafluorophosphate (1a·PF_6)

^{13}C-NMR, 75 MHz, CD_3CN

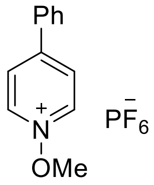

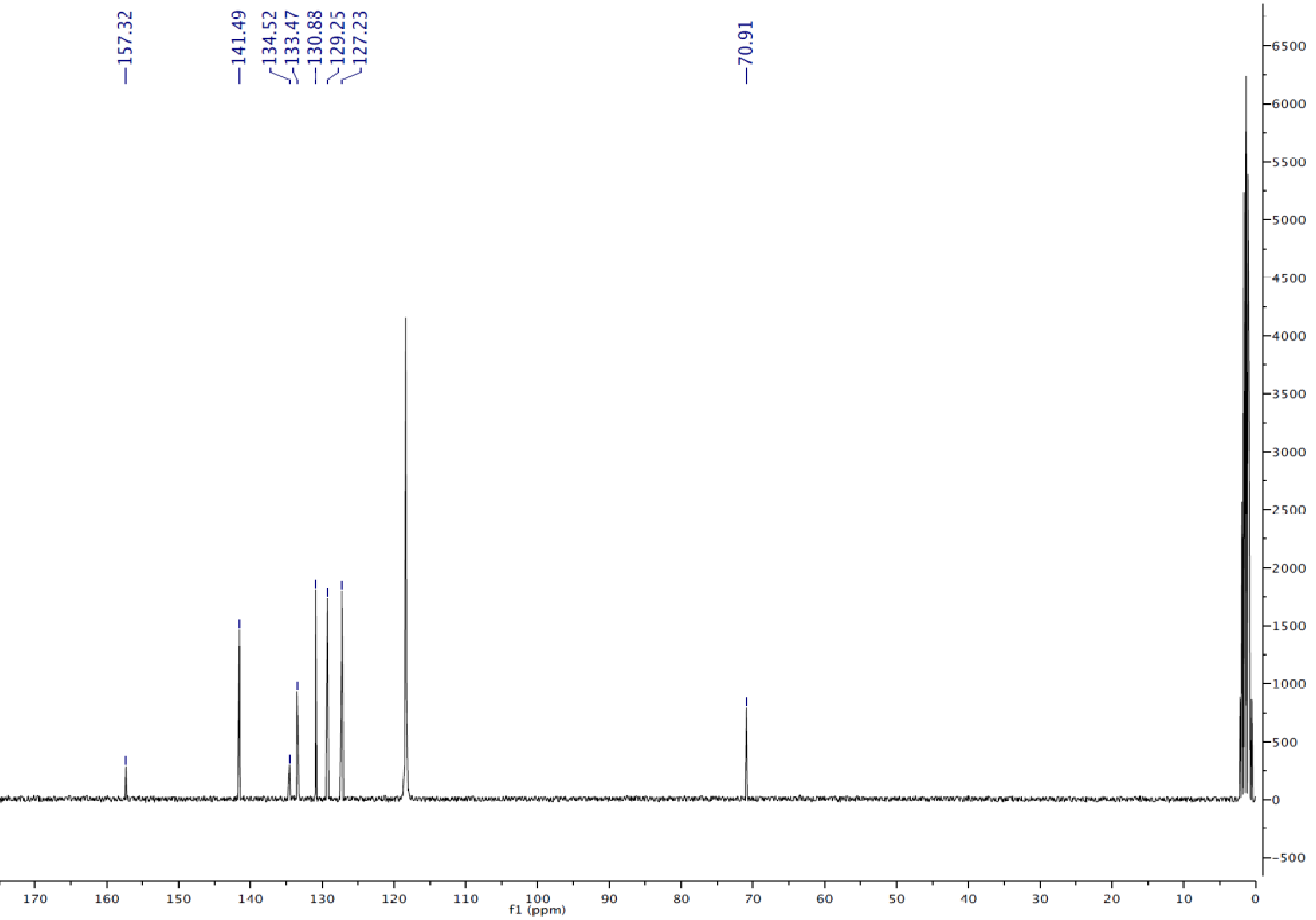

1-Methoxy-4-methylquinolin-1-ium tetrafluoroborate (1b·BF_4)

^1H-NMR, 300 MHz, CD_3CN

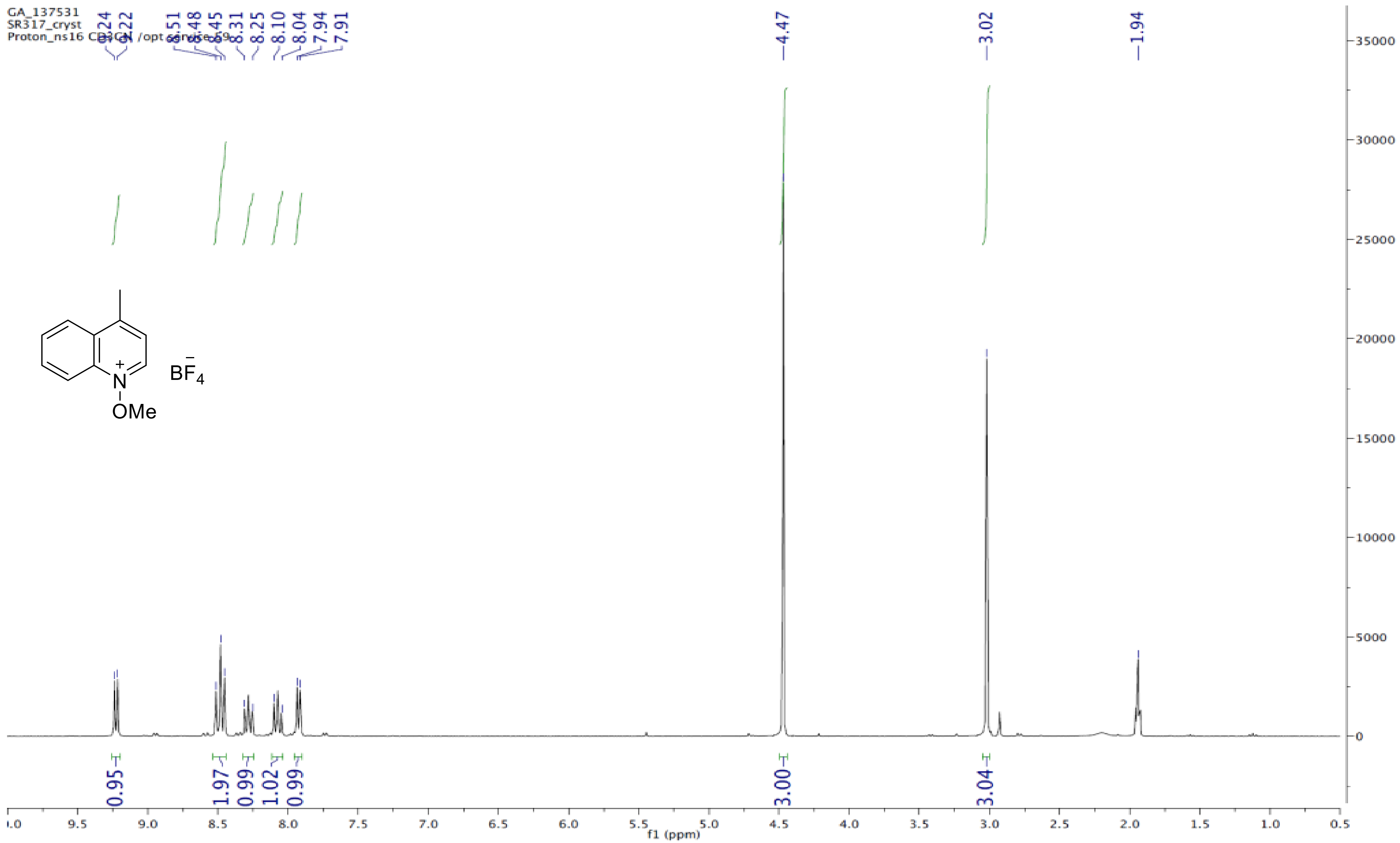

1-Methoxy-4-methylquinolin-1-ium tetrafluoroborate (1b·BF_4)

^{13}C-NMR, 75 MHz, CD_3CN

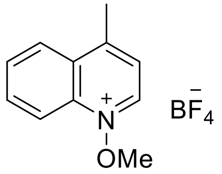

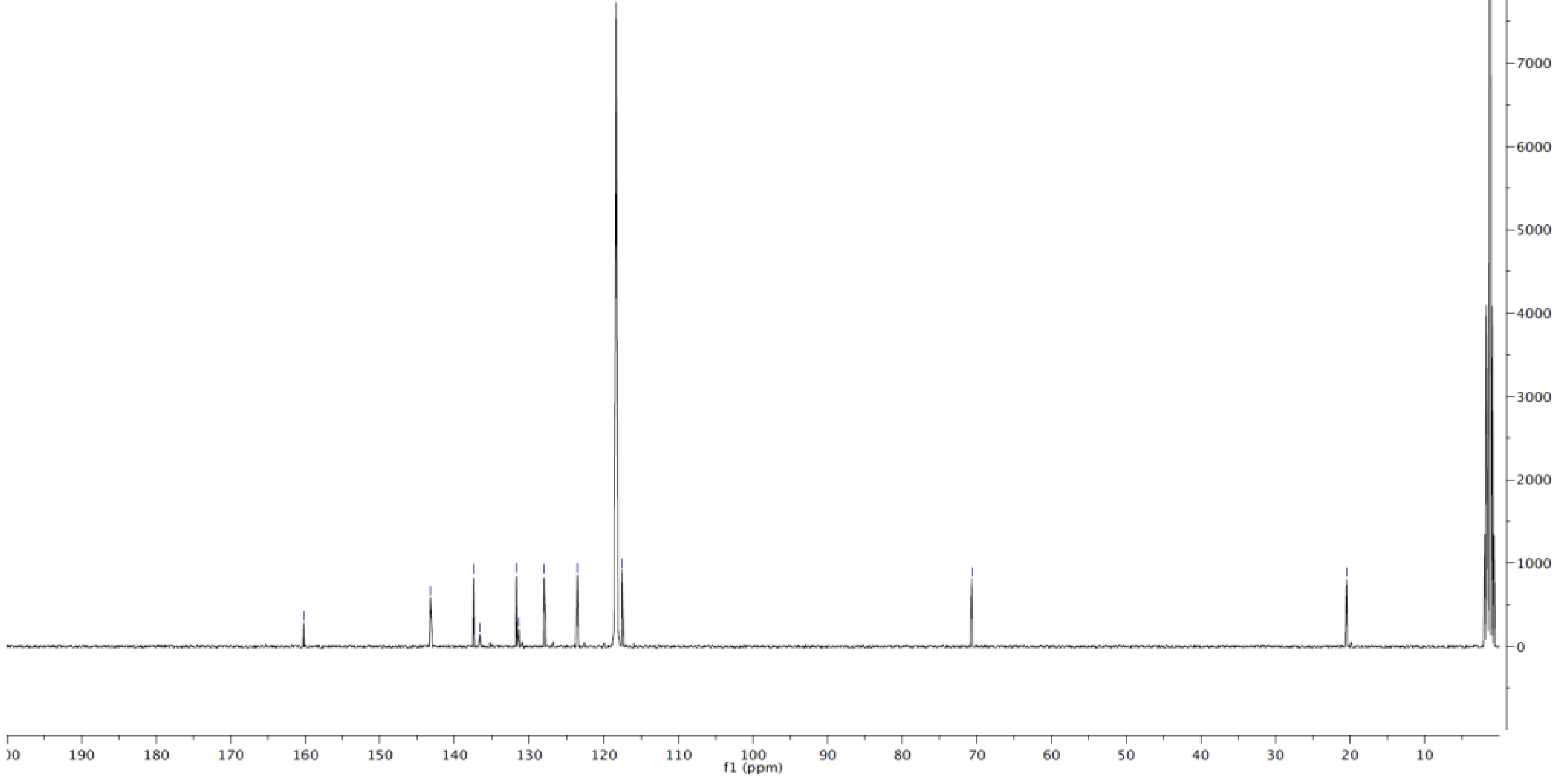

1-Methoxy-4-chloroquinolin-1-ium tetrafluoroborate (1c·BF_4)

^{1}H-NMR, 300 MHz, CD_3CN

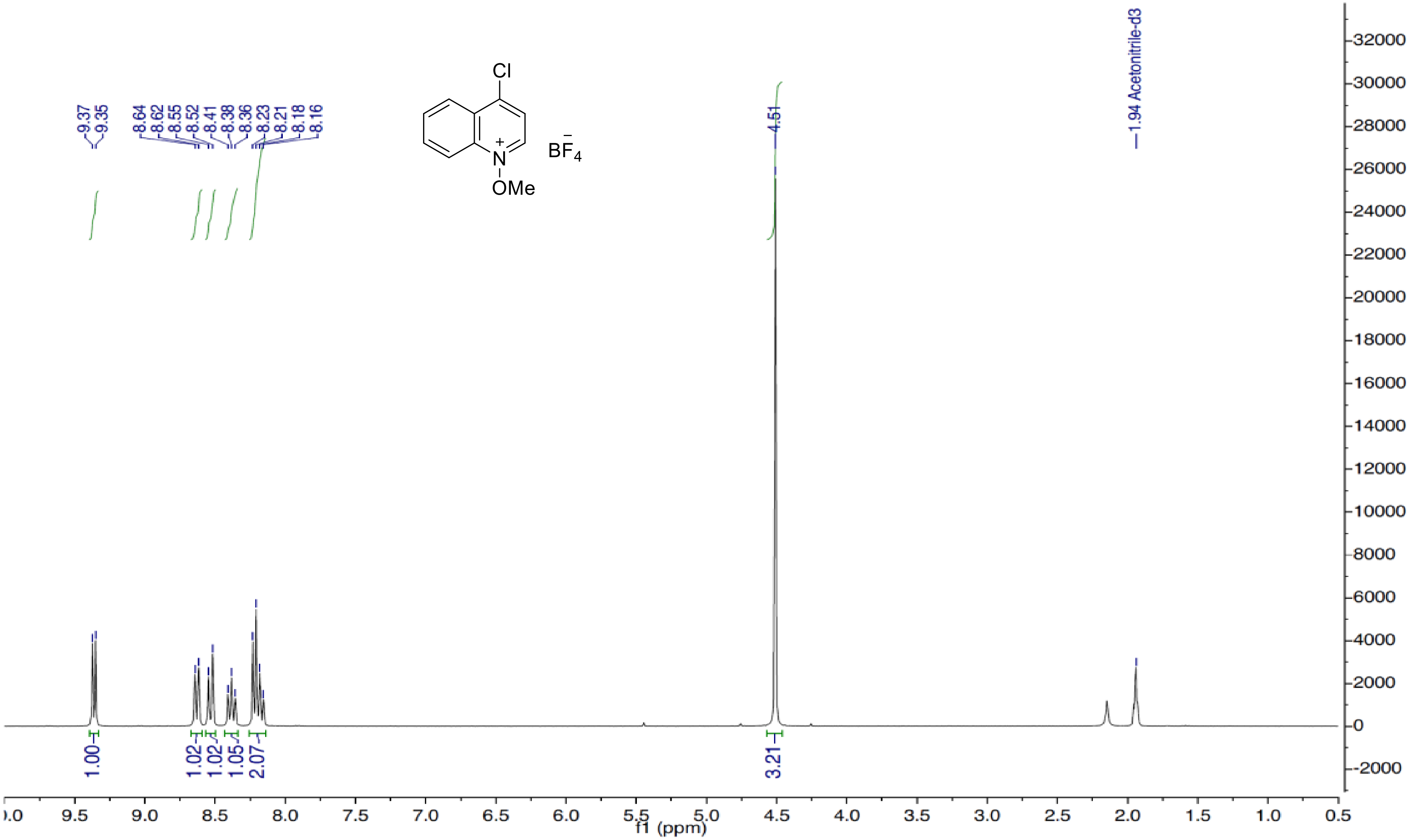

1-Methoxy-4-chloroquinolin-1-ium tetrafluoroborate (1c·BF_4)

^{13}C-NMR, 75 MHz, CD_3CN

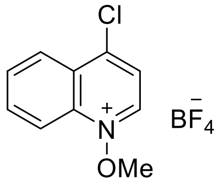

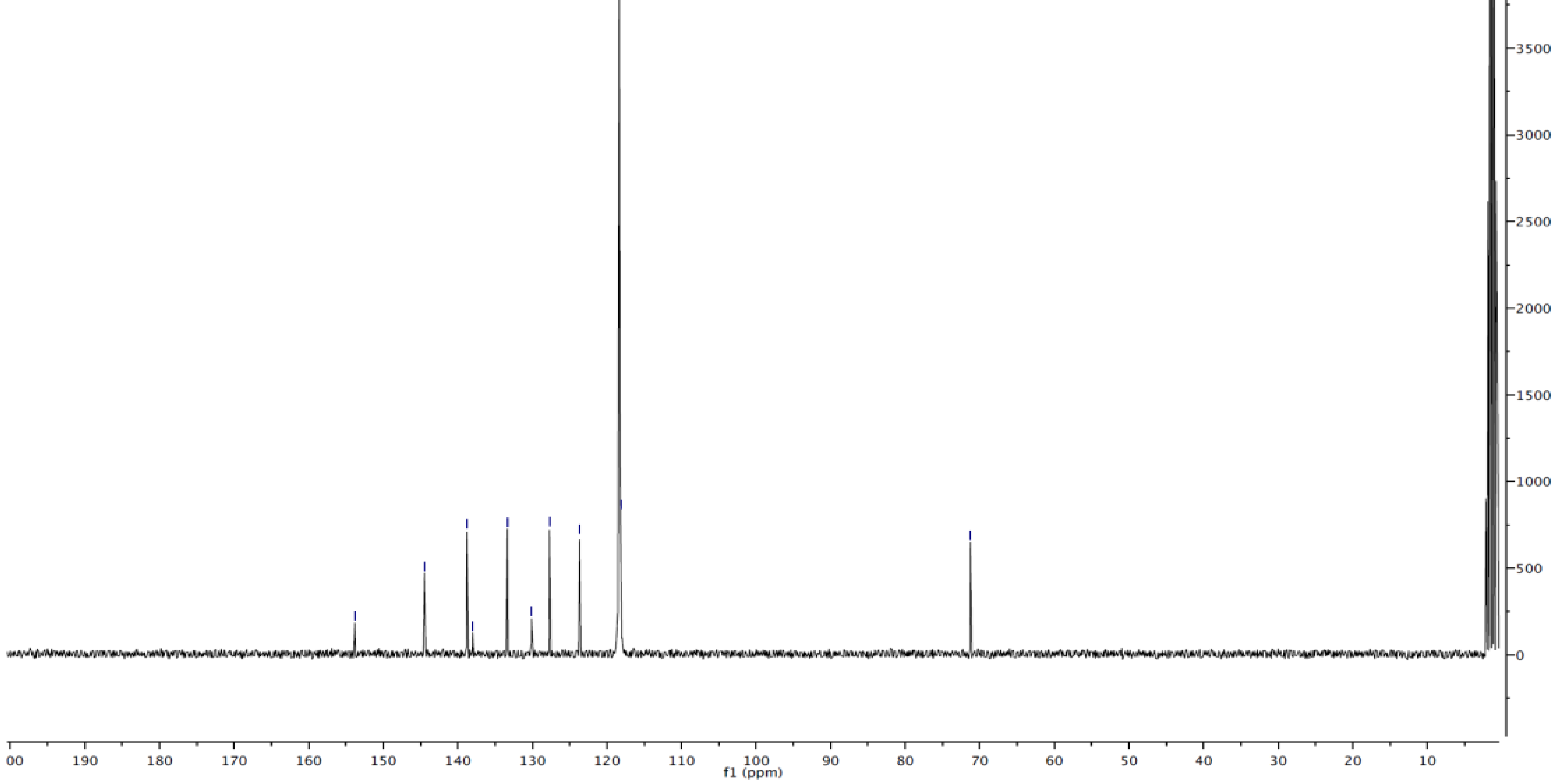

5-Methoxyphenanthridin-5-ium tetrafluoroborate (1d·BF_4)

^{1}H-NMR,300 MHz, CD_3CN

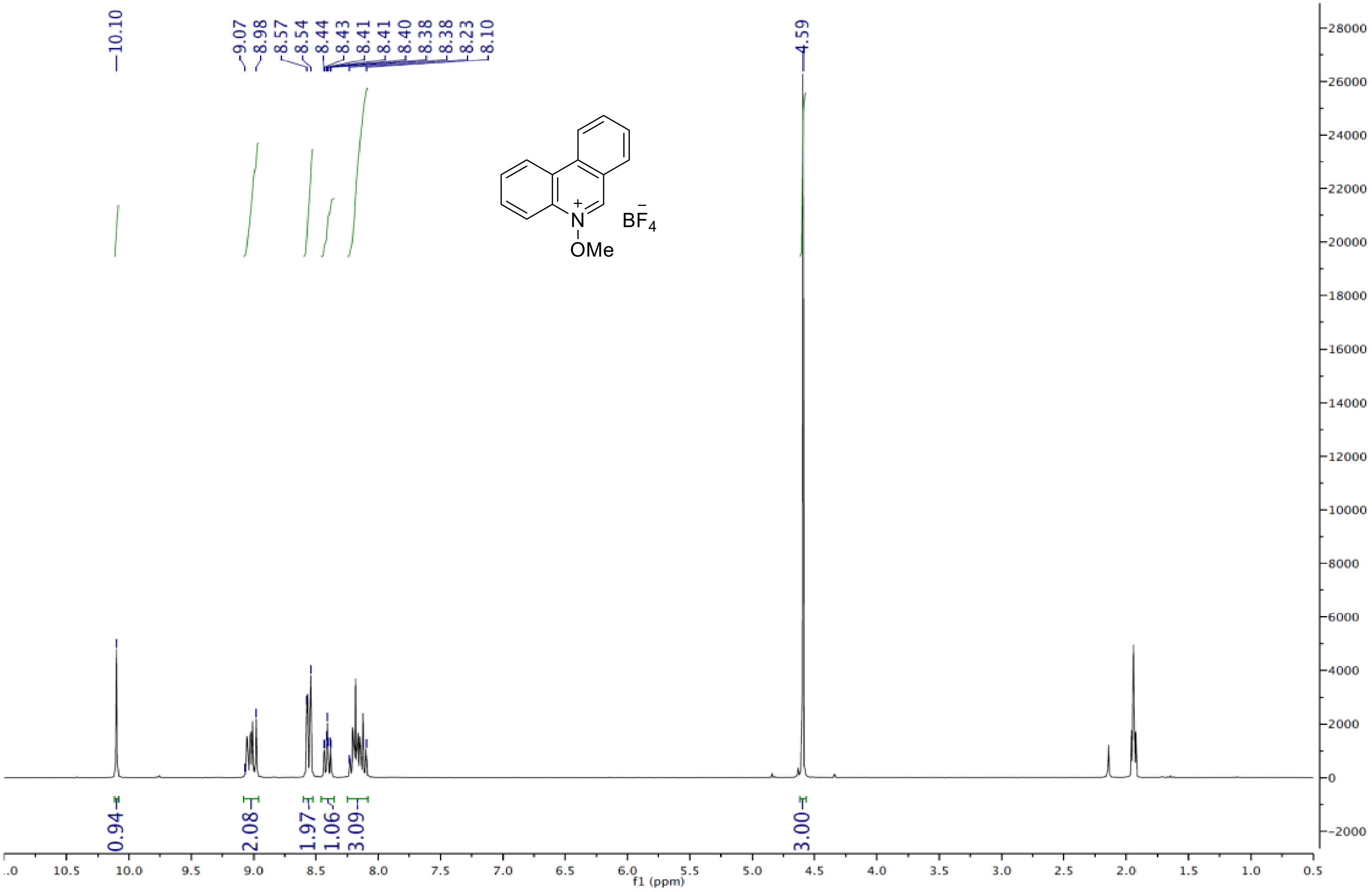

5-Methoxyphenanthridin-5-ium tetrafluoroborate (1d·BF_4)

^{13}C-NMR, 75 MHz, CD_3CN

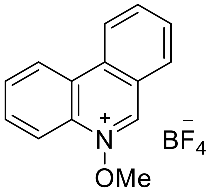

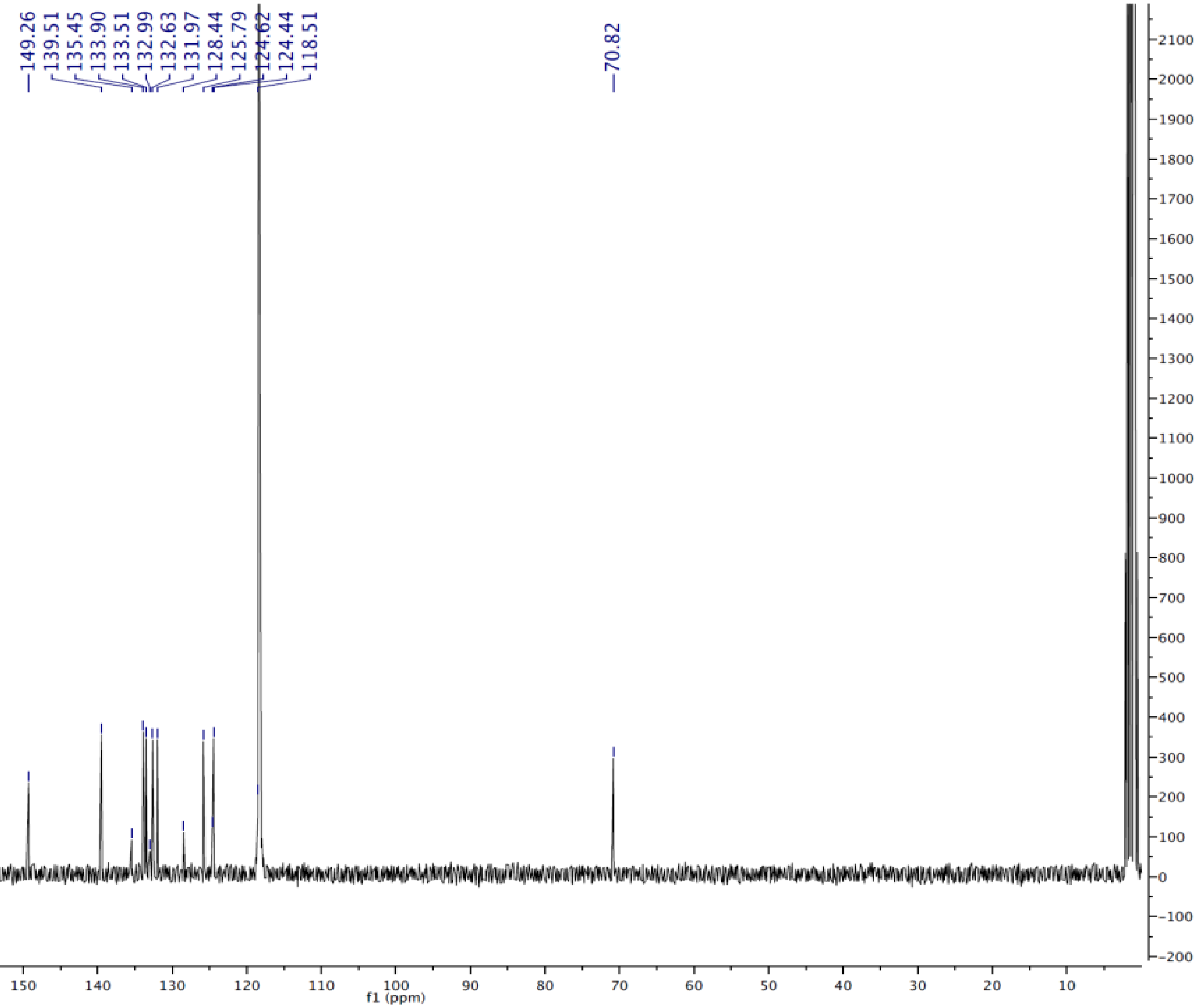

5-Methoxyphenanthridin-5-ium tetrafluoroborate (1e·BF_4)

^1H-NMR, 300 MHz, CD_3CN

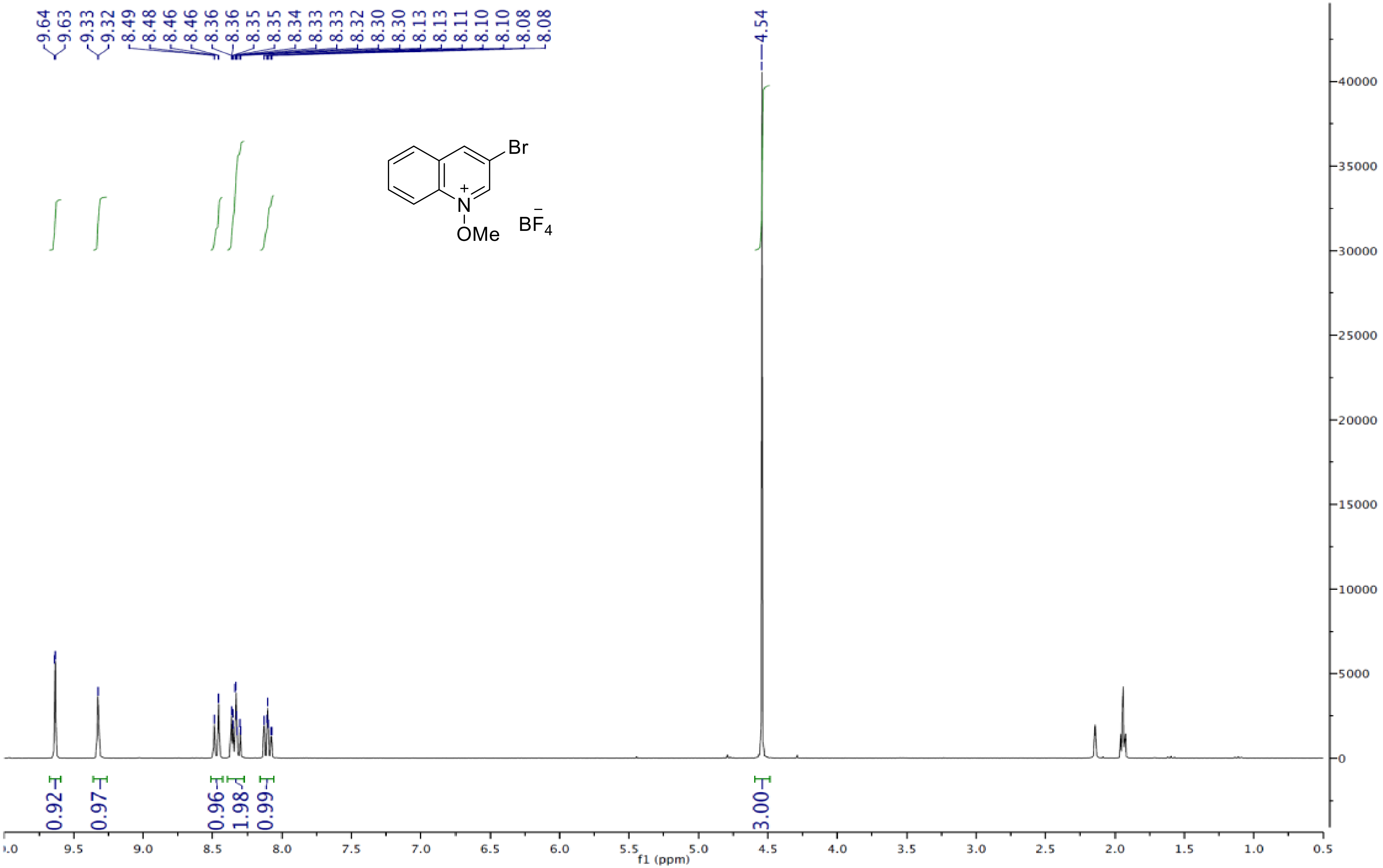

5-Methoxyphenanthridin-5-ium tetrafluoroborate (1e·BF_4) ^{13}C-NMR, 75 MHz, CD_3CN

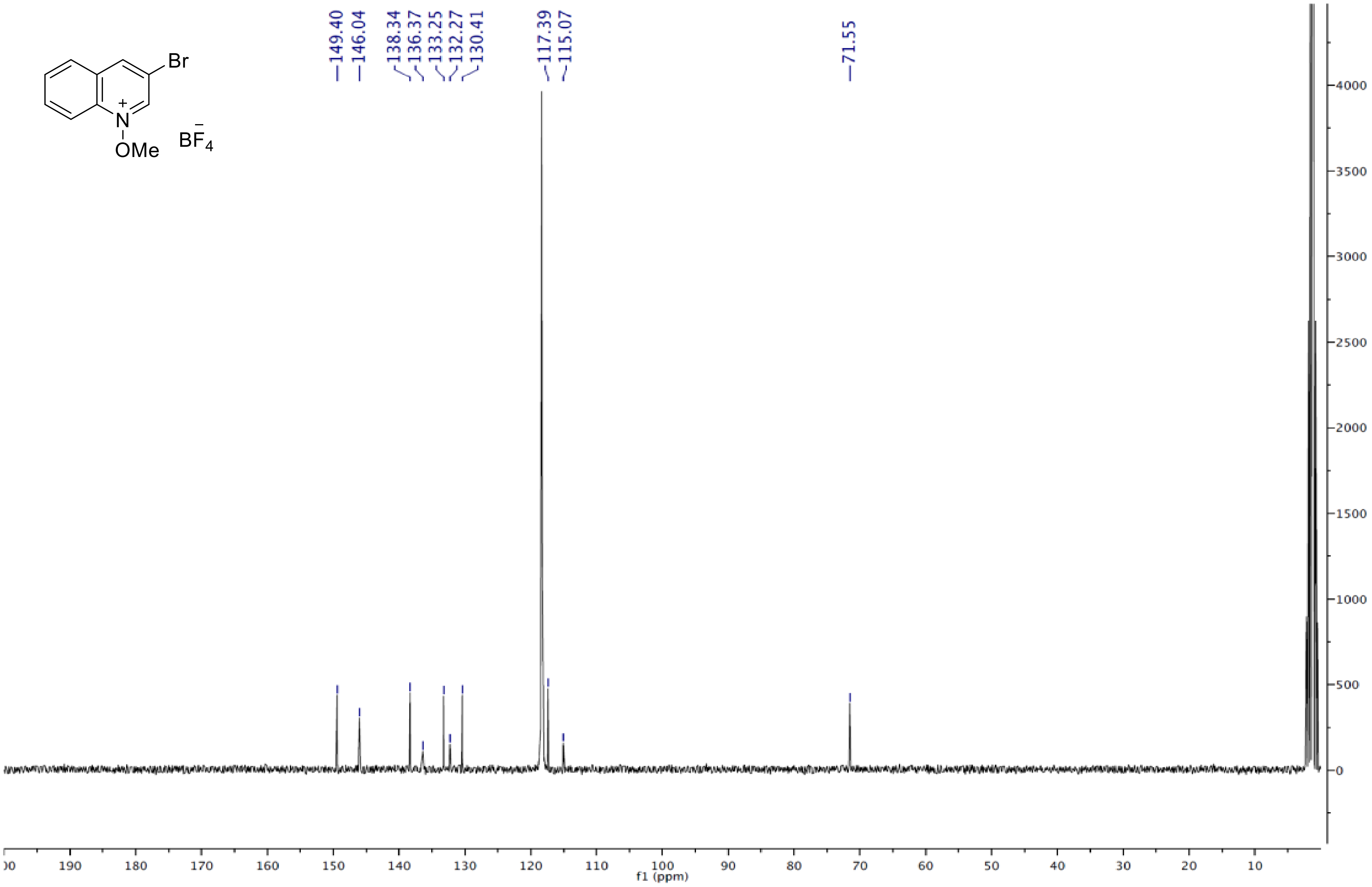

1-Methoxy-2-methylquinolin-1-ium tetrafluoroborate (1f·BF_4)

^{1}H-NMR, 300 MHz, CD_3CN

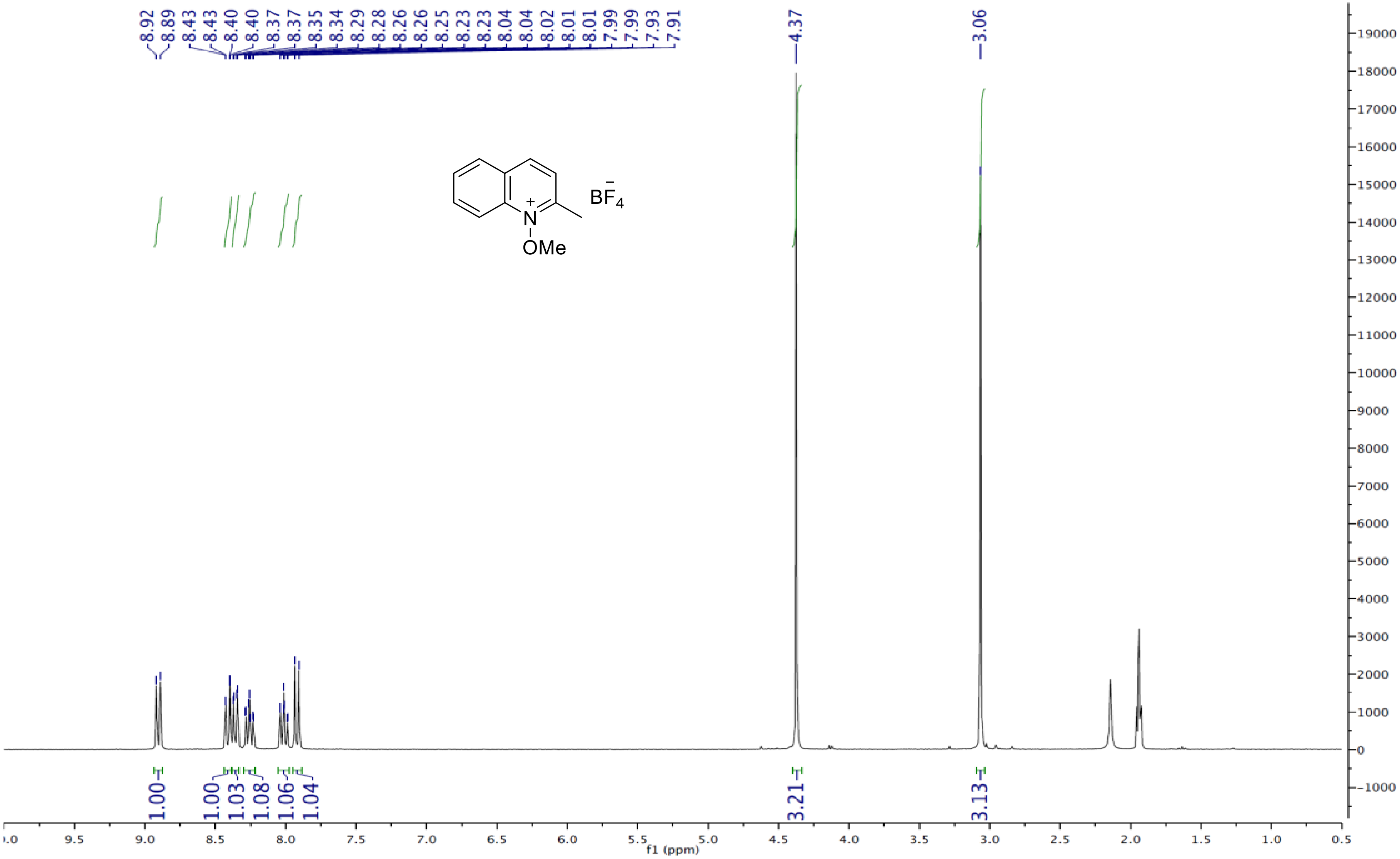

1-Methoxy-2-methylquinolin-1-ium tetrafluoroborate (1f·BF_4)

^{13}C-NMR, 75 MHz, CD_3CN

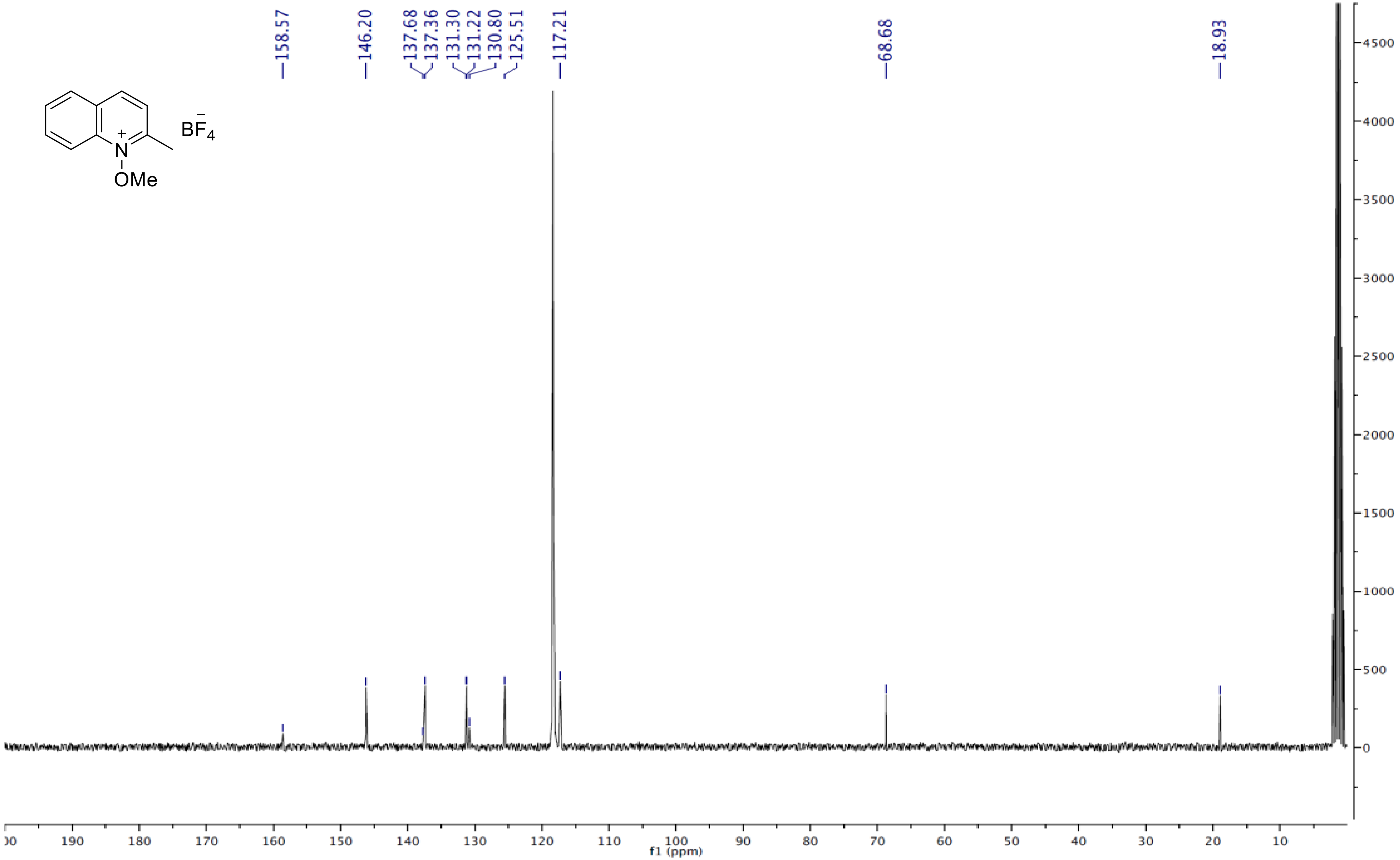

1-Methoxy-4-*tert*-butylpyridinium hexafluorophosphate (1g·PF_6)

^{1}H-NMR, 300 MHz, CD_3CN

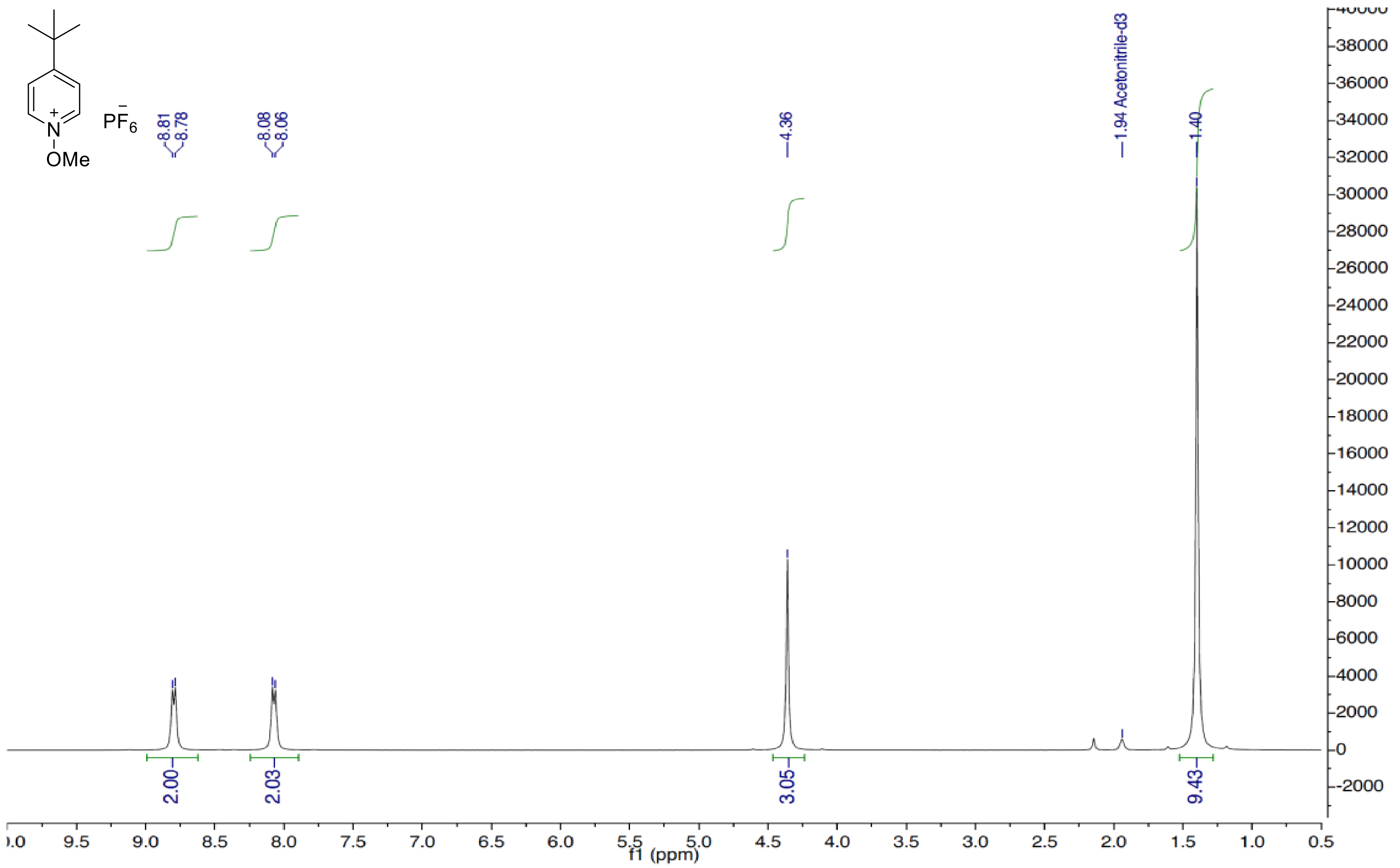

1-Methoxy-4-*tert*-butylpyridinium hexafluorophosphate (1g·PF_6)

^{13}C-NMR, 75 MHz, CD_3CN

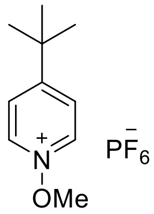

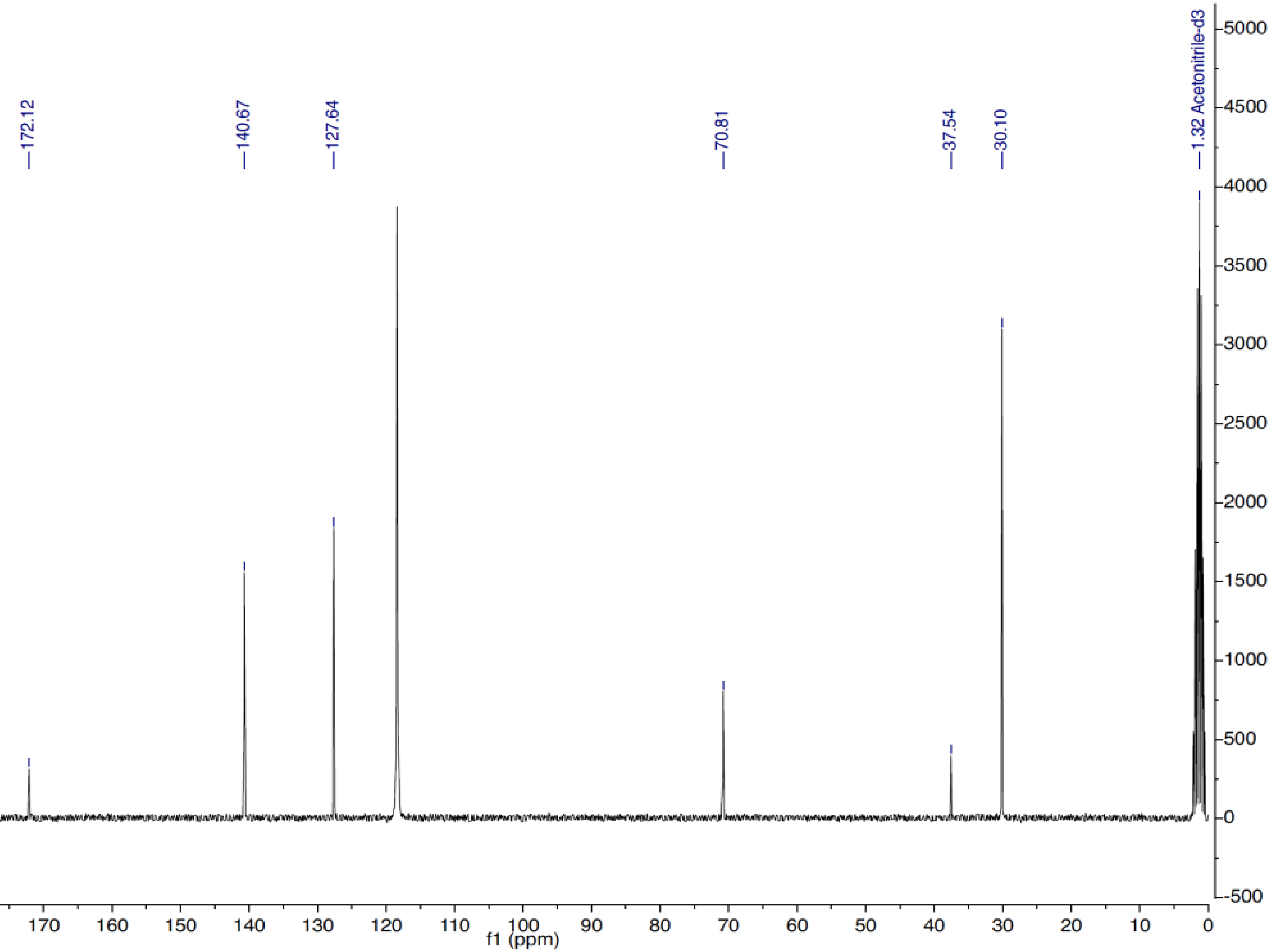

1-Methoxy-2,6-dimethylpyridin-1-ium tetrafluoroborate (1i·BF4)

^{1}H-NMR, 300 MHz, CD_3CN

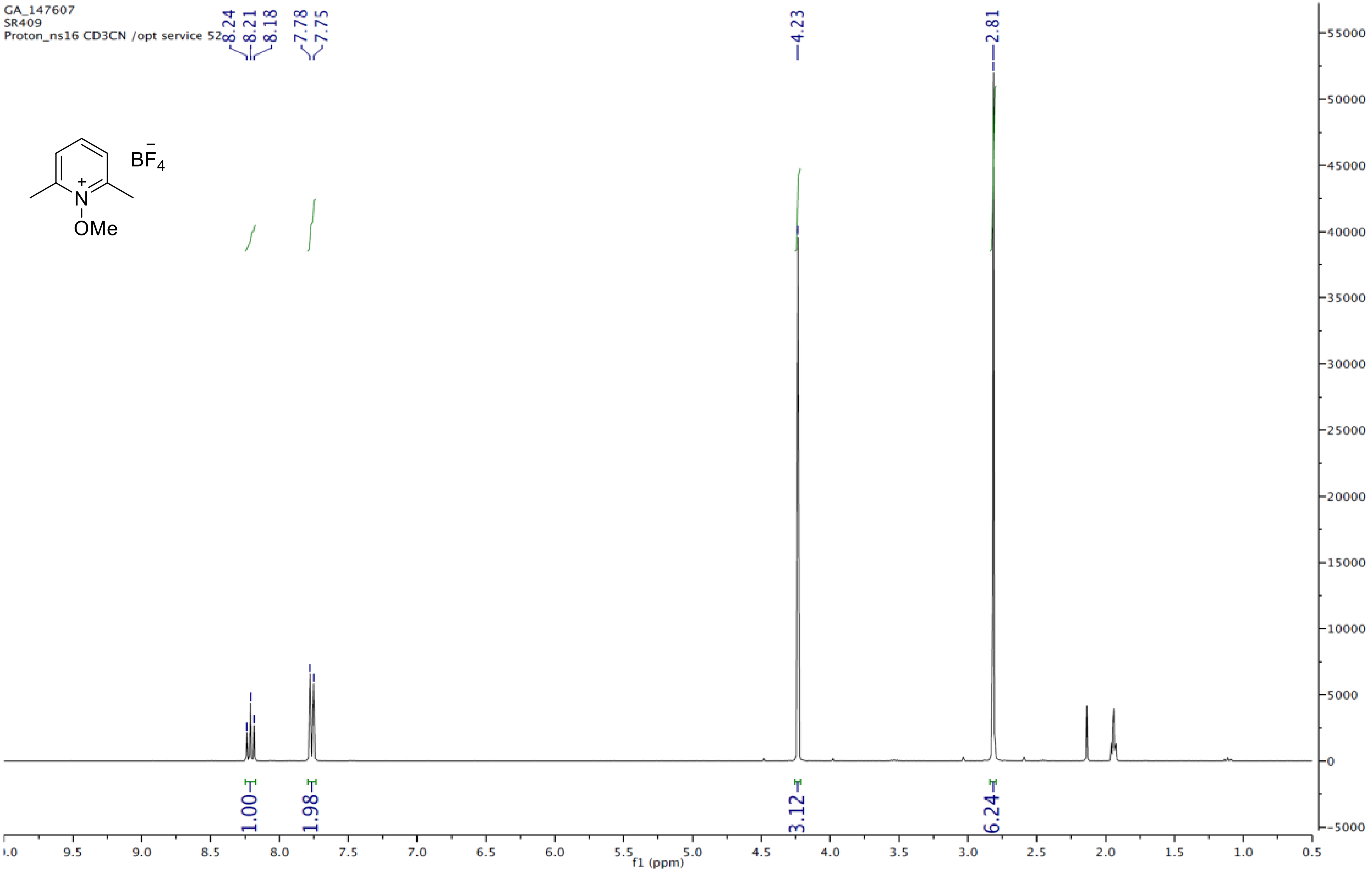

1-Methoxy-2,6-dimethylpyridin-1-ium tetrafluoroborate (1i·BF4)

^{13}C-NMR, 75 MHz, DMSO-d6

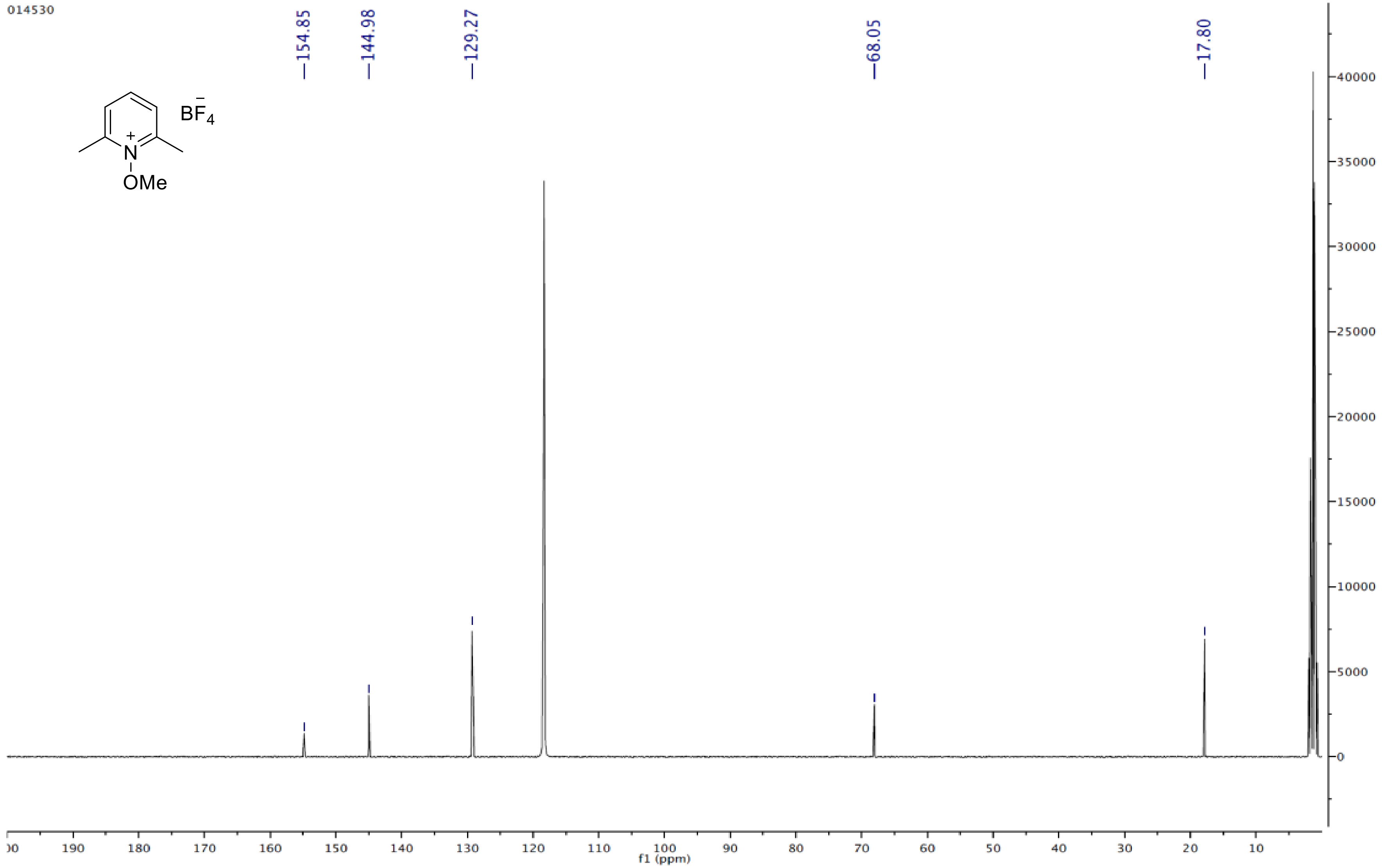

4-Chloro-1-methoxypyridin-1-ium tetrafluoroborate (1j·BF_4)

^{1}H-NMR, 300 MHz, DMSO-d6

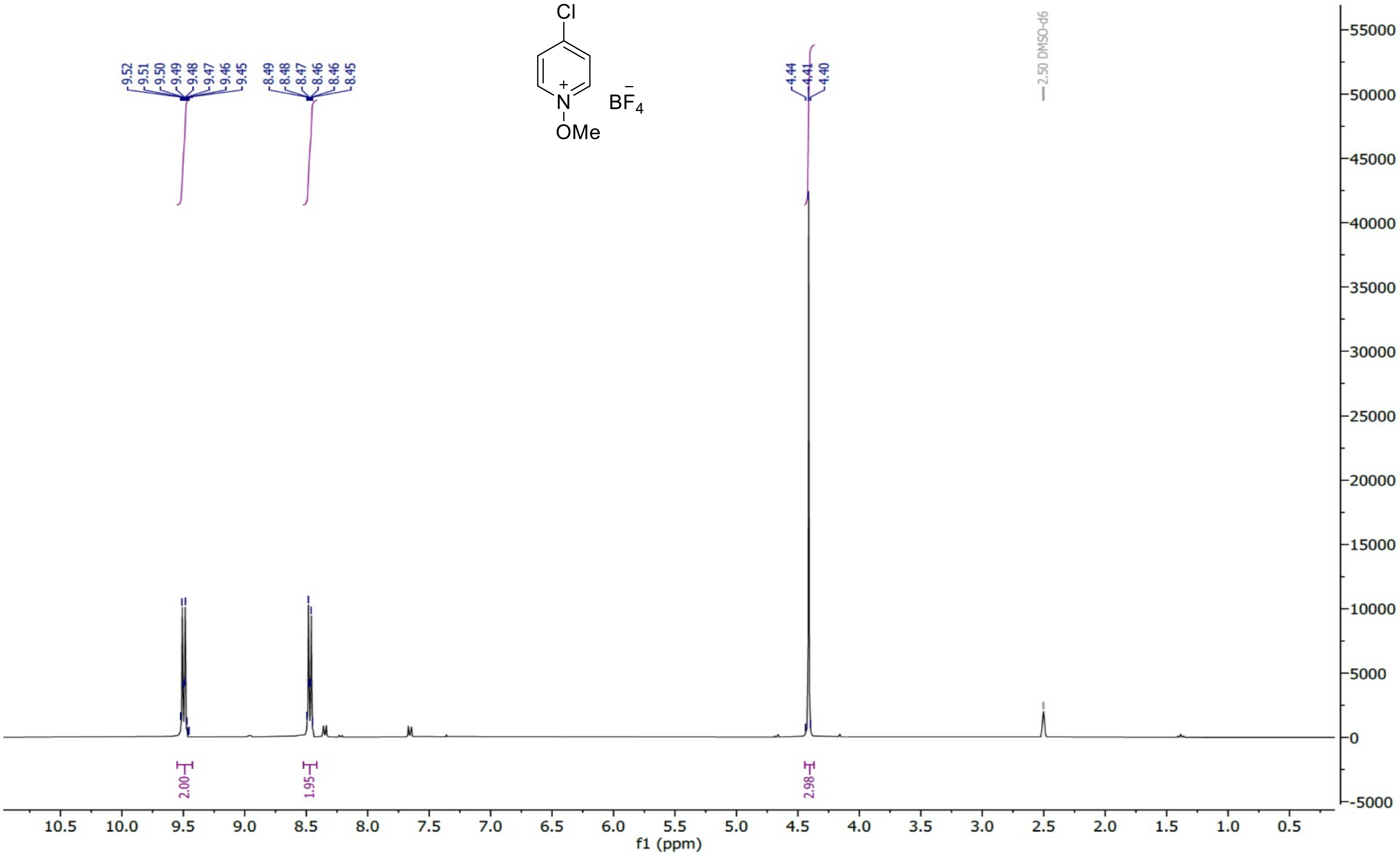

4-Chloro-1-methoxypyridin-1-ium tetrafluoroborate (1j·BF_4)

^{13}C-NMR, 75 MHz, DMSO-d6

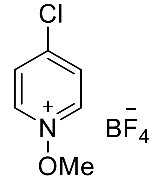

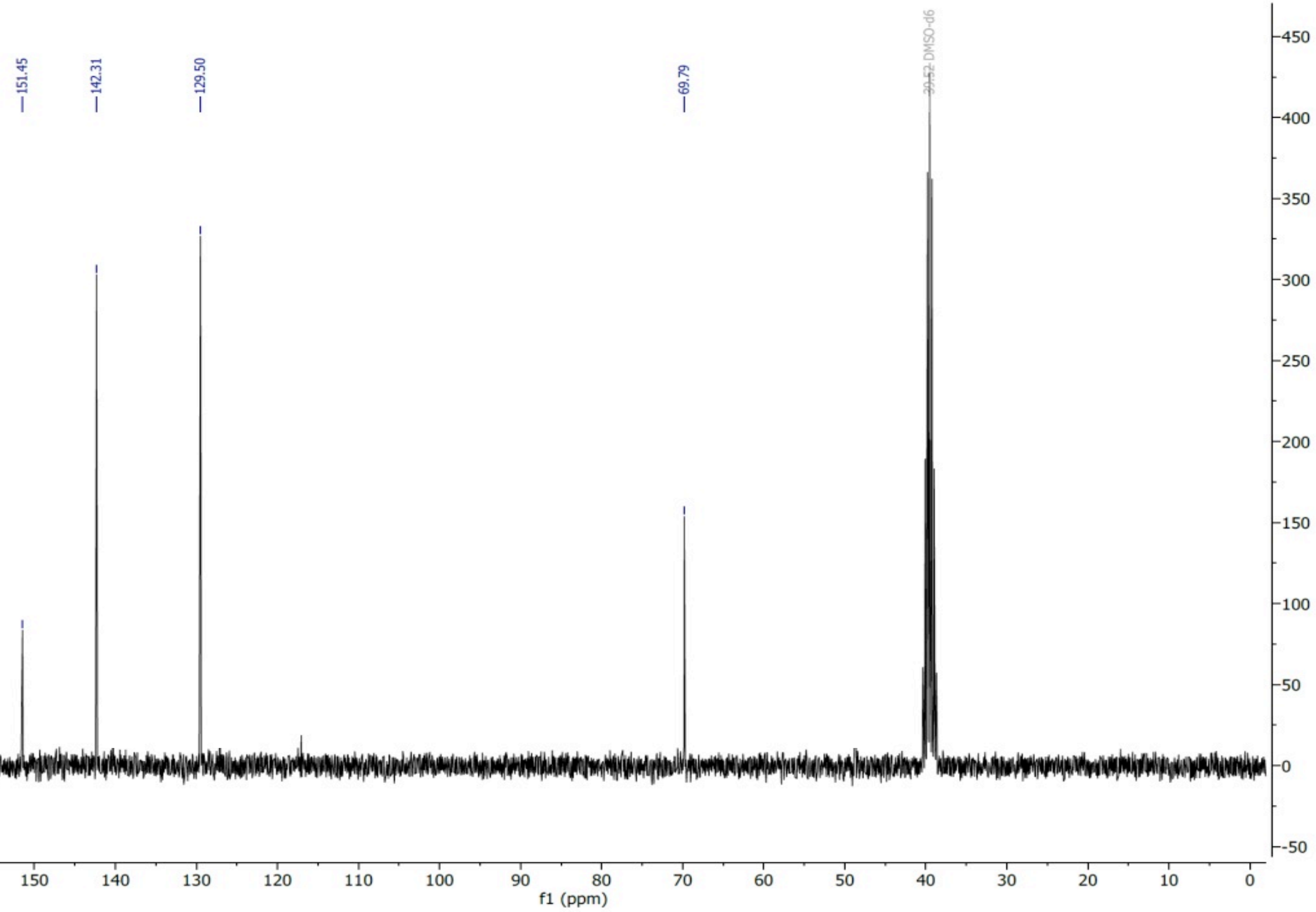

4-Bromo-1-methoxypyridin-1-ium tetrafluoroborate (1k·BF_4)

^{13}C-NMR, 300 MHz, DMSO-d6

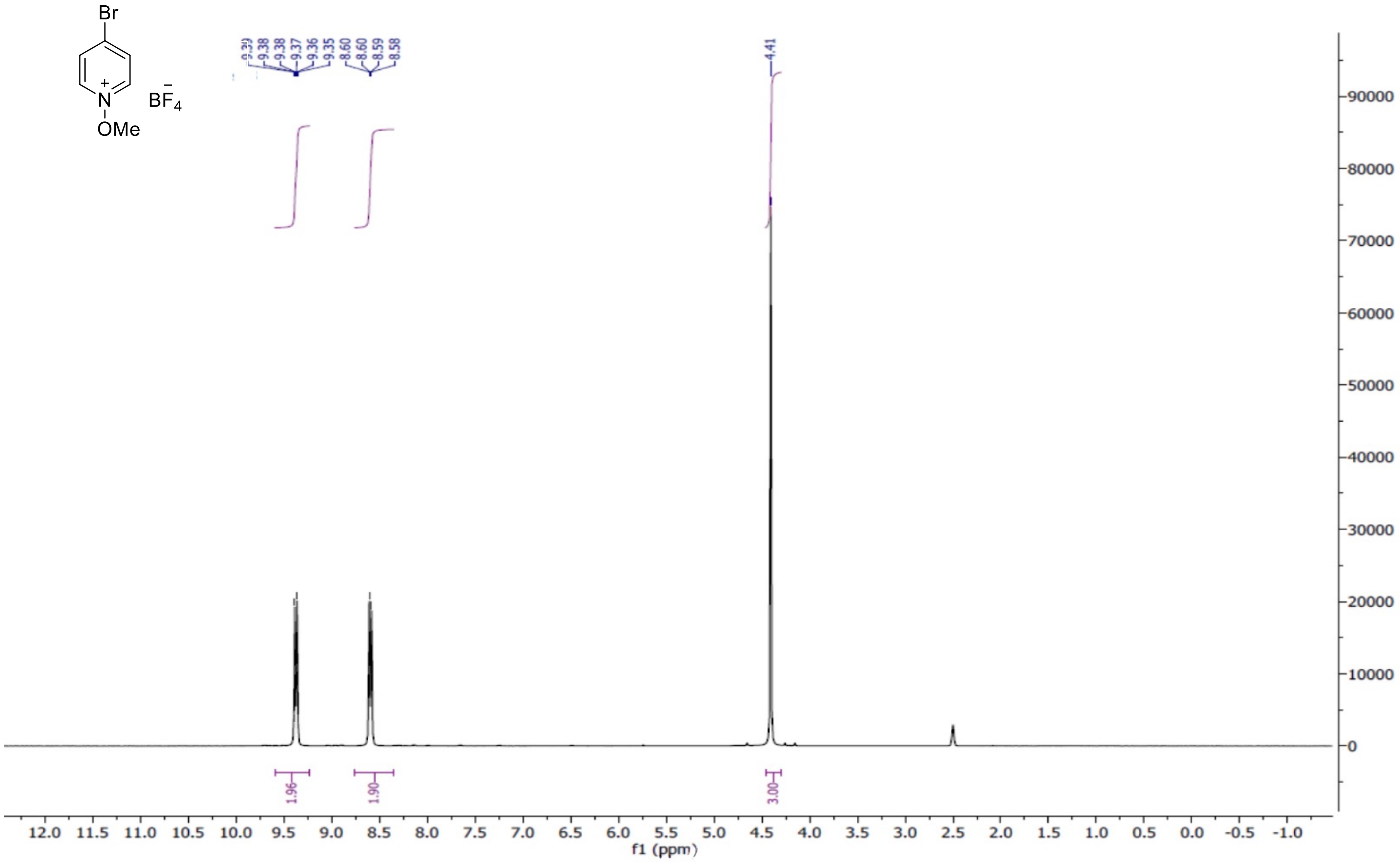

4-Bromo-1-methoxypyridin-1-ium tetrafluoroborate (1k·BF_4)

^{13}C-NMR, 75 MHz, DMSO-d6

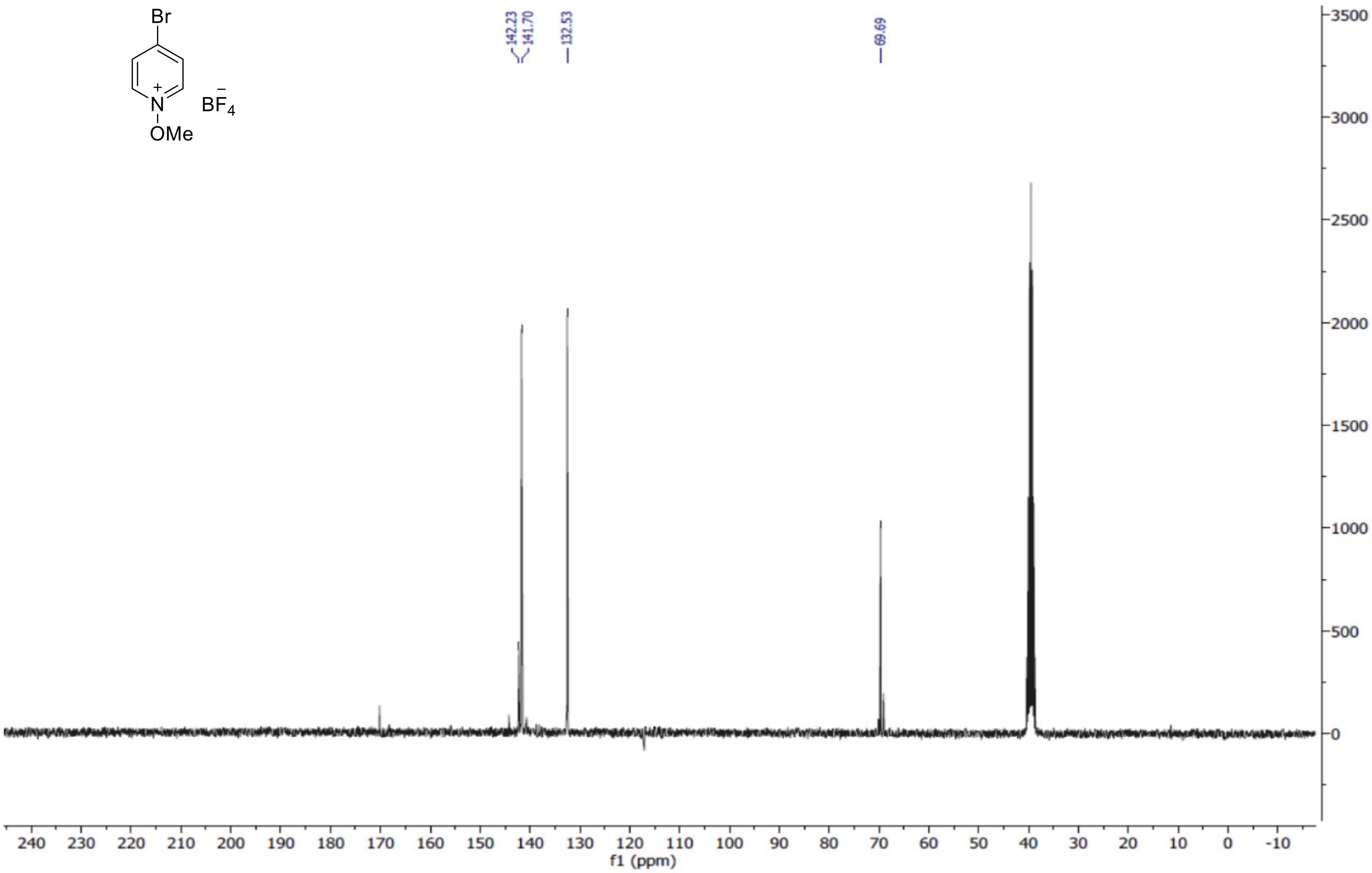

2,6-Dichloro-1-methoxypyridin-1-ium tetrafluoroborate (1l·BF_4)

^{1}H-NMR, 300 MHz, DMSO-d6

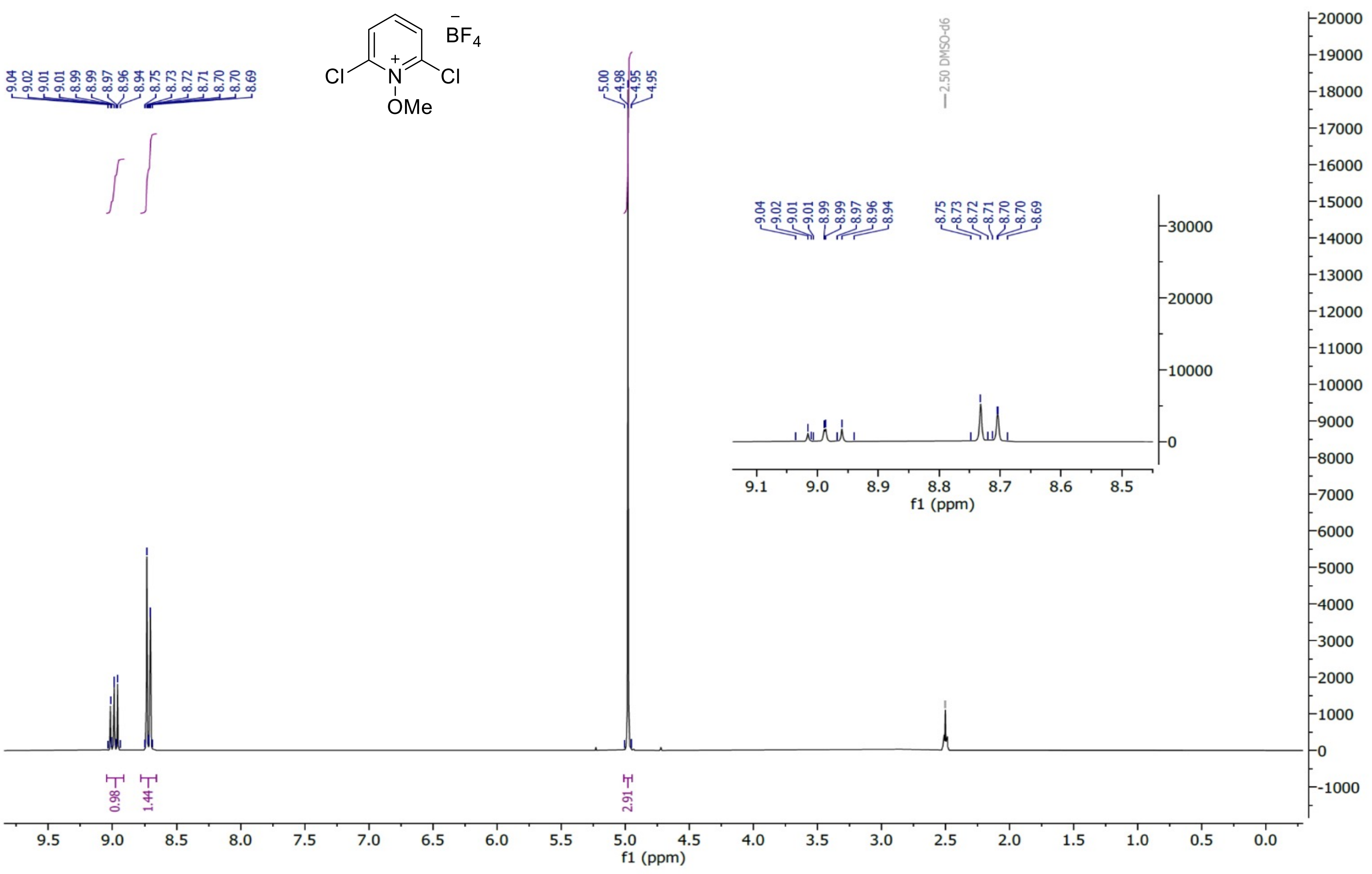

2,6-Dichloro-1-methoxypyridin-1-ium tetrafluoroborate (1l·BF_4)

^{13}C-NMR, 75 MHz, CD_3CN

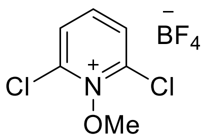

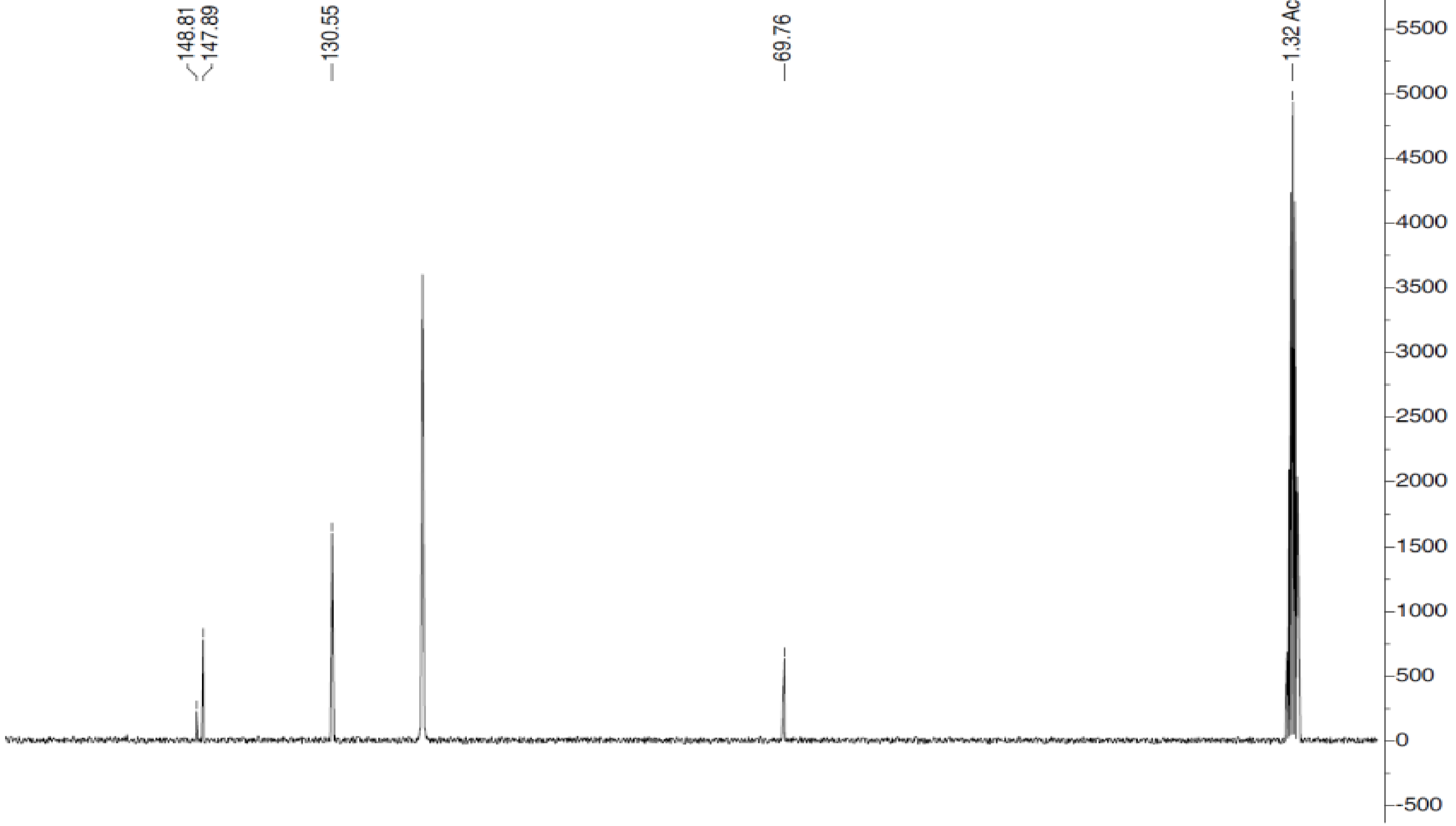

2,4-Dichloro-1-methoxypyridin-1-ium tetrafluoroborate (1m·BF_4)

^{13}C-NMR, 75 MHz, CD_3CN

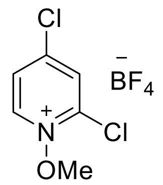

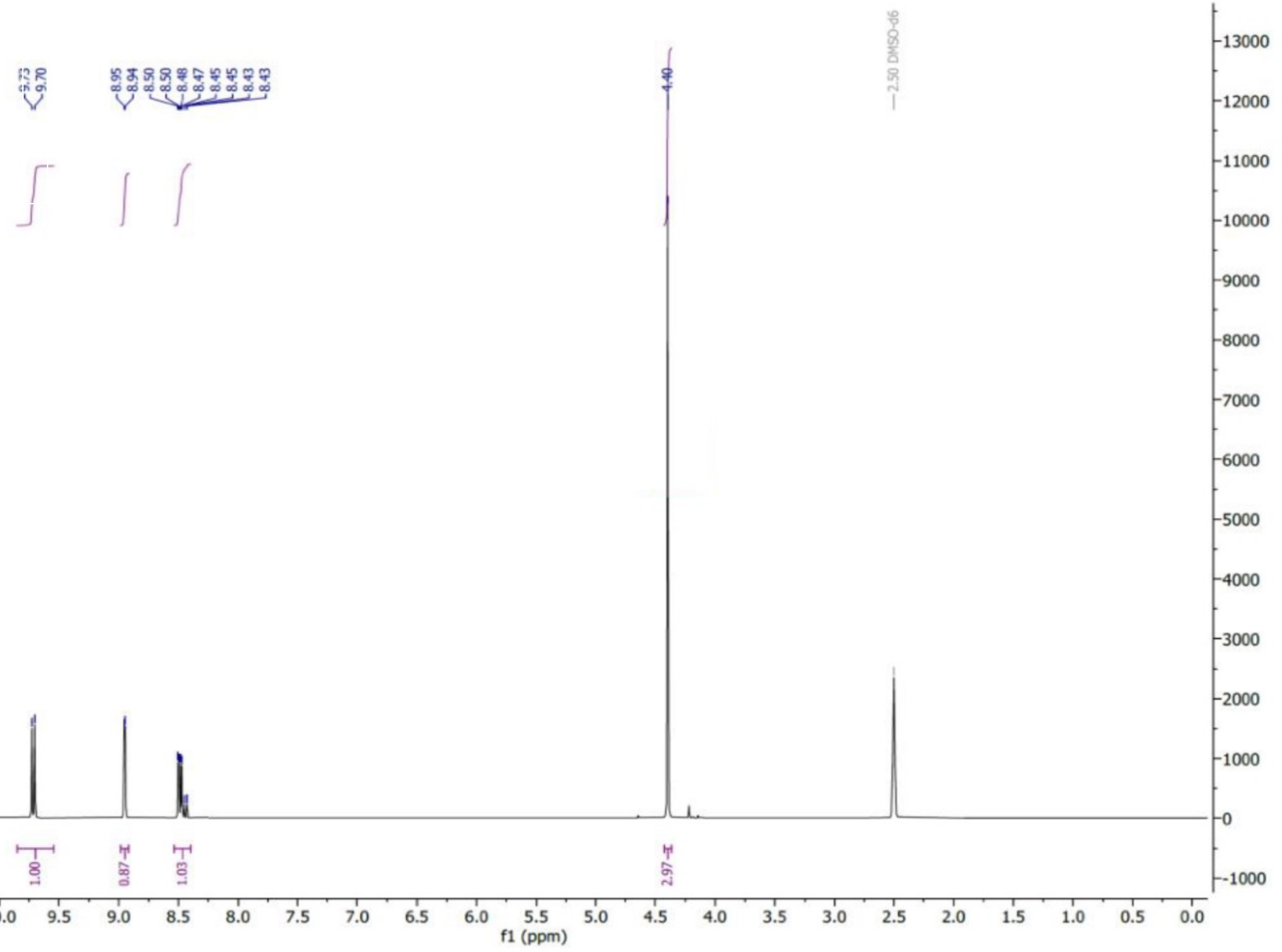

2,4-Dichloro-1-methoxypyridin-1-ium tetrafluoroborate (1m·BF_4)

^{13}C-NMR, 75MHz, $CDCl_3$

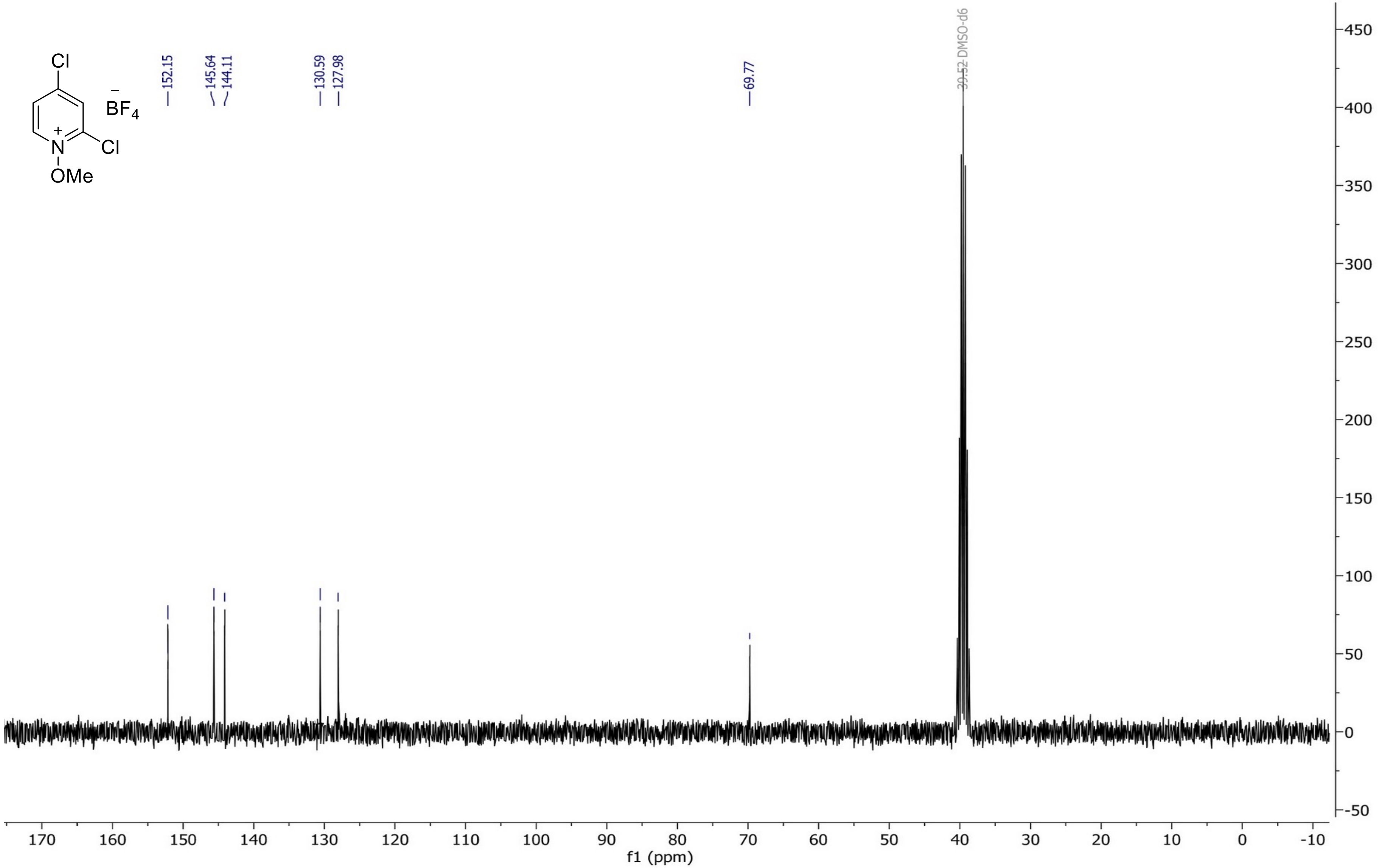

2-Chloro-1-methoxypyridin-1-ium tetrafluoroborate (1n·BF_4)

^{1}H-NMR, 300 MHz, DMSO-d6

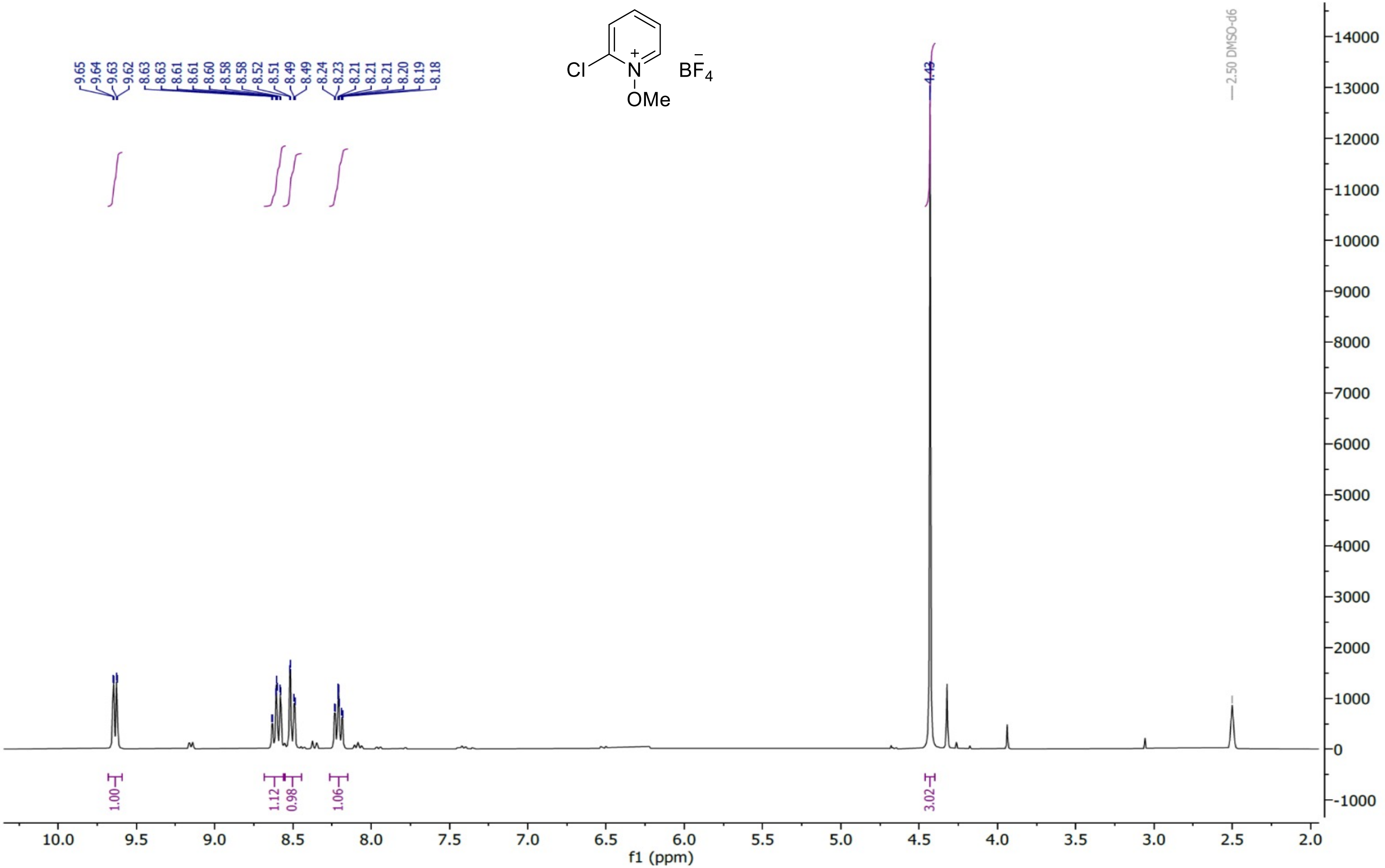

2-Chloro-1-methoxypyridin-1-ium tetrafluoroborate (1n·BF_4)

^{13}C-NMR, 75 MHz, DMSO-d6

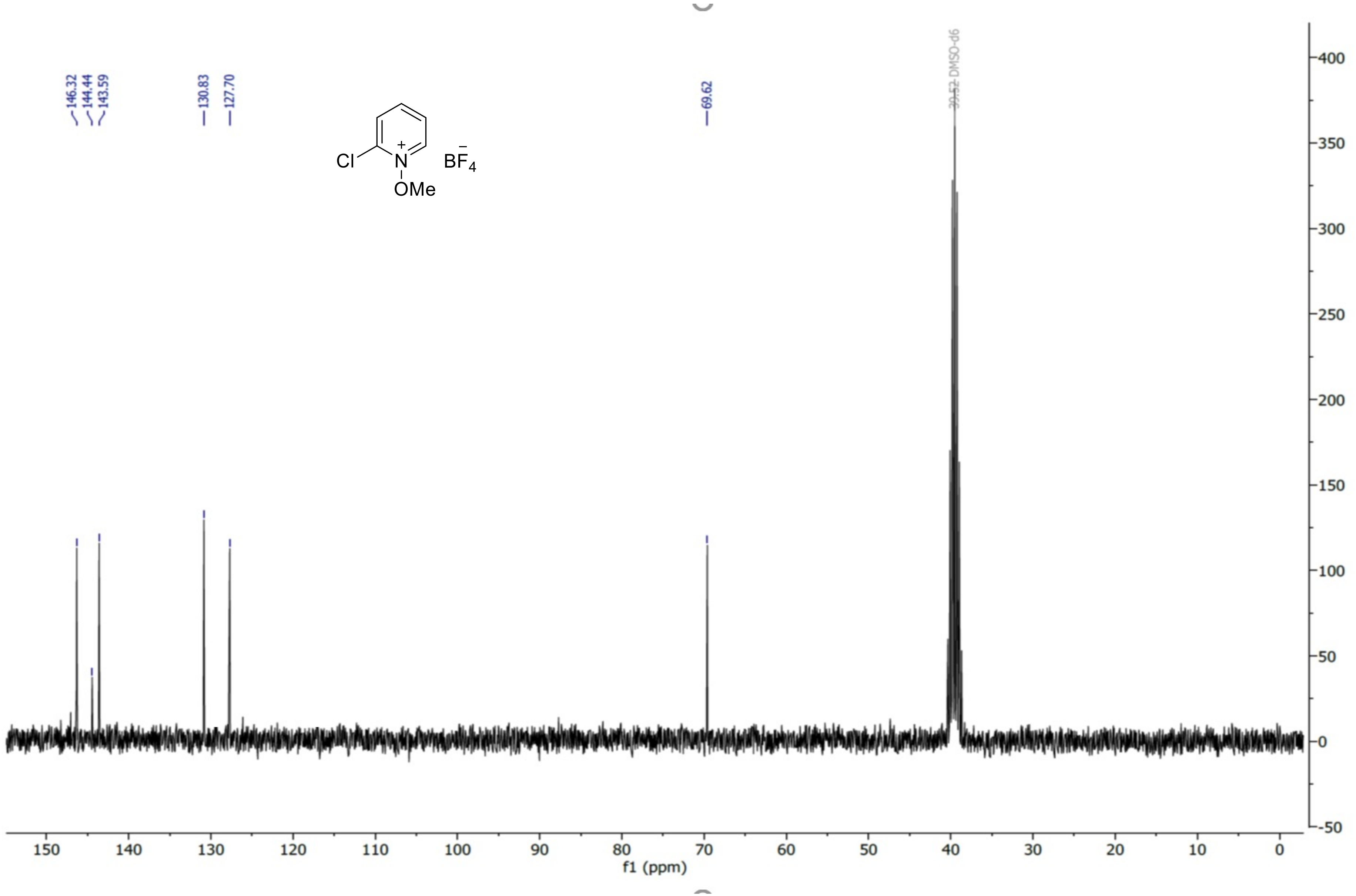

1-Methoxy-2-(trifluoromethyl)pyridin-1-ium tetrafluoroborate (1o·BF_4)

^{1}H-NMR (400 MHz, $CDCl_3$)

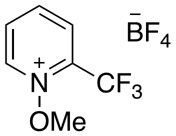

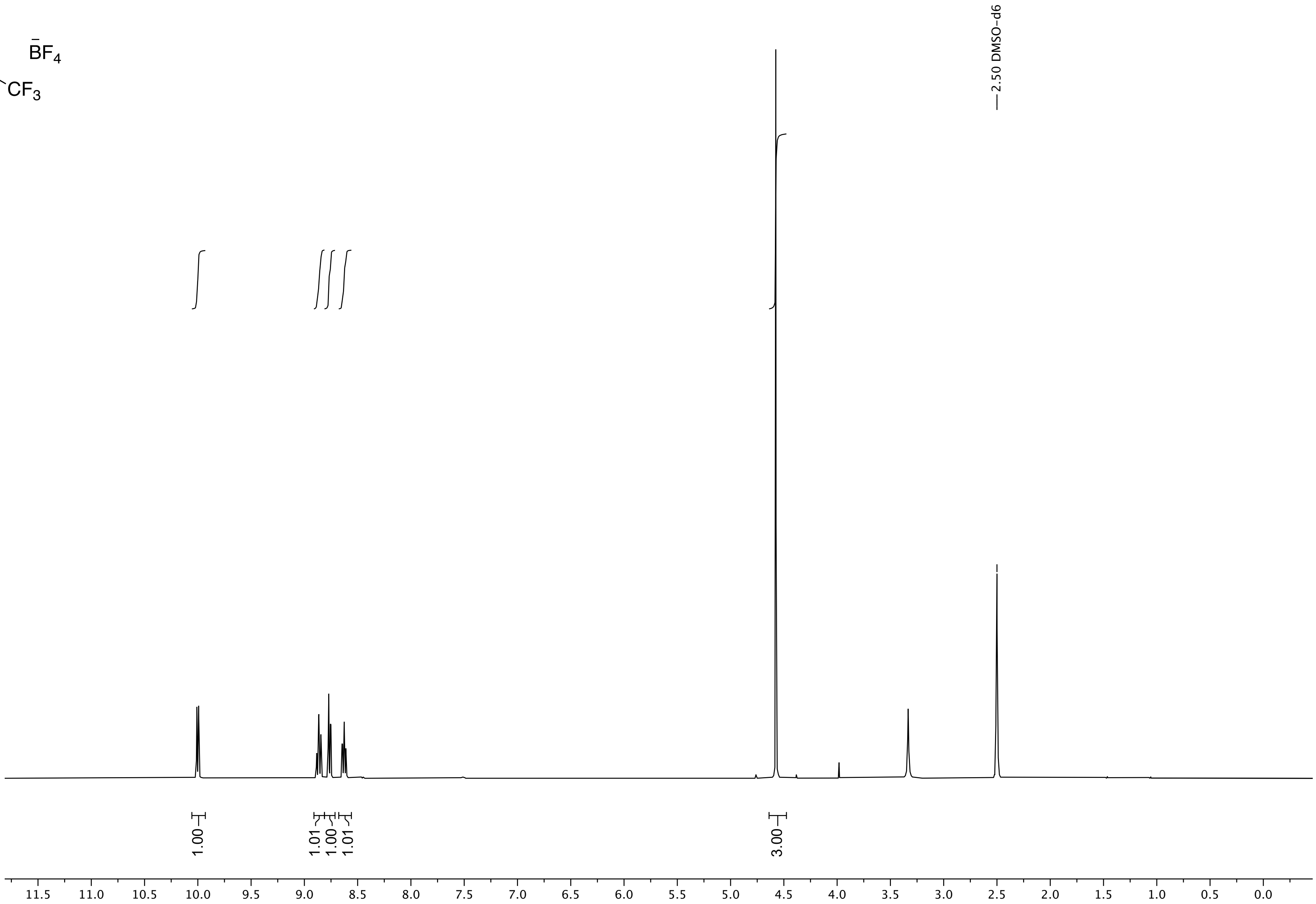

1-Methoxy-2-(trifluoromethyl)pyridin-1-ium tetrafluoroborate (1o·BF_4)

^{1}H-NMR (400 MHz, $CDCl_3$)

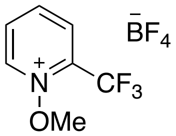

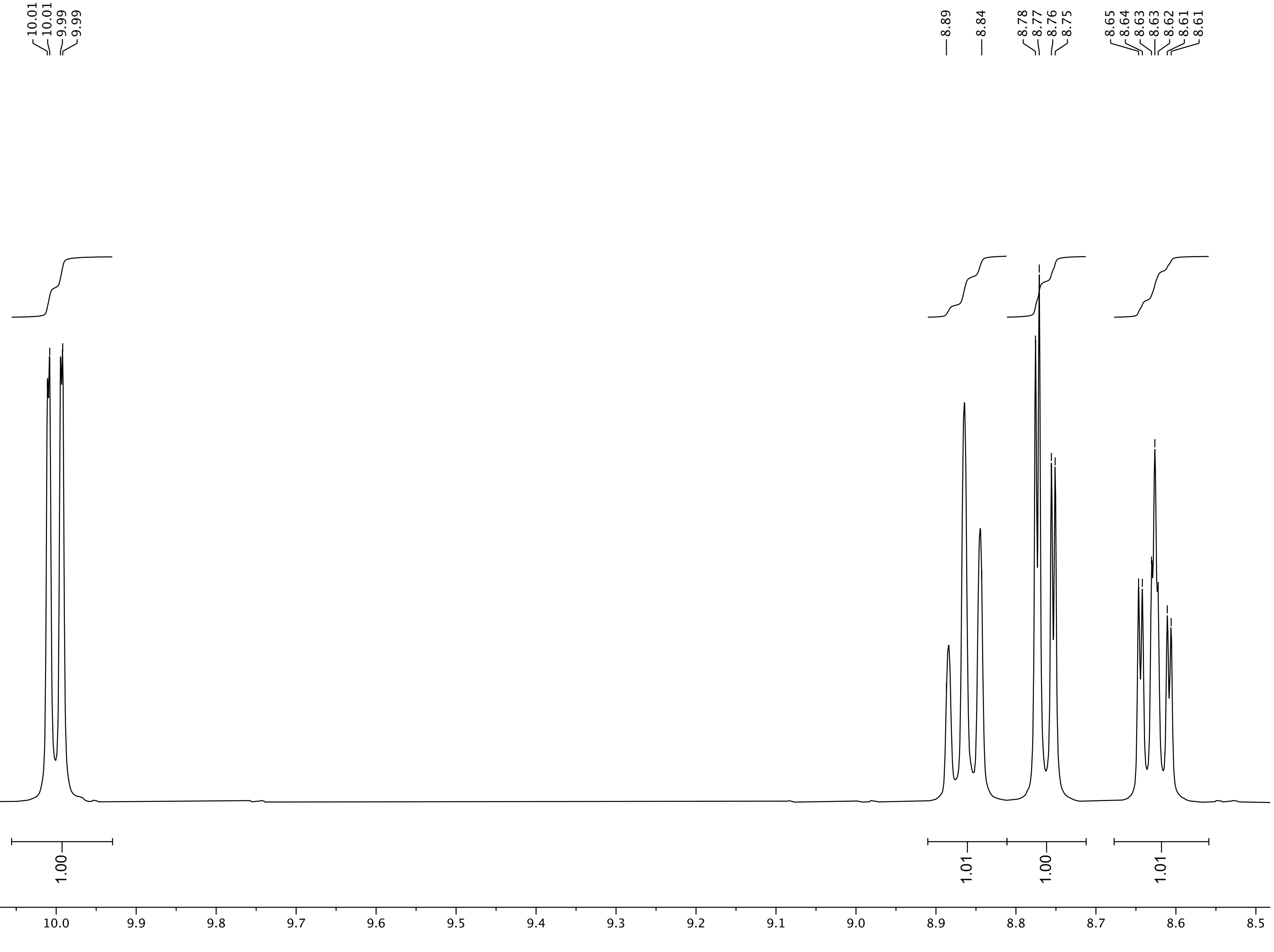

1-Methoxy-2-(trifluoromethyl)pyridin-1-ium tetrafluoroborate (1o·BF_4)

13C-NMR (101 MHz, $CDCl_3$)

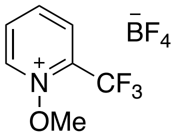

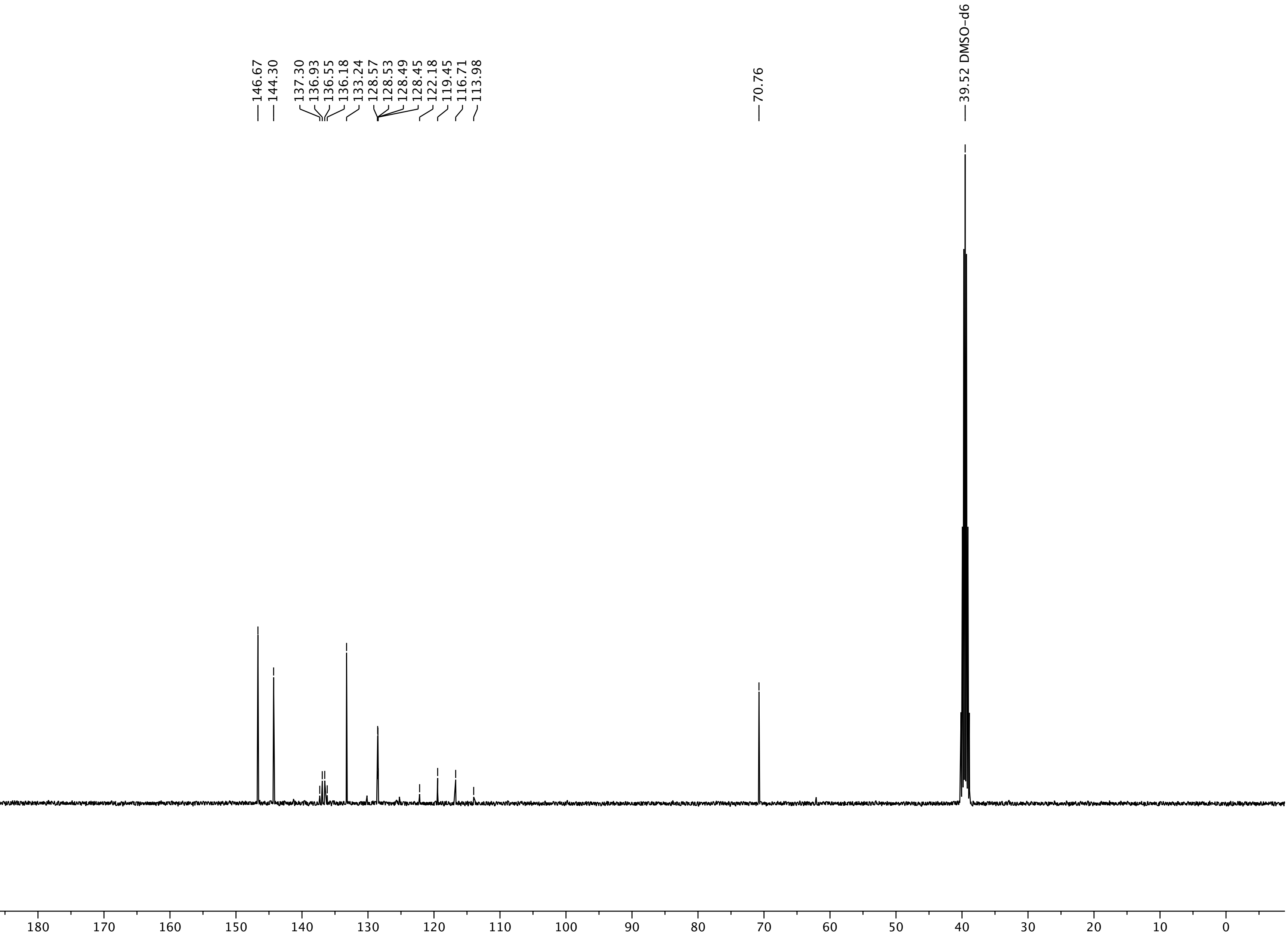

1-Methoxy-2-(trifluoromethyl)pyridin-1-ium tetrafluoroborate (1o·BF_4)

13C-NMR (101 MHz, $CDCl_3$)

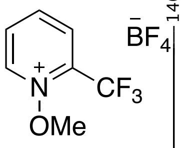

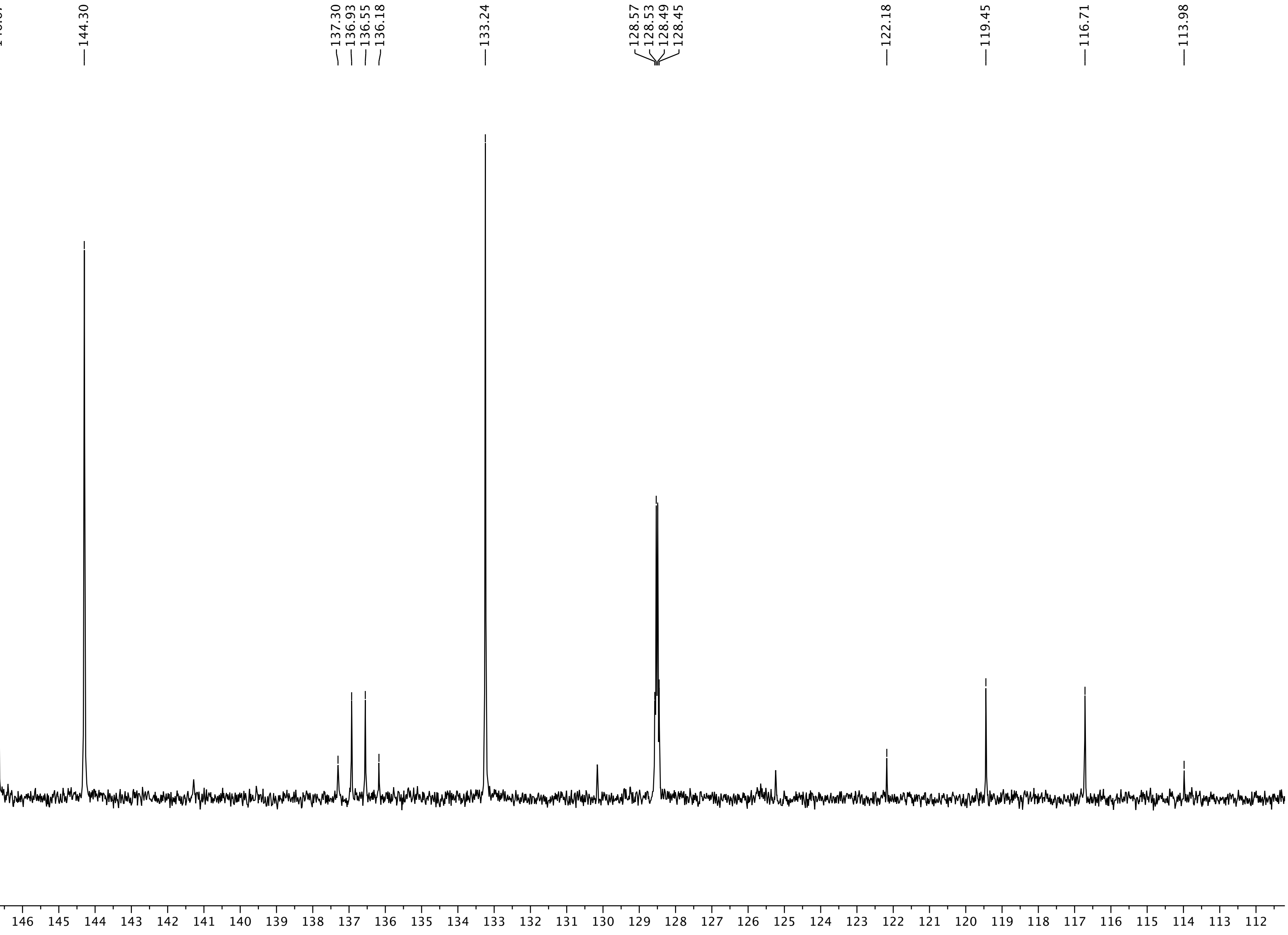

1-Methoxy-2-(trifluoromethyl)pyridin-1-ium tetrafluoroborate (1o·BF_4)

^{19}F-NMR (376 MHz, $CDCl_3$)

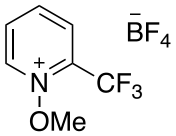

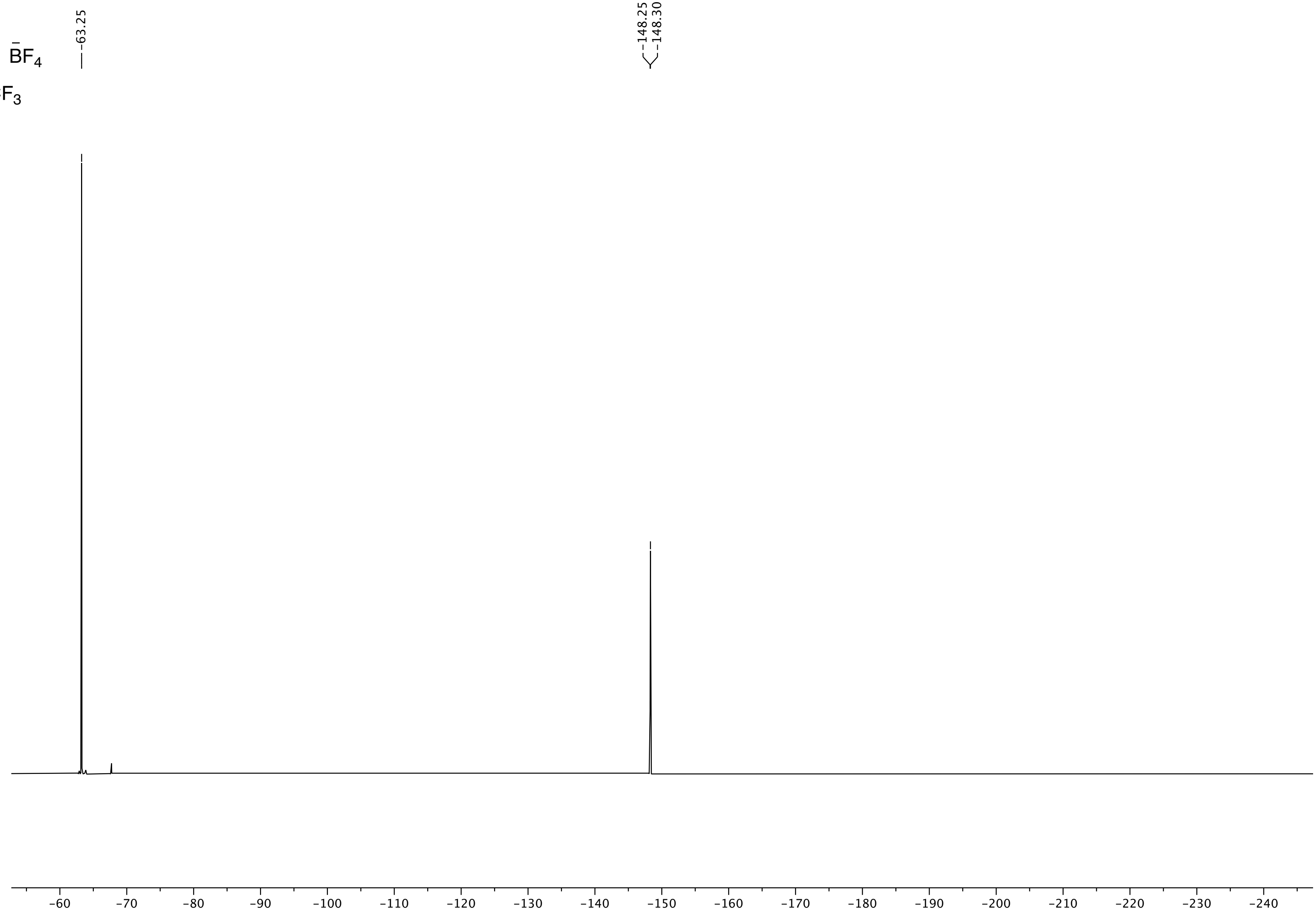

1-Methoxy-2-(trifluoromethyl)pyridin-1-ium tetrafluoroborate (1o·BF_4)

^{19}F-NMR (376 MHz, $CDCl_3$)

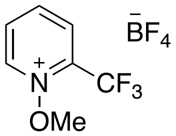

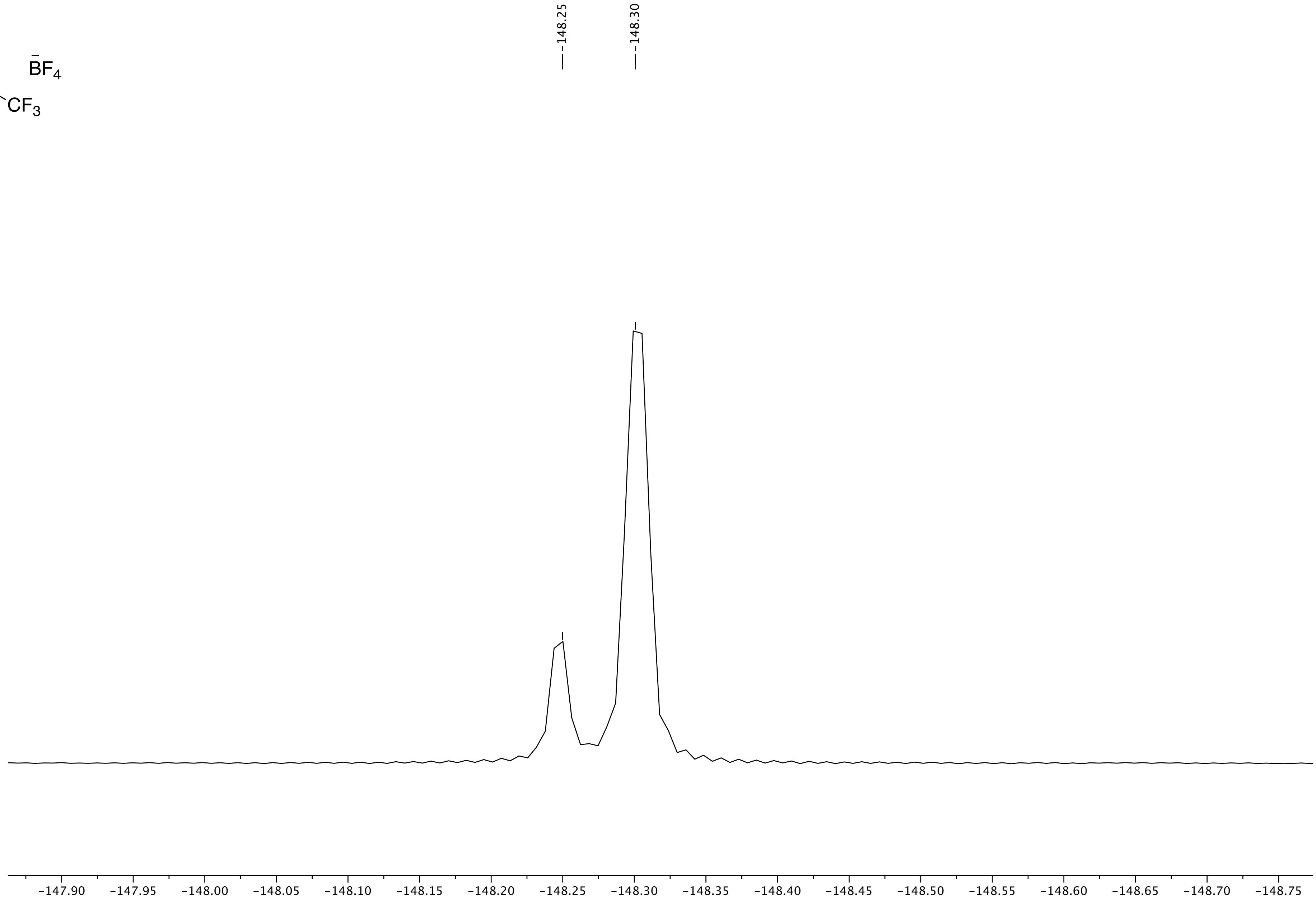

6-Fluoro-1-methoxyquinolin-1-ium tetrafluoroborate (1p·BF_4)

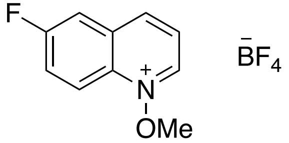

^{1}H-NMR (400 MHz, $CDCl_3$)

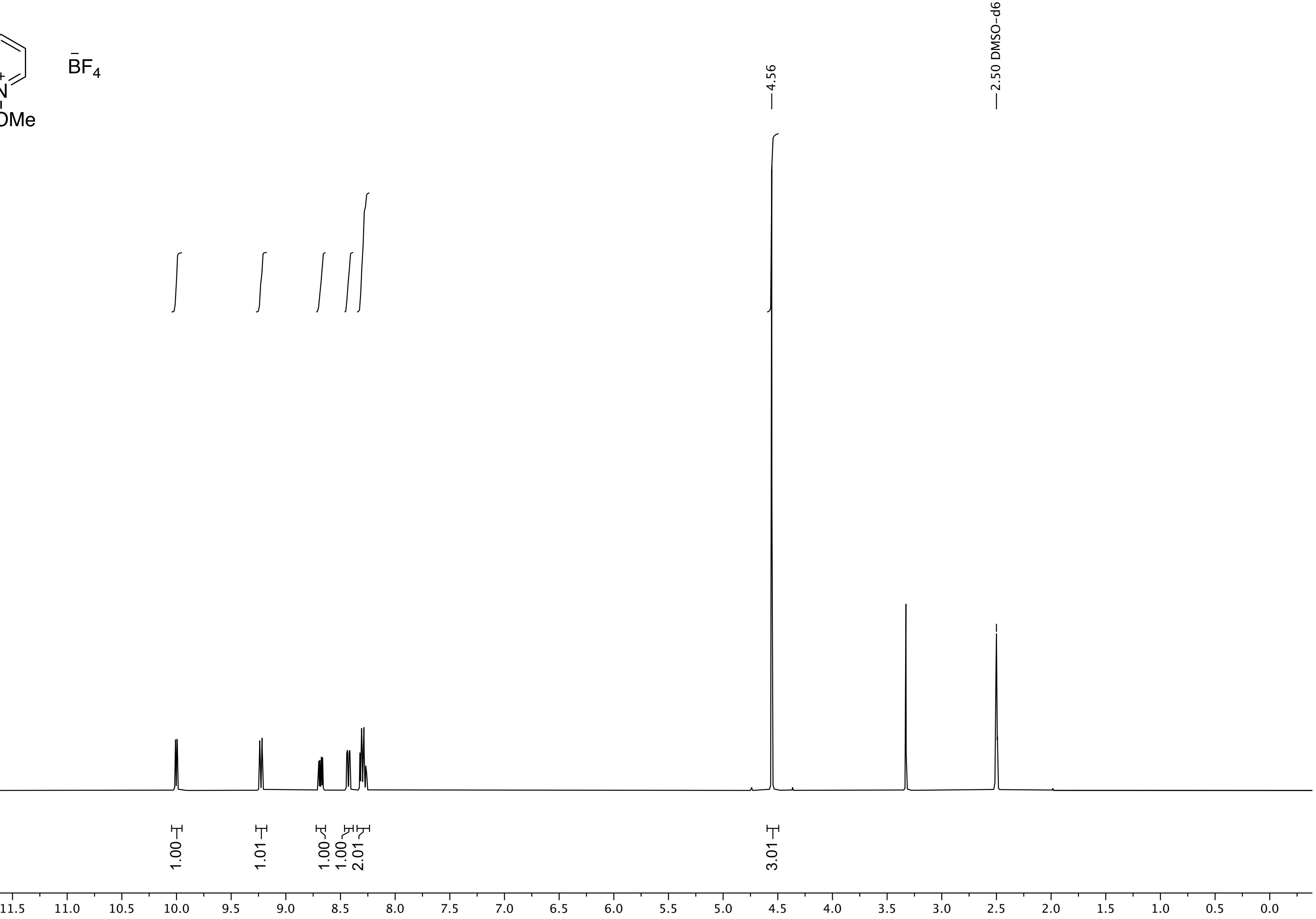

6-Fluoro-1-methoxyquinolin-1-ium tetrafluoroborate (1p·BF_4)

^{1}H-NMR (400 MHz, $CDCl_3$)

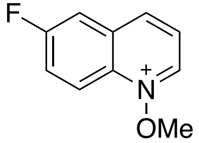

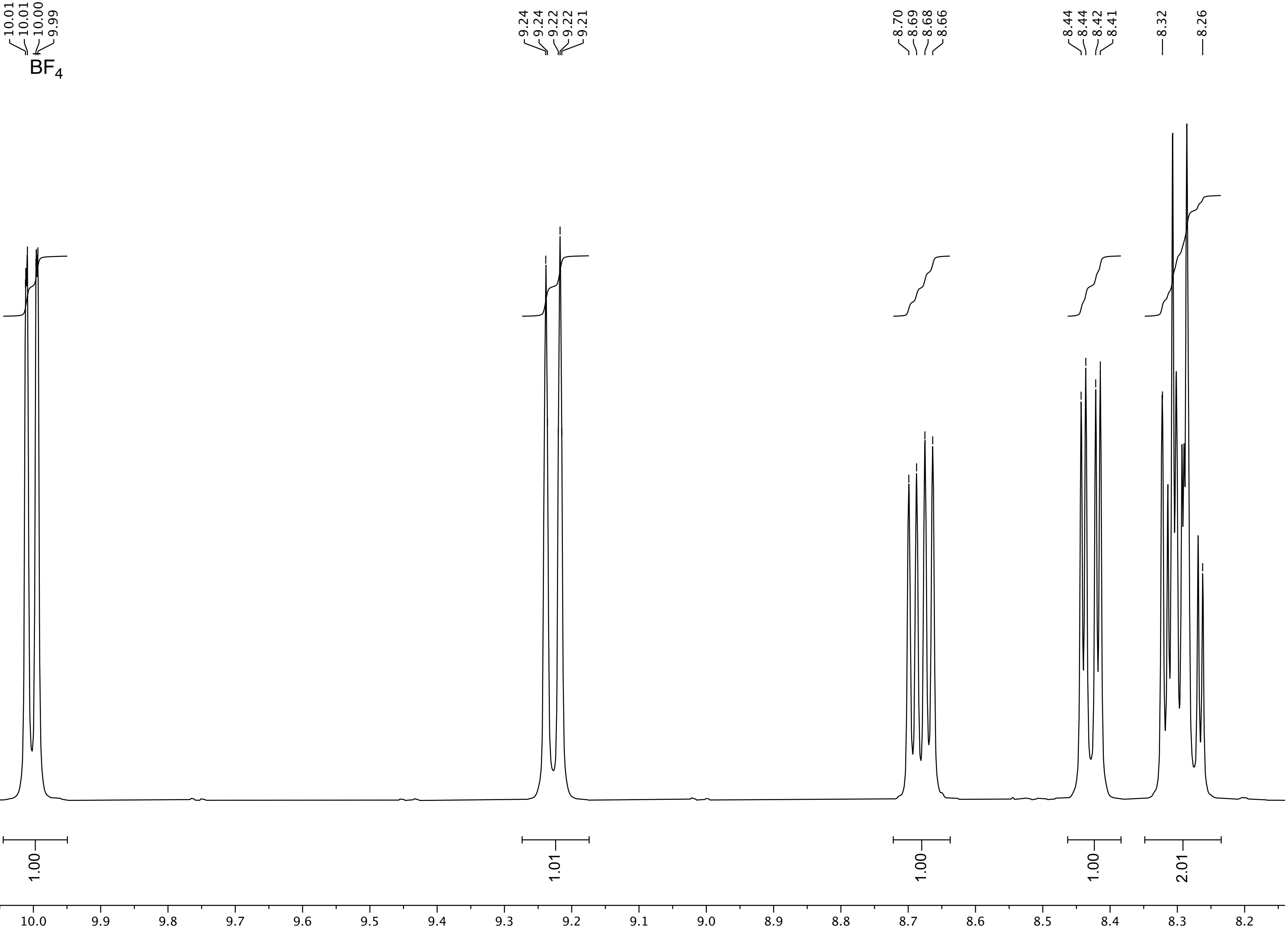

6-Fluoro-1-methoxyquinolin-1-ium tetrafluoroborate (1p·BF_4)

^{13}C-NMR (101 MHz, $CDCl_3$)

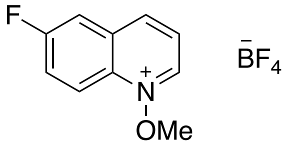

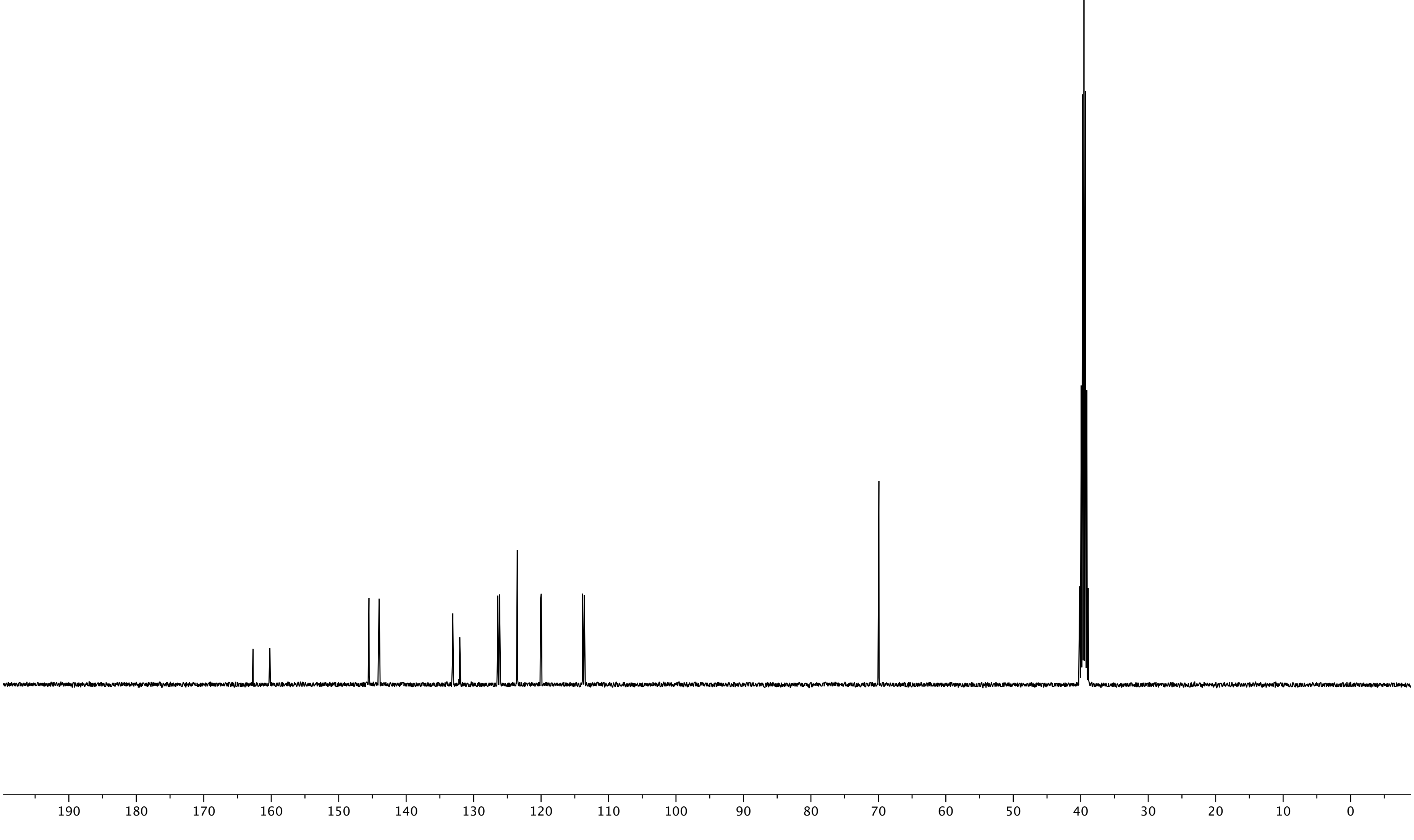

6-Fluoro-1-methoxyquinolin-1-ium tetrafluoroborate (1p·BF_4)

^{13}C-NMR (101 MHz, $CDCl_3$)

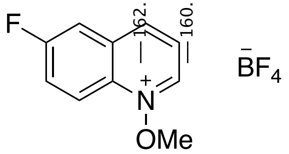

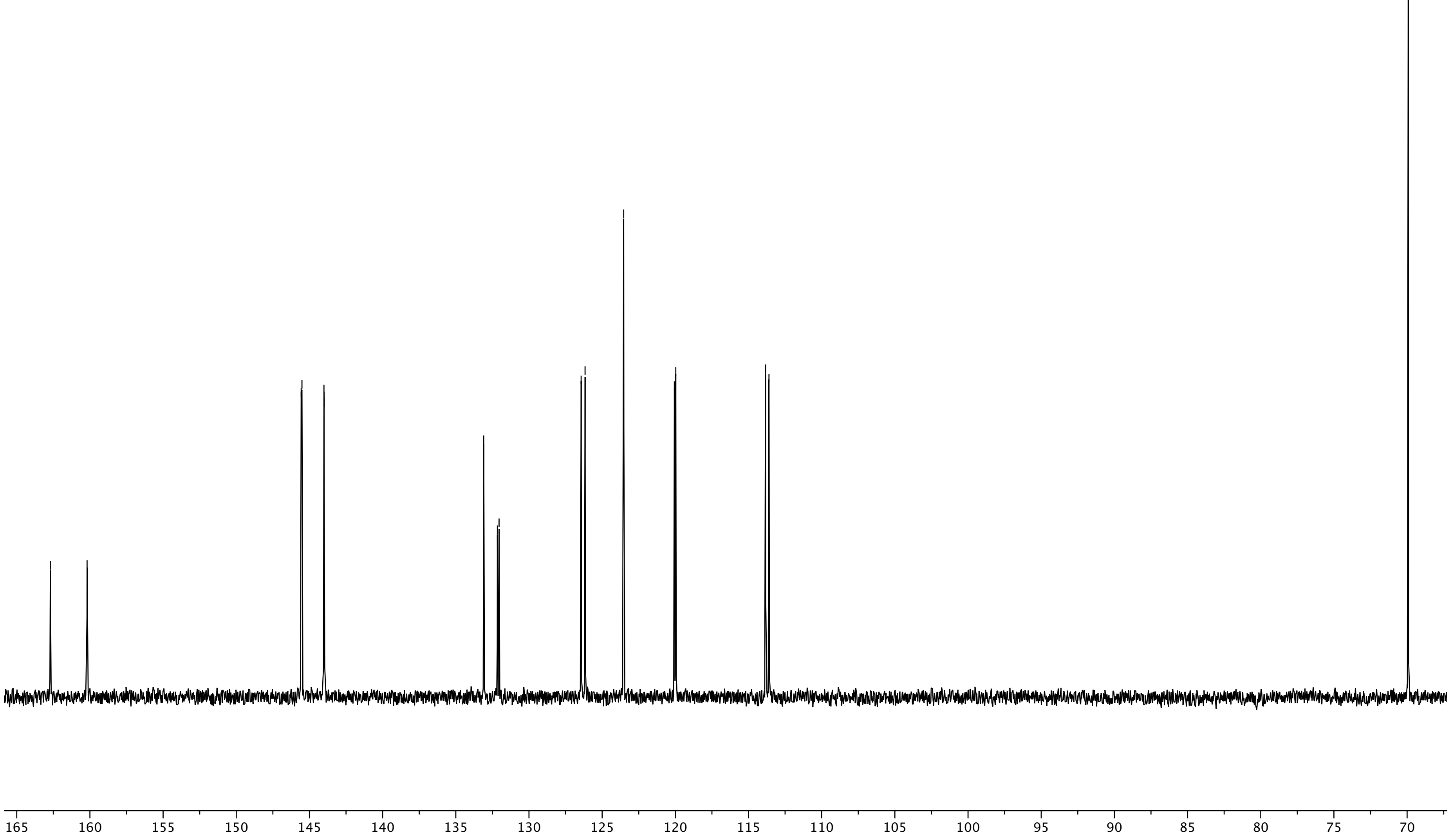

6-Fluoro-1-methoxyquinolin-1-ium tetrafluoroborate (1p·BF_4)

Dept-135 (101 MHz, $CDCl_3$)

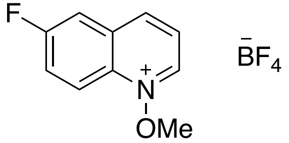

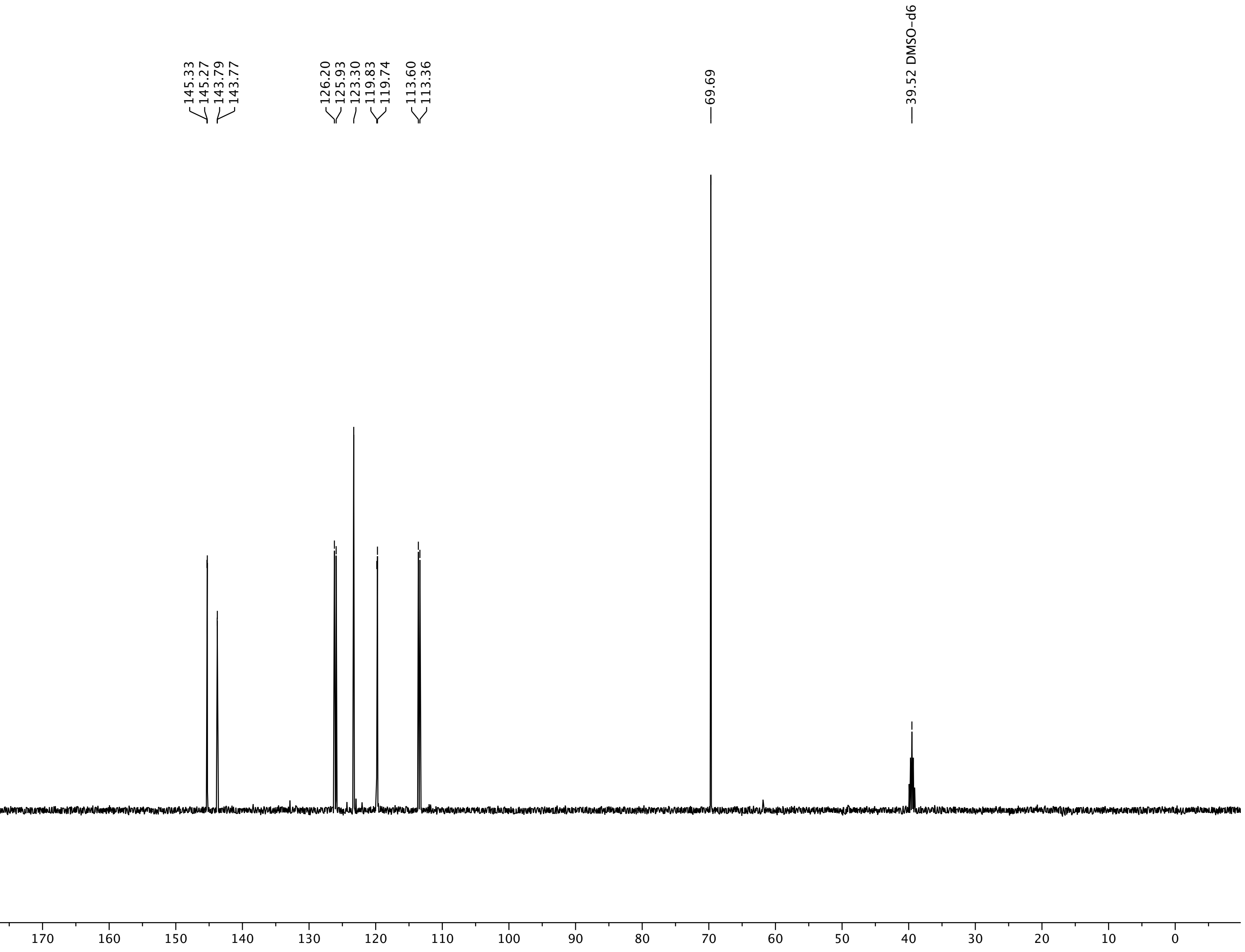

6-Fluoro-1-methoxyquinolin-1-ium tetrafluoroborate (1p·BF_4)

HSQC (400 MHz, $CDCl_3$)

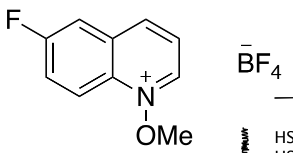

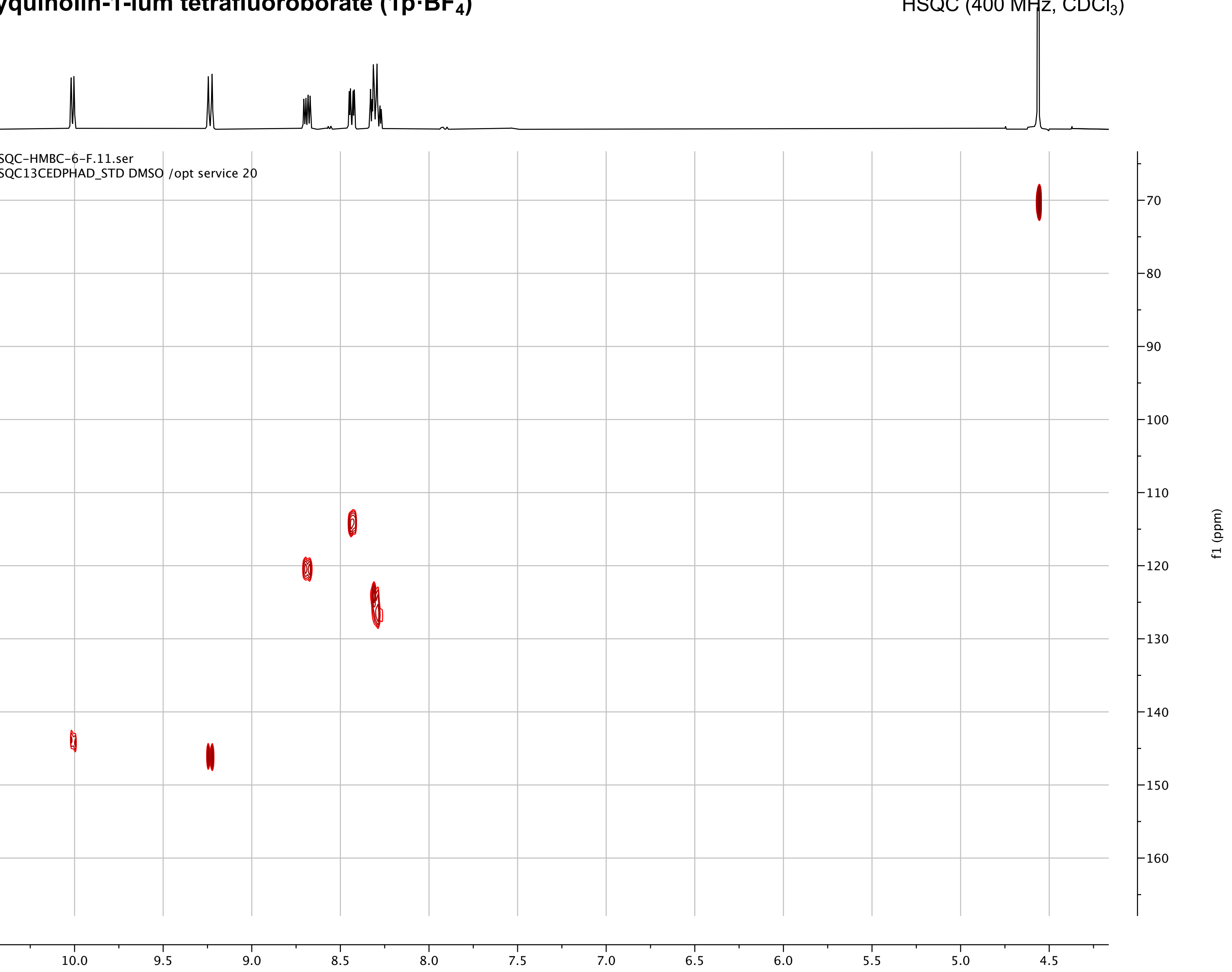

6-Fluoro-1-methoxyquinolin-1-ium tetrafluoroborate (1p·BF_4)

HBMC (400 MHz, $CDCl_3$)

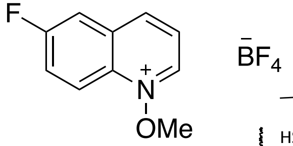

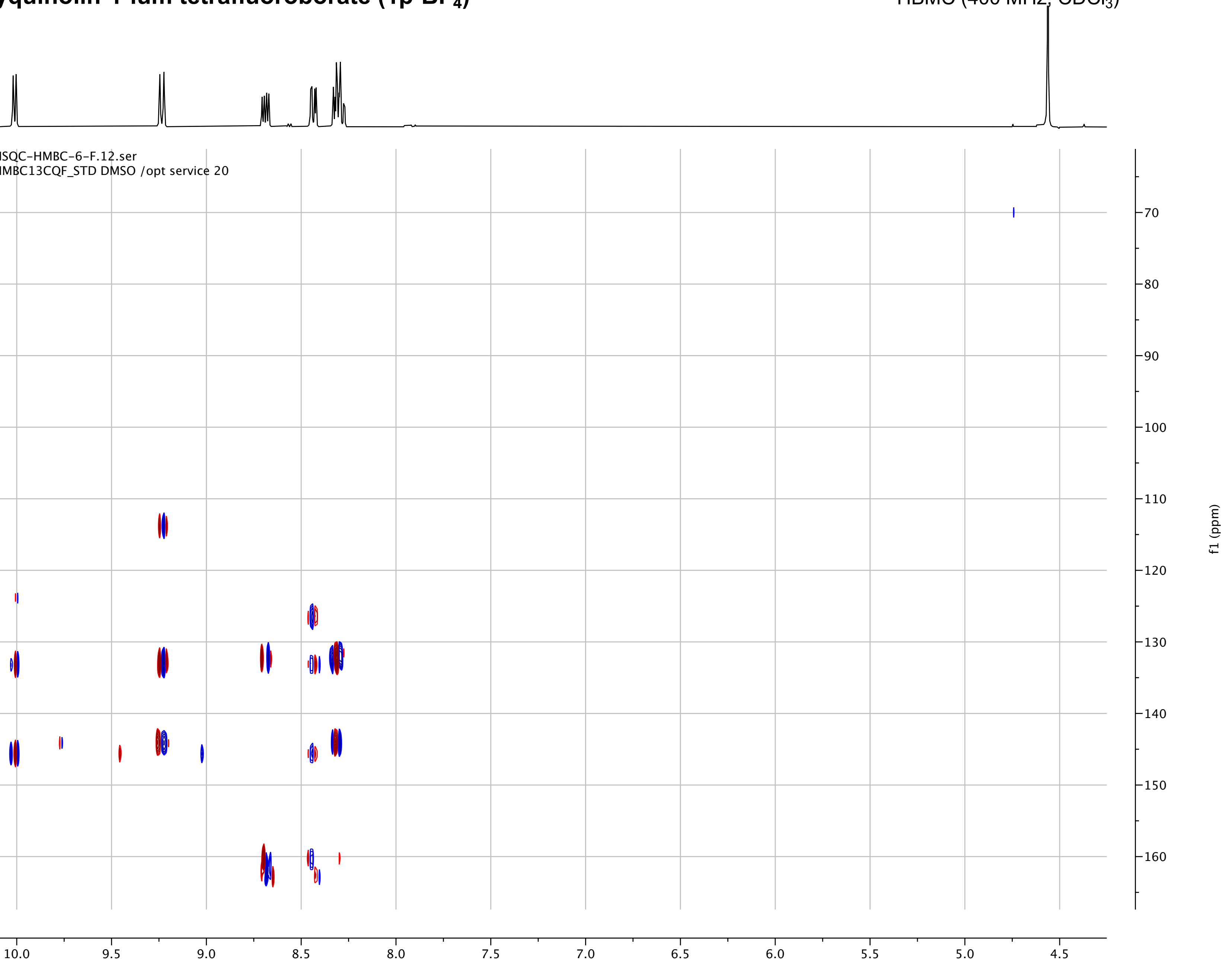

6-Fluoro-1-methoxyquinolin-1-ium tetrafluoroborate (1p·BF_4)

HBMC (400 MHz, $CDCl_3$)

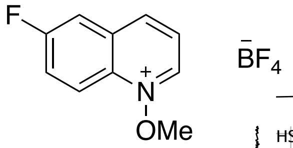

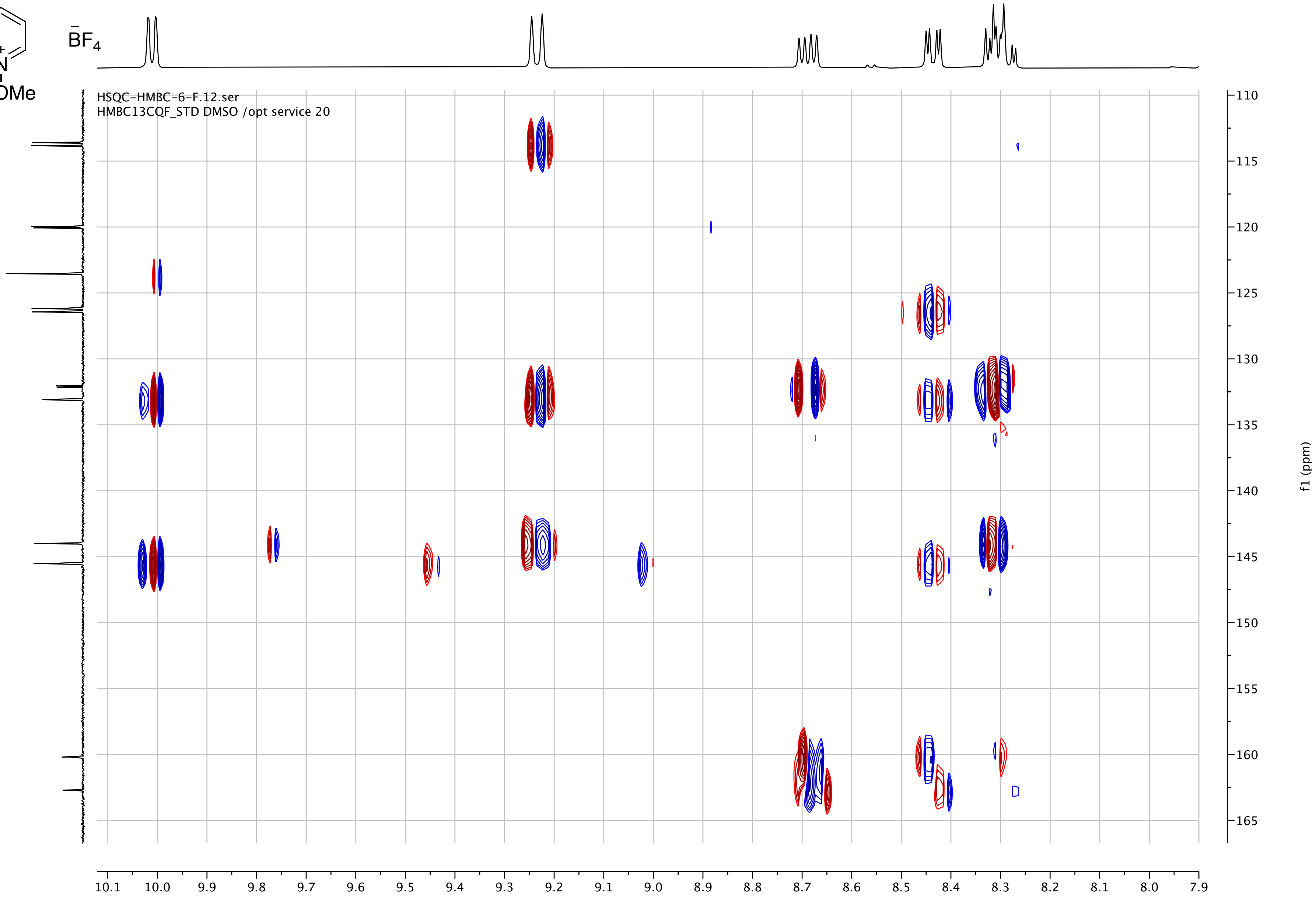

6-Fluoro-1-methoxyquinolin-1-ium tetrafluoroborate (1p·BF_4)

^{19}F-NMR (376 MHz, $CDCl_3$)

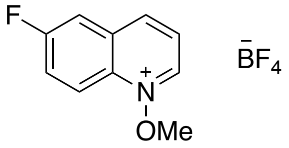

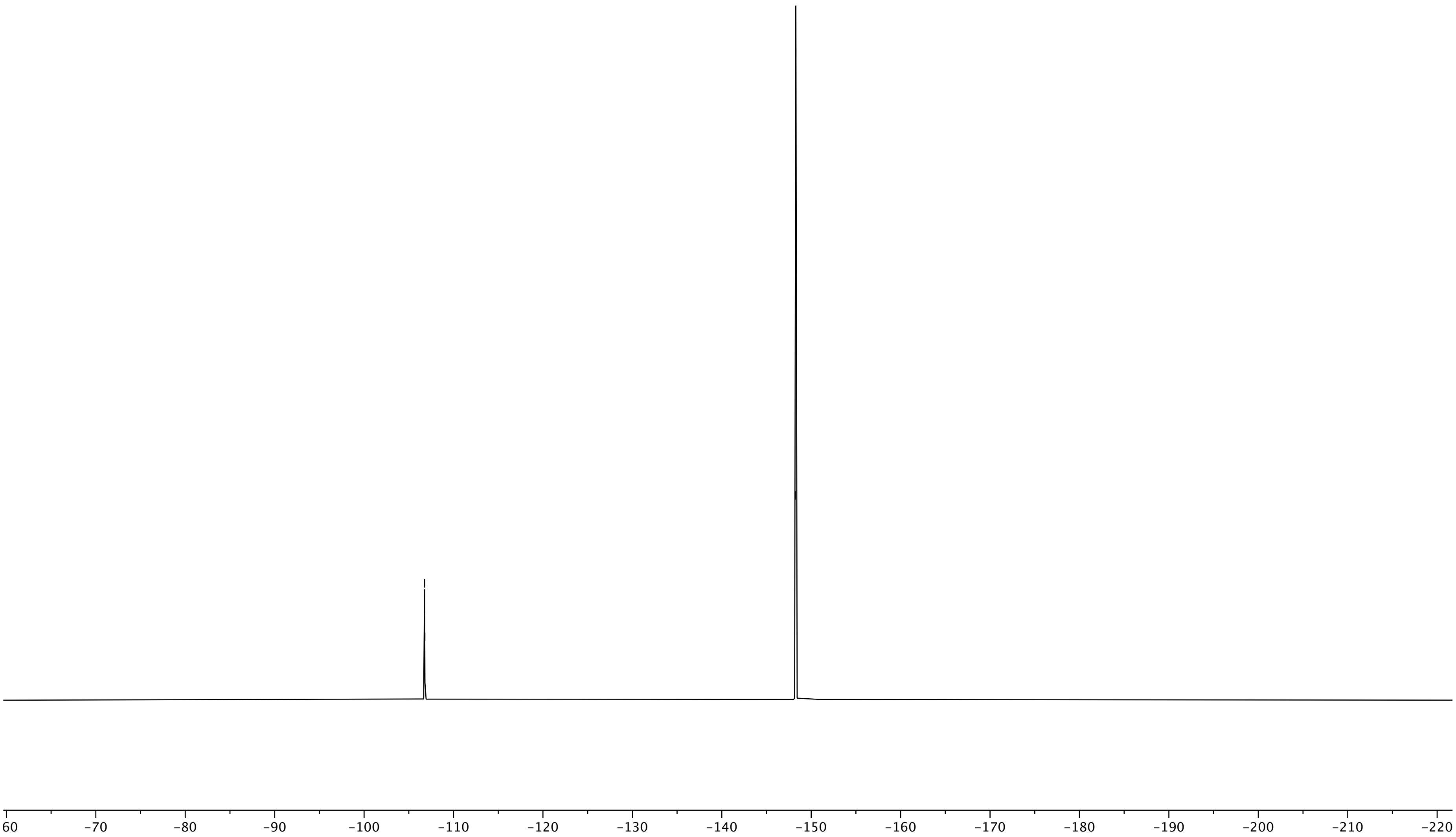

6-Fluoro-1-methoxyquinolin-1-ium tetrafluoroborate (1p·BF_4)

^{19}F-NMR (376 MHz, $CDCl_3$)

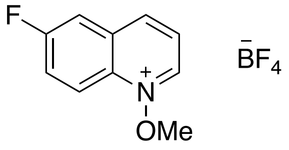

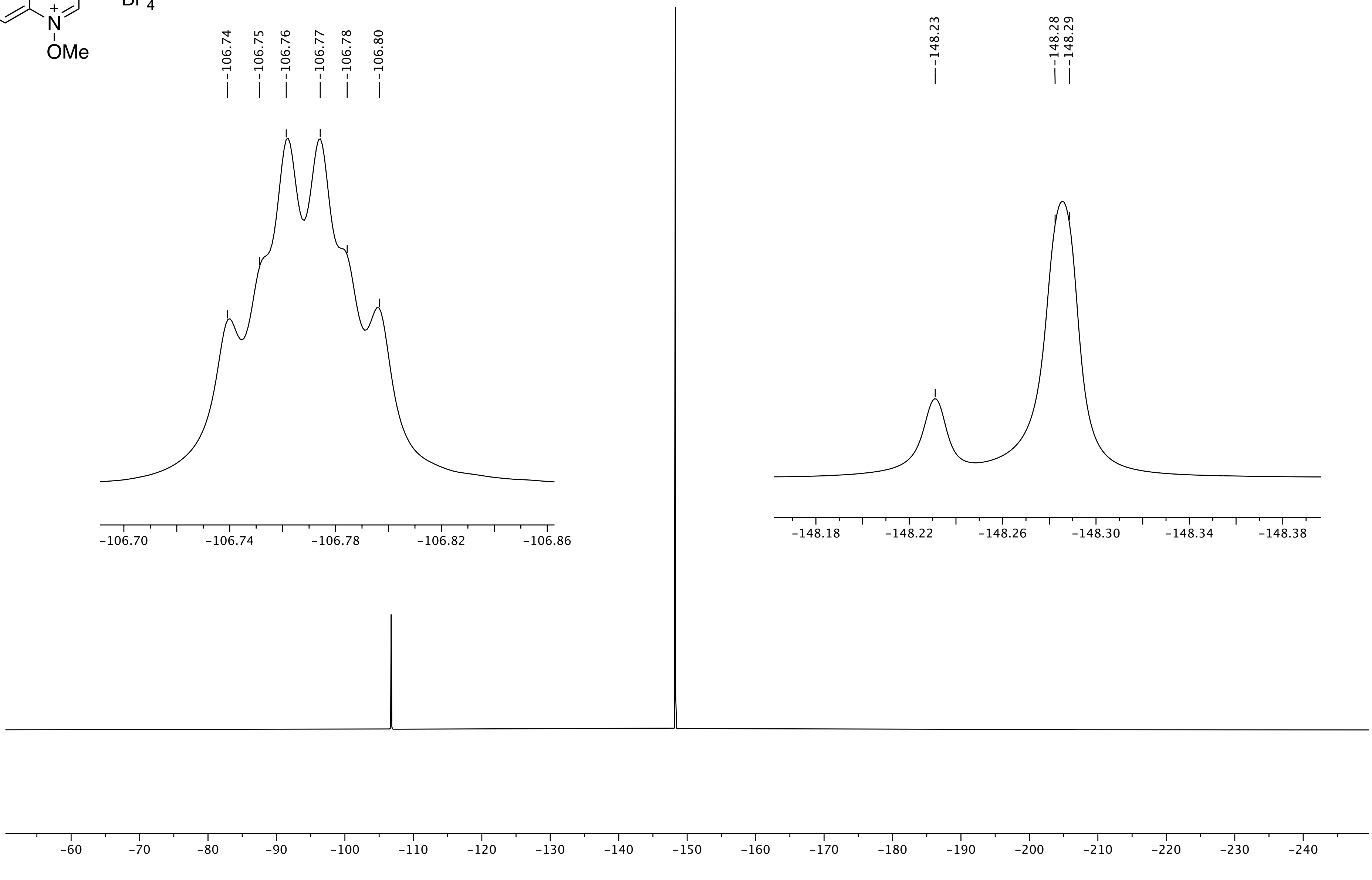

2-Methoxyisoquinolin-2-ium tetrafluoroborate (1q·BF_4)

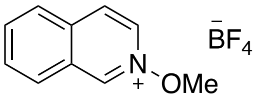

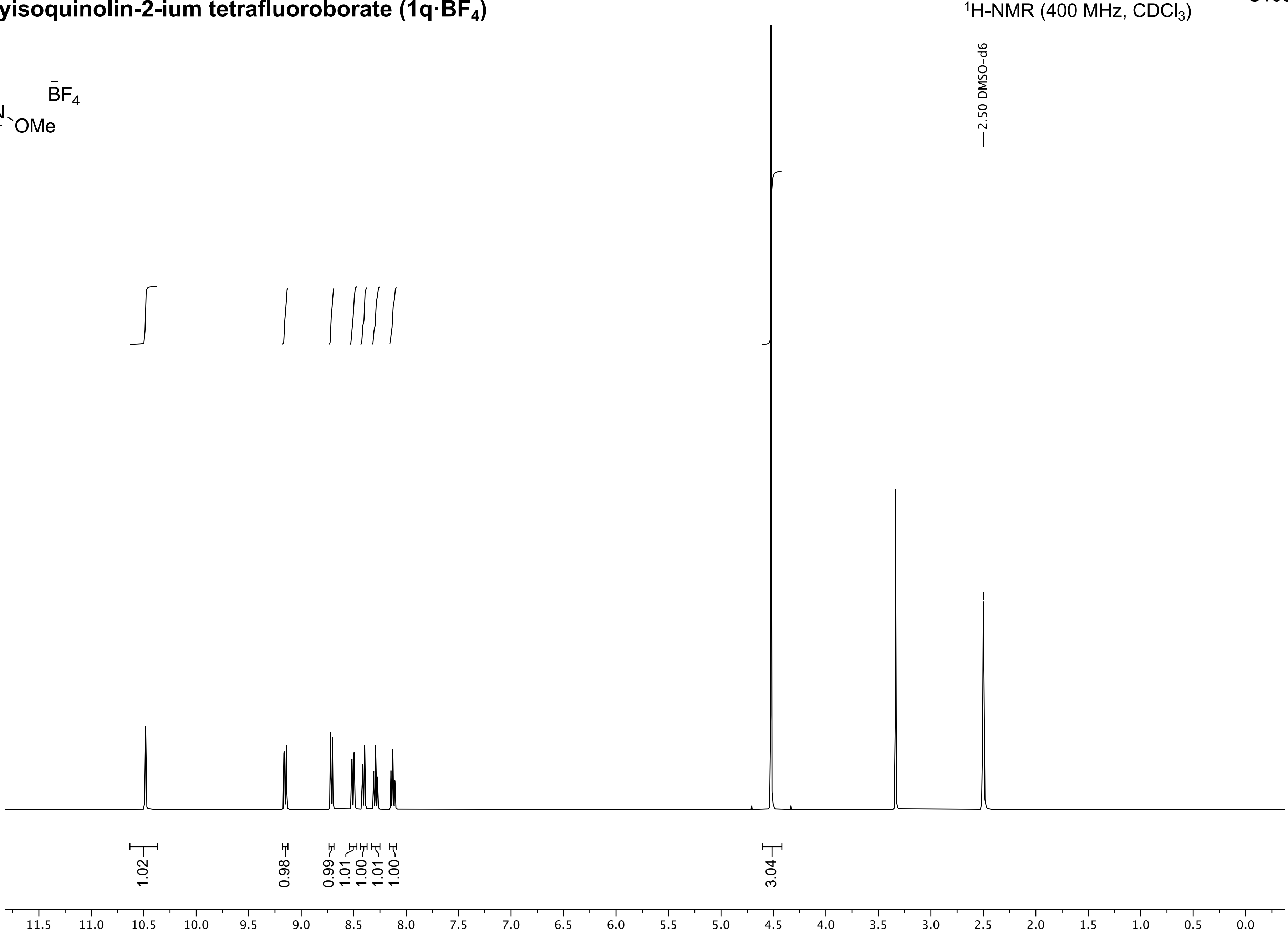

2-Methoxyisoquinolin-2-ium tetrafluoroborate (1q·BF_4)

^{1}H-NMR (400 MHz, $CDCl_3$)

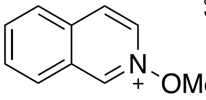

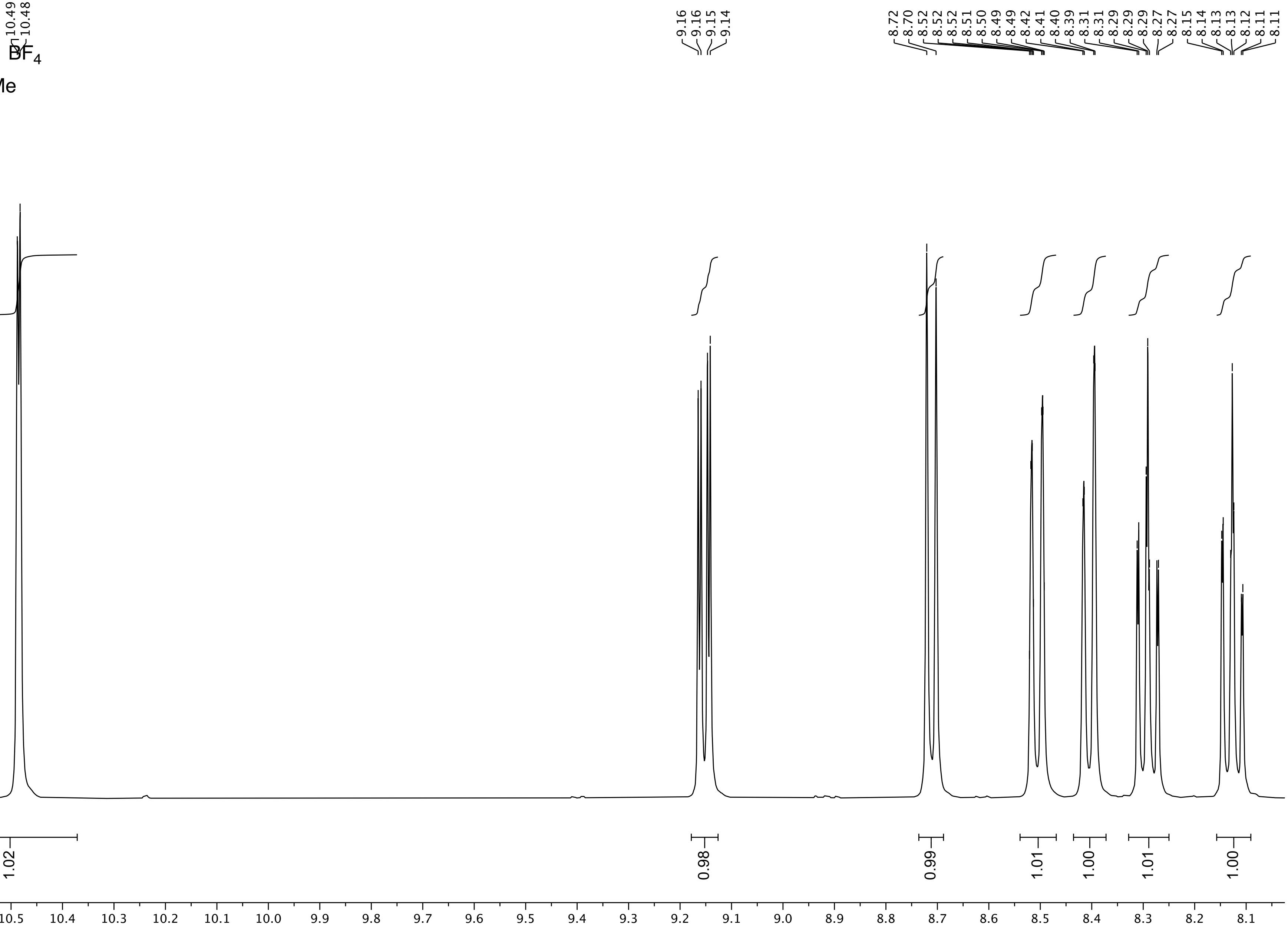

2-Methoxyisoquinolin-2-ium tetrafluoroborate (1q·BF_4)

^{13}C-NMR (101 MHz, $CDCl_3$)

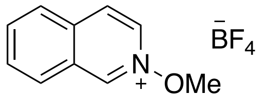

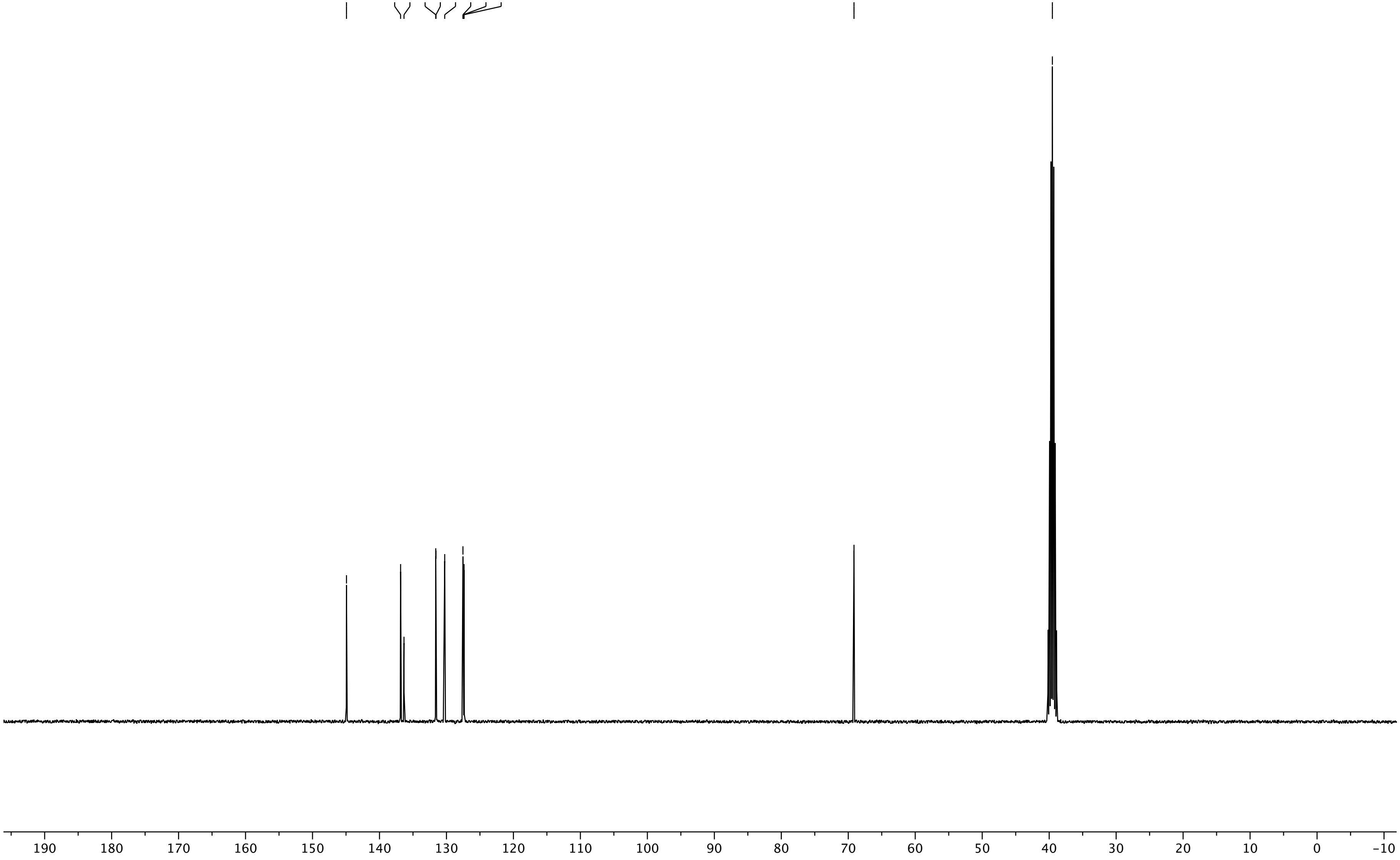

2-Methoxyisoquinolin-2-ium tetrafluoroborate (1q·BF_4)

^{13}C-NMR (101 MHz, $CDCl_3$)

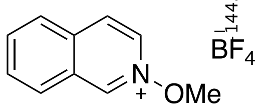

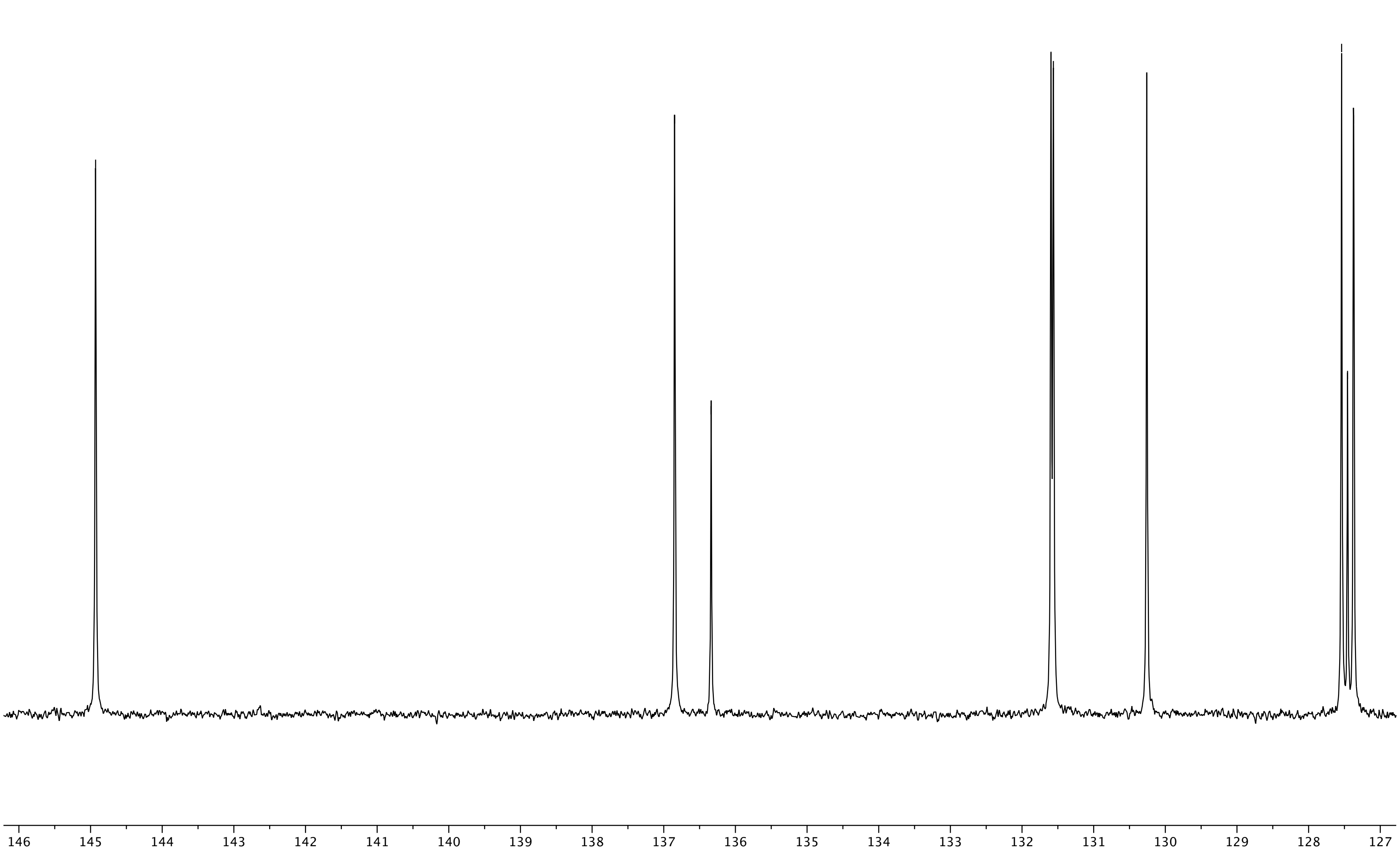

2-Cyclohexyl-4-phenylpyridine (2)

^{1}H-NMR, 300 MHz, $CDCl_3$

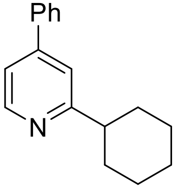

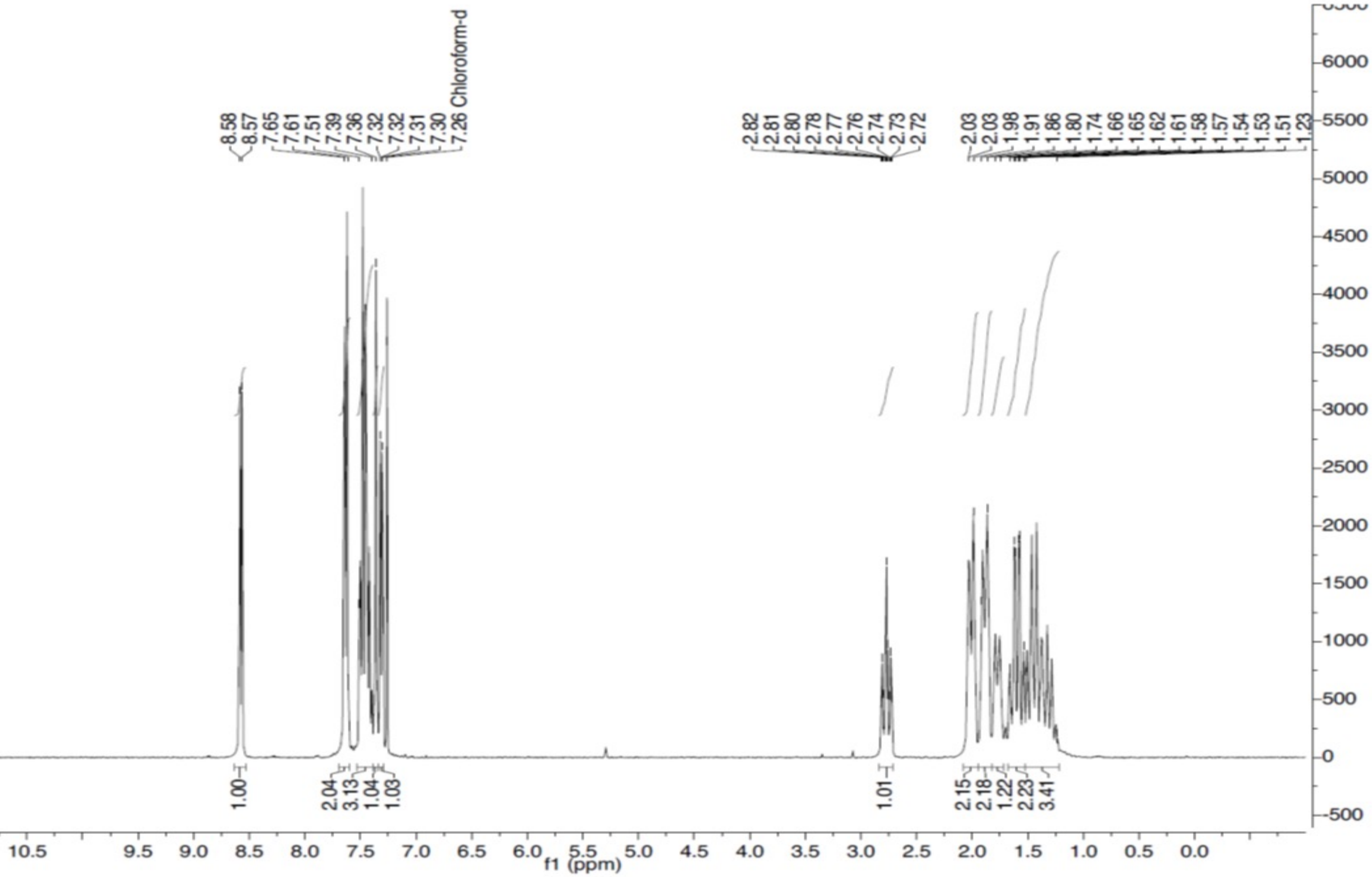

2-Cyclohexyl-4-phenylpyridine (2)

^{13}C-NMR, 75MHz, $CDCl_3$

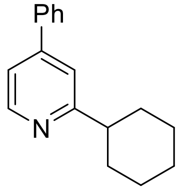

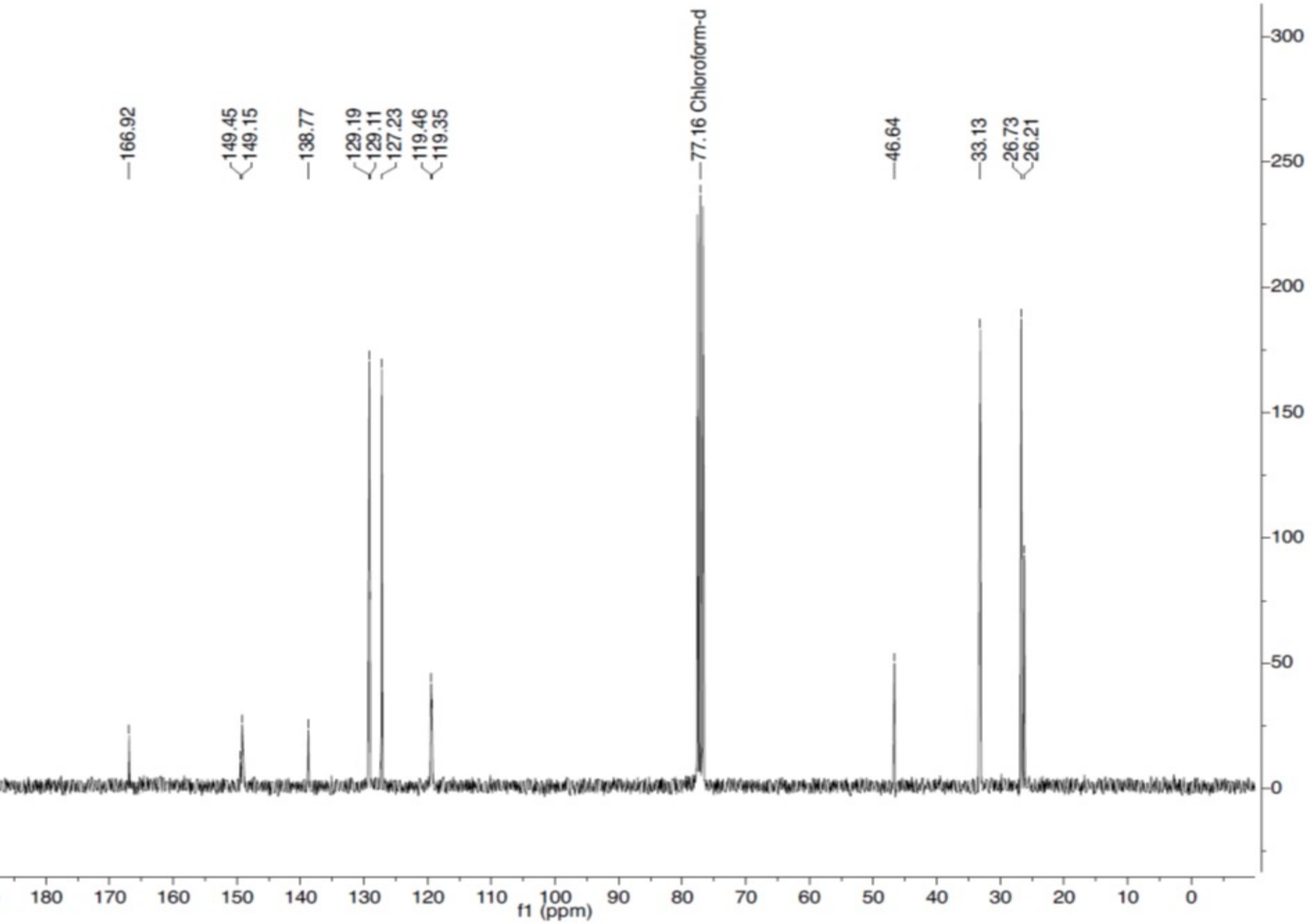

2-Cyclohexyl-4-methylquinoline (3)

^{1}H-NMR, 300 MHz, $CDCl_3$

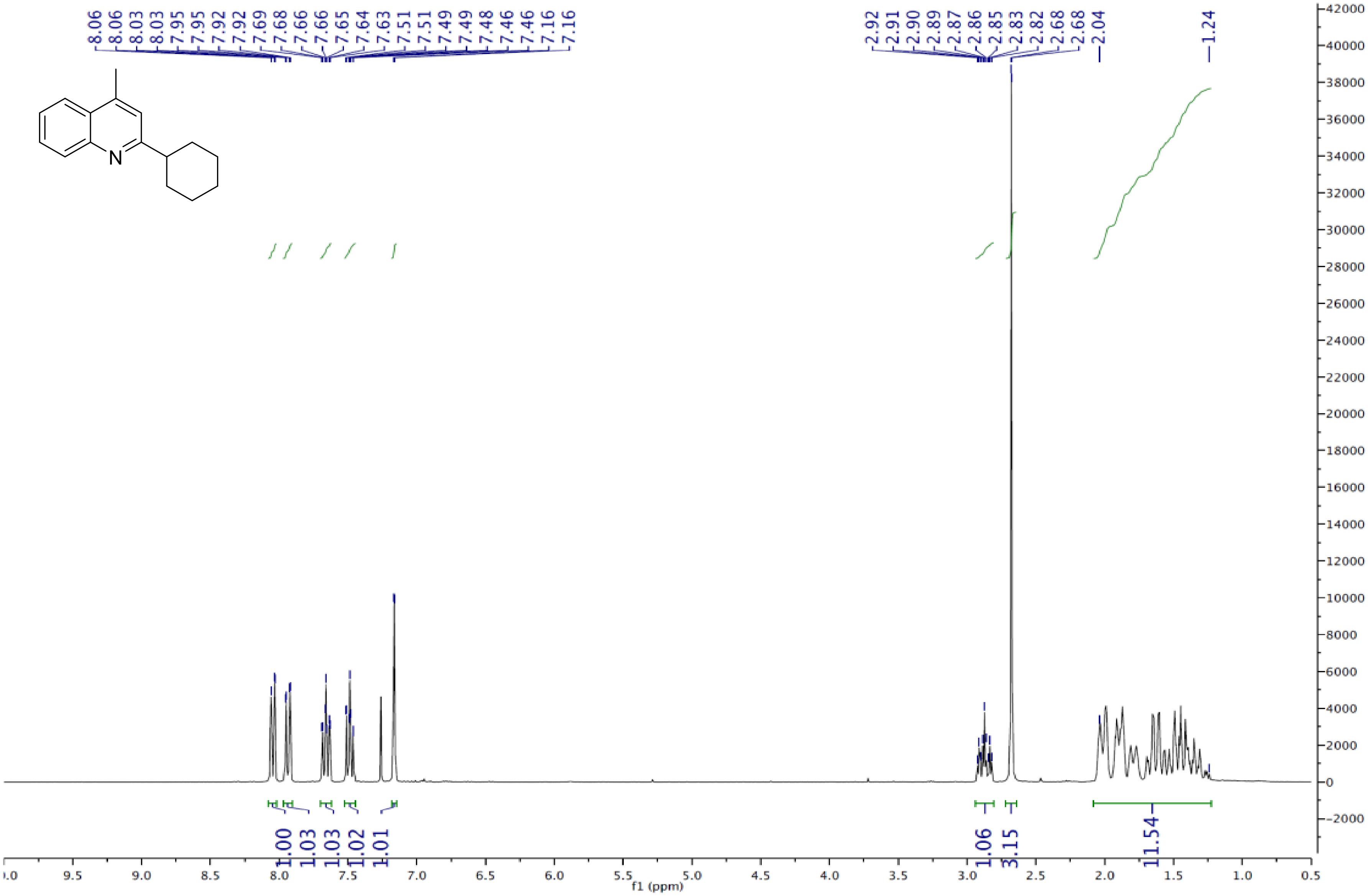

2-Cyclohexyl-4-methylquinoline (3)

^{13}C-NMR, 75MHz, $CDCl_3$

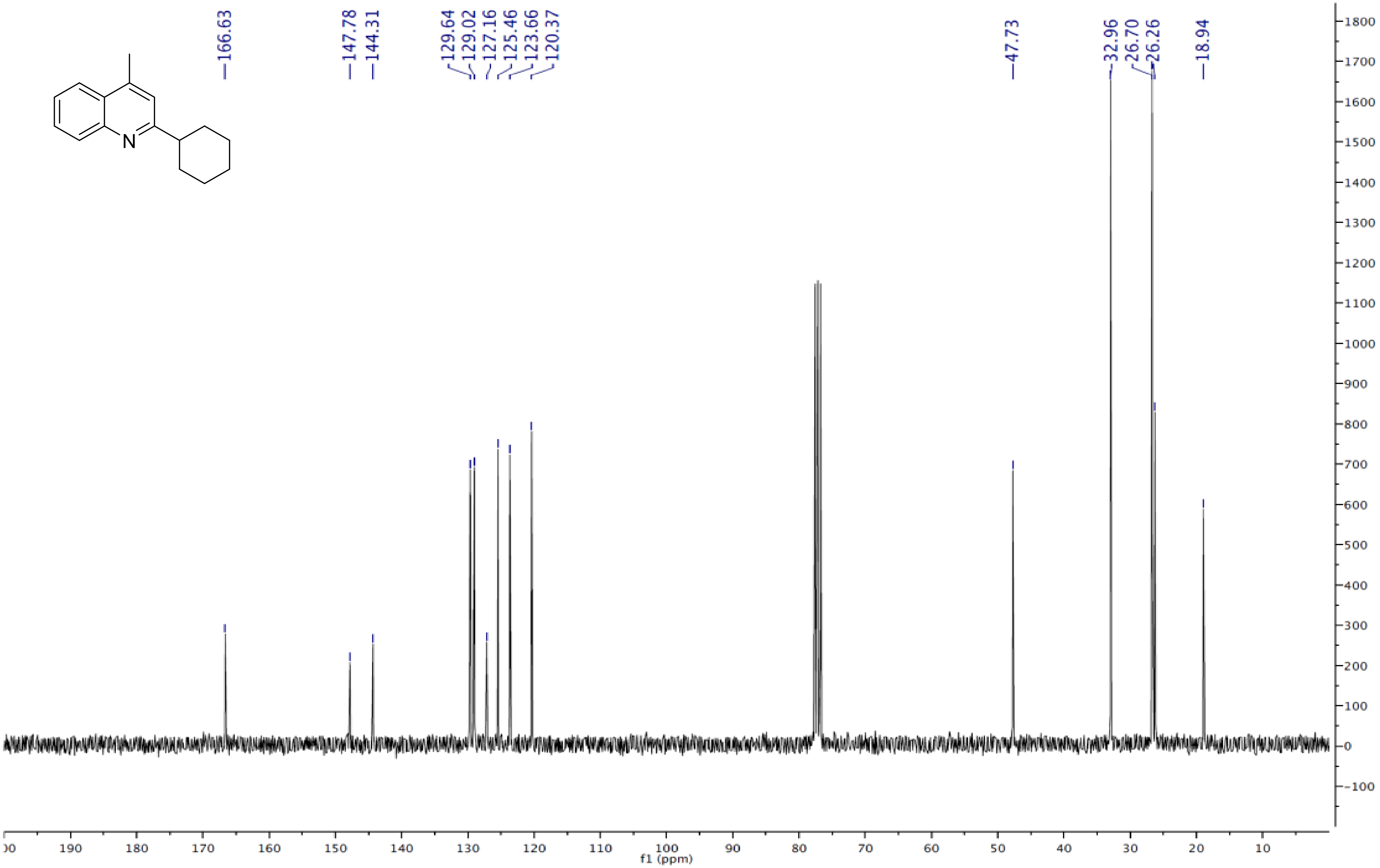

2-Hexyl-4-methylquinoline (5)

^{1}H-NMR, 300 MHz, $CDCl_3$

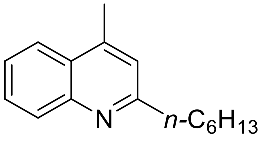

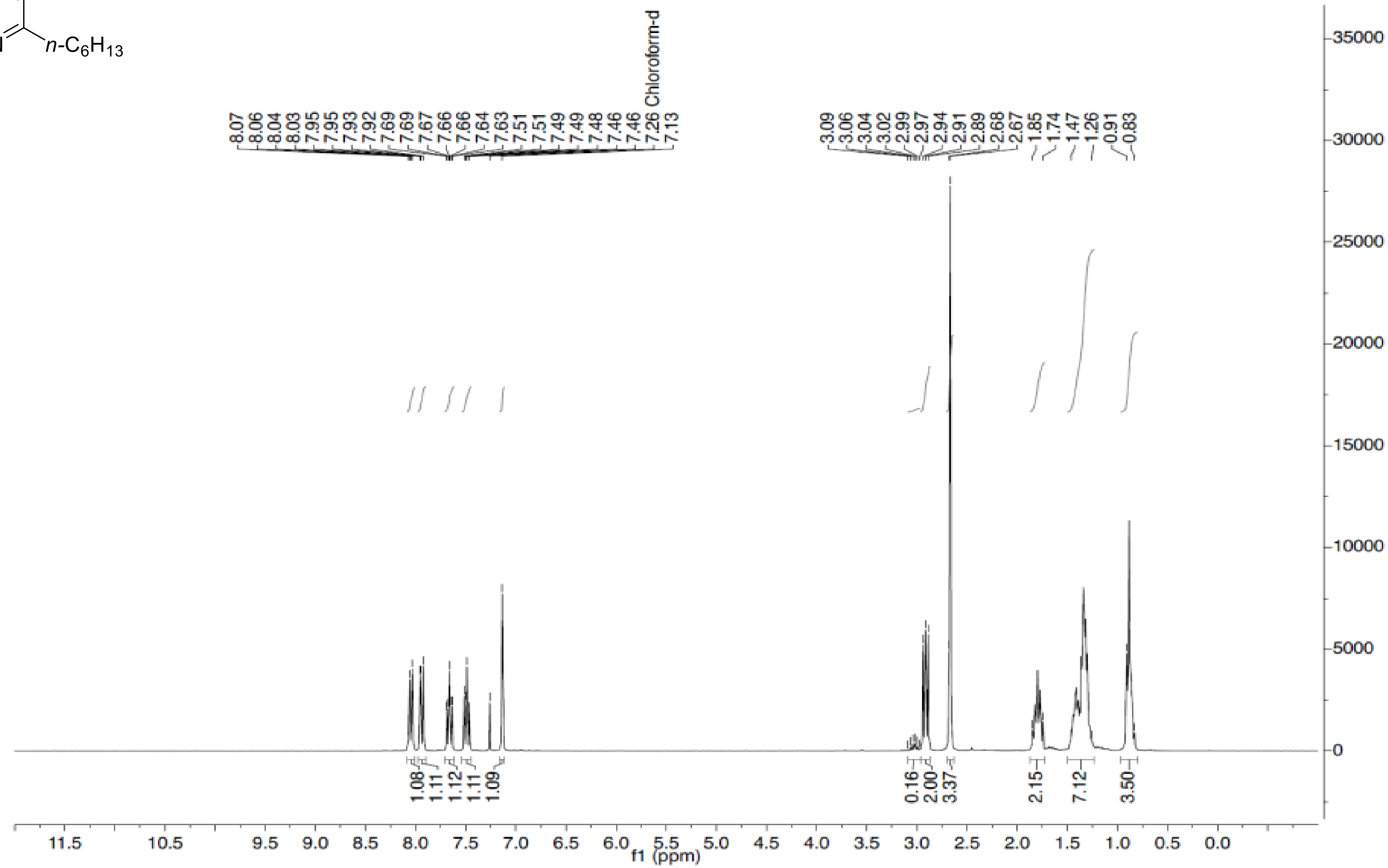

2-Hexyl-4-methylquinoline (5)

^{13}C-NMR, 75 MHz, $CDCl_3$

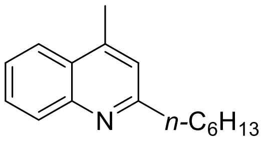

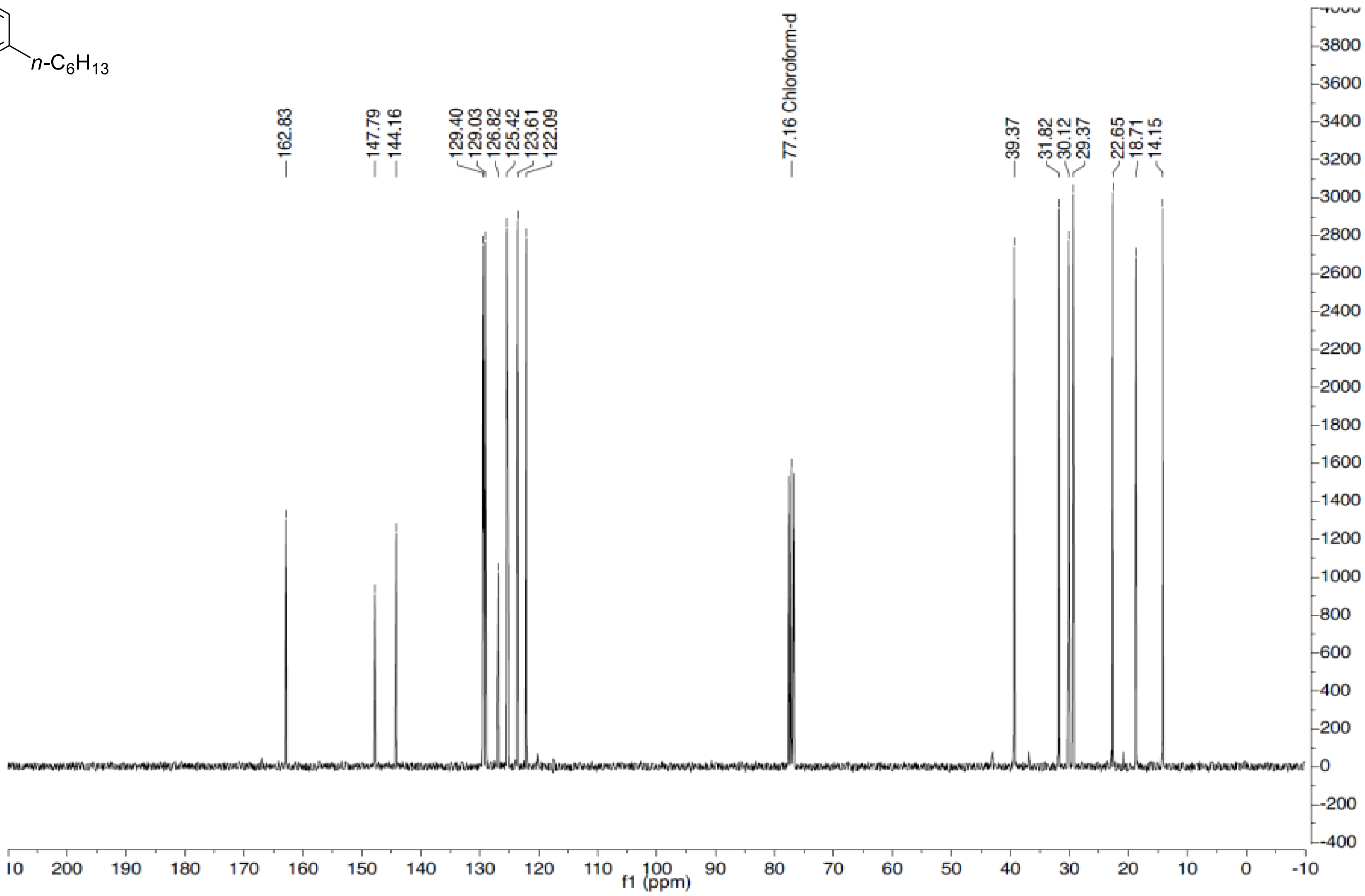

2-Decyl-4-methylquinoline (6)

^{1}H-NMR, 300 MHz, $CDCl_3$

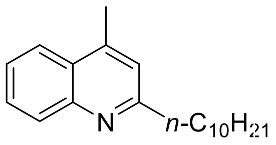

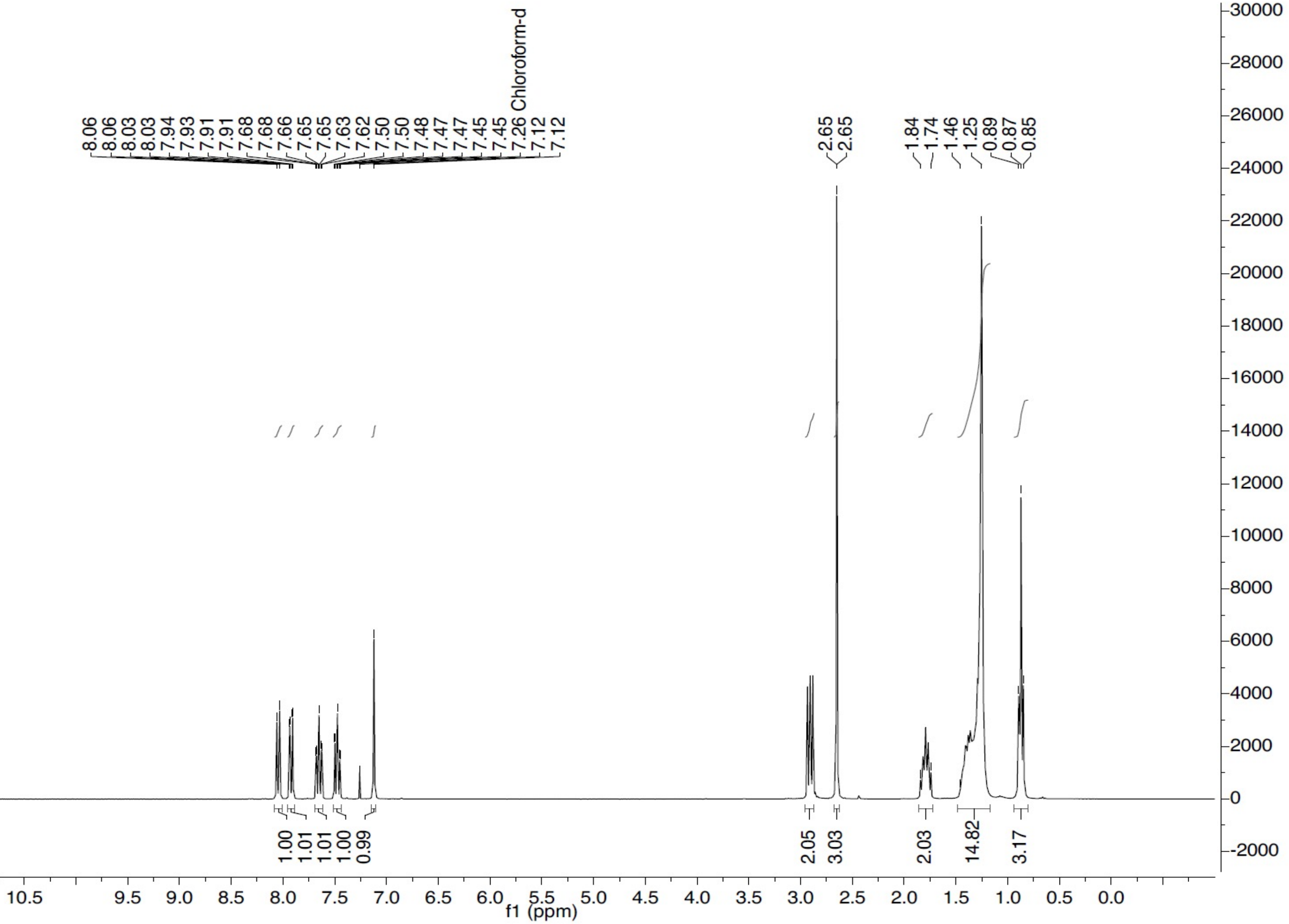

2-Decyl-4-methylquinoline (6)

^{13}C-NMR, 300 MHz, $CDCl_3$

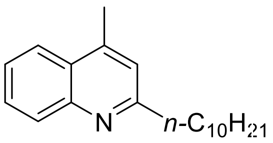

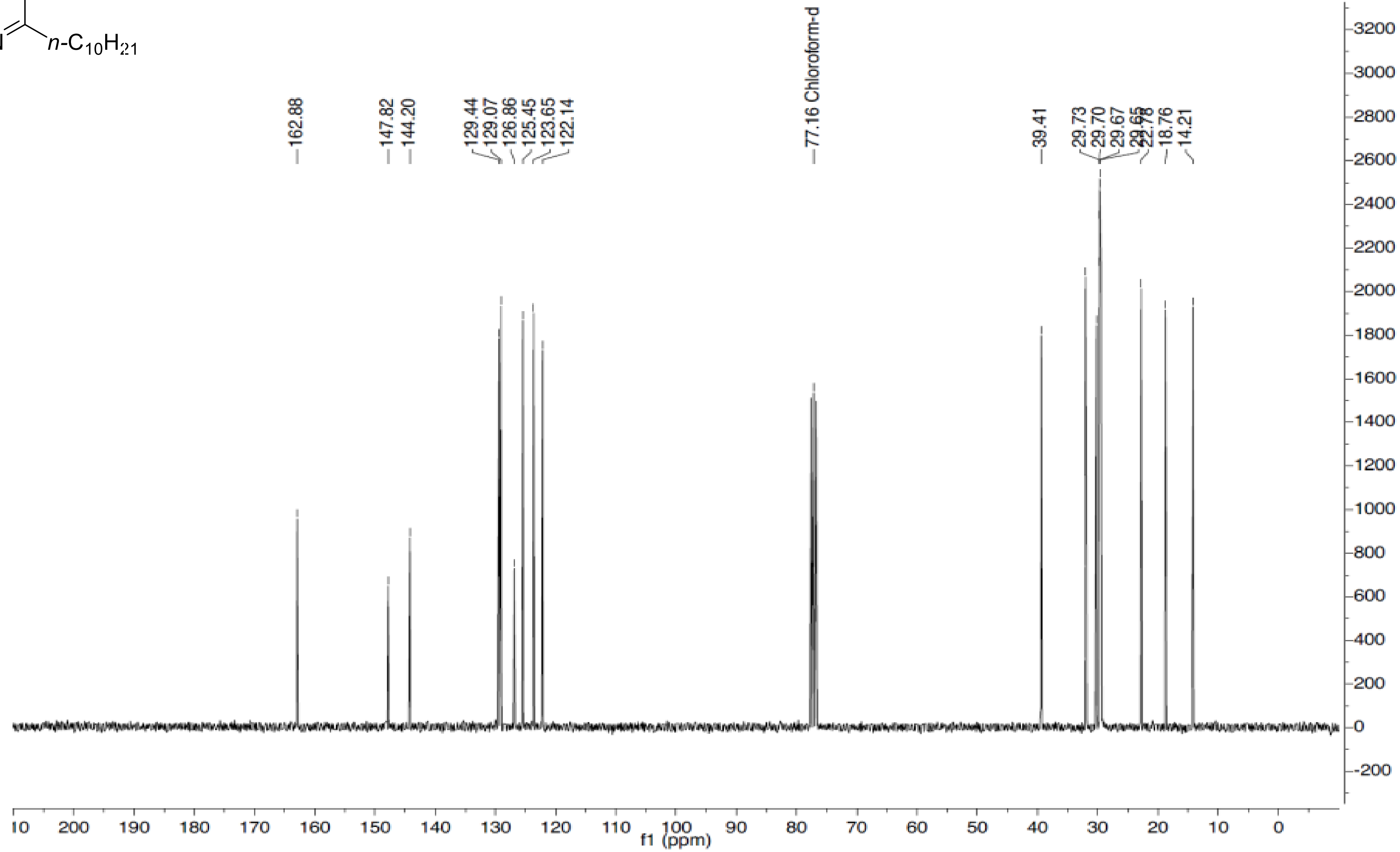

2-Ethyl-4-methylquinoline (7)

^{13}C-NMR, 300 MHz, C_6D_6

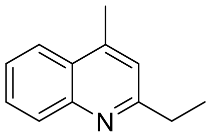

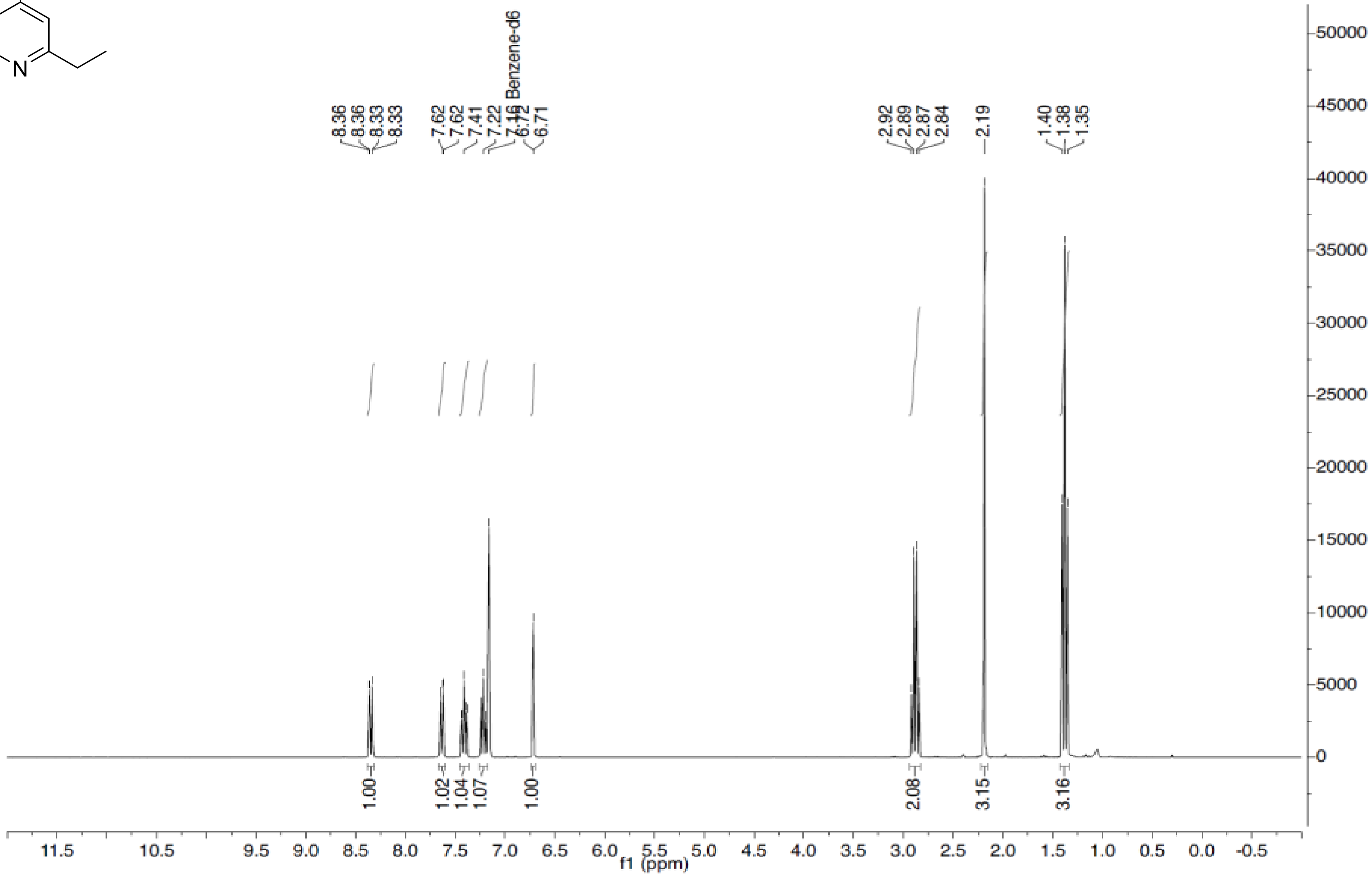

2-Ethyl-4-methylquinoline (7)

^{13}C-NMR, 75 MHz, C_6D_6

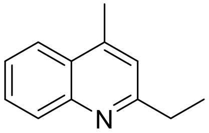

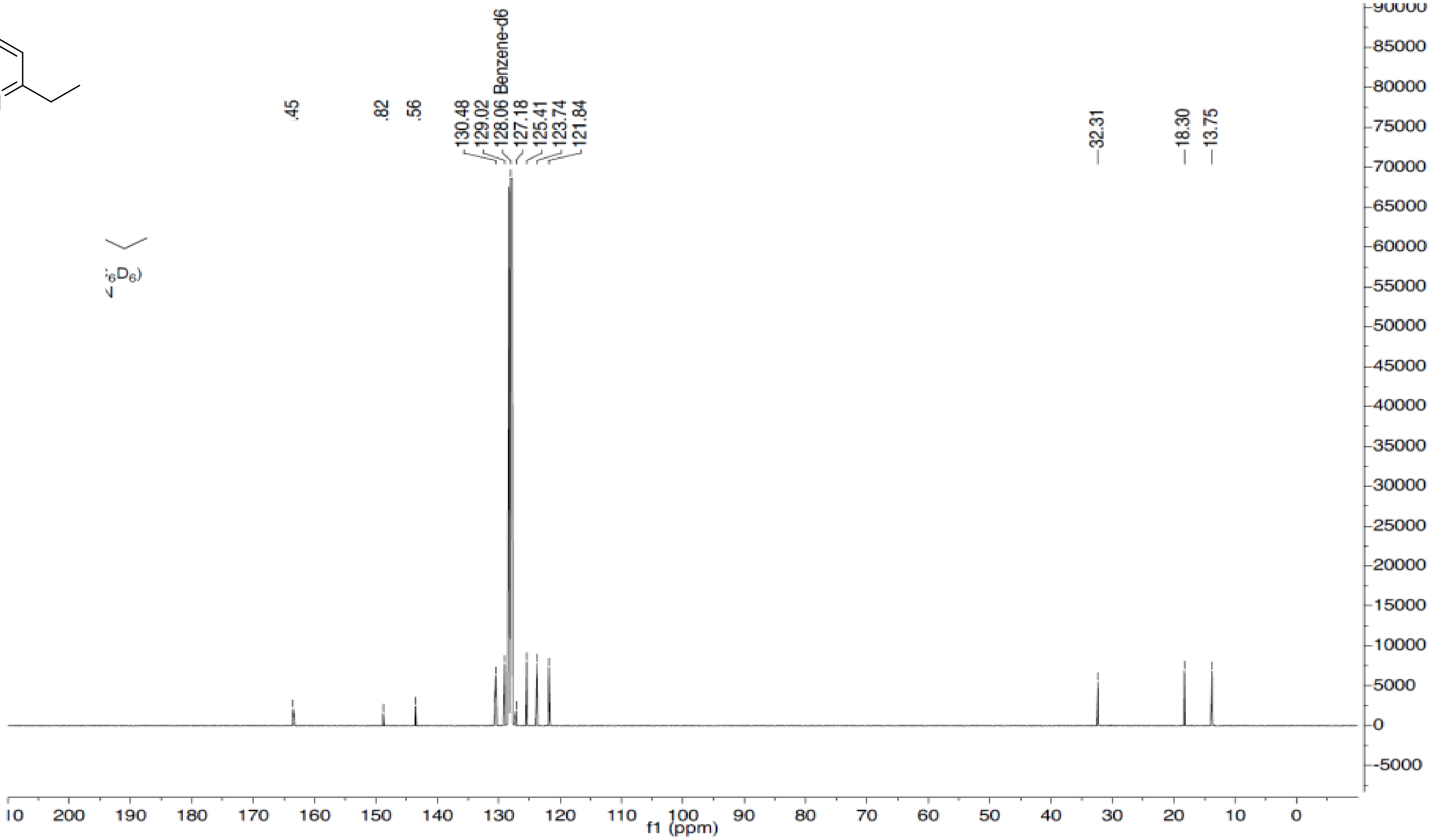

2-(2,3-Dimethylbutan-2-yl)-4-methylquinoline (8)

^{1}H-NMR, 300 MHz, $CDCl_3$

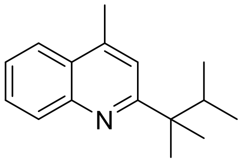

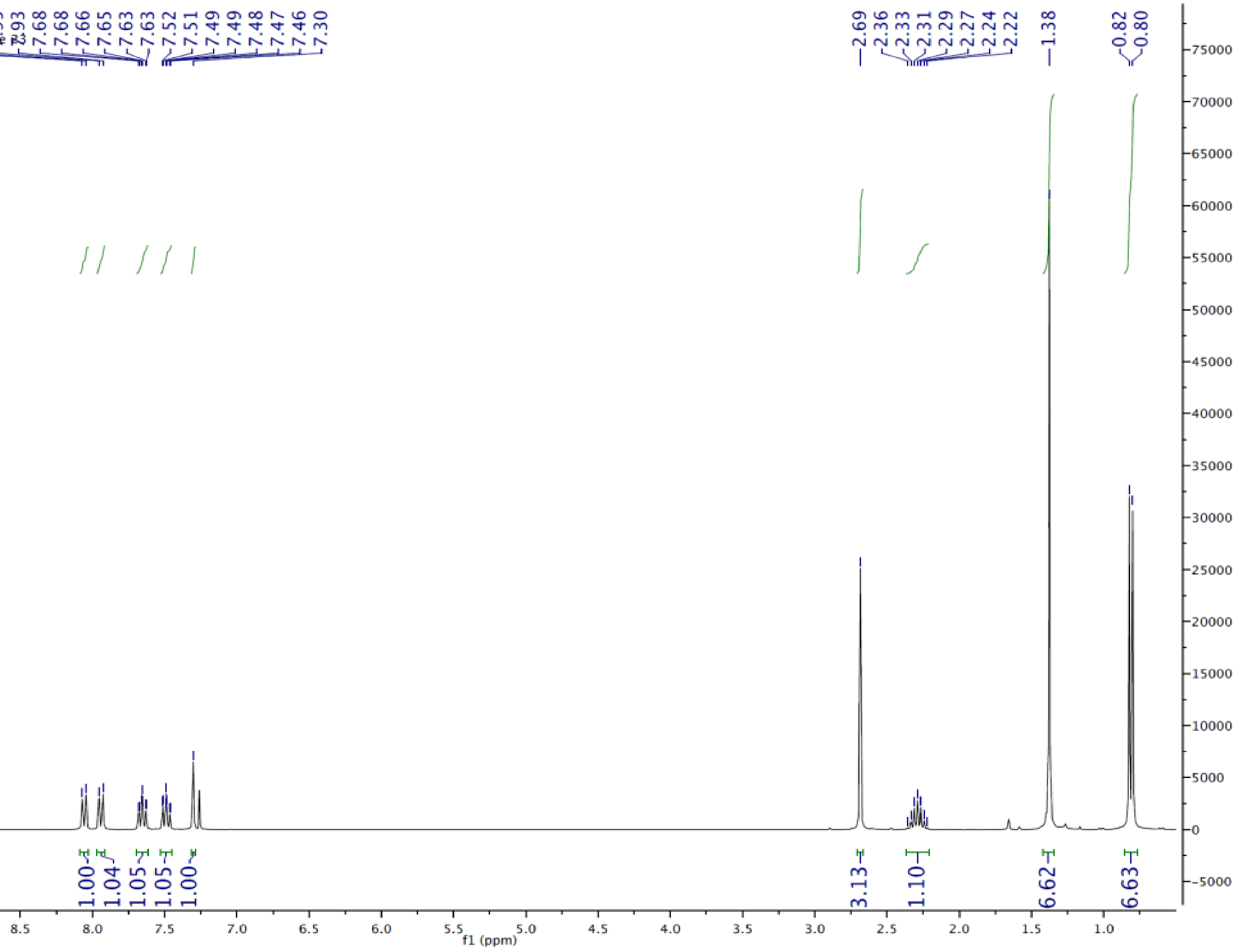

2-(2,3-Dimethylbutan-2-yl)-4-methylquinoline (8)

^{13}C-NMR, 300 MHz, $CDCl_3$

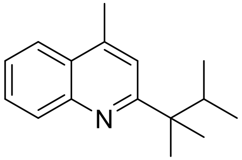

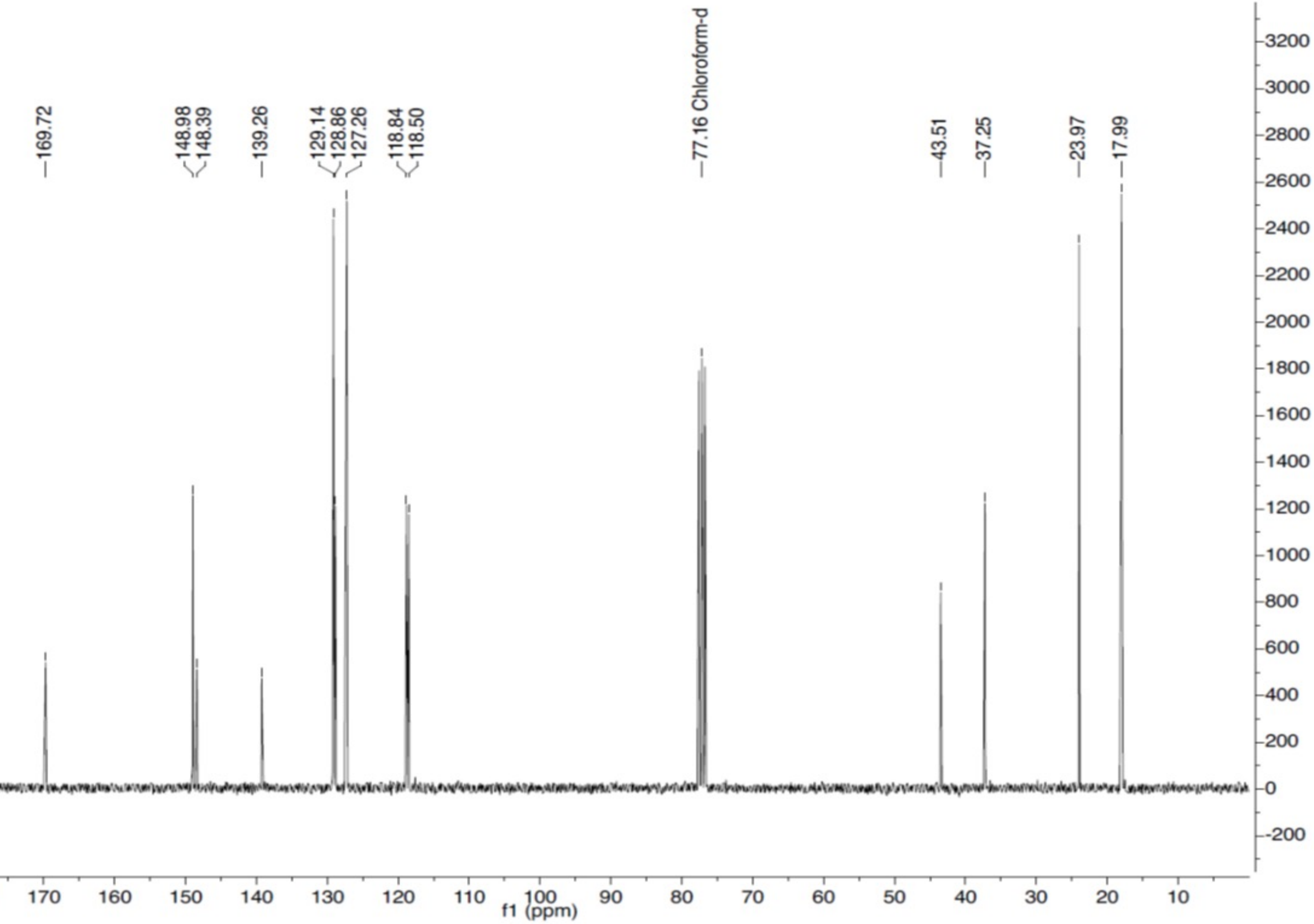

trans-4-Methyl-2-(2-methylcyclohexyl)quinoline (9)

^{1}H-NMR, 300 MHz, C_6D_6

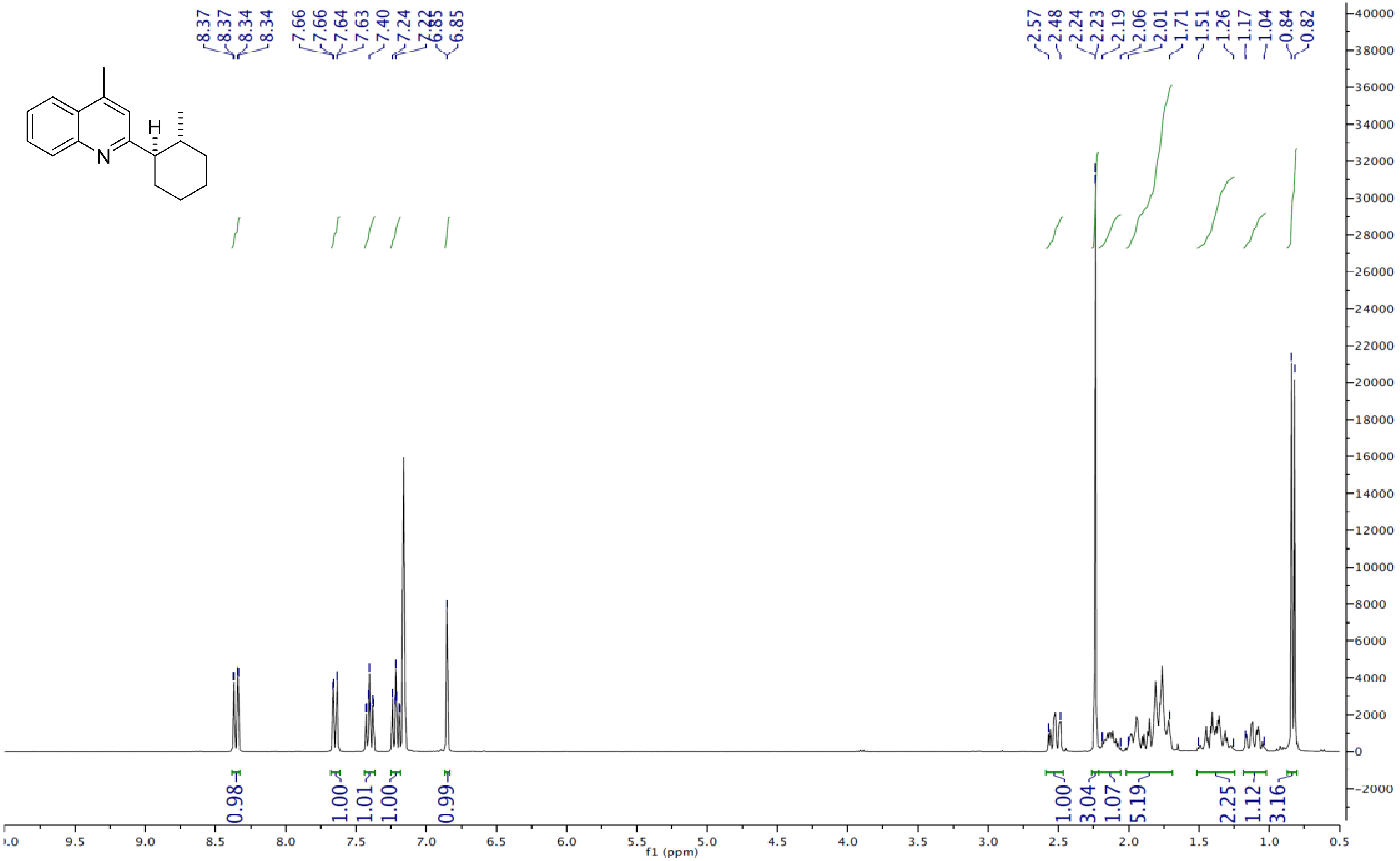

***trans*-4-Methyl-2-(2-methylcyclohexyl)quinoline (9)**

^{13}C-NMR, 75 MHz, C_6D_6

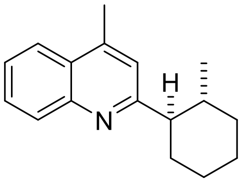

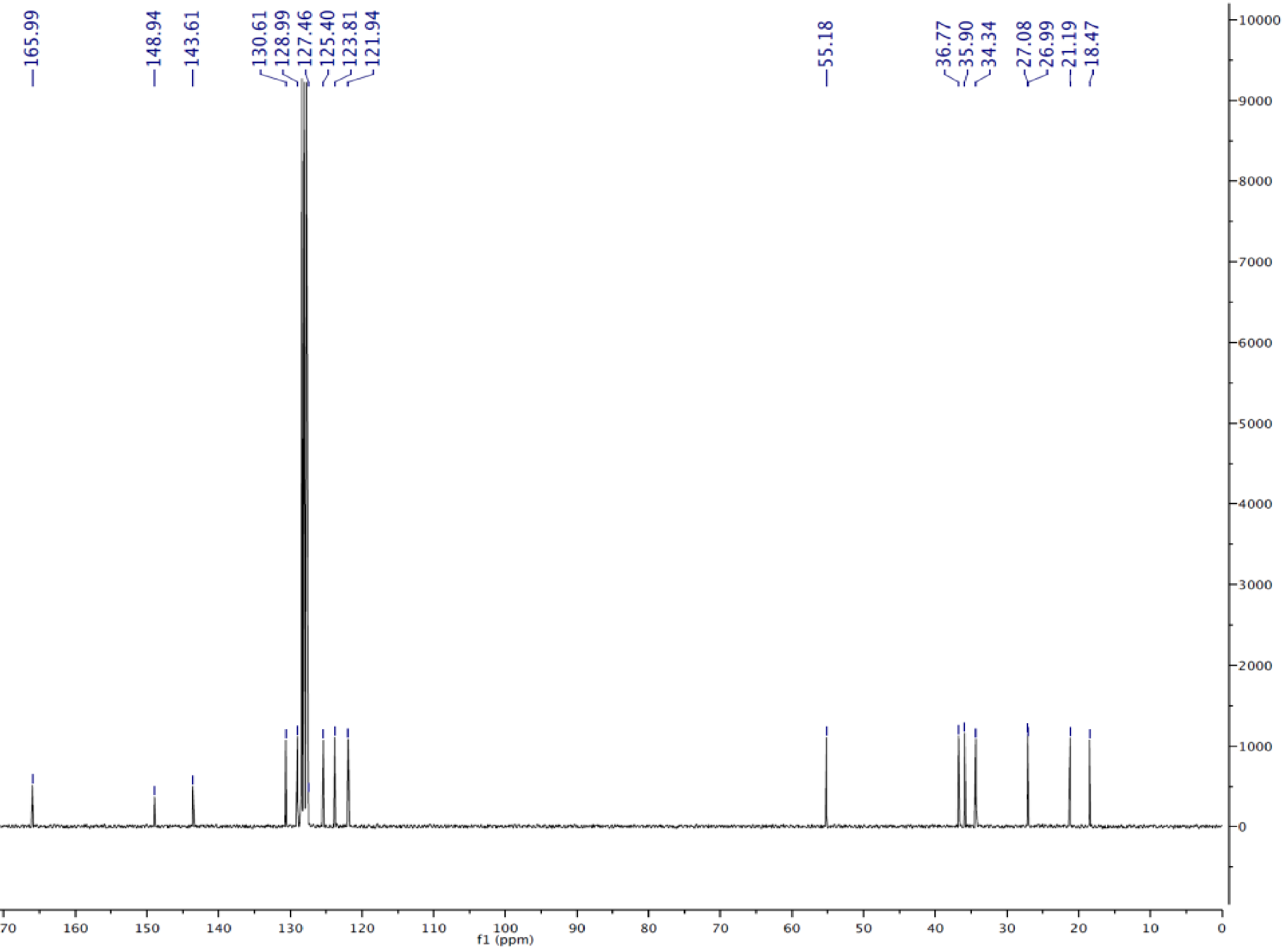

11-(4-Methylquinolin-2-yl)undecan-1-ol (10)

^{1}H-NMR (400 MHz, $CDCl_3$)

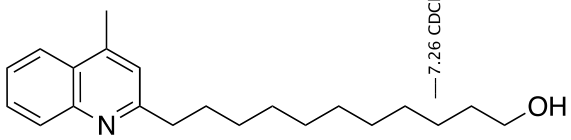

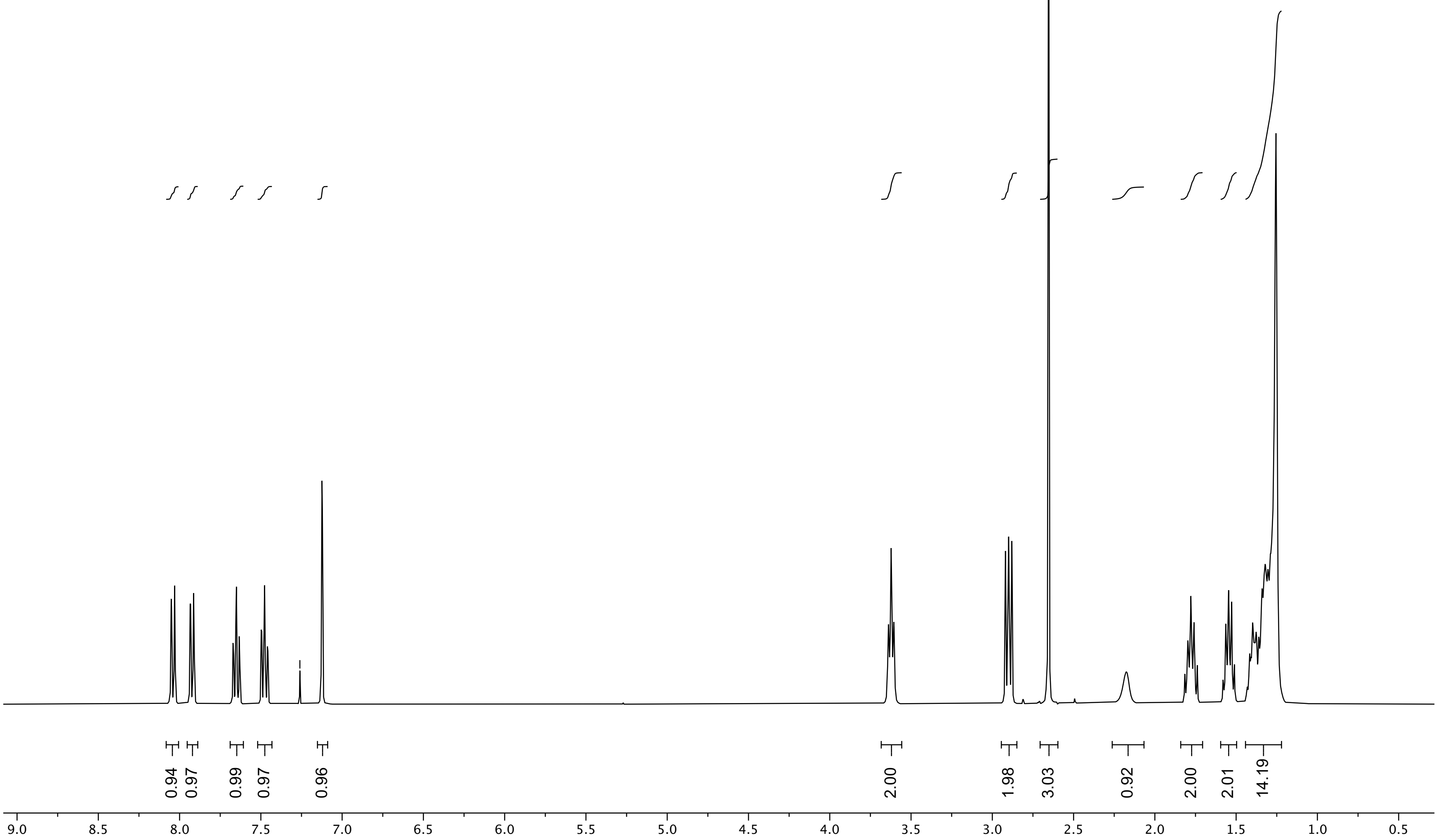

11-(4-Methylquinolin-2-yl)undecan-1-ol (10)

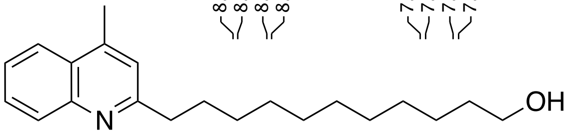

^{1}H-NMR (400 MHz, $CDCl_3$)

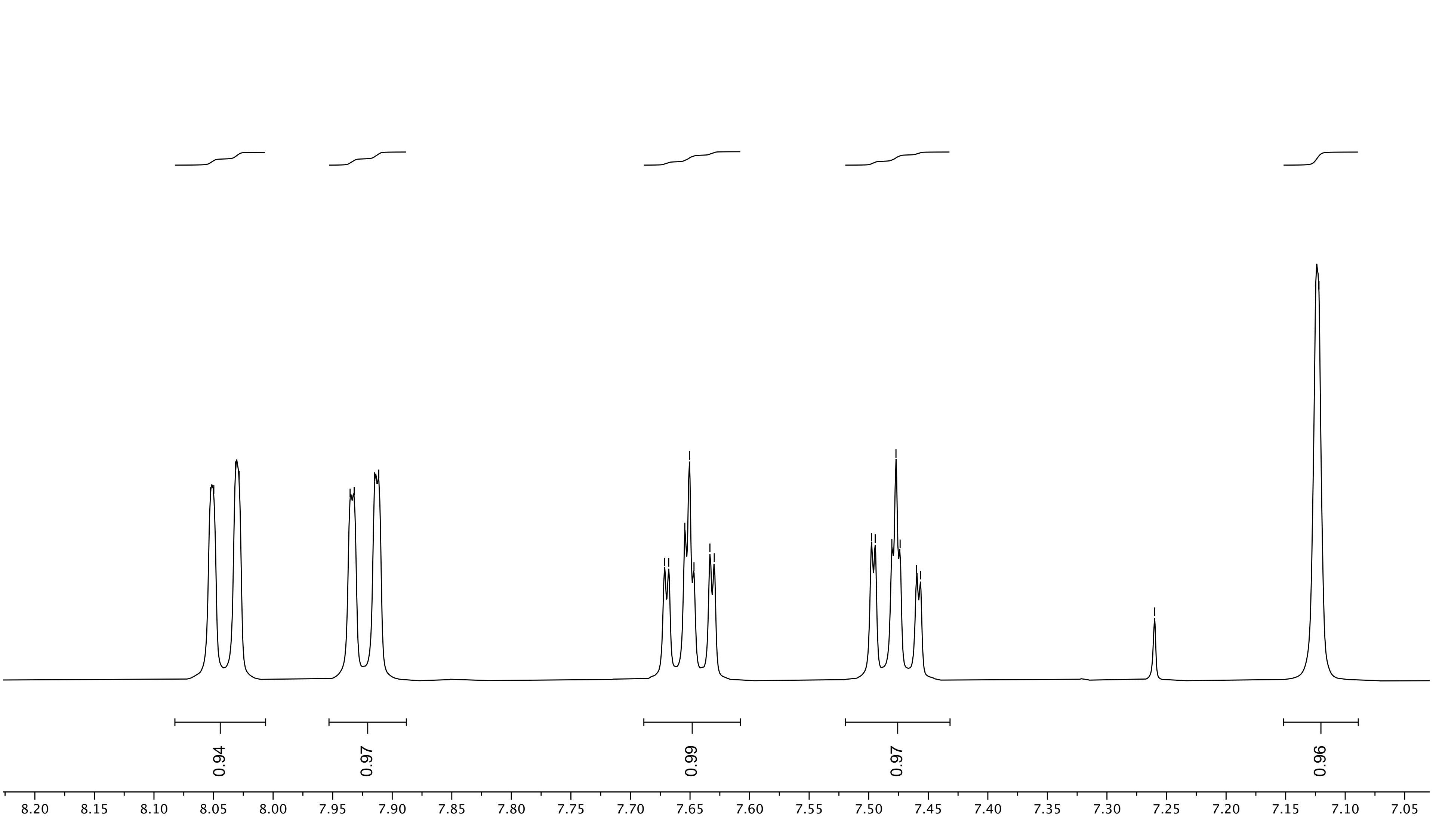

11-(4-Methylquinolin-2-yl)undecan-1-ol (10)

^{1}H-NMR (400 MHz, $CDCl_3$)

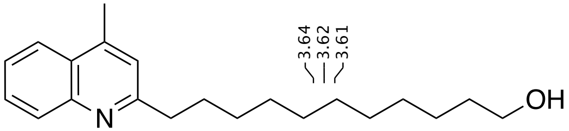

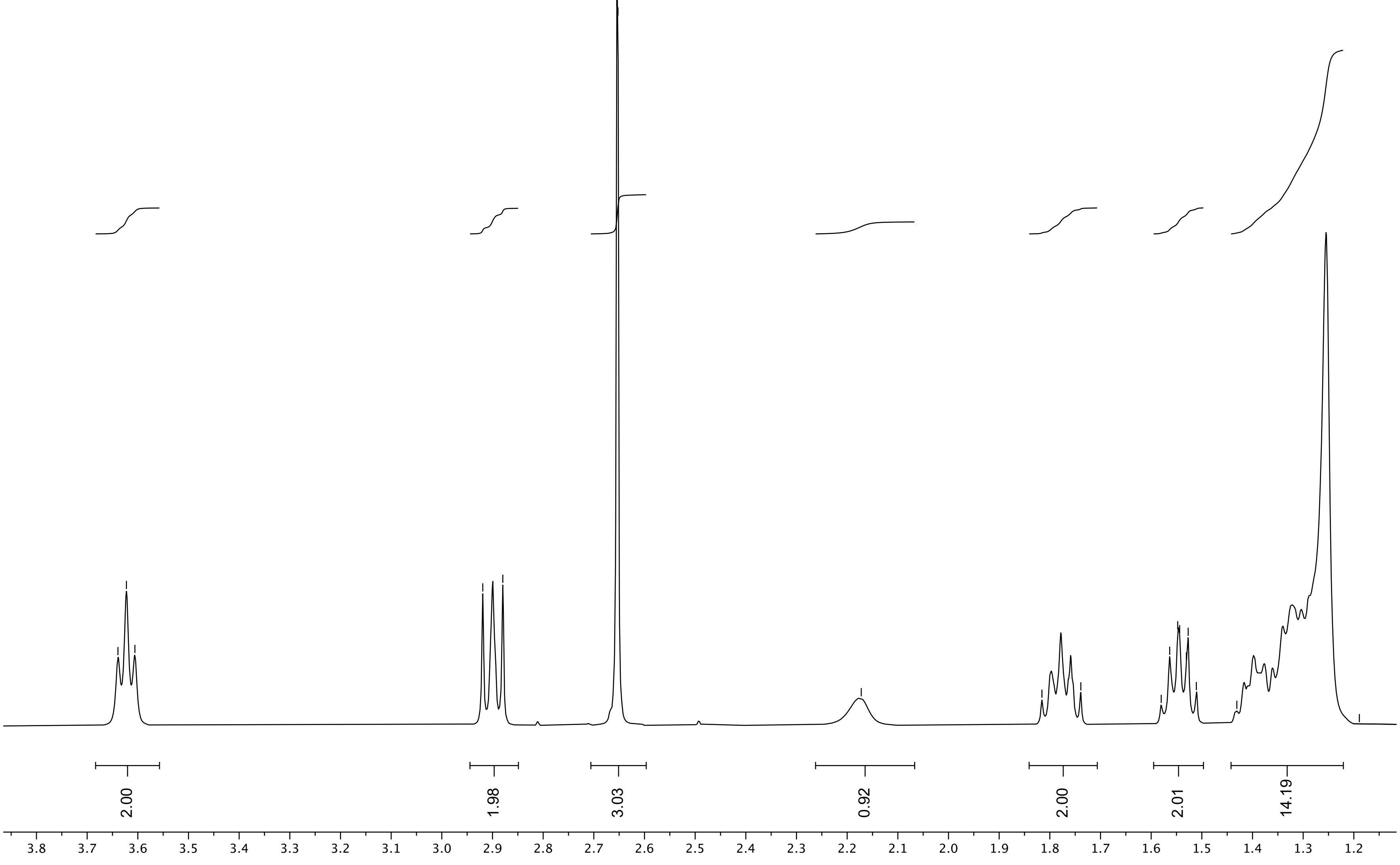

11-(4-Methylquinolin-2-yl)undecan-1-ol (10)

^{13}C-NMR (101 MHz, $CDCl_3$)

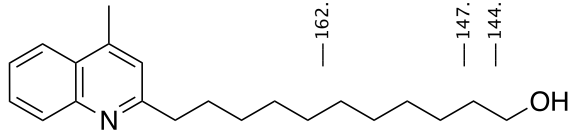

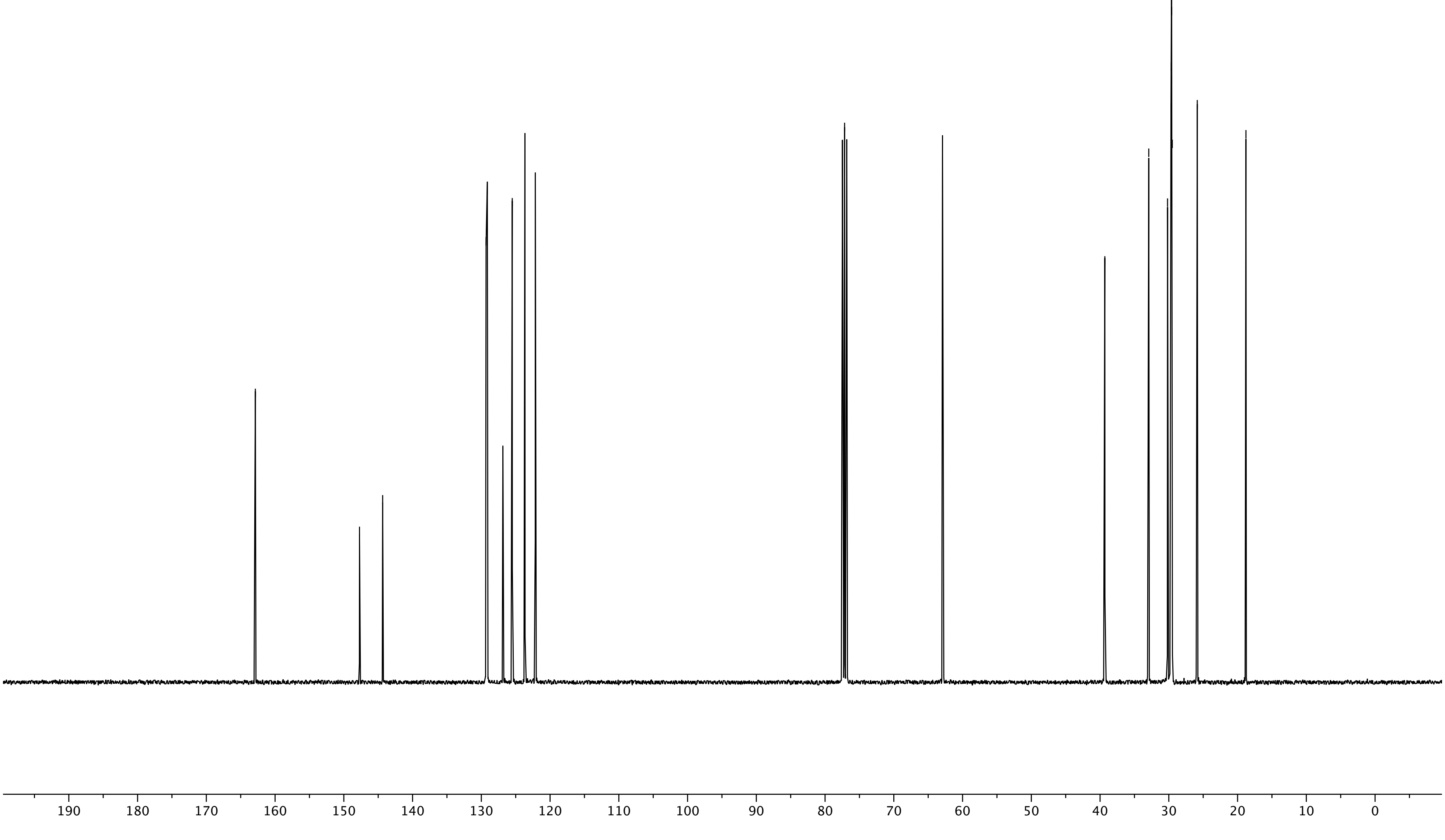

11-(4-Methylquinolin-2-yl)undecan-1-ol (10)

^{13}C-NMR (101 MHz, $CDCl_3$)

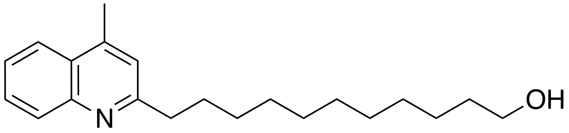

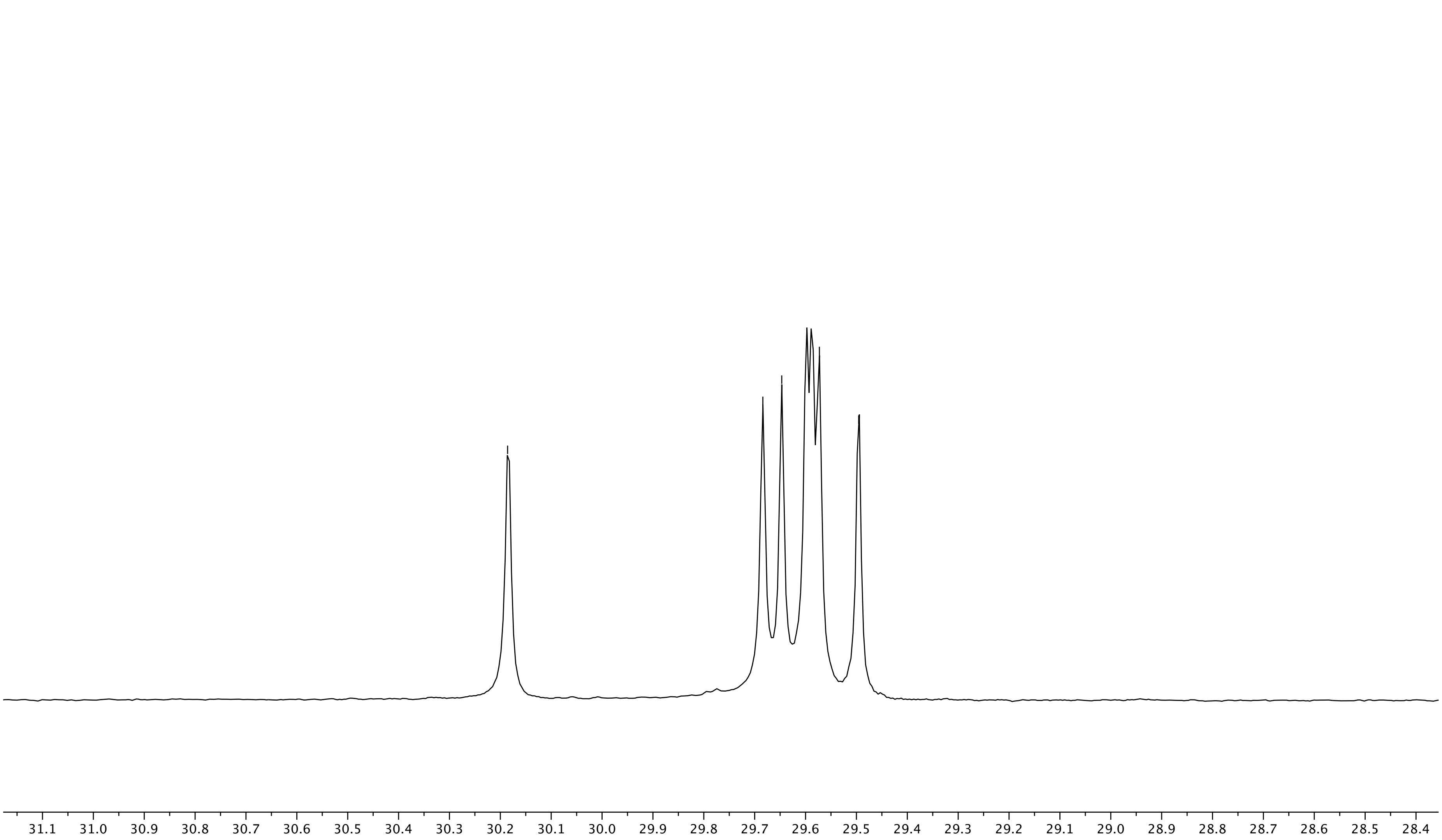

11-(4-Methylquinolin-2-yl)undecan-1-ol (10)

Dept-135 (101 MHz, $CDCl_3$)

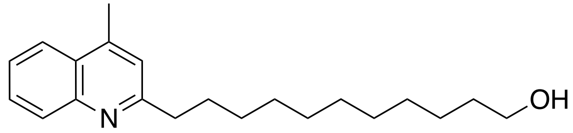

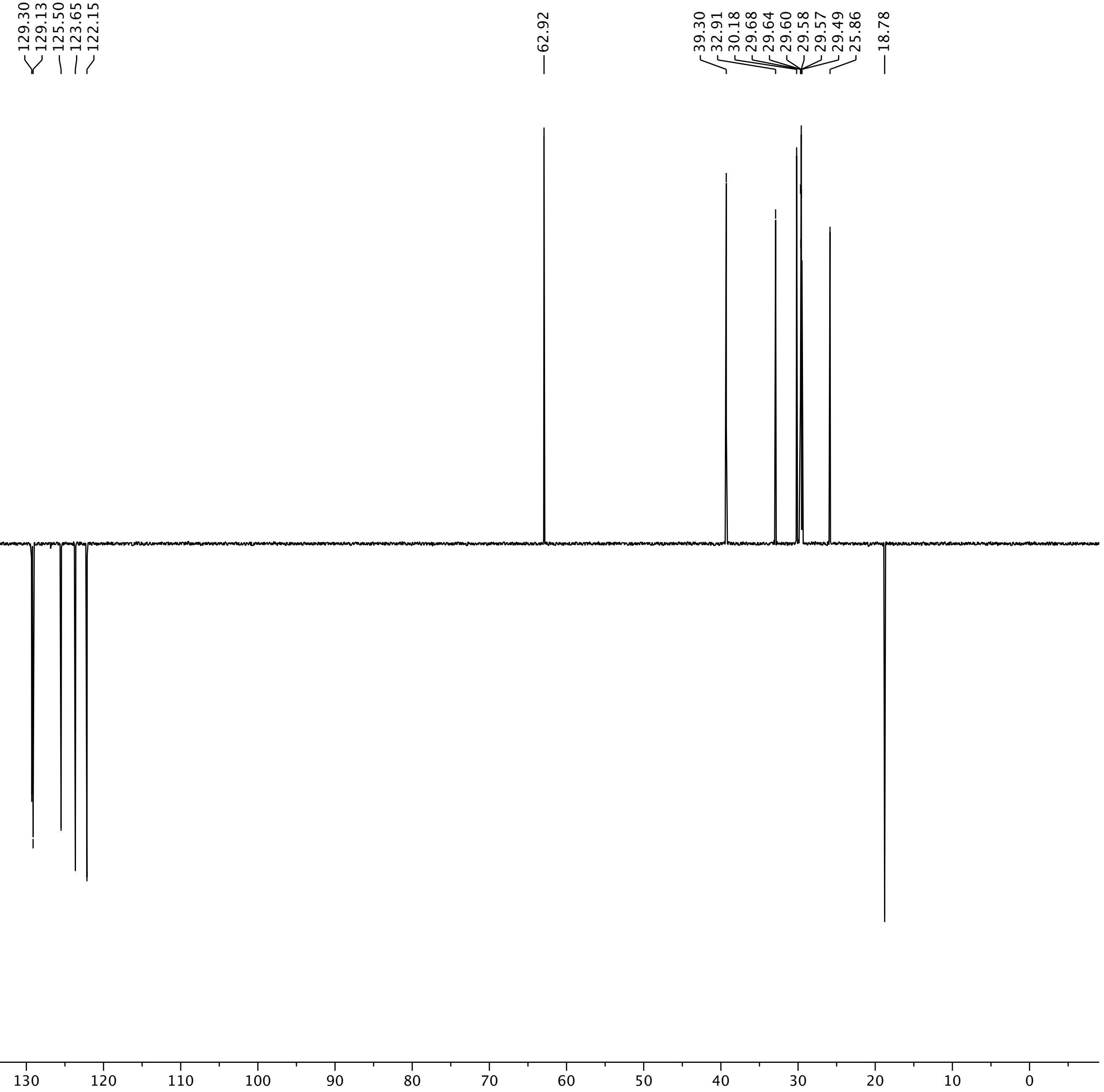

11-(4-Methylquinolin-2-yl)undecan-1-ol (10)

^1H-^1H COSY (400 MHz, $CDCl_3$)

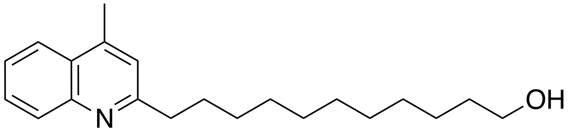

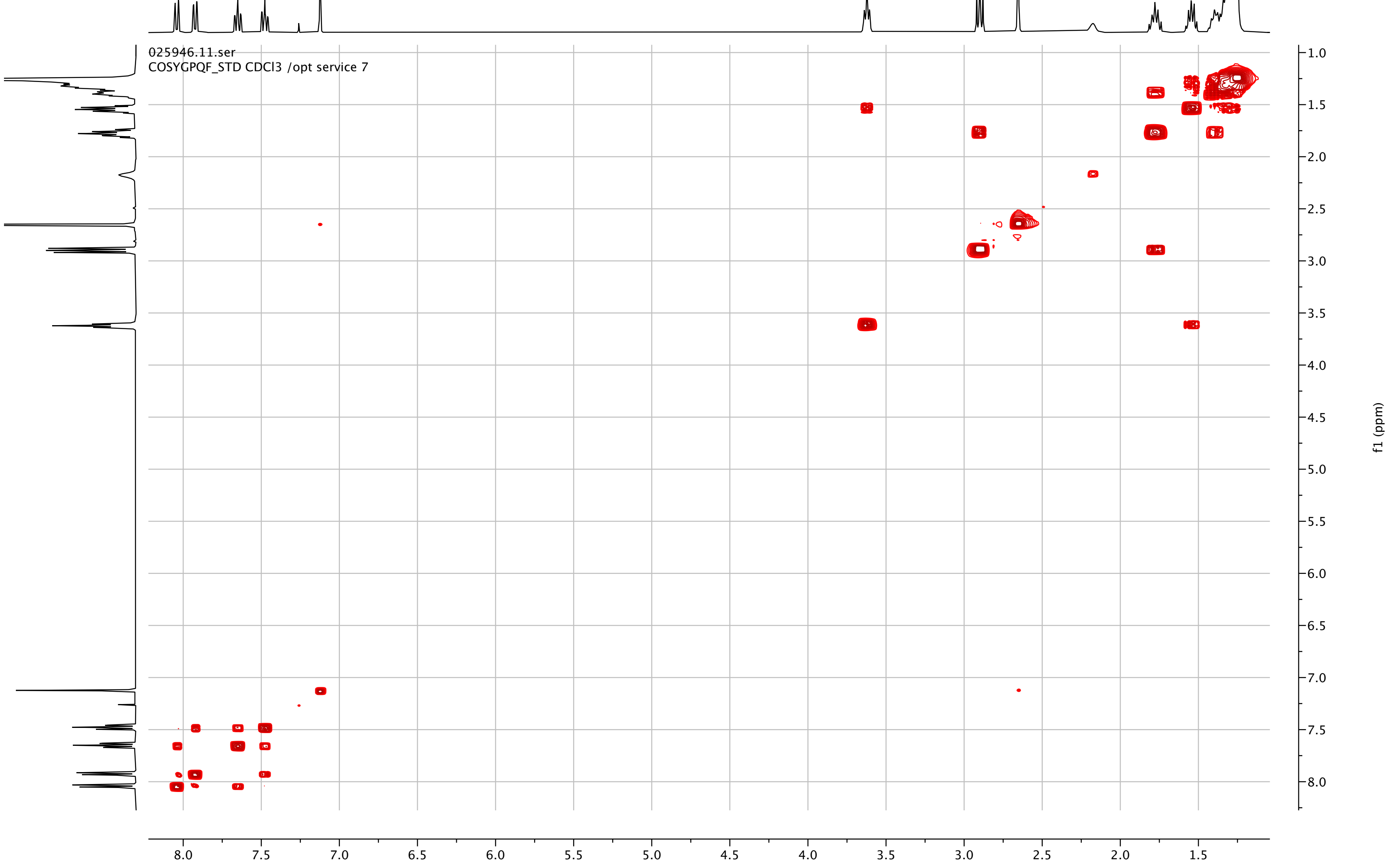

11-(4-Methylquinolin-2-yl)undecan-1-ol (10)

^1H-^1H COSY (400 MHz, $CDCl_3$)

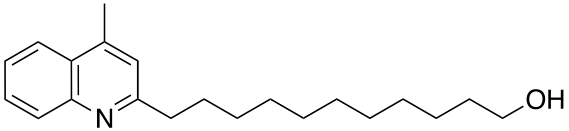

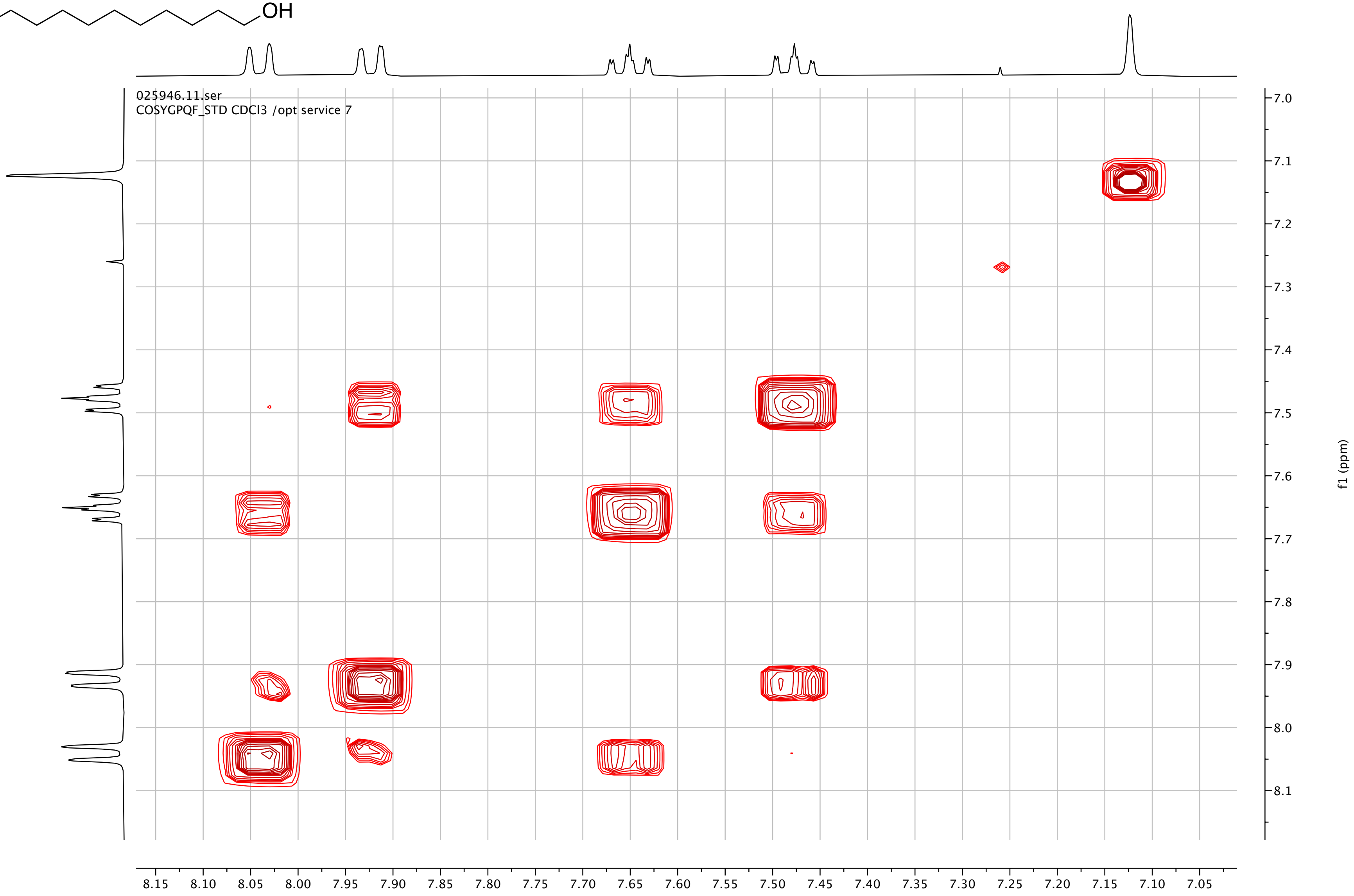

11-(4-Methylquinolin-2-yl)undecan-1-ol (10)

^{1}H-^{1}H COSY (400 MHz, $CDCl_3$)

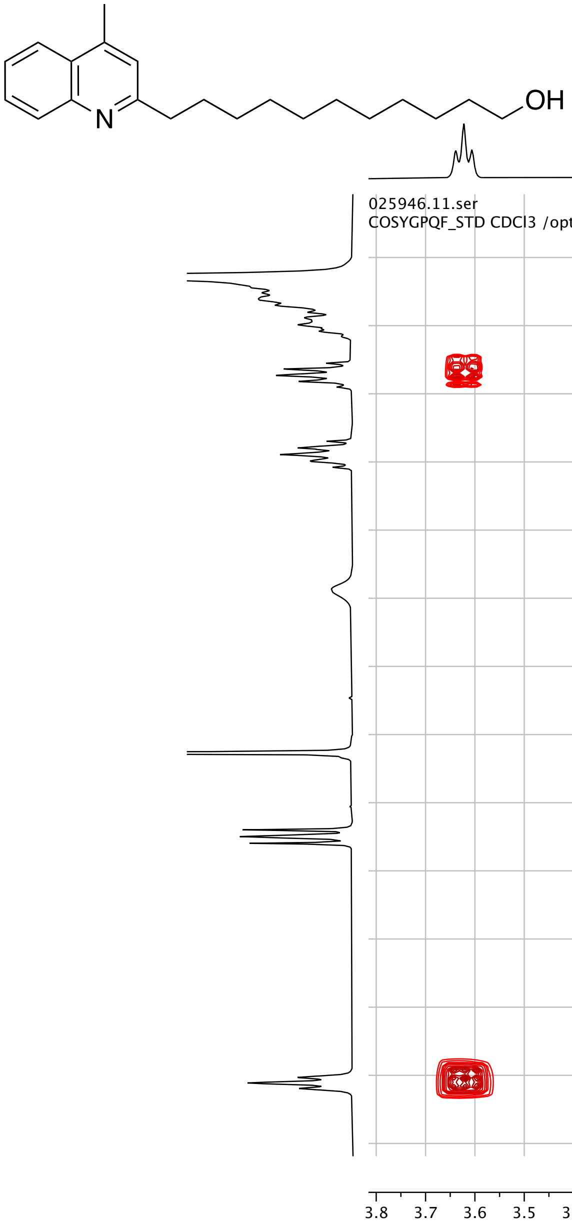

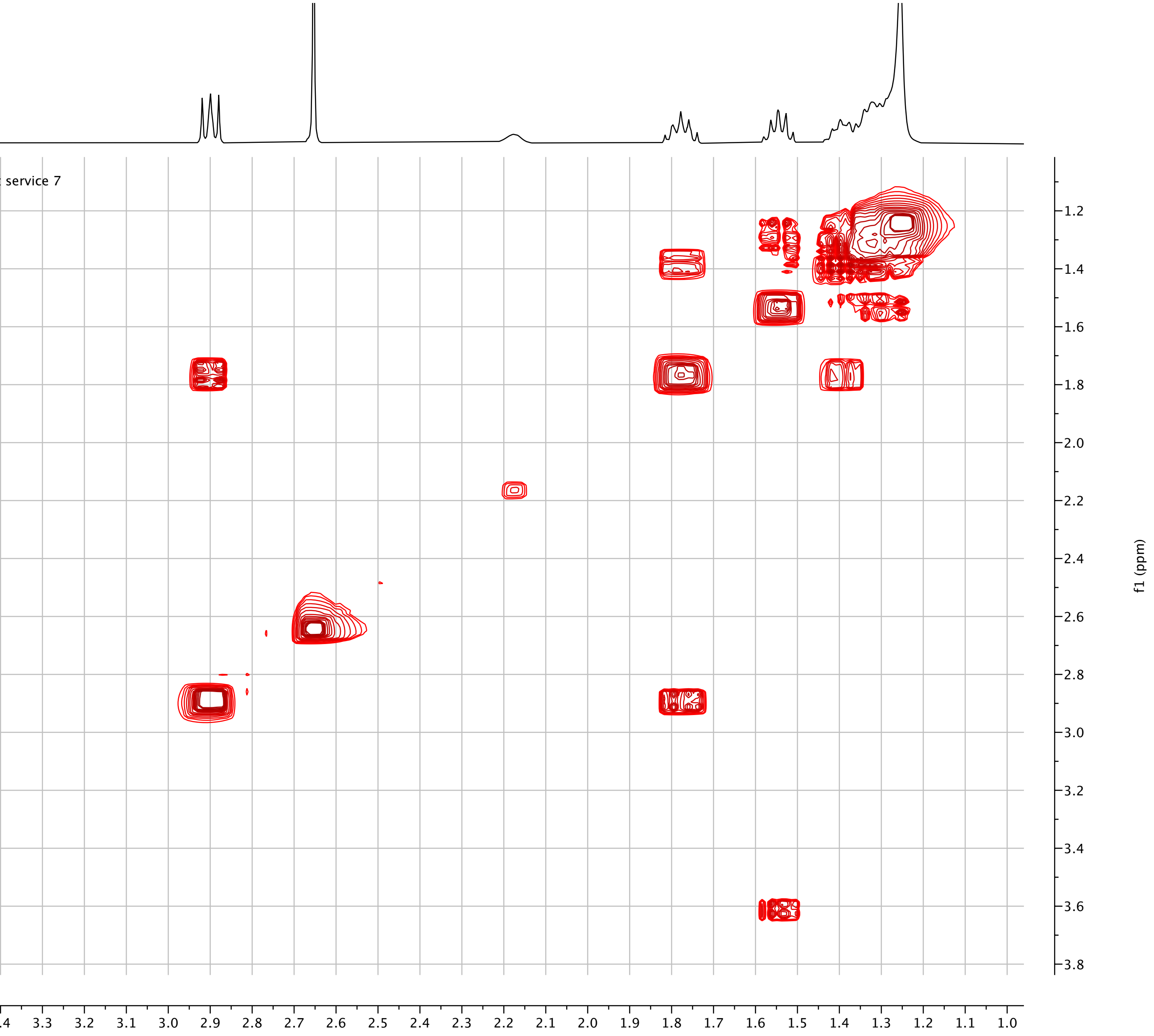

11-(4-Methylquinolin-2-yl)undecan-1-ol (10)

HSQC-EDT (400 MHz, $CDCl_3$)

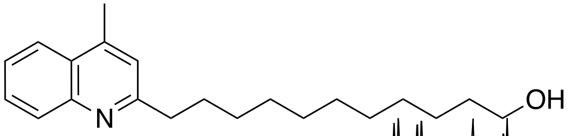

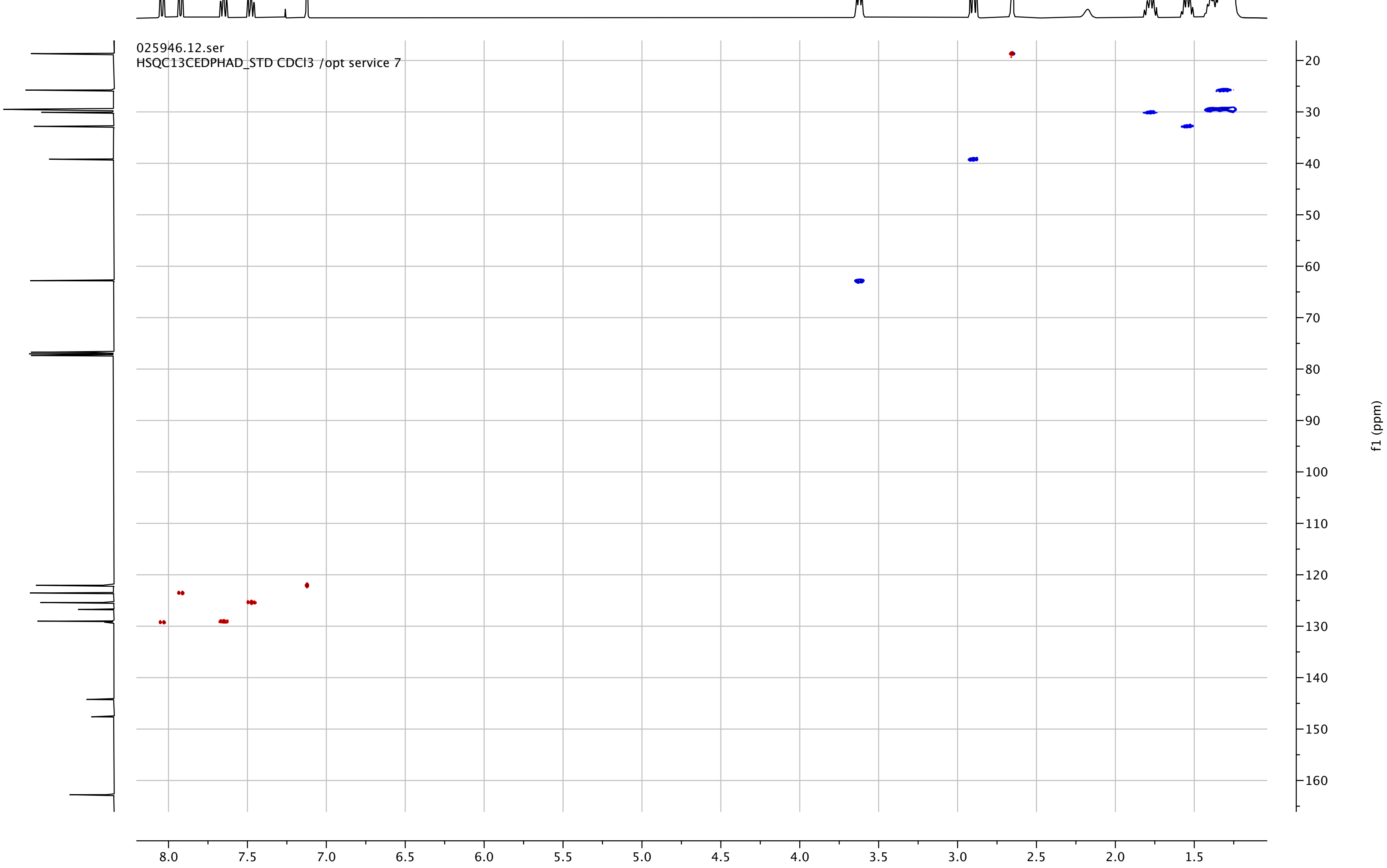

11-(4-Methylquinolin-2-yl)undecan-1-ol (10)

HSQC-EDT (400 MHz, $CDCl_3$)

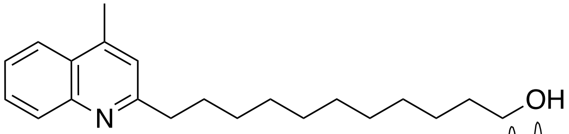

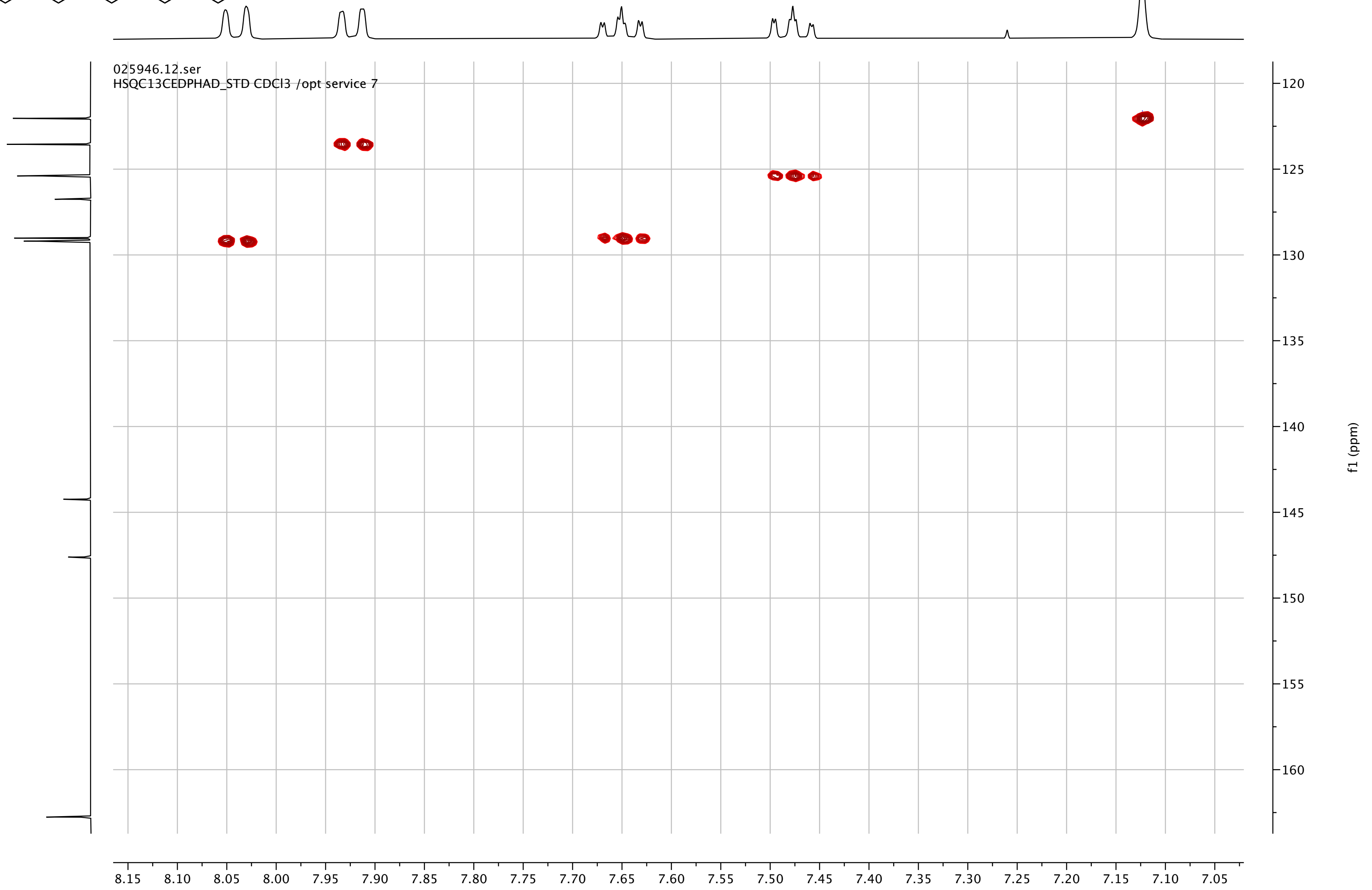

11-(4-Methylquinolin-2-yl)undecan-1-ol (10)

HSQC-EDT (400 MHz, $CDCl_3$)

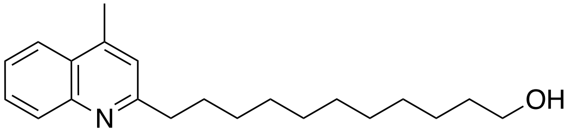

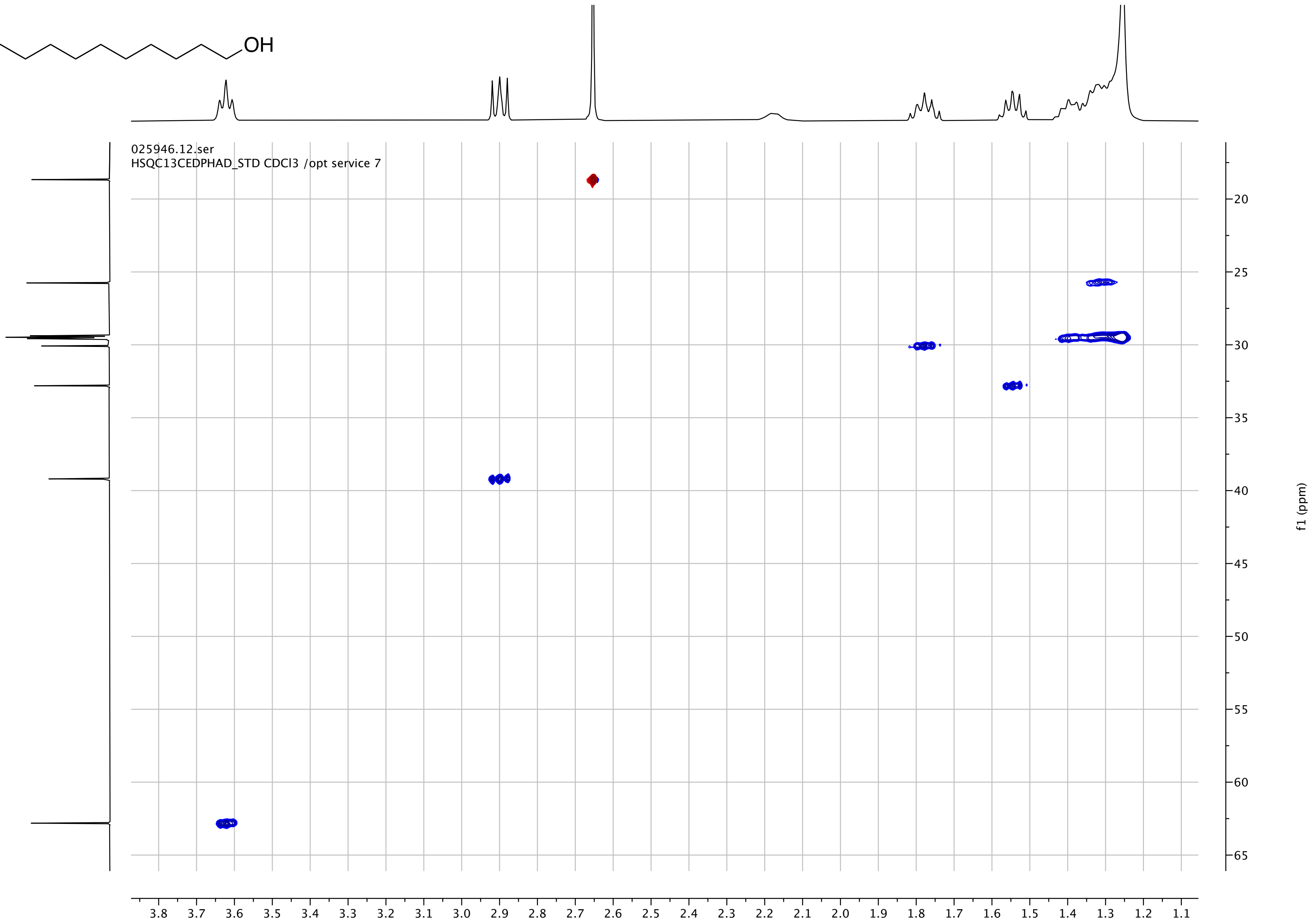

10-(4-Methylquinolin-2-yl)undecan-1-ol (minor)

^{1}H-NMR (400 MHz, $CDCl_3$)

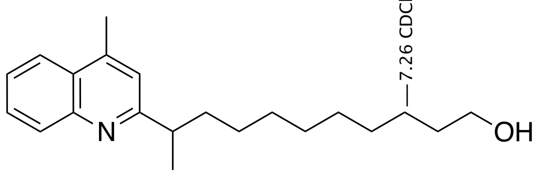

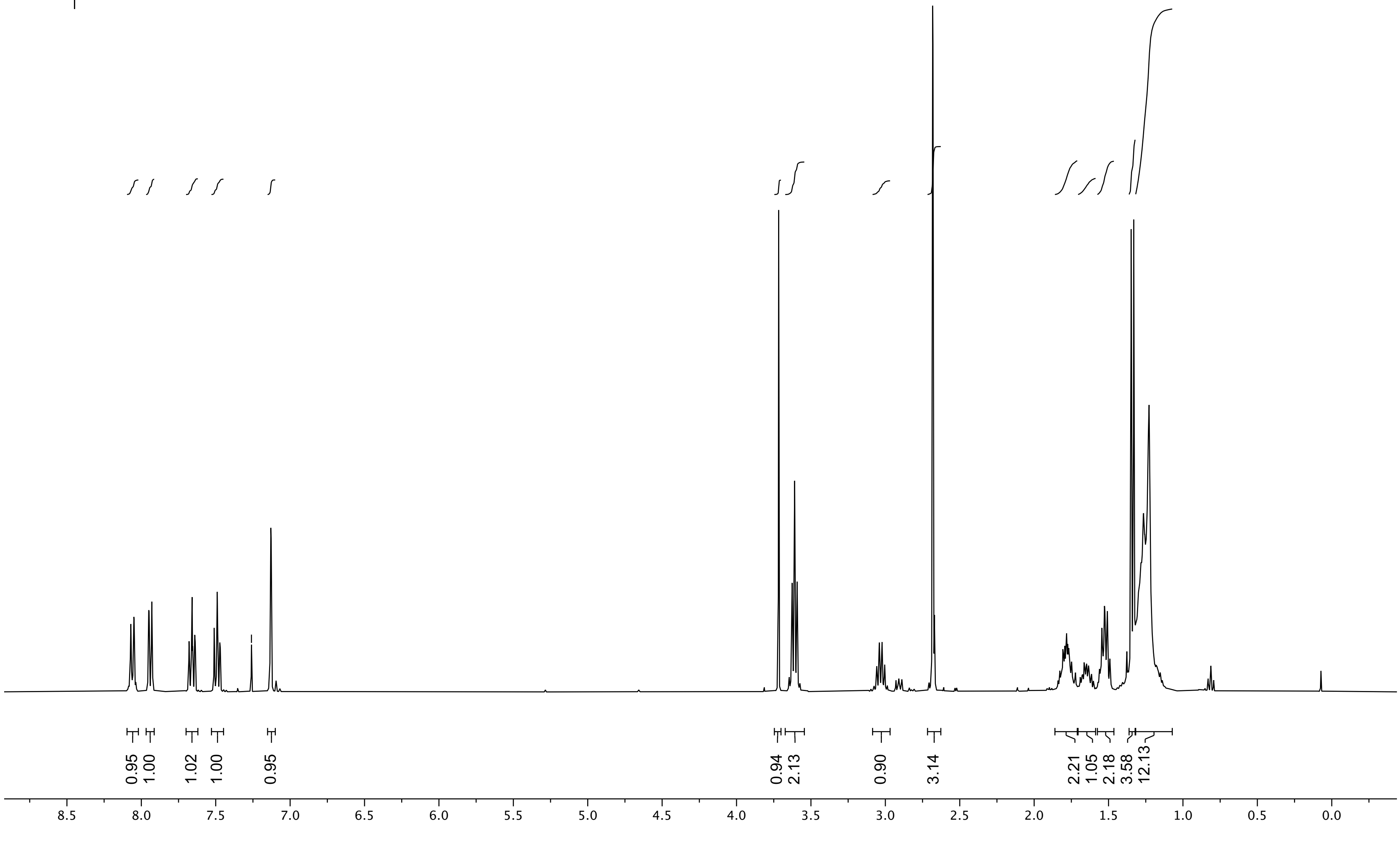

10-(4-Methylquinolin-2-yl)undecan-1-ol (minor)

^{1}H-NMR (400 MHz, $CDCl_3$)

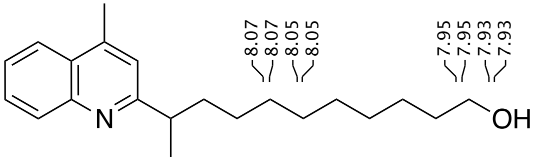

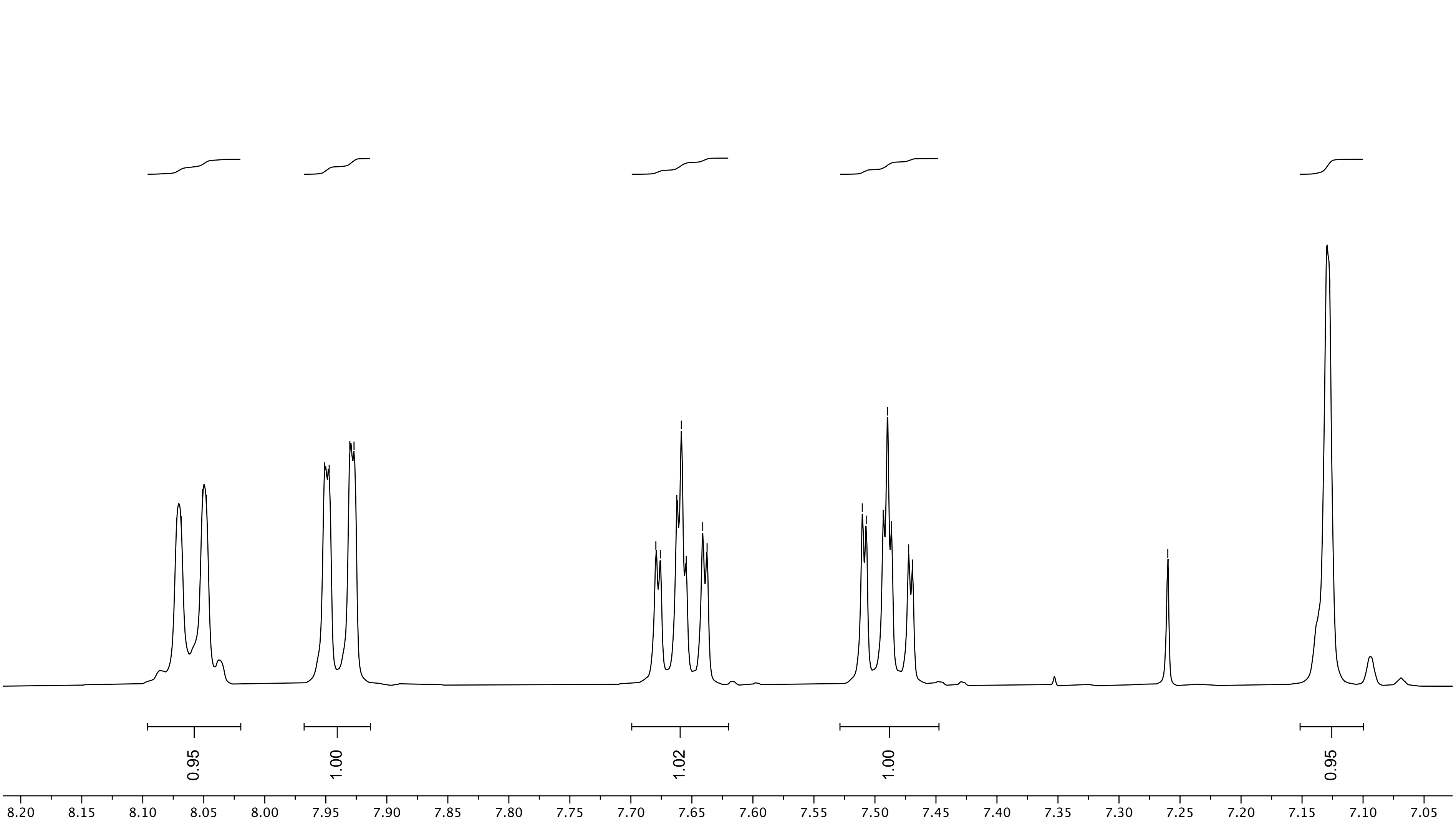

10-(4-Methylquinolin-2-yl)undecan-1-ol (minor)

^{1}H-NMR (400 MHz, $CDCl_3$)

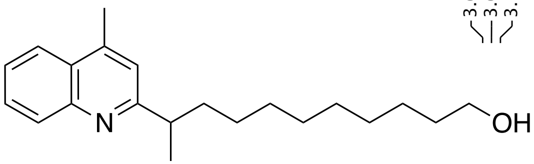

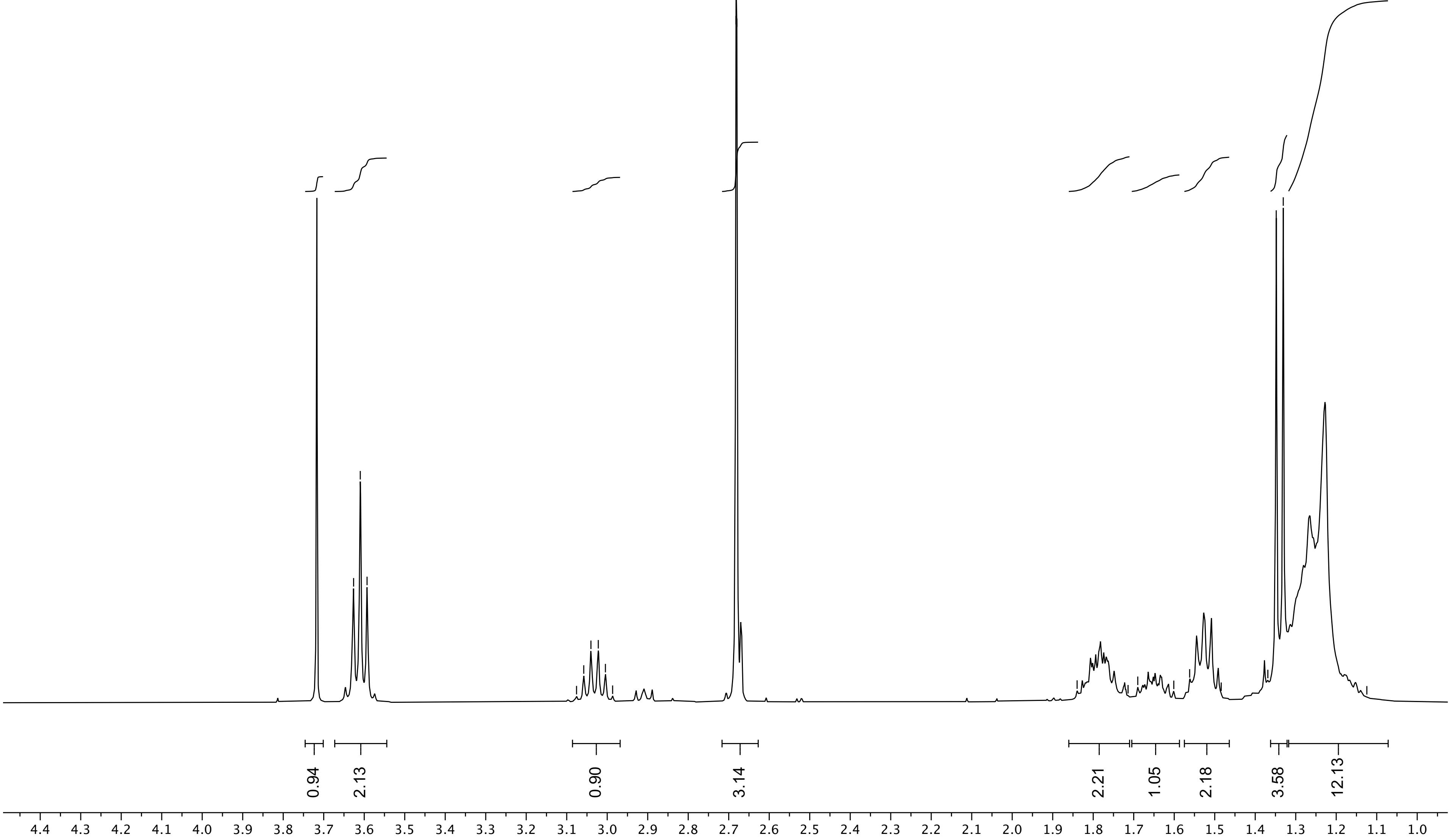

10-(4-Methylquinolin-2-yl)undecan-1-ol (minor)

^{13}C-NMR (101 MHz, $CDCl_3$)

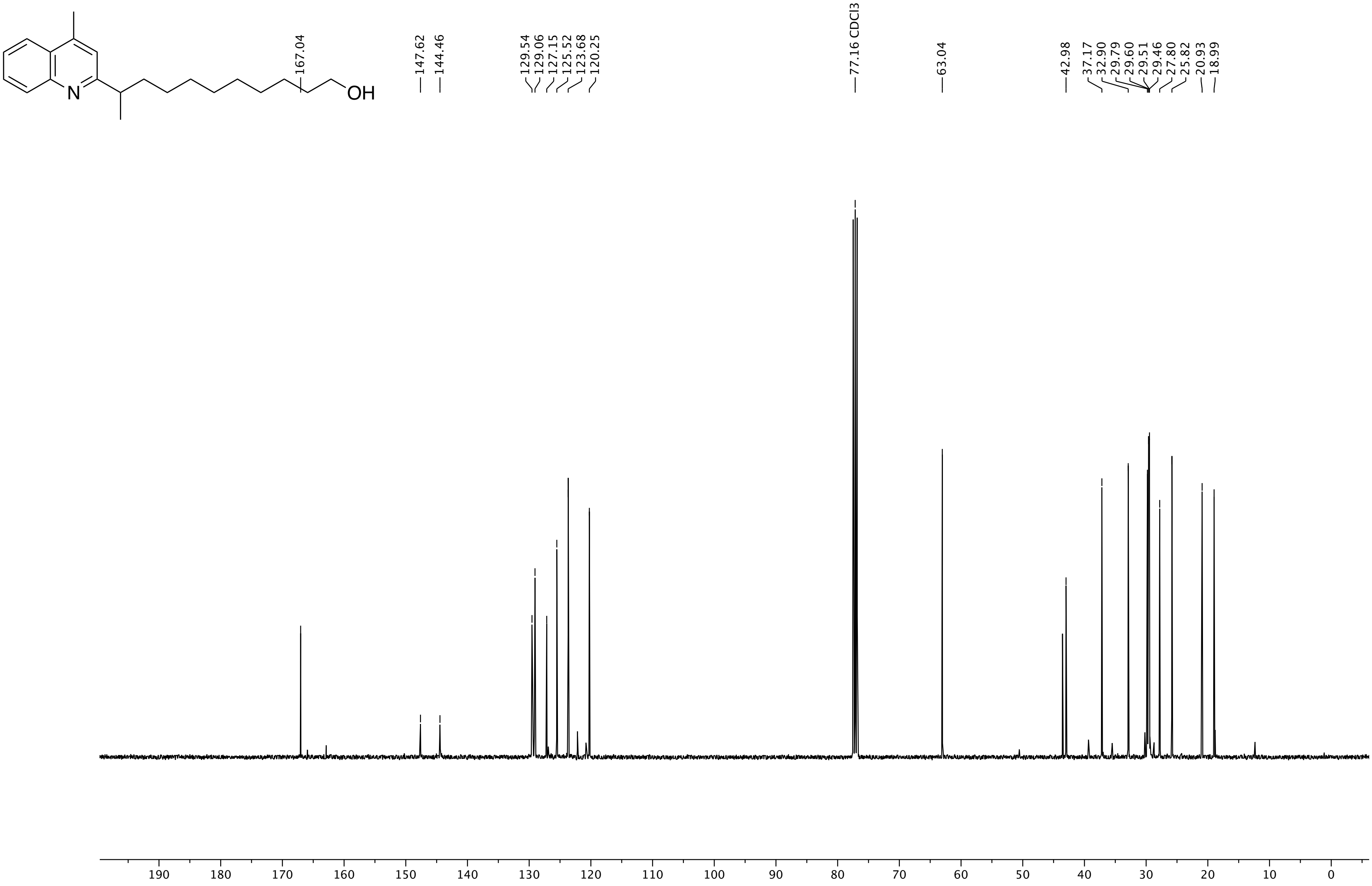

10-(4-Methylquinolin-2-yl)undecan-1-ol (minor)

^{13}C-NMR (101 MHz, $CDCl_3$)

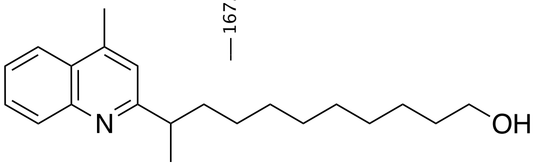

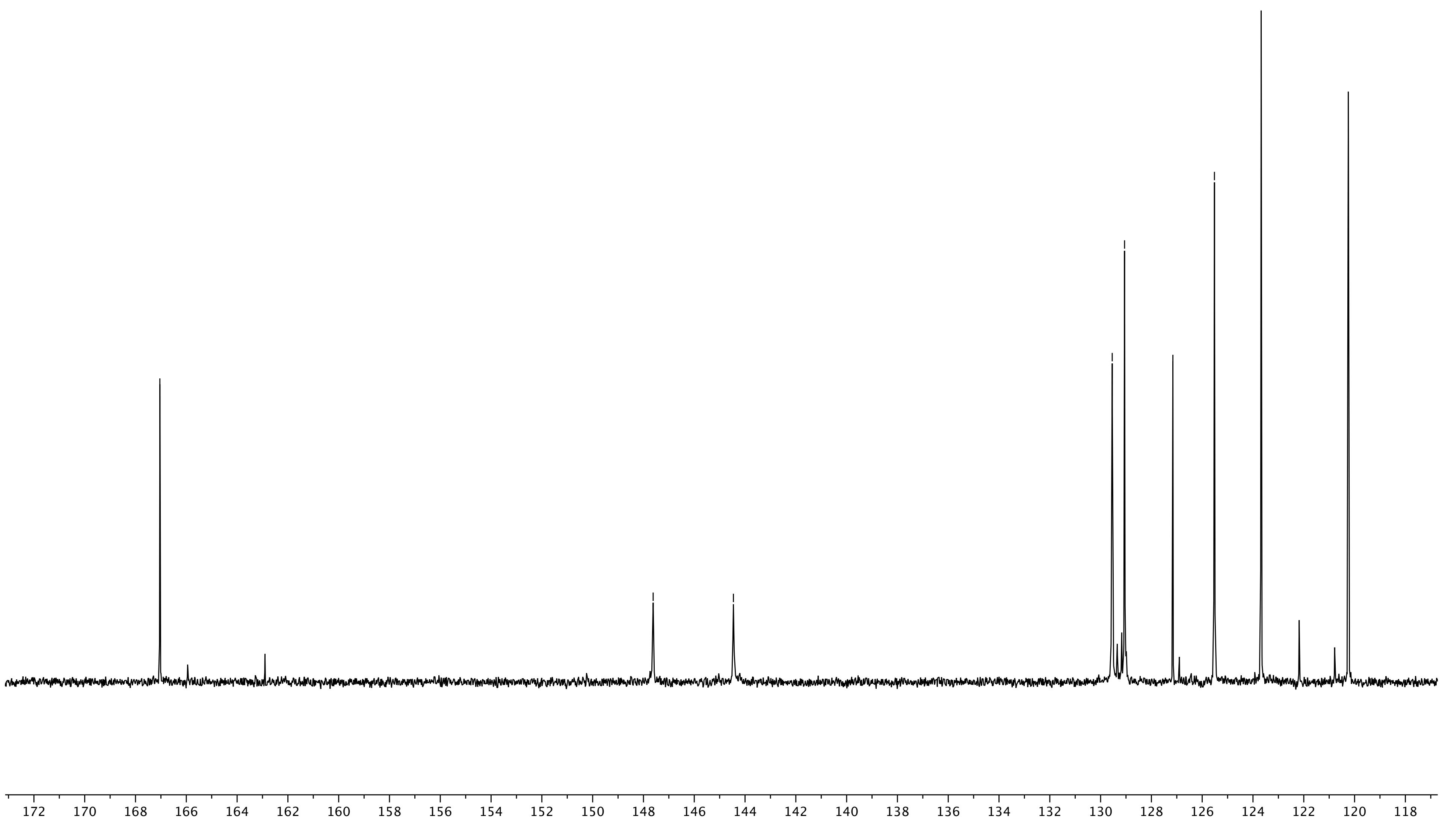

10-(4-Methylquinolin-2-yl)undecan-1-ol (minor)

^{13}C-NMR (101 MHz, CDCl$_3$)

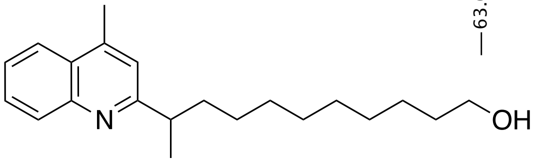

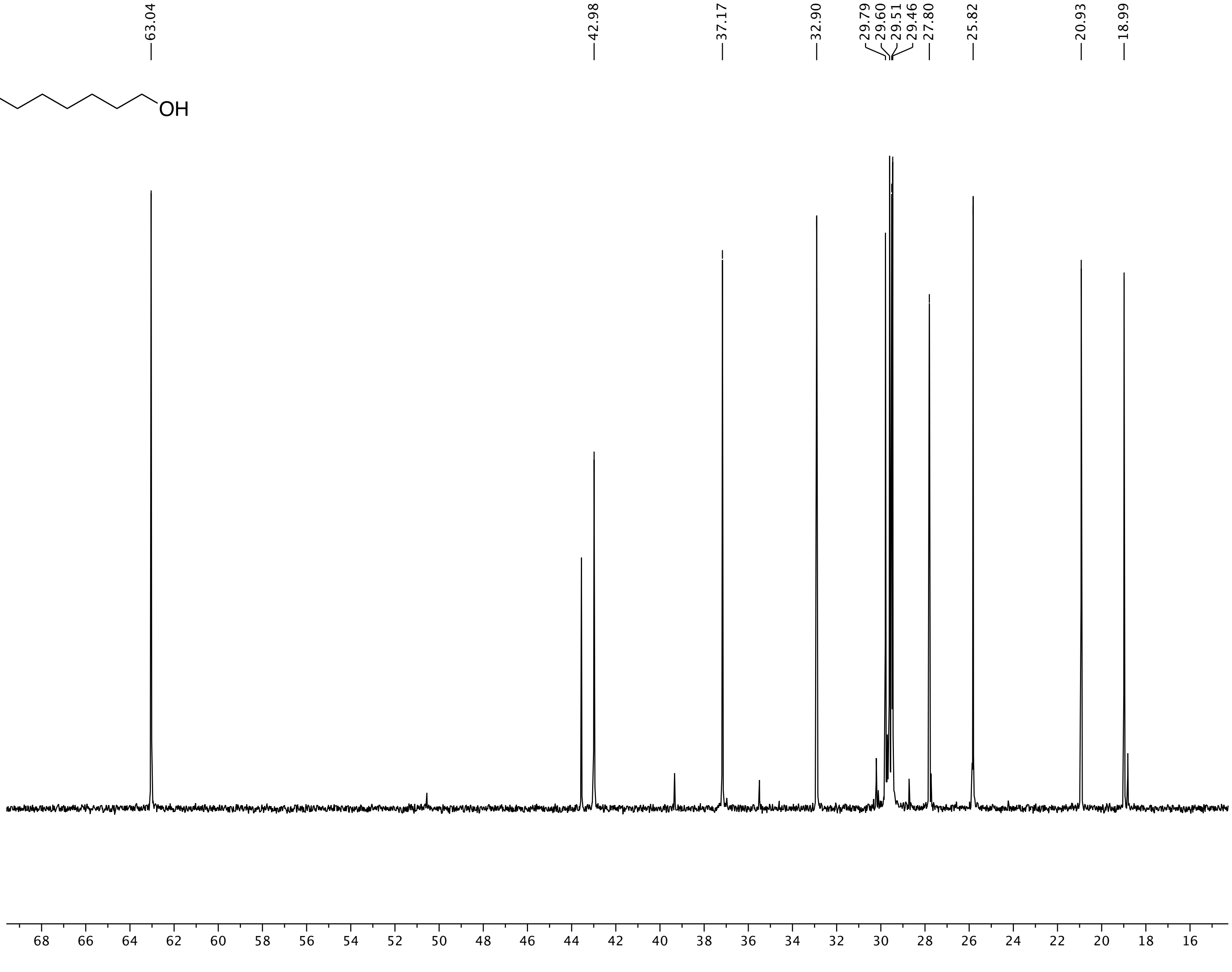

10-(4-Methylquinolin-2-yl)undecan-1-ol (minor)

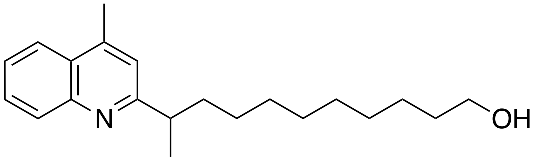

DEPT-135 (101 MHz, $CDCl_3$)

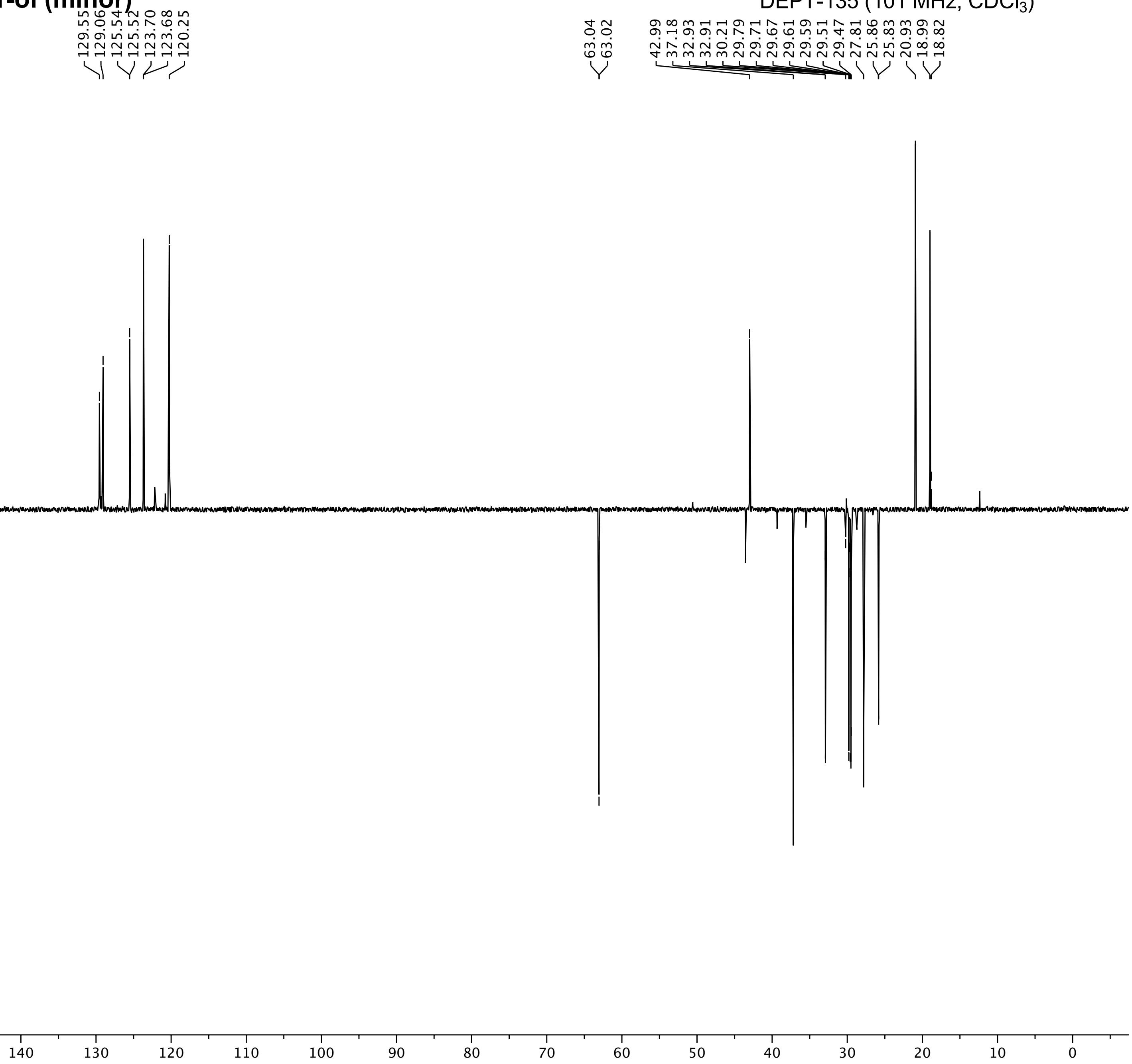

10-(4-Methylquinolin-2-yl)undecan-1-ol (minor)

DEPT-135 (101 MHz, $CDCl_3$)

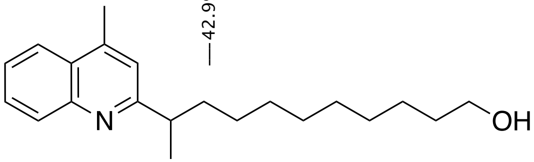

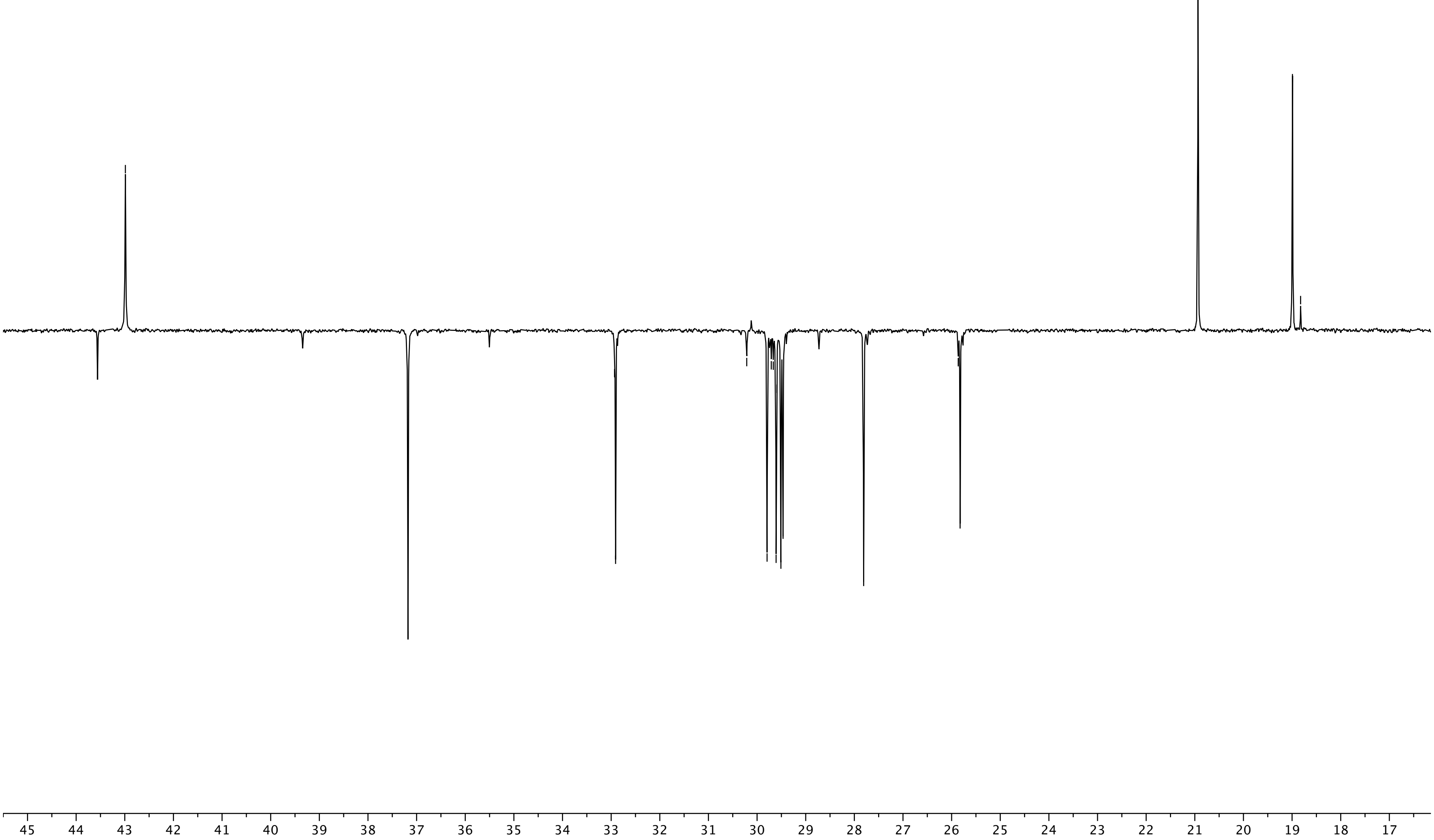

10-(4-Methylquinolin-2-yl)undecan-1-ol (minor)

^1H-^1H COSY (400 MHz, $CDCl_3$)

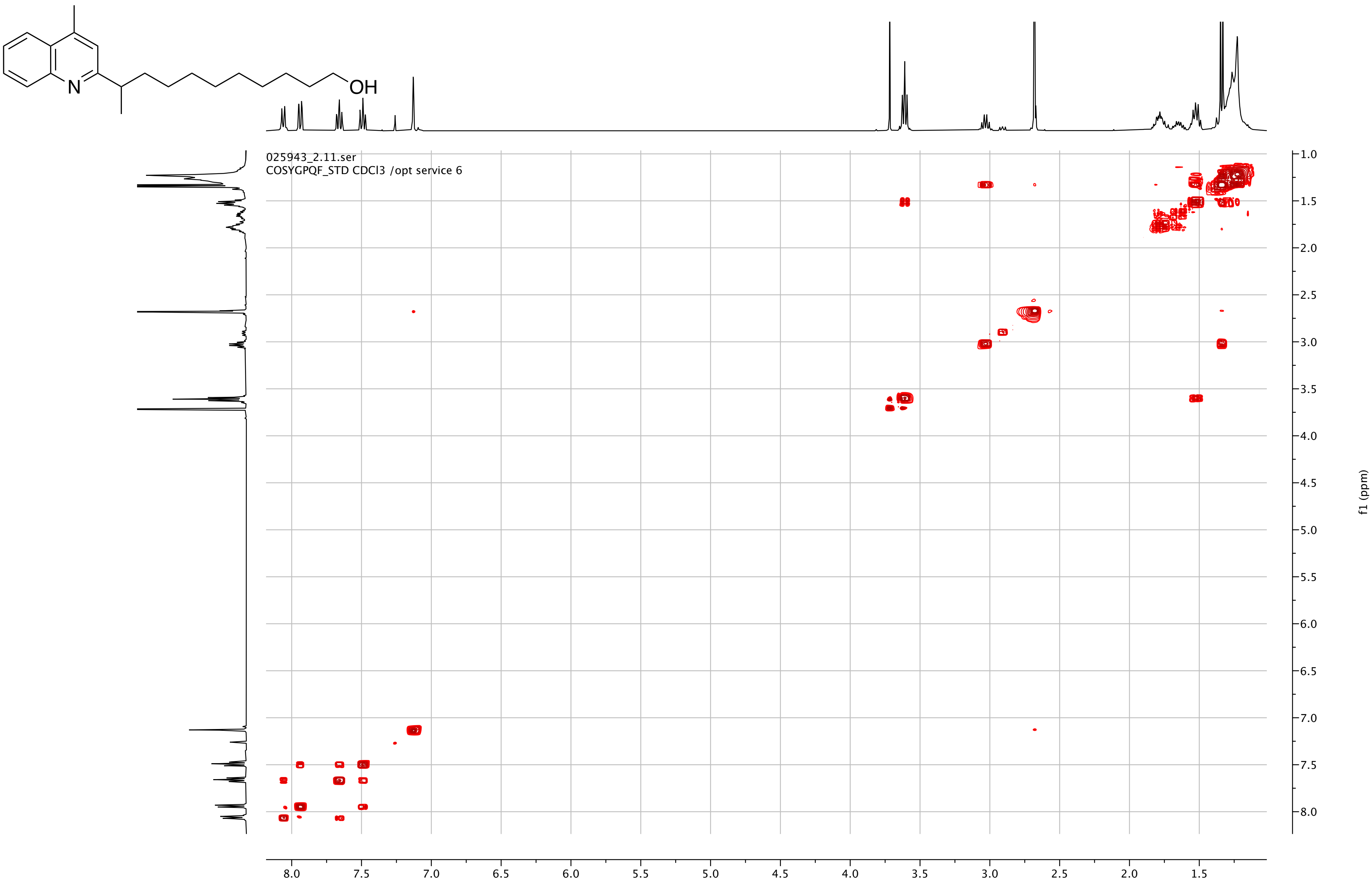

10-(4-Methylquinolin-2-yl)undecan-1-ol (minor)

^{1}H-^{1}H COSY (400 MHz, $CDCl_3$)

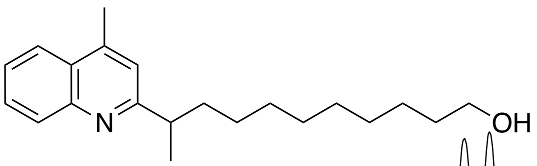

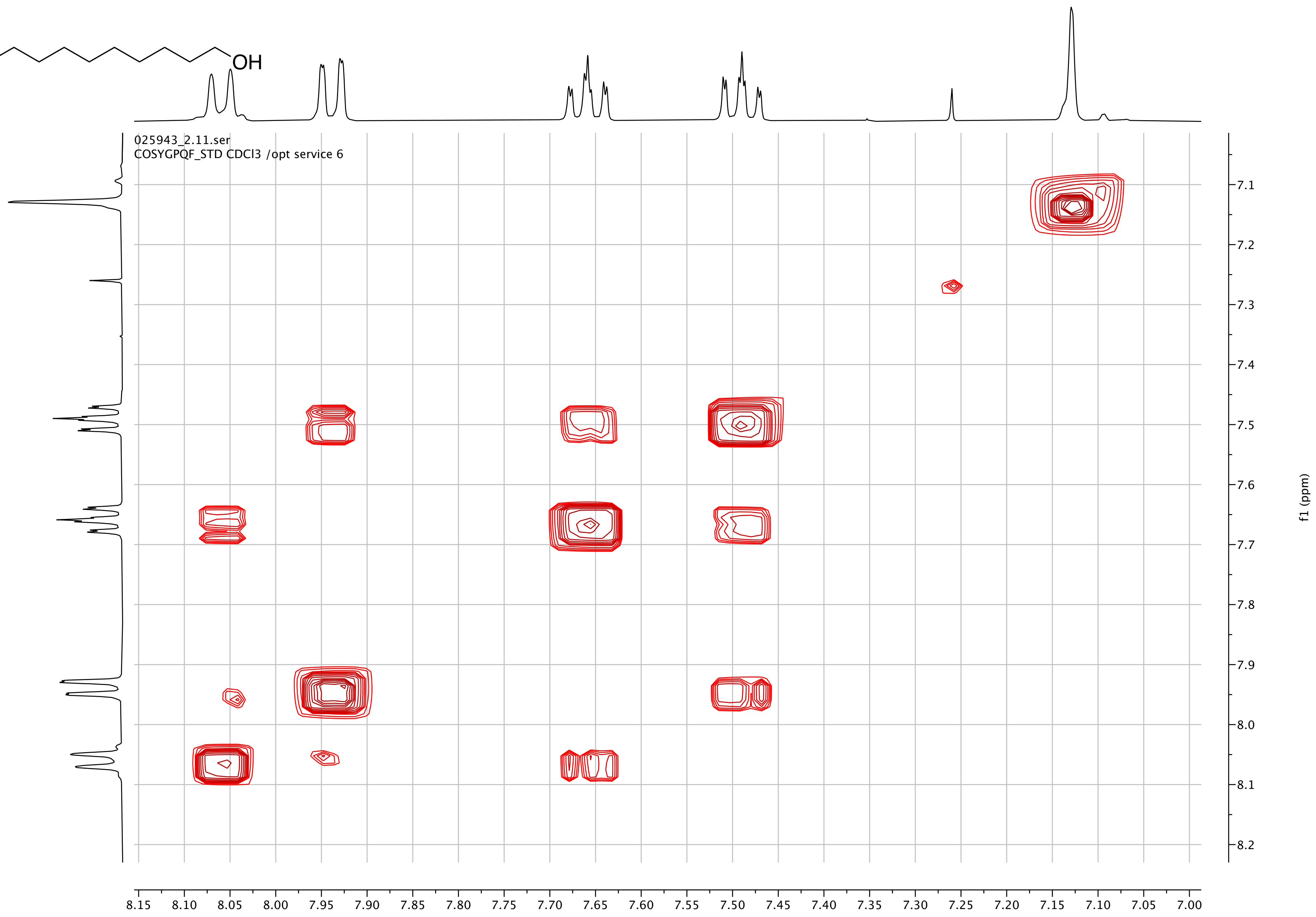

10-(4-Methylquinolin-2-yl)undecan-1-ol (minor)

^1H-^1H COSY (400 MHz, $CDCl_3$)

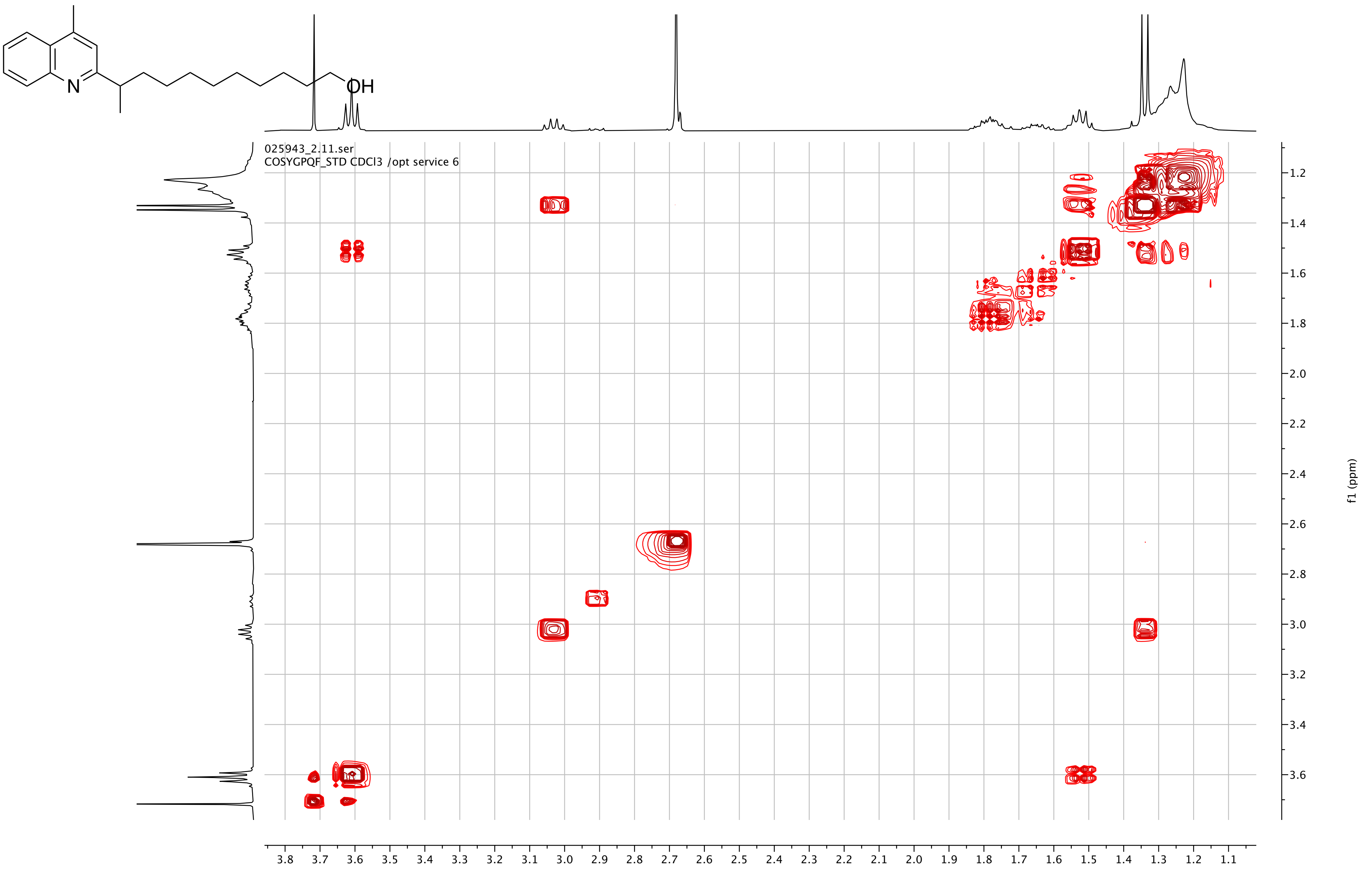

10-(4-Methylquinolin-2-yl)undecan-1-ol (minor)

HSQC-EDT (400 MHz, $CDCl_3$)

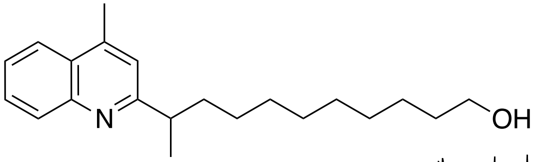

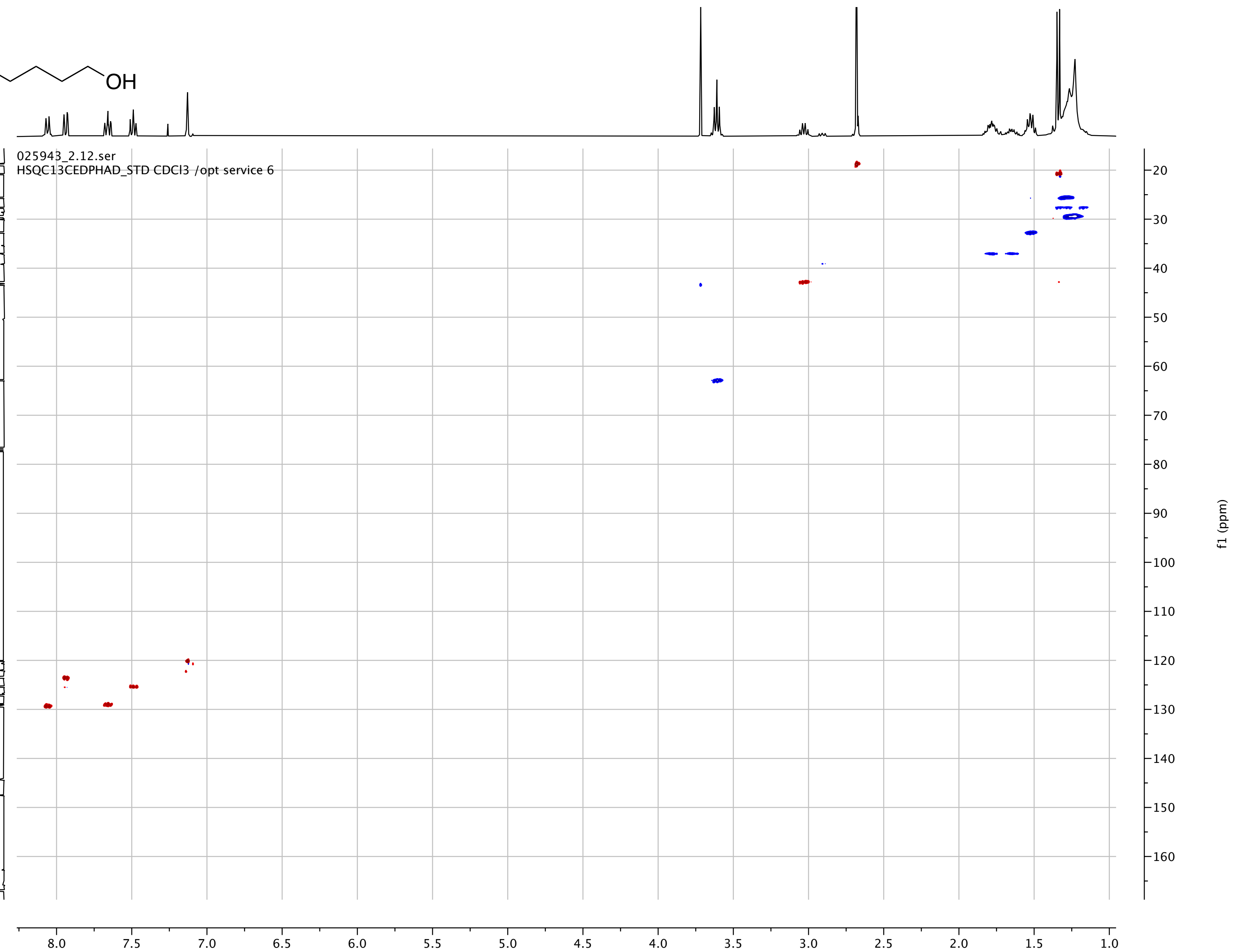

10-(4-Methylquinolin-2-yl)undecan-1-ol (minor)

HSQC-EDT (400 MHz, $CDCl_3$)

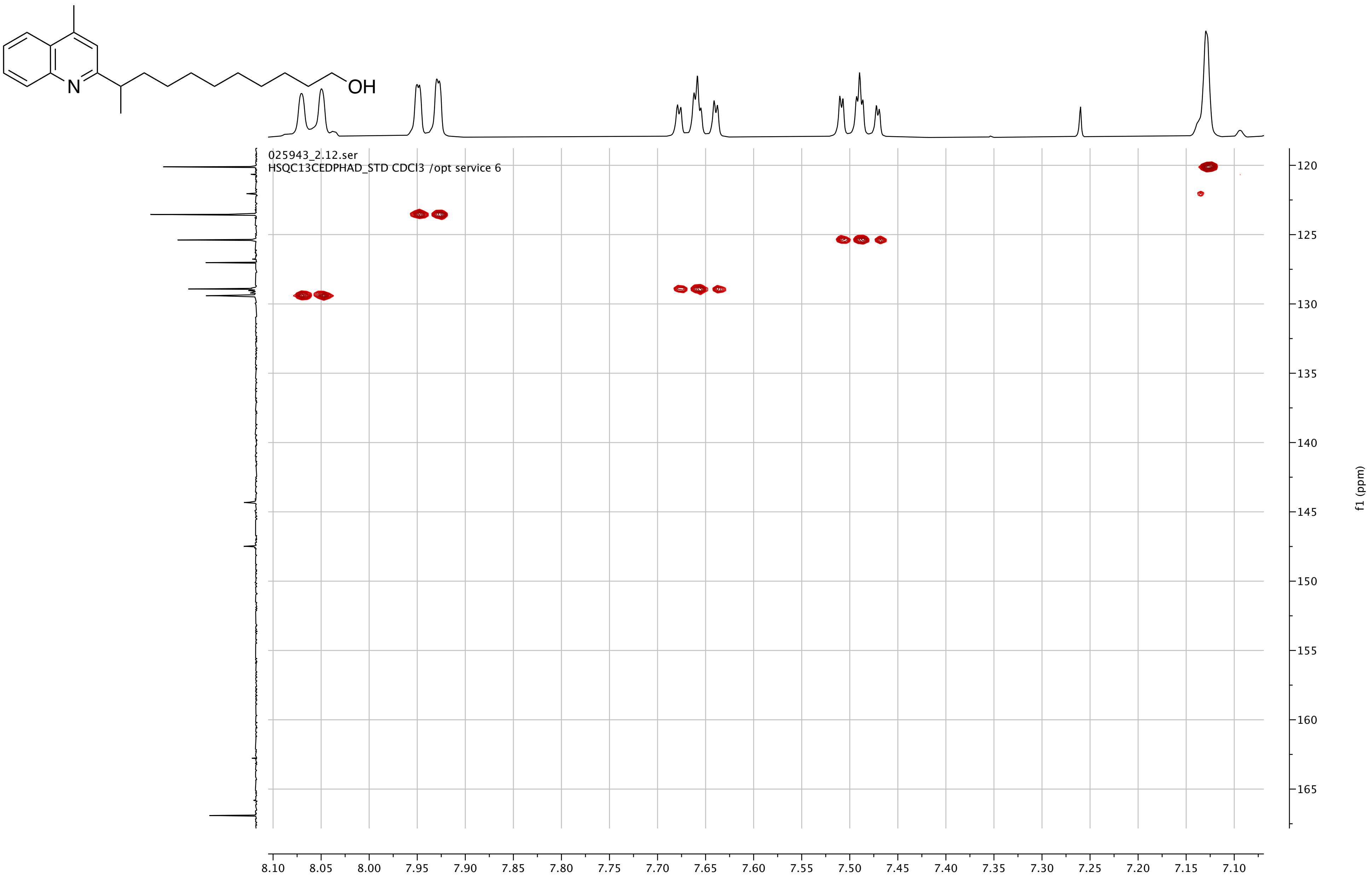

10-(4-Methylquinolin-2-yl)undecan-1-ol (minor)

HSQC-EDT (400 MHz, $CDCl_3$)

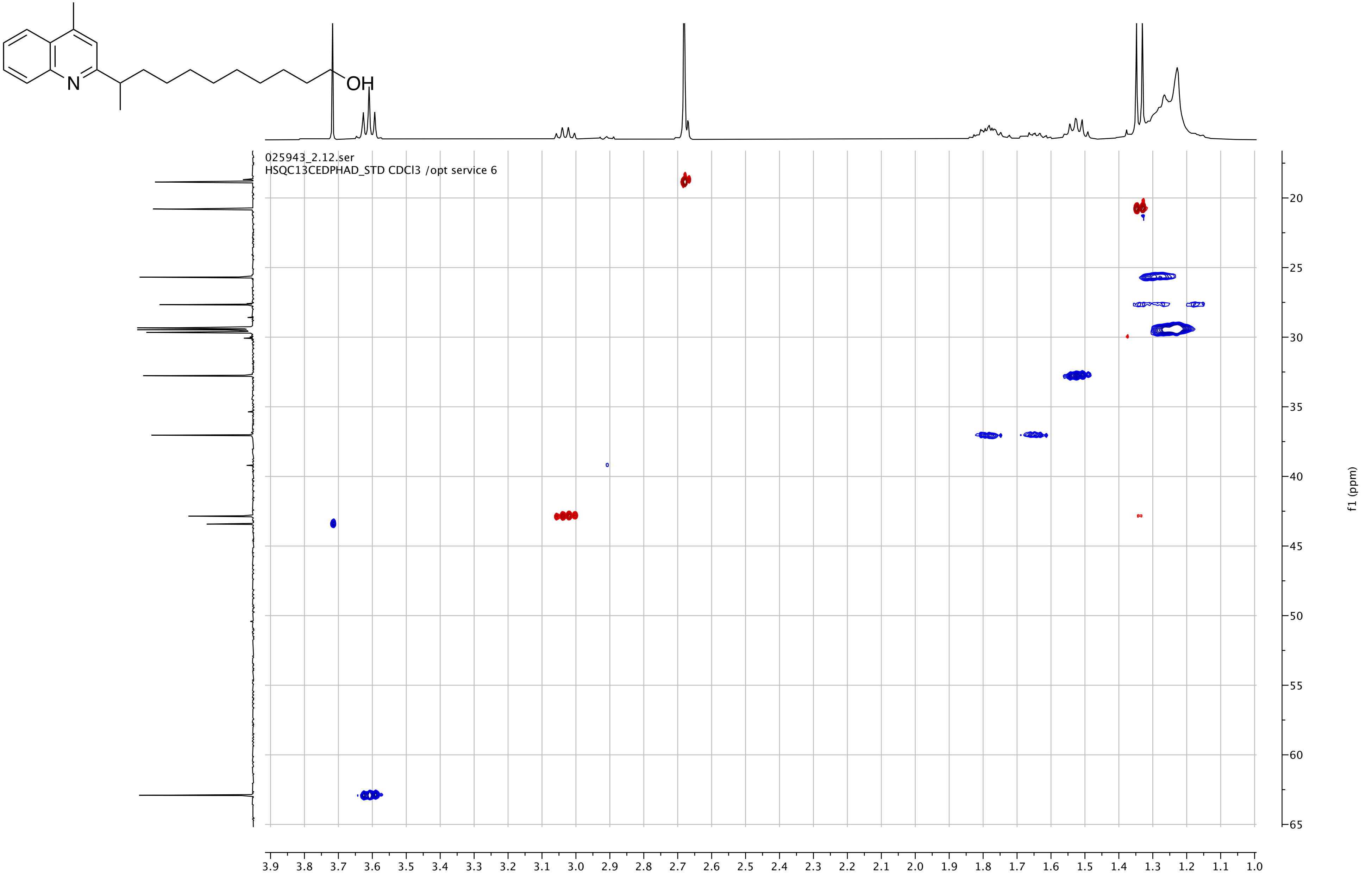

2-(3-((*tert*-Butyldiphenylsilyl)oxy)-2-methylpropyl)-4-methylquinoline (11)

^{1}H-NMR (400 MHz, $CDCl_3$)

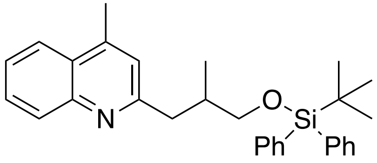

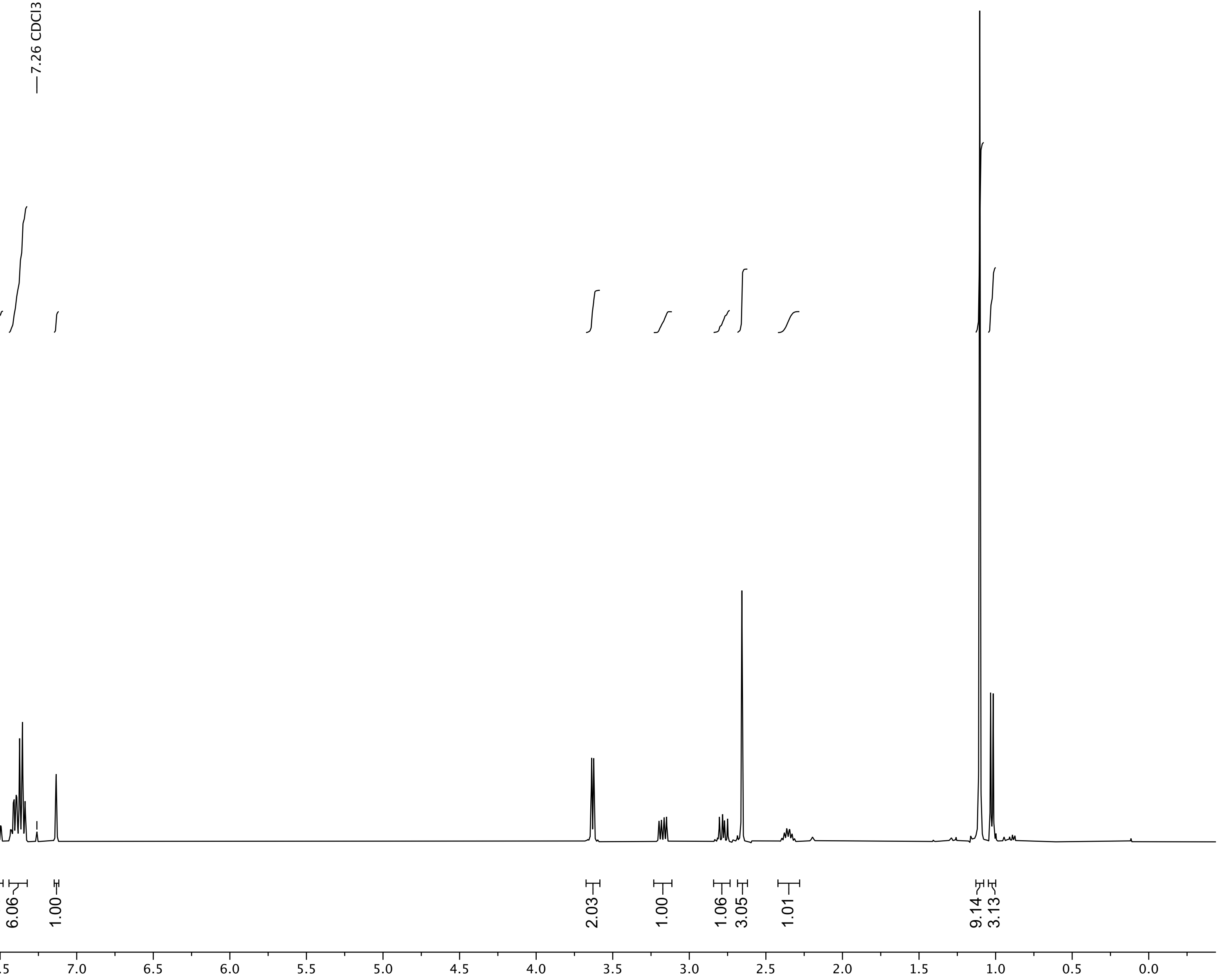

2-(3-((*tert*-Butyldiphenylsilyl)oxy)-2-methylpropyl)-4-methylquinoline (11)

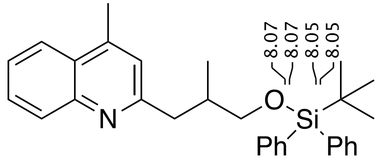

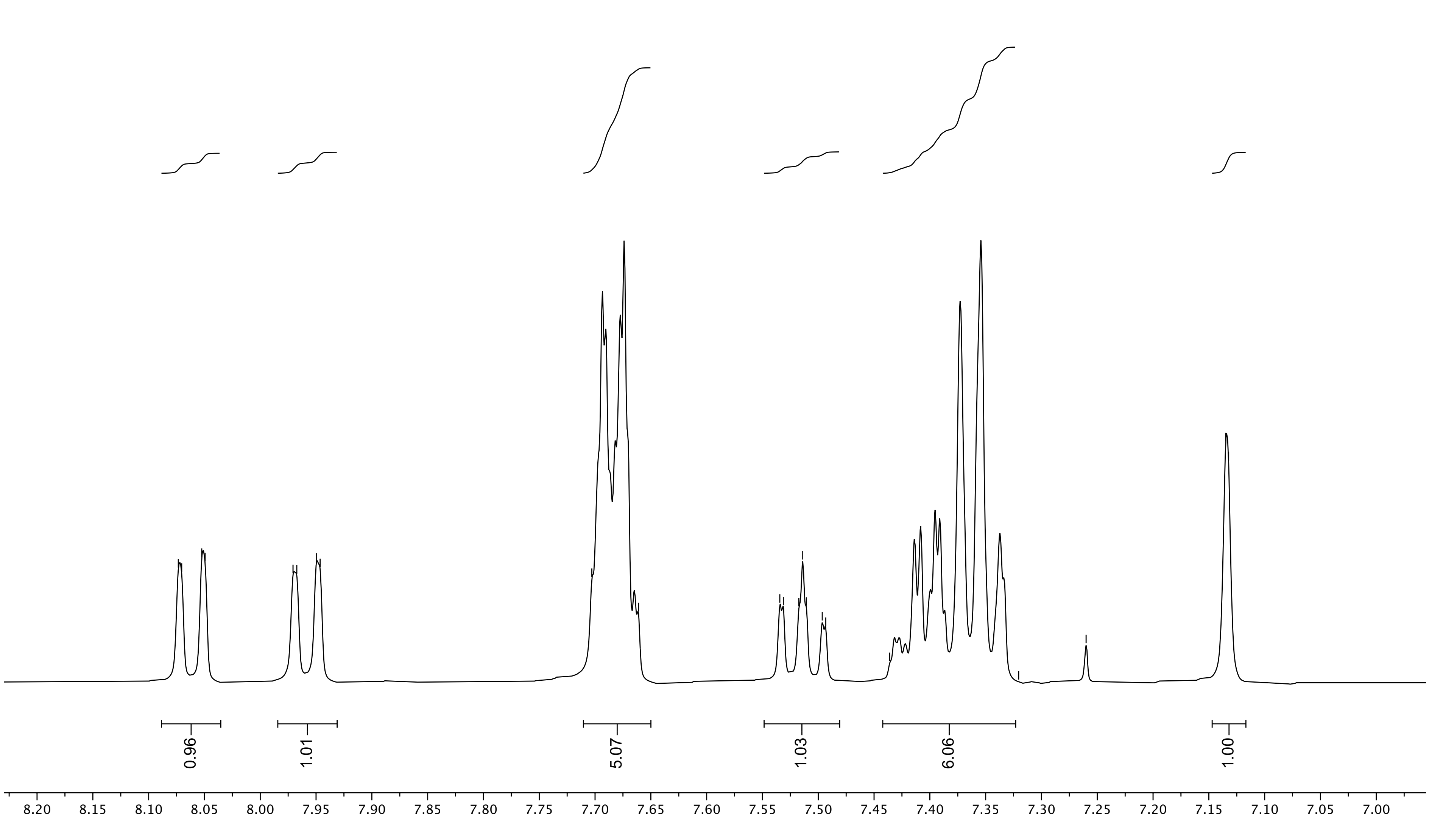

2-(3-((*tert*-Butyldiphenylsilyl)oxy)-2-methylpropyl)-4-methylquinoline (11)

^{1}H-NMR (400 MHz, $CDCl_3$)

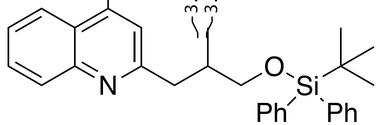

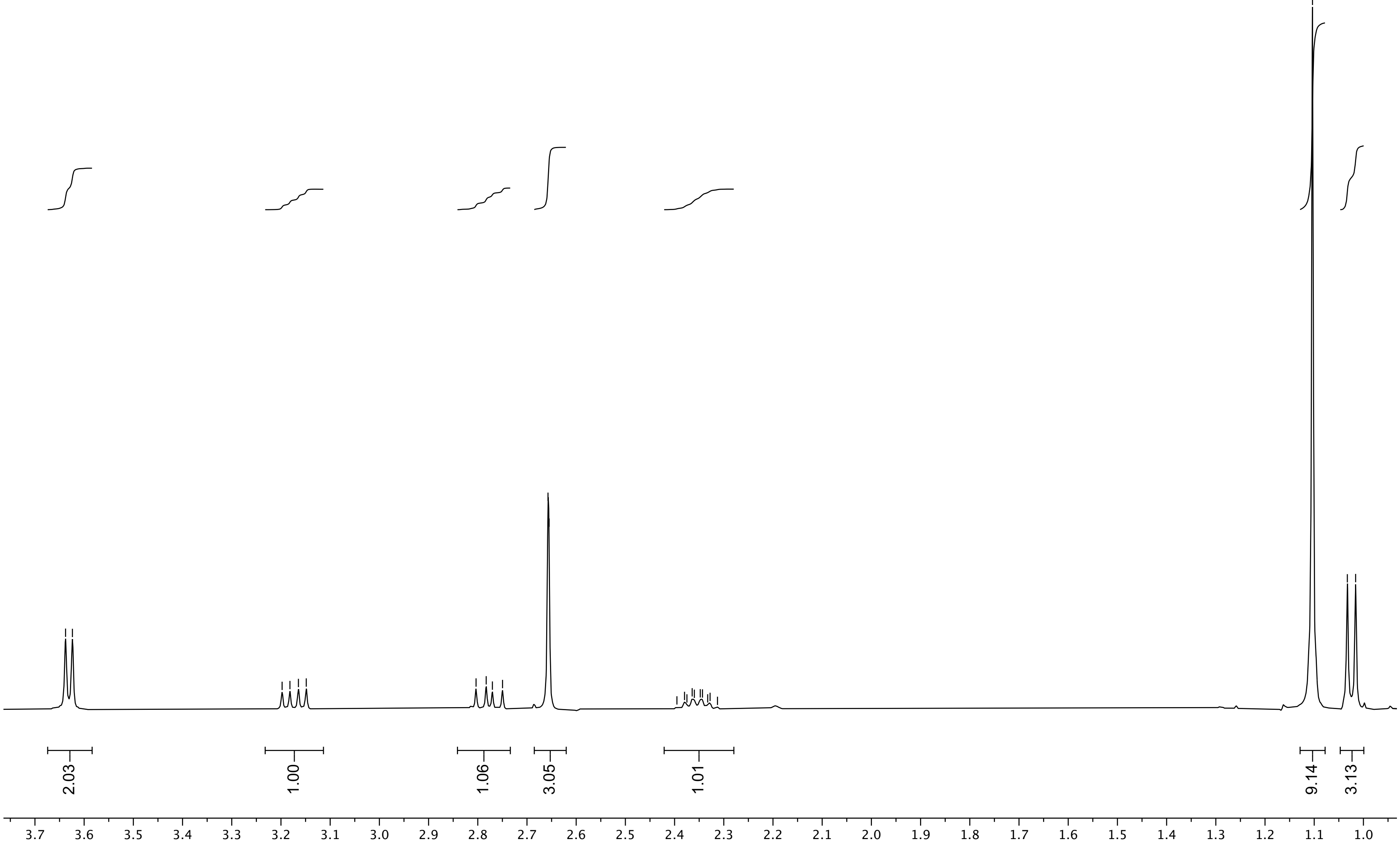

2-(3-((*tert*-Butyldiphenylsilyl)oxy)-2-methylpropyl)-4-methylquinoline (11)

^{13}C-NMR (101 MHz, $CDCl_3$)

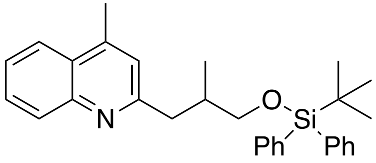

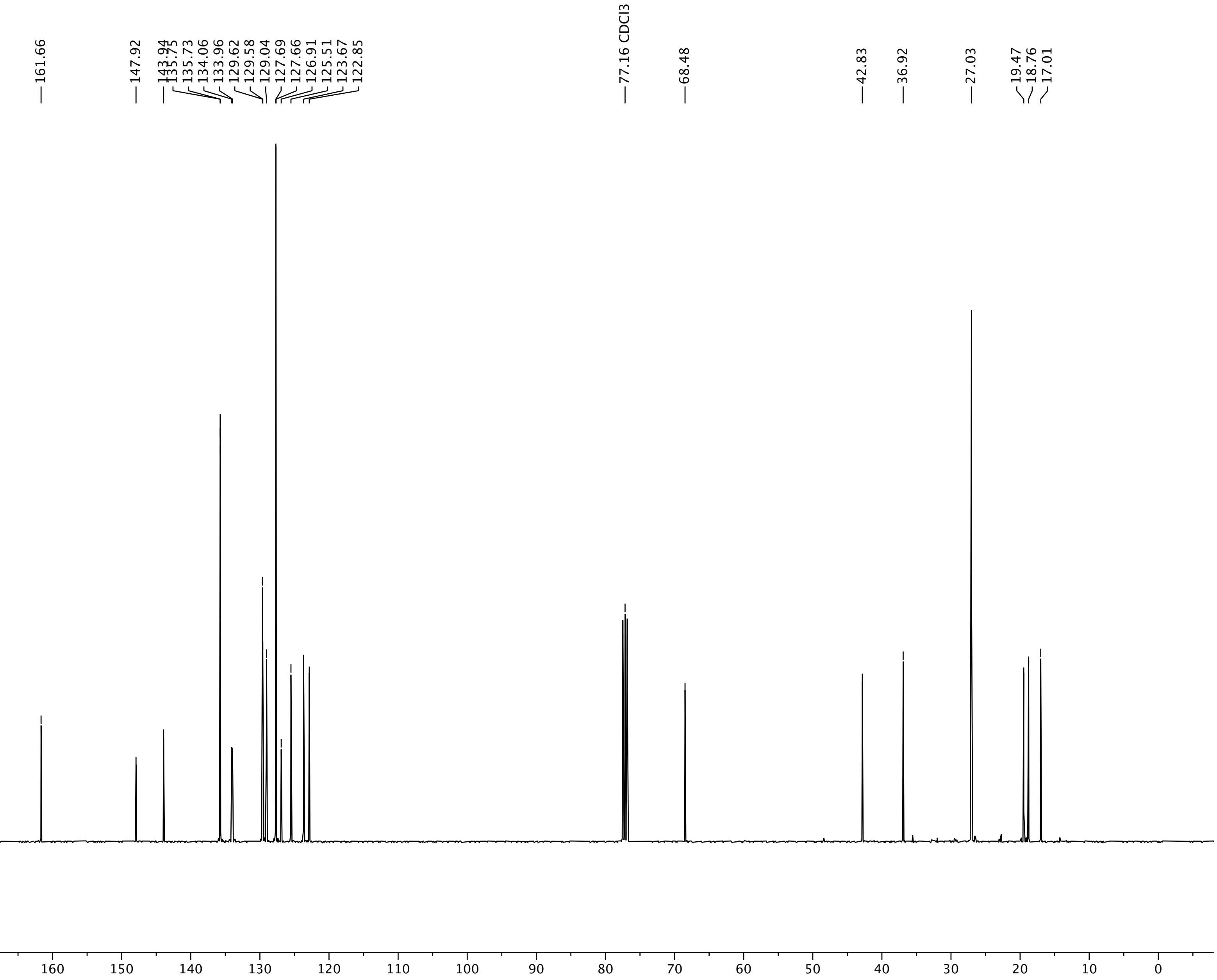

2-(3-((*tert*-Butyldiphenylsilyl)oxy)-2-methylpropyl)-4-methylquinoline (11)

^{1}H-NMR (400 MHz, $CDCl_3$)

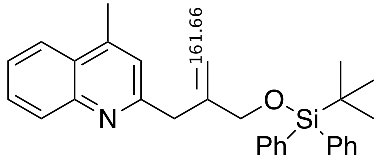

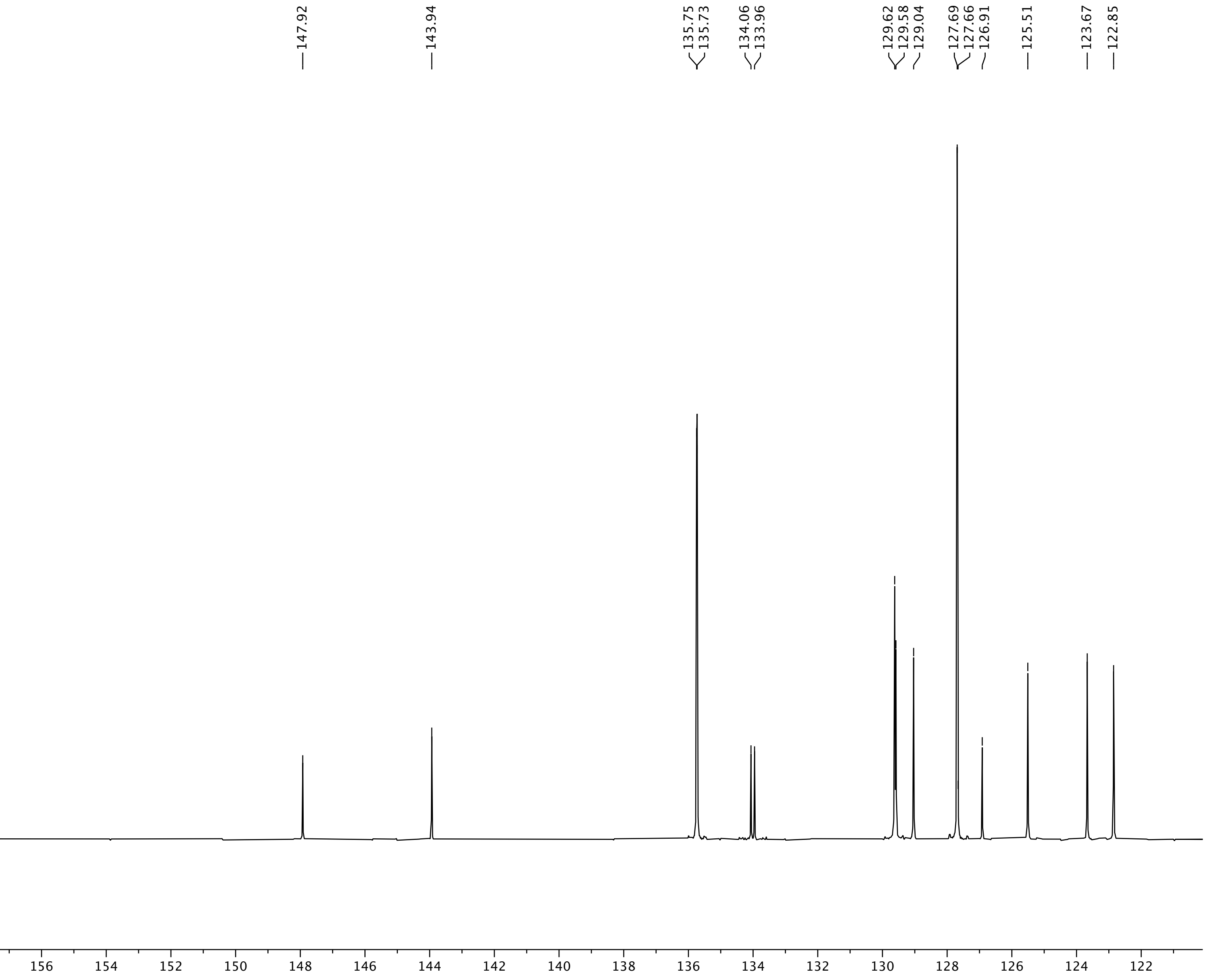

2-(3-((*tert*-Butyldiphenylsilyl)oxy)-2-methylpropyl)-4-methylquinoline (11)

^{13}C-NMR (101 MHz, $CDCl_3$)

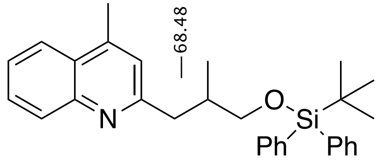

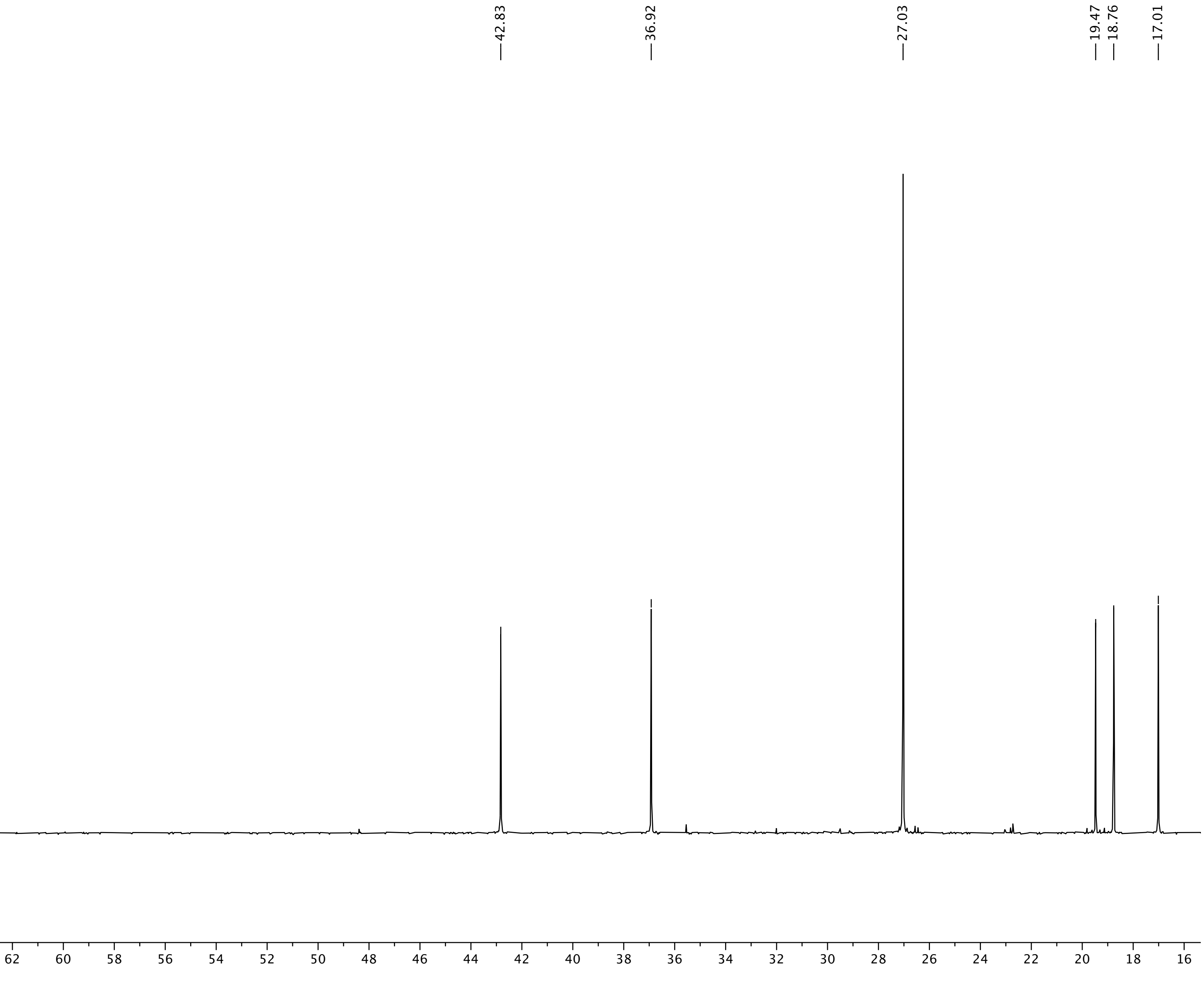

2-(3-((*tert*-Butyldiphenylsilyl)oxy)-2-methylpropyl)-4-methylquinoline (11)

Dept-135 (101 MHz, $CDCl_3$)

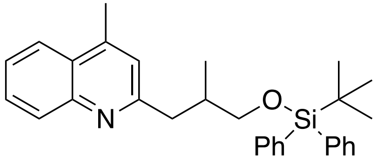

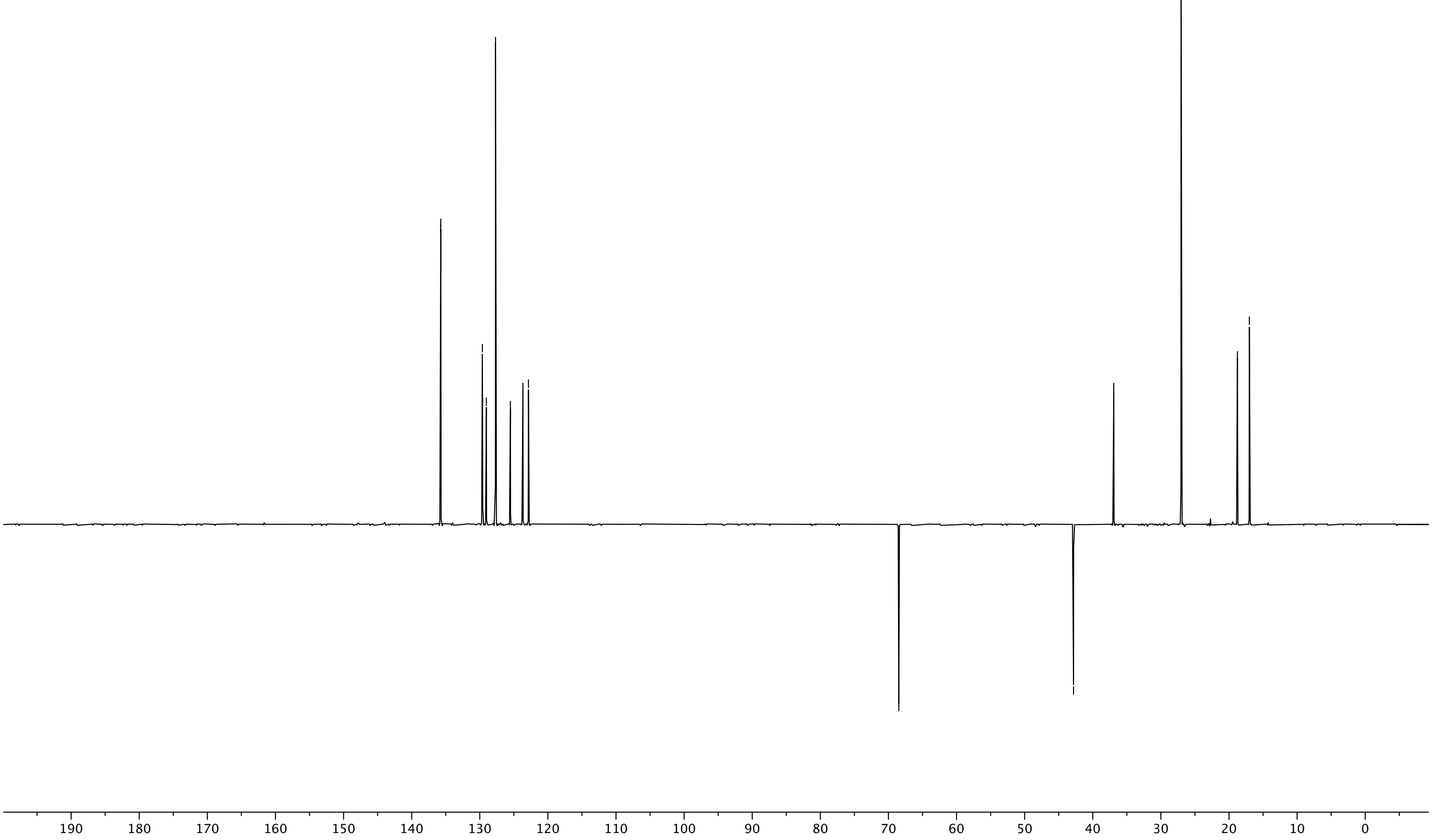

2-(3-((*tert*-Butyldiphenylsilyl)oxy)-2-methylpropyl)-4-methylquinoline (11)

Dept-135 (101 MHz, $CDCl_3$)

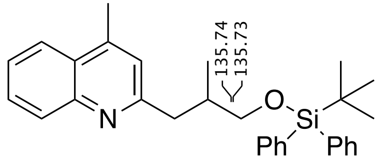

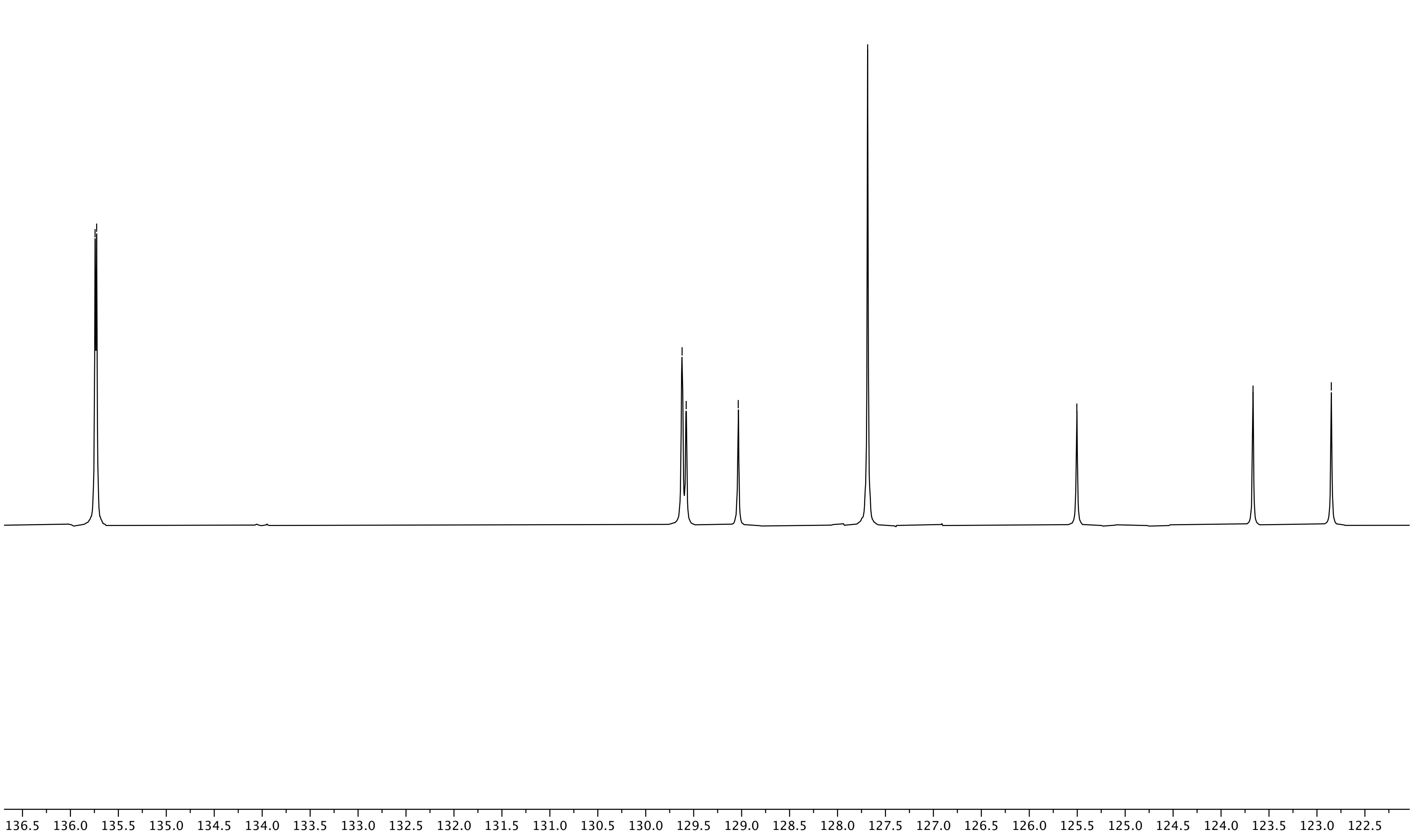

2-(3-((*tert*-Butyldiphenylsilyl)oxy)-2-methylpropyl)-4-methylquinoline (11)

^{1}H-^{1}H COSY(400 MHz, $CDCl_3$)

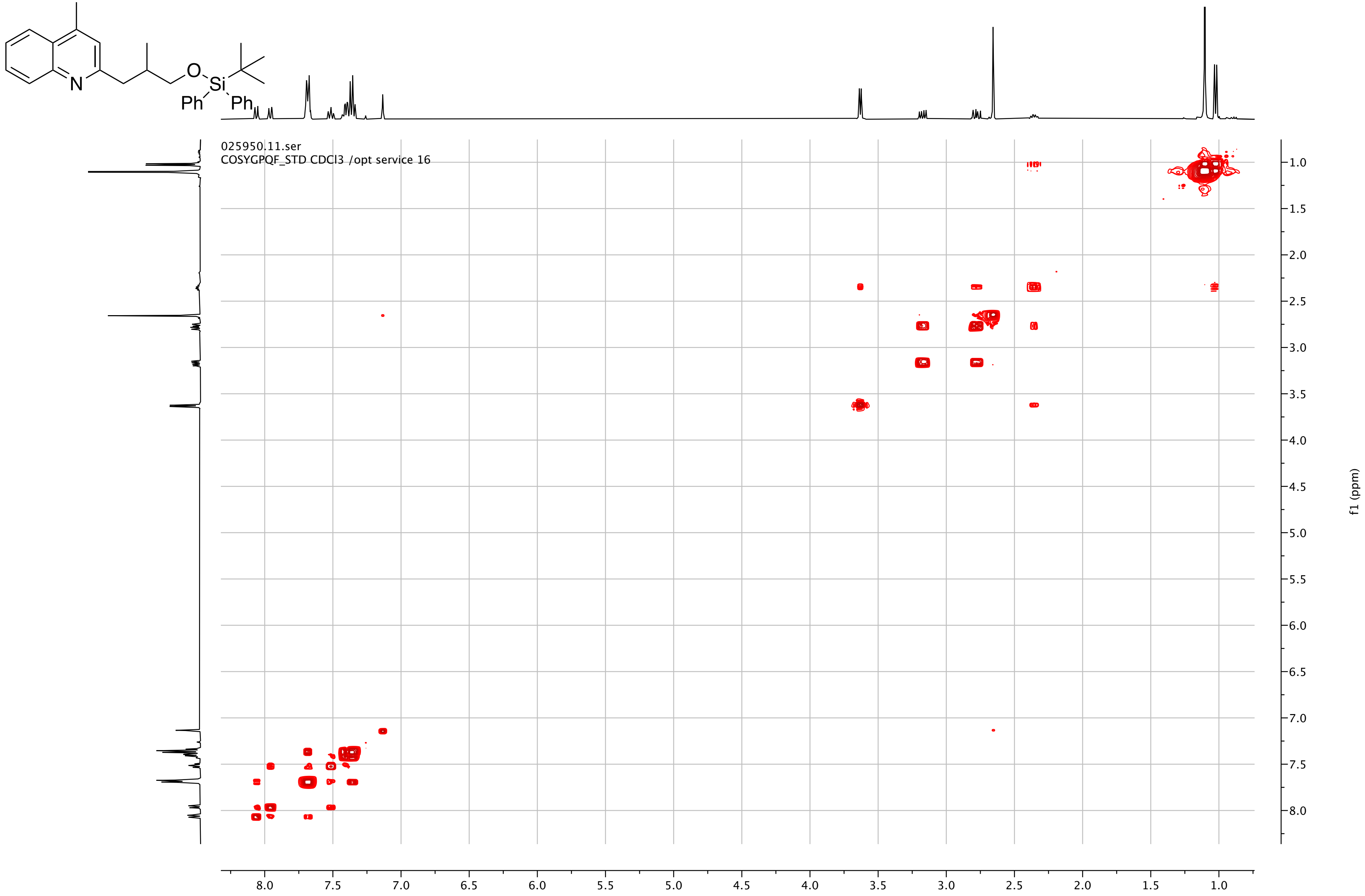

2-(3-((*tert*-Butyldiphenylsilyl)oxy)-2-methylpropyl)-4-methylquinoline (11)

^{1}H-^{1}H COSY(400 MHz, $CDCl_3$)

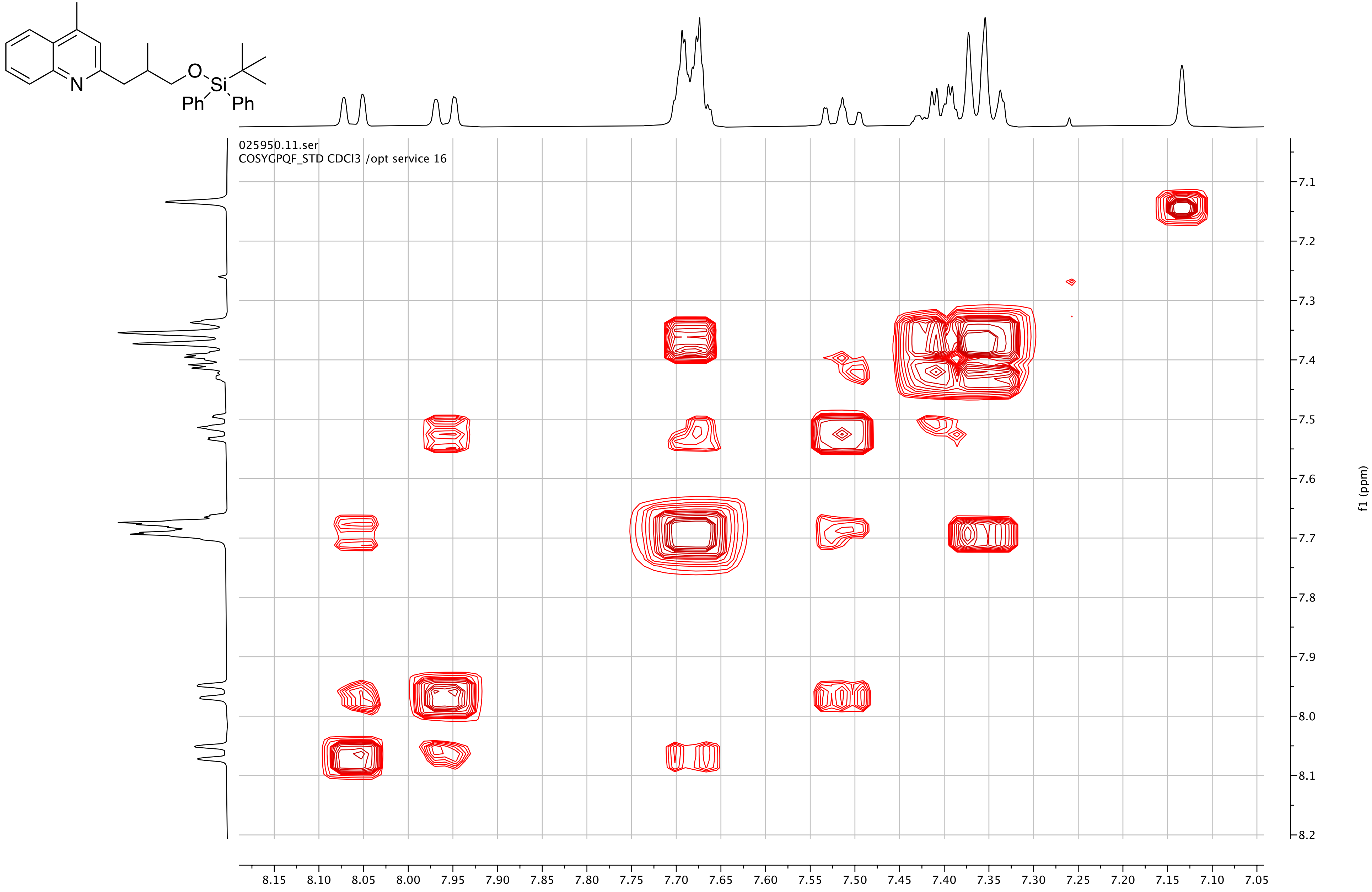

2-(3-((*tert*-Butyldiphenylsilyl)oxy)-2-methylpropyl)-4-methylquinoline (11)

^{1}H-^{1}H COSY(400 MHz, $CDCl_3$)

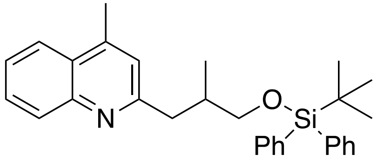

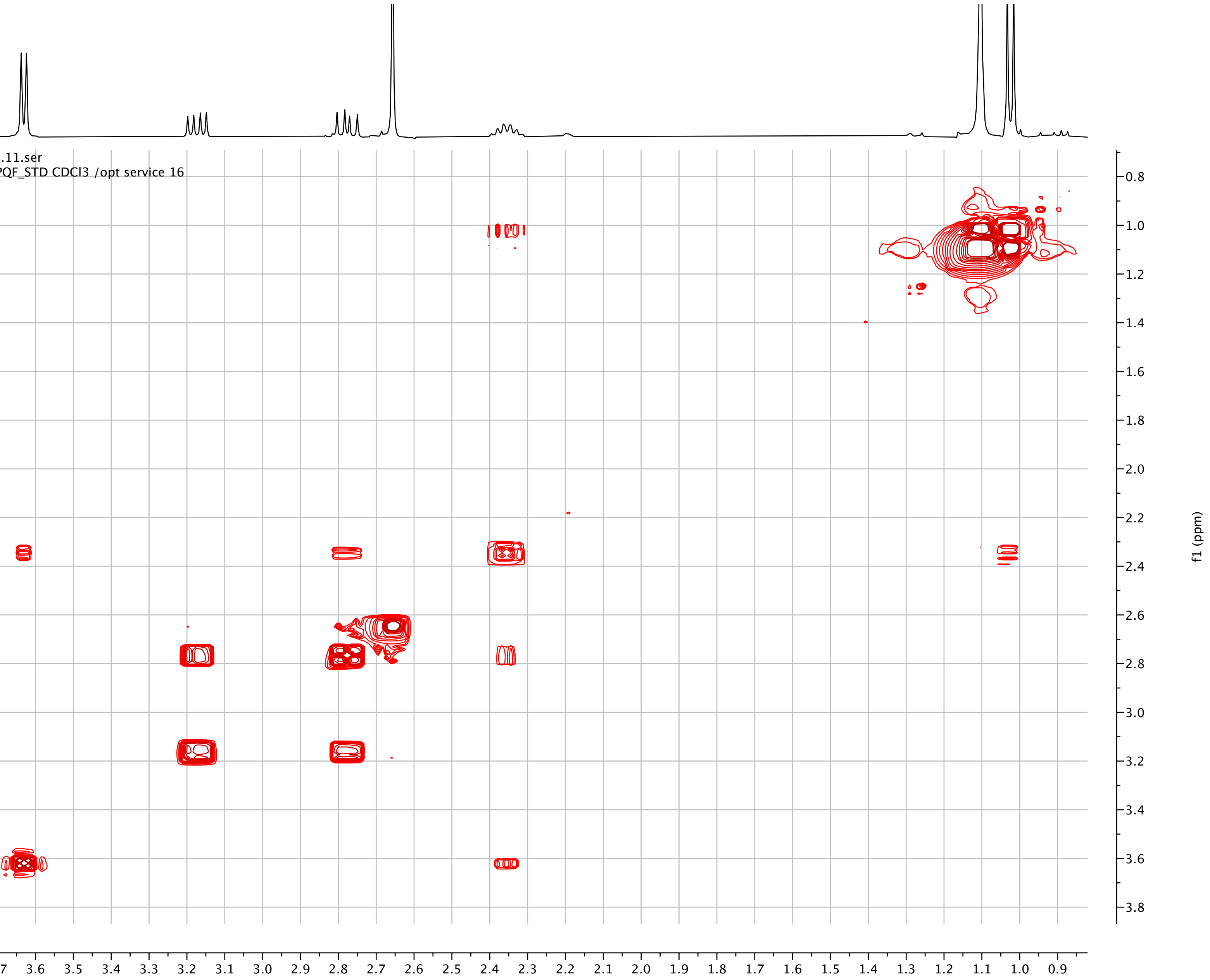

2-(3-((*tert*-Butyldiphenylsilyl)oxy)-2-methylpropyl)-4-methylquinoline (11)

HSQC (400 MHz, $CDCl_3$)

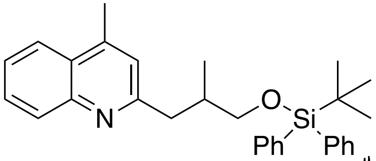

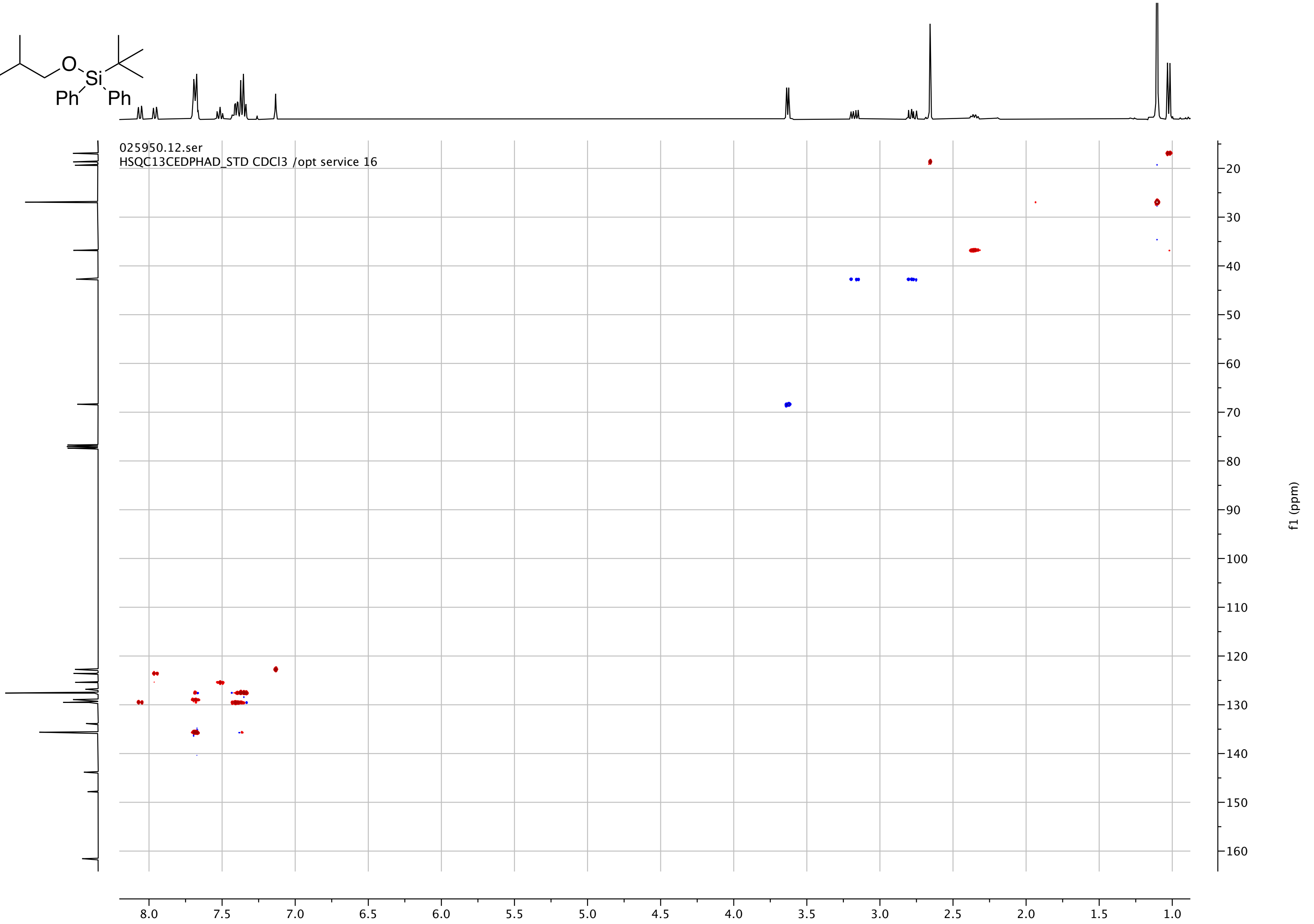

2-(3-((*tert*-Butyldiphenylsilyl)oxy)-2-methylpropyl)-4-methylquinoline (11)

HSQC (400 MHz, $CDCl_3$)

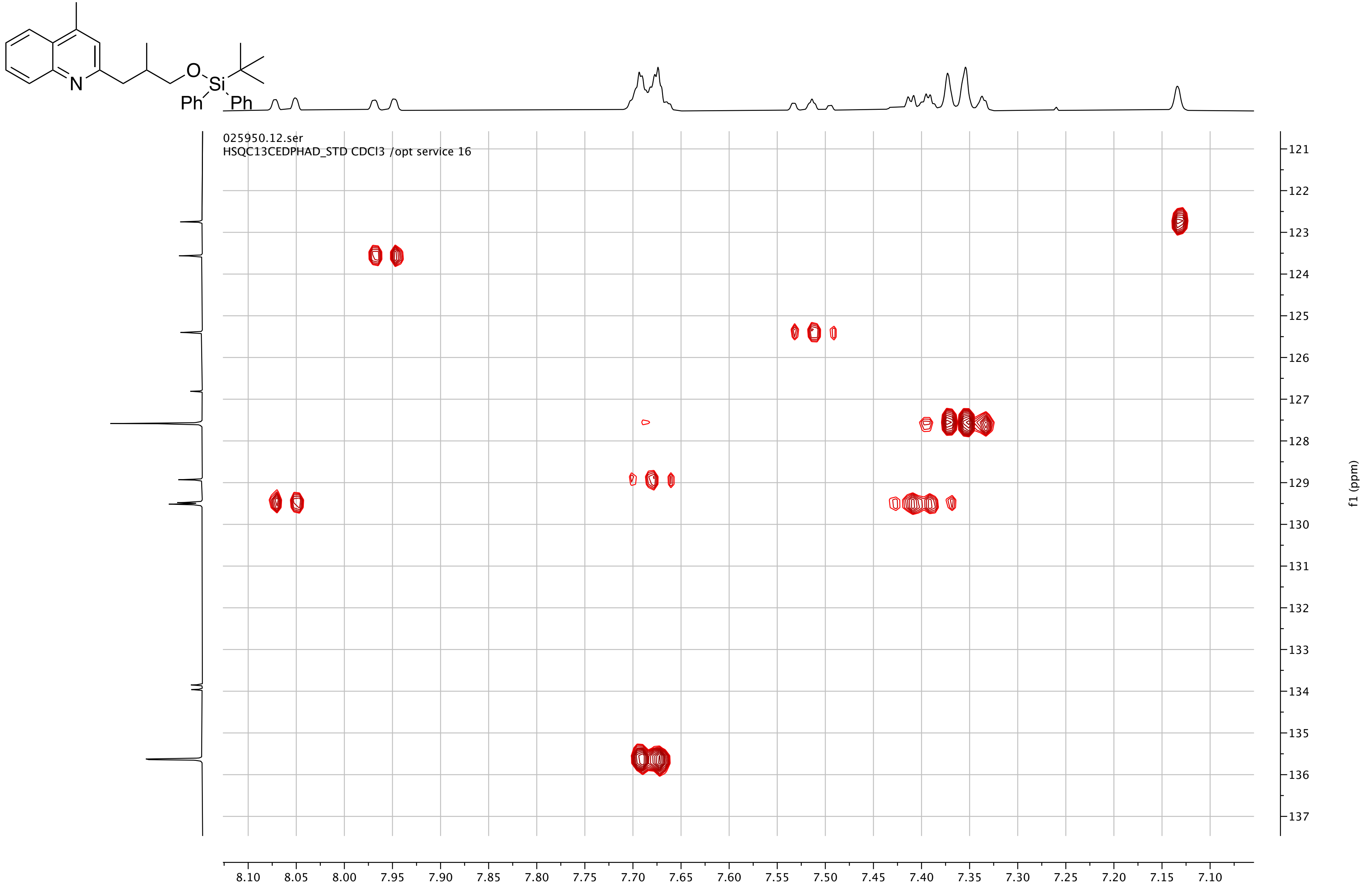

2-(3-((*tert*-Butyldiphenylsilyl)oxy)-2-methylpropyl)-4-methylquinoline (11)

HSQC (400 MHz, $CDCl_3$)

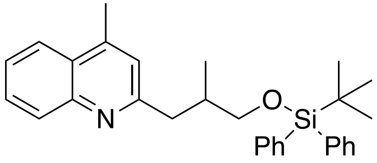

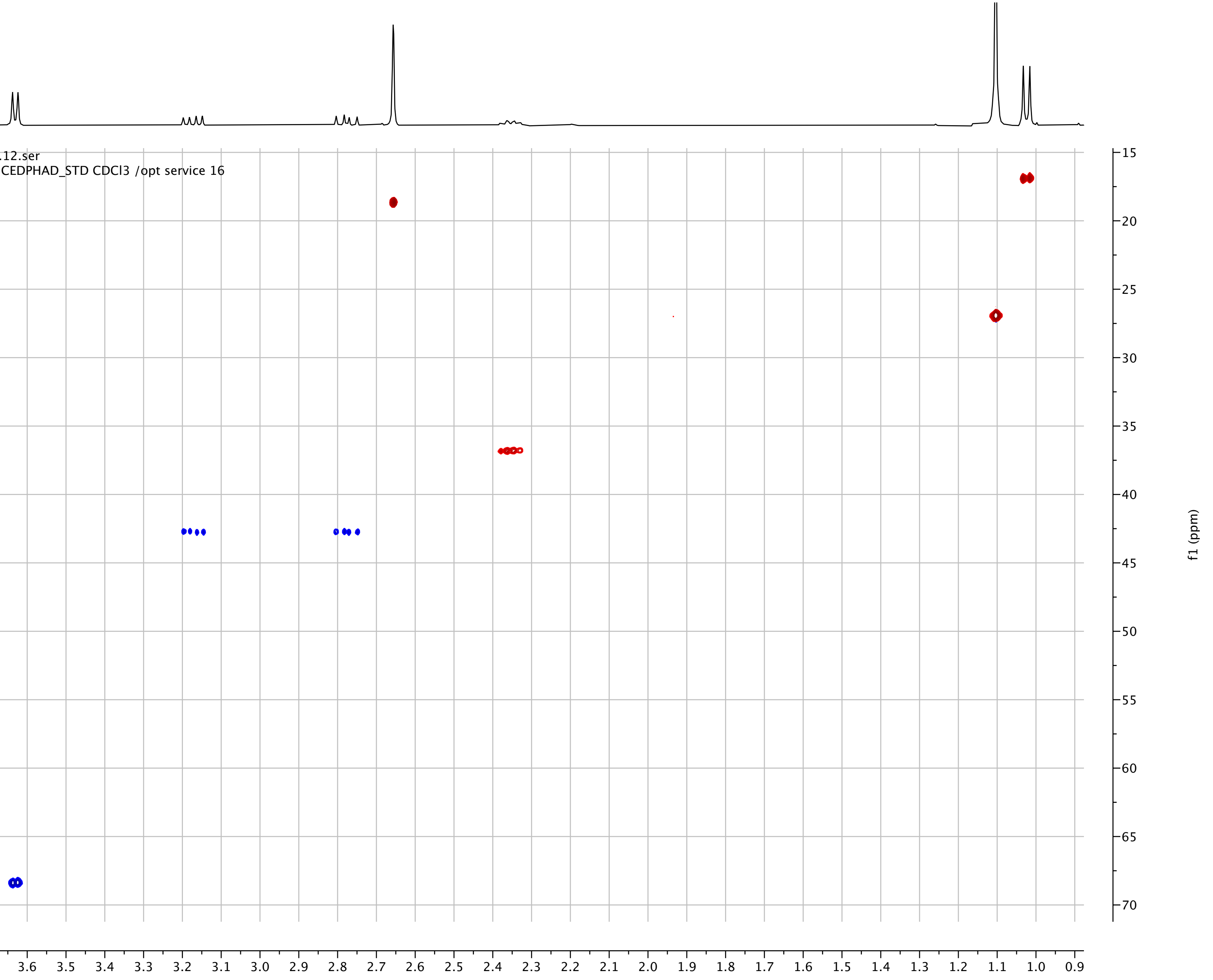

2-(3-((*tert*-Butyldiphenylsilyl)oxy)-2-methylpropyl)-4-methylquinoline (11)

HMBC (400 MHz, $CDCl_3$)

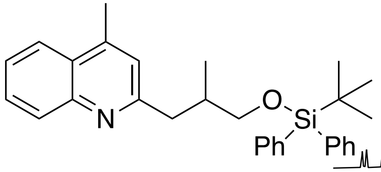

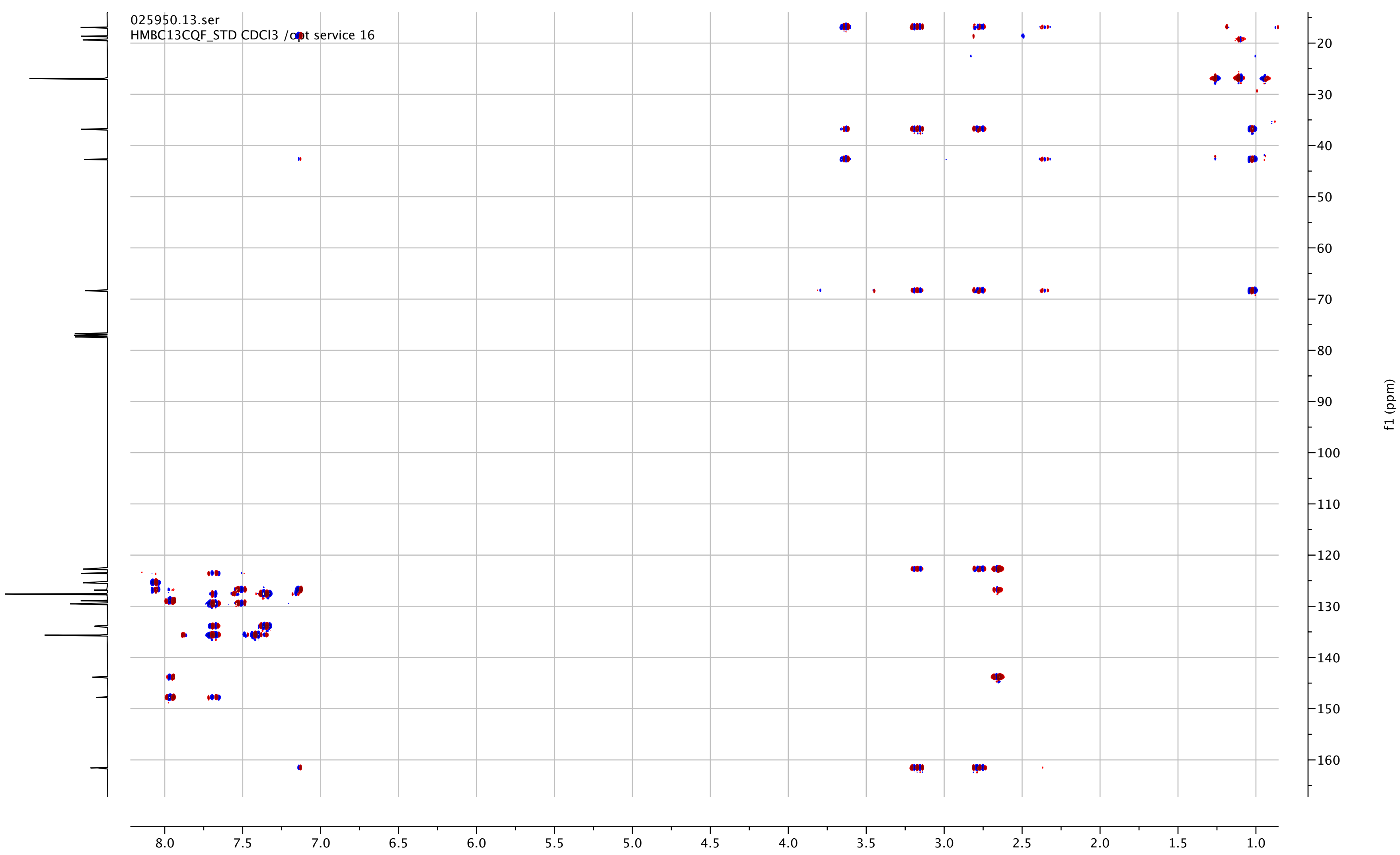

2-(3-((*tert*-Butyldiphenylsilyl)oxy)-2-methylpropyl)-4-methylquinoline (11)

HMBC (400 MHz, $CDCl_3$)

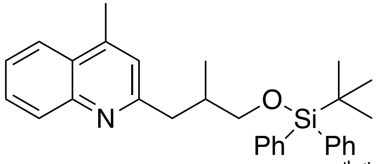

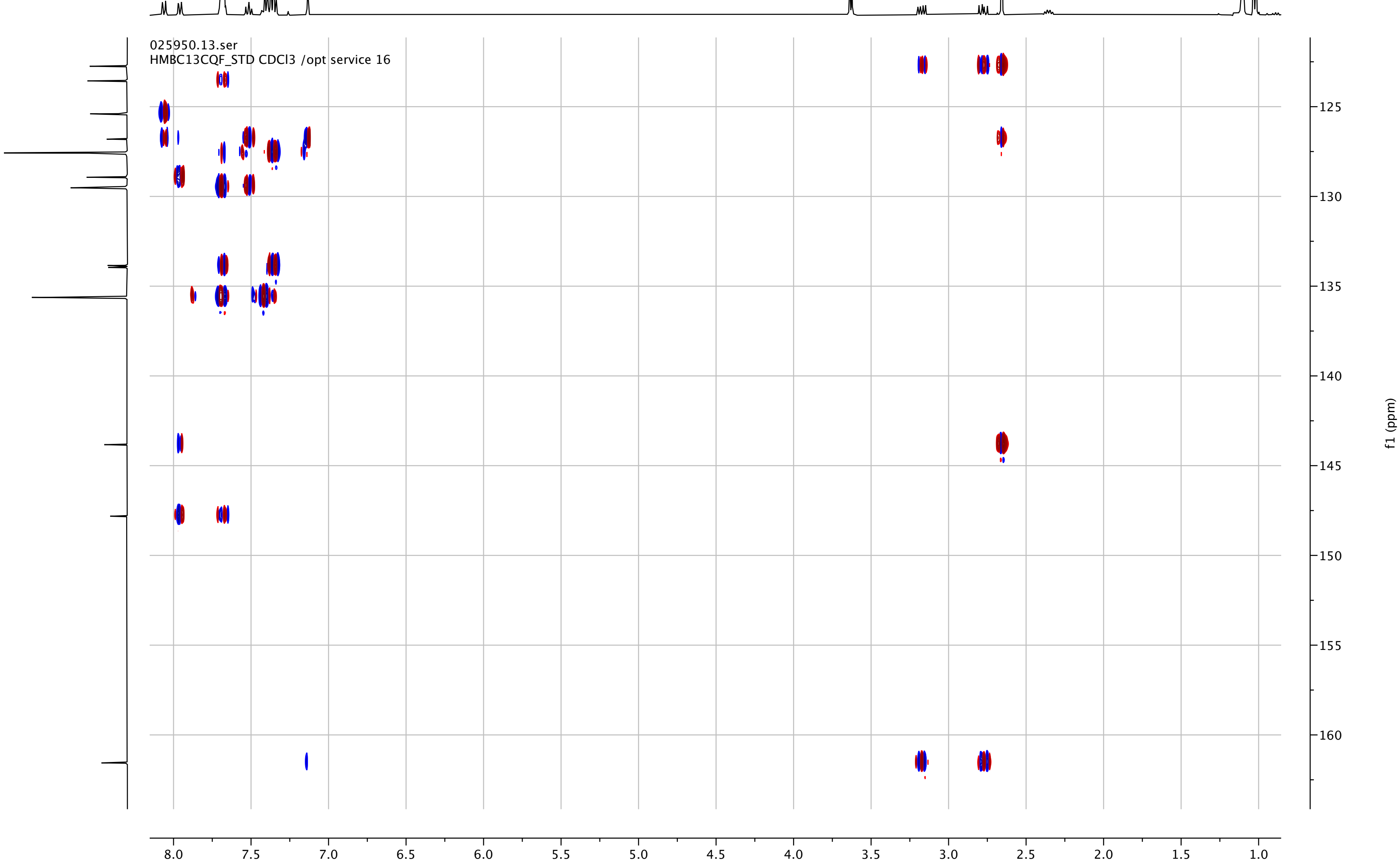

2-(3-((*tert*-Butyldiphenylsilyl)oxy)-2-methylpropyl)-4-methylquinoline (11)

HMBC (400 MHz, $CDCl_3$)

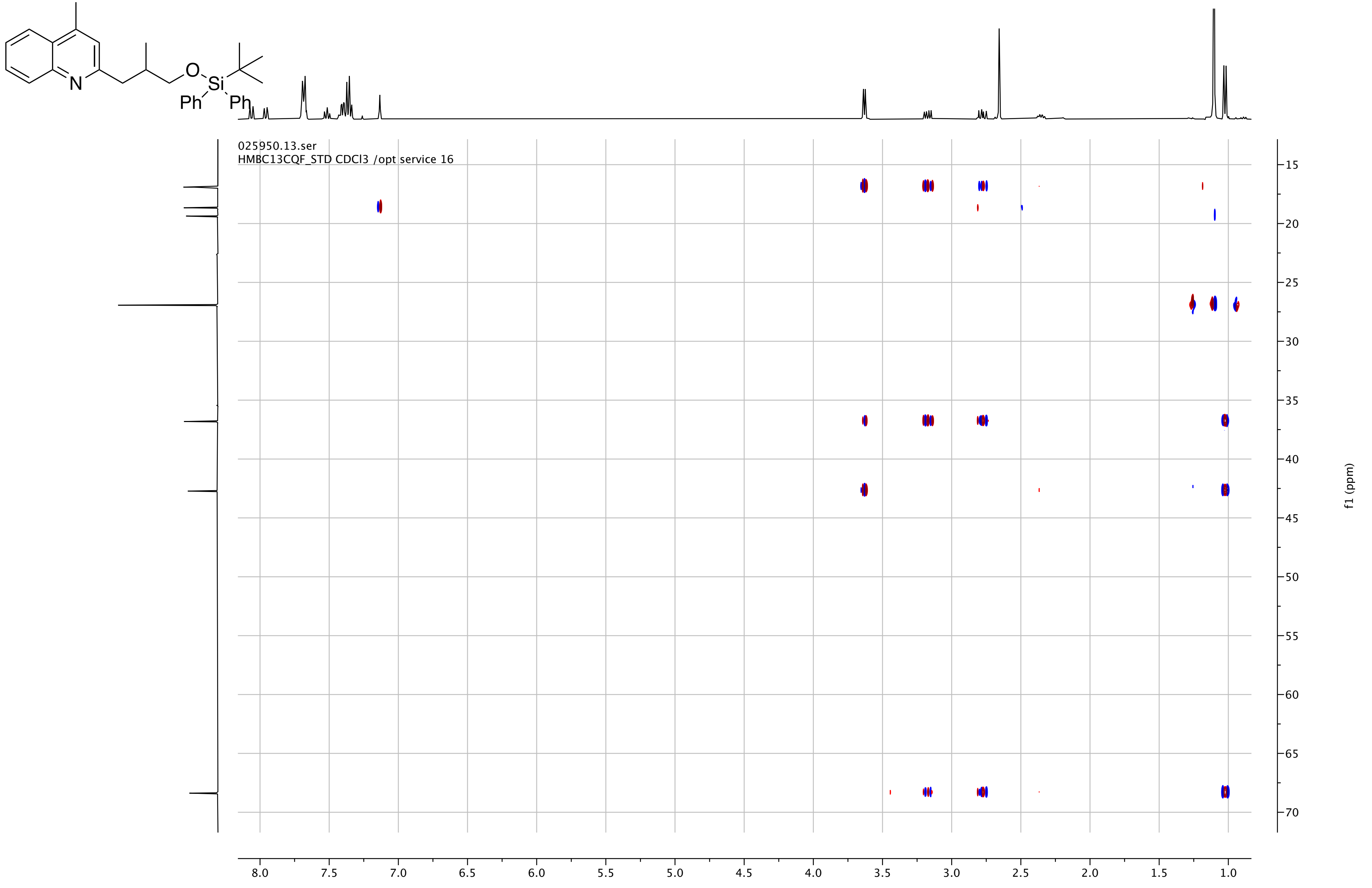

4-Chloro-2-cyclohexylquinoline (12)

^{1}H-NMR, 300 MHz, $CDCl_3$

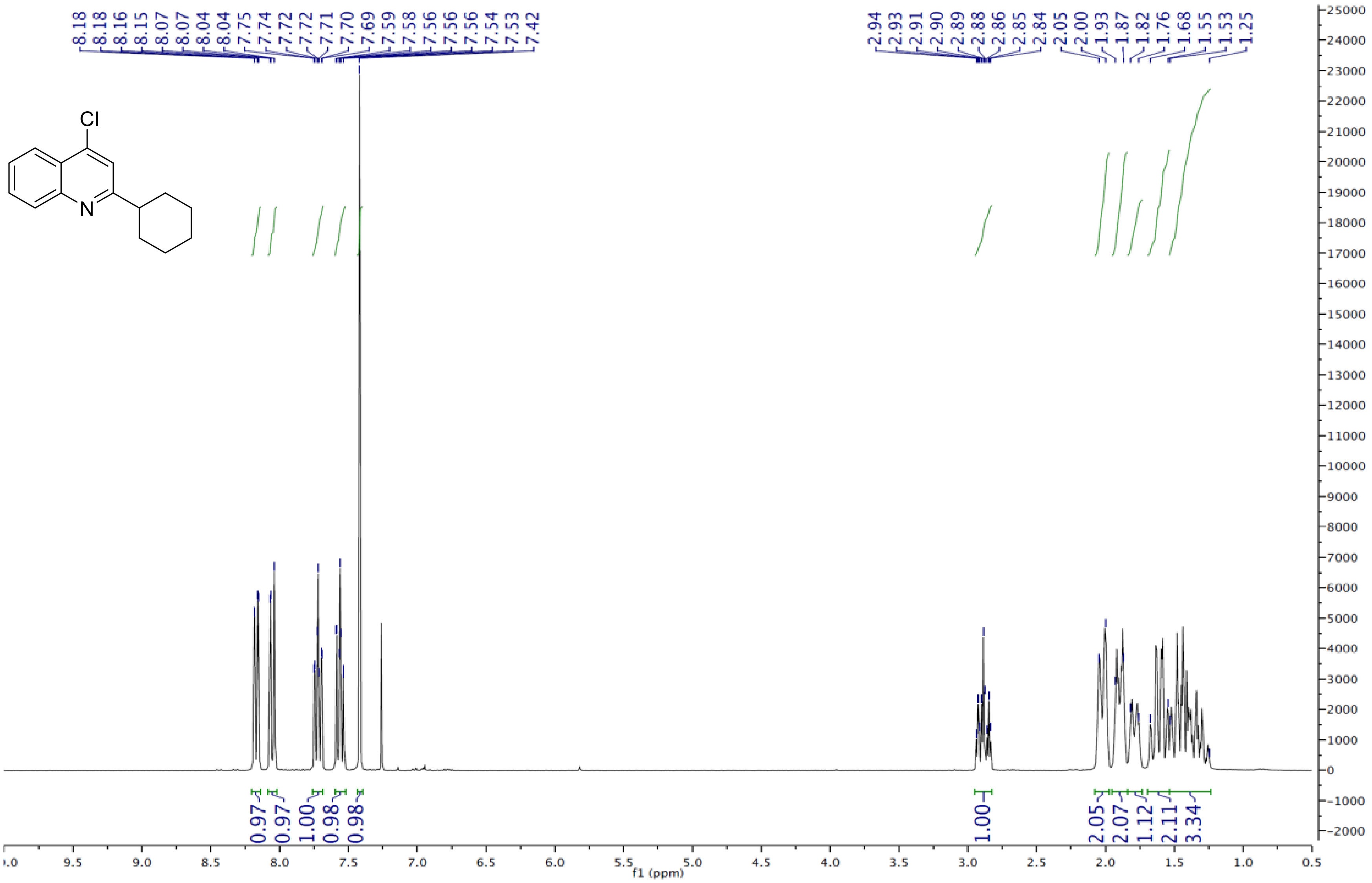

4-Chloro-2-cyclohexylquinoline (12)

^{13}C-NMR, 75 MHz, $CDCl_3$

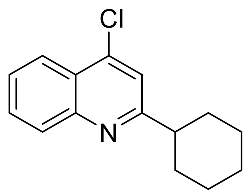

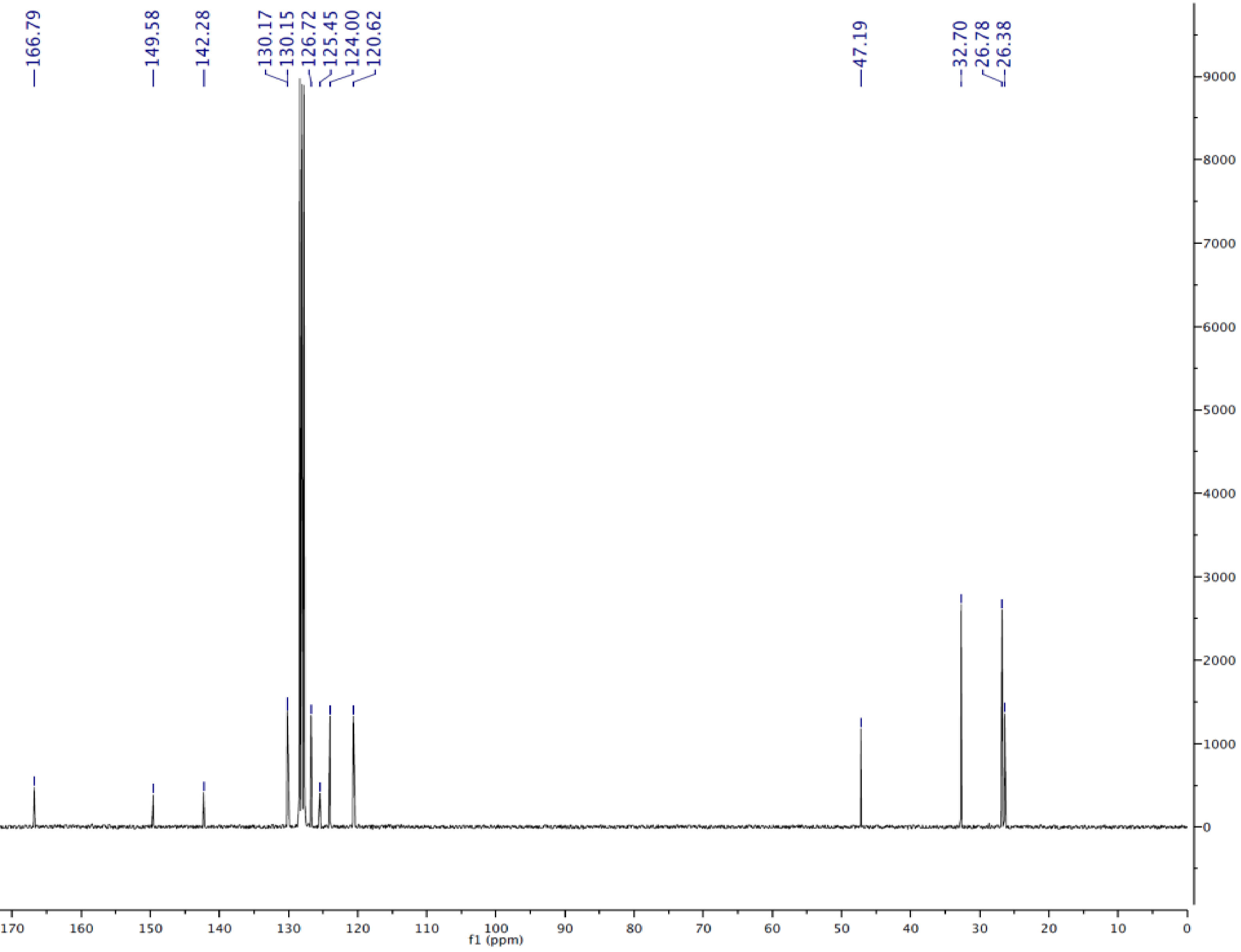

6-Cyclohexylphenanthridine (13)

^{1}H-NMR, 300 MHz, $CDCl_3$

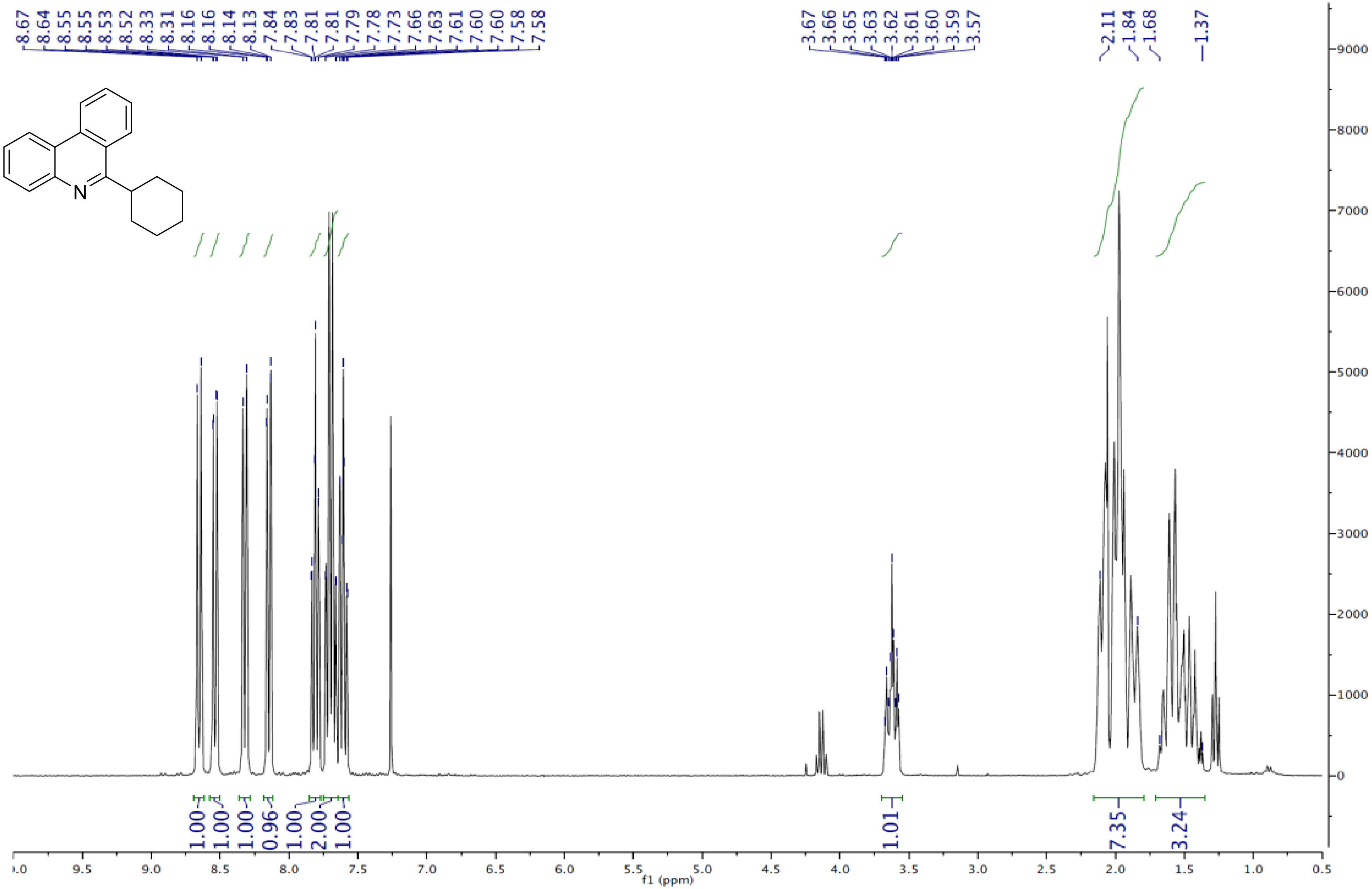

6-Cyclohexylphenanthridine (13)

^{13}C-NMR, 75 MHz, $CDCl_3$

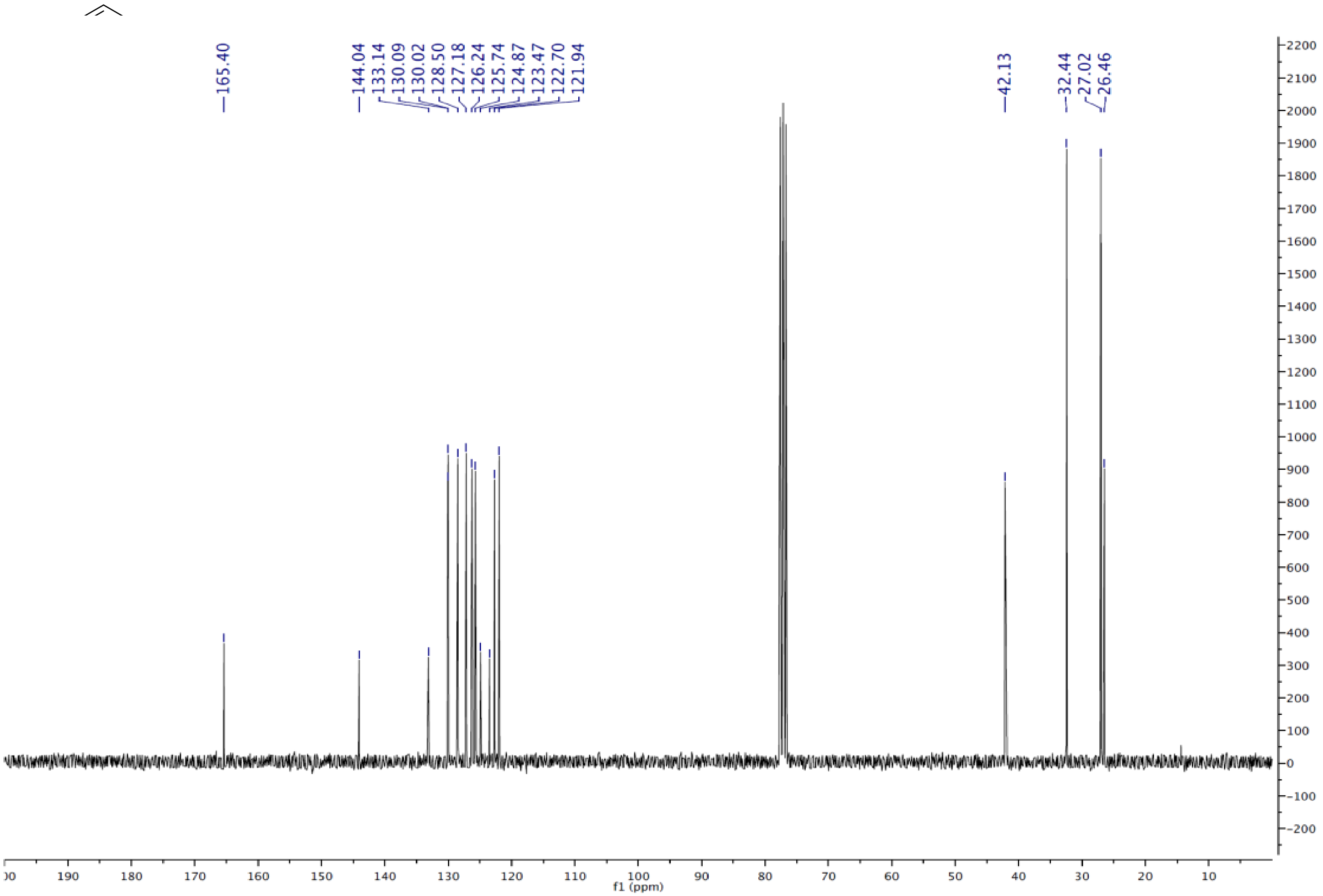

3-Bromo-2-cyclohexylquinoline (14)

^{1}H-NMR, 300 MHz, $CDCl_3$

^{1}H-NMR, 300 MHz, $CDCl_3$

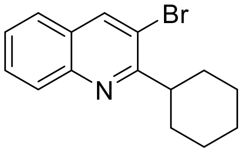

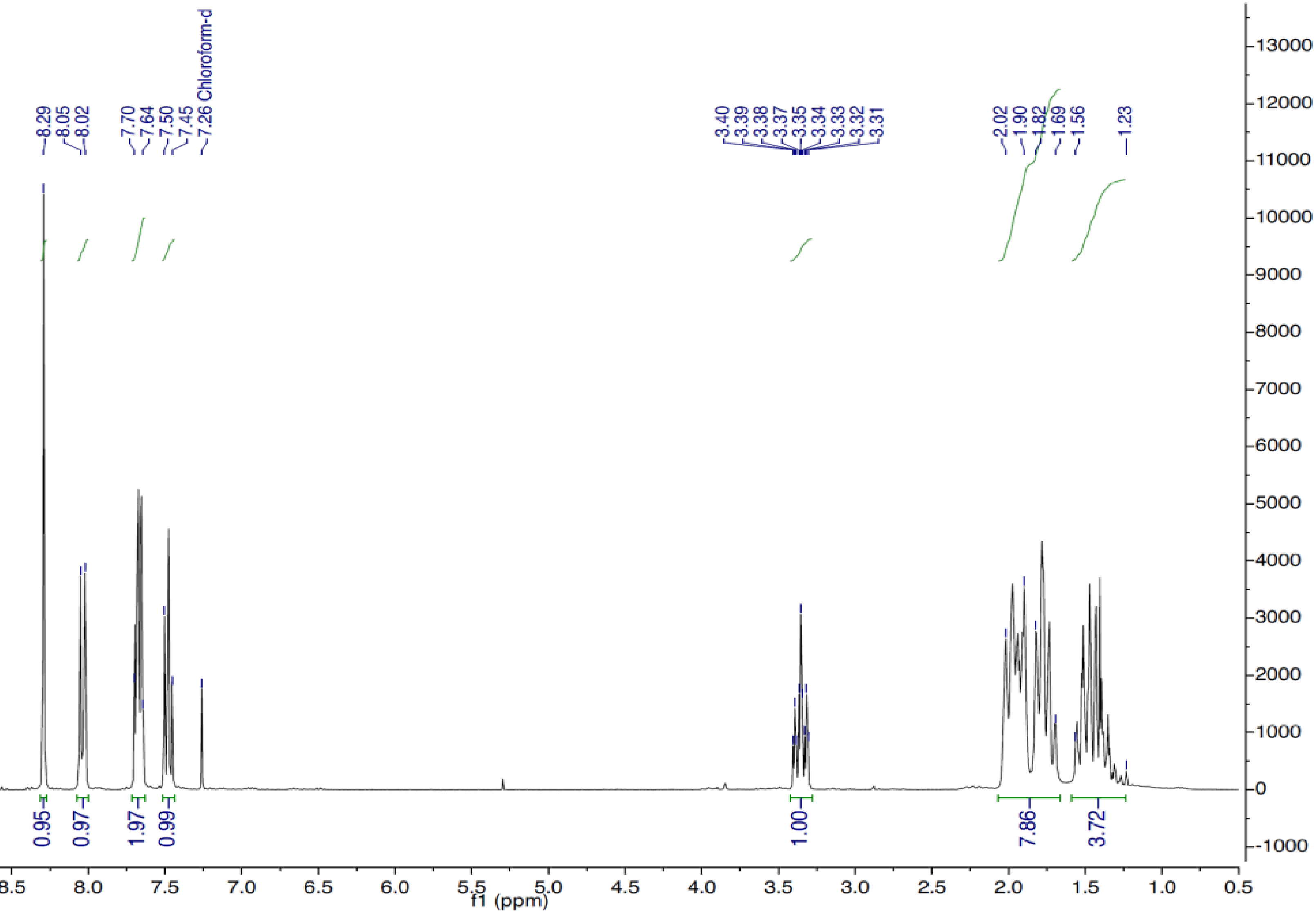

3-Bromo-2-cyclohexylquinoline (14)

^{13}C-NMR, 75 MHz, $CDCl_3$

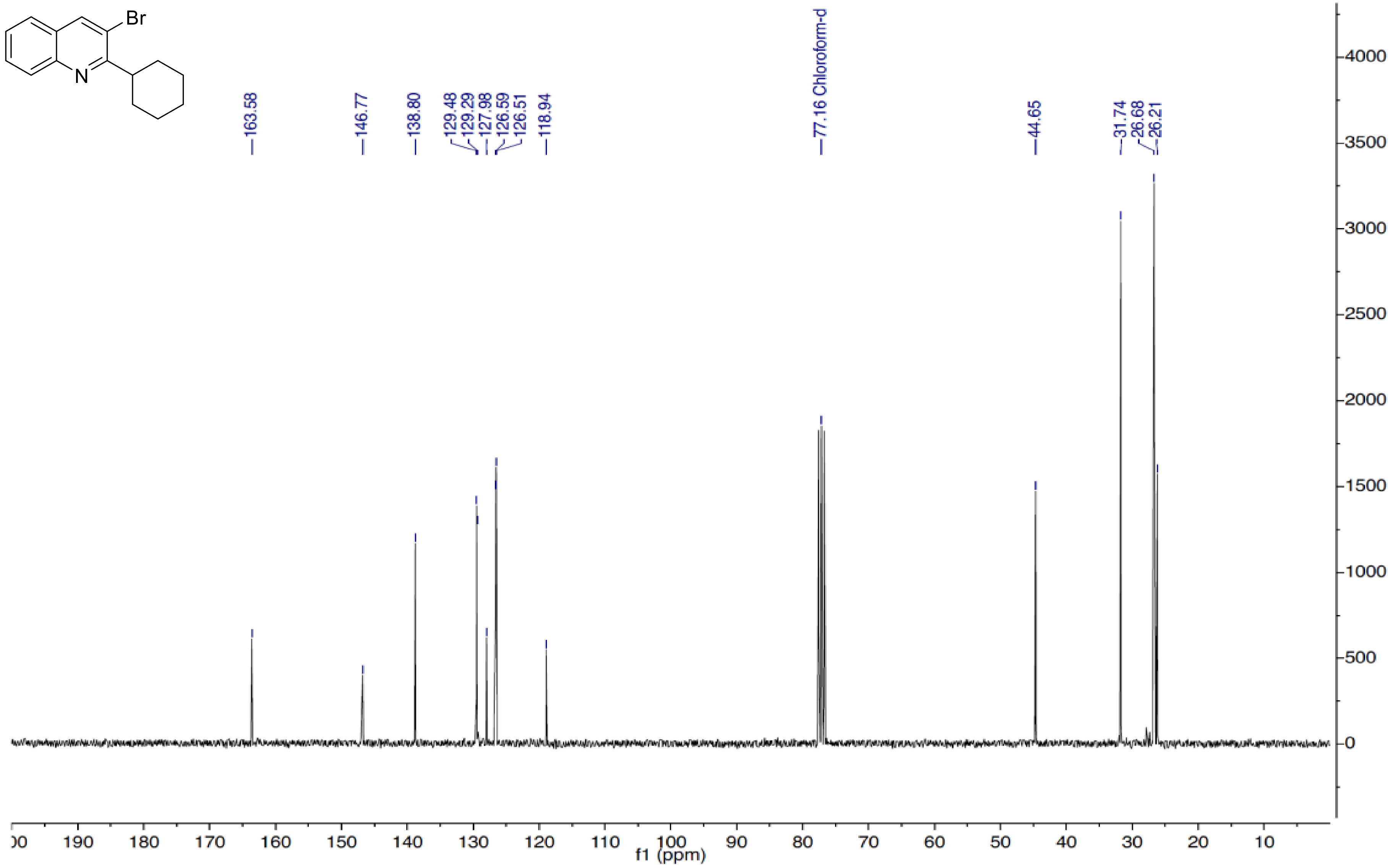

4-Cyclohexyl-2-methylquinoline (15)

^{1}H-NMR, 300 MHz, $CDCl_3$

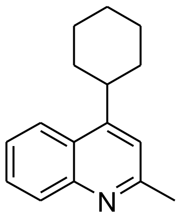

$C_{15}H_{16}N$
MW = 210.13

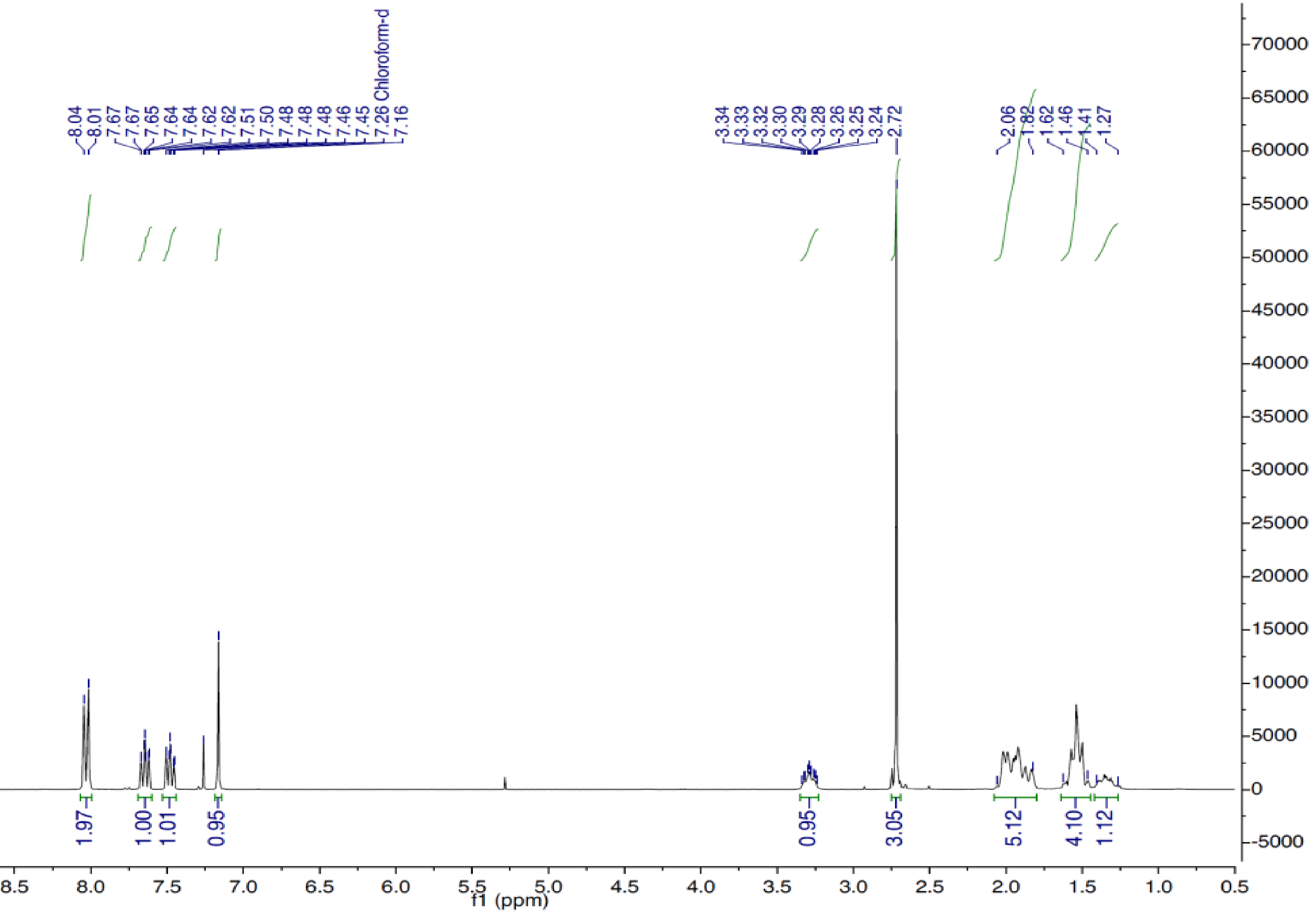

4-Cyclohexyl-2-methylquinoline (15)

^{1}H-NMR, 300 MHz, $CDCl_3$

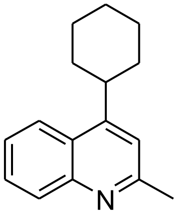

$C_{15}H_{16}N$
MW = 210.13

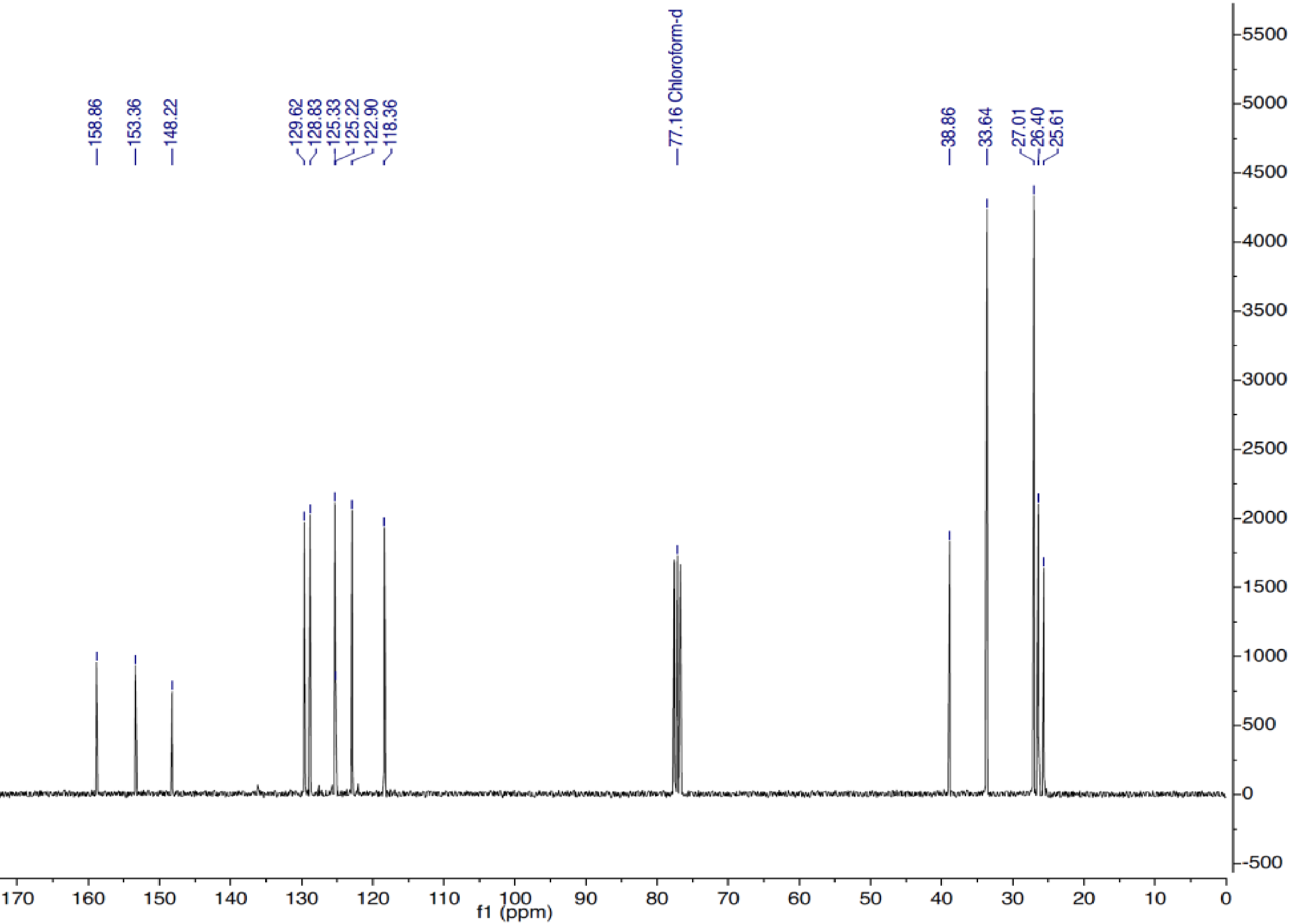

2-Dodecyl-4-phenylpyridine (16)

^{1}H-NMR, 300 MHz, $CDCl_3$

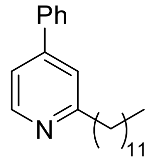

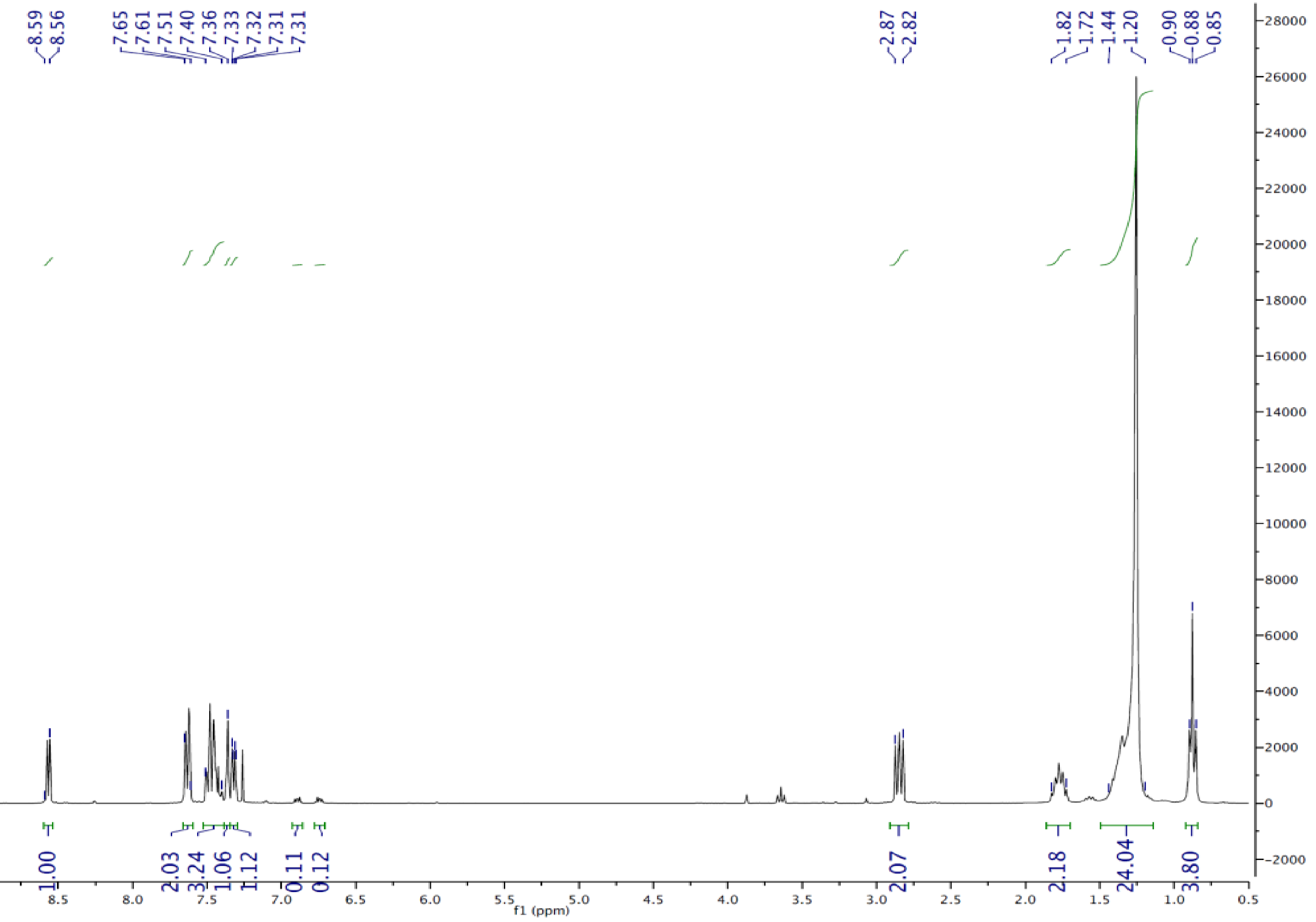

2-Dodecyl-4-phenylpyridine (16)

^{13}C-NMR, 75 MHz, $CDCl_3$

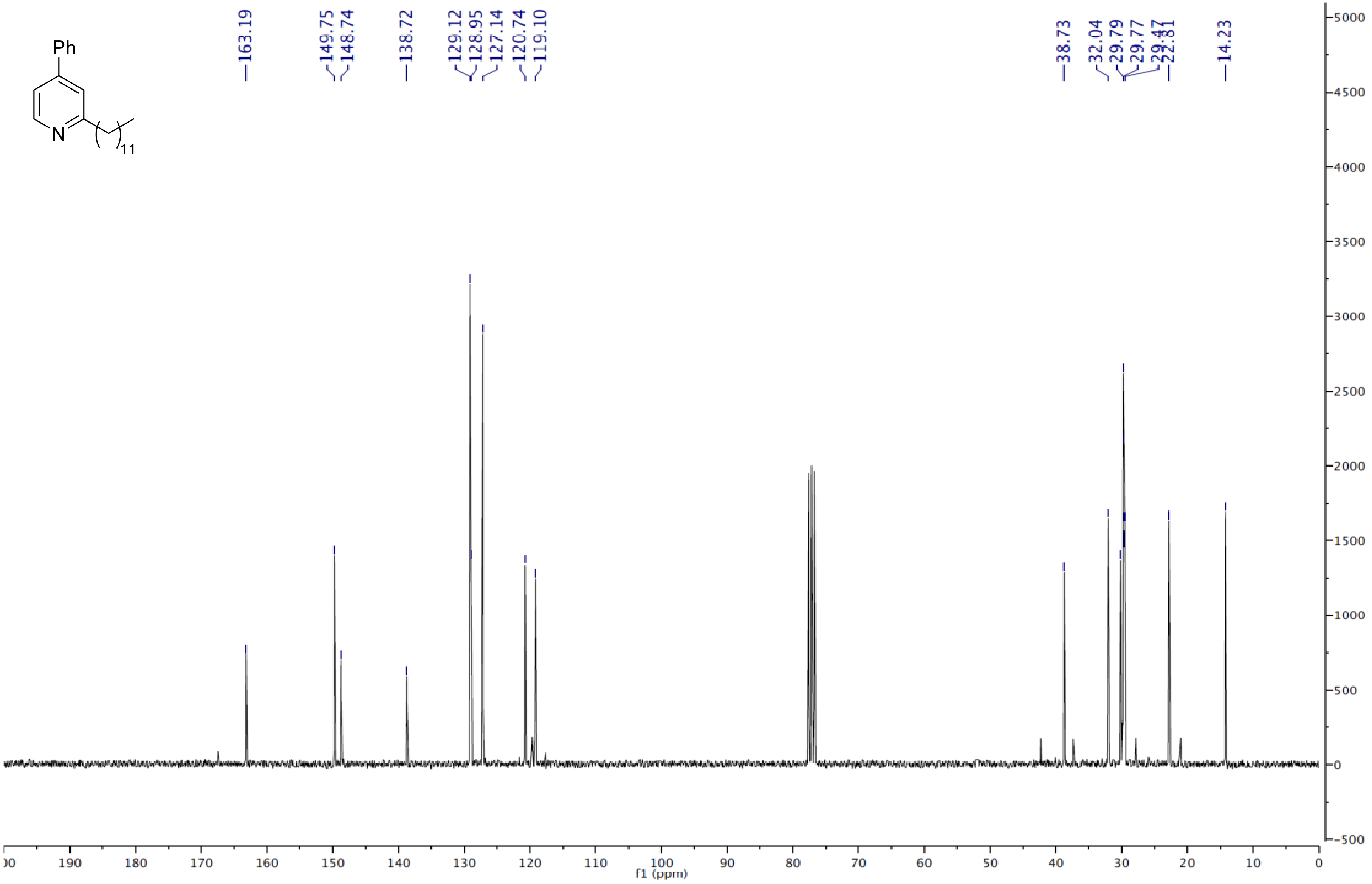

2-(2,3-Dimethylbutan-2-yl)-4-phenylpyridine (17)

^{1}H-NMR, 300 MHz, $CDCl_3$

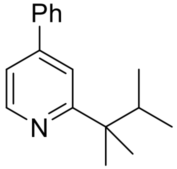

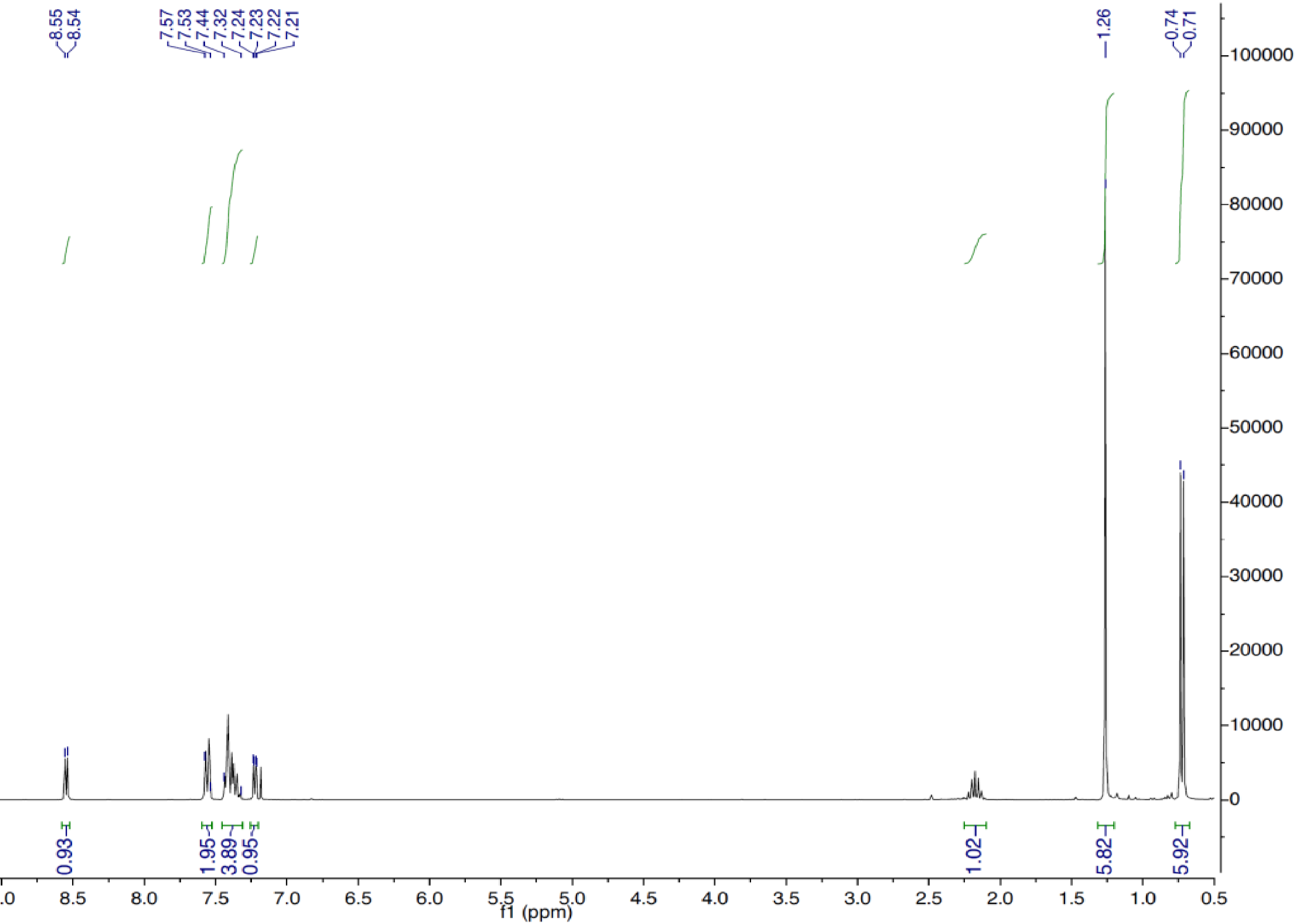

2-(2,3-Dimethylbutan-2-yl)-4-phenylpyridine (17)

^{13}C-NMR, 75 MHz, $CDCl_3$

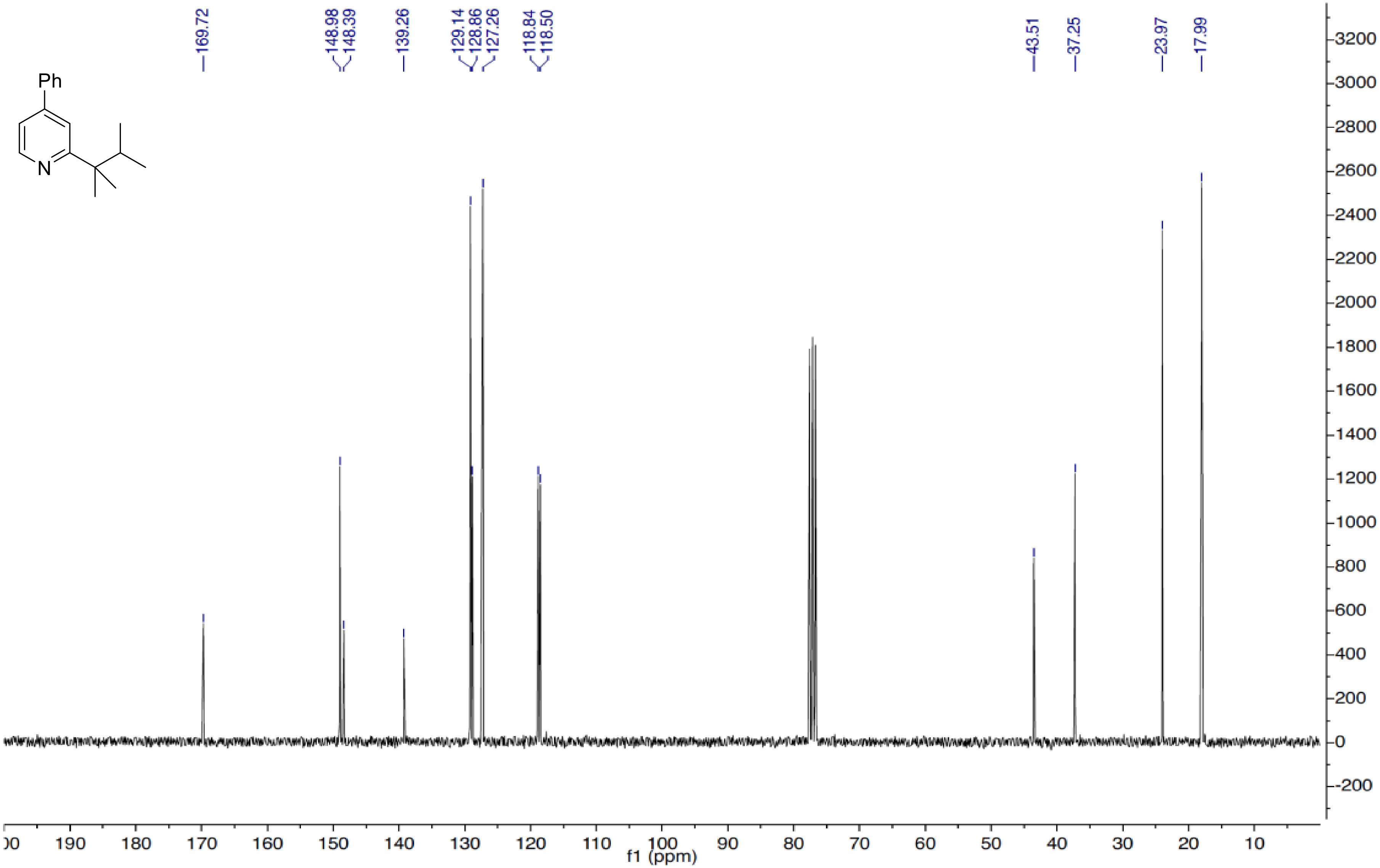

2-Cyclohexyl-4-*tert*-butyl-pyridine (18)

^{1}H-NMR, 300 MHz, $CDCl_3$

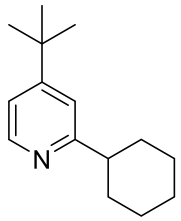

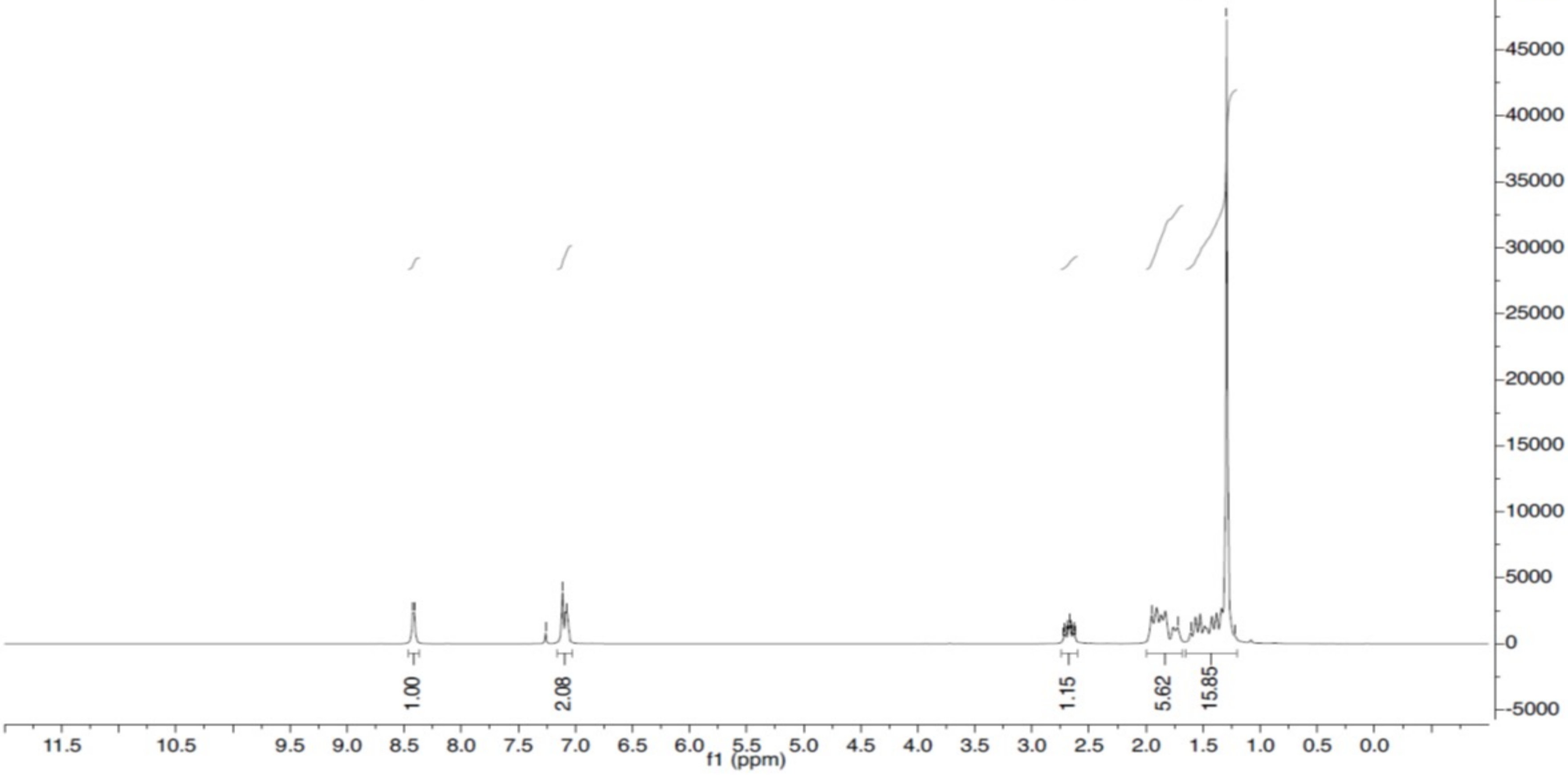

2-Cyclohexyl-4-*tert*-butyl-pyridine (18)

^{13}C-NMR, 75 MHz, $CDCl_3$

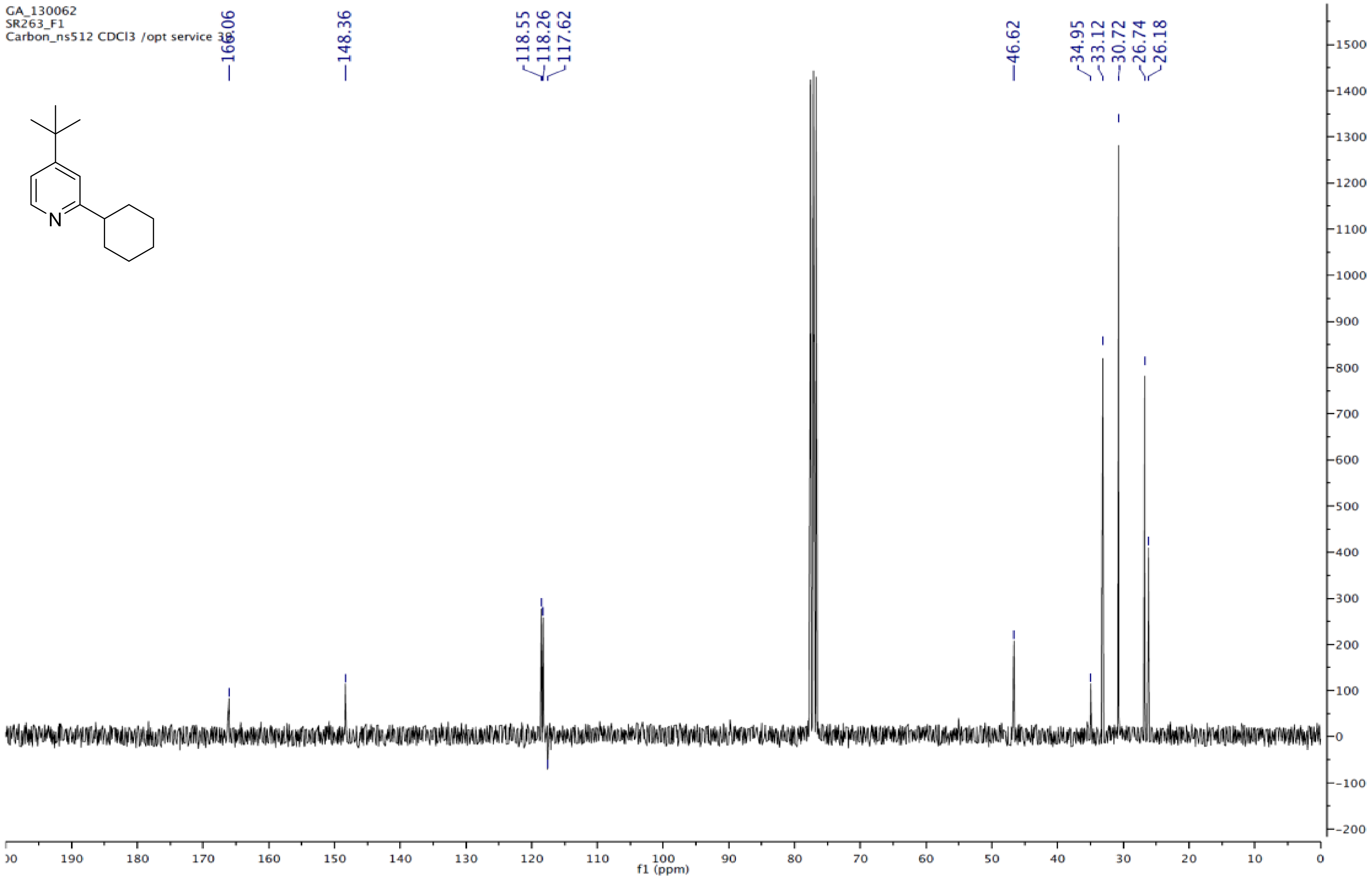

Ethyl 2-cyclohexylisonicotinate (19)

^{1}H-NMR, 300 MHz, $CDCl_3$

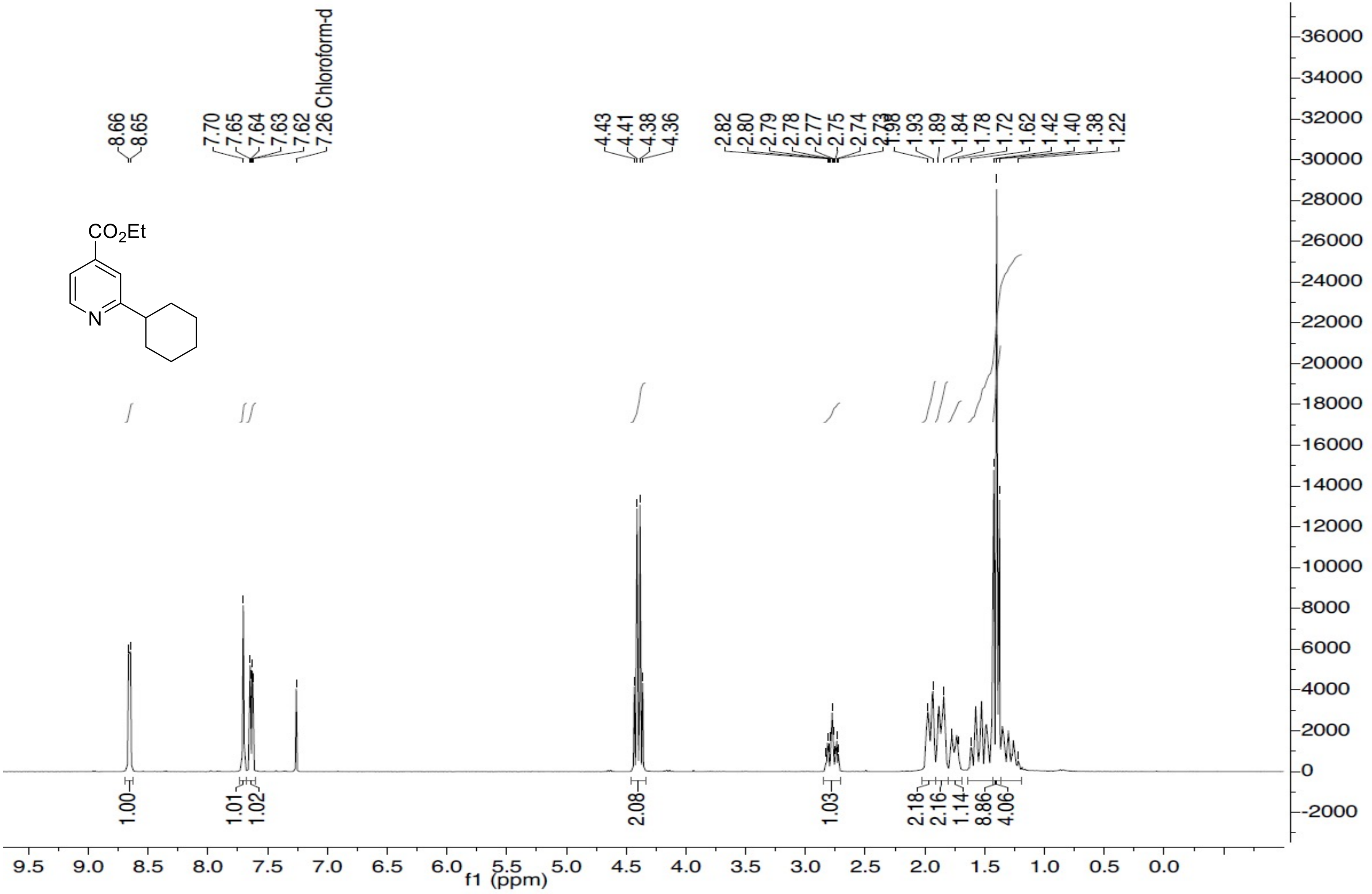

Ethyl 2-cyclohexylisonicotinate (19)

^{13}C-NMR, 75 MHz, $CDCl_3$

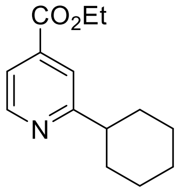

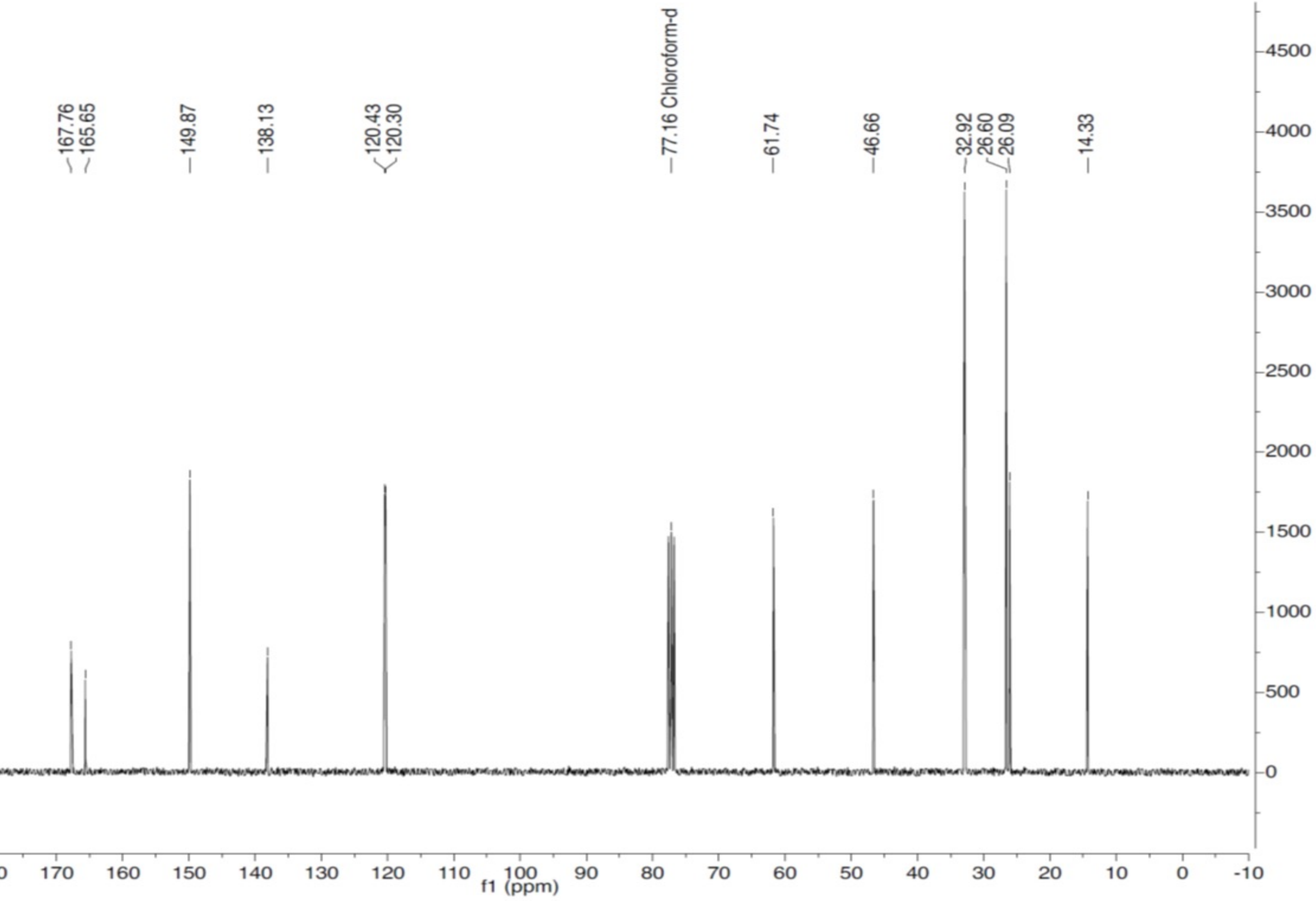

4-Methyl-2-((1*R*,2*R*,3*R*,5*S*)-2,6,6-trimethylbicyclo[3.1.1]heptan-3-yl) (20)

^{1}H-NMR, 300 MHz, $CDCl_3$

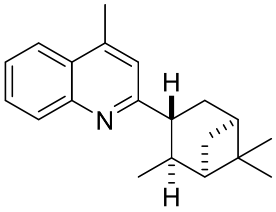

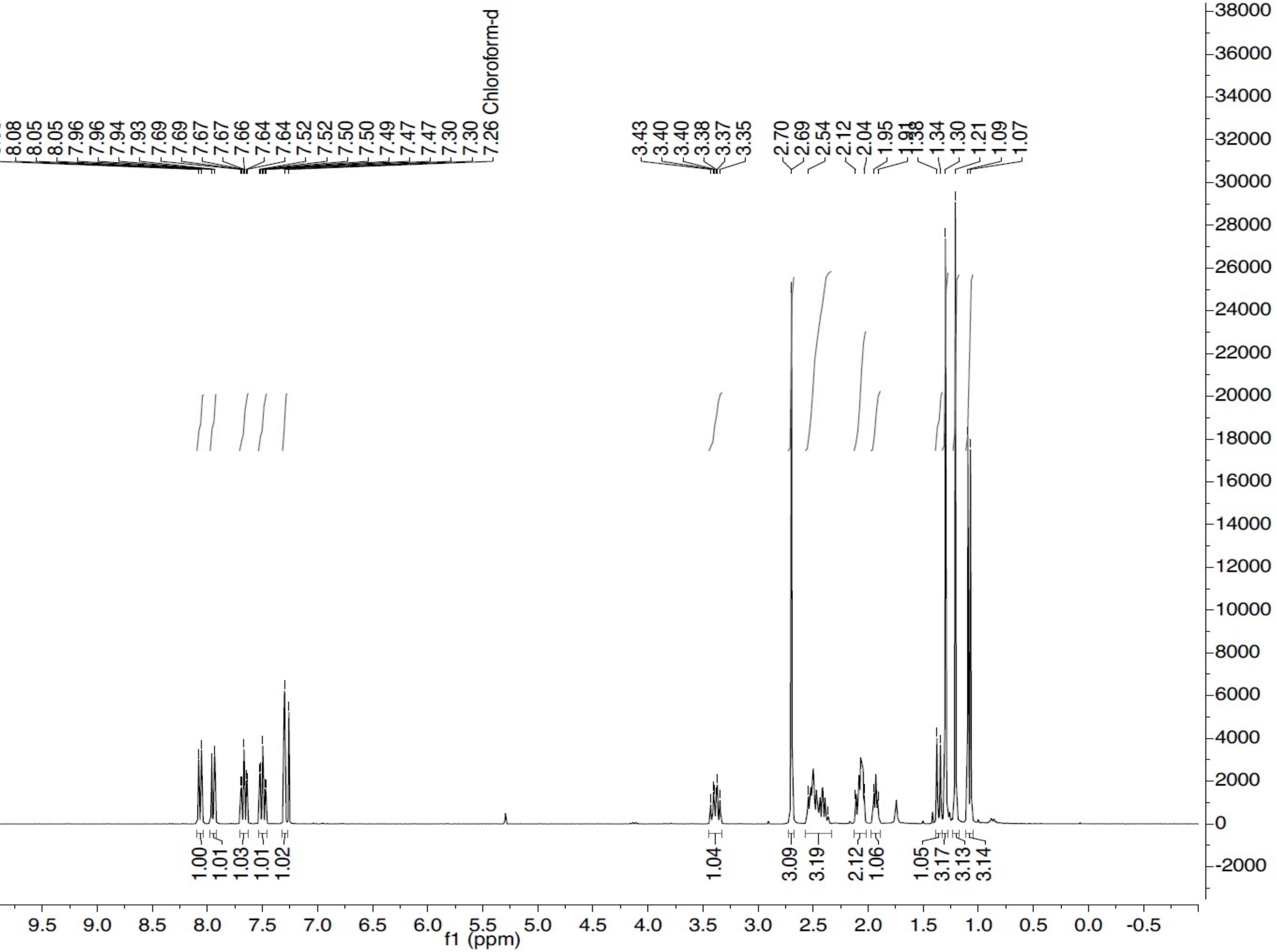

4-Methyl-2-((1*R*,2*R*,3*R*,5*S*)-2,6,6-trimethylbicyclo[3.1.1]heptan-3-yl) (20)

^{13}C-NMR, 75 MHz, $CDCl_3$

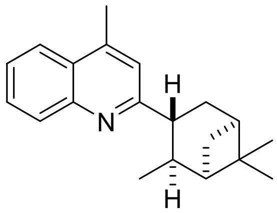

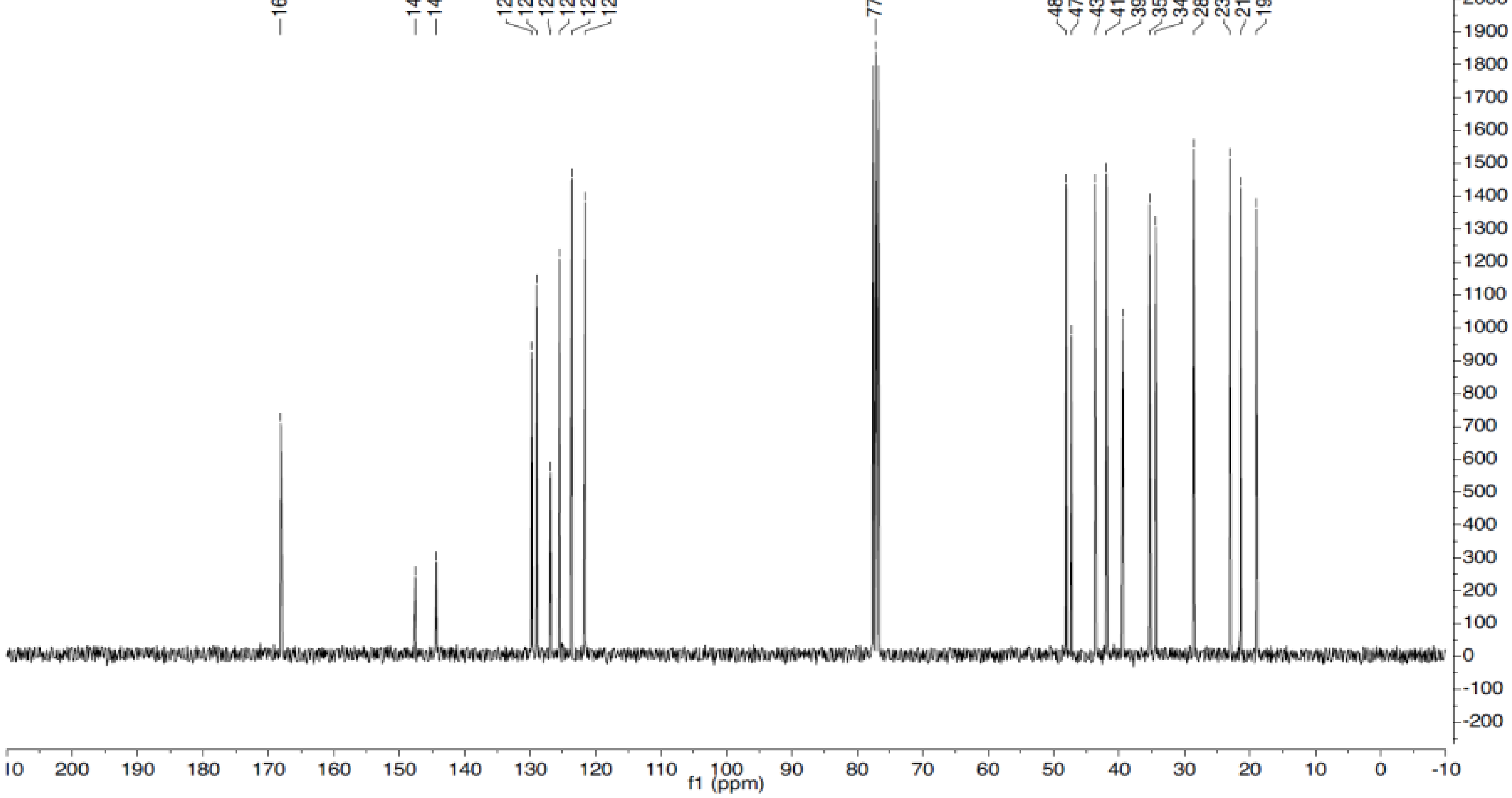

2-(((1*S*,2*S*,5*S*)-6,6-Dimethylbicyclo[3.1.1]heptan-2-yl)methyl)-4-methylquinoline (21)

^{1}H-NMR, 300 MHz, $CDCl_3$

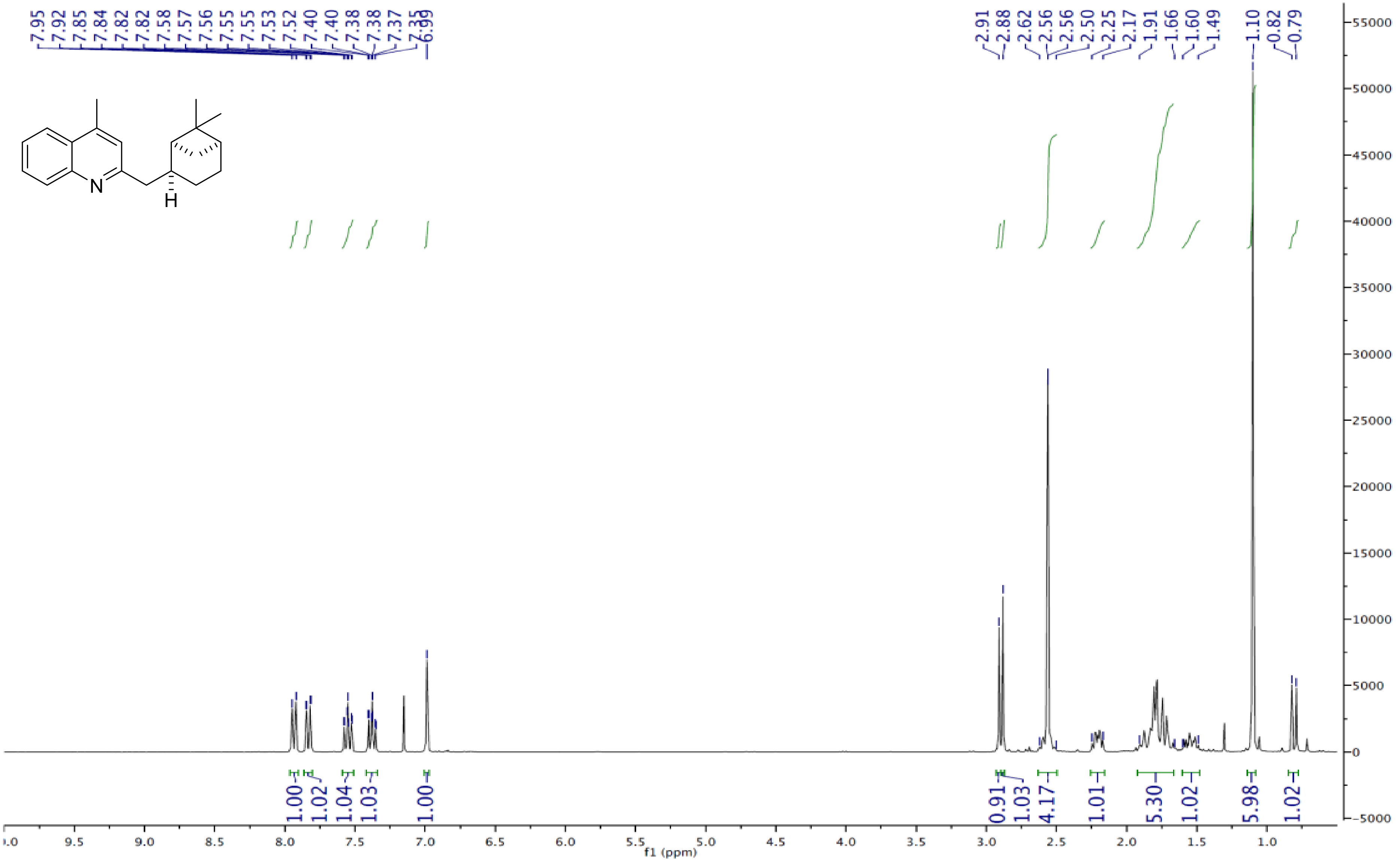

2-(((1*S*,2*S*,5*S*)-6,6-Dimethylbicyclo[3.1.1]heptan-2-yl)methyl)-4-methylquinoline (21)

^{13}C-NMR, 75 MHz, $CDCl_3$

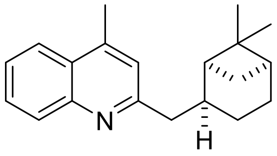

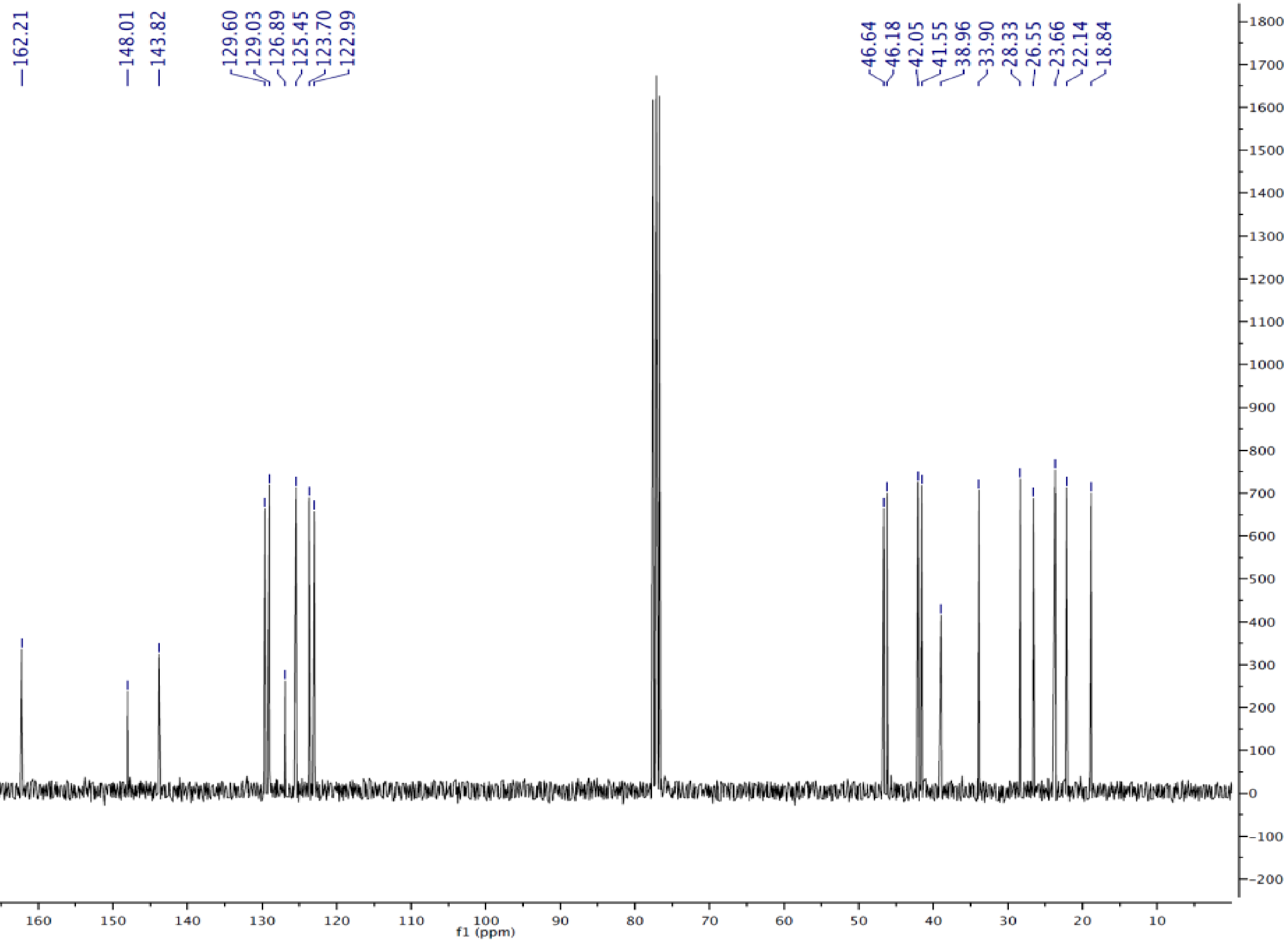

2-(2-(2-(Benzyloxy)ethyl)-6,6-dimethylbicyclo[3.1.1]heptan-3-yl)-4-methylquinoline (22)

^{1}H NMR (400 MHz, C_6D_6)

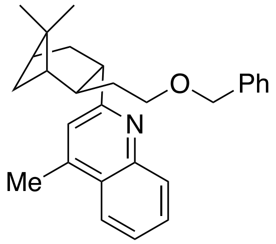

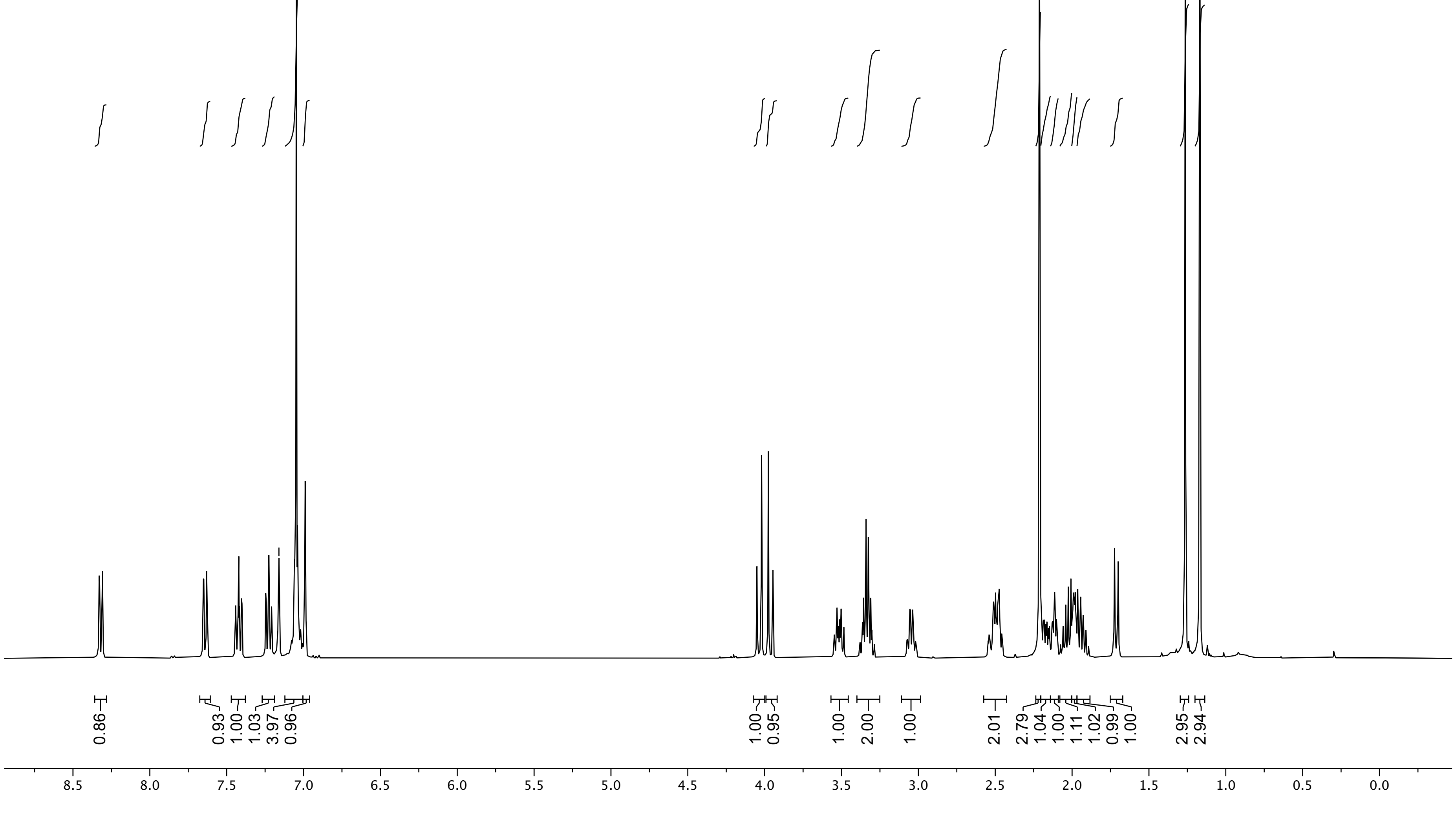

2-(2-(2-(Benzyloxy)ethyl)-6,6-dimethylbicyclo[3.1.1]heptan-3-yl)-4-methylquinoline (22)

^{1}H NMR (400 MHz, C_6D_6)

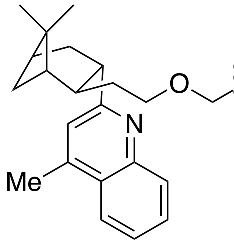

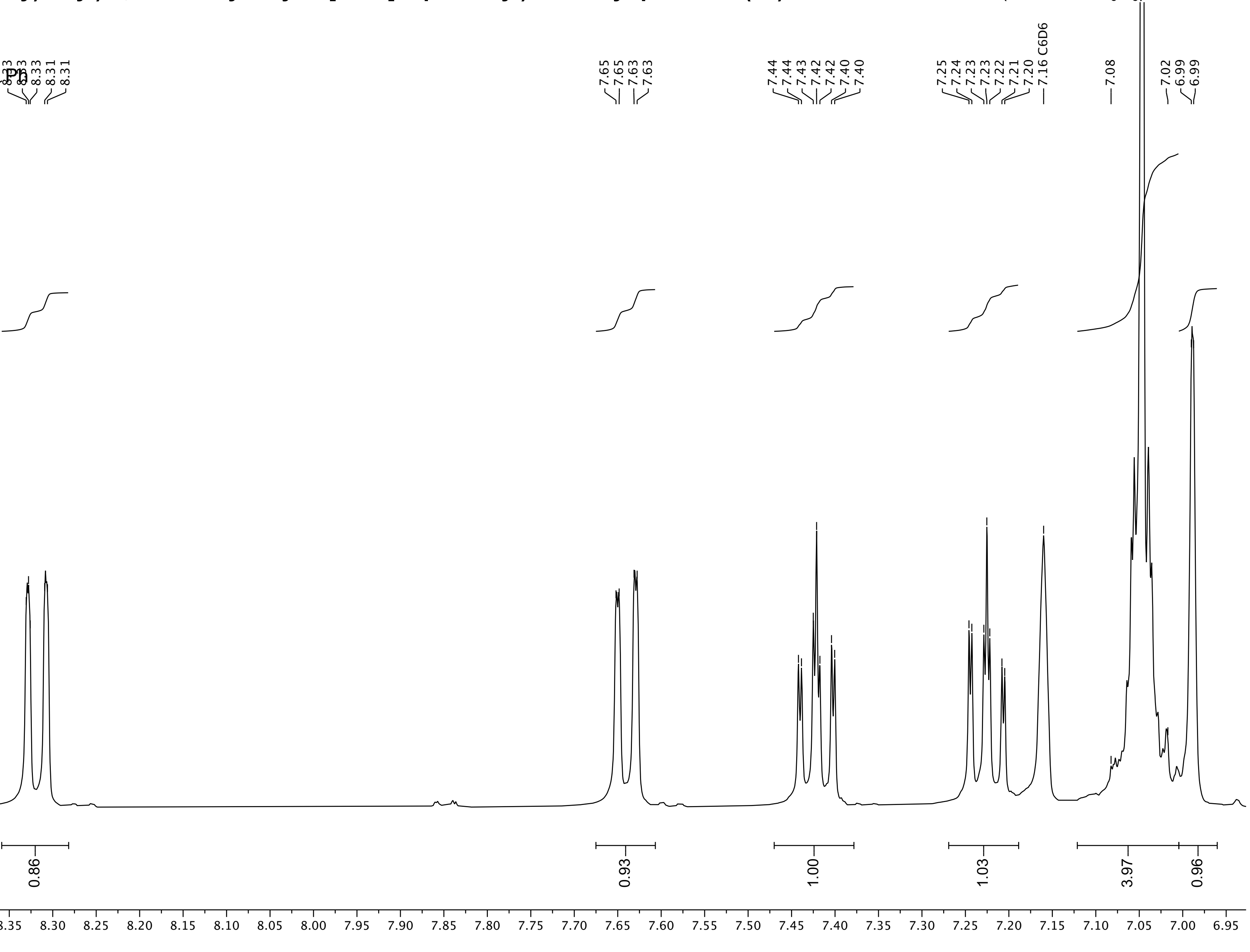

2-(2-(2-(Benzyloxy)ethyl)-6,6-dimethylbicyclo[3.1.1]heptan-3-yl)-4-methylquinoline (22)

^{1}H NMR (400 MHz, C_6D_6)

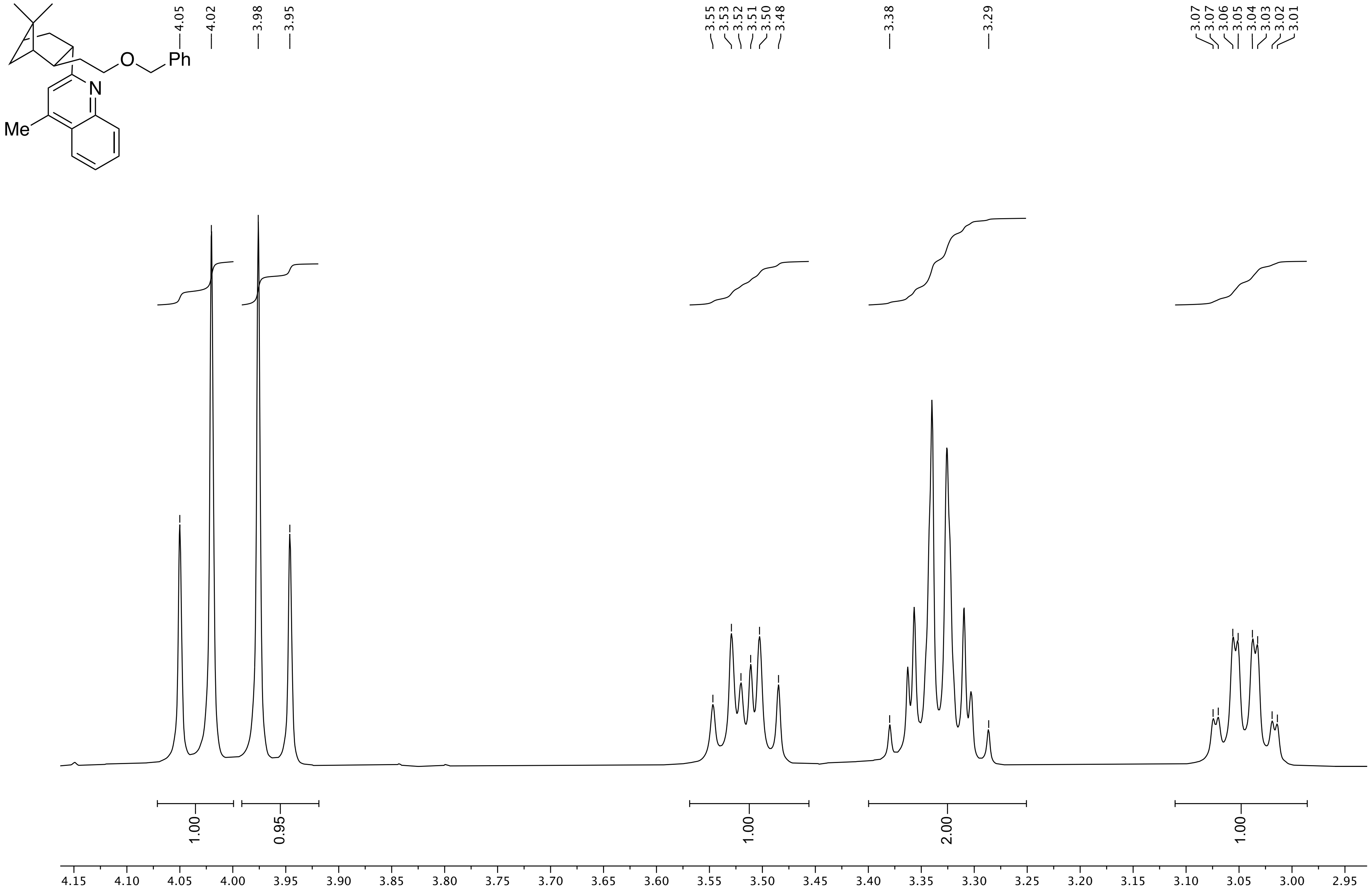

2-(2-(2-(Benzyloxy)ethyl)-6,6-dimethylbicyclo[3.1.1]heptan-3-yl)-4-methylquinoline (22)

^{1}H NMR (400 MHz, C_6D_6)

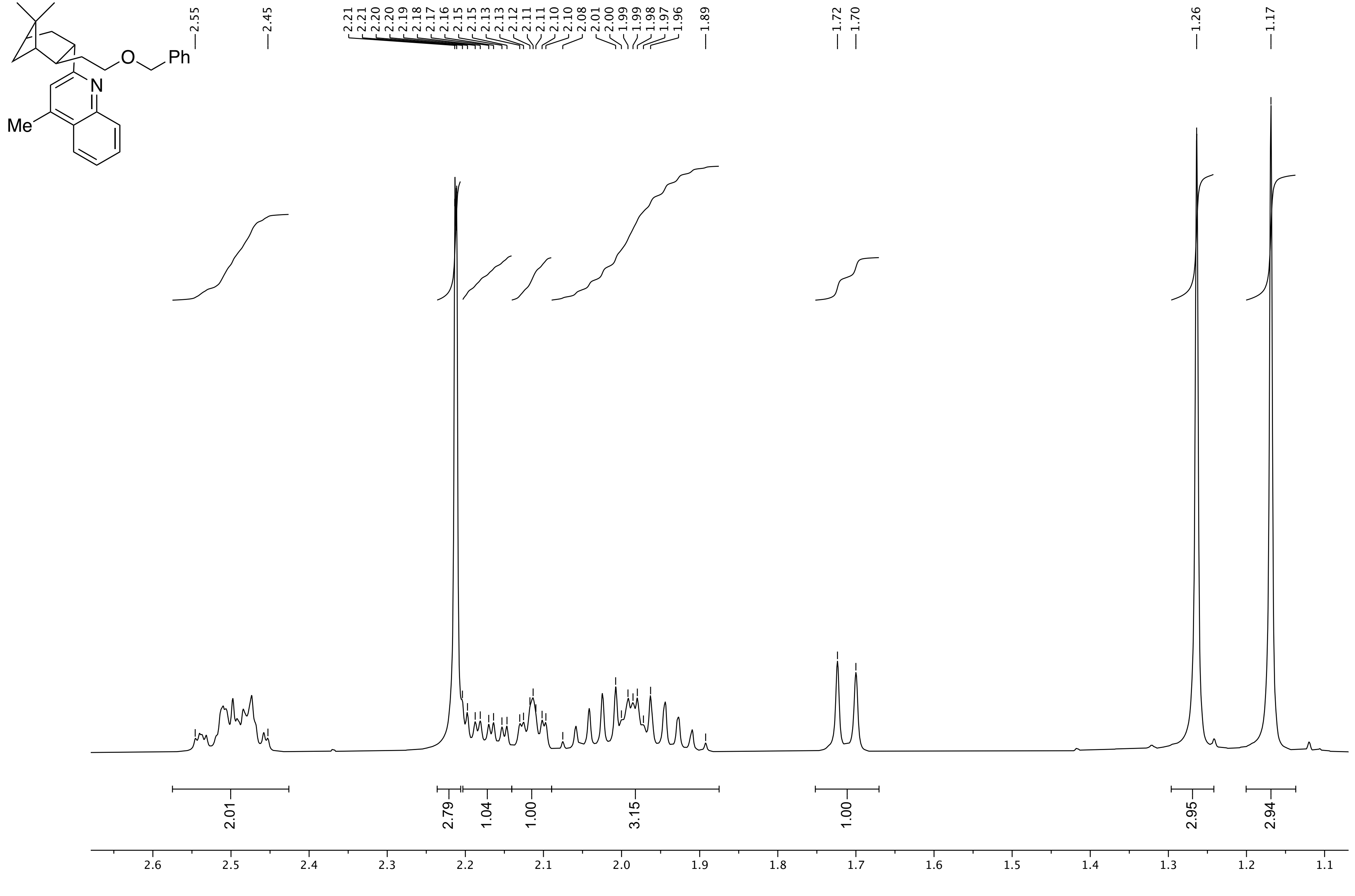

2-(2-(2-(Benzyloxy)ethyl)-6,6-dimethylbicyclo[3.1.1]heptan-3-yl)-4-methylquinoline (22)

^{1}H NMR (400 MHz, C_6D_6)

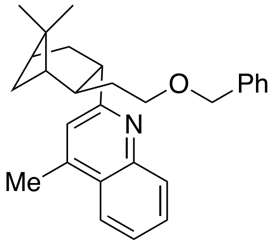

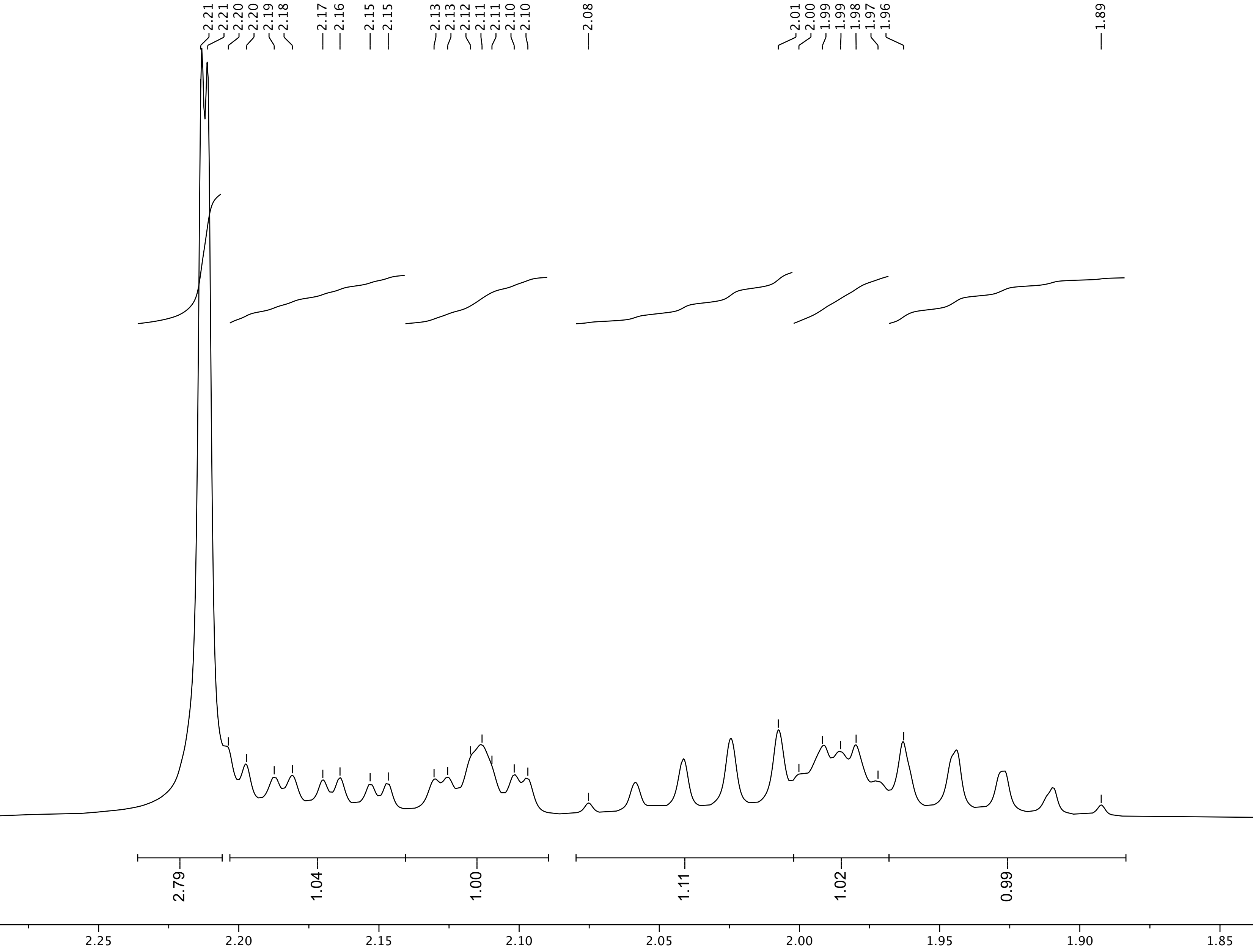

2-(2-(2-(Benzyloxy)ethyl)-6,6-dimethylbicyclo[3.1.1]heptan-3-yl)-4-methylquinoline (22)

^{13}C NMR (101 MHz, C_6D_6)

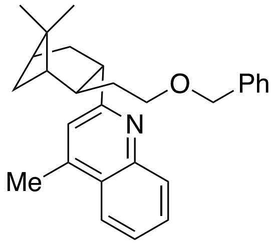

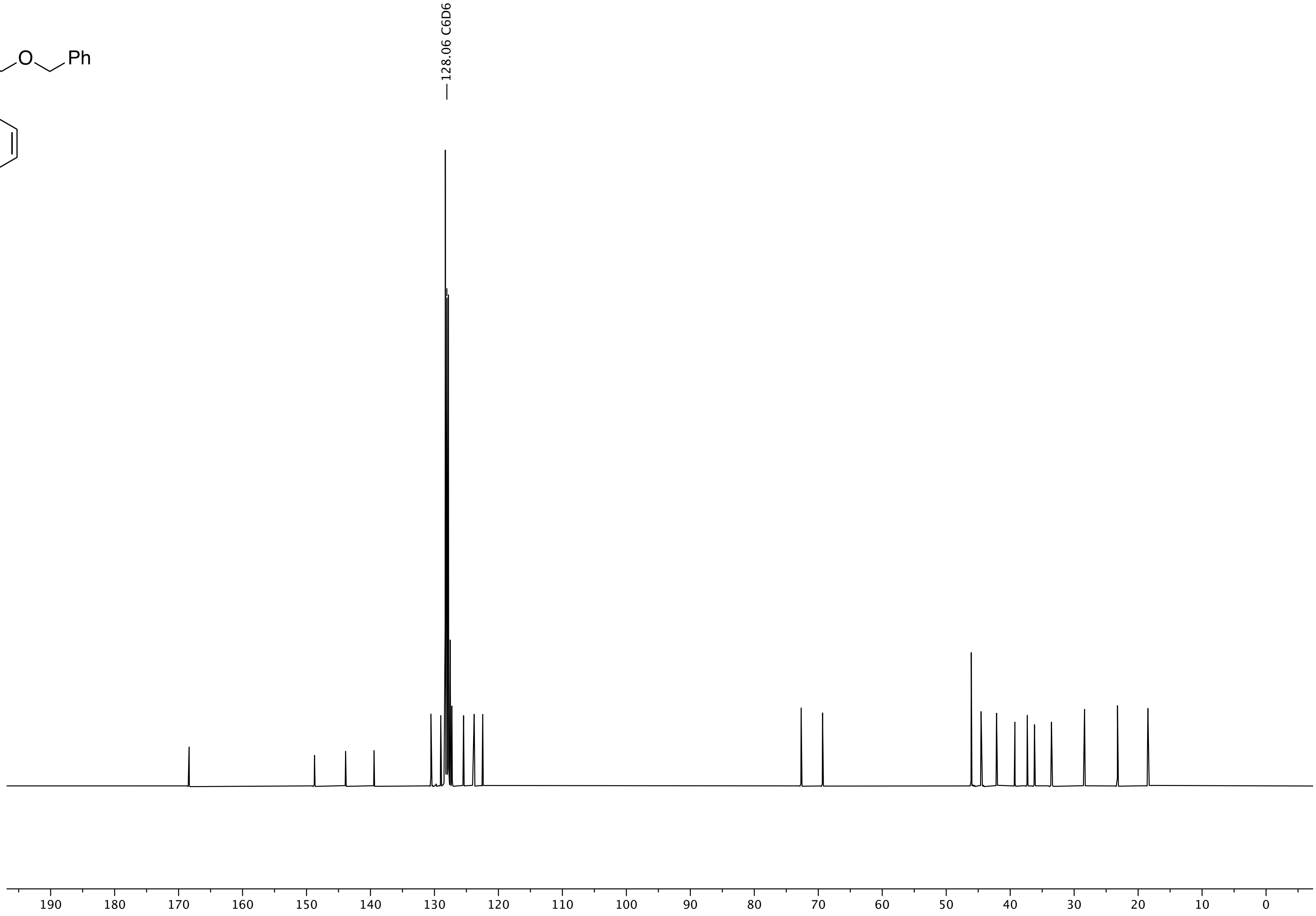

2-(2-(2-(Benzyloxy)ethyl)-6,6-dimethylbicyclo[3.1.1]heptan-3-yl)-4-methylquinoline (22)

^{13}C NMR (101 MHz, C_6D_6)

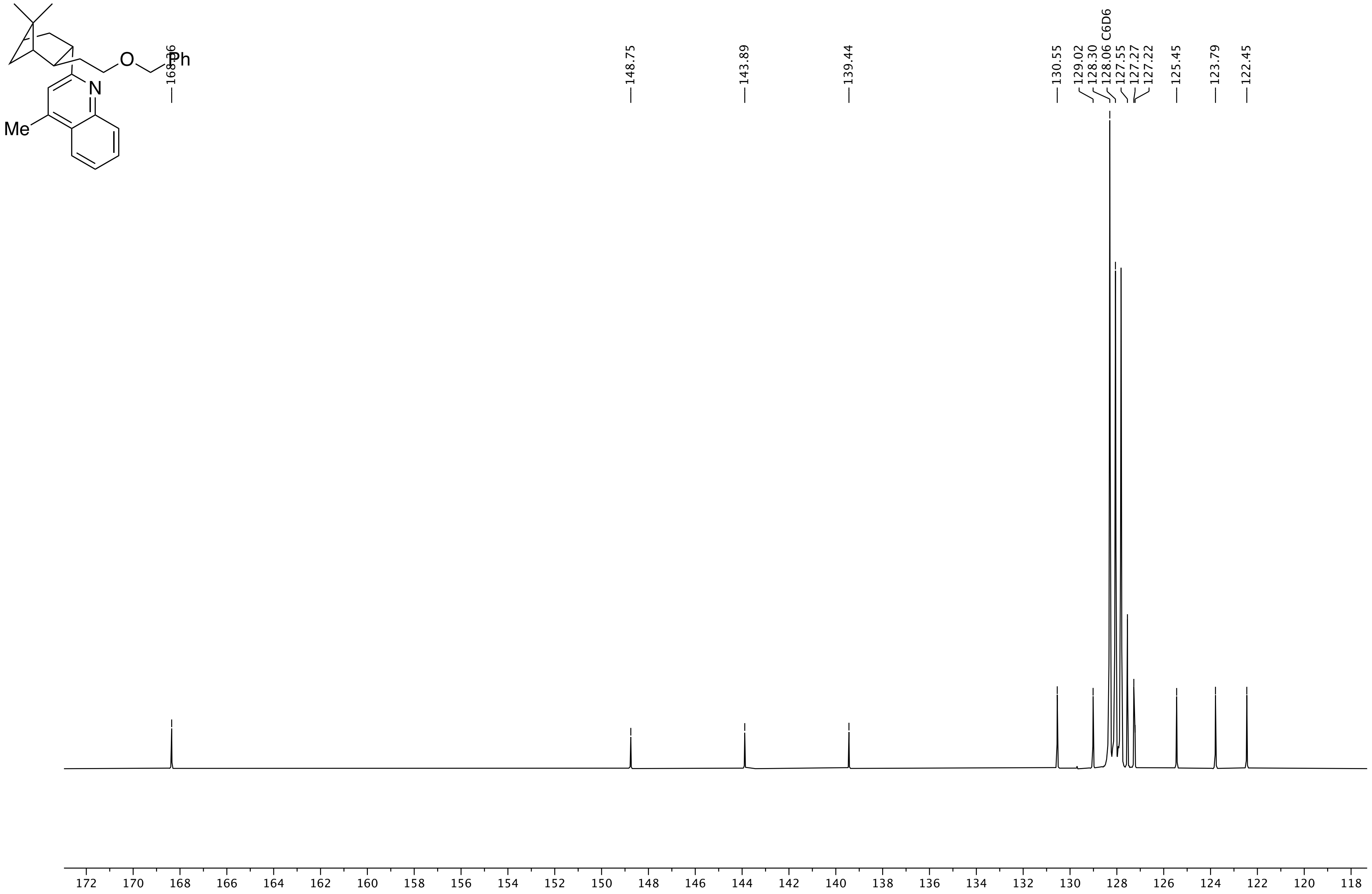

2-(2-(2-(Benzyloxy)ethyl)-6,6-dimethylbicyclo[3.1.1]heptan-3-yl)-4-methylquinoline (22)

^{13}C NMR (101 MHz, C_6D_6)

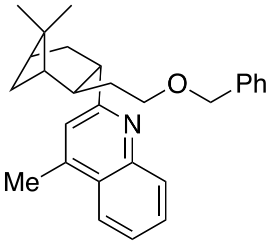

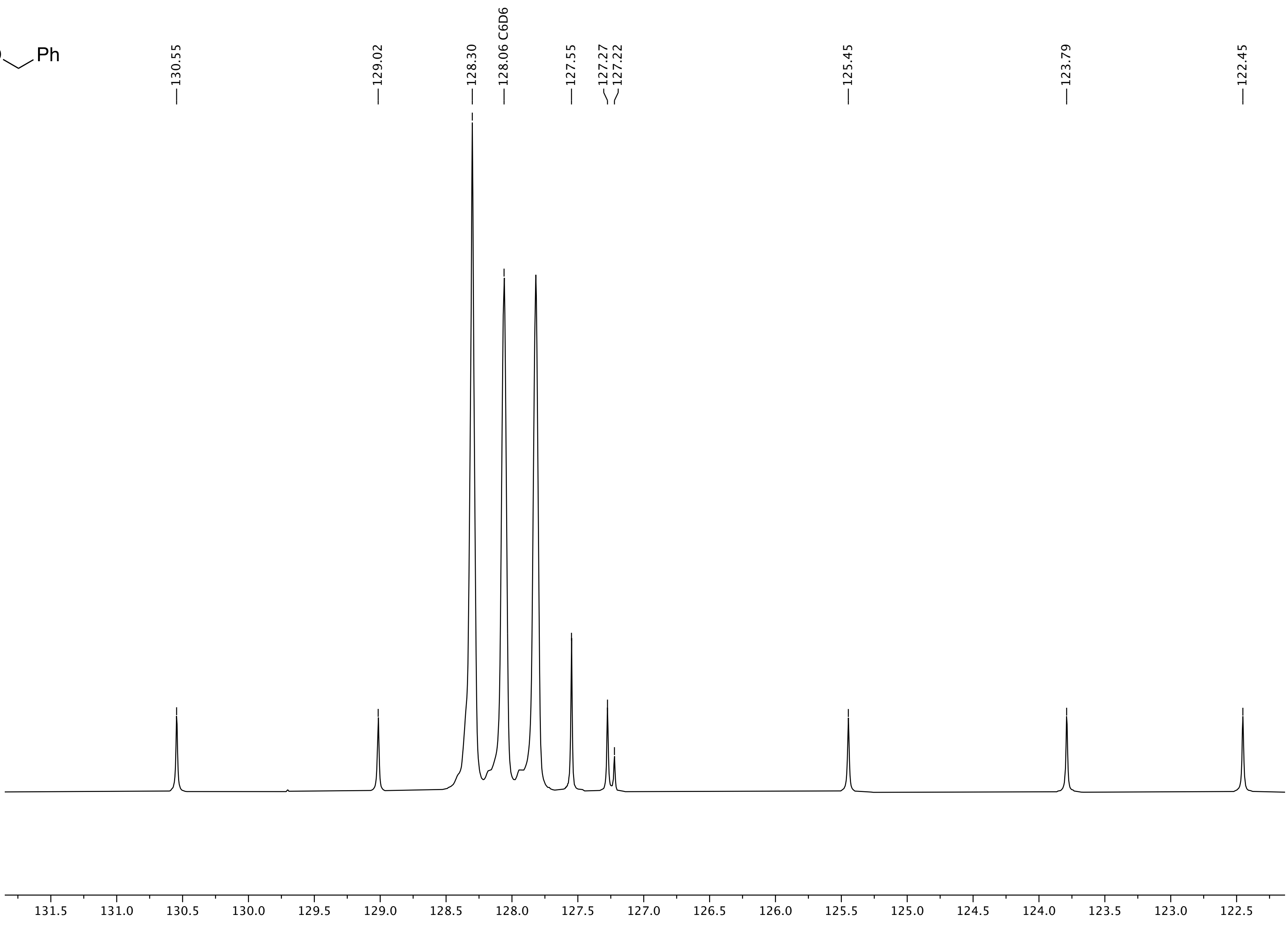

2-(2-(2-(Benzyloxy)ethyl)-6,6-dimethylbicyclo[3.1.1]heptan-3-yl)-4-methylquinoline (22)

^{13}C NMR (101 MHz, C_6D_6)

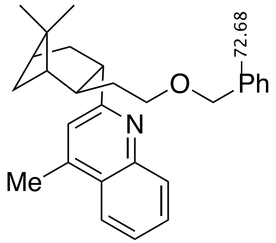

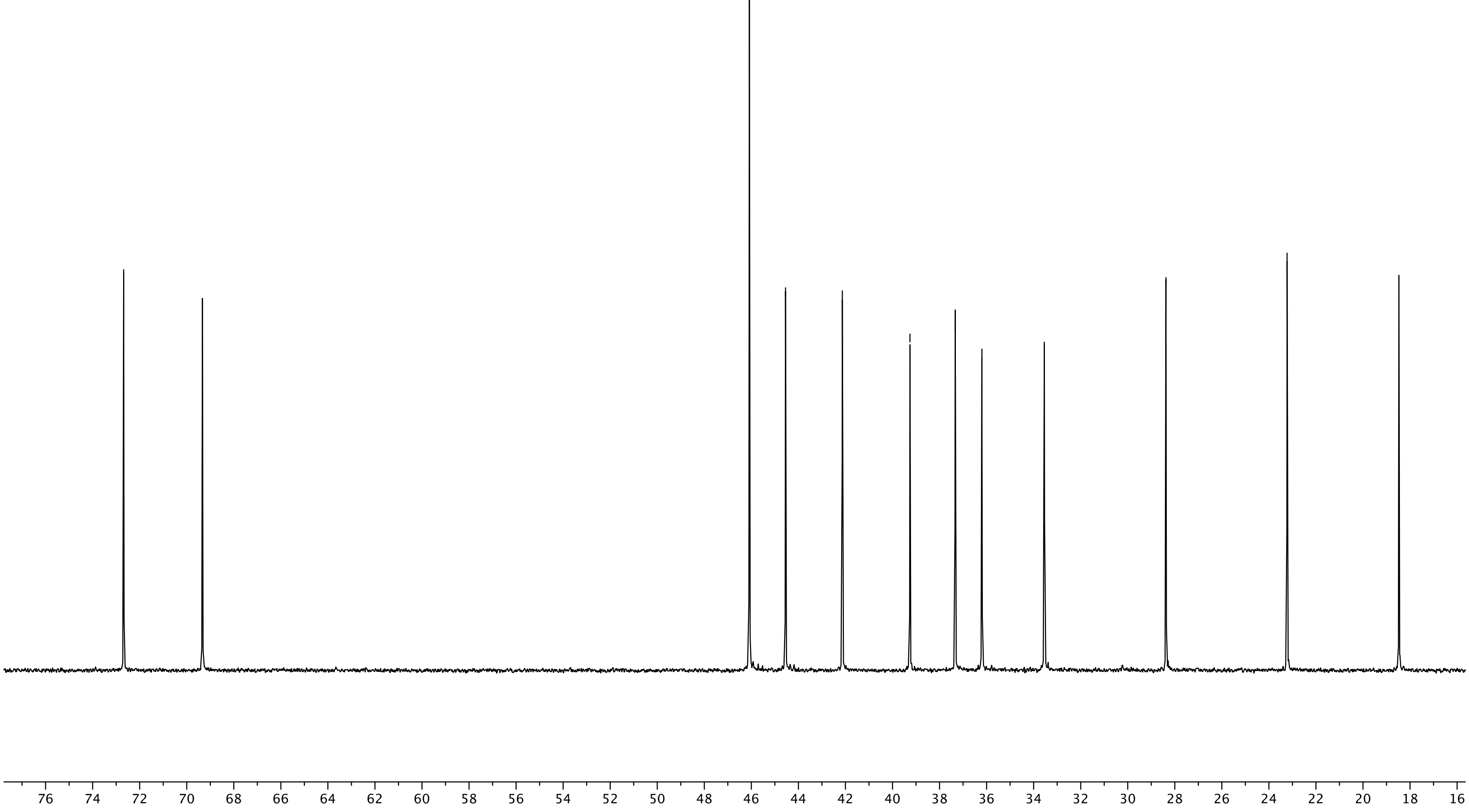

2-(2-(2-(Benzyloxy)ethyl)-6,6-dimethylbicyclo[3.1.1]heptan-3-yl)-4-methylquinoline (22)

Dept–135 (101 MHz, C_6D_6)

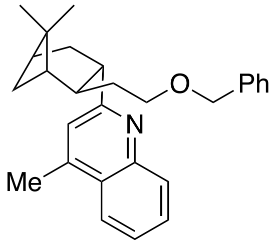

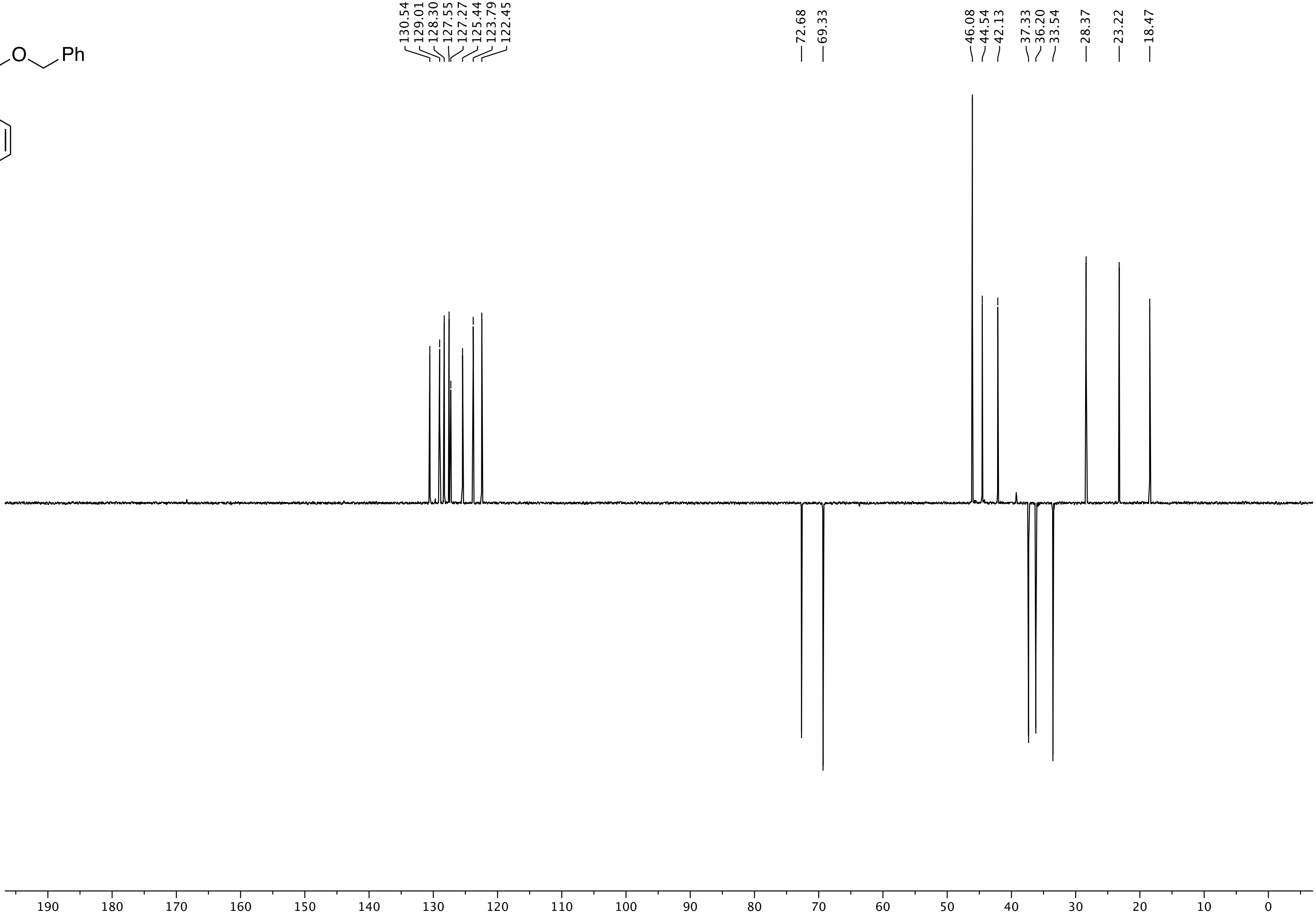

2-(2-(2-(Benzyloxy)ethyl)-6,6-dimethylbicyclo[3.1.1]heptan-3-yl)-4-methylquinoline (22)

Dept–135 (101 MHz, C_6D_6)

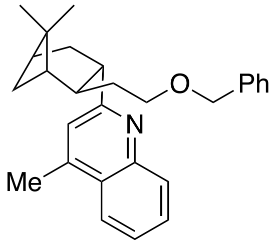

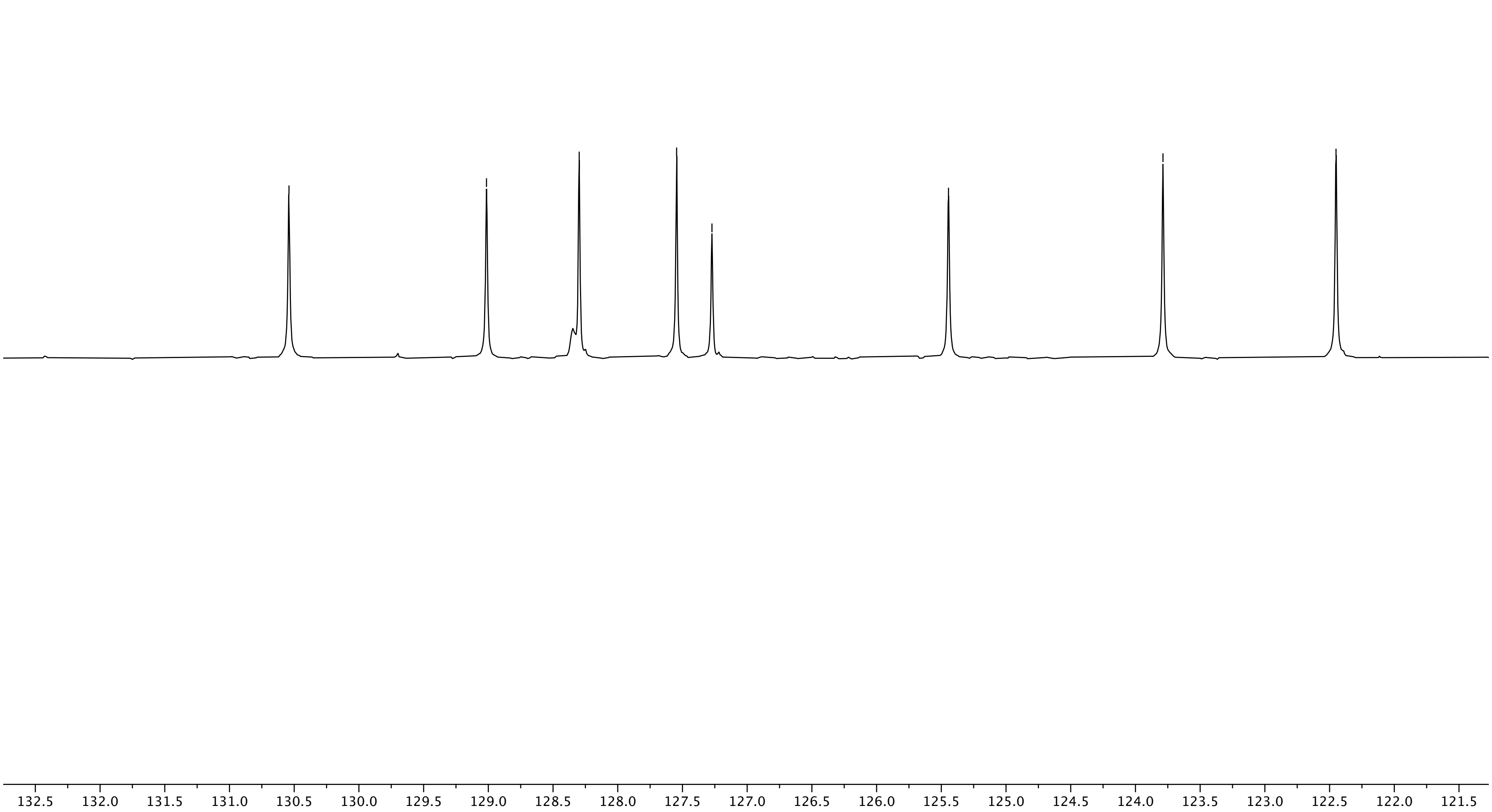

2-(2-(2-(Benzyloxy)ethyl)-6,6-dimethylbicyclo[3.1.1]heptan-3-yl)-4-methylquinoline (22)

Dept–135 (101 MHz, C_6D_6)

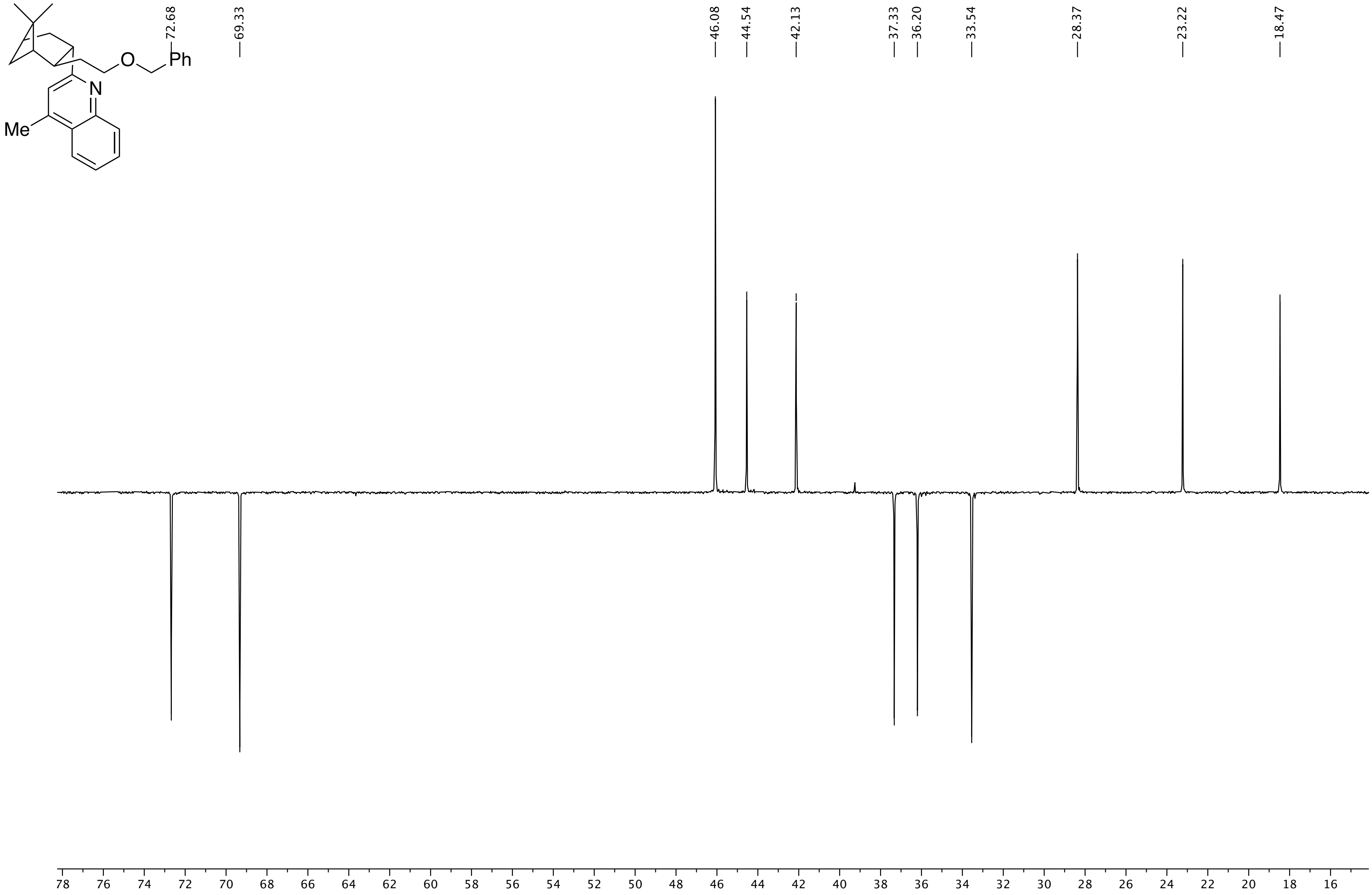

2-(2-(2-(Benzyloxy)ethyl)-6,6-dimethylbicyclo[3.1.1]heptan-3-yl)-4-methylquinoline (22)

COSY (400 MHz, C_6D_6)

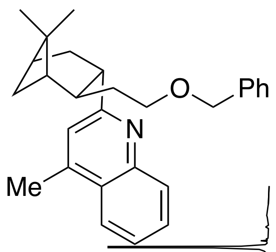

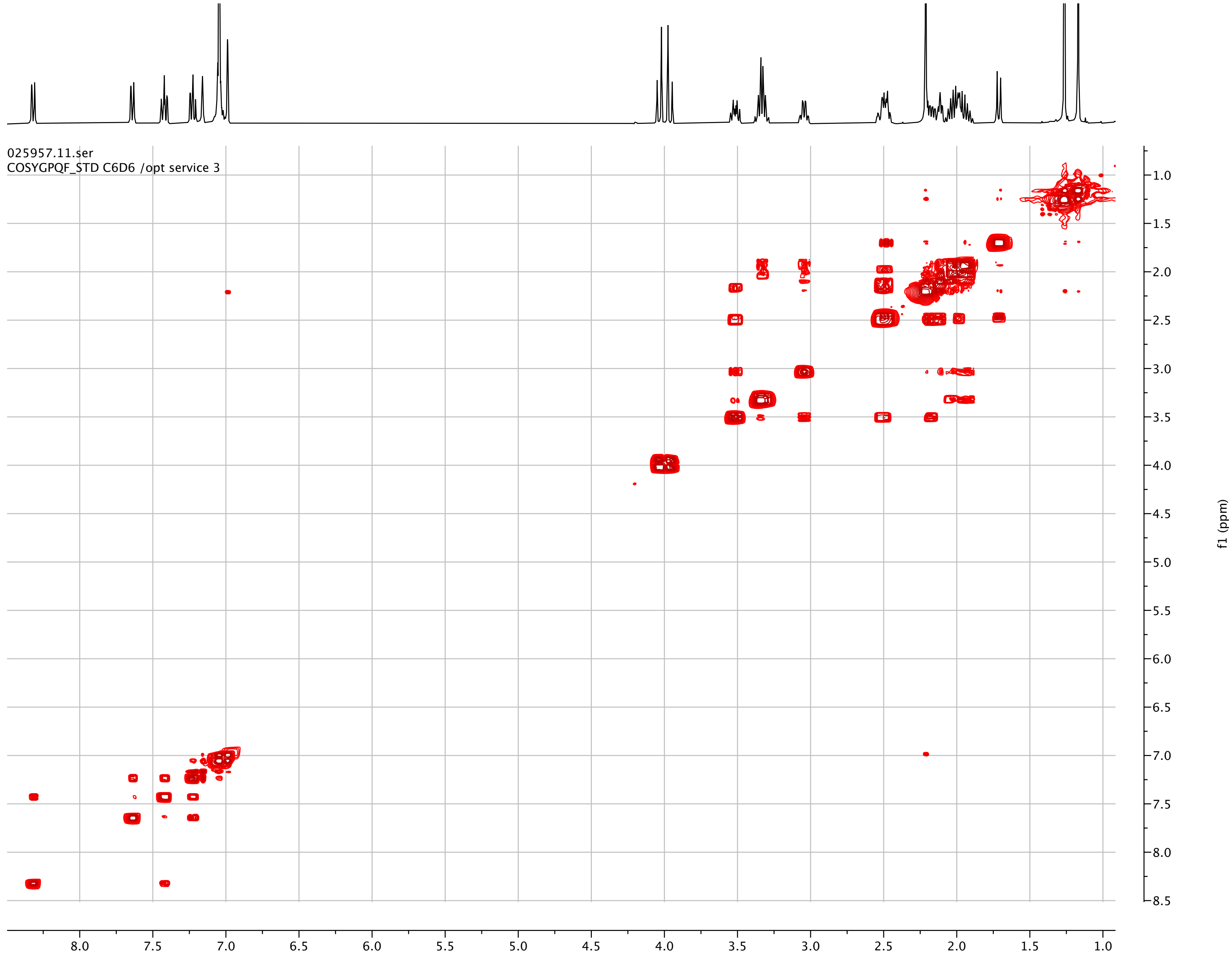

2-(2-(2-(Benzyloxy)ethyl)-6,6-dimethylbicyclo[3.1.1]heptan-3-yl)-4-methylquinoline (22)

COSY (400 MHz, C_6D_6)

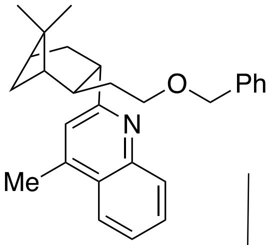

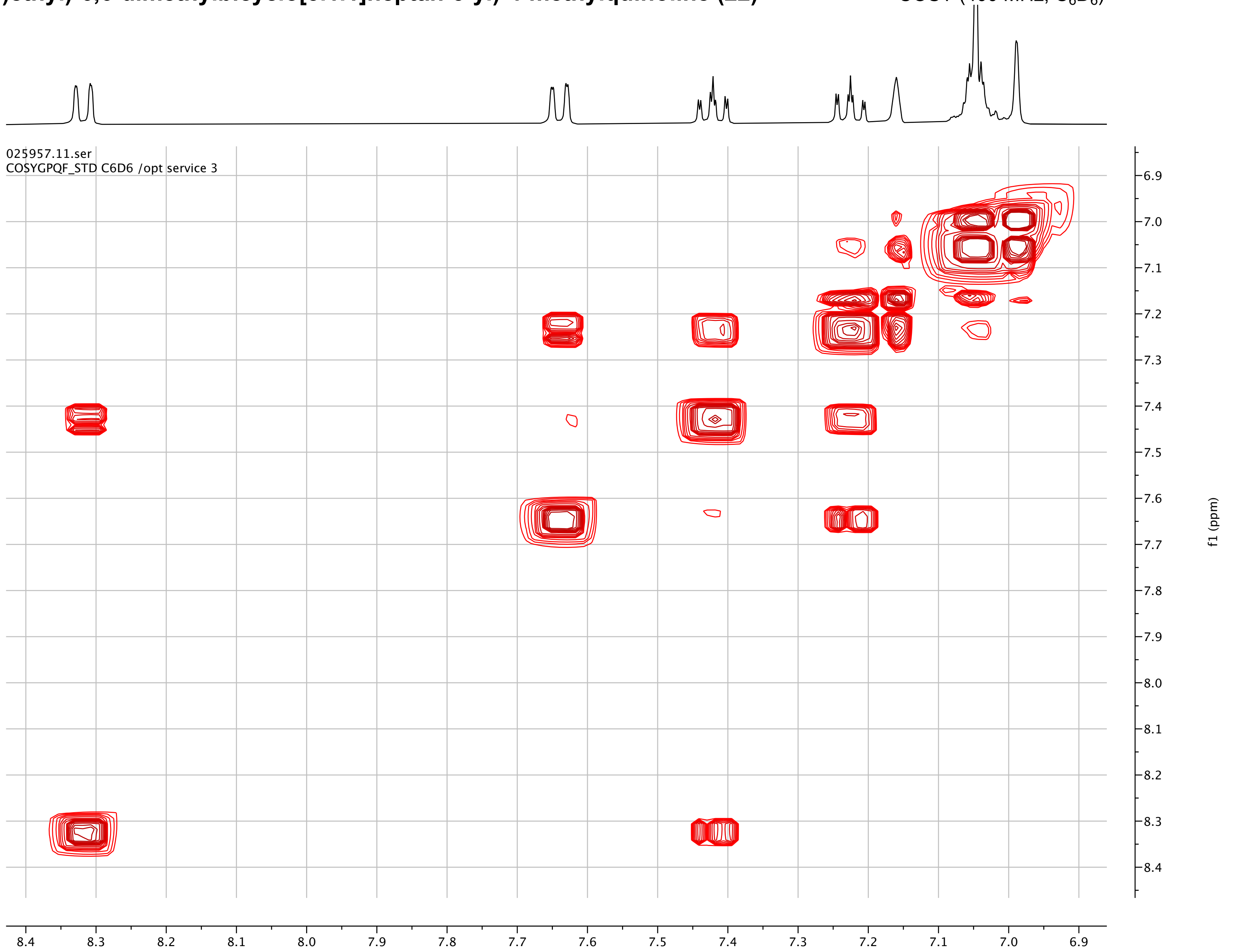

2-(2-(2-(Benzyloxy)ethyl)-6,6-dimethylbicyclo[3.1.1]heptan-3-yl)-4-methylquinoline (22)

COSY (400 MHz, C_6D_6)

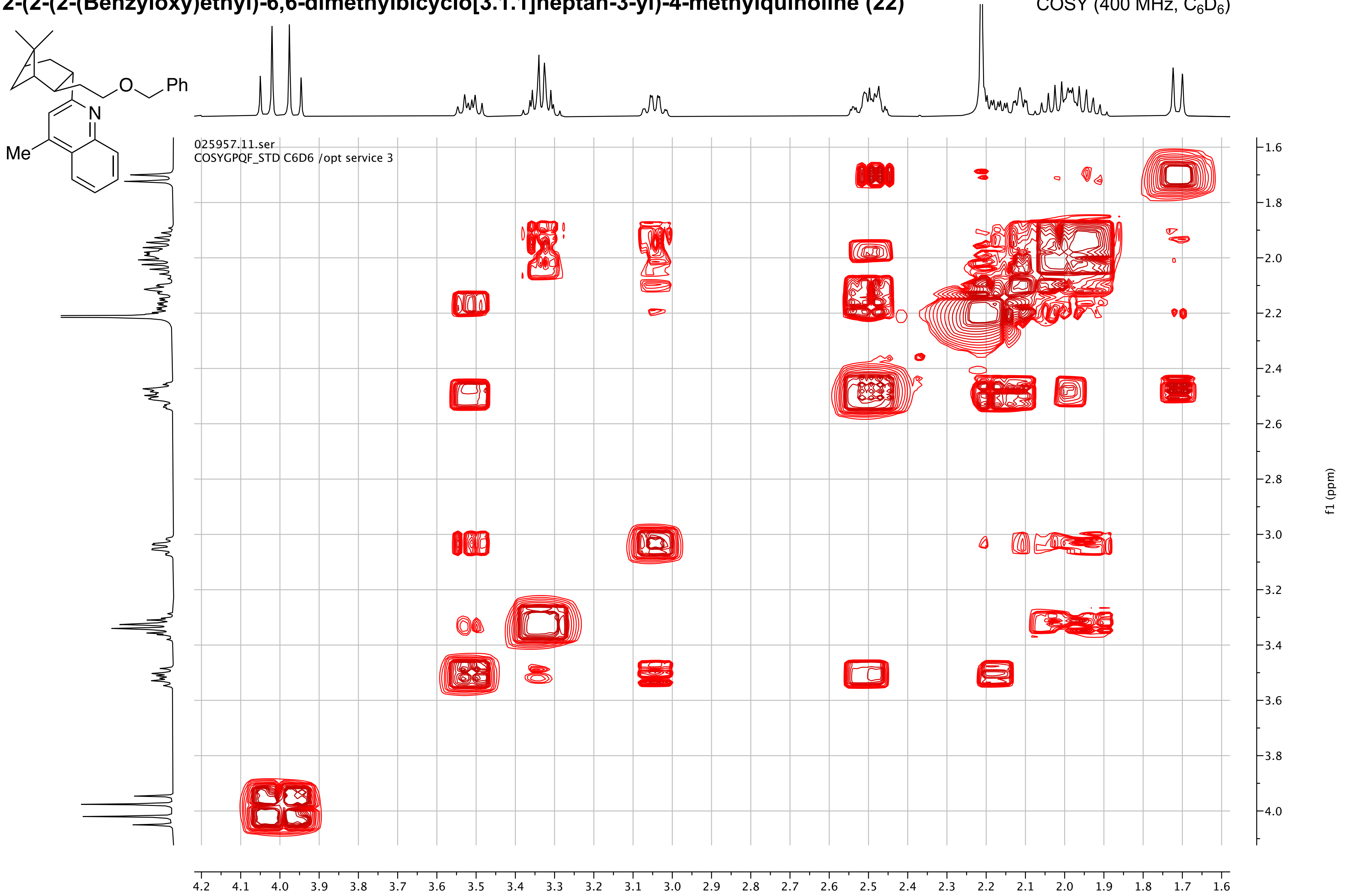

2-(2-(2-(Benzyloxy)ethyl)-6,6-dimethylbicyclo[3.1.1]heptan-3-yl)-4-methylquinoline (22)

HSQC (400 MHz, C_6D_6)

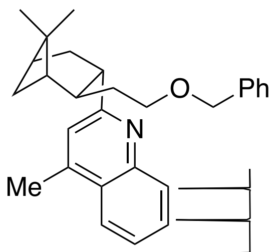

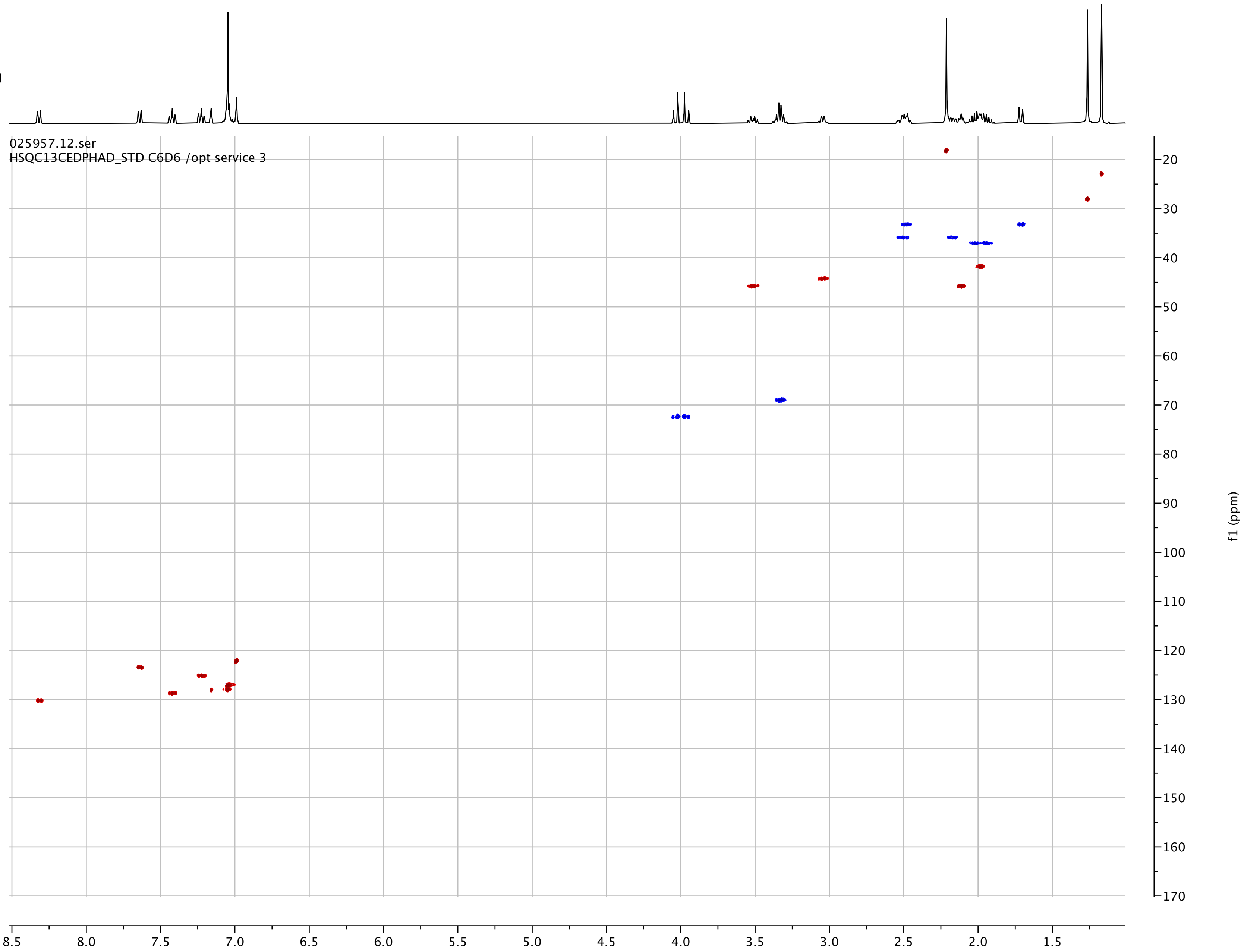

2-(2-(2-(Benzyloxy)ethyl)-6,6-dimethylbicyclo[3.1.1]heptan-3-yl)-4-methylquinoline (22)

HSQC (400 MHz, C_6D_6)

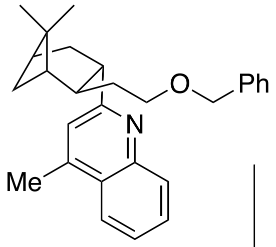

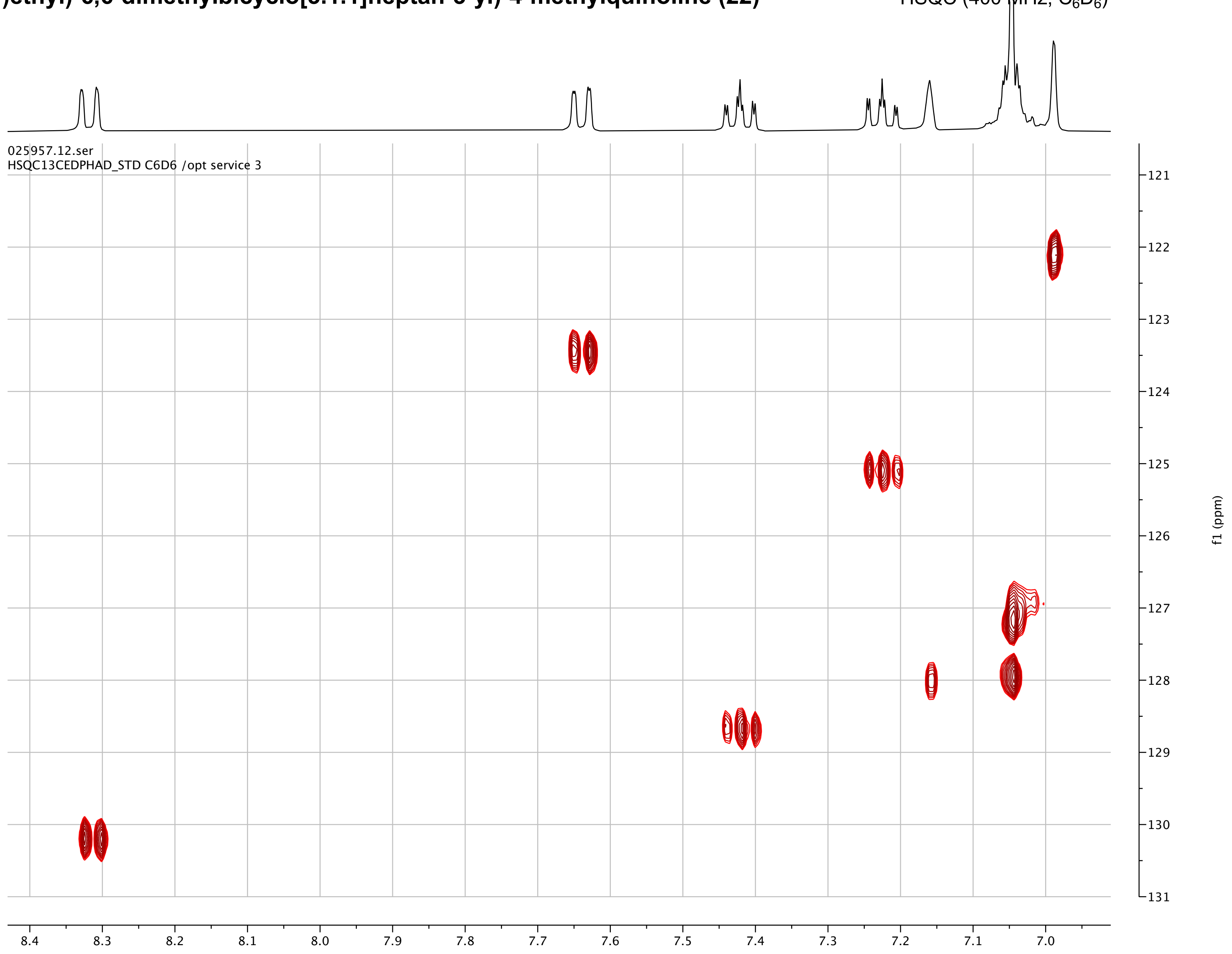

2-(2-(2-(Benzyloxy)ethyl)-6,6-dimethylbicyclo[3.1.1]heptan-3-yl)-4-methylquinoline (22)

HSQC (400 MHz, C_6D_6)

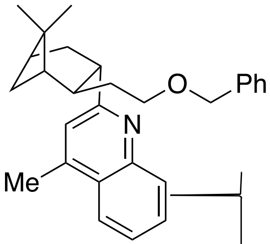

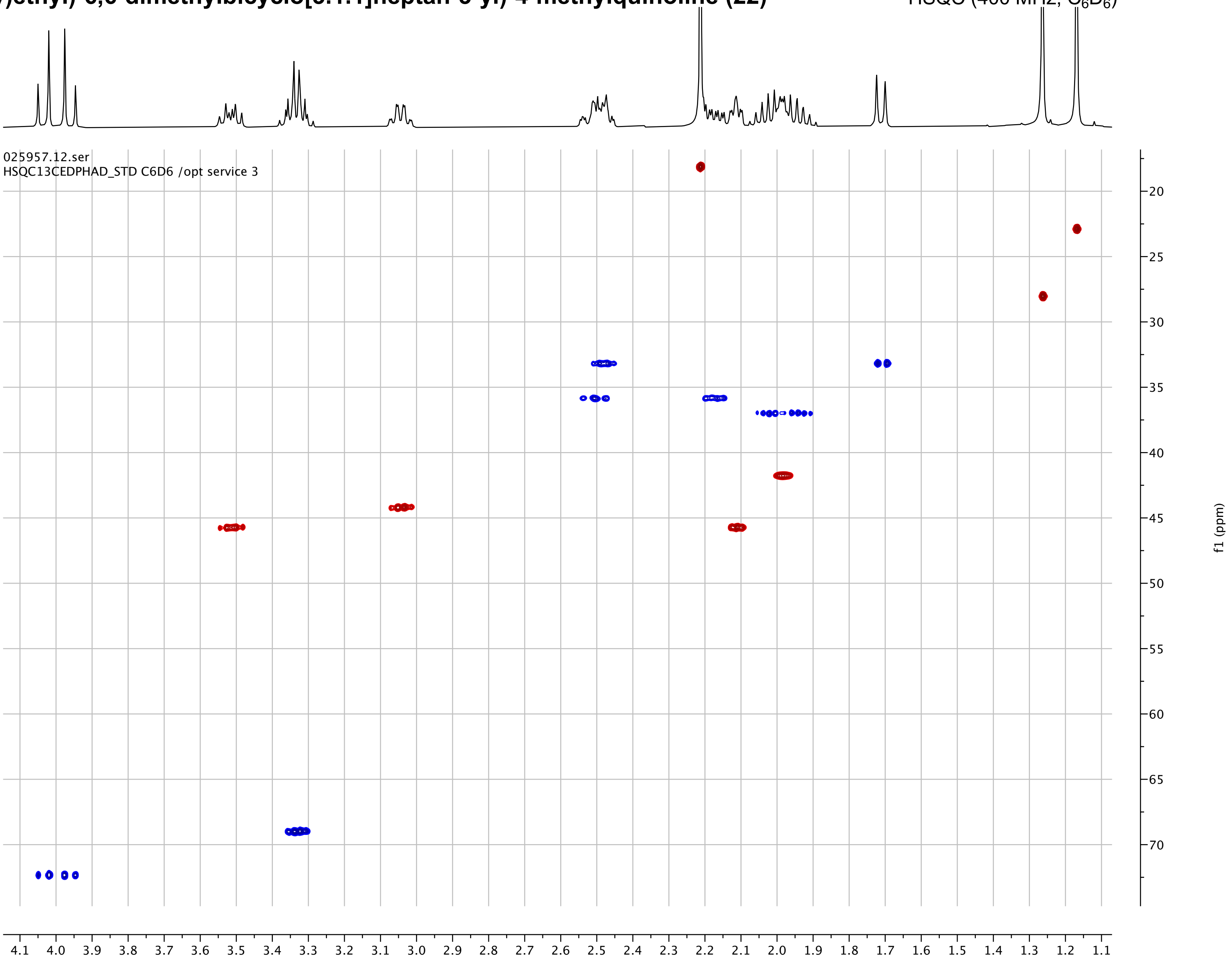

2-(2-(2-(Benzyloxy)ethyl)-6,6-dimethylbicyclo[3.1.1]heptan-3-yl)-4-methylquinoline (22)

HMBC (400 MHz, C_6D_6)

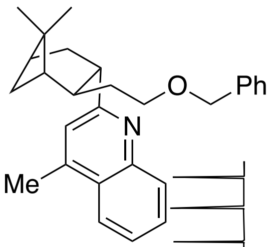

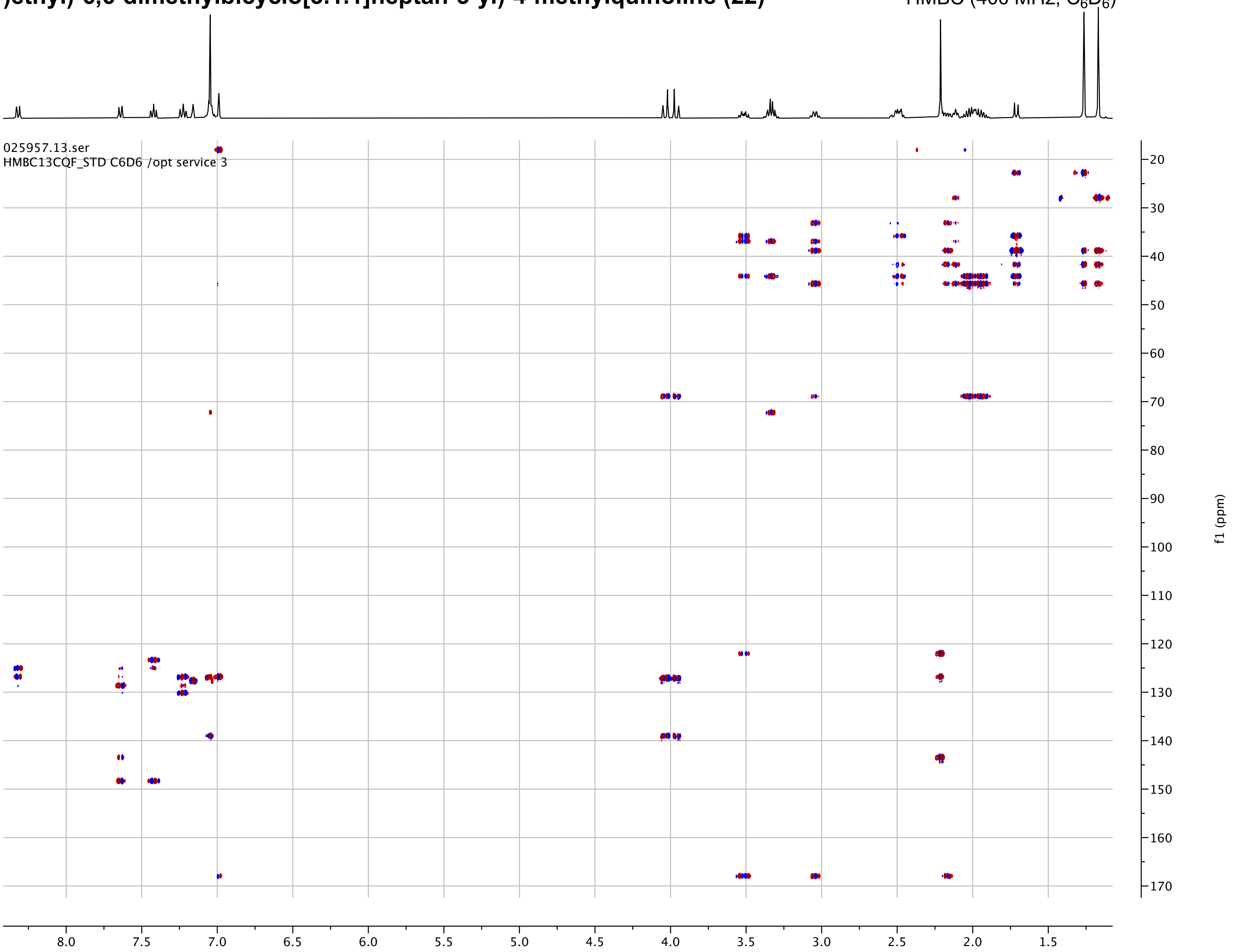

2-(2-(2-(Benzyloxy)ethyl)-6,6-dimethylbicyclo[3.1.1]heptan-3-yl)-4-methylquinoline (22)

HMBC (400 MHz, C_6D_6)

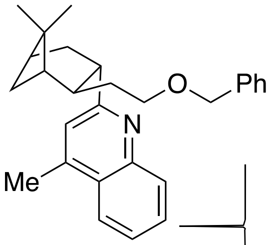

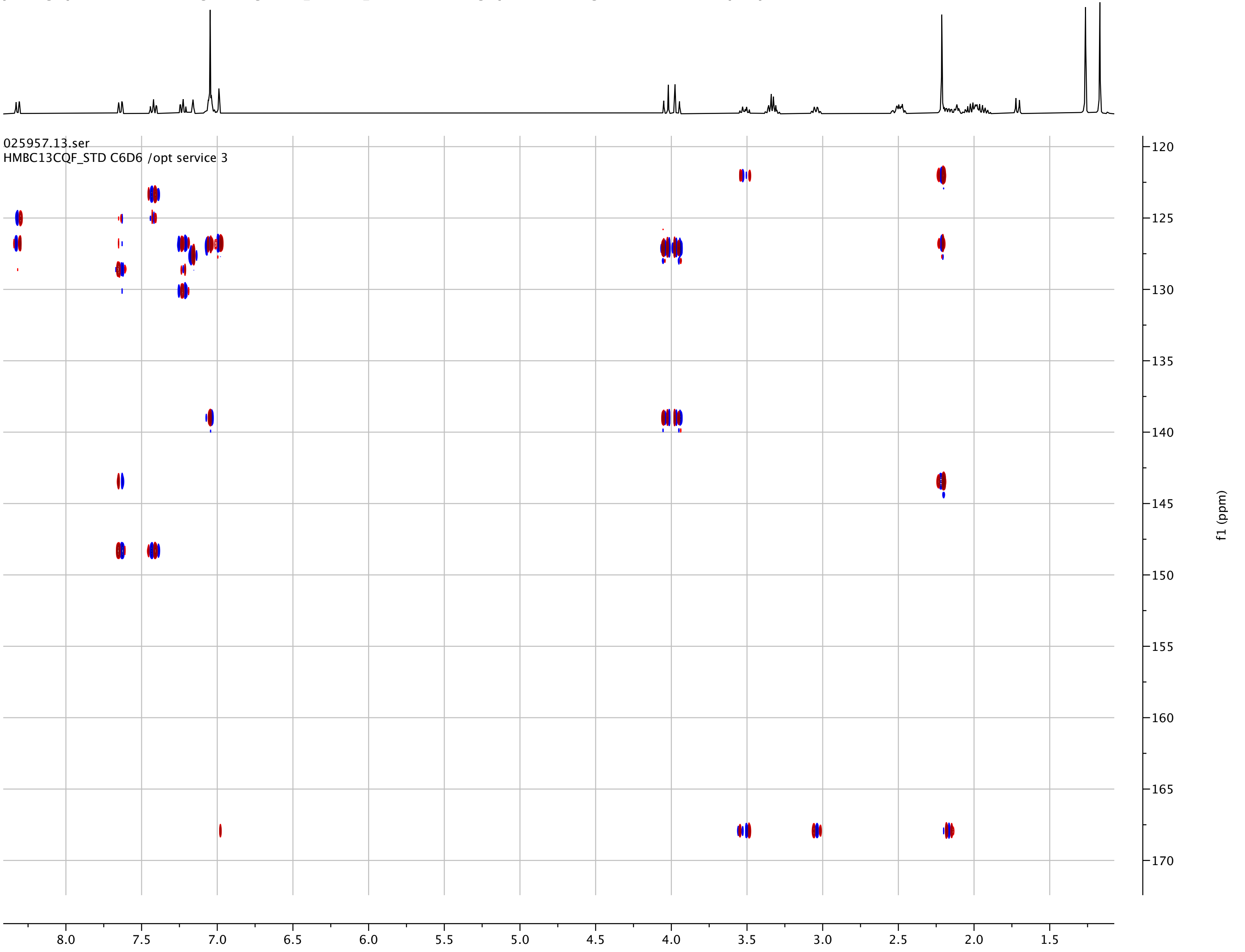

2-(2-(2-(Benzyloxy)ethyl)-6,6-dimethylbicyclo[3.1.1]heptan-3-yl)-4-methylquinoline (22)

HMBC (400 MHz, C_6D_6)

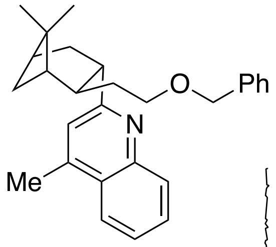

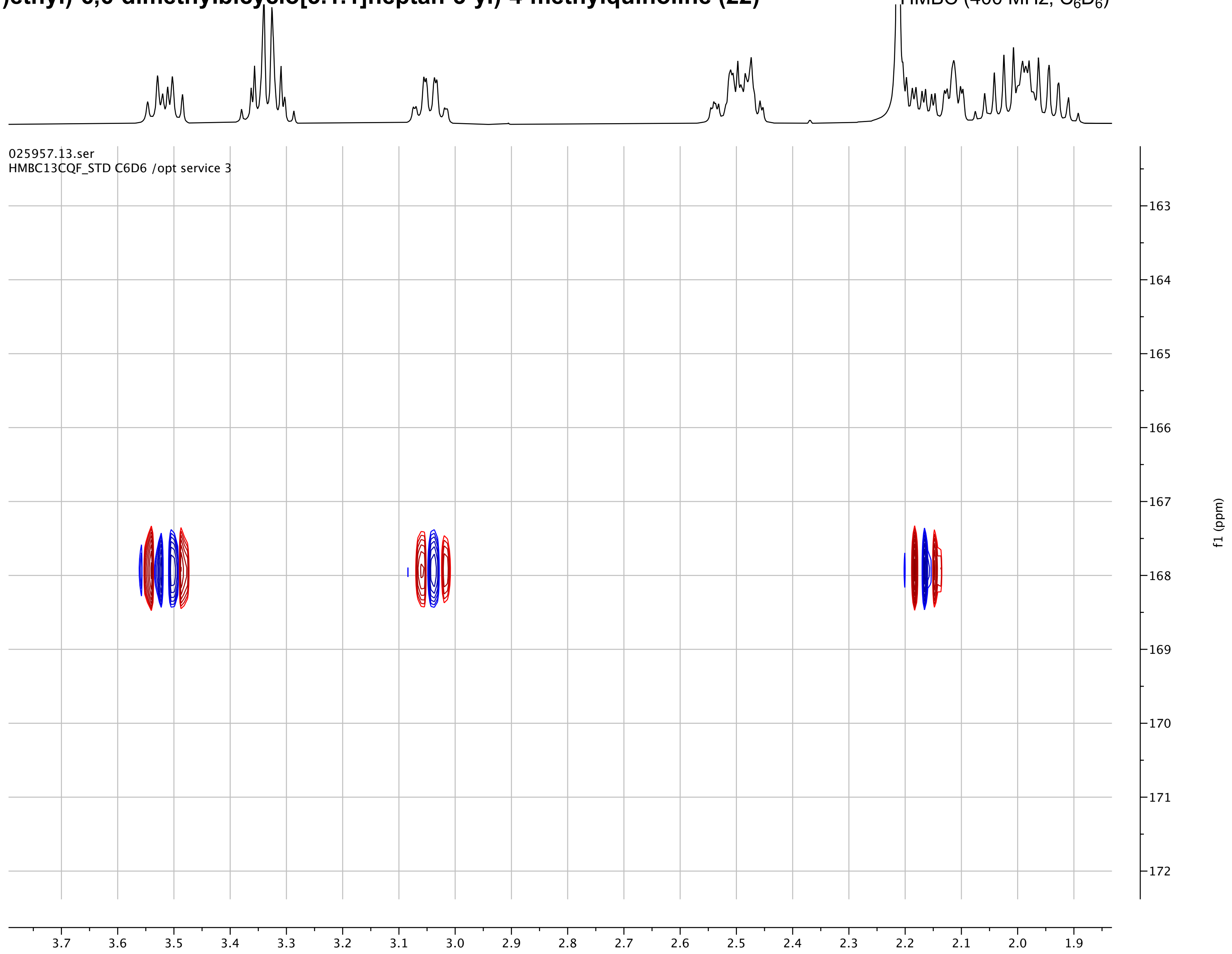

2-(2-(2-(Benzyloxy)ethyl)-6,6-dimethylbicyclo[3.1.1]heptan-3-yl)-4-methylquinoline (22)

HMBC (400 MHz, C_6D_6)

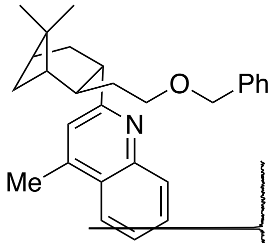

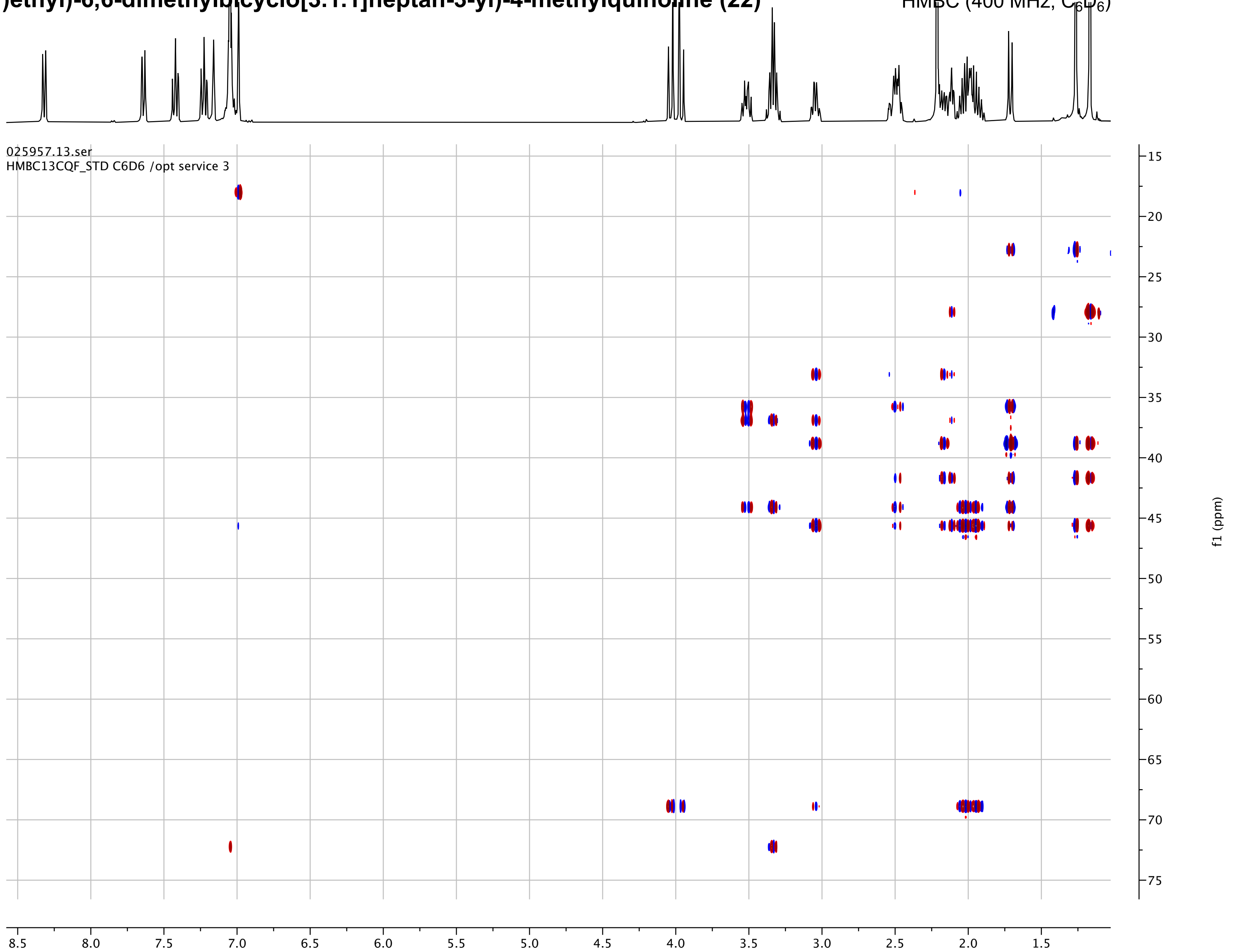

2-(2-(2-(Benzyloxy)ethyl)-6,6-dimethylbicyclo[3.1.1]heptan-3-yl)-4-methylquinoline (22)

^{1}H–^{1}H NOESY(400 MHz, C_6D_6)

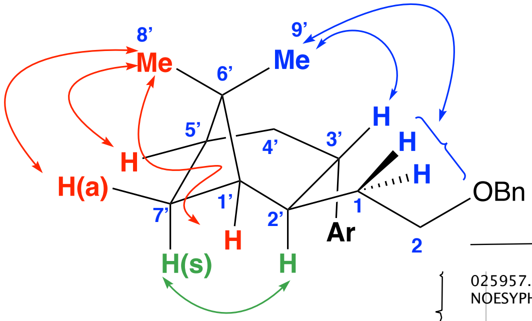

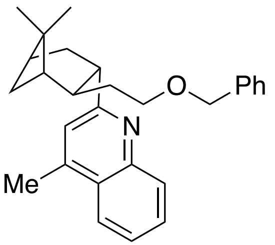

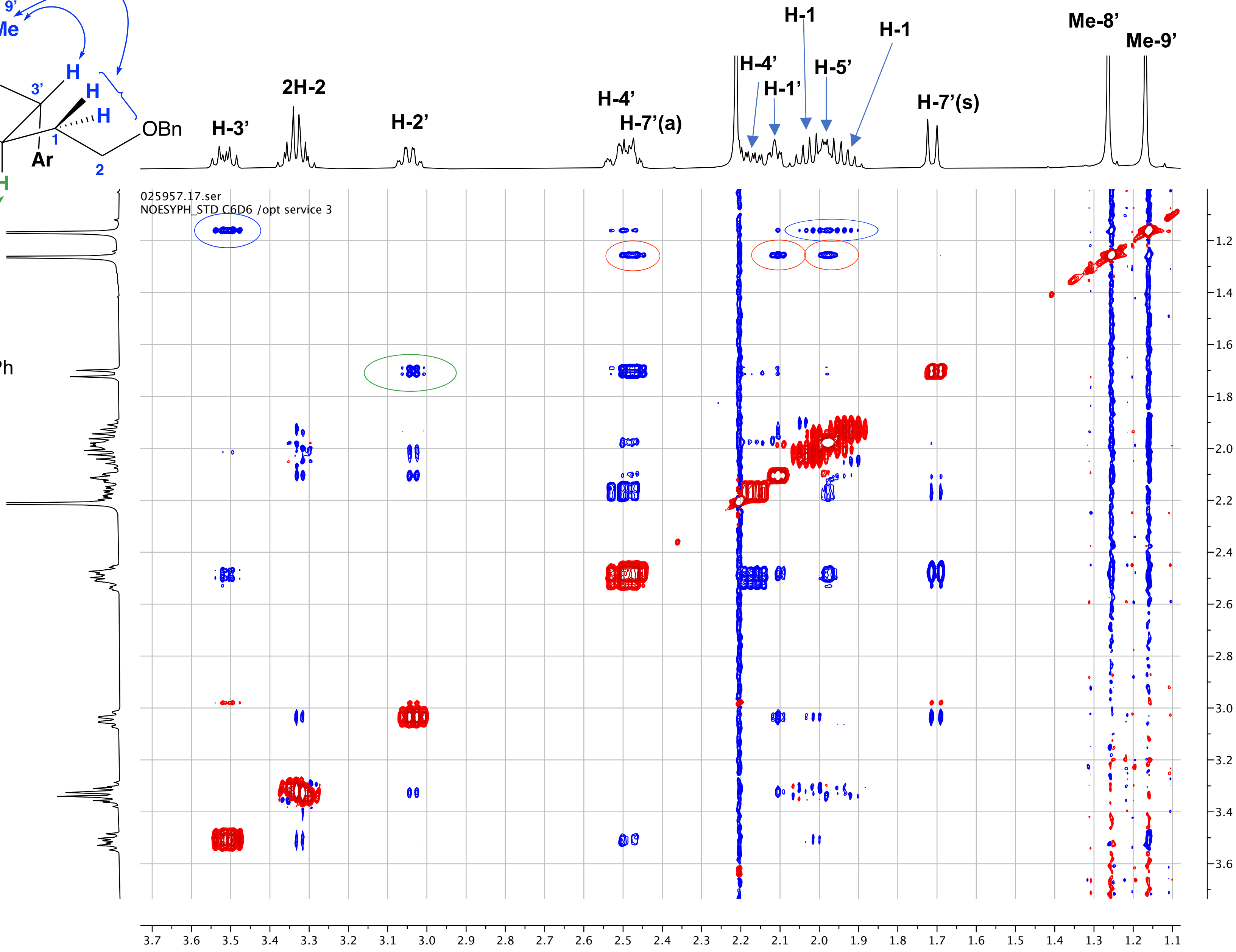

1-(2-(2-(Benzyloxy)ethyl)-6,6-dimethylbicyclo[3.1.1]heptan-3-yl)isoquinoline (23)

^{1}H-NMR (400 MHz, $CDCl_3$)

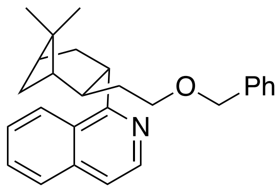

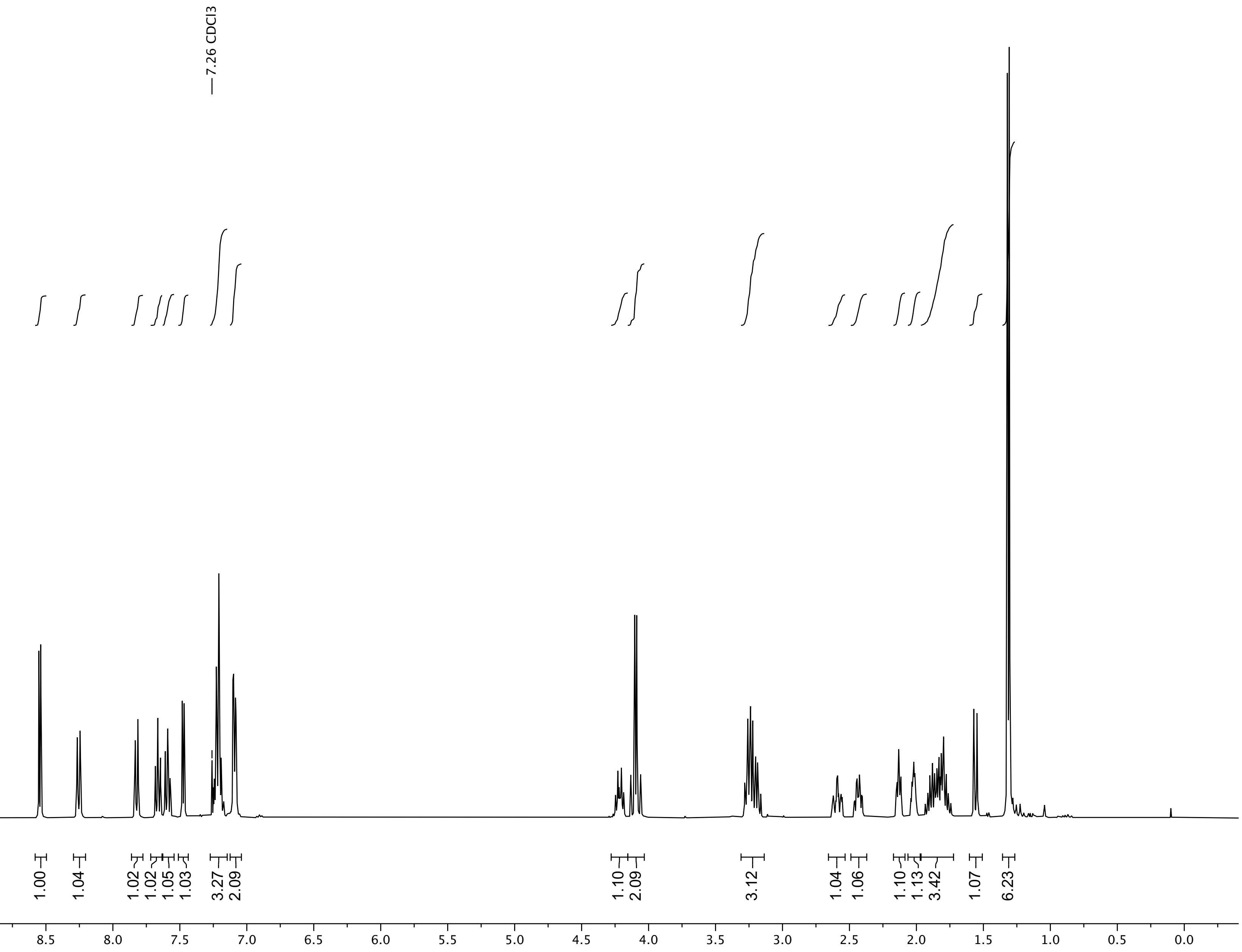

1-(2-(2-(Benzyloxy)ethyl)-6,6-dimethylbicyclo[3.1.1]heptan-3-yl)isoquinoline (23)

^{1}H-NMR (400 MHz, $CDCl_3$)

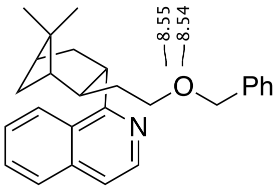

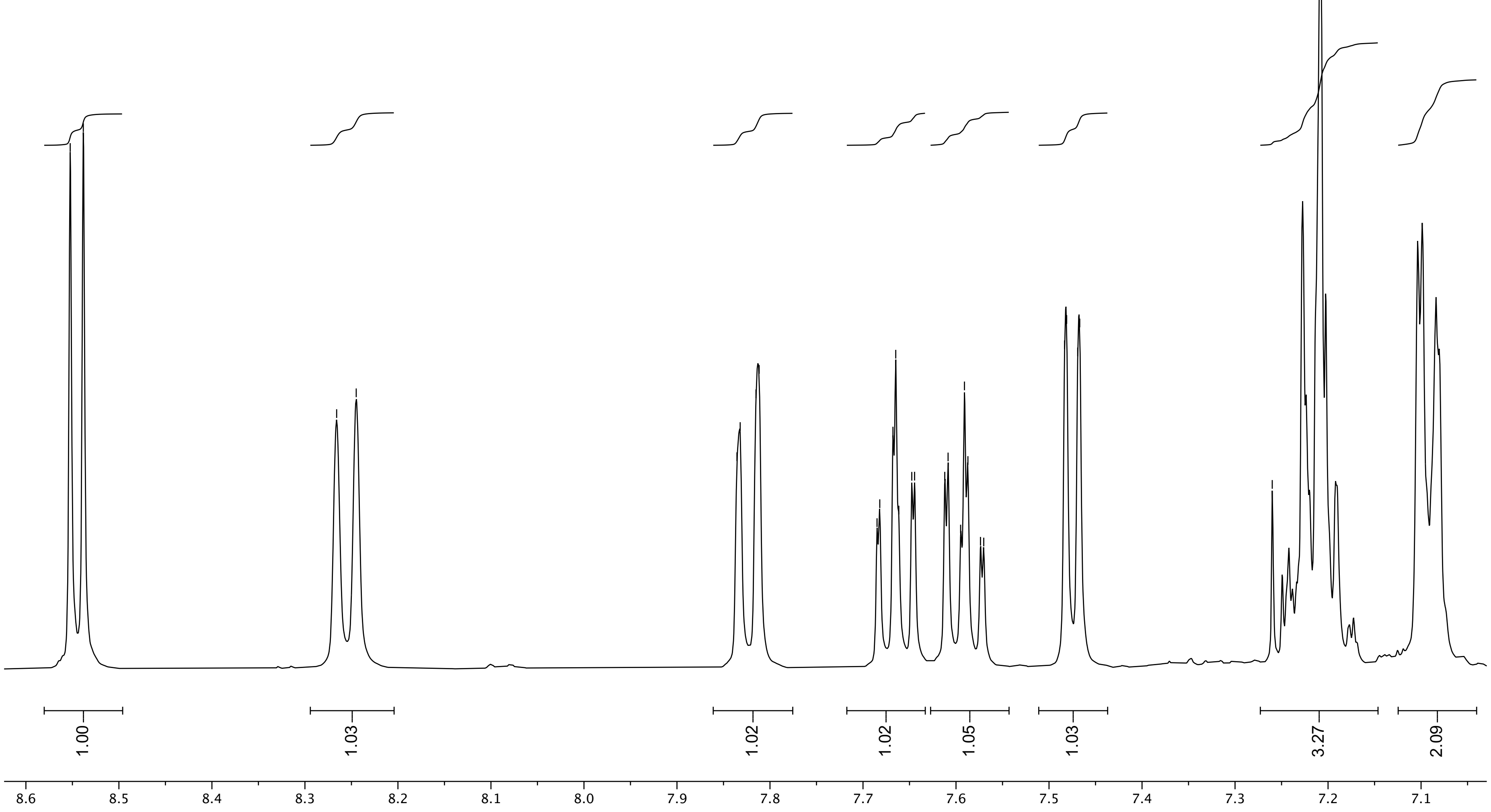

1-(2-(2-(Benzyloxy)ethyl)-6,6-dimethylbicyclo[3.1.1]heptan-3-yl)isoquinoline (23)

^{1}H-NMR (400 MHz, $CDCl_3$)

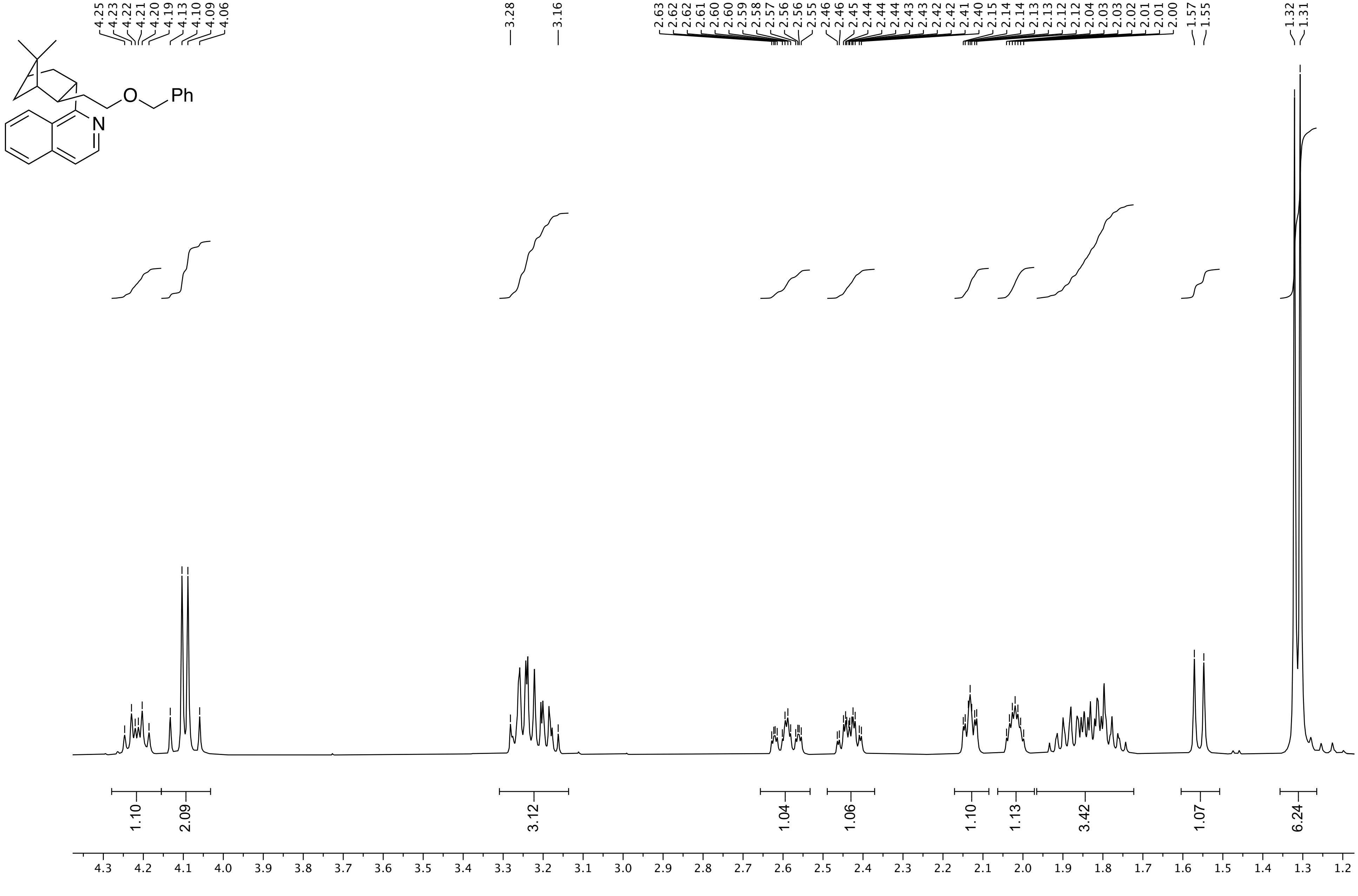

1-(2-(2-(Benzyloxy)ethyl)-6,6-dimethylbicyclo[3.1.1]heptan-3-yl)isoquinoline (23)

^{1}H-NMR (400 MHz, $CDCl_3$)

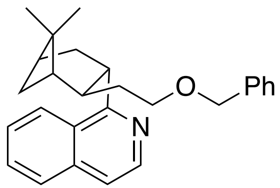

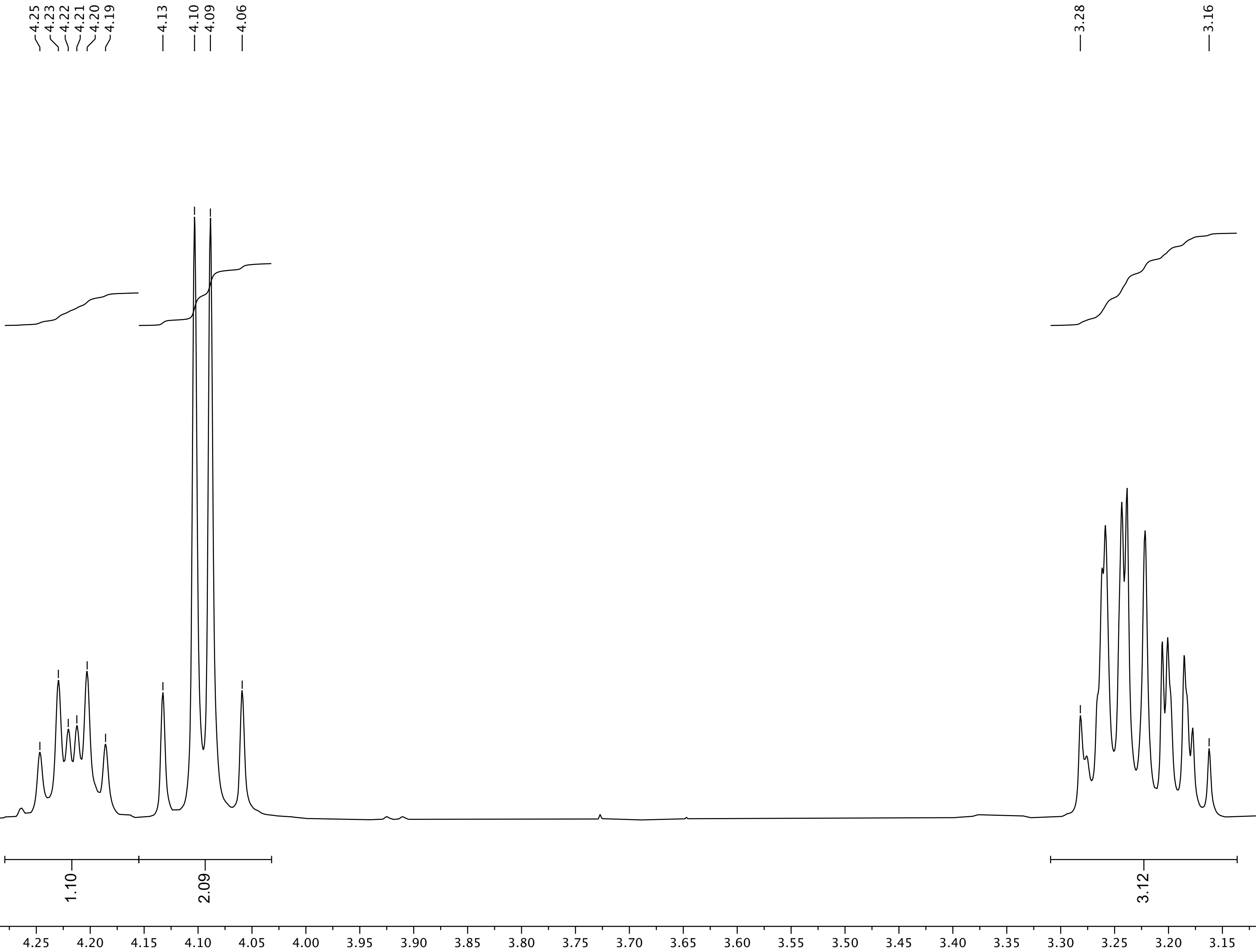

1-(2-(2-(Benzyloxy)ethyl)-6,6-dimethylbicyclo[3.1.1]heptan-3-yl)isoquinoline (23)

^{1}H-NMR (400 MHz, $CDCl_3$)

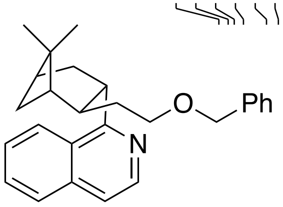

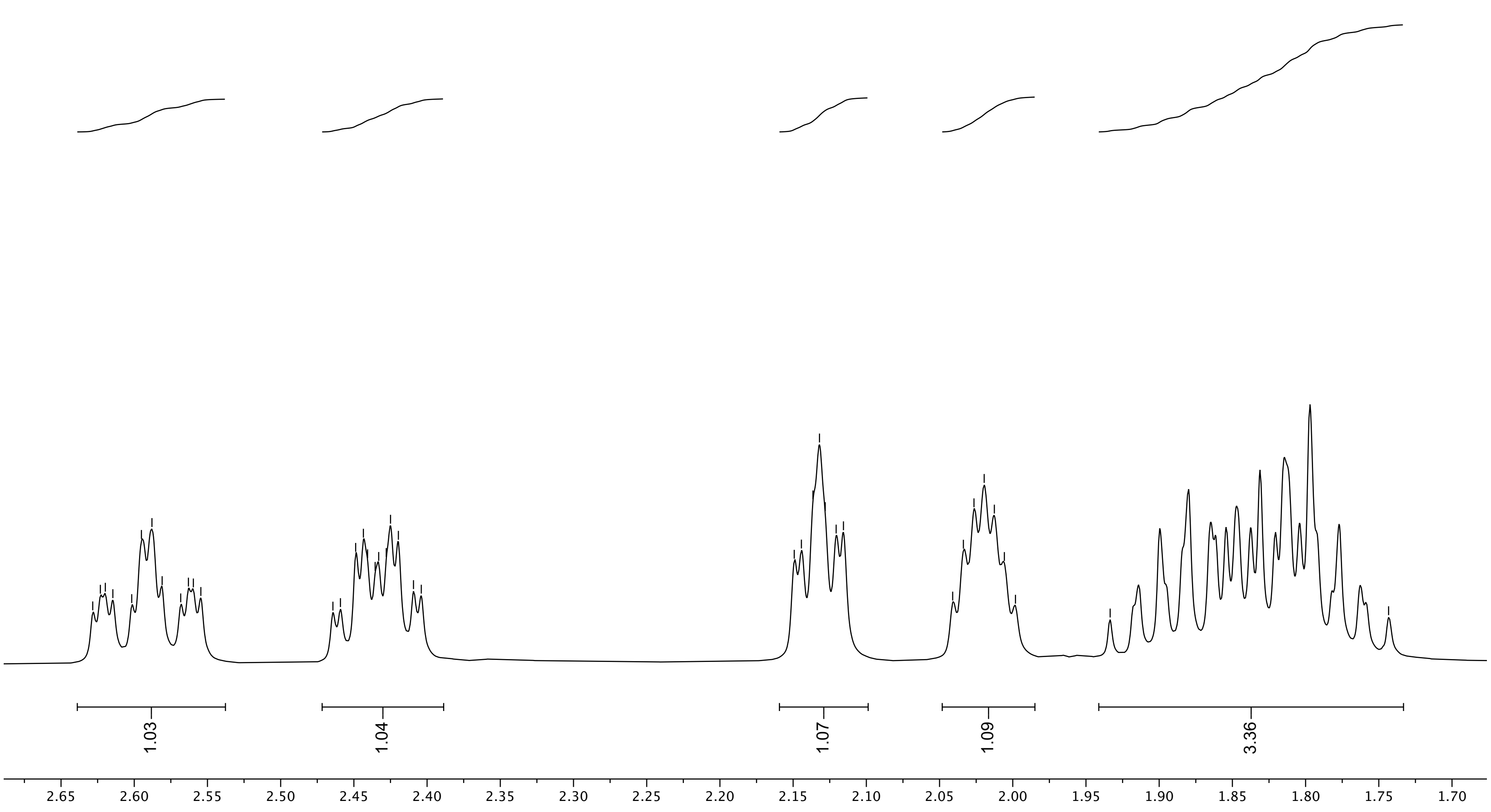

1-(2-(2-(Benzyloxy)ethyl)-6,6-dimethylbicyclo[3.1.1]heptan-3-yl)isoquinoline (23)

^{1}H-NMR (400 MHz, $CDCl_3$)

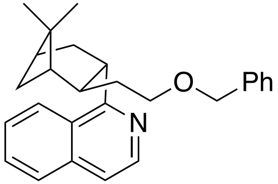

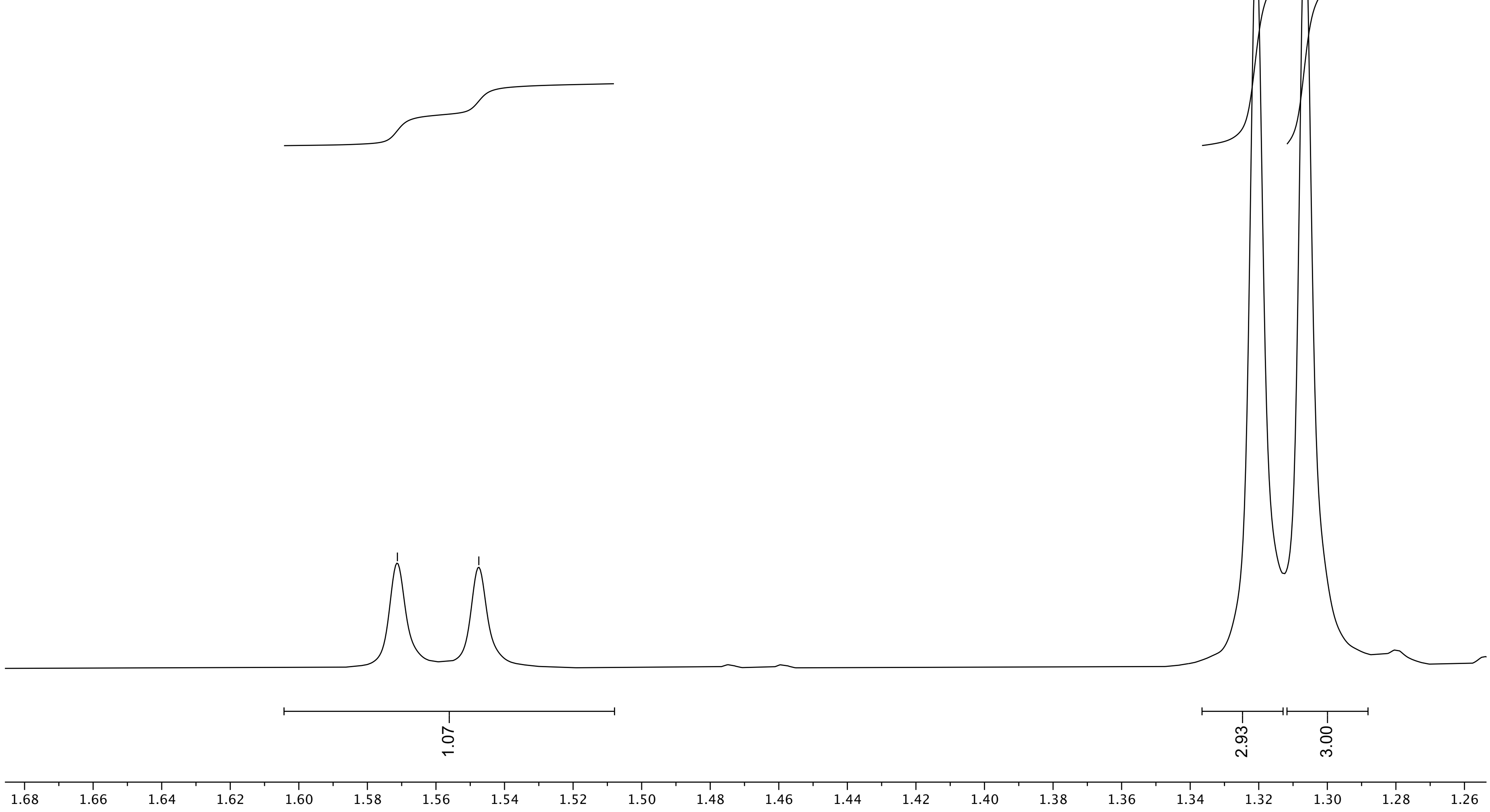

1-(2-(2-(Benzyloxy)ethyl)-6,6-dimethylbicyclo[3.1.1]heptan-3-yl)isoquinoline (23)

^{13}C-NMR (101 MHz, $CDCl_3$)

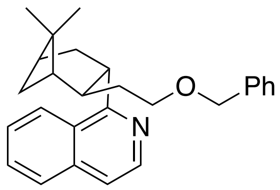

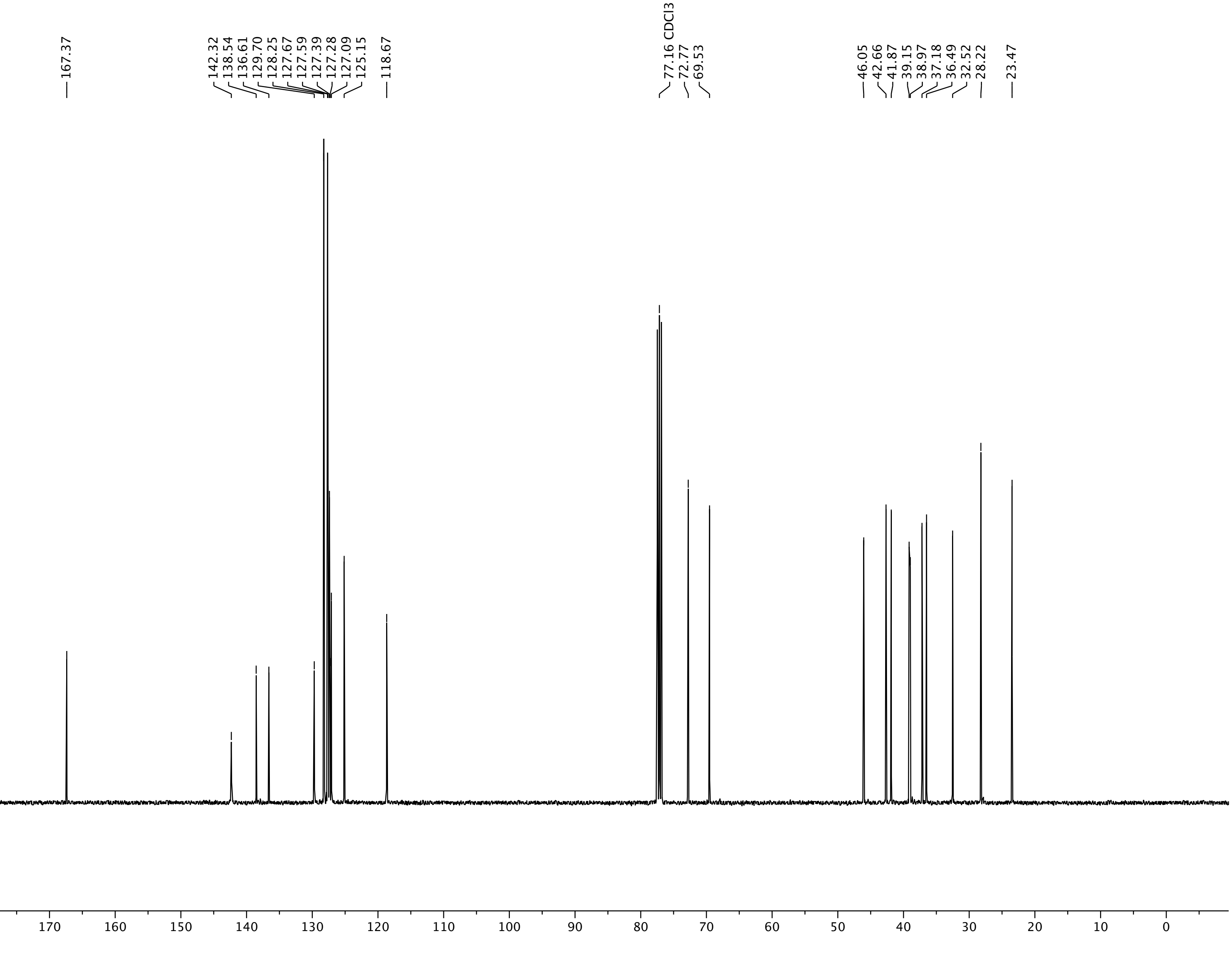

1-(2-(2-(Benzyloxy)ethyl)-6,6-dimethylbicyclo[3.1.1]heptan-3-yl)isoquinoline (23)

^{13}C-NMR (101 MHz, $CDCl_3$)

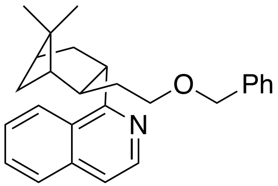

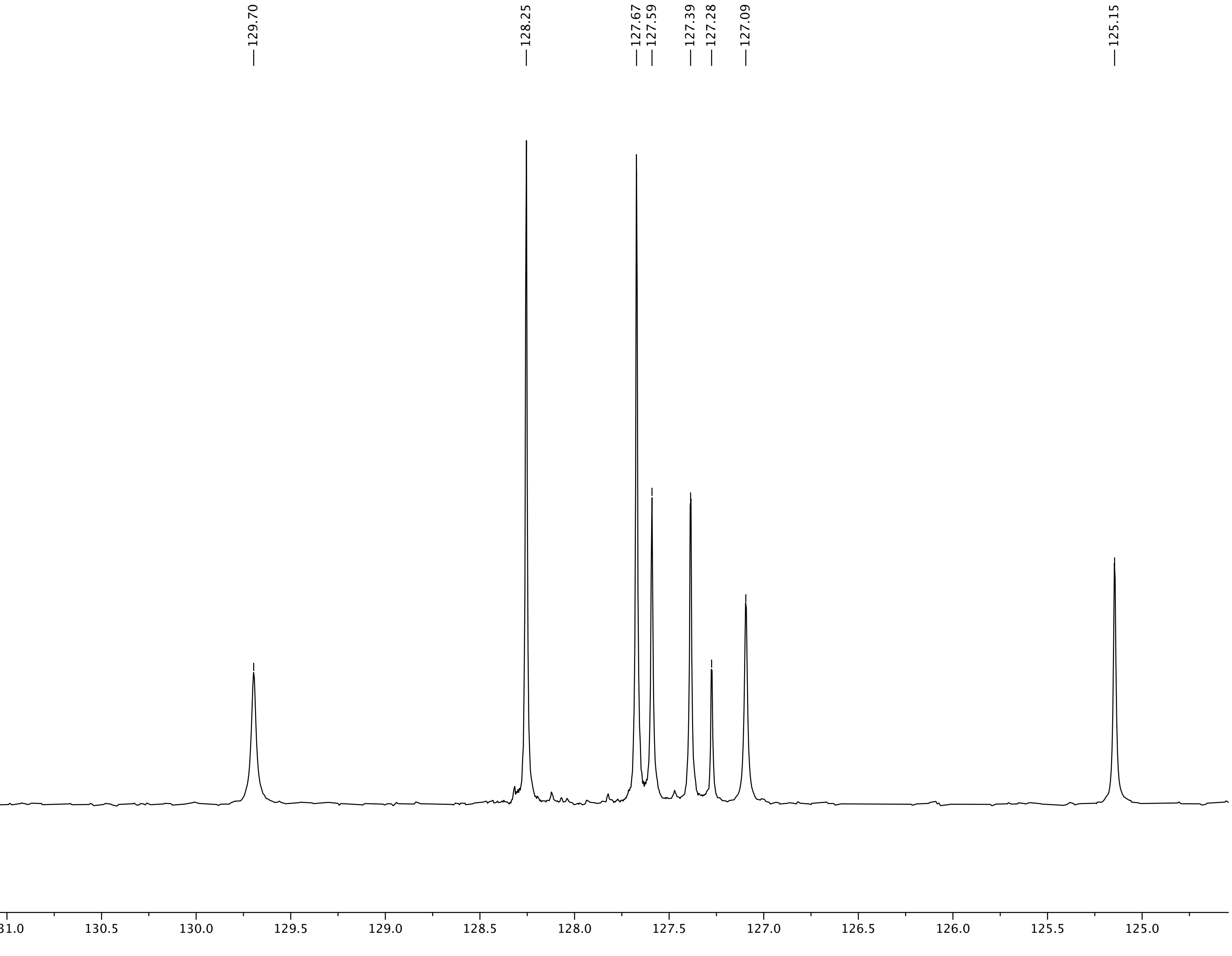

1-(2-(2-(Benzyloxy)ethyl)-6,6-dimethylbicyclo[3.1.1]heptan-3-yl)isoquinoline (23)

^{13}C-NMR (101 MHz, $CDCl_3$)

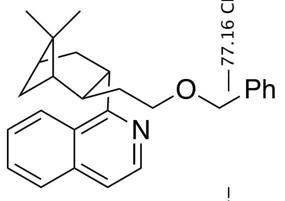

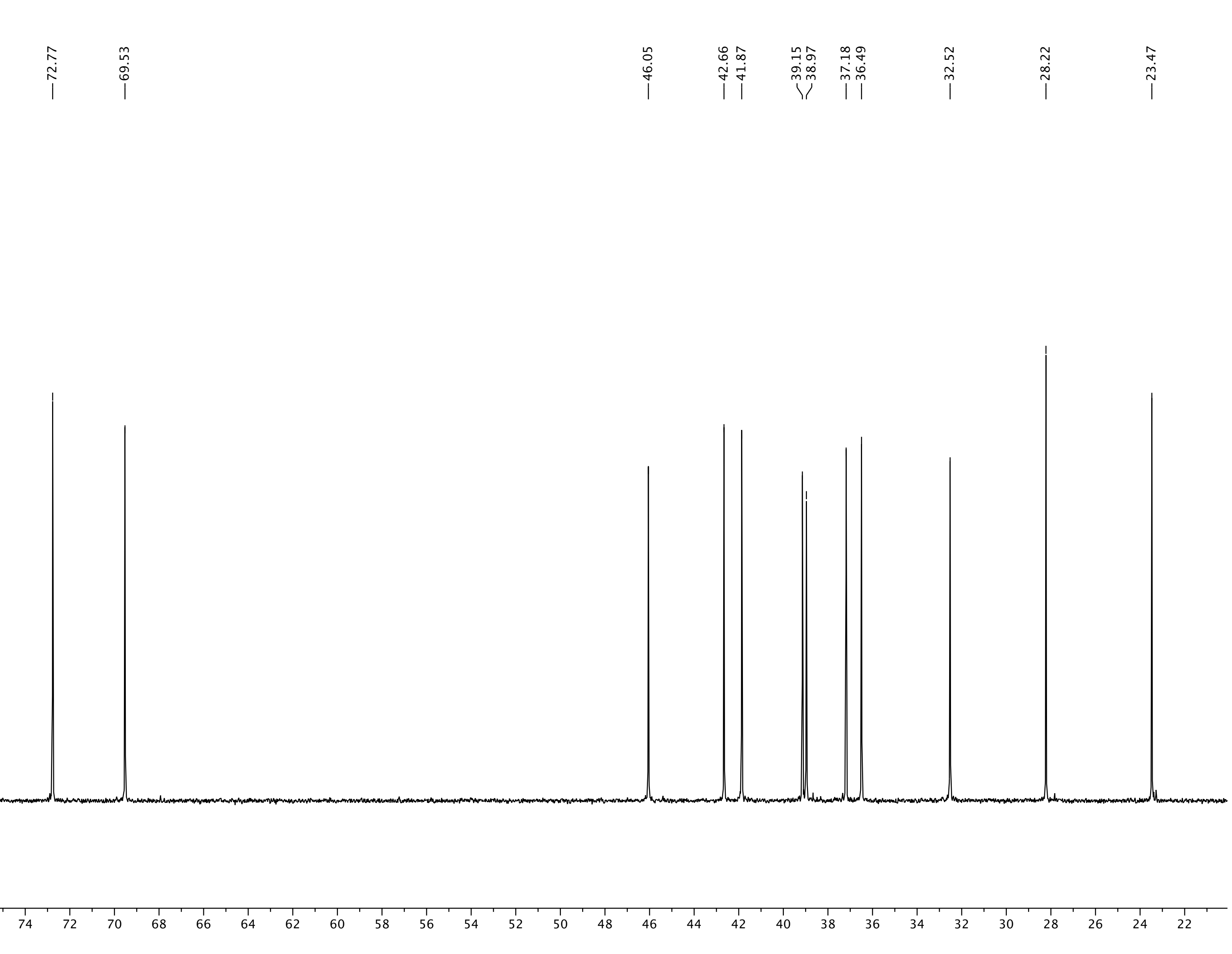

1-(2-(2-(Benzyloxy)ethyl)-6,6-dimethylbicyclo[3.1.1]heptan-3-yl)isoquinoline (23)

Dept-135 (101 MHz, $CDCl_3$)

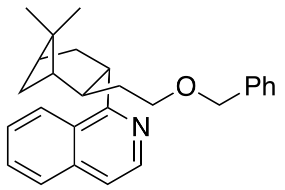

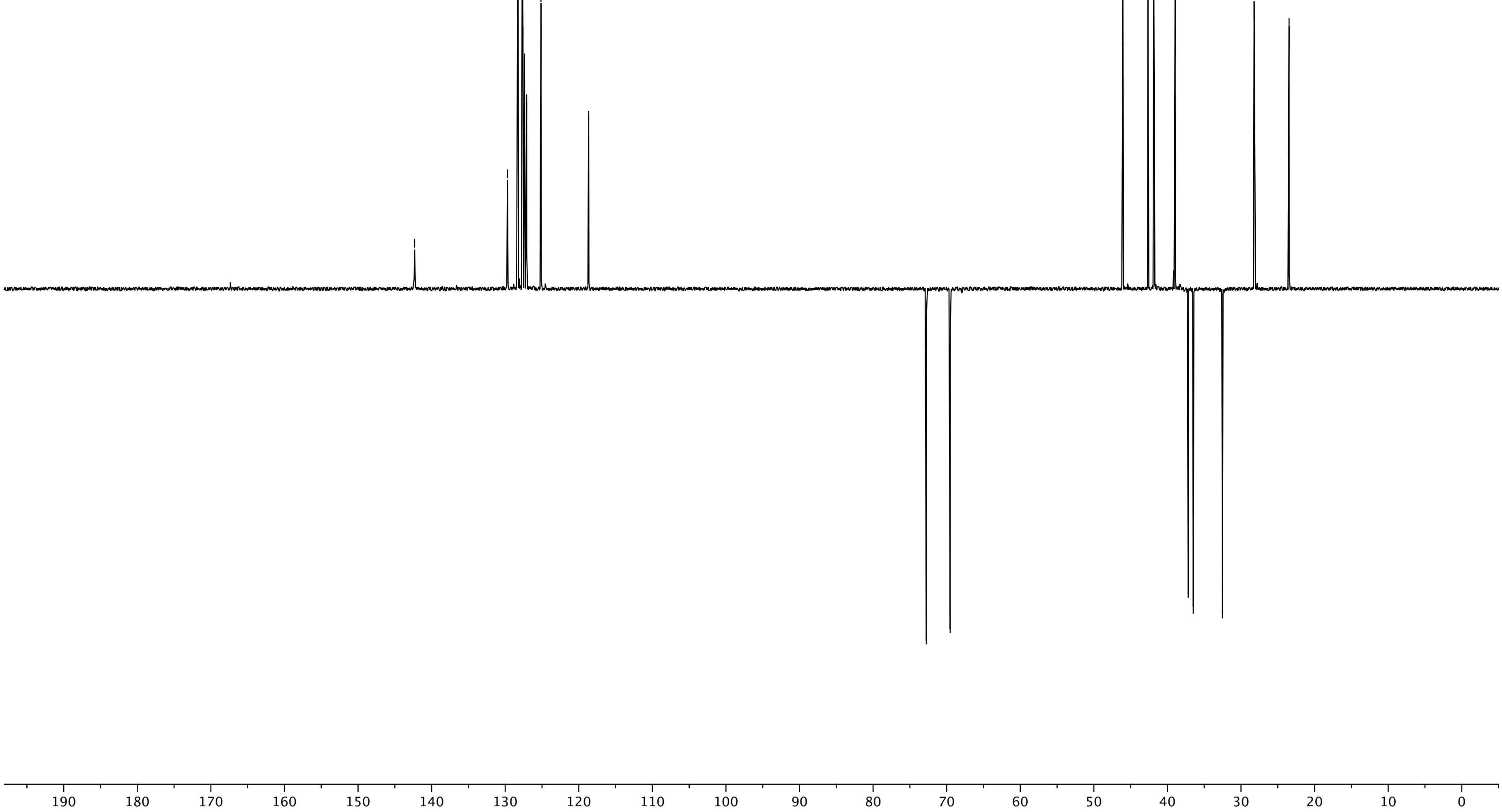

1-(2-(2-(Benzyloxy)ethyl)-6,6-dimethylbicyclo[3.1.1]heptan-3-yl)isoquinoline (23)

Dept-135 (101 MHz, $CDCl_3$)

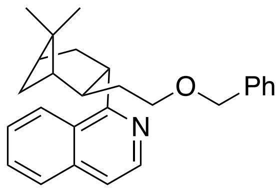

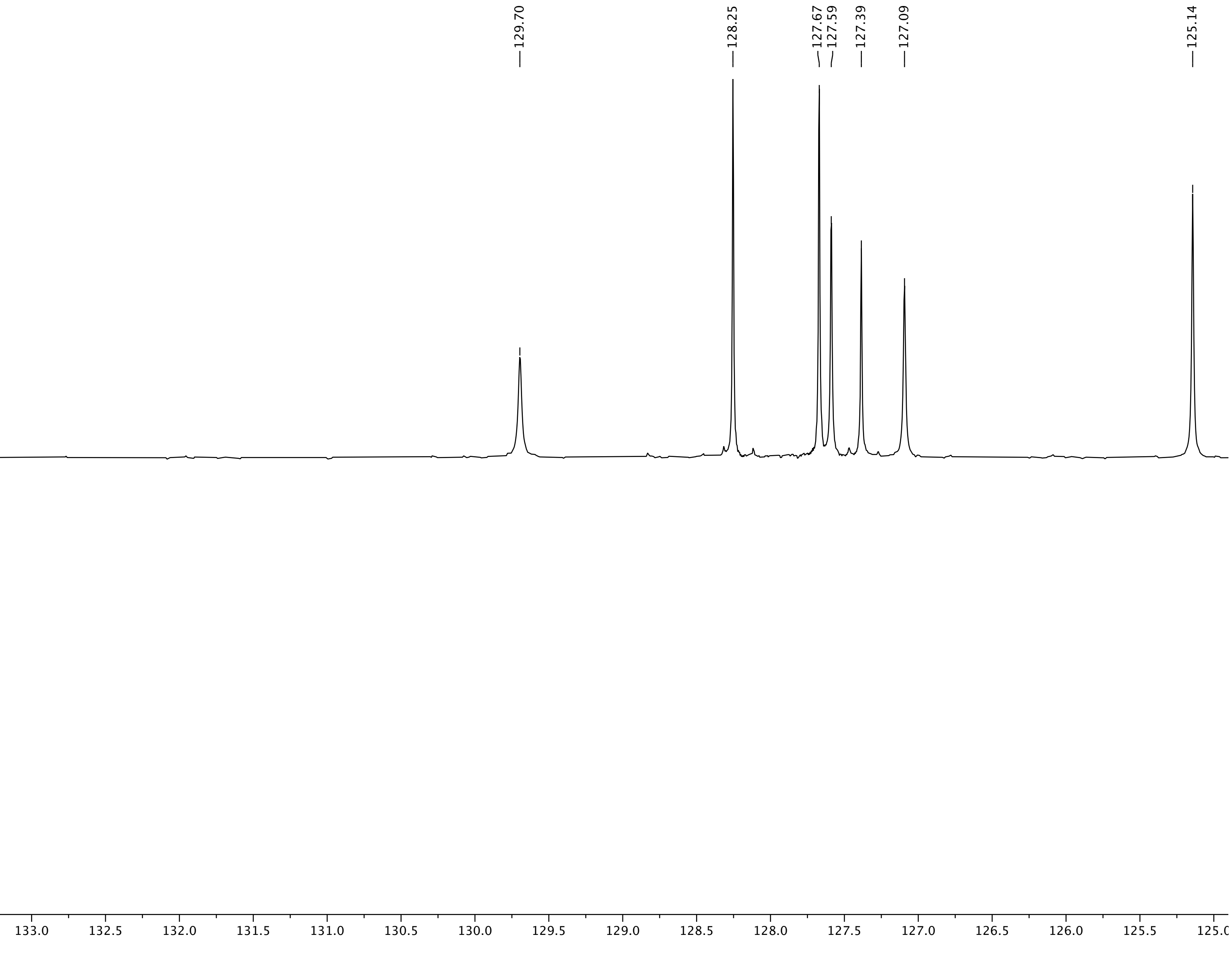

1-(2-(2-(Benzyloxy)ethyl)-6,6-dimethylbicyclo[3.1.1]heptan-3-yl)isoquinoline (23)

Dept-135 (101 MHz, $CDCl_3$)

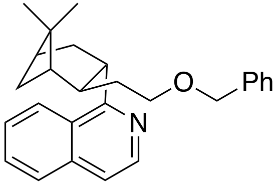

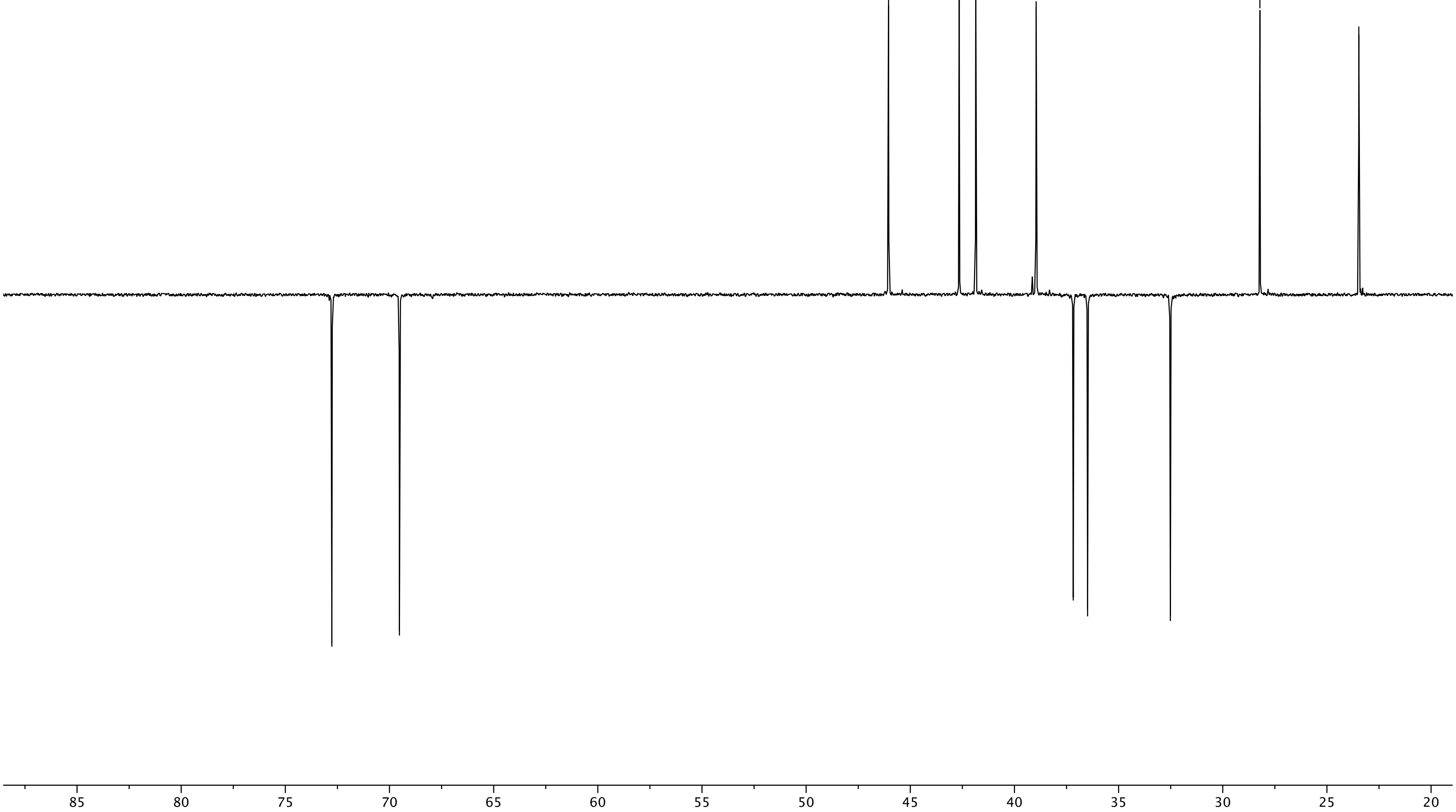

1-(2-(2-(Benzyloxy)ethyl)-6,6-dimethylbicyclo[3.1.1]heptan-3-yl)isoquinoline (23)

^1H-^1H COSY (400 MHz, $CDCl_3$)

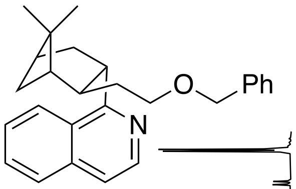

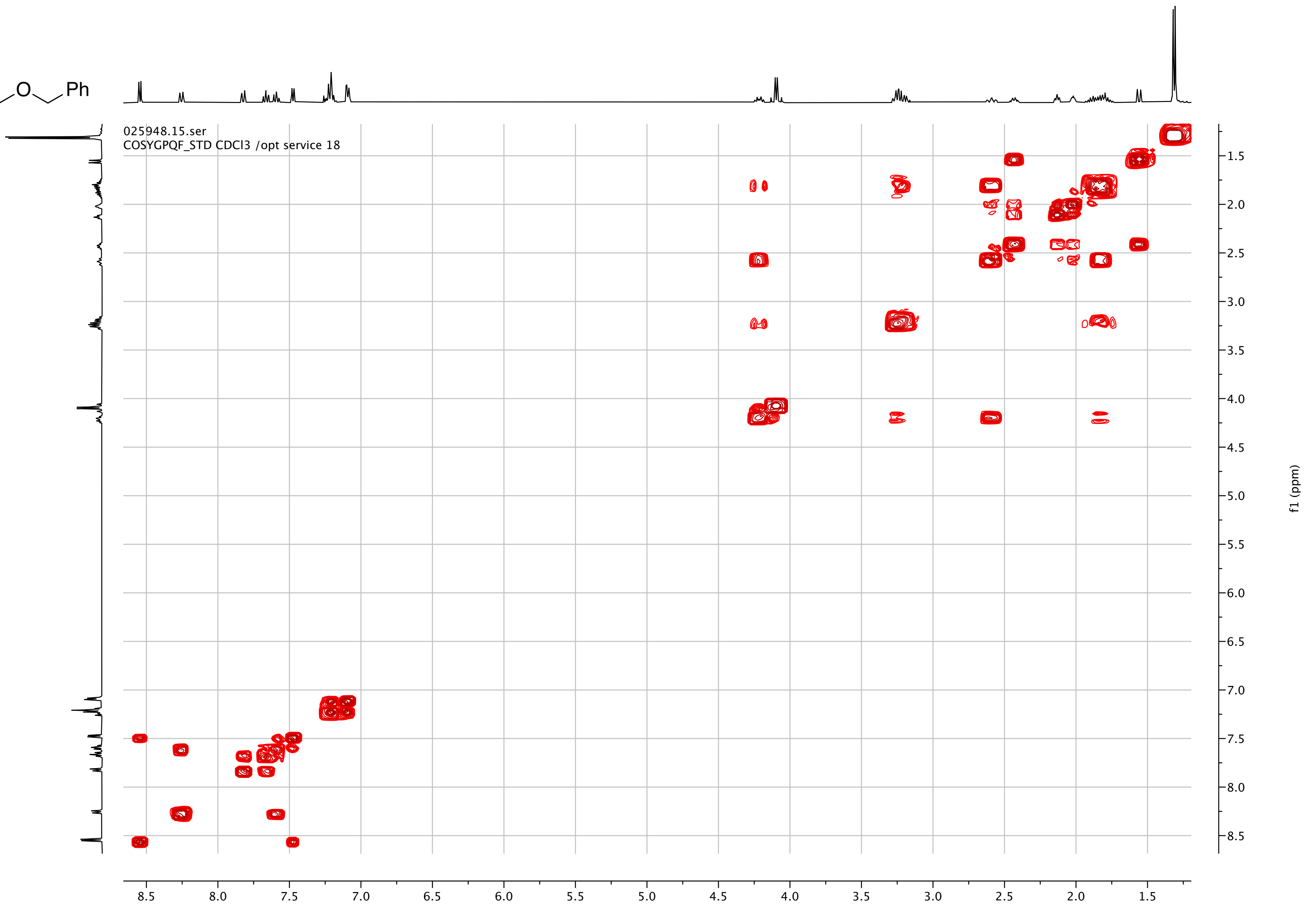

1-(2-(2-(Benzyloxy)ethyl)-6,6-dimethylbicyclo[3.1.1]heptan-3-yl)isoquinoline (23)

^{1}H-^{1}H COSY (400 MHz, $CDCl_3$)

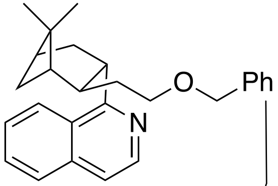

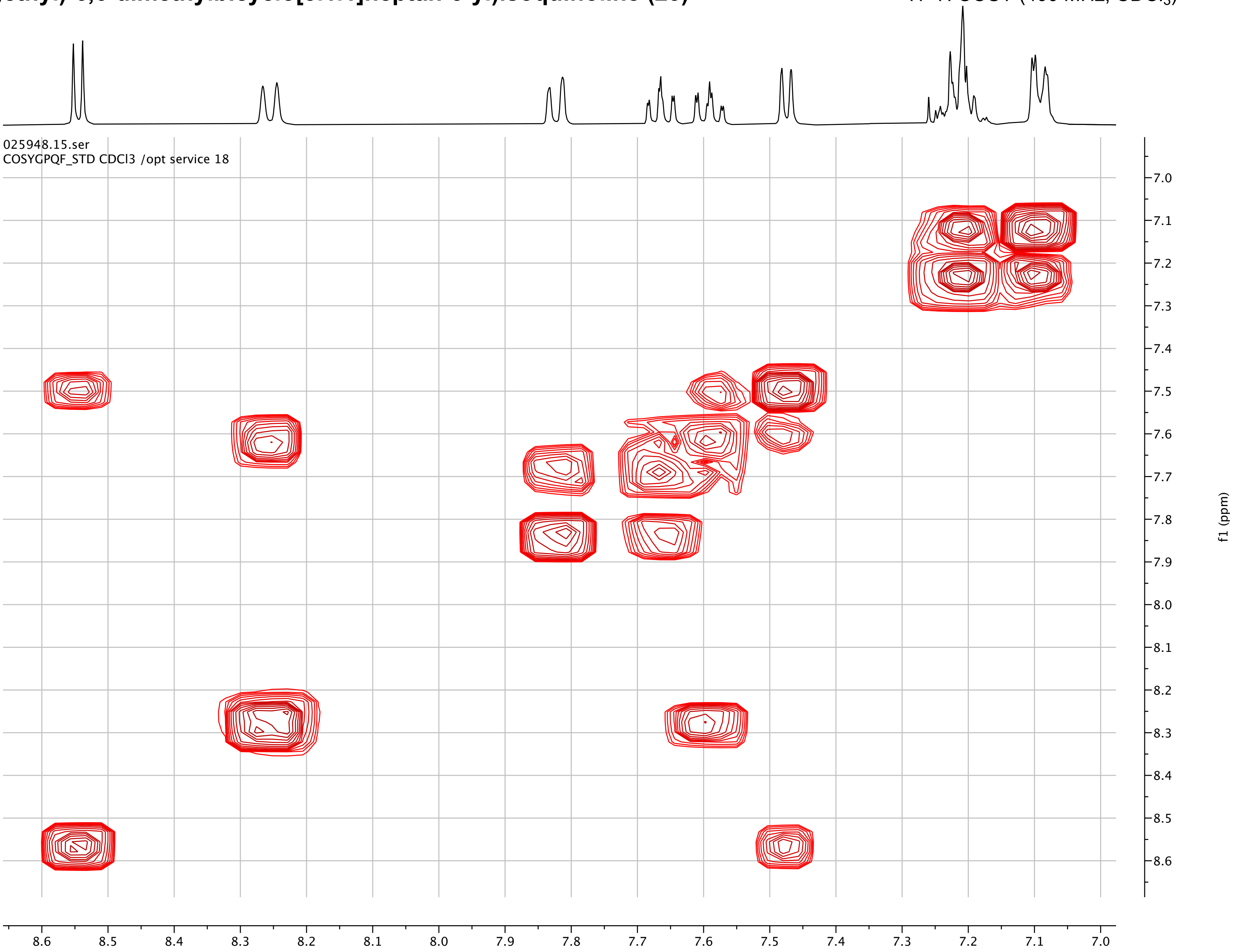

1-(2-(2-(Benzyloxy)ethyl)-6,6-dimethylbicyclo[3.1.1]heptan-3-yl)isoquinoline (23)

^{1}H-^{1}H COSY (400 MHz, $CDCl_3$)

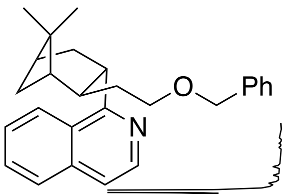

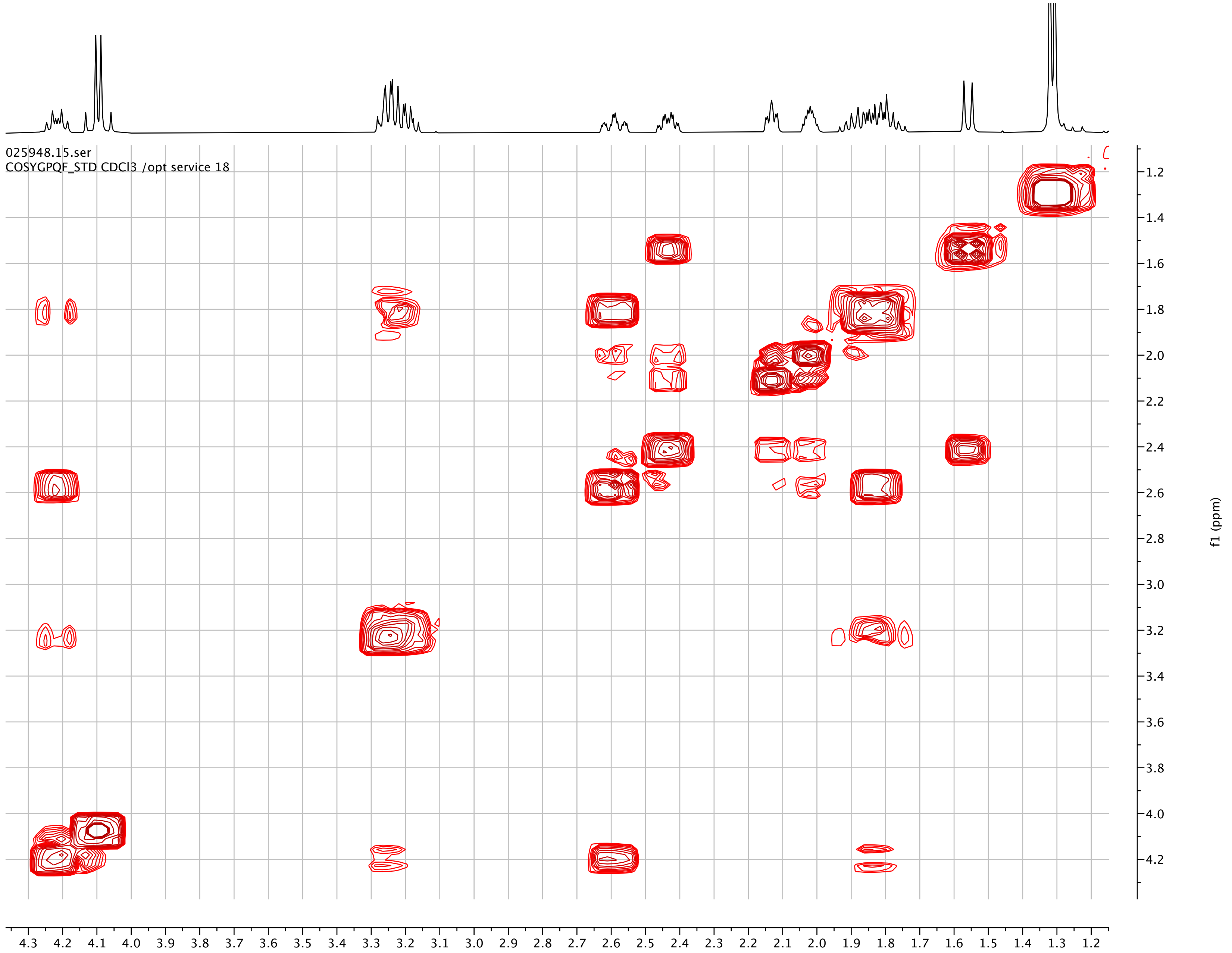

1-(2-(2-(Benzyloxy)ethyl)-6,6-dimethylbicyclo[3.1.1]heptan-3-yl)isoquinoline (23)

HSQC (400 MHz, $CDCl_3$)

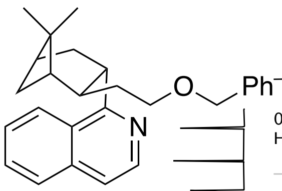

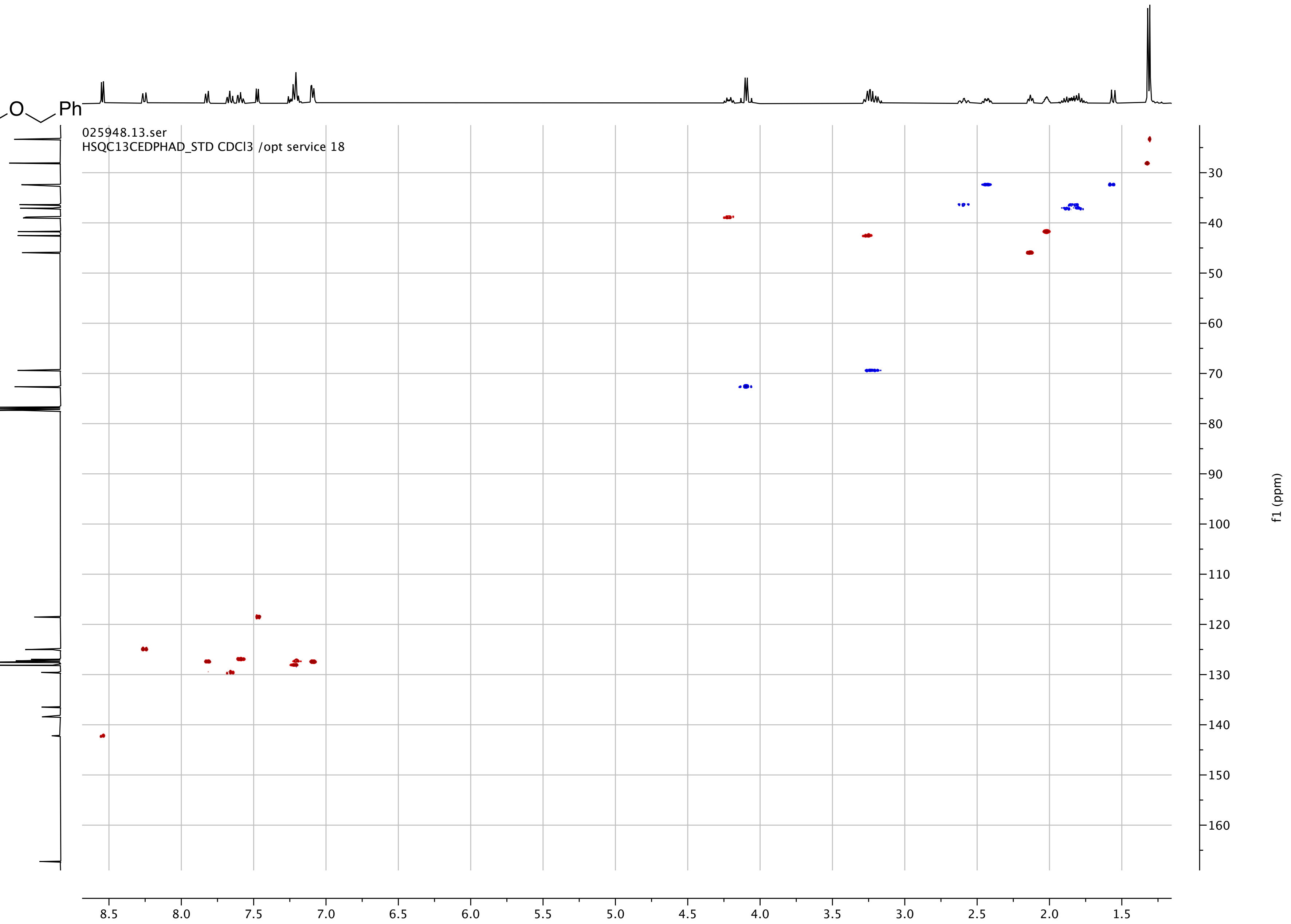

1-(2-(2-(Benzyloxy)ethyl)-6,6-dimethylbicyclo[3.1.1]heptan-3-yl)isoquinoline (23)

HSQC (400 MHz, $CDCl_3$)

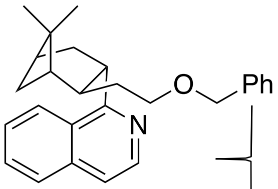

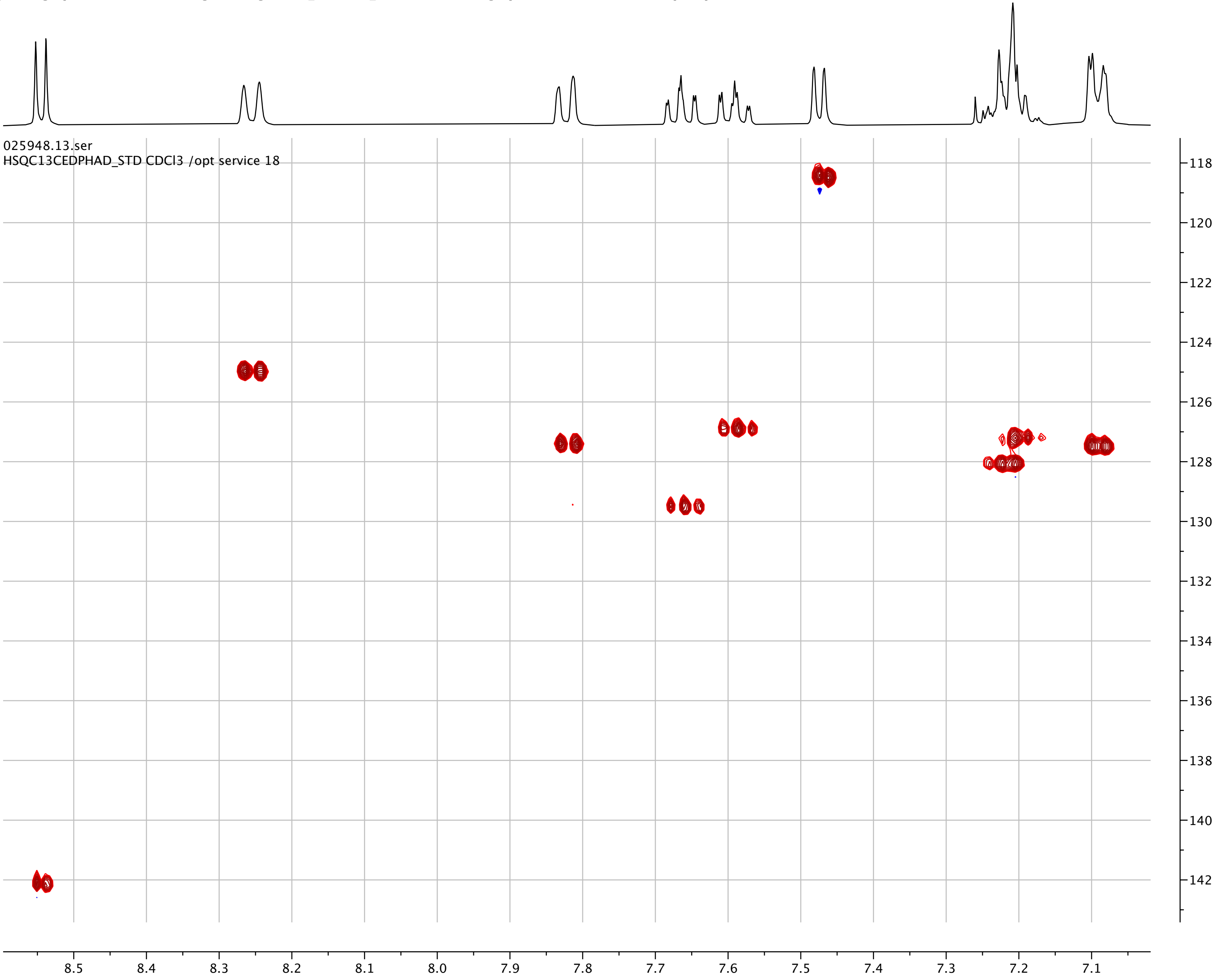

1-(2-(2-(Benzyloxy)ethyl)-6,6-dimethylbicyclo[3.1.1]heptan-3-yl)isoquinoline (23)

HSQC (400 MHz, $CDCl_3$)

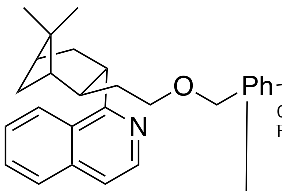

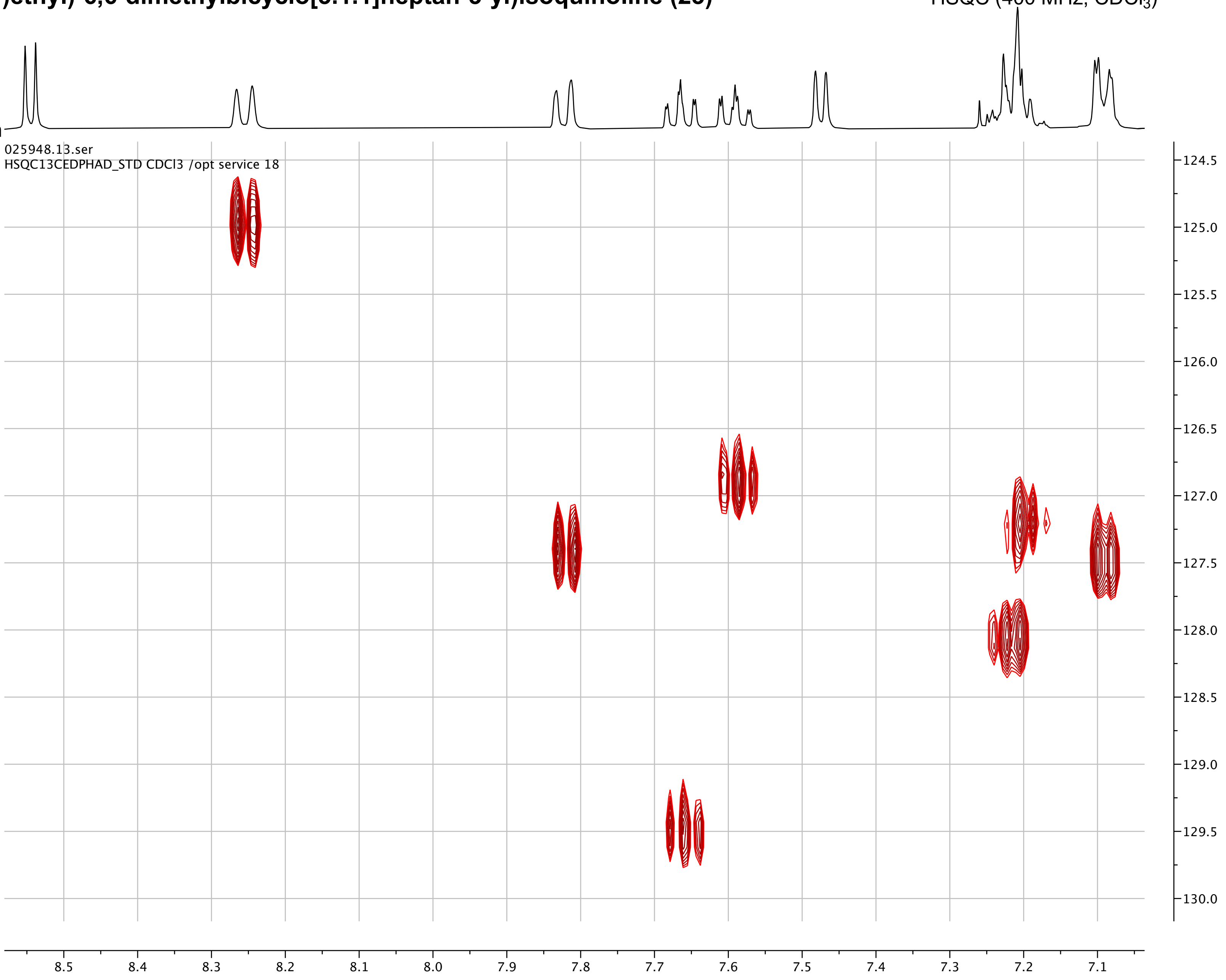

1-(2-(2-(Benzyloxy)ethyl)-6,6-dimethylbicyclo[3.1.1]heptan-3-yl)isoquinoline (23)

HSQC (400 MHz, $CDCl_3$)

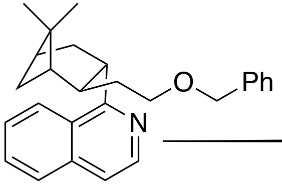

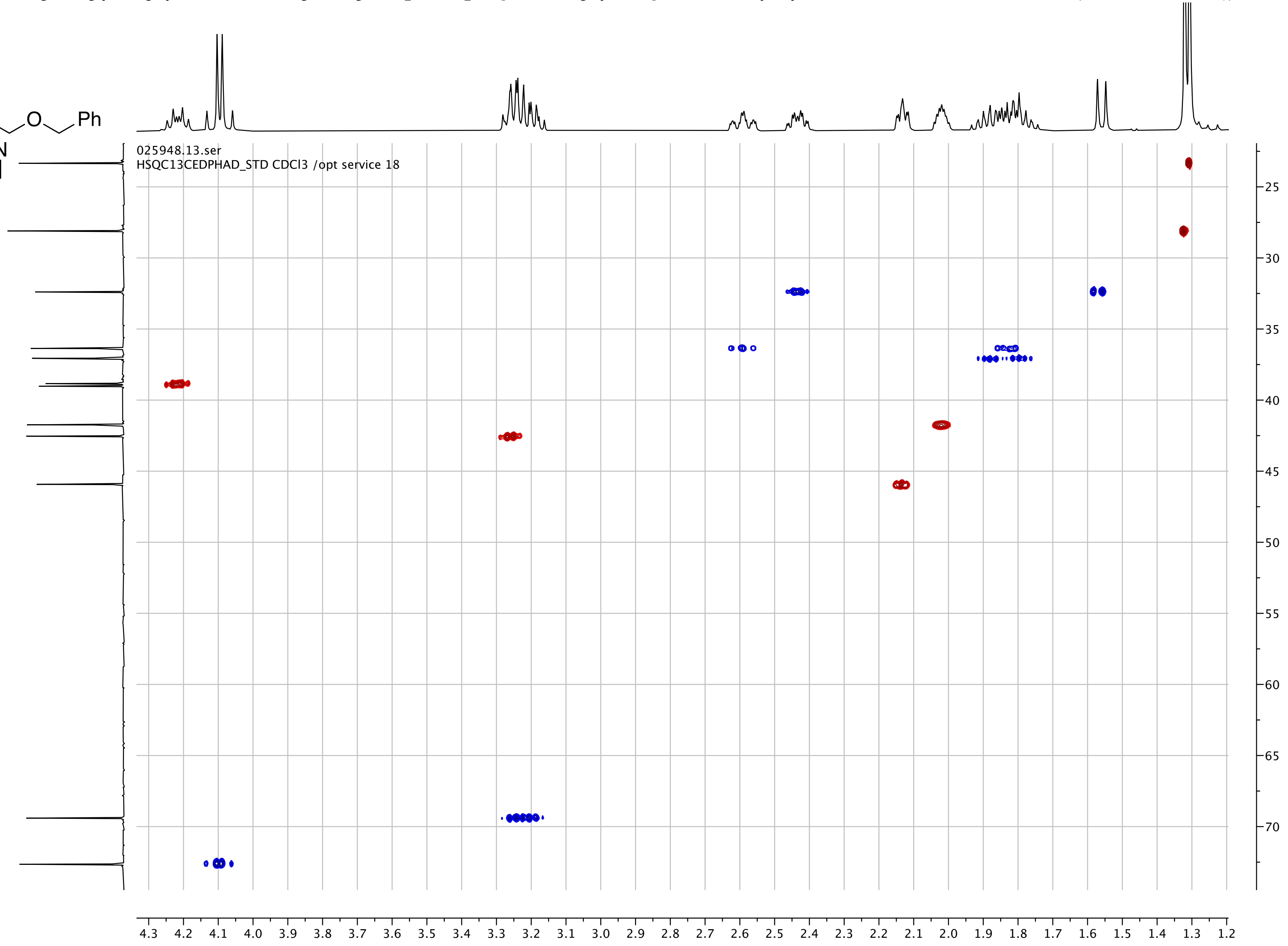

1-(2-(2-(Benzyloxy)ethyl)-6,6-dimethylbicyclo[3.1.1]heptan-3-yl)isoquinoline (23)

HMBC (400 MHz, $CDCl_3$)

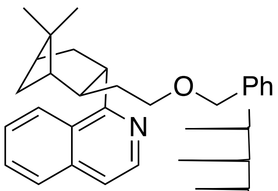

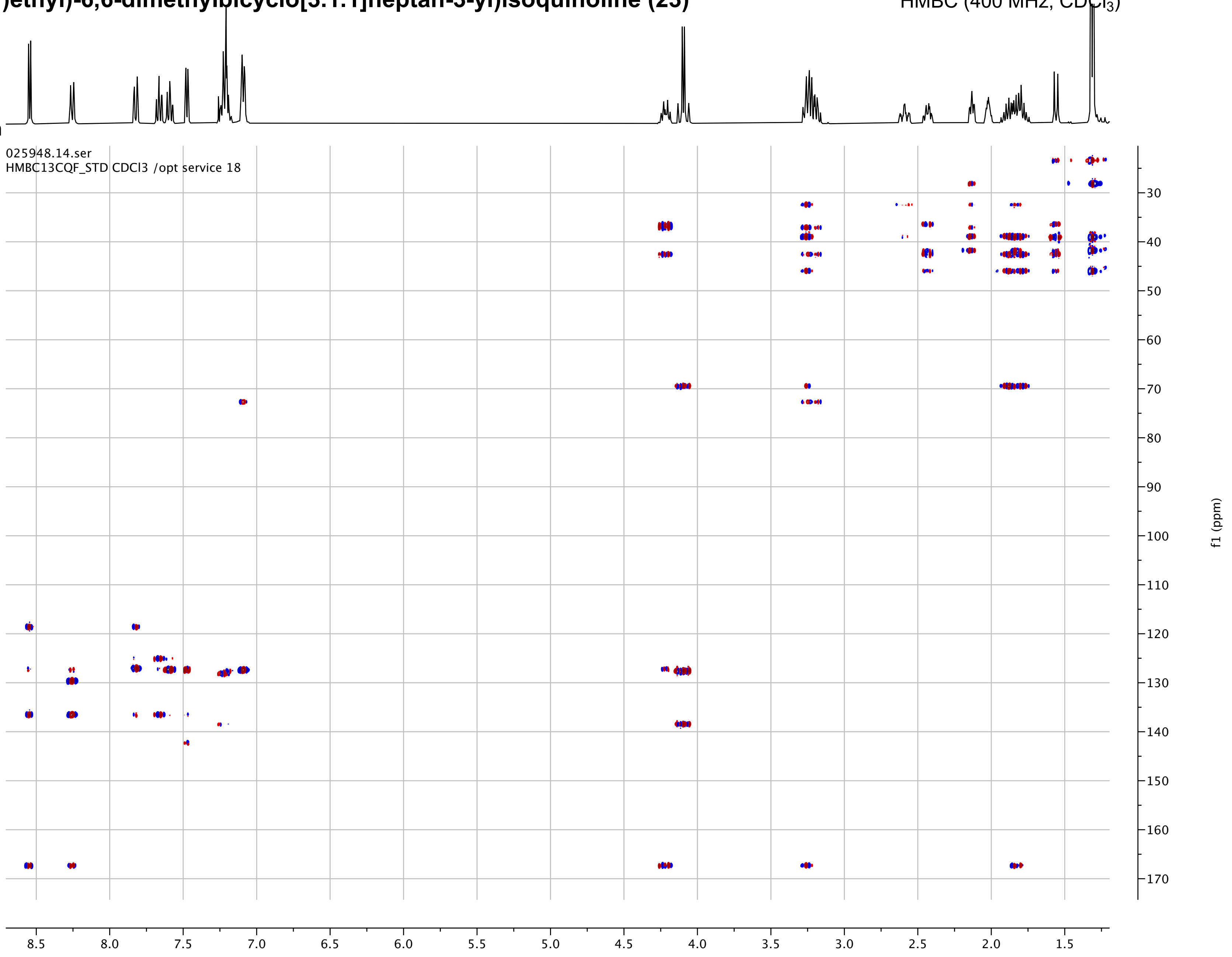

1-(2-(2-(Benzyloxy)ethyl)-6,6-dimethylbicyclo[3.1.1]heptan-3-yl)isoquinoline (23)

HMBC (400 MHz, $CDCl_3$)

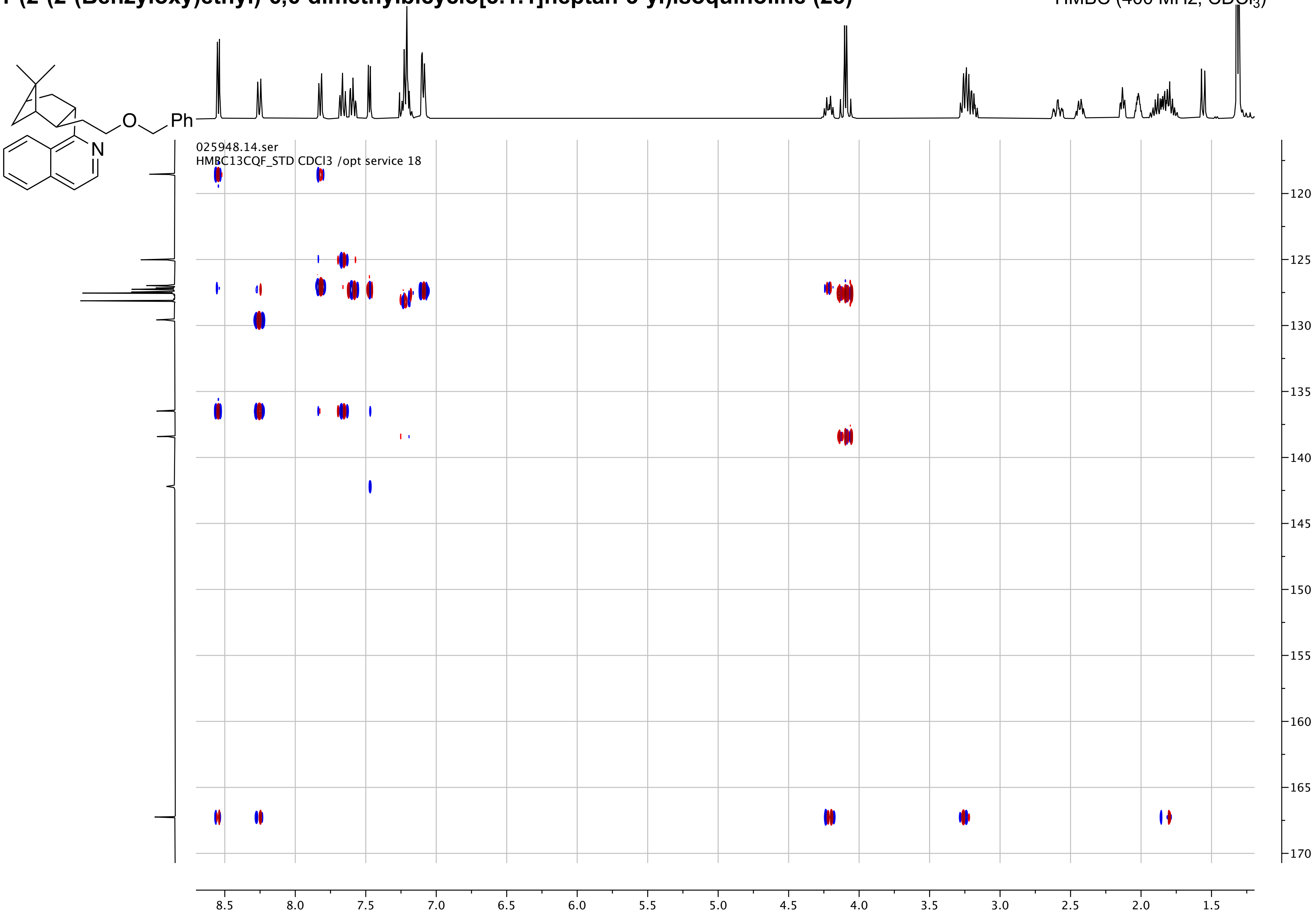

1-(2-(2-(Benzyloxy)ethyl)-6,6-dimethylbicyclo[3.1.1]heptan-3-yl)isoquinoline (23)

HMBC (400 MHz, $CDCl_3$)

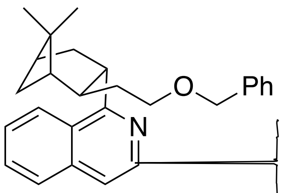

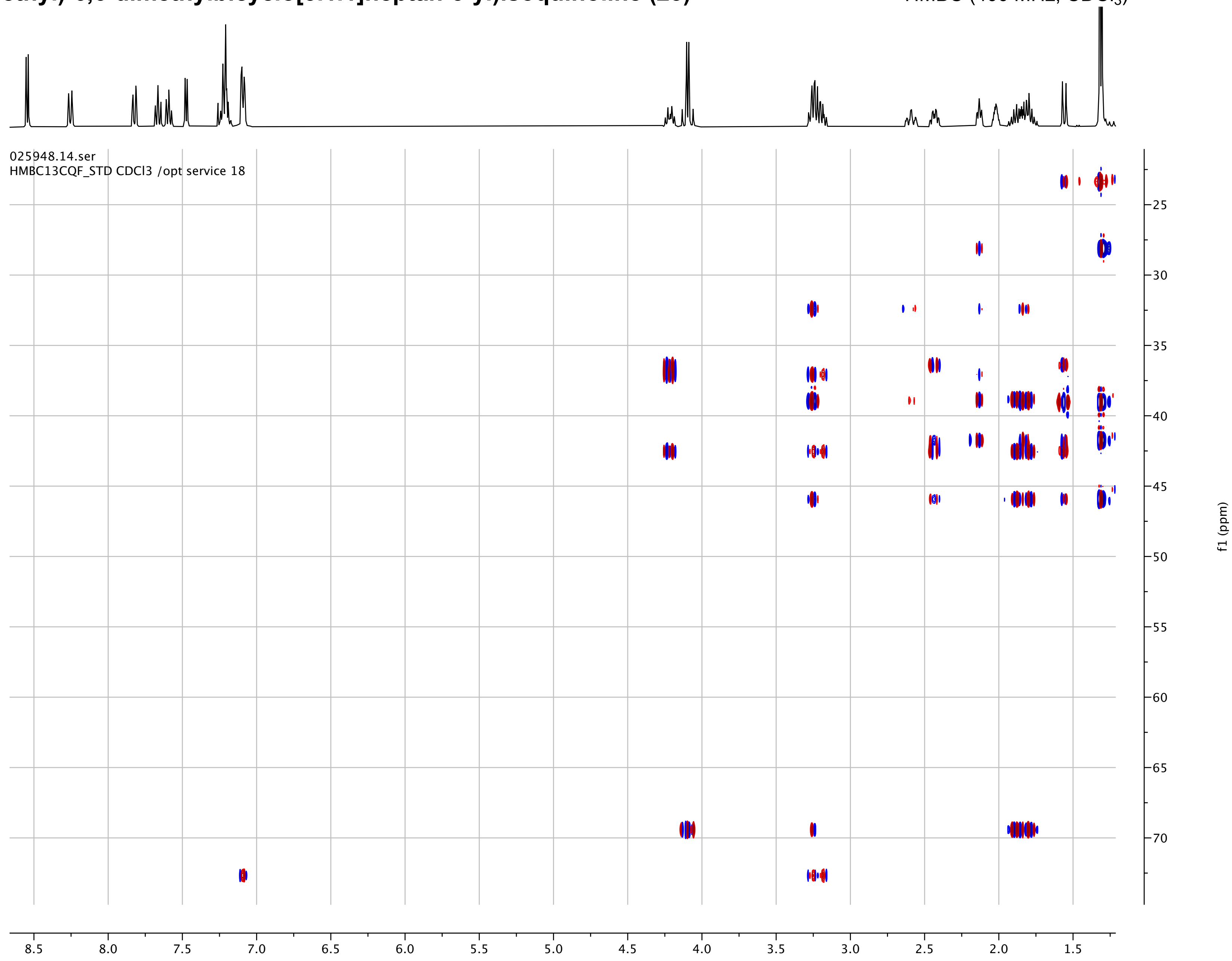

3-(2-(2-(Benzyloxy)ethyl)-6,6-dimethylbicyclo[3.1.1]heptan-3-yl)isoquinoline (C3-23, minor)

^{1}H-NMR (400 MHz, $CDCl_3$)

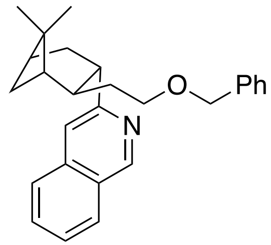

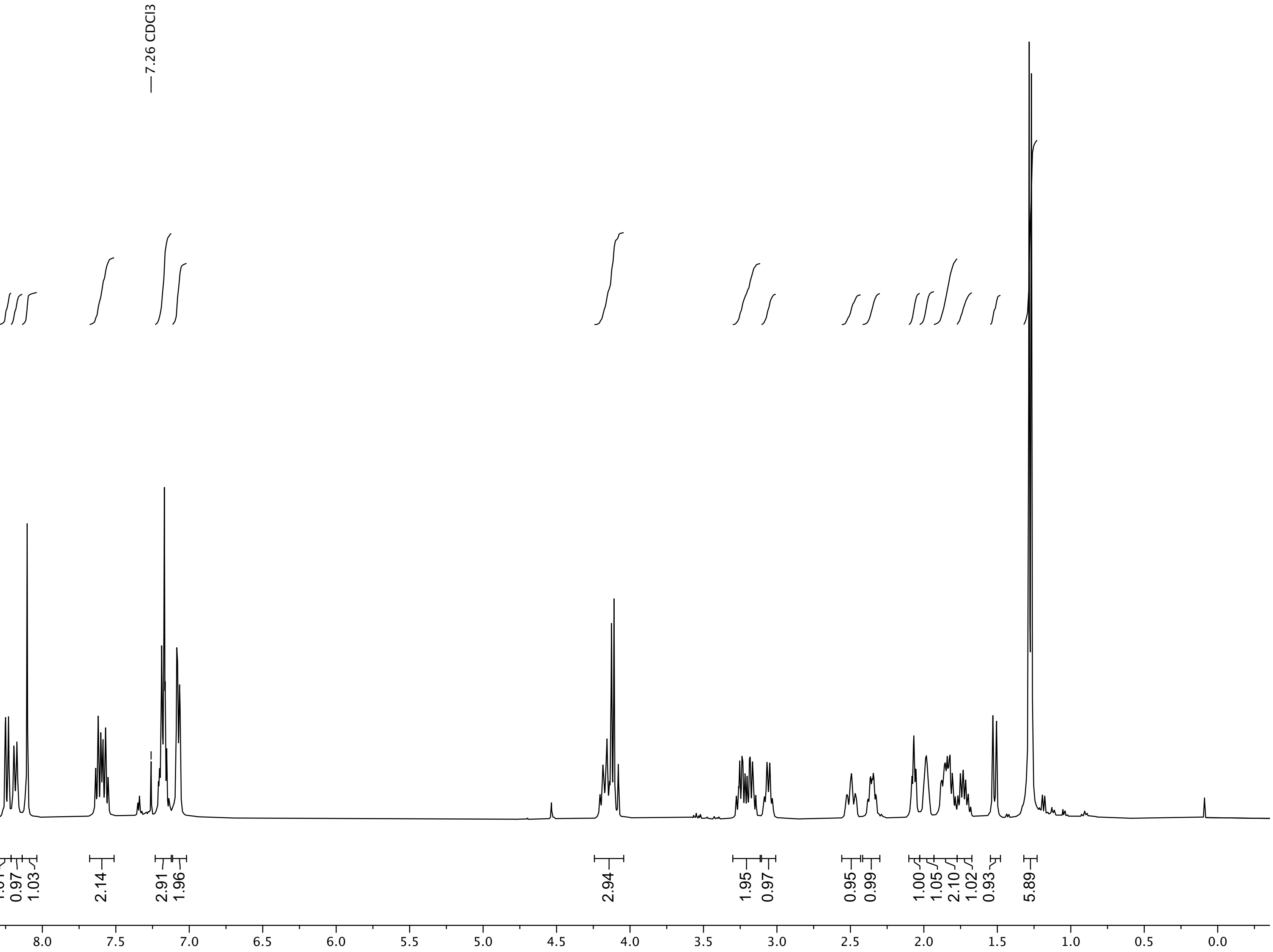

3-(2-(2-(Benzyloxy)ethyl)-6,6-dimethylbicyclo[3.1.1]heptan-3-yl)isoquinoline (C3-23, minor)

^{1}H-NMR (400 MHz, $CDCl_3$)

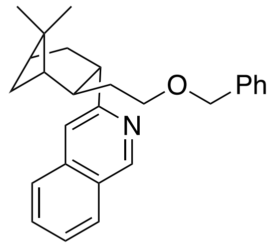

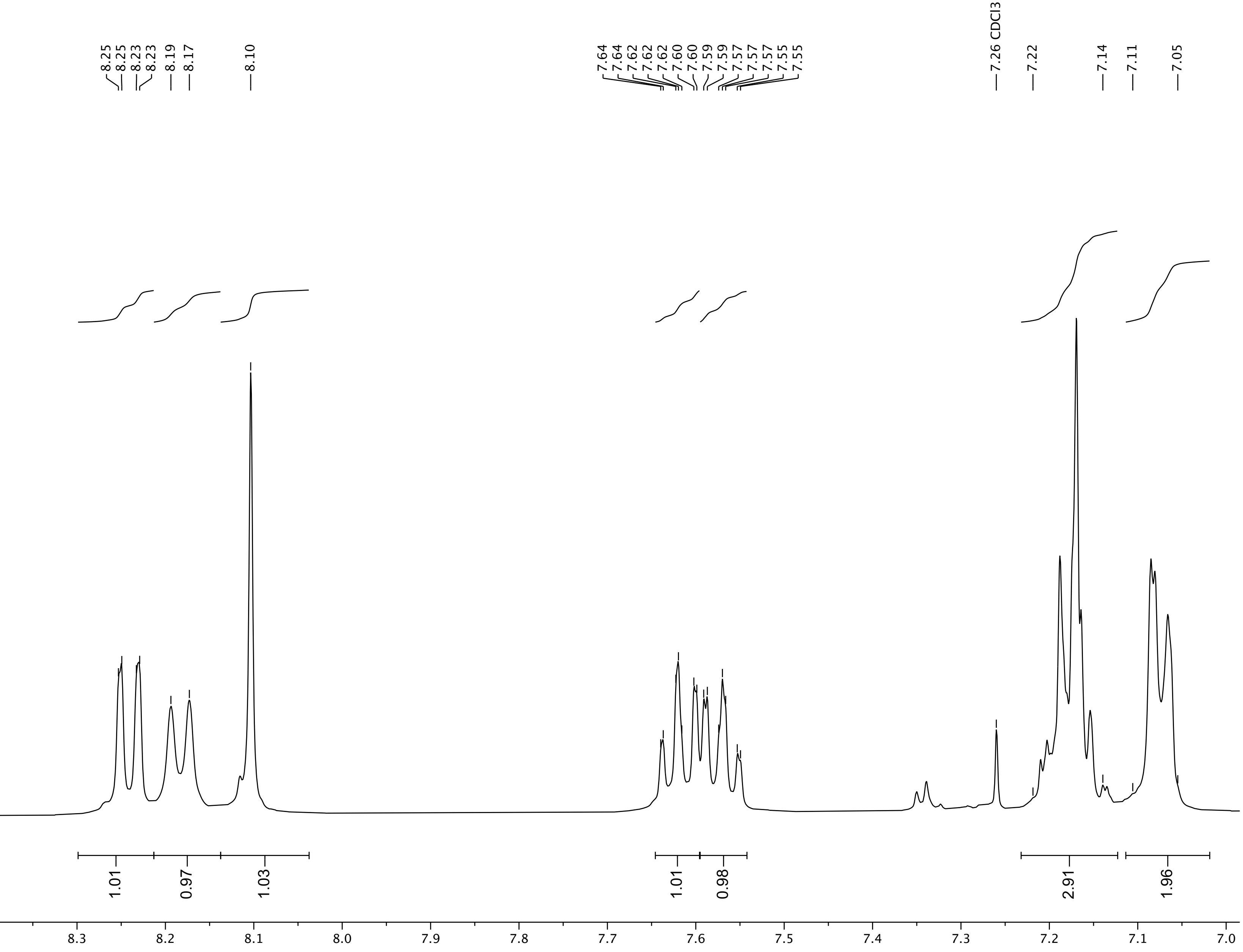

3-(2-(2-(Benzyloxy)ethyl)-6,6-dimethylbicyclo[3.1.1]heptan-3-yl)isoquinoline (C3-23, minor)

^{1}H-NMR (400 MHz, $CDCl_3$)

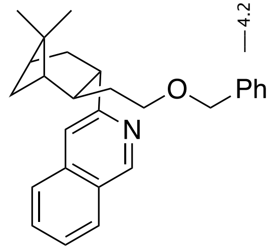

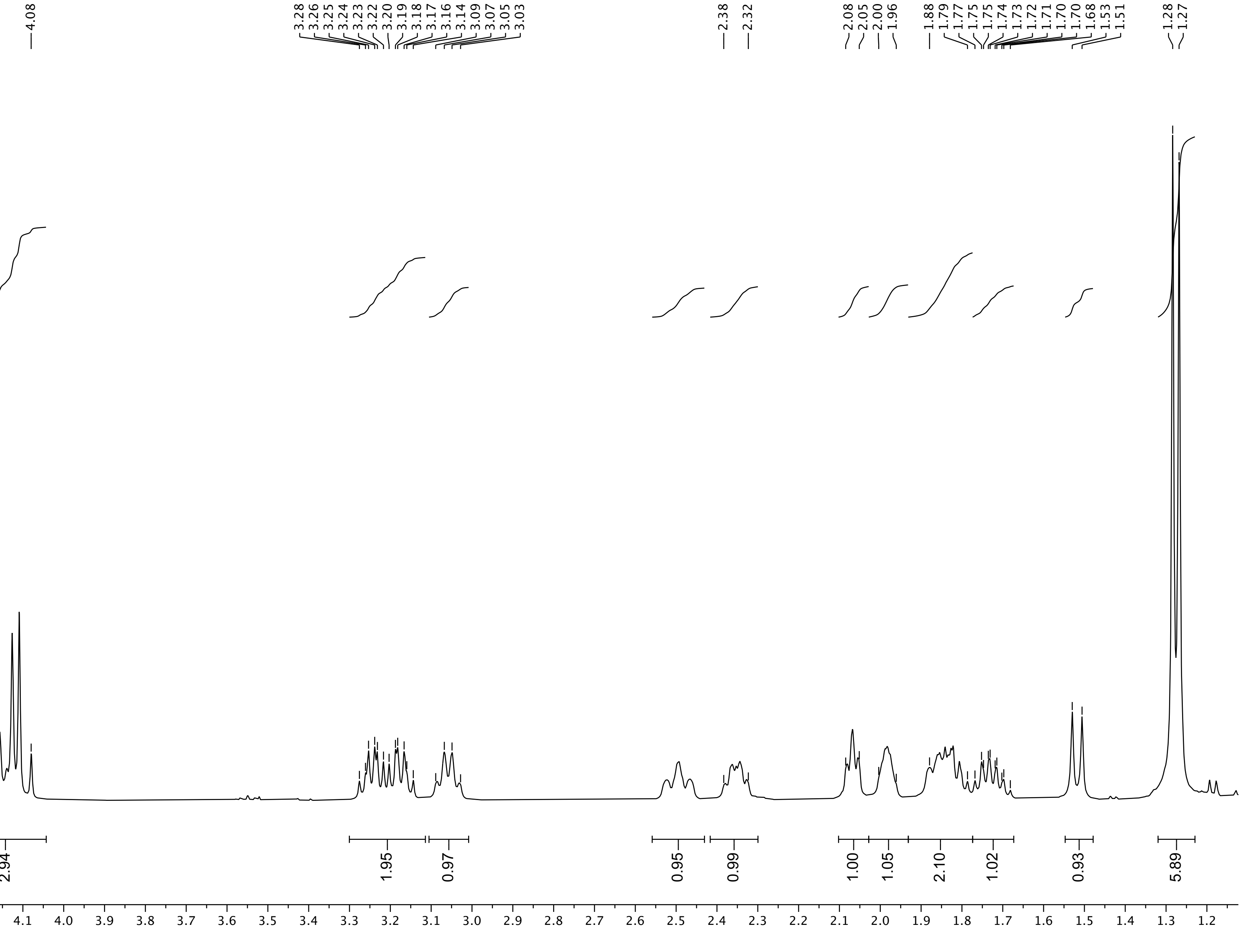

3-(2-(2-(Benzyloxy)ethyl)-6,6-dimethylbicyclo[3.1.1]heptan-3-yl)isoquinoline (C3-23, minor)

^{1}H-NMR (400 MHz, $CDCl_3$)

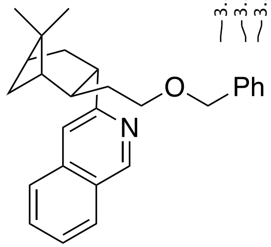

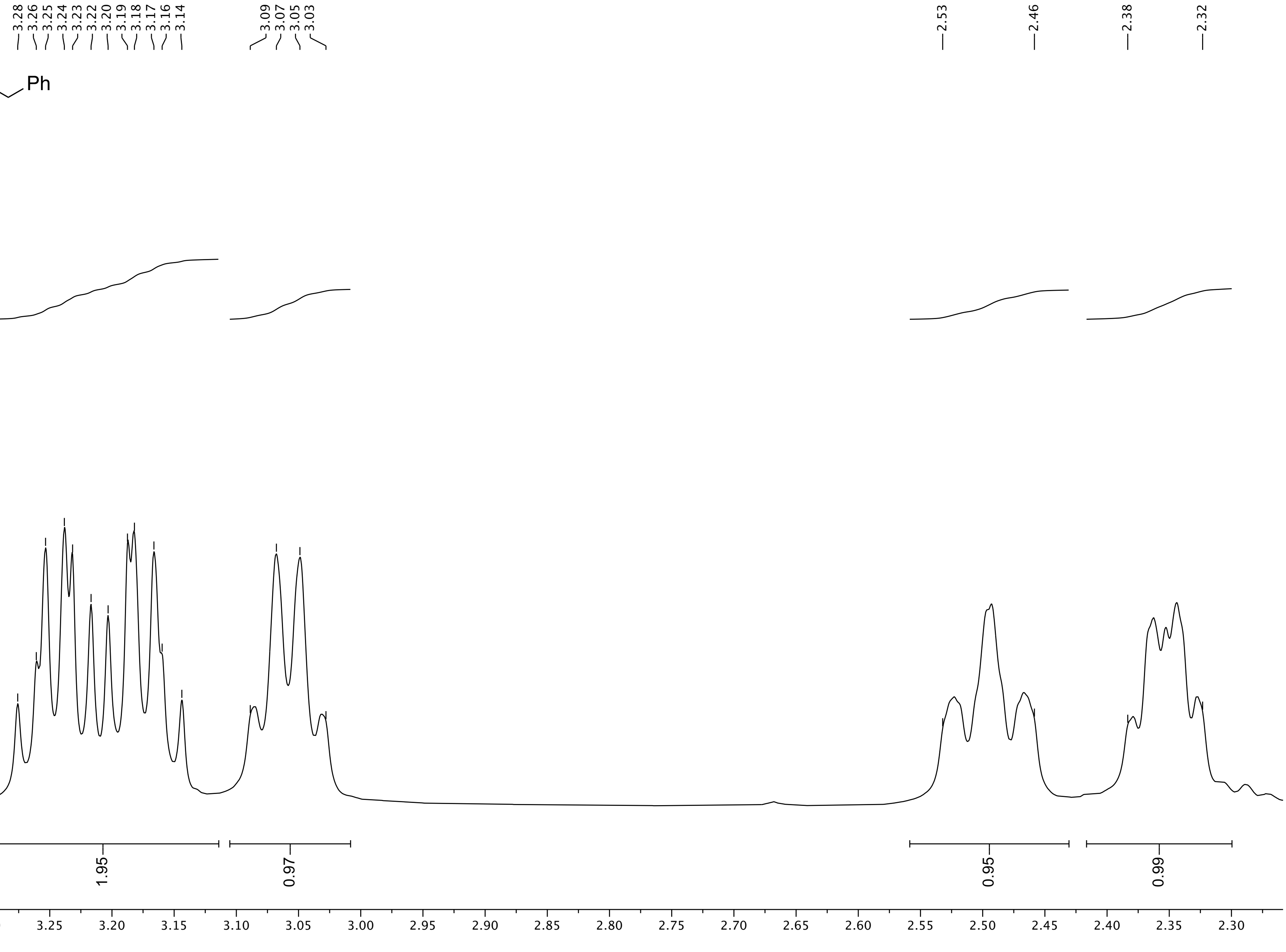

3-(2-(2-(Benzyloxy)ethyl)-6,6-dimethylbicyclo[3.1.1]heptan-3-yl)isoquinoline (C3-23, minor)

^{1}H-NMR (400 MHz, $CDCl_3$)

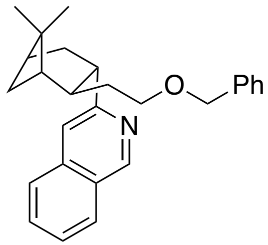

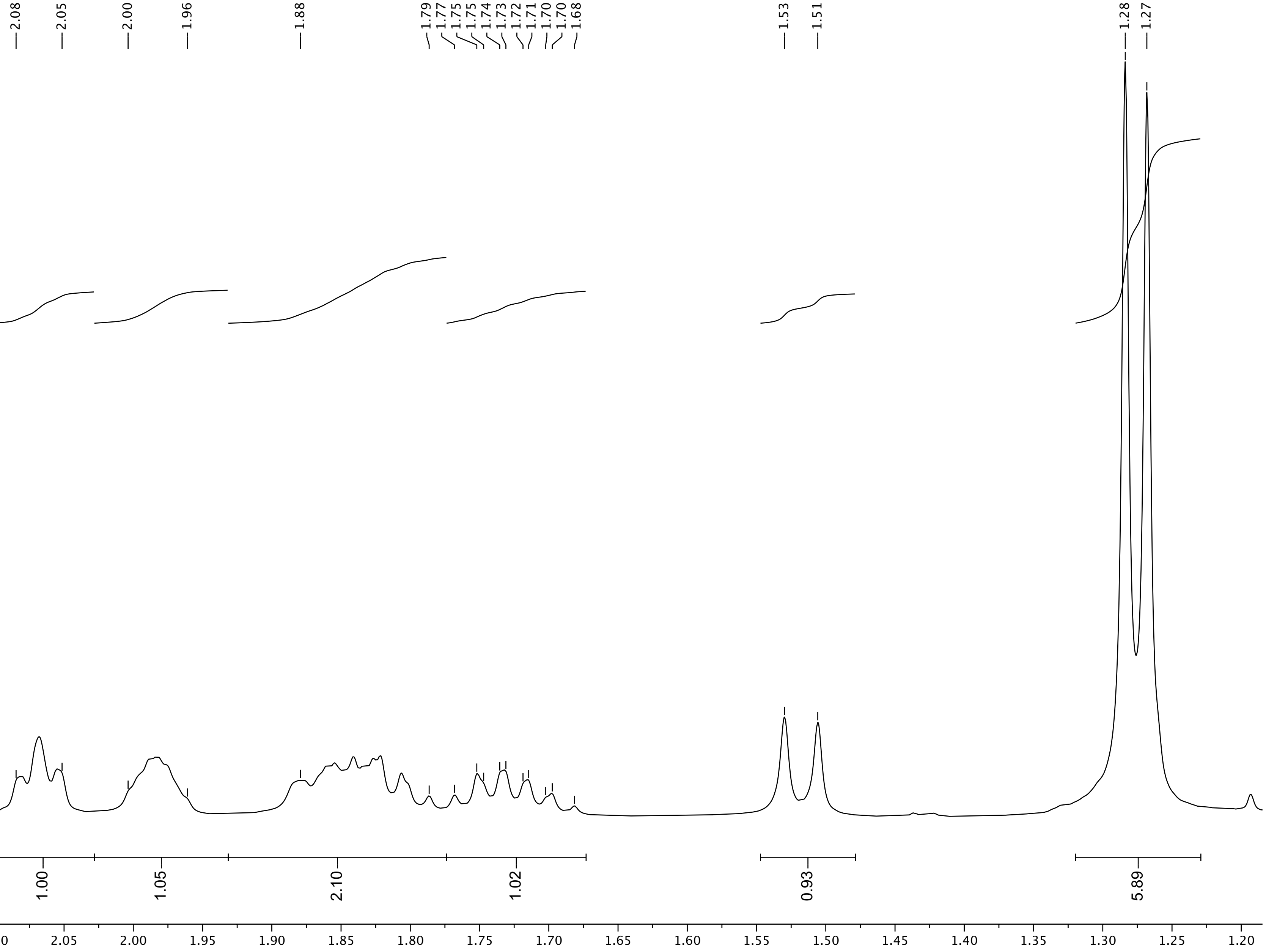

3-(2-(2-(Benzyloxy)ethyl)-6,6-dimethylbicyclo[3.1.1]heptan-3-yl)isoquinoline (C3-23, minor)

^{13}C-NMR (101 MHz, $CDCl_3$)

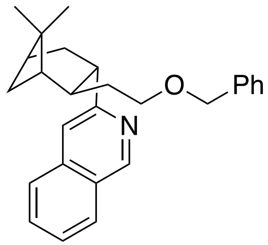

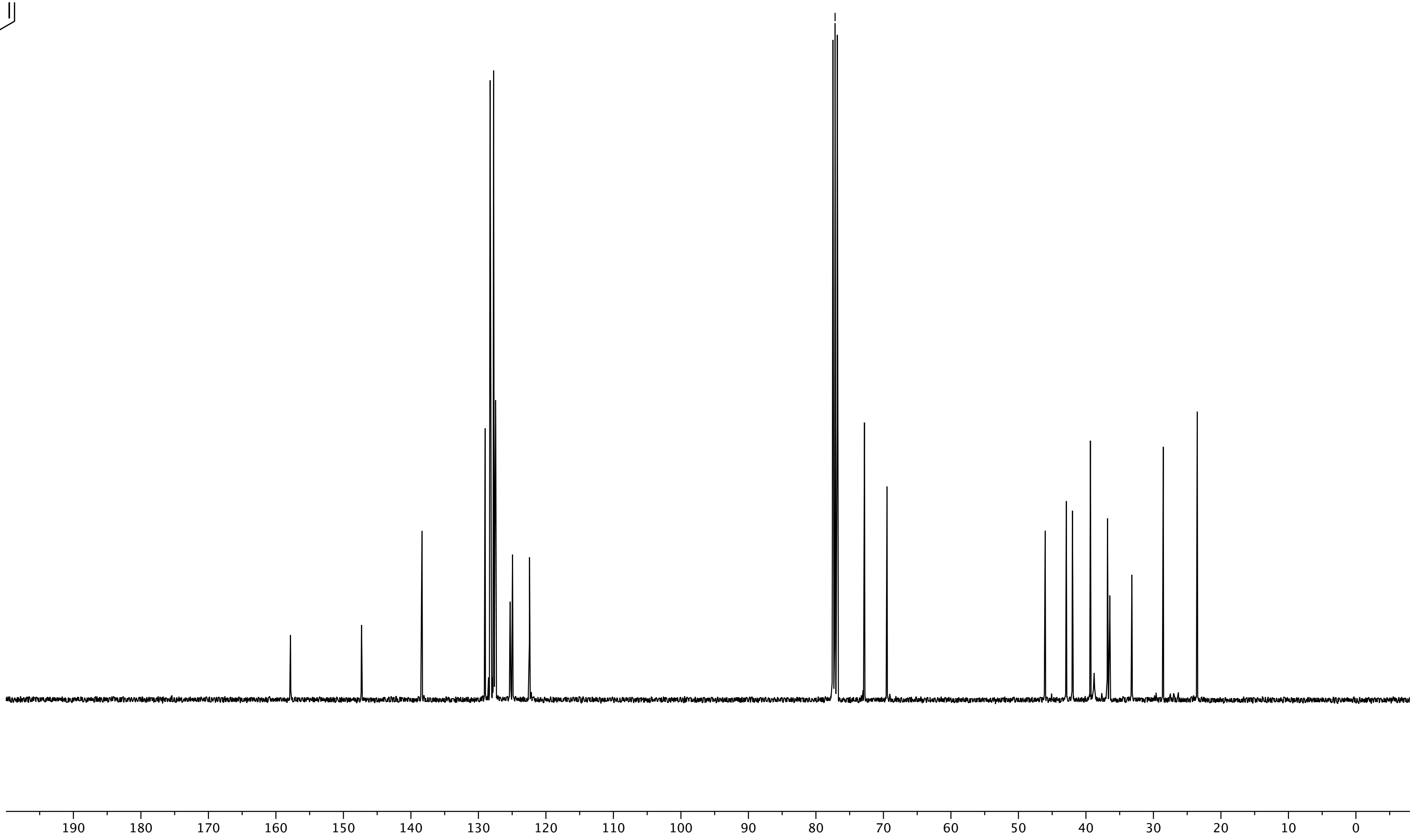

3-(2-(2-(Benzyloxy)ethyl)-6,6-dimethylbicyclo[3.1.1]heptan-3-yl)isoquinoline (C3-23, minor)

^{13}C-NMR (101 MHz, $CDCl_3$)

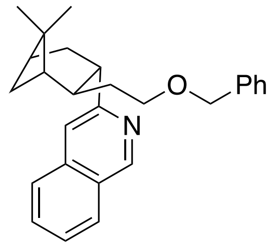

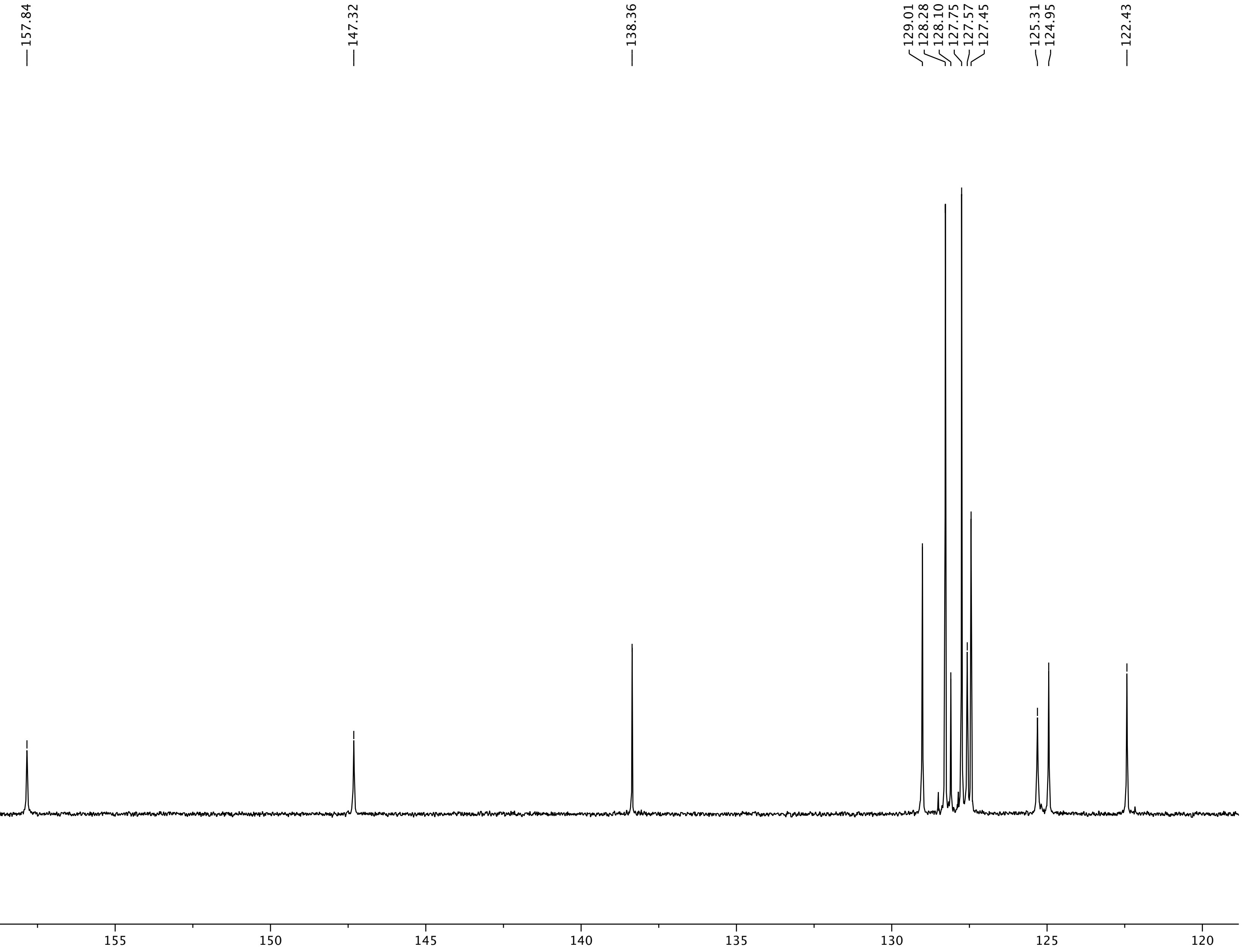

3-(2-(2-(Benzyloxy)ethyl)-6,6-dimethylbicyclo[3.1.1]heptan-3-yl)isoquinoline (C3-23, minor)

^{13}C-NMR (101 MHz, $CDCl_3$)

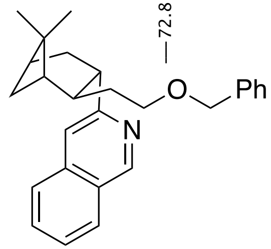

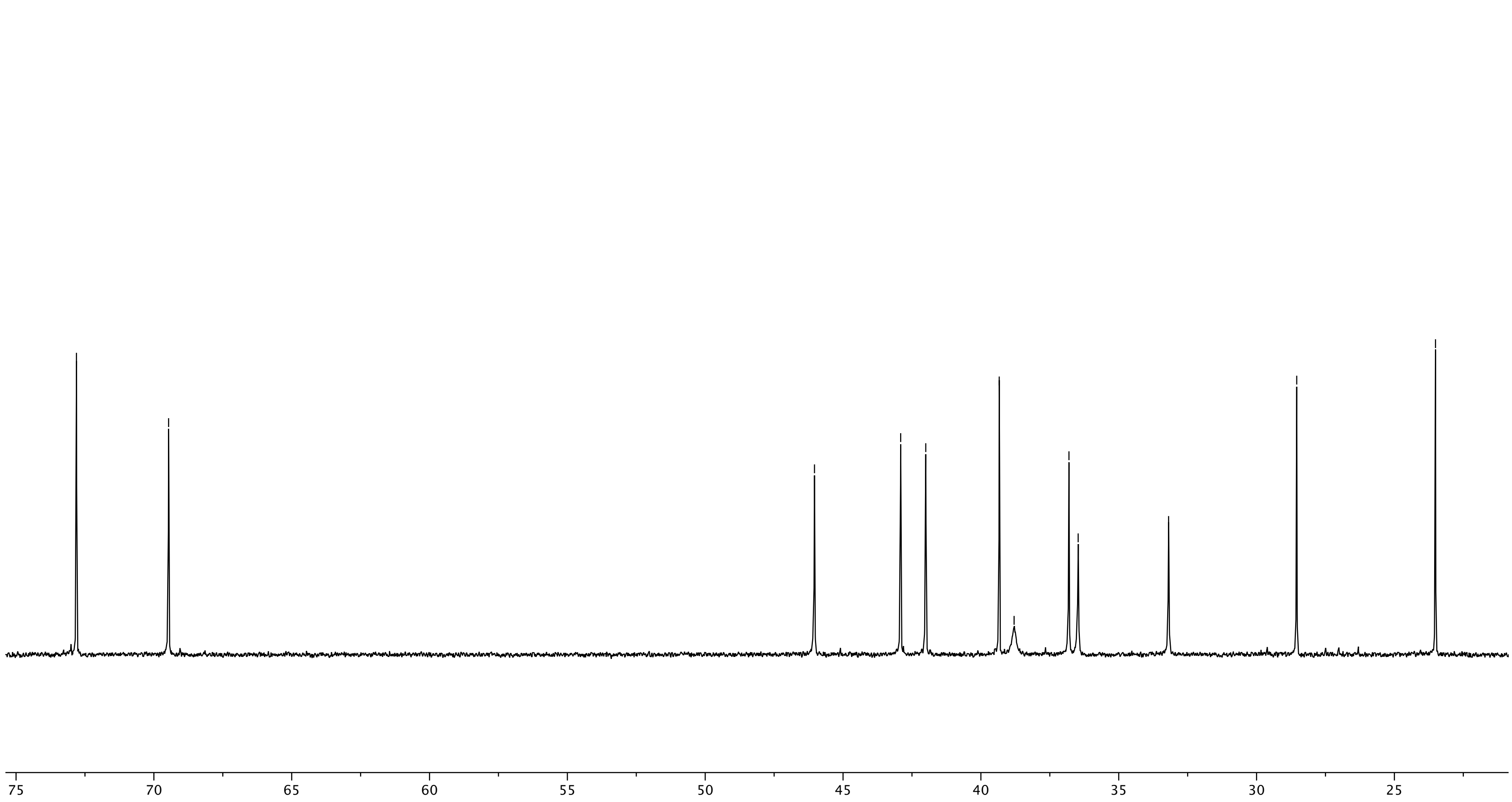

3-(2-(2-(Benzyloxy)ethyl)-6,6-dimethylbicyclo[3.1.1]heptan-3-yl)isoquinoline (C3-23, minor) Dept-135 (101 MHz, $CDCl_3$)

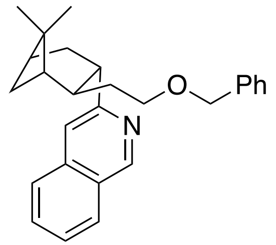

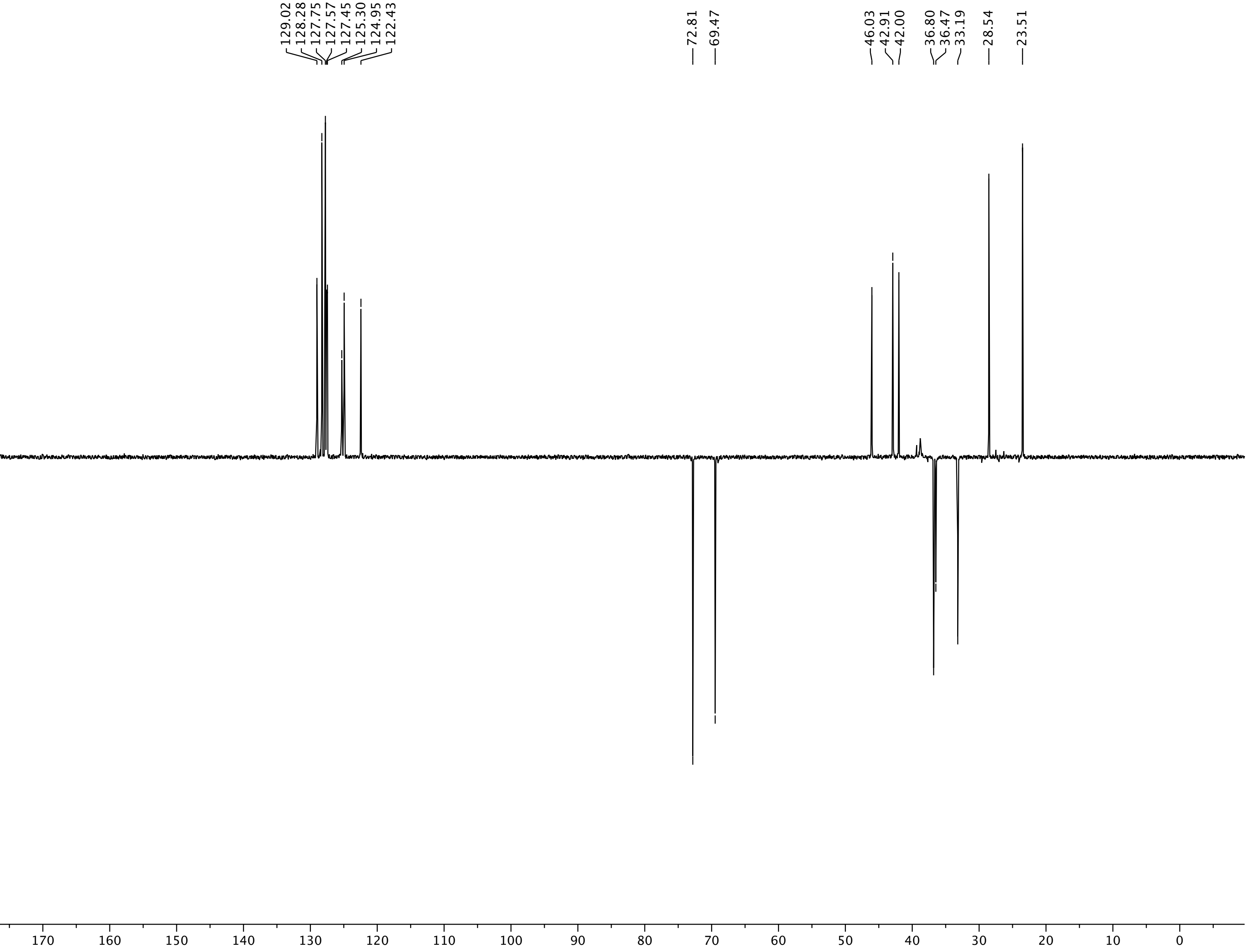

3-(2-(2-(Benzyloxy)ethyl)-6,6-dimethylbicyclo[3.1.1]heptan-3-yl)isoquinoline (C3-23, minor)

^1H-^1H COSY(400 MHz, $CDCl_3$)

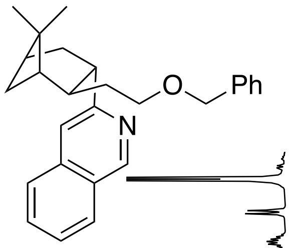

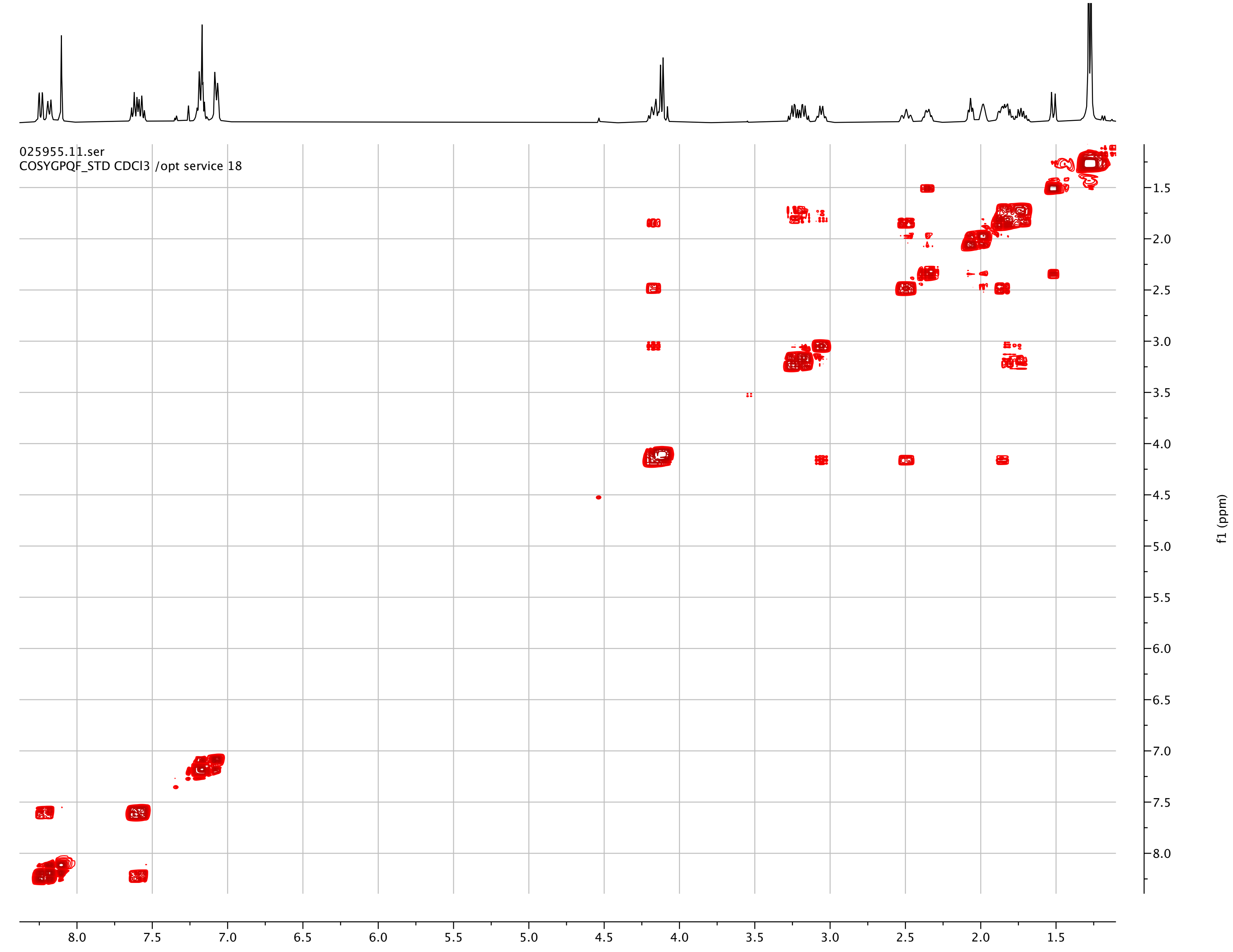

3-(2-(2-(Benzyloxy)ethyl)-6,6-dimethylbicyclo[3.1.1]heptan-3-yl)isoquinoline (C3-23, minor)

^{1}H-^{1}H COSY(400 MHz, $CDCl_3$)

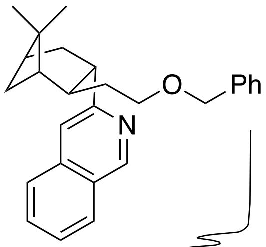

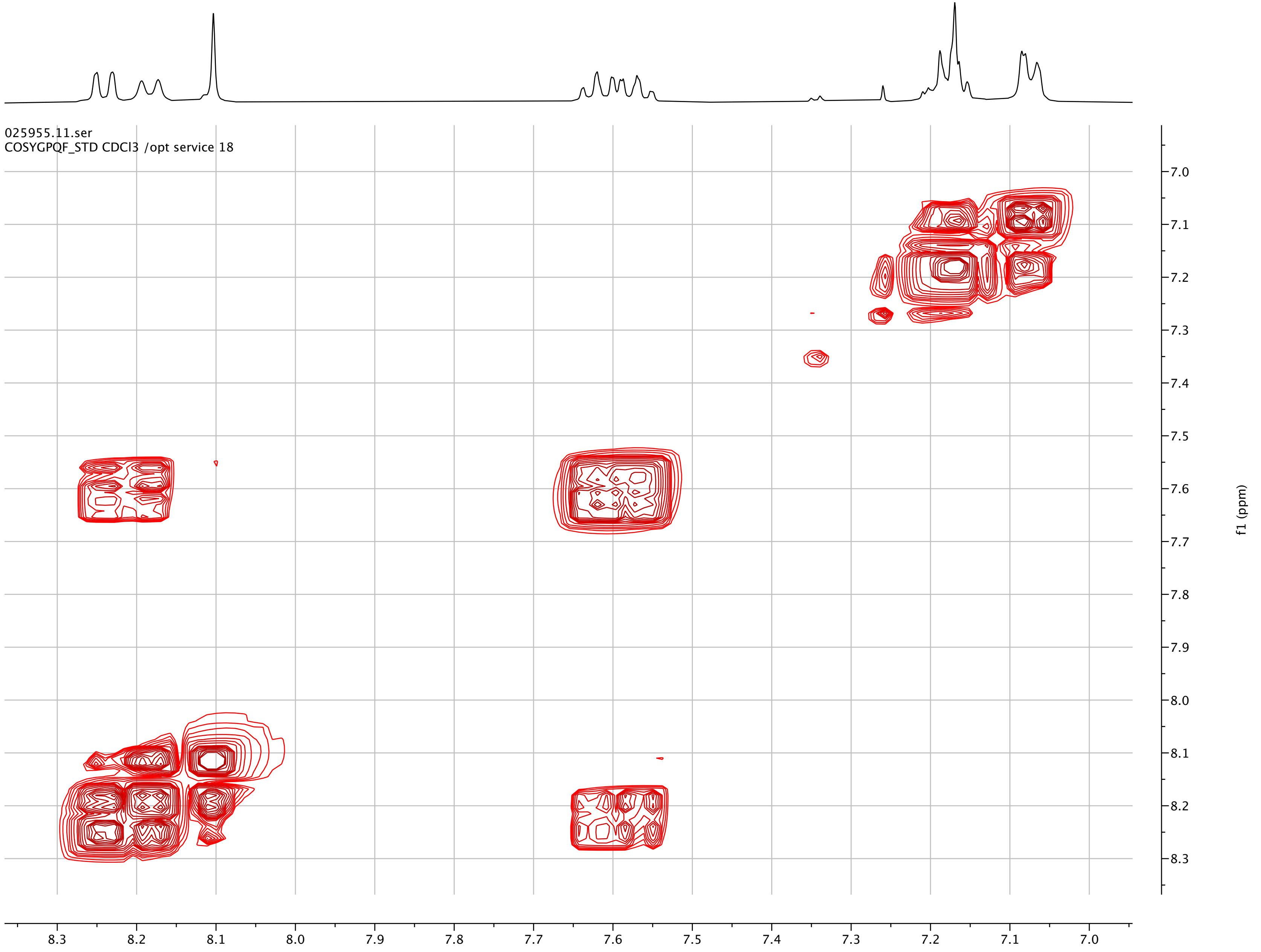

3-(2-(2-(Benzyloxy)ethyl)-6,6-dimethylbicyclo[3.1.1]heptan-3-yl)isoquinoline (C3-23, minor)

^{1}H-^{1}H COSY(400 MHz, $CDCl_3$)

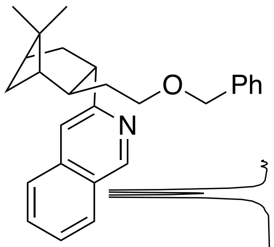

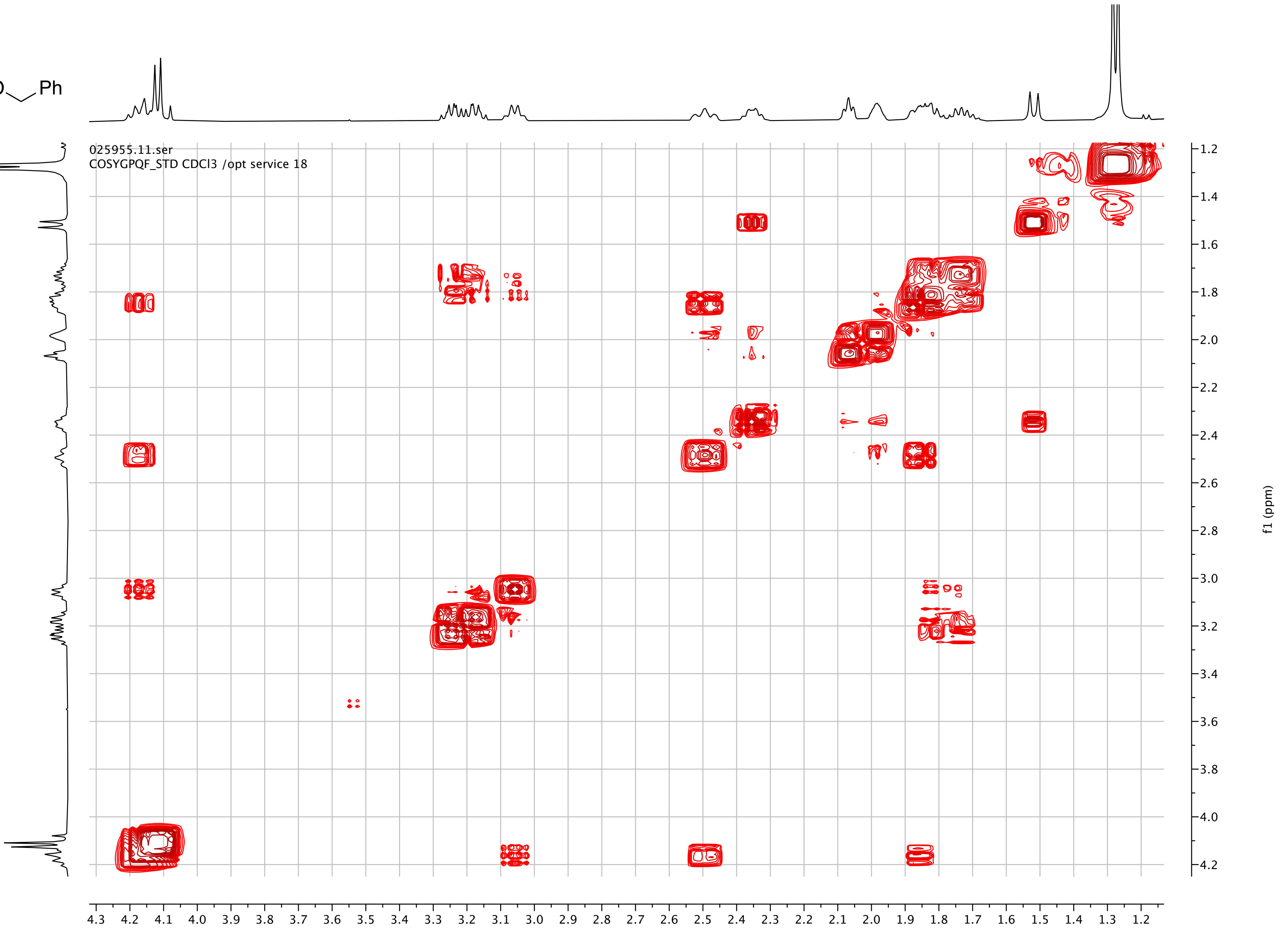

3-(2-(2-(Benzyloxy)ethyl)-6,6-dimethylbicyclo[3.1.1]heptan-3-yl)isoquinoline (C3-23, minor)

HSQC (400 MHz, $CDCl_3$)

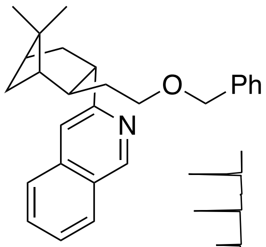

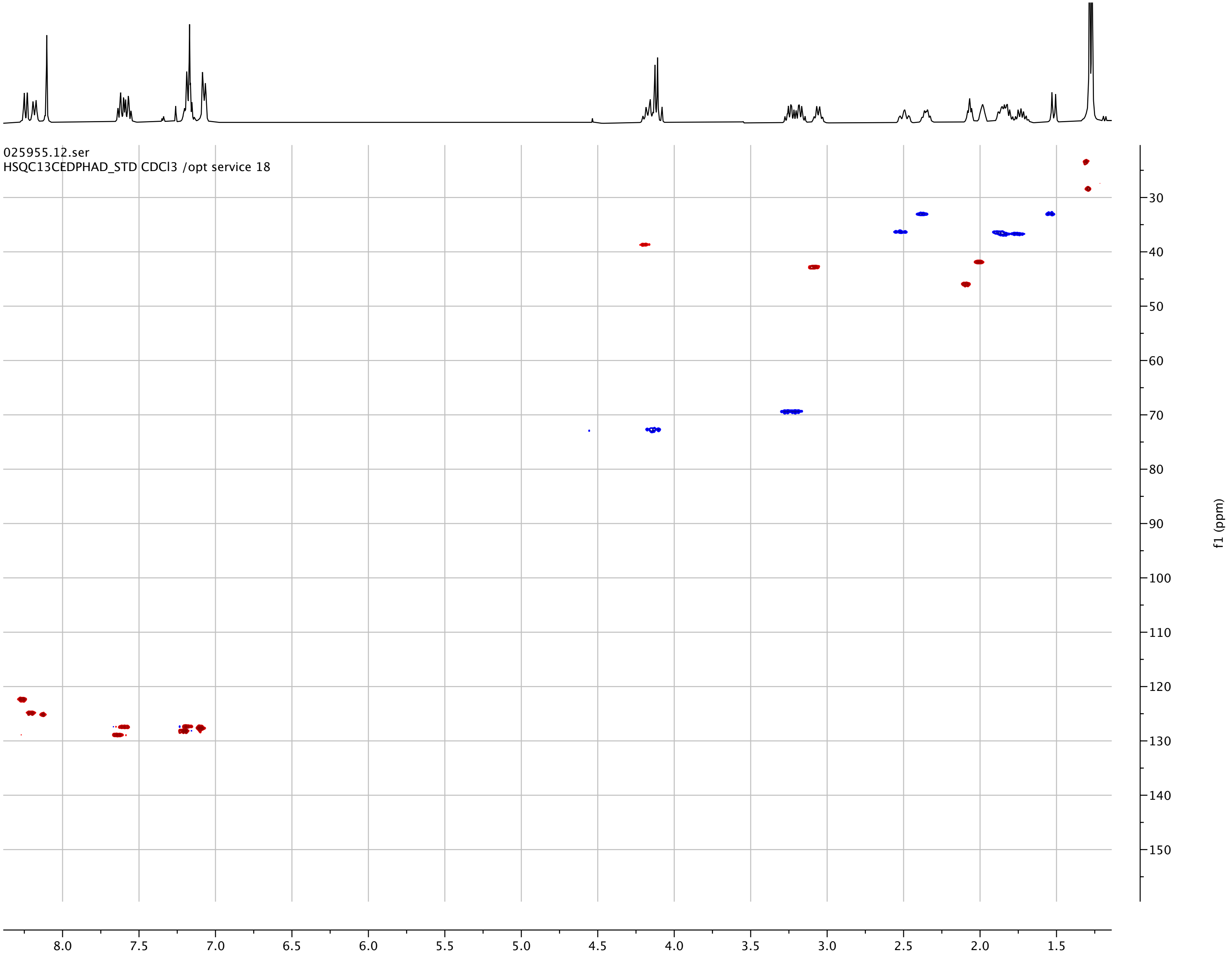

3-(2-(2-(Benzyloxy)ethyl)-6,6-dimethylbicyclo[3.1.1]heptan-3-yl)isoquinoline (C3-23, minor)

HSQC (400 MHz, $CDCl_3$)

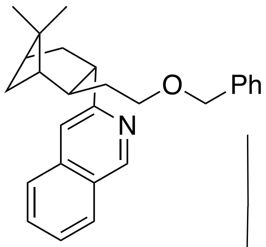

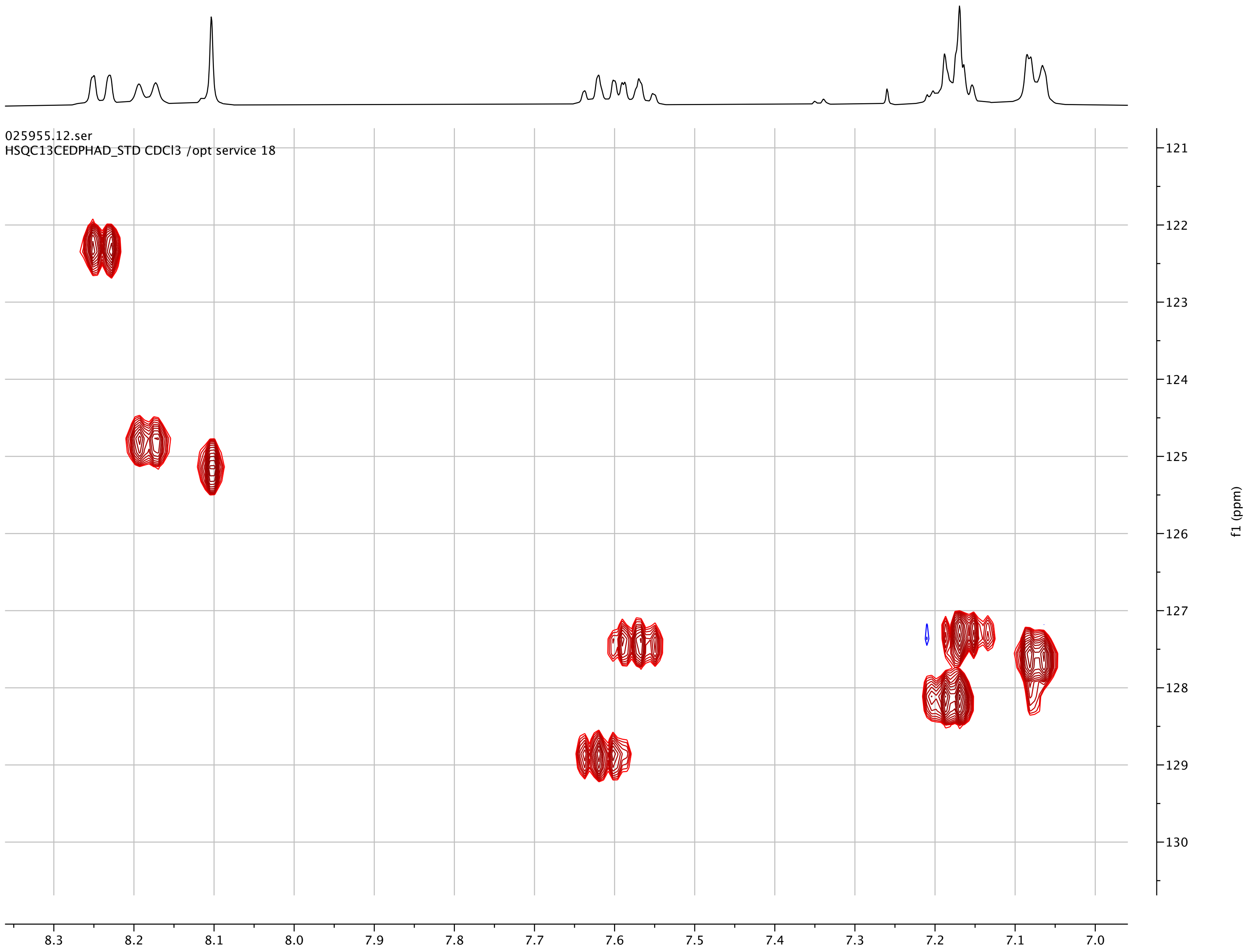

3-(2-(2-(Benzyloxy)ethyl)-6,6-dimethylbicyclo[3.1.1]heptan-3-yl)isoquinoline (C3-23, minor)

HSQC (400 MHz, $CDCl_3$)

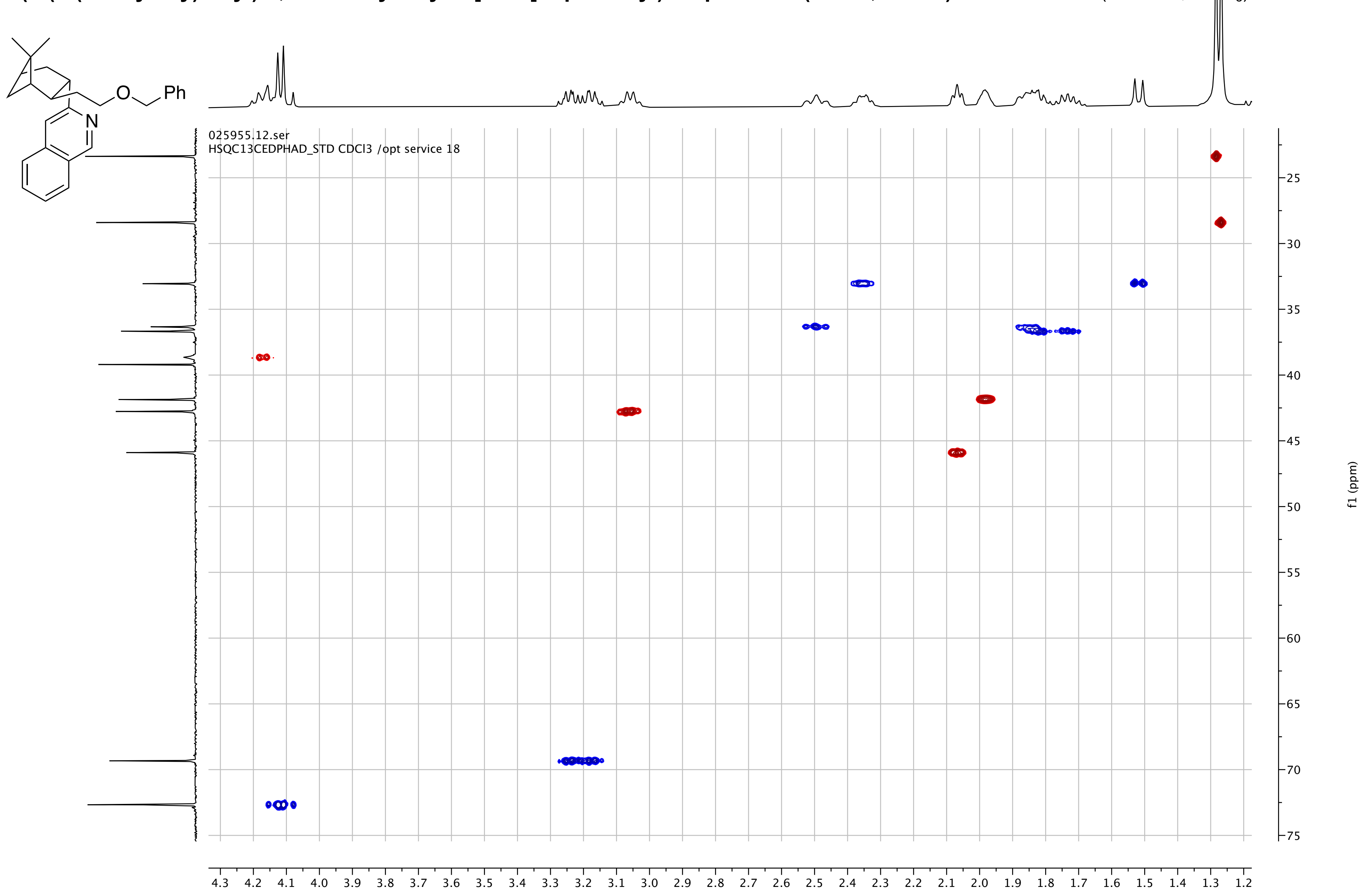

3-(2-(2-(Benzyloxy)ethyl)-6,6-dimethylbicyclo[3.1.1]heptan-3-yl)isoquinoline (C3-23, minor) HMBC (400 MHz, $CDCl_3$)

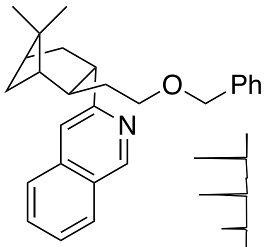

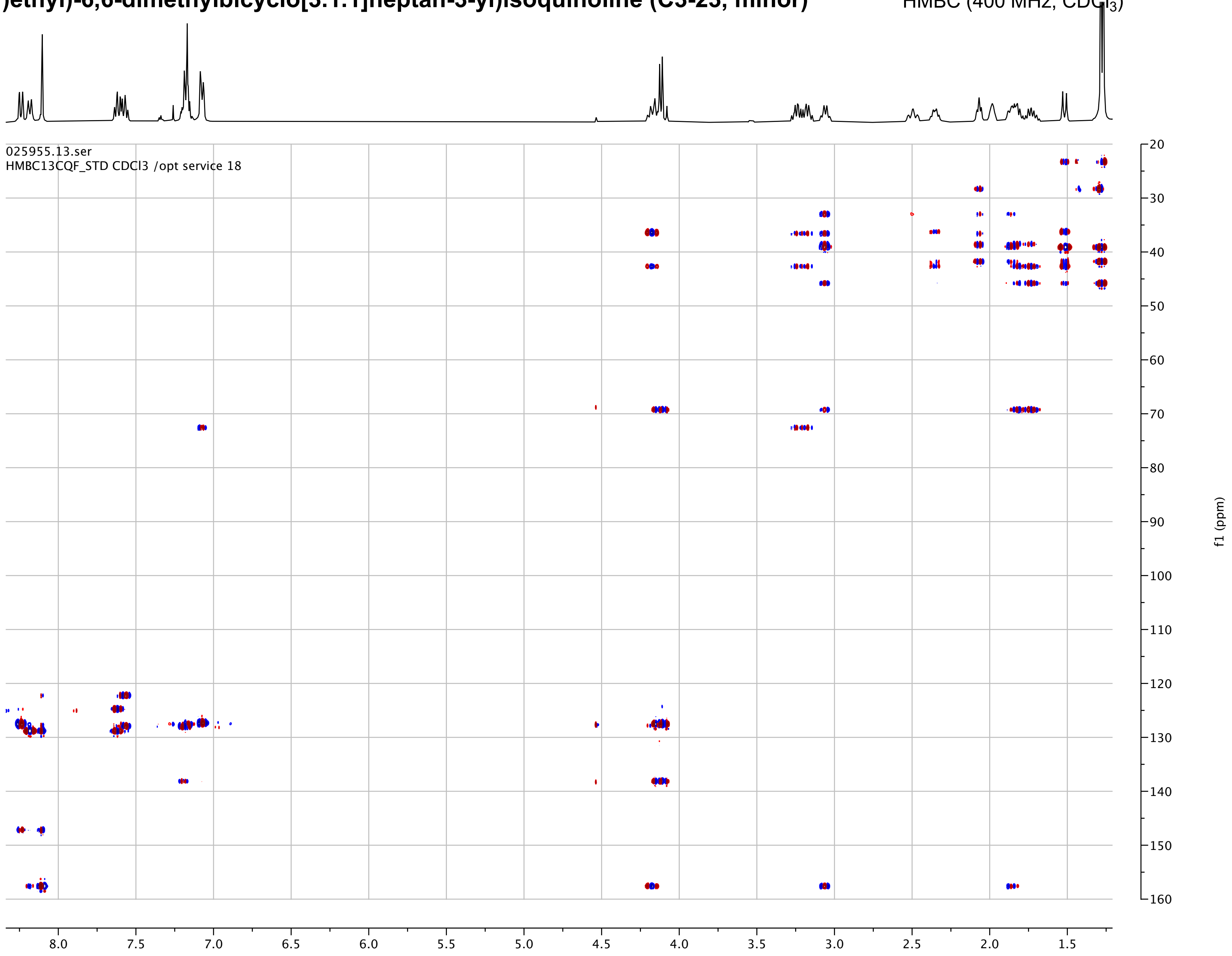

3-(2-(2-(Benzyloxy)ethyl)-6,6-dimethylbicyclo[3.1.1]heptan-3-yl)isoquinoline (C3-23, minor)

HMBC (400 MHz, $CDCl_3$)

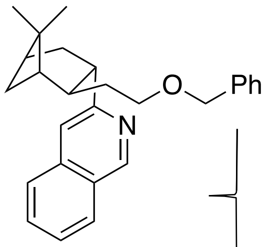

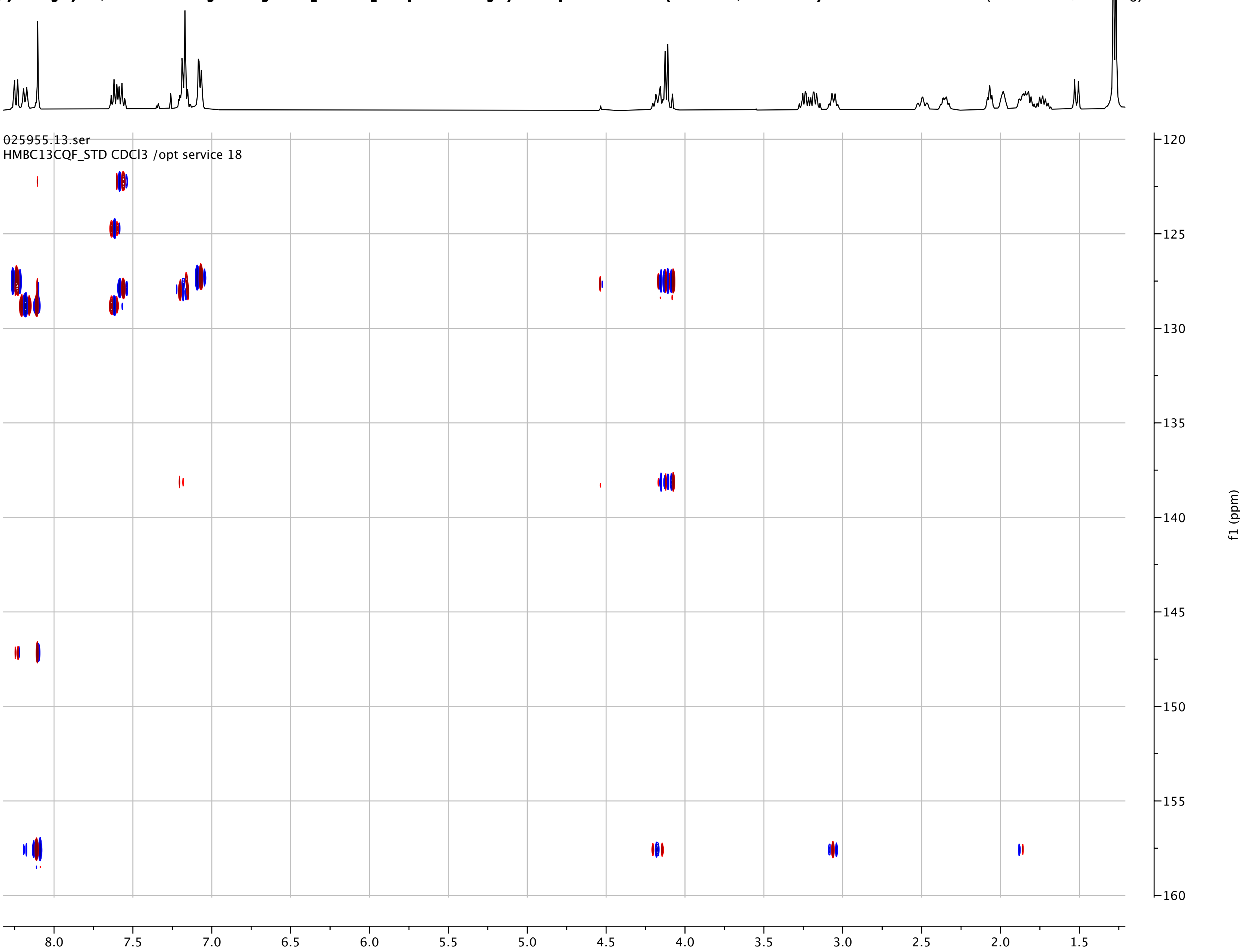

3-(2-(2-(Benzyloxy)ethyl)-6,6-dimethylbicyclo[3.1.1]heptan-3-yl)isoquinoline (C3-23, minor)

HMBC (400 MHz, $CDCl_3$)

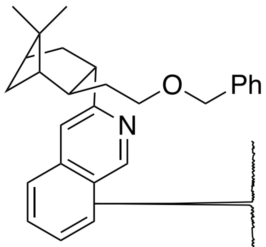

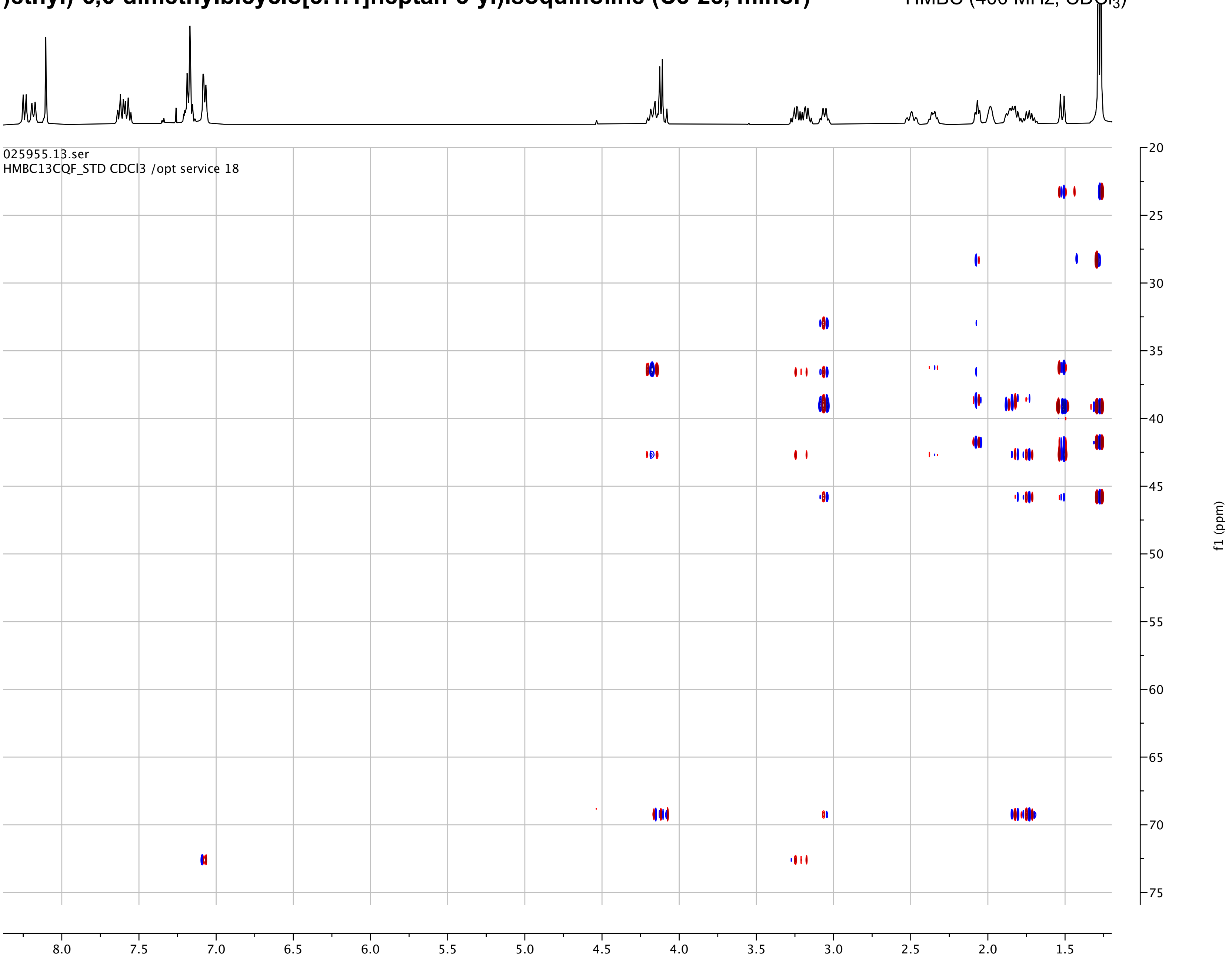

2-(2-(2-(Benzyloxy)ethyl)-6,6-dimethylbicyclo[3.1.1]heptan-3-yl)-6-fluoroquinoline (24)

^{1}H-NMR (400 MHz, $CDCl_3$)

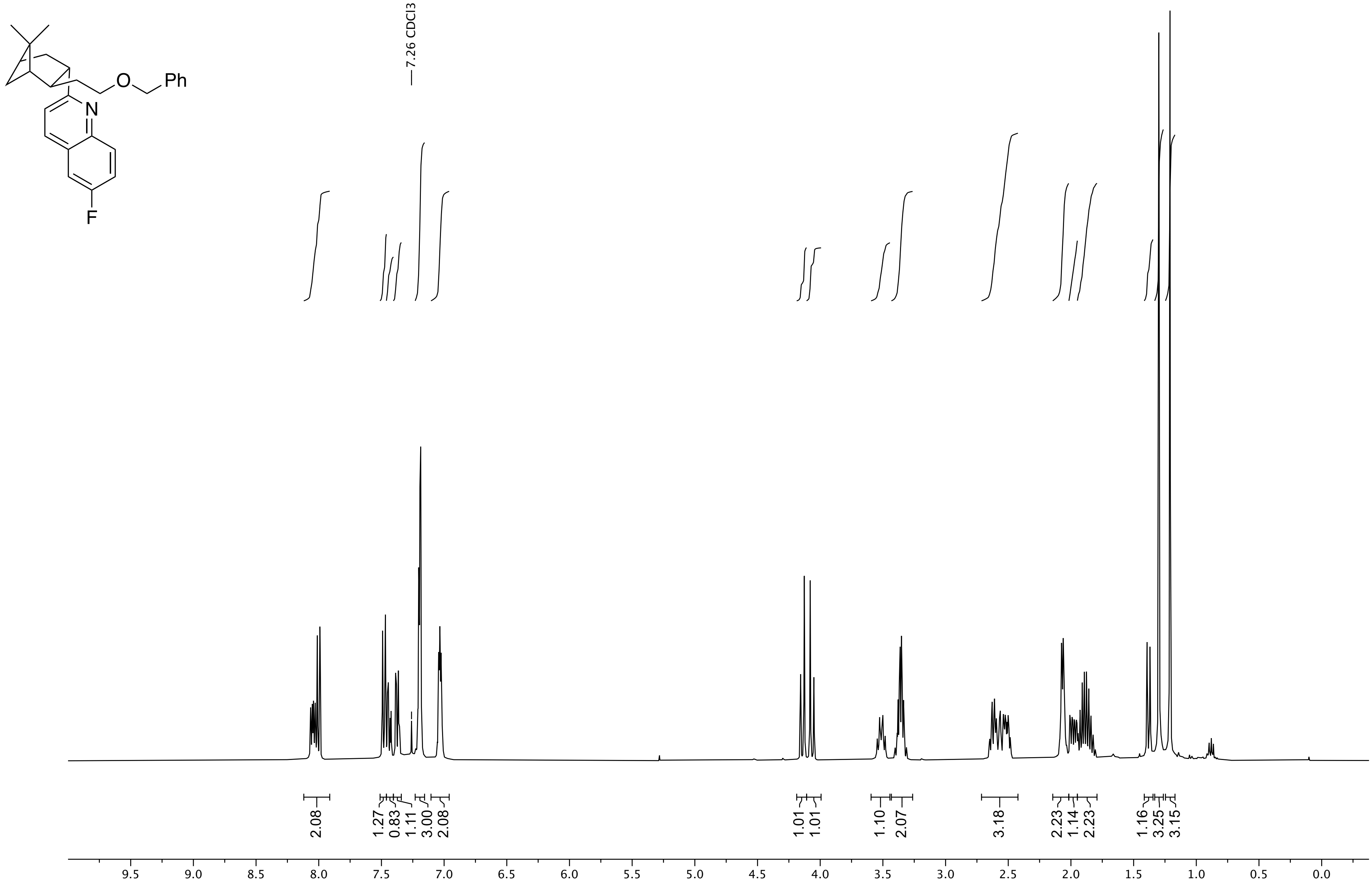

2-(2-(2-(Benzyloxy)ethyl)-6,6-dimethylbicyclo[3.1.1]heptan-3-yl)-6-fluoroquinoline (24)

^{1}H-NMR (400 MHz, $CDCl_3$)

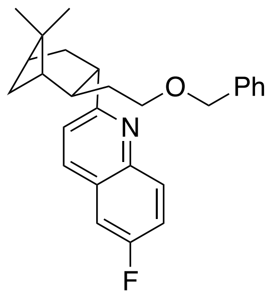

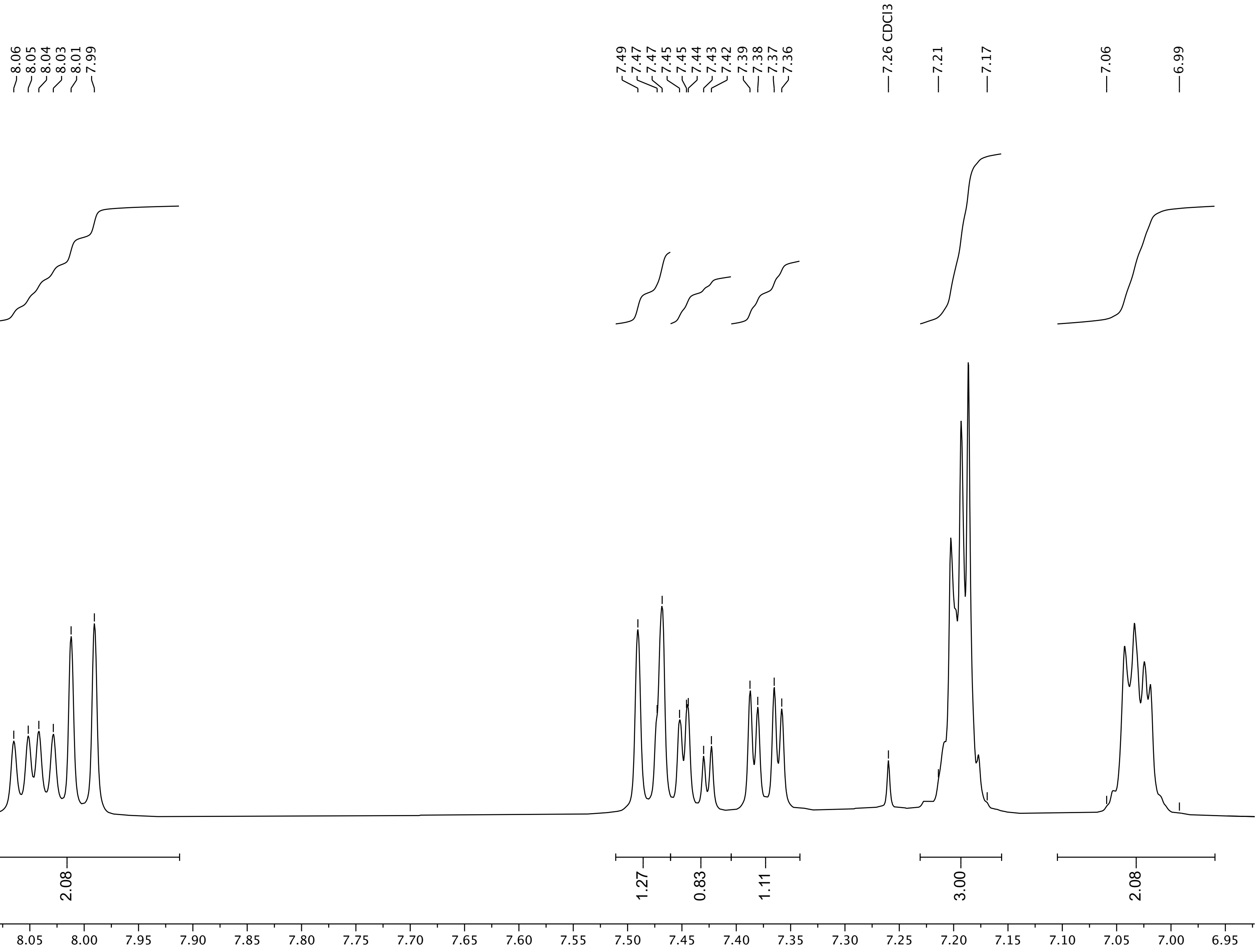

2-(2-(2-(Benzyloxy)ethyl)-6,6-dimethylbicyclo[3.1.1]heptan-3-yl)-6-fluoroquinoline (24)

^{1}H-NMR (400 MHz, $CDCl_3$)

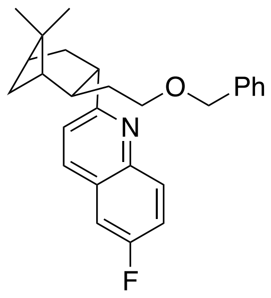

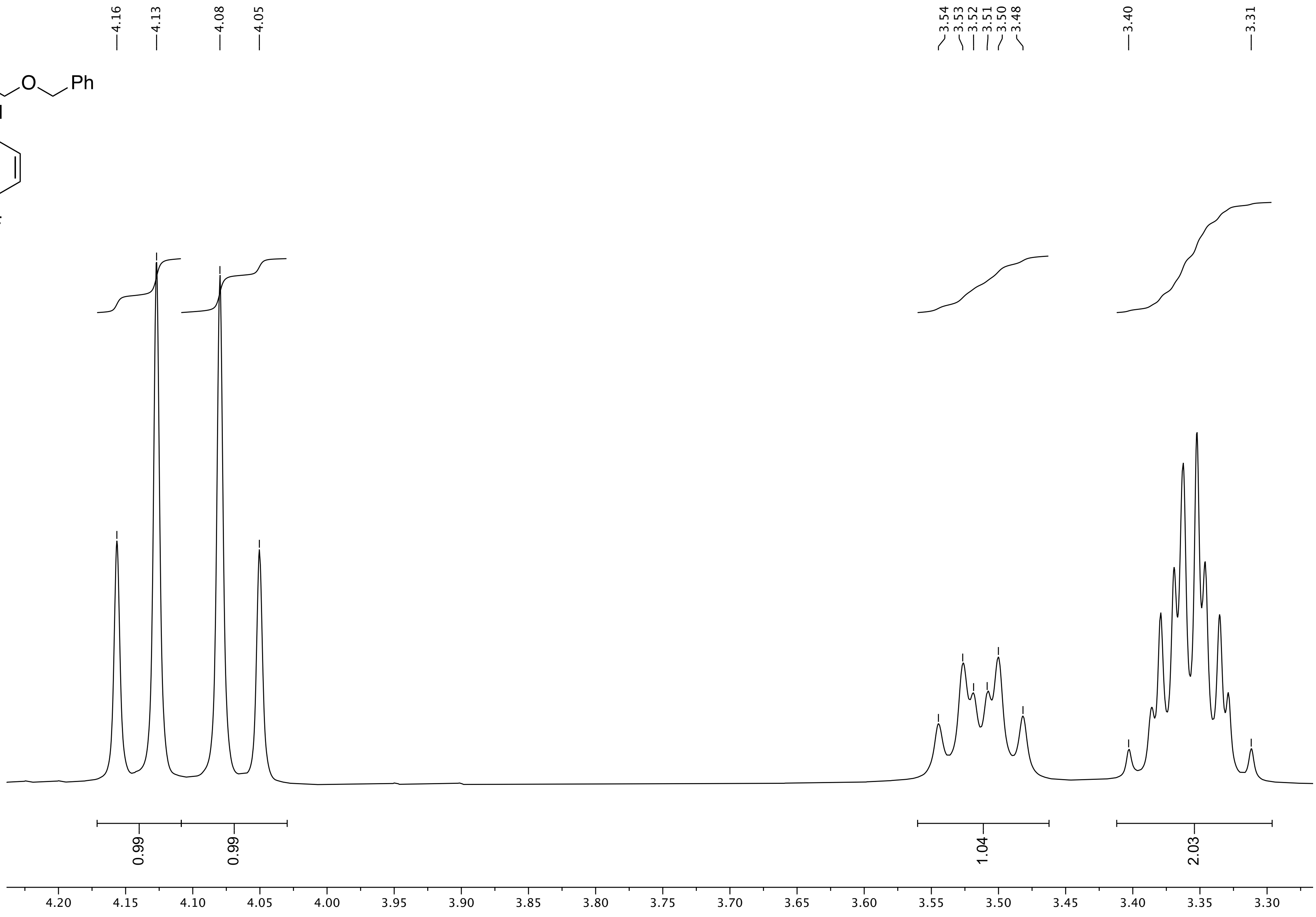

2-(2-(2-(Benzyloxy)ethyl)-6,6-dimethylbicyclo[3.1.1]heptan-3-yl)-6-fluoroquinoline (24)

^{1}H-NMR (400 MHz, $CDCl_3$)

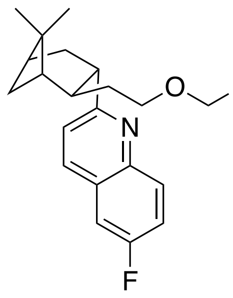

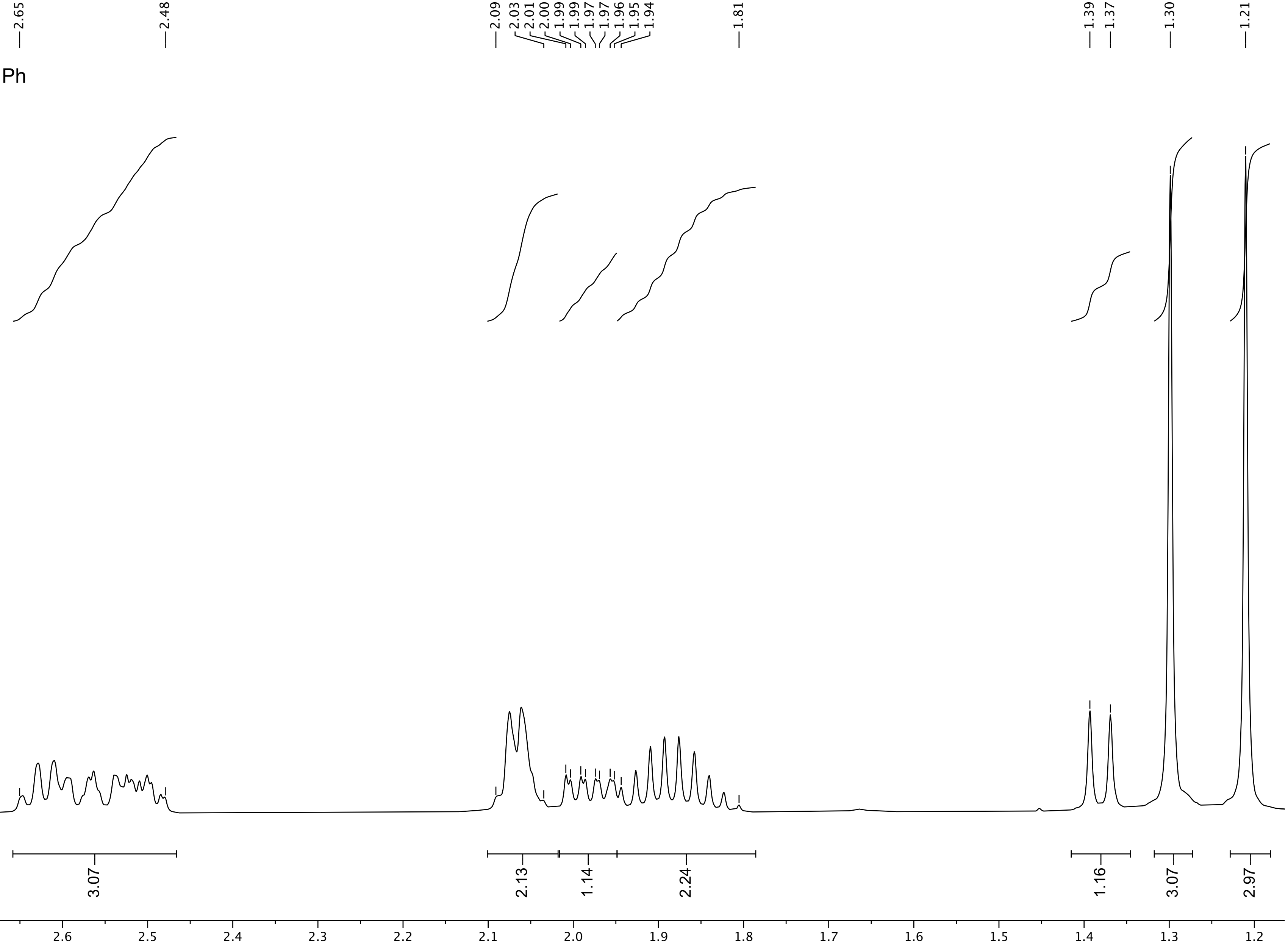

2-(2-(2-(Benzyloxy)ethyl)-6,6-dimethylbicyclo[3.1.1]heptan-3-yl)-6-fluoroquinoline (24)

^{13}C-NMR (101 MHz, $CDCl_3$)

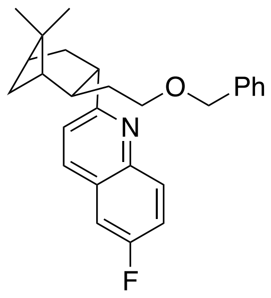

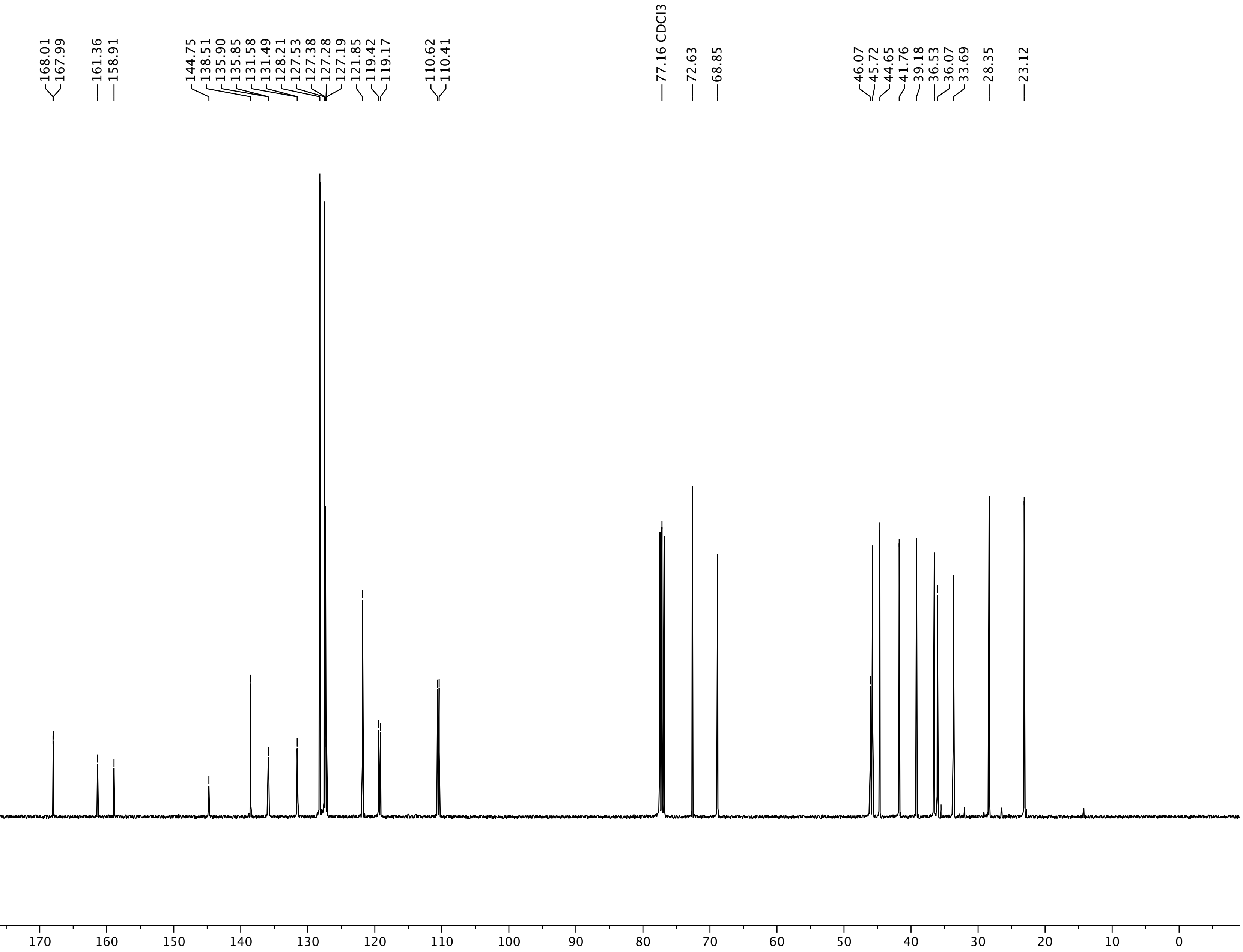

2-(2-(2-(Benzyloxy)ethyl)-6,6-dimethylbicyclo[3.1.1]heptan-3-yl)-6-fluoroquinoline (24)

^{13}C-NMR (101 MHz, $CDCl_3$)

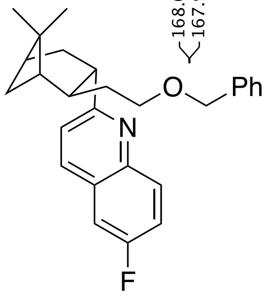

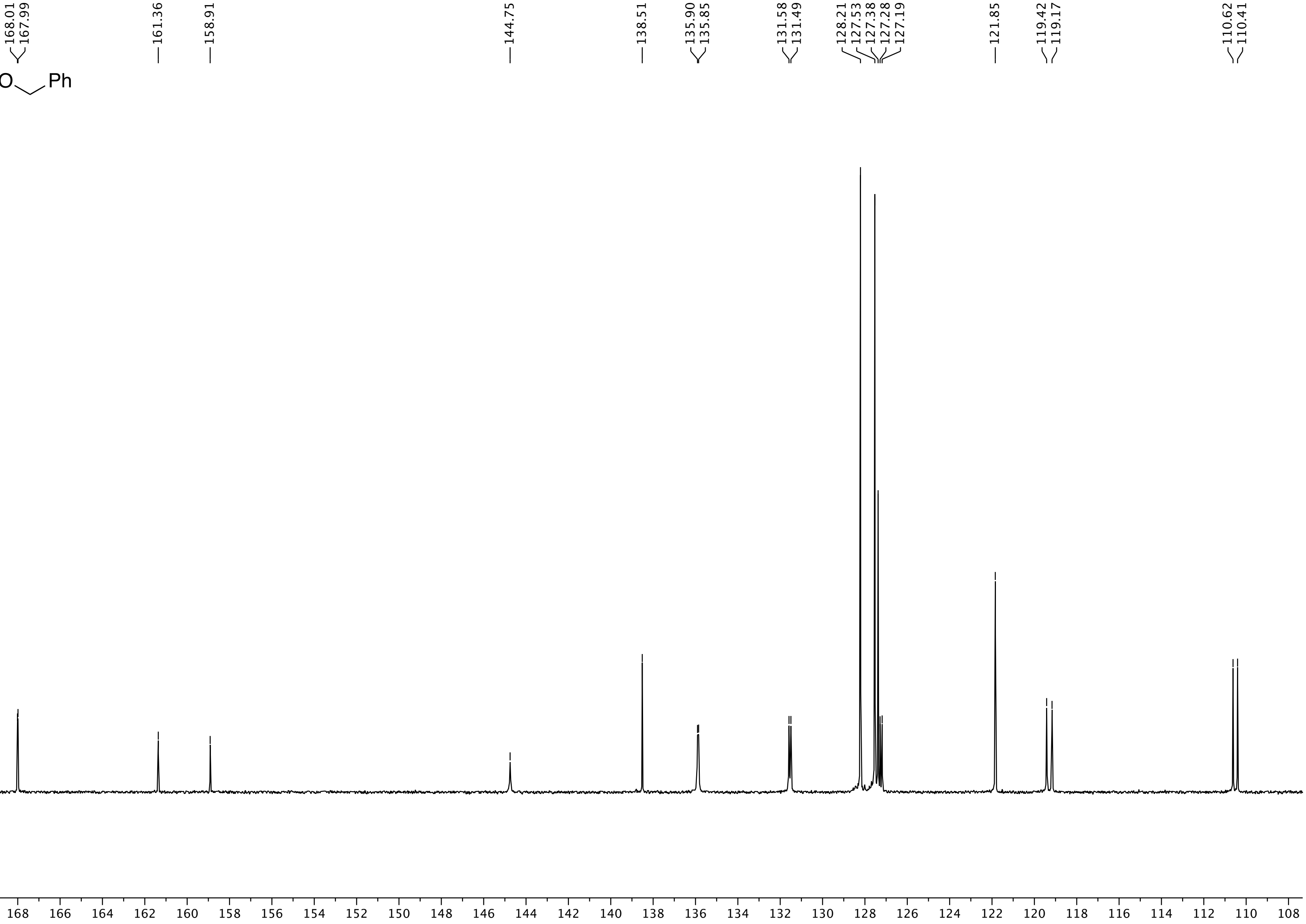

2-(2-(2-(Benzyloxy)ethyl)-6,6-dimethylbicyclo[3.1.1]heptan-3-yl)-6-fluoroquinoline (24)

^{13}C-NMR (101 MHz, CDCl$_3$)

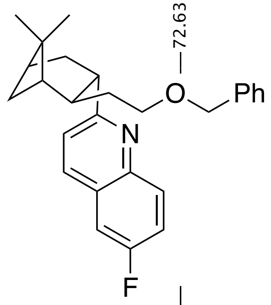

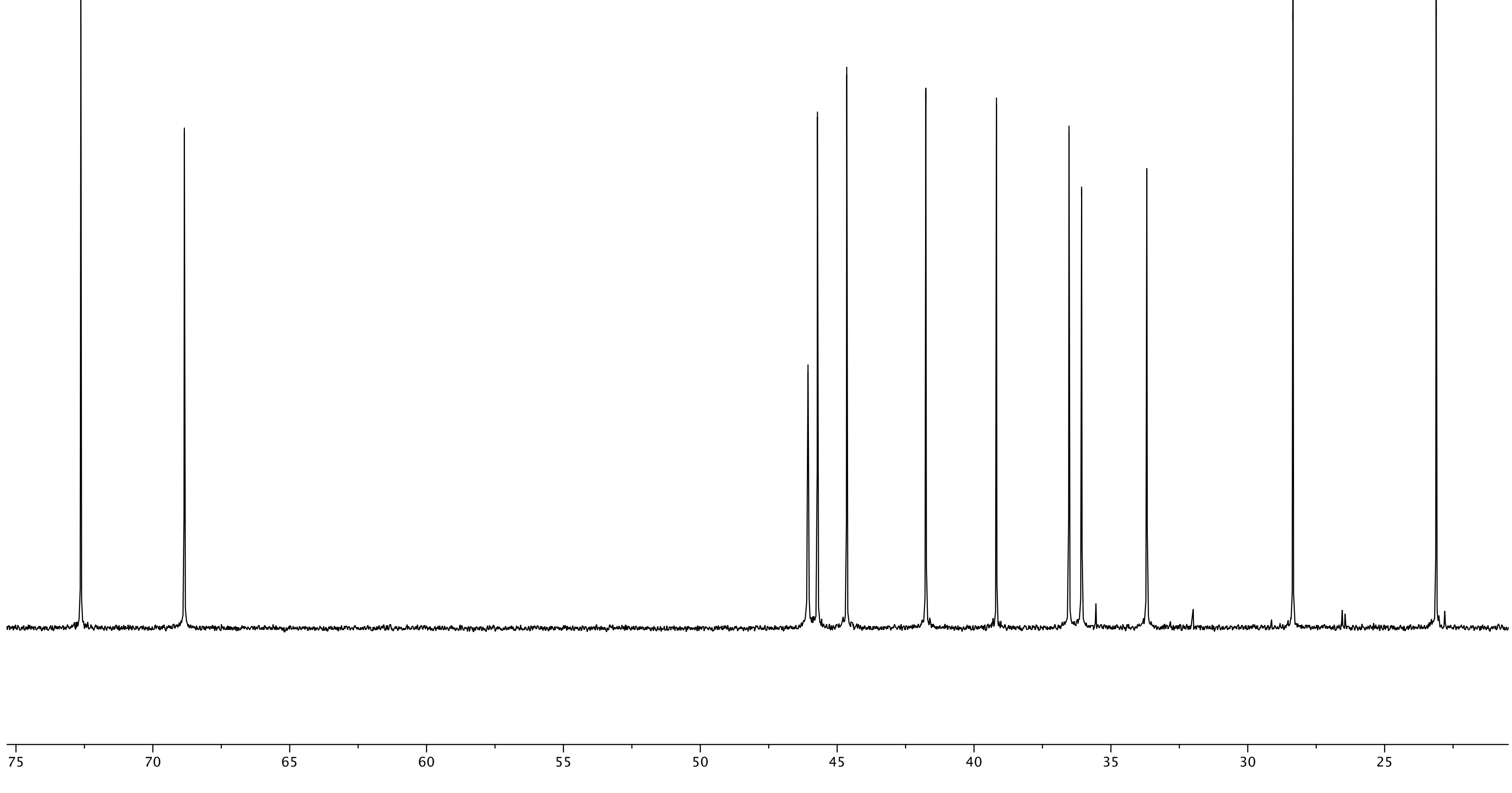

2-(2-(2-(Benzyloxy)ethyl)-6,6-dimethylbicyclo[3.1.1]heptan-3-yl)-6-fluoroquinoline (24)

Dept-135 (101 MHz, $CDCl_3$)

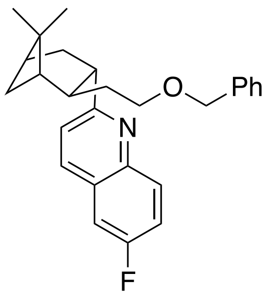

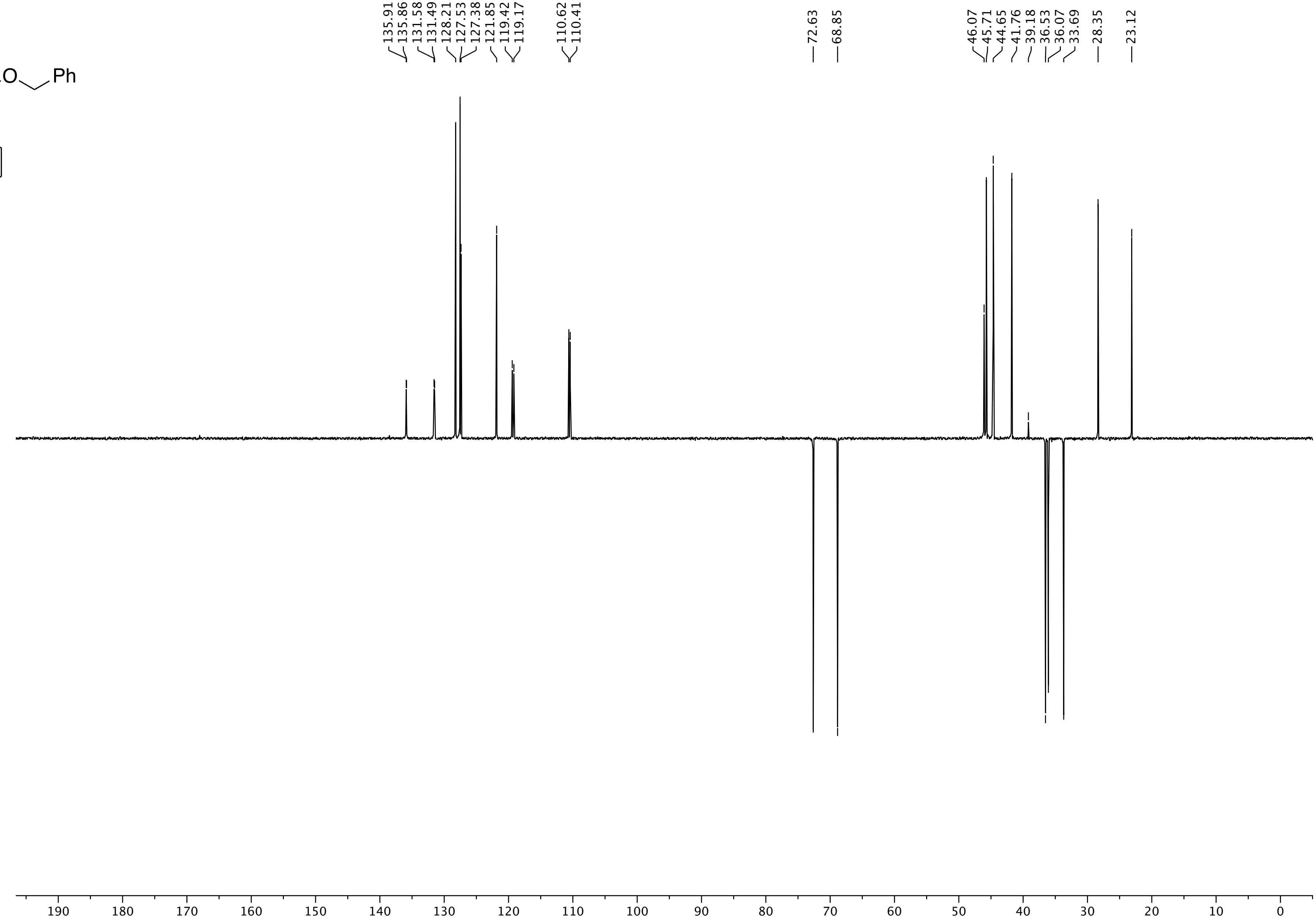

2-(2-(2-(Benzyloxy)ethyl)-6,6-dimethylbicyclo[3.1.1]heptan-3-yl)-6-fluoroquinoline (24)

Dept-135 (101 MHz, $CDCl_3$)

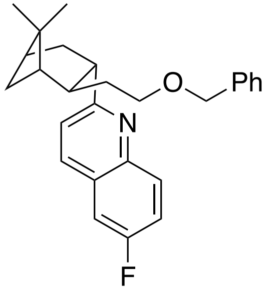

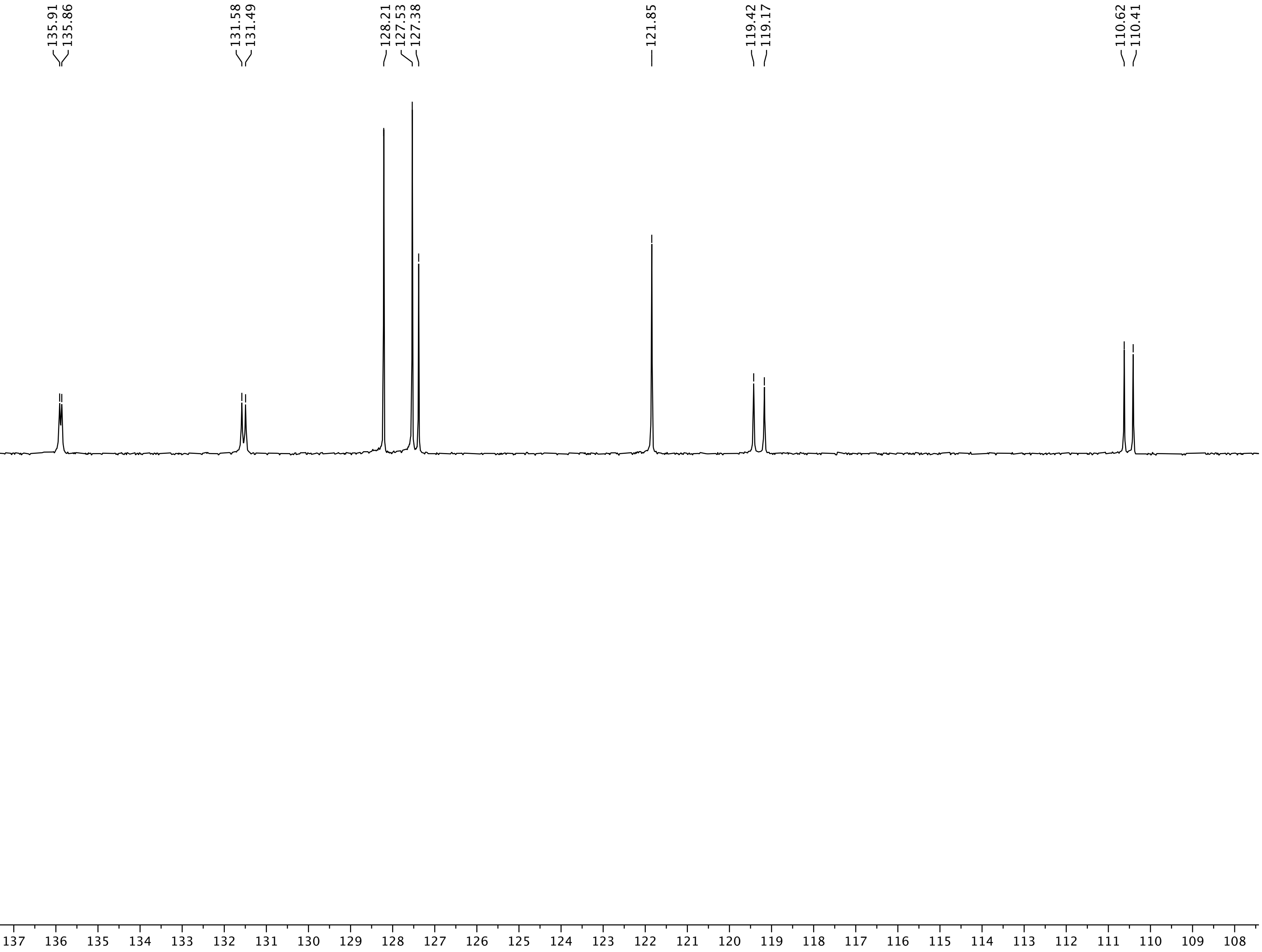

2-(2-(2-(Benzyloxy)ethyl)-6,6-dimethylbicyclo[3.1.1]heptan-3-yl)-6-fluoroquinoline (24)

Dept-135 (101 MHz, $CDCl_3$)

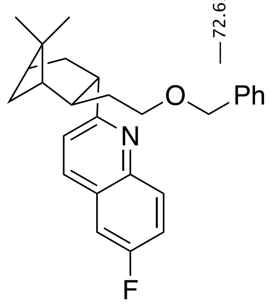

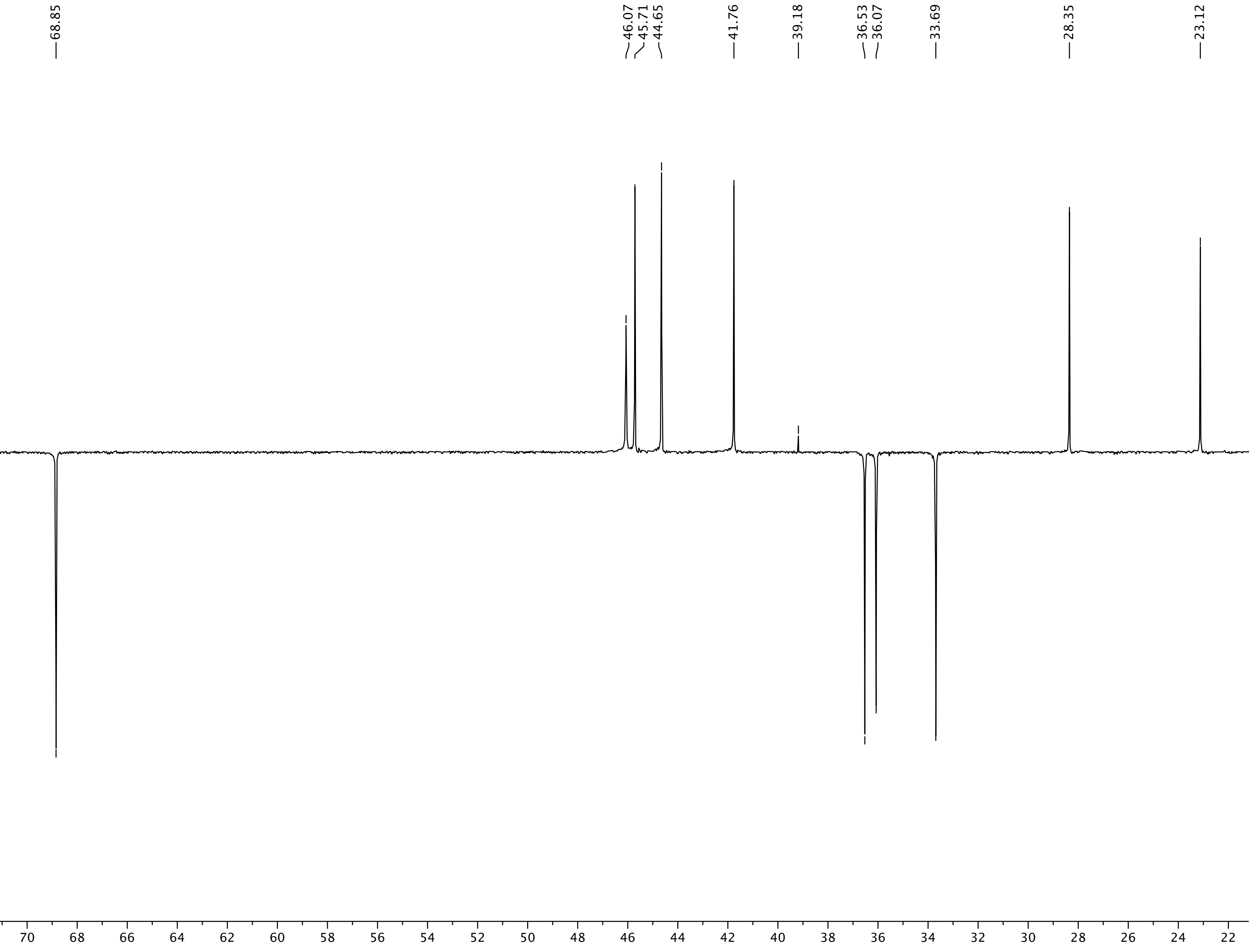

2-(2-(2-(Benzyloxy)ethyl)-6,6-dimethylbicyclo[3.1.1]heptan-3-yl)-6-fluoroquinoline (24)

^1H-^1H COSY (400 MHz, $CDCl_3$)

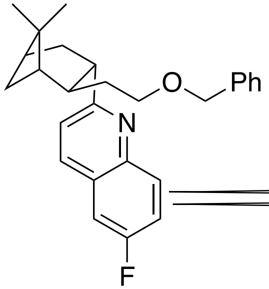

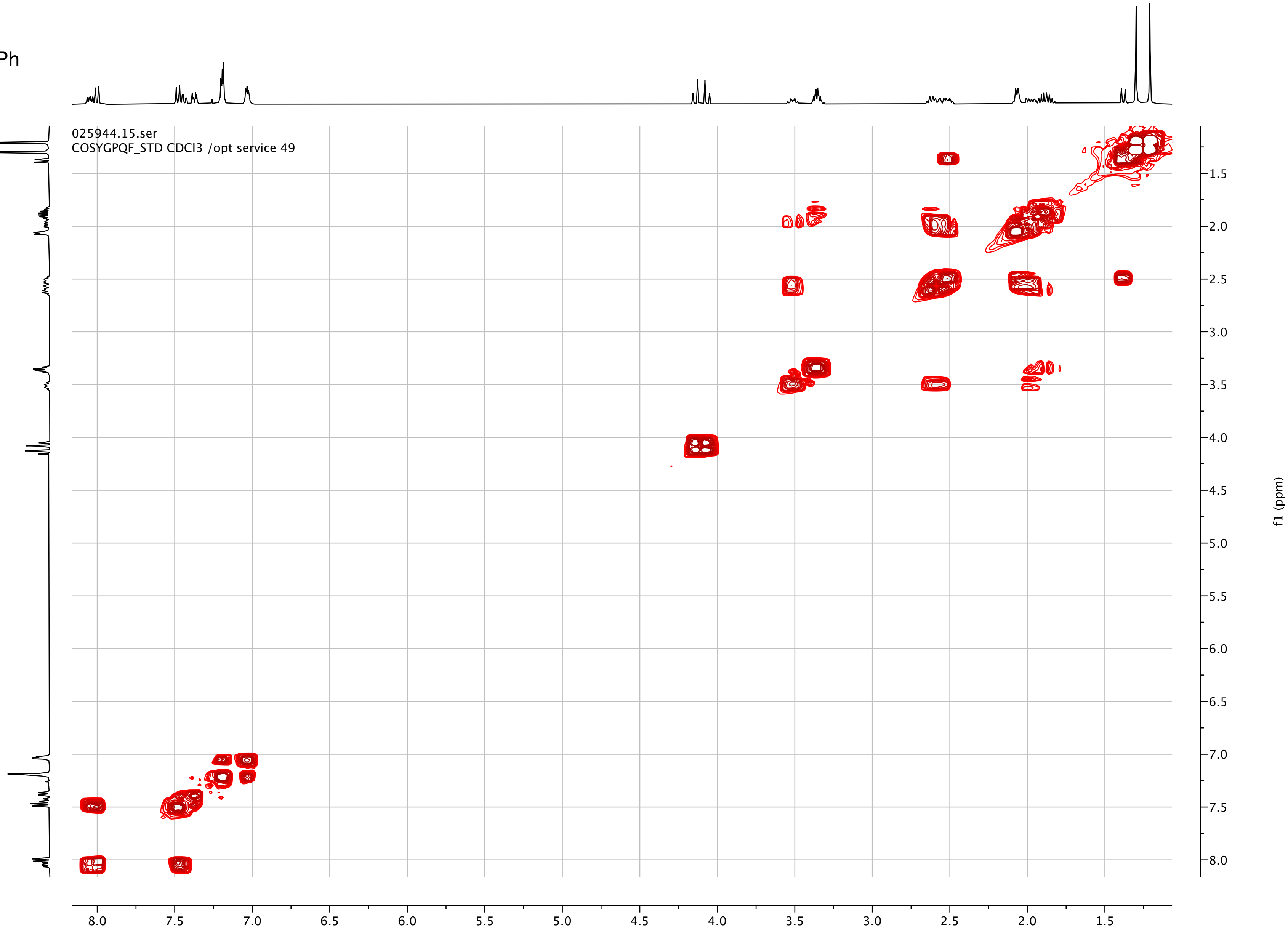

2-(2-(2-(Benzyloxy)ethyl)-6,6-dimethylbicyclo[3.1.1]heptan-3-yl)-6-fluoroquinoline (24)

^{1}H-^{1}H COSY (400 MHz, $CDCl_3$)

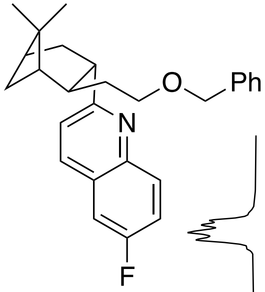

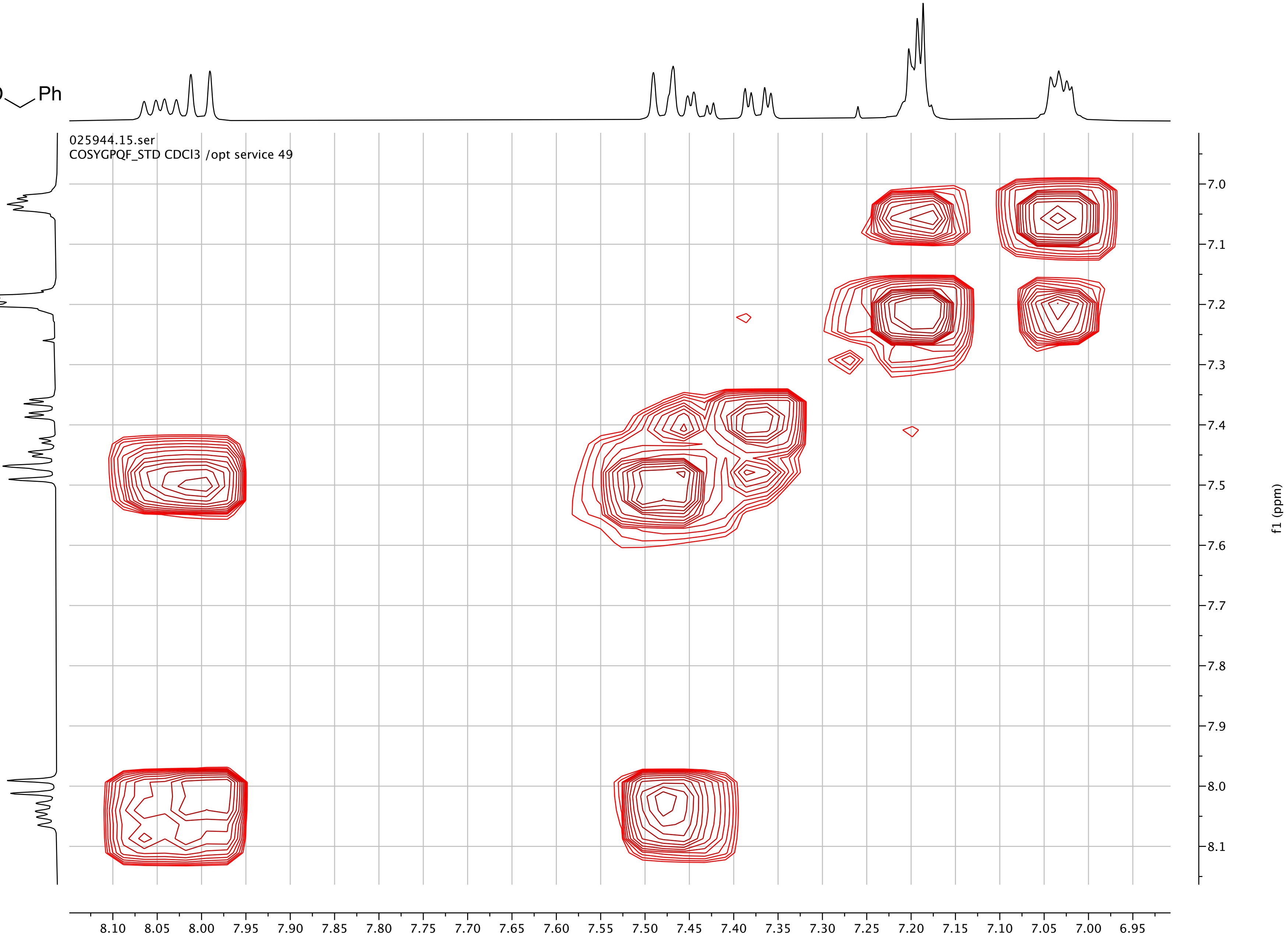

2-(2-(2-(Benzyloxy)ethyl)-6,6-dimethylbicyclo[3.1.1]heptan-3-yl)-6-fluoroquinoline (24)

^{1}H-^{1}H COSY (400 MHz, $CDCl_3$)

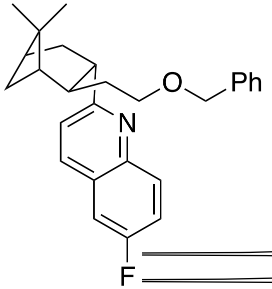

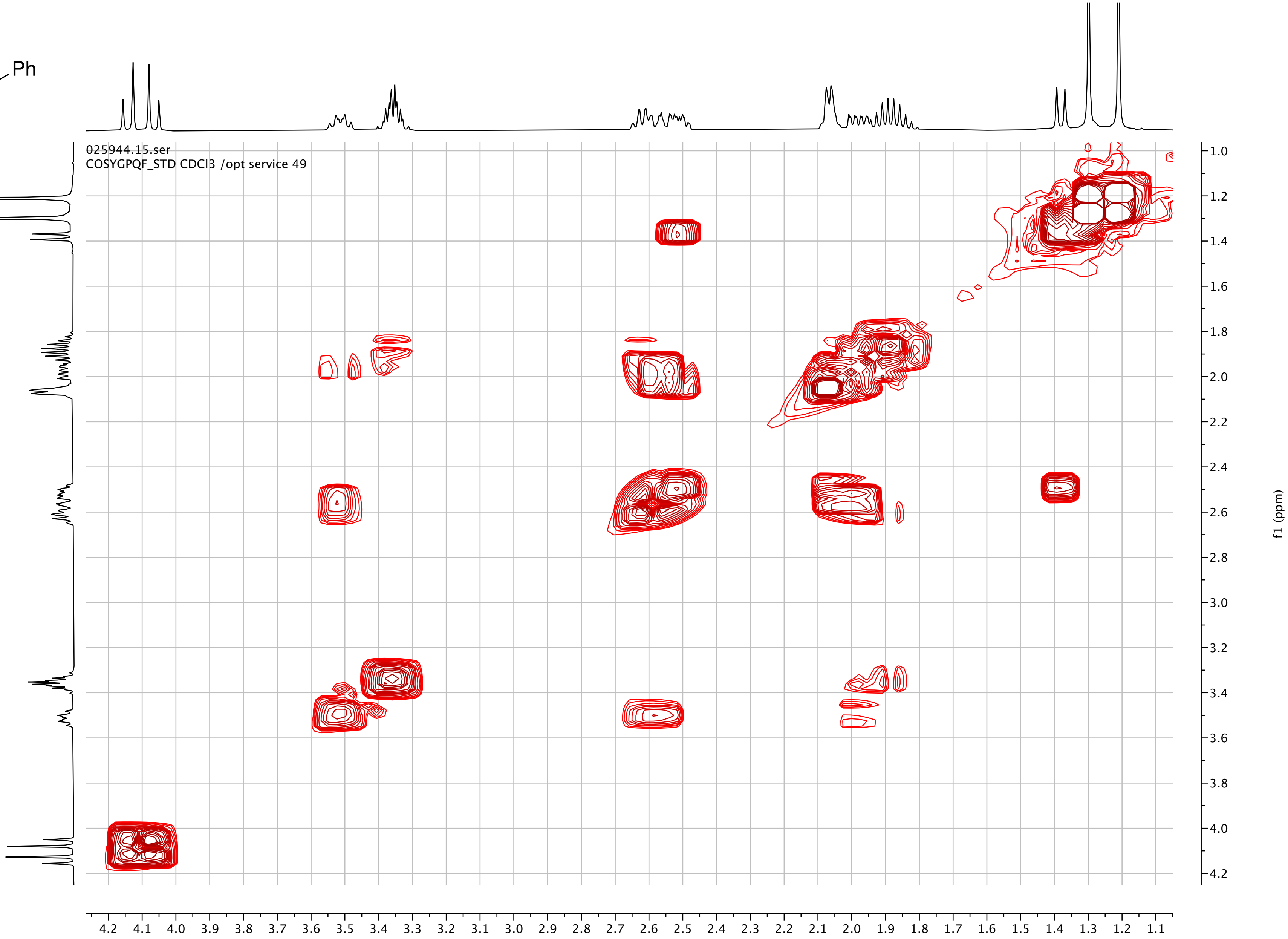

2-(2-(2-(Benzyloxy)ethyl)-6,6-dimethylbicyclo[3.1.1]heptan-3-yl)-6-fluoroquinoline (24) HSQC (400 MHz, $CDCl_3$)

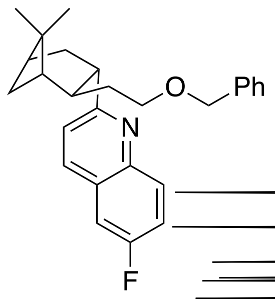

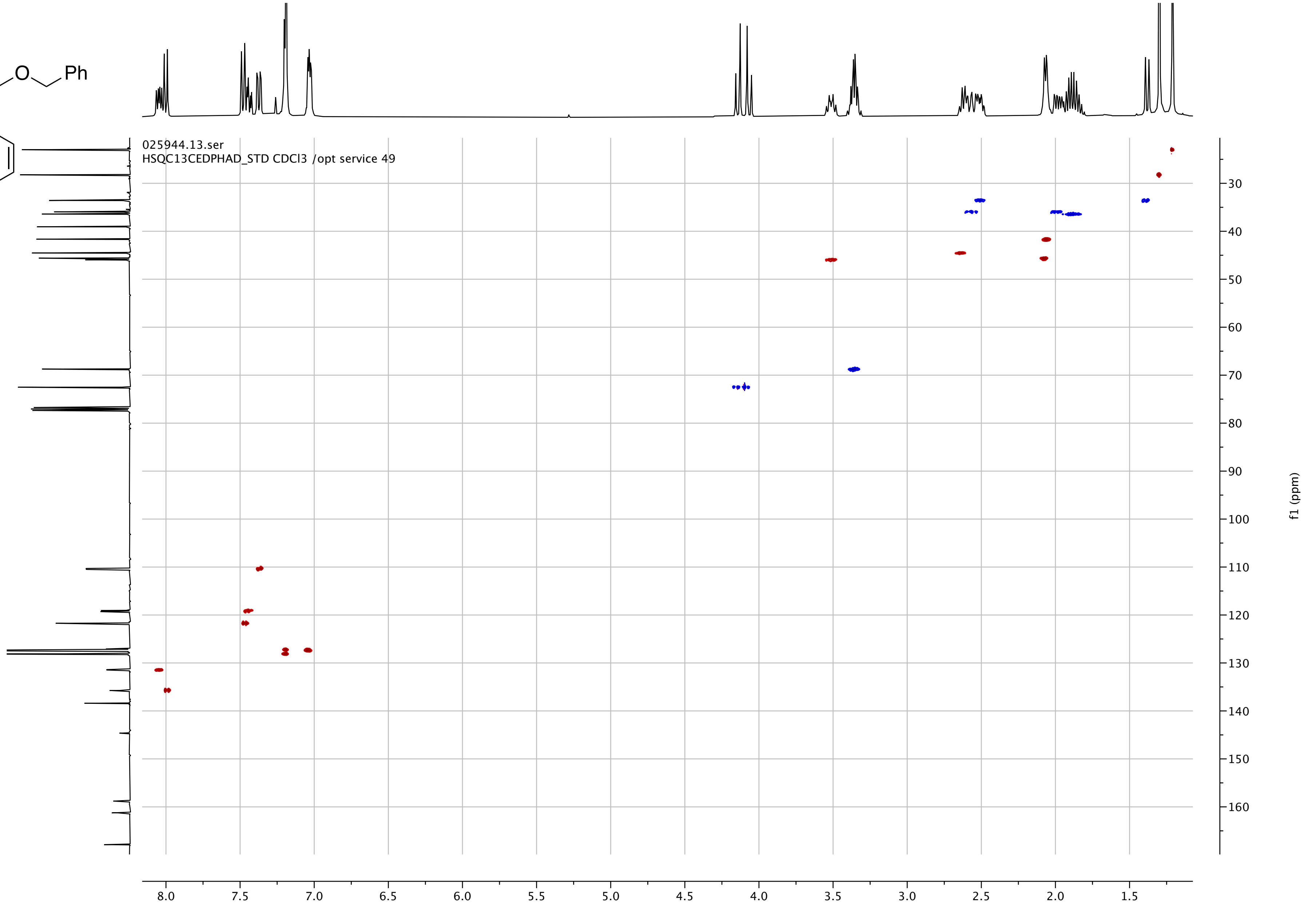

2-(2-(2-(Benzyloxy)ethyl)-6,6-dimethylbicyclo[3.1.1]heptan-3-yl)-6-fluoroquinoline (24)

HSQC (400 MHz, $CDCl_3$)

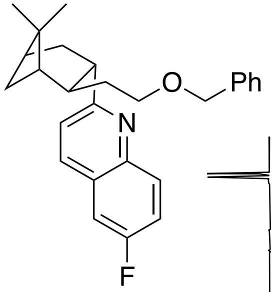

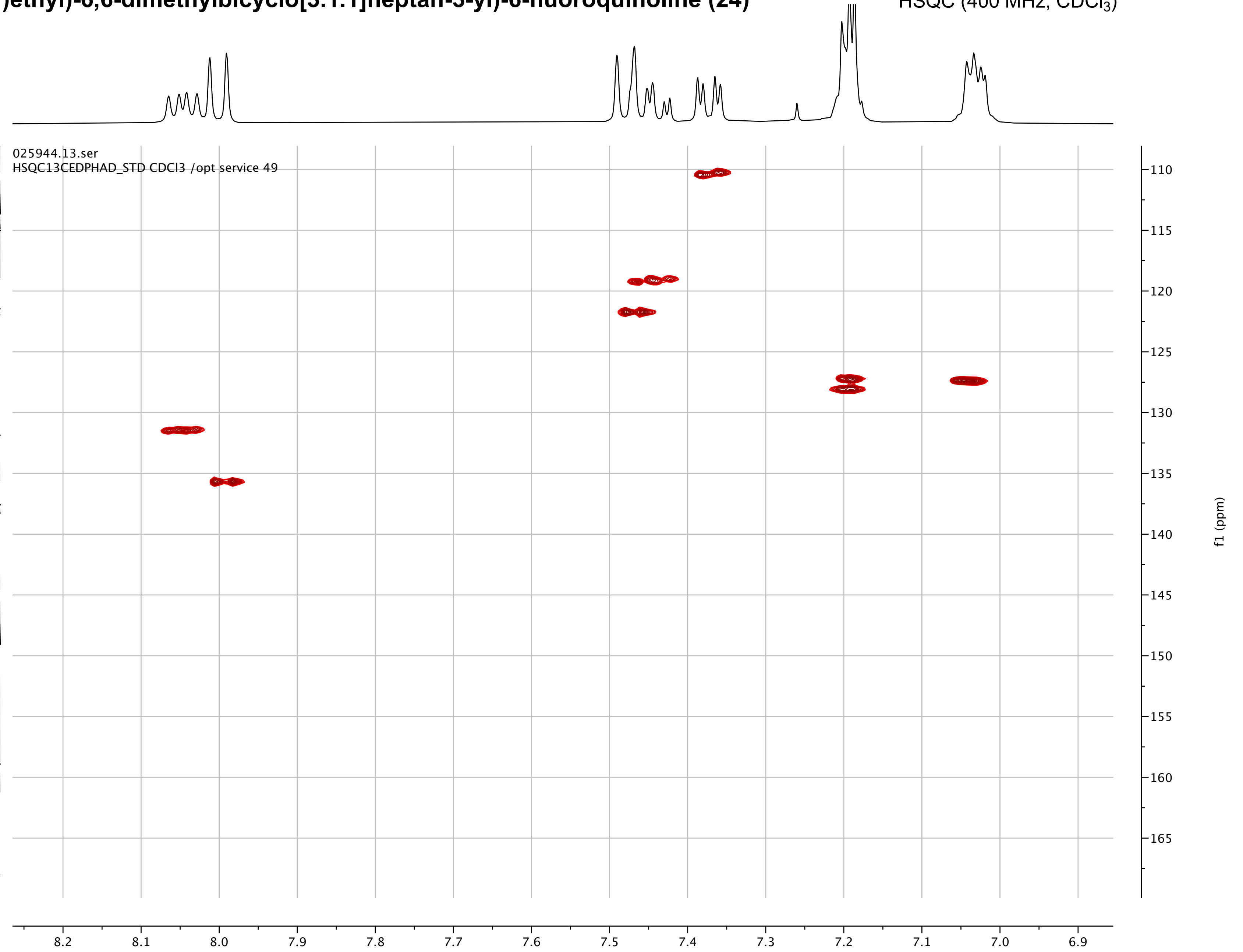

2-(2-(2-(Benzyloxy)ethyl)-6,6-dimethylbicyclo[3.1.1]heptan-3-yl)-6-fluoroquinoline (24)

HSQC (400 MHz, $CDCl_3$)

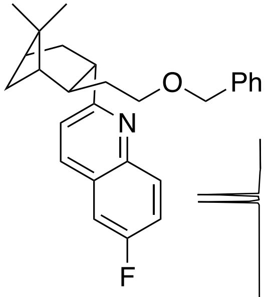

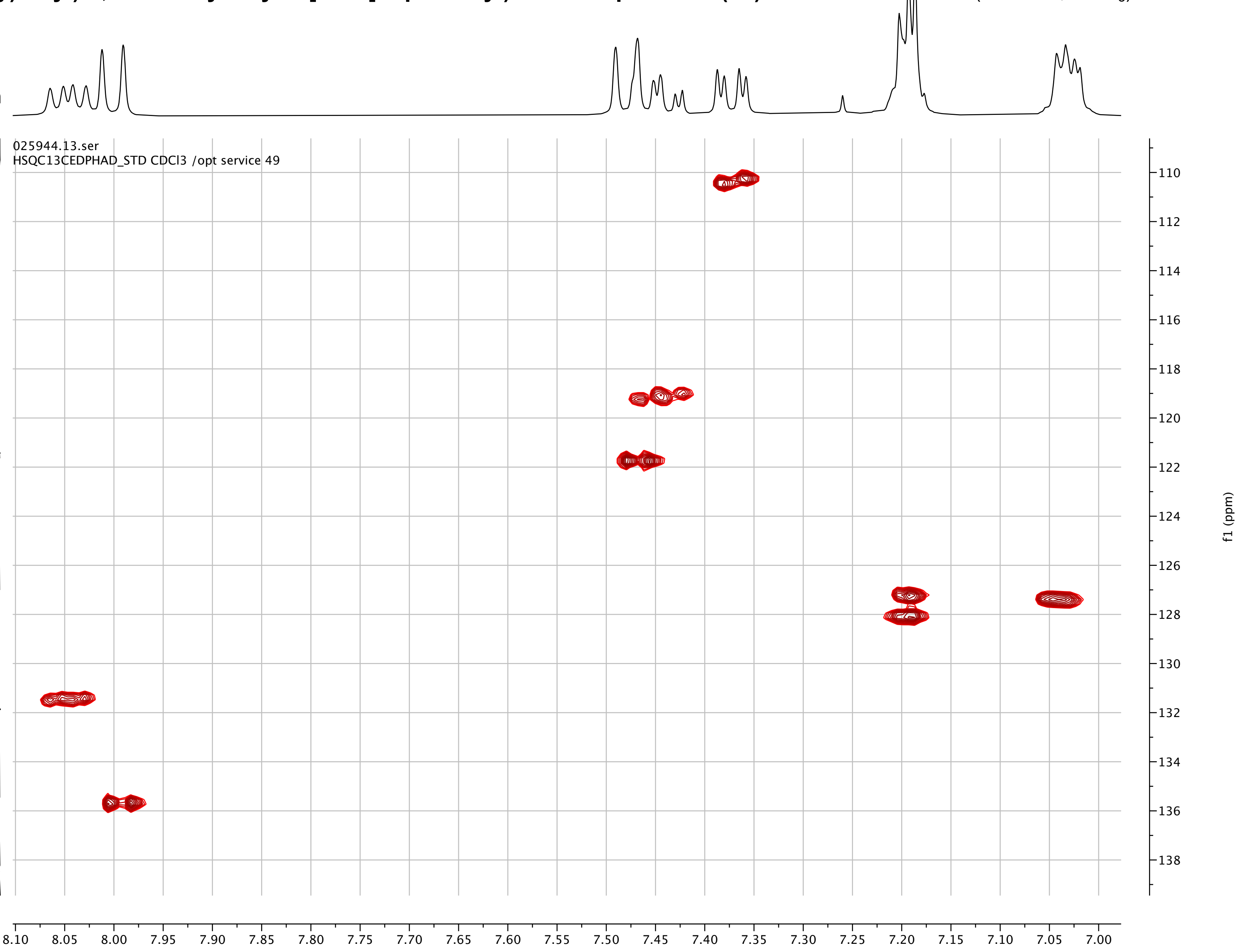

2-(2-(2-(Benzyloxy)ethyl)-6,6-dimethylbicyclo[3.1.1]heptan-3-yl)-6-fluoroquinoline (24)

HSQC (400 MHz, $CDCl_3$)

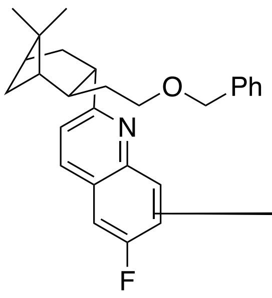

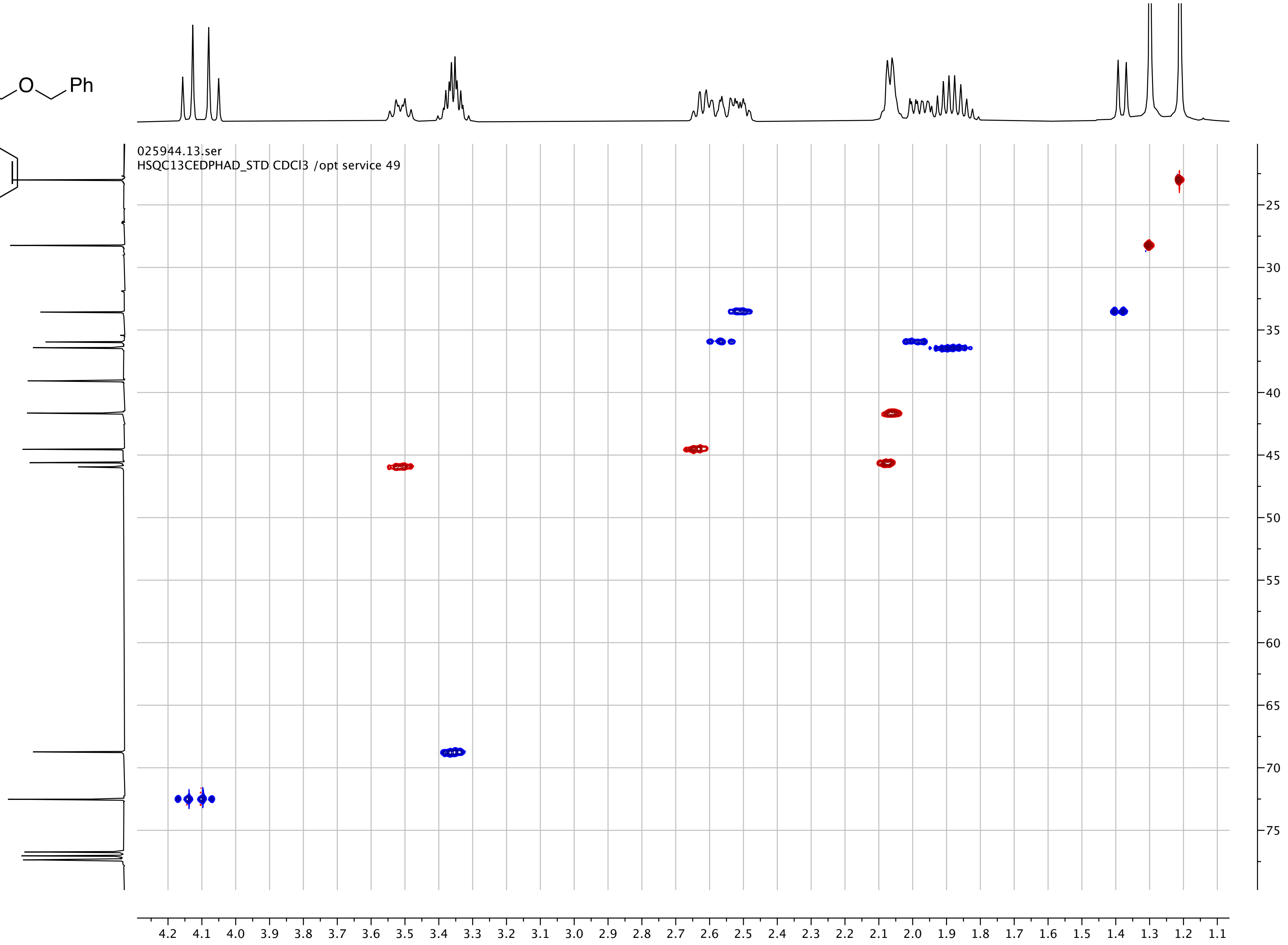

2-(2-(2-(Benzyloxy)ethyl)-6,6-dimethylbicyclo[3.1.1]heptan-3-yl)-6-fluoroquinoline (24)

HMBC (400 MHz, $CDCl_3$)

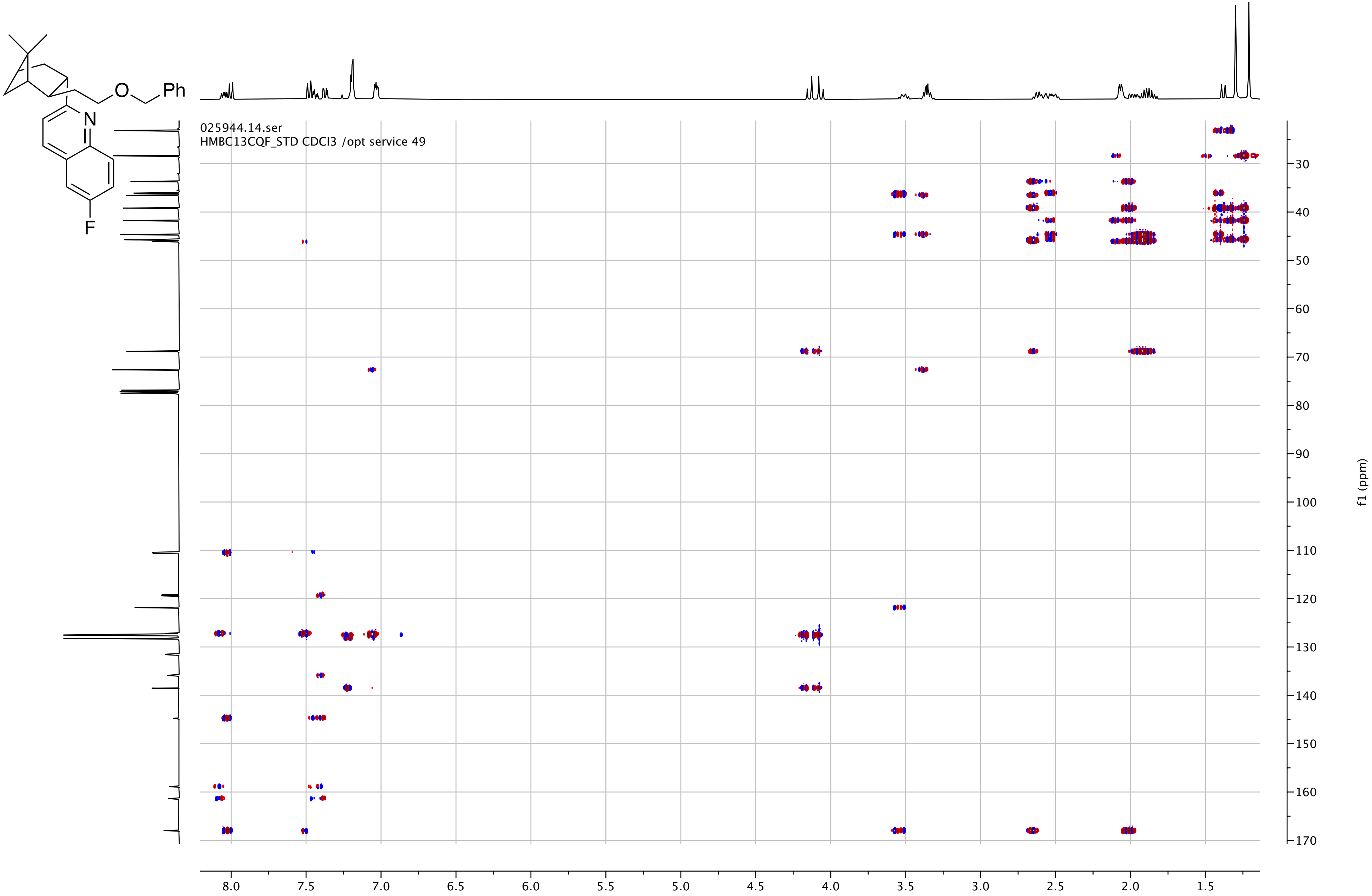

2-(2-(2-(Benzyloxy)ethyl)-6,6-dimethylbicyclo[3.1.1]heptan-3-yl)-6-fluoroquinoline (24)

HMBC (400 MHz, $CDCl_3$)

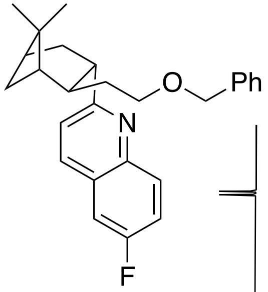

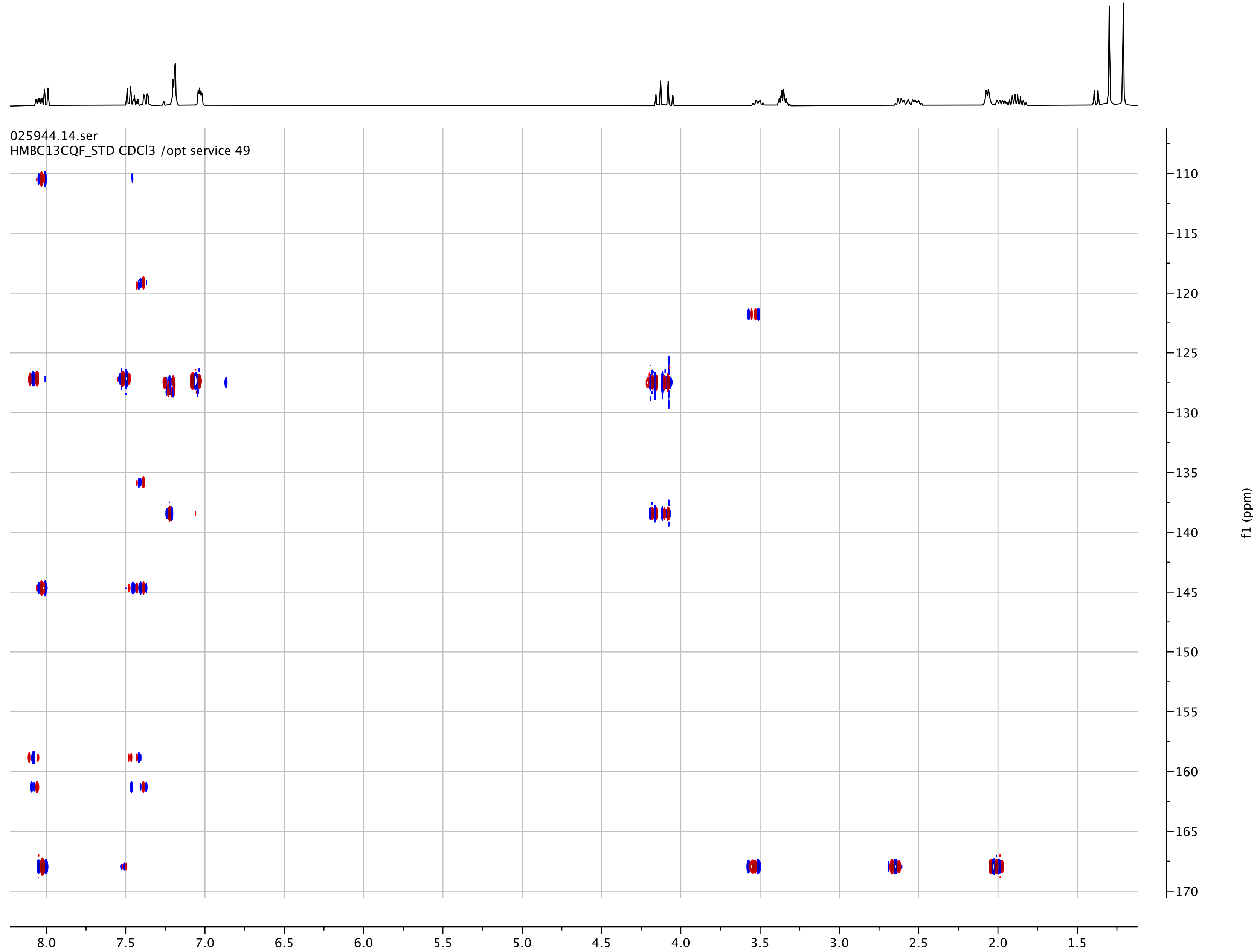

2-(2-(2-(Benzyloxy)ethyl)-6,6-dimethylbicyclo[3.1.1]heptan-3-yl)-6-fluoroquinoline (24)

HMBC (400 MHz, $CDCl_3$)

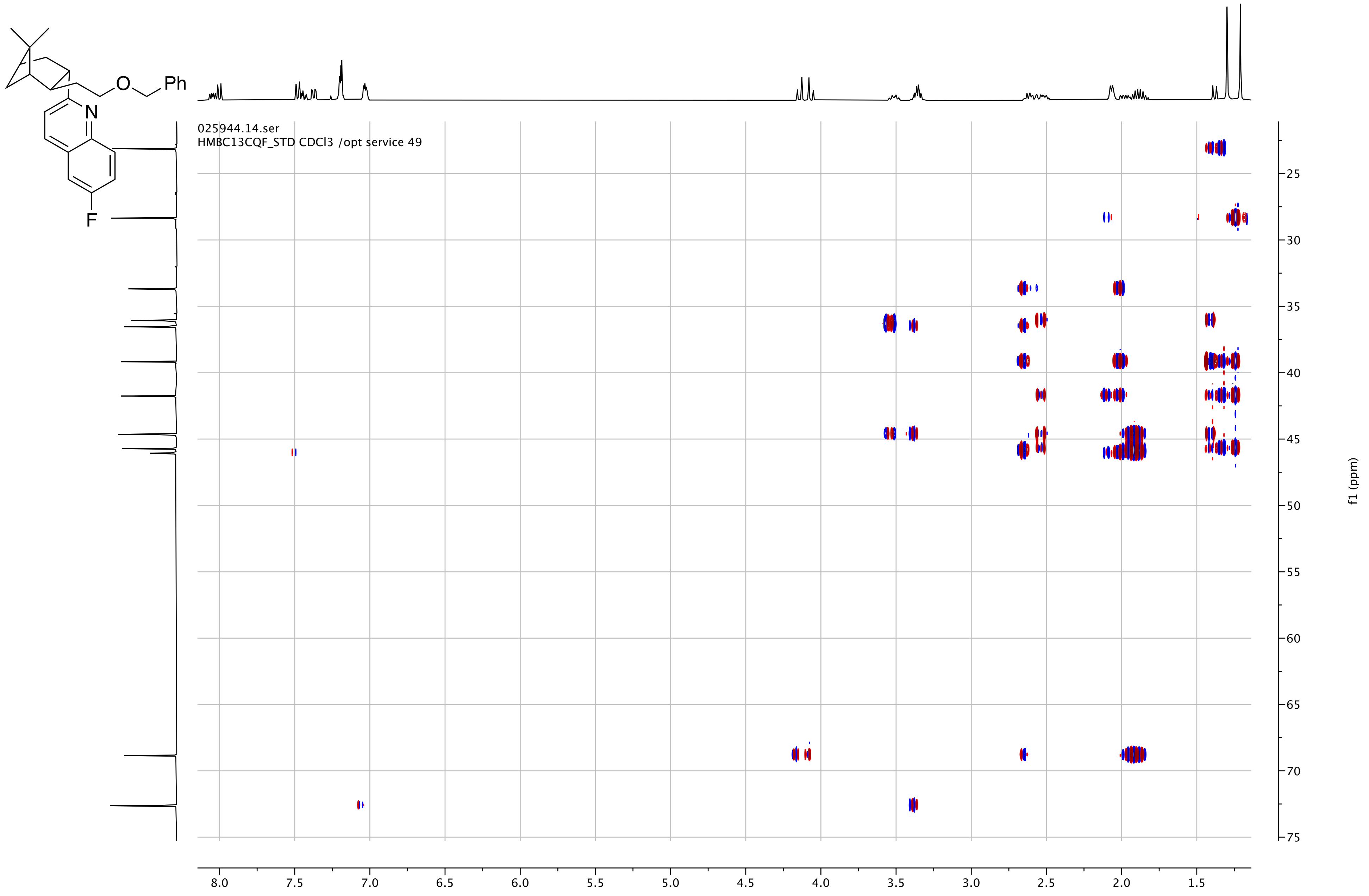

2-(2-(2-(Benzyloxy)ethyl)-6,6-dimethylbicyclo[3.1.1]heptan-3-yl)-6-fluoroquinoline (24)

NOESY (400 MHz, $CDCl_3$)

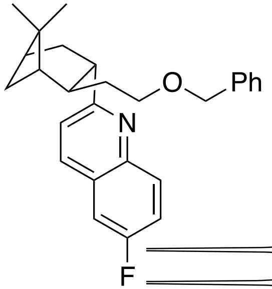

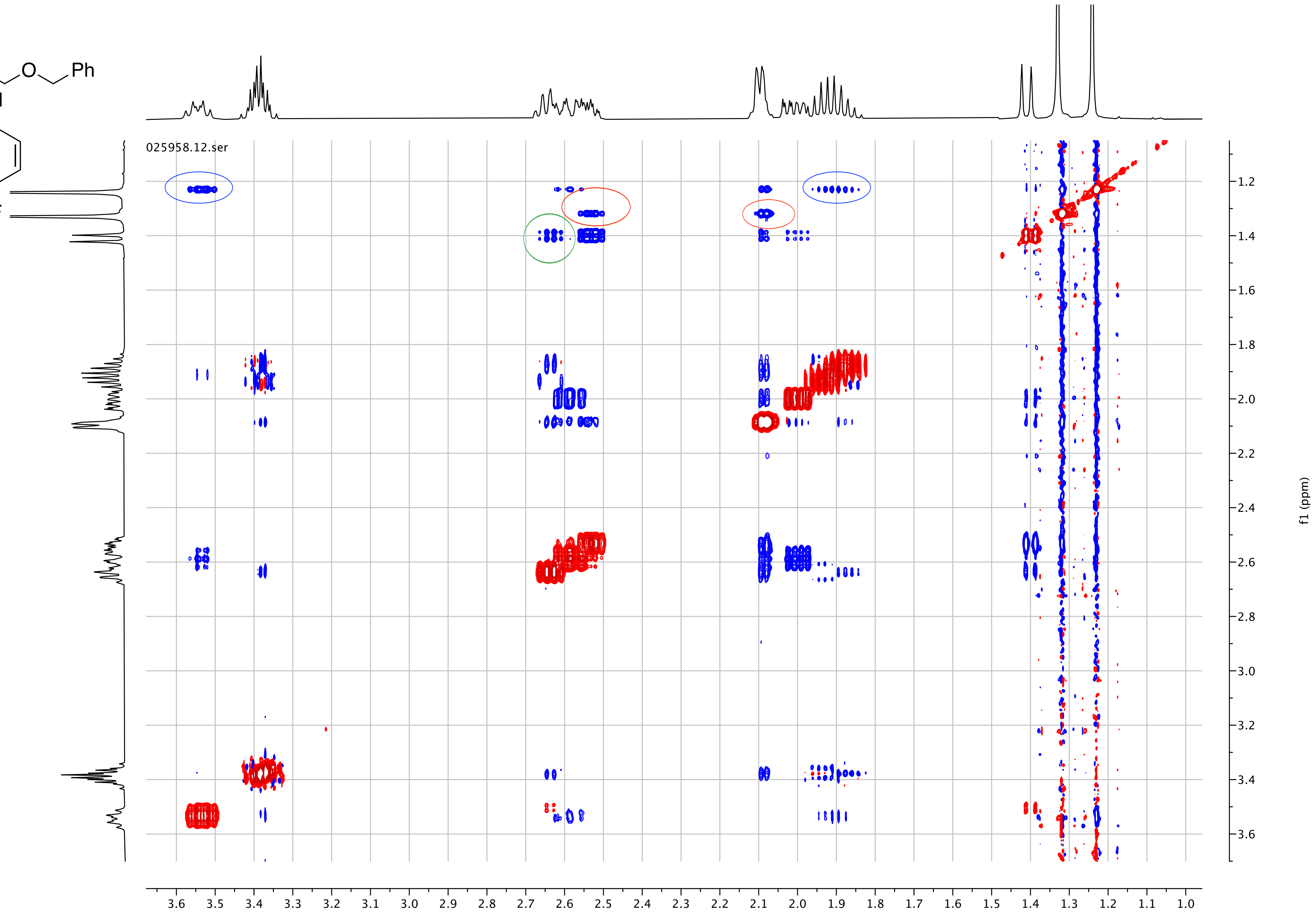

2-(2-(2-(Benzyloxy)ethyl)-6,6-dimethylbicyclo[3.1.1]heptan-3-yl)-6-fluoroquinoline

NOESY (400 MHz, $CDCl_3$)

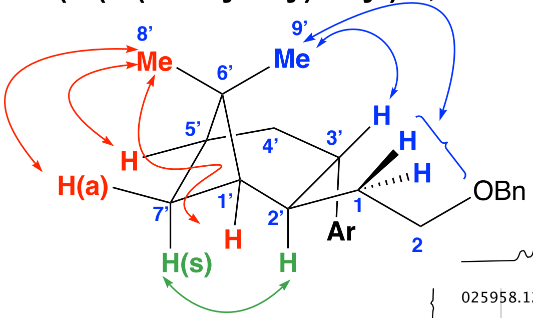

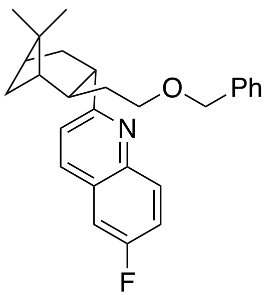

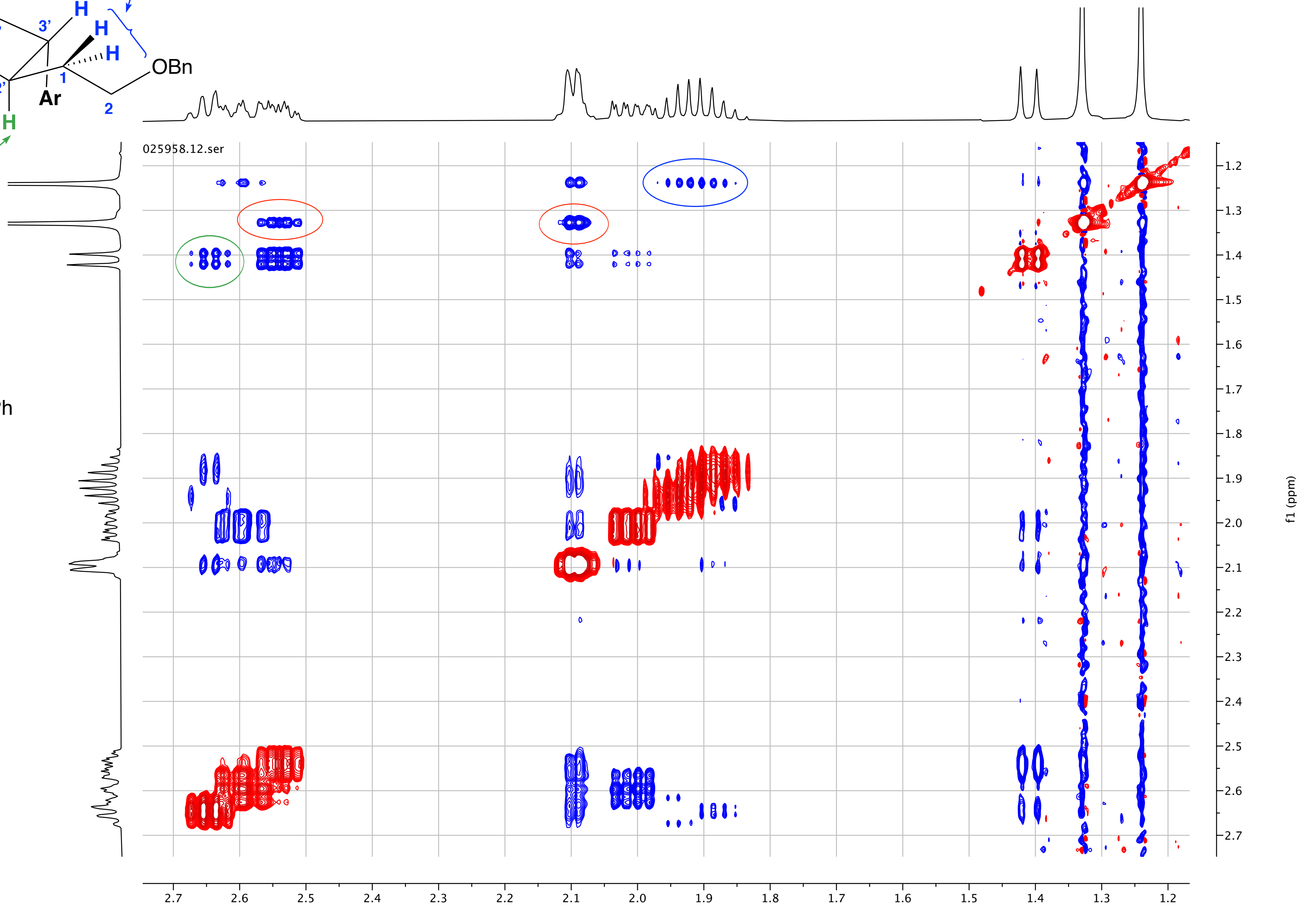

2-(2-(2-(Benzyloxy)ethyl)-6,6-dimethylbicyclo[3.1.1]heptan-3-yl)-6-fluoroquinoline (24)

^{19}F-NMR (376 MHz, $CDCl_3$)

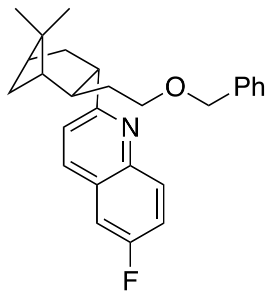

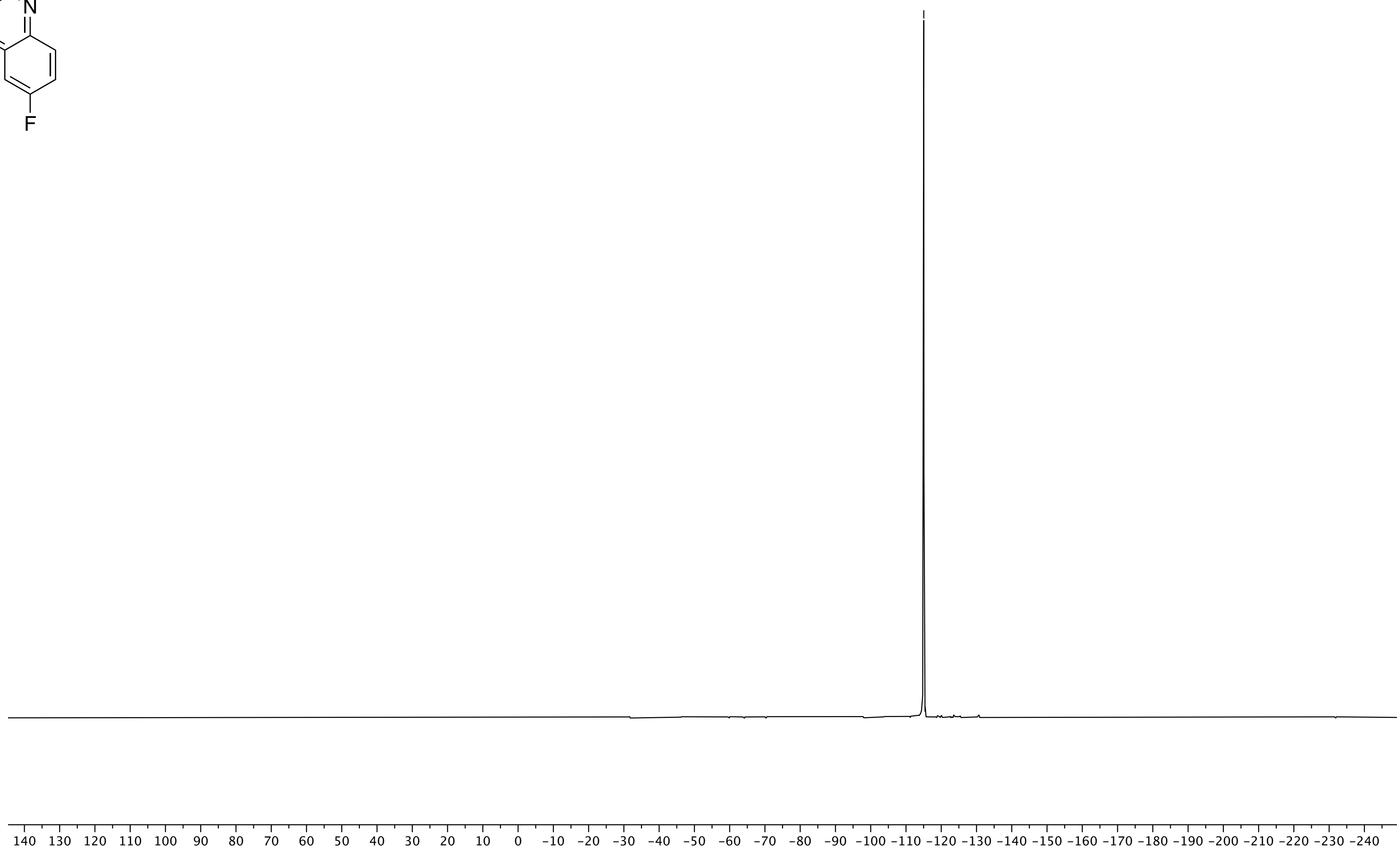

4-(2-(2-(Benzyloxy)ethyl)-6,6-dimethylbicyclo[3.1.1]heptan-3-yl)-6-fluoroquinoline (C_4-24, minor) ^{1}H-NMR (400 MHz, $CDCl_3$)

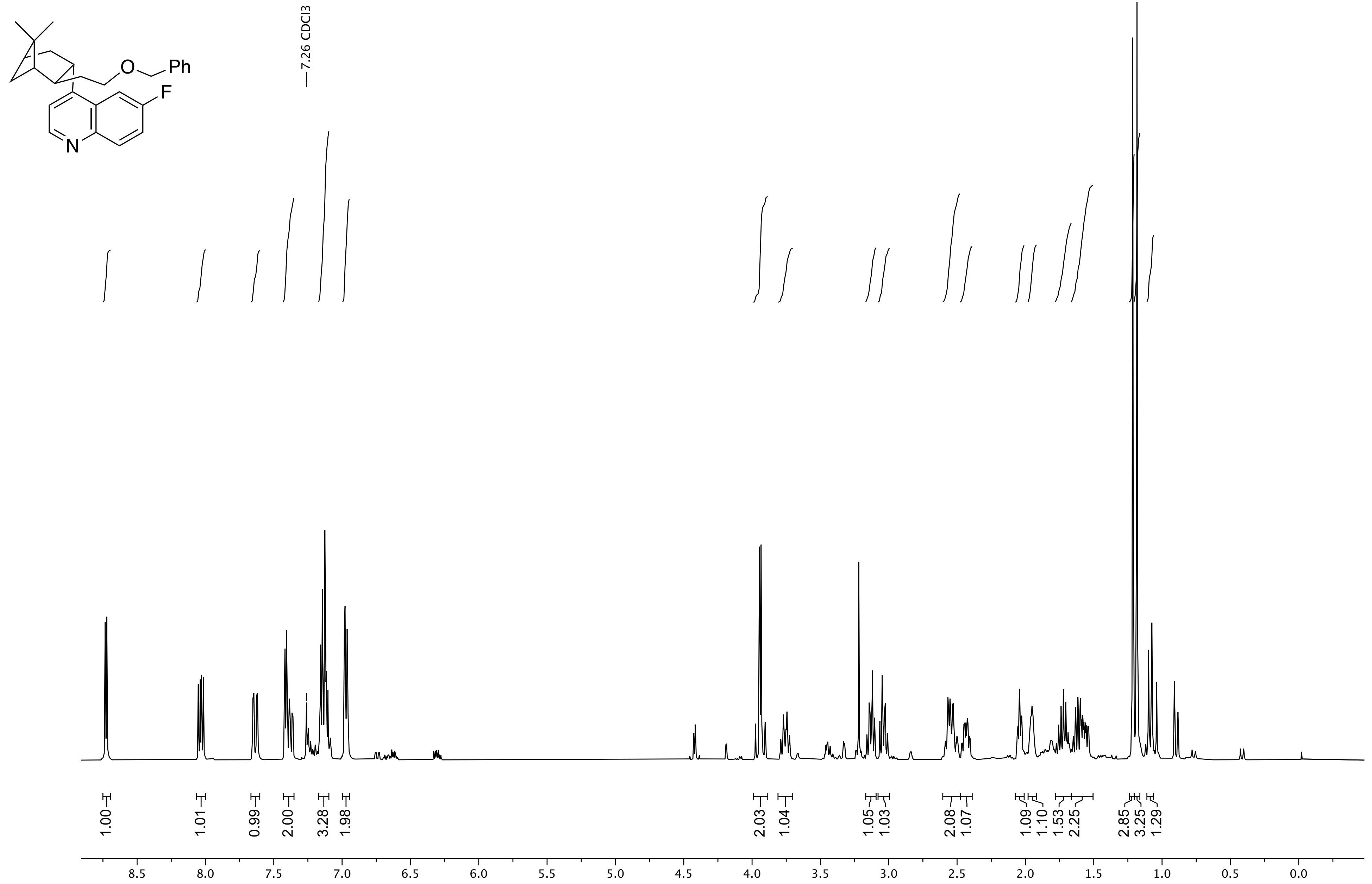

4-(2-(2-(Benzyloxy)ethyl)-6,6-dimethylbicyclo[3.1.1]heptan-3-yl)-6-fluoroquinoline (C_4-24, minor) ^{1}H-NMR (400 MHz, $CDCl_3$)

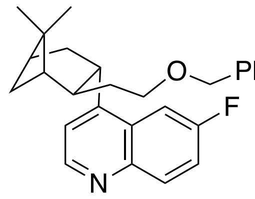

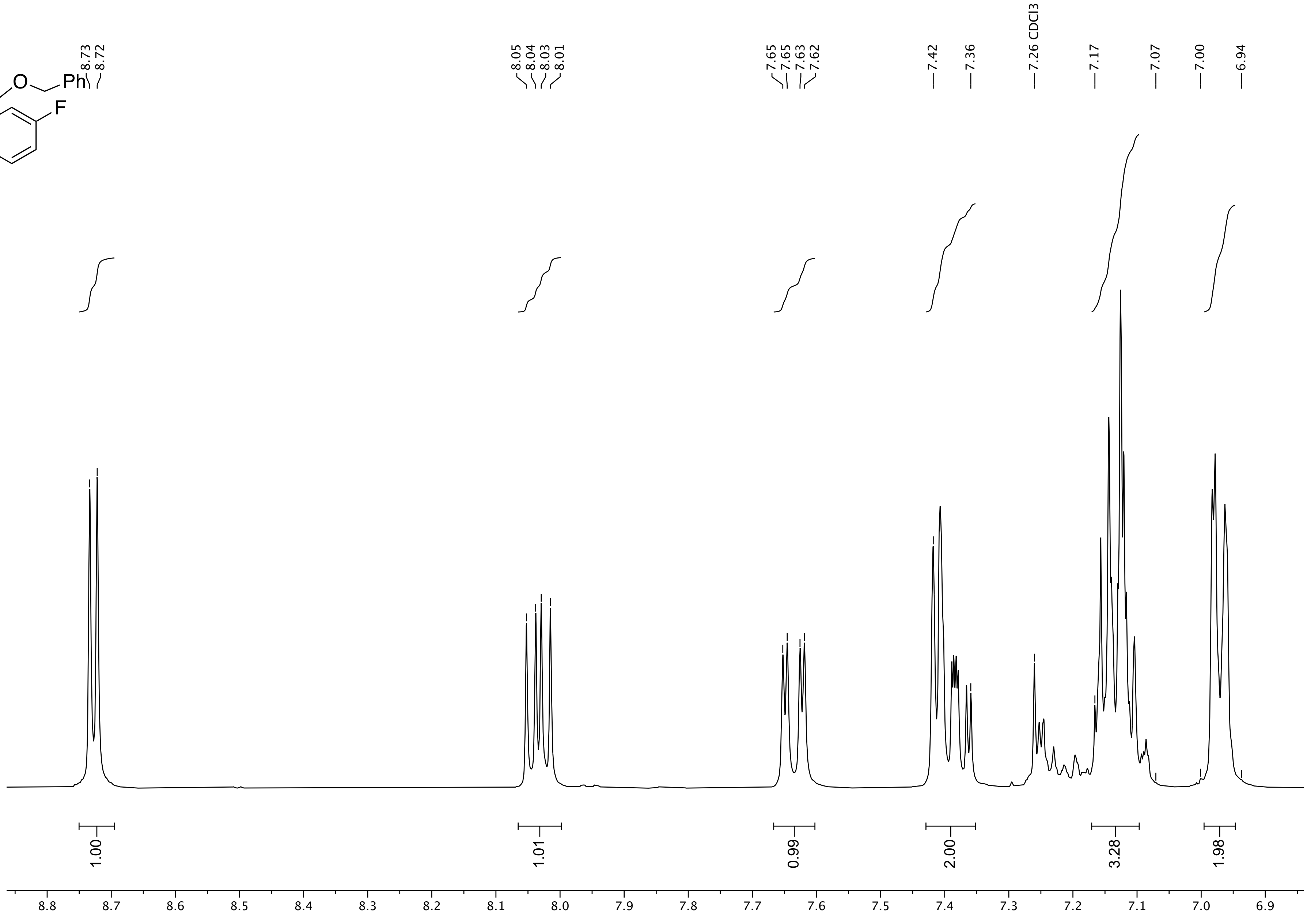

4-(2-(2-(Benzyloxy)ethyl)-6,6-dimethylbicyclo[3.1.1]heptan-3-yl)-6-fluoroquinoline (C_4-24, minor) ^{1}H-NMR (400 MHz, $CDCl_3$)

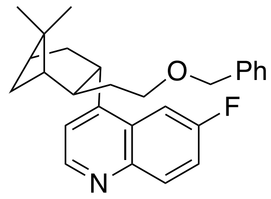

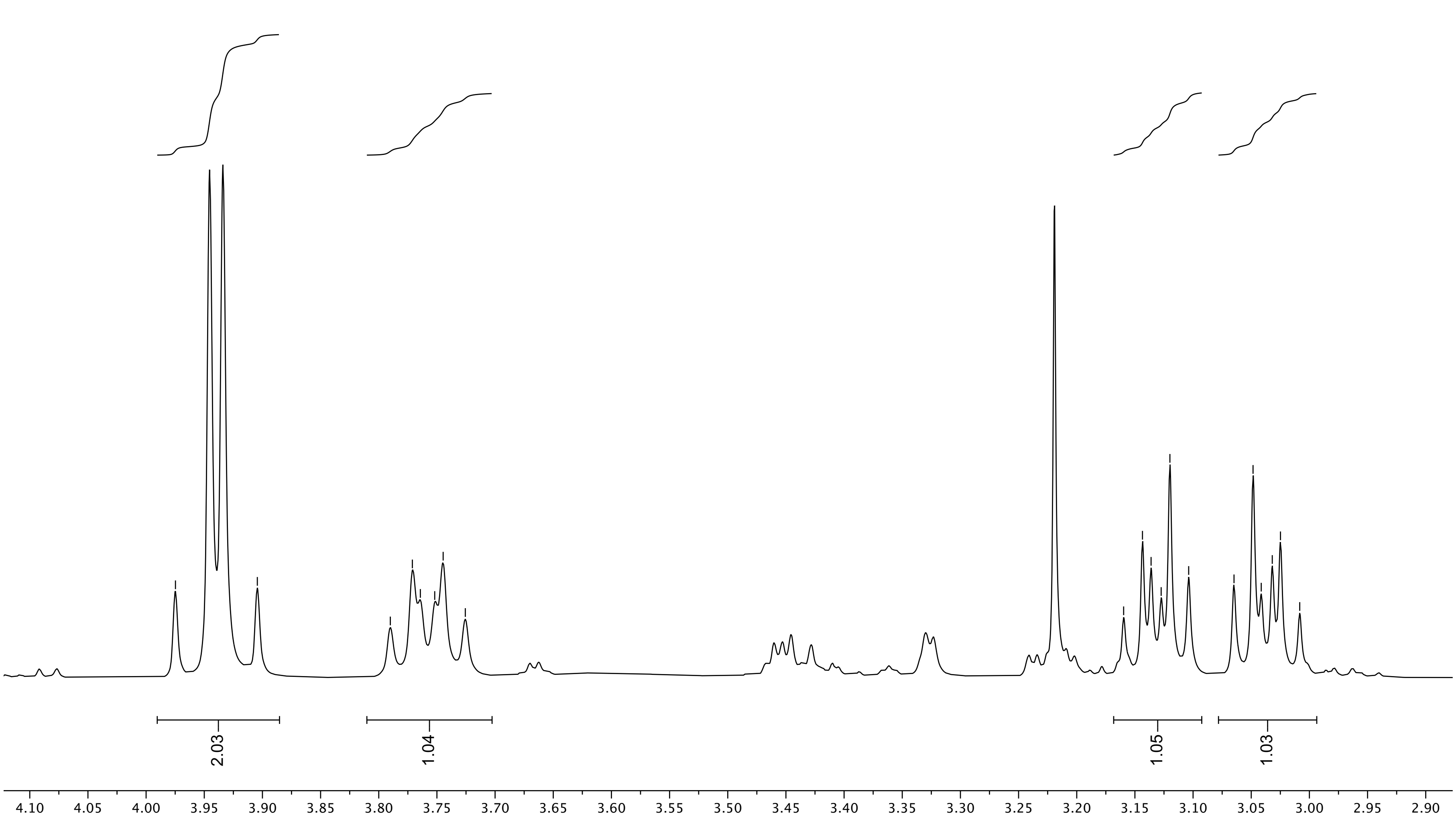

4-(2-(2-(Benzyloxy)ethyl)-6,6-dimethylbicyclo[3.1.1]heptan-3-yl)-6-fluoroquinoline (C_4-24, minor) ^{1}H-NMR (400 MHz, $CDCl_3$)

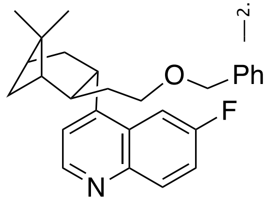

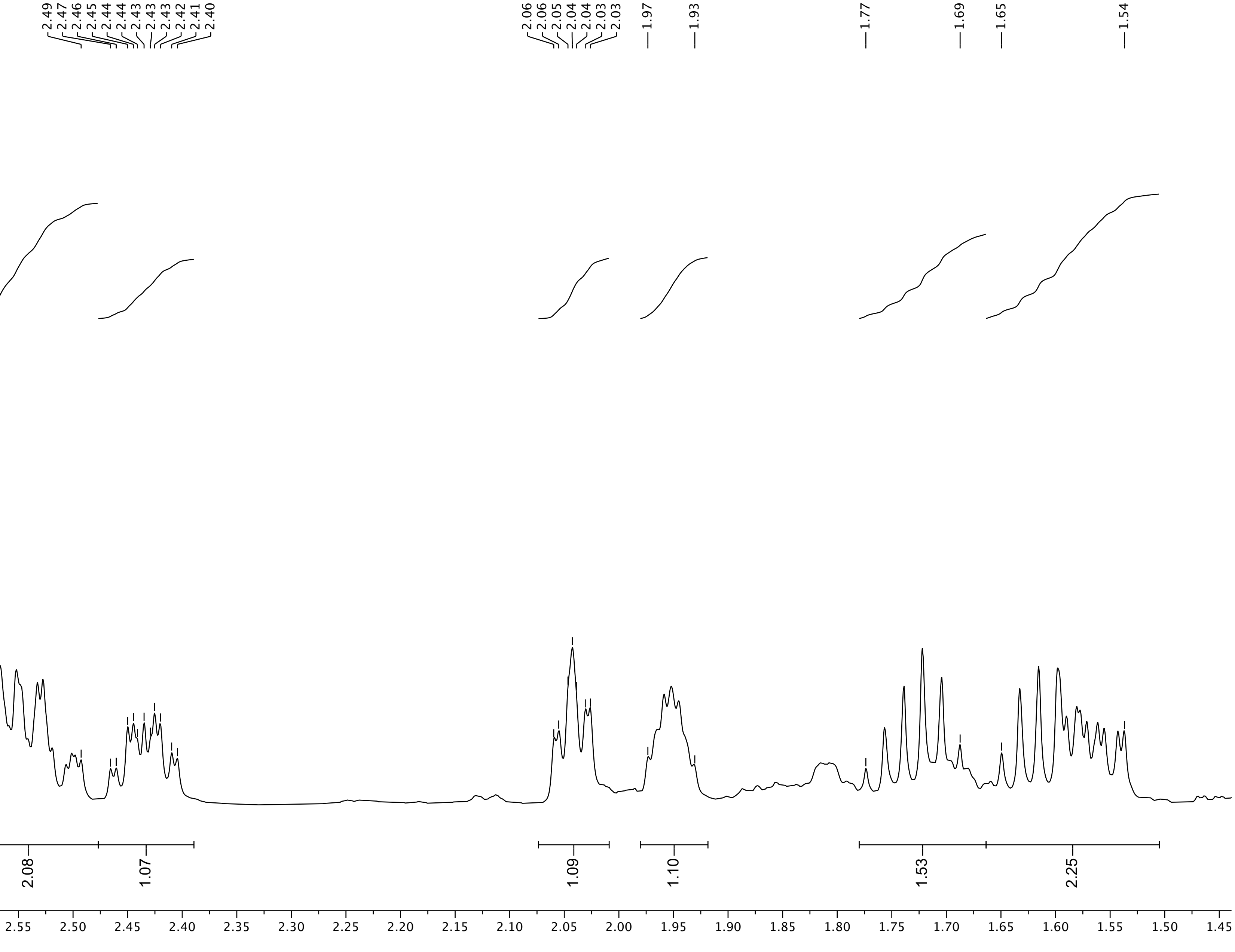

4-(2-(2-(Benzyloxy)ethyl)-6,6-dimethylbicyclo[3.1.1]heptan-3-yl)-6-fluoroquinoline (C_4-24, minor) ^{13}C-NMR (101 MHz, $CDCl_3$)

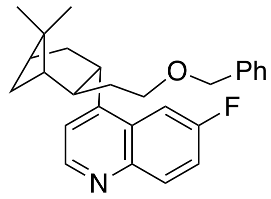

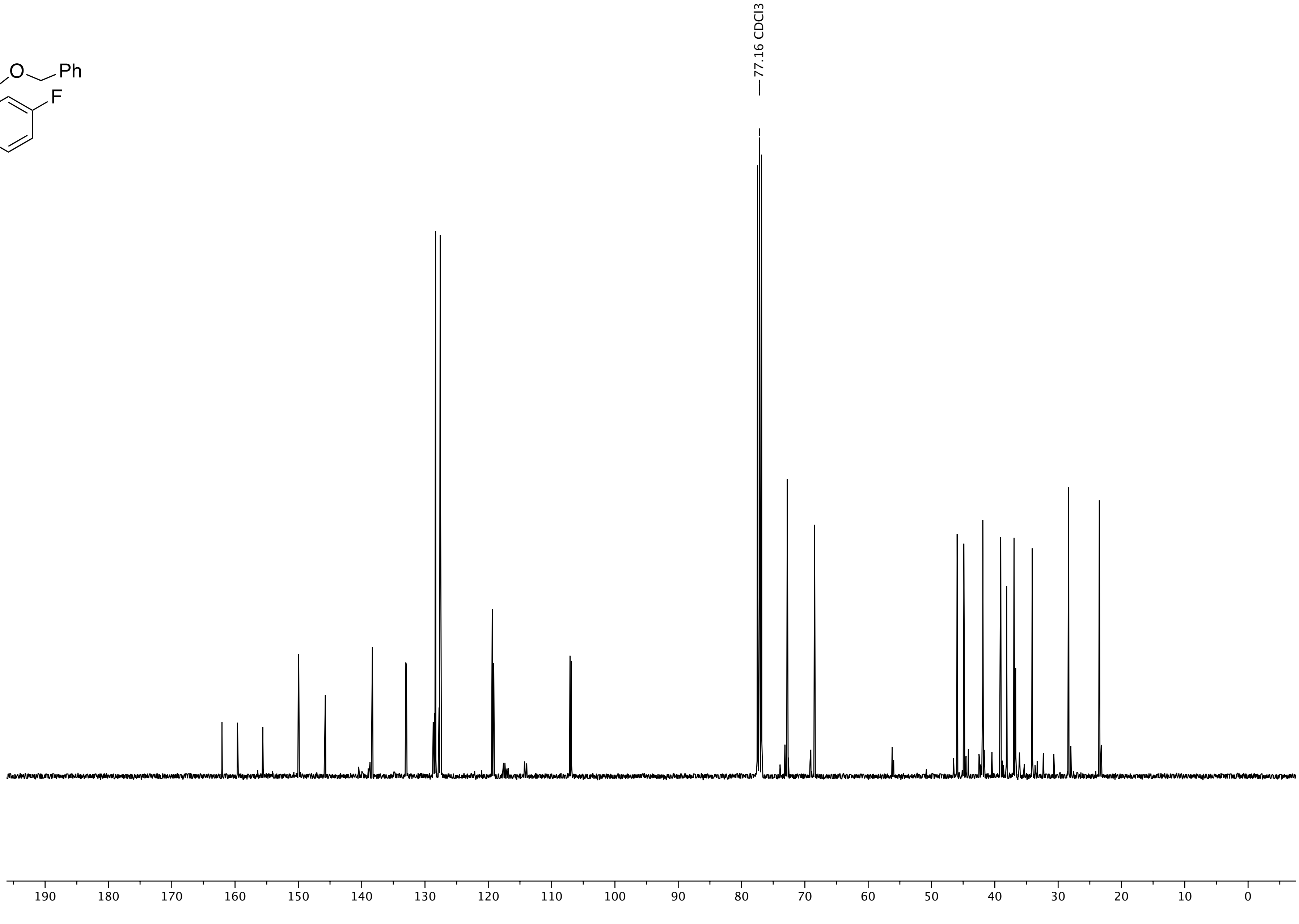

4-(2-(2-(Benzyloxy)ethyl)-6,6-dimethylbicyclo[3.1.1]heptan-3-yl)-6-fluoroquinoline (C_4-24, minor) ^{13}C-NMR (101 MHz, $CDCl_3$)

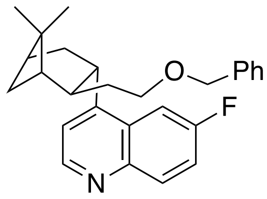

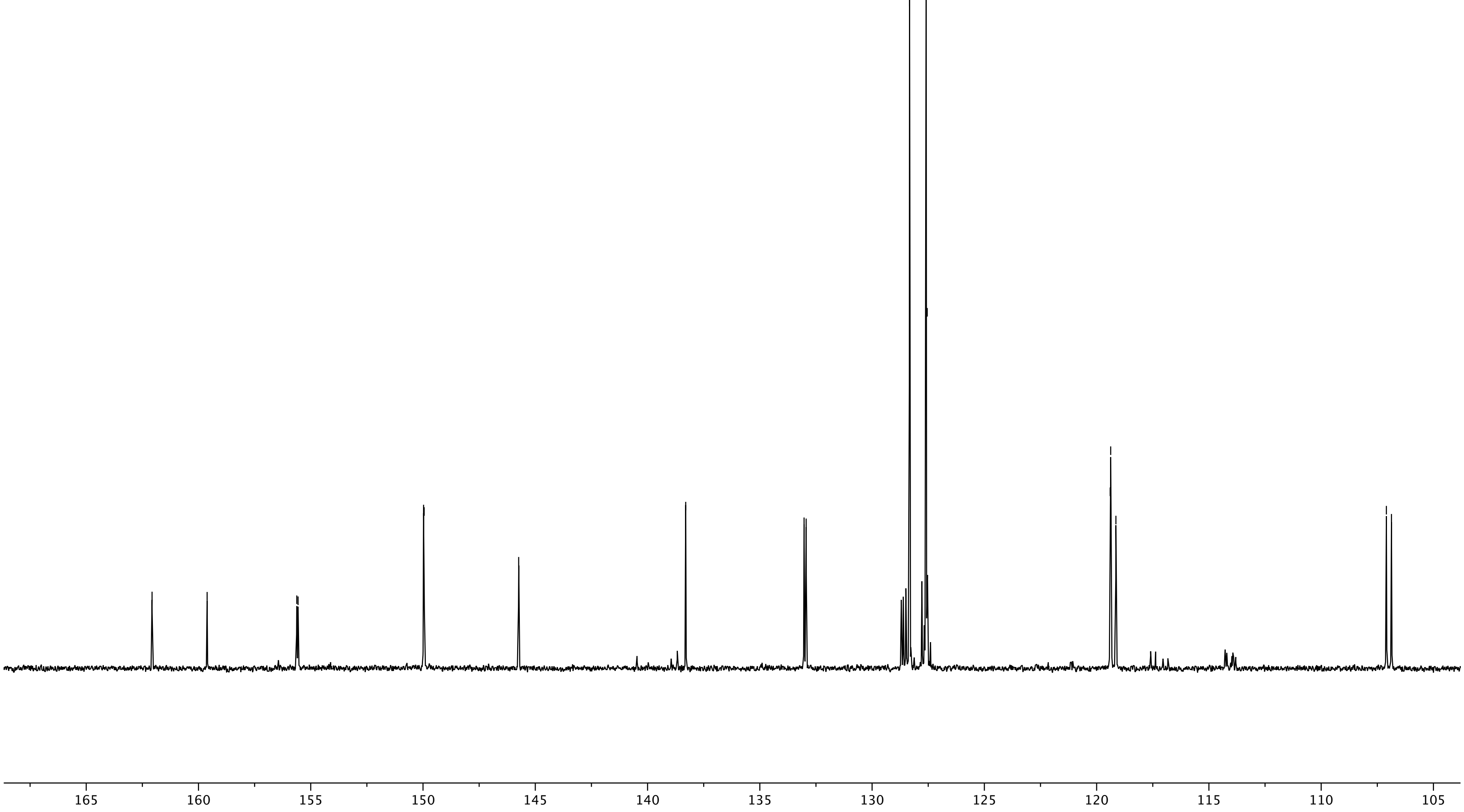

4-(2-(2-(Benzyloxy)ethyl)-6,6-dimethylbicyclo[3.1.1]heptan-3-yl)-6-fluoroquinoline (C_4-24, minor) ^{13}C-NMR (101 MHz, $CDCl_3$)

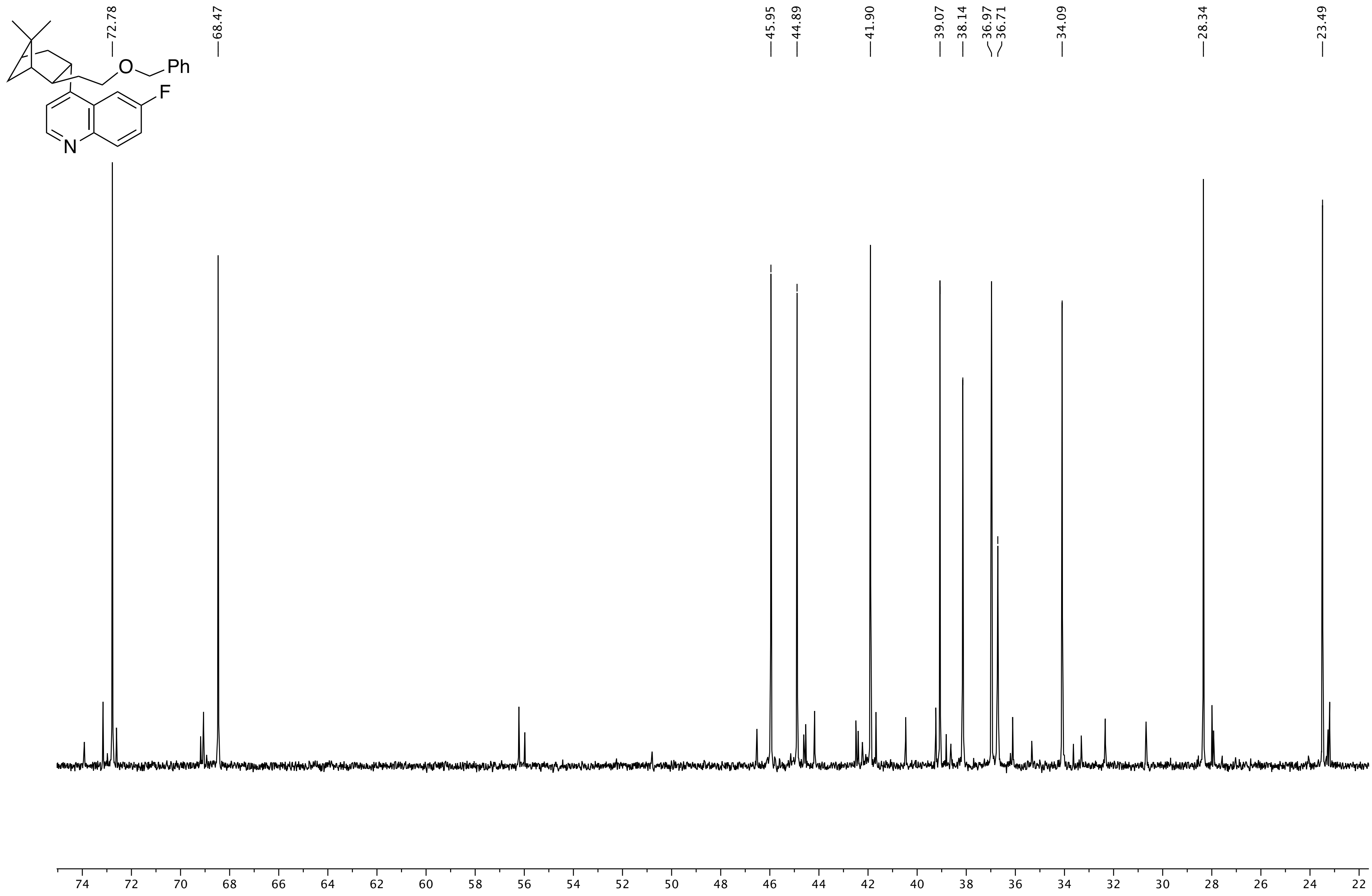

4-(2-(2-(Benzyloxy)ethyl)-6,6-dimethylbicyclo[3.1.1]heptan-3-yl)-6-fluoroquinoline (C_4-24, minor) Dept-135 (101 MHz, $CDCl_3$)

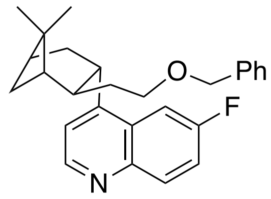

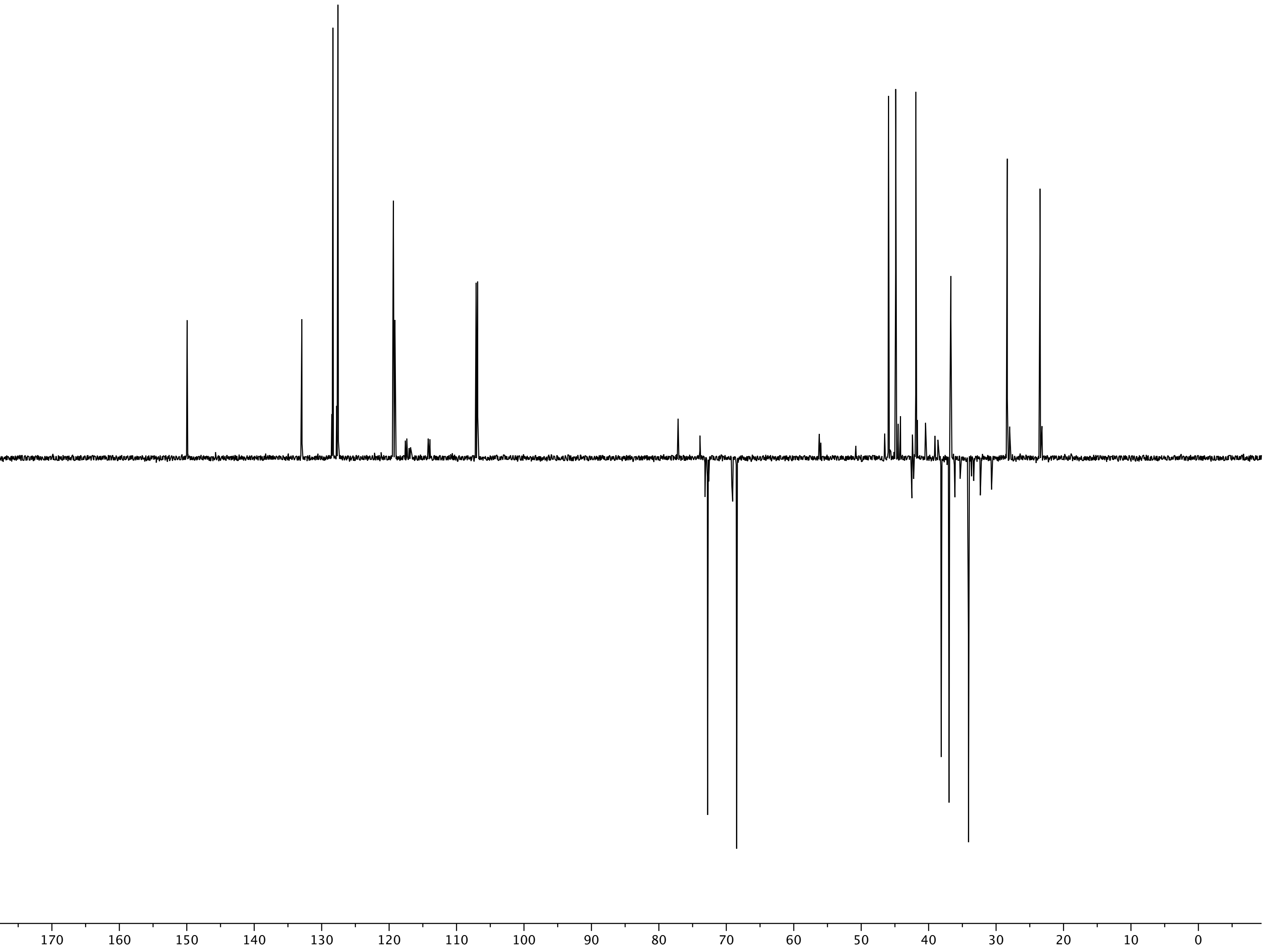

4-(2-(2-(Benzyloxy)ethyl)-6,6-dimethylbicyclo[3.1.1]heptan-3-yl)-6-fluoroquinoline (C_4-24, minor) Dept-135 (101 MHz, $CDCl_3$)

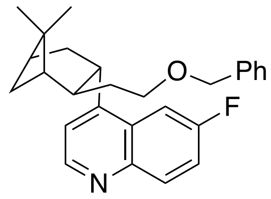

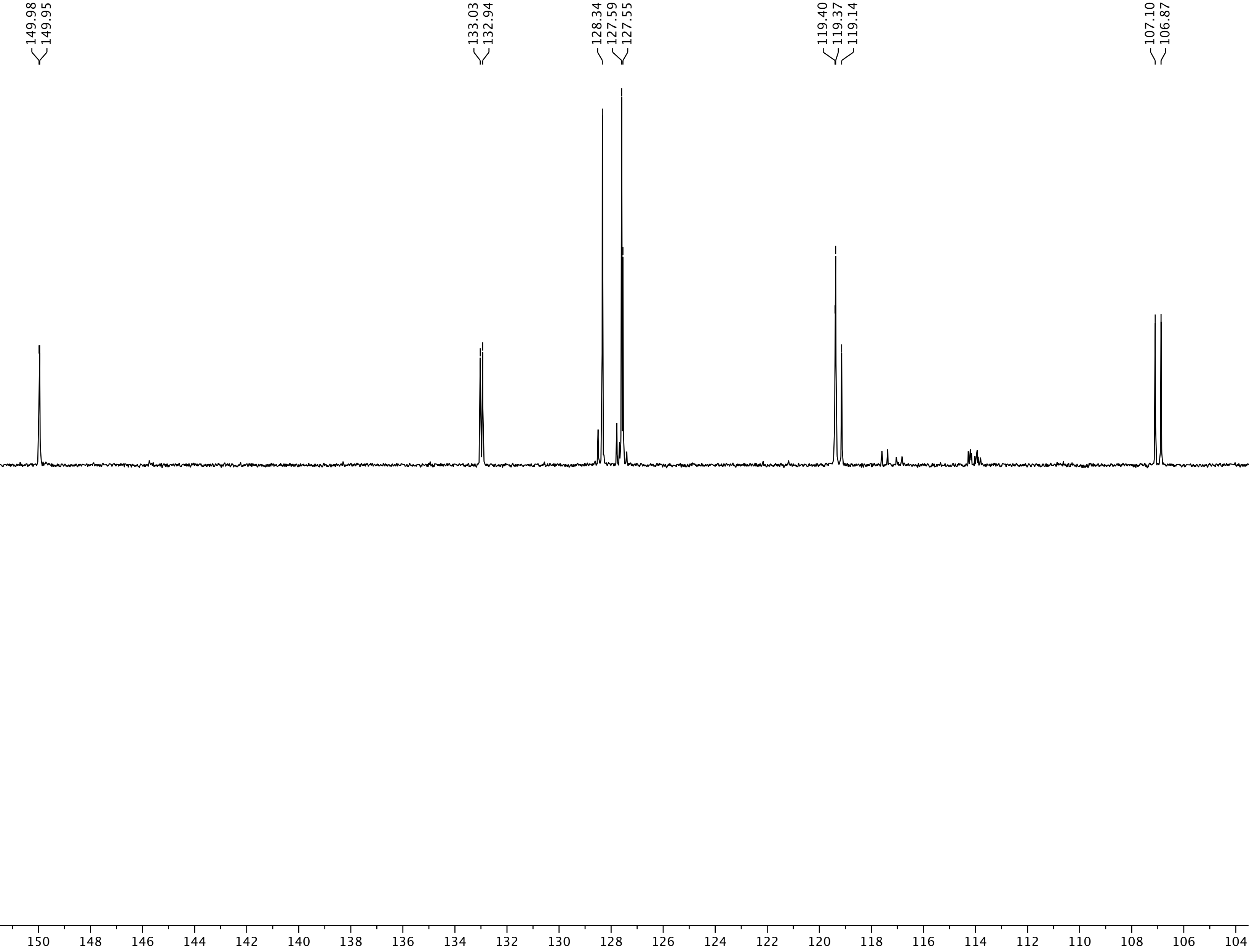

4-(2-(2-(Benzyloxy)ethyl)-6,6-dimethylbicyclo[3.1.1]heptan-3-yl)-6-fluoroquinoline (C_4-24, minor) Dept-135 (101 MHz, $CDCl_3$)

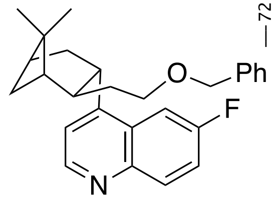

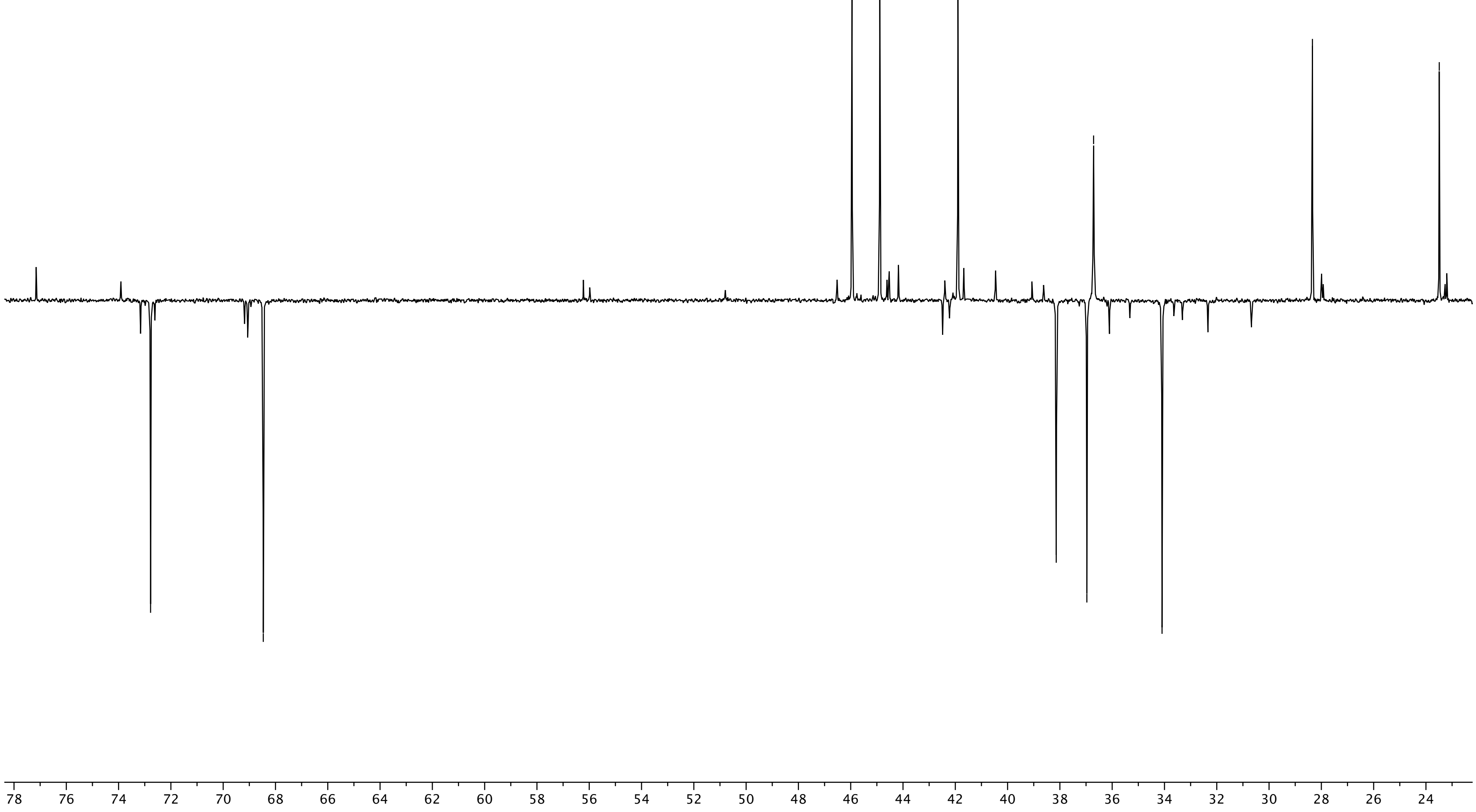

4-(2-(2-(Benzyloxy)ethyl)-6,6-dimethylbicyclo[3.1.1]heptan-3-yl)-6-fluoroquinoline (C_4-24, minor) ^1H-^1H COSY (400 MHz, $CDCl_3$)

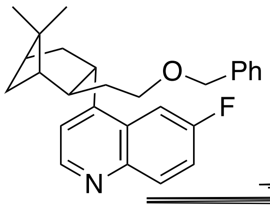

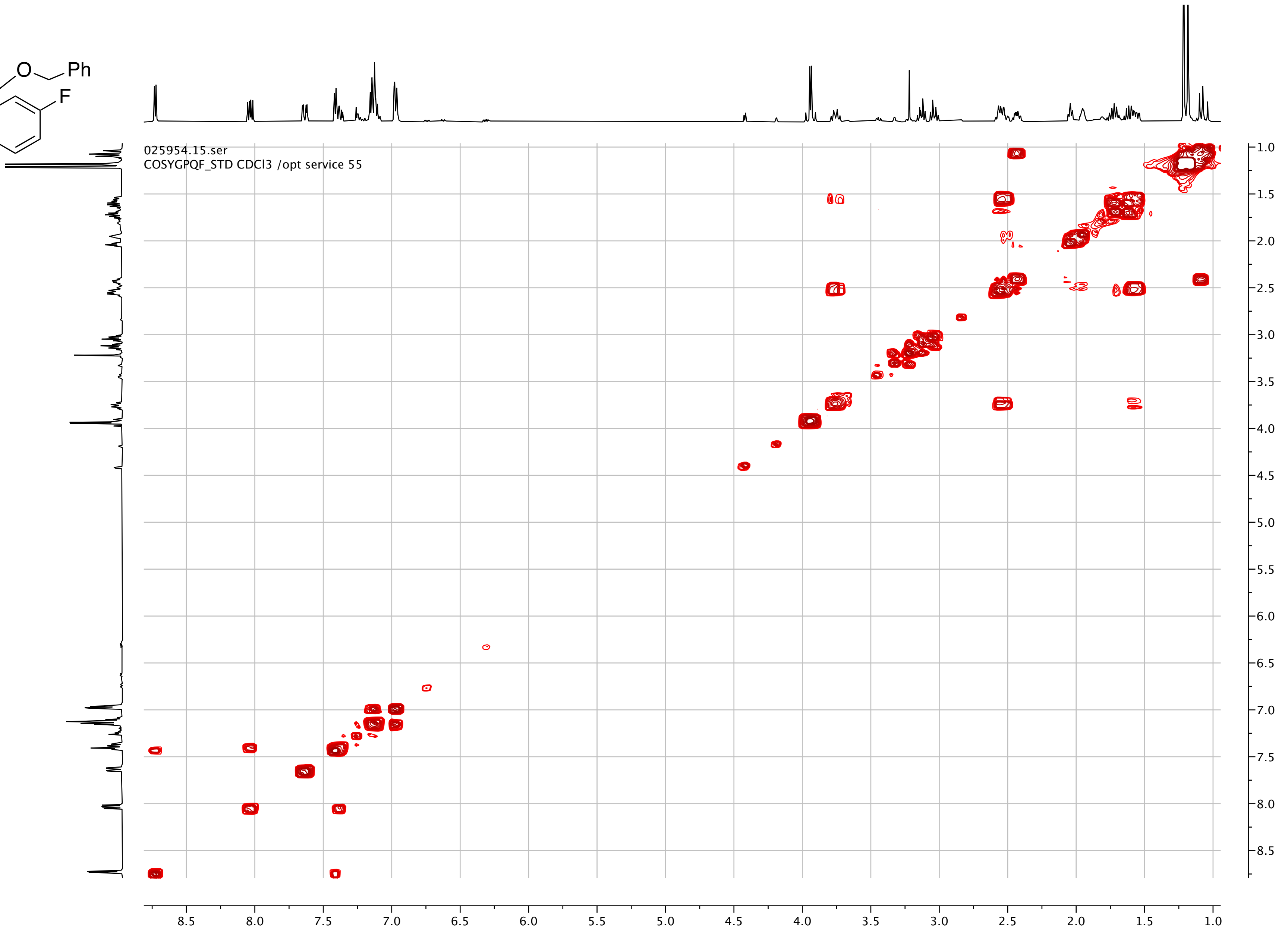

4-(2-(2-(Benzyloxy)ethyl)-6,6-dimethylbicyclo[3.1.1]heptan-3-yl)-6-fluoroquinoline (C_4-24, minor) ^{1}H-^{1}H COSY (400 MHz, $CDCl_3$)

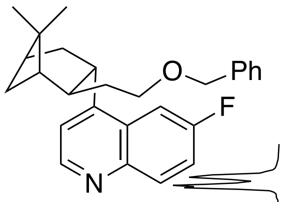

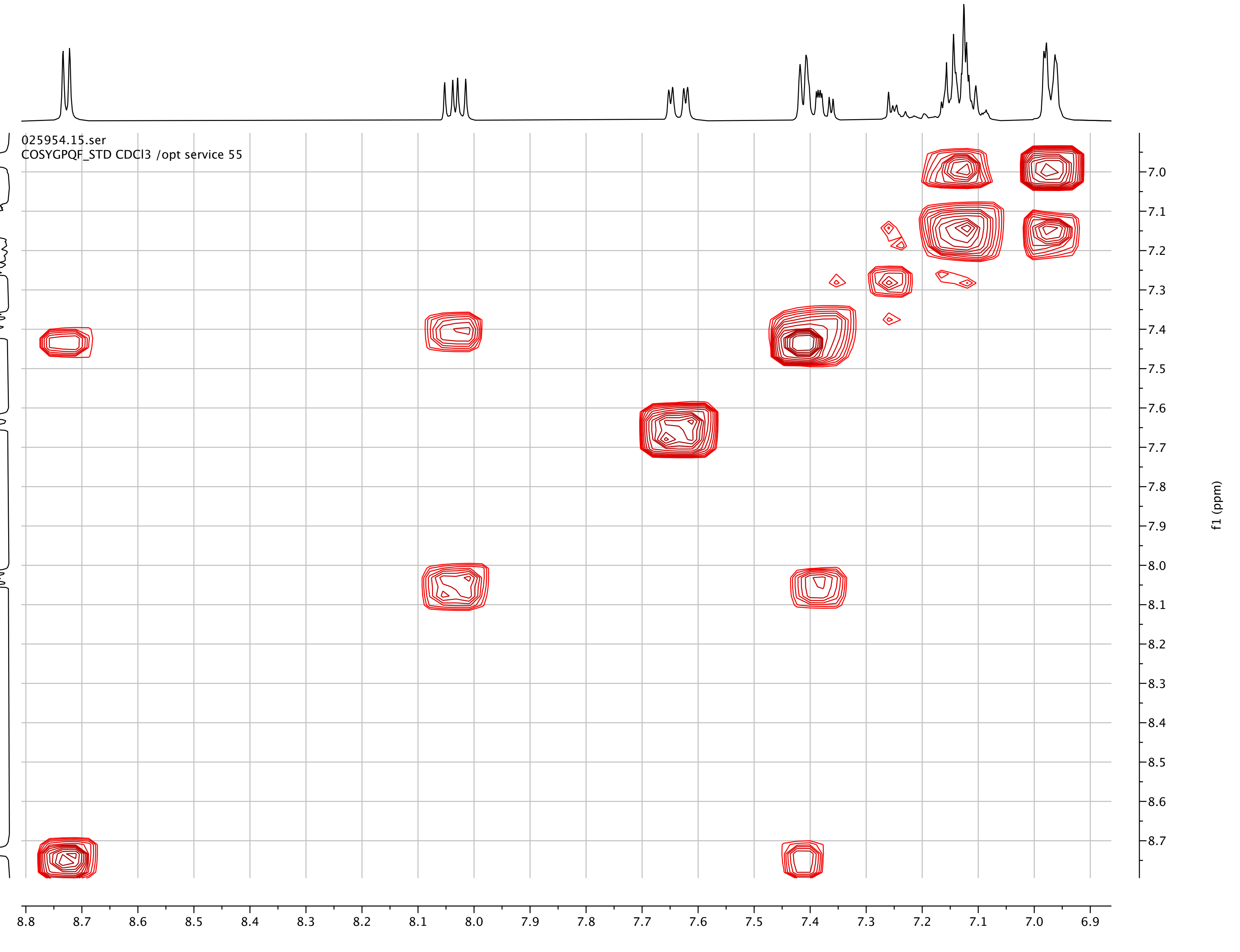

4-(2-(2-(Benzyloxy)ethyl)-6,6-dimethylbicyclo[3.1.1]heptan-3-yl)-6-fluoroquinoline (C_4-24, minor) ^1H-^1H COSY (400 MHz, $CDCl_3$)

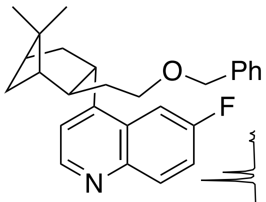

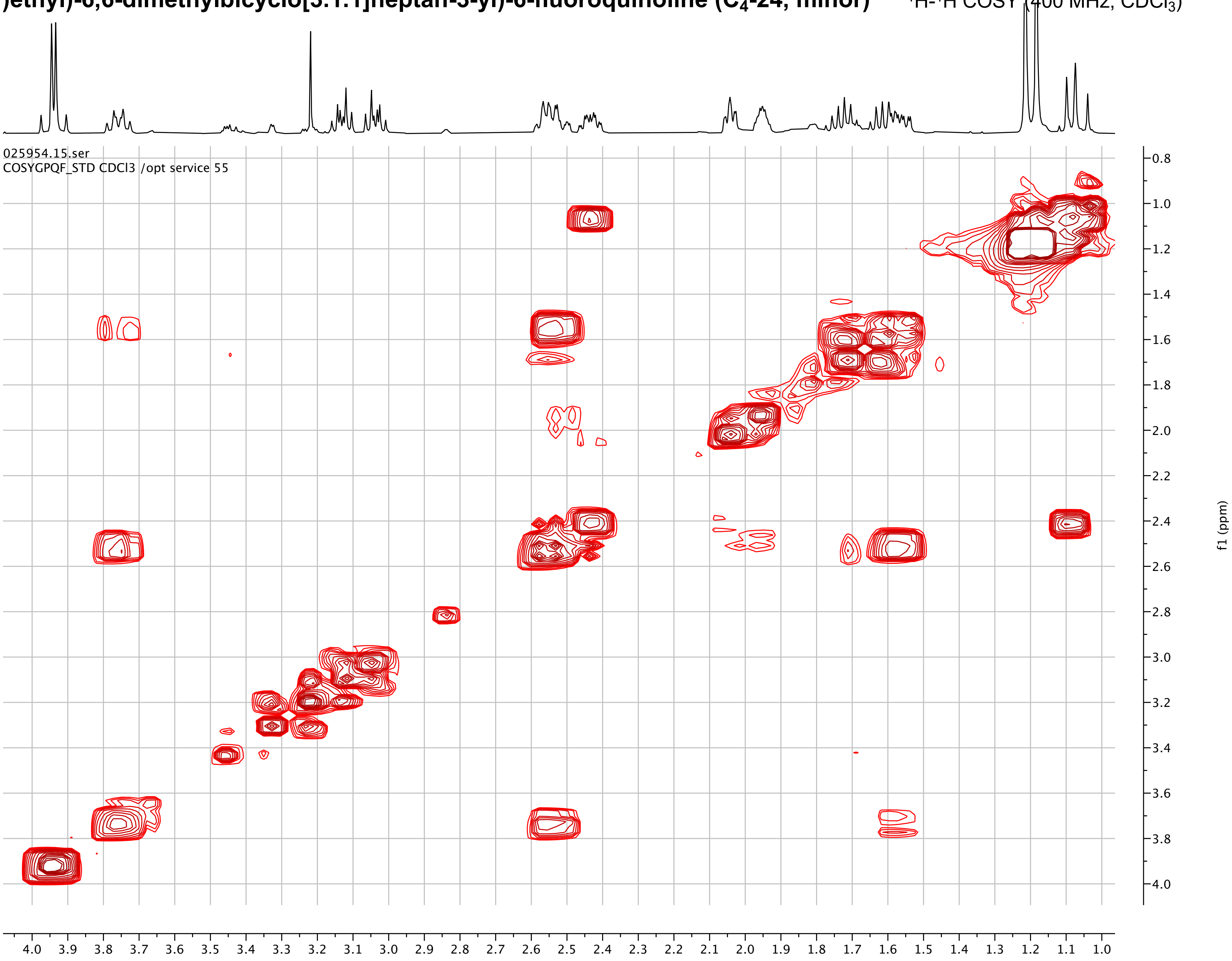

4-(2-(2-(Benzyloxy)ethyl)-6,6-dimethylbicyclo[3.1.1]heptan-3-yl)-6-fluoroquinoline (C_4-24, minor) HSQC (400 MHz, $CDCl_3$)

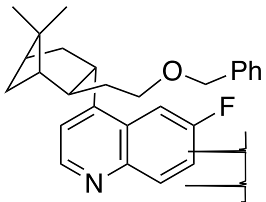

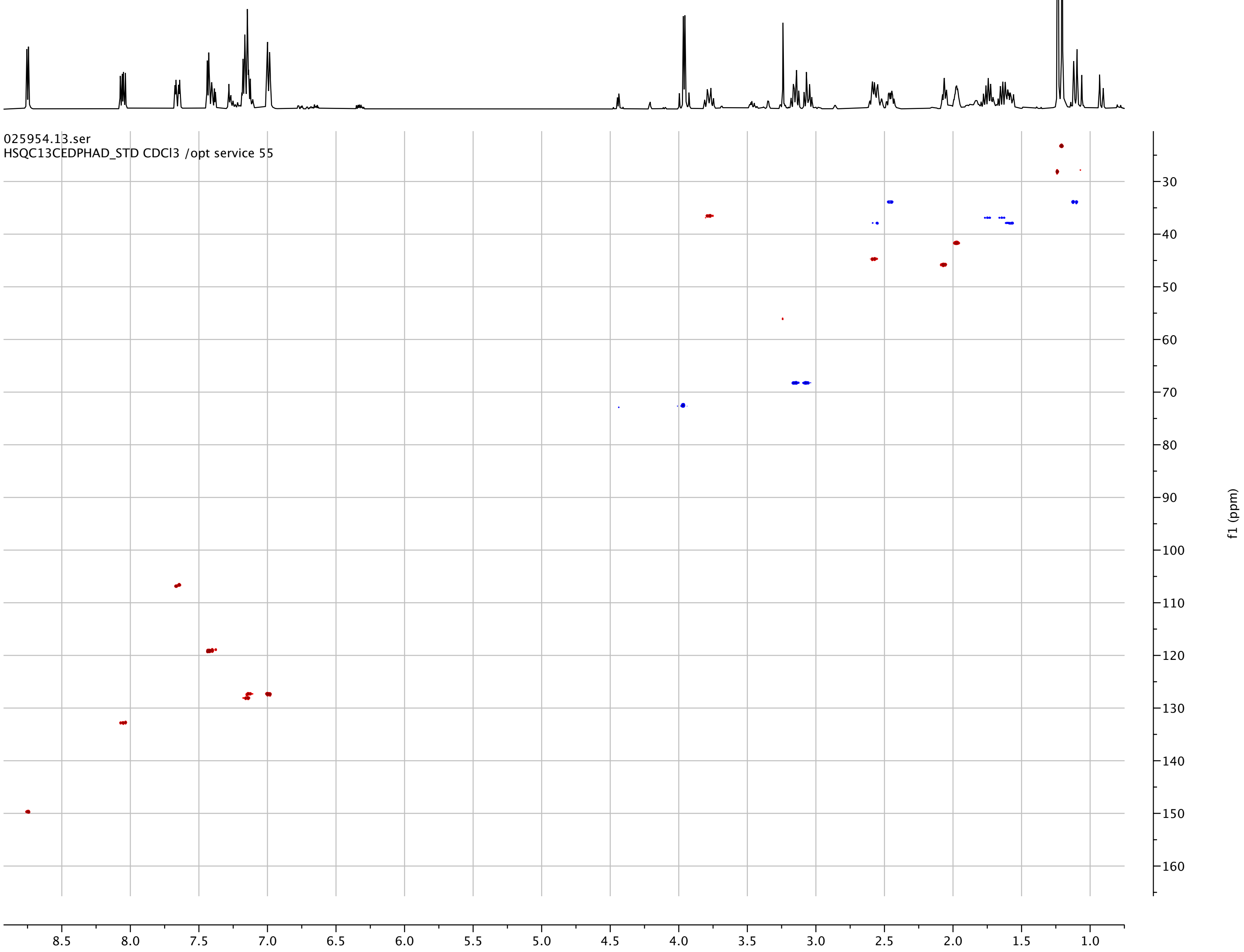

4-(2-(2-(Benzyloxy)ethyl)-6,6-dimethylbicyclo[3.1.1]heptan-3-yl)-6-fluoroquinoline (C_4-24, minor) HSQC (400 MHz, $CDCl_3$)

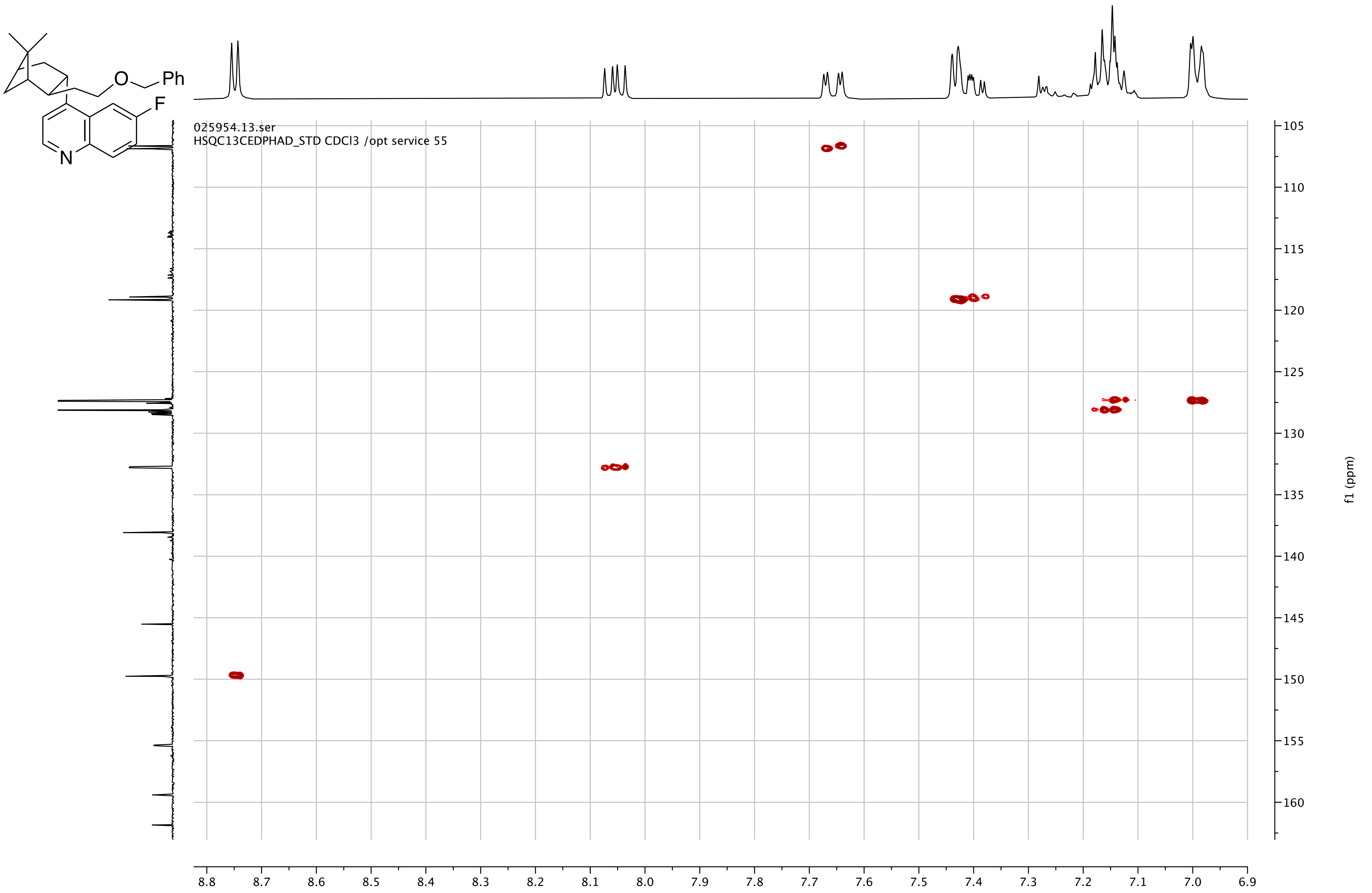

4-(2-(2-(Benzyloxy)ethyl)-6,6-dimethylbicyclo[3.1.1]heptan-3-yl)-6-fluoroquinoline (C_4-24, minor) HSQC (400 MHz, $CDCl_3$)

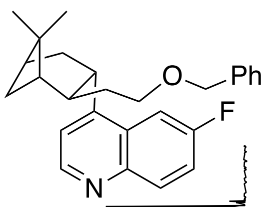

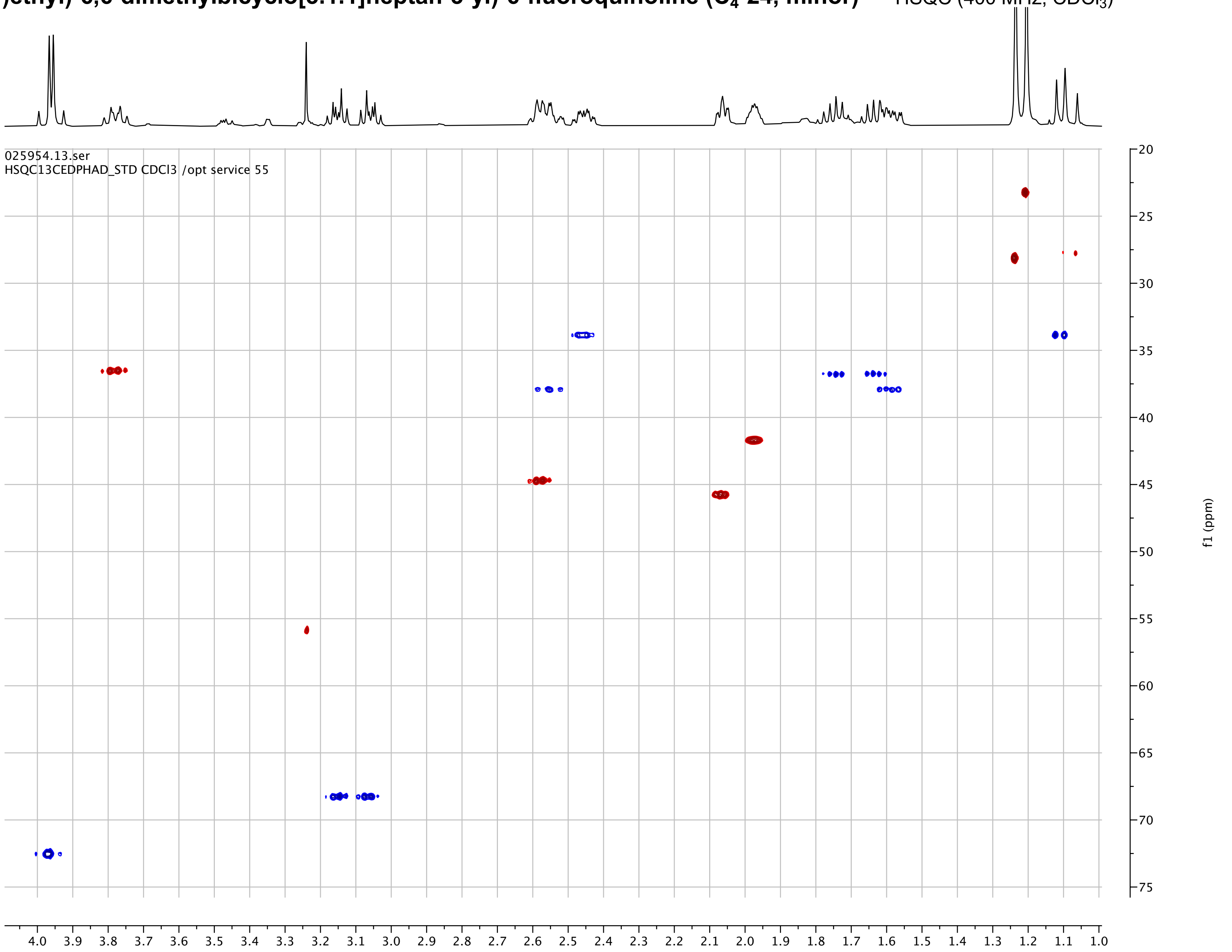

4-(2-(2-(Benzyloxy)ethyl)-6,6-dimethylbicyclo[3.1.1]heptan-3-yl)-6-fluoroquinoline (C_4-24, minor) HMBC (400 MHz, $CDCl_3$)

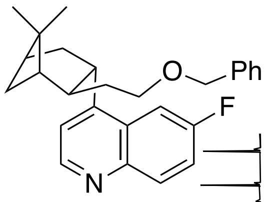

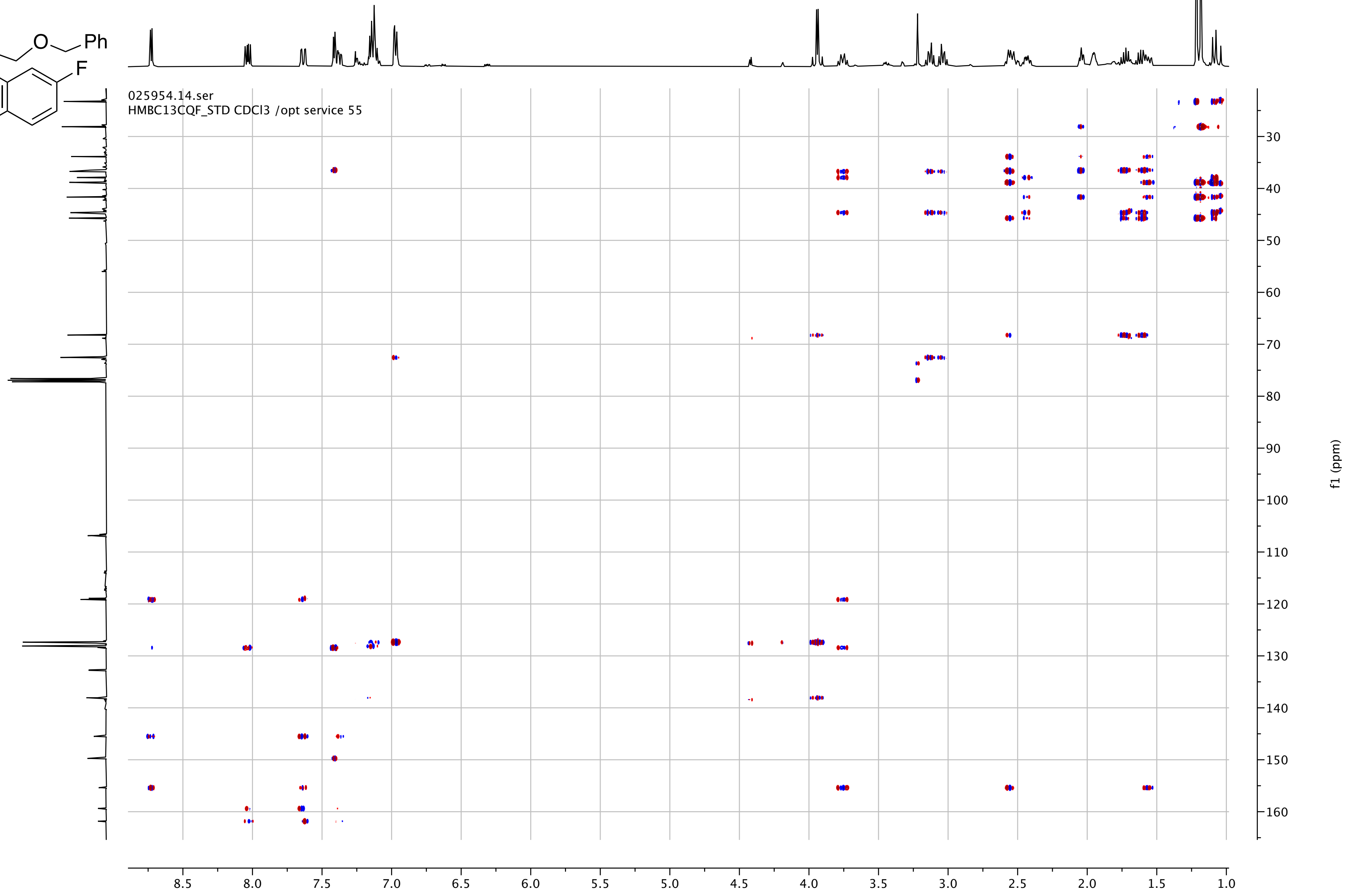

4-(2-(2-(Benzyloxy)ethyl)-6,6-dimethylbicyclo[3.1.1]heptan-3-yl)-6-fluoroquinoline (C_4-24, minor) HMBC (400 MHz, $CDCl_3$)

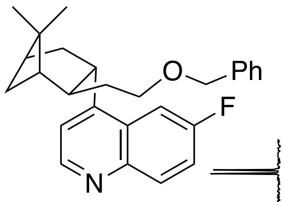

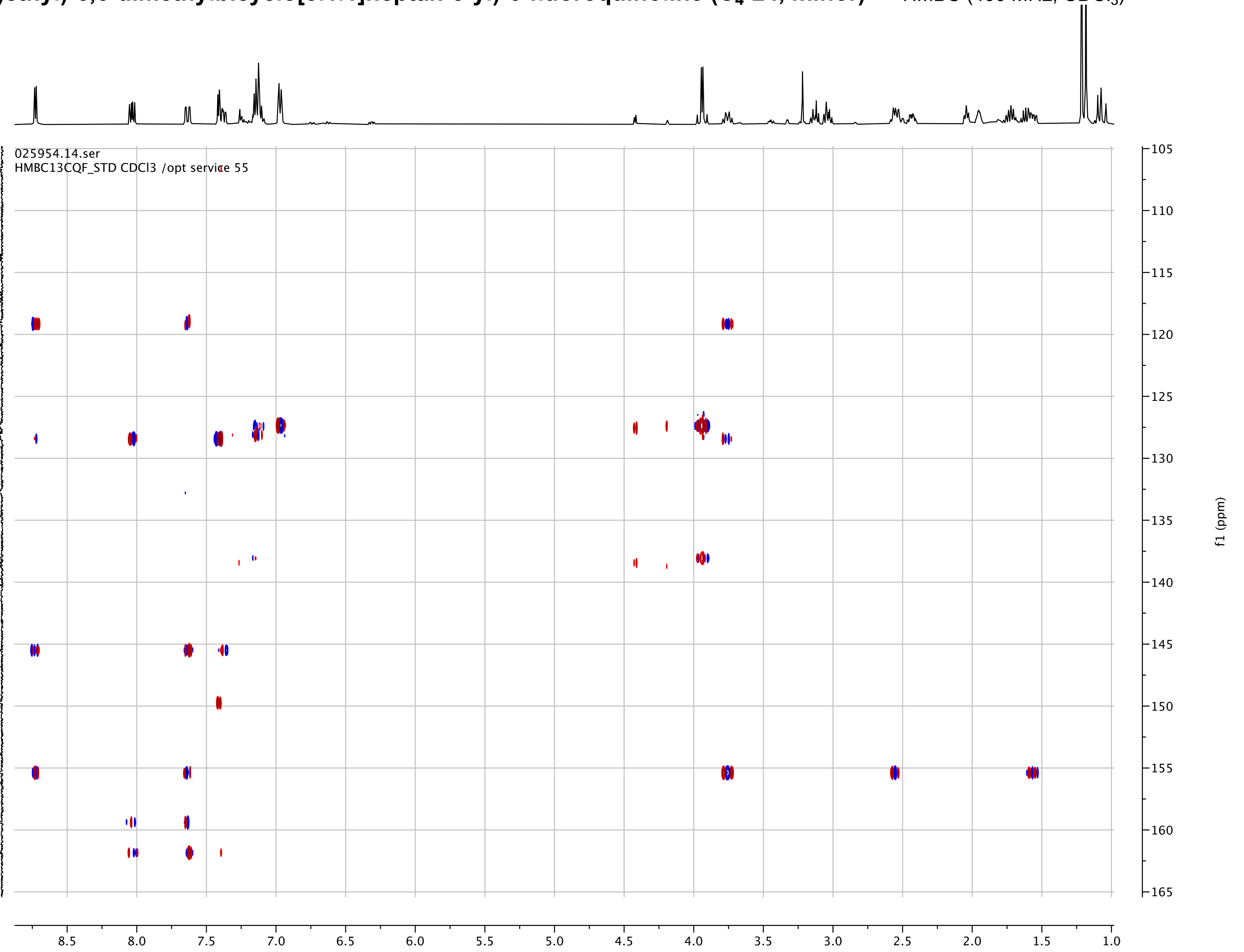

4-(2-(2-(Benzyloxy)ethyl)-6,6-dimethylbicyclo[3.1.1]heptan-3-yl)-6-fluoroquinoline (C_4-24, minor) HMBC (400 MHz, $CDCl_3$)

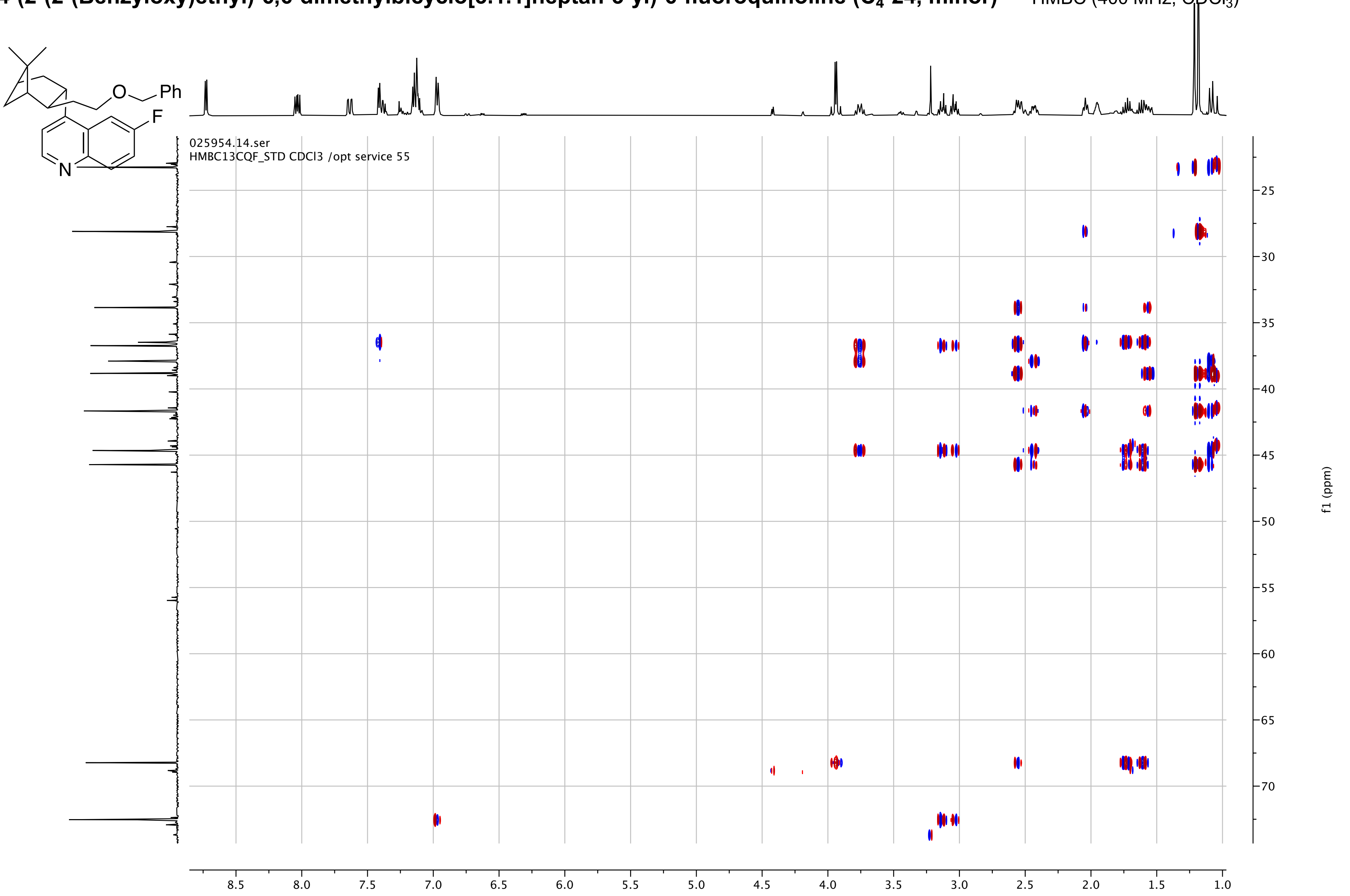

4-(2-(2-(Benzyloxy)ethyl)-6,6-dimethylbicyclo[3.1.1]heptan-3-yl)-6-fluoroquinoline (C_4-24, minor) ^{19}F (376 MHz, $CDCl_3$)

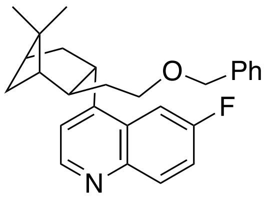

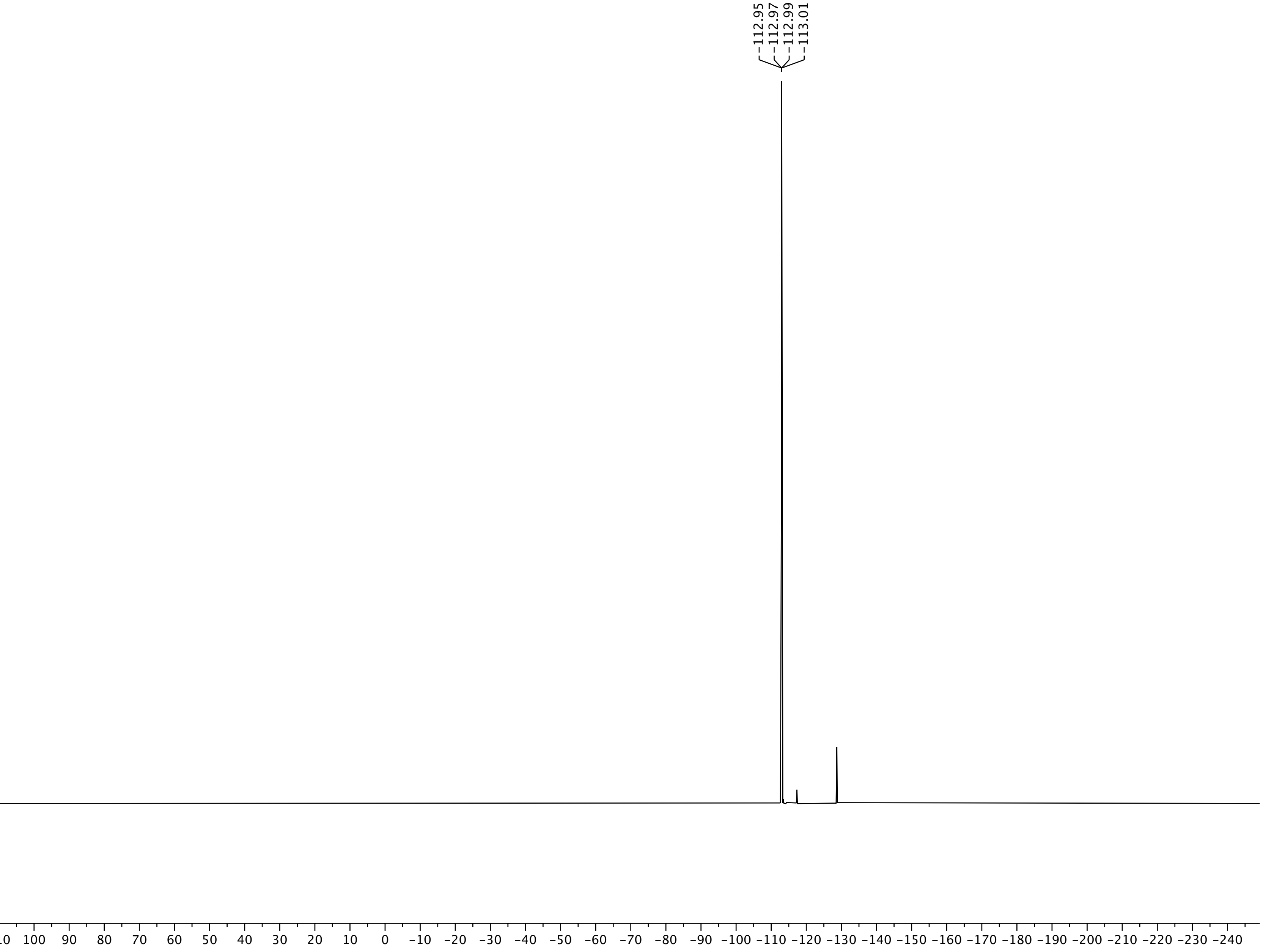

2-(2-(2-(Benzyloxy)ethyl)-6,6-dimethylbicyclo[3.1.1]heptan-3-yl)-6-chloropyridine (25)

^{1}H-NMR (400 MHz, $CDCl_3$)

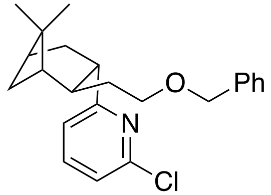

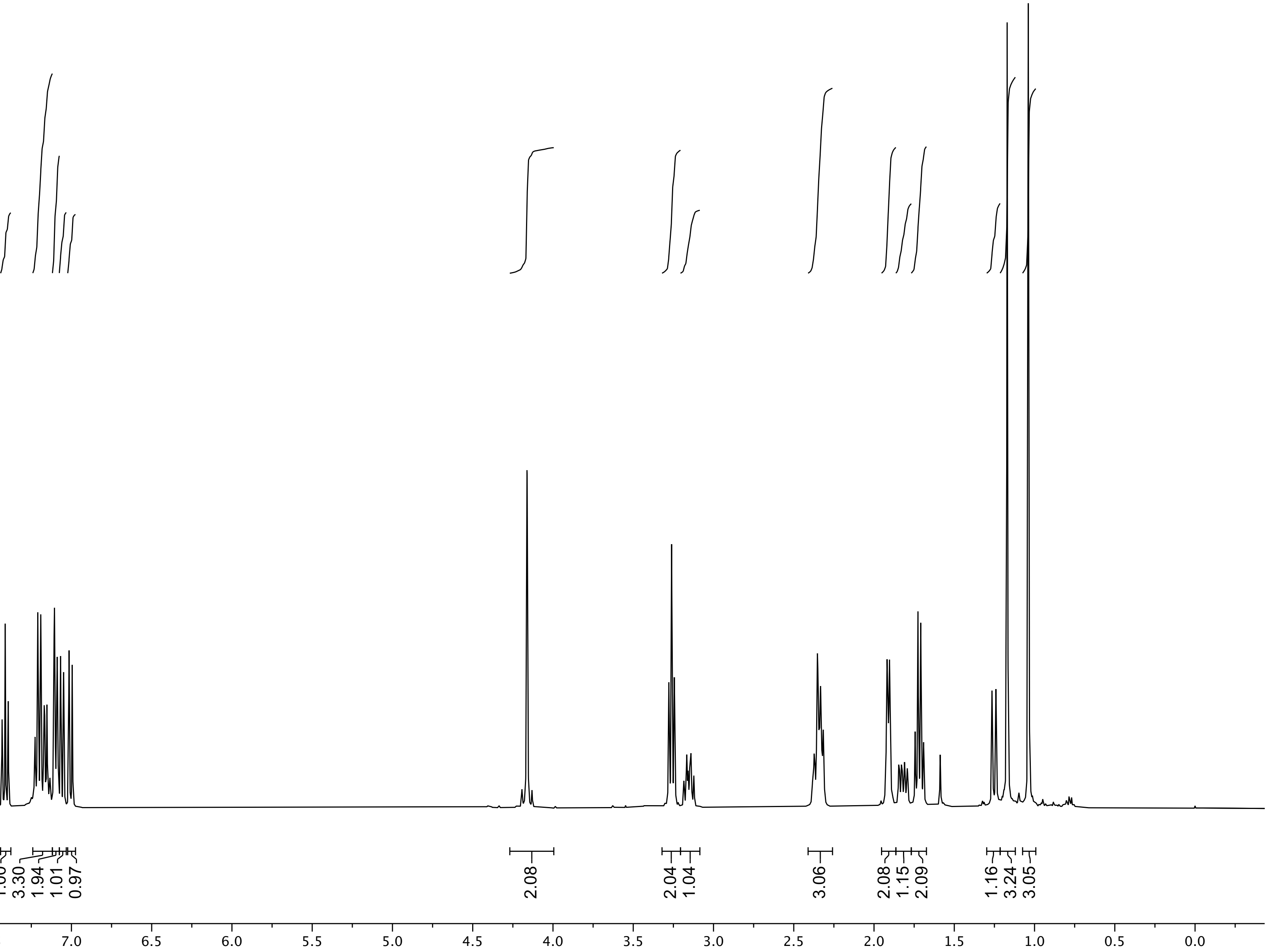

2-(2-(2-(Benzyloxy)ethyl)-6,6-dimethylbicyclo[3.1.1]heptan-3-yl)-6-chloropyridine (25)

^{1}H-NMR (400 MHz, $CDCl_3$)

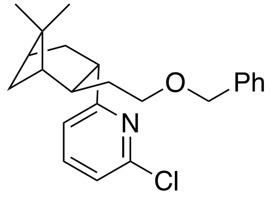

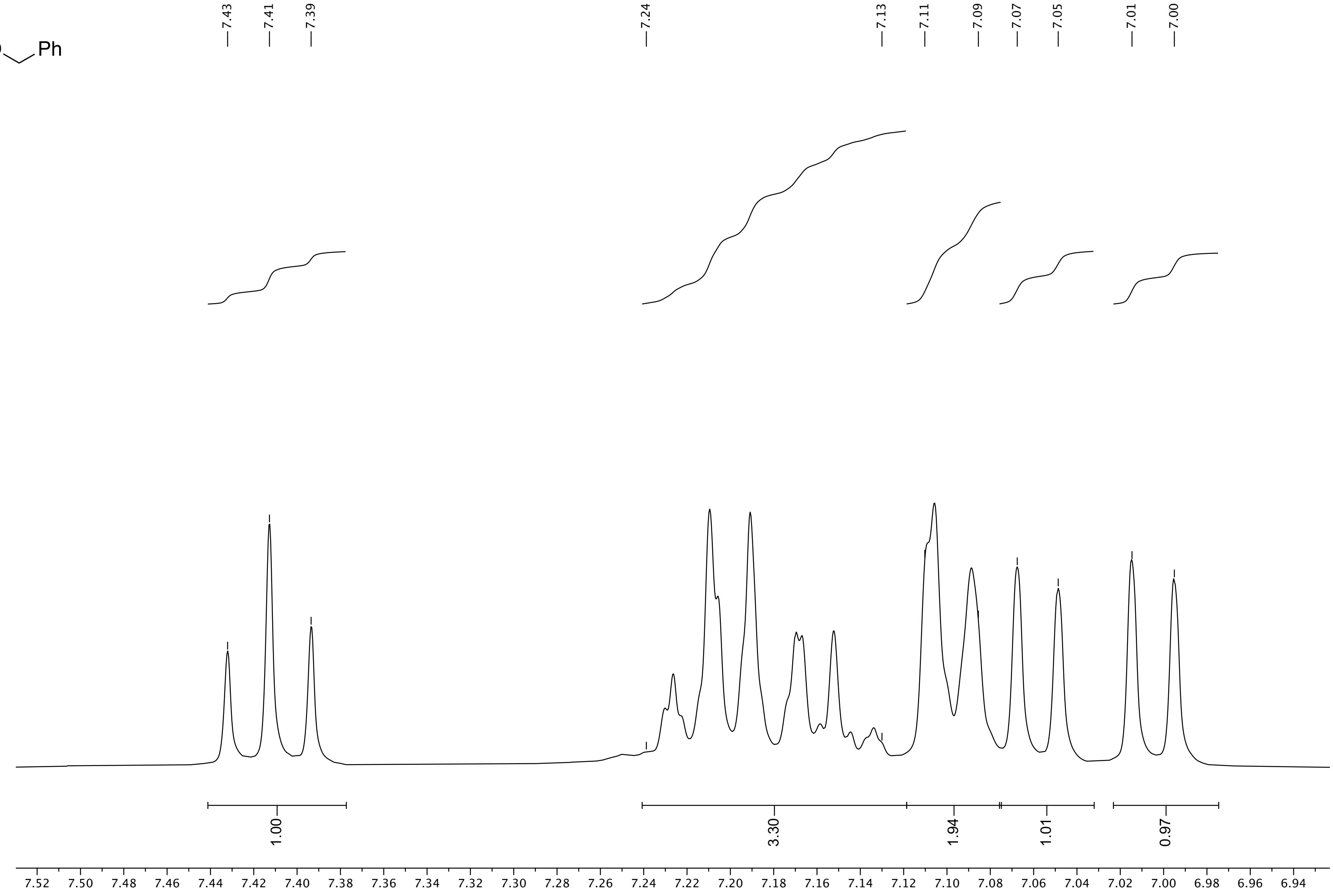

2-(2-(2-(Benzyloxy)ethyl)-6,6-dimethylbicyclo[3.1.1]heptan-3-yl)-6-chloropyridine (25)

^{1}H-NMR (400 MHz, $CDCl_3$)

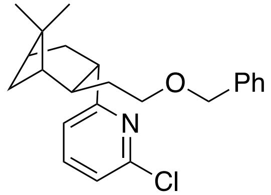

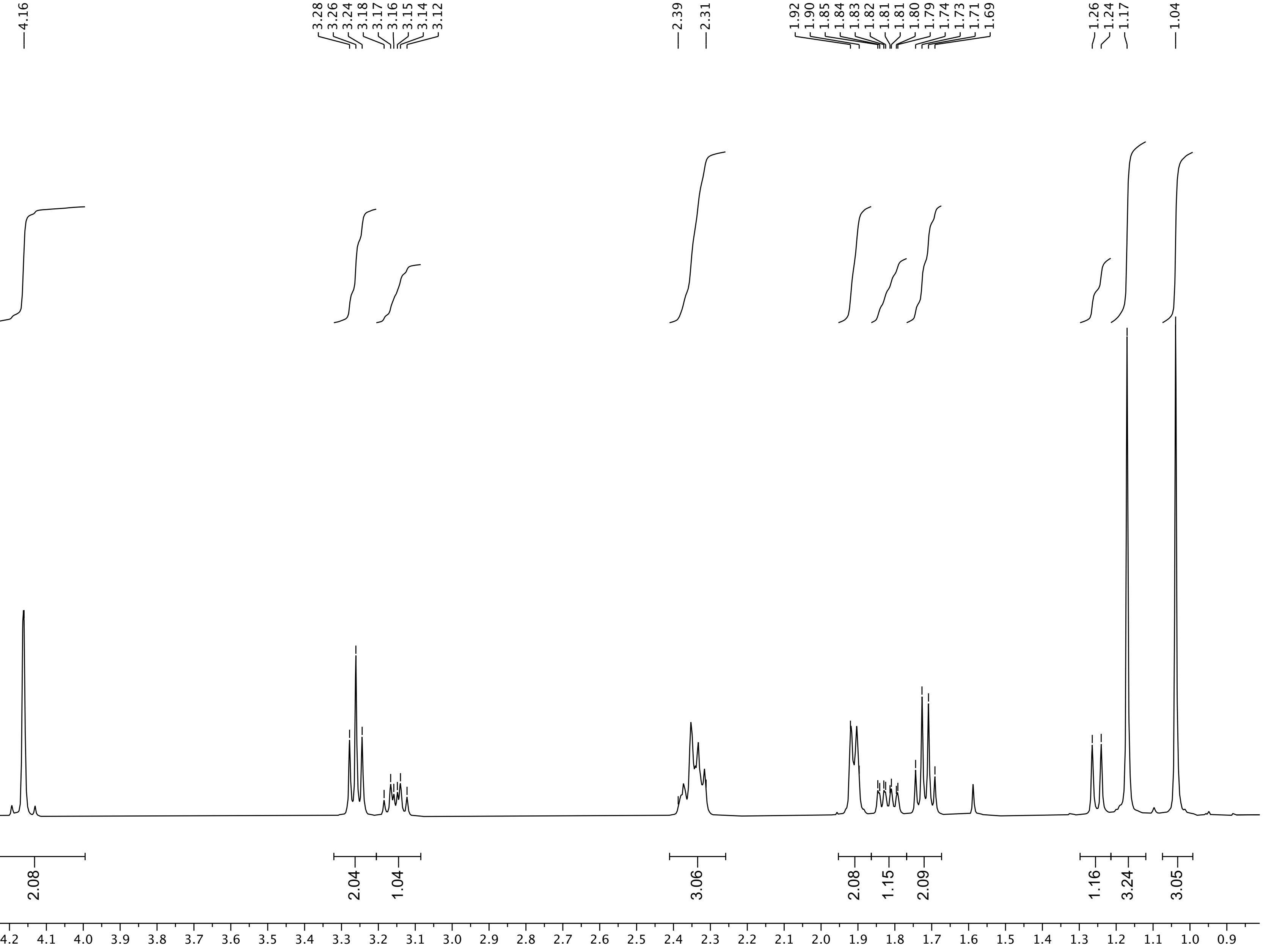

2-(2-(2-(Benzyloxy)ethyl)-6,6-dimethylbicyclo[3.1.1]heptan-3-yl)-6-chloropyridine (25)

^{1}H-NMR (400 MHz, $CDCl_3$)

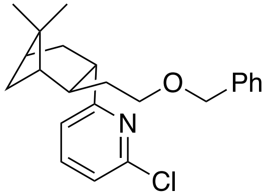

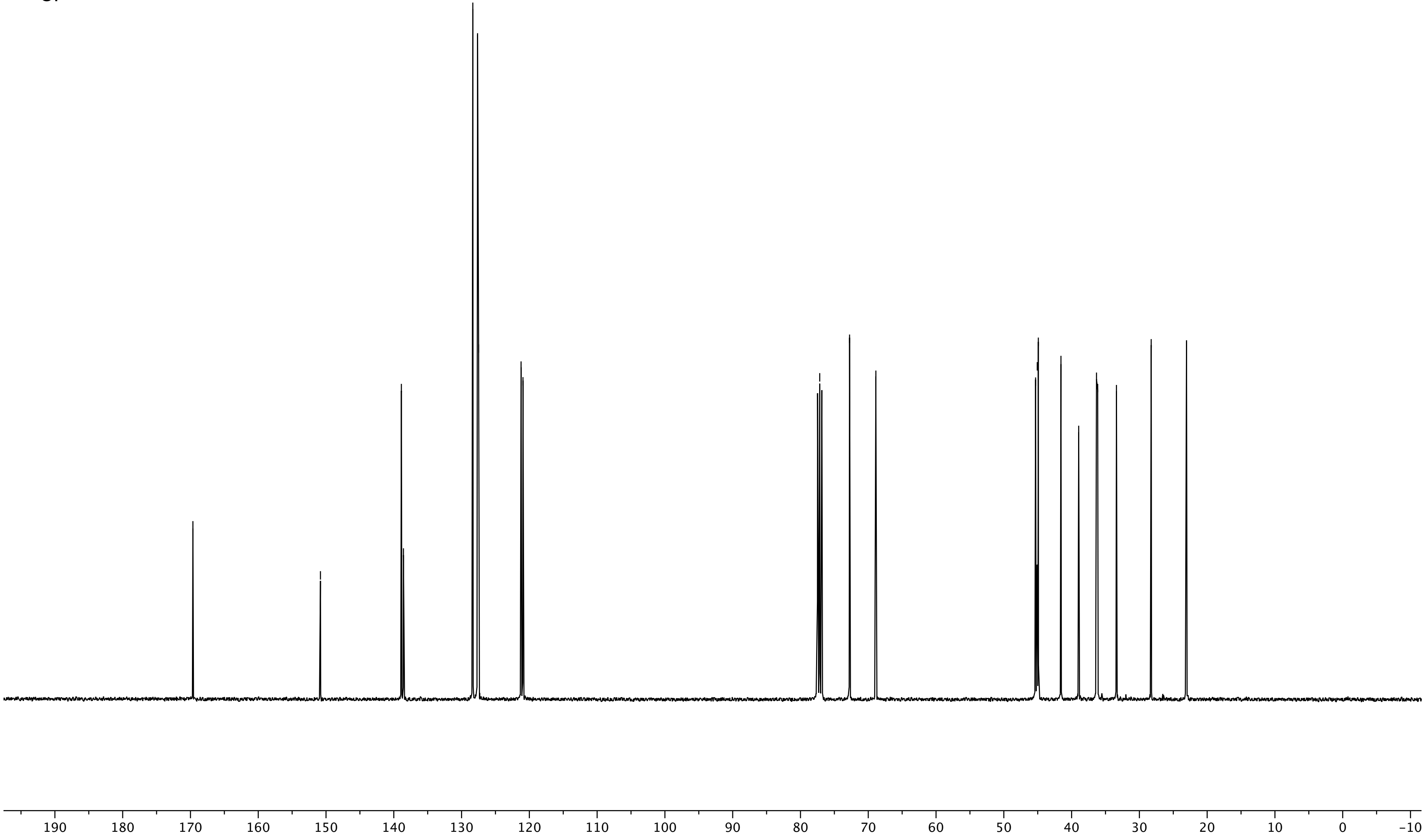

2-(2-(2-(Benzyloxy)ethyl)-6,6-dimethylbicyclo[3.1.1]heptan-3-yl)-6-chloropyridine (25)

^{1}H-NMR (400 MHz, $CDCl_3$)

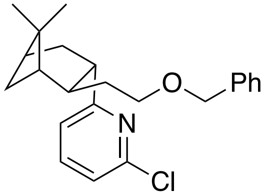

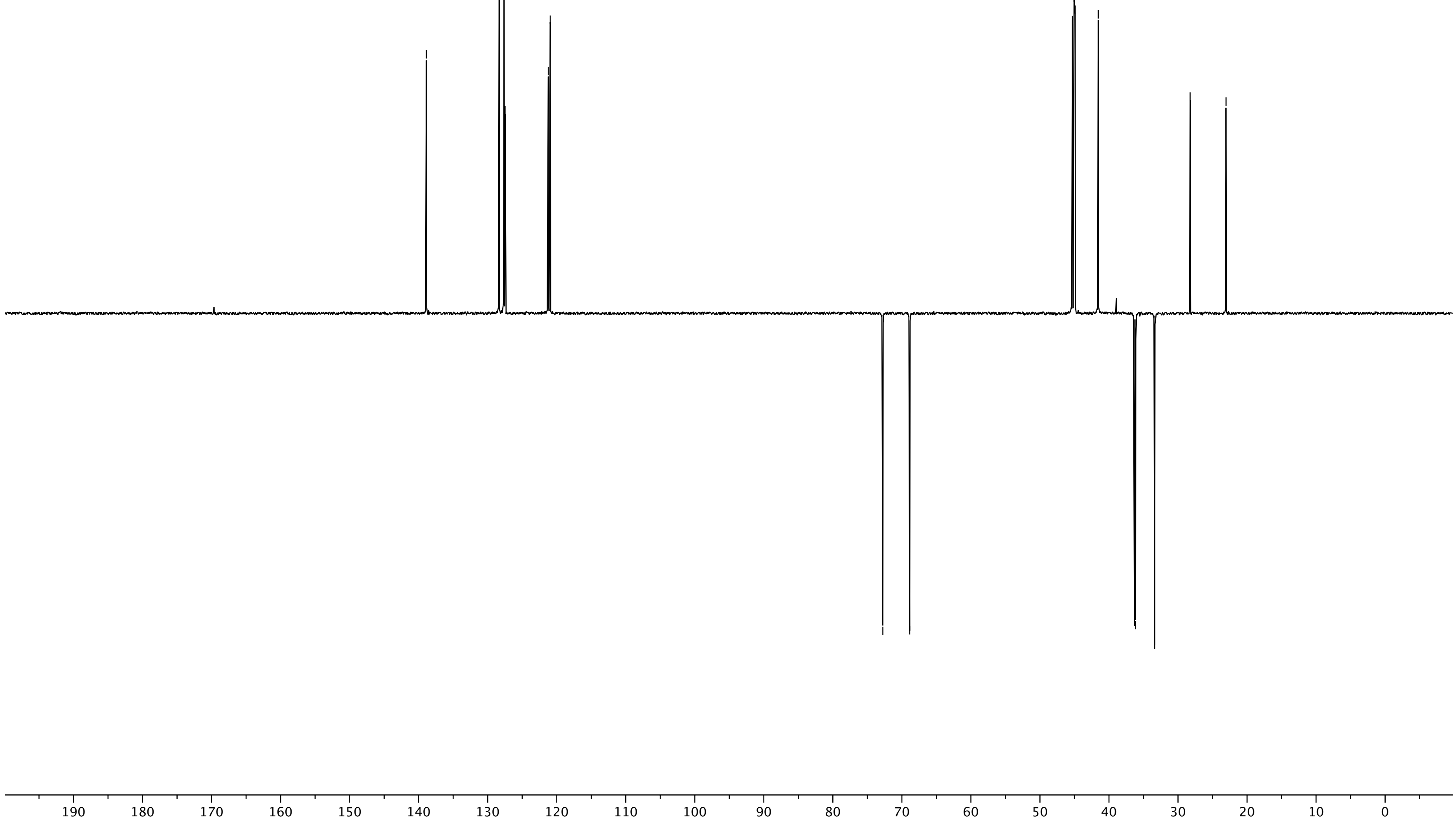

2-(2-(2-(Benzyloxy)ethyl)-6,6-dimethylbicyclo[3.1.1]heptan-3-yl)-6-chloropyridine (25)

^{1}H-^{1}H COSY (400 MHz, $CDCl_3$)

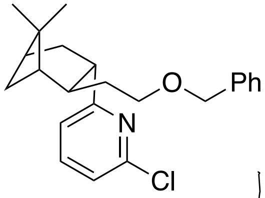

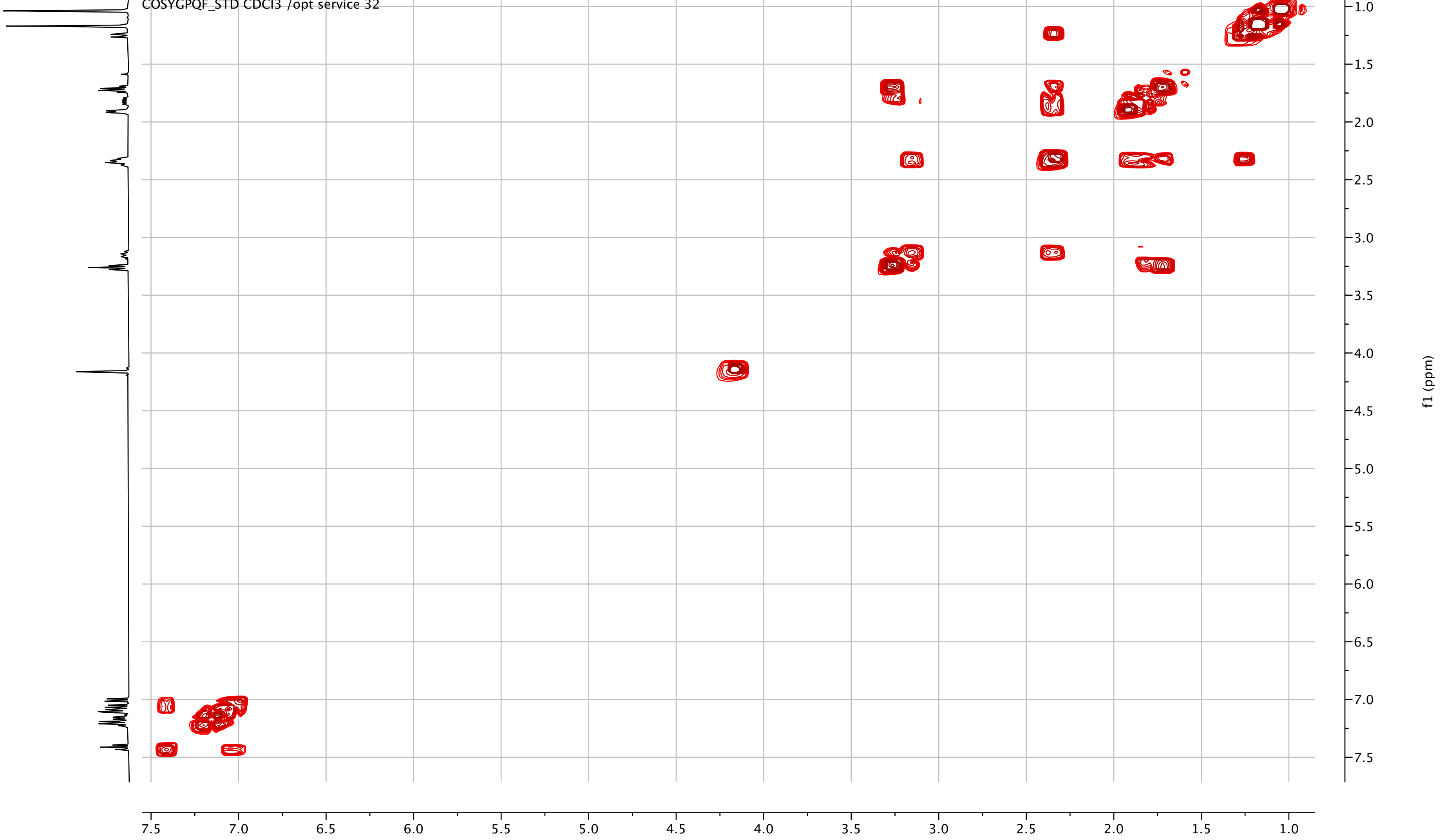

2-(2-(2-(Benzyloxy)ethyl)-6,6-dimethylbicyclo[3.1.1]heptan-3-yl)-6-chloropyridine (25)

^{1}H-^{1}H COSY (400 MHz, $CDCl_3$)

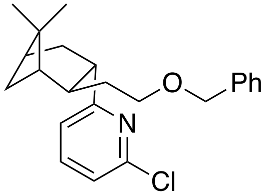

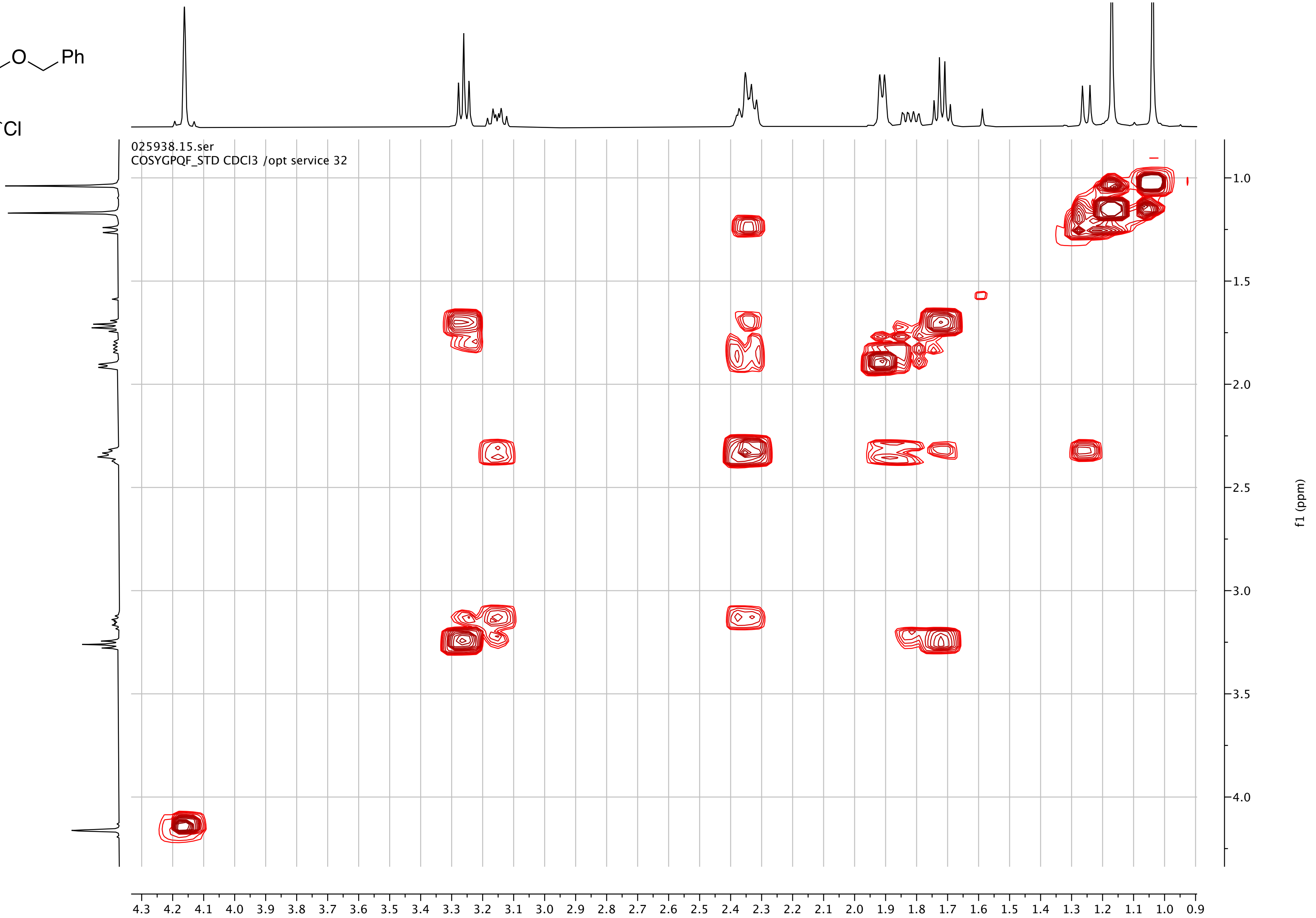

2-(2-(2-(Benzyloxy)ethyl)-6,6-dimethylbicyclo[3.1.1]heptan-3-yl)-6-chloropyridine (25)

HSQC-EDT (400 MHz, $CDCl_3$)

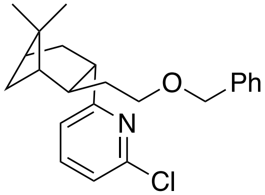

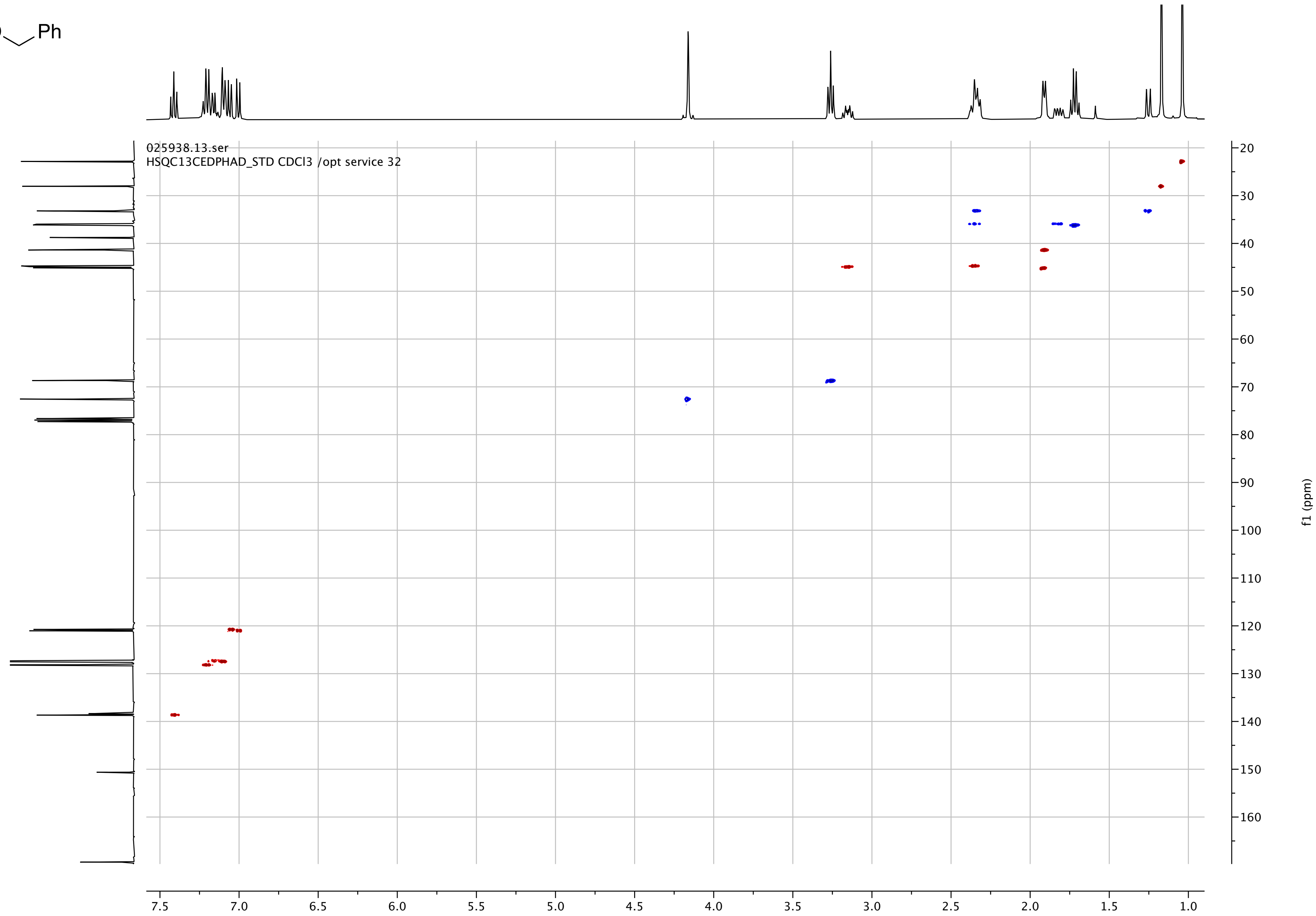

2-(2-(2-(Benzyloxy)ethyl)-6,6-dimethylbicyclo[3.1.1]heptan-3-yl)-6-chloropyridine (25)

HSQC-EDT (400 MHz, $CDCl_3$)

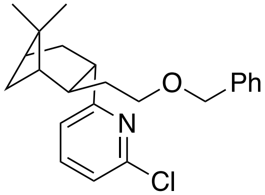

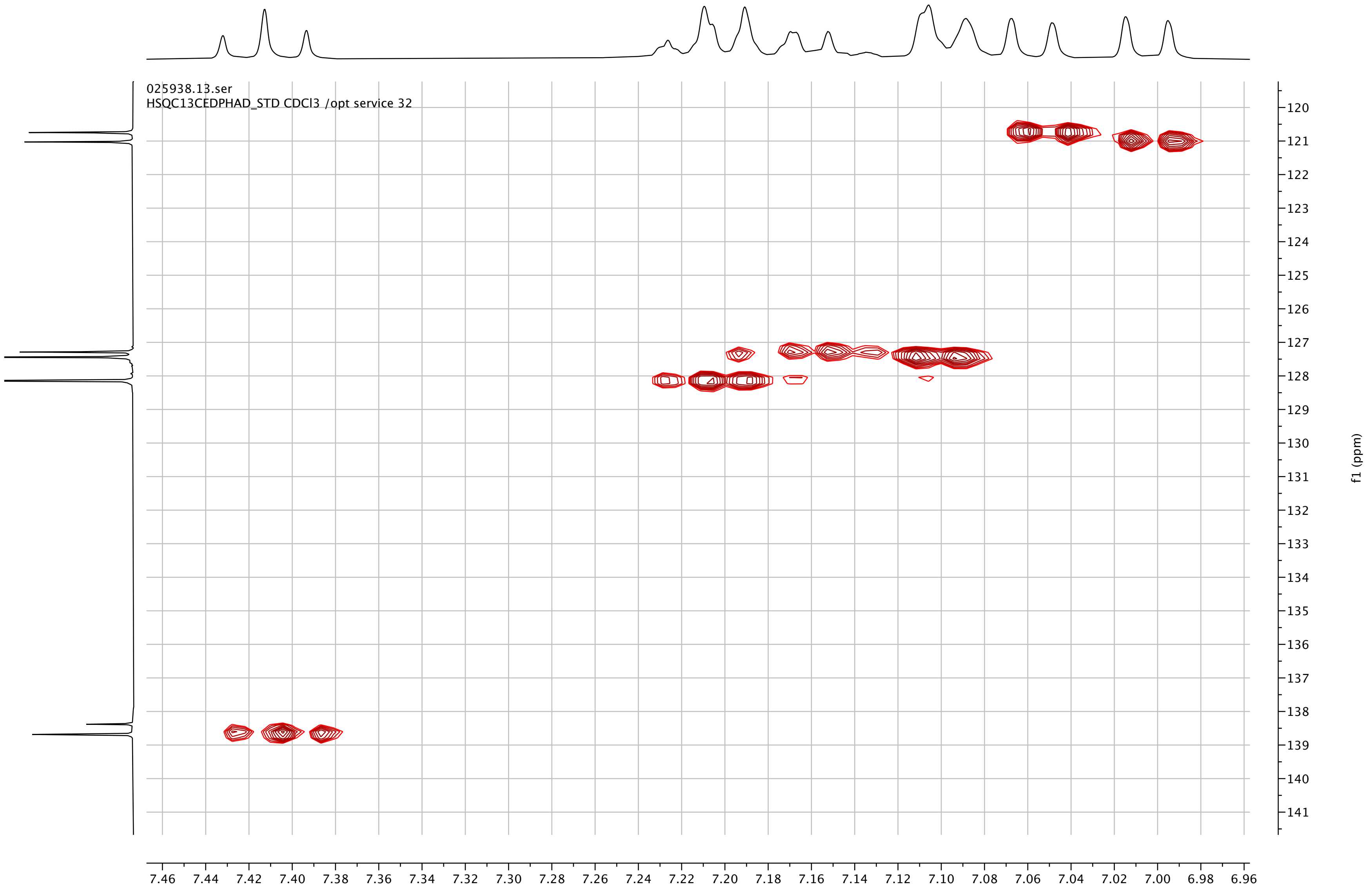

2-(2-(2-(Benzyloxy)ethyl)-6,6-dimethylbicyclo[3.1.1]heptan-3-yl)-6-chloropyridine (25)

HSQC-EDT (400 MHz, $CDCl_3$)

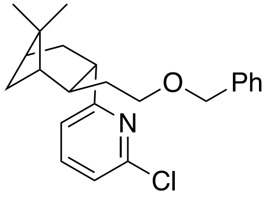

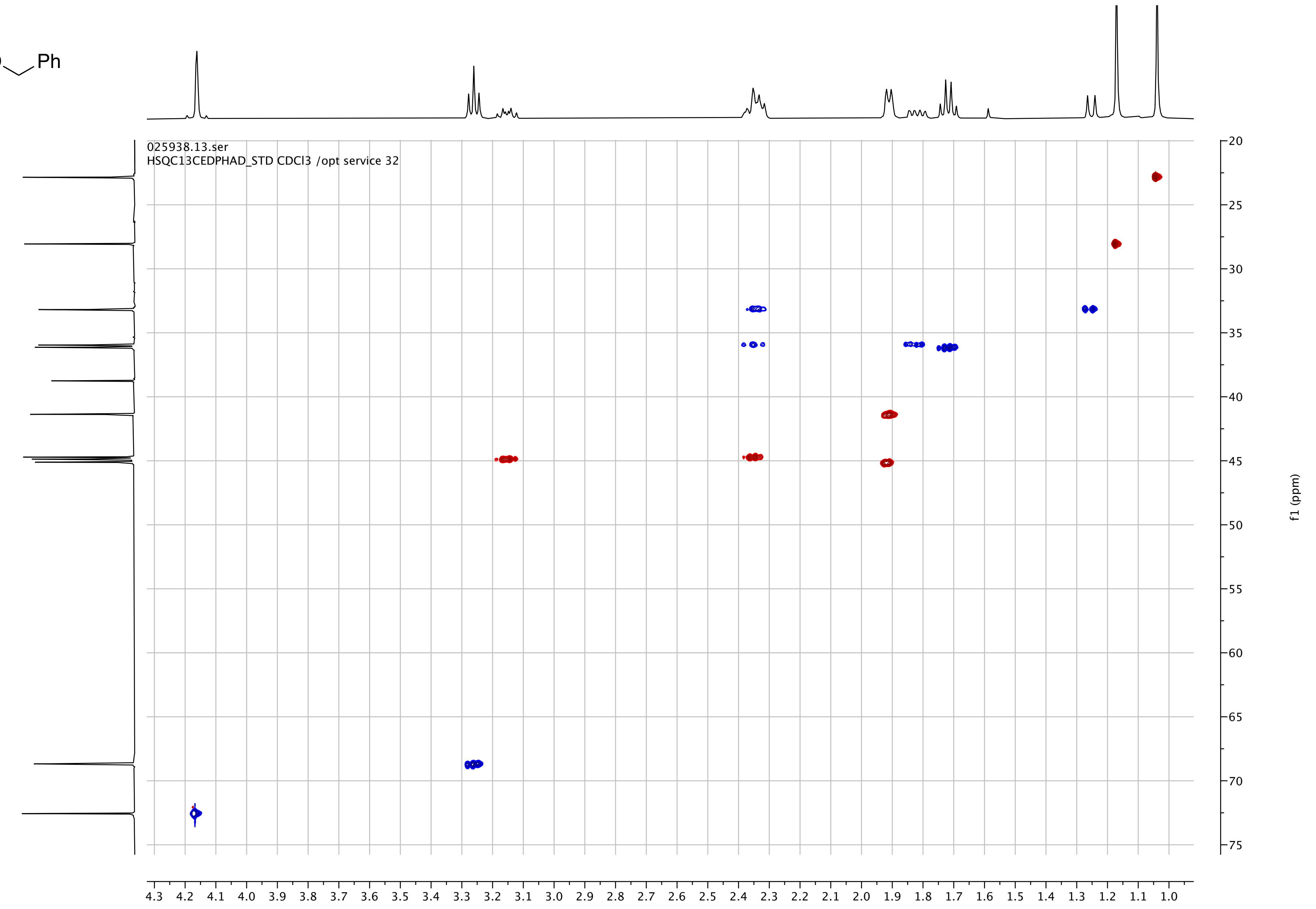

2-(2-(2-(Benzyloxy)ethyl)-6,6-dimethylbicyclo[3.1.1]heptan-3-yl)-6-chloropyridine (25)

HMBC (400 MHz, $CDCl_3$)

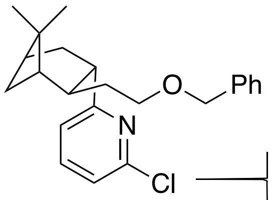

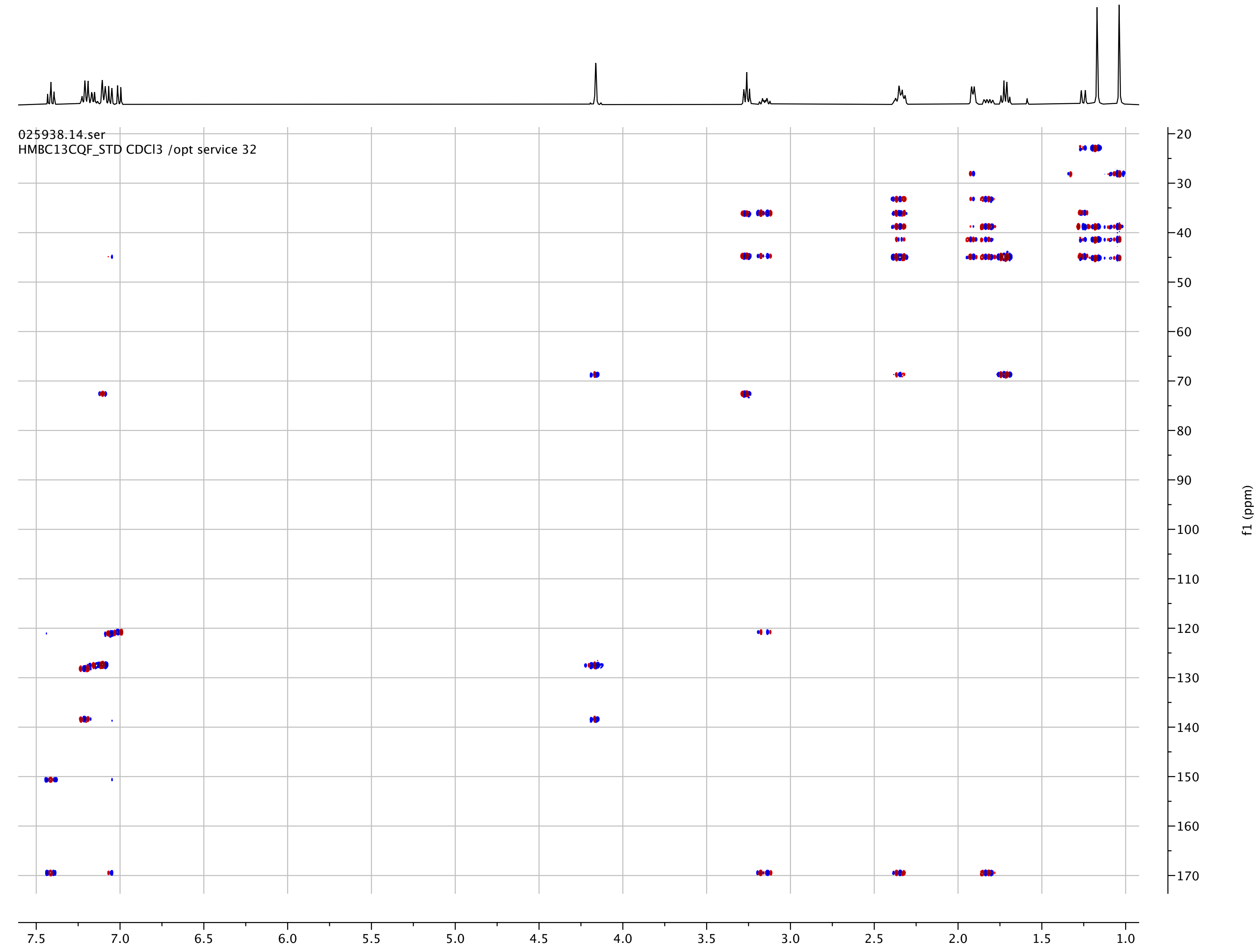

2-(2-(2-(Benzyloxy)ethyl)-6,6-dimethylbicyclo[3.1.1]heptan-3-yl)-6-chloropyridine (25)

HMBC (400 MHz, $CDCl_3$)

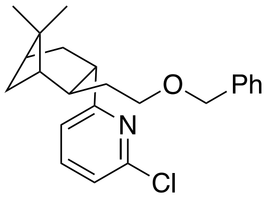

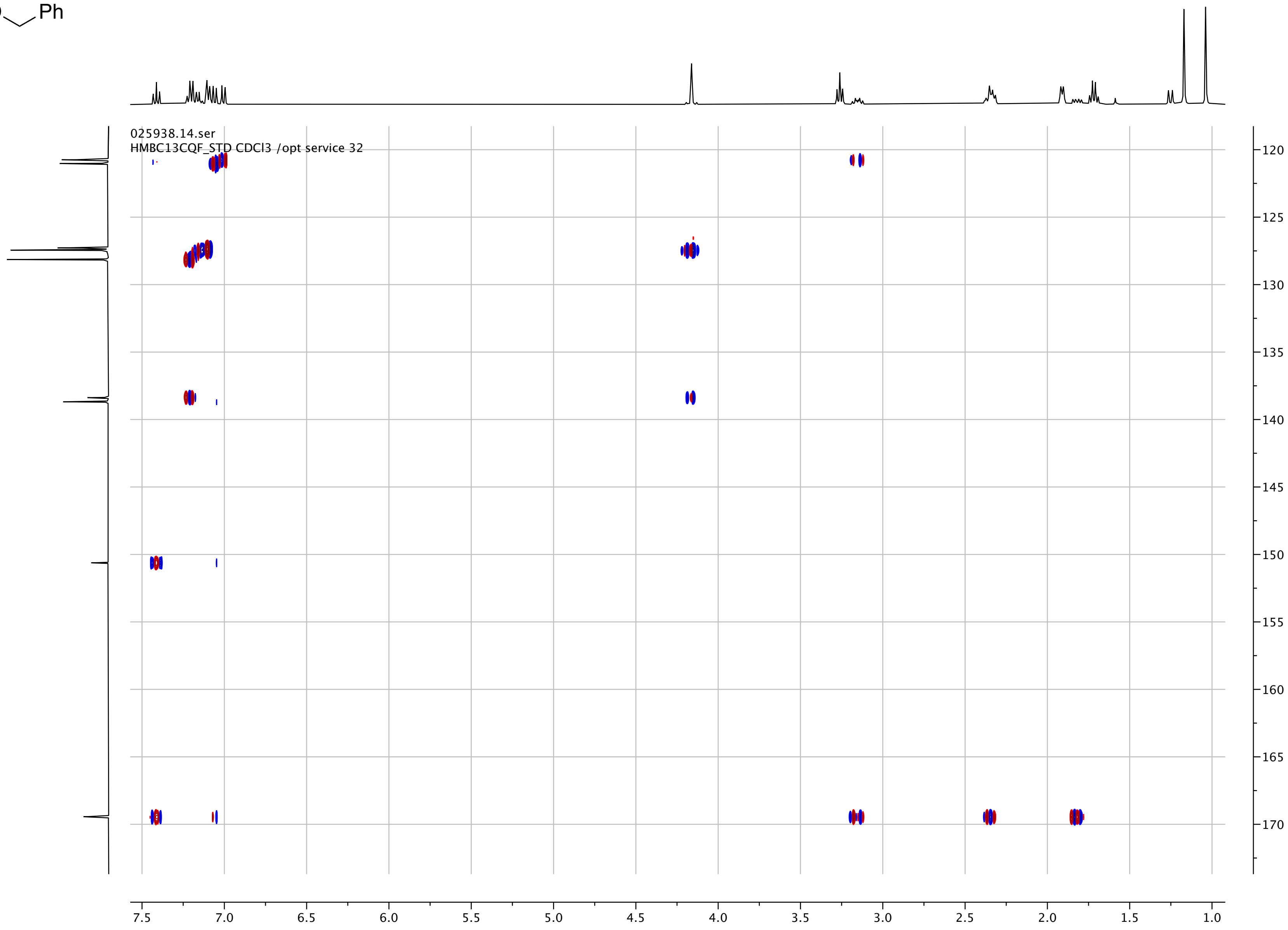

2-(2-(2-(Benzyloxy)ethyl)-6,6-dimethylbicyclo[3.1.1]heptan-3-yl)-6-chloropyridine (25)

HMBC (400 MHz, $CDCl_3$)

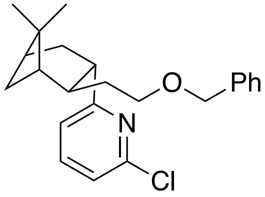

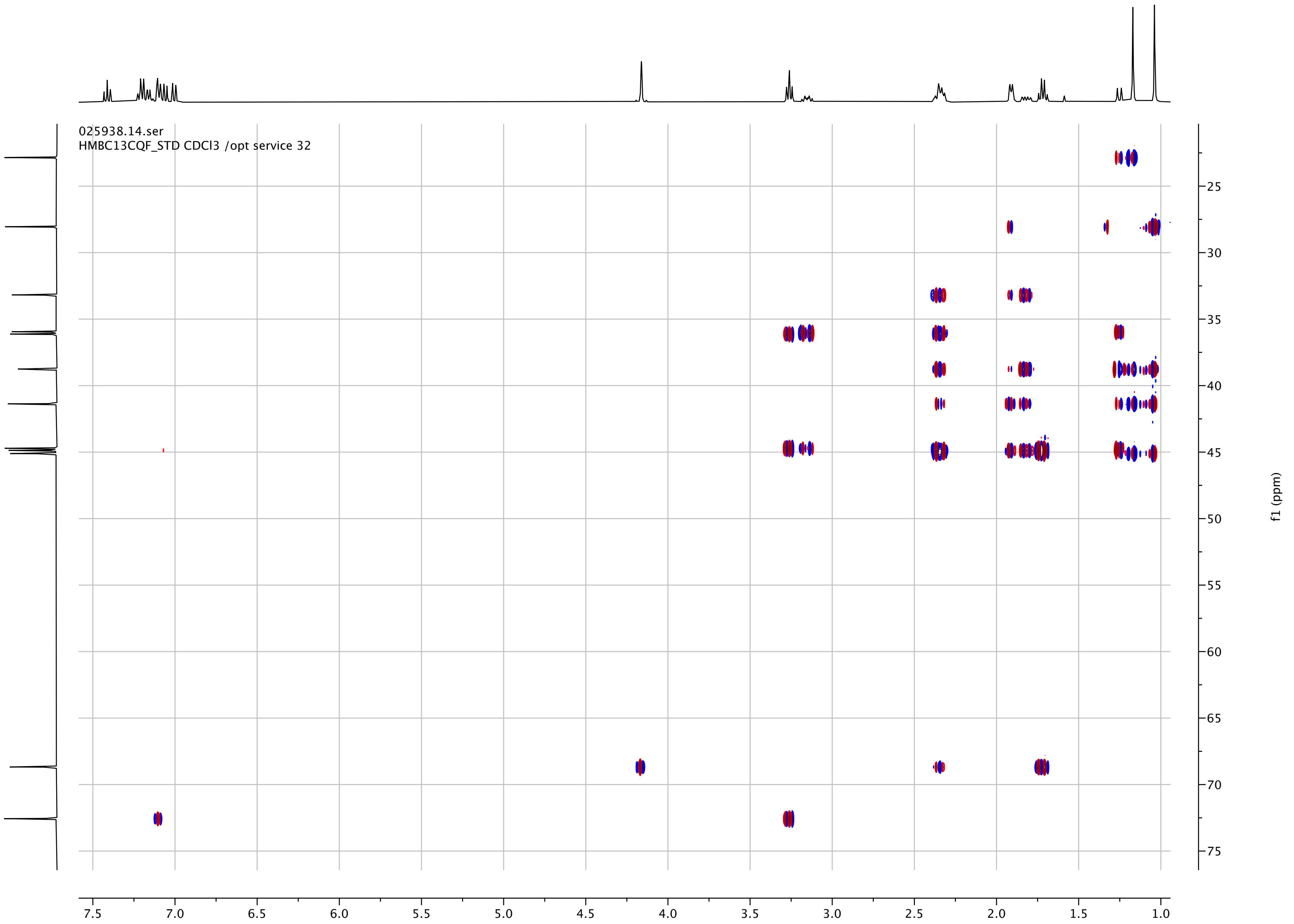

4-(2-(2-(Benzyloxy)ethyl)-6,6-dimethylbicyclo[3.1.1]heptan-3-yl)-2-chloropyridine (C_4-25, minor) ^{1}H-NMR (400 MHz, $CDCl_3$)

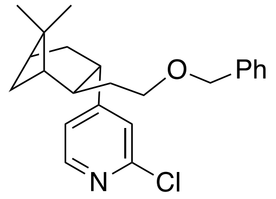

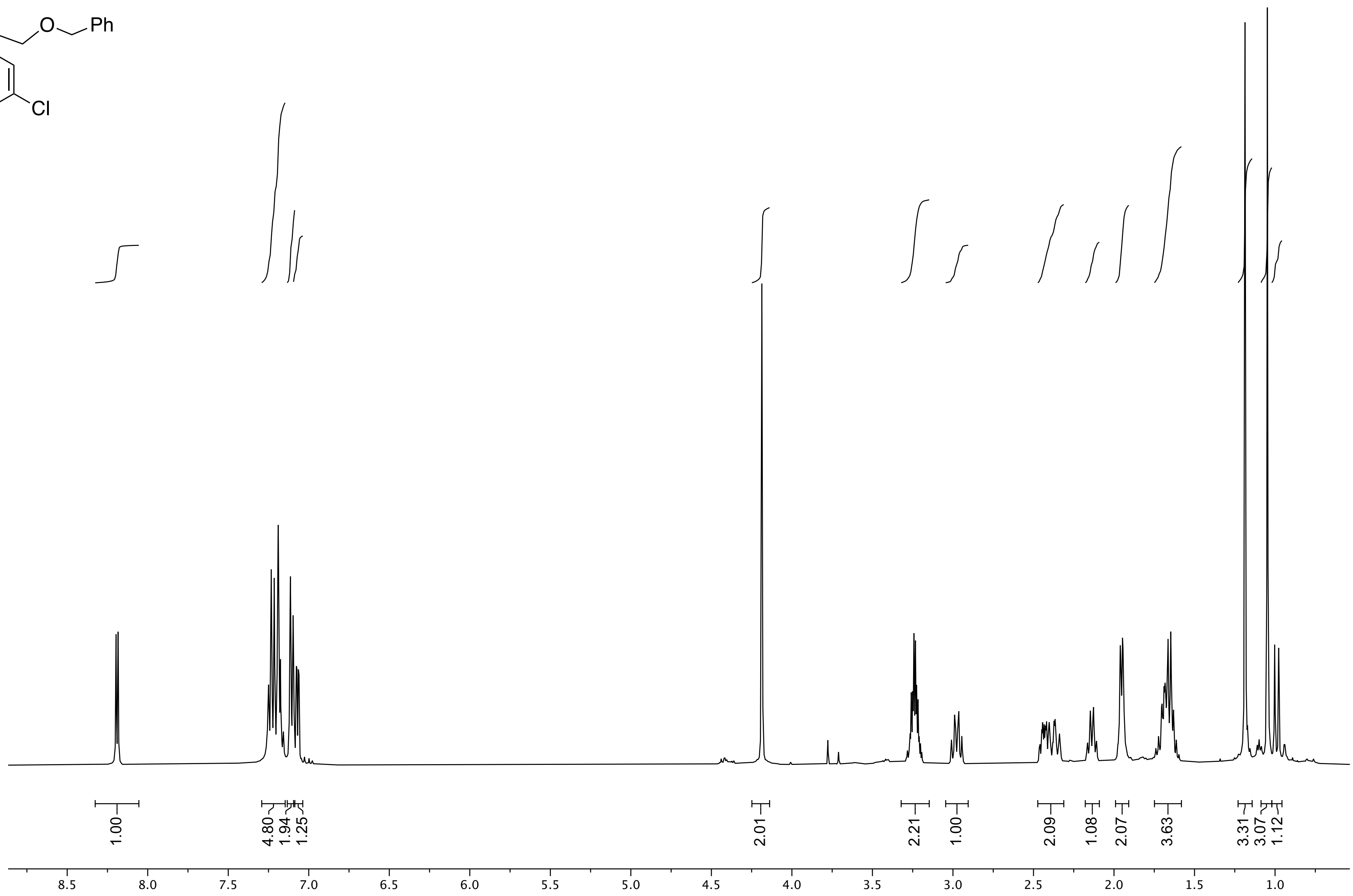

4-(2-(2-(Benzyloxy)ethyl)-6,6-dimethylbicyclo[3.1.1]heptan-3-yl)-2-chloropyridine (C_4-25, minor) ^{1}H-NMR (400 MHz, $CDCl_3$)

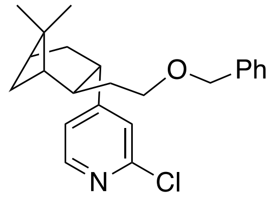

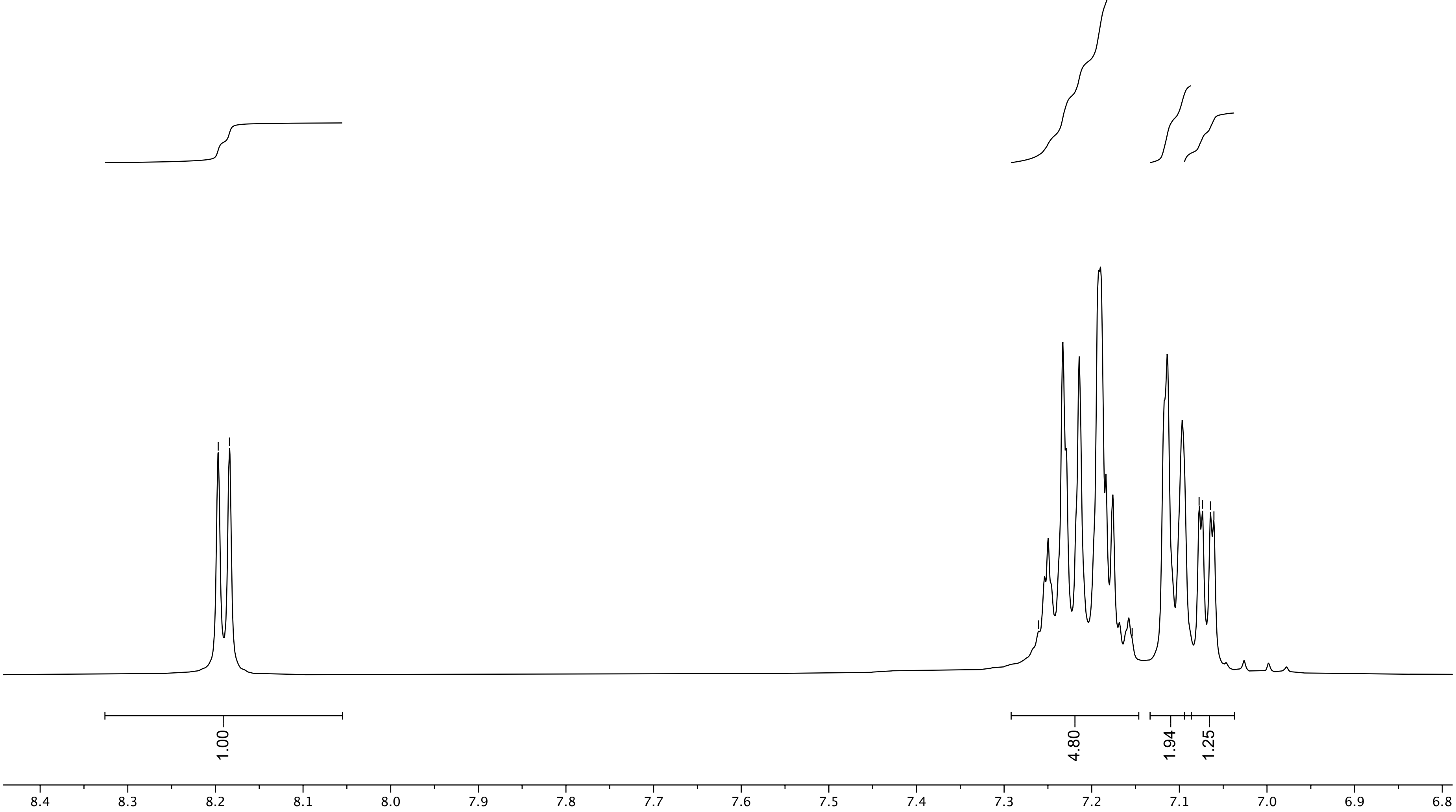

4-(2-(2-(Benzyloxy)ethyl)-6,6-dimethylbicyclo[3.1.1]heptan-3-yl)-2-chloropyridine (C_4-25, minor) ^{1}H-NMR (400 MHz, $CDCl_3$)

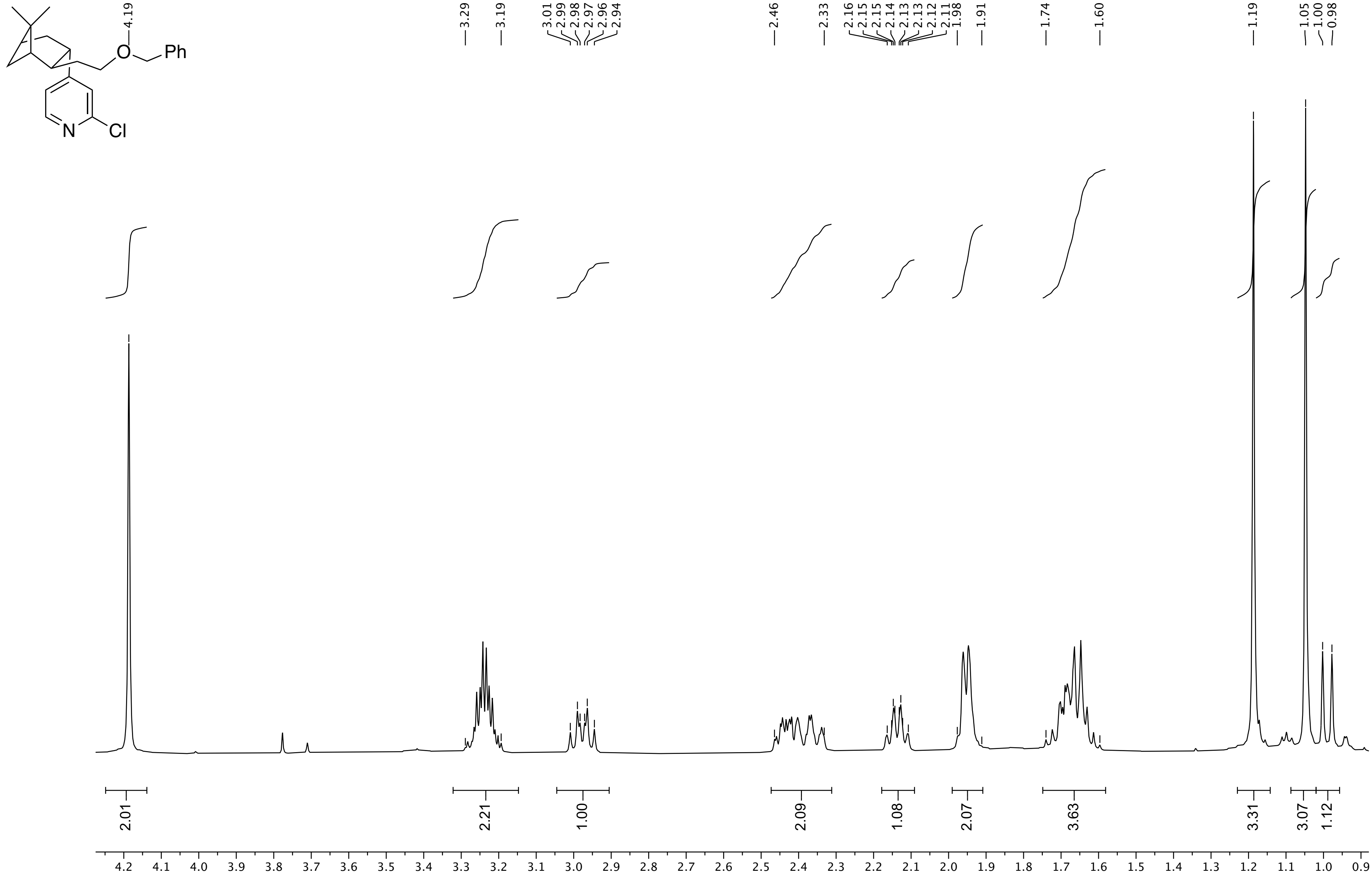

4-(2-(2-(Benzyloxy)ethyl)-6,6-dimethylbicyclo[3.1.1]heptan-3-yl)-2-chloropyridine (C_4-25, minor) ^{13}C-NMR (101 MHz, $CDCl_3$)

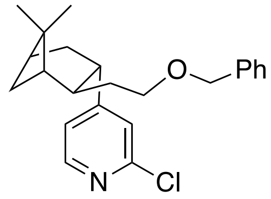

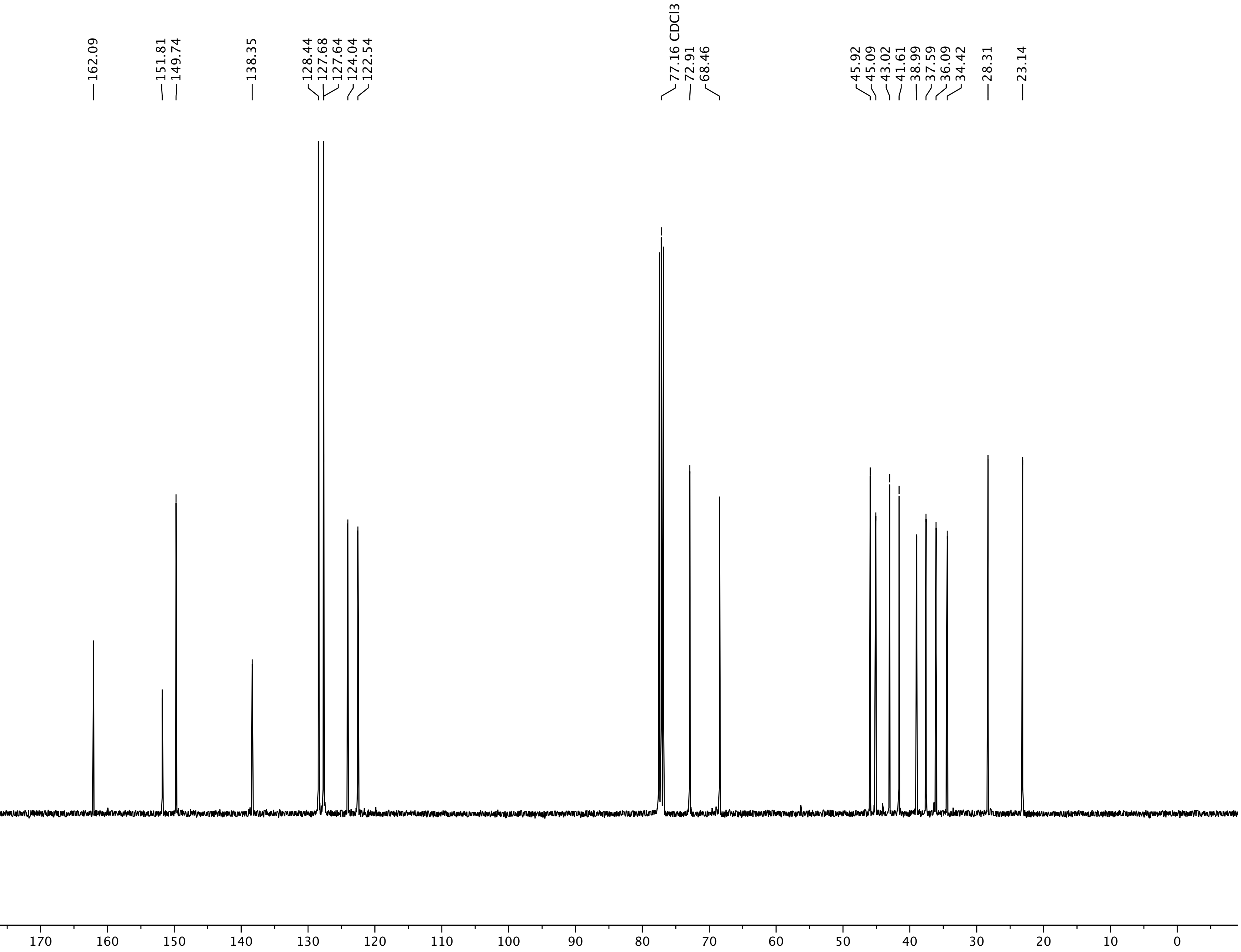

4-(2-(2-(Benzyloxy)ethyl)-6,6-dimethylbicyclo[3.1.1]heptan-3-yl)-2-chloropyridine (C_4-25, minor) ^{1}H-NMR (400 MHz, $CDCl_3$)

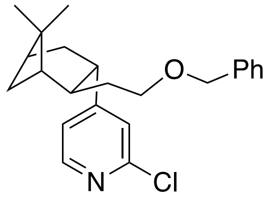

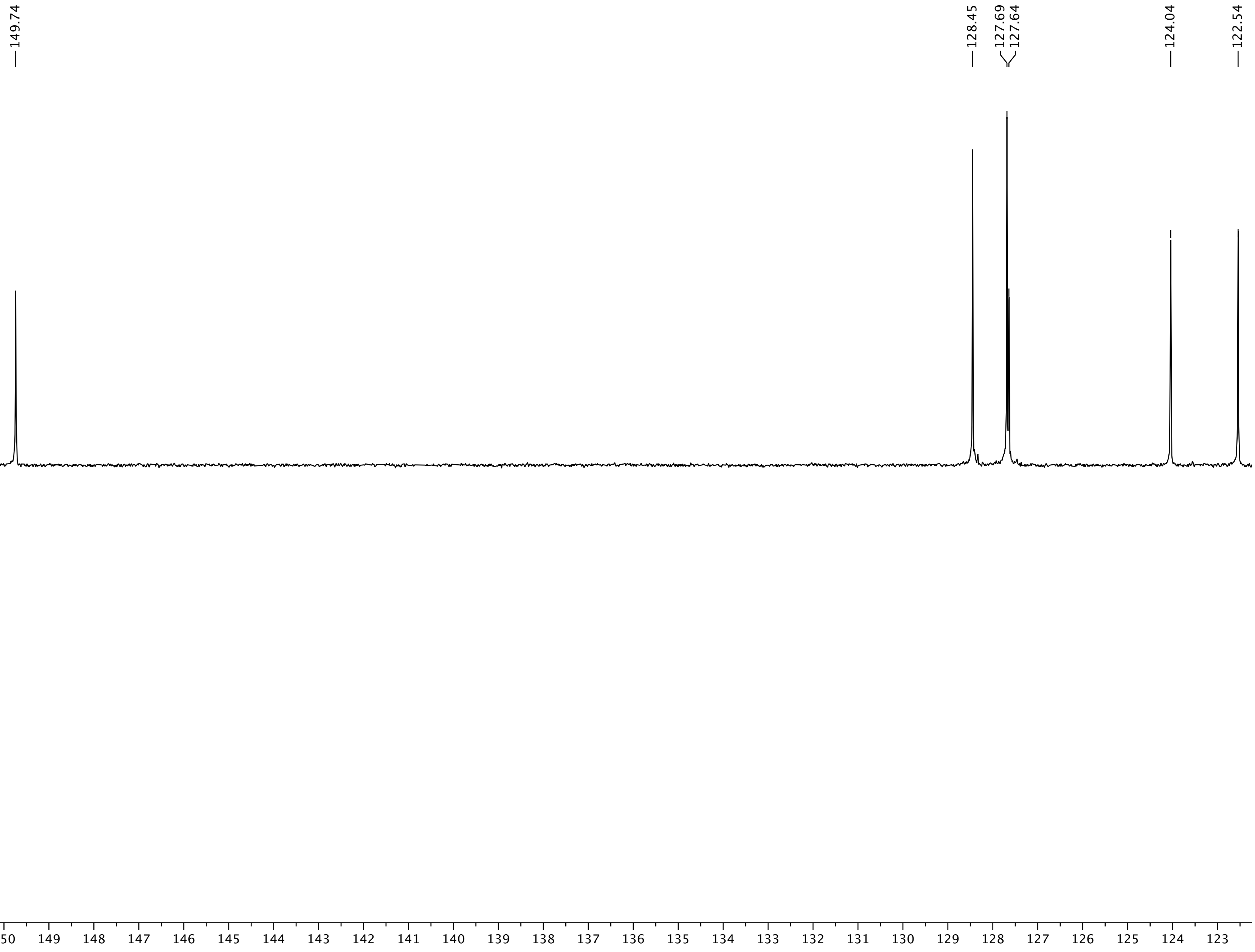

4-(2-(2-(Benzyloxy)ethyl)-6,6-dimethylbicyclo[3.1.1]heptan-3-yl)-2-chloropyridine (C_4-25, minor) ^{1}H-NMR (400 MHz, $CDCl_3$)

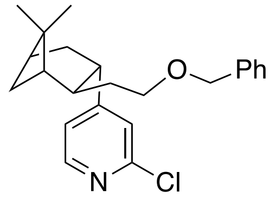

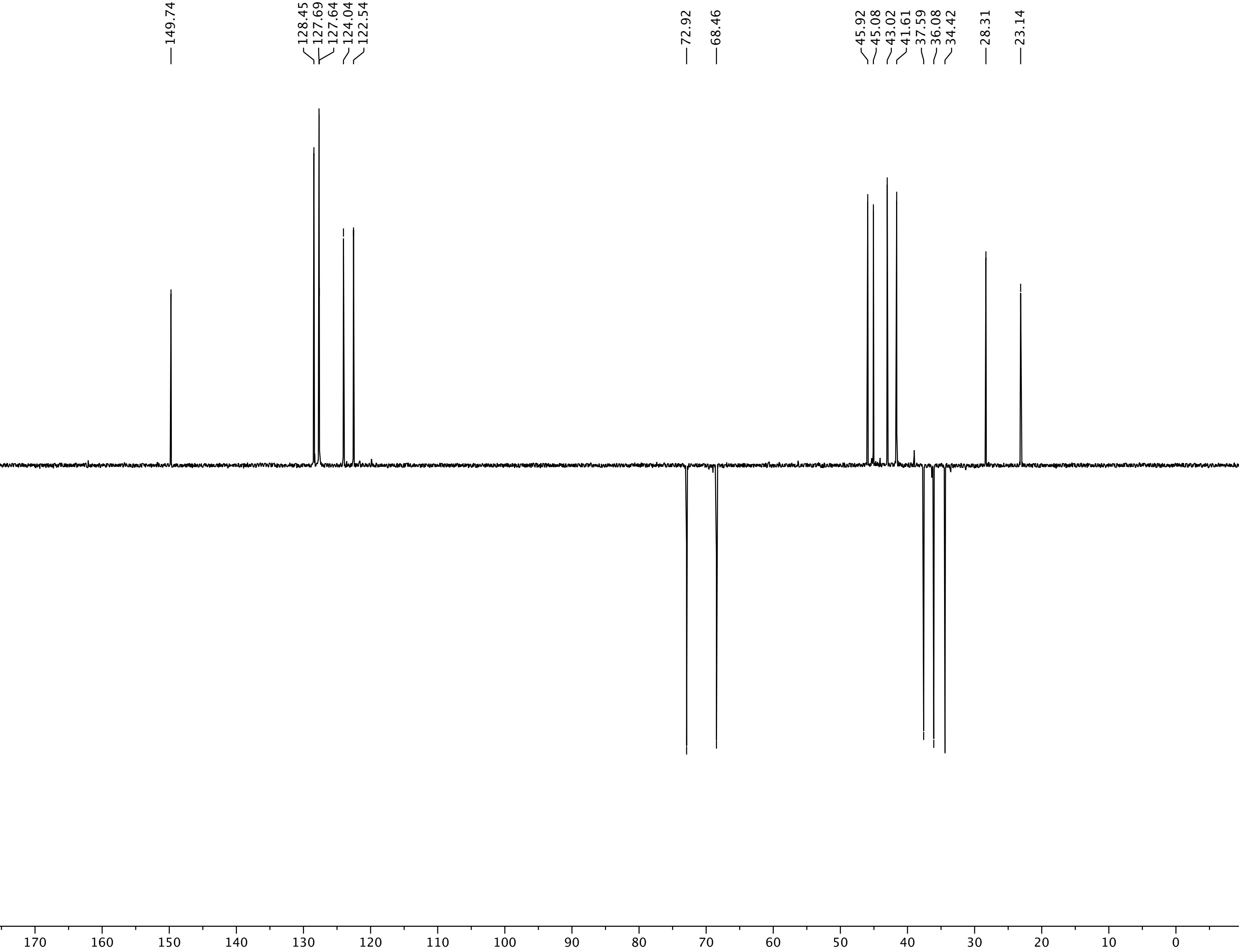

4-(2-(2-(Benzyloxy)ethyl)-6,6-dimethylbicyclo[3.1.1]heptan-3-yl)-2-chloropyridine (C_4-25, minor)

^1H-^1H COSY (400 MHz, $CDCl_3$)

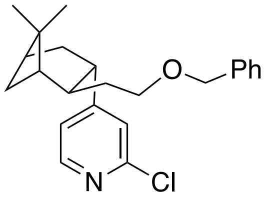

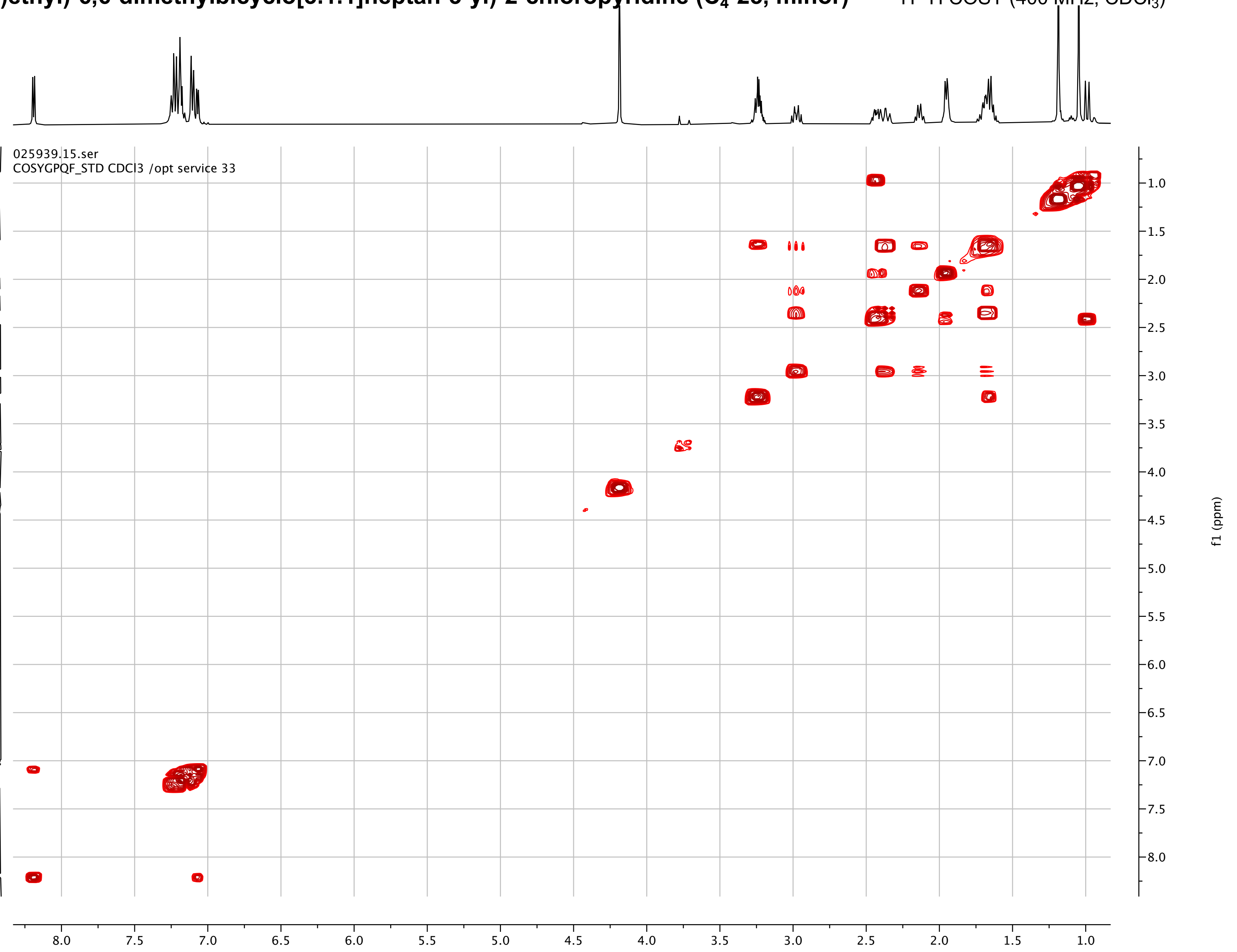

4-(2-(2-(Benzyloxy)ethyl)-6,6-dimethylbicyclo[3.1.1]heptan-3-yl)-2-chloropyridine (C_4-25, minor) ^1H-^1H COSY (400 MHz, $CDCl_3$)

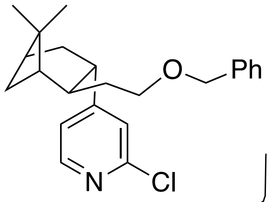

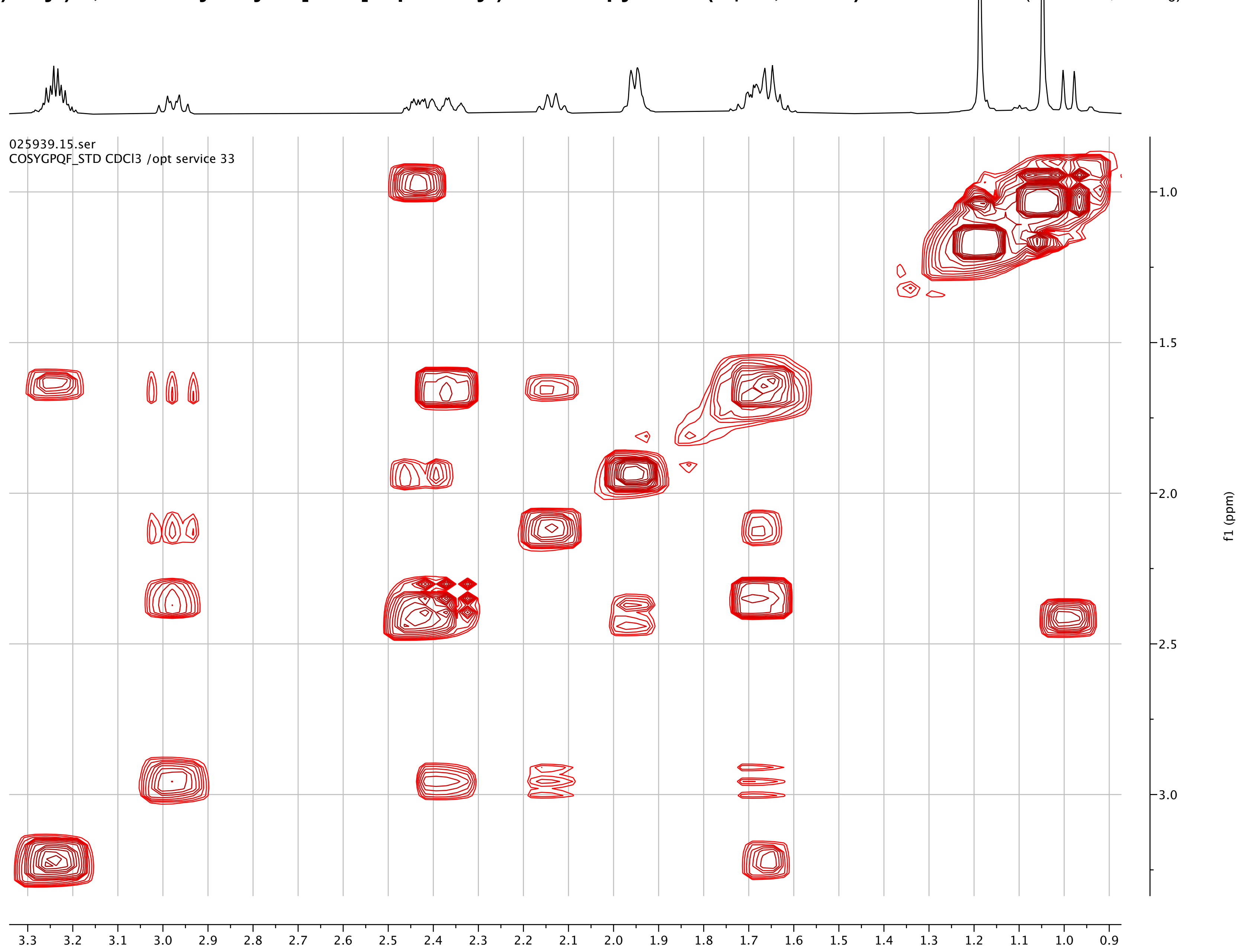

4-(2-(2-(Benzyloxy)ethyl)-6,6-dimethylbicyclo[3.1.1]heptan-3-yl)-2-chloropyridine (C_4-25, minor) ^{1}H-NMR (400 MHz, $CDCl_3$)

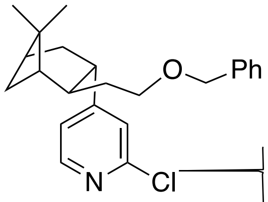

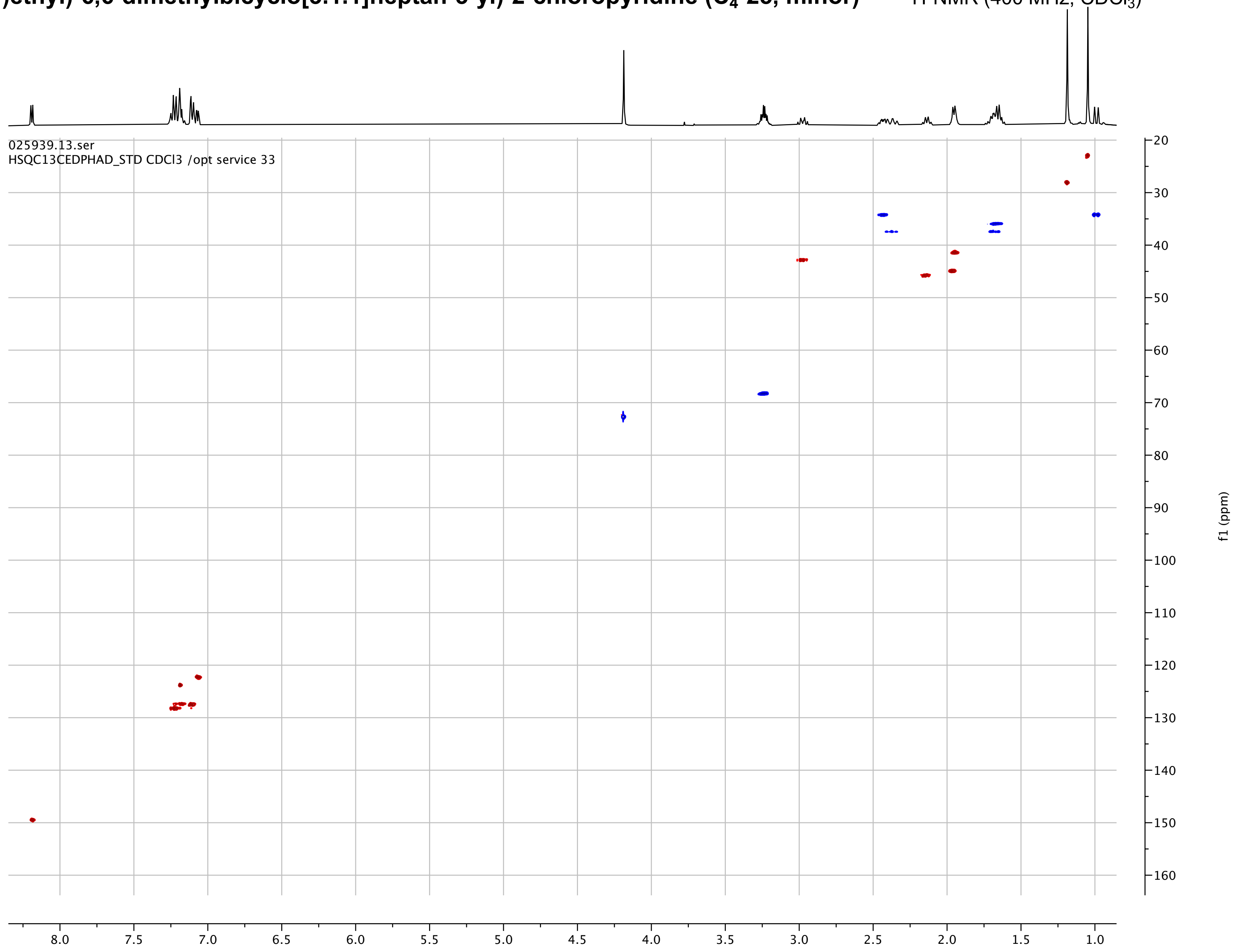

4-(2-(2-(Benzyloxy)ethyl)-6,6-dimethylbicyclo[3.1.1]heptan-3-yl)-2-chloropyridine (C_4-25, minor)

^{1}H-NMR (400 MHz, $CDCl_3$)

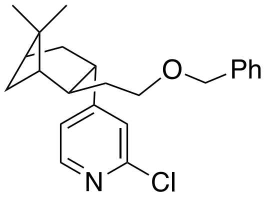

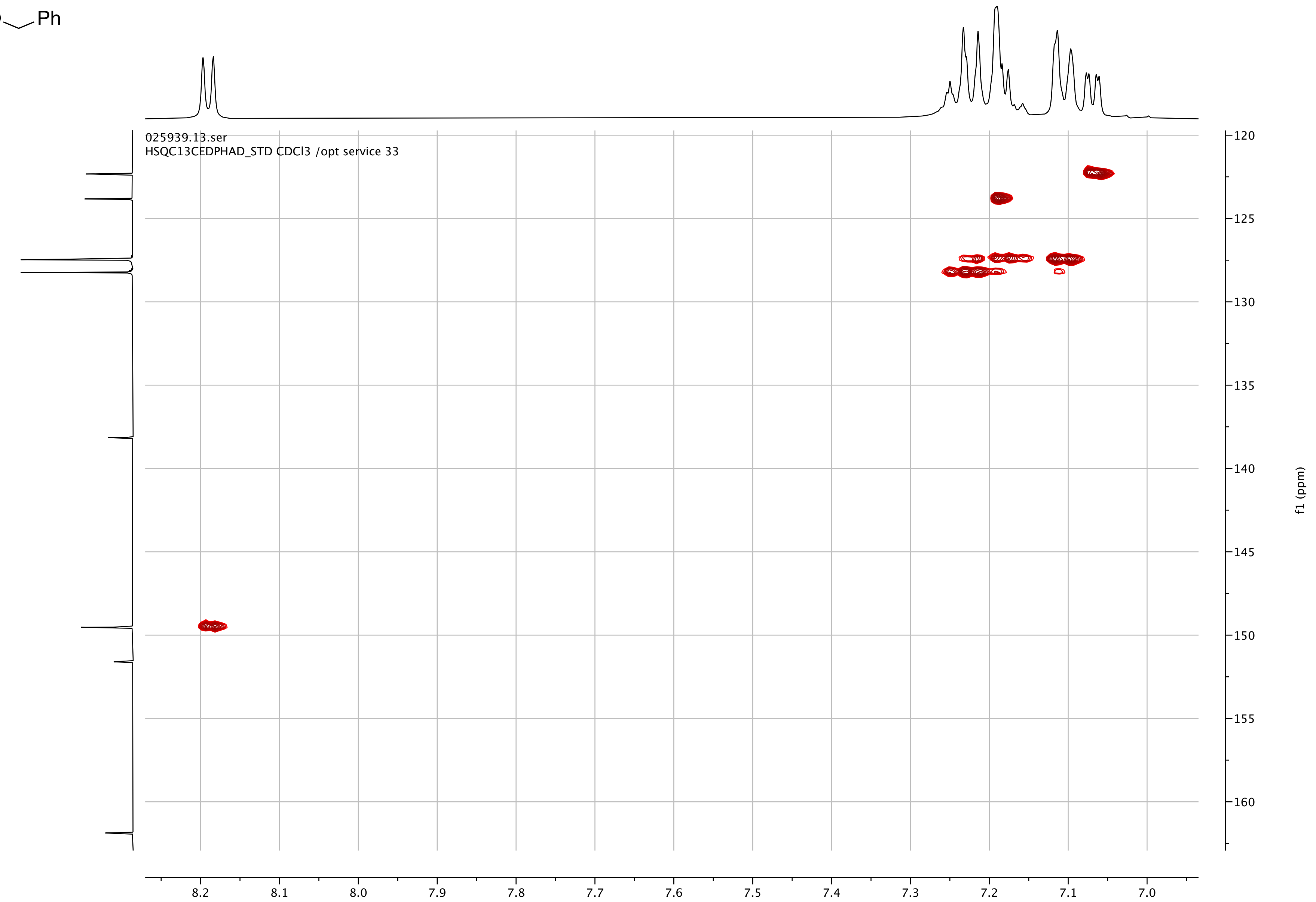

4-(2-(2-(Benzyloxy)ethyl)-6,6-dimethylbicyclo[3.1.1]heptan-3-yl)-2-chloropyridine (C_4-25, minor)

^{1}H-NMR (400 MHz, $CDCl_3$)

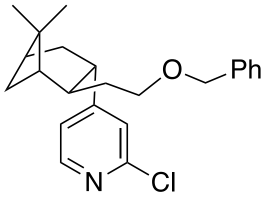

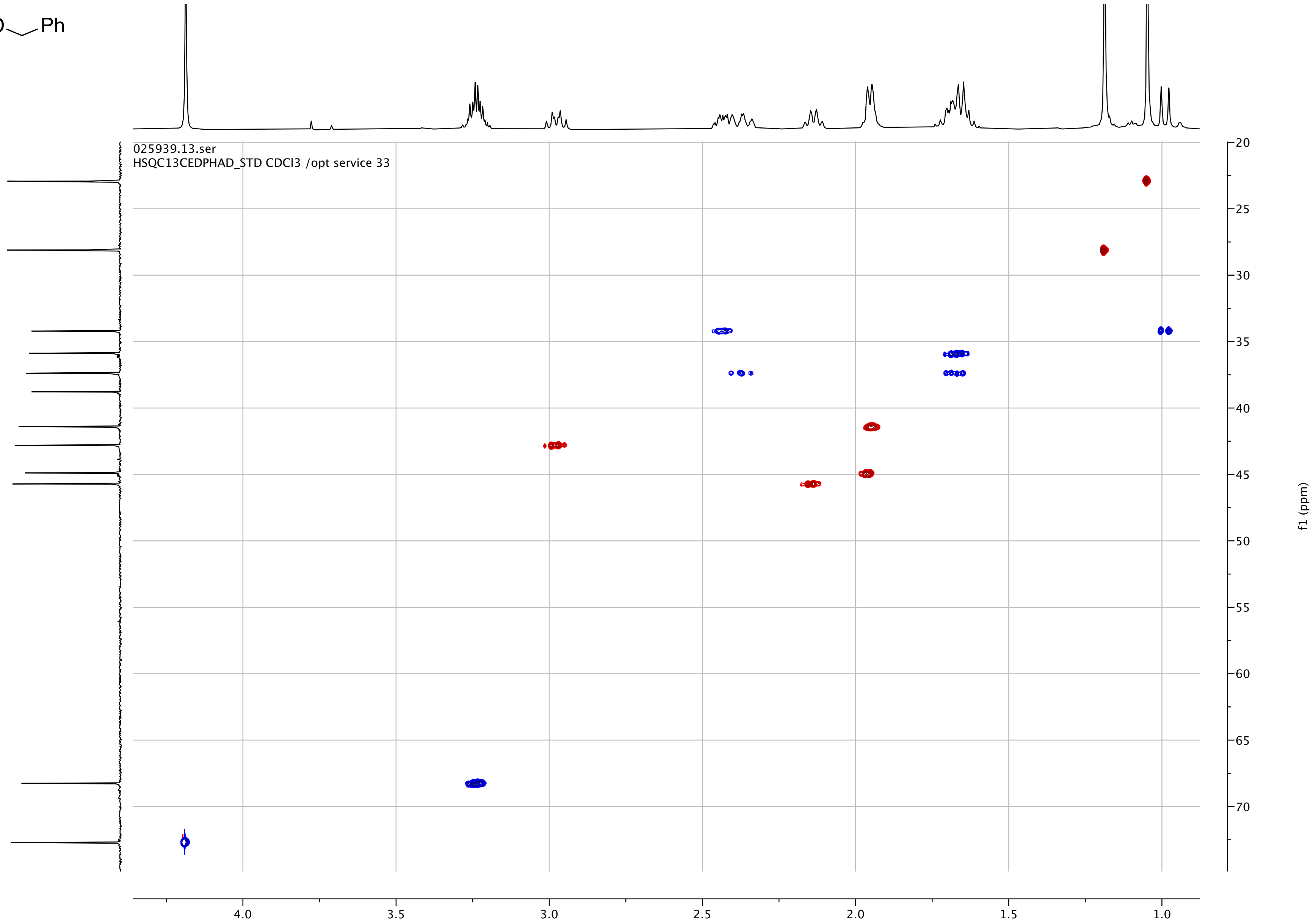

4-(2-(2-(Benzyloxy)ethyl)-6,6-dimethylbicyclo[3.1.1]heptan-3-yl)-2-chloropyridine (C_4-25, minor) HMBC (400 MHz, $CDCl_3$)

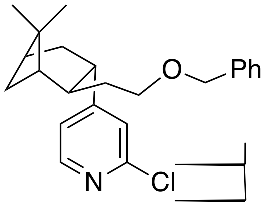

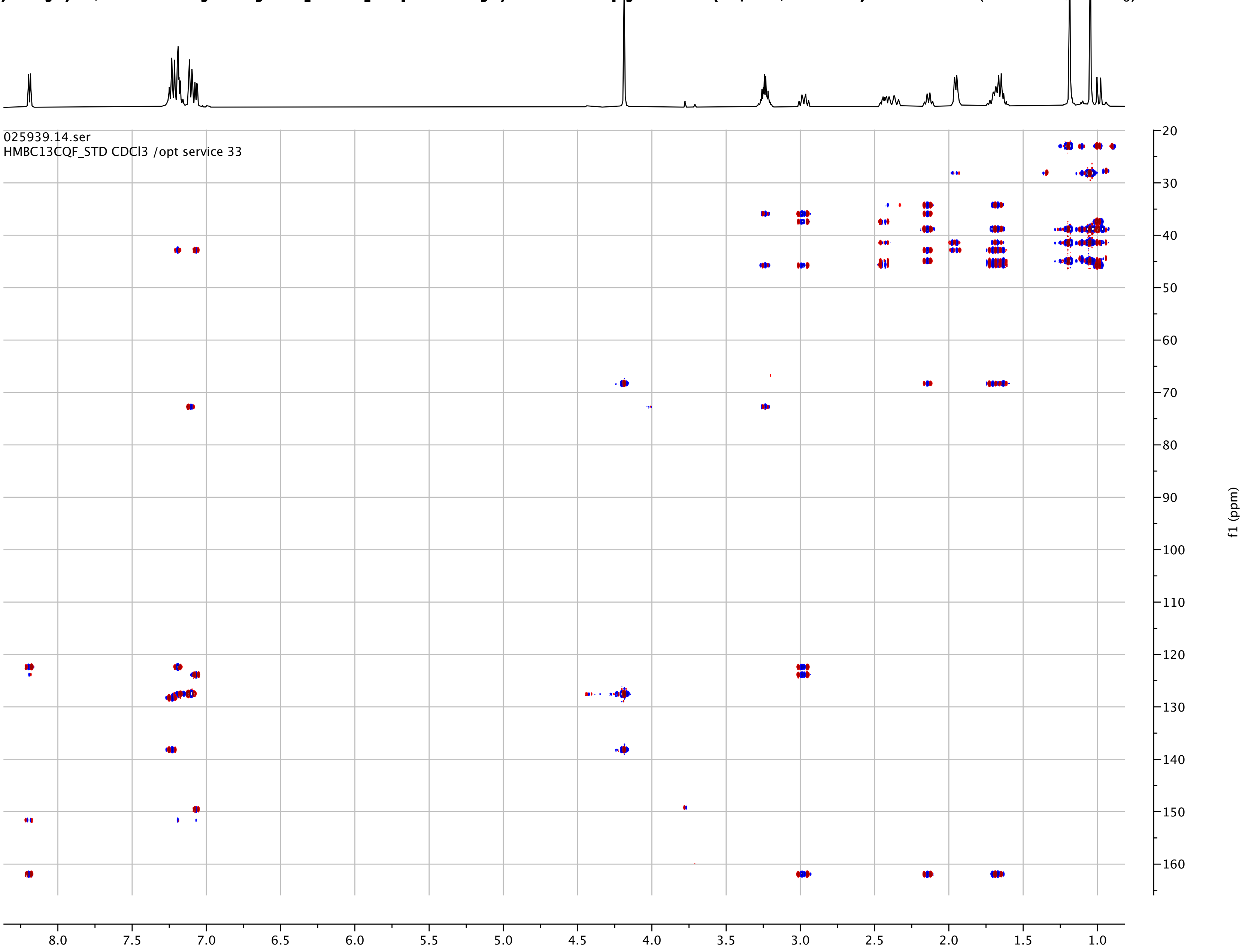

4-(2-(2-(Benzyloxy)ethyl)-6,6-dimethylbicyclo[3.1.1]heptan-3-yl)-2-chloropyridine (C_4-25, minor)

HMBC (400 MHz, $CDCl_3$)

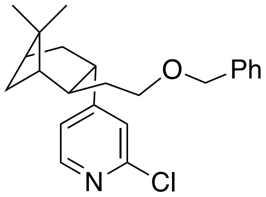

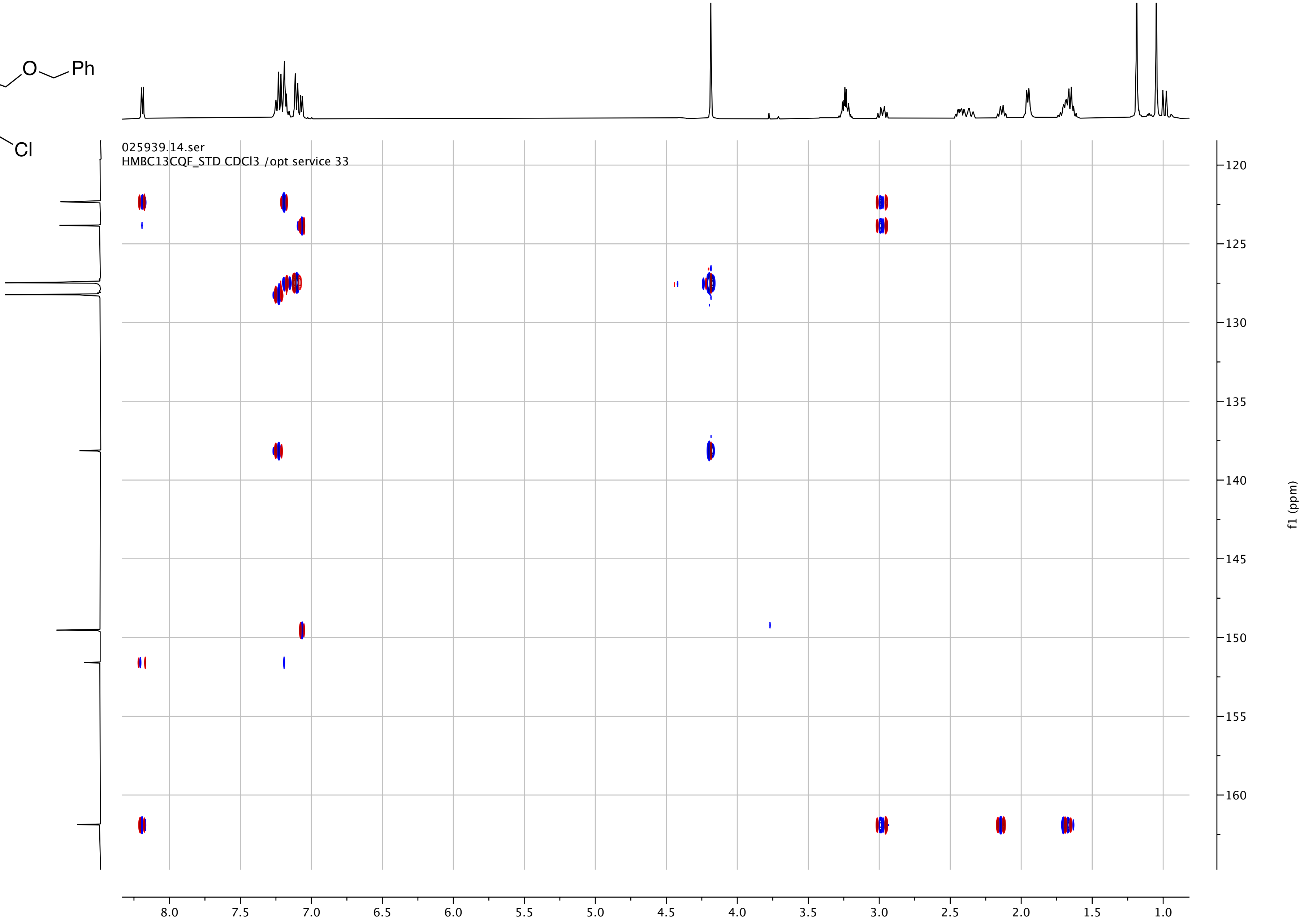

4-(2-(2-(Benzyloxy)ethyl)-6,6-dimethylbicyclo[3.1.1]heptan-3-yl)-2-chloropyridine (C_4-25, minor) HMBC (400 MHz, $CDCl_3$)

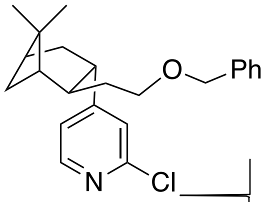

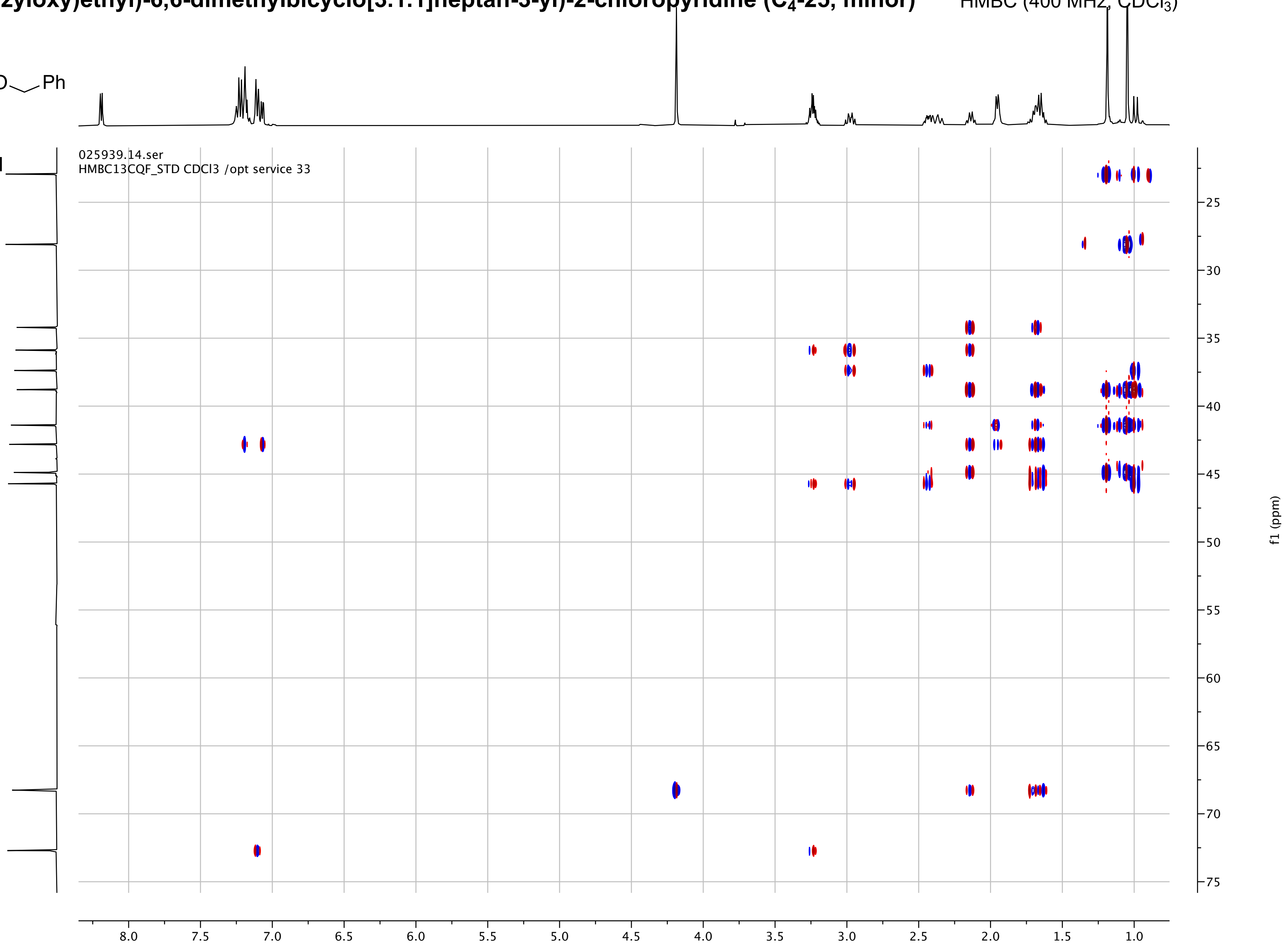

4-(2-(2-(Benzyloxy)ethyl)-6,6-dimethylbicyclo[3.1.1]heptan-3-yl)-2-chloropyridine (C_4-25, minor) NOESY (400 MHz, $CDCl_3$)

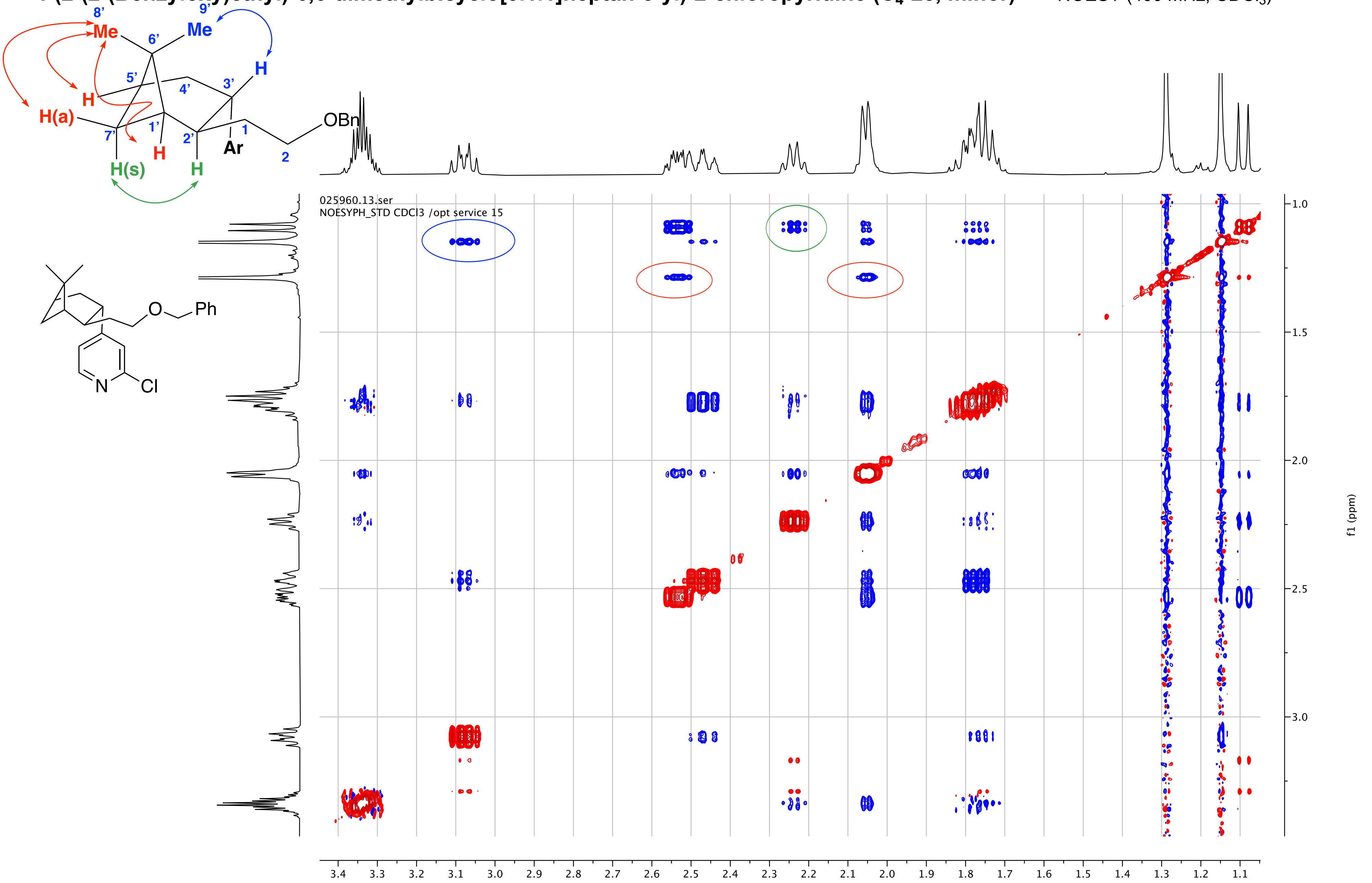

2-(2-(2-(Benzyloxy)ethyl)-6,6-dimethylbicyclo[3.1.1]heptan-3-yl)-6-(trifluoromethyl)pyridine (26)

^{1}H-NMR (400 MHz, $CDCl_3$)

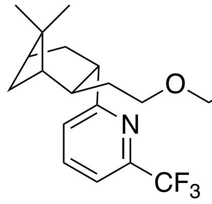

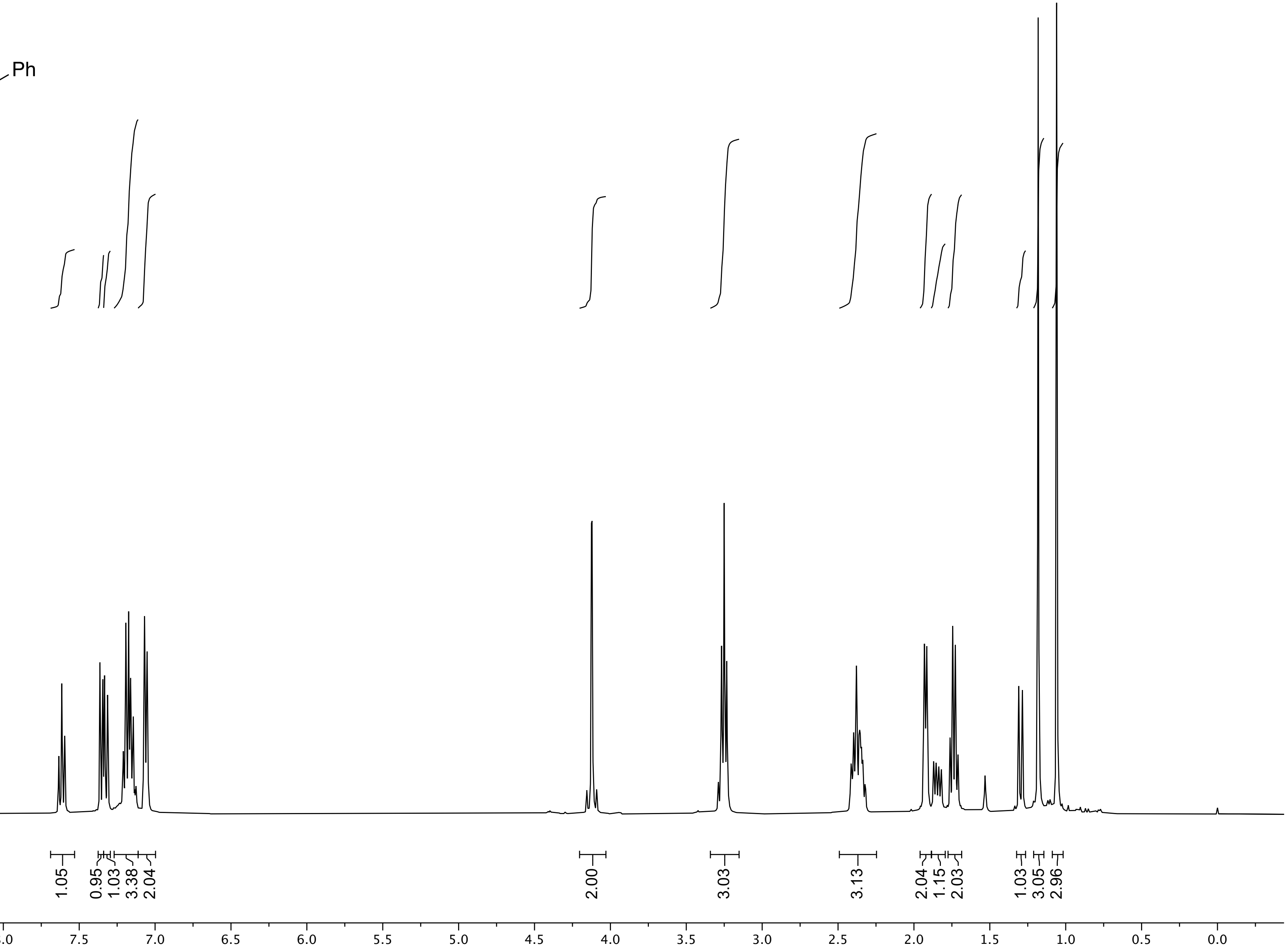

2-(2-(2-(Benzyloxy)ethyl)-6,6-dimethylbicyclo[3.1.1]heptan-3-yl)-6-(trifluoromethyl)pyridine (26) ^{1}H-NMR (400 MHz, $CDCl_3$)

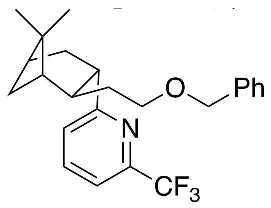

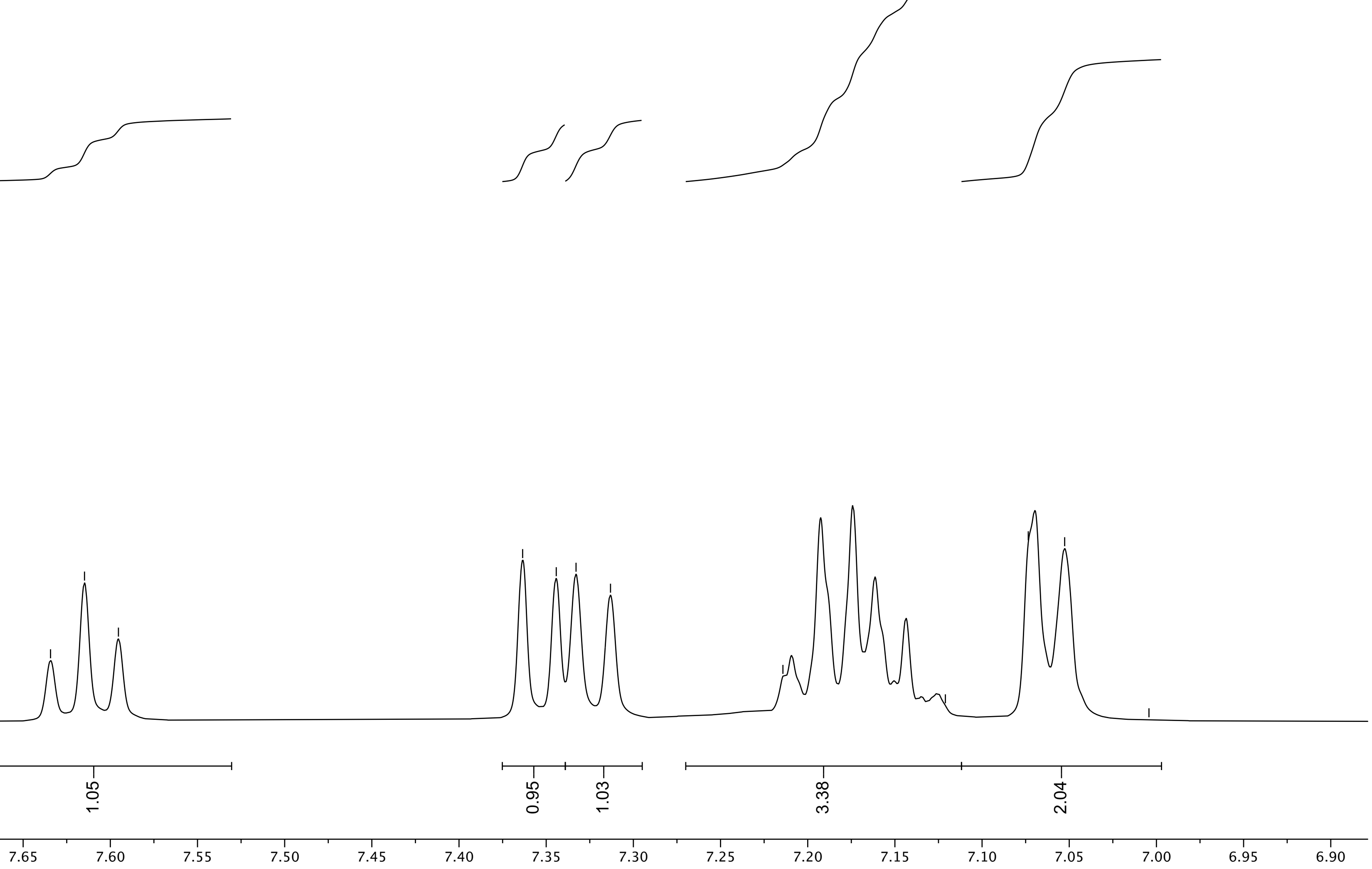

2-(2-(2-(Benzyloxy)ethyl)-6,6-dimethylbicyclo[3.1.1]heptan-3-yl)-6-(trifluoromethyl)pyridine (26) ^{1}H-NMR (400 MHz, $CDCl_3$)

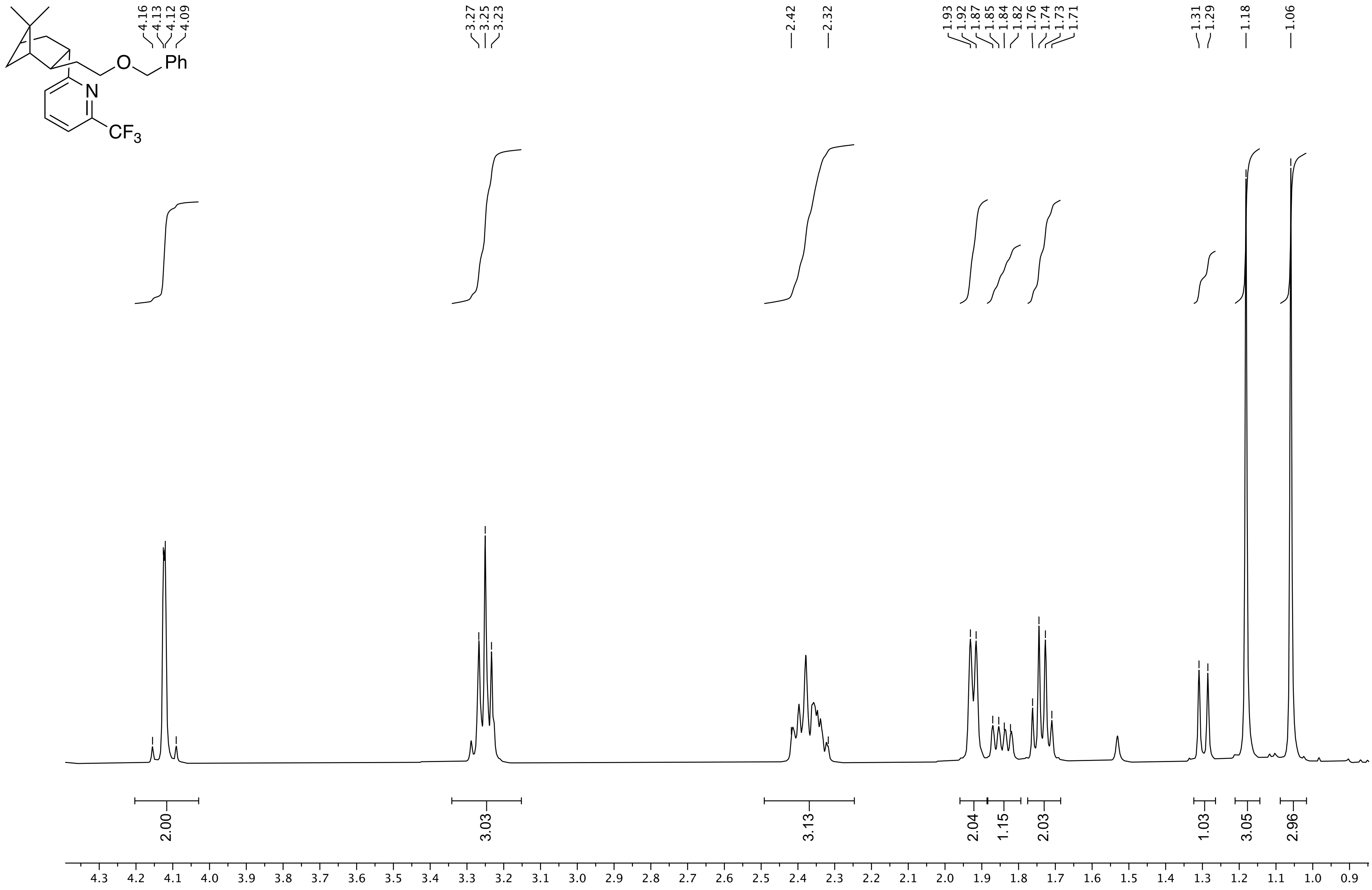

2-(2-(2-(Benzyloxy)ethyl)-6,6-dimethylbicyclo[3.1.1]heptan-3-yl)-6-(trifluoromethyl)pyridine (26) 13C-NMR (101 MHz, $CDCl_3$)

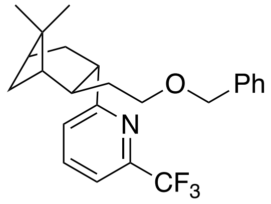

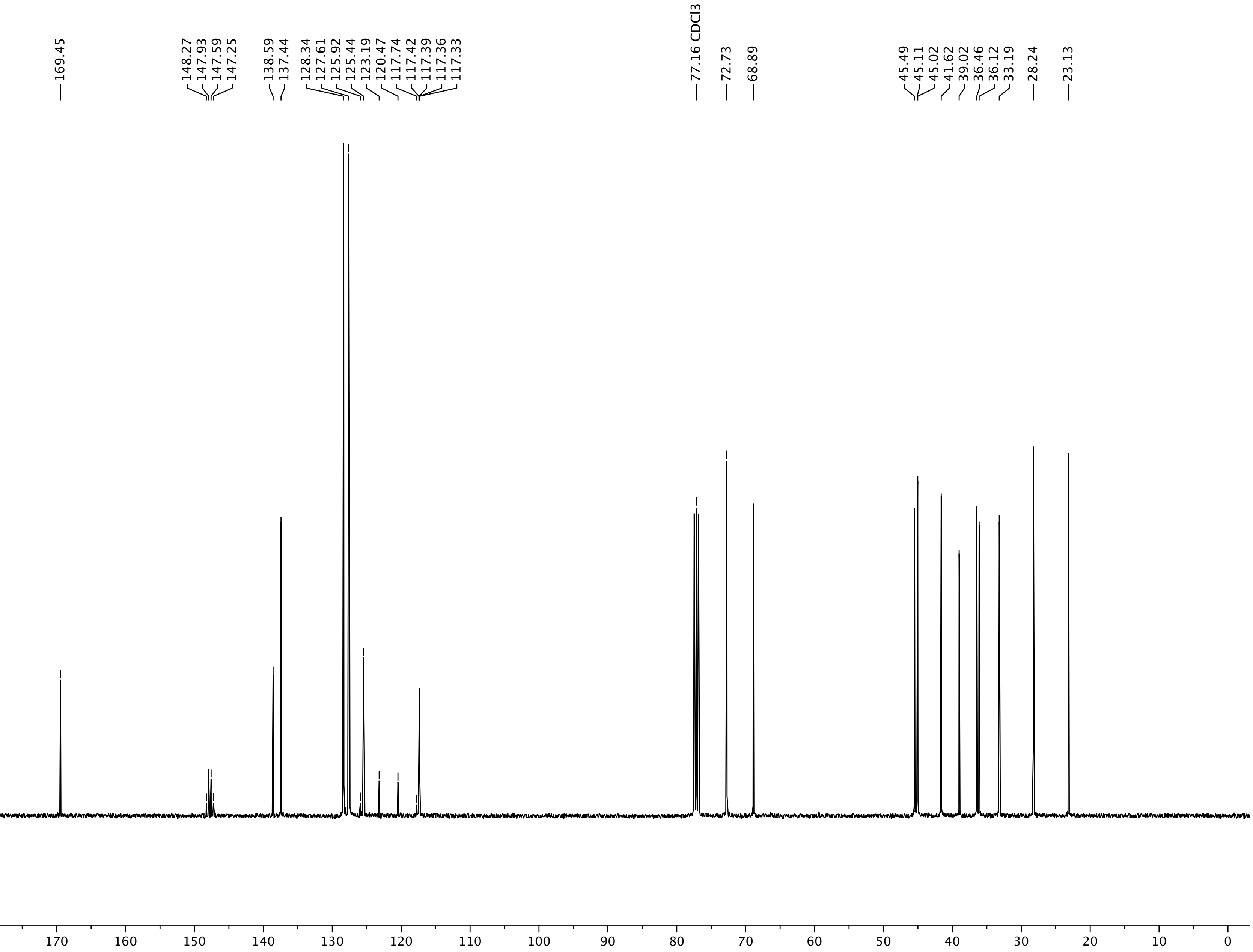

2-(2-(2-(Benzyloxy)ethyl)-6,6-dimethylbicyclo[3.1.1]heptan-3-yl)-6-(trifluoromethyl)pyridine (26) 13C-NMR (101 MHz, $CDCl_3$)

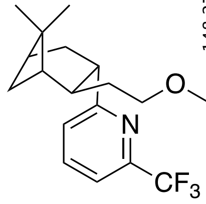

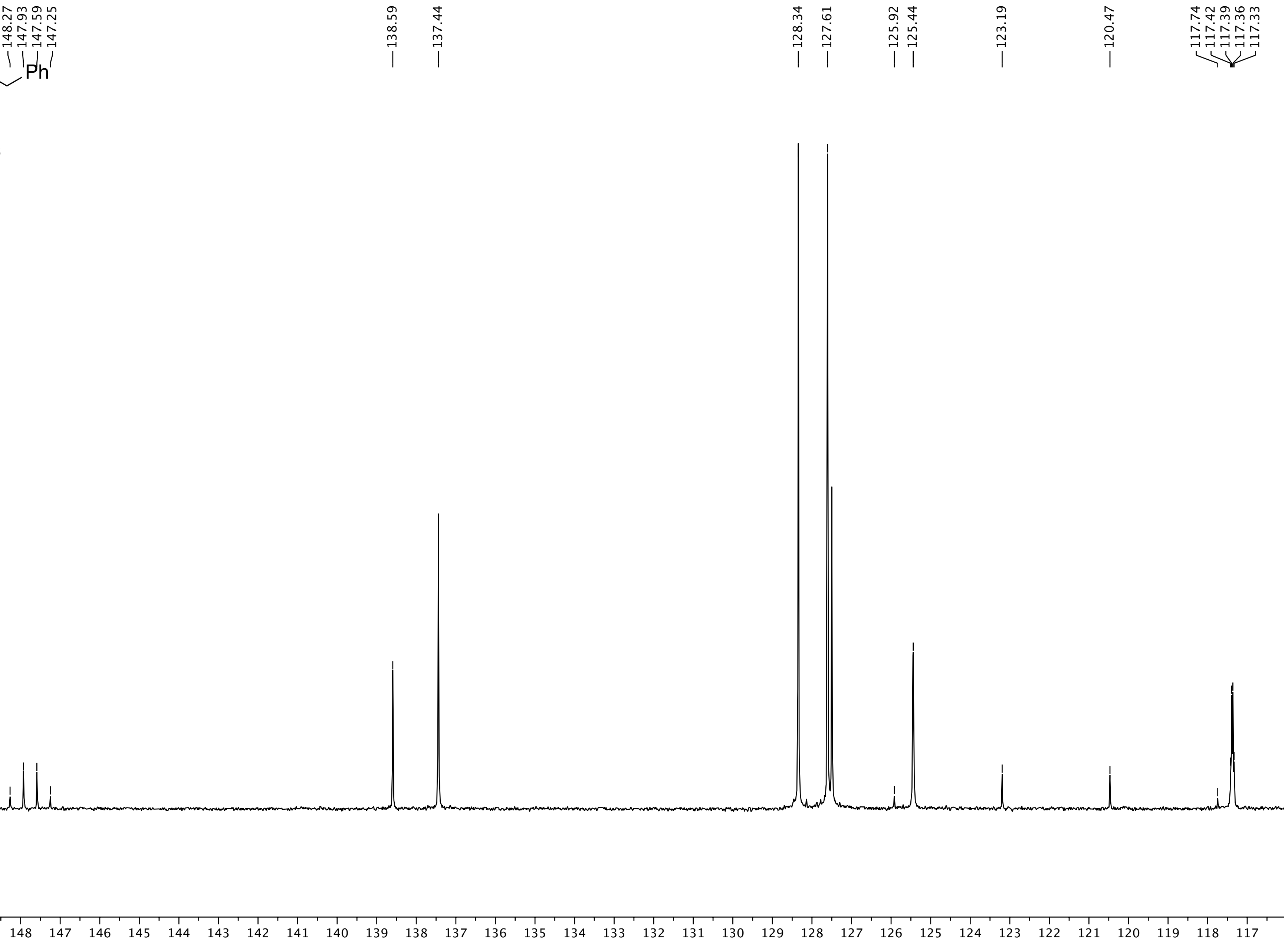

2-(2-(2-(Benzyloxy)ethyl)-6,6-dimethylbicyclo[3.1.1]heptan-3-yl)-6-(trifluoromethyl)pyridine (26) 13C-NMR (101 MHz, $CDCl_3$)

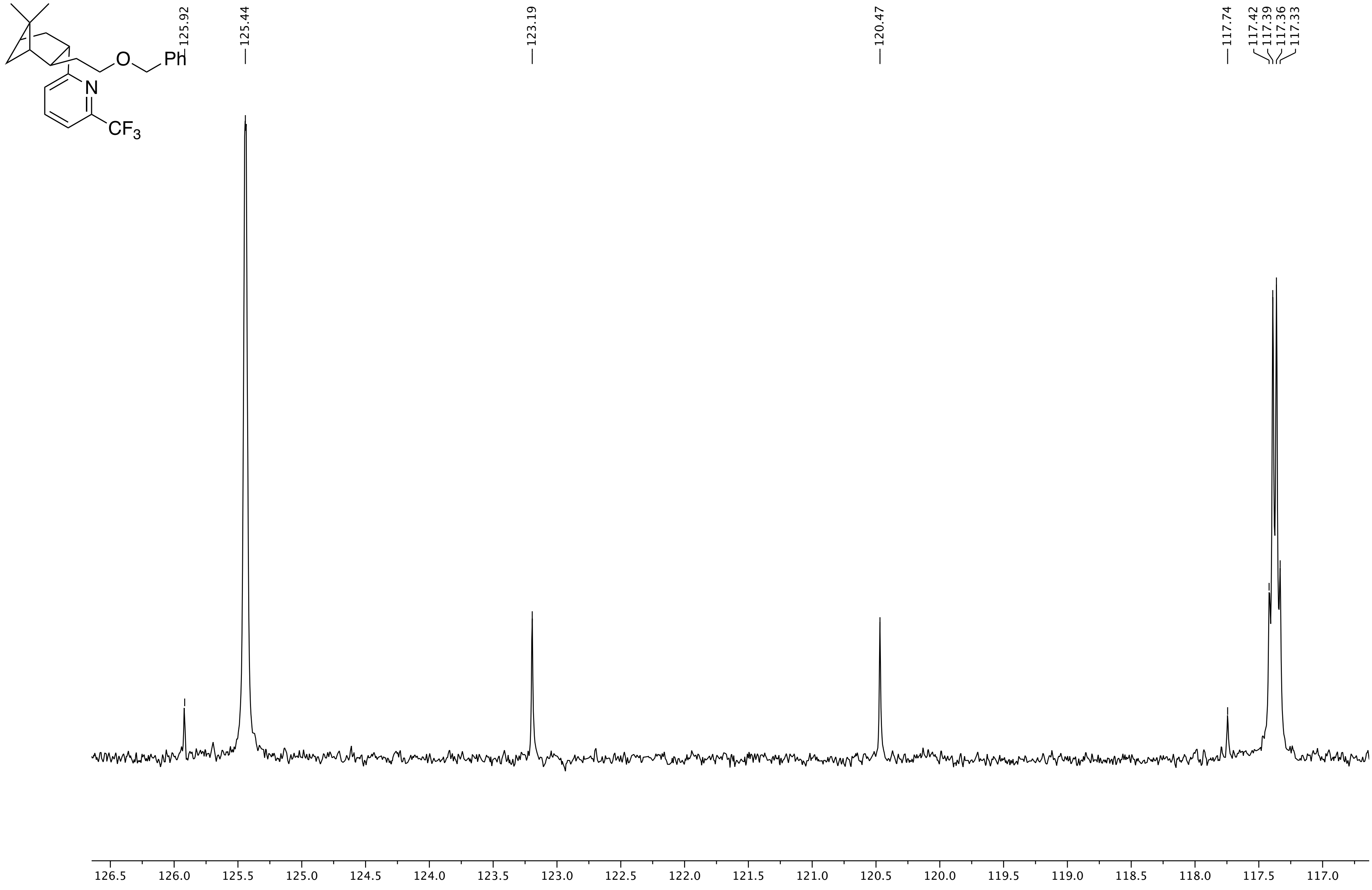

2-(2-(2-(Benzyloxy)ethyl)-6,6-dimethylbicyclo[3.1.1]heptan-3-yl)-6-(trifluoromethyl)pyridine (26) 13C-NMR (101 MHz, $CDCl_3$)

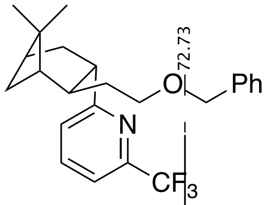

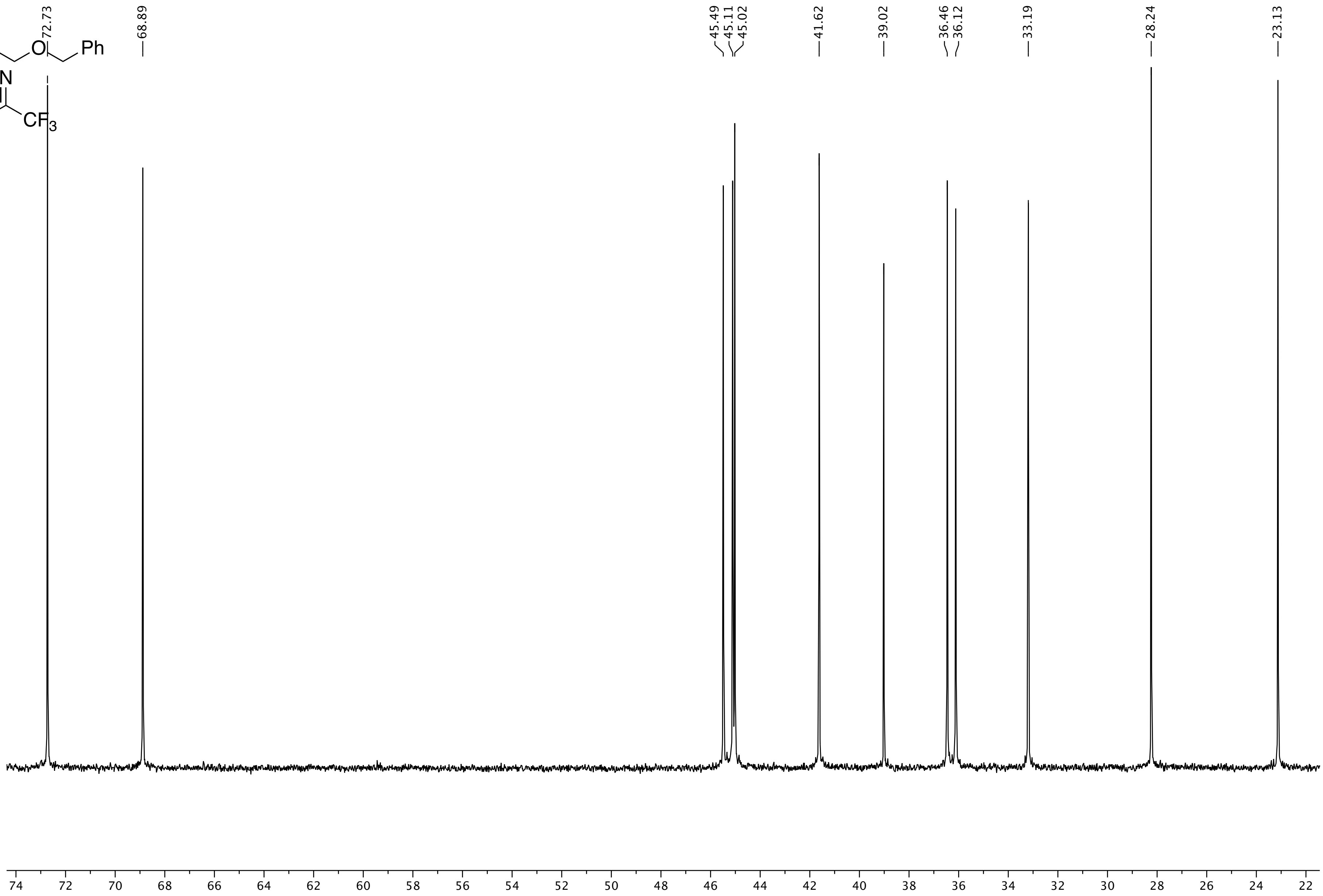

2-(2-(2-(Benzyloxy)ethyl)-6,6-dimethylbicyclo[3.1.1]heptan-3-yl)-6-(trifluoromethyl)pyridine

DEPT135 (101 MHz, $CDCl_3$)

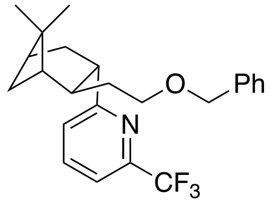

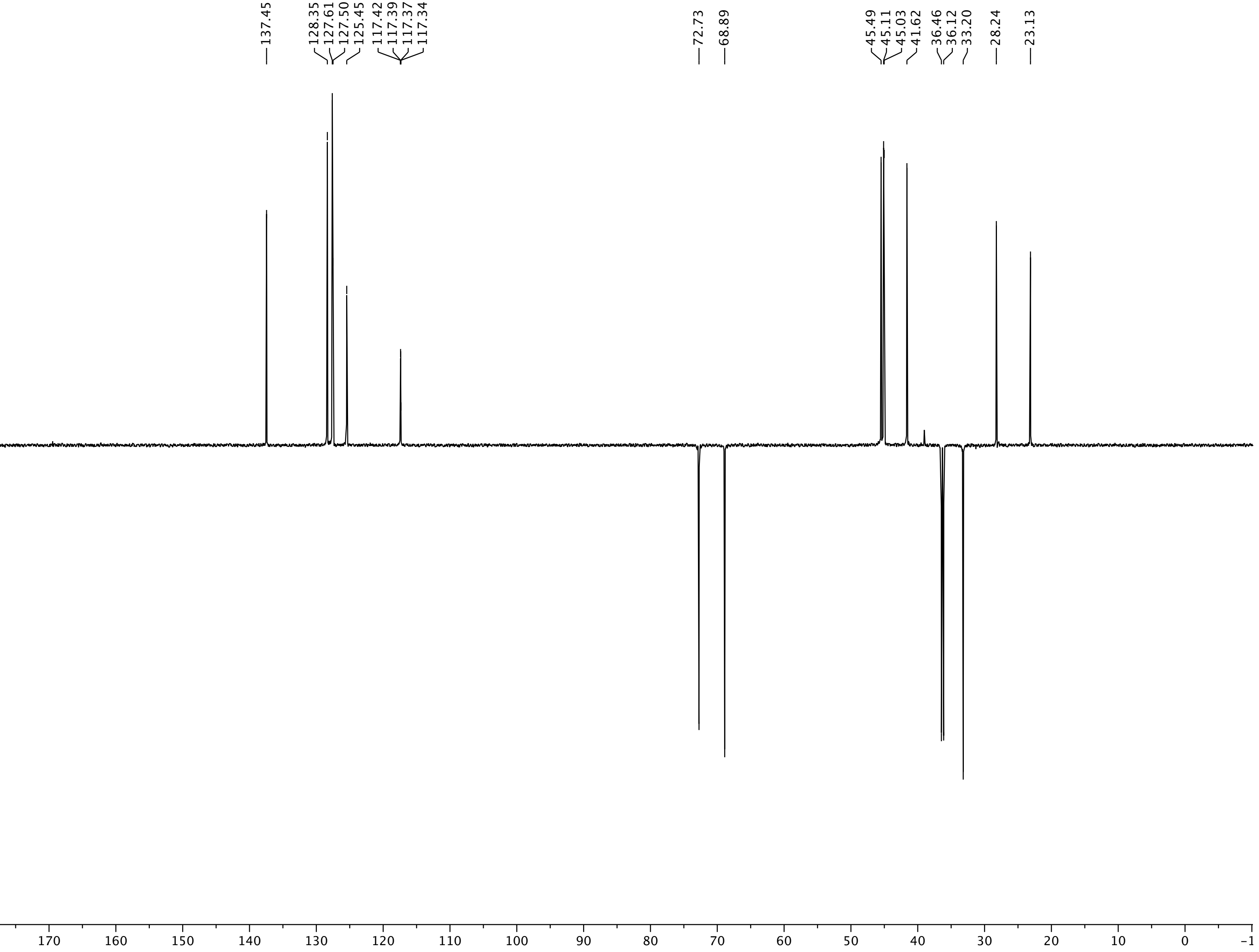

2-(2-(2-(Benzyloxy)ethyl)-6,6-dimethylbicyclo[3.1.1]heptan-3-yl)-6-(trifluoromethyl)pyridine (26) ^{1}H-^{1}H COSY (400 MHz, $CDCl_3$)

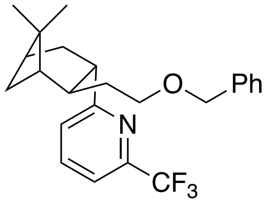

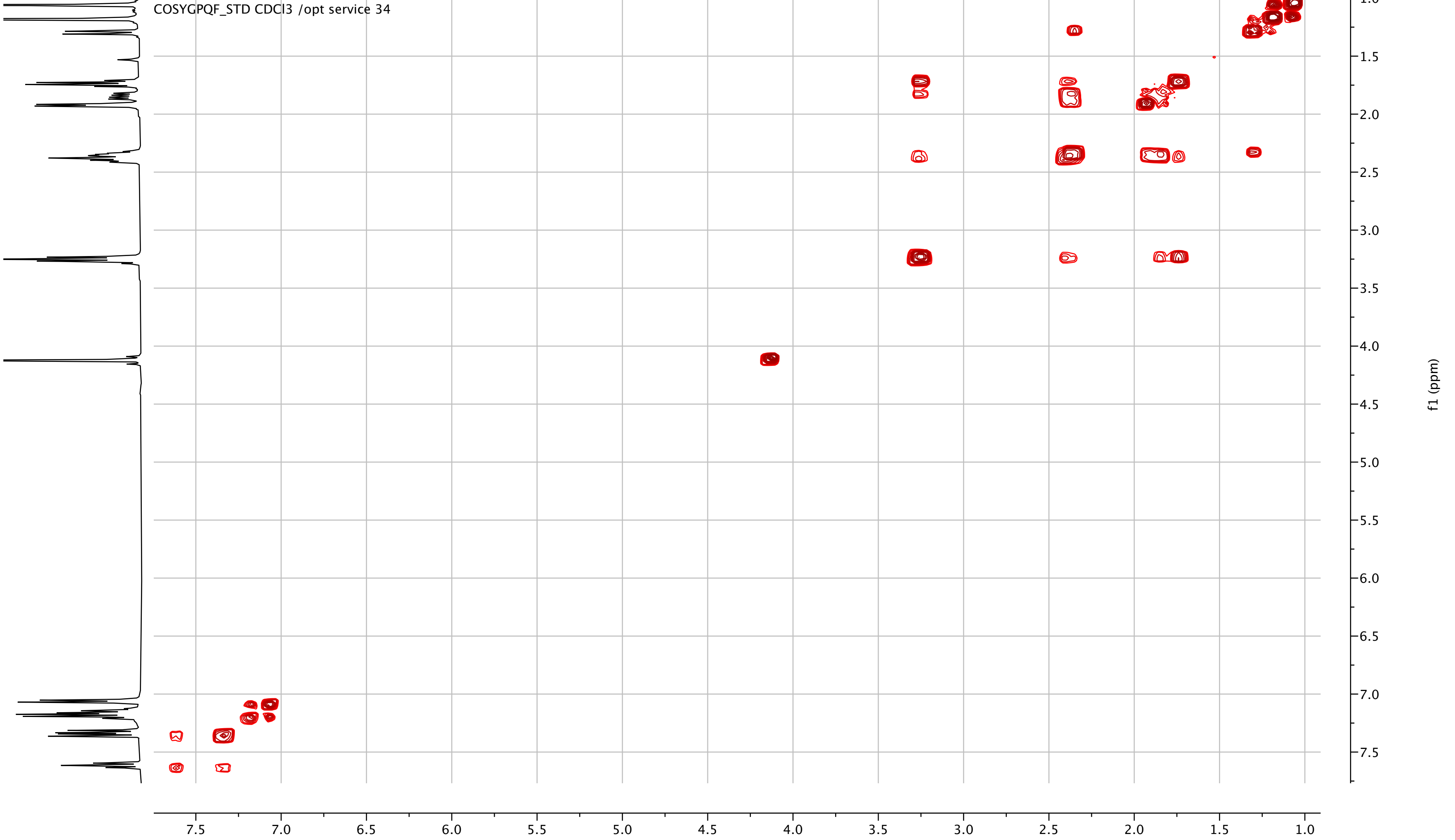

2-(2-(2-(Benzyloxy)ethyl)-6,6-dimethylbicyclo[3.1.1]heptan-3-yl)-6-(trifluoromethyl)pyridine (26)

^{1}H-^{1}H COSY (400 MHz, $CDCl_3$)

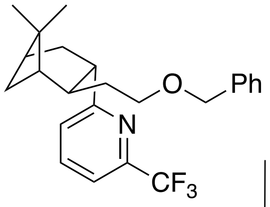

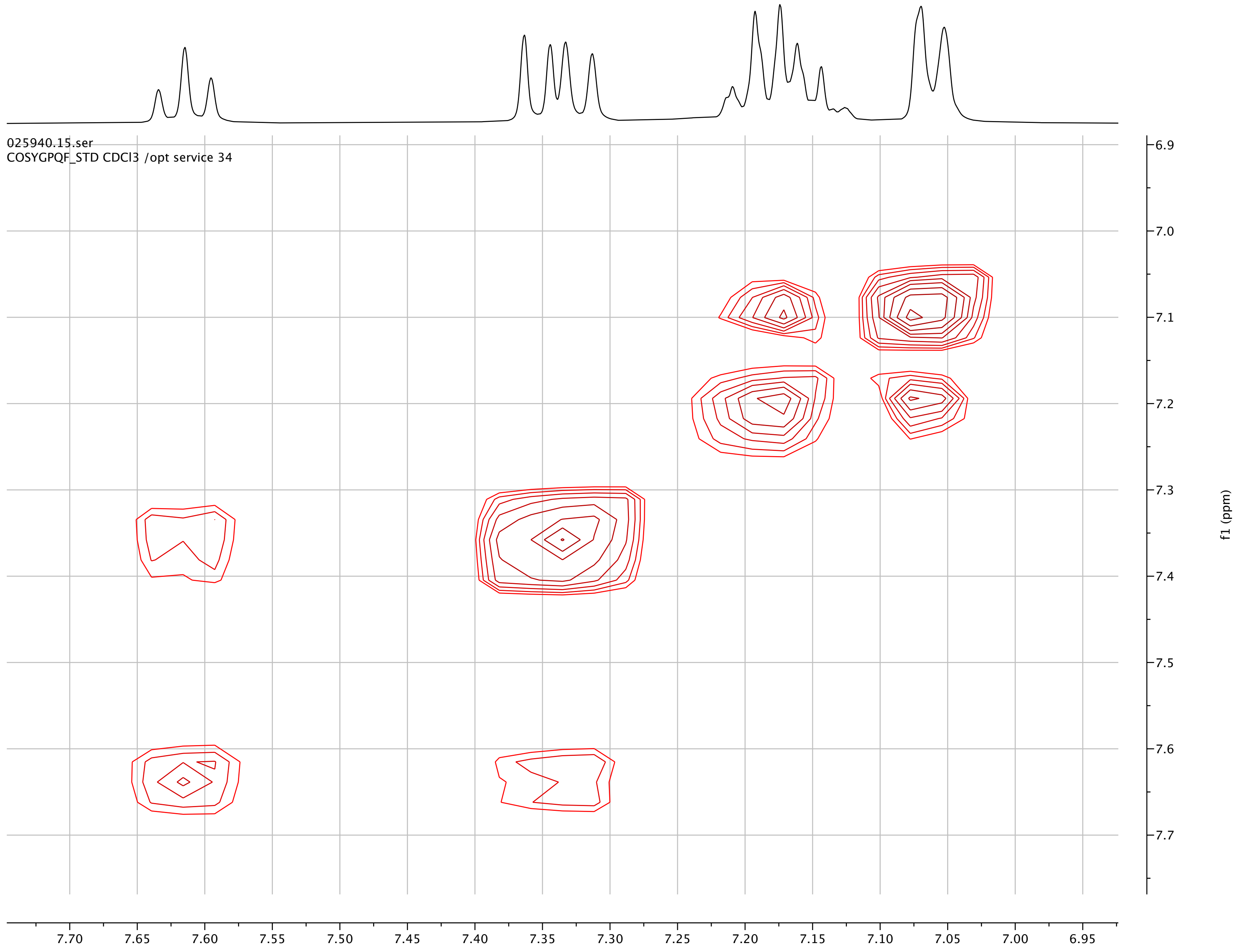

2-(2-(2-(Benzyloxy)ethyl)-6,6-dimethylbicyclo[3.1.1]heptan-3-yl)-6-(trifluoromethyl)pyridine (26)

^{1}H-^{1}H COSY (400 MHz, $CDCl_3$)

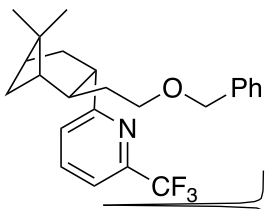

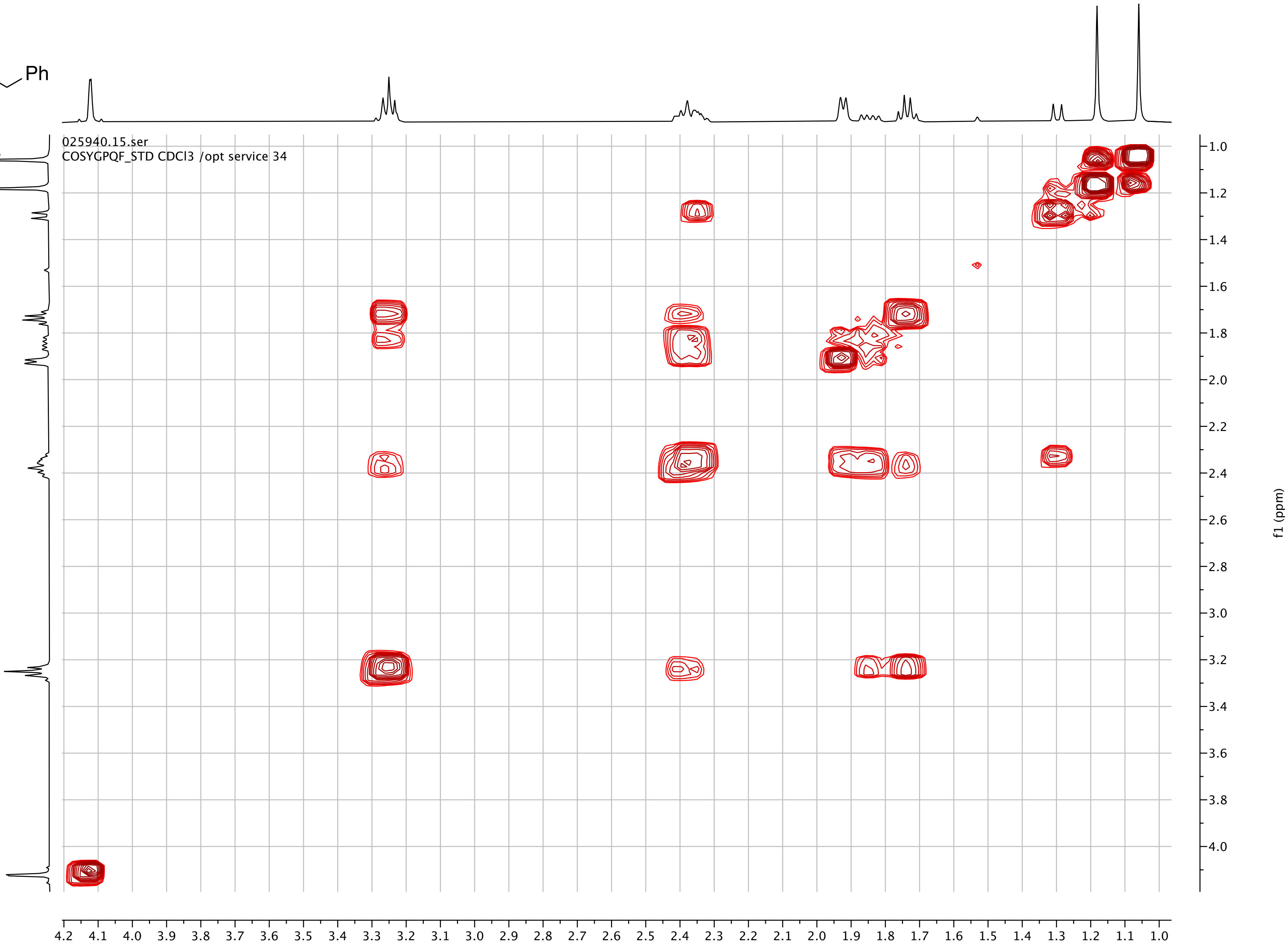

2-(2-(2-(Benzyloxy)ethyl)-6,6-dimethylbicyclo[3.1.1]heptan-3-yl)-6-(trifluoromethyl)pyridine (26) HSQC (101 MHz, $CDCl_3$)

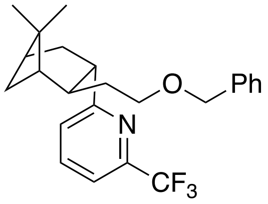

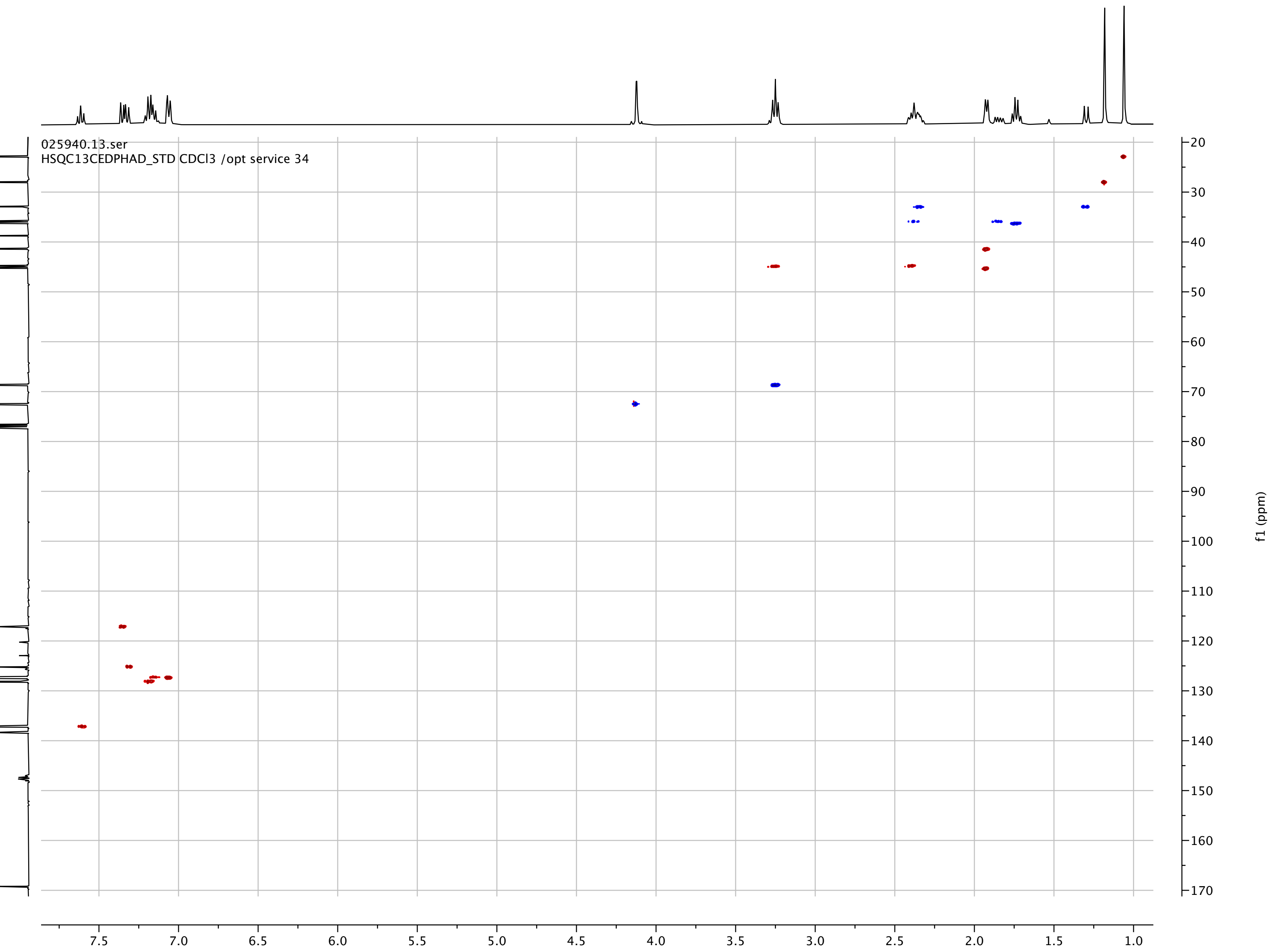

2-(2-(2-(Benzyloxy)ethyl)-6,6-dimethylbicyclo[3.1.1]heptan-3-yl)-6-(trifluoromethyl)pyridine (26) HSQC (101 MHz, $CDCl_3$)

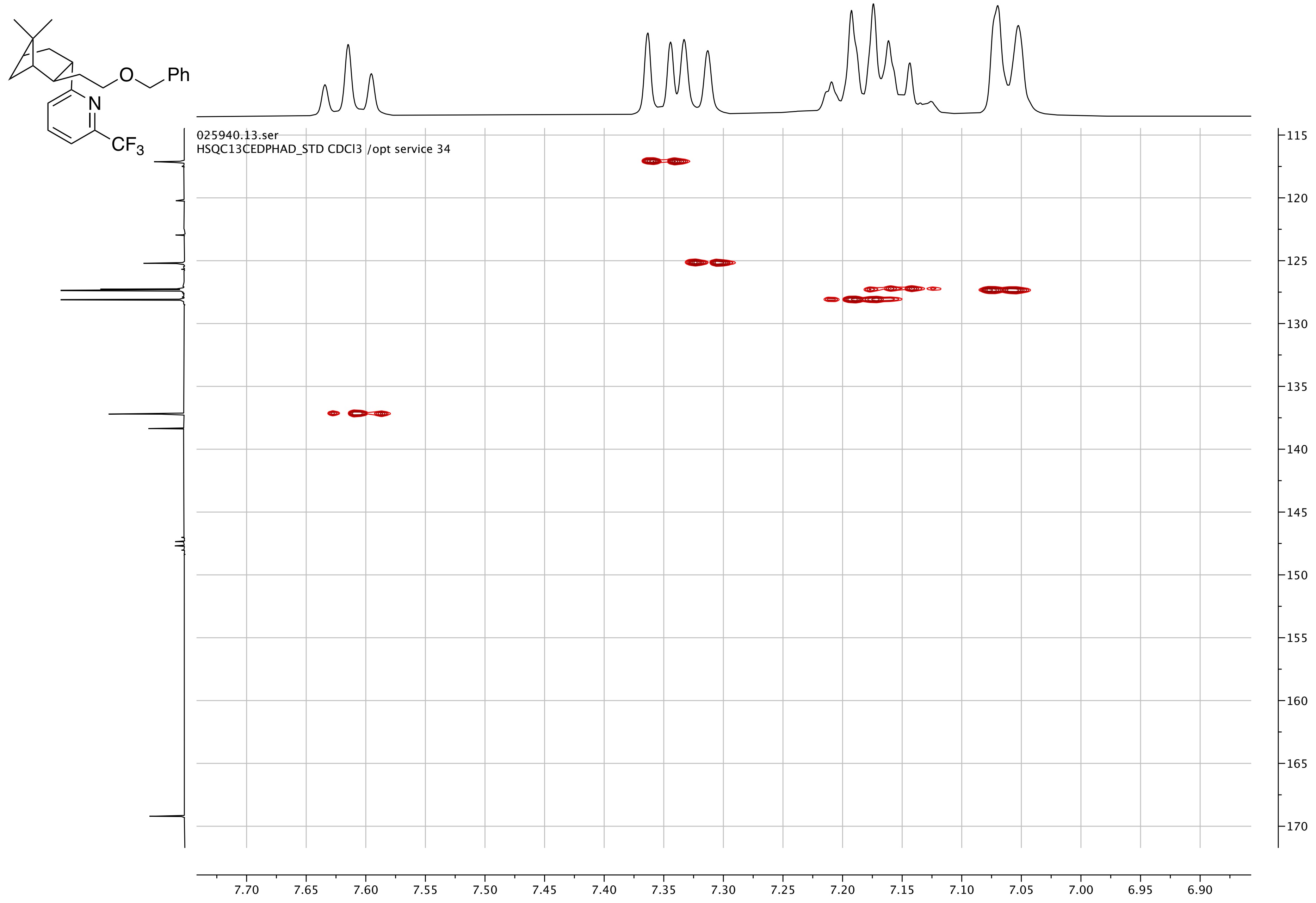

2-(2-(2-(Benzyloxy)ethyl)-6,6-dimethylbicyclo[3.1.1]heptan-3-yl)-6-(trifluoromethyl)pyridine (26)

HSQC (101 MHz, $CDCl_3$)

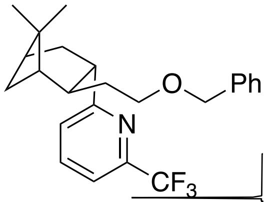

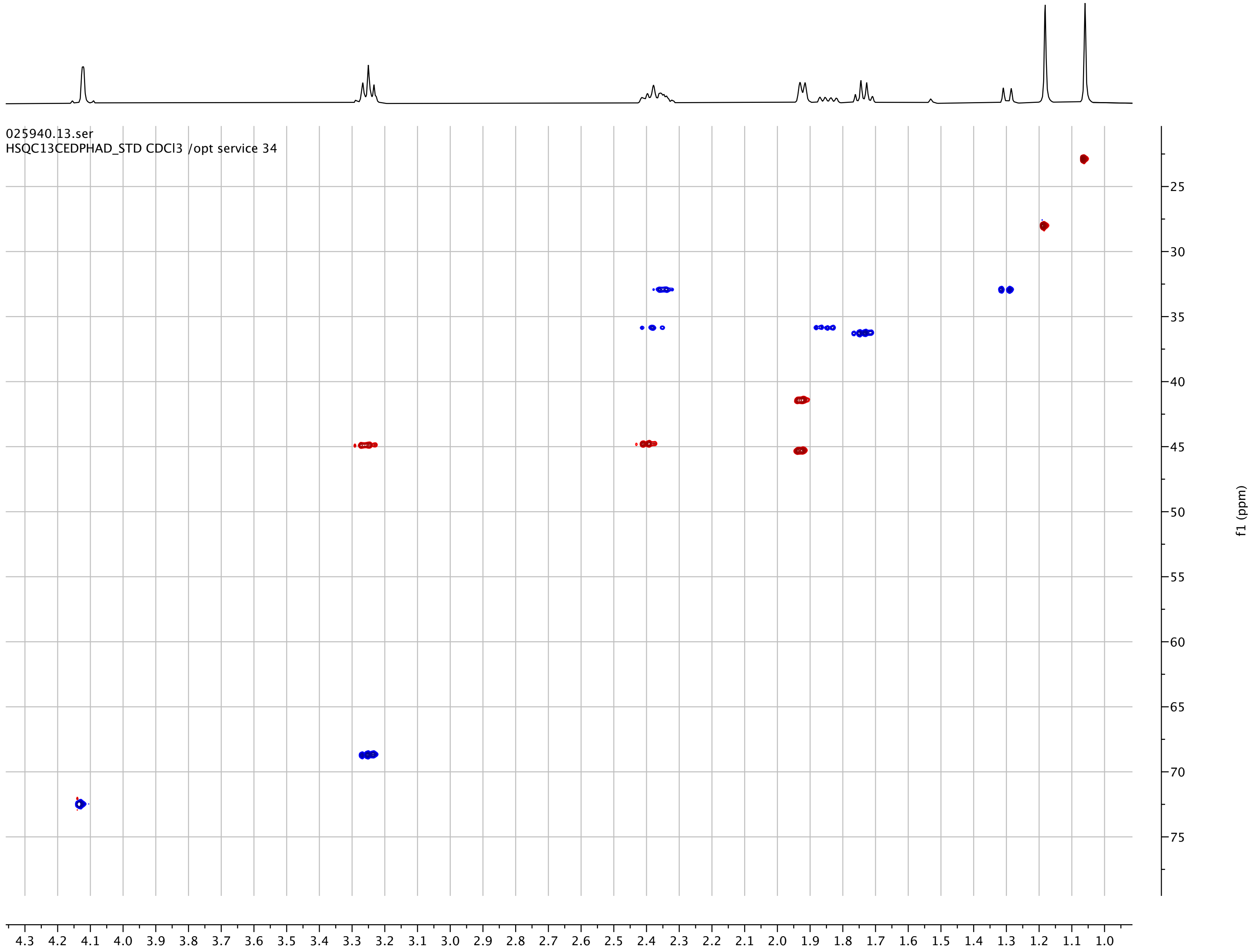

2-(2-(2-(Benzyloxy)ethyl)-6,6-dimethylbicyclo[3.1.1]heptan-3-yl)-6-(trifluoromethyl)pyridine (26) HMBC (101 MHz, $CDCl_3$)

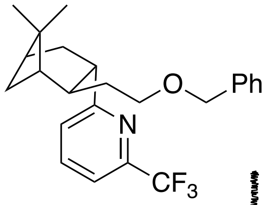

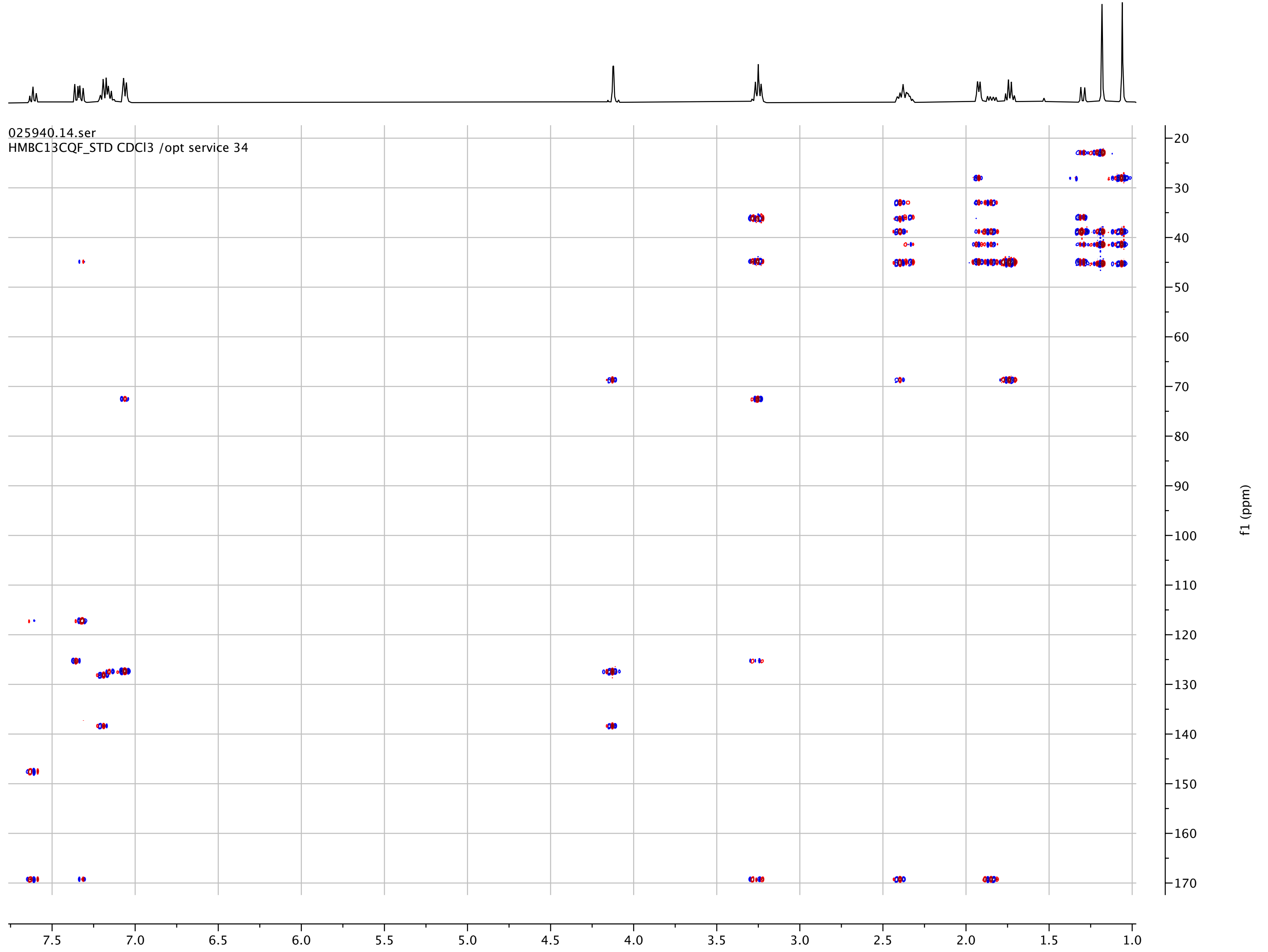

S339

2-(2-(2-(Benzyloxy)ethyl)-6,6-dimethylbicyclo[3.1.1]heptan-3-yl)-6-(trifluoromethyl)pyridine (26) HMBC (101 MHz, $CDCl_3$)

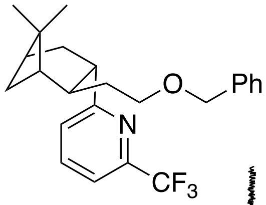

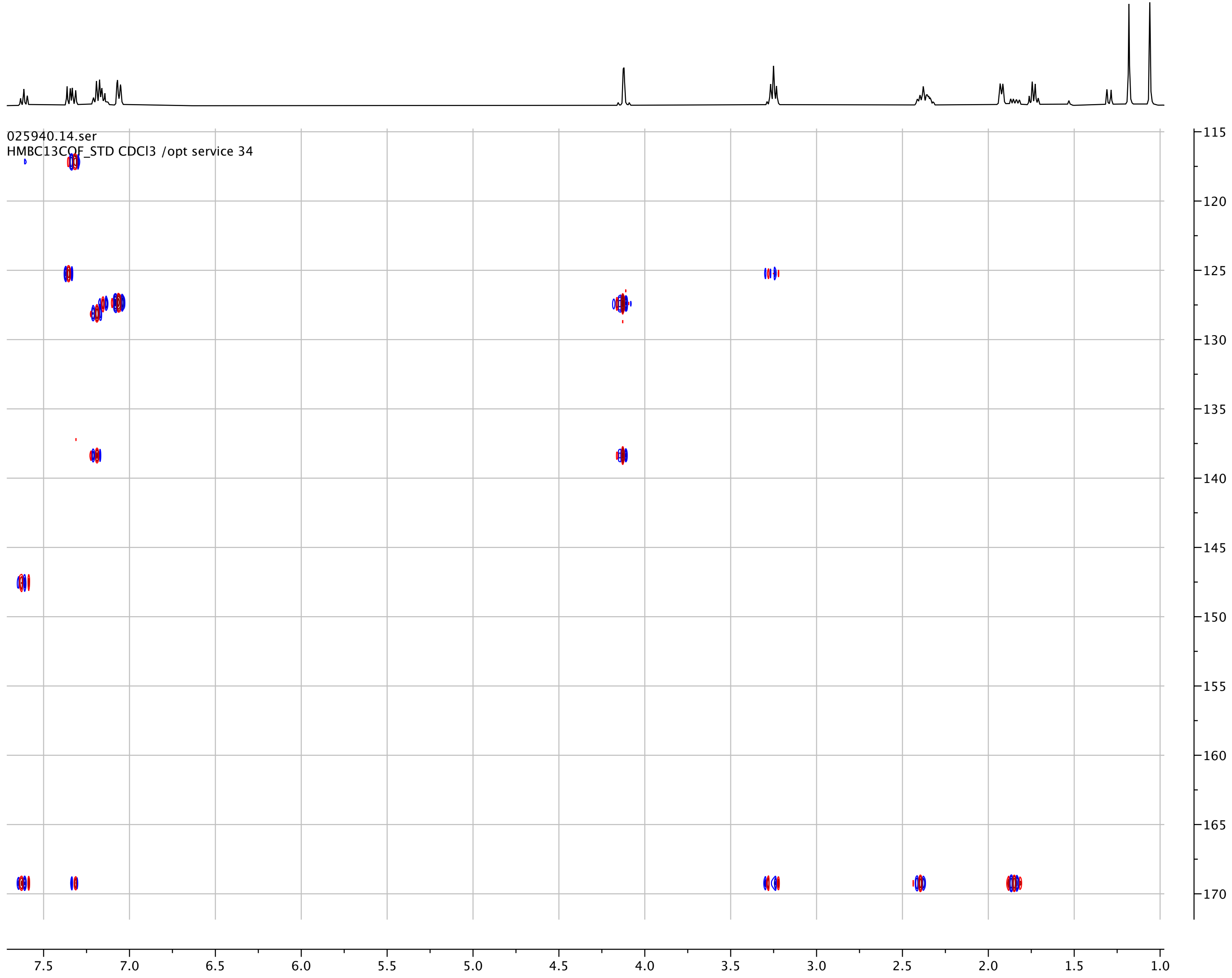

2-(2-(2-(Benzyloxy)ethyl)-6,6-dimethylbicyclo[3.1.1]heptan-3-yl)-6-(trifluoromethyl)pyridine (26) HMBC (101 MHz, $CDCl_3$)

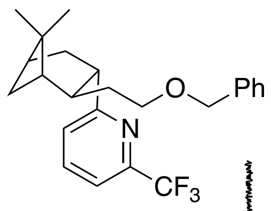

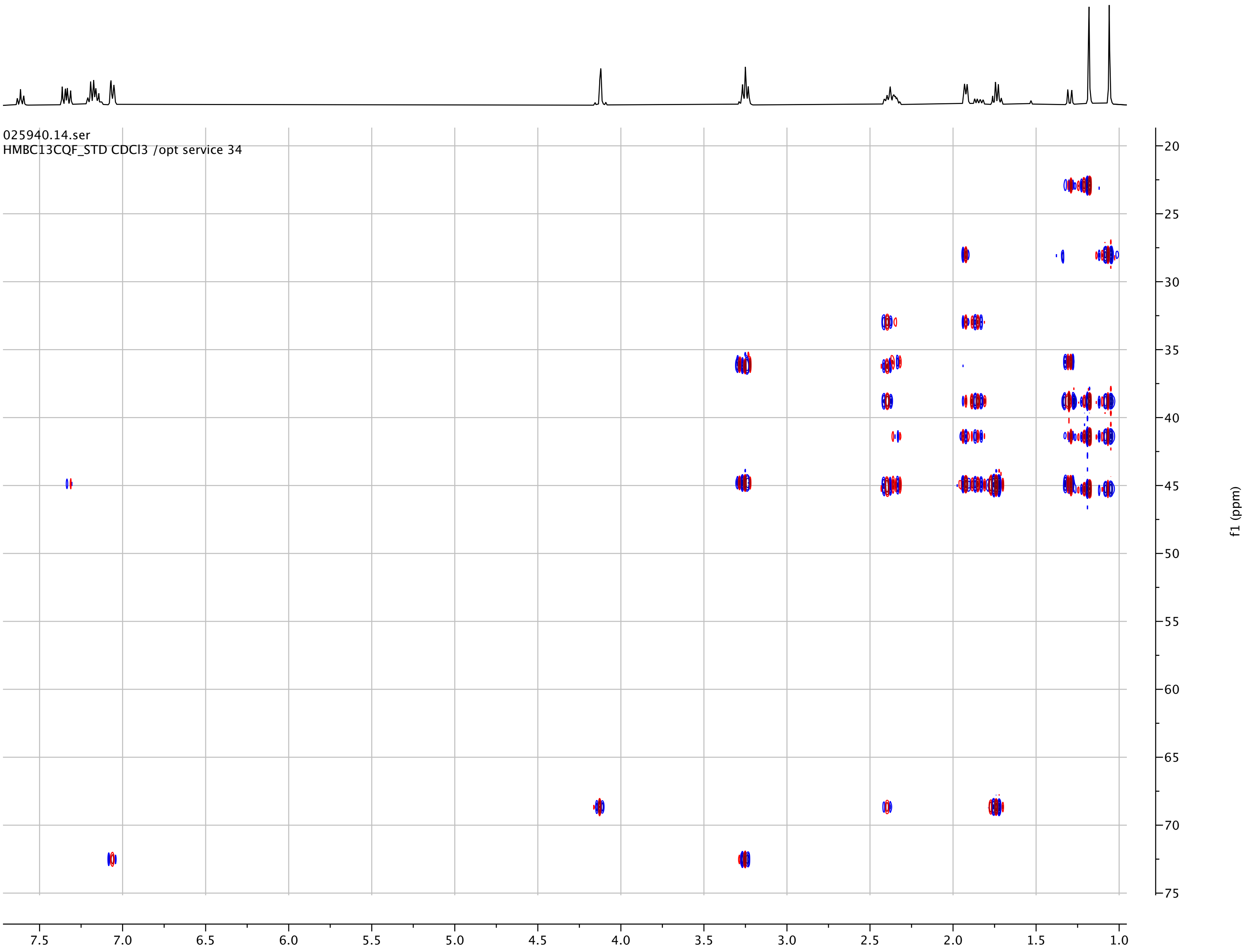

2-(2-(2-(Benzyloxy)ethyl)-6,6-dimethylbicyclo[3.1.1]heptan-3-yl)-6-(trifluoromethyl)pyridine (26) ^{19}F (376 MHz, $CDCl_3$)

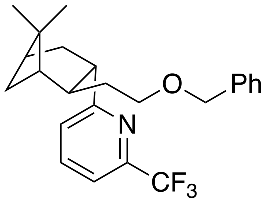

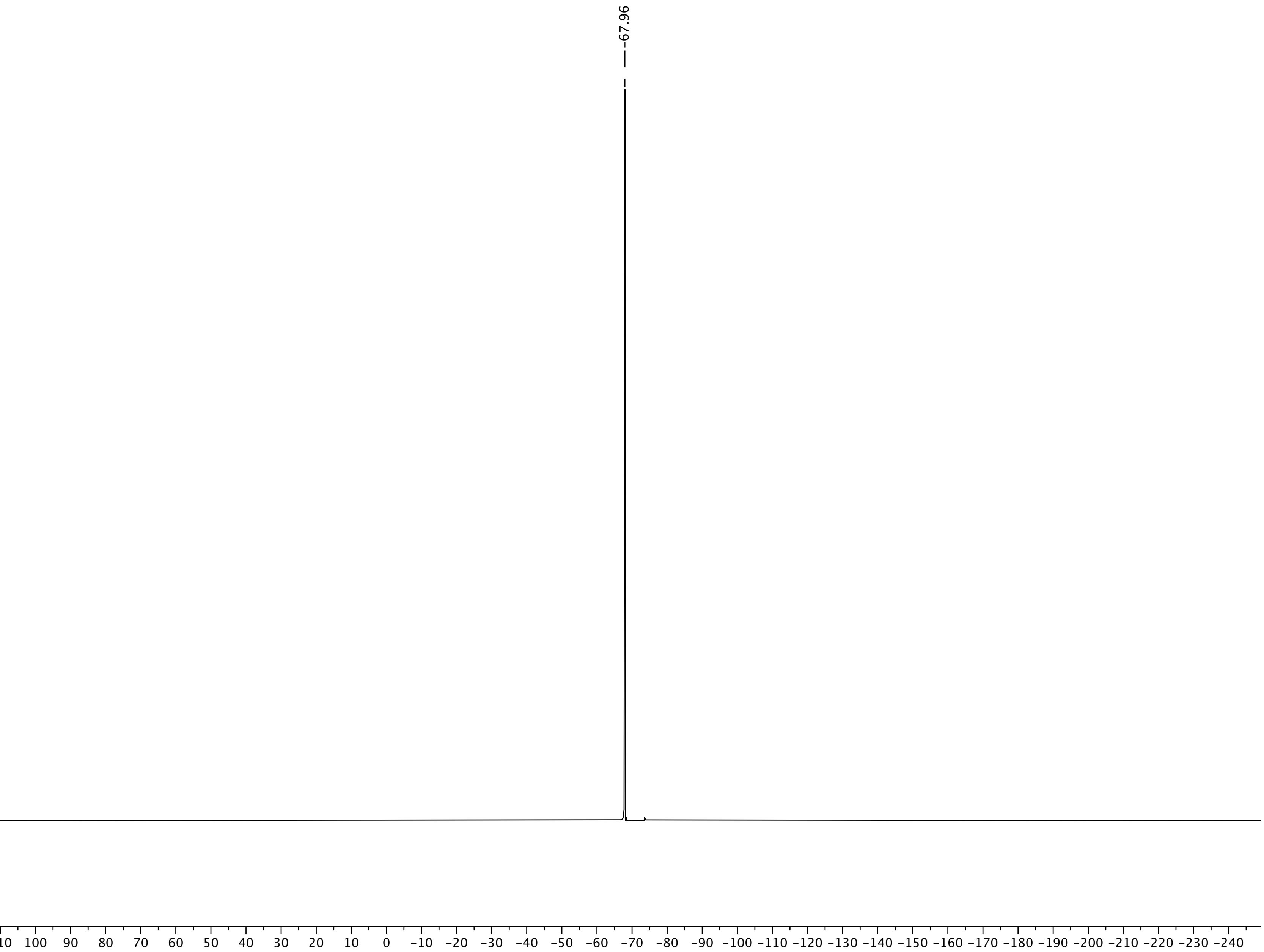

4-(2-(2-(Benzyloxy)ethyl)-6,6-dimethylbicyclo[3.1.1]heptan-3-yl)-2-(trifluoromethyl)pyridine (C_4-26) ^{1}H-NMR (400 MHz, $CDCl_3$)

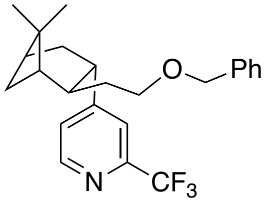

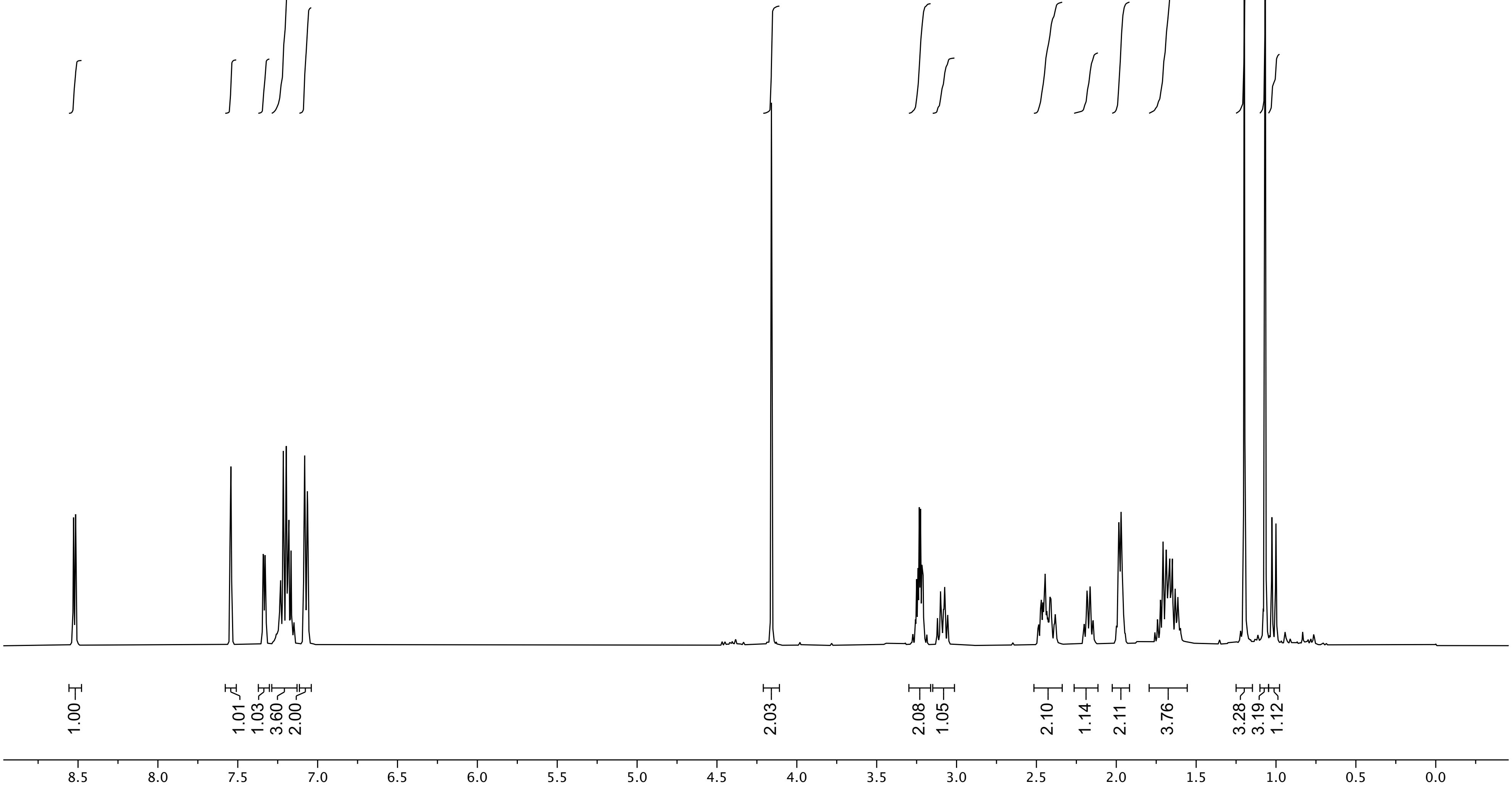

4-(2-(2-(Benzyloxy)ethyl)-6,6-dimethylbicyclo[3.1.1]heptan-3-yl)-2-(trifluoromethyl)pyridine (C_4-26) ^{1}H-NMR (400 MHz, $CDCl_3$)

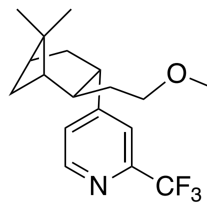

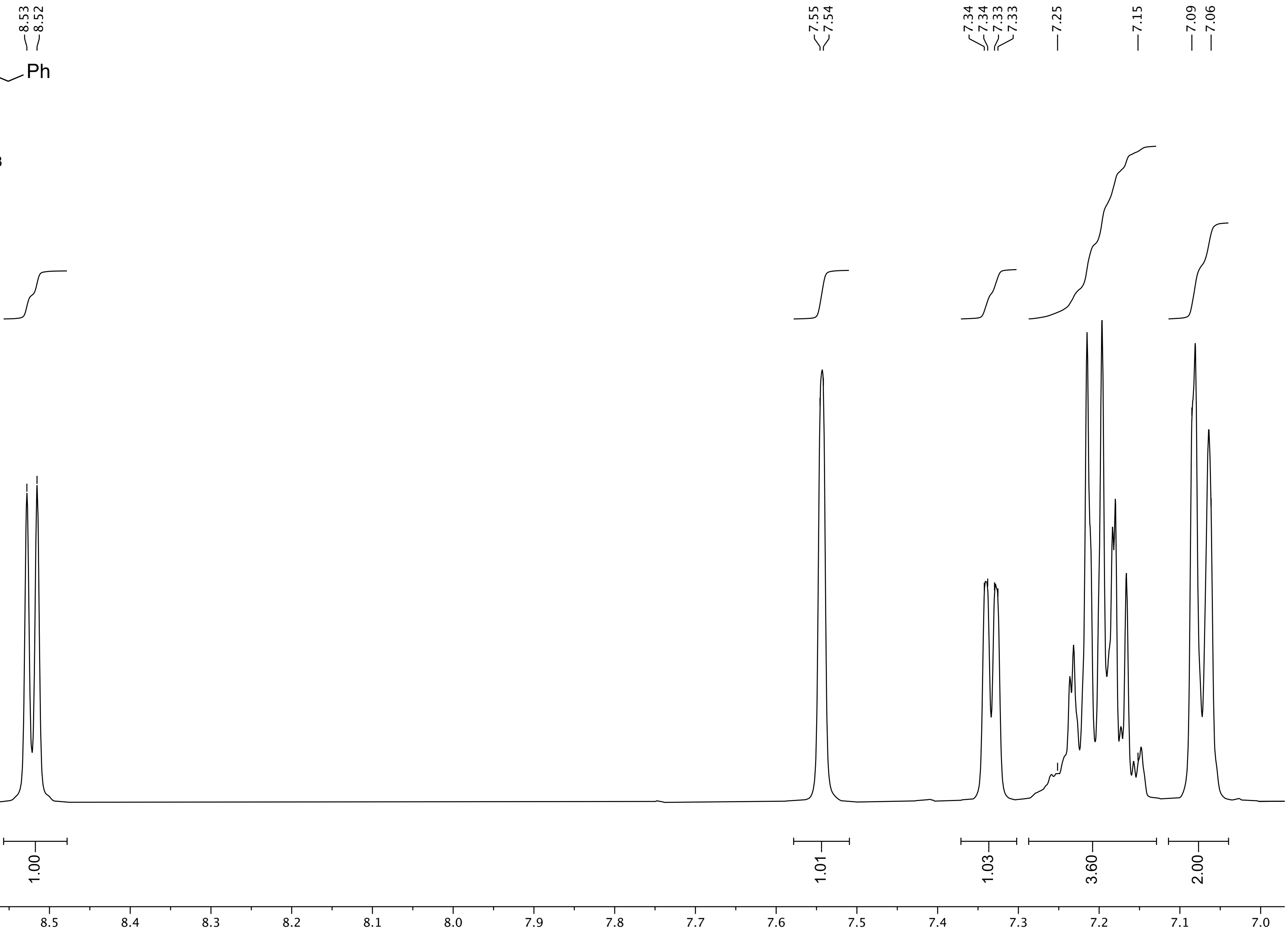

4-(2-(2-(Benzyloxy)ethyl)-6,6-dimethylbicyclo[3.1.1]heptan-3-yl)-2-(trifluoromethyl)pyridine (C_4-26) ^{1}H-NMR (400 MHz, $CDCl_3$)

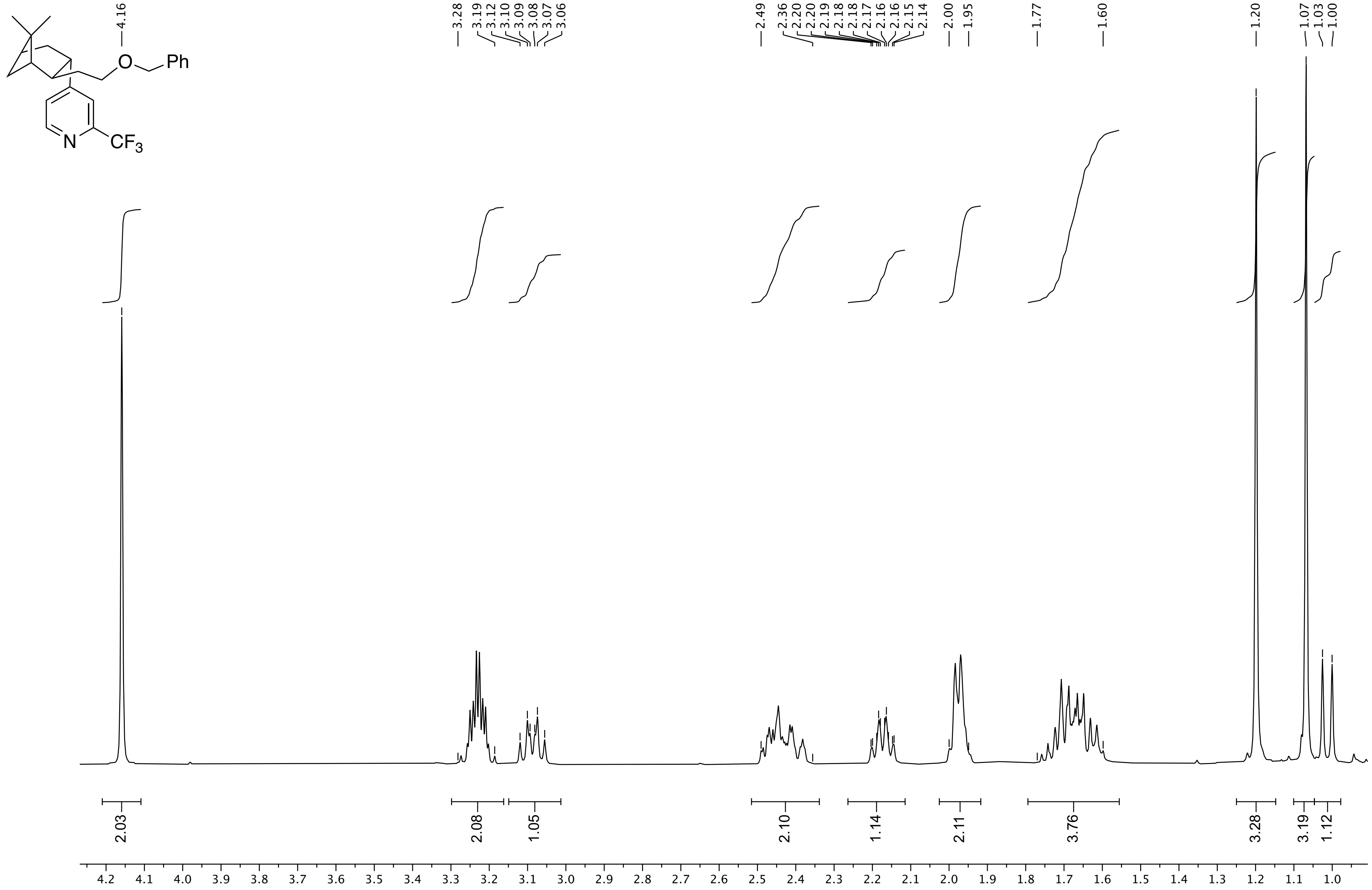

4-(2-(2-(Benzyloxy)ethyl)-6,6-dimethylbicyclo[3.1.1]heptan-3-yl)-2-(trifluoromethyl)pyridine (C_4-26) ^{13}C-NMR (101 MHz, $CDCl_3$)

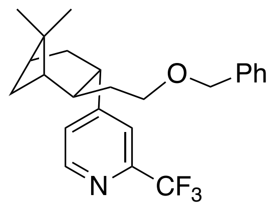

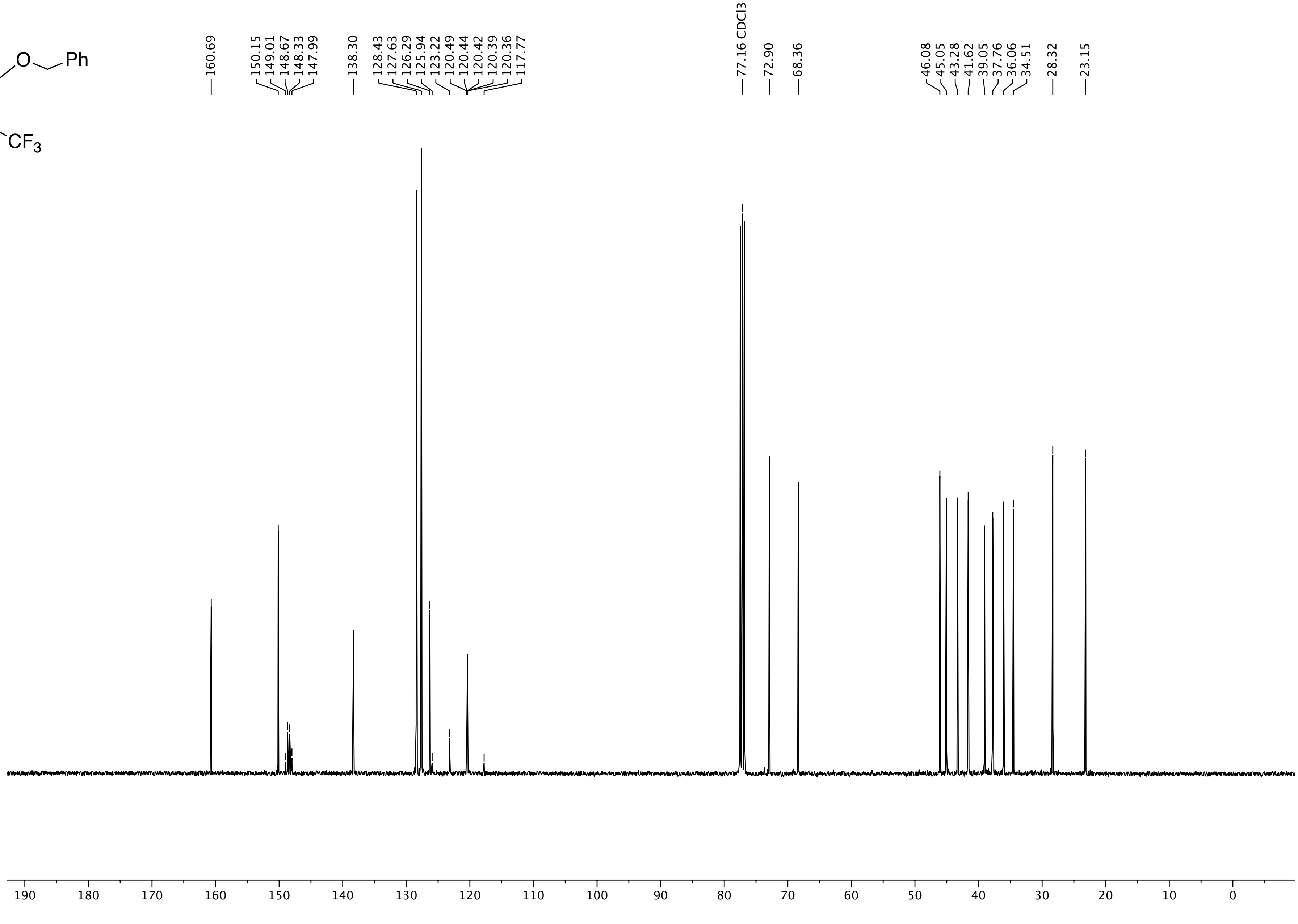

4-(2-(2-(Benzyloxy)ethyl)-6,6-dimethylbicyclo[3.1.1]heptan-3-yl)-2-(trifluoromethyl)pyridine (C_4-26) ^{13}C-NMR (101 MHz, $CDCl_3$)

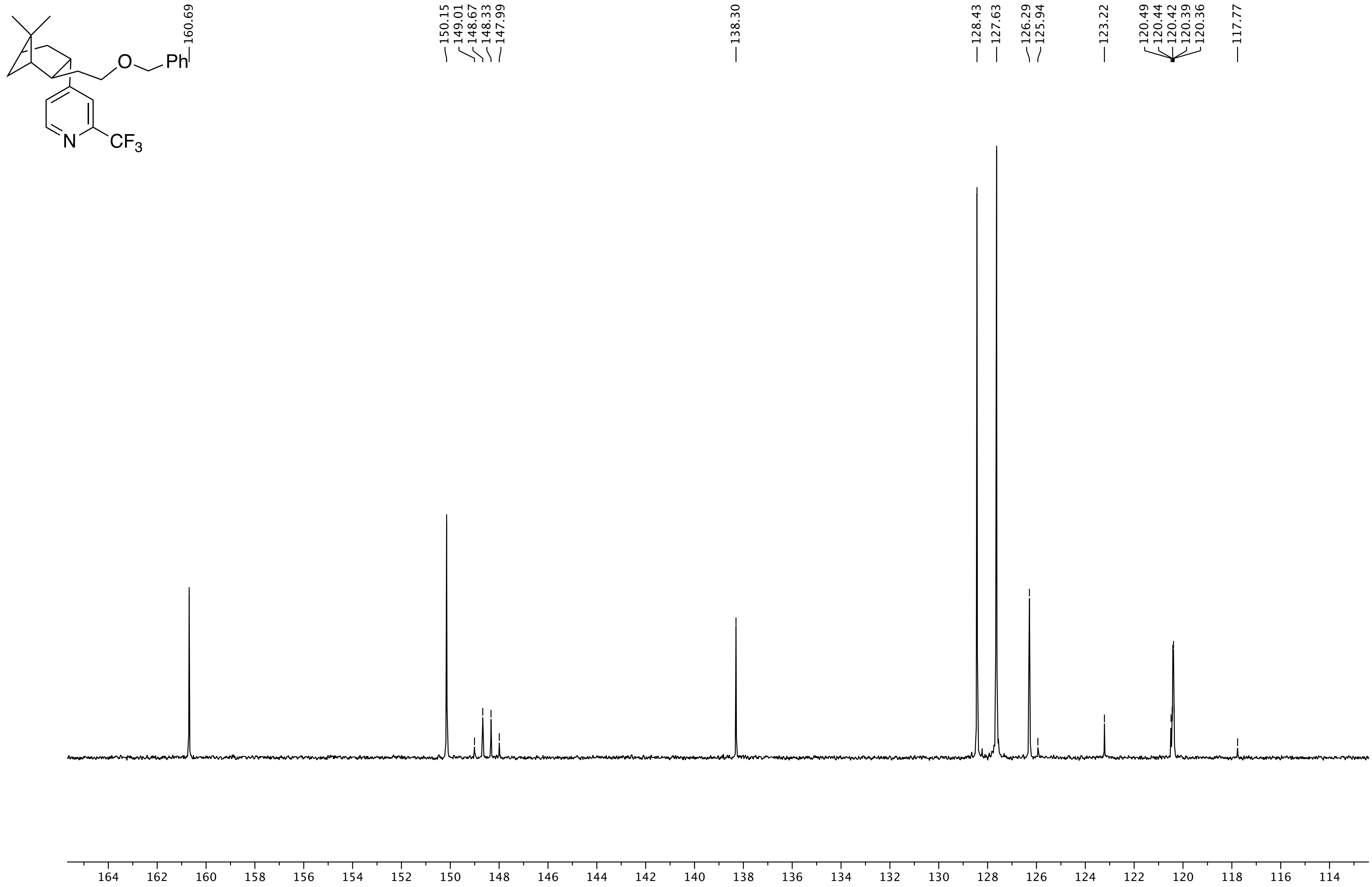

4-(2-(2-(Benzyloxy)ethyl)-6,6-dimethylbicyclo[3.1.1]heptan-3-yl)-2-(trifluoromethyl)pyridine (C_4-26) ^{13}C-NMR (101 MHz, $CDCl_3$)

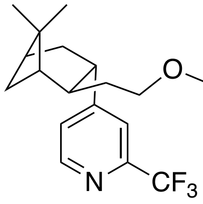

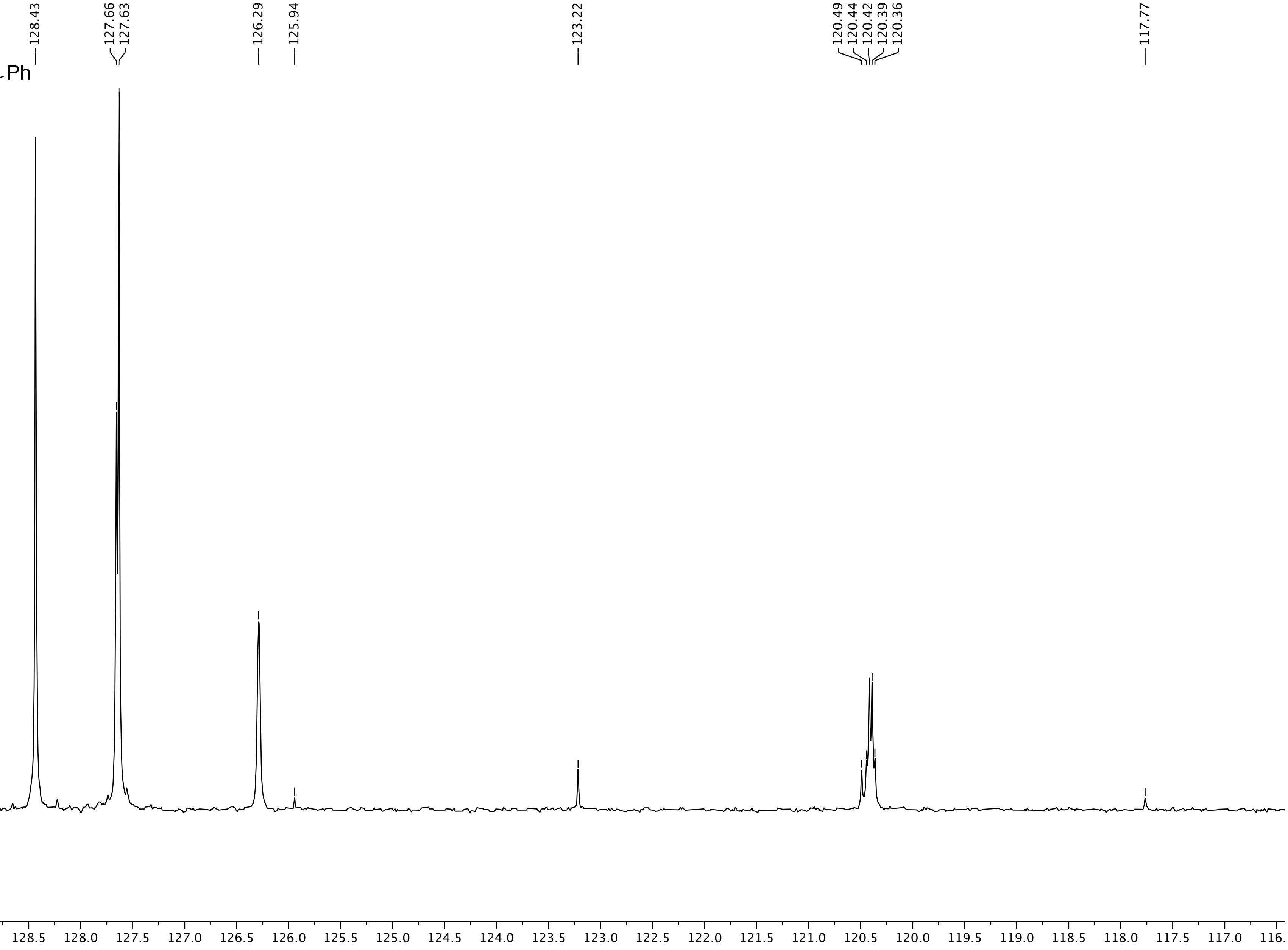

4-(2-(2-(Benzyloxy)ethyl)-6,6-dimethylbicyclo[3.1.1]heptan-3-yl)-2-(trifluoromethyl)pyridine (C_4-26) ^{13}C-NMR (101 MHz, $CDCl_3$)

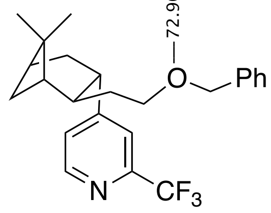

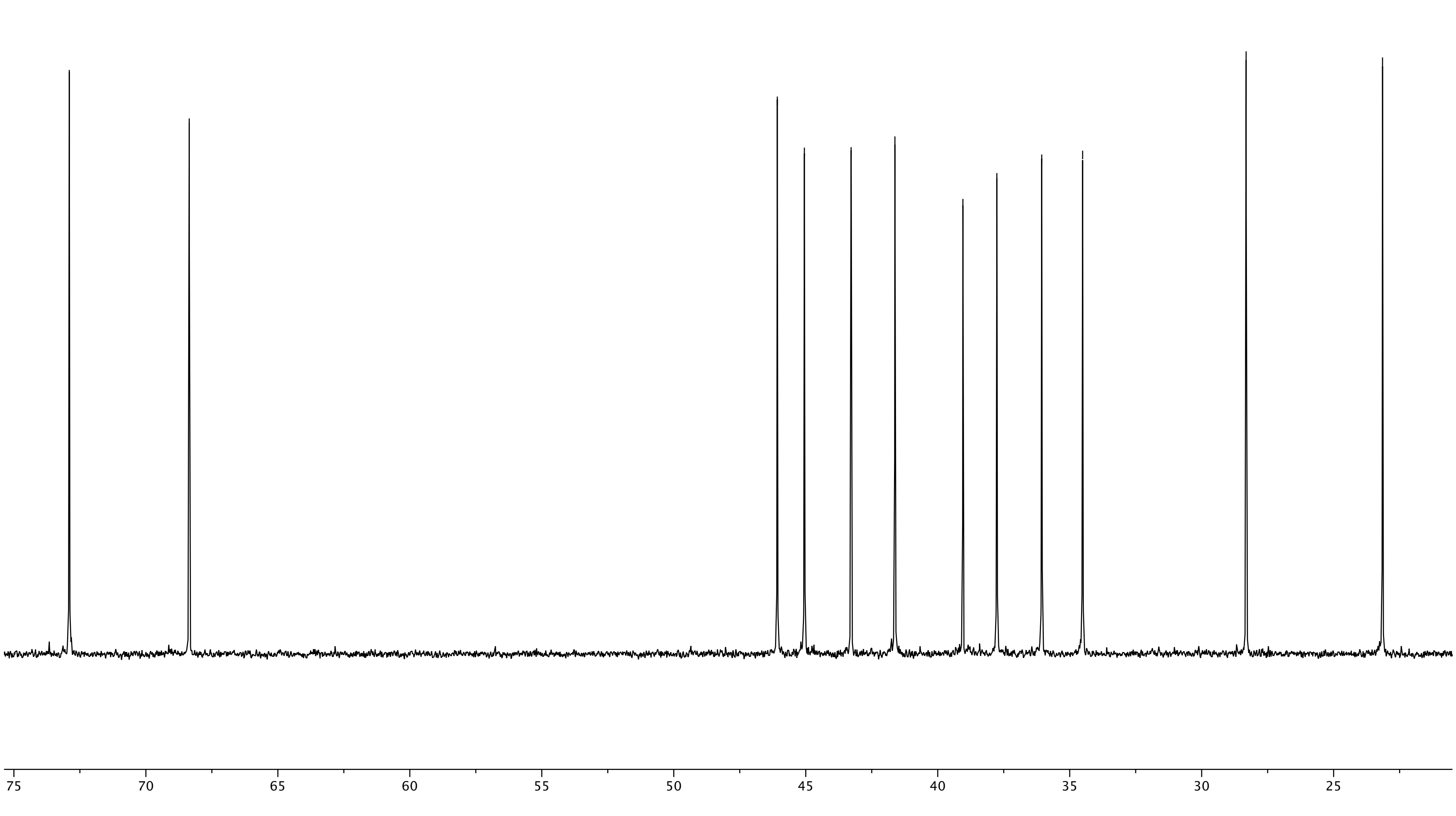

4-(2-(2-(Benzyloxy)ethyl)-6,6-dimethylbicyclo[3.1.1]heptan-3-yl)-2-(trifluoromethyl)pyridine (C_4-26) Dept135 (101 MHz, $CDCl_3$)

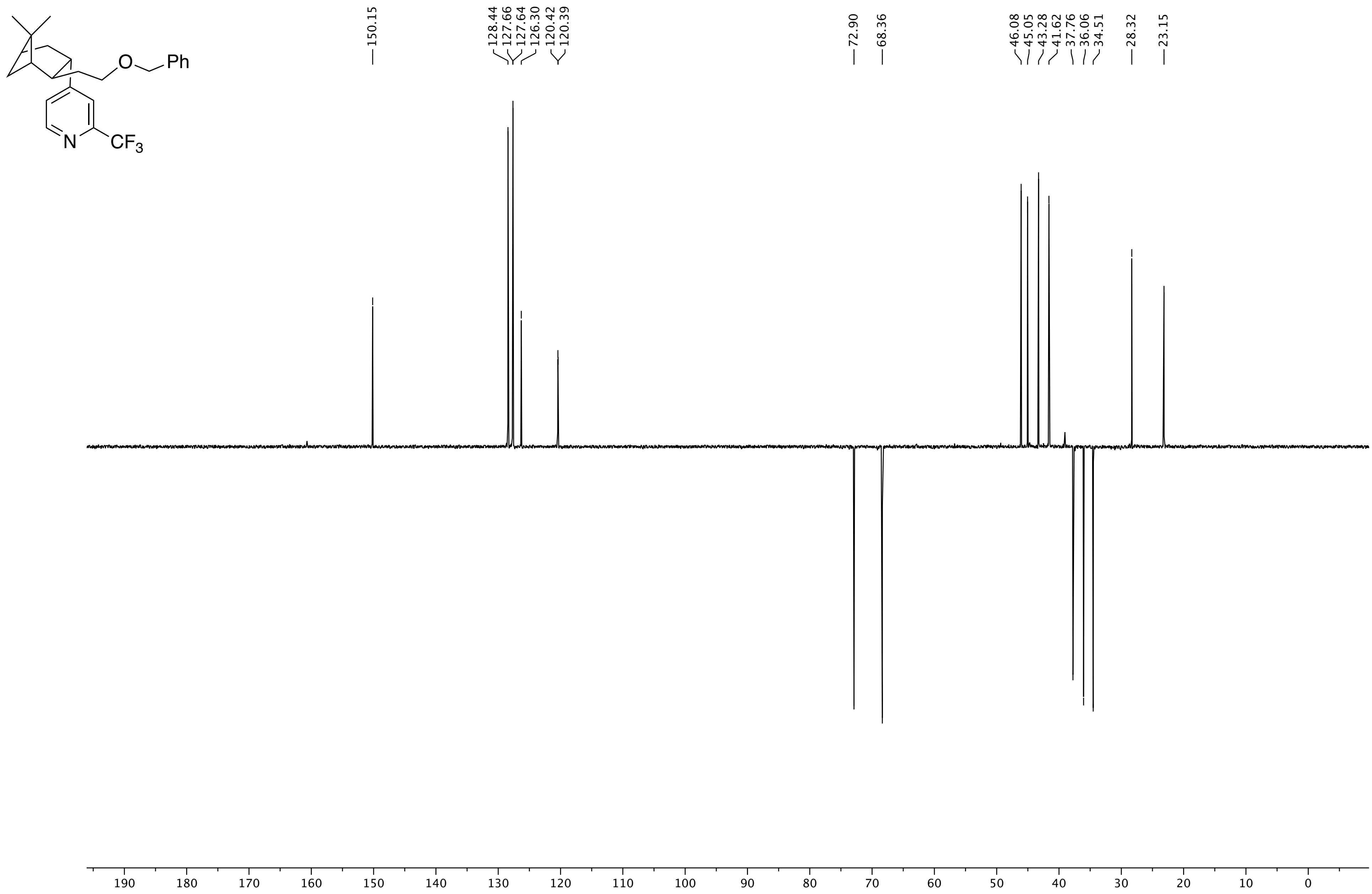

4-(2-(2-(Benzyloxy)ethyl)-6,6-dimethylbicyclo[3.1.1]heptan-3-yl)-2-(trifluoromethyl)pyridine (C_4-26) ^{1}H-^{1}H COSY (400 MHz, $CDCl_3$)

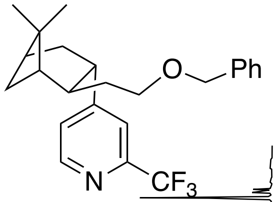

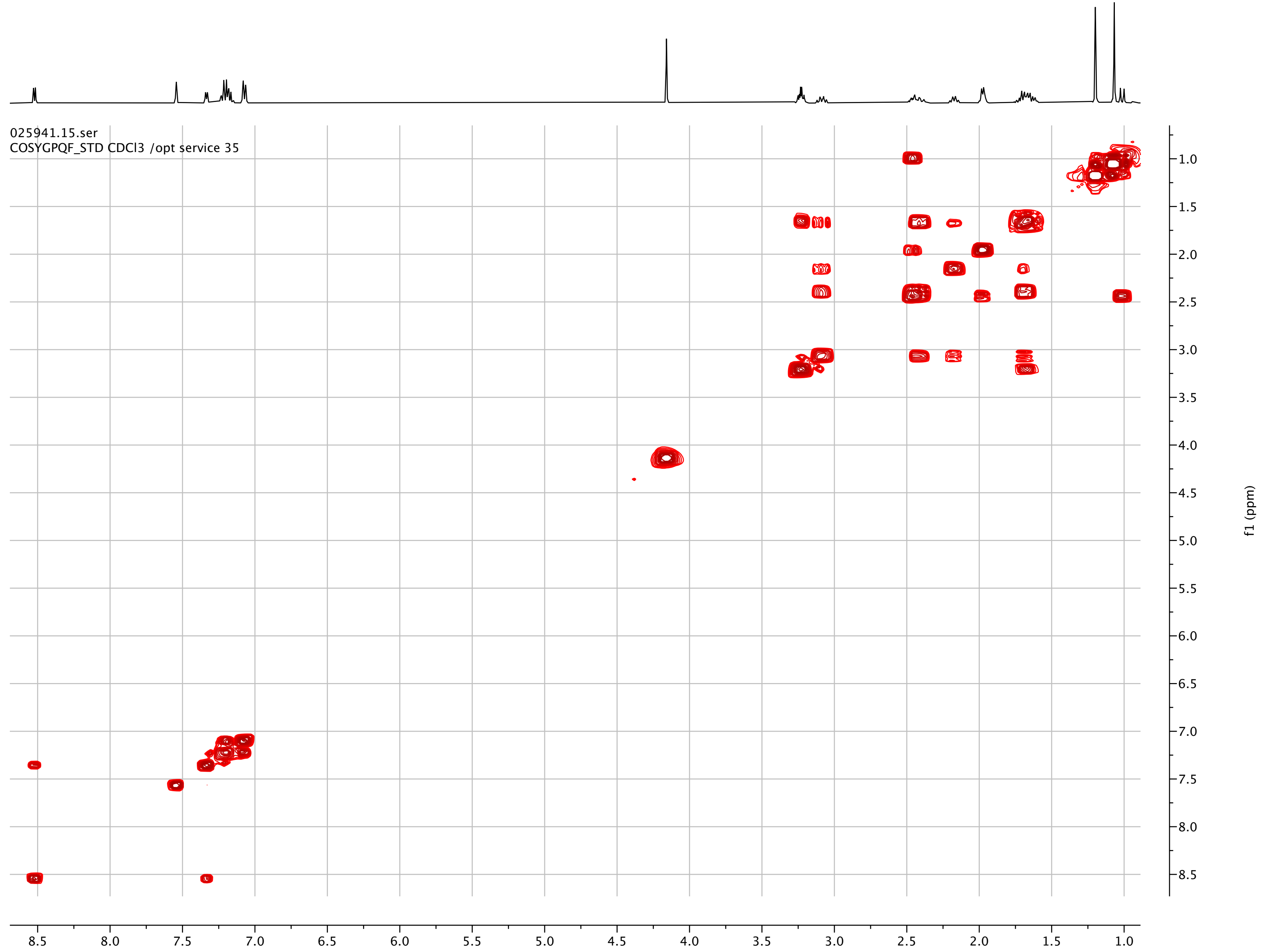

4-(2-(2-(Benzyloxy)ethyl)-6,6-dimethylbicyclo[3.1.1]heptan-3-yl)-2-(trifluoromethyl)pyridine (C_4-26) ^1H-^1H COSY (400 MHz, $CDCl_3$)

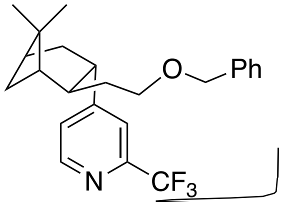

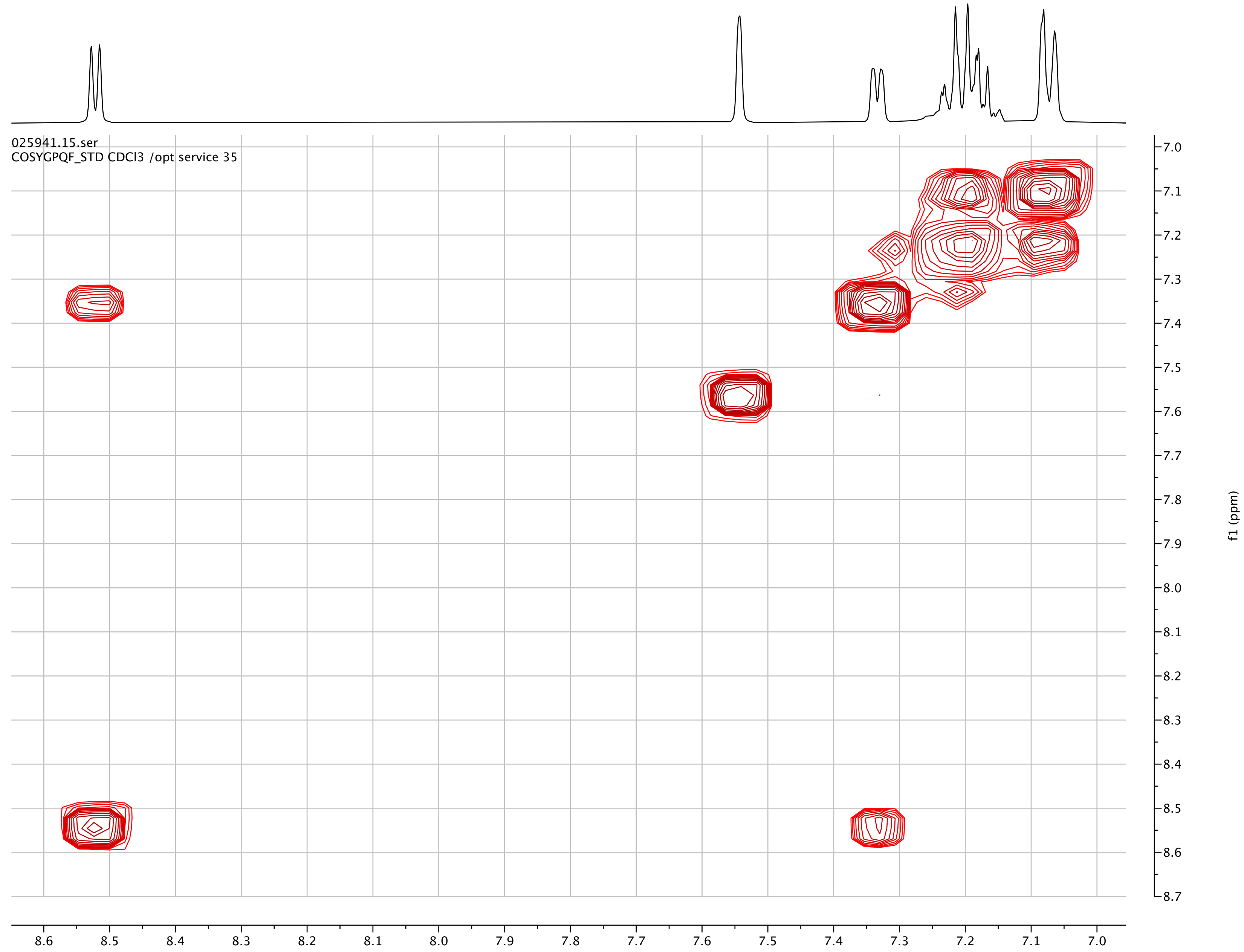

4-(2-(2-(Benzyloxy)ethyl)-6,6-dimethylbicyclo[3.1.1]heptan-3-yl)-2-(trifluoromethyl)pyridine (C_4-26) ^{1}H-^{1}H COSY (400 MHz, $CDCl_3$)

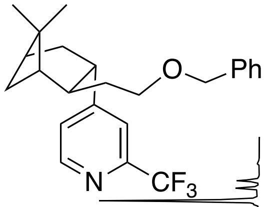

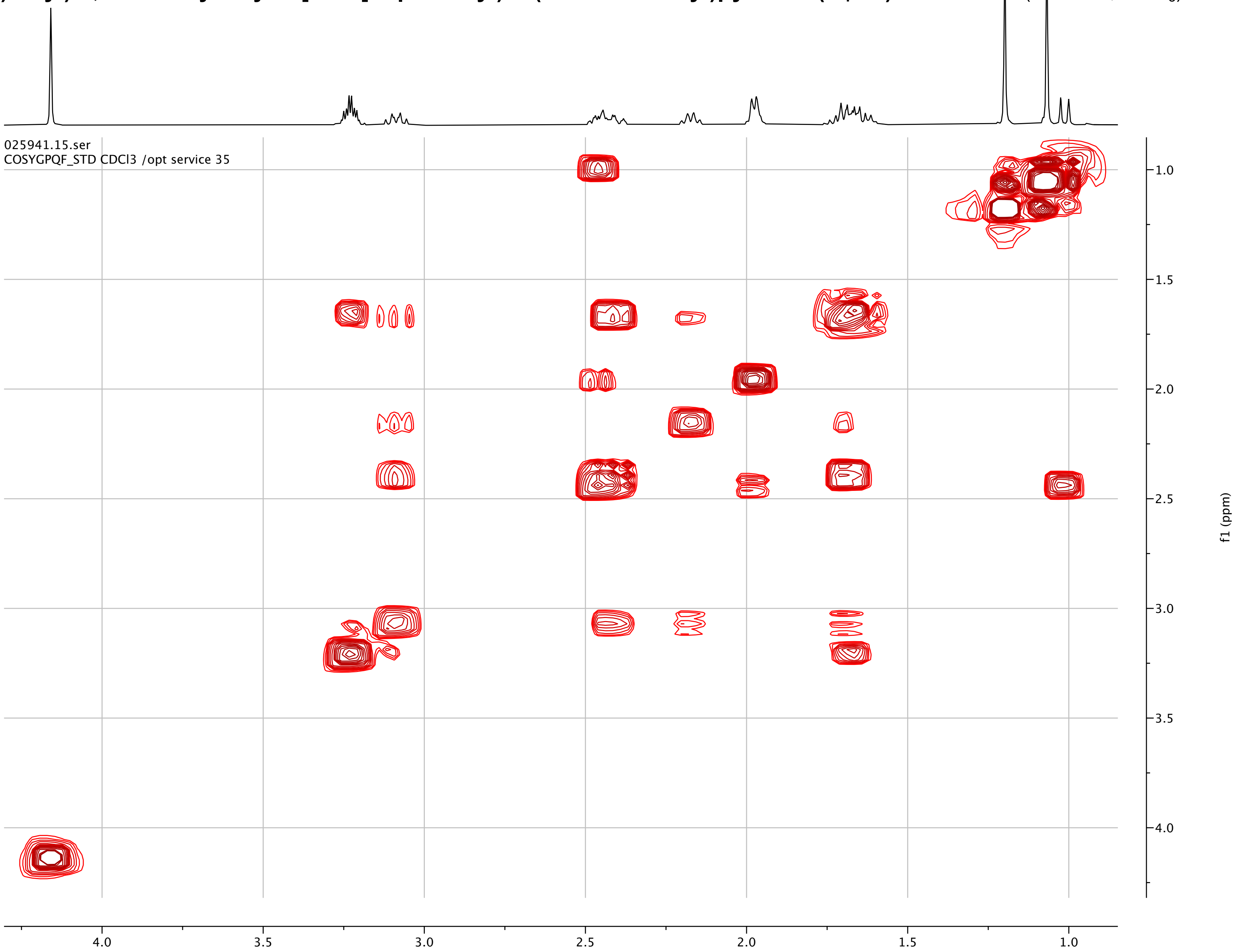

4-(2-(2-(Benzyloxy)ethyl)-6,6-dimethylbicyclo[3.1.1]heptan-3-yl)-2-(trifluoromethyl)pyridine (C_4-26) HSQC (400 MHz, $CDCl_3$)

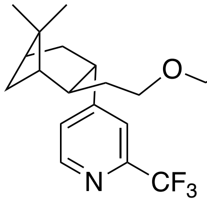

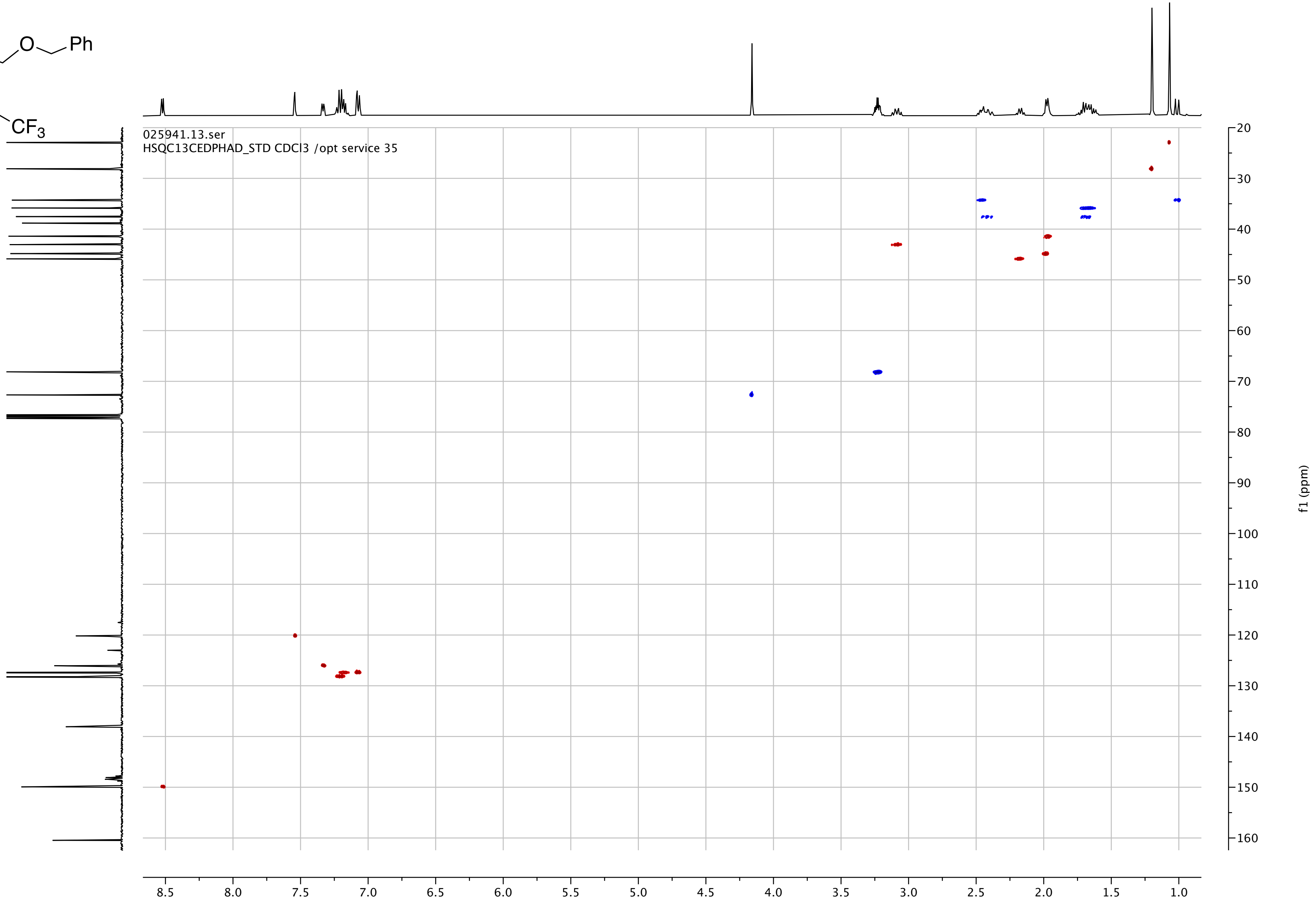

4-(2-(2-(Benzyloxy)ethyl)-6,6-dimethylbicyclo[3.1.1]heptan-3-yl)-2-(trifluoromethyl)pyridine (C_4-26) HSQC (400 MHz, $CDCl_3$)

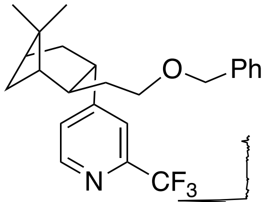

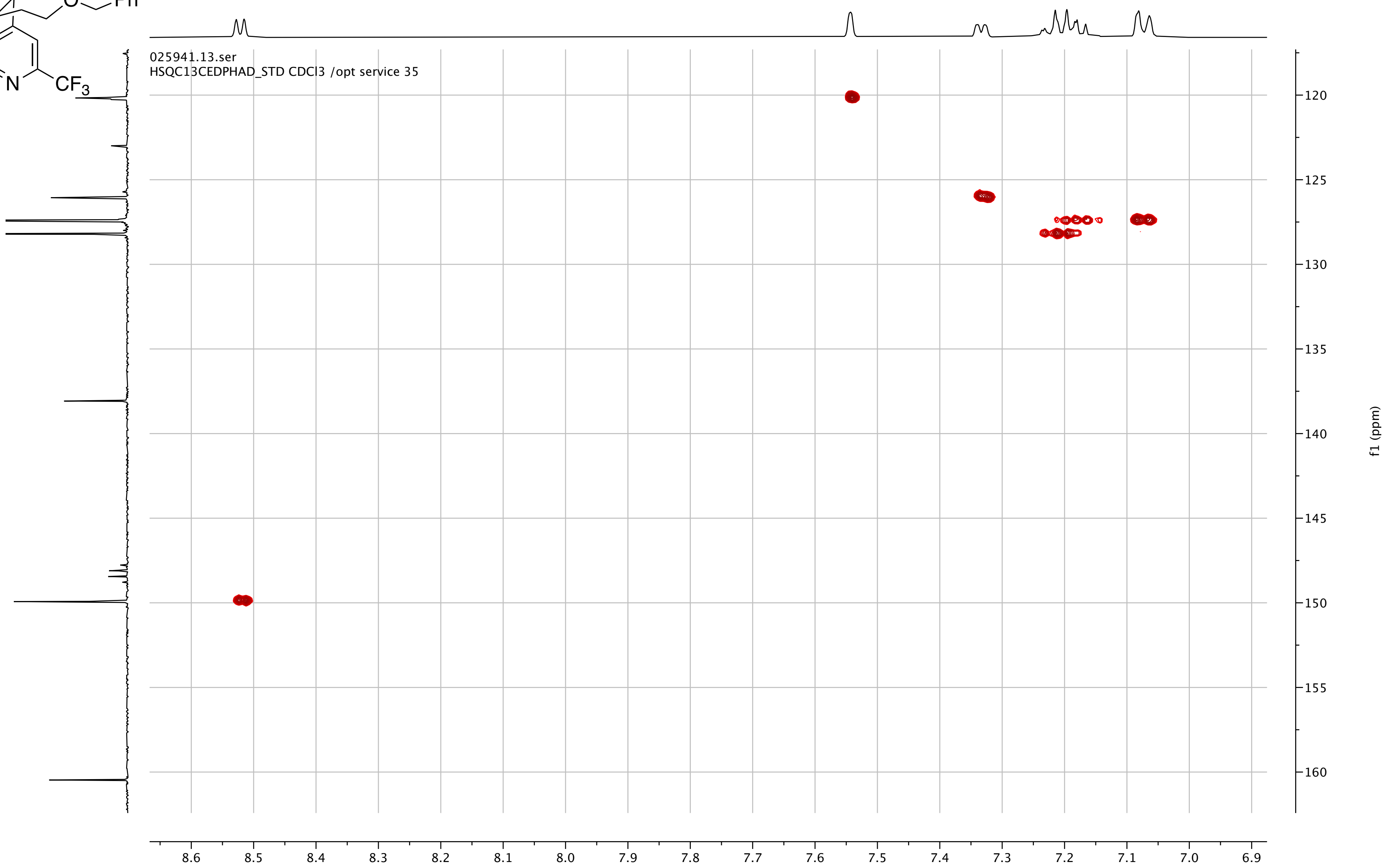

4-(2-(2-(Benzyloxy)ethyl)-6,6-dimethylbicyclo[3.1.1]heptan-3-yl)-2-(trifluoromethyl)pyridine (C_4-26) HSQC (400 MHz, $CDCl_3$)

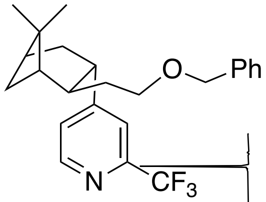

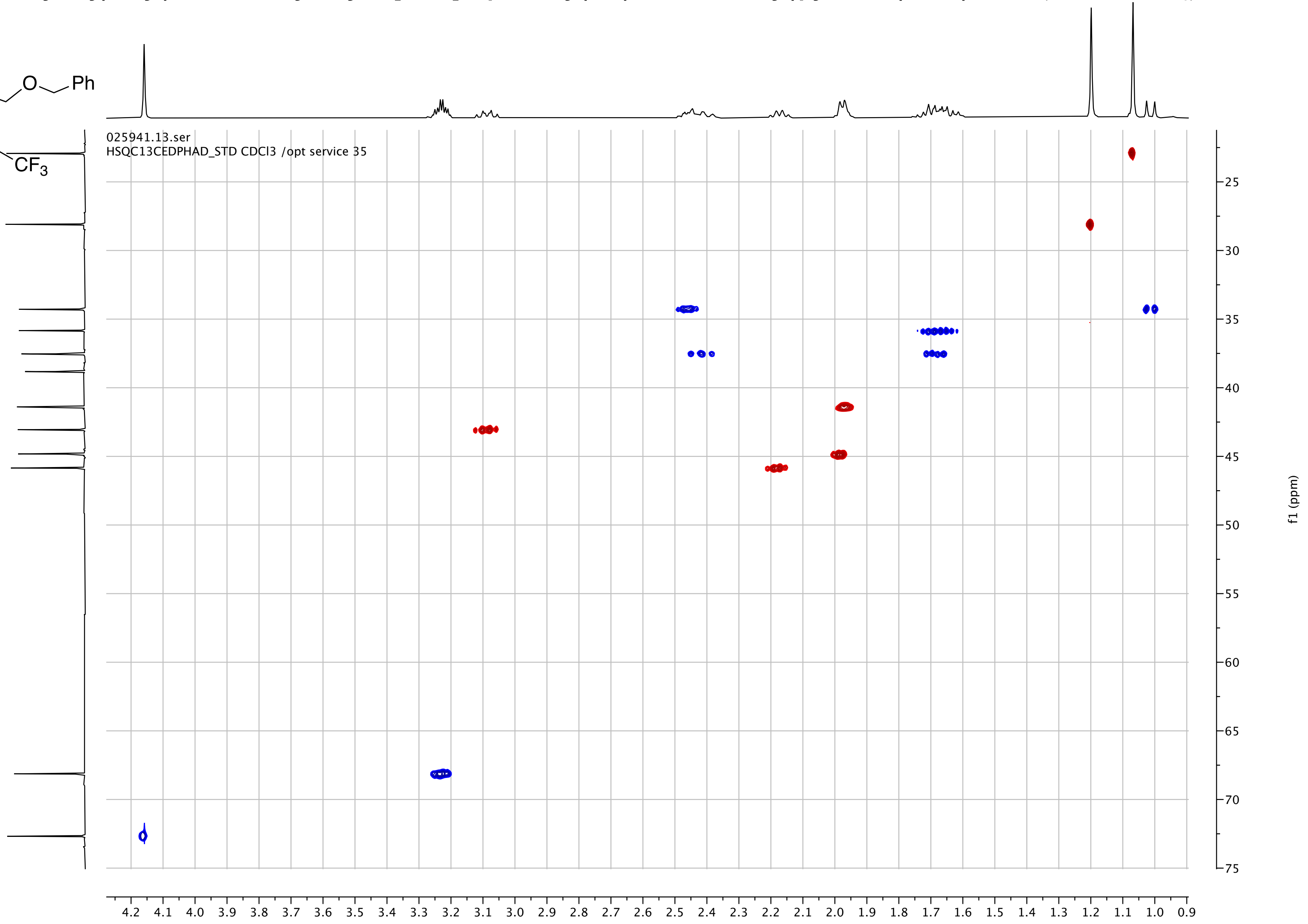

4-(2-(2-(Benzyloxy)ethyl)-6,6-dimethylbicyclo[3.1.1]heptan-3-yl)-2-(trifluoromethyl)pyridine (C_4-26) HMBC (400 MHz, $CDCl_3$)

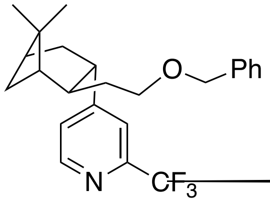

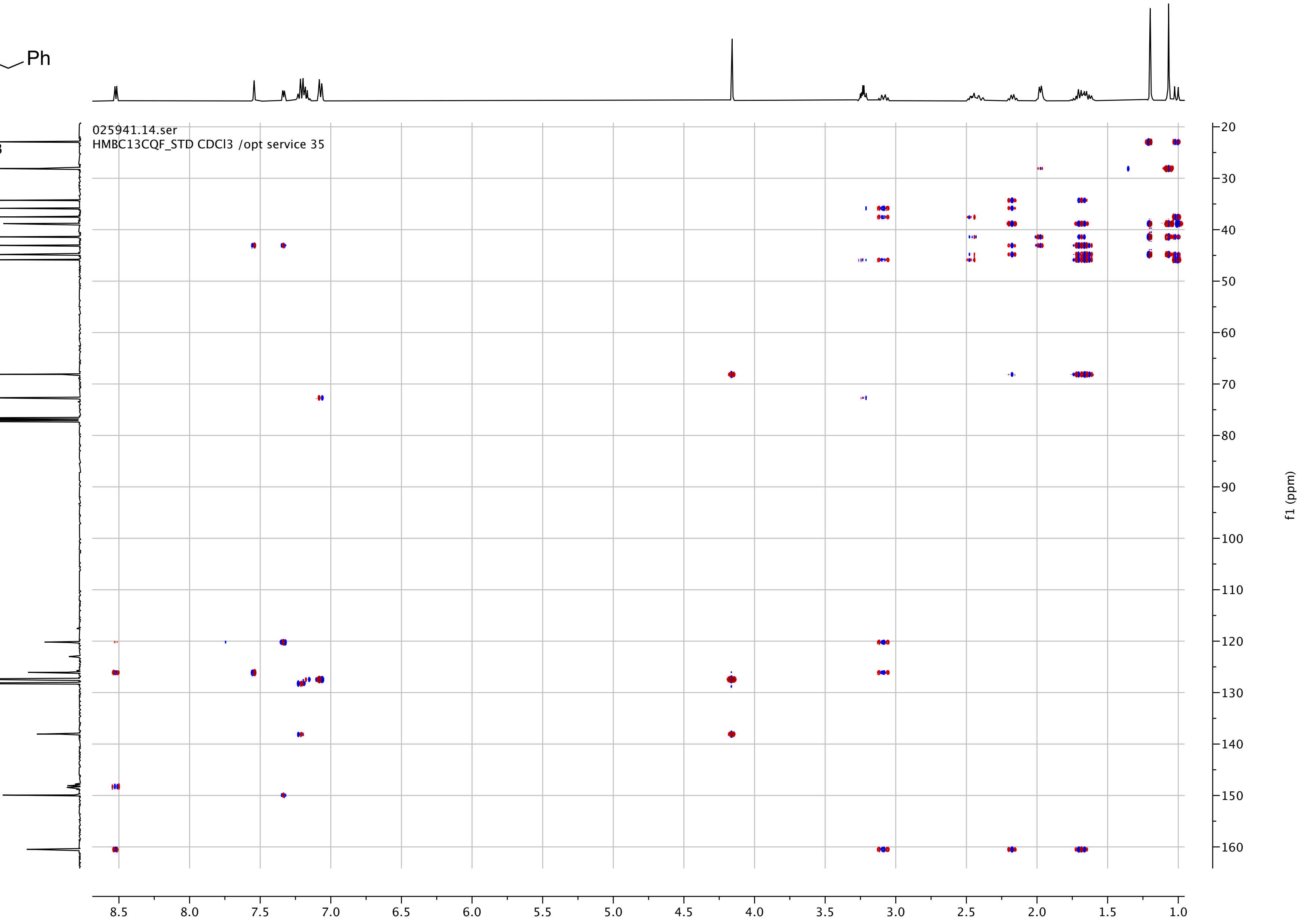

4-(2-(2-(Benzyloxy)ethyl)-6,6-dimethylbicyclo[3.1.1]heptan-3-yl)-2-(trifluoromethyl)pyridine (C_4-26) HMBC (400 MHz, $CDCl_3$)

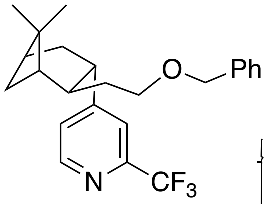

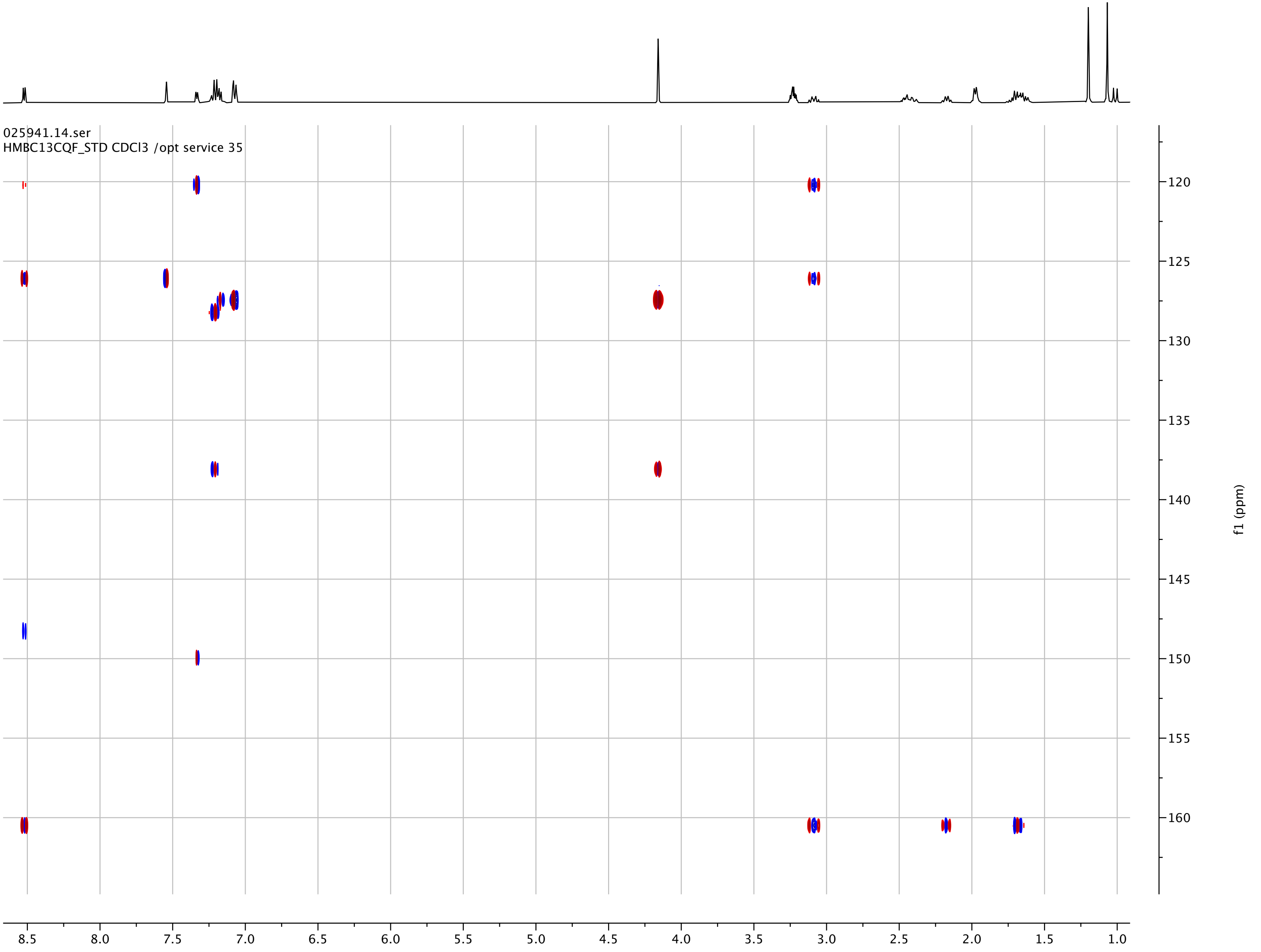

4-(2-(2-(Benzyloxy)ethyl)-6,6-dimethylbicyclo[3.1.1]heptan-3-yl)-2-(trifluoromethyl)pyridine (C_4-26) HMBC (400 MHz, $CDCl_3$)

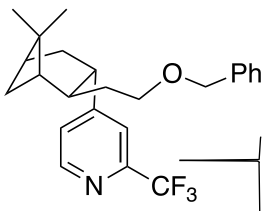

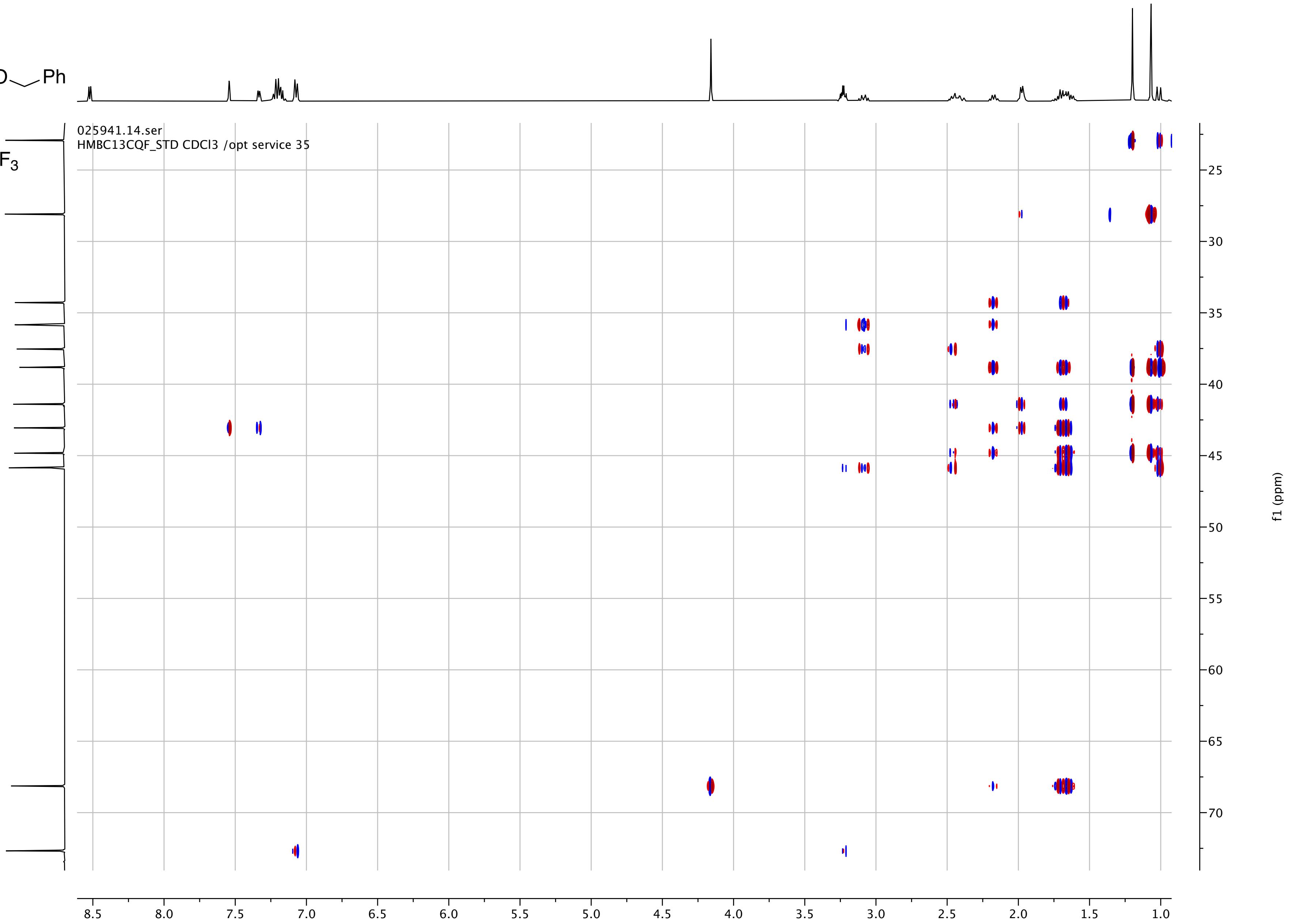

4-(2-(2-(Benzyloxy)ethyl)-6,6-dimethylbicyclo[3.1.1]heptan-3-yl)-2-(trifluoromethyl)pyridine (C_4-26) NOESY (400 MHz, $CDCl_3$)

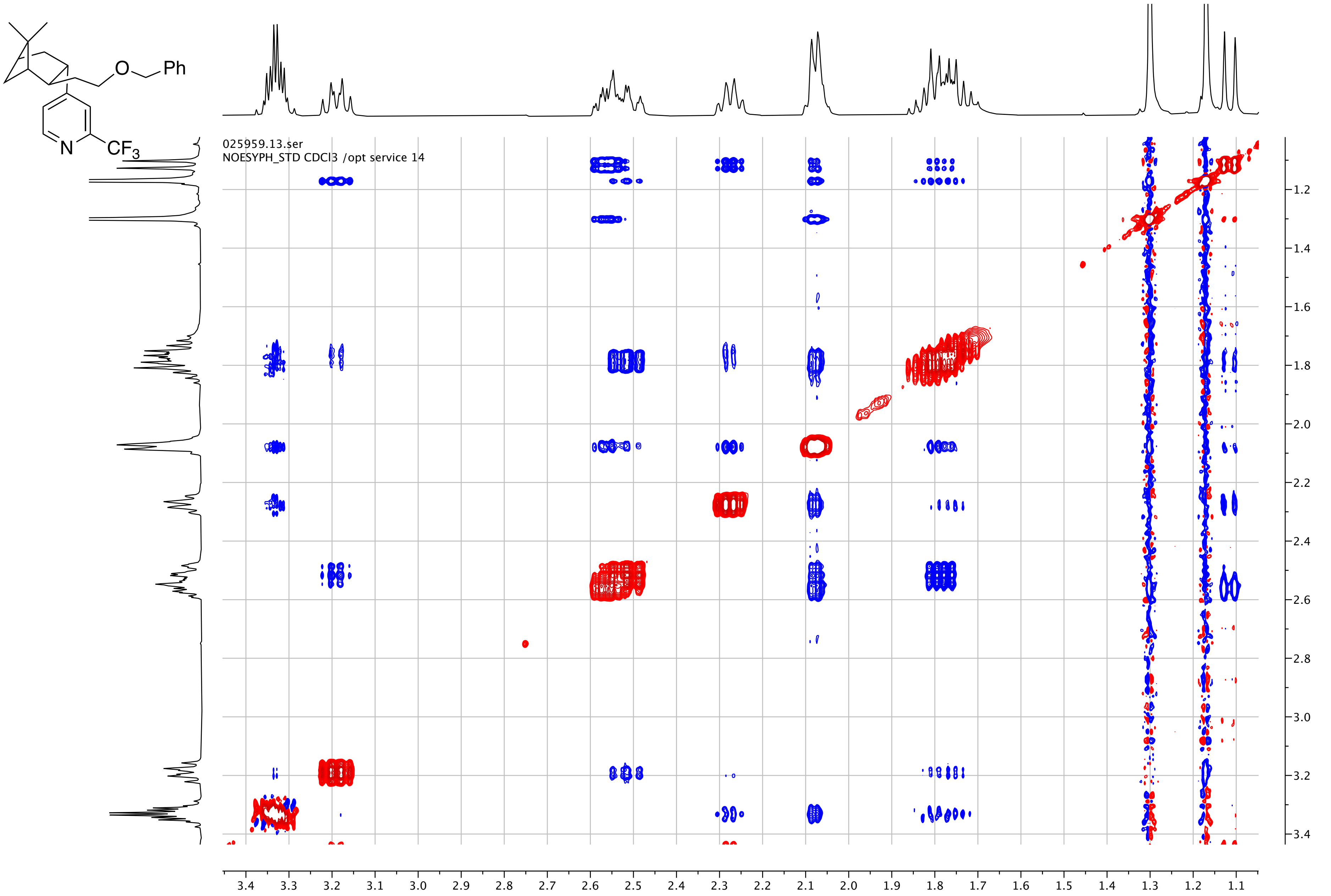

4-(2-(2-(Benzyloxy)ethyl)-6,6-dimethylbicyclo[3.1.1]heptan-3-yl)-2-(trifluoromethyl)pyridine (C_4-26) NOESY (400 MHz, $CDCl_3$)

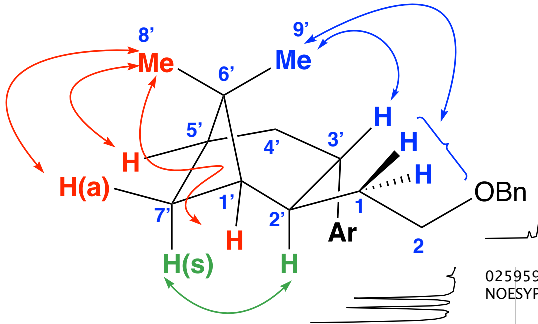

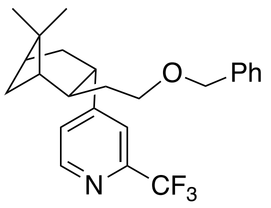

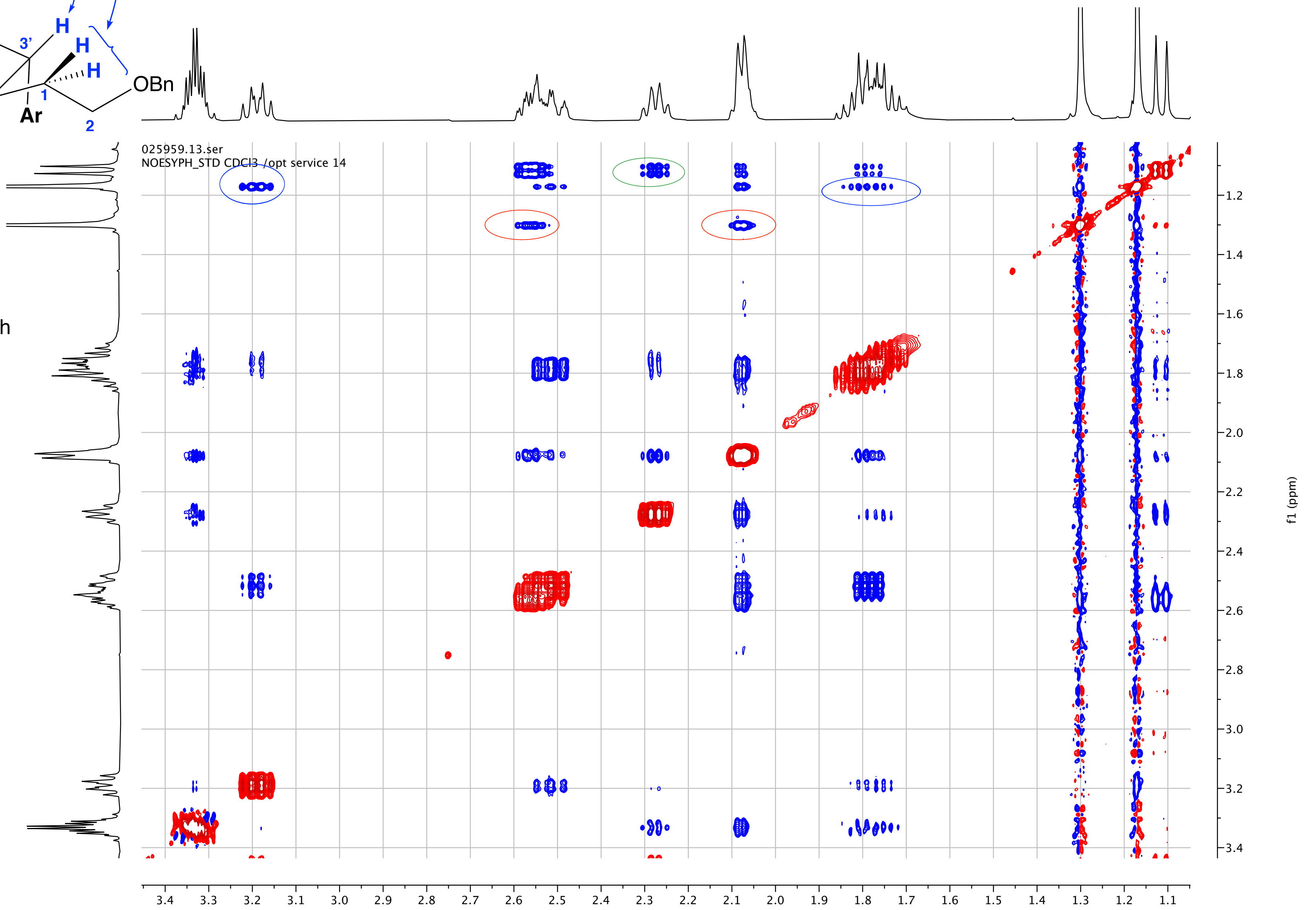

4-(2-(2-(Benzyloxy)ethyl)-6,6-dimethylbicyclo[3.1.1]heptan-3-yl)-2-(trifluoromethyl)pyridine (C_4-26) 19-F (376 MHz, $CDCl_3$)

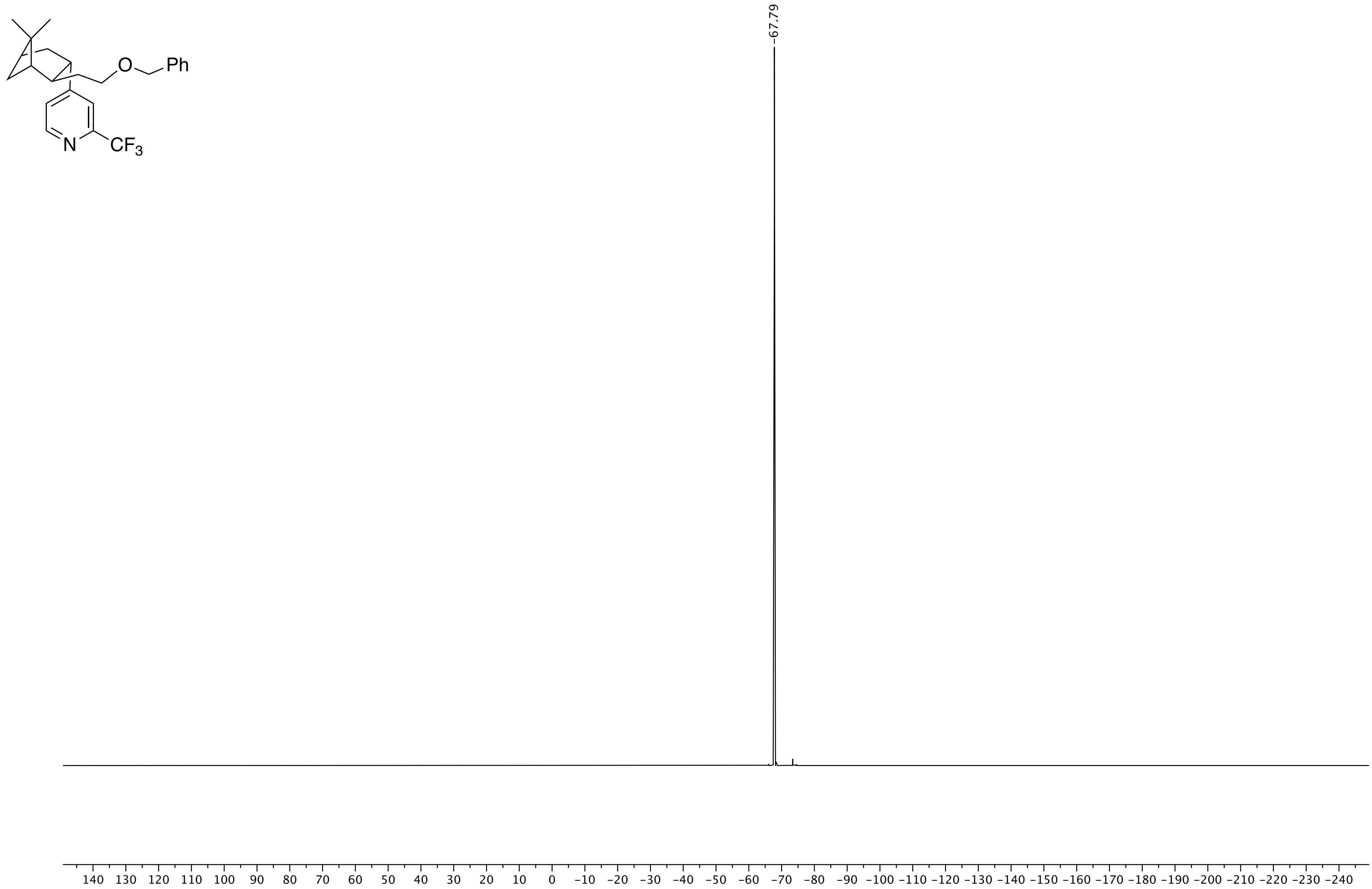

2-(6,6-Dimethyl-3-(4-methylquinolin-2-yl)bicyclo[3.1.1]heptan-2-yl)ethan-1-ol (27)

^{1}H-NMR (400 MHz, $CDCl_3$)

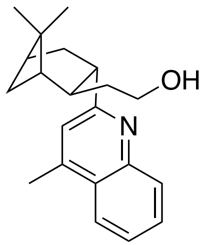

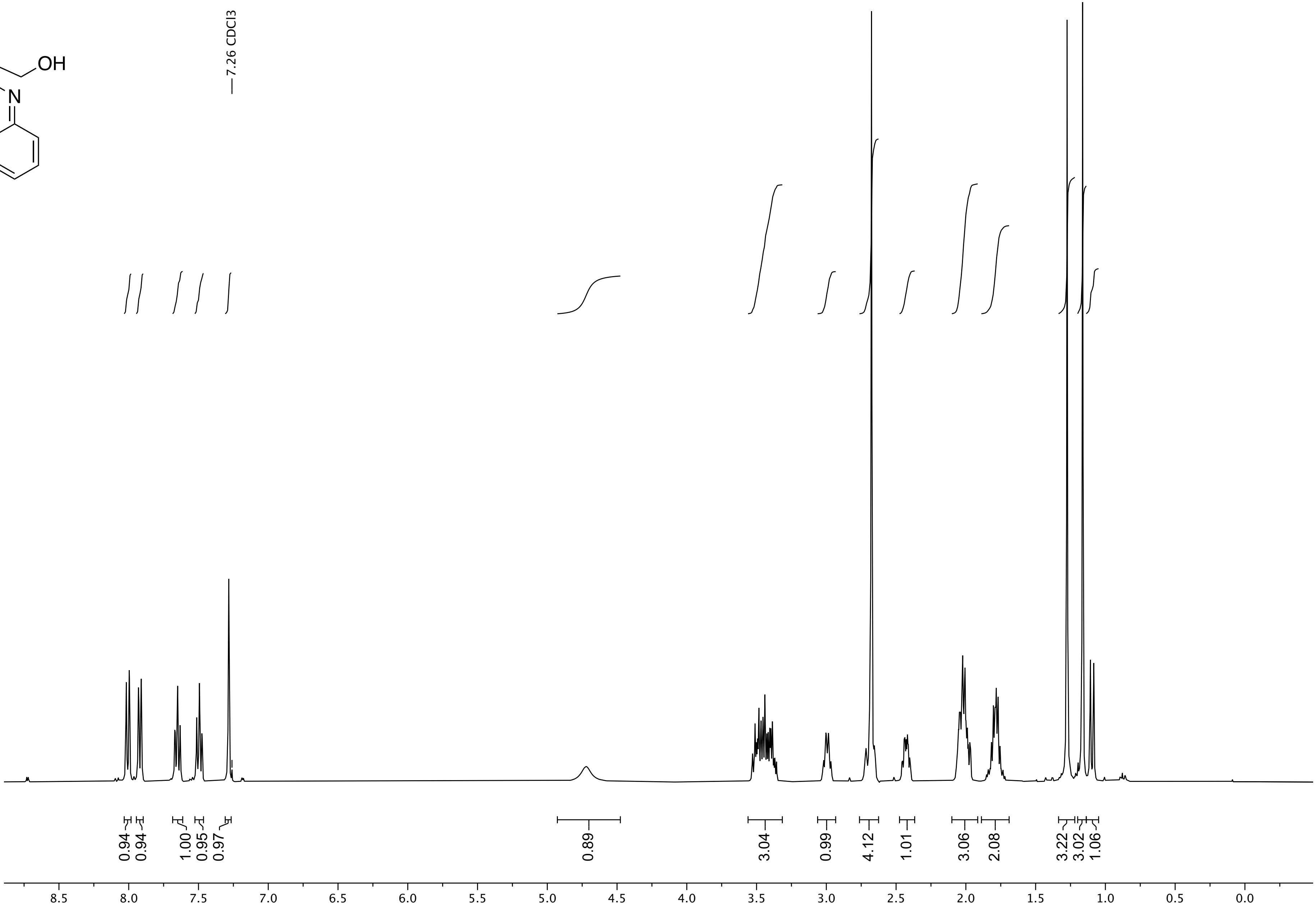

2-(6,6-Dimethyl-3-(4-methylquinolin-2-yl)bicyclo[3.1.1]heptan-2-yl)ethan-1-ol (27)

^{1}H-NMR (400 MHz, $CDCl_3$)

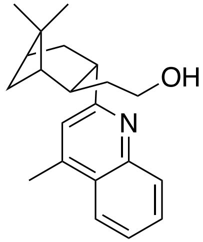

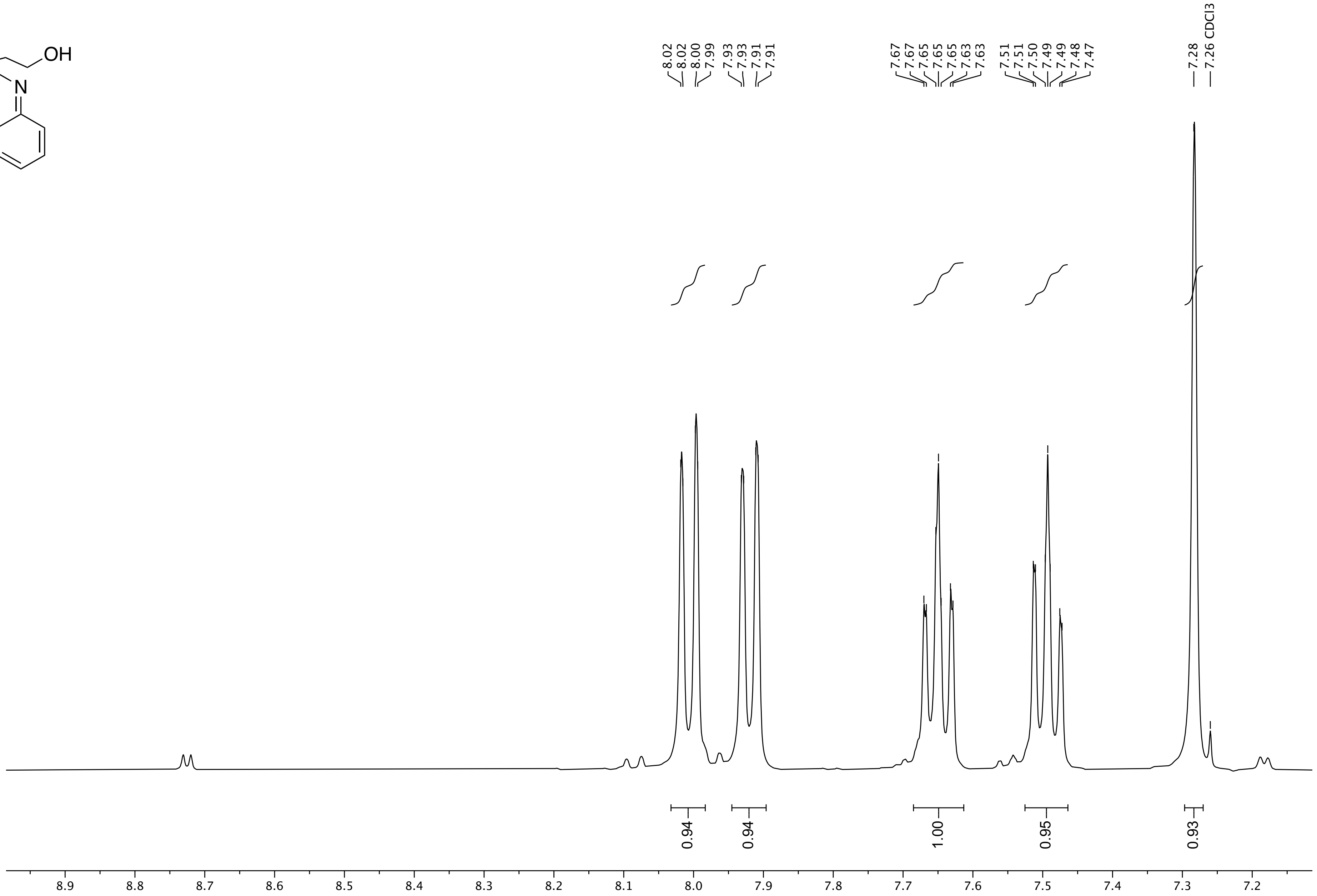

2-(6,6-Dimethyl-3-(4-methylquinolin-2-yl)bicyclo[3.1.1]heptan-2-yl)ethan-1-ol (27)

^{1}H-NMR (400 MHz, $CDCl_3$)

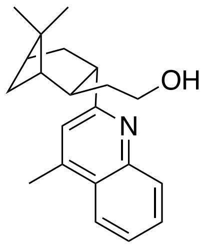

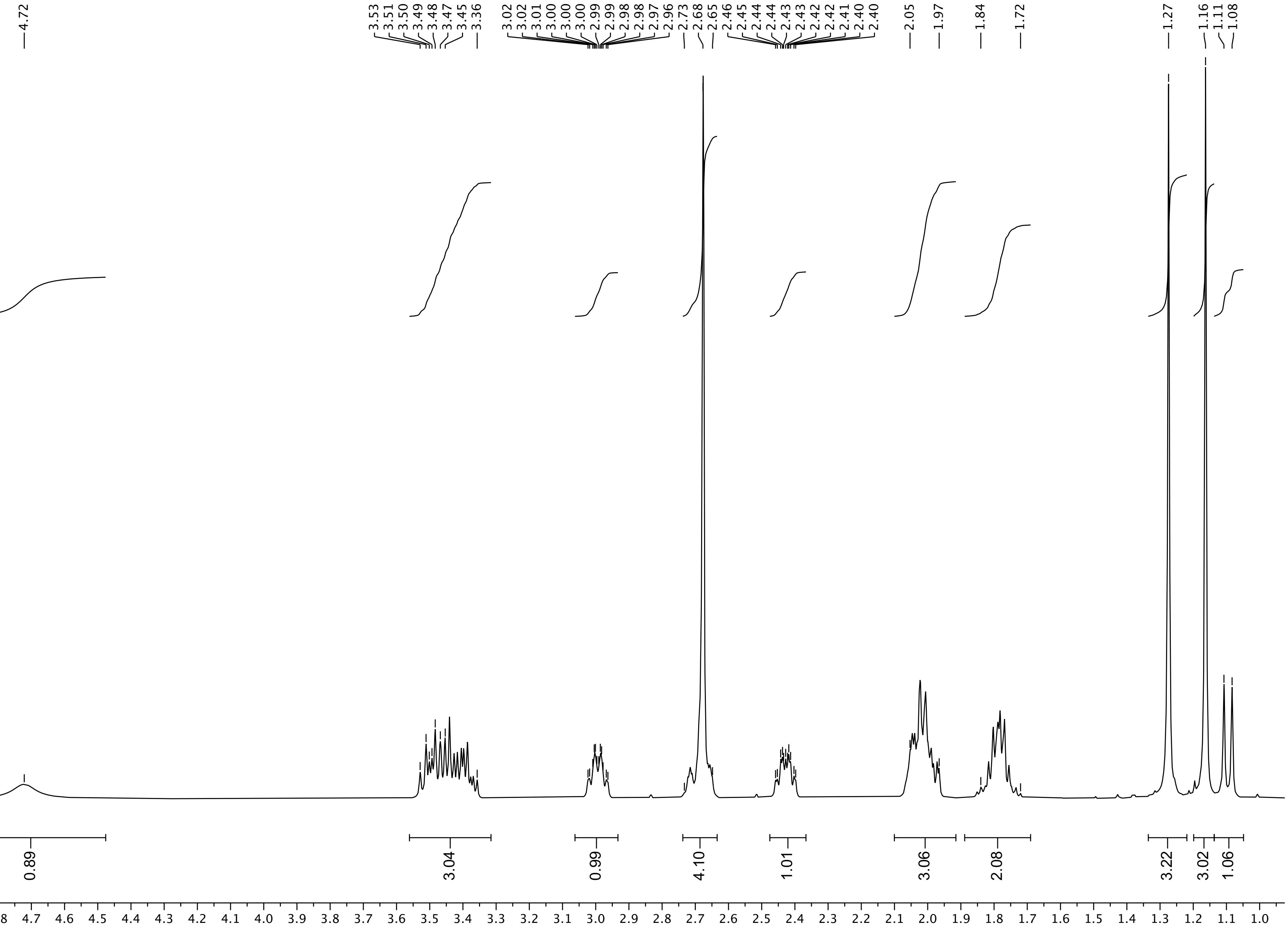

2-(6,6-Dimethyl-3-(4-methylquinolin-2-yl)bicyclo[3.1.1]heptan-2-yl)ethan-1-ol (27)

^{1}H-NMR (400 MHz, $CDCl_3$)

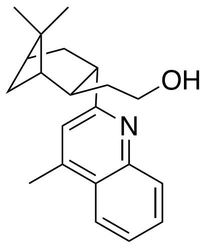

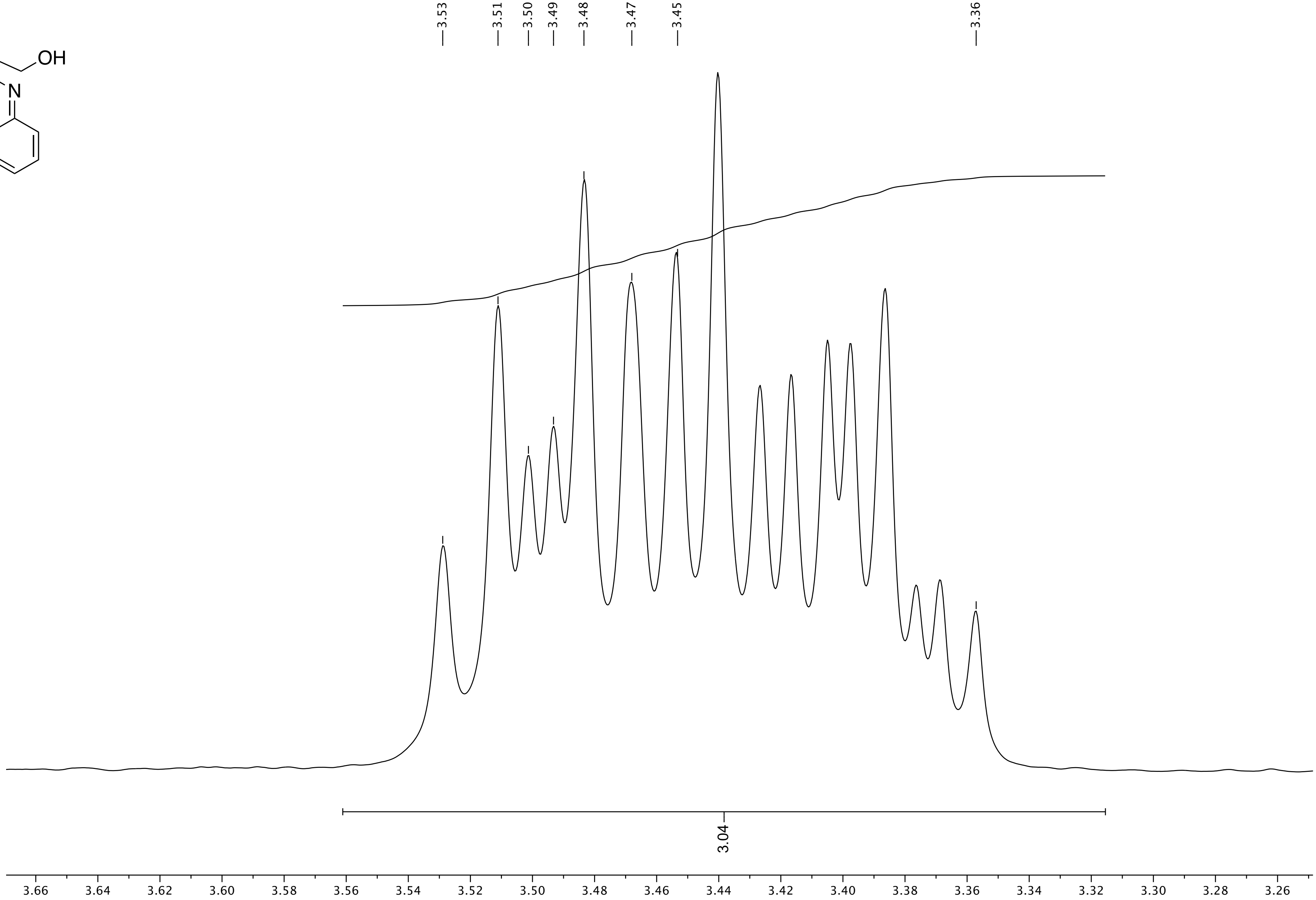

2-(6,6-Dimethyl-3-(4-methylquinolin-2-yl)bicyclo[3.1.1]heptan-2-yl)ethan-1-ol (27)

^{1}H-NMR (400 MHz, $CDCl_3$)

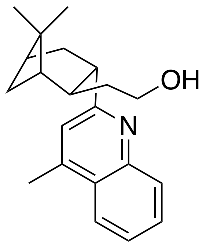

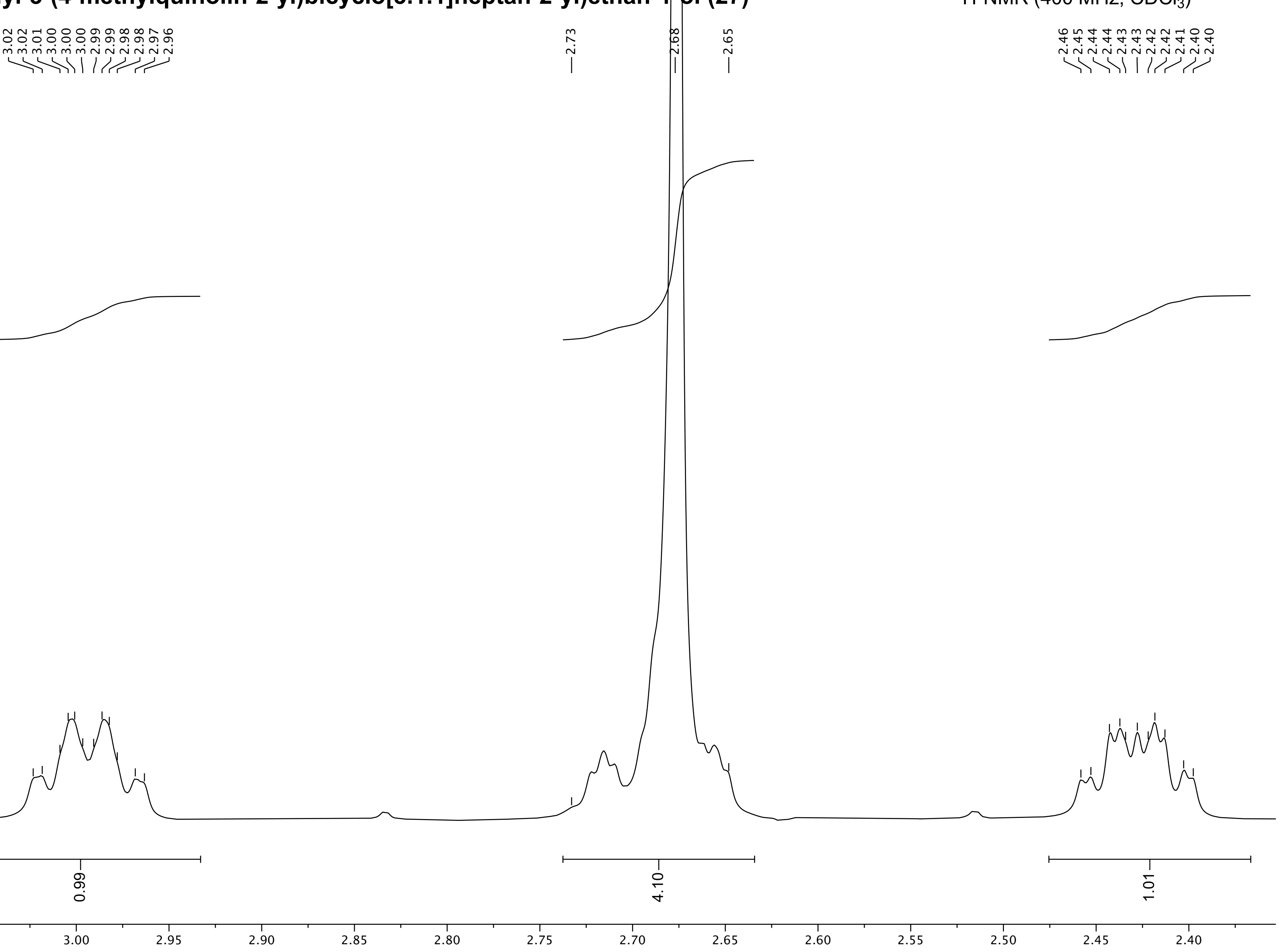

2-(6,6-Dimethyl-3-(4-methylquinolin-2-yl)bicyclo[3.1.1]heptan-2-yl)ethan-1-ol (27)

^{13}C-NMR (101 MHz, CDCl$_3$)

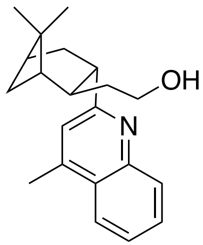

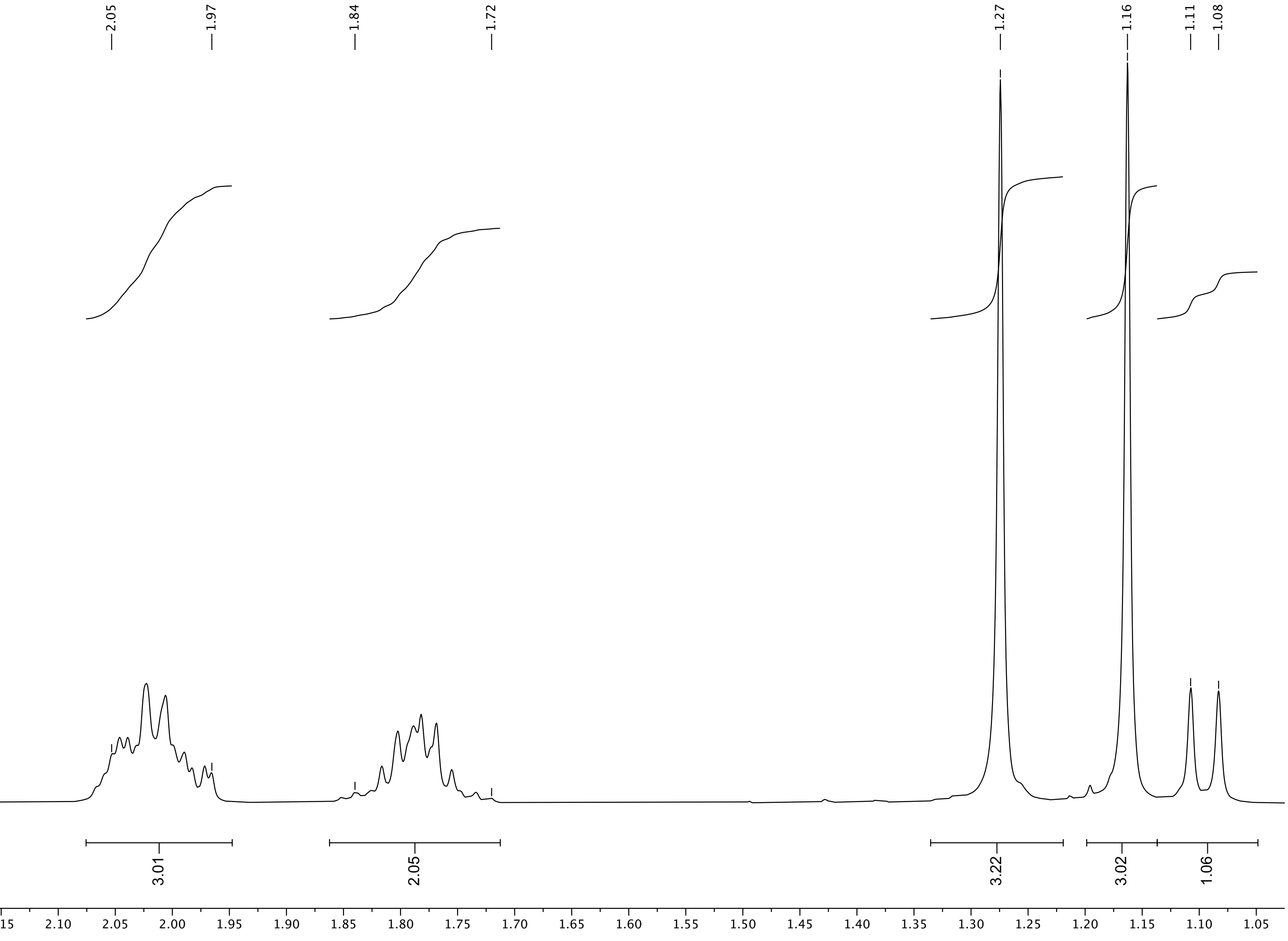

2-(6,6-Dimethyl-3-(4-methylquinolin-2-yl)bicyclo[3.1.1]heptan-2-yl)ethan-1-ol (27)

^{13}C-NMR (101 MHz, $CDCl_3$)

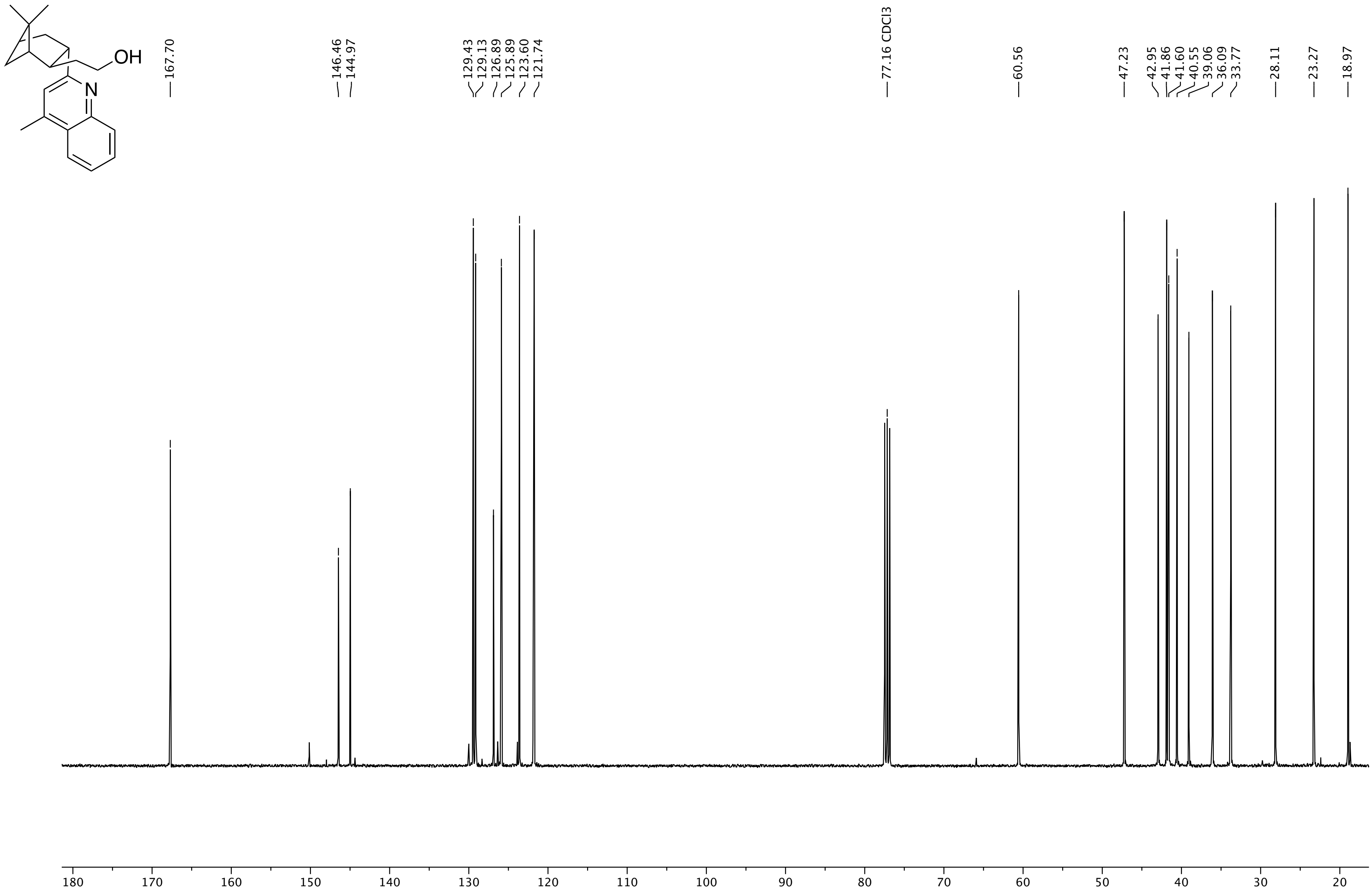

2-(6,6-Dimethyl-3-(4-methylquinolin-2-yl)bicyclo[3.1.1]heptan-2-yl)ethan-1-ol (27)

^{13}C-NMR (101 MHz, $CDCl_3$)

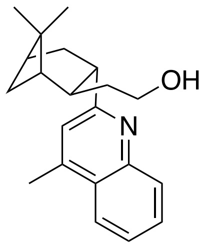

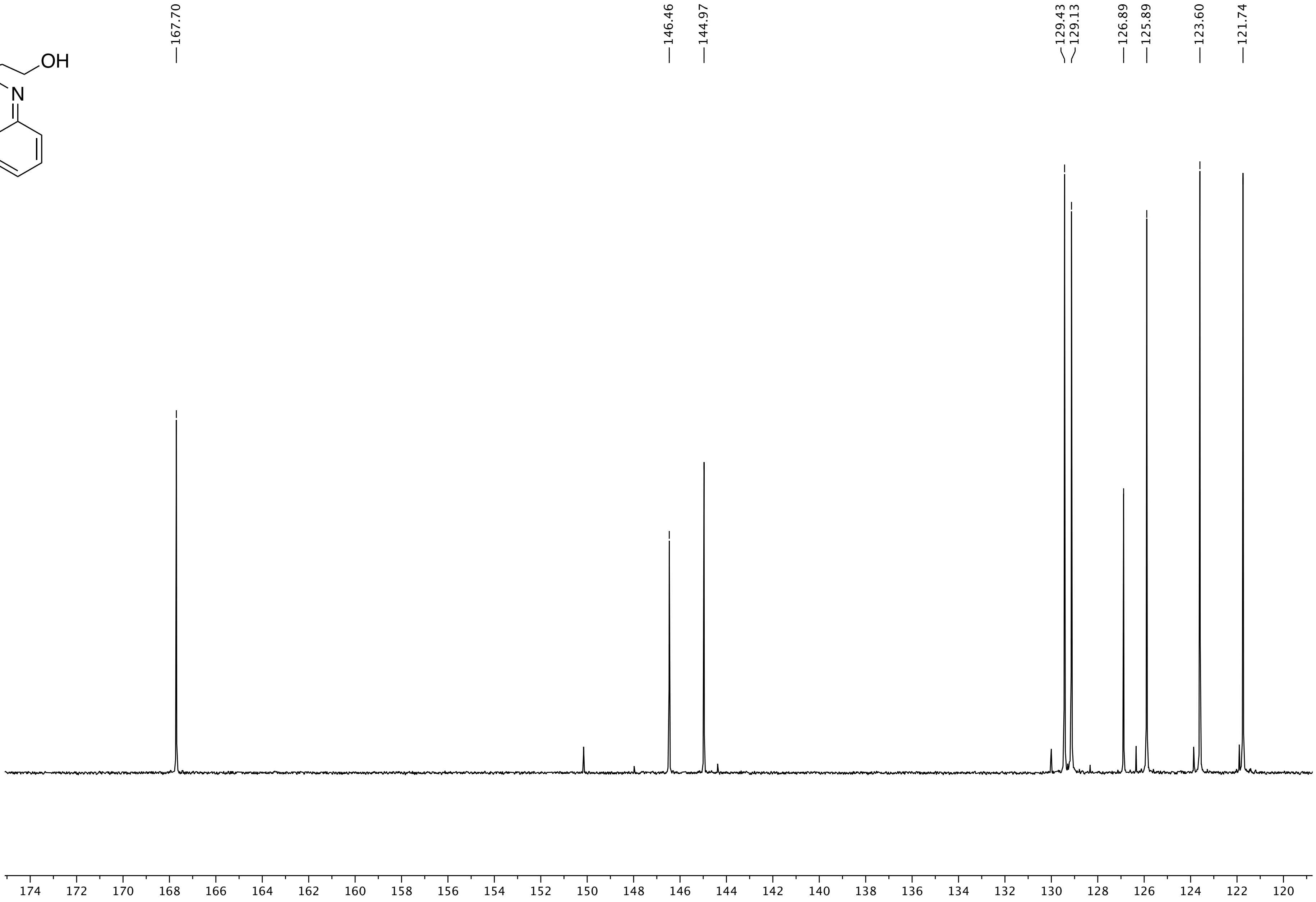

2-(6,6-Dimethyl-3-(4-methylquinolin-2-yl)bicyclo[3.1.1]heptan-2-yl)ethan-1-ol (27)

^{13}C-NMR (101 MHz, CDCl$_3$)

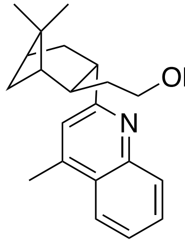

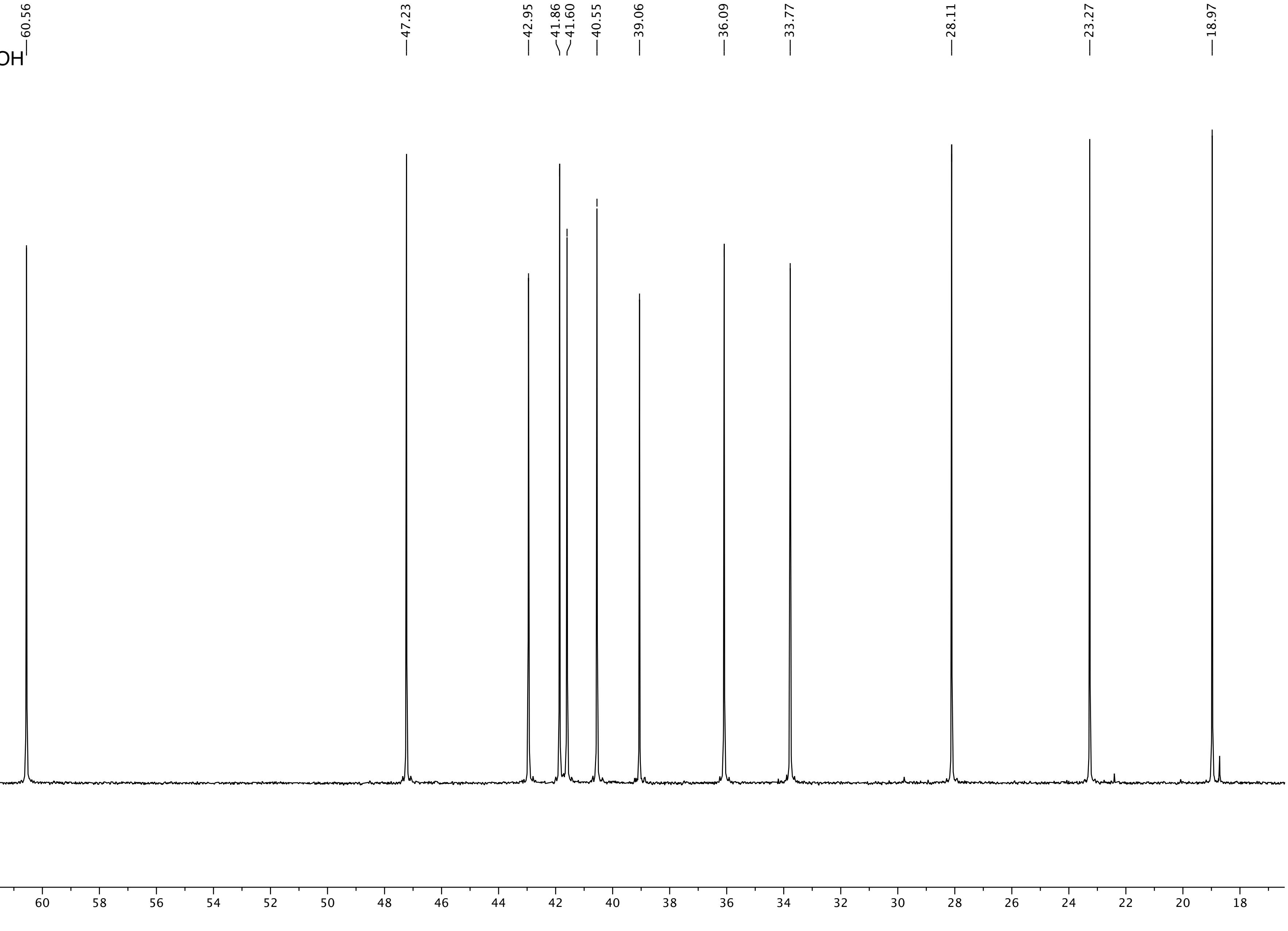

2-(6,6-Dimethyl-3-(4-methylquinolin-2-yl)bicyclo[3.1.1]heptan-2-yl)ethan-1-ol (27)

Dept-135 (101 MHz, $CDCl_3$)

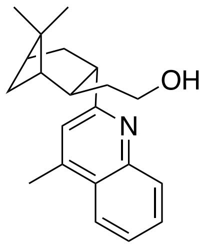

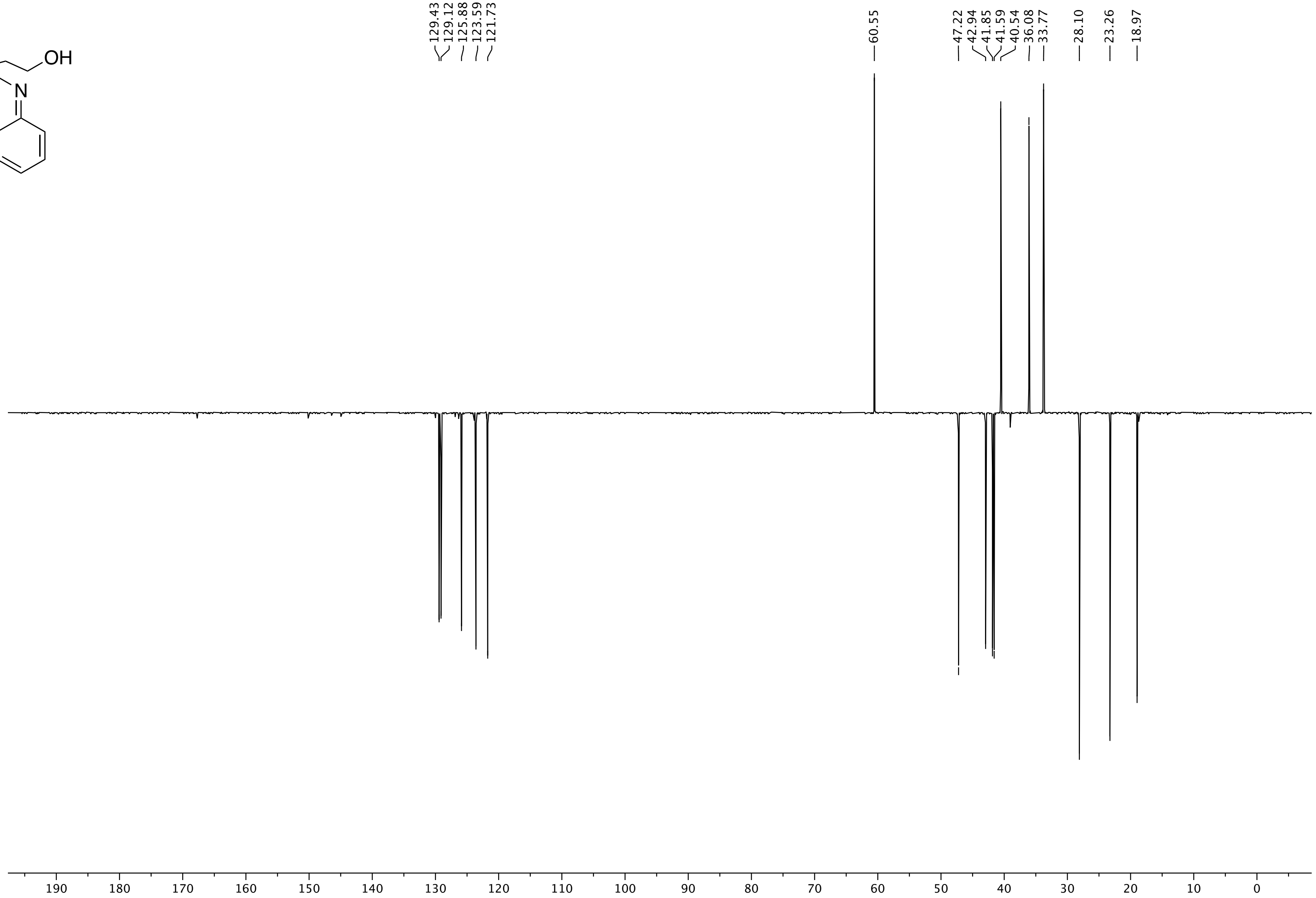

2-(6,6-Dimethyl-3-(4-methylquinolin-2-yl)bicyclo[3.1.1]heptan-2-yl)ethan-1-ol (27)

^1H-^1H COSY (400 MHz, $CDCl_3$)

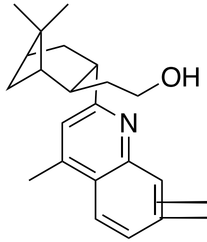

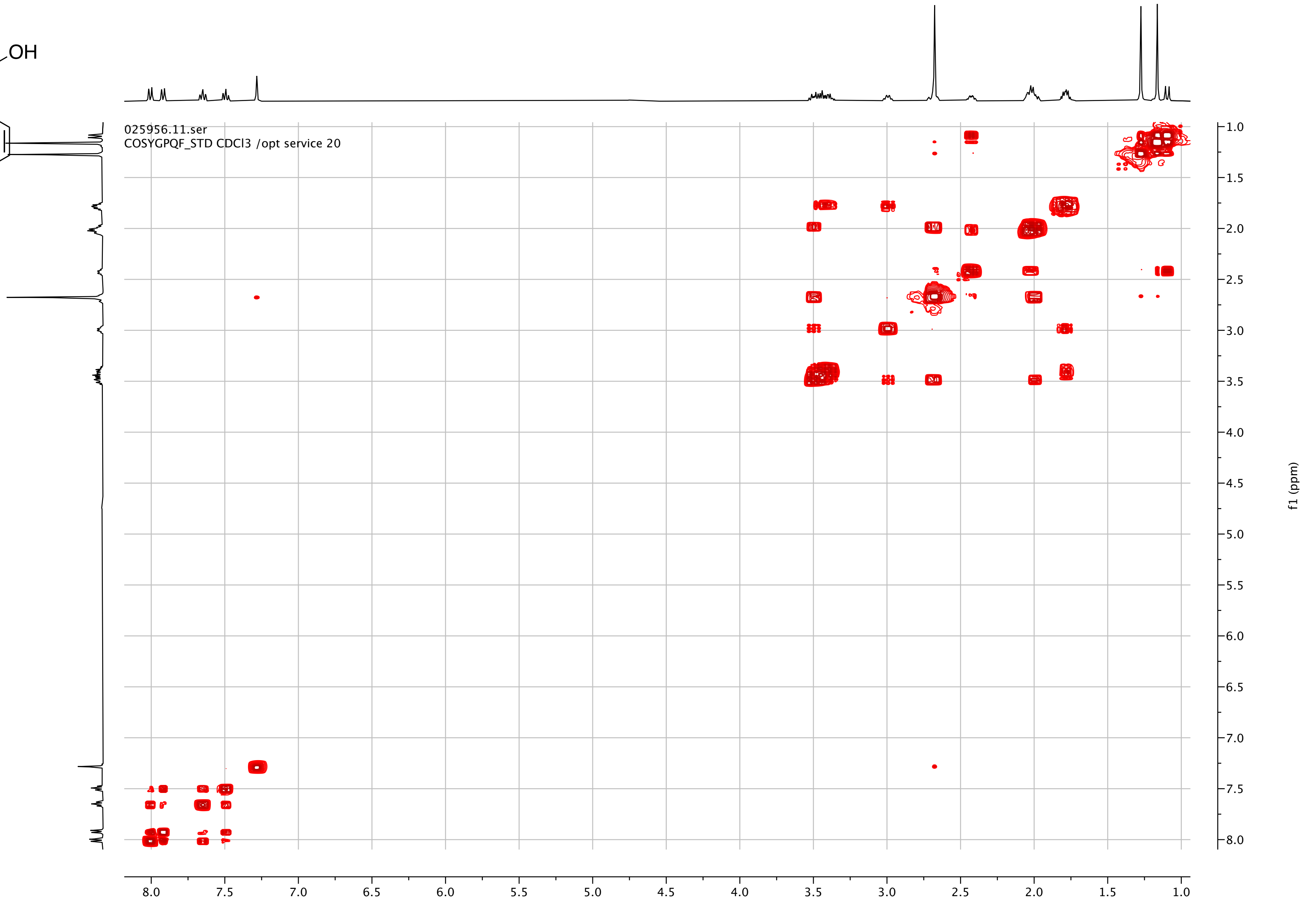

2-(6,6-Dimethyl-3-(4-methylquinolin-2-yl)bicyclo[3.1.1]heptan-2-yl)ethan-1-ol (27)

^1H-^1H COSY (400 MHz, $CDCl_3$)

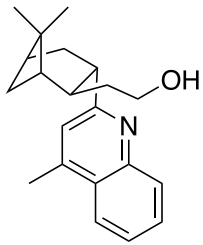

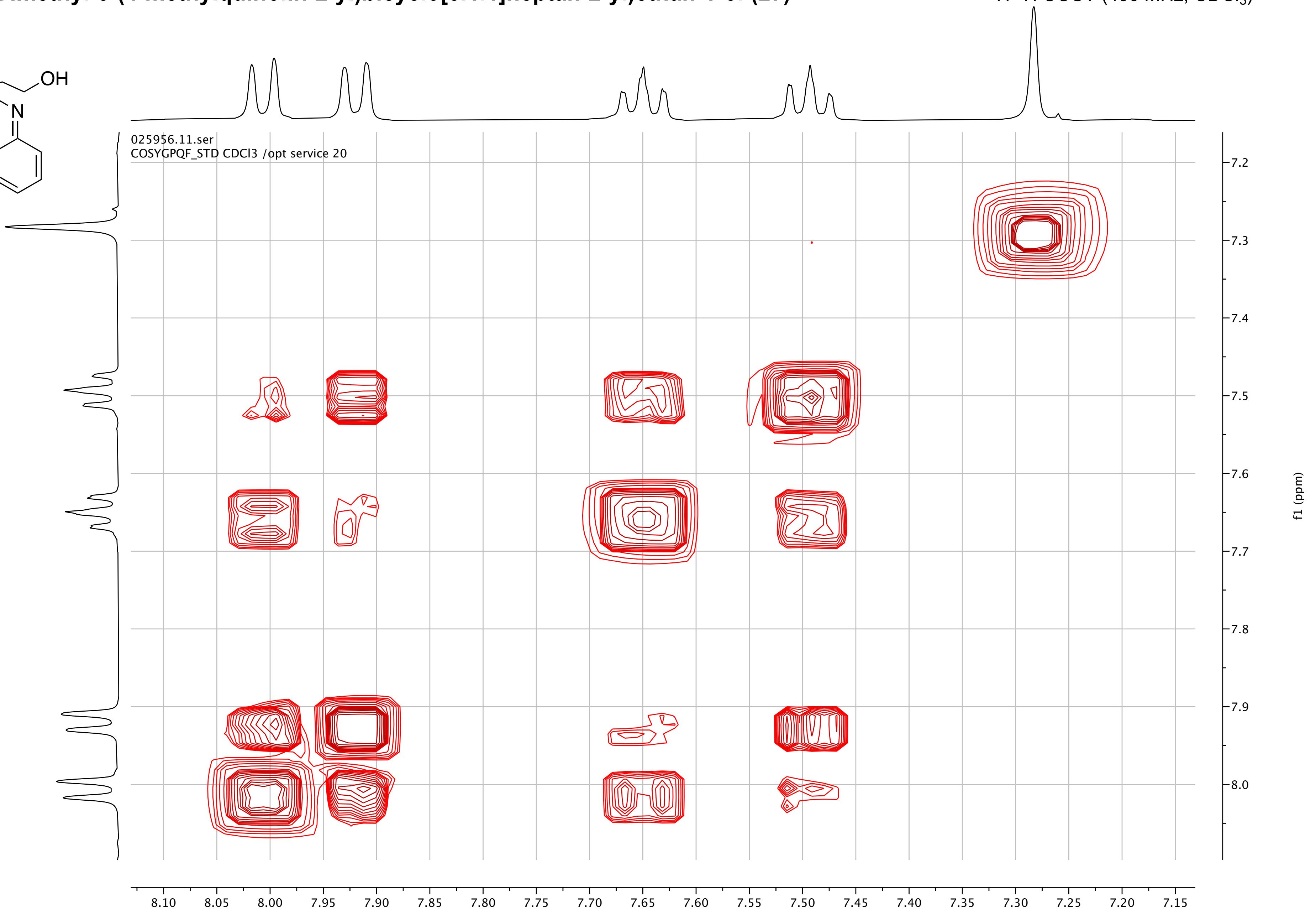

2-(6,6-Dimethyl-3-(4-methylquinolin-2-yl)bicyclo[3.1.1]heptan-2-yl)ethan-1-ol (27)

^{1}H-^{1}H COSY (400 MHz, $CDCl_3$)

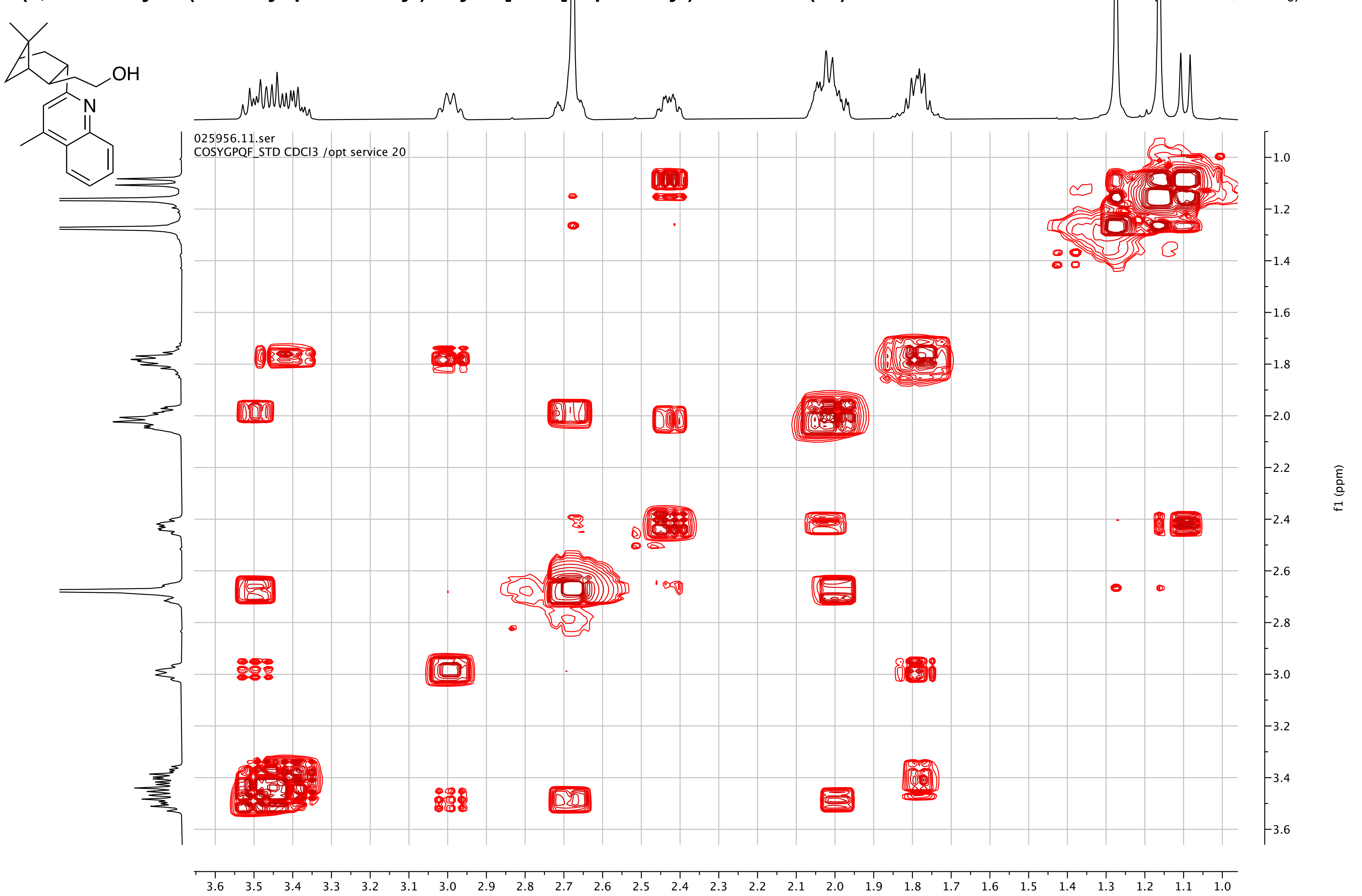

2-(6,6-Dimethyl-3-(4-methylquinolin-2-yl)bicyclo[3.1.1]heptan-2-yl)ethan-1-ol (27)

HSQC (400 MHz, $CDCl_3$)

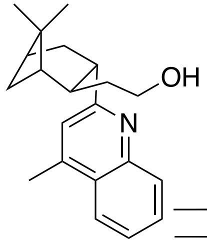

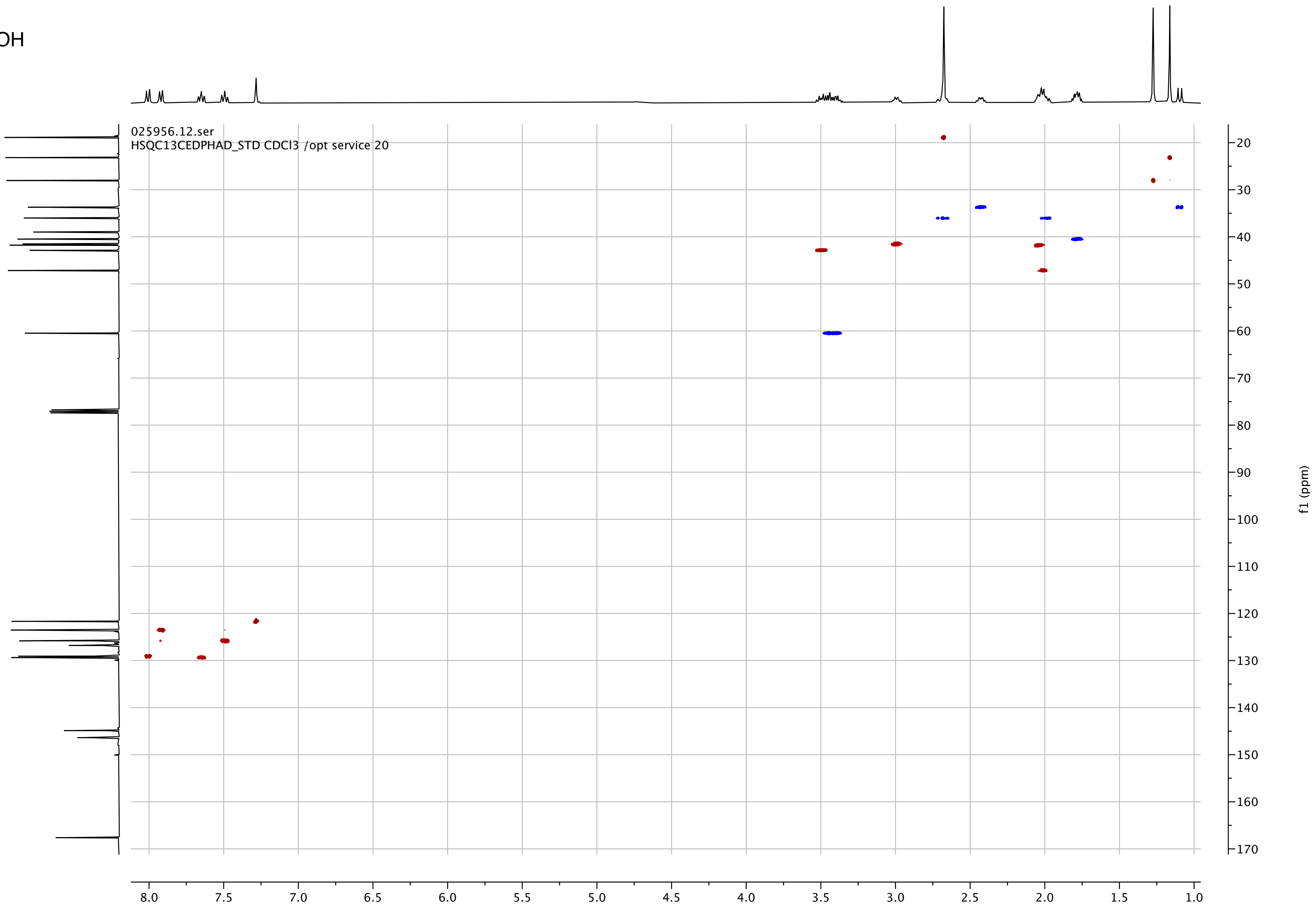

2-(6,6-Dimethyl-3-(4-methylquinolin-2-yl)bicyclo[3.1.1]heptan-2-yl)ethan-1-ol (27)

HSQC (400 MHz, $CDCl_3$)

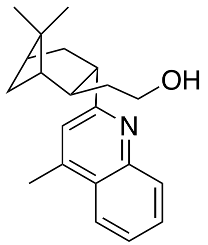

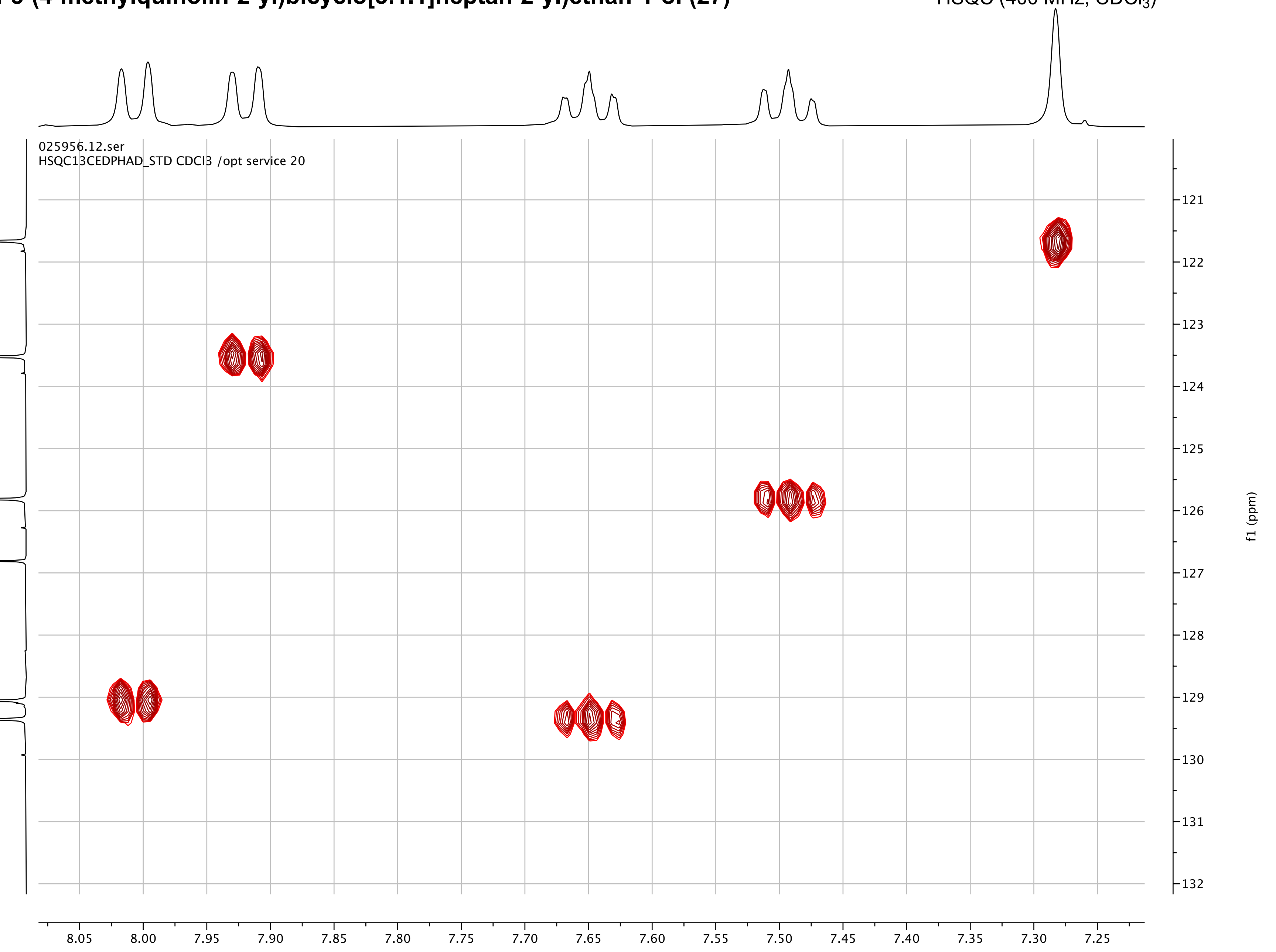

2-(6,6-Dimethyl-3-(4-methylquinolin-2-yl)bicyclo[3.1.1]heptan-2-yl)ethan-1-ol (27)

HSQC (400 MHz, $CDCl_3$)

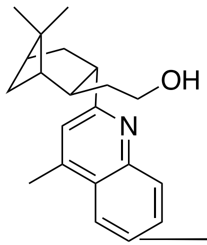

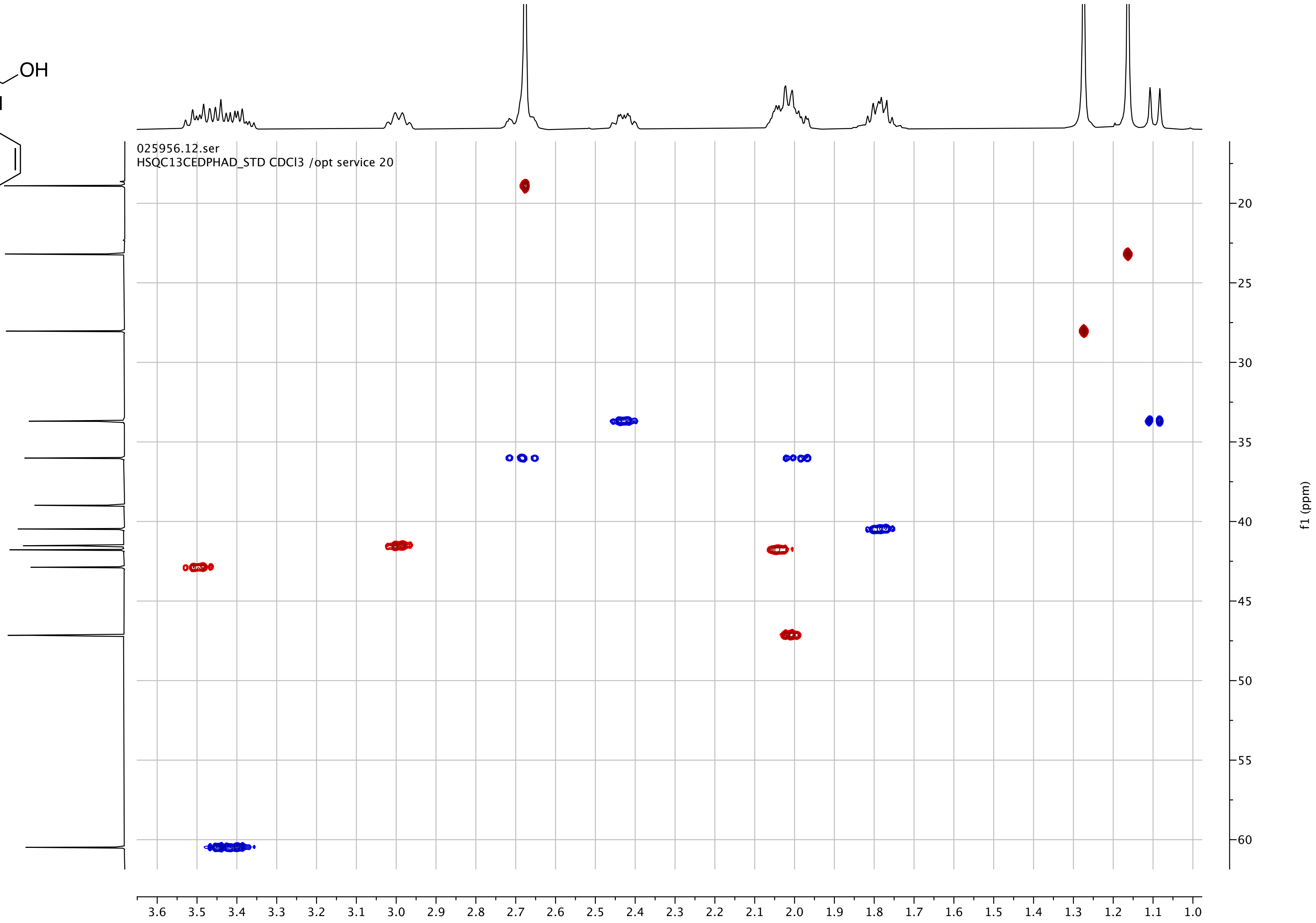

2-(6,6-Dimethyl-3-(4-methylquinolin-2-yl)bicyclo[3.1.1]heptan-2-yl)ethan-1-ol (27)

HMBC (400 MHz, $CDCl_3$)

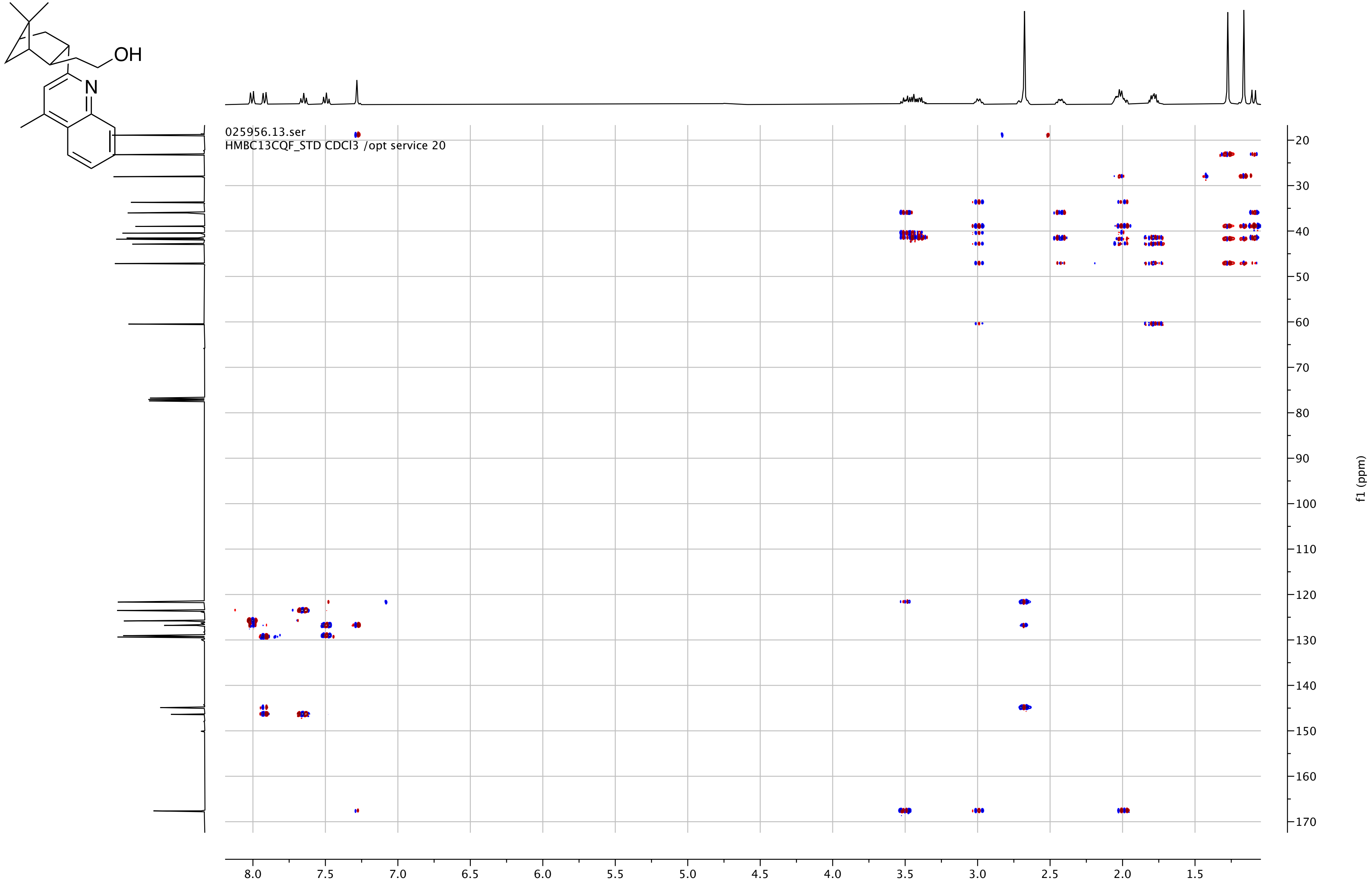

2-(6,6-Dimethyl-3-(4-methylquinolin-2-yl)bicyclo[3.1.1]heptan-2-yl)ethan-1-ol (27)

HMBC (400 MHz, $CDCl_3$)

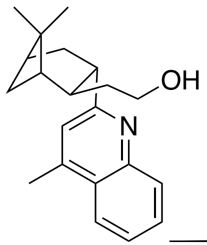

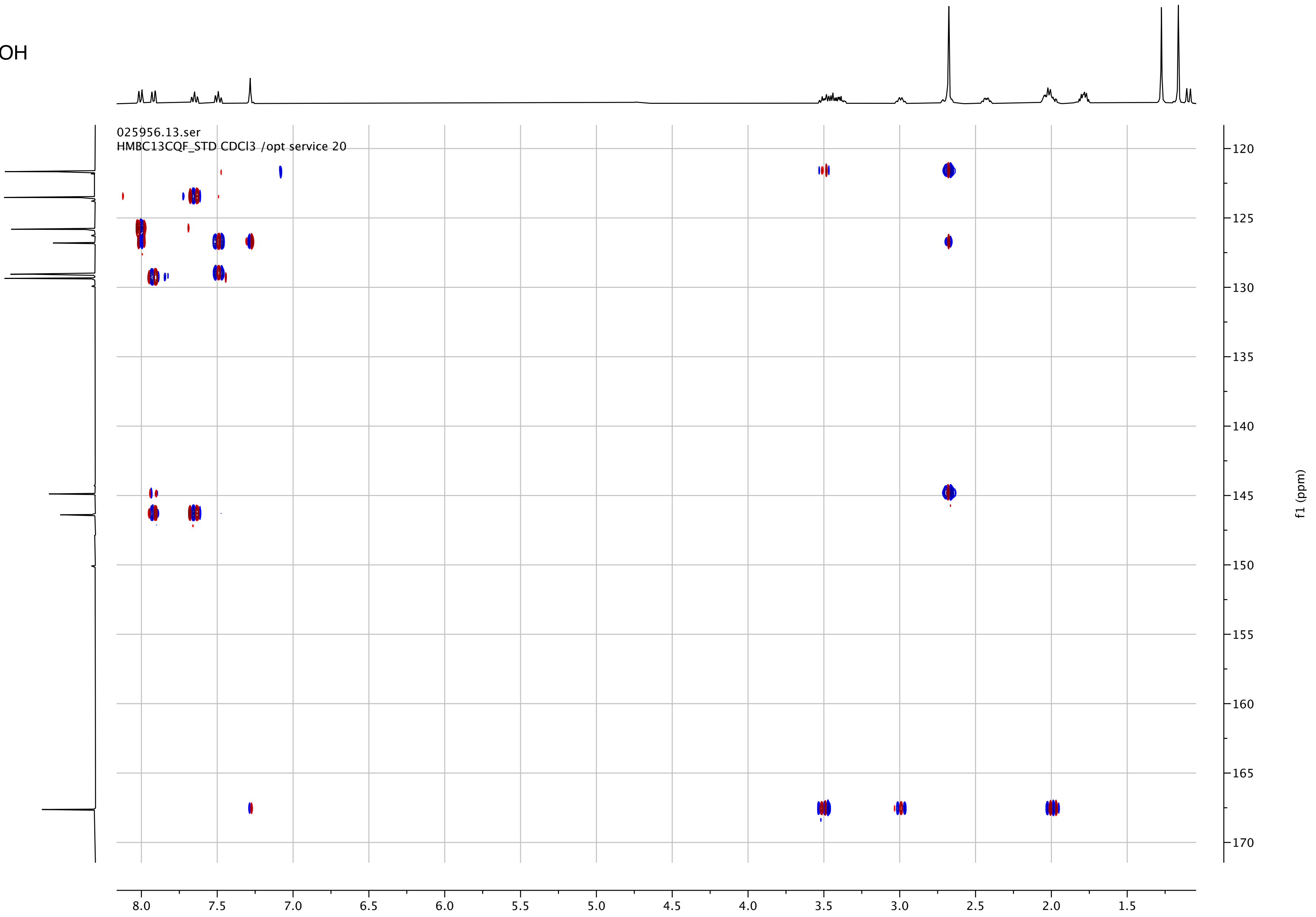

2-(6,6-Dimethyl-3-(4-methylquinolin-2-yl)bicyclo[3.1.1]heptan-2-yl)ethan-1-ol (27)

HMBC (400 MHz, $CDCl_3$)

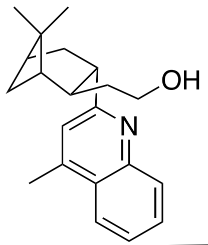

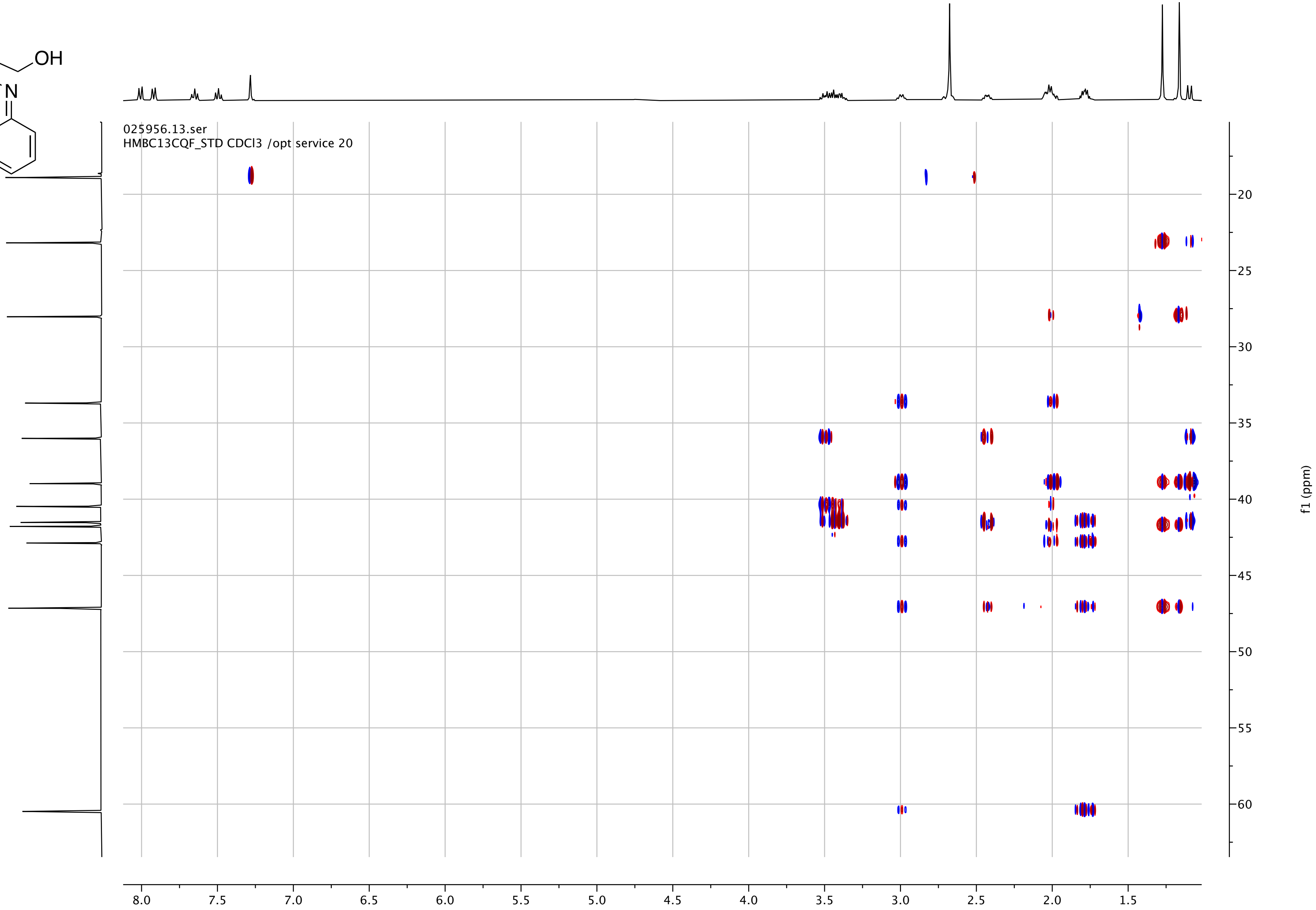

2-(6,6-Dimethyl-3-(4-methylquinolin-2-yl)bicyclo[3.1.1]heptan-2-yl)ethan-1-ol (27)

HMBC (400 MHz, $CDCl_3$)

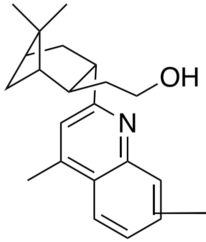

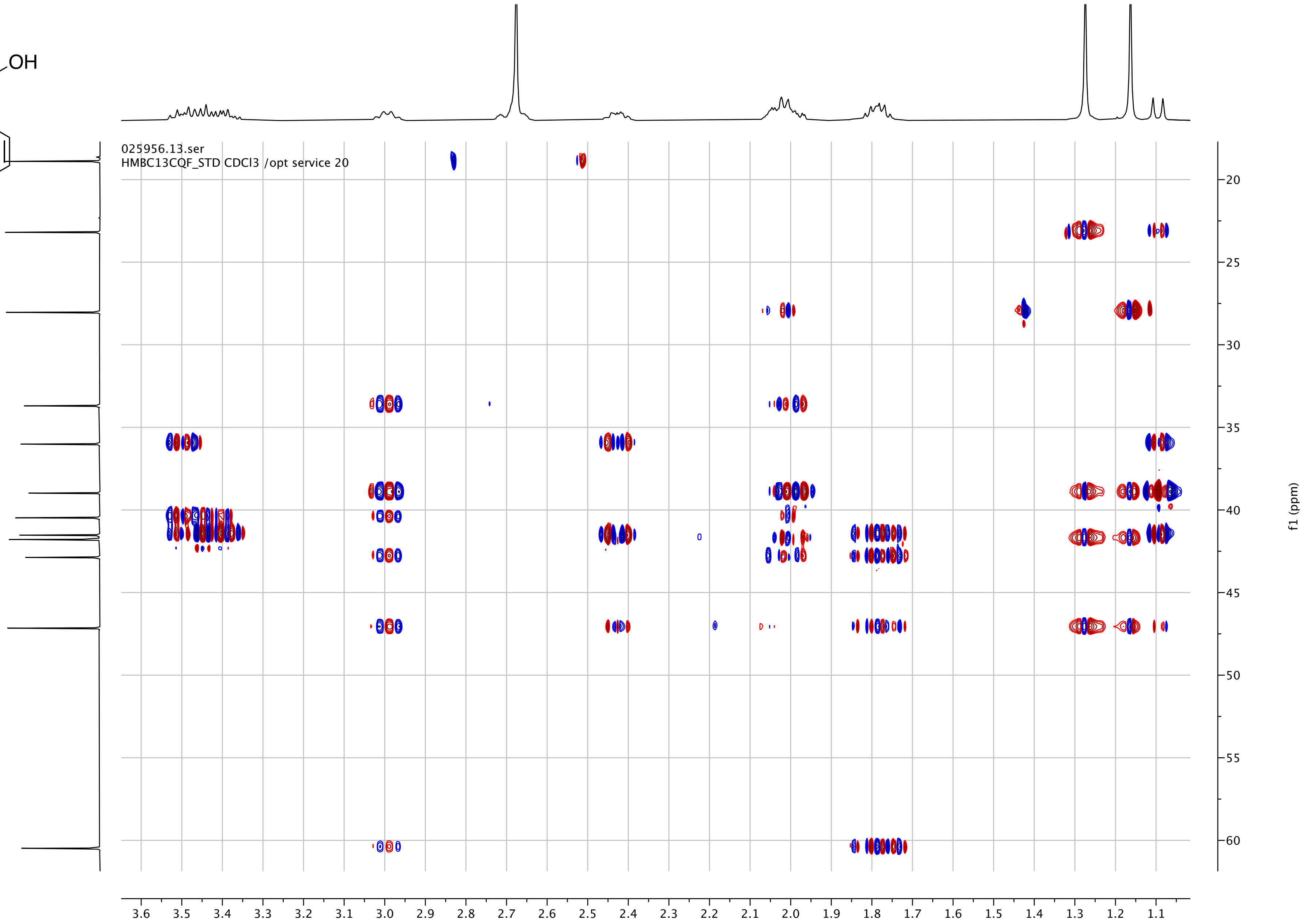

2-(((1*S*,4*R*)-3,3-Dimethylbicyclo[2.2.1]heptan-2-yl)methyl)-4-methylquinoline (28)

^{1}H-NMR (400 MHz, $CDCl_3$)

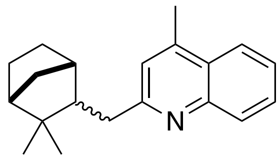

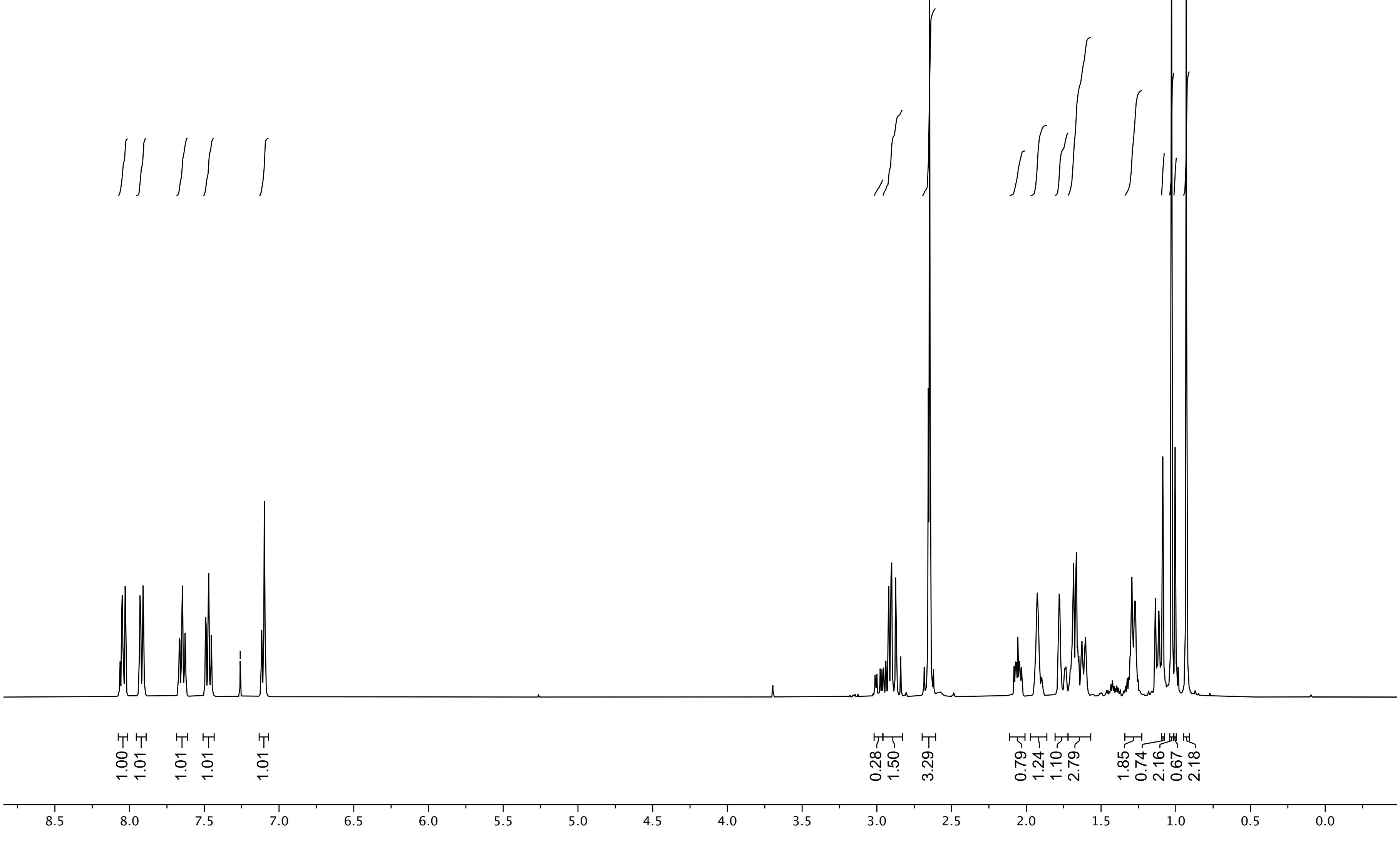

2-(((1*S*,4*R*)-3,3-Dimethylbicyclo[2.2.1]heptan-2-yl)methyl)-4-methylquinoline (28)

^{1}H-NMR (400 MHz, $CDCl_3$)

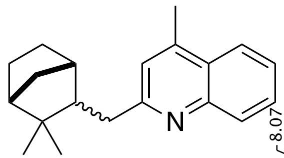

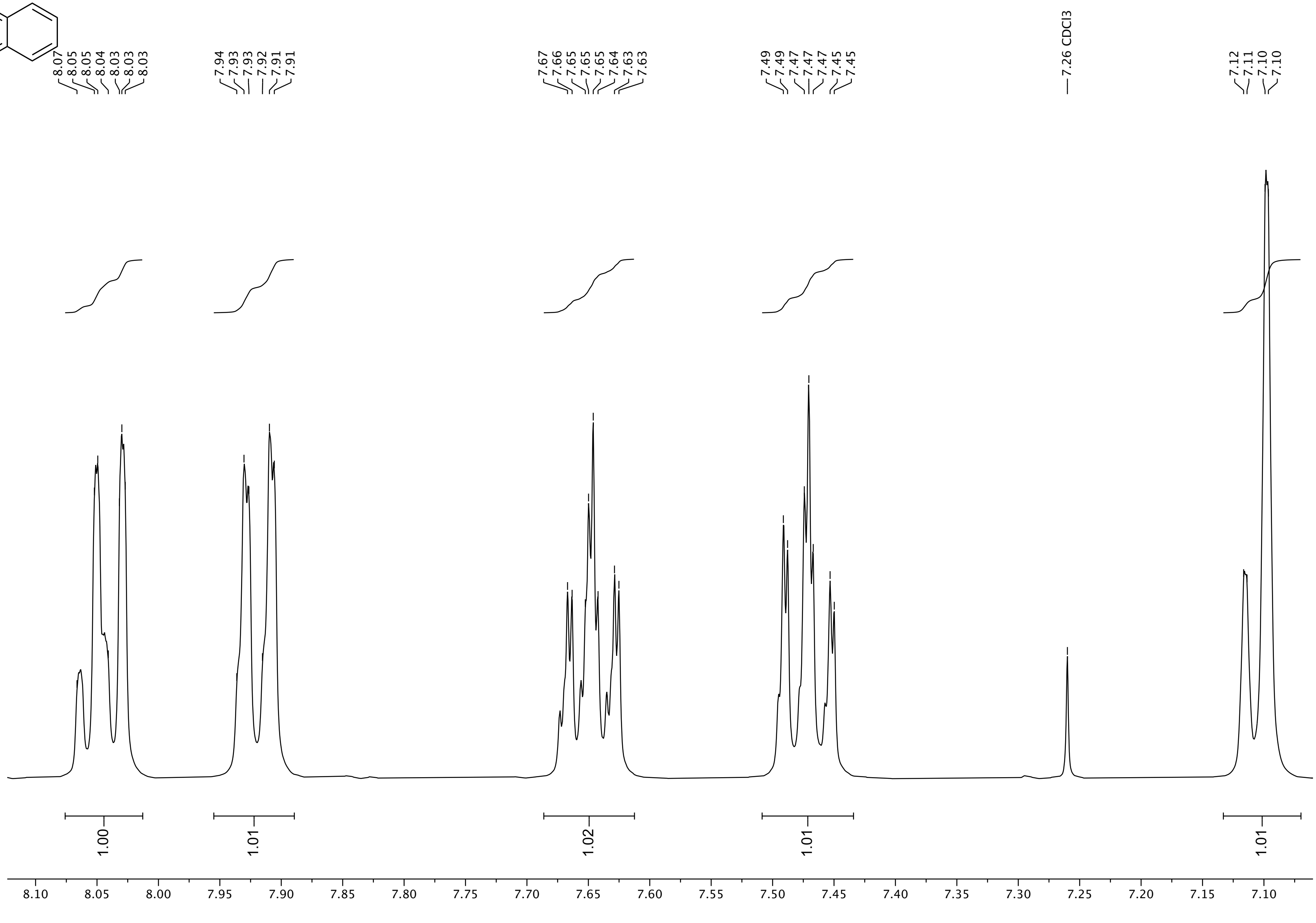

2-(((1*S*,4*R*)-3,3-Dimethylbicyclo[2.2.1]heptan-2-yl)methyl)-4-methylquinoline (28)

^{1}H-NMR (400 MHz, $CDCl_3$)

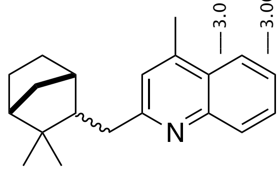

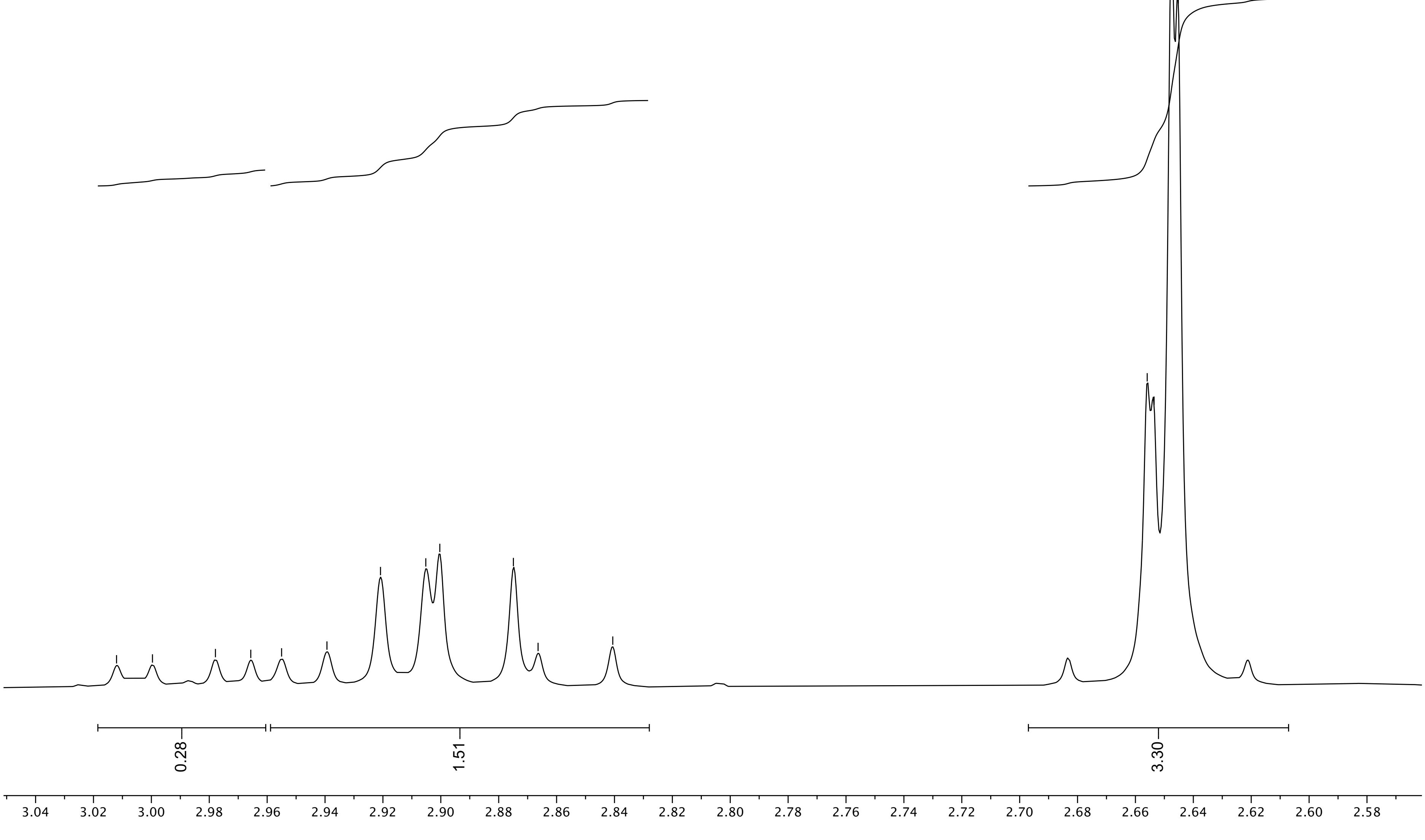

2-(((1*S*,4*R*)-3,3-Dimethylbicyclo[2.2.1]heptan-2-yl)methyl)-4-methylquinoline (28)

^{1}H-NMR (400 MHz, $CDCl_3$)

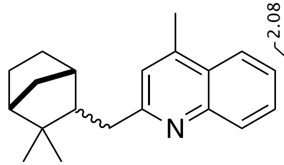

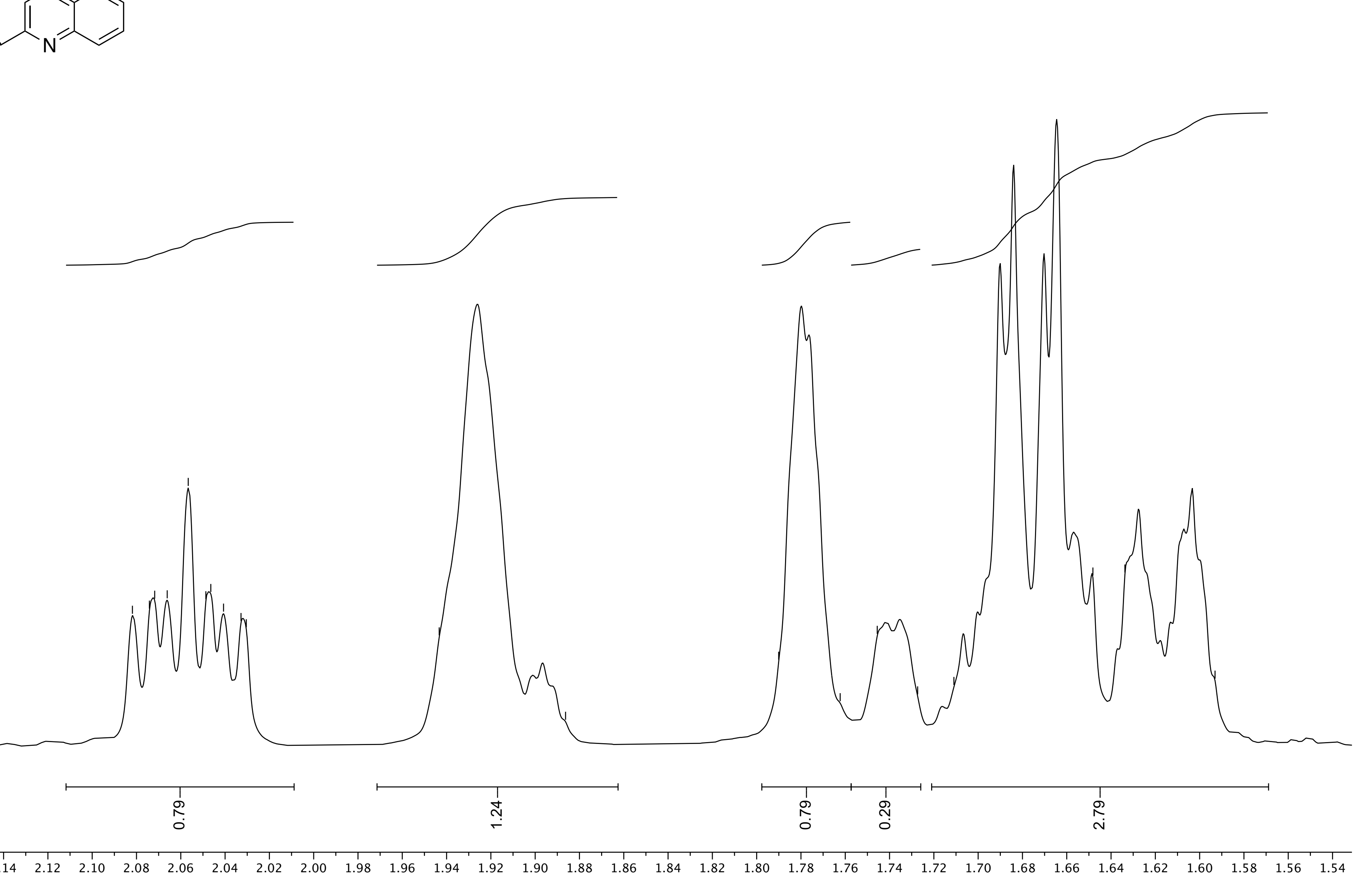

2-(((1*S*,4*R*)-3,3-Dimethylbicyclo[2.2.1]heptan-2-yl)methyl)-4-methylquinoline (28)

^{1}H-NMR (400 MHz, $CDCl_3$)

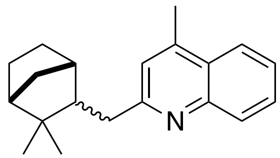

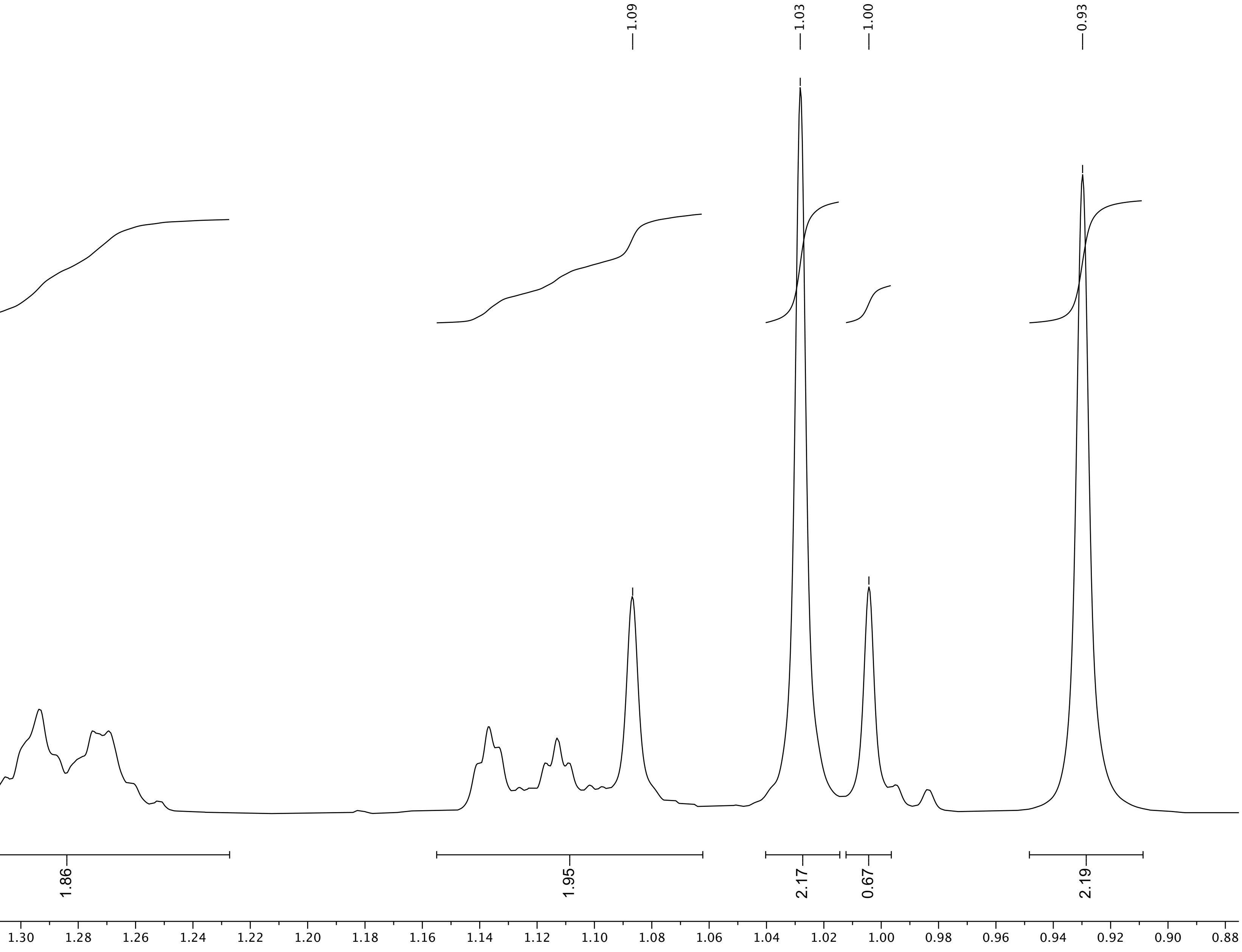

2-(((1*S*,4*R*)-3,3-Dimethylbicyclo[2.2.1]heptan-2-yl)methyl)-4-methylquinoline (28)

^{13}C-NMR (101 MHz, $CDCl_3$)

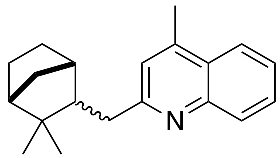

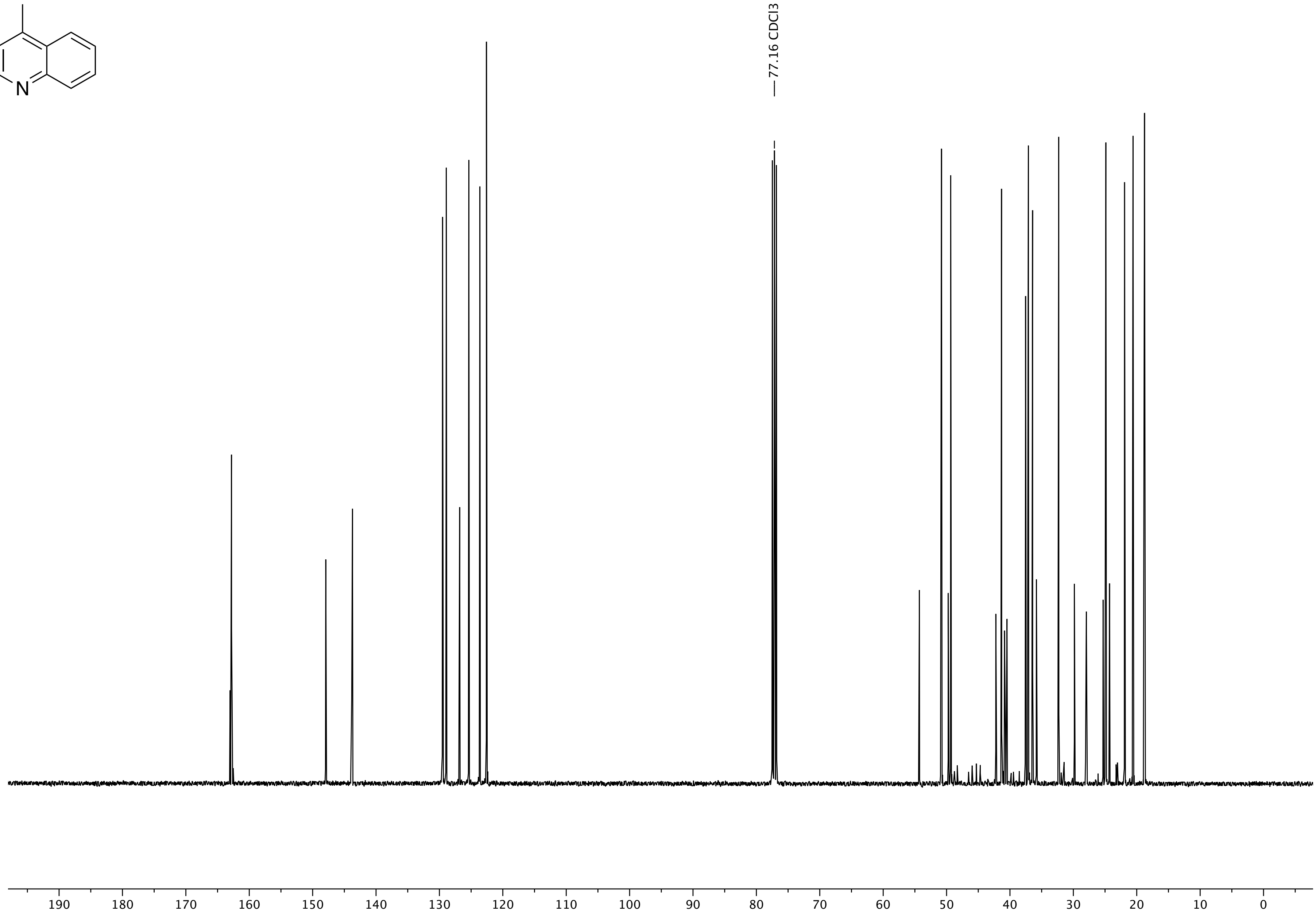

2-(((1*S*,4*R*)-3,3-Dimethylbicyclo[2.2.1]heptan-2-yl)methyl)-4-methylquinoline (28)

^{13}C-NMR (101 MHz, $CDCl_3$)

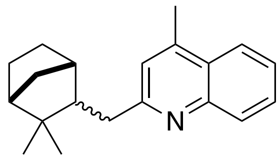

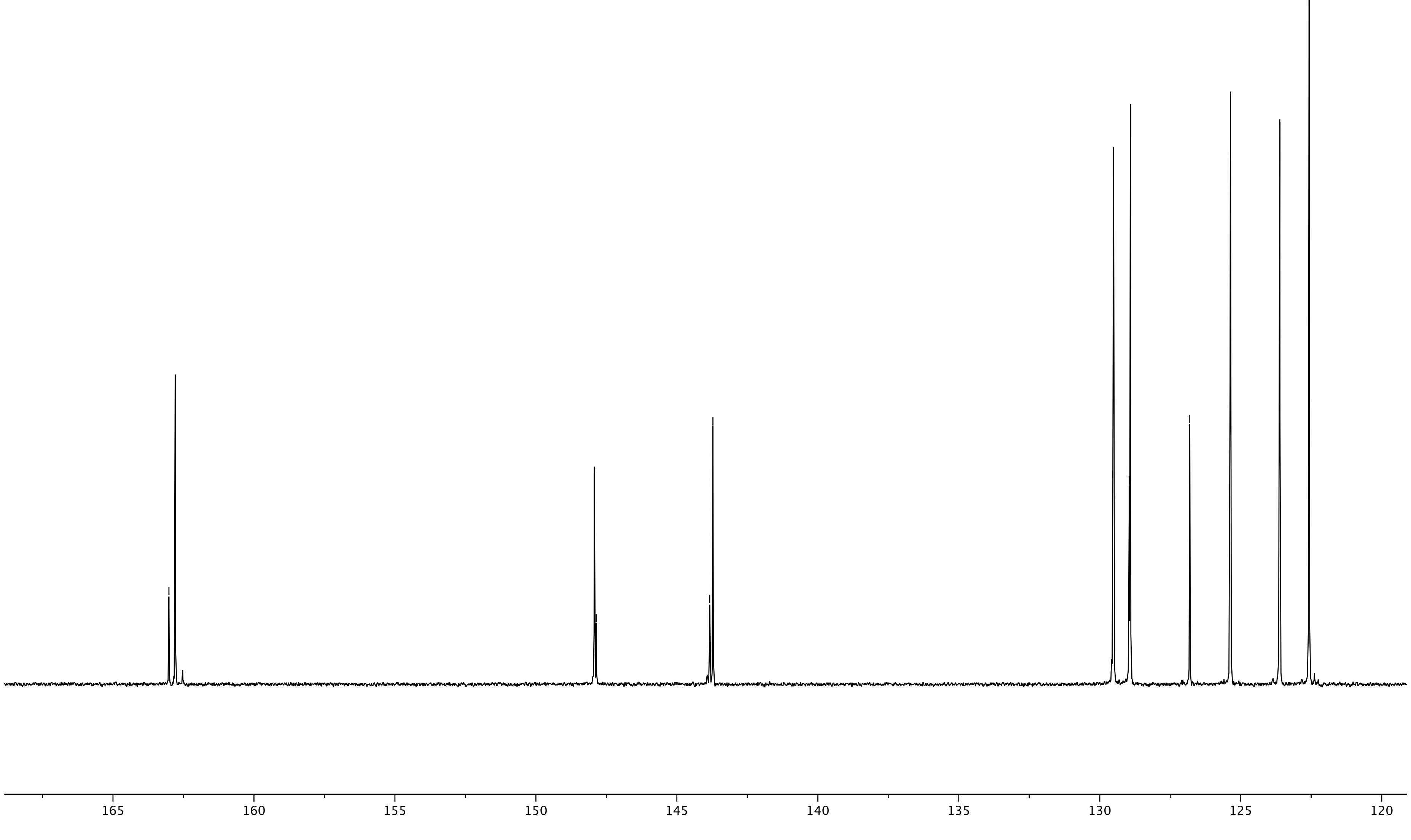

2-(((1*S*,4*R*)-3,3-Dimethylbicyclo[2.2.1]heptan-2-yl)methyl)-4-methylquinoline (28)

^{13}C-NMR (101 MHz, $CDCl_3$)

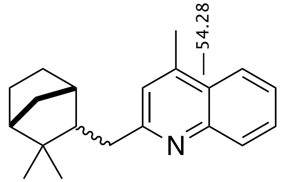

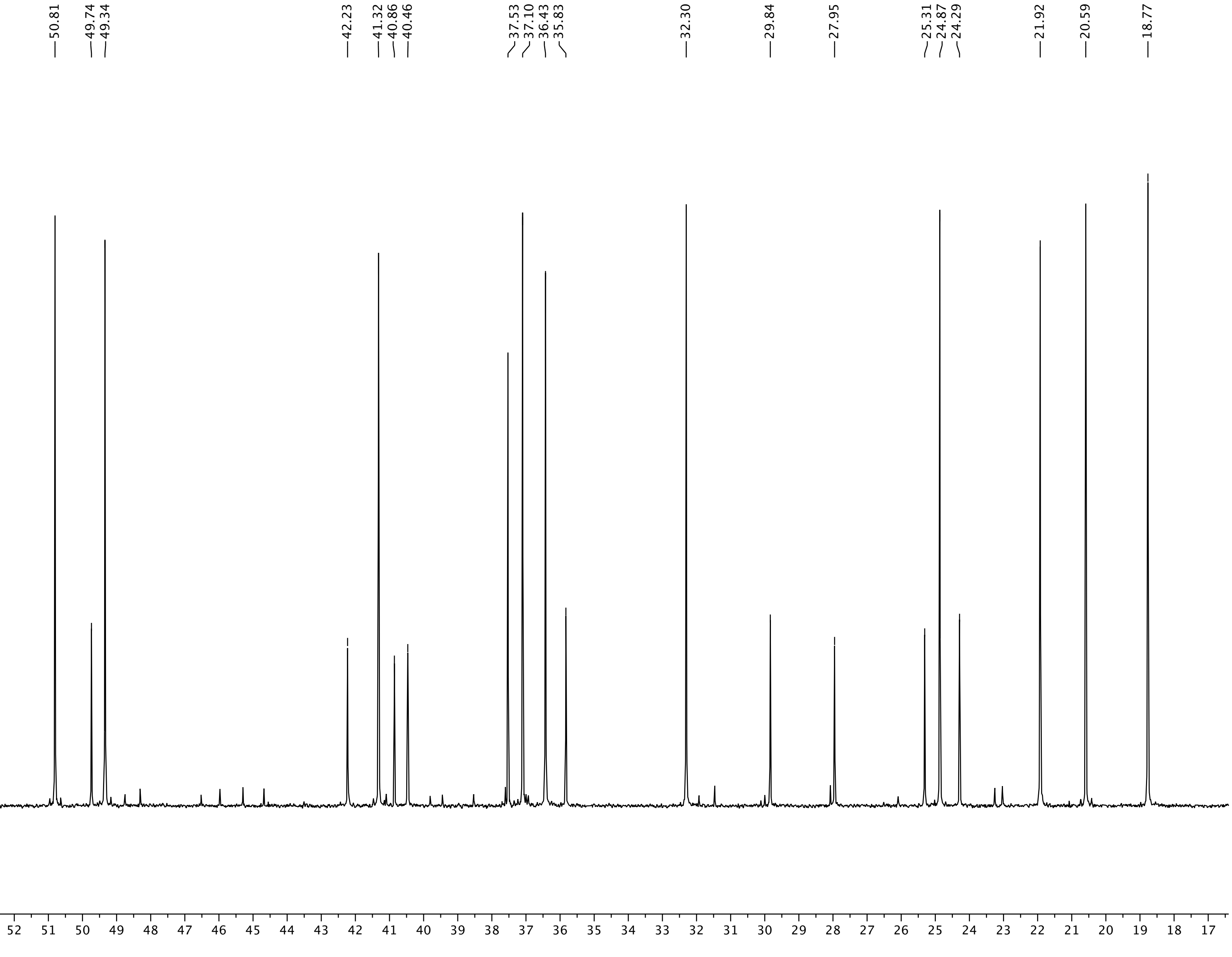

2-(((1*S*,4*R*)-3,3-Dimethylbicyclo[2.2.1]heptan-2-yl)methyl)-4-methylquinoline (28)

Dept-135 (101 MHz, $CDCl_3$)

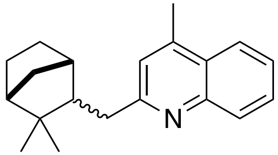

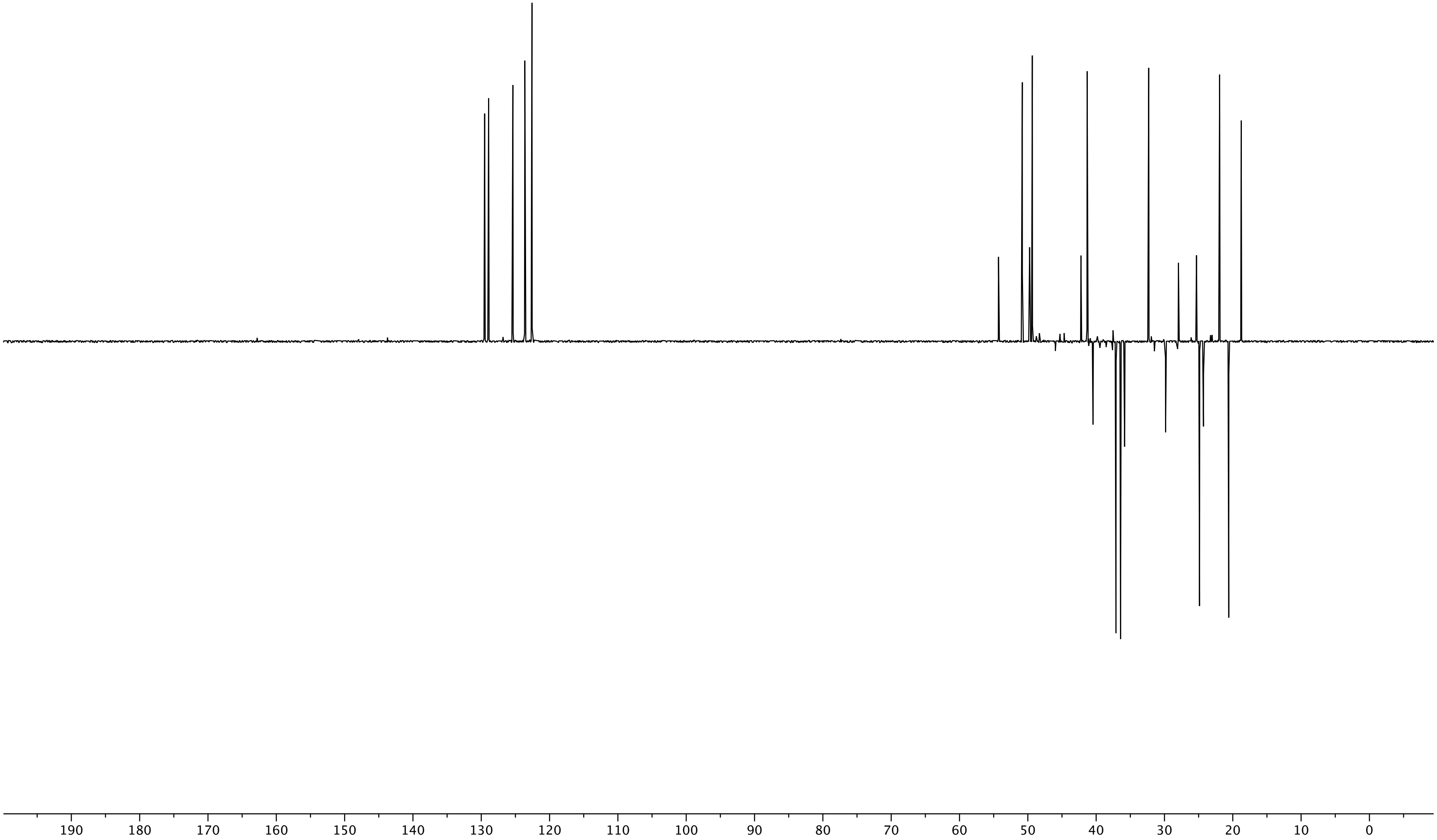

2-(((1*S*,4*R*)-3,3-Dimethylbicyclo[2.2.1]heptan-2-yl)methyl)-4-methylquinoline (28)

Dept-135 (101 MHz, $CDCl_3$)

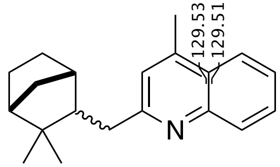

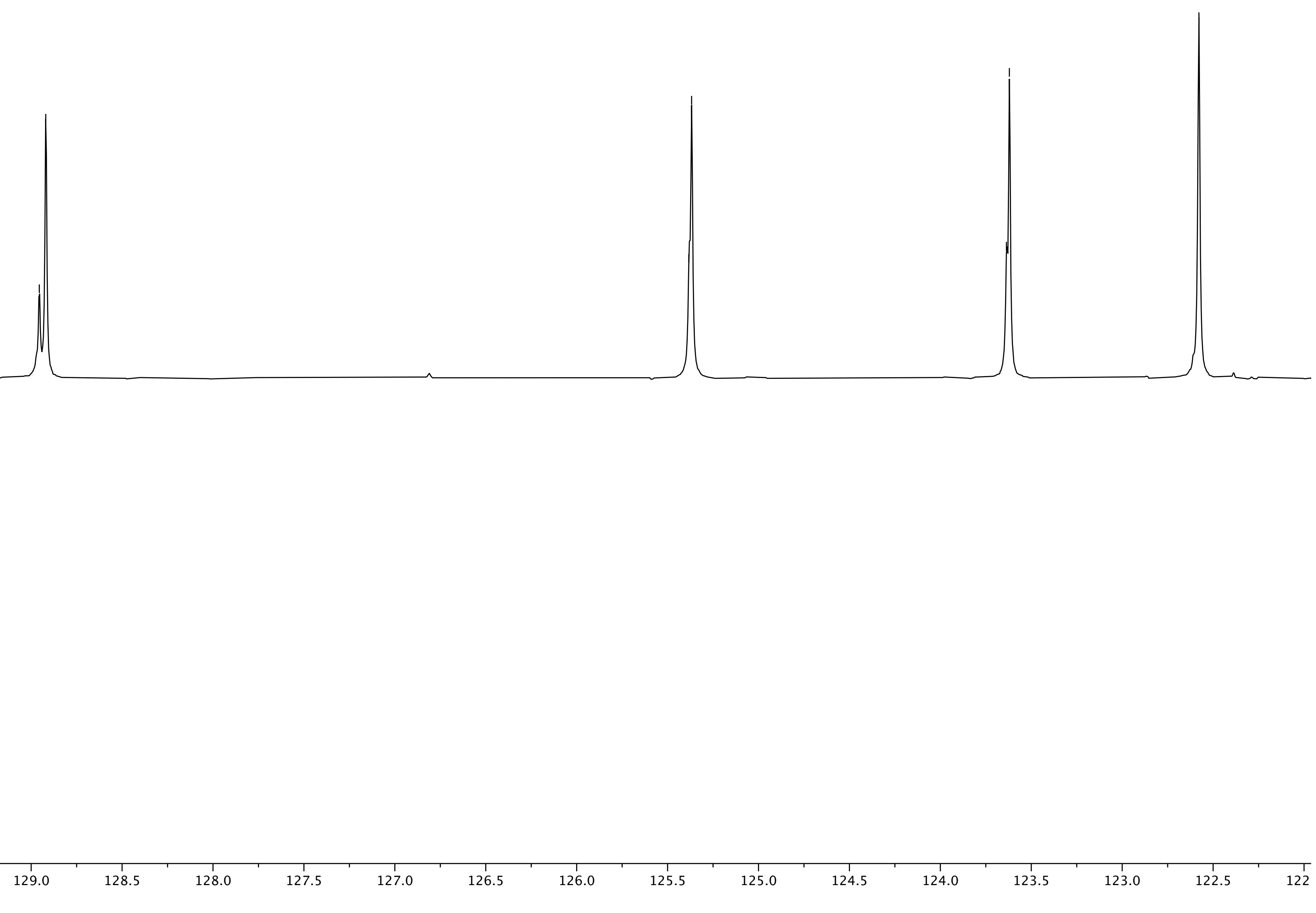

2-(((1*S*,4*R*)-3,3-Dimethylbicyclo[2.2.1]heptan-2-yl)methyl)-4-methylquinoline (28)

Dept-135 (101 MHz, $CDCl_3$)

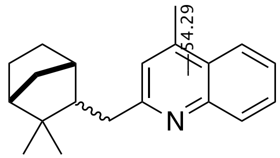

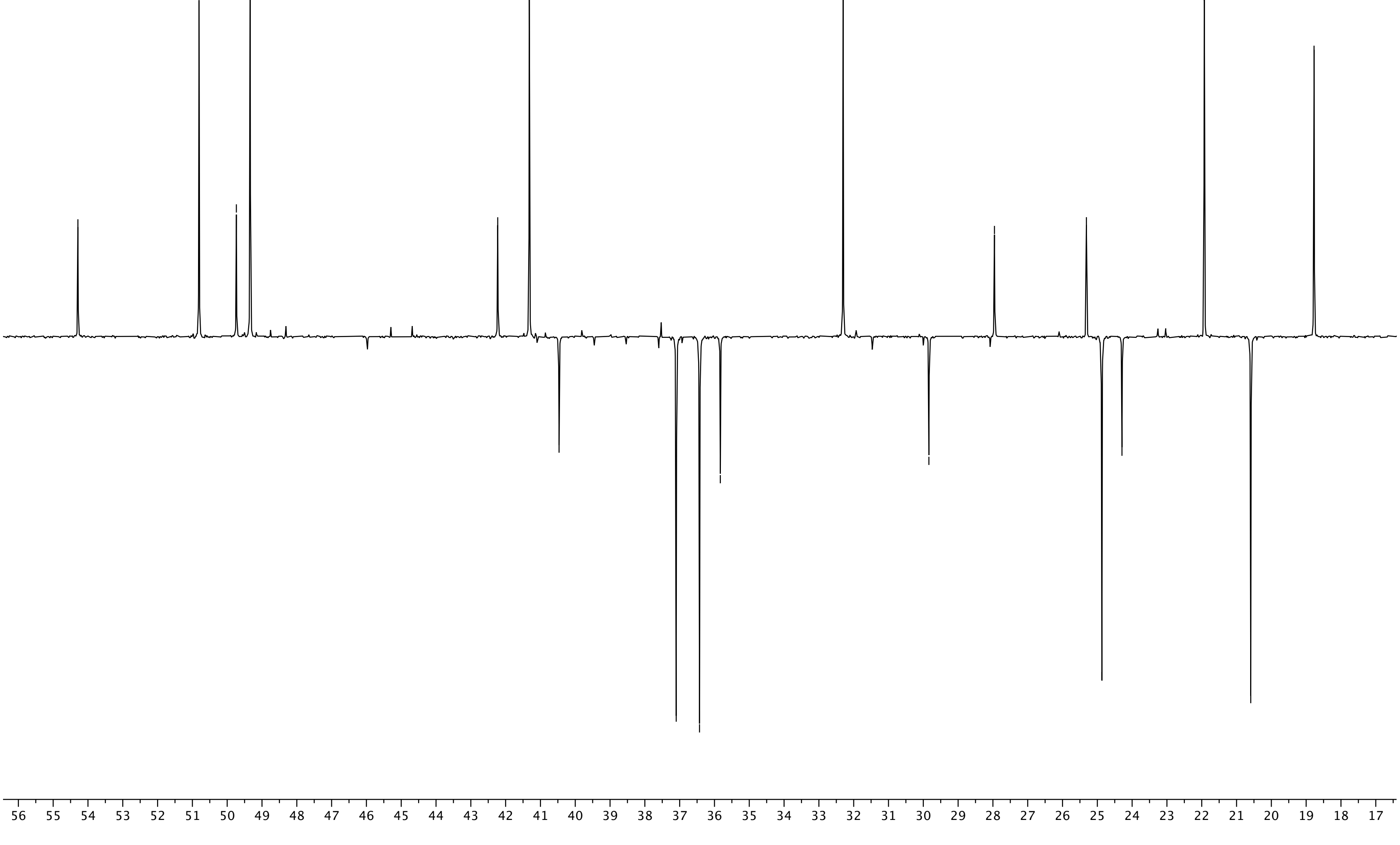

2-(((1*S*,4*R*)-3,3-Dimethylbicyclo[2.2.1]heptan-2-yl)methyl)-4-methylquinoline (28)

^{1}H-^{1}H COSY (400 MHz, $CDCl_3$)

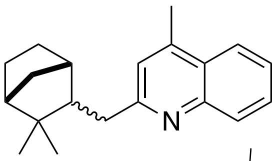

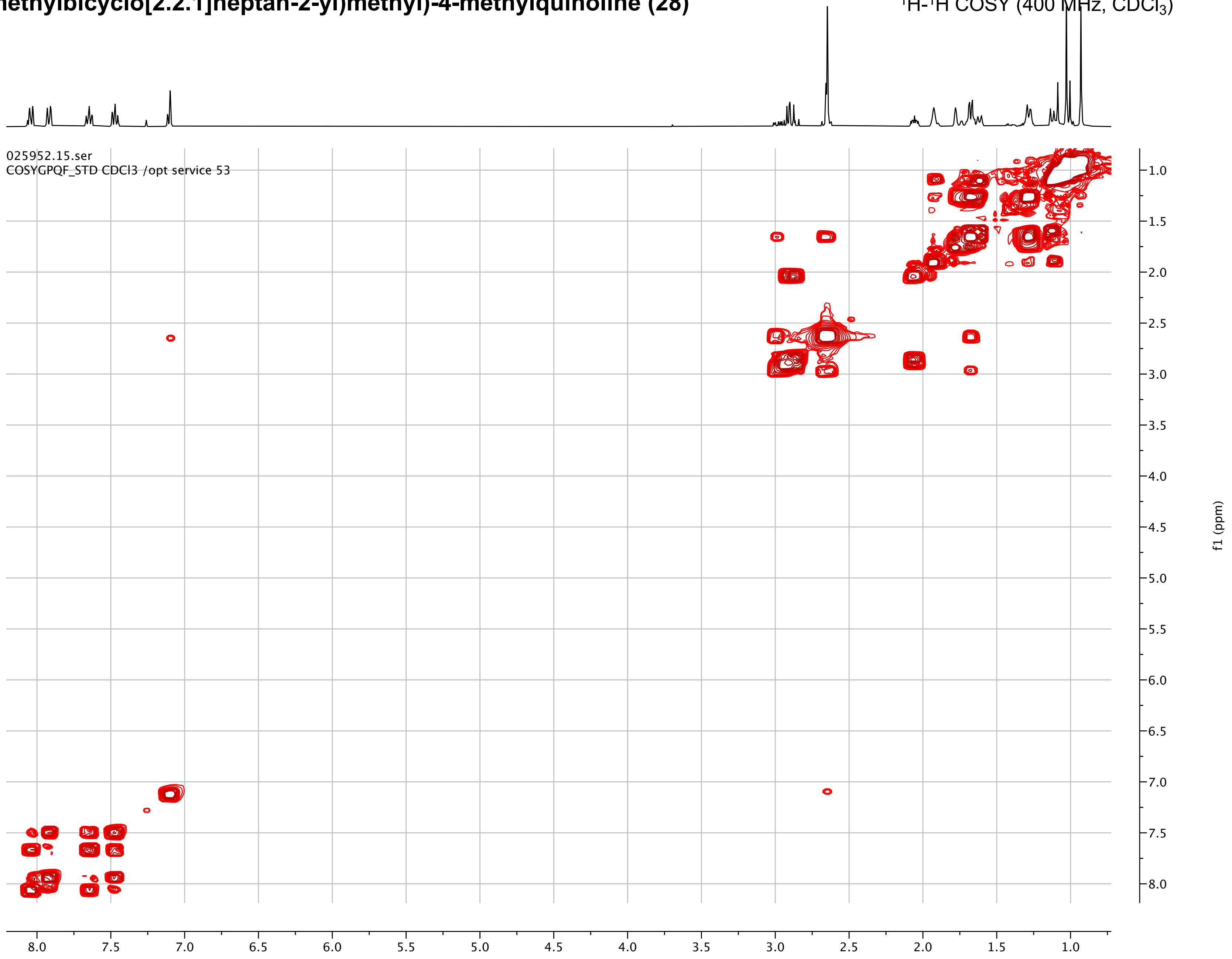

2-(((1*S*,4*R*)-3,3-Dimethylbicyclo[2.2.1]heptan-2-yl)methyl)-4-methylquinoline (28)

^{1}H-^{1}H COSY (400 MHz, $CDCl_3$)

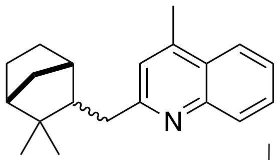

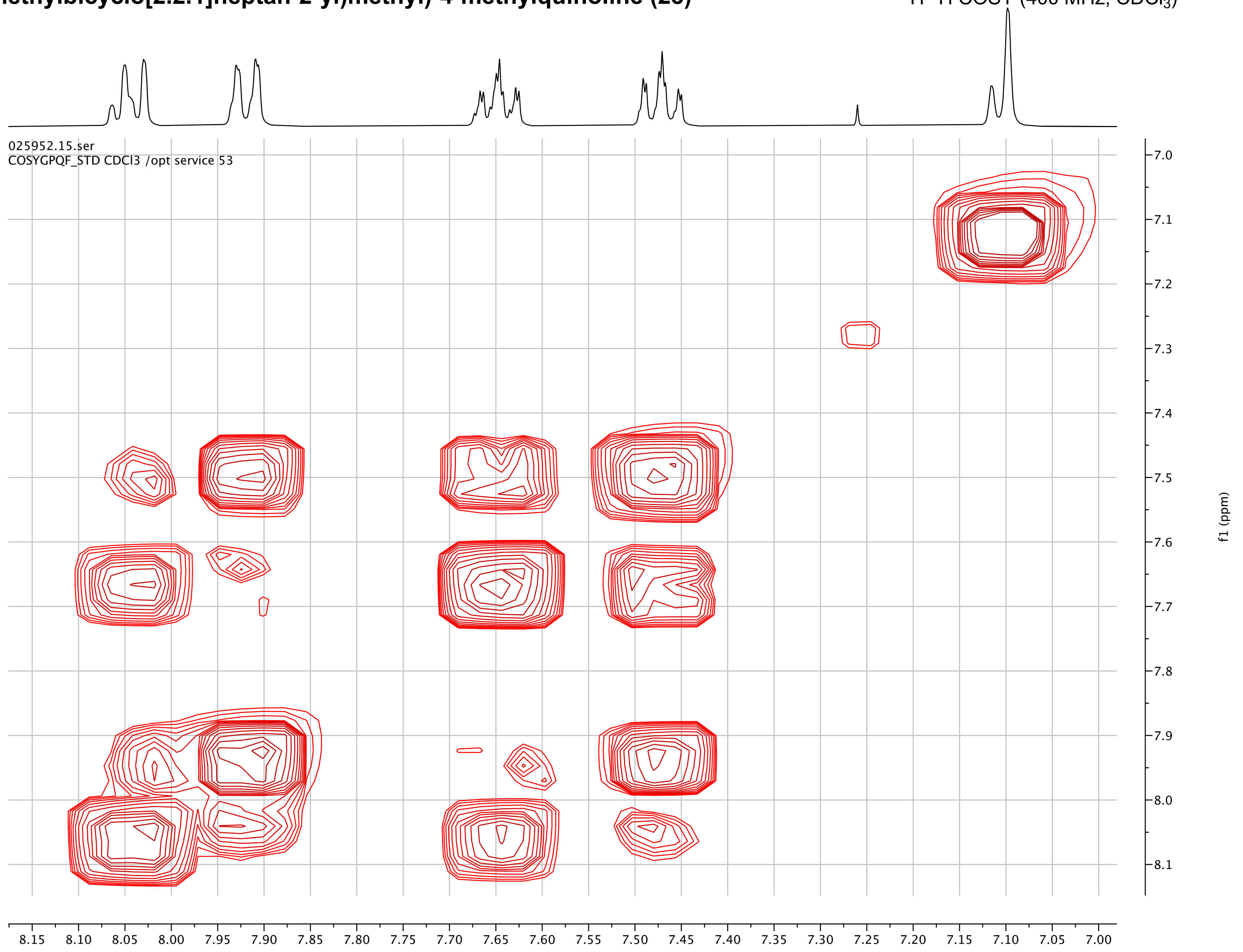

2-(((1*S*,4*R*)-3,3-Dimethylbicyclo[2.2.1]heptan-2-yl)methyl)-4-methylquinoline (28)

^{1}H-^{1}H COSY (400 MHz, $CDCl_3$)

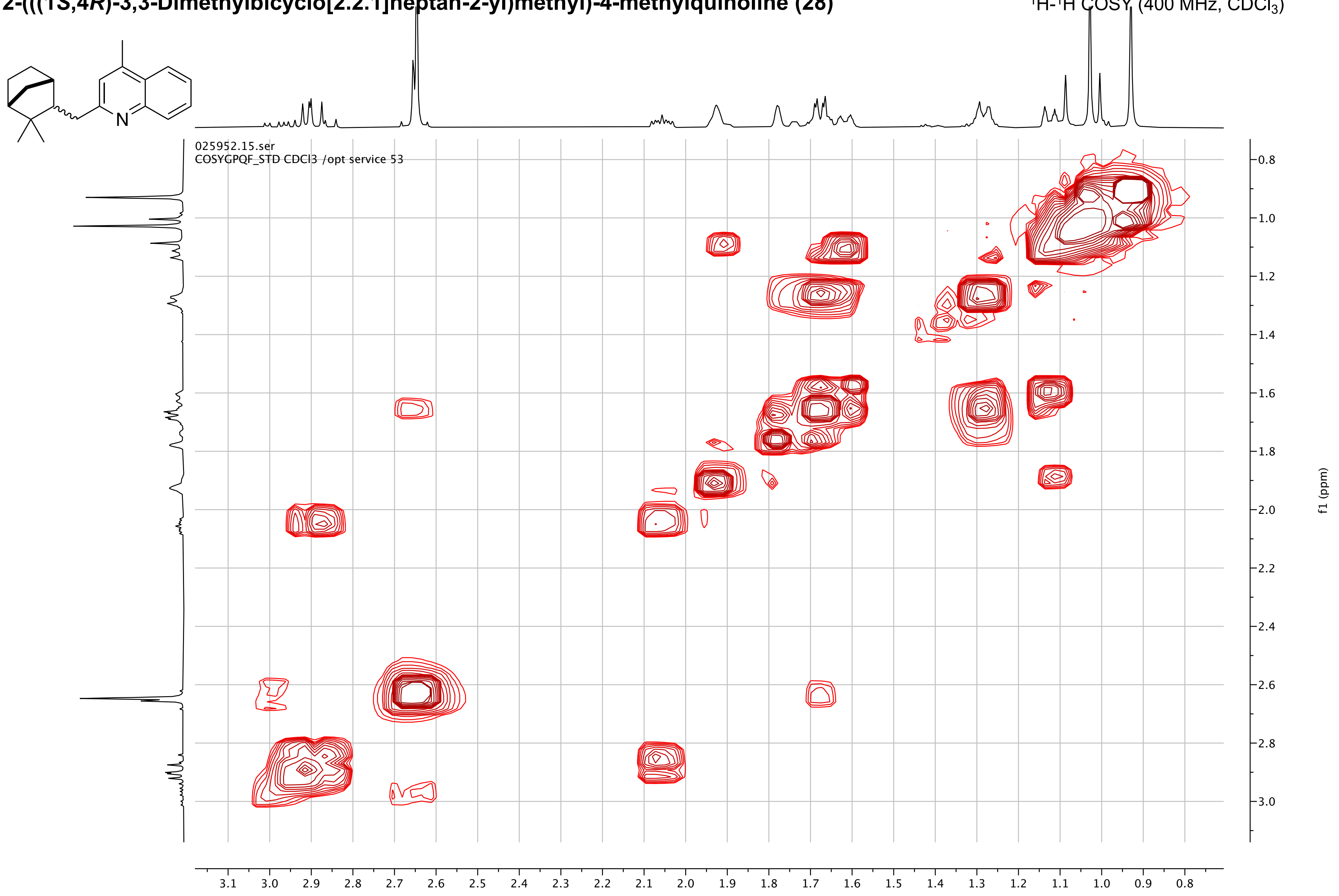

2-(((1*S*,4*R*)-3,3-Dimethylbicyclo[2.2.1]heptan-2-yl)methyl)-4-methylquinoline (28)

HSQC(400 MHz, $CDCl_3$)

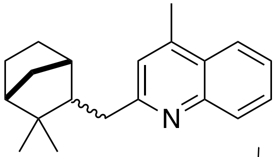

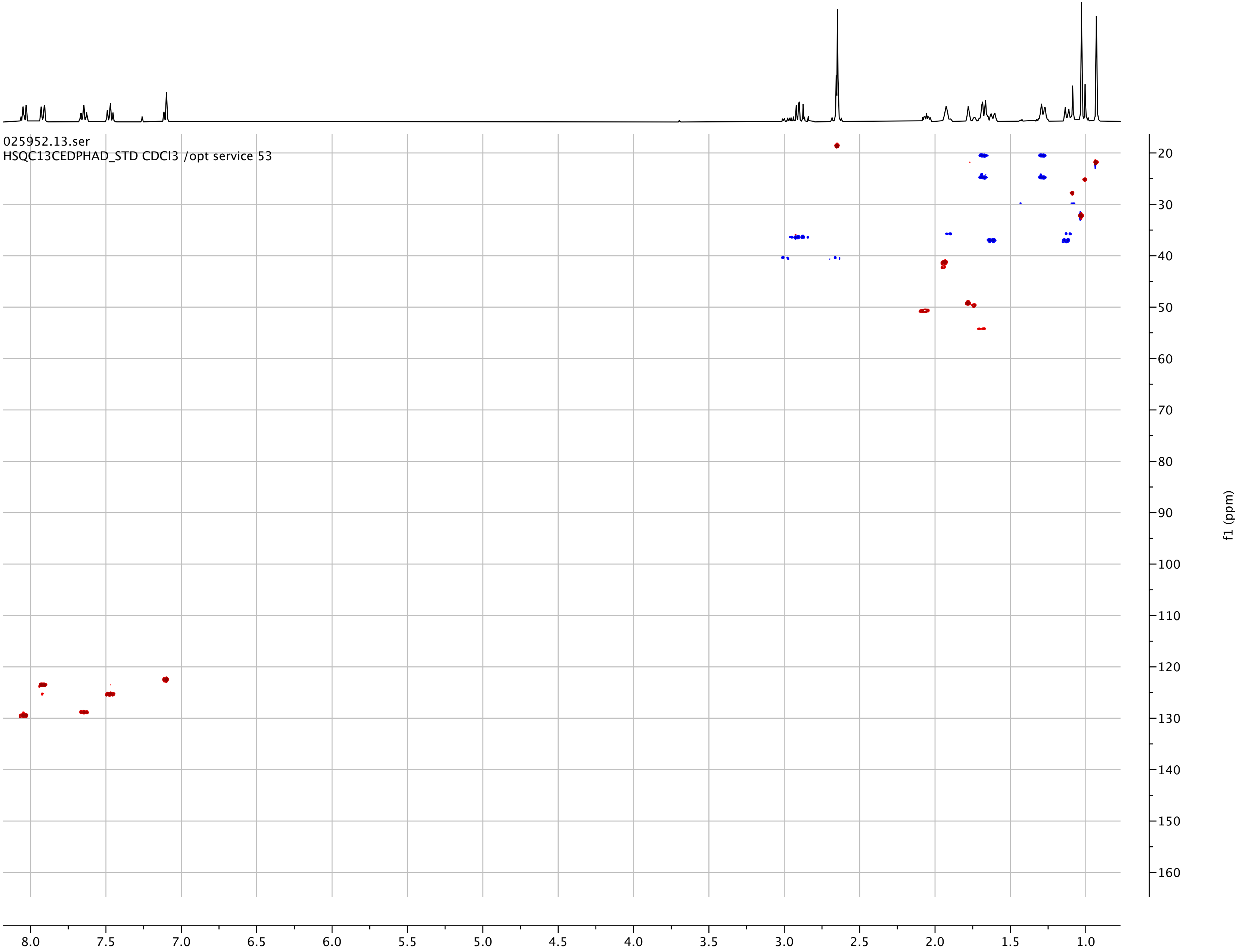

2-(((1*S*,4*R*)-3,3-dDmethylbicyclo[2.2.1]heptan-2-yl)methyl)-4-methylquinoline (28)

HSQC(400 MHz, $CDCl_3$)

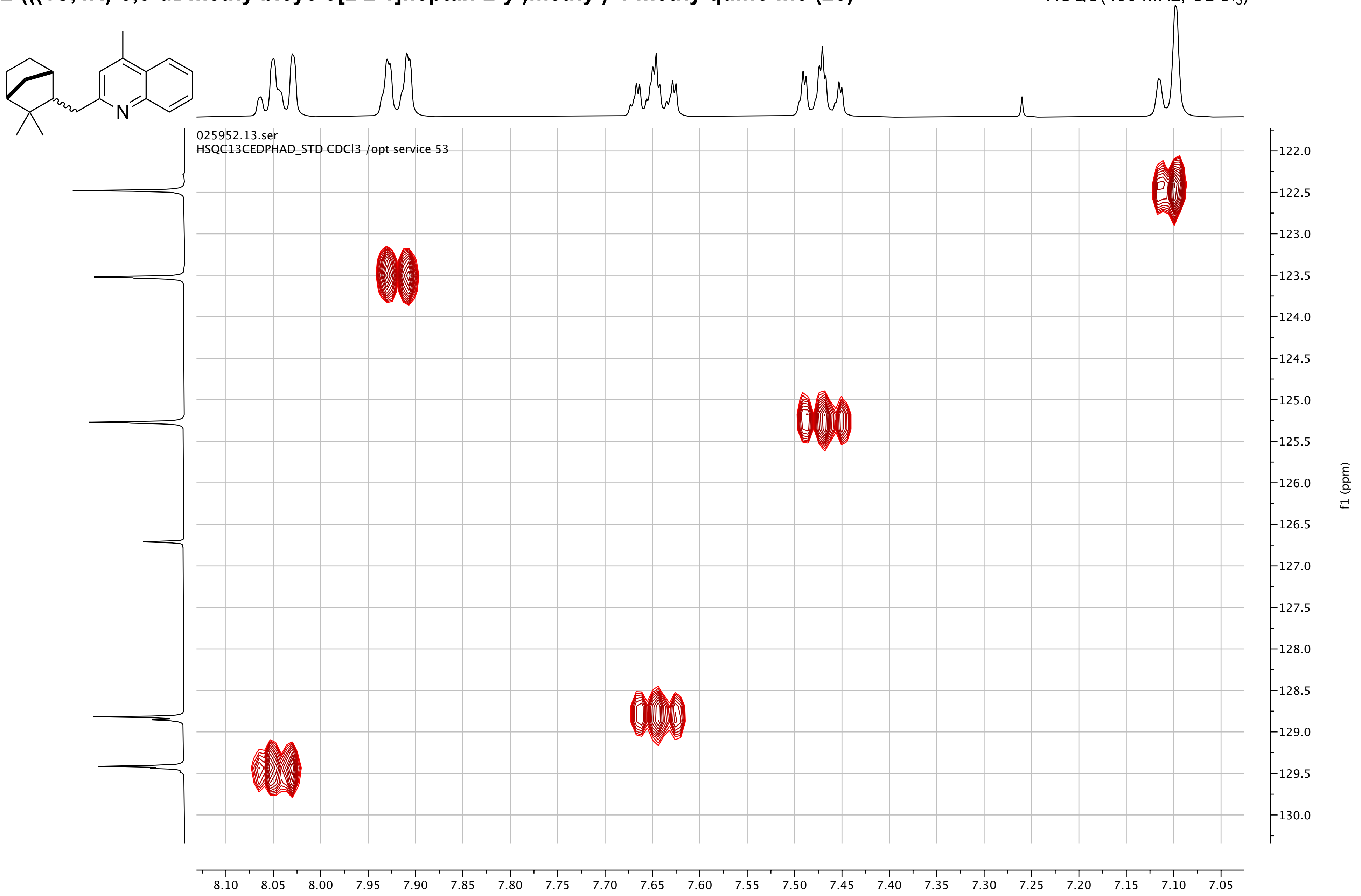

2-(((1*S*,4*R*)-3,3-Dimethylbicyclo[2.2.1]heptan-2-yl)methyl)-4-methylquinoline (28)

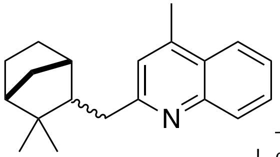

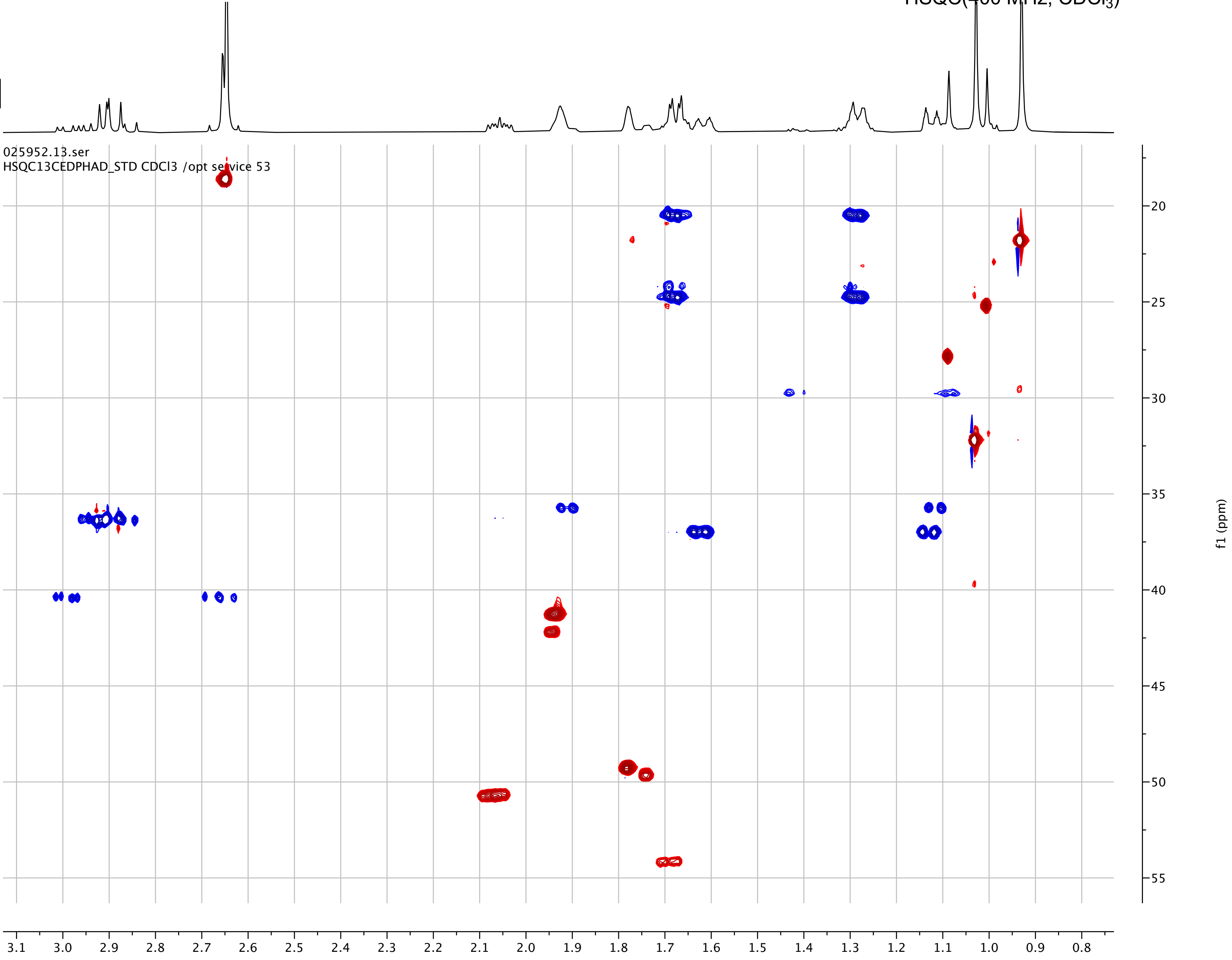

2-(((1*S*,4*R*)-3,3-Dimethylbicyclo[2.2.1]heptan-2-yl)methyl)-4-methylquinoline (28)

HSQC(400 MHz, $CDCl_3$)

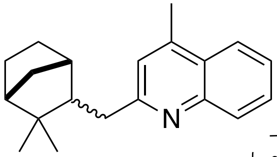

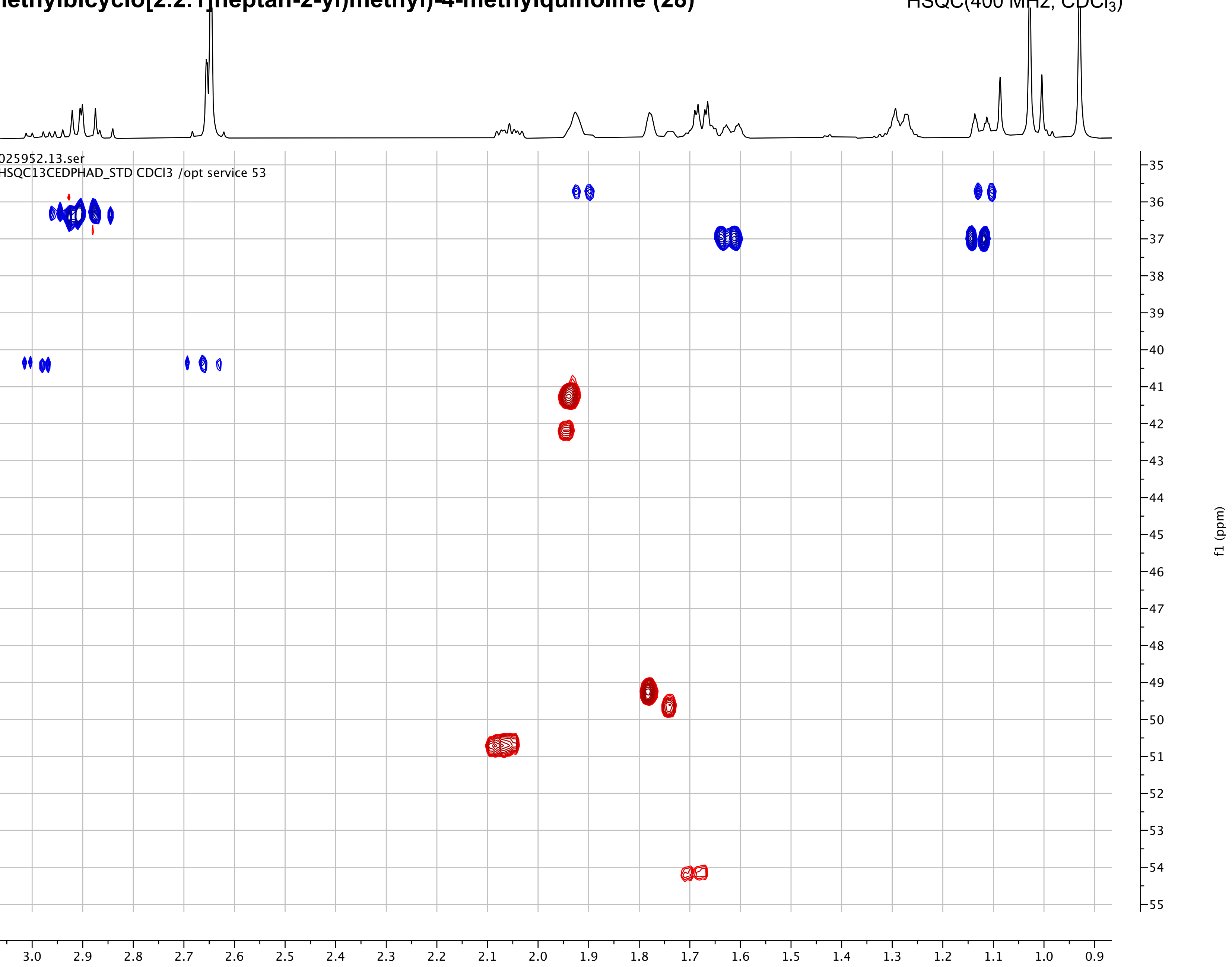

2-(((1*S*,4*R*)-3,3-Dimethylbicyclo[2.2.1]heptan-2-yl)methyl)-4-methylquinoline (28)

HMBC(400 MHz, $CDCl_3$)

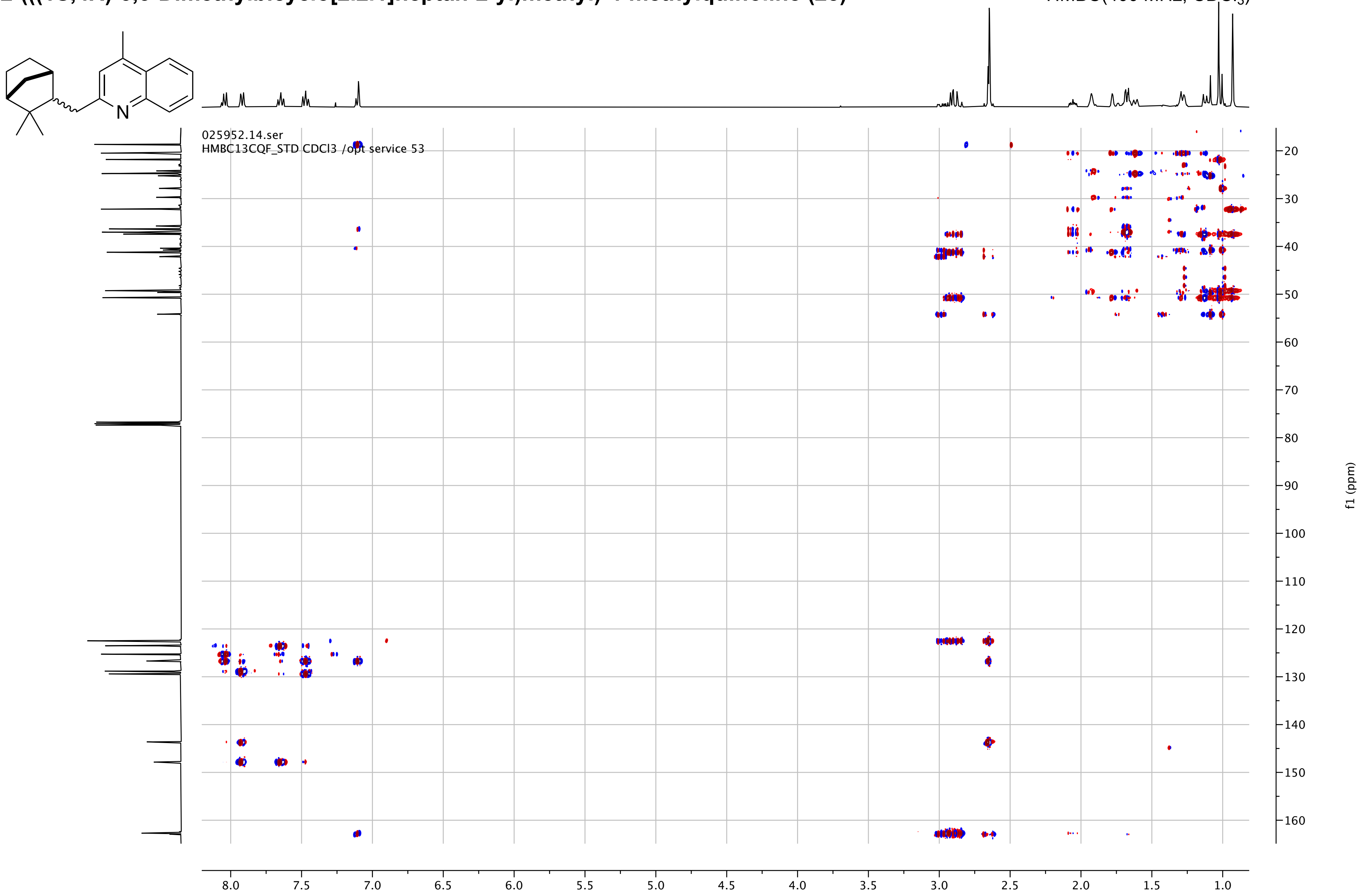

2-(((1*S*,4*R*)-3,3-Dimethylbicyclo[2.2.1]heptan-2-yl)methyl)-4-methylquinoline (28)

HMBC(400 MHz, $CDCl_3$)

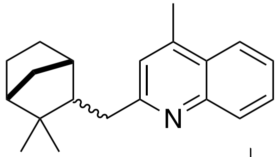

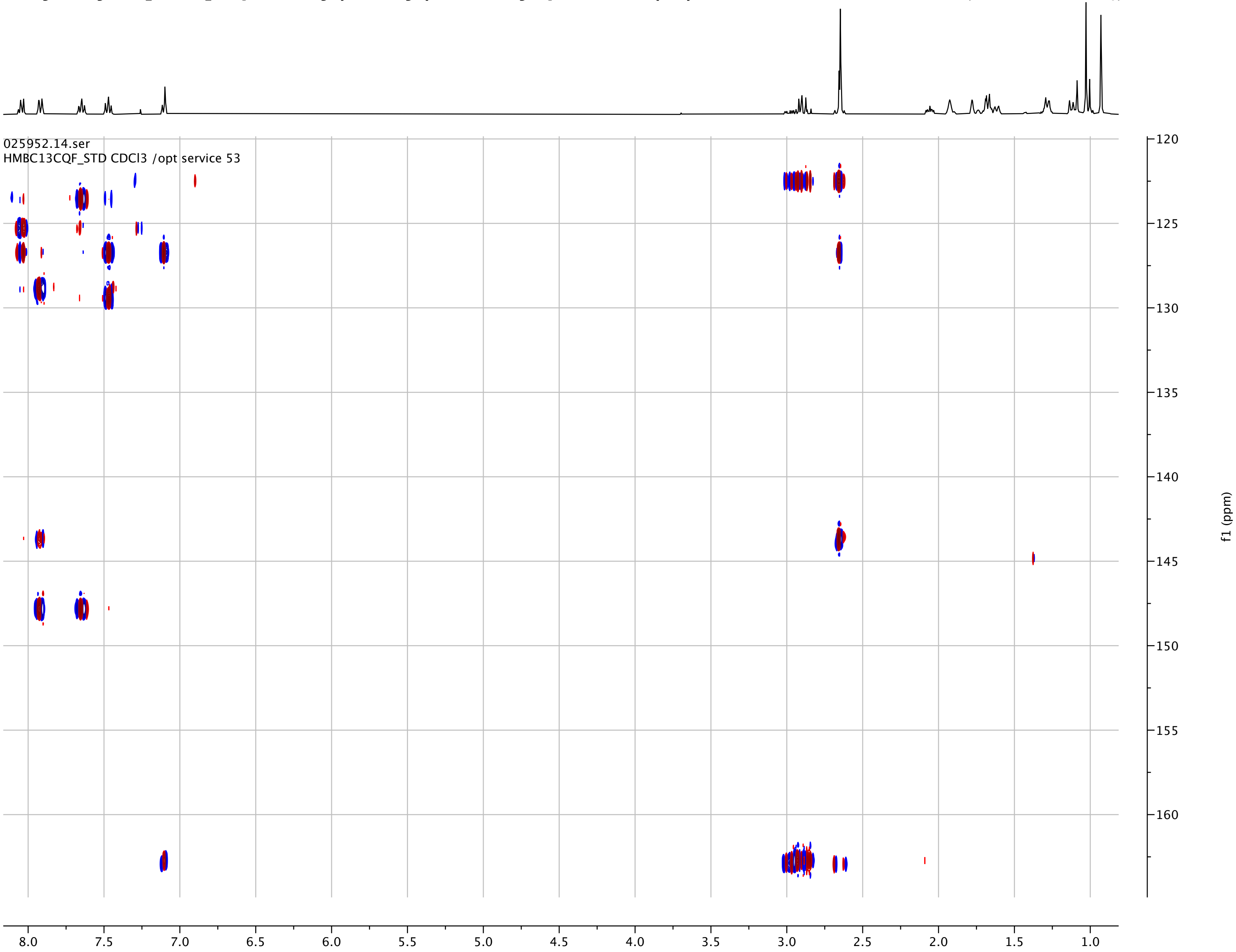

2-(((1*S*,4*R*)-3,3-Dimethylbicyclo[2.2.1]heptan-2-yl)methyl)-4-methylquinoline (28)

HMBC(400 MHz, $CDCl_3$)

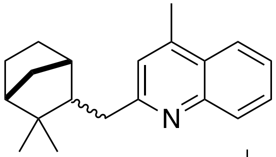

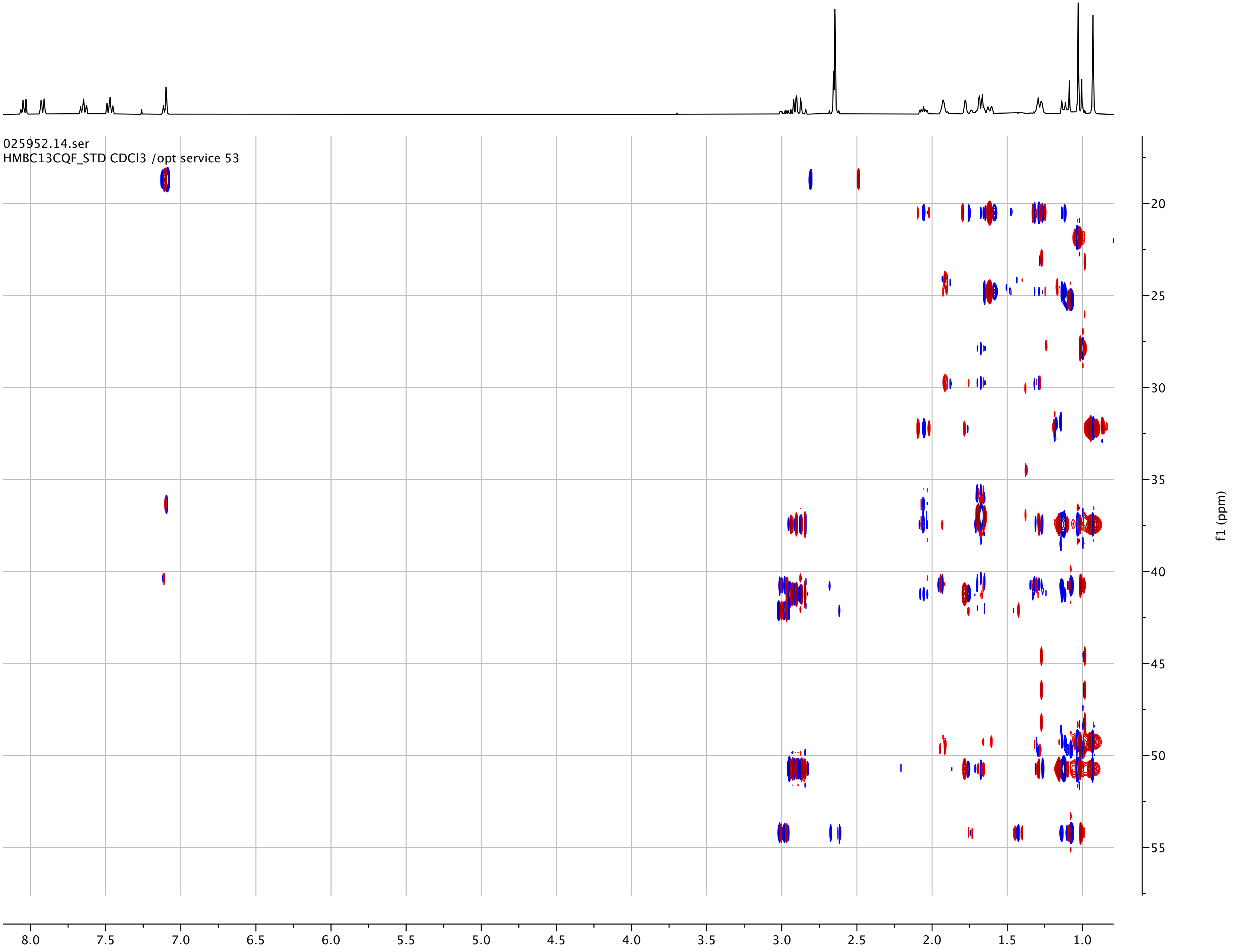

(3*S*,5*R*,6*S*,8*S*,9*S*,10*R*,13*R*,14*S*,17*R*)-10,13-Dimethyl-17-((*R*)-6-methylheptan-2-yl)-6-(4-methylquinolin-2-yl)hexadecahydro-1*H*-cyclopenta[*a*]phenanthren-3-yl benzoate (29)

^{1}H-NMR (400 MHz, $CDCl_3$)

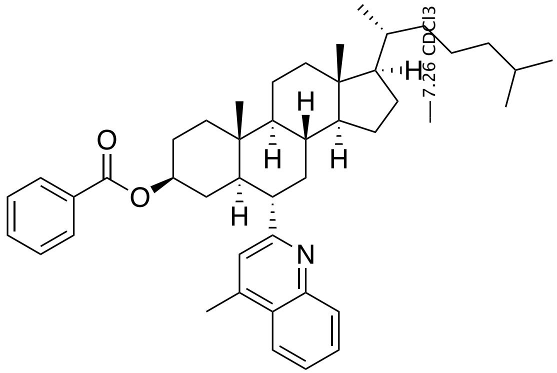

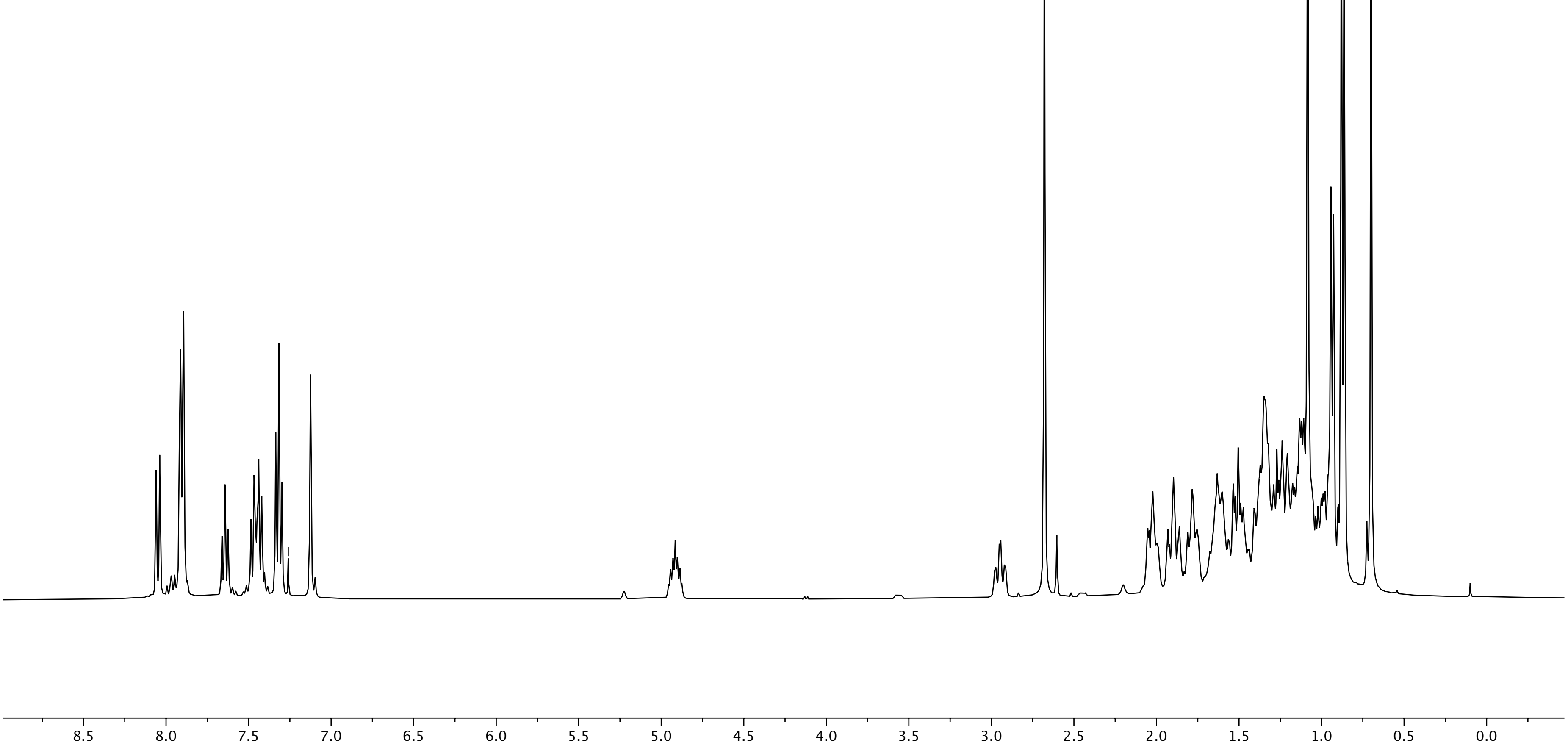

(3*S*,5*R*,6*S*,8*S*,9*S*,10*R*,13*R*,14*S*,17*R*)-10,13-Dimethyl-17-((*R*)-6-methylheptan-2-yl)-6-(4-methylquinolin-2-yl)hexadecahydro-1*H*-cyclopenta[*a*]phenanthren-3-yl benzoate (29)

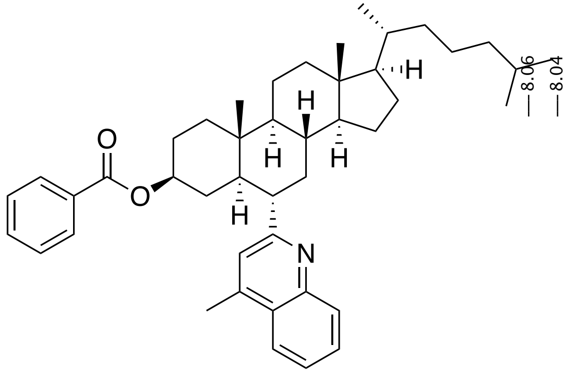

^{1}H-NMR (400 MHz, $CDCl_3$)

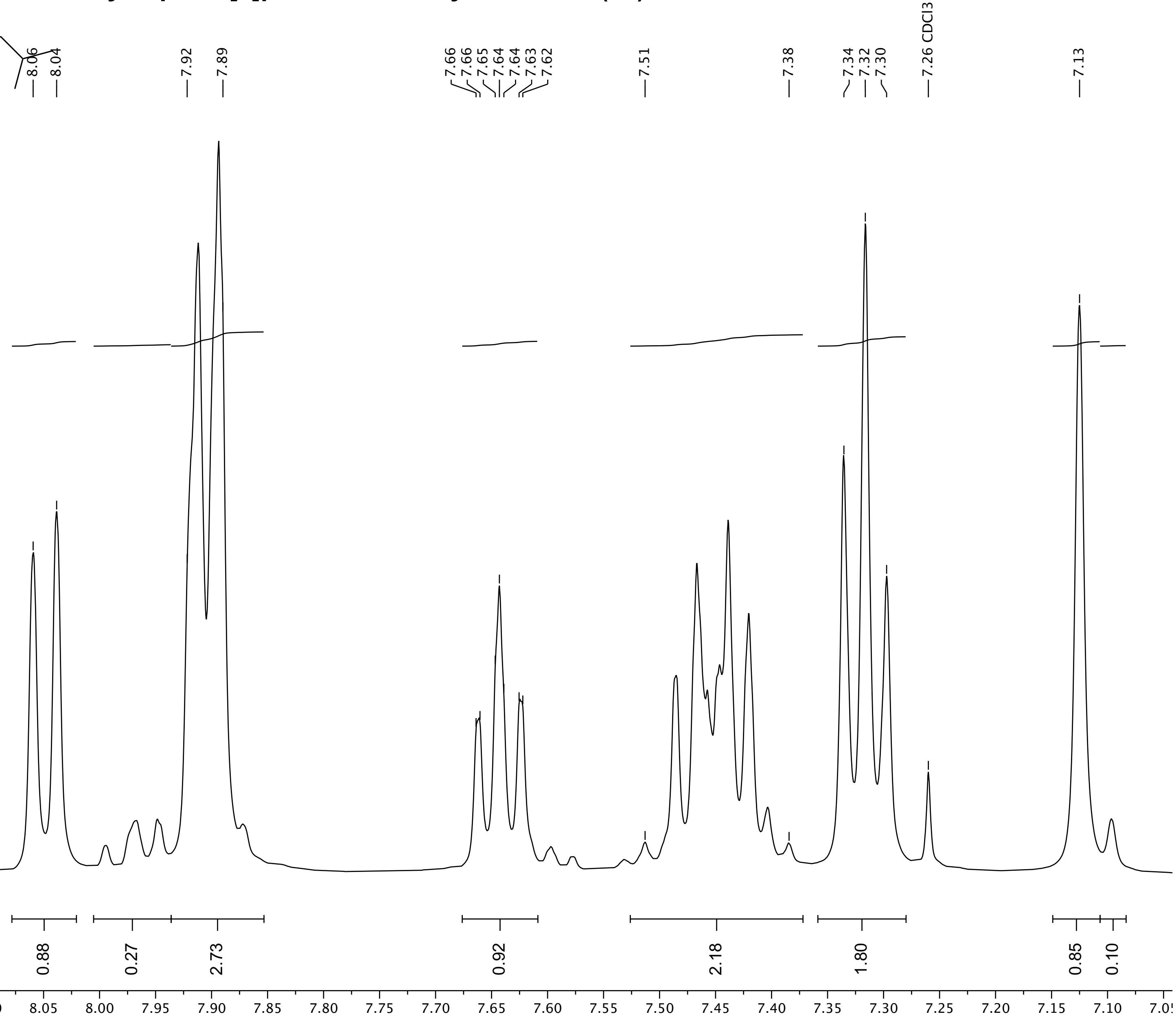

(3*S*,5*R*,6*S*,8*S*,9*S*,10*R*,13*R*,14*S*,17*R*)-10,13-Dimethyl-17-((*R*)-6-methylheptan-2-yl)-6-(4-methylquinolin-2-yl)hexadecahydro-1*H*-cyclopenta[*a*]phenanthren-3-yl benzoate (29)

^{1}H-NMR (400 MHz, $CDCl_3$)

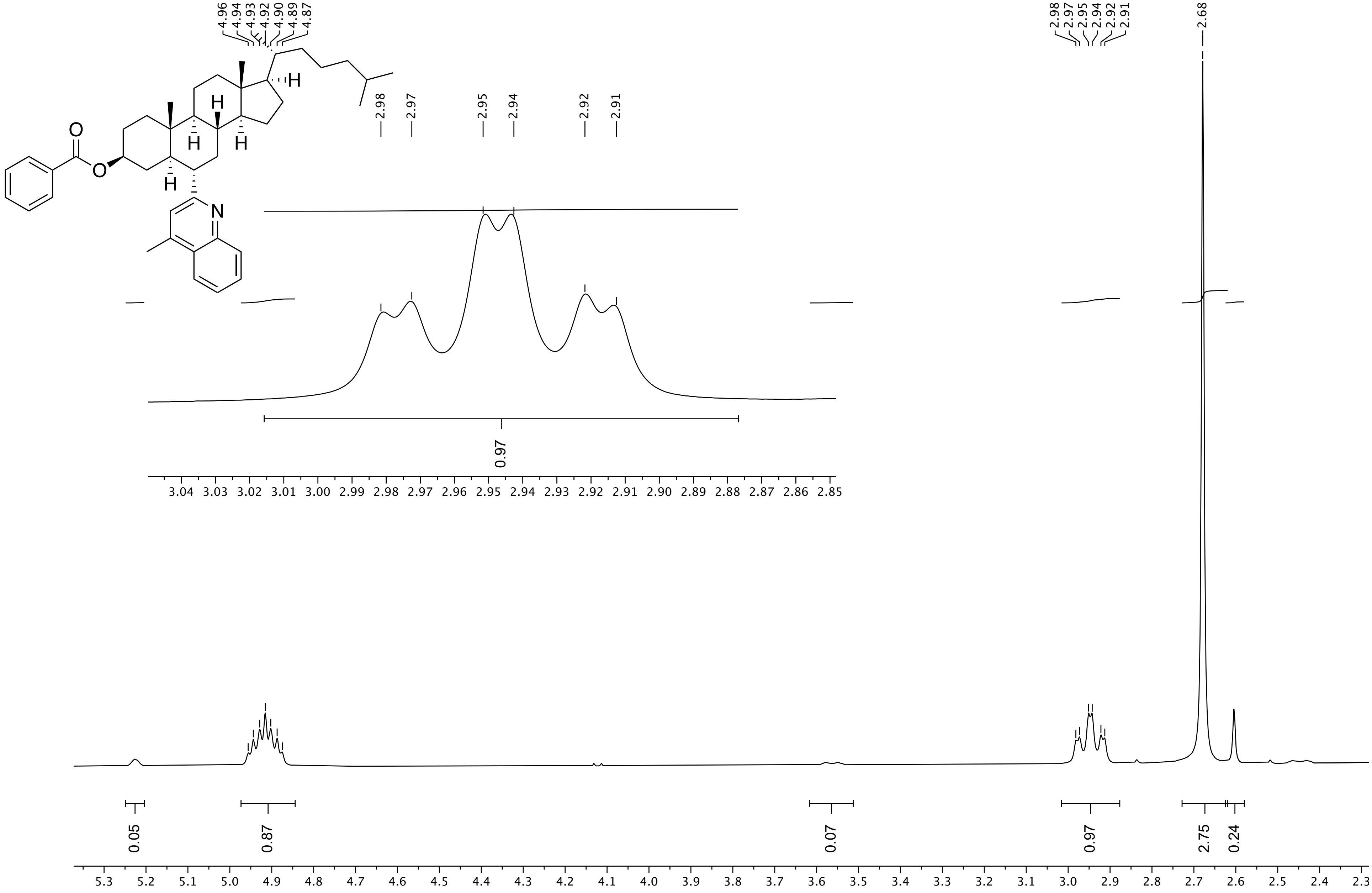

(3*S*,5*R*,6*S*,8*S*,9*S*,10*R*,13*R*,14*S*,17*R*)-10,13-Dimethyl-17-((*R*)-6-methylheptan-2-yl)-6-(4-methylquinolin-2-yl)hexadecahydro-1*H*-cyclopenta[*a*]phenanthren-3-yl benzoate (29)

^{1}H-NMR (400 MHz, $CDCl_3$)

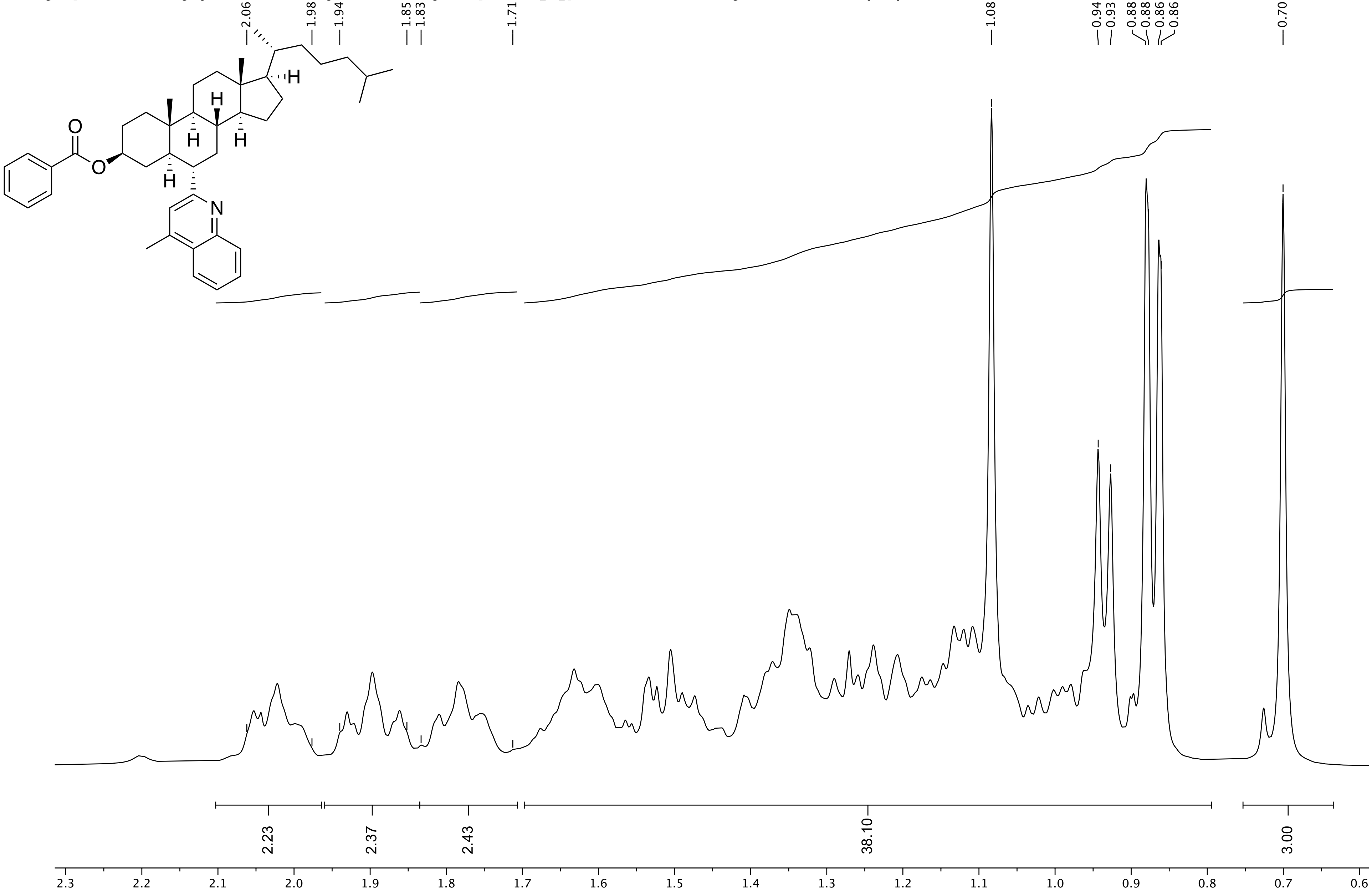

(3*S*,5*R*,6*S*,8*S*,9*S*,10*R*,13*R*,14*S*,17*R*)-10,13-Dimethyl-17-((*R*)-6-methylheptan-2-yl)-6-(4-methylquinolin-2-yl)hexadecahydro-1*H*-cyclopenta[*a*]phenanthren-3-yl benzoate (29)

^{13}C-NMR (101 MHz, $CDCl_3$)

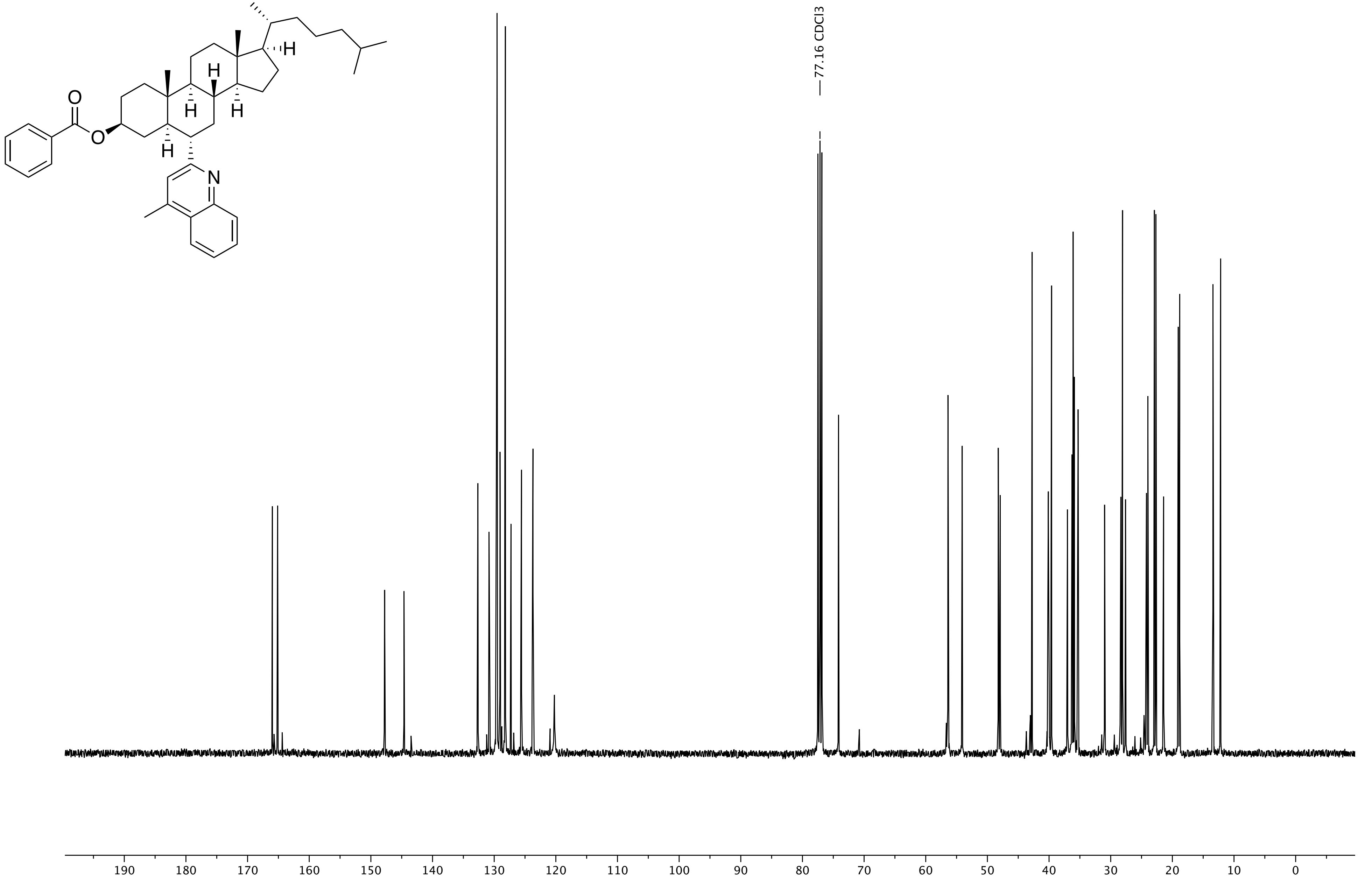

(3*S*,5*R*,6*S*,8*S*,9*S*,10*R*,13*R*,14*S*,17*R*)-10,13-Dimethyl-17-((*R*)-6-methylheptan-2-yl)-6-(4-methylquinolin-2-yl)hexadecahydro-1*H*-cyclopenta[*a*]phenanthren-3-yl benzoate (29)

^{13}C-NMR (101 MHz, $CDCl_3$)

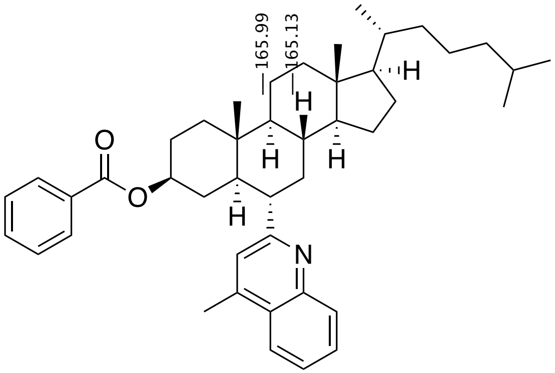

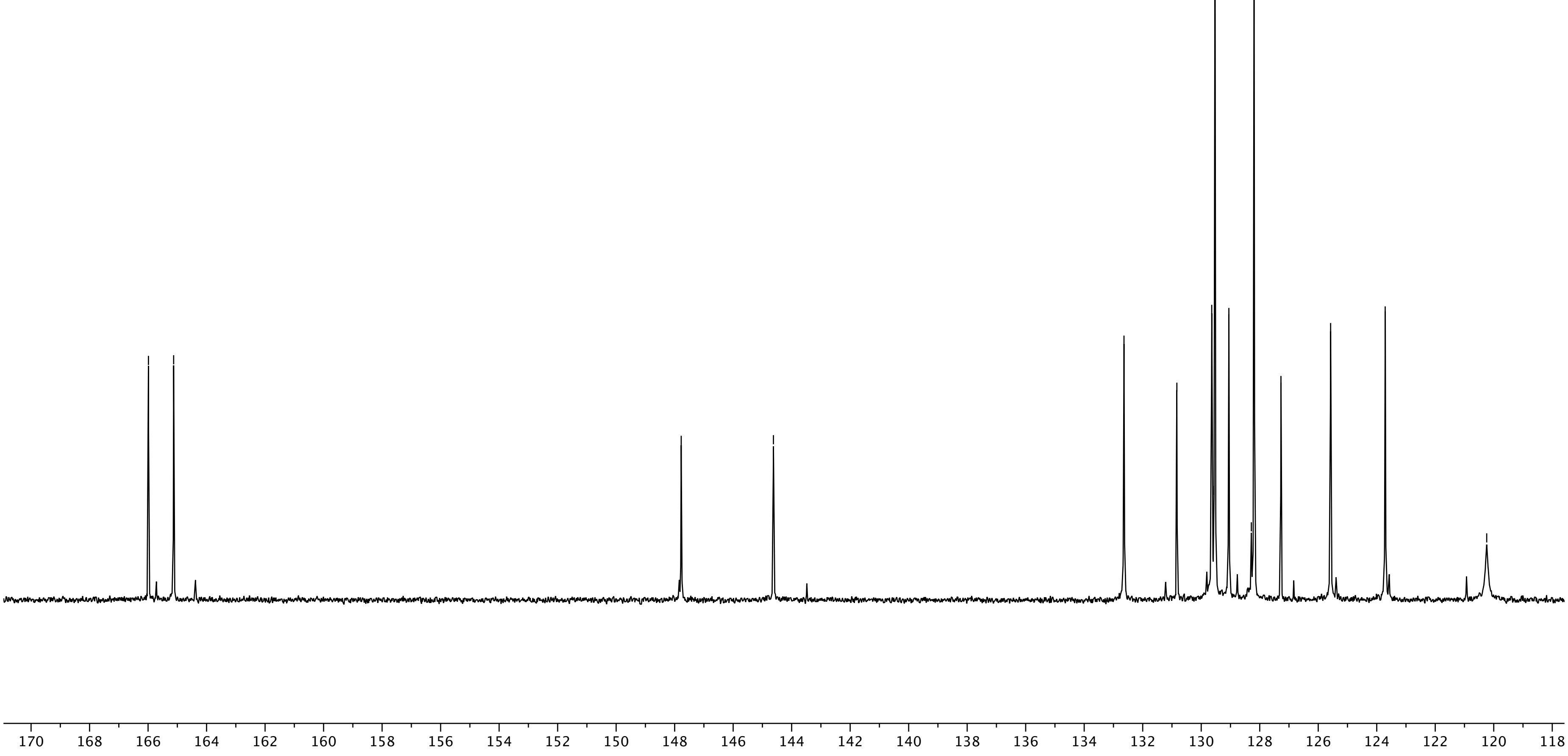

(3*S*,5*R*,6*S*,8*S*,9*S*,10*R*,13*R*,14*S*,17*R*)-10,13-Dimethyl-17-((*R*)-6-methylheptan-2-yl)-6-(4-methylquinolin-2-yl)hexadecahydro-1*H*-cyclopenta[*a*]phenanthren-3-yl benzoate (29)

^{13}C-NMR (101 MHz, $CDCl_3$)

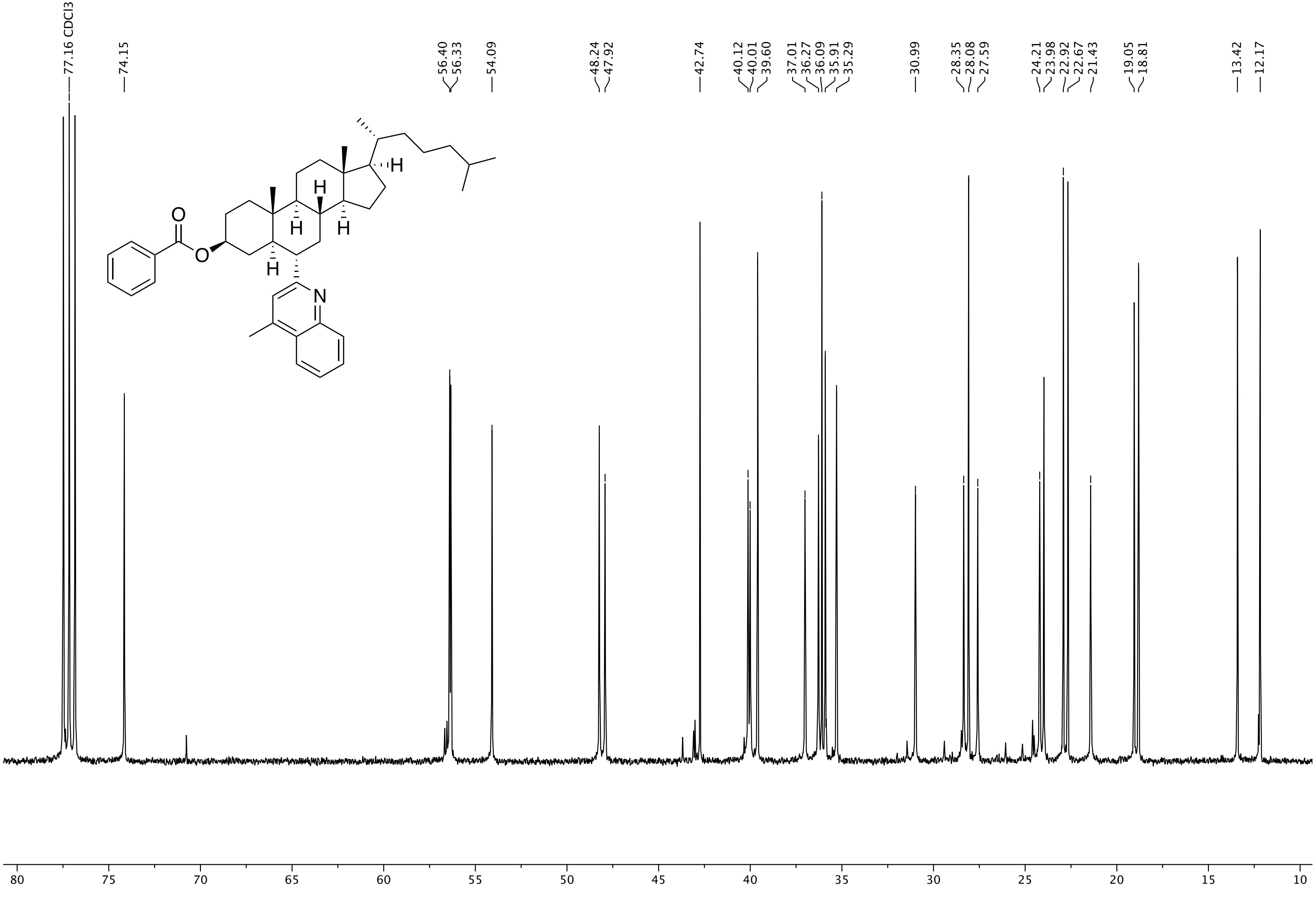

(3*S*,5*R*,6*S*,8*S*,9*S*,10*R*,13*R*,14*S*,17*R*)-10,13-Dimethyl-17-((*R*)-6-methylheptan-2-yl)-6-(4-methylquinolin-2-yl)hexadecahydro-1*H*-cyclopenta[*a*]phenanthren-3-yl benzoate (29)

Dept-135 (101 MHz, $CDCl_3$)

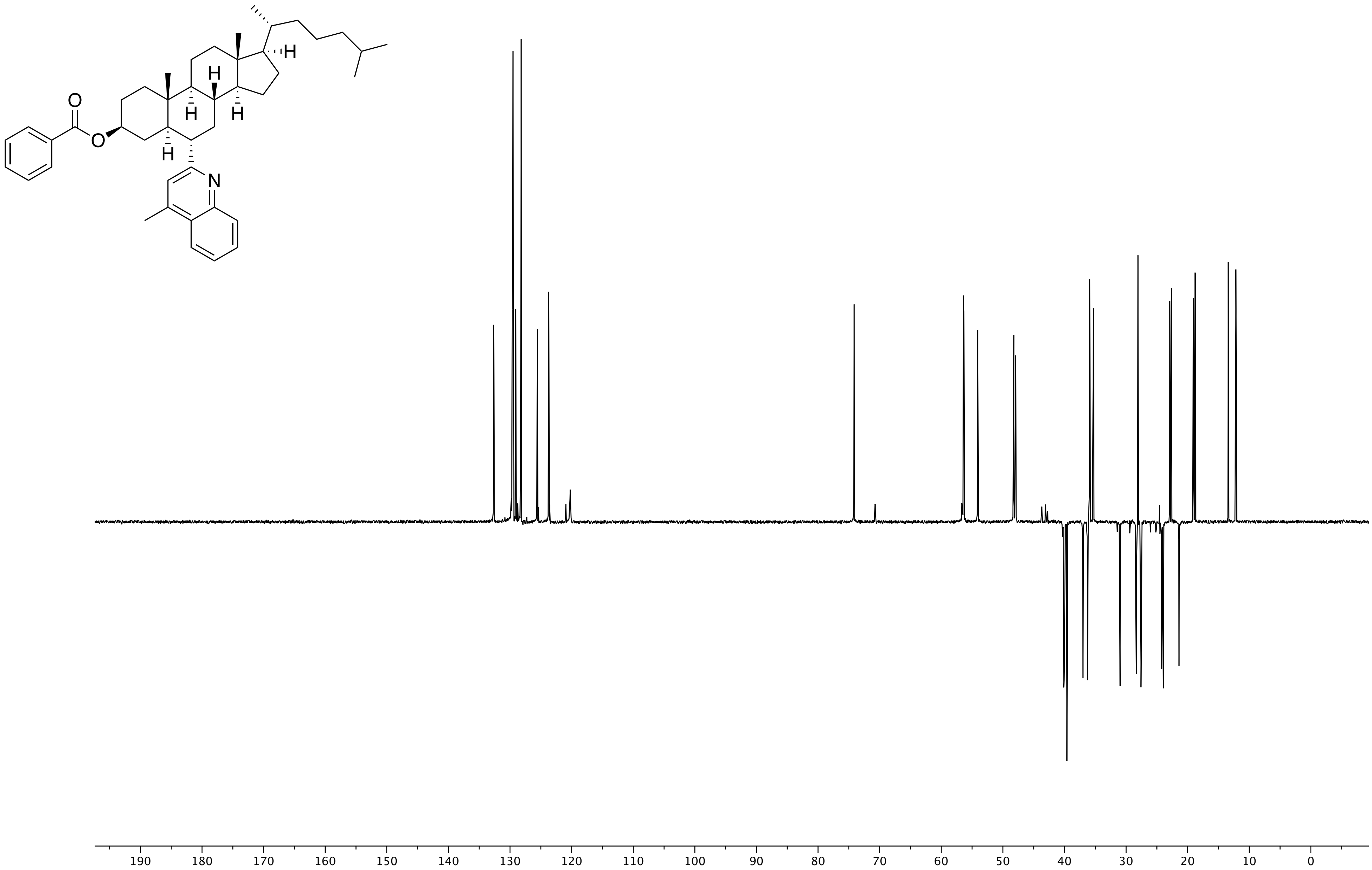

(3*S*,5*R*,6*S*,8*S*,9*S*,10*R*,13*R*,14*S*,17*R*)-10,13-Dimethyl-17-((*R*)-6-methylheptan-2-yl)-6-(4-methylquinolin-2-yl)hexadecahydro-1*H*-cyclopenta[*a*]phenanthren-3-yl benzoate (29)

Dept-135 (101 MHz, $CDCl_3$)

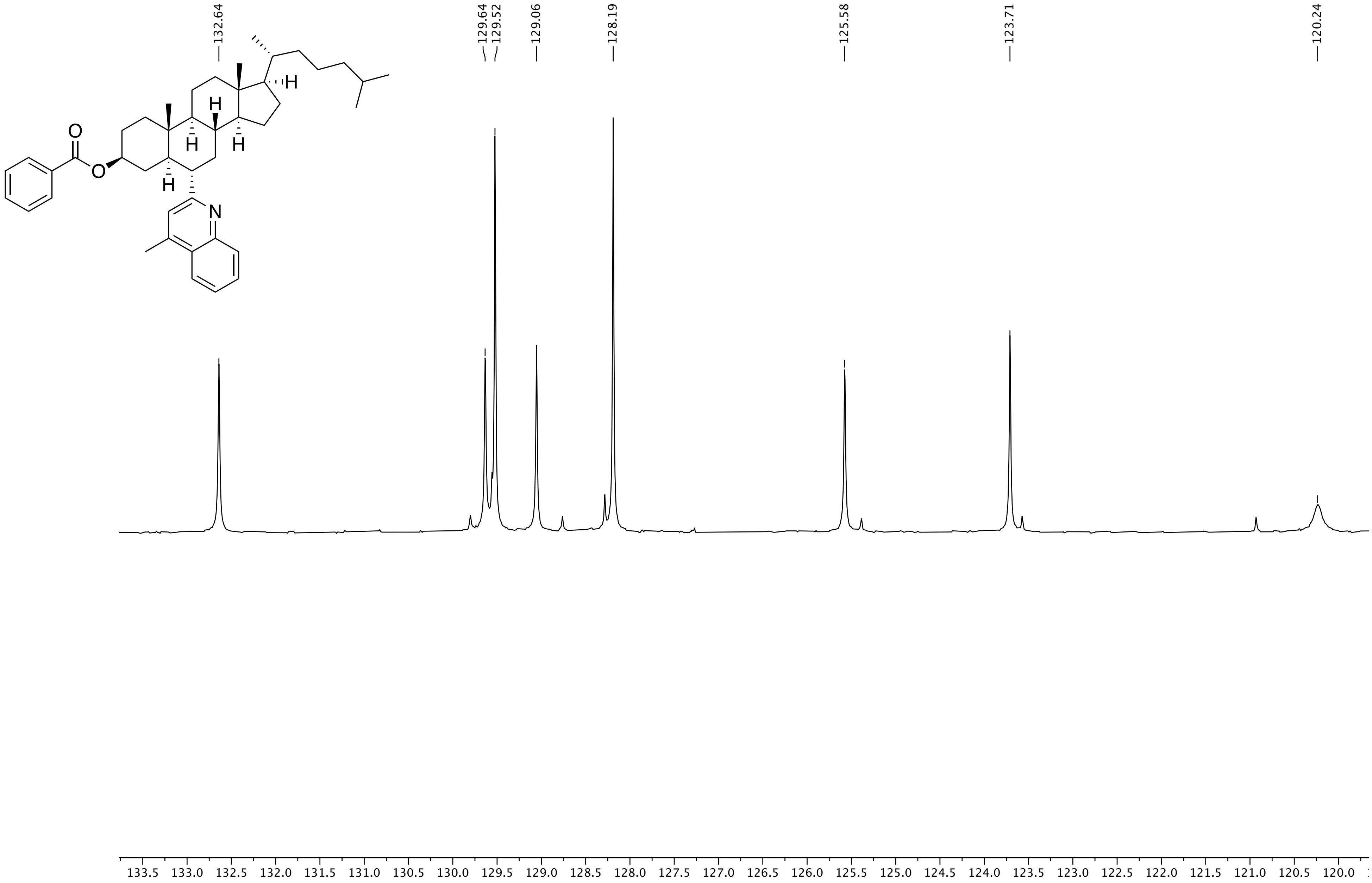

(3*S*,5*R*,6*S*,8*S*,9*S*,10*R*,13*R*,14*S*,17*R*)-10,13-Dimethyl-17-((*R*)-6-methylheptan-2-yl)-6-(4-methylquinolin-2-yl)hexadecahydro-1*H*-cyclopenta[*a*]phenanthren-3-yl benzoate (29)

Dept-135 (101 MHz, $CDCl_3$)

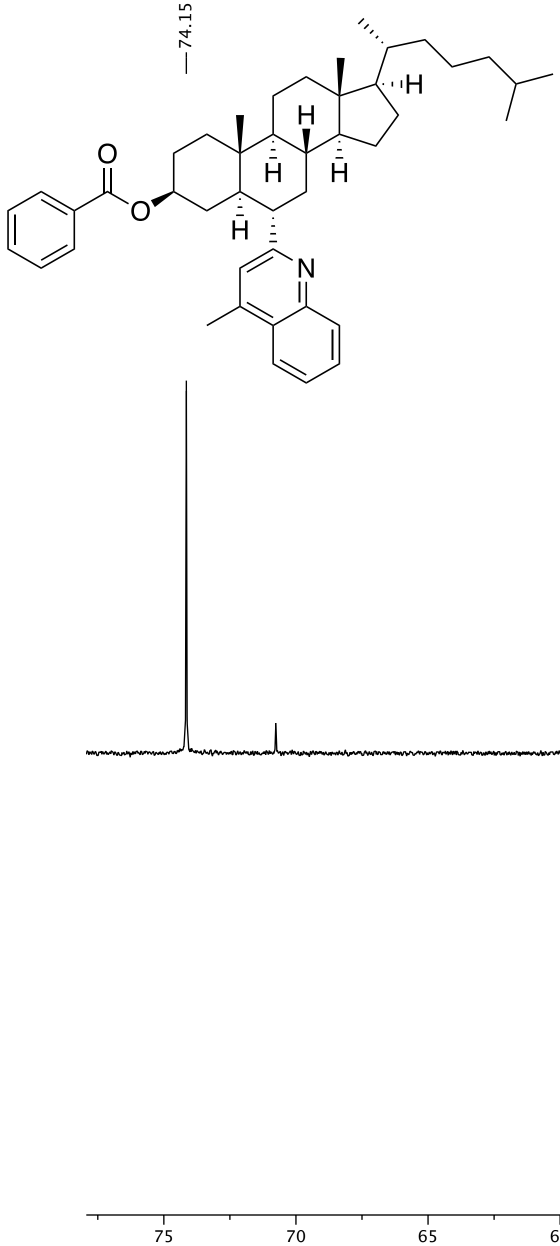

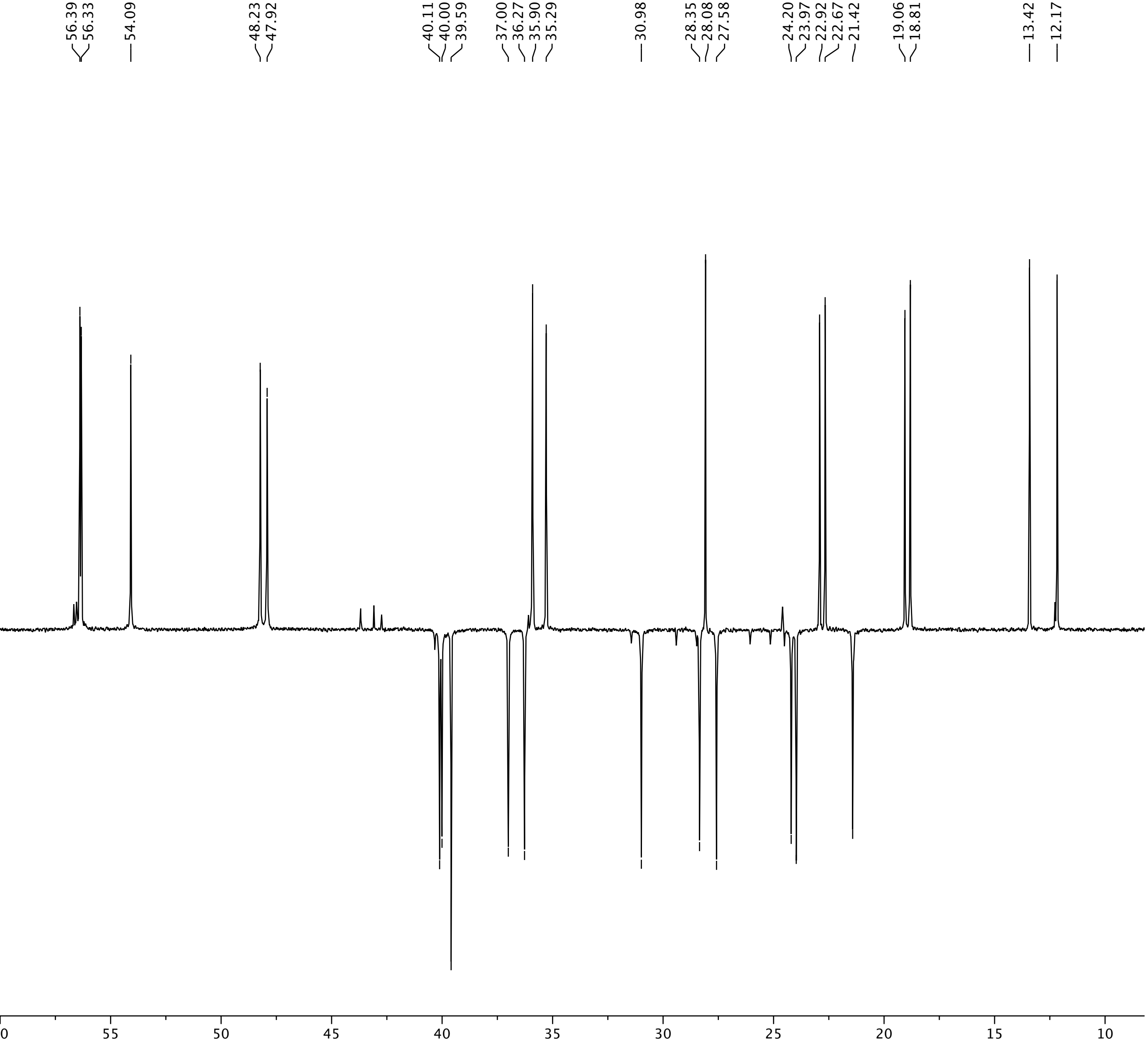

(3*S*,5*R*,6*S*,8*S*,9*S*,10*R*,13*R*,14*S*,17*R*)-10,13-Dimethyl-17-((*R*)-6-methylheptan-2-yl)-6-(4-methylquinolin-2-yl)hexadecahydro-1*H*-cyclopenta[*a*]phenanthren-3-yl benzoate (29)

^{1}H-^{1}H COSY 400 MHz, $CDCl_3$)

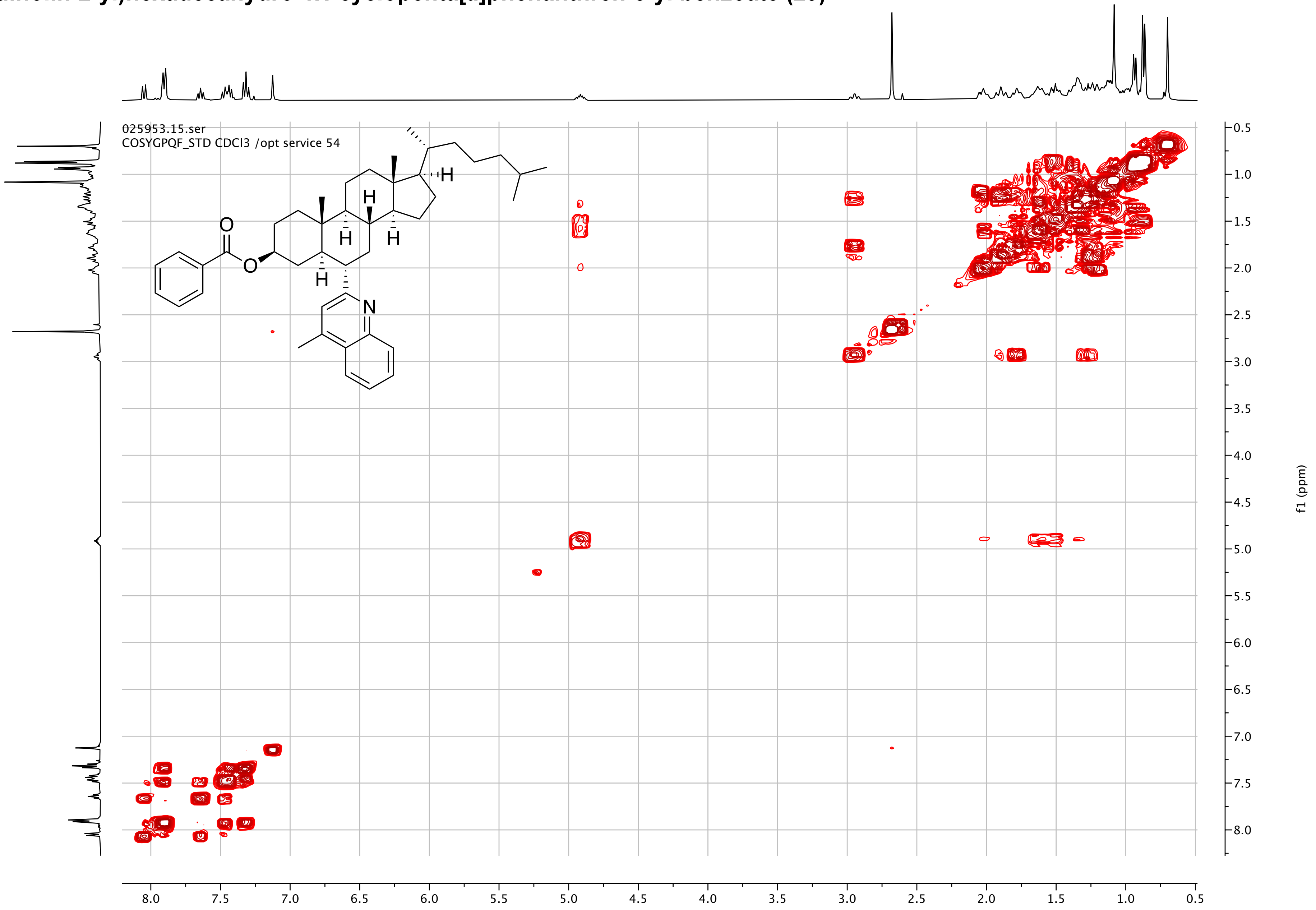

(3*S*,5*R*,6*S*,8*S*,9*S*,10*R*,13*R*,14*S*,17*R*)-10,13-Dimethyl-17-((*R*)-6-methylheptan-2-yl)-6-(4-methylquinolin-2-yl)hexadecahydro-1*H*-cyclopenta[*a*]phenanthren-3-yl benzoate (29)

^{1}H-^{1}H COSY 400 MHz, CDCl$_3$)

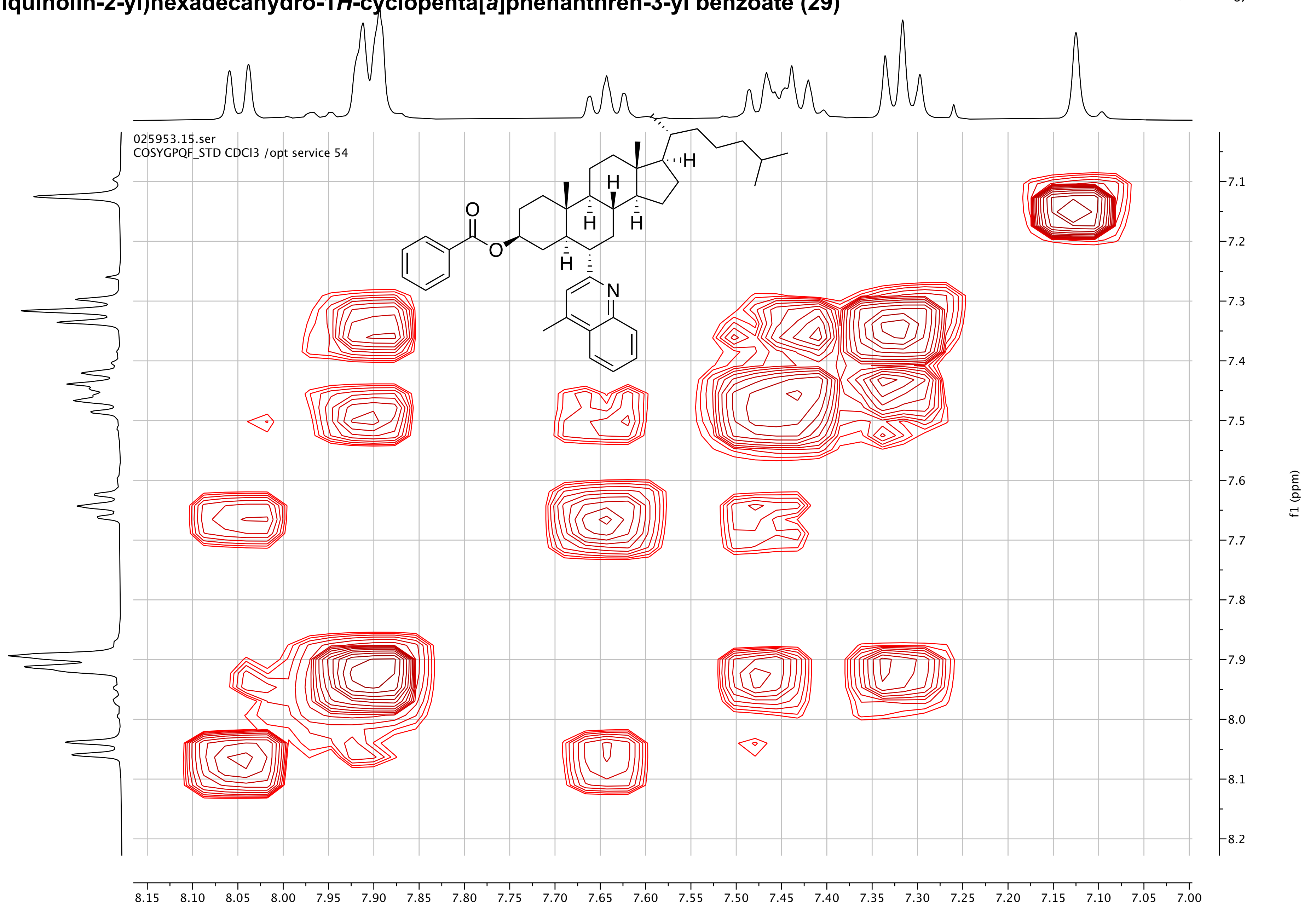

(3*S*,5*R*,6*S*,8*S*,9*S*,10*R*,13*R*,14*S*,17*R*)-10,13-Dimethyl-17-((*R*)-6-methylheptan-2-yl)-6-(4-methylquinolin-2-yl)hexadecahydro-1*H*-cyclopenta[*a*]phenanthren-3-yl benzoate (29)

^{1}H-^{1}H COSY 400 MHz, CDCl$_3$)

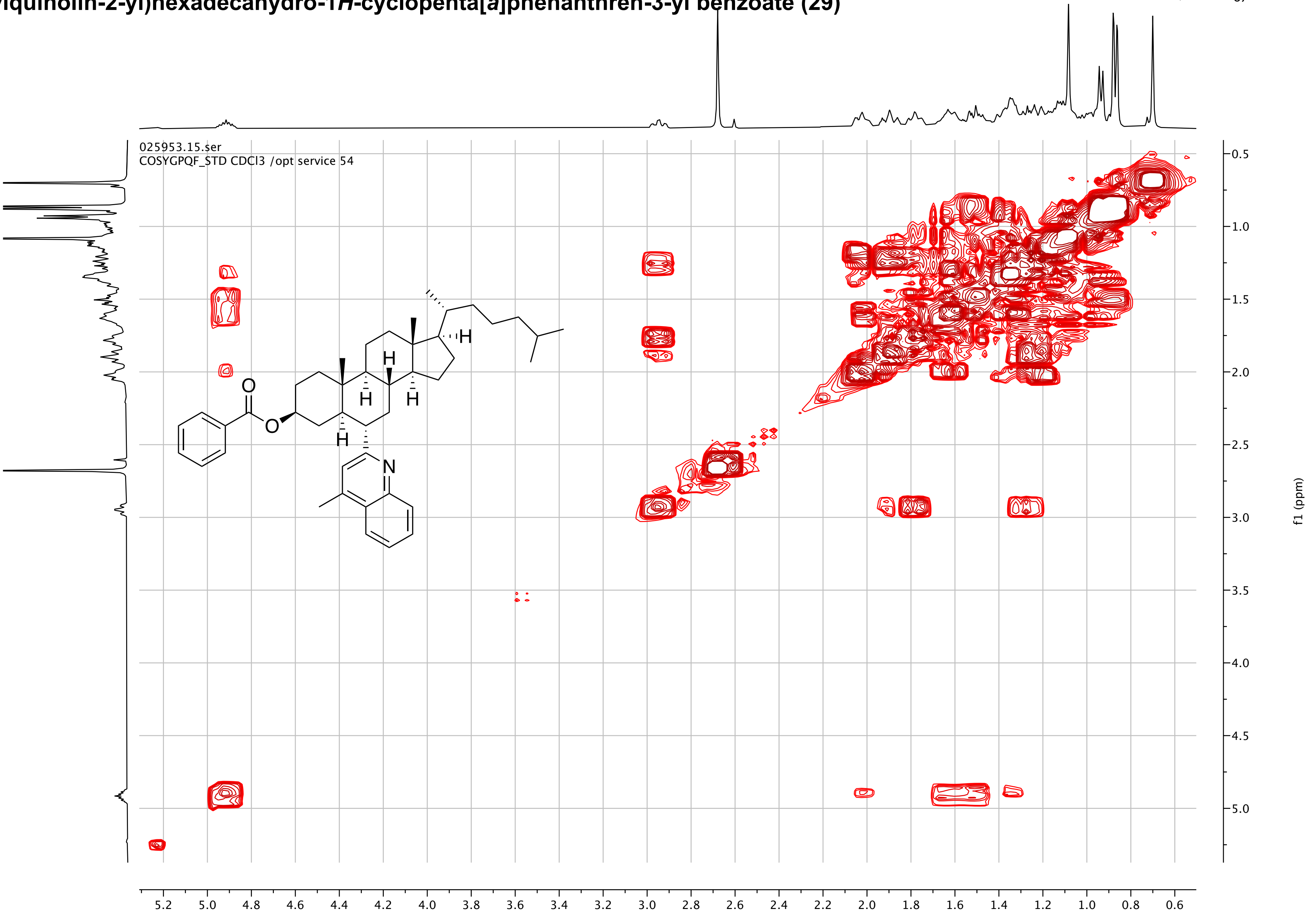

(3*S*,5*R*,6*S*,8*S*,9*S*,10*R*,13*R*,14*S*,17*R*)-10,13-Dimethyl-17-((*R*)-6-methylheptan-2-yl)-6-(4-methylquinolin-2-yl)hexadecahydro-1*H*-cyclopenta[*a*]phenanthren-3-yl benzoate (29)

HSQC (400 MHz, $CDCl_3$)

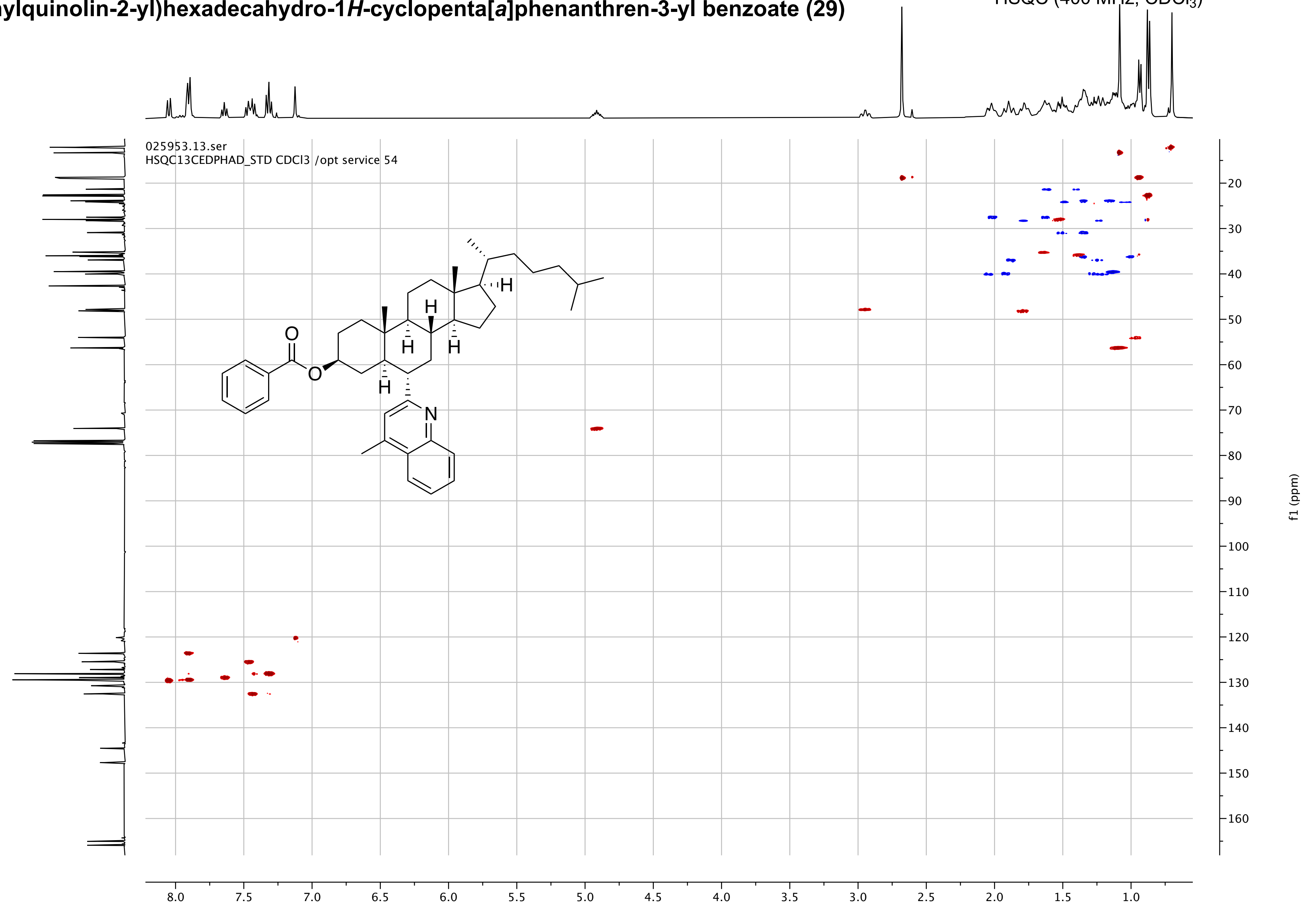

(3*S*,5*R*,6*S*,8*S*,9*S*,10*R*,13*R*,14*S*,17*R*)-10,13-Dimethyl-17-((*R*)-6-methylheptan-2-yl)-6-(4-methylquinolin-2-yl)hexadecahydro-1*H*-cyclopenta[*a*]phenanthren-3-yl benzoate (29)

HSQC (400 MHz, $CDCl_3$)

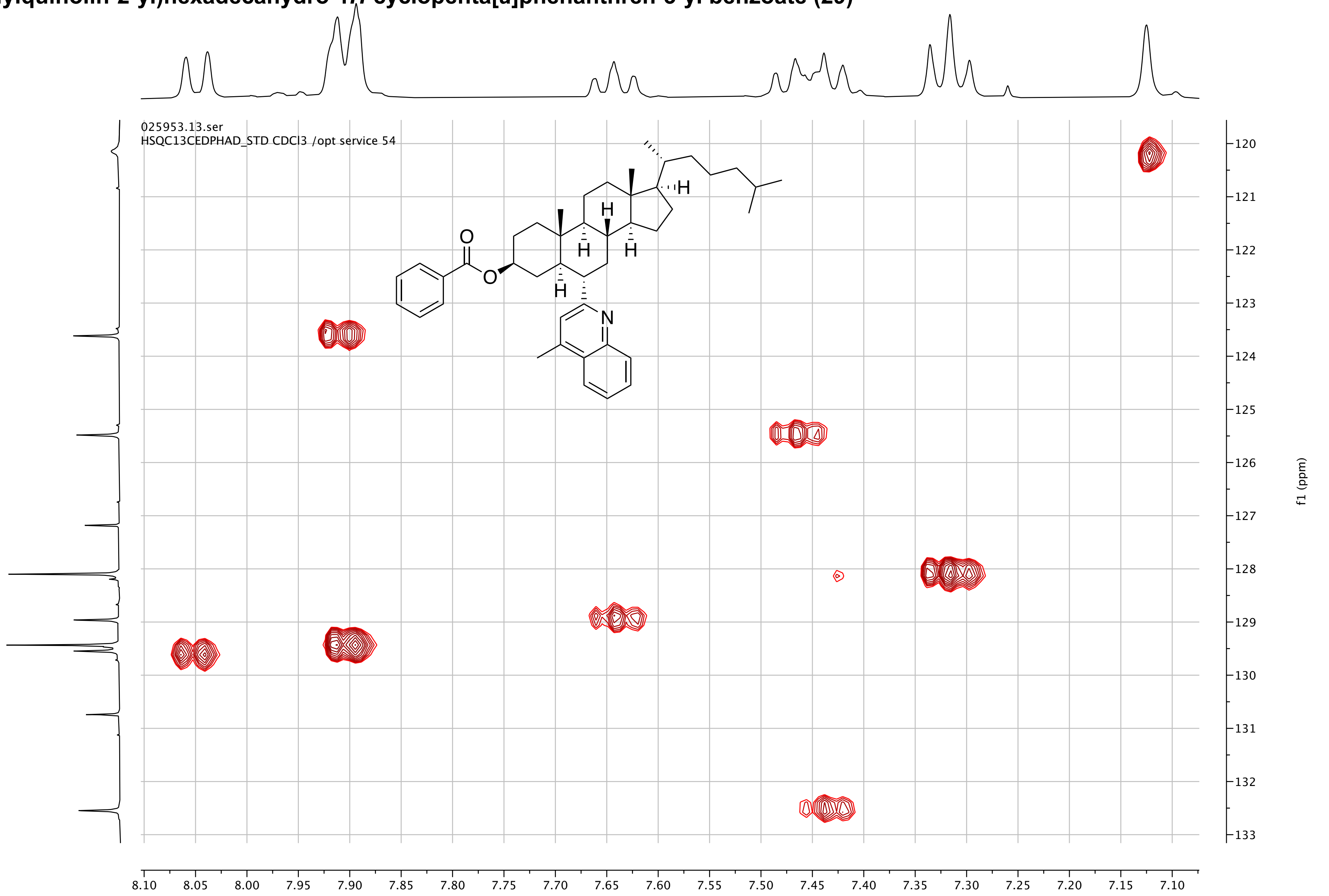

(3*S*,5*R*,6*S*,8*S*,9*S*,10*R*,13*R*,14*S*,17*R*)-10,13-Dimethyl-17-((*R*)-6-methylheptan-2-yl)-6-(4-methylquinolin-2-yl)hexadecahydro-1*H*-cyclopenta[*a*]phenanthren-3-yl benzoate (29)

HSQC (400 MHz, $CDCl_3$)

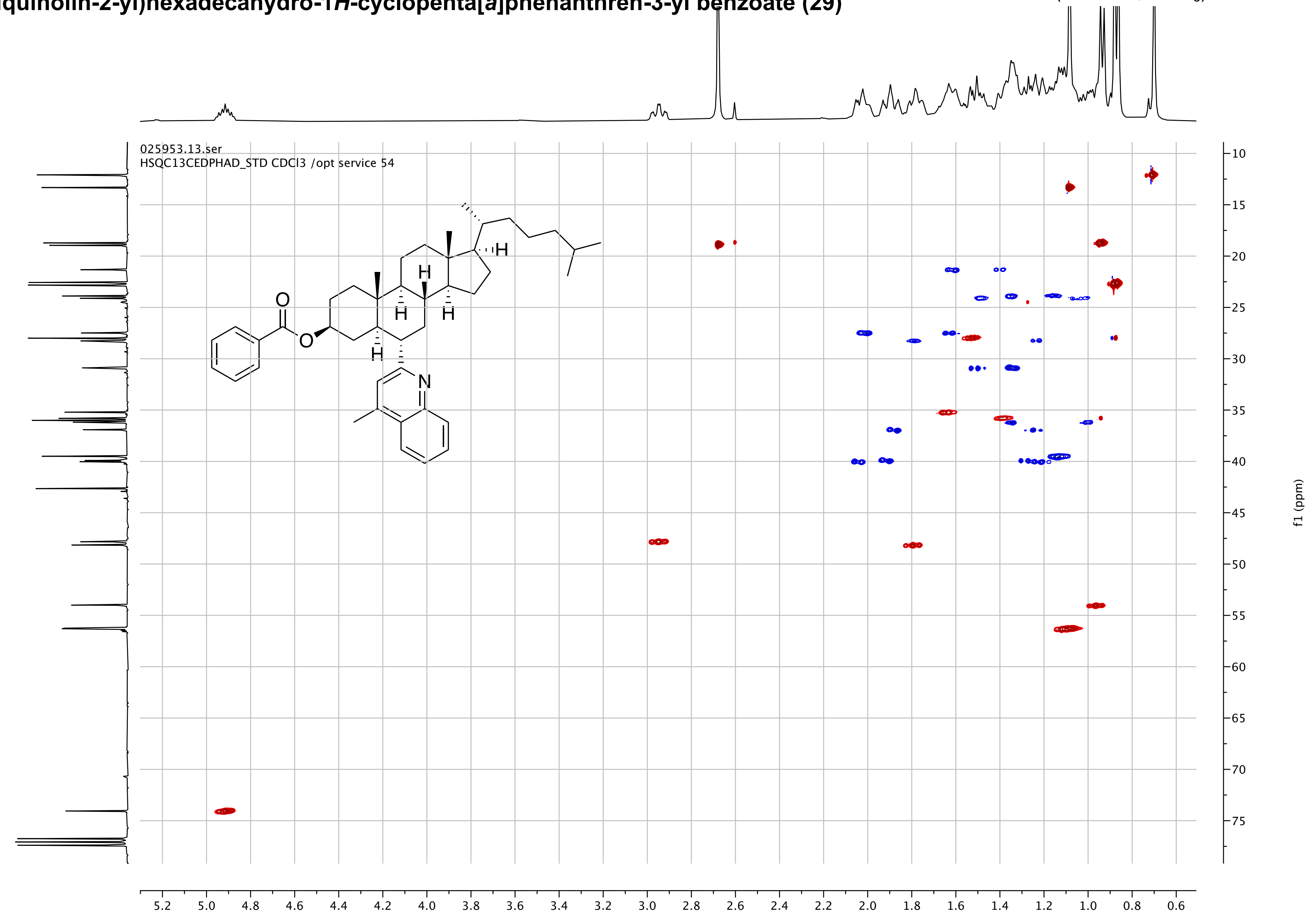

(3*S*,5*R*,6*S*,8*S*,9*S*,10*R*,13*R*,14*S*,17*R*)-10,13-Dimethyl-17-((*R*)-6-methylheptan-2-yl)-6-(4-methylquinolin-2-yl)hexadecahydro-1*H*-cyclopenta[*a*]phenanthren-3-yl benzoate (29)

HSQC (400 MHz, $CDCl_3$)

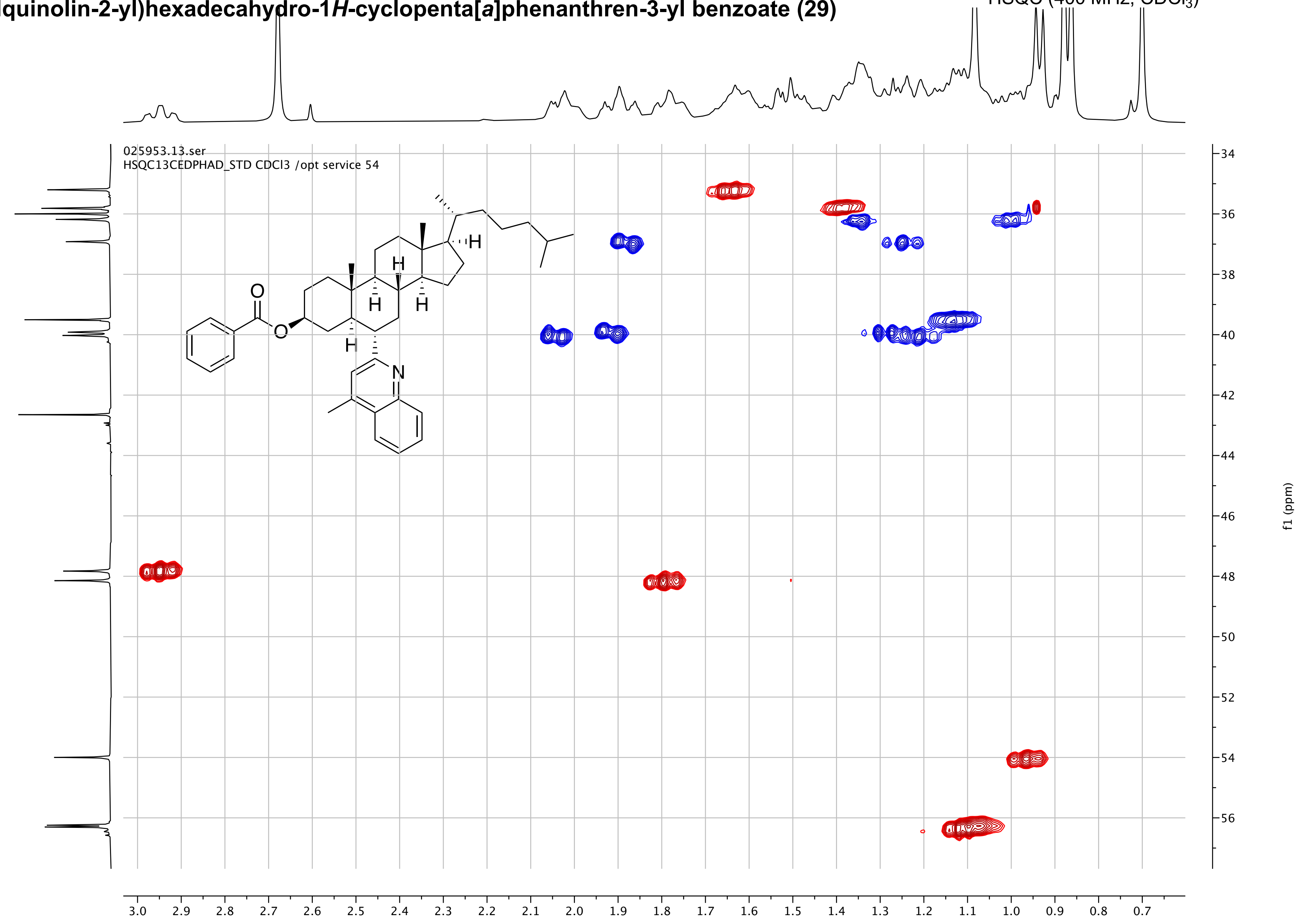

(3*S*,5*R*,6*S*,8*S*,9*S*,10*R*,13*R*,14*S*,17*R*)-10,13-Dimethyl-17-((*R*)-6-methylheptan-2-yl)-6-(4-methylquinolin-2-yl)hexadecahydro-1*H*-cyclopenta[*a*]phenanthren-3-yl benzoate (29)

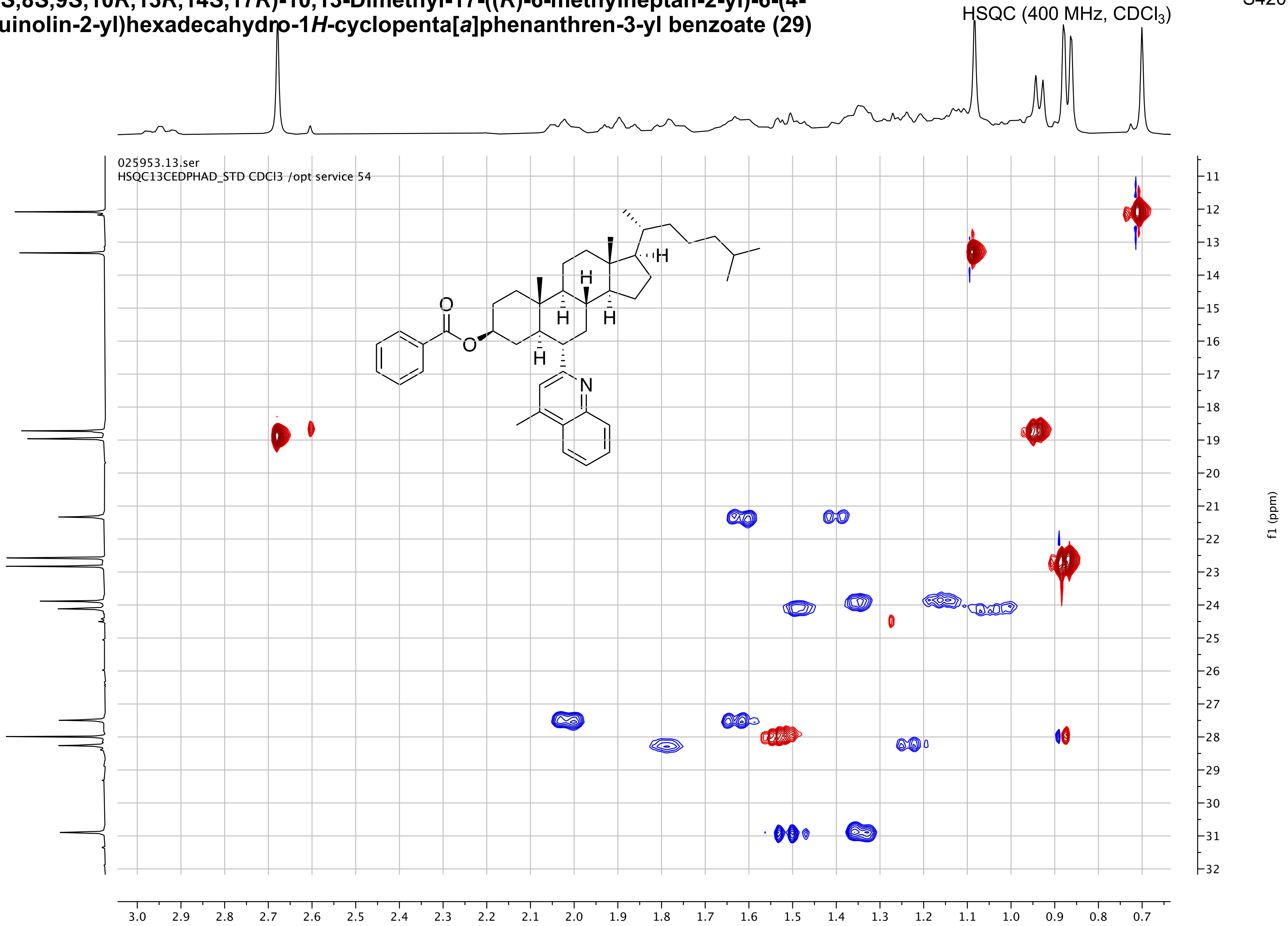

(3*S*,5*R*,6*S*,8*S*,9*S*,10*R*,13*R*,14*S*,17*R*)-10,13-Dimethyl-17-((*R*)-6-methylheptan-2-yl)-6-(4-methylquinolin-2-yl)hexadecahydro-1*H*-cyclopenta[*a*]phenanthren-3-yl benzoate (29)

HMBC (400 MHz, $CDCl_3$)

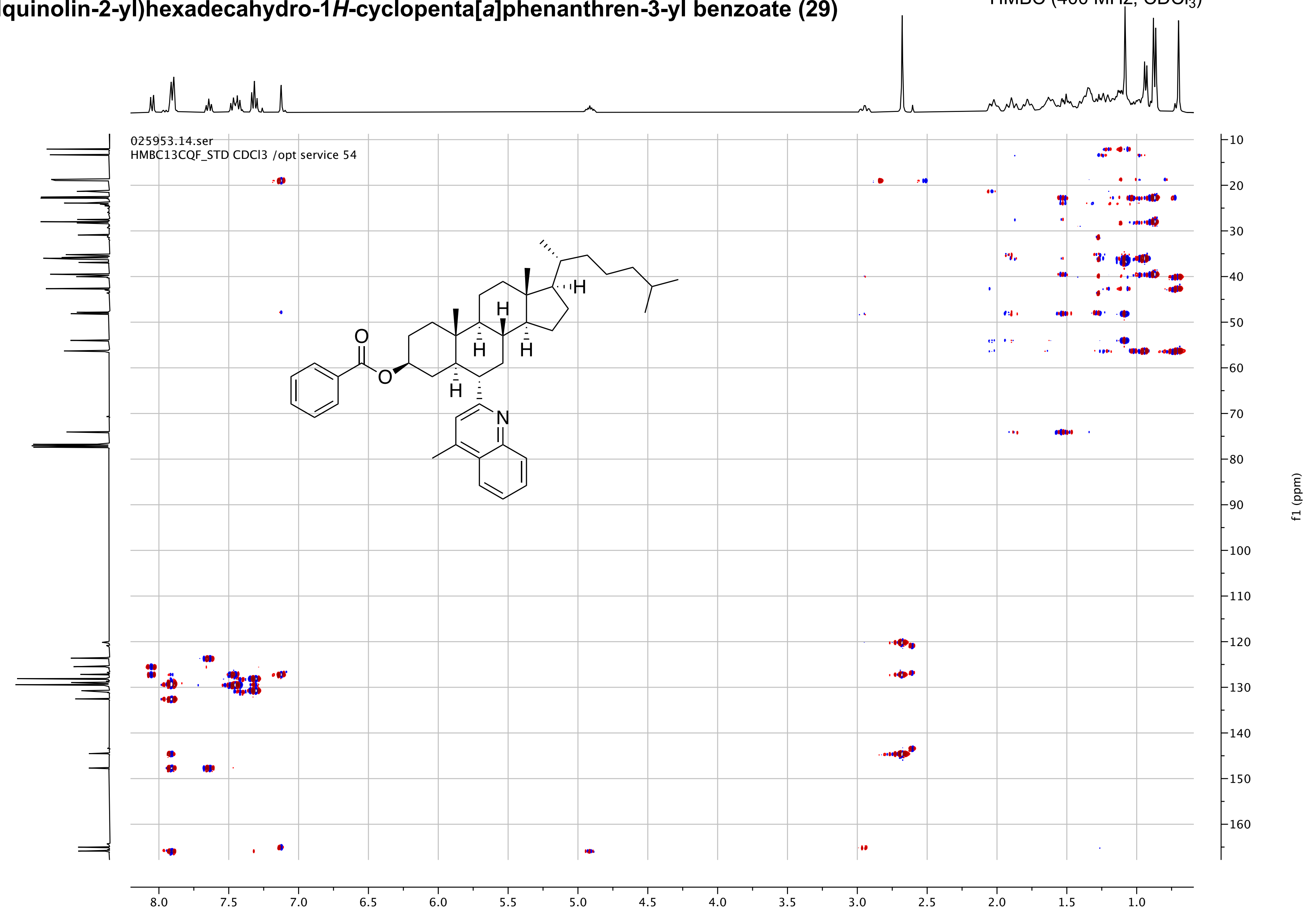

(3*S*,5*R*,6*S*,8*S*,9*S*,10*R*,13*R*,14*S*,17*R*)-10,13-Dimethyl-17-((*R*)-6-methylheptan-2-yl)-6-(4-methylquinolin-2-yl)hexadecahydro-1*H*-cyclopenta[*a*]phenanthren-3-yl benzoate (29)

HMBC (400 MHz, $CDCl_3$)

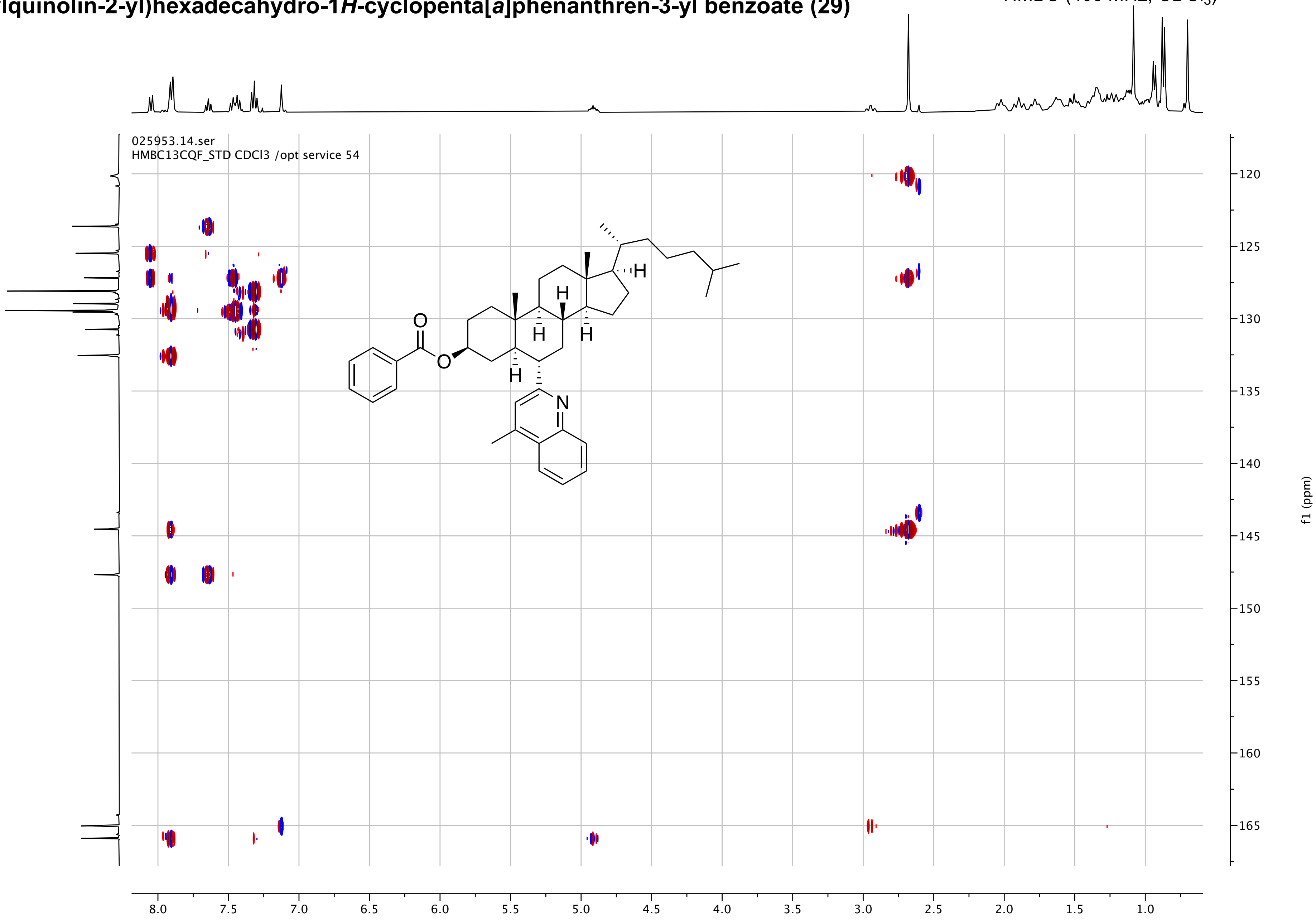

(3*S*,5*R*,6*S*,8*S*,9*S*,10*R*,13*R*,14*S*,17*R*)-10,13-Dimethyl-17-((*R*)-6-methylheptan-2-yl)-6-(4-methylquinolin-2-yl)hexadecahydro-1*H*-cyclopenta[*a*]phenanthren-3-yl benzoate (29)

HMBC (400 MHz, $CDCl_3$)

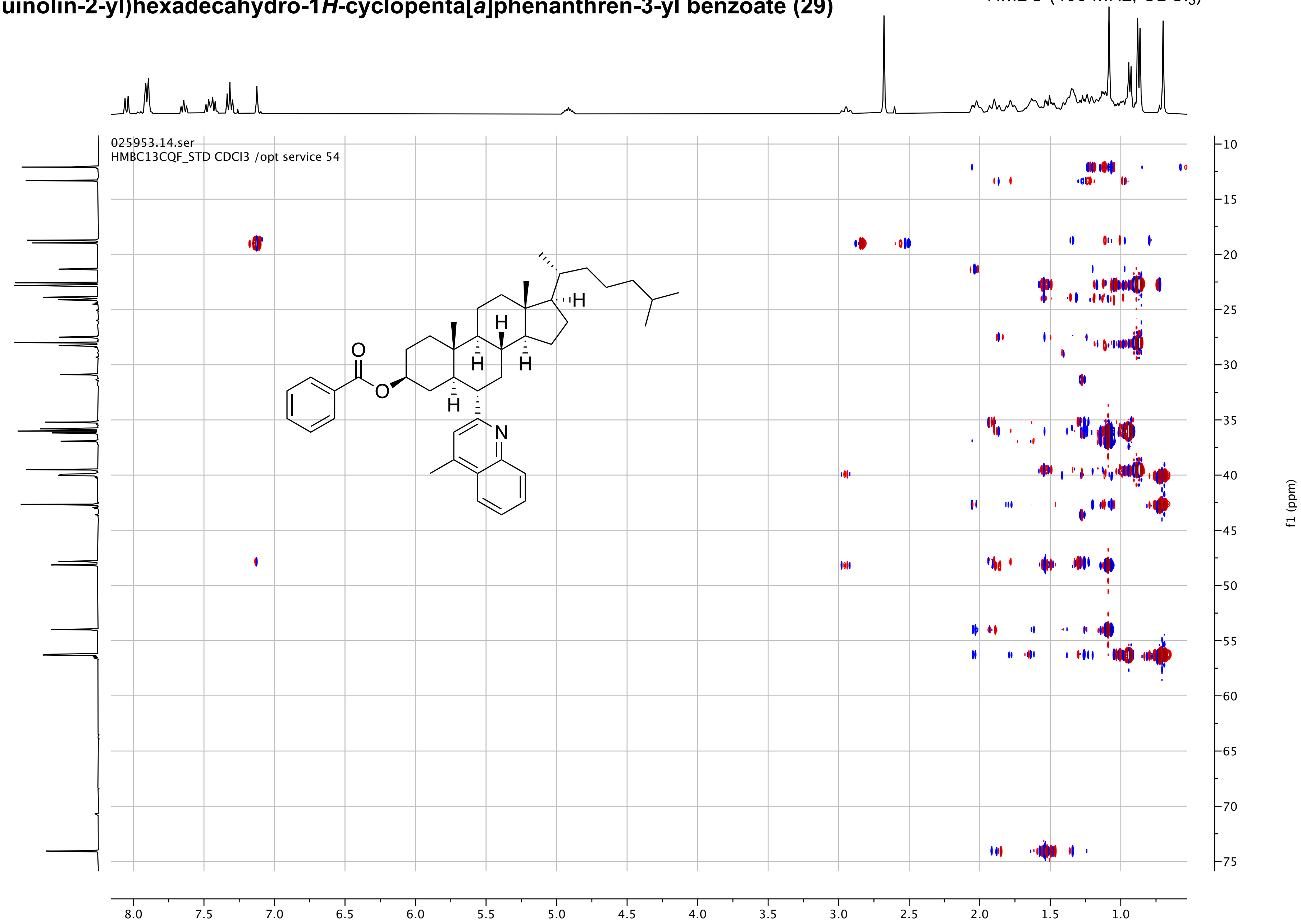

4-Methyl-2-[1-methyl-1-[(1R,4S)-4-methylcyclohex-2-en-1-yl]ethyl]quinoline (30)

^{1}H-NMR, 300 MHz, $CDCl_3$

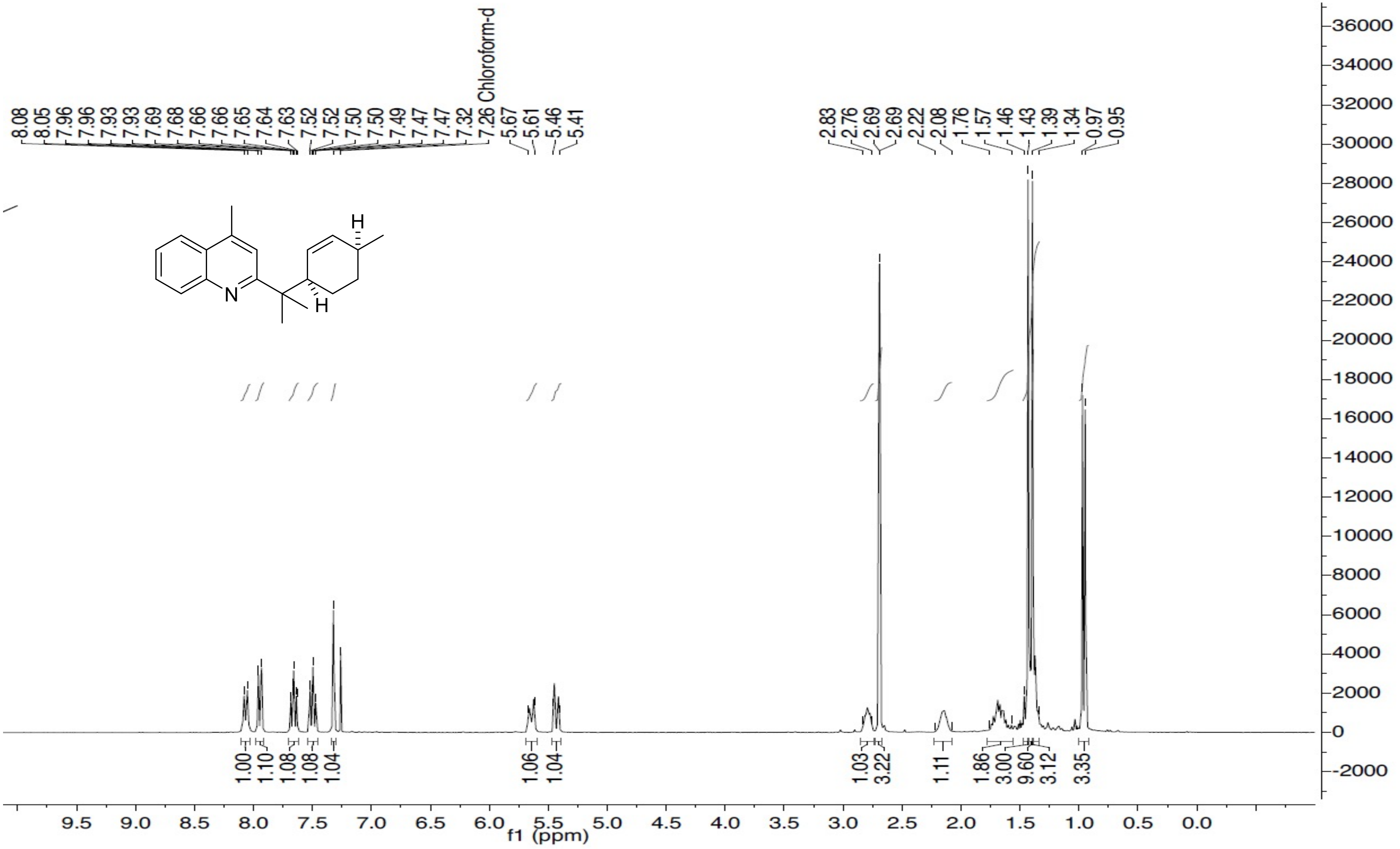

4-Methyl-2-[1-methyl-1-[(1R,4S)-4-methylcyclohex-2-en-1-yl]ethyl]quinoline (30)

^{13}C-NMR, 75 MHz, $CDCl_3$

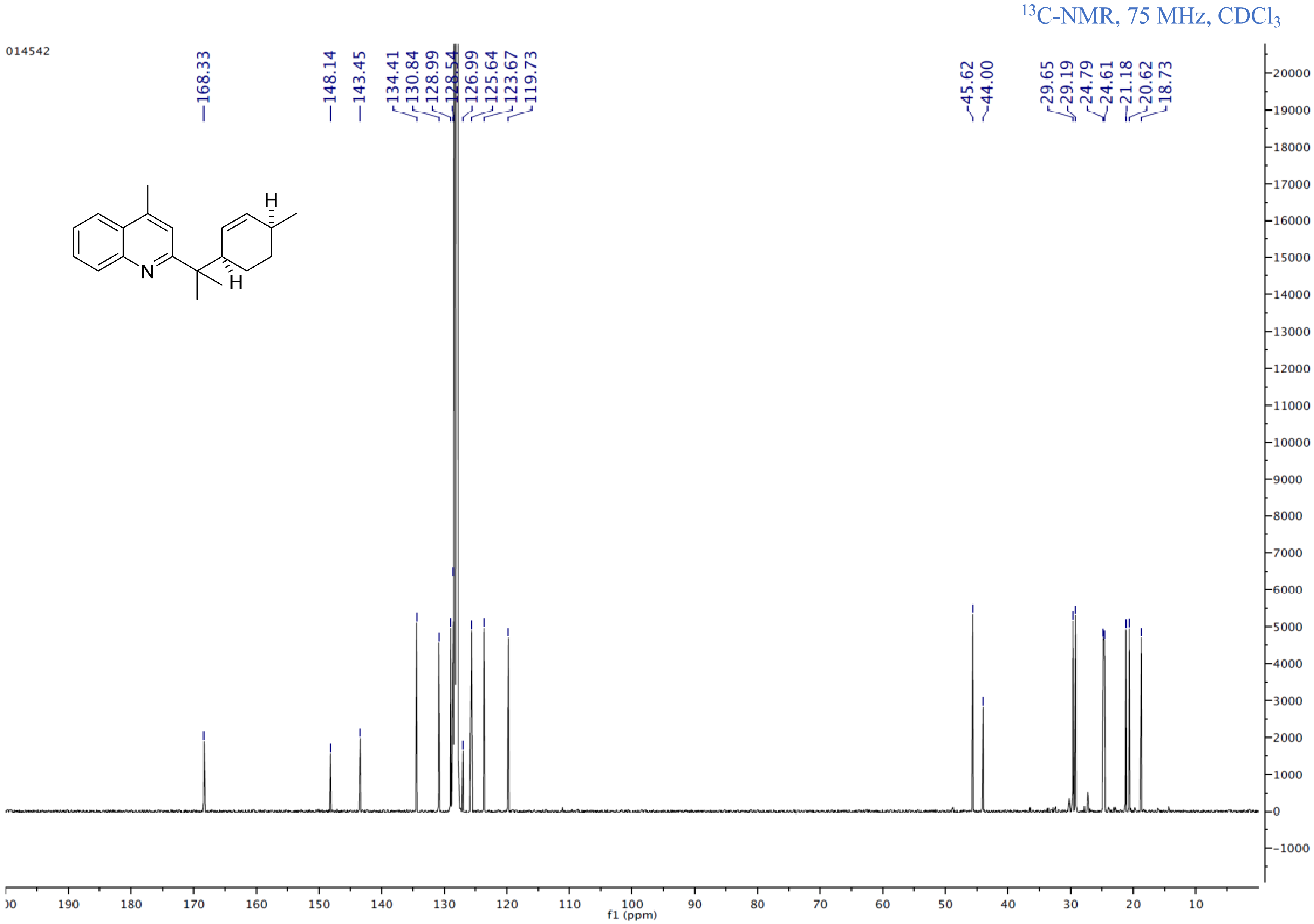

2-Isopropyl-4-methylquinoline (31)

^{1}H-NMR, 300 MHz, $CDCl_3$

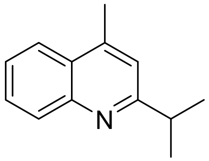

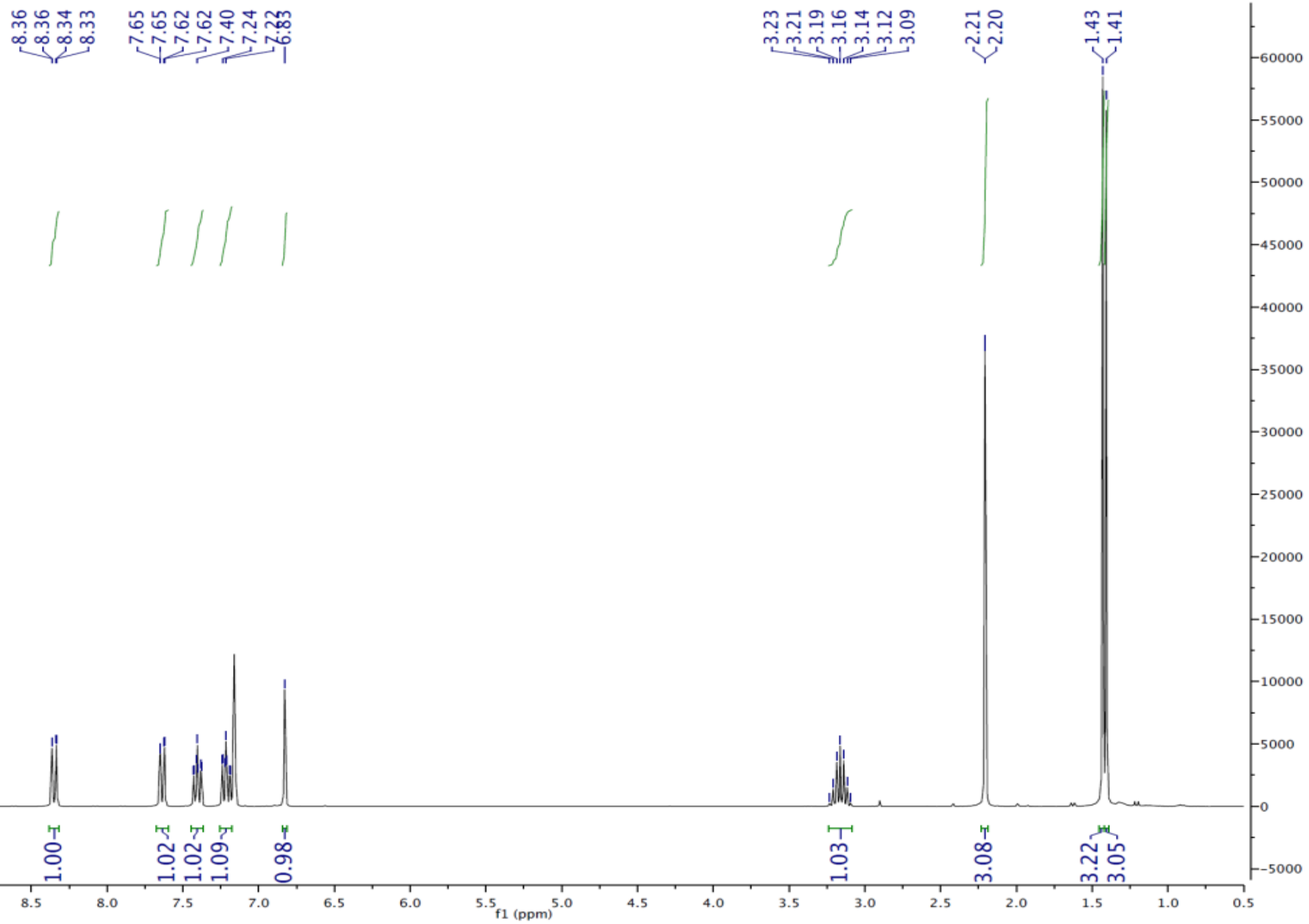

2-Isopropyl-4-methylquinoline (31)

^{13}C-NMR, 75 MHz, $CDCl_3$

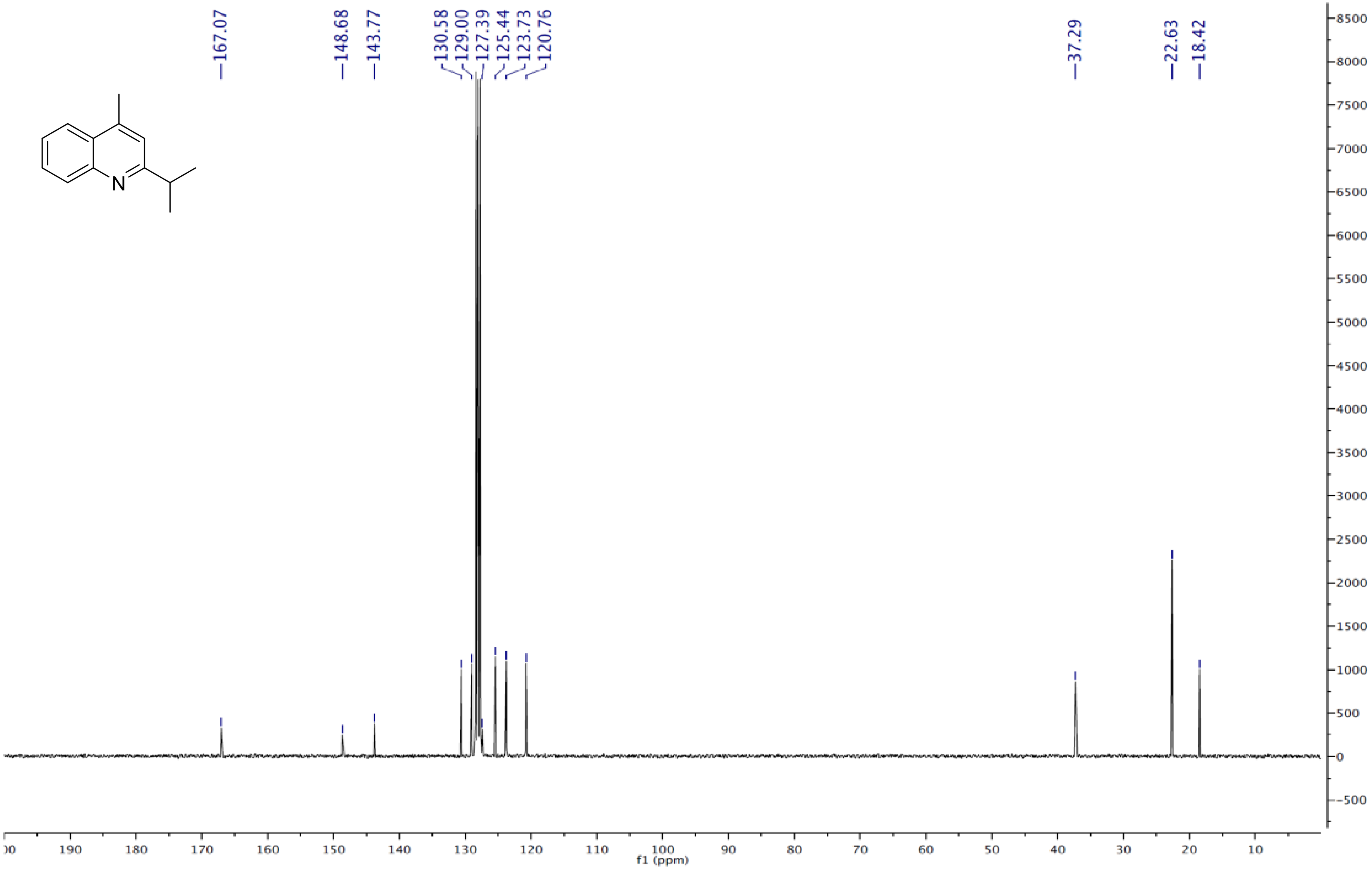

2-(tert-Butyl)-4-phenylpyridine (32)

^{1}H-NMR, 300 MHz, CD_2Cl_2

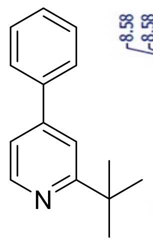

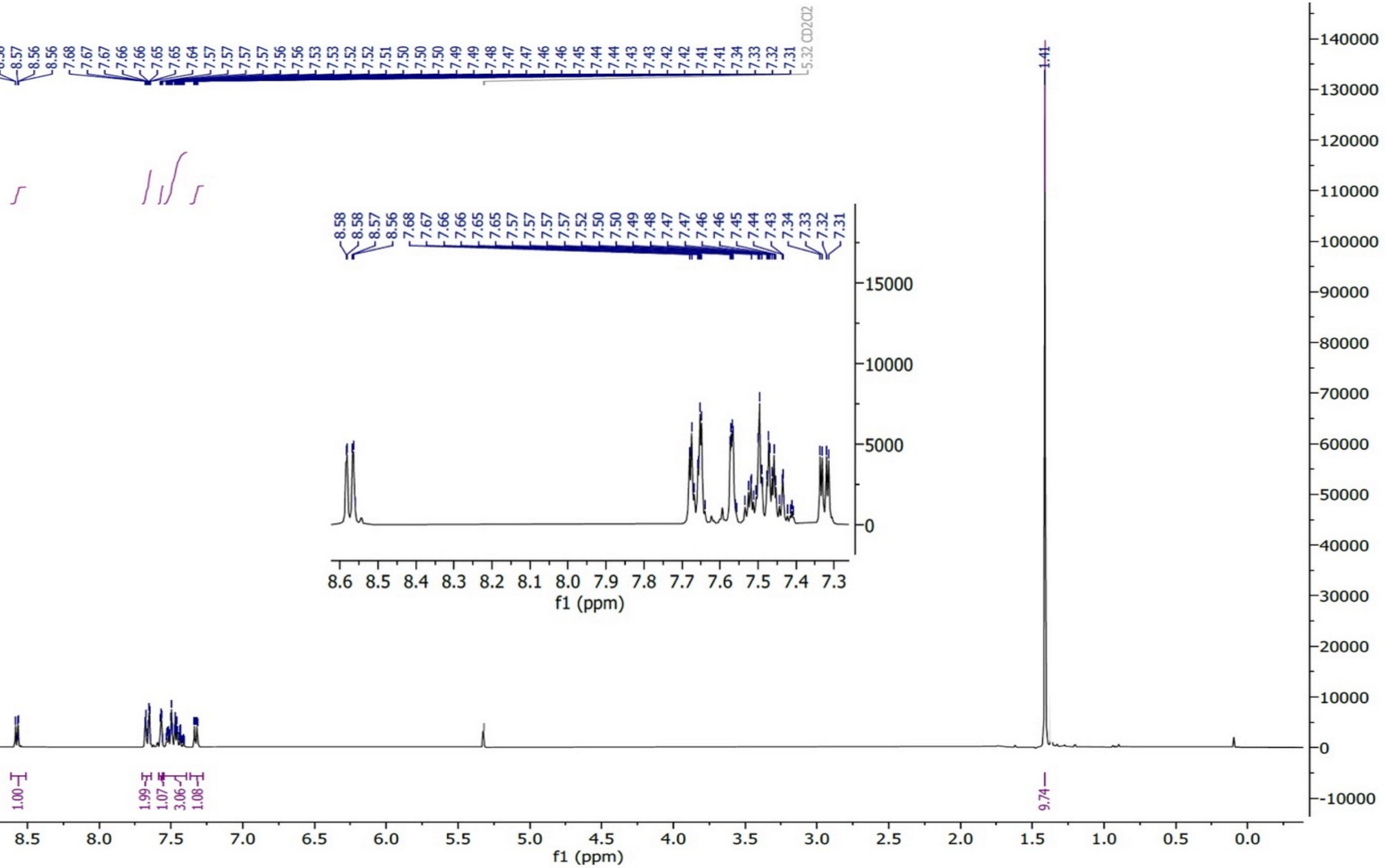

2-(tert-Butyl)-4-phenylpyridine (32)

^{13}C-NMR, 75 MHz, $CDCl_3$

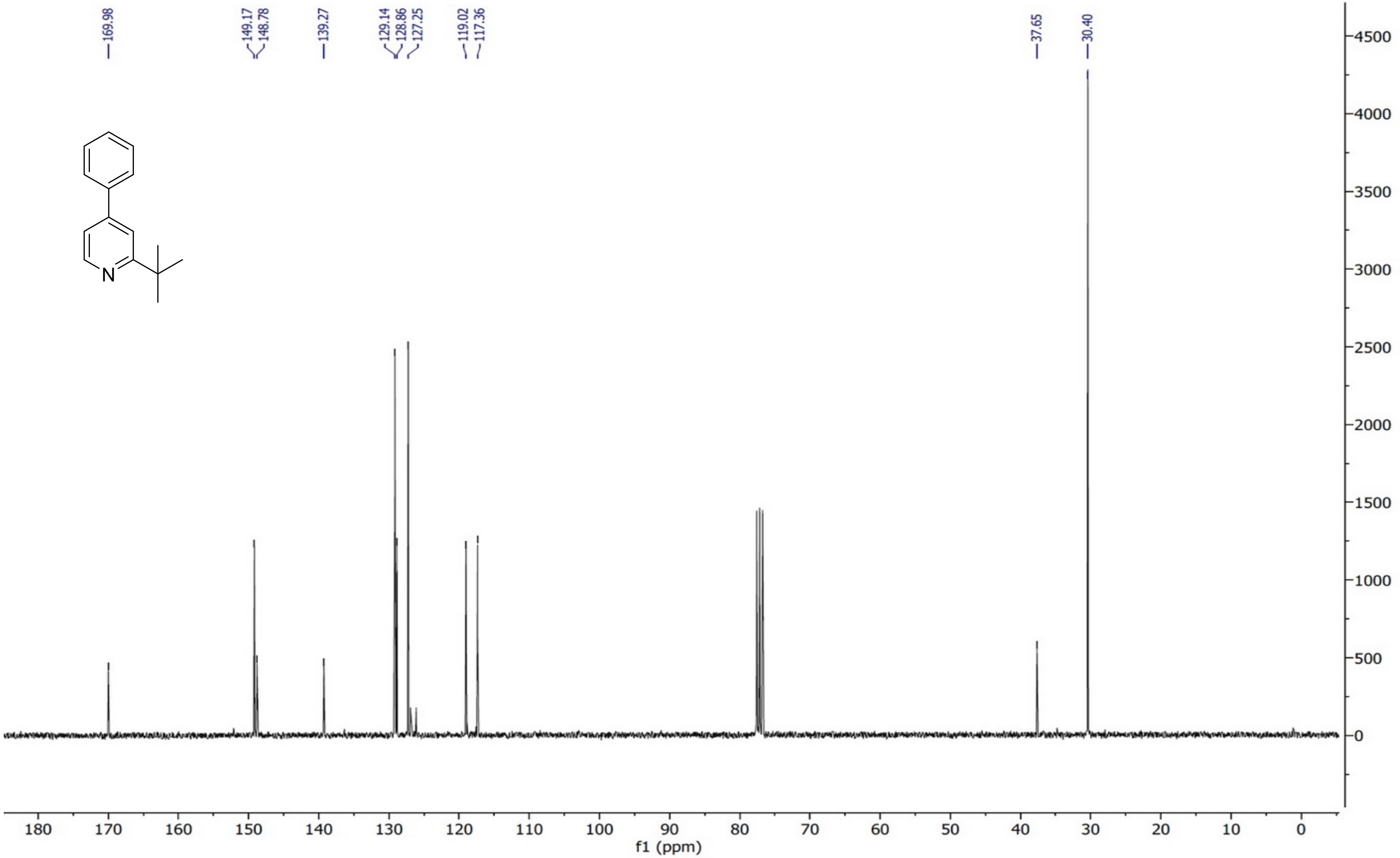

4-Cyclohexyl-2,6-dimethylpyridine (33)

^{1}H-NMR, 300 MHz, $CDCl_3$

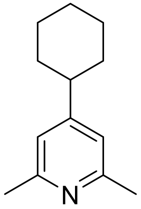

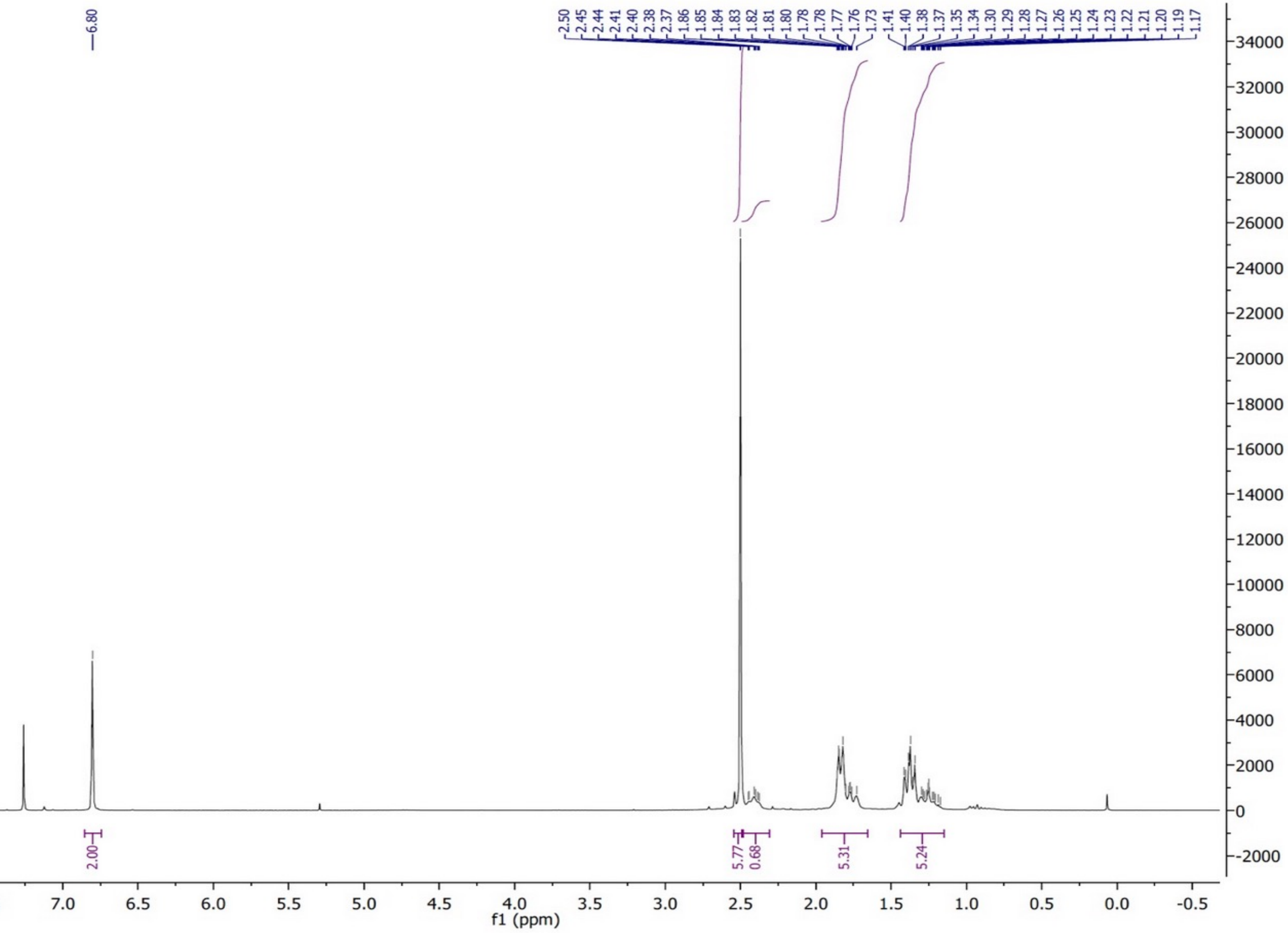

4-Cyclohexyl-2,6-dimethylpyridine (33)

^{13}C-NMR, 75 MHz, $CDCl_3$

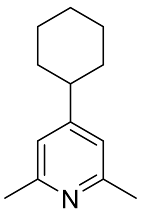

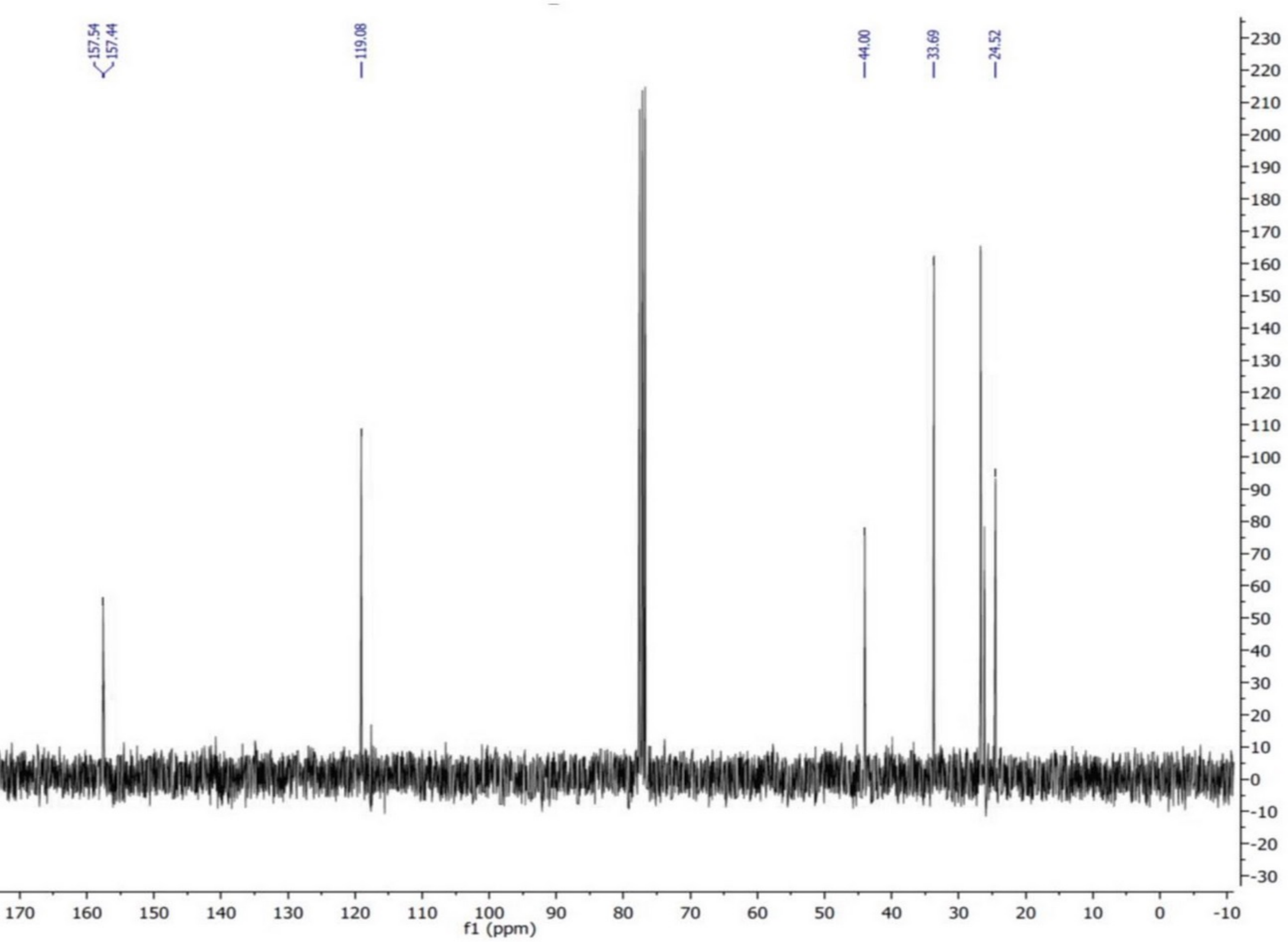

4-Isopropyl-2,6-dimethylpyridine (34)

^{1}H-NMR, 300 MHz, $CDCl_3$

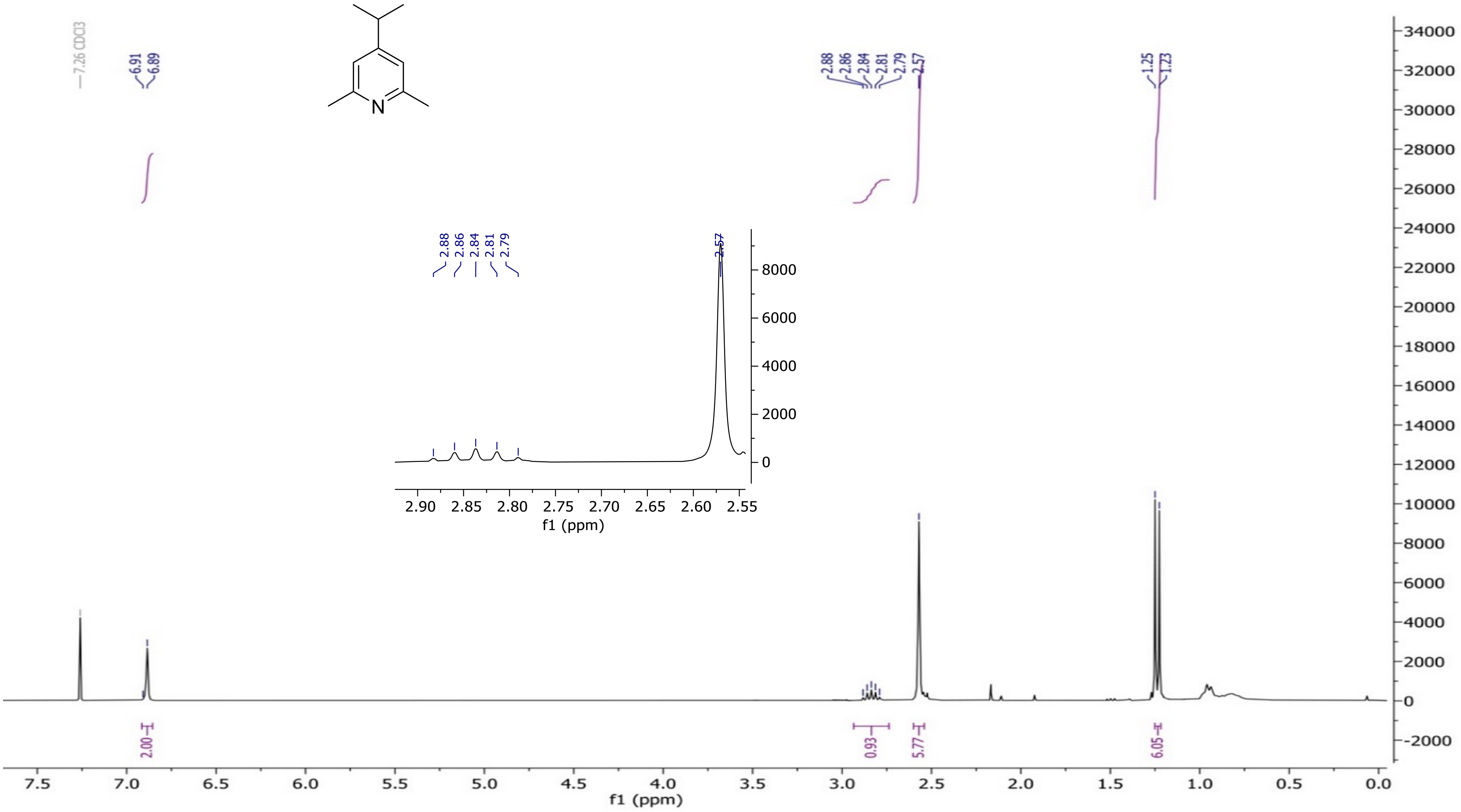

4-Isopropyl-2,6-dimethylpyridine (34)

^{13}C-NMR, 75 MHz, $CDCl_3$

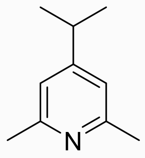

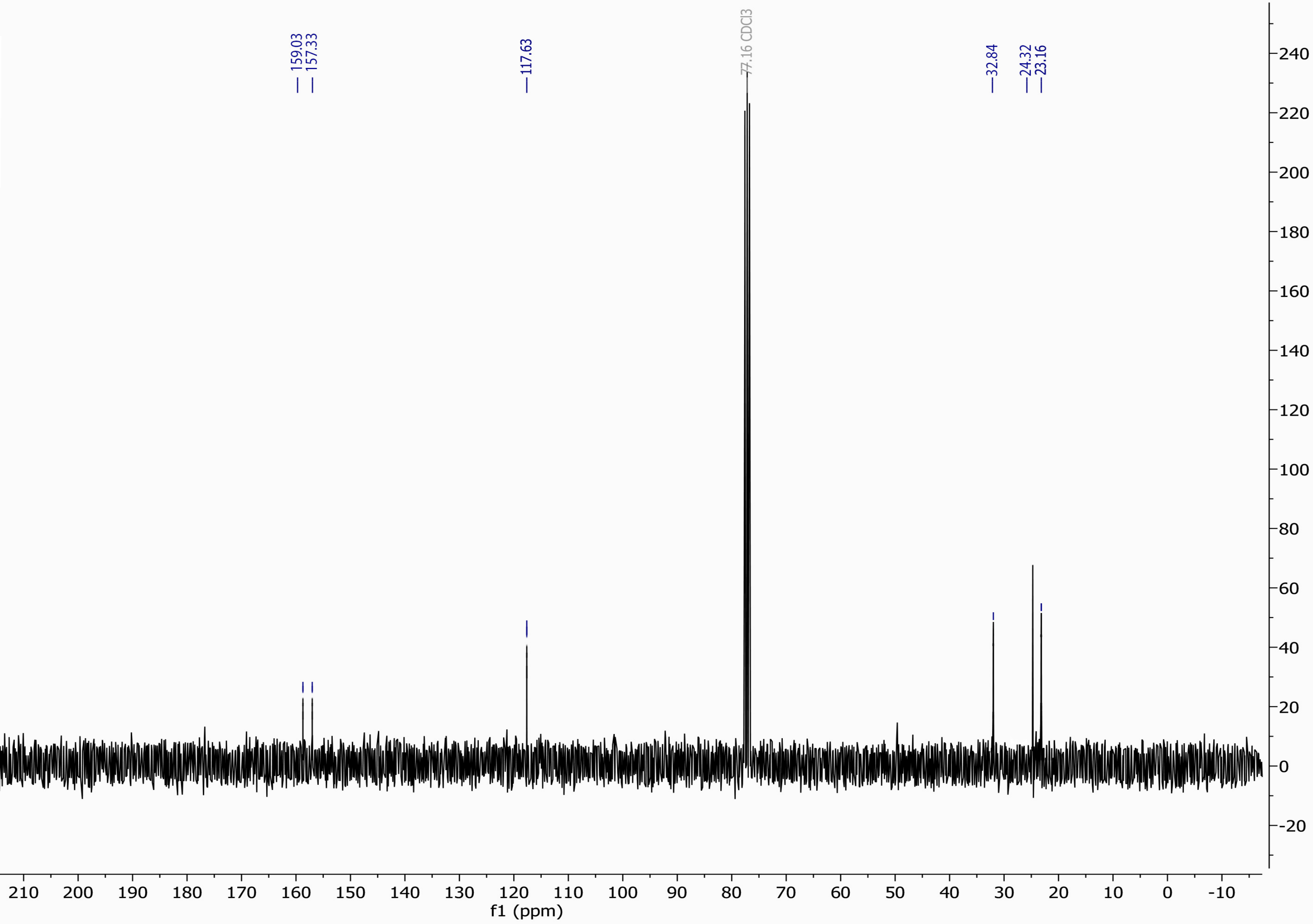

4-(tert-butyl)-2,6-dimethylpyridine (35)

^{1}H-NMR, 300 MHz, $CDCl_3$

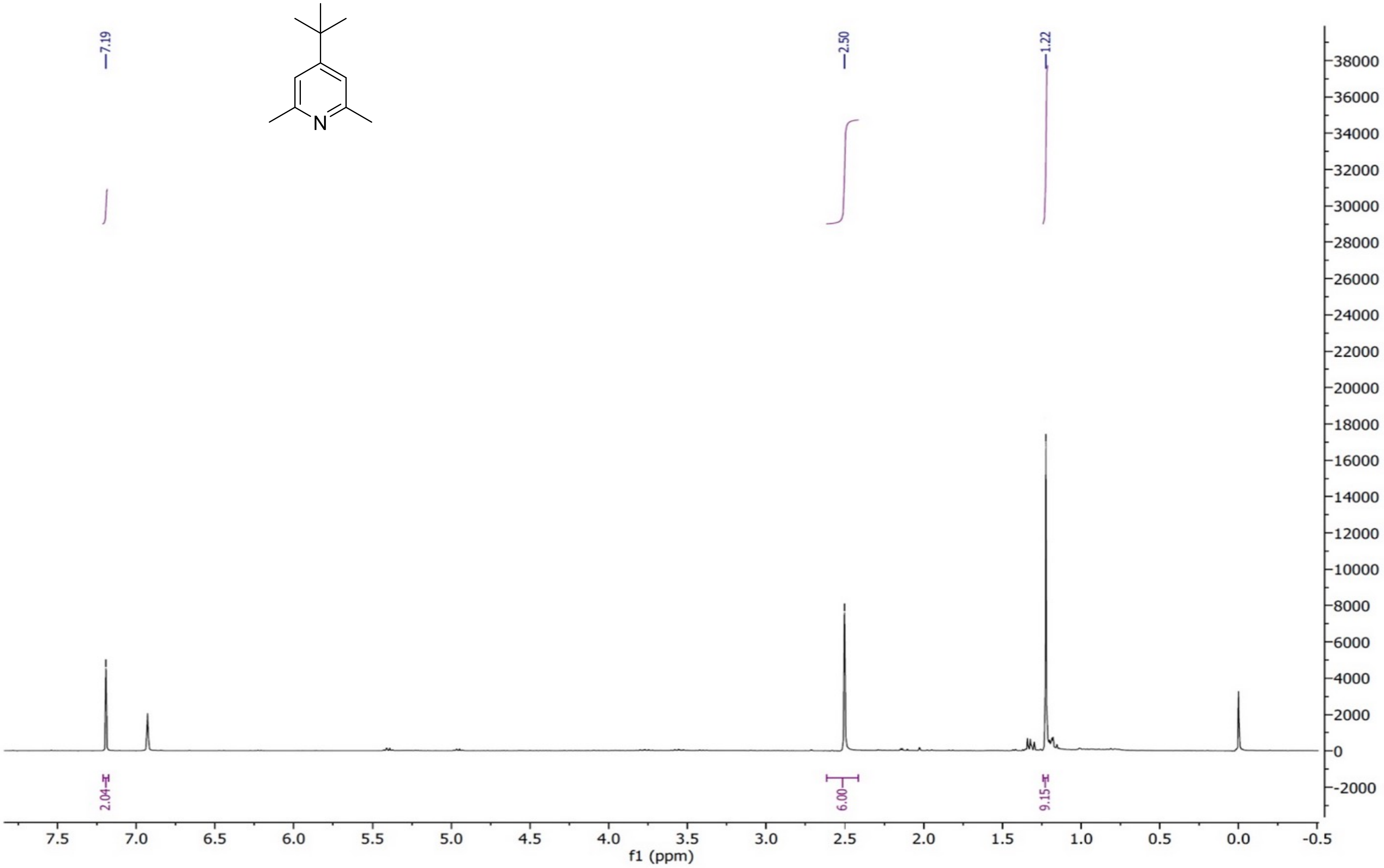

4-(tert-Butyl)-2,6-dimethylpyridine (35)

^{13}C-NMR, 75 MHz, $CDCl_3$

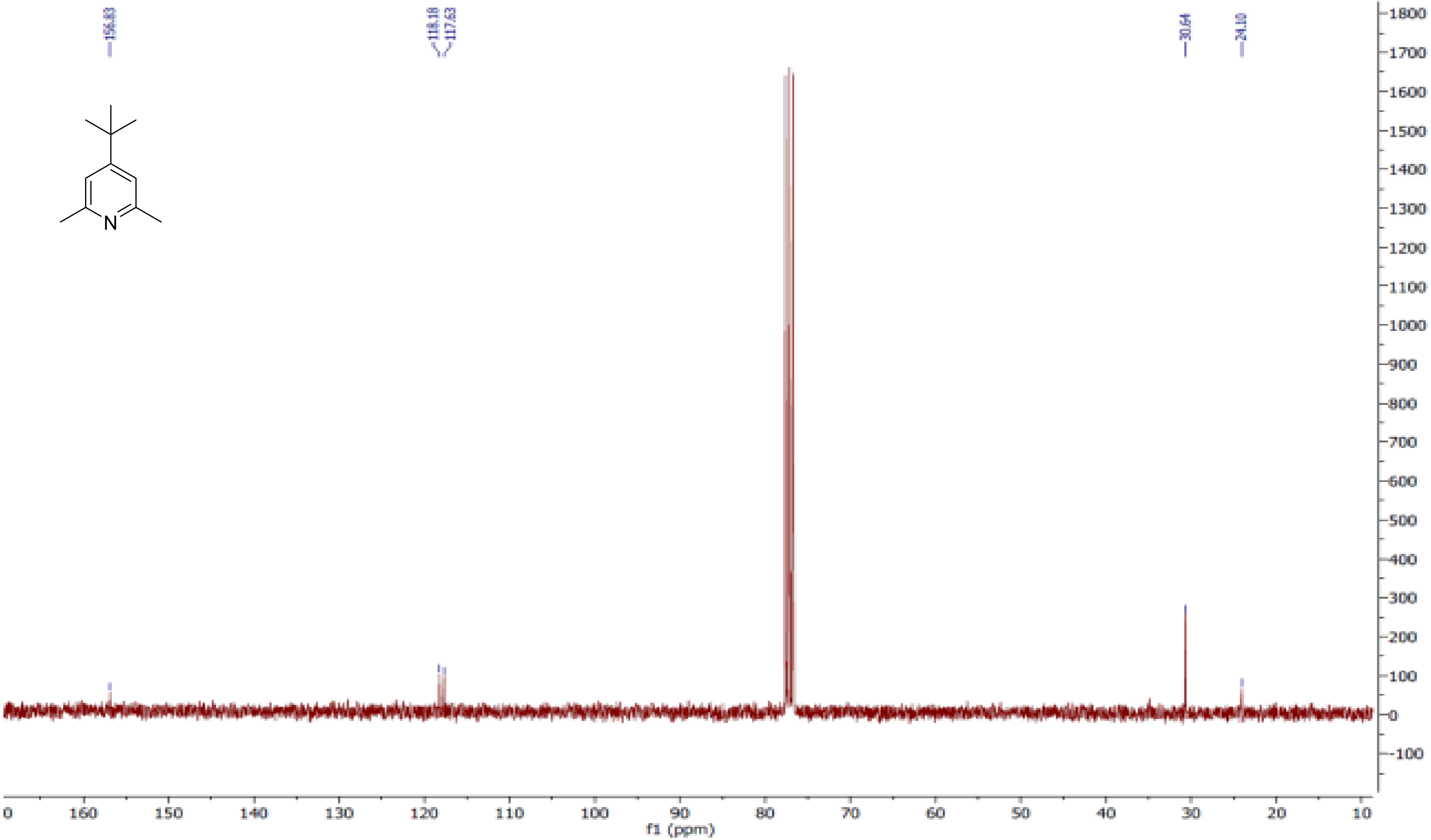

4-Adamantyl-2,6-dimethylpyridine (36)

^{1}H-NMR, 300 MHz, $CDCl_3$

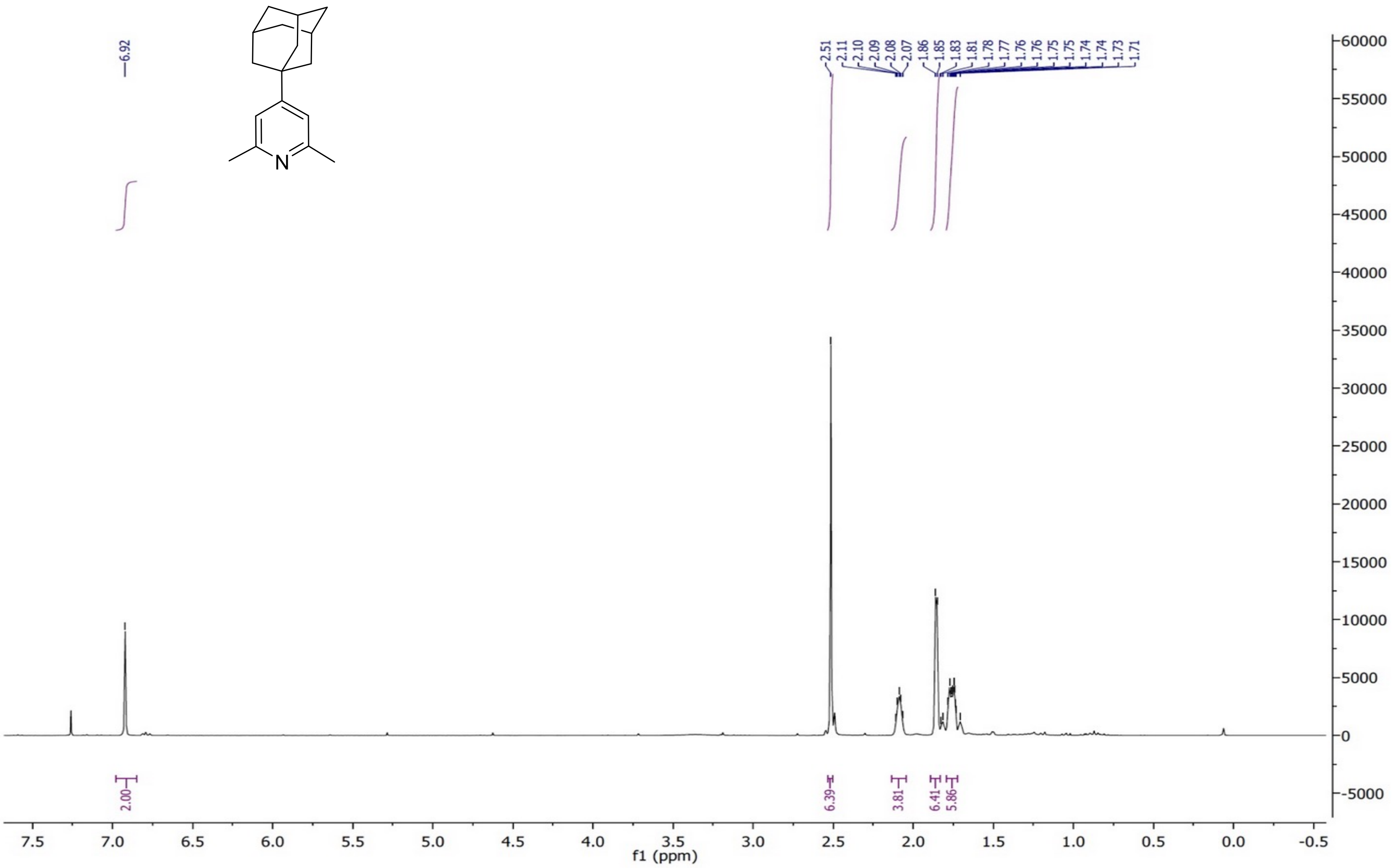

4-Adamantyl-2,6-dimethylpyridine (36)

^{13}C-NMR, 75 MHz, $CDCl_3$

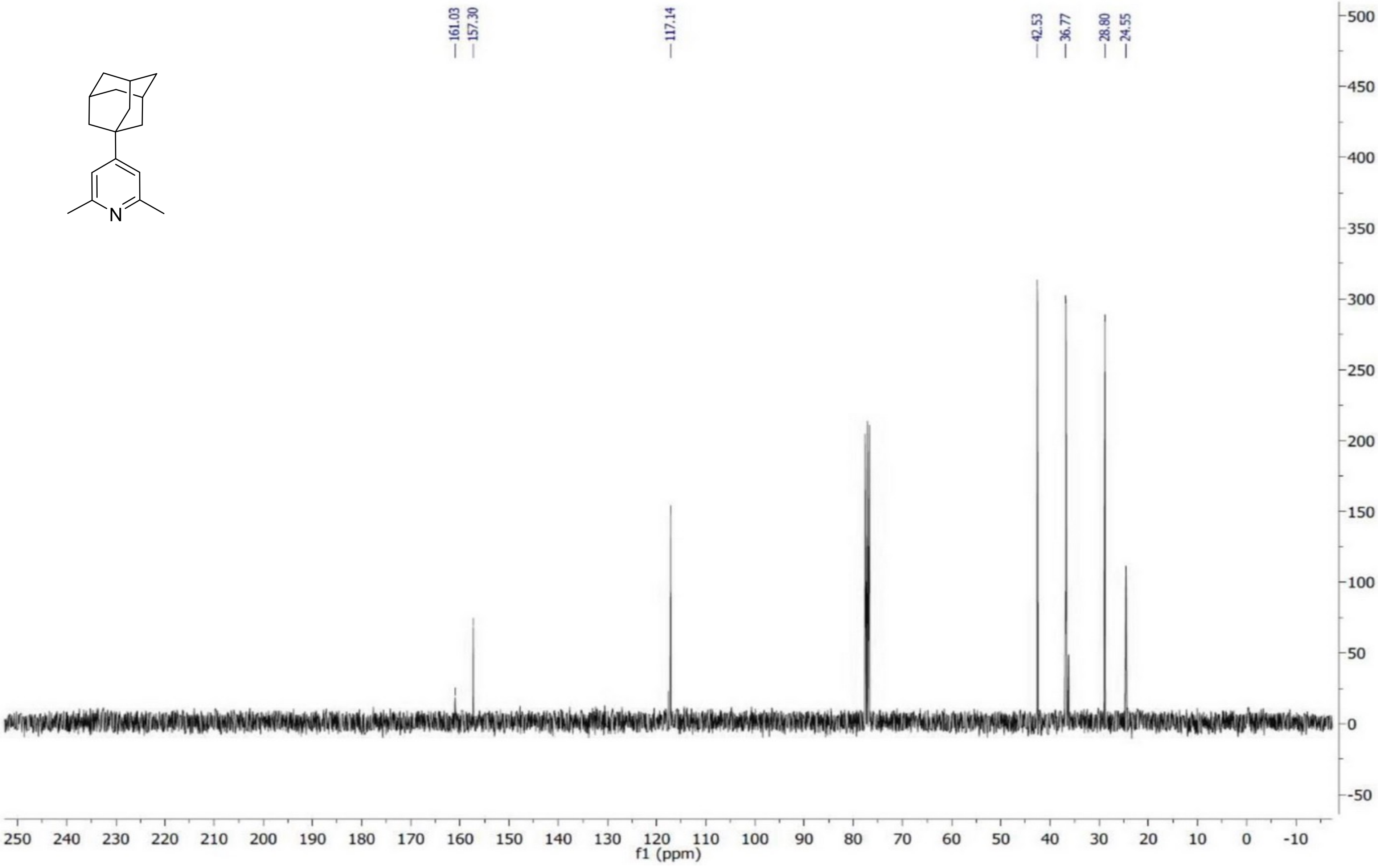

4-((3R,8S,9S,10R,13R,14S,17R)-10,13-Dimethyl-17-((R)-6-methylheptan-2-yl)-2,3,4,7,8,9,10,11,12,13,14,15,16,17-tetradecahydro-1H-cyclopenta[a]phenanthren-3-yl)-2,6-dimethylpyridine (37)

^{1}H-NMR, 300 MHz, $CDCl_3$

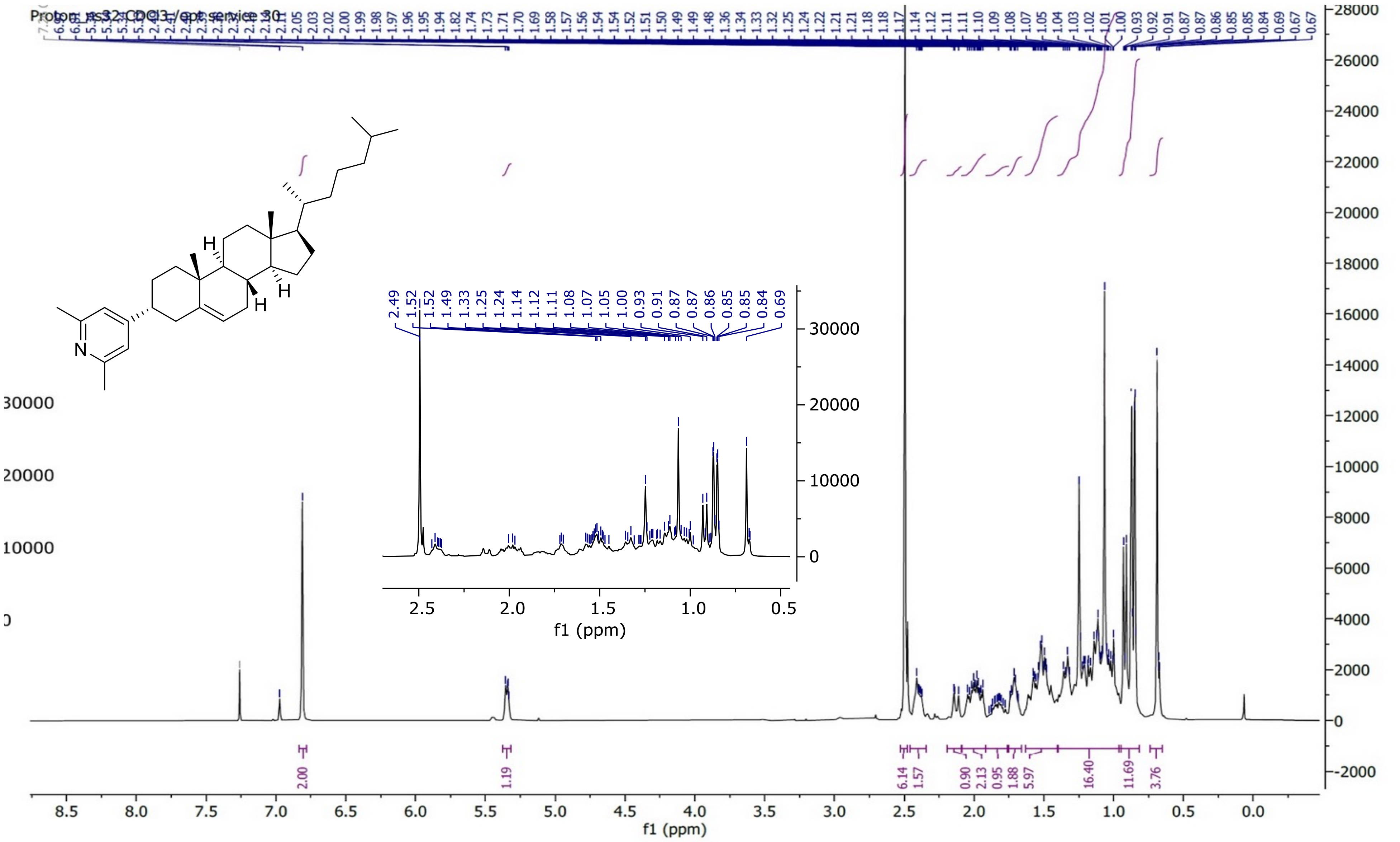

4-((3R,8S,9S,10R,13R,14S,17R)-10,13-Dimethyl-17-((R)-6-methylheptan-2-yl)-2,3,4,7,8,9,10,11,12,13,14,15,16,17-tetradecahydro-1H-cyclopenta[a]phenanthren-3-yl)-2,6-dimethylpyridine (37)

^{13}C-NMR, 75 MHz, $CDCl_3$

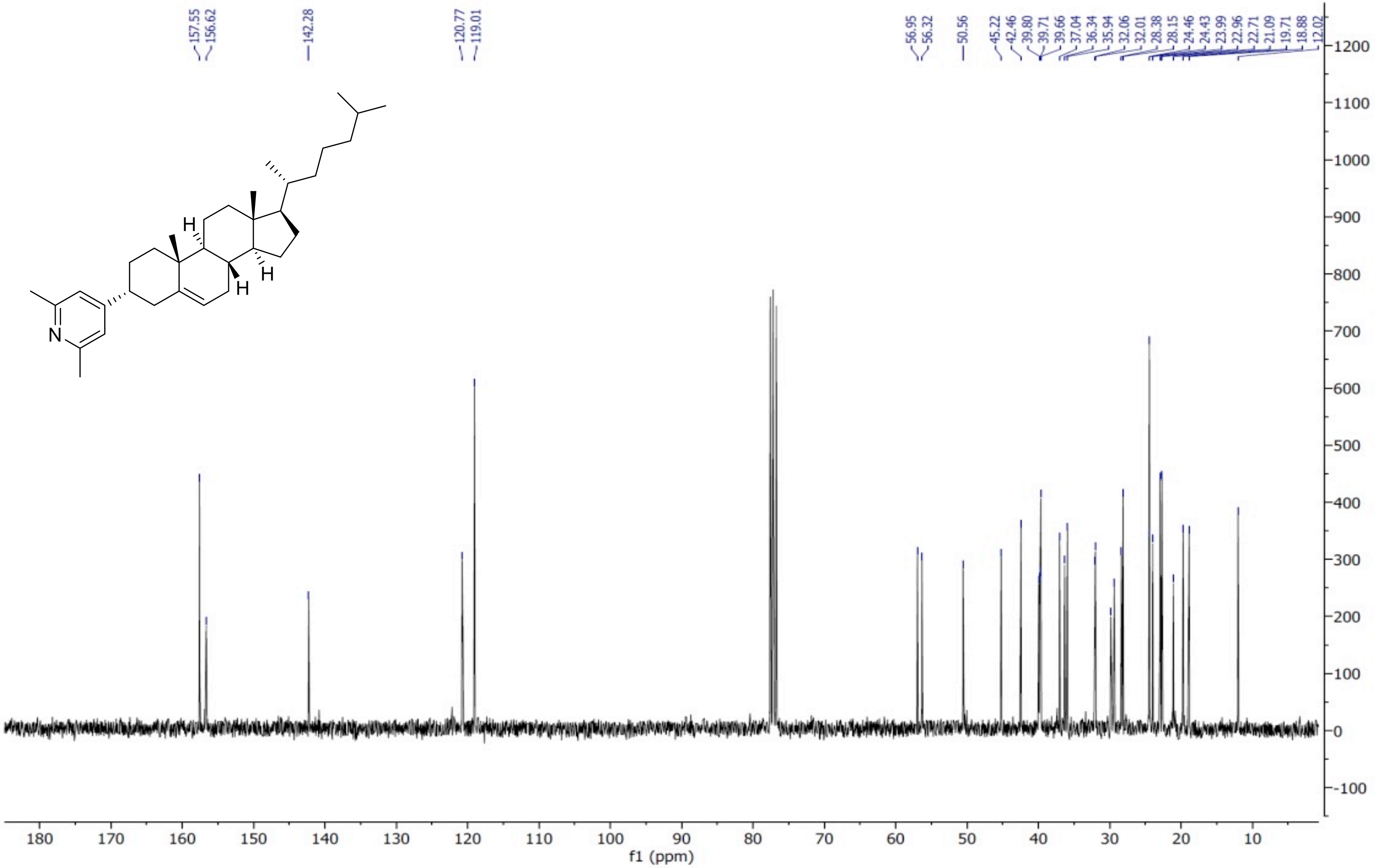

4-Chloro-2-cyclohexylpyridine (38)

^{1}H-NMR, 300 MHz, $CDCl_3$

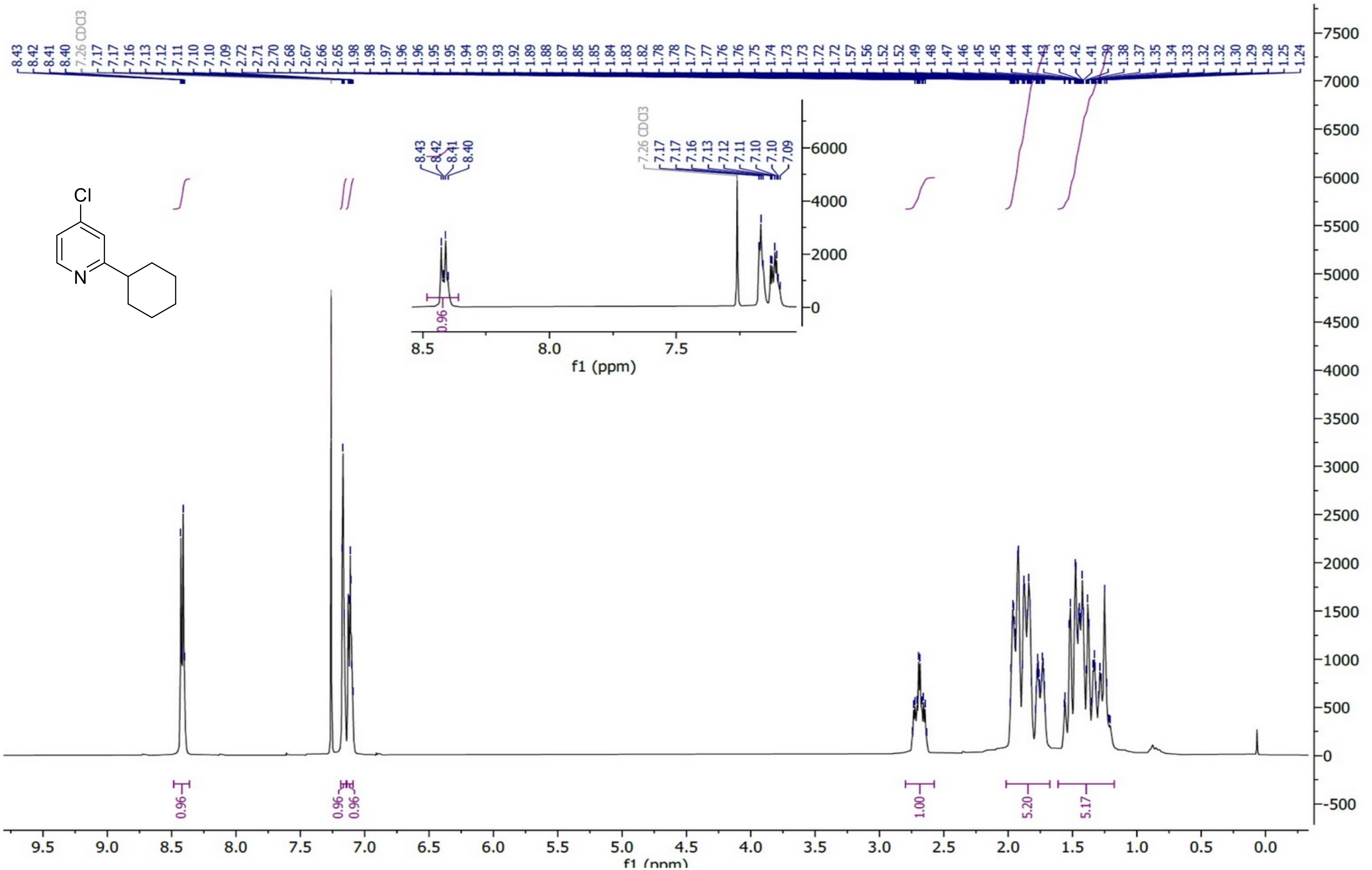

4-Chloro-2-cyclohexylpyridine (38)

^{13}C-NMR, 75 MHz, $CDCl_3$

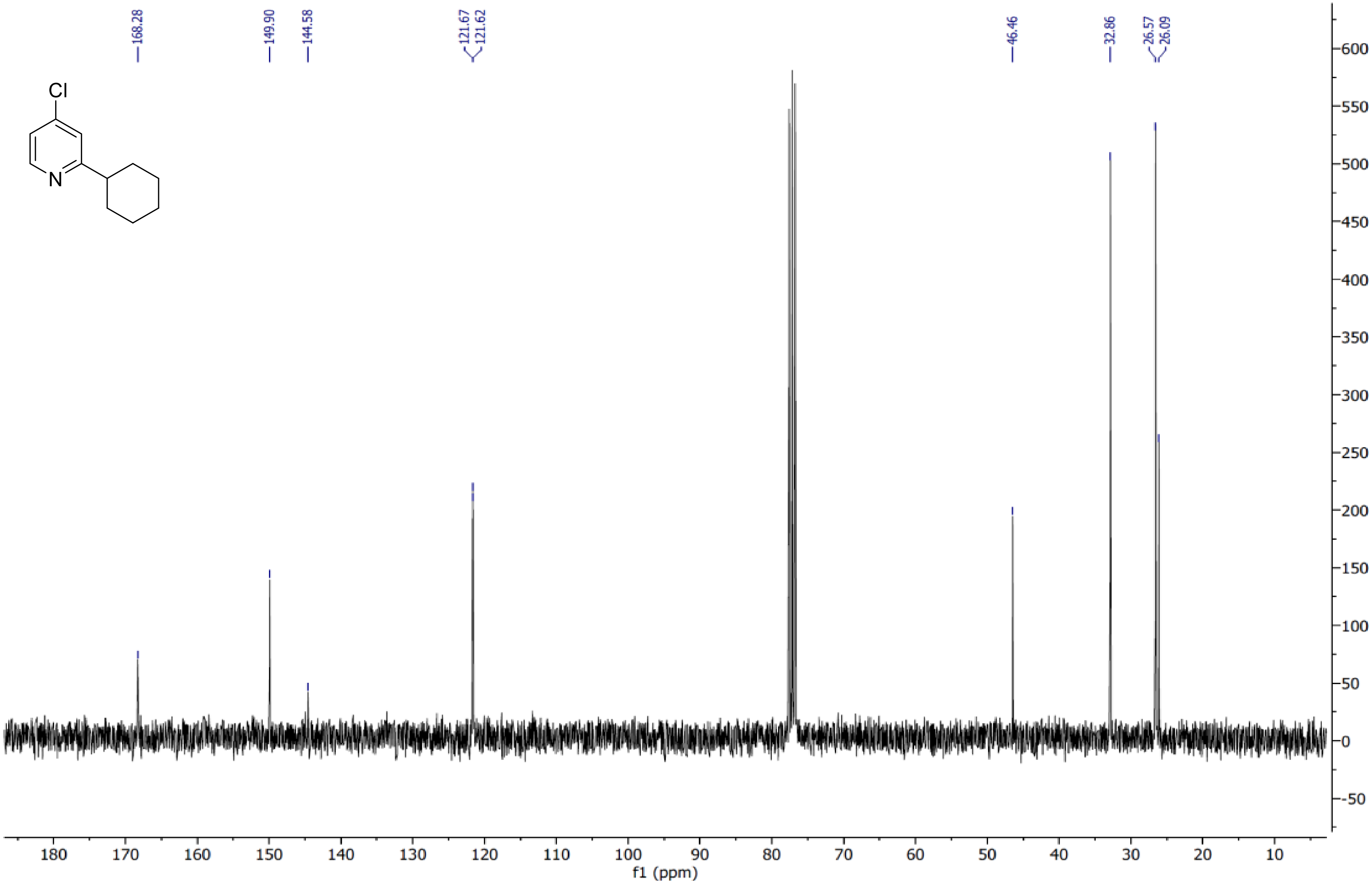

4-Chloro-2-isopropylpyridine (39)

^{1}H-NMR, 300 MHz, $CDCl_3$

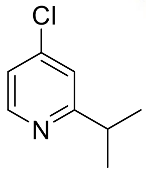

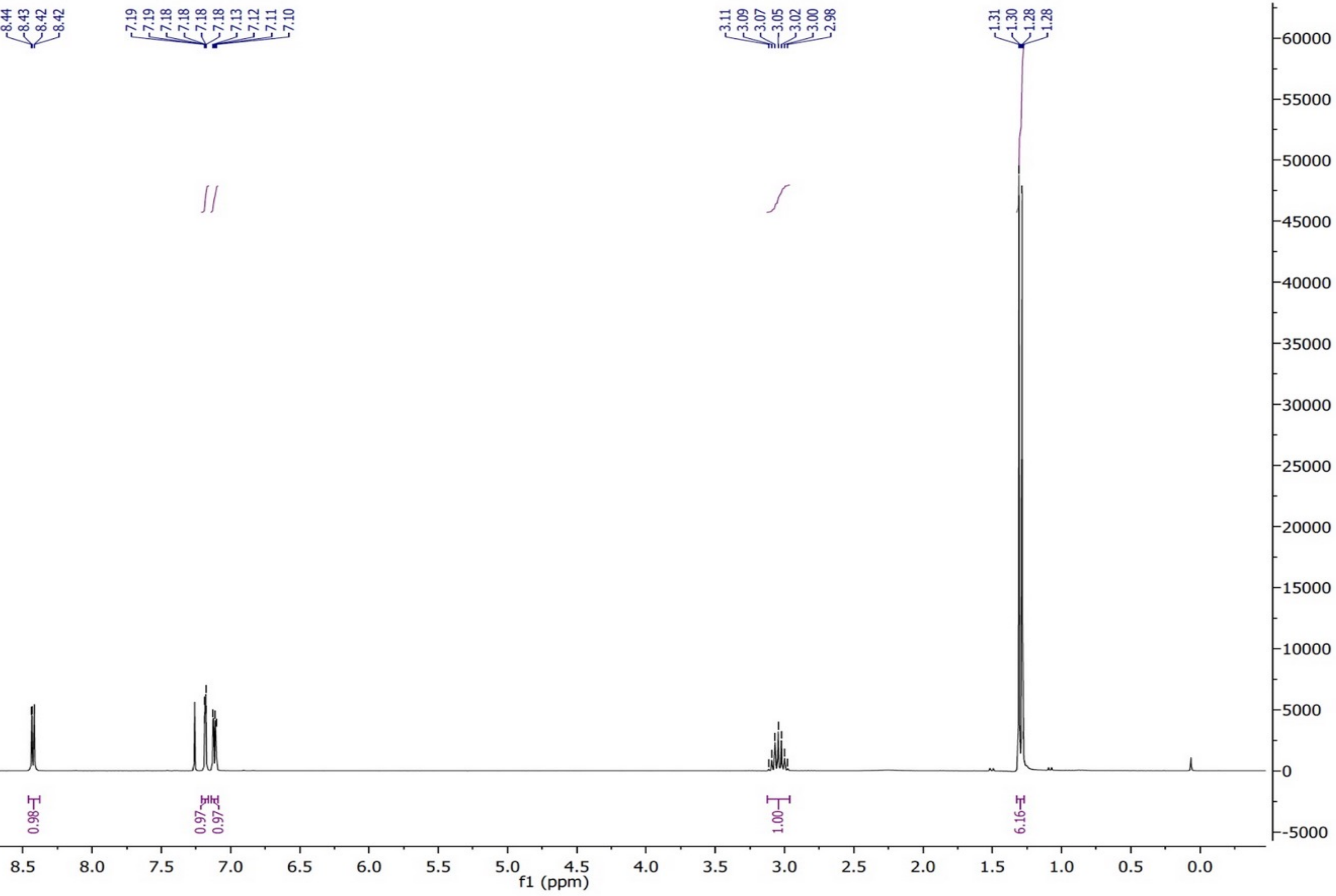

4-Chloro-2-isopropylpyridine (39)

^{13}C-NMR, 75 MHz, $CDCl_3$

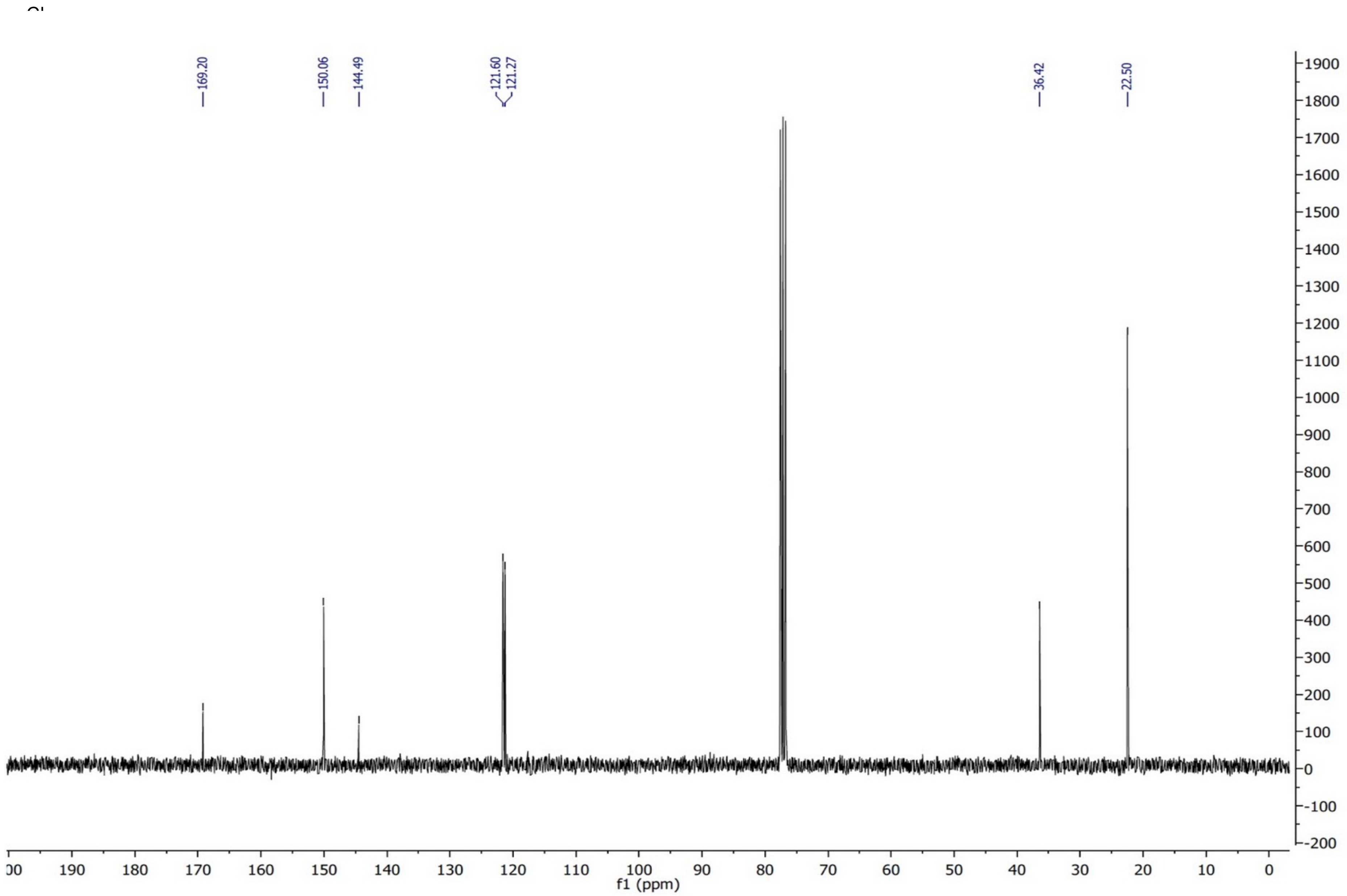

4-Chloro-2-isopropylpyridine (40)

^{1}H-NMR, 300 MHz, $CDCl_3$

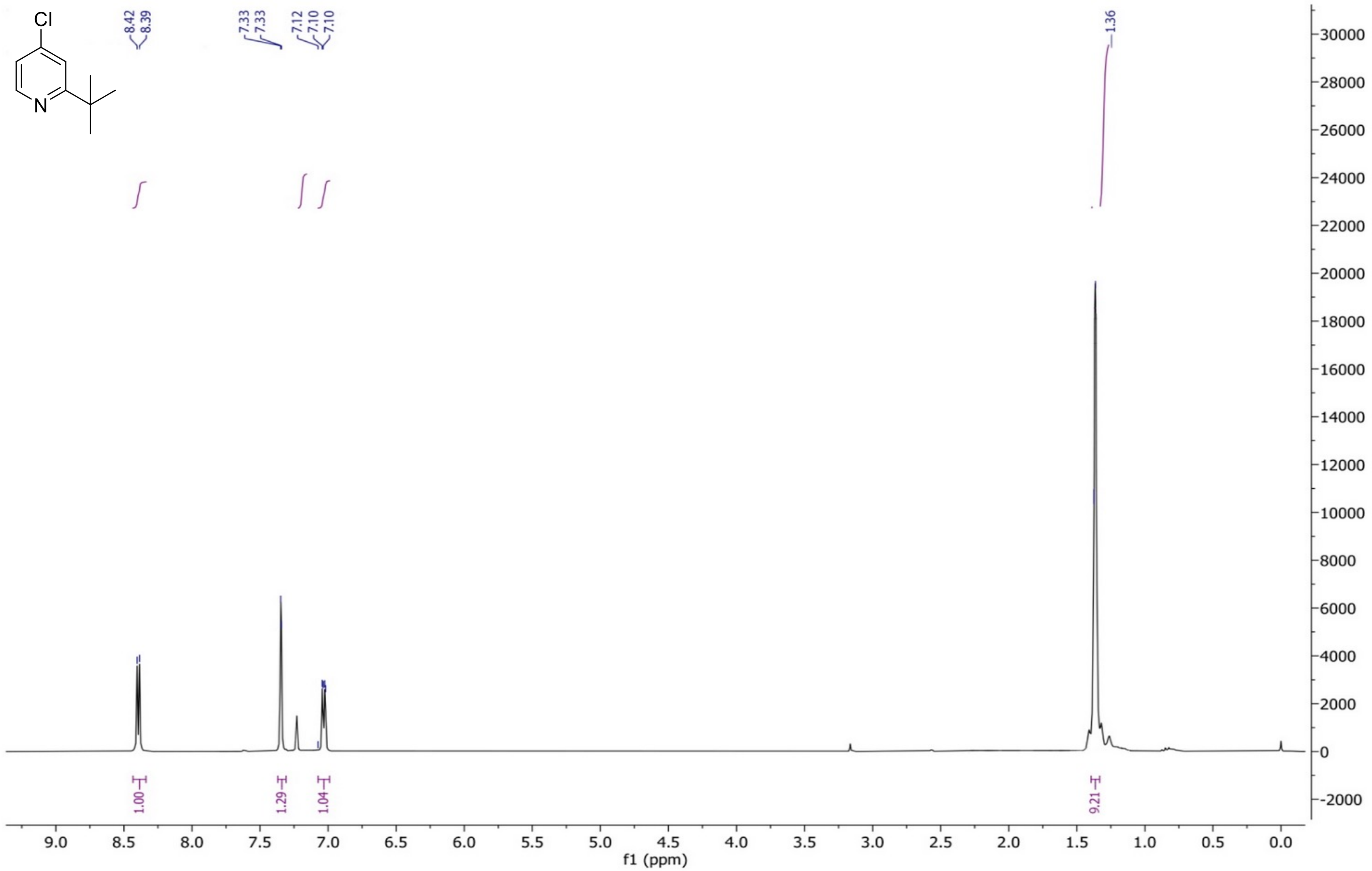

4-Chloro-2-isopropylpyridine (40)

^{13}C-NMR, 75 MHz, $CDCl_3$

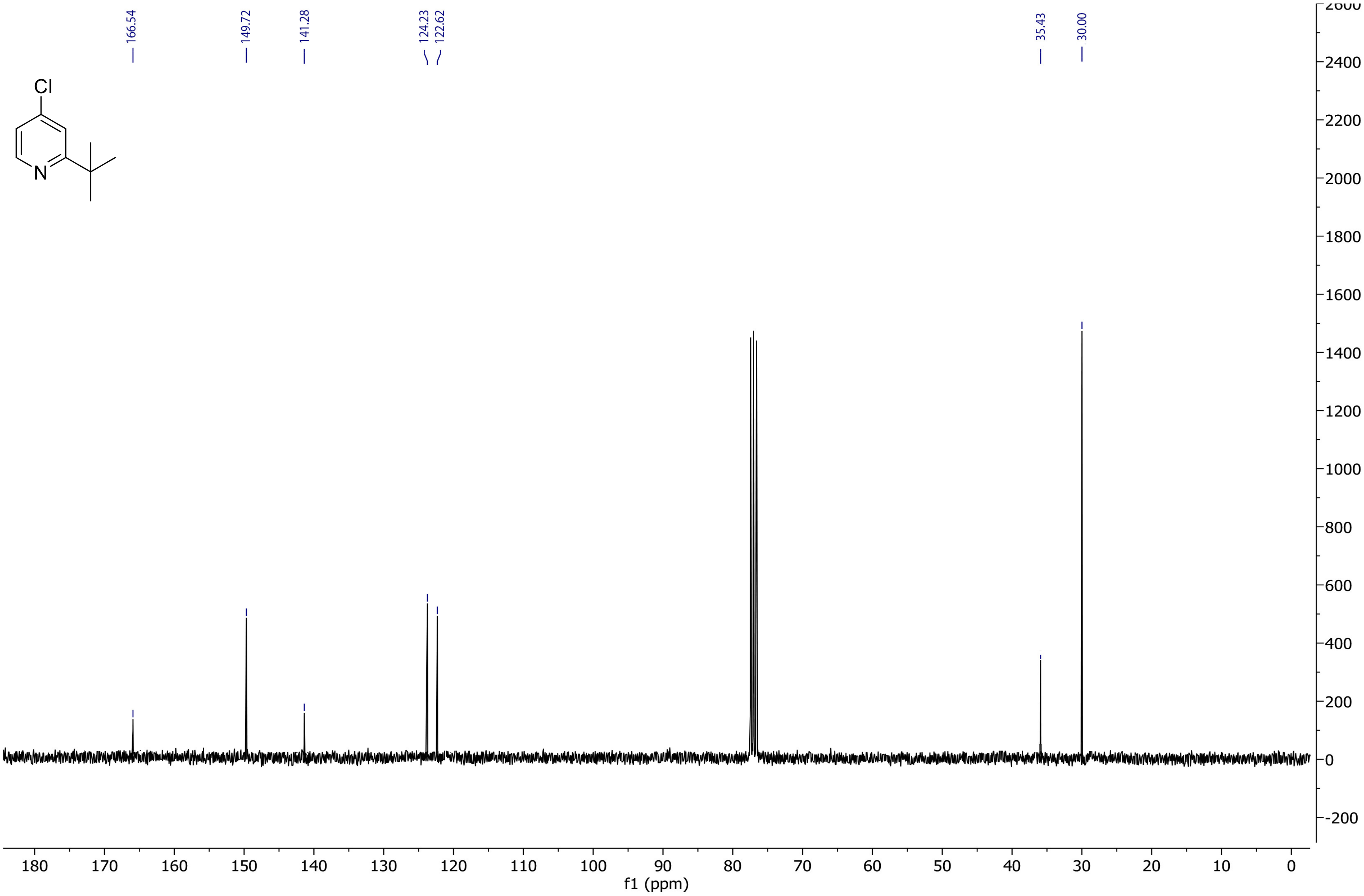

4-Chloro-2-adamantylpyridine (41)

^{1}H-NMR, 300 MHz, $CDCl_3$

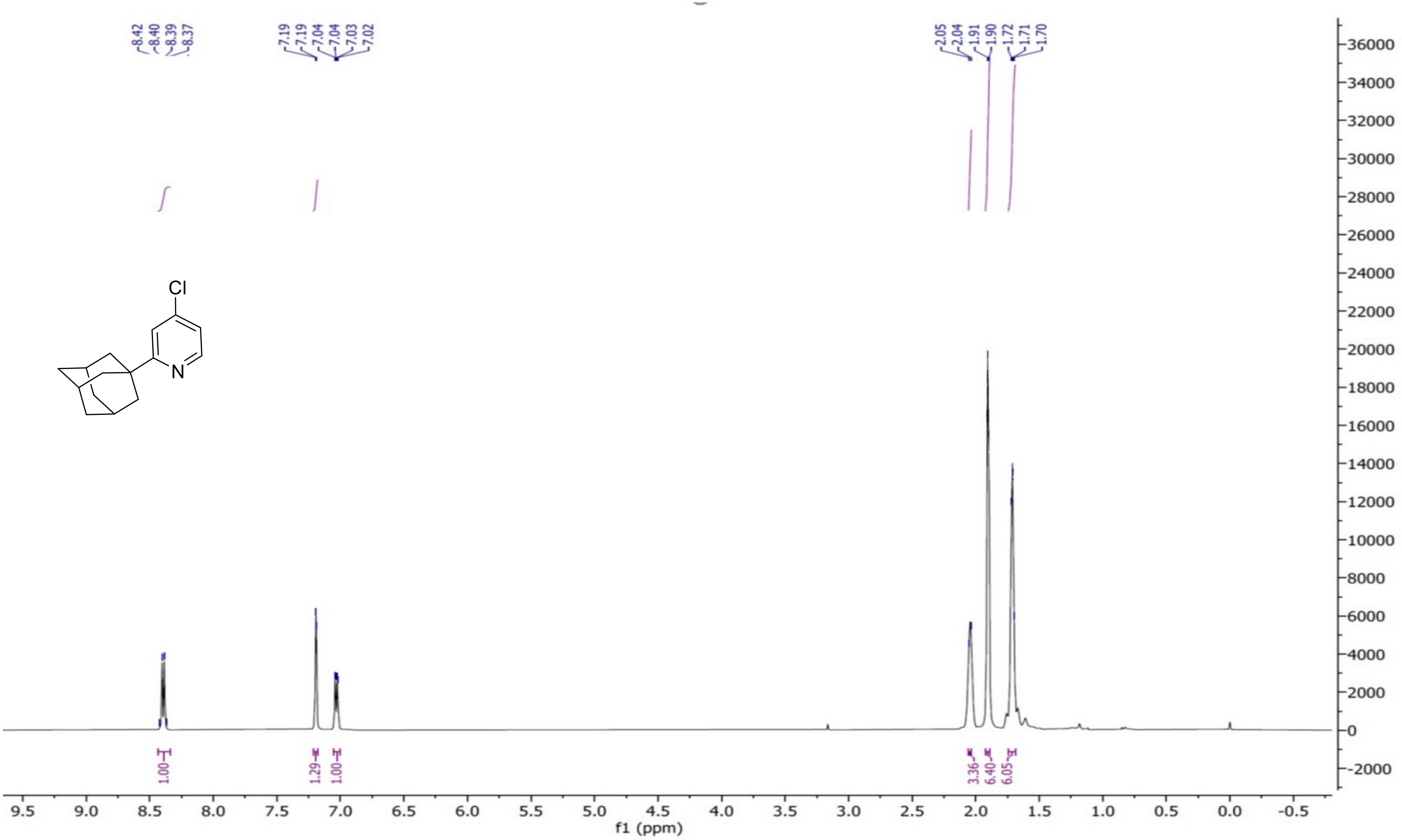

4-Chloro-2-adamantylpyridine (41)

^{13}C-NMR, 75 MHz, $CDCl_3$

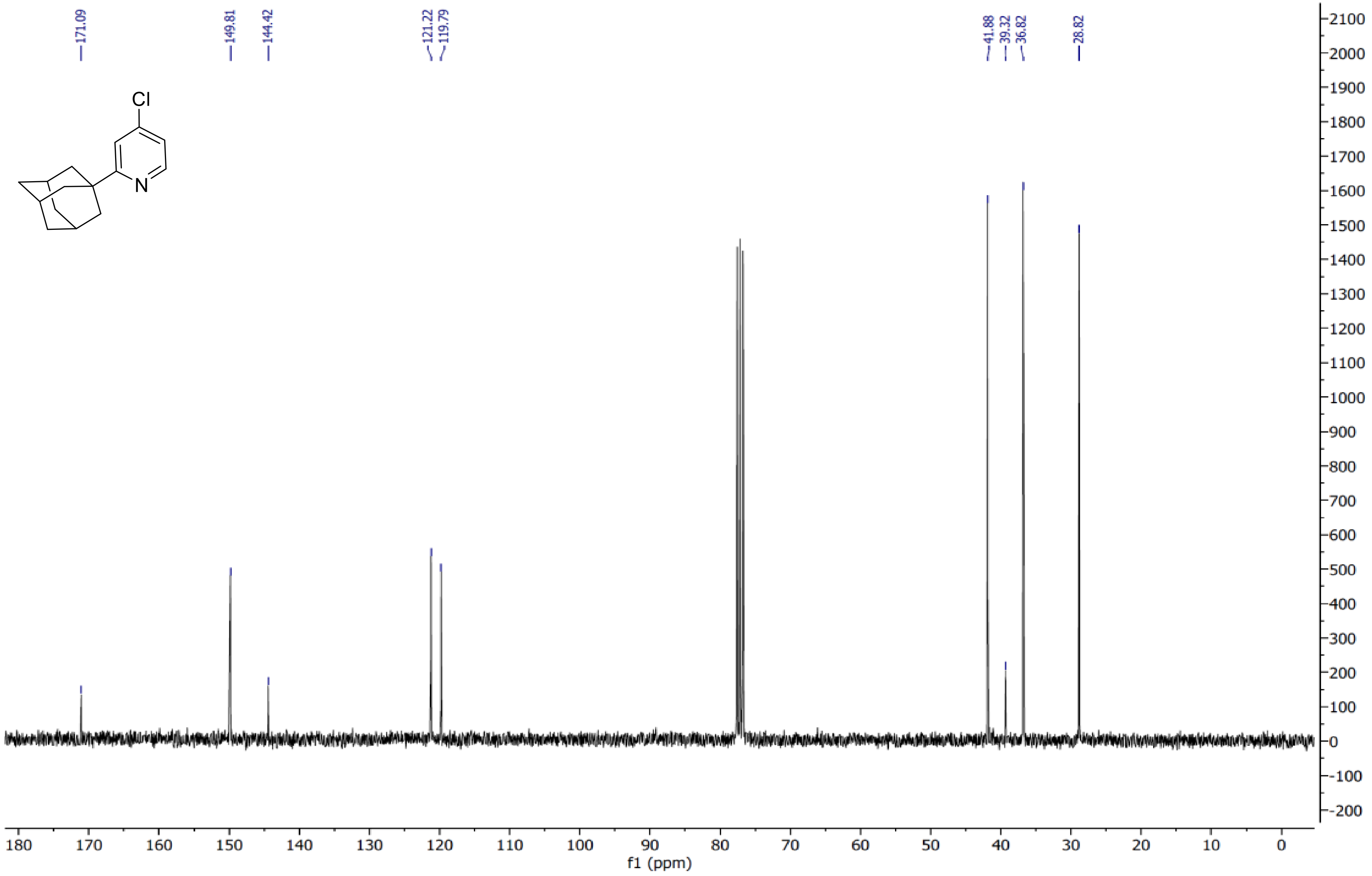

4-Bromo-2-isopropylpyridine (42)

^{1}H-NMR, 300 MHz, $CDCl_3$

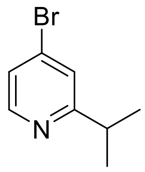

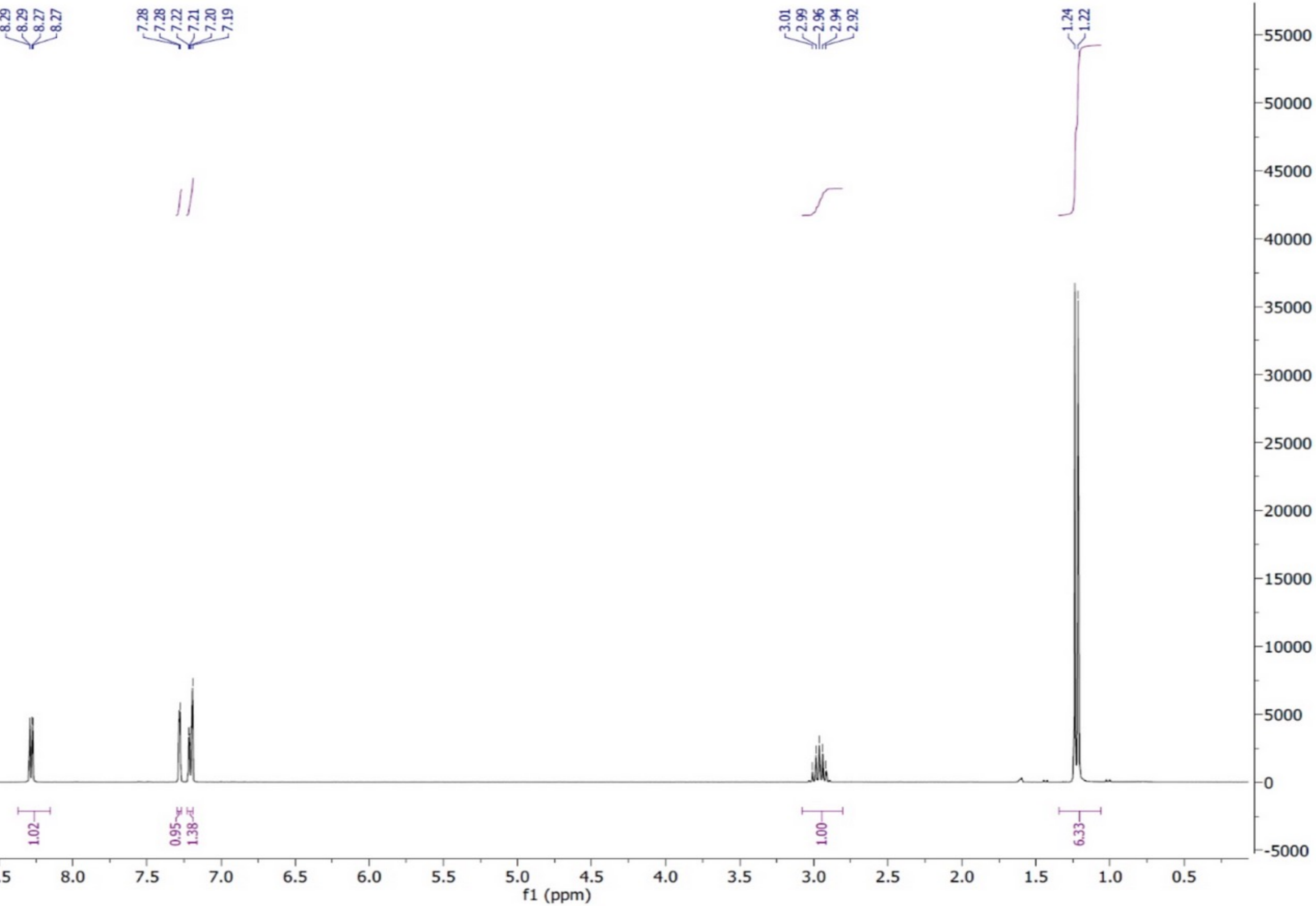

4-Bromo-2-isopropylpyridine (42)

^{13}C-NMR, 75 MHz, $CDCl_3$

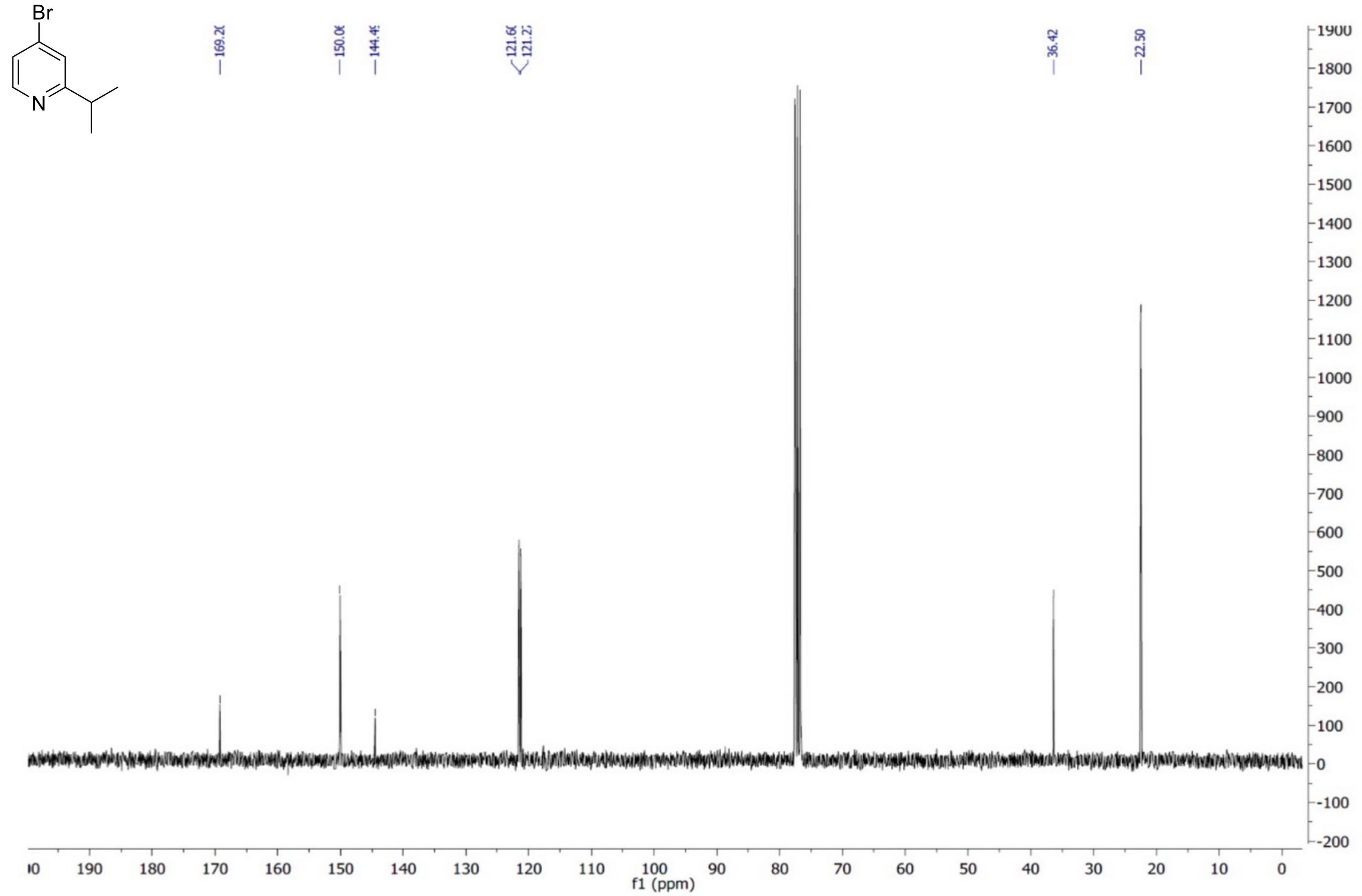

4-Bromo-2-adamantylpyridine (43)

^{1}H-NMR, 300 MHz, $CDCl_3$

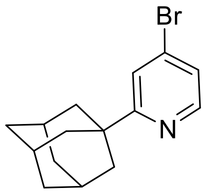

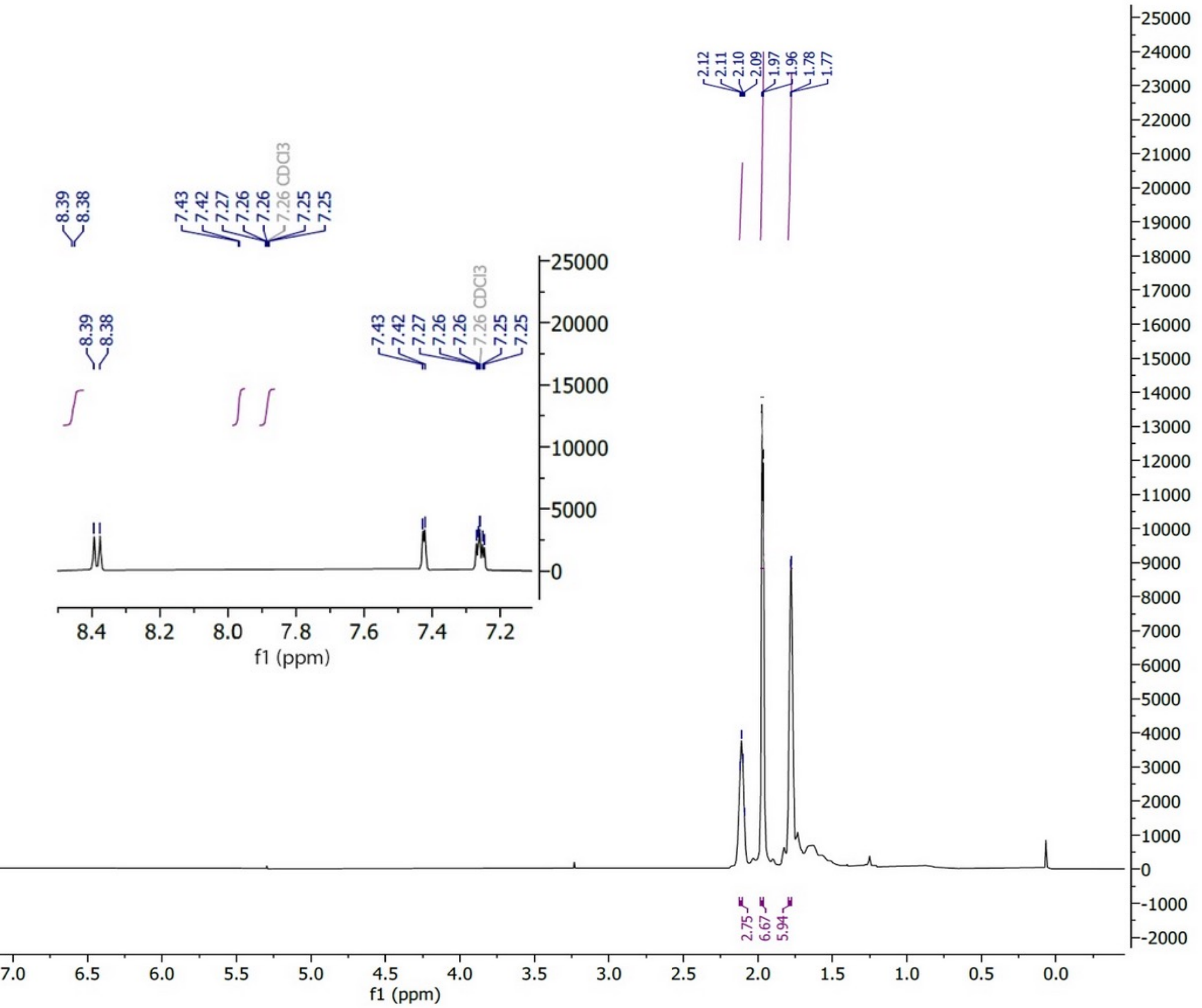

4-Bromo-2-adamantylpyridine (43)

^{13}C-NMR, 75 MHz, $CDCl_3$

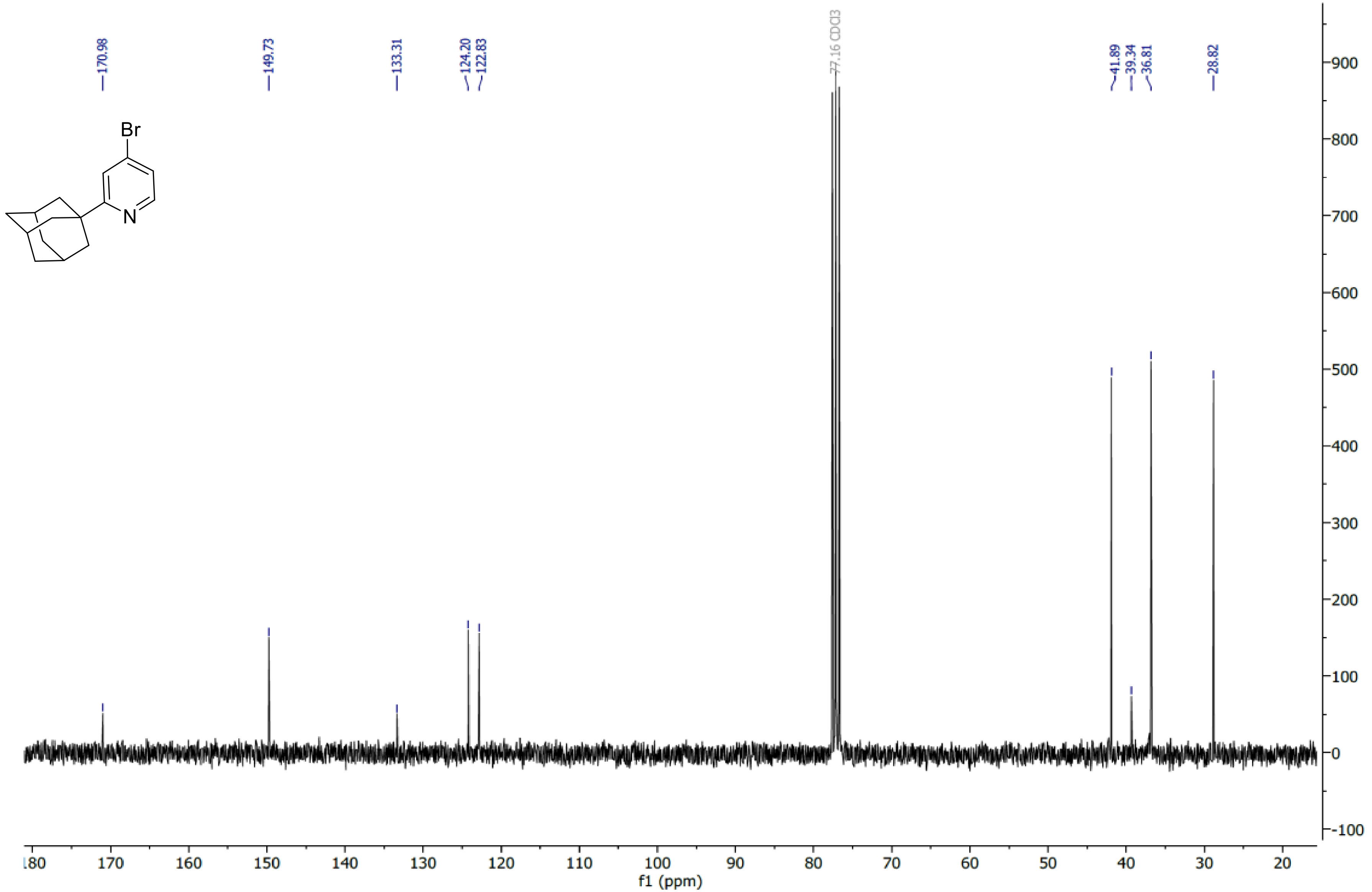

tert-Butyl 6-(2,4-dichloropyridin-4-yl)piperidine-1-carboxylate (44)

^{1}H-NMR, 100 MHz, $CDCl_3$

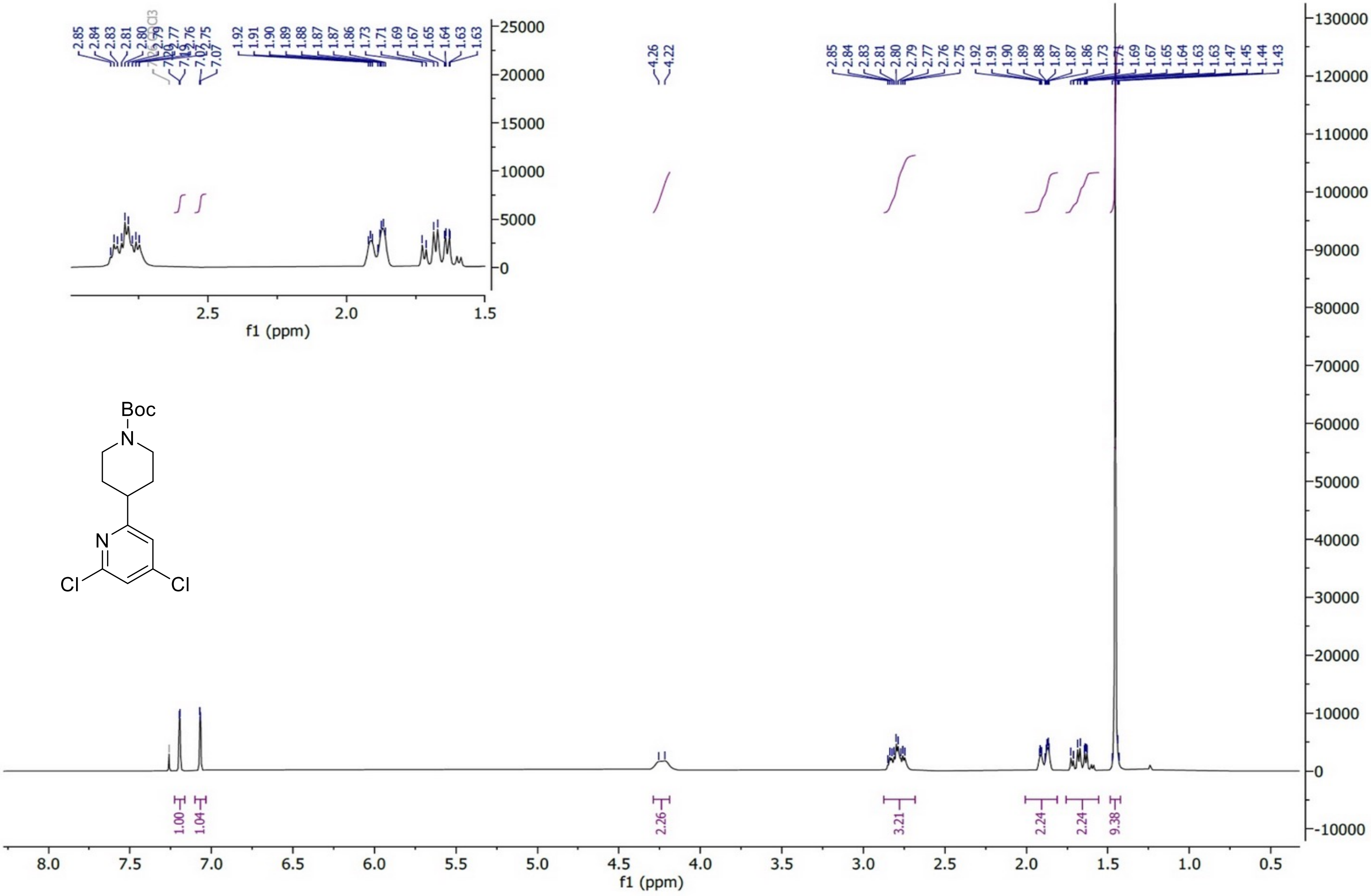

tert-Butyl 6-(2,4-dichloropyridin-4-yl)piperidine-1-carboxylate (44)

^{13}C-NMR, 75 MHz, $CDCl_3$

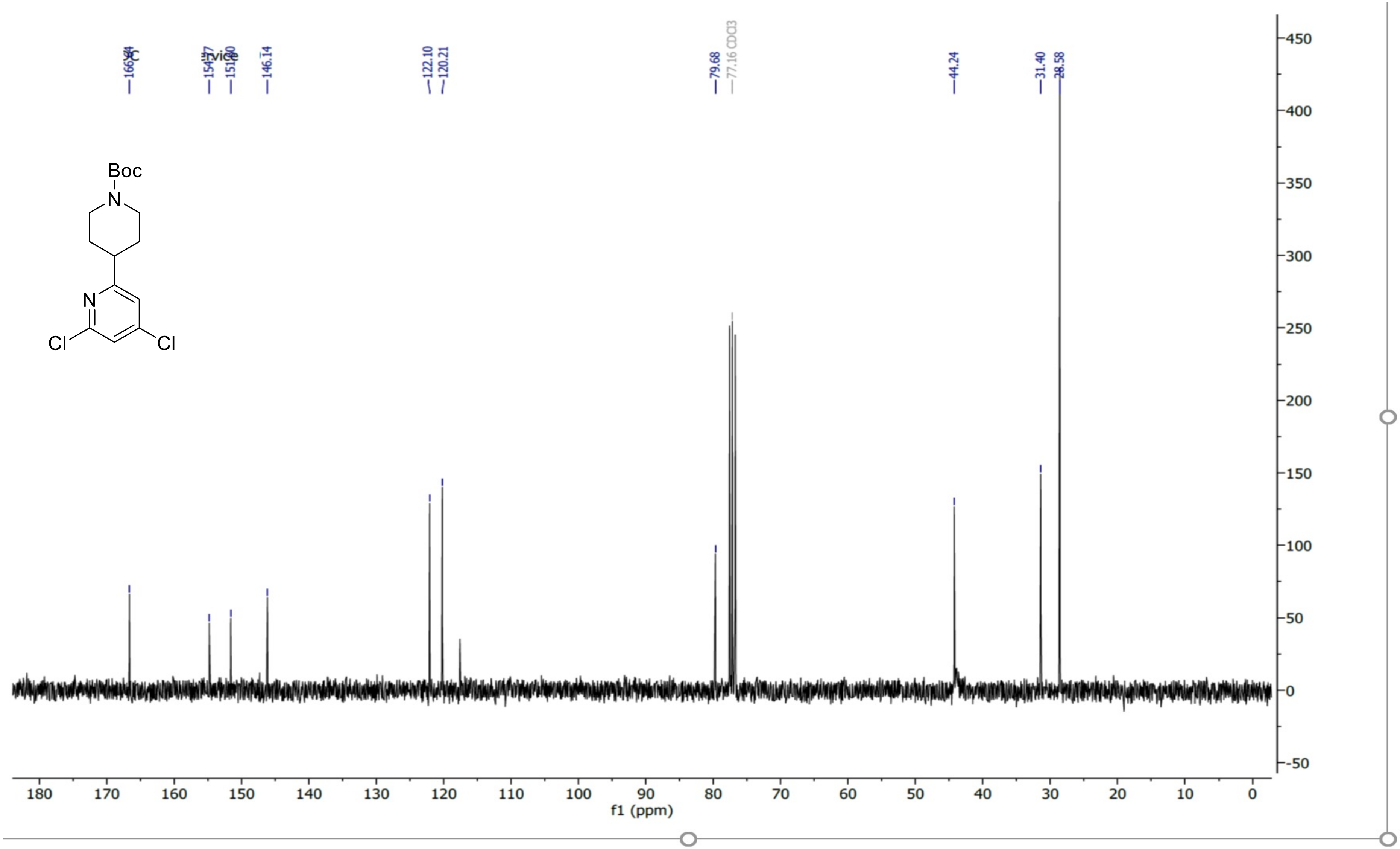

2,6-Dichloro-4-adamantylpyrindine (45)

^{1}H-NMR, 300 MHz, $CDCl_3$

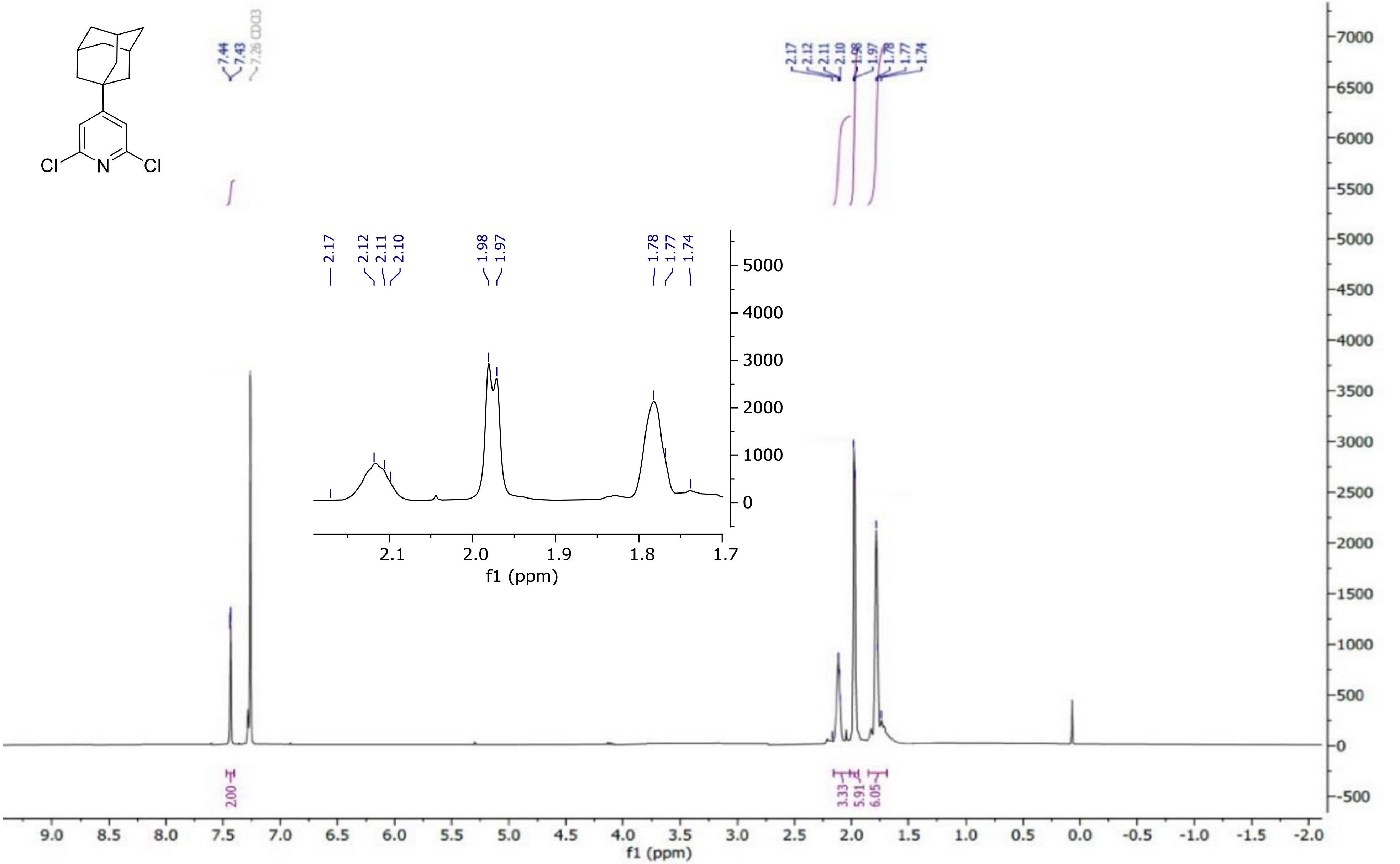

2,6-Dichloro-4-adamantylpyrindine (45)

^{13}C-NMR, 75 MHz, $CDCl_3$

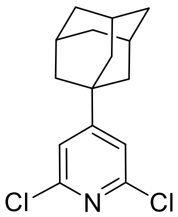

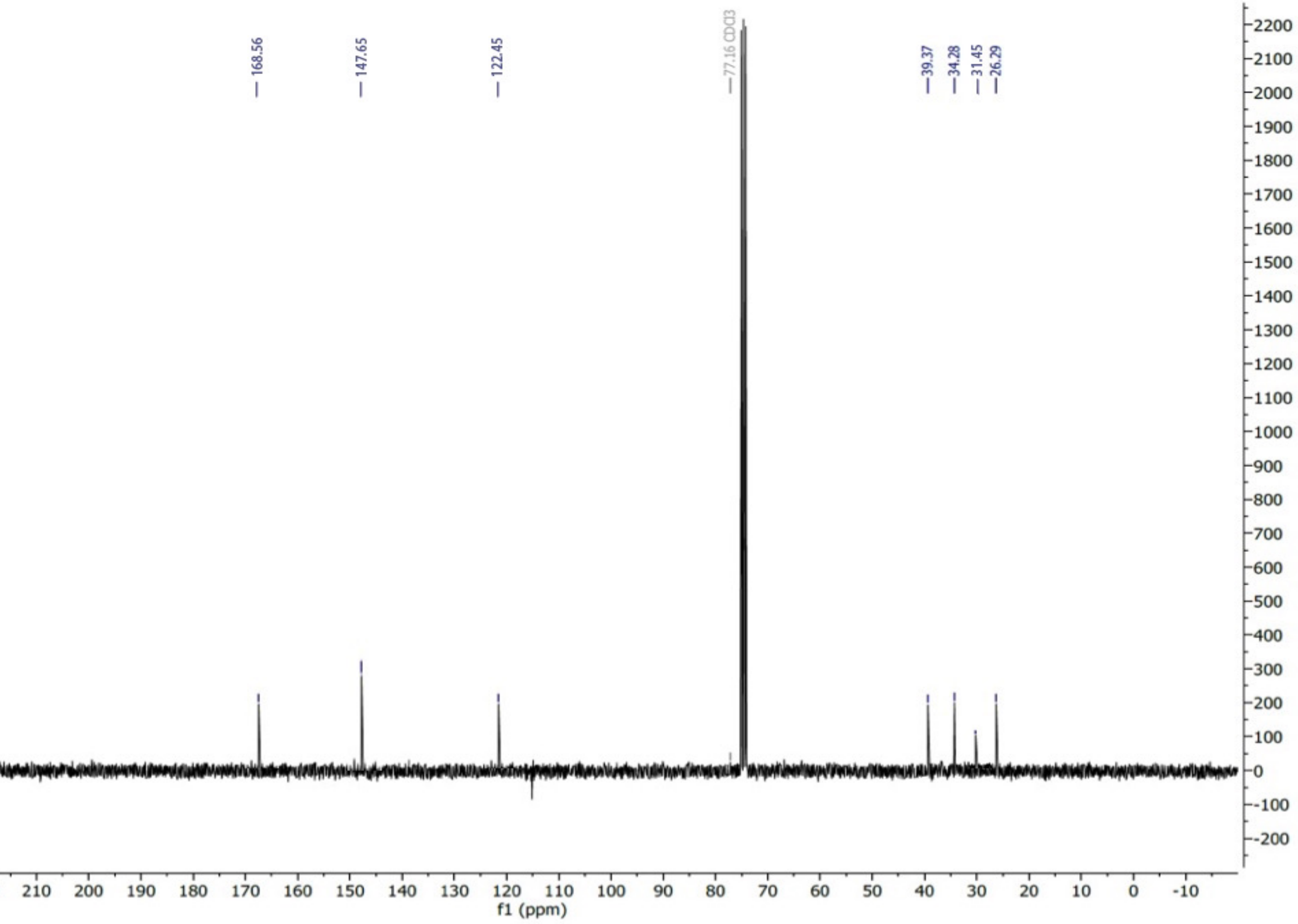

2,6-Dichloro-4-(4-phenylcyclohexyl)pyridine (46)

^{1}H-NMR, 300 MHz, $CDCl_3$

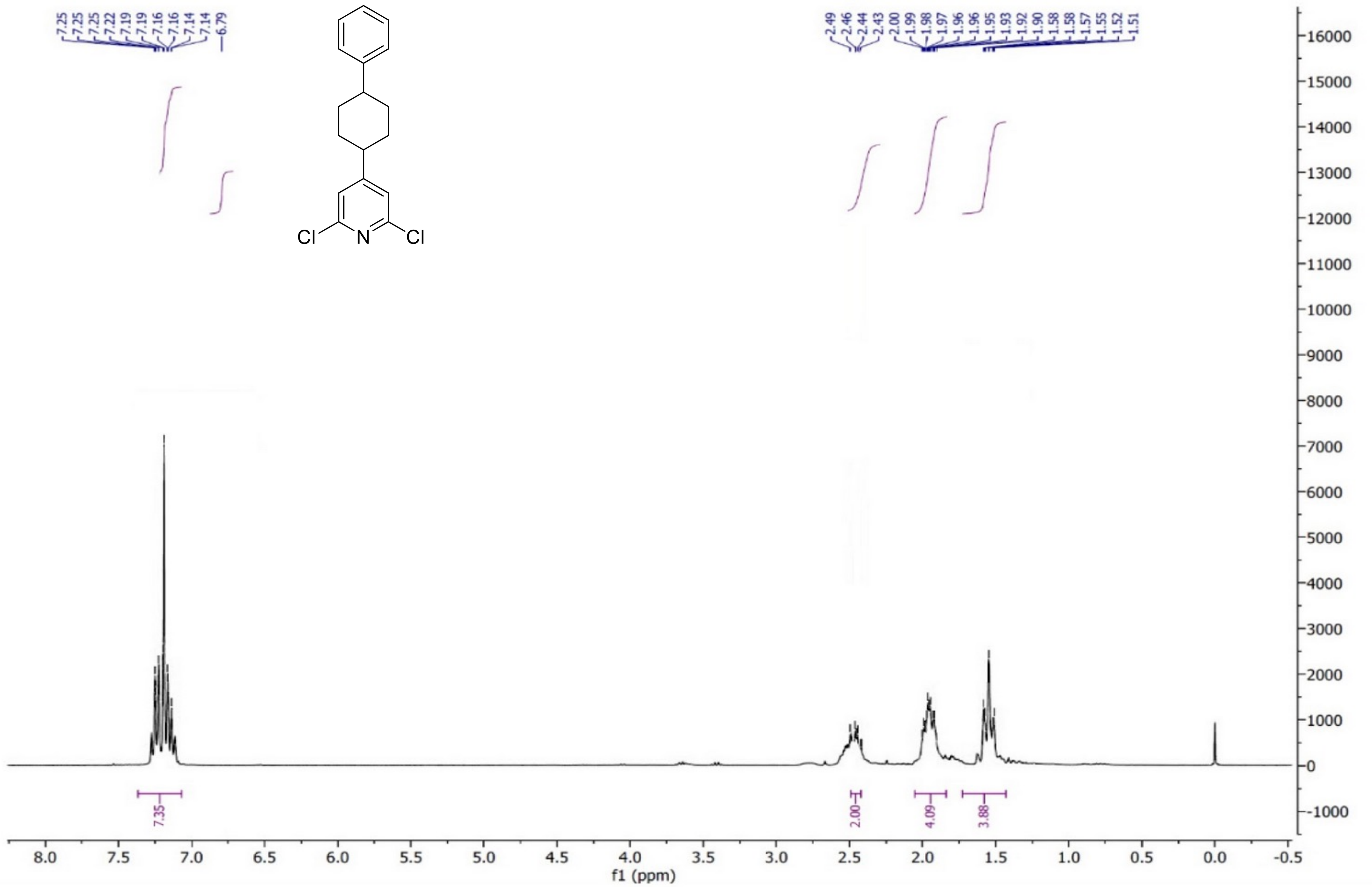

2,6-Dichloro-4-(4-phenylcyclohexyl)pyridine (46)

^{13}C-NMR, 75 MHz, $CDCl_3$

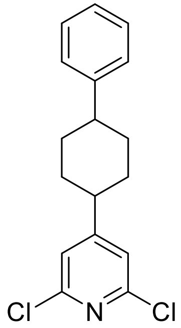

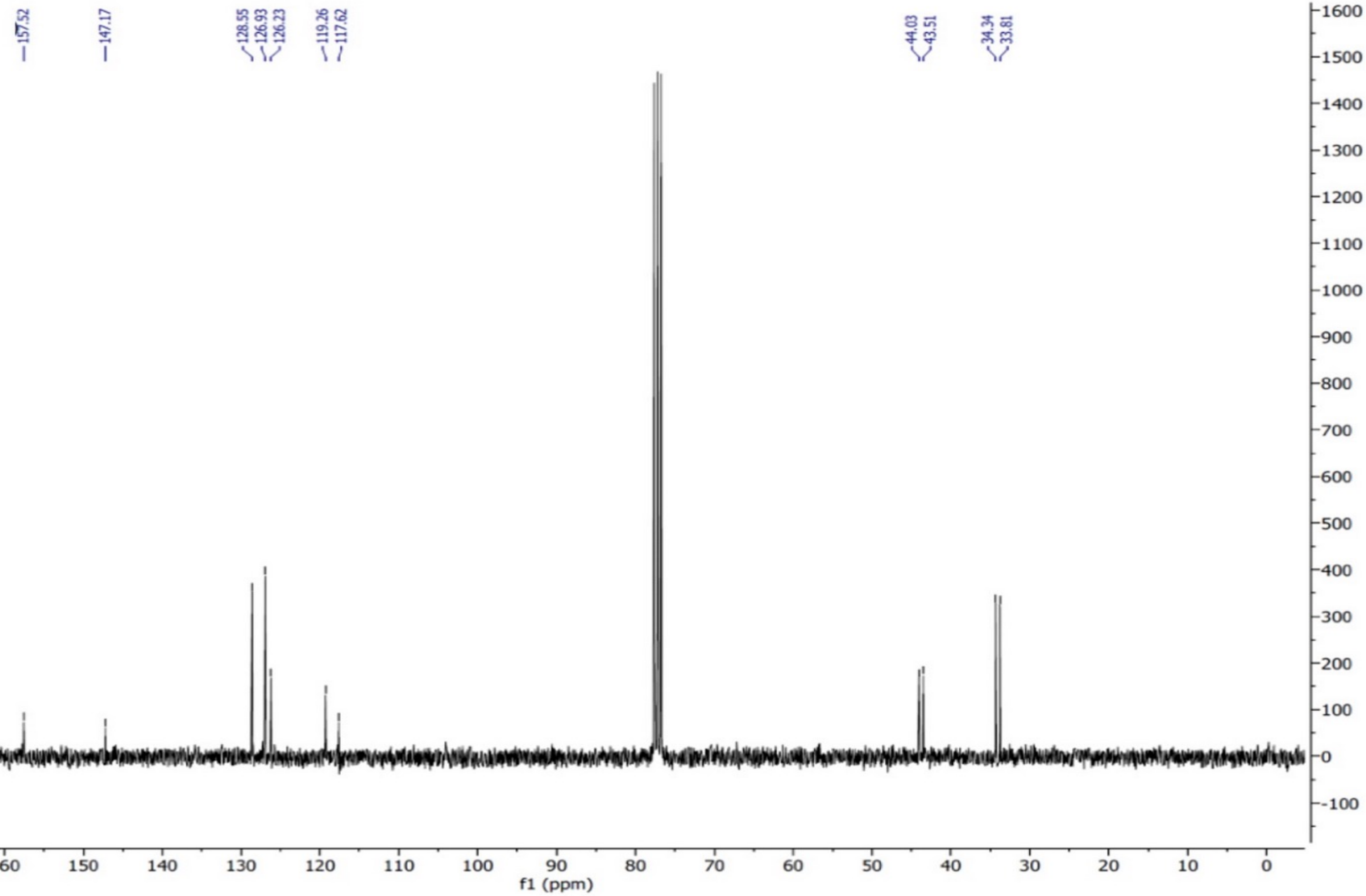

tert-Butyl 4-(2,6-dichloropyridin-4-yl)piperidine-1-carboxylate (47)

^{1}H-NMR, 300 MHz, $CDCl_3$

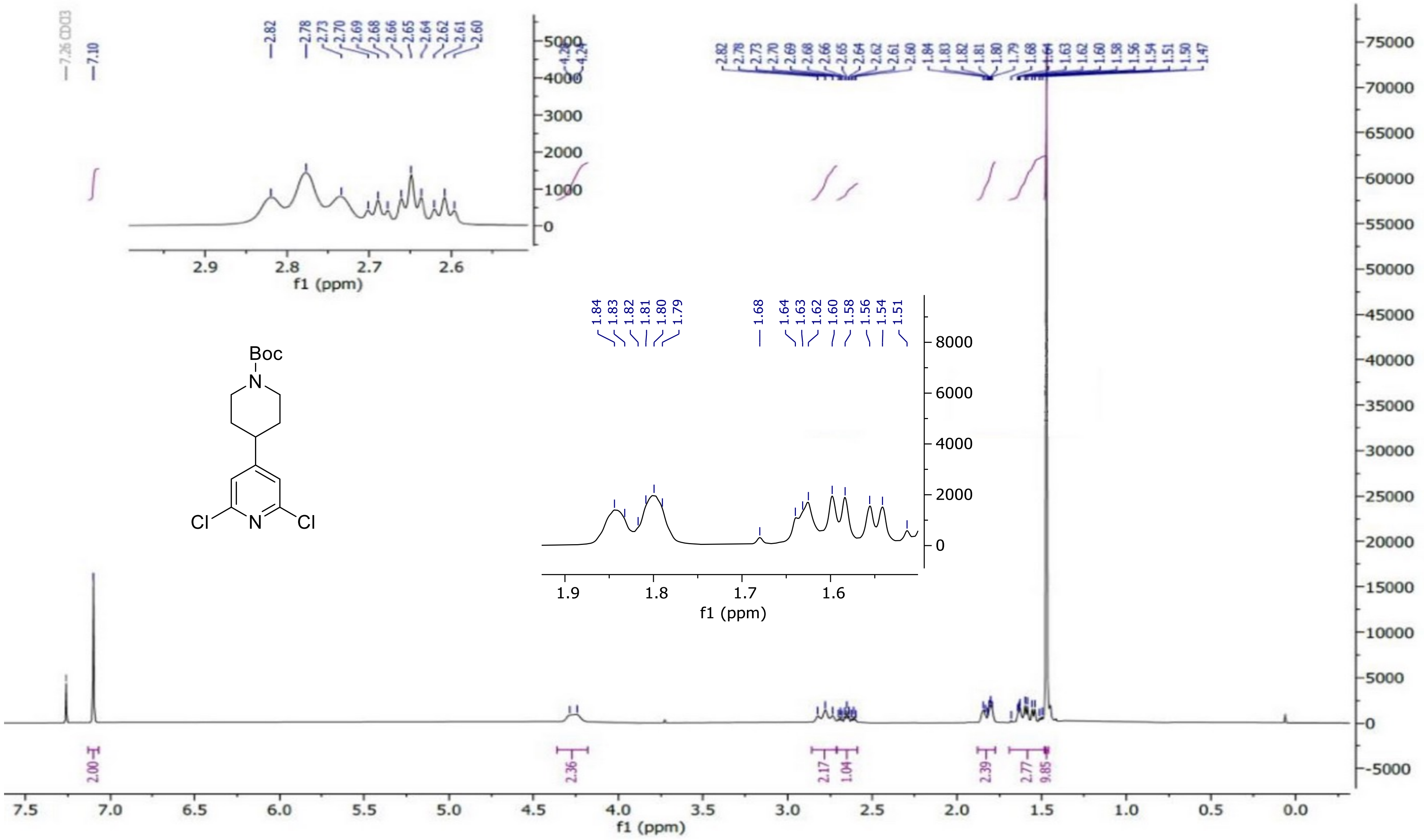

tert-Butyl 4-(2,6-dichloropyridin-4-yl)piperidine-1-carboxylate (47)

^{13}C-NMR, 75 MHz, $CDCl_3$

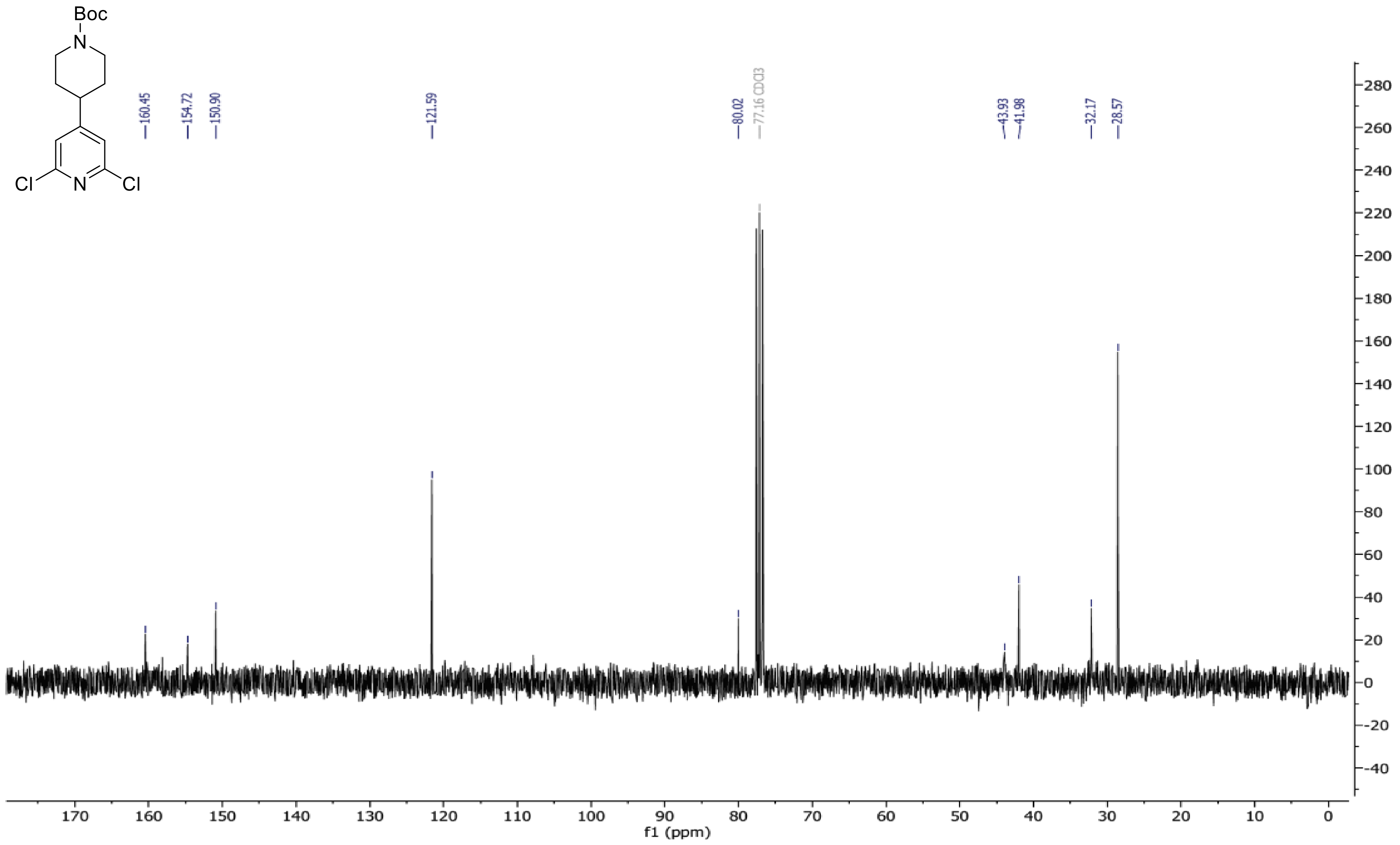

Ethyl-4-((ethoxycarbonothioyl)thio)undecanoate (48a)

^{1}H-NMR, 300 MHz, $CDCl_3$

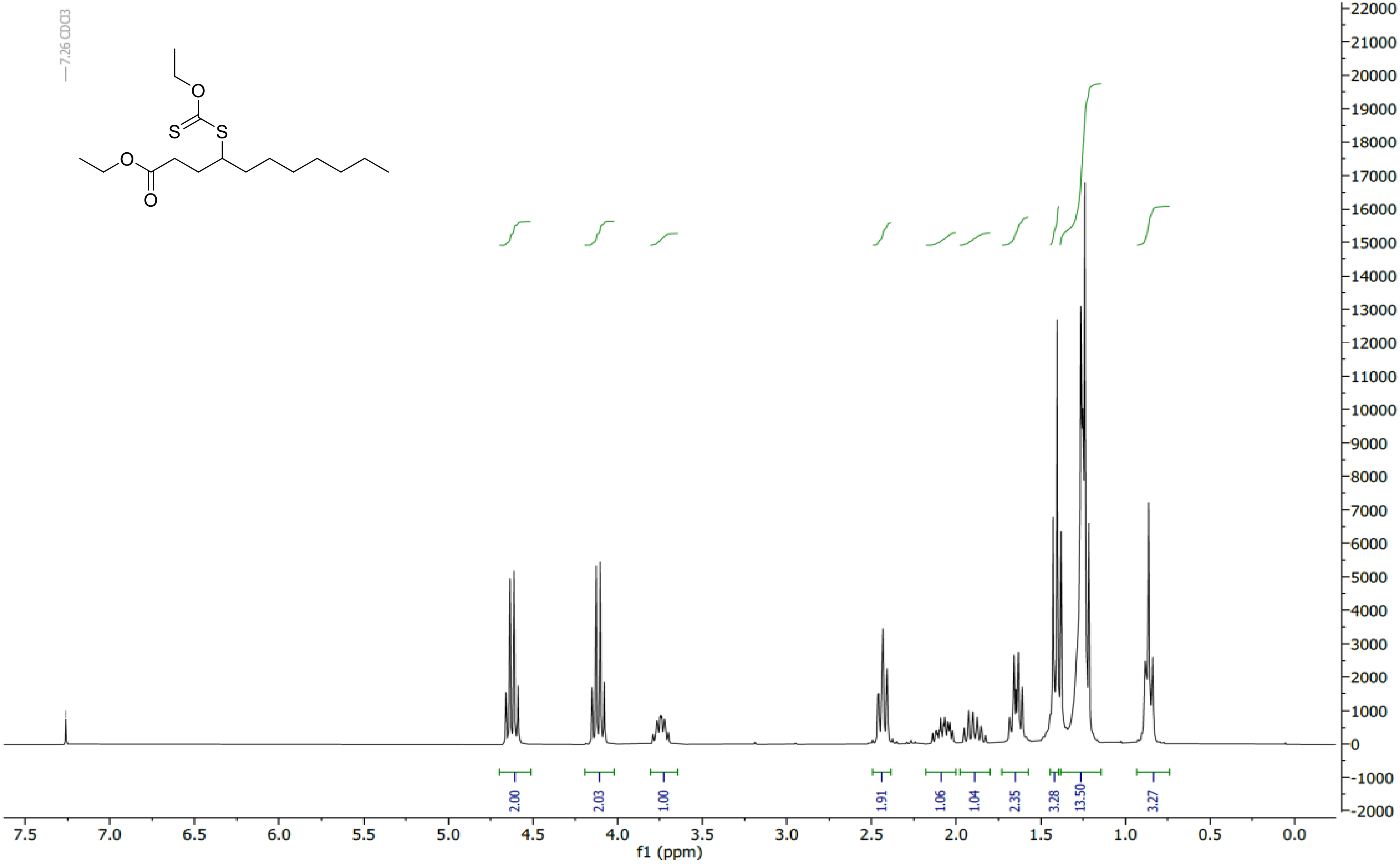

Ethyl-4-((ethoxycarbonothioyl)thio)undecanoate (48a)

^{13}C-NMR, 75 MHz, $CDCl_3$

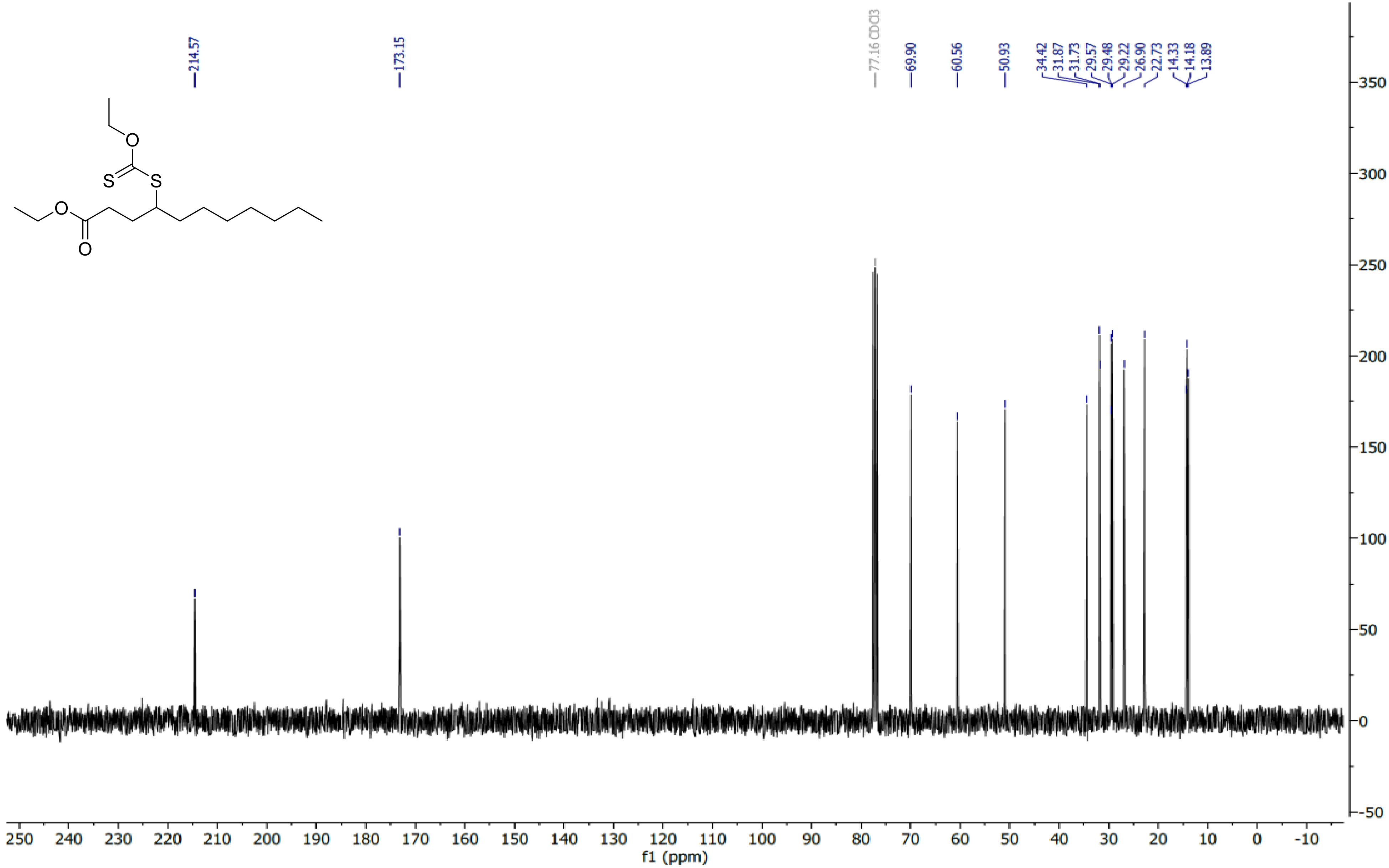

Ethyl 4-butoxy-4-((ethoxycarbonothioyl)thio)butanoate (48d)

^{1}H-NMR, 300 MHz, $CDCl_3$

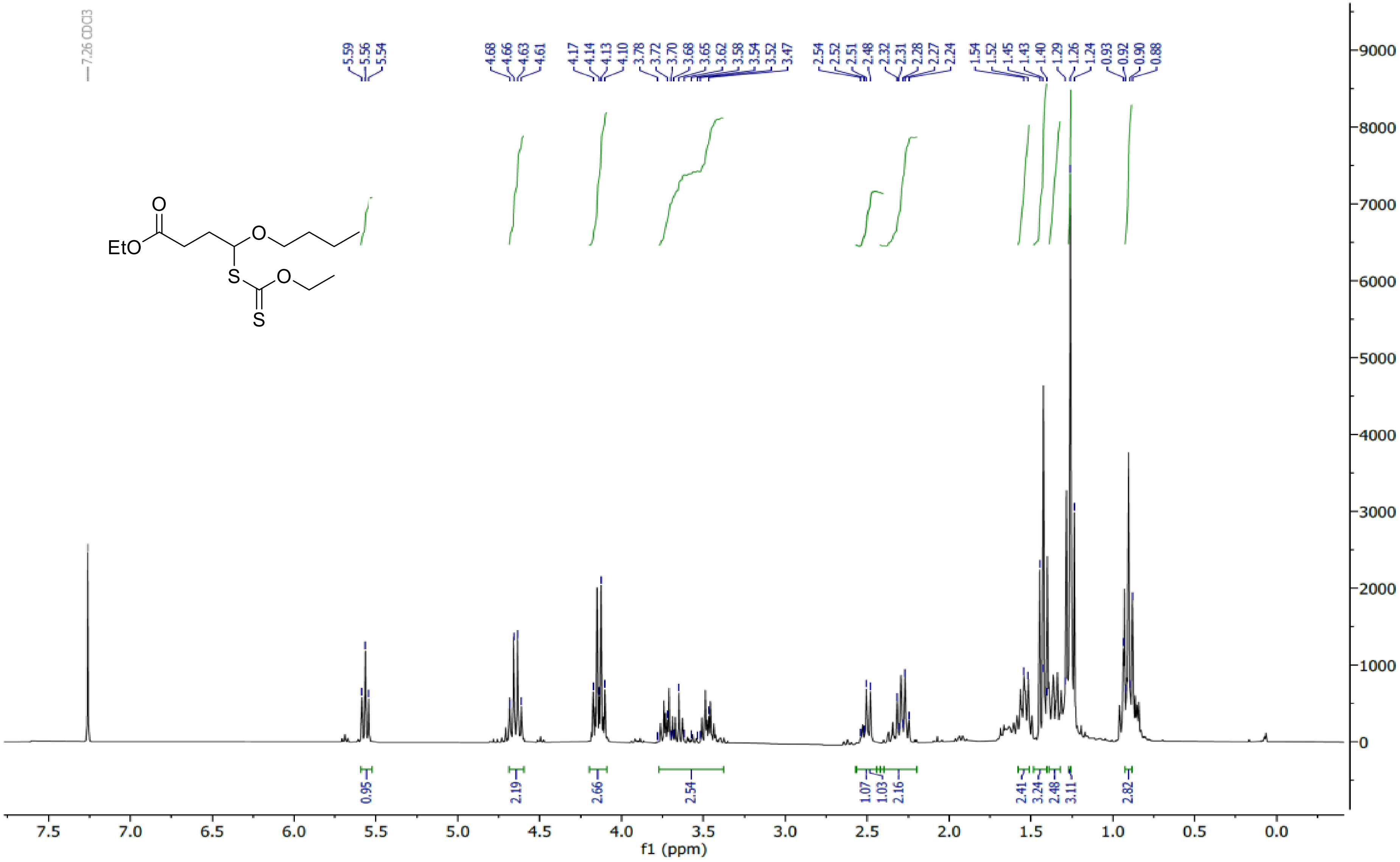

Ethyl 4-butoxy-4-((ethoxycarbonothioyl)thio)butanoate (48d)

^{13}C-NMR, 75 MHz, $CDCl_3$

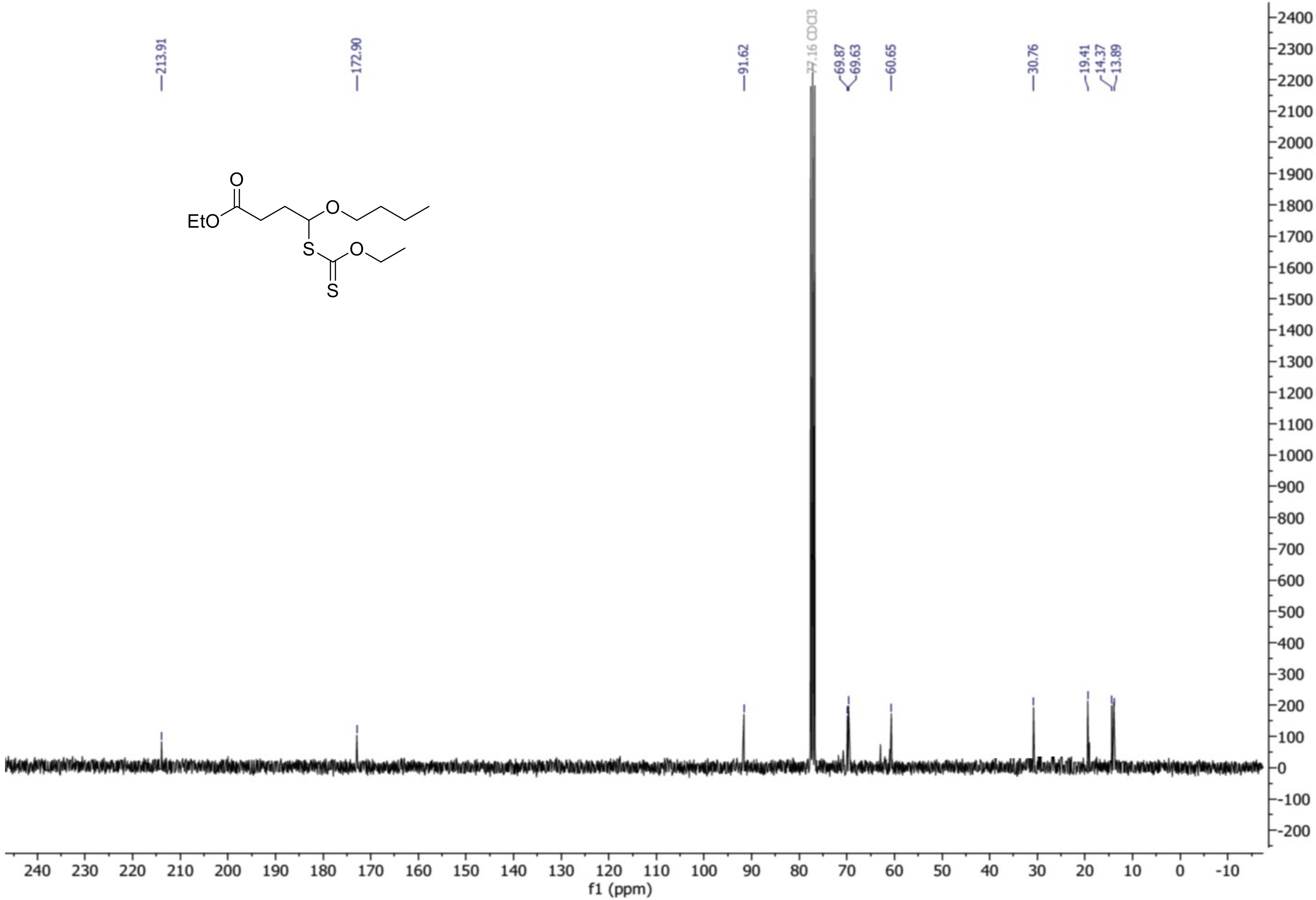

Ethyl 4-(2,6-dimethylpyridin-4-yl)undecanoate (49)

^{1}H-NMR, 300 MHz, $CDCl_3$

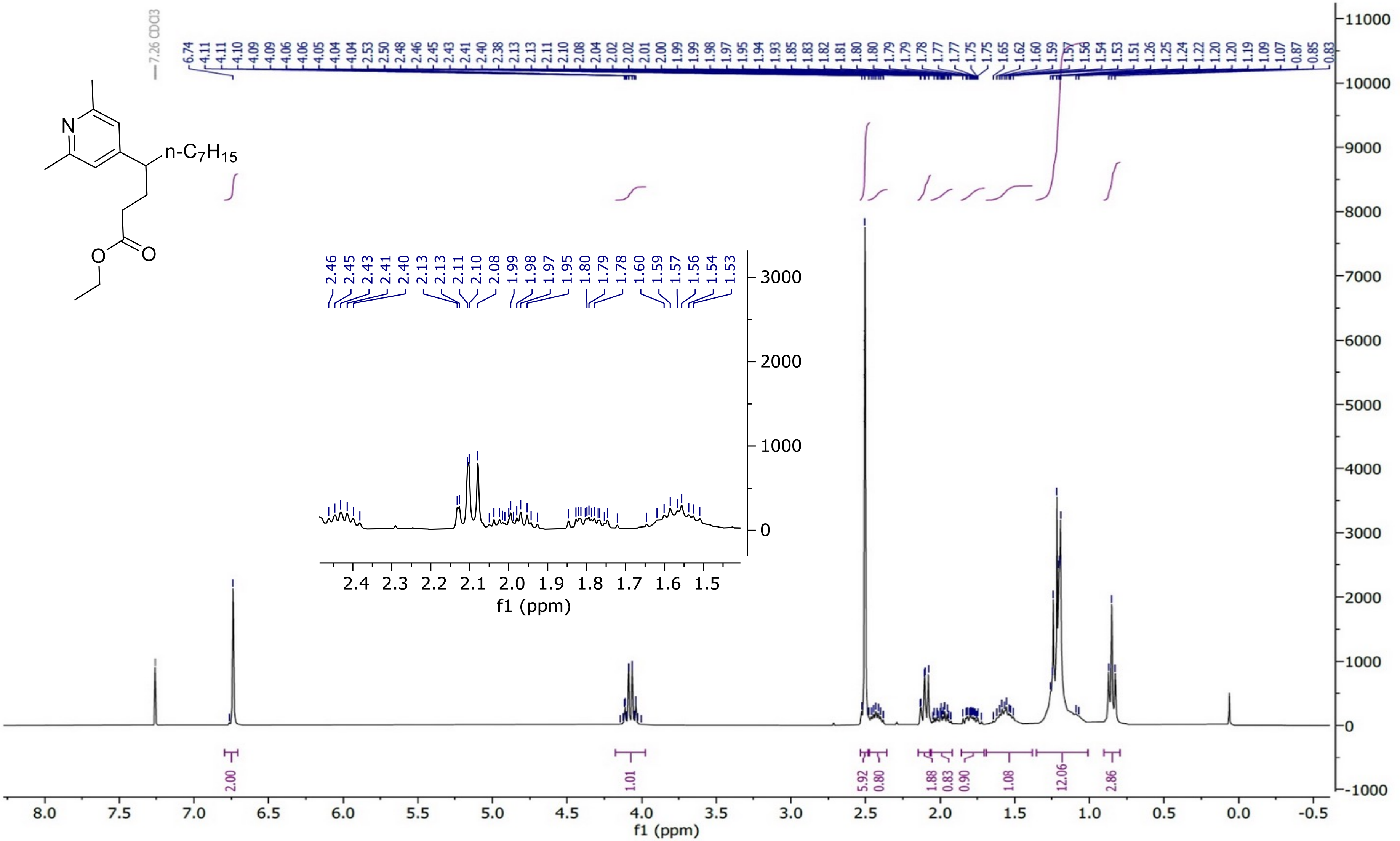

Ethyl 4-(2,6-dimethylpyridin-4-yl)undecanoate (49)

^{13}C-NMR, 75 MHz, $CDCl_3$

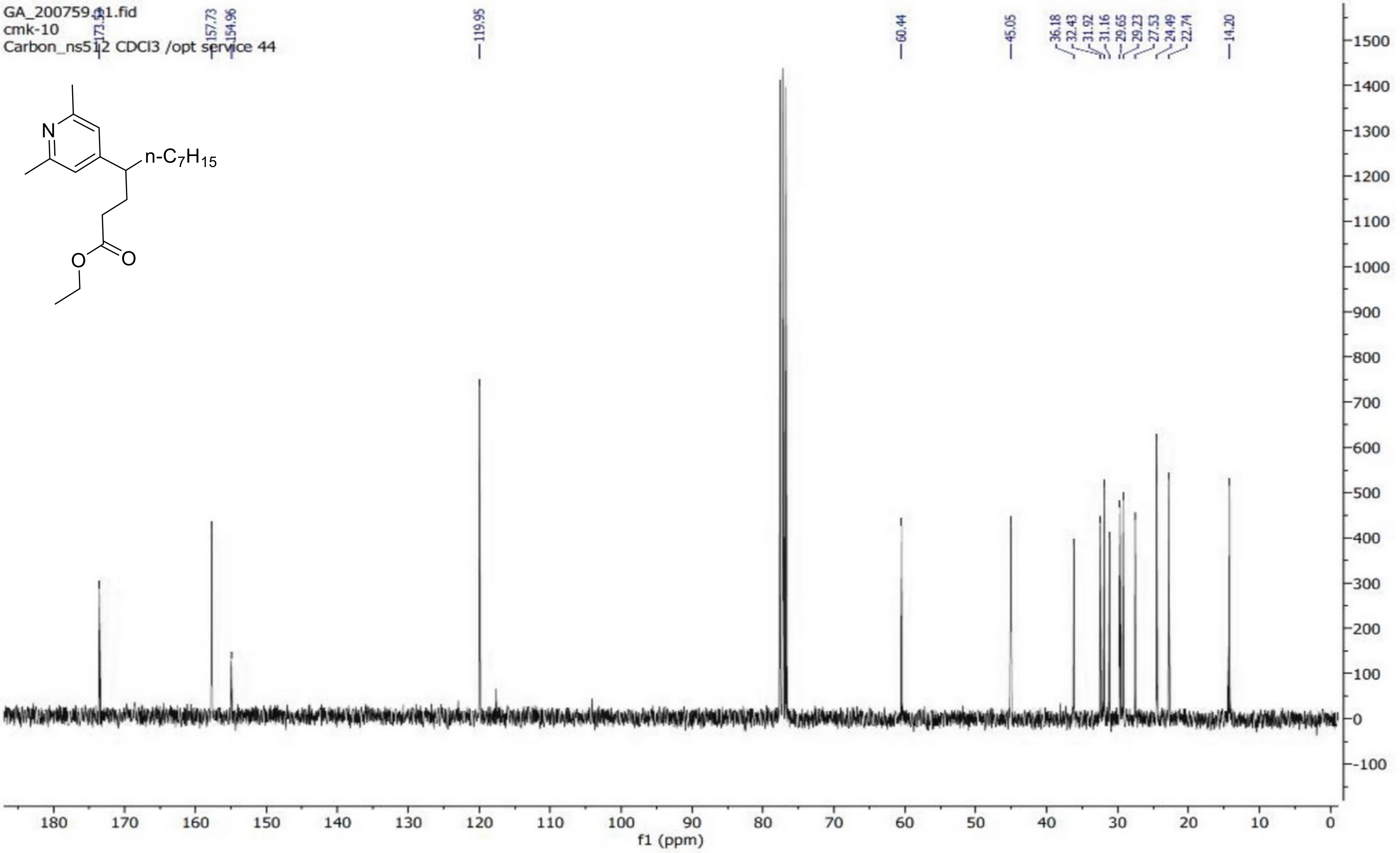

Ethyl 4-(4-chloropyridin-2-yl)-5-(trimethylsilyl)pentanoate (50)

^{1}H-NMR, 300 MHz, $CDCl_3$

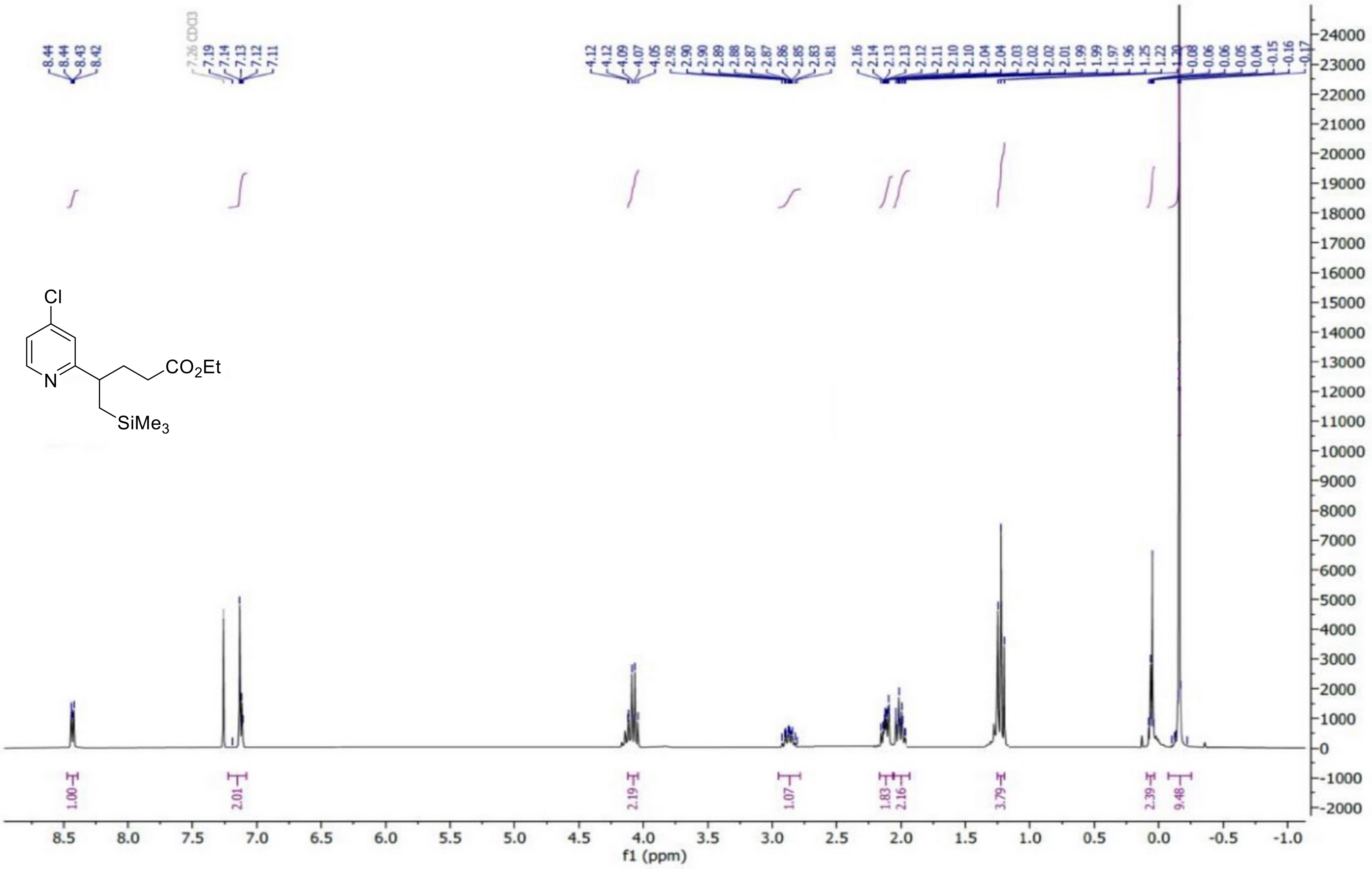

Ethyl 4-(4-chloropyridin-2-yl)-5-(trimethylsilyl)pentanoate (50)

^{13}C-NMR, 75 MHz, $CDCl_3$

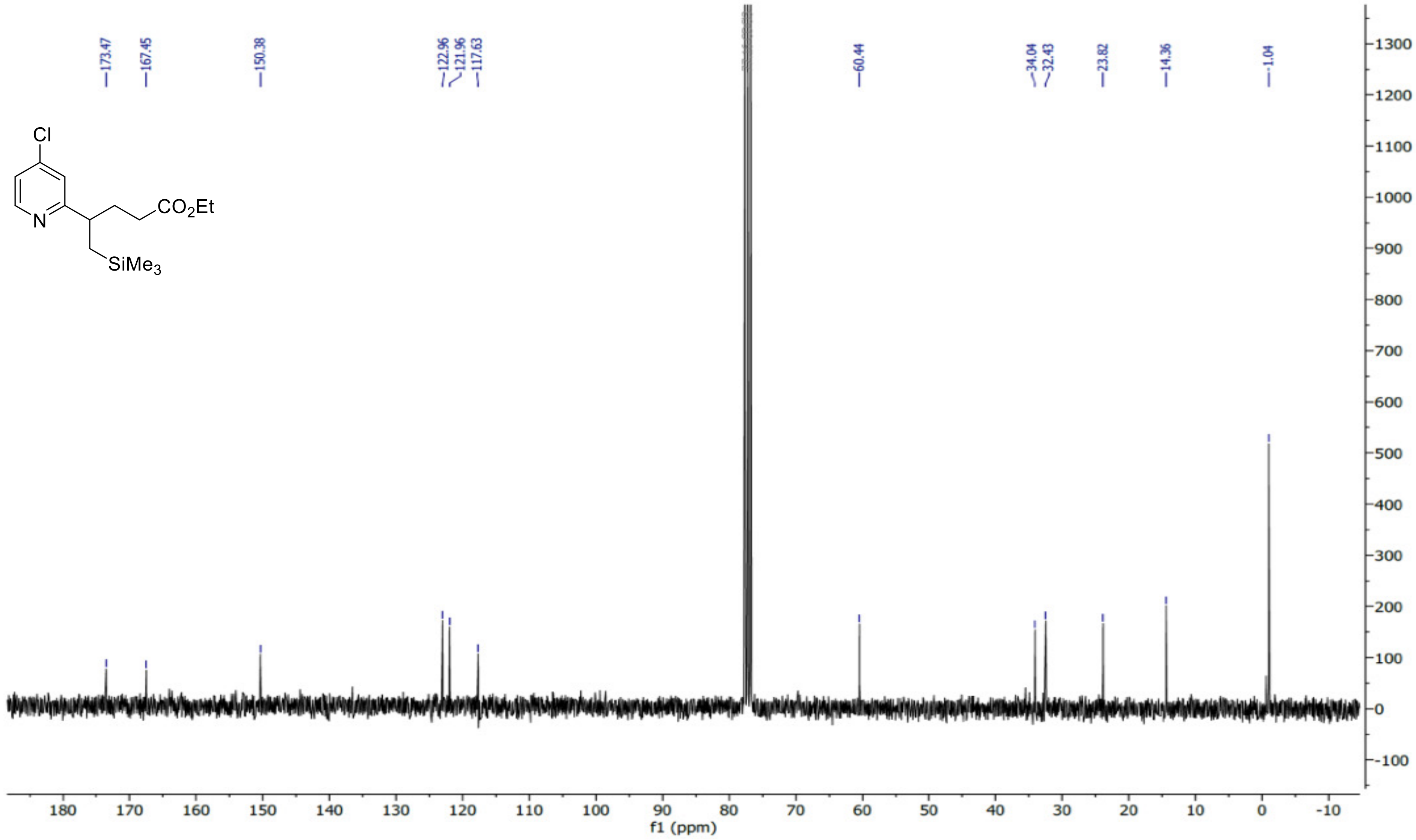

Ethyl 4-acetoxy-4-(2,6-dimethylpyridin-4-yl)butanoate (51)

^{1}H-NMR, 100 MHz, $CDCl_3$

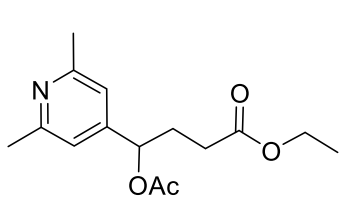

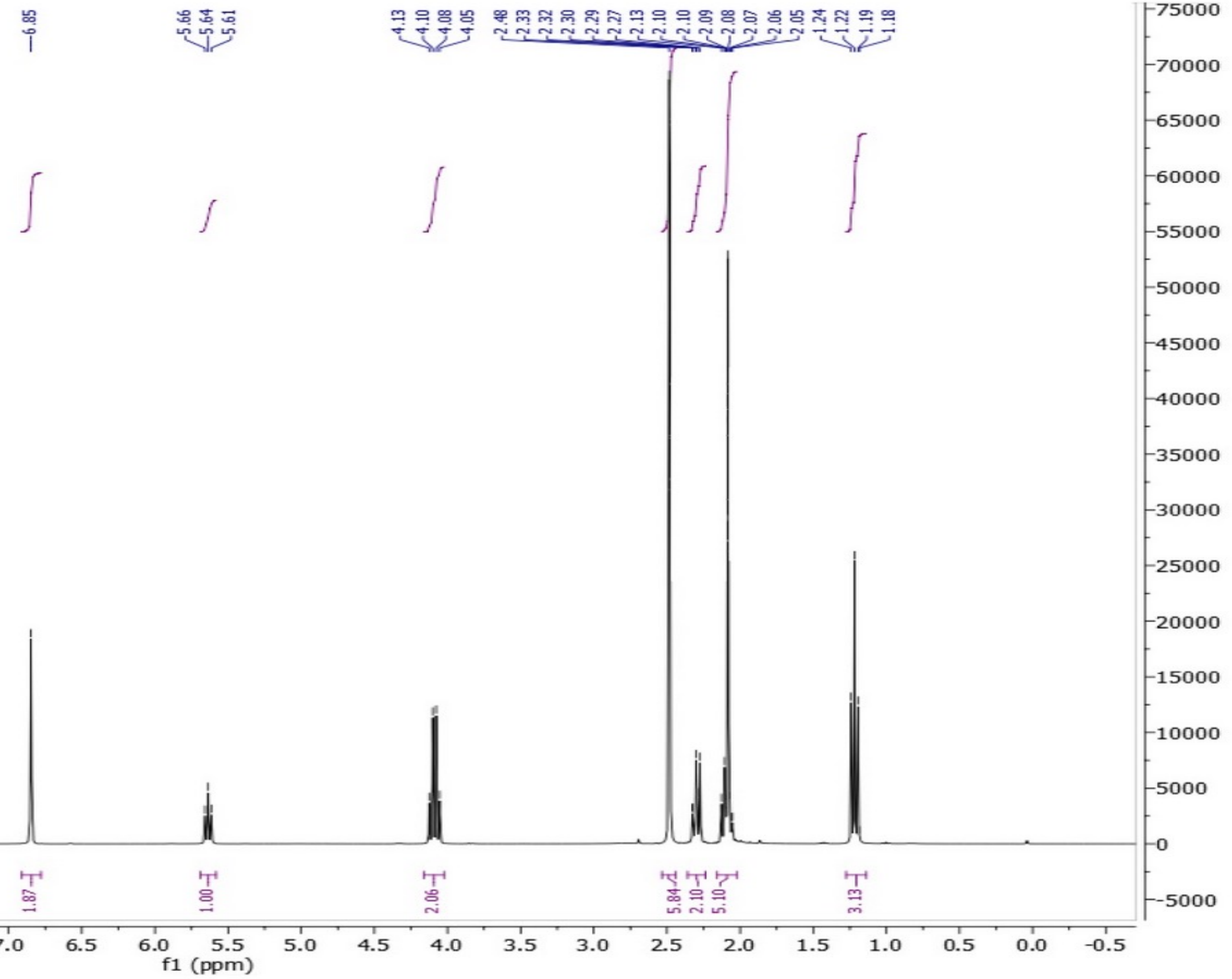

Ethyl 4-acetoxy-4-(2,6-dimethylpyridin-4-yl)butanoate (51)

^{13}C-NMR, 75 MHz, $CDCl_3$

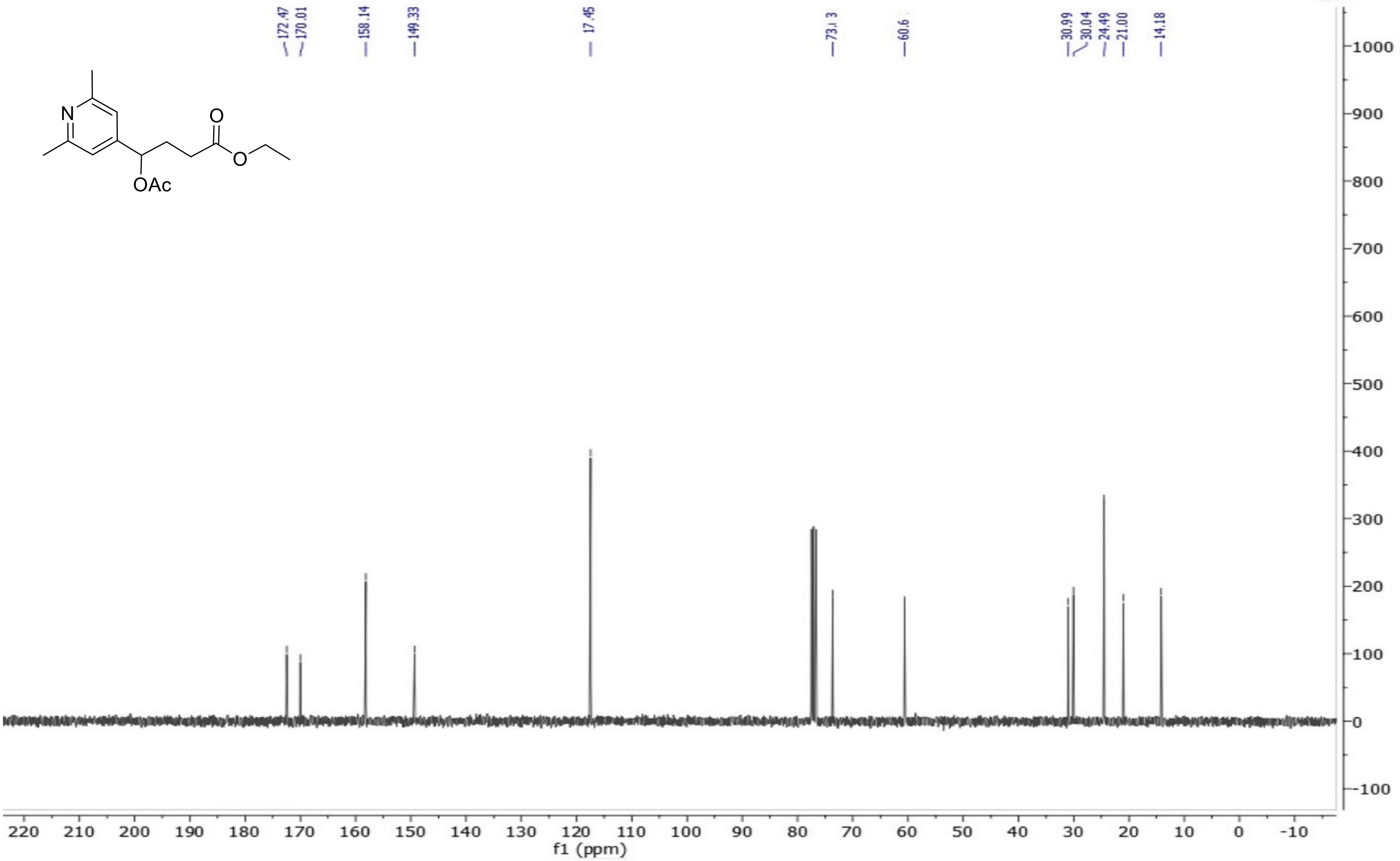

Ethyl 4-acetoxy-4-(4-chloropyridin-2-yl)butanoate (52)

^{1}H-NMR, 300 MHz, $CDCl_3$

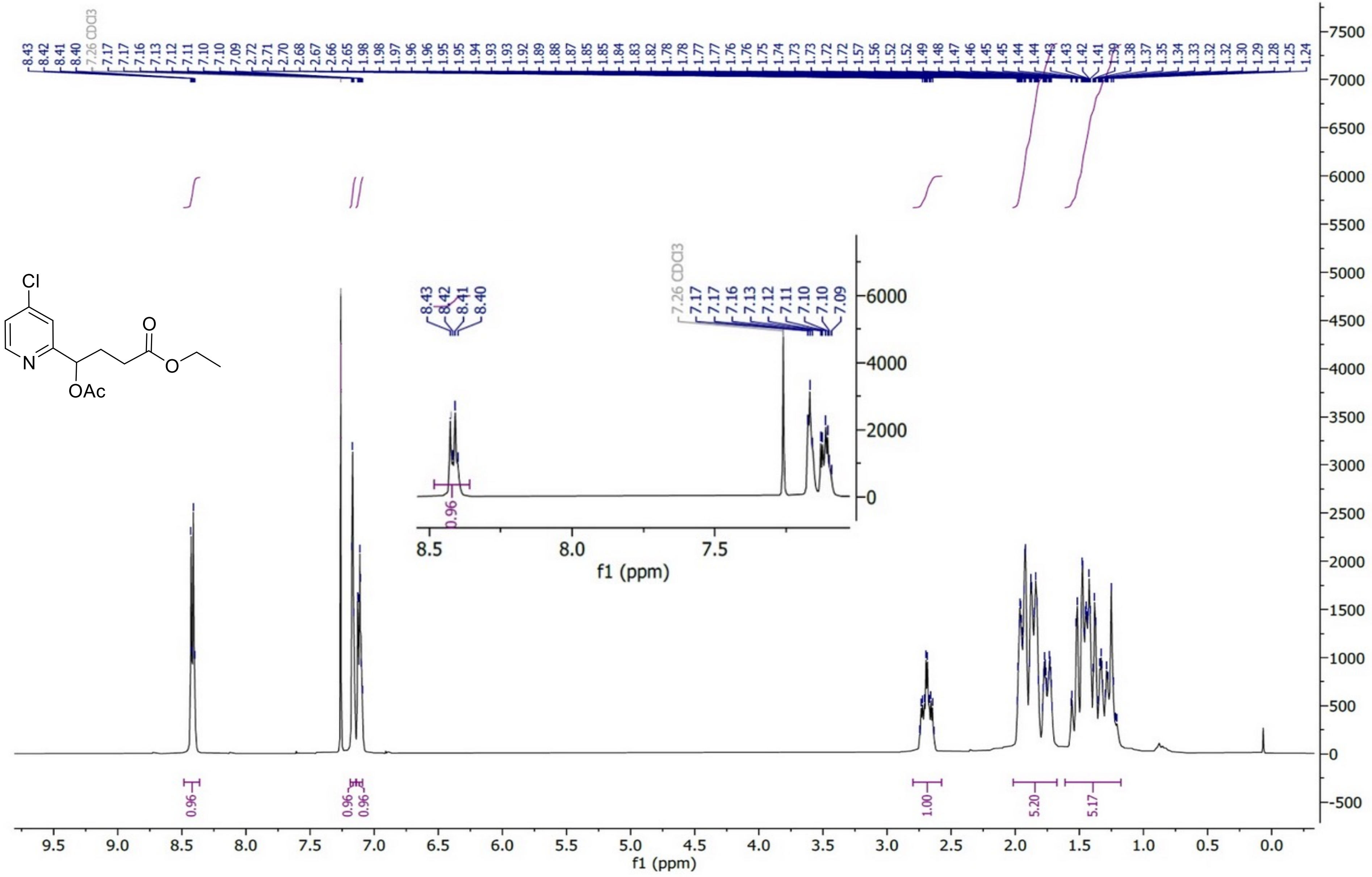

Ethyl 4-acetoxy-4-(4-chloropyridin-2-yl)butanoate (52)

^{13}C-NMR, 75 MHz, $CDCl_3$

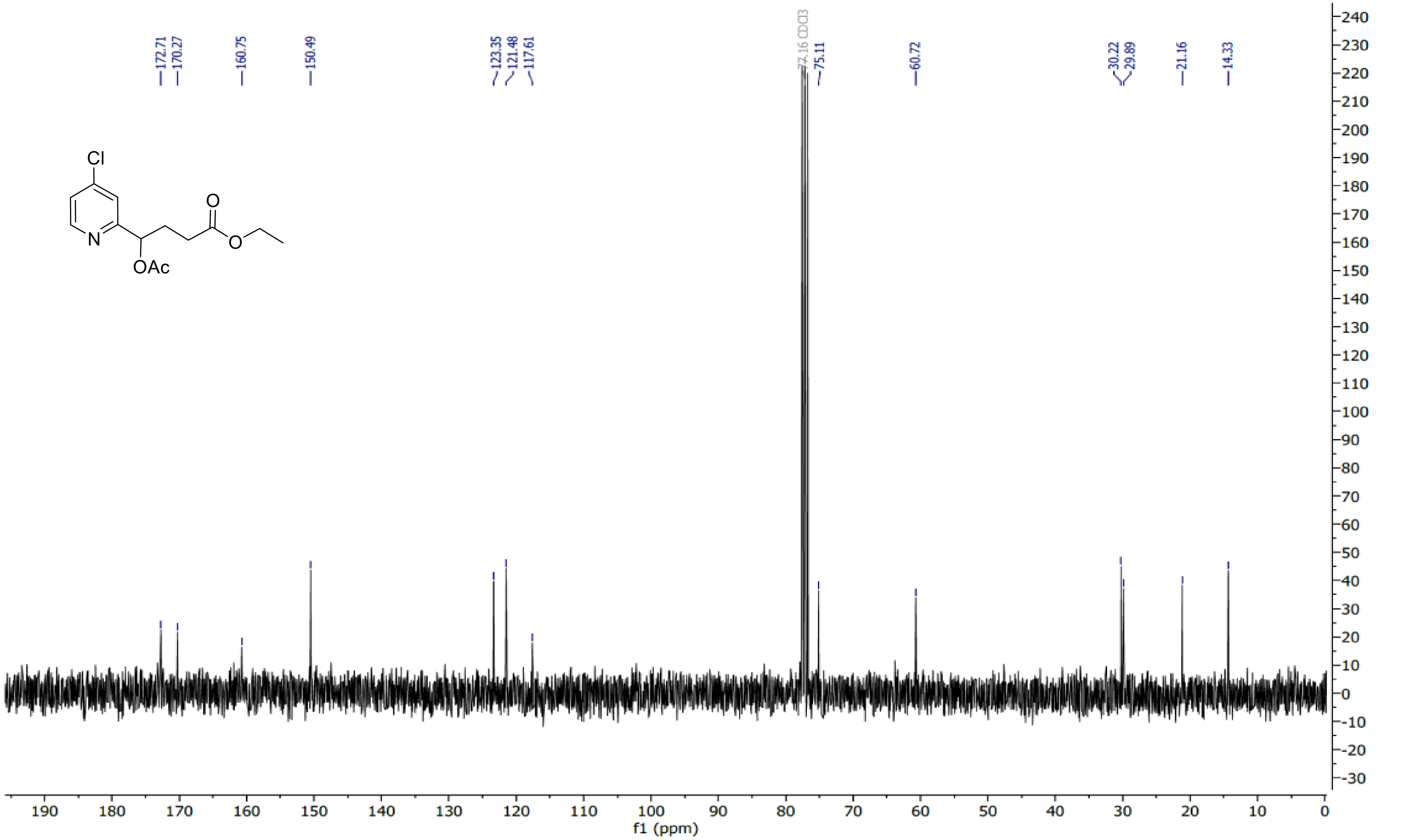

Ethyl 4-acetoxy-4-(4-bromopyridin-2-yl)butanoate (53)

^{1}H-NMR, 300 MHz, $CDCl_3$

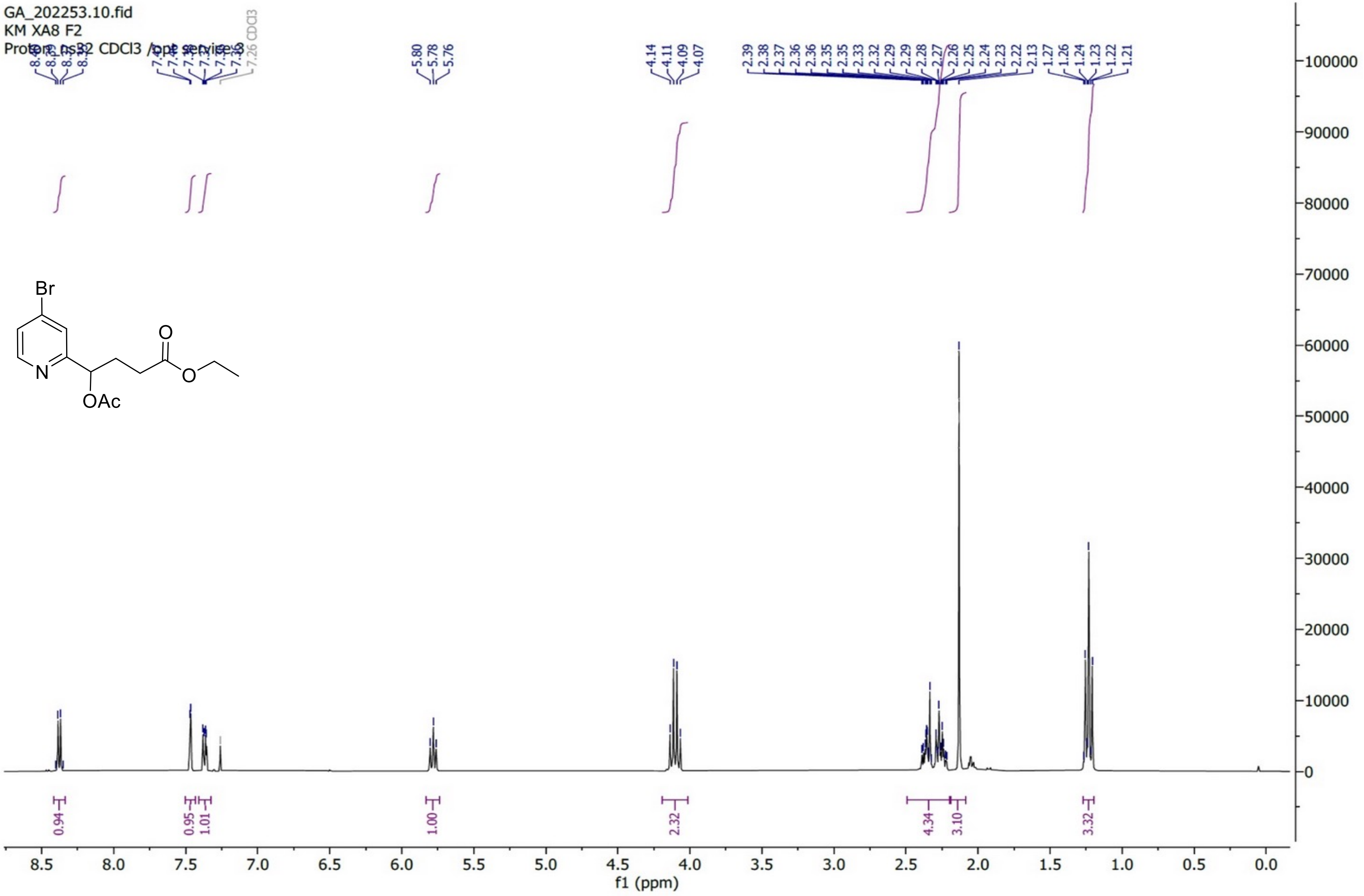

Ethyl 4-acetoxy-4-(4-bromopyridin-2-yl)butanoate (53)

^{13}C-NMR, 75 MHz, $CDCl_3$

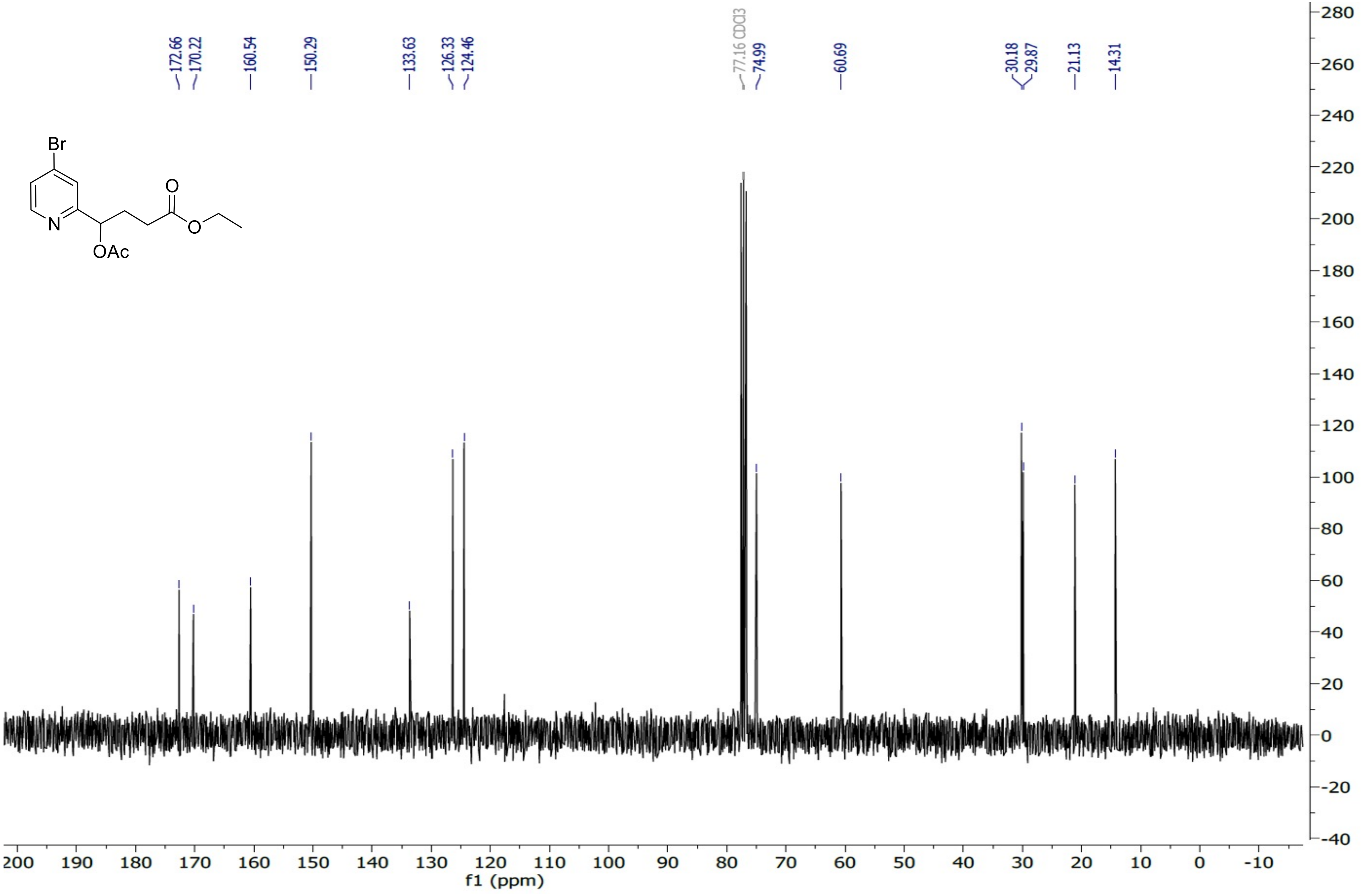

Ethyl 4-(4-bromopyridin-2-yl)-4-butoxybutanoate (54)

^{1}H-NMR, 300 MHz, $CDCl_3$

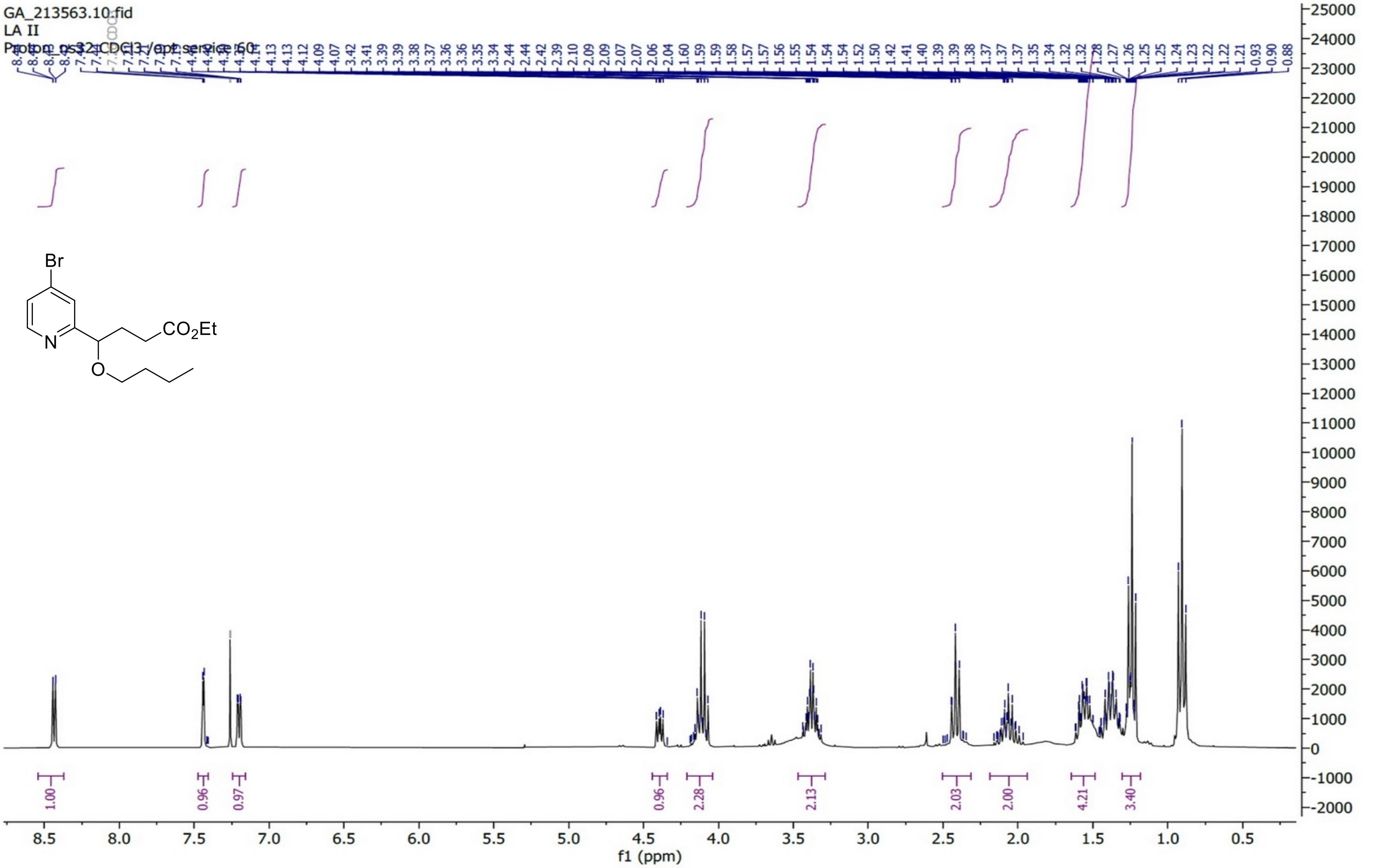

Ethyl 4-(4-bromopyridin-2-yl)-4-butoxybutanoate (54)

^{13}C-NMR, 75 MHz, $CDCl_3$

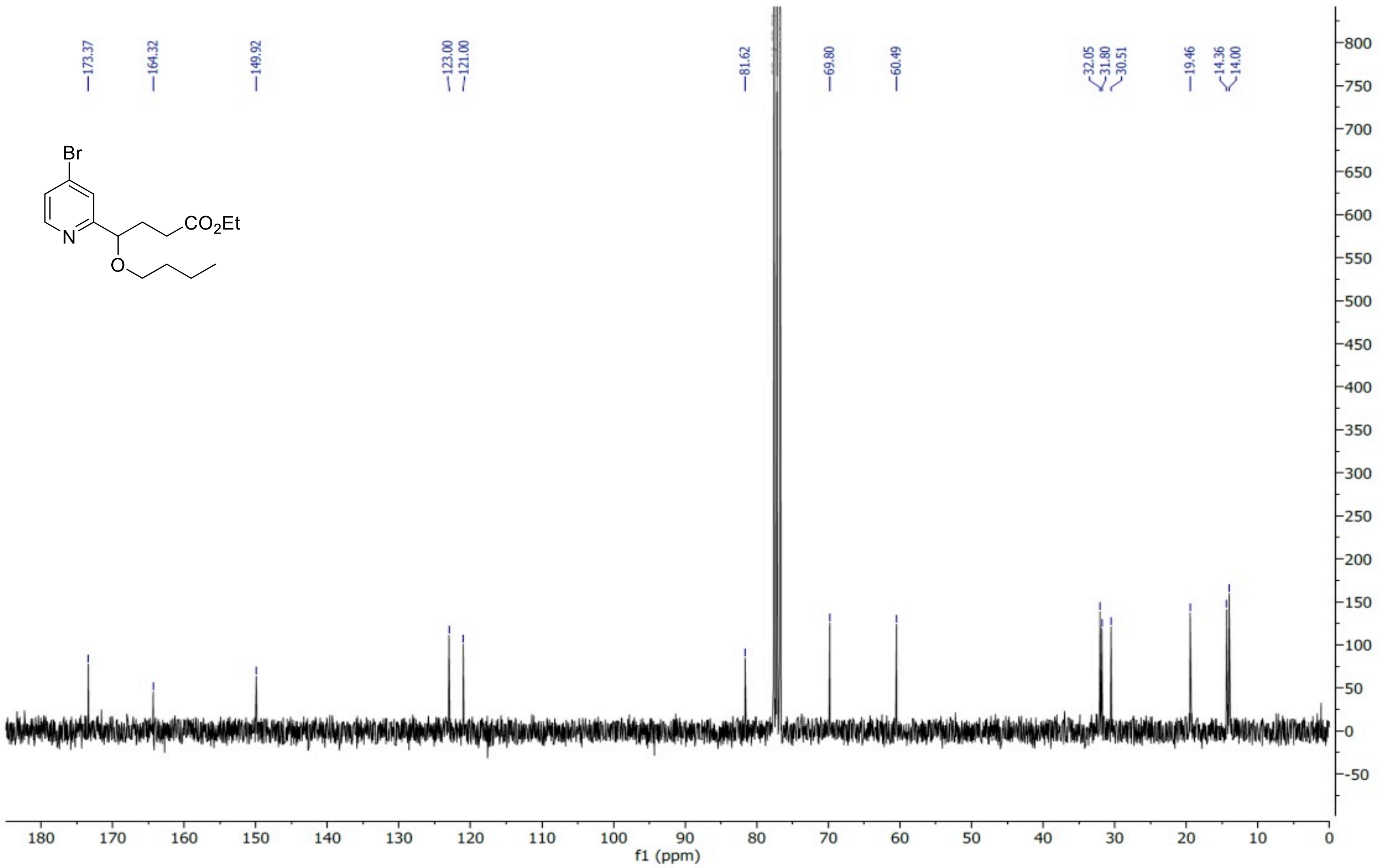

_S_-Methyl _O_-(undecan-2-yl) carbonodithioate (55)

^{1}H-NMR (300 MHz, $CDCl_3$)

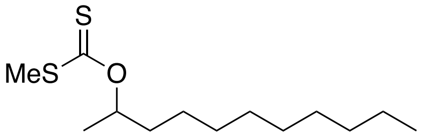

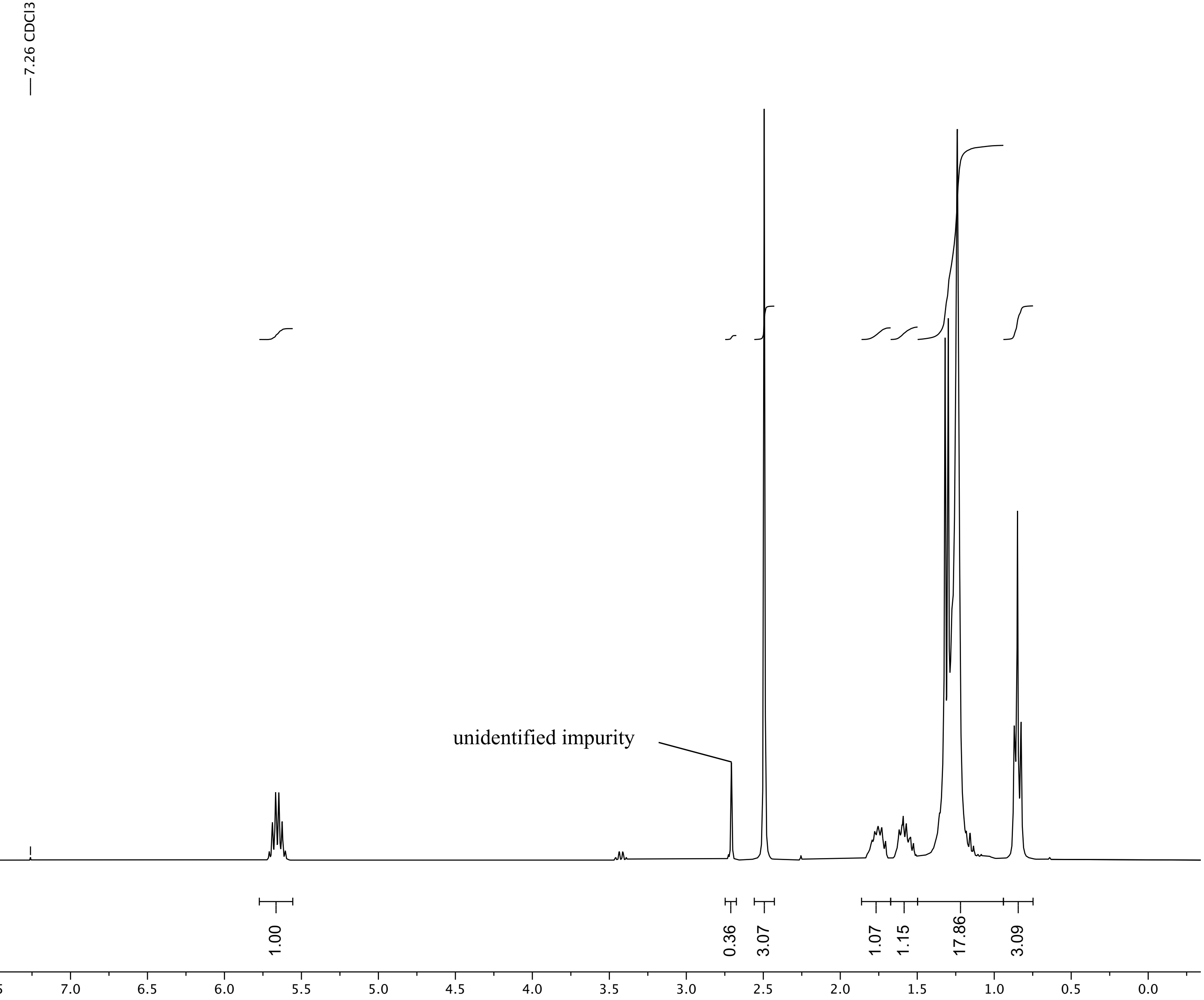

_S_-Methyl _O_-(undecan-2-yl) carbonodithioate (55)

^{1}H-NMR (300 MHz, $CDCl_3$)

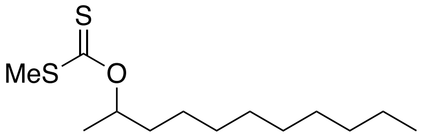

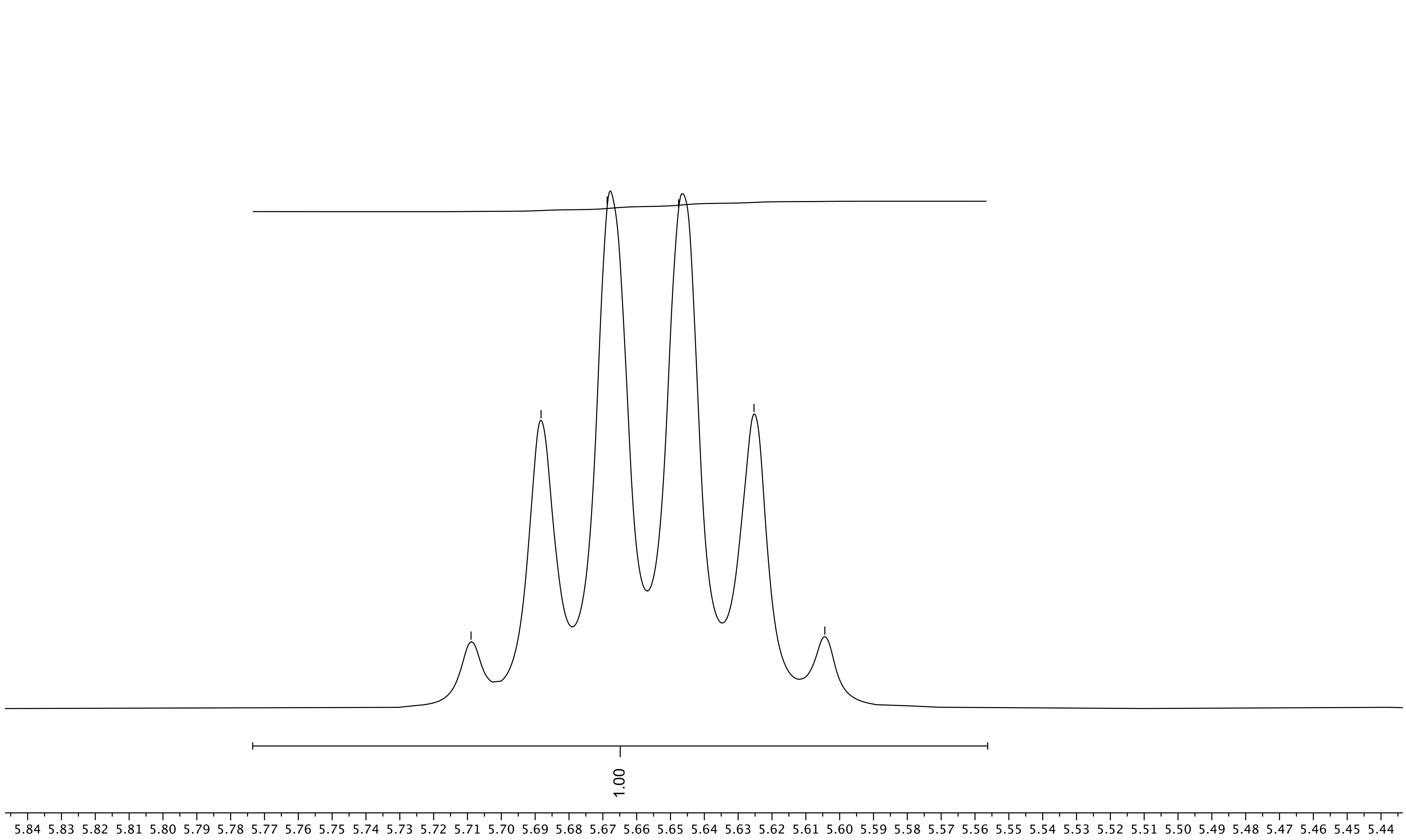

S-Methyl O-(undecan-2-yl) carbonodithioate (55)

^{1}H-NMR (300 MHz, $CDCl_3$)

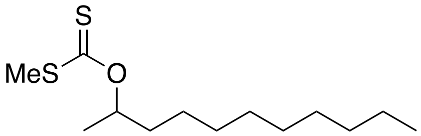

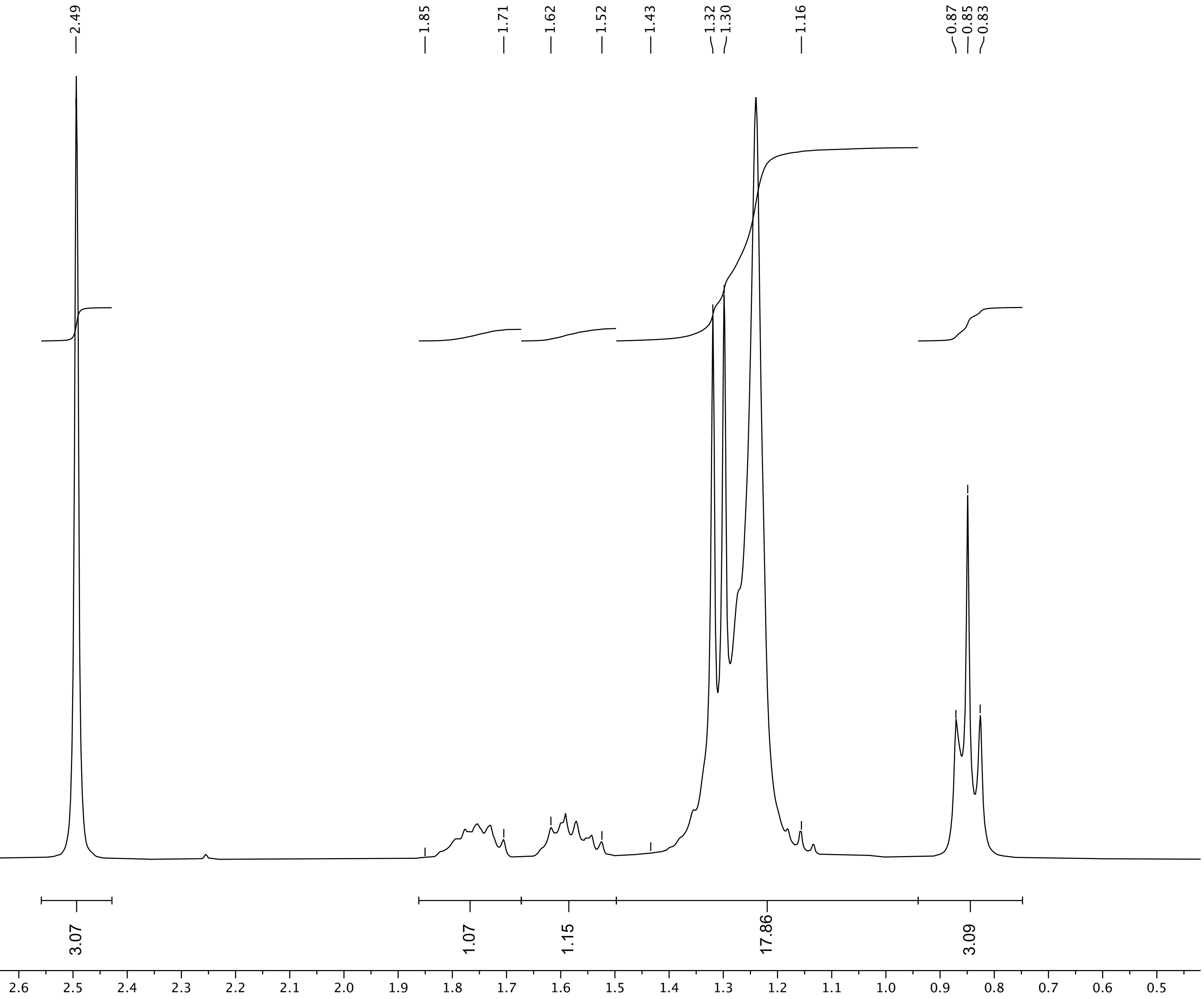

_S_-Methyl _O_-(undecan-2-yl) carbonodithioate (55)

^{13}C-NMR (75 MHz, $CDCl_3$)

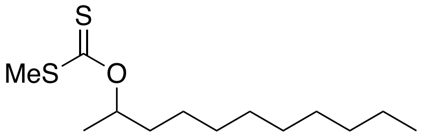

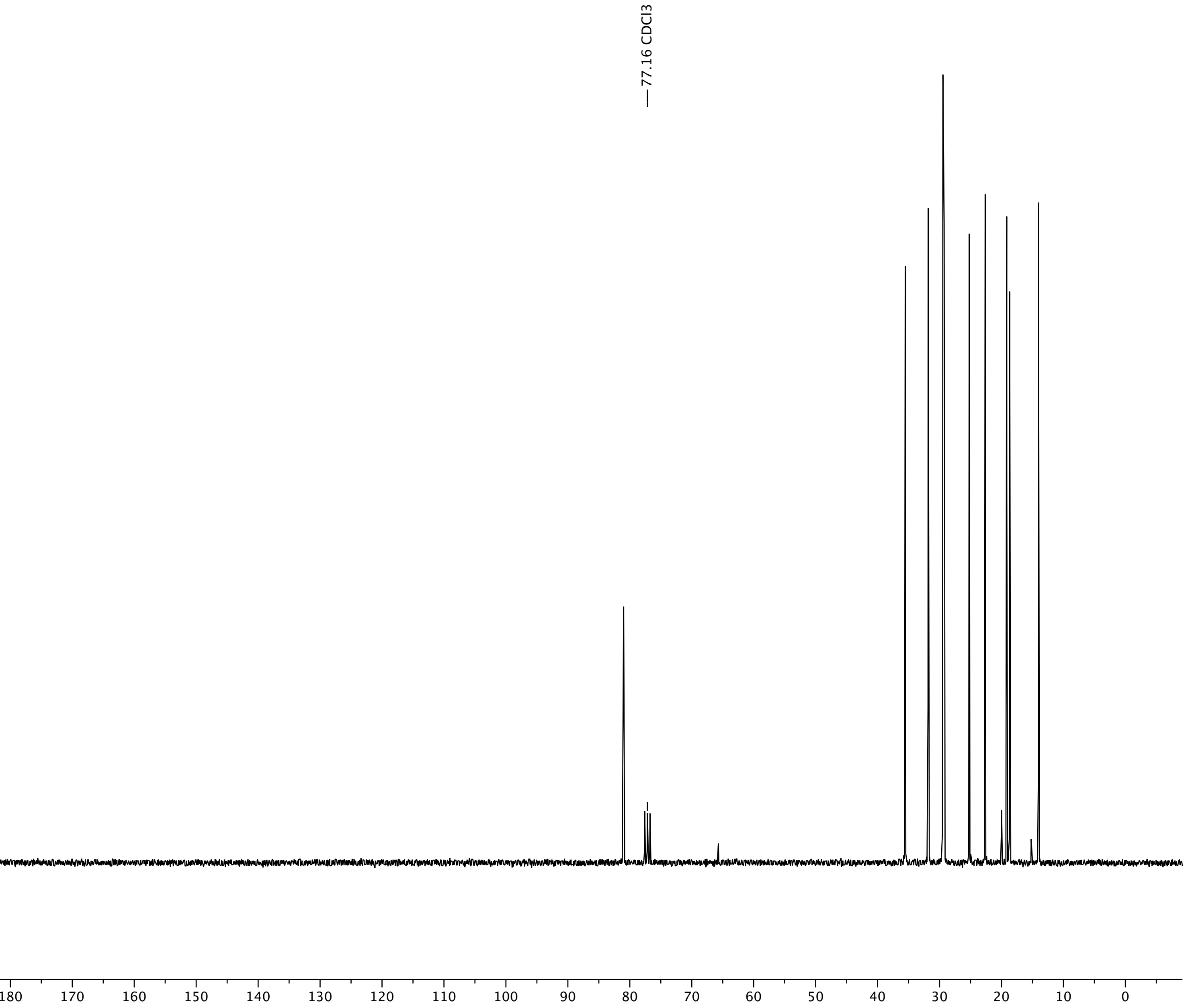

S-Methyl O-(undecan-2-yl) carbonodithioate (55)

^{1}H-NMR (75 MHz, $CDCl_3$)

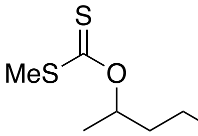

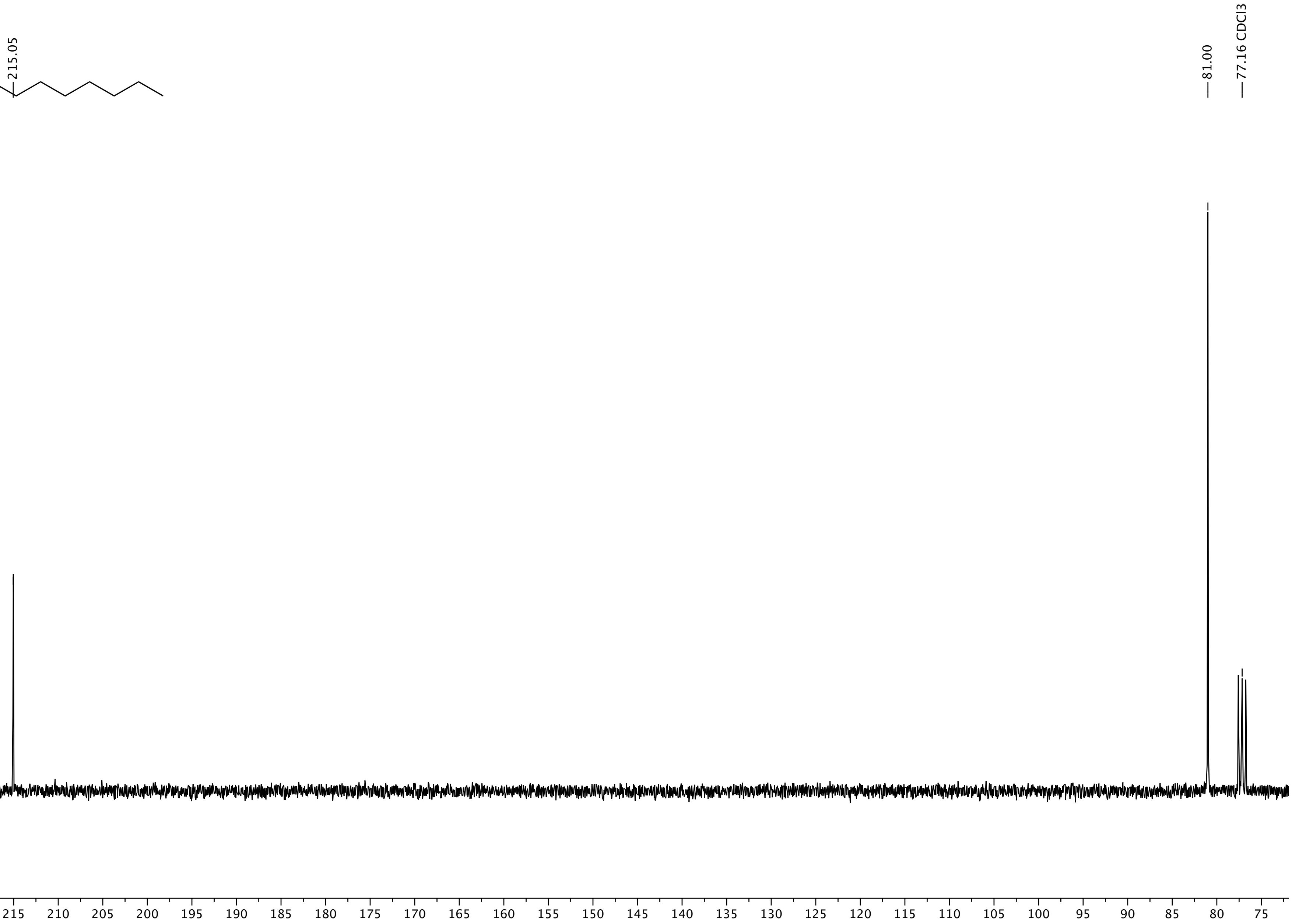

_S_-Methyl _O_-(undecan-2-yl) carbonodithioate (55)

^{13}C-NMR (75 MHz, $CDCl_3$)

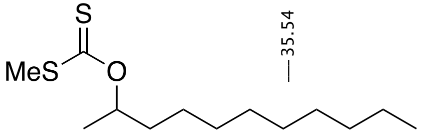

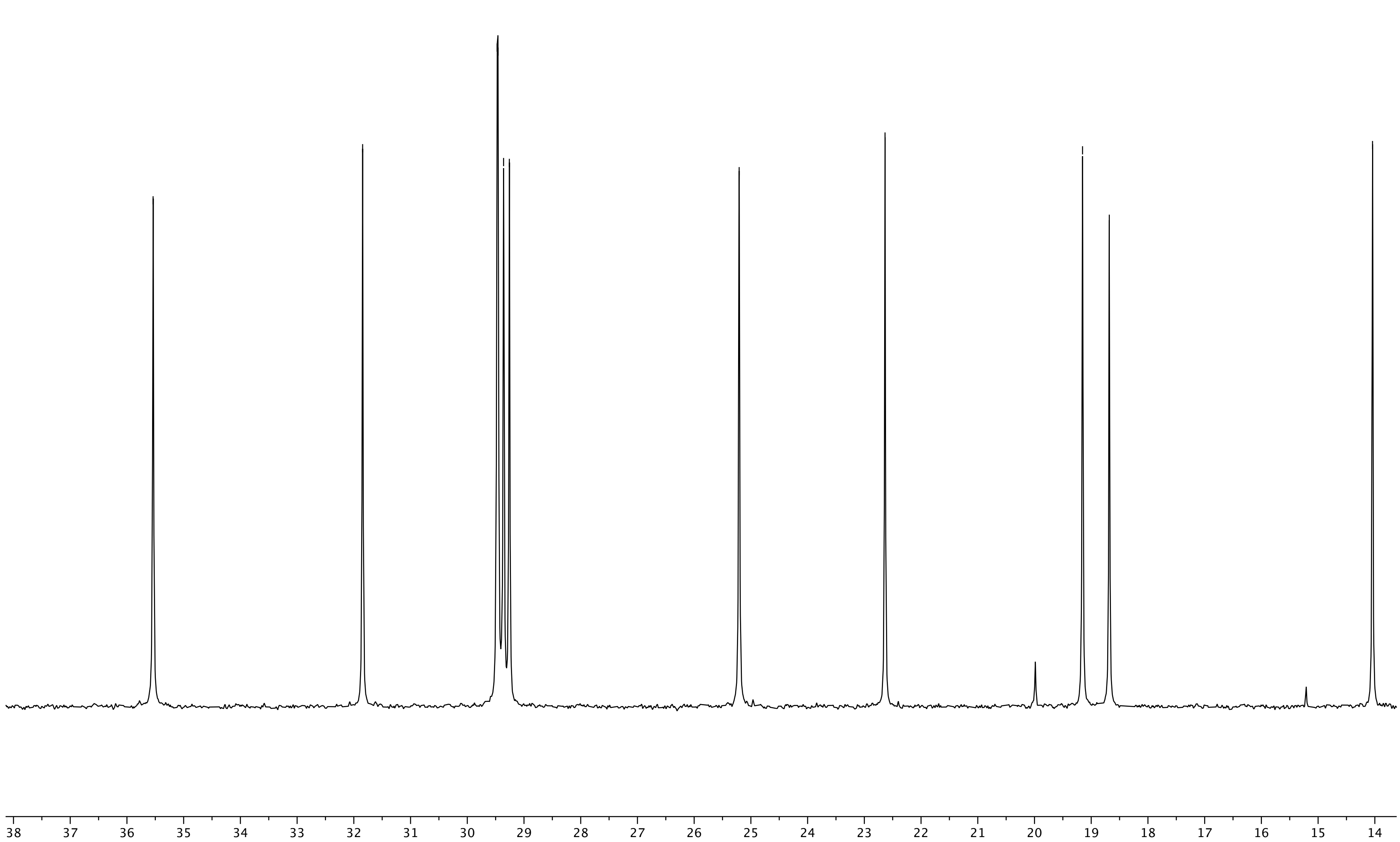

_S_-Methyl _O_-(undecan-2-yl) carbonodithioate (55)

Dept-135 (75 MHz, $CDCl_3$)

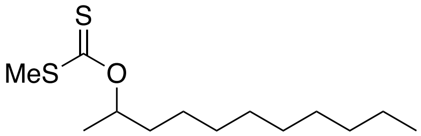

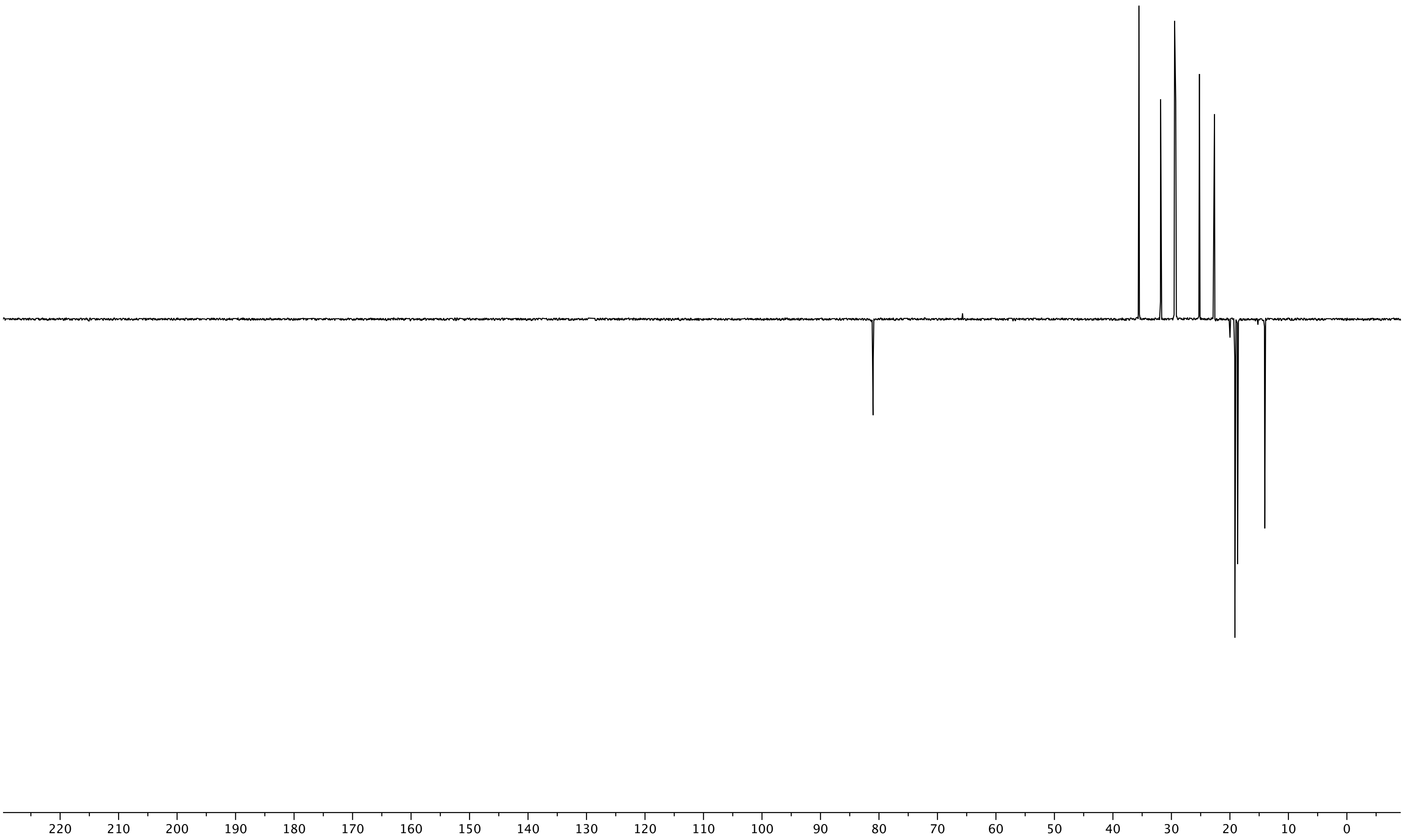

S-Methyl O-(undecan-2-yl) carbonodithioate (55)

Dept-135 (75 MHz, $CDCl_3$)

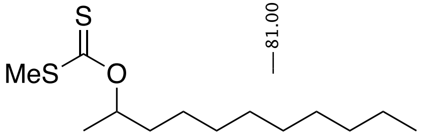

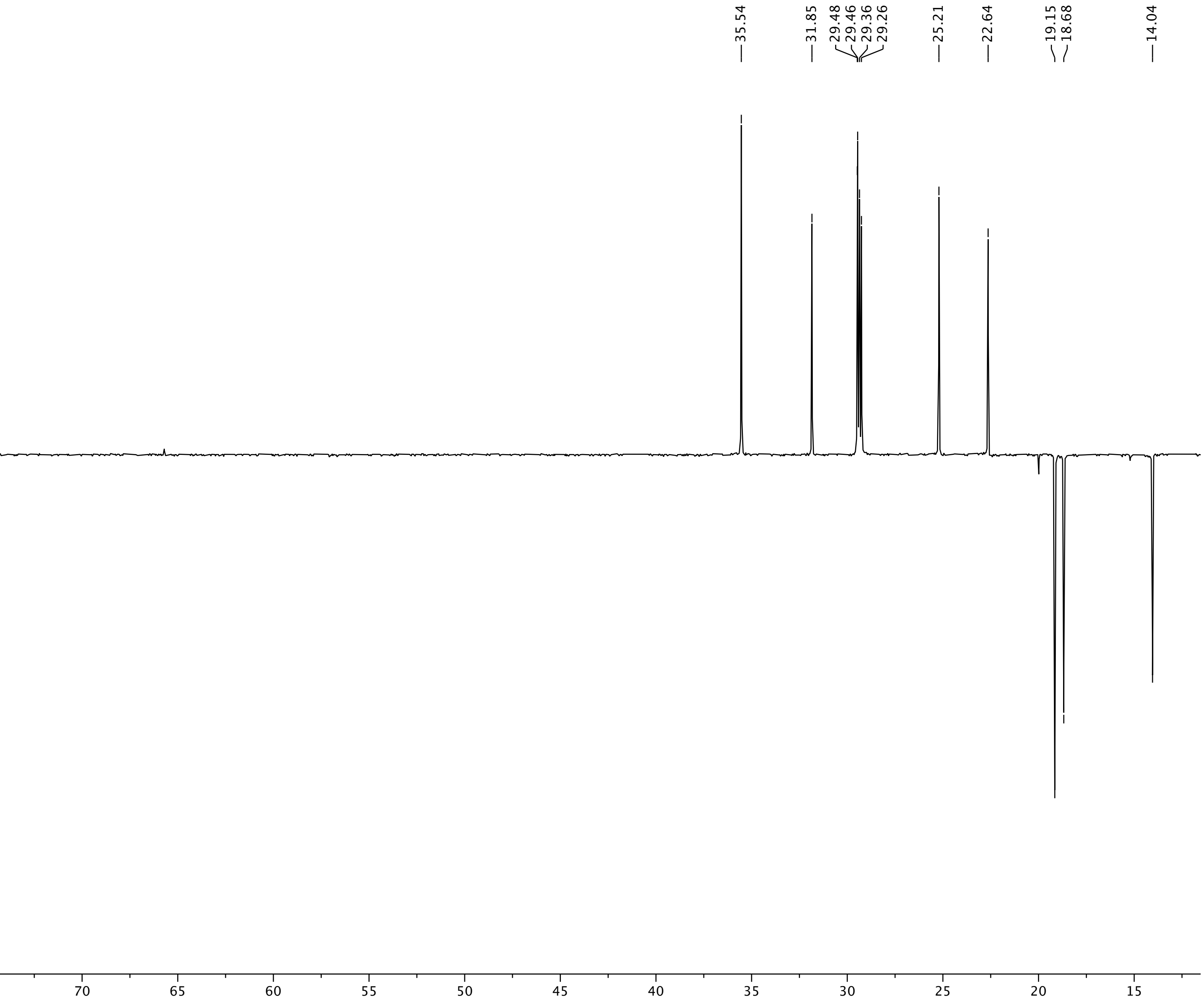

2,6-Dimethyl-4-(undecan-2-yl)pyridine (56)

^{1}H-NMR (400 MHz, $CDCl_3$)

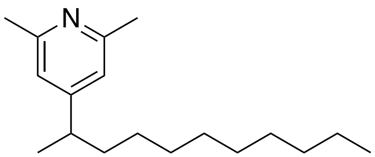

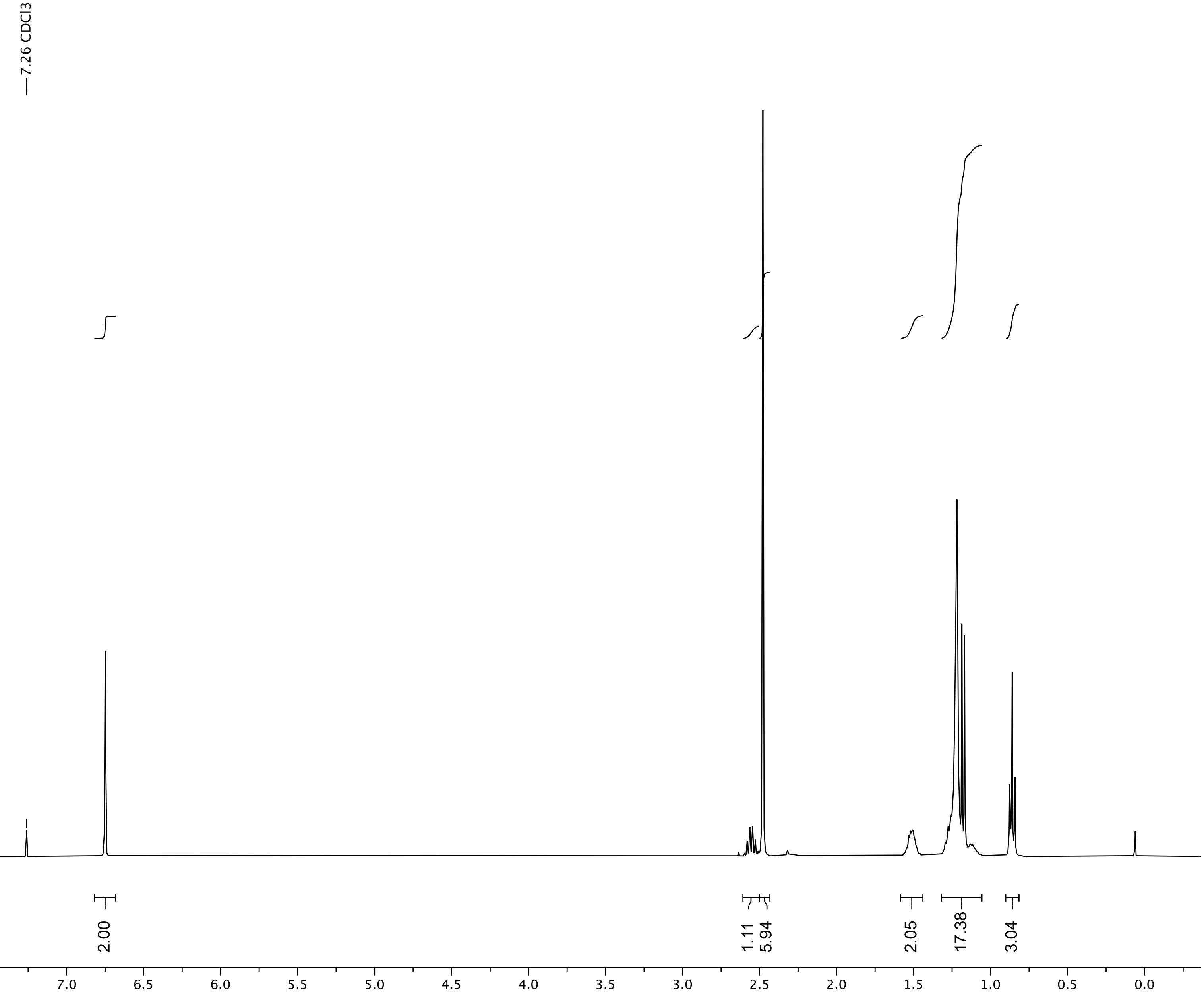

2,6-Dimethyl-4-(undecan-2-yl)pyridine (56)

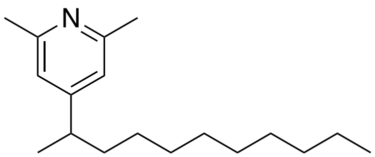

^{1}H-NMR (400 MHz, $CDCl_3$)

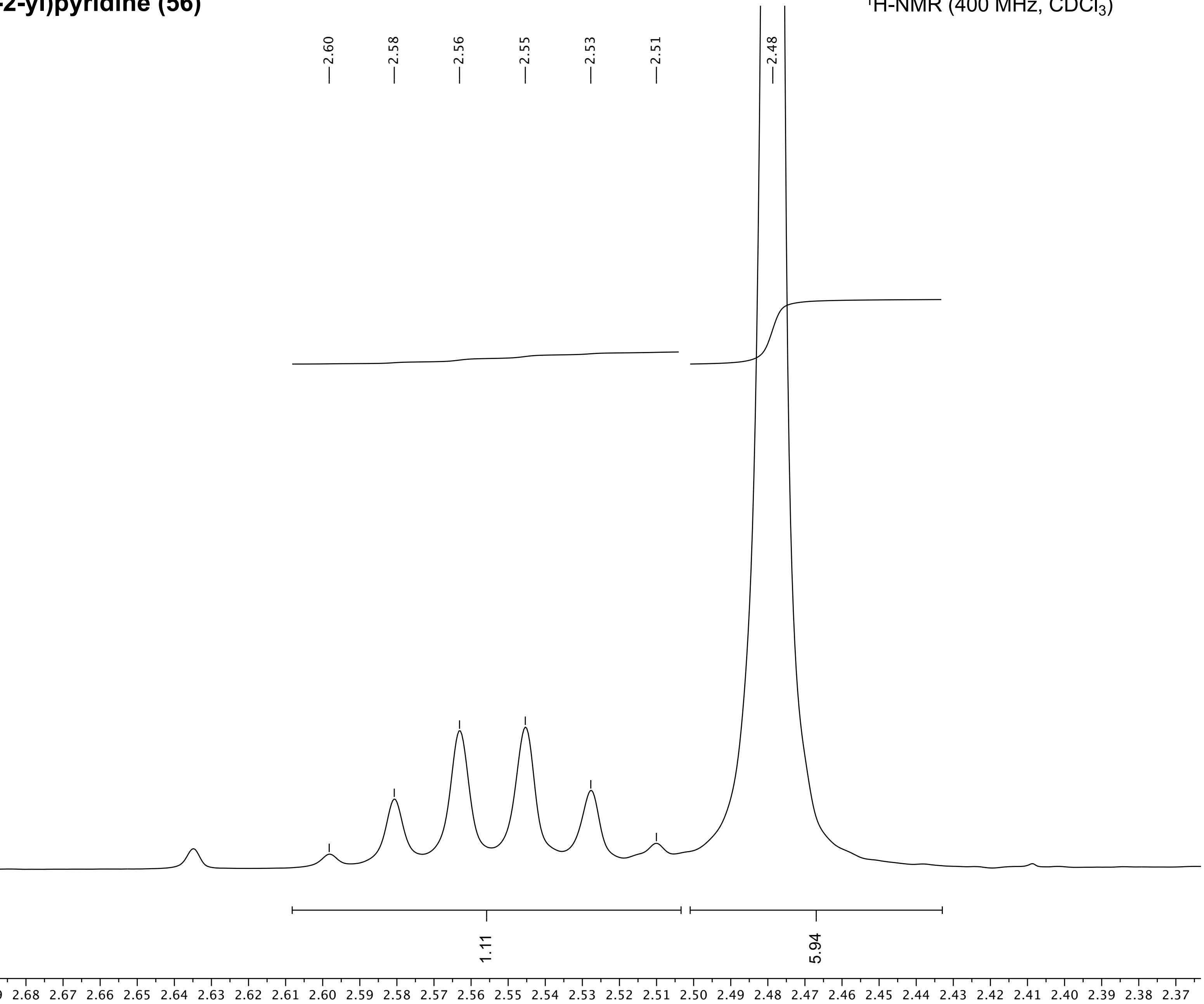

2,6-Dimethyl-4-(undecan-2-yl)pyridine (56)

^{1}H-NMR (400 MHz, $CDCl_3$)

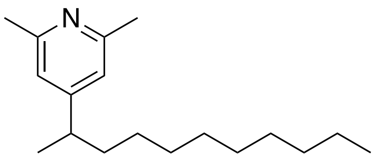

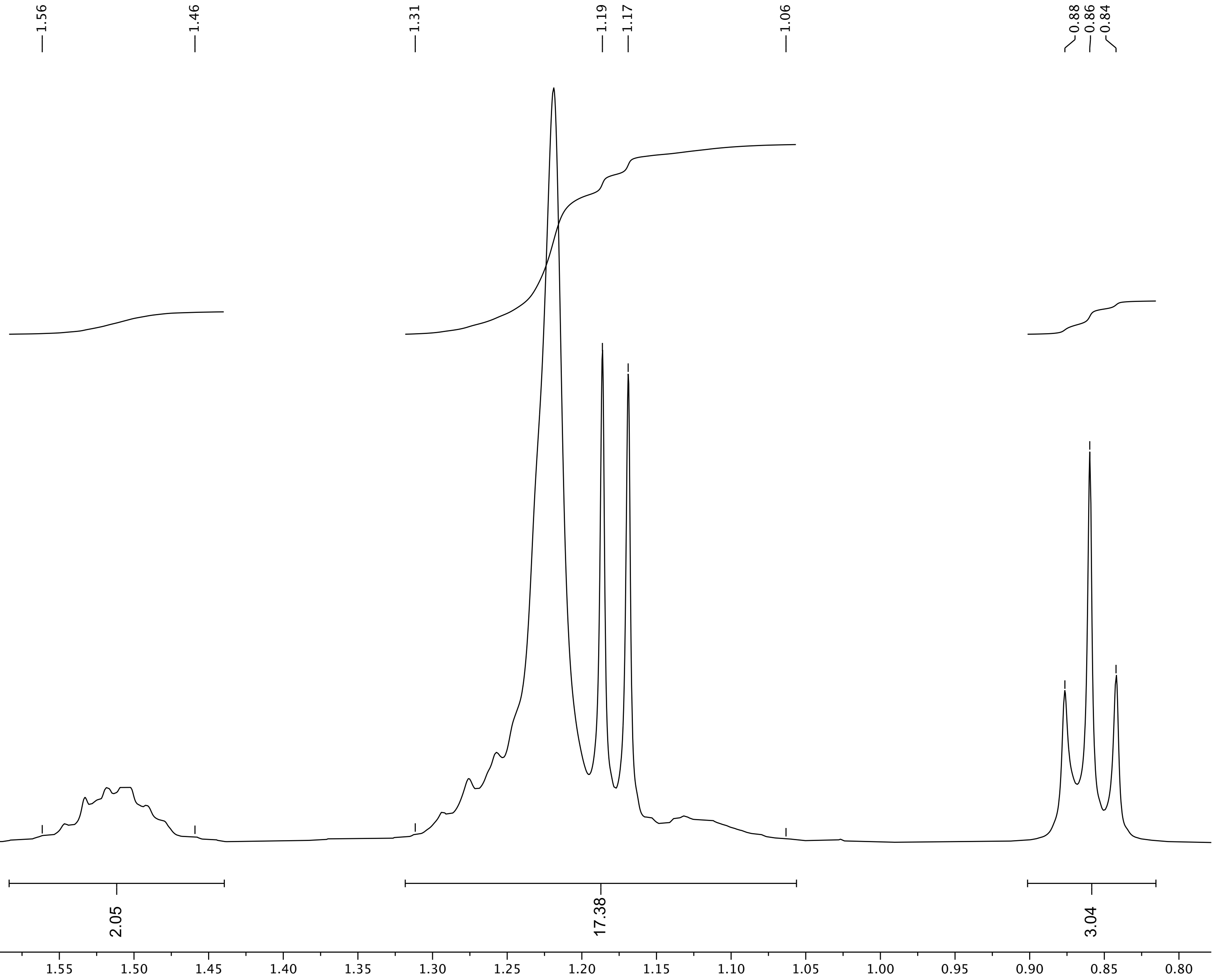

2,6-Dimethyl-4-(undecan-2-yl)pyridine (56)

^{13}C-NMR (101 MHz, $CDCl_3$)

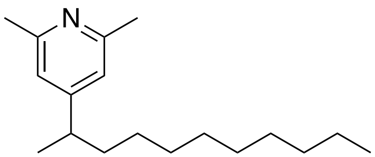

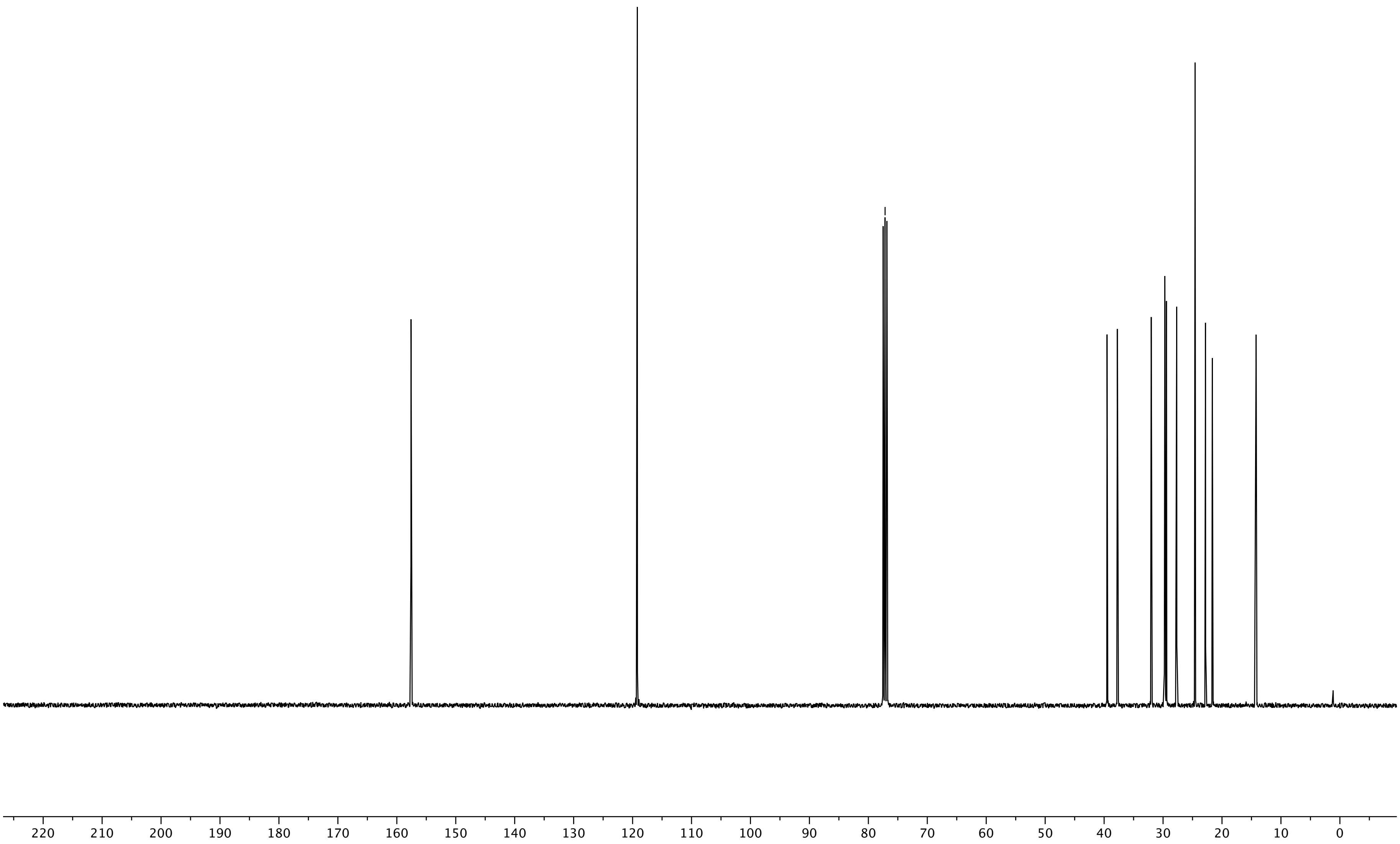

2,6-Dimethyl-4-(undecan-2-yl)pyridine (56)

^{13}C-NMR (101 MHz, $CDCl_3$)

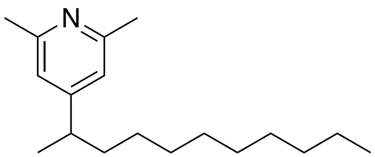

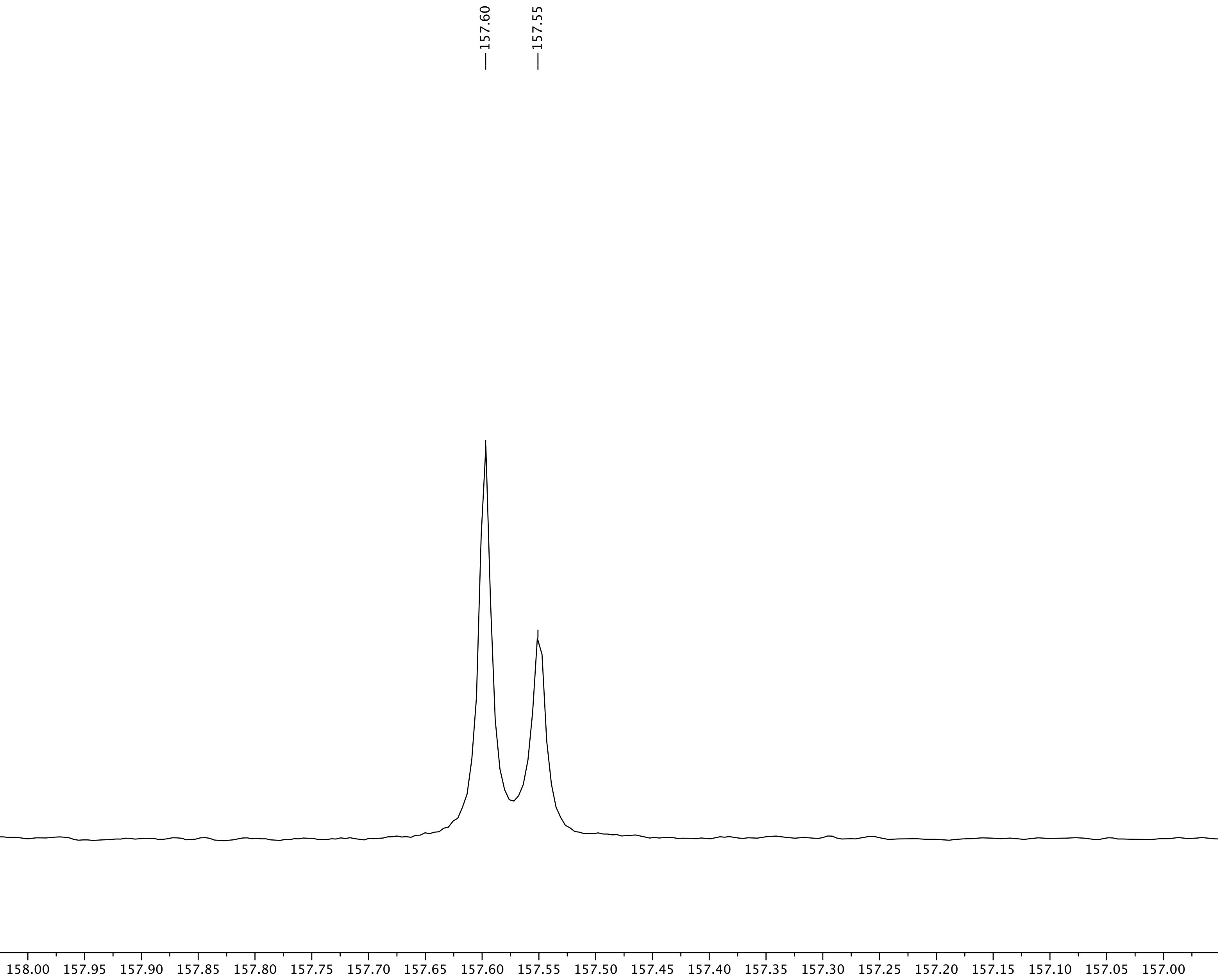

2,6-Dimethyl-4-(undecan-2-yl)pyridine (56)

^{13}C-NMR (101 MHz, $CDCl_3$)

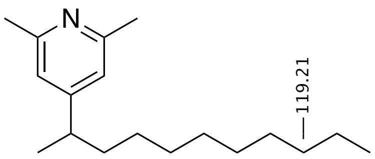

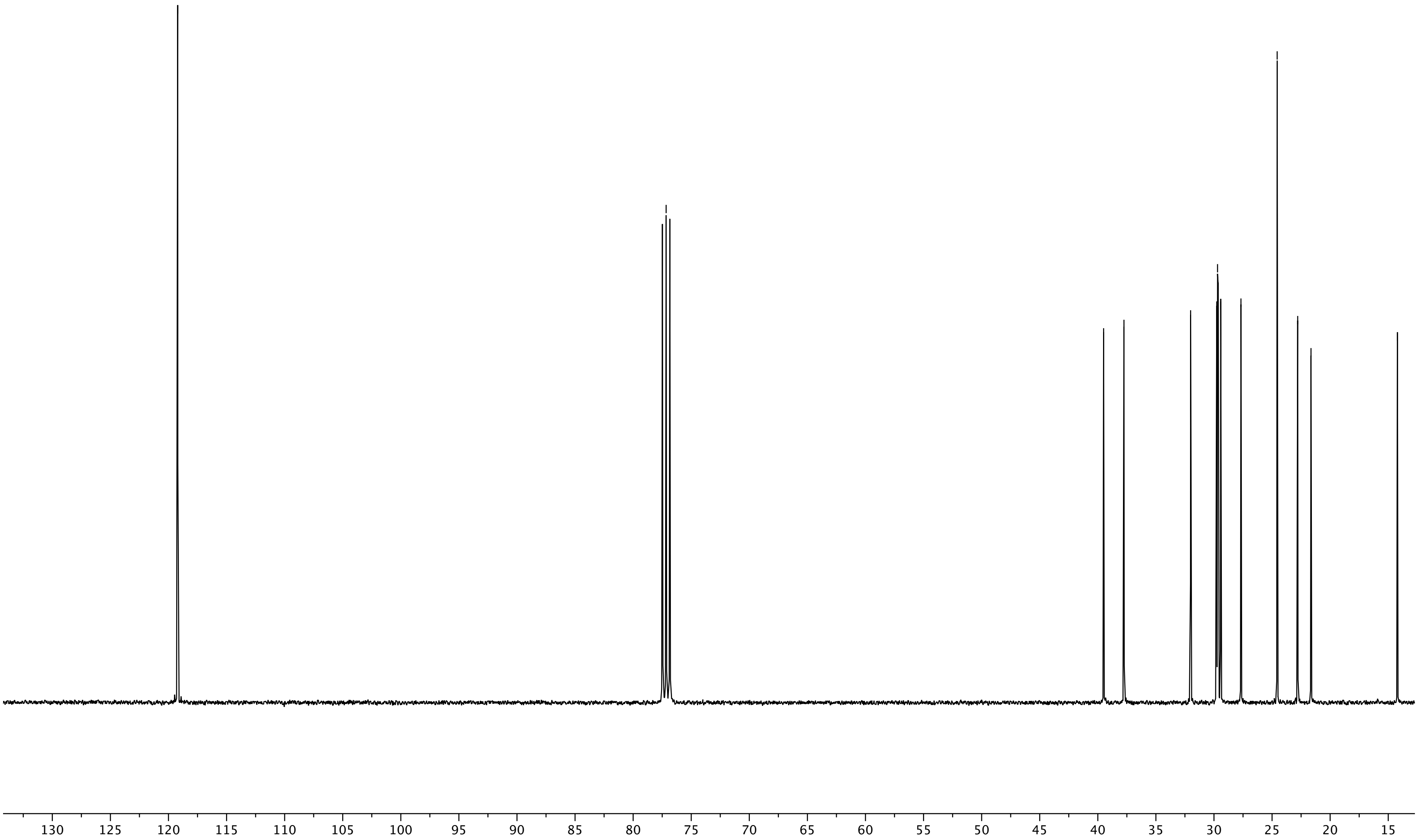

2,6-Dimethyl-4-(undecan-2-yl)pyridine (56)

^{13}C-NMR (101 MHz, $CDCl_3$)

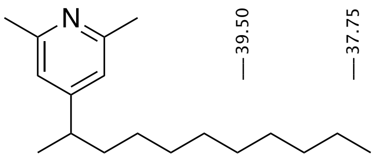

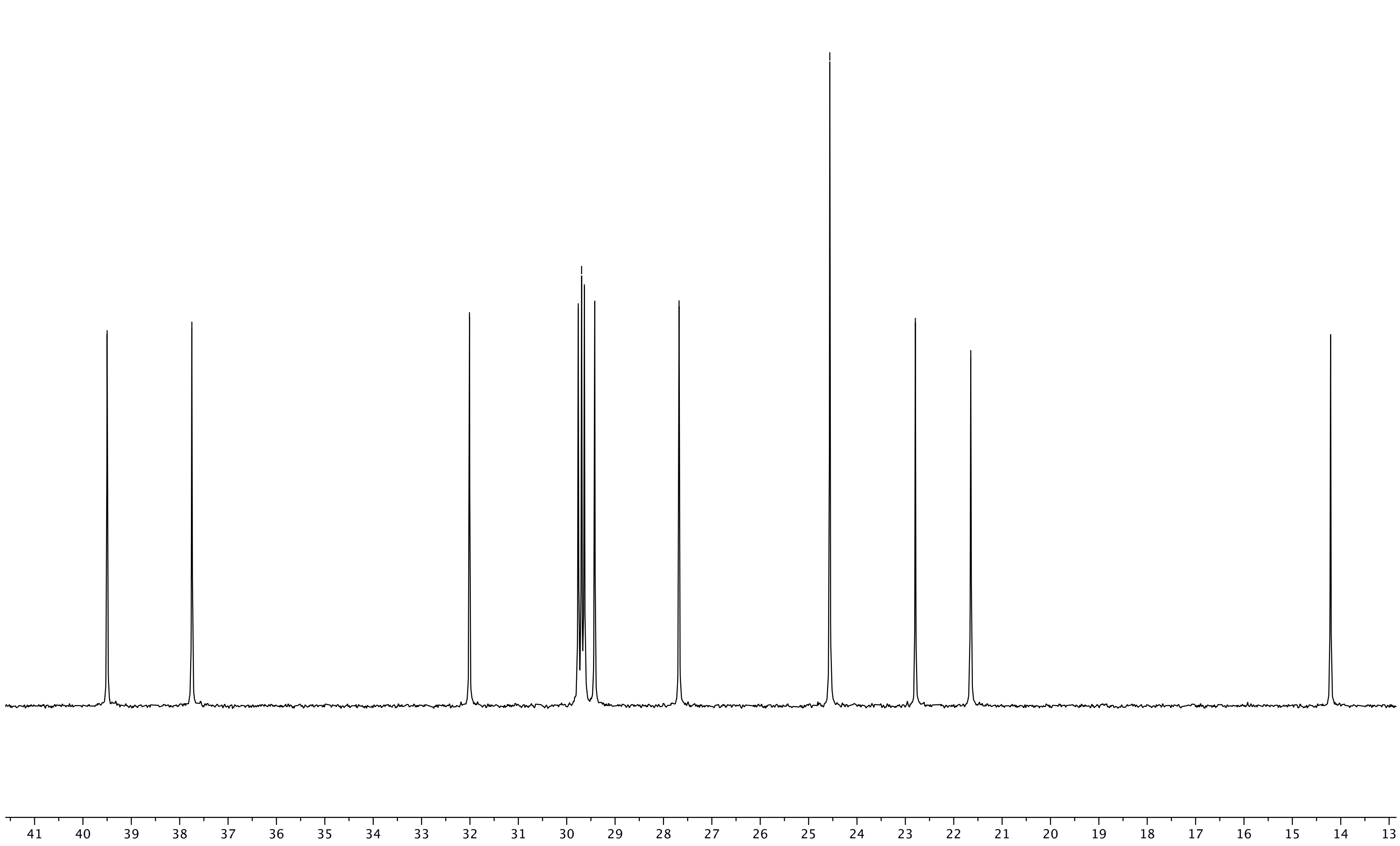

2,6-Dimethyl-4-(undecan-2-yl)pyridine (56)

Dept-135 (101 MHz, $CDCl_3$)

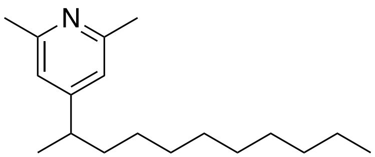

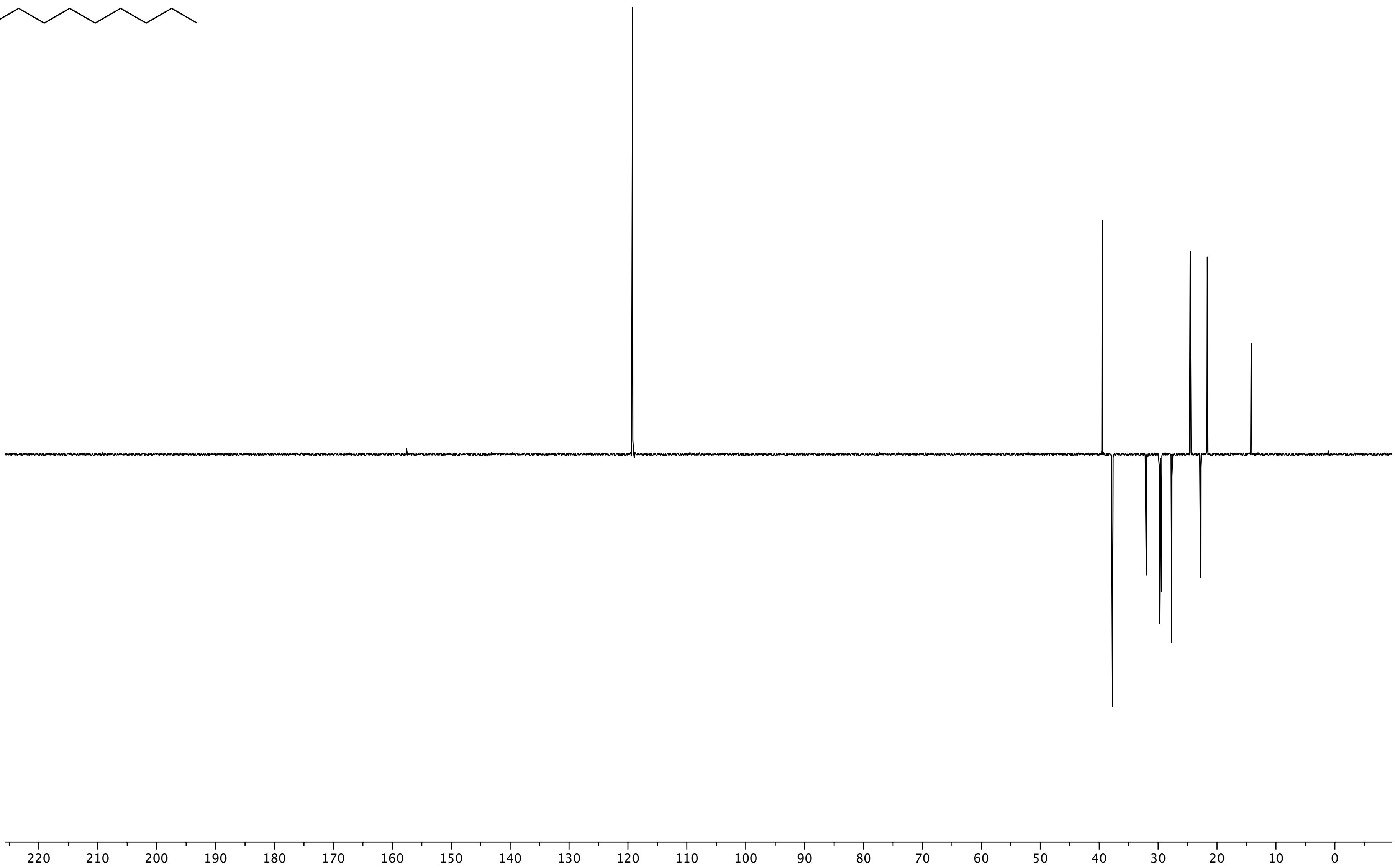

2,6-Dimethyl-4-(undecan-2-yl)pyridine (56)

Dept-135 (101 MHz, $CDCl_3$)

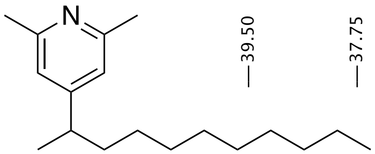

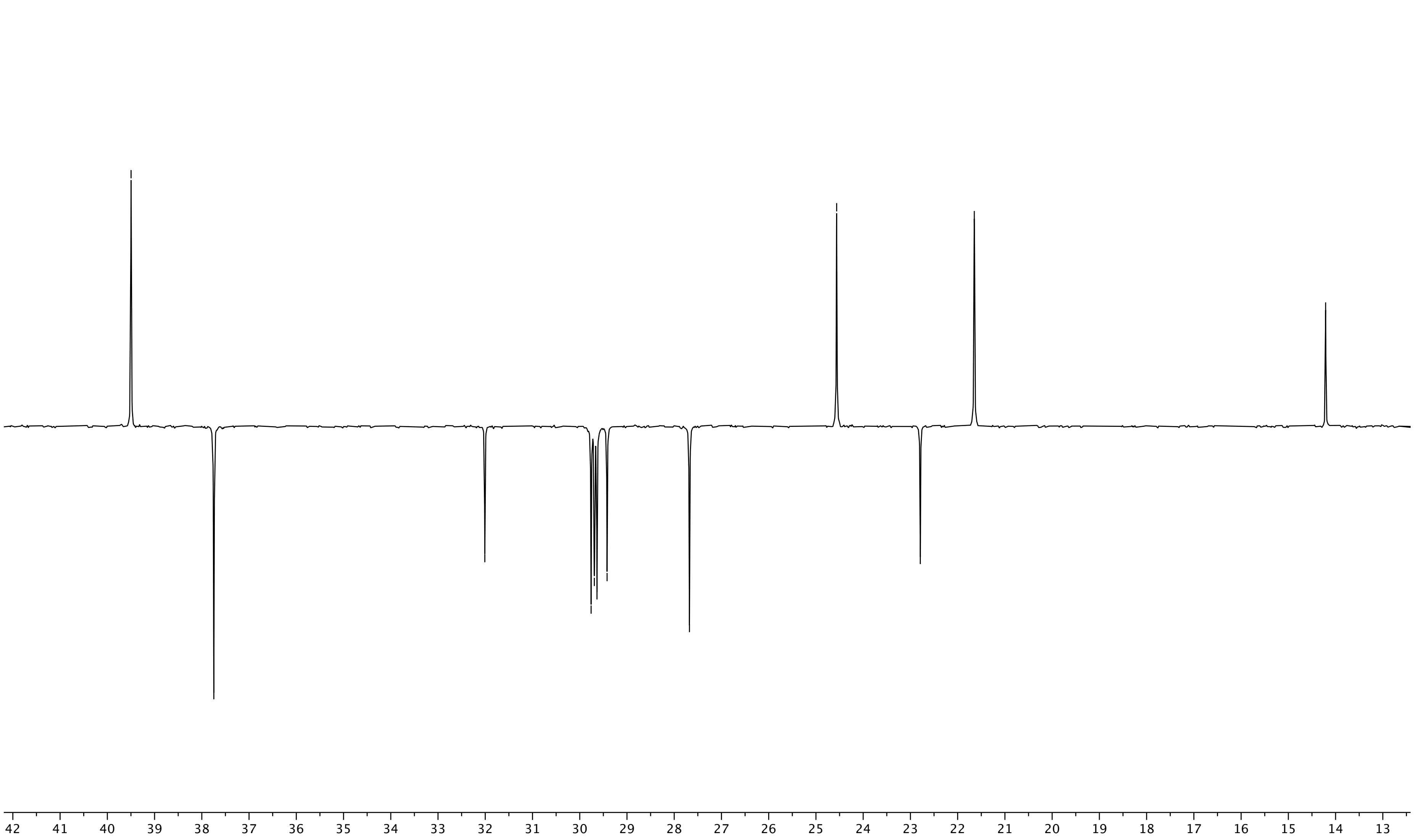

2,6-Dimethyl-4-(undecan-2-yl)pyridine (56)

^1H-^1H COSY(400 MHz, $CDCl_3$)

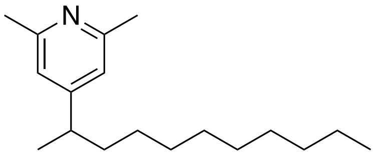

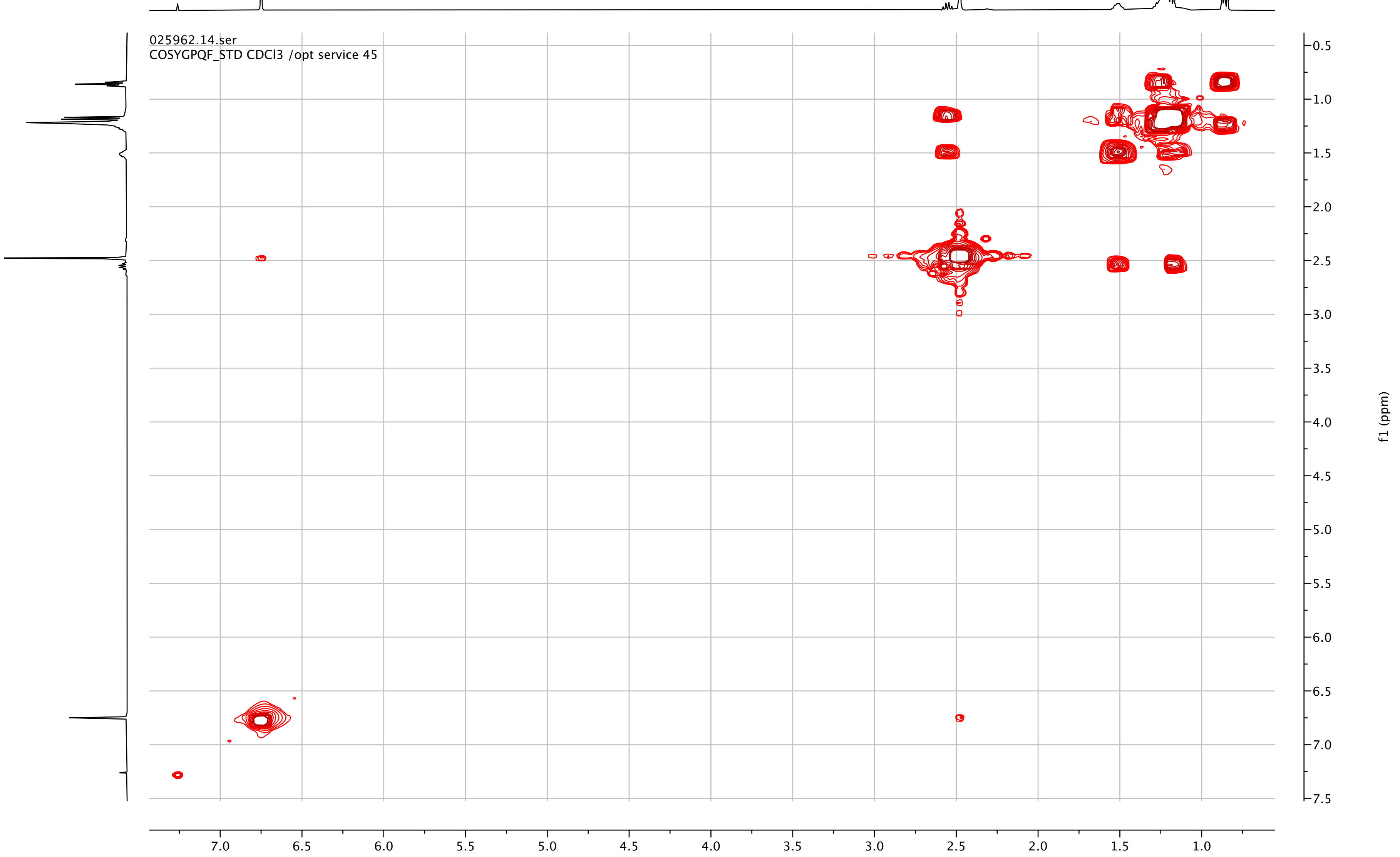

2,6-Dimethyl-4-(undecan-2-yl)pyridine (56)

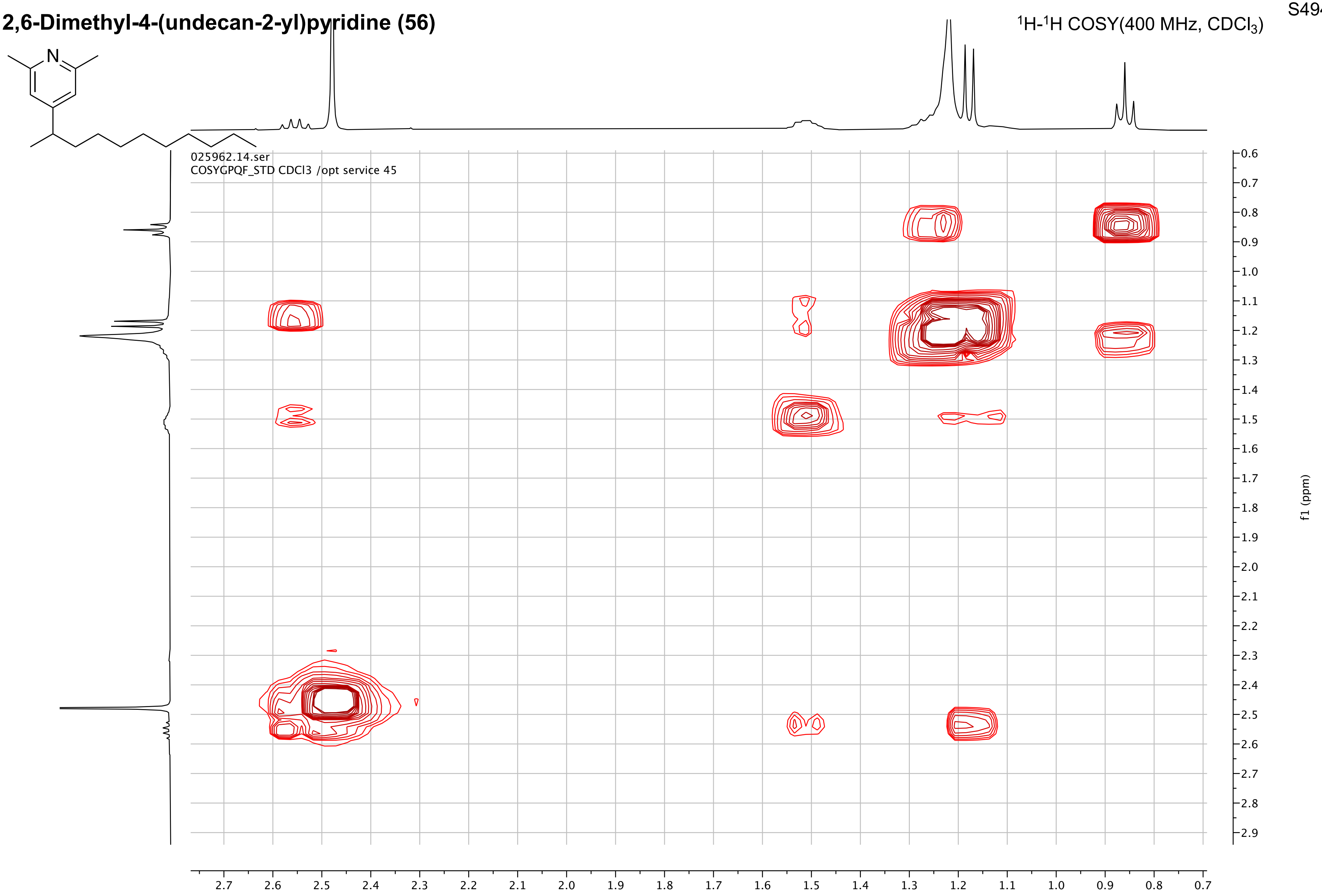

2,6-Dimethyl-4-(undecan-2-yl)pyridine (56)

HSQC (400 MHz, $CDCl_3$)

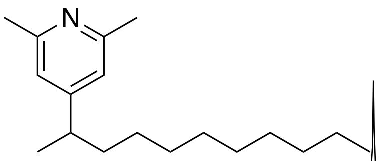

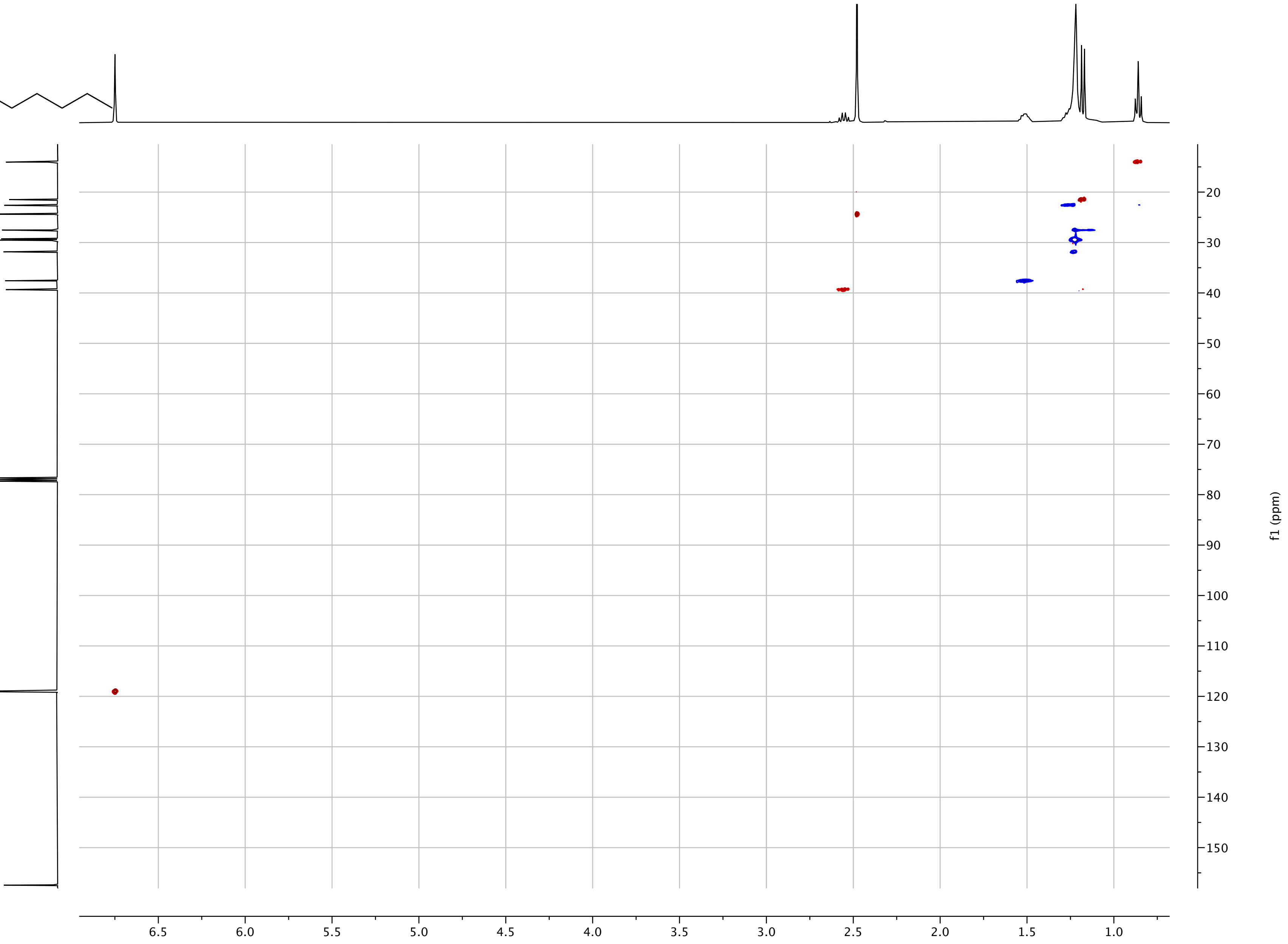

2,6-Dimethyl-4-(undecan-2-yl)pyridine (56)

HSQC (400 MHz, $CDCl_3$)

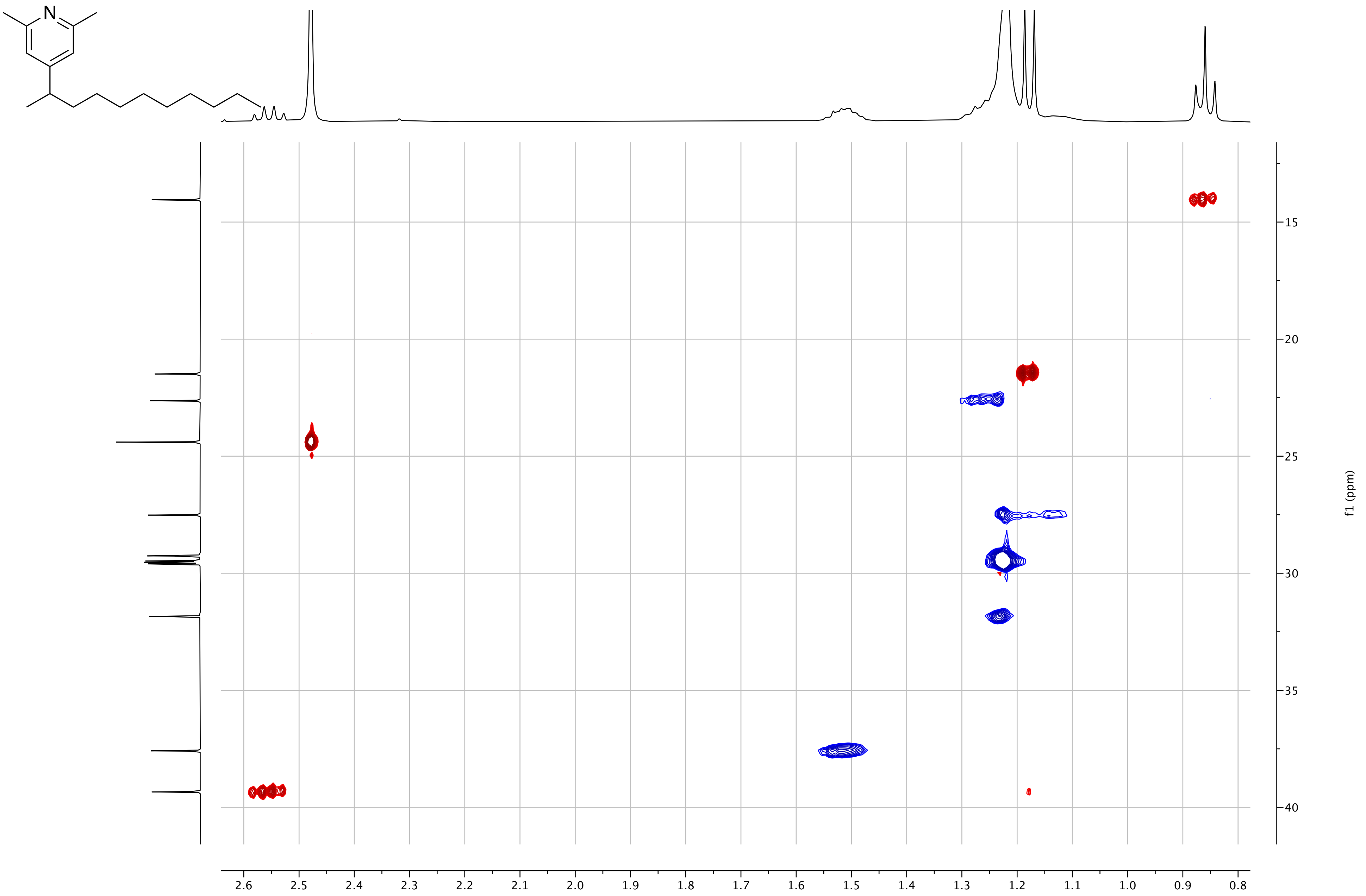

S-Methyl S-((1S,2S,3S,5R)-2,6,6-trimethylbicyclo[3.1.1]heptan-3-yl) carbonodithioate (58)

^{1}H-NMR (400 MHz, $CDCl_3$)

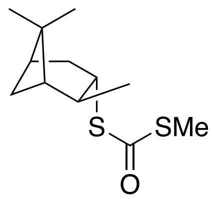

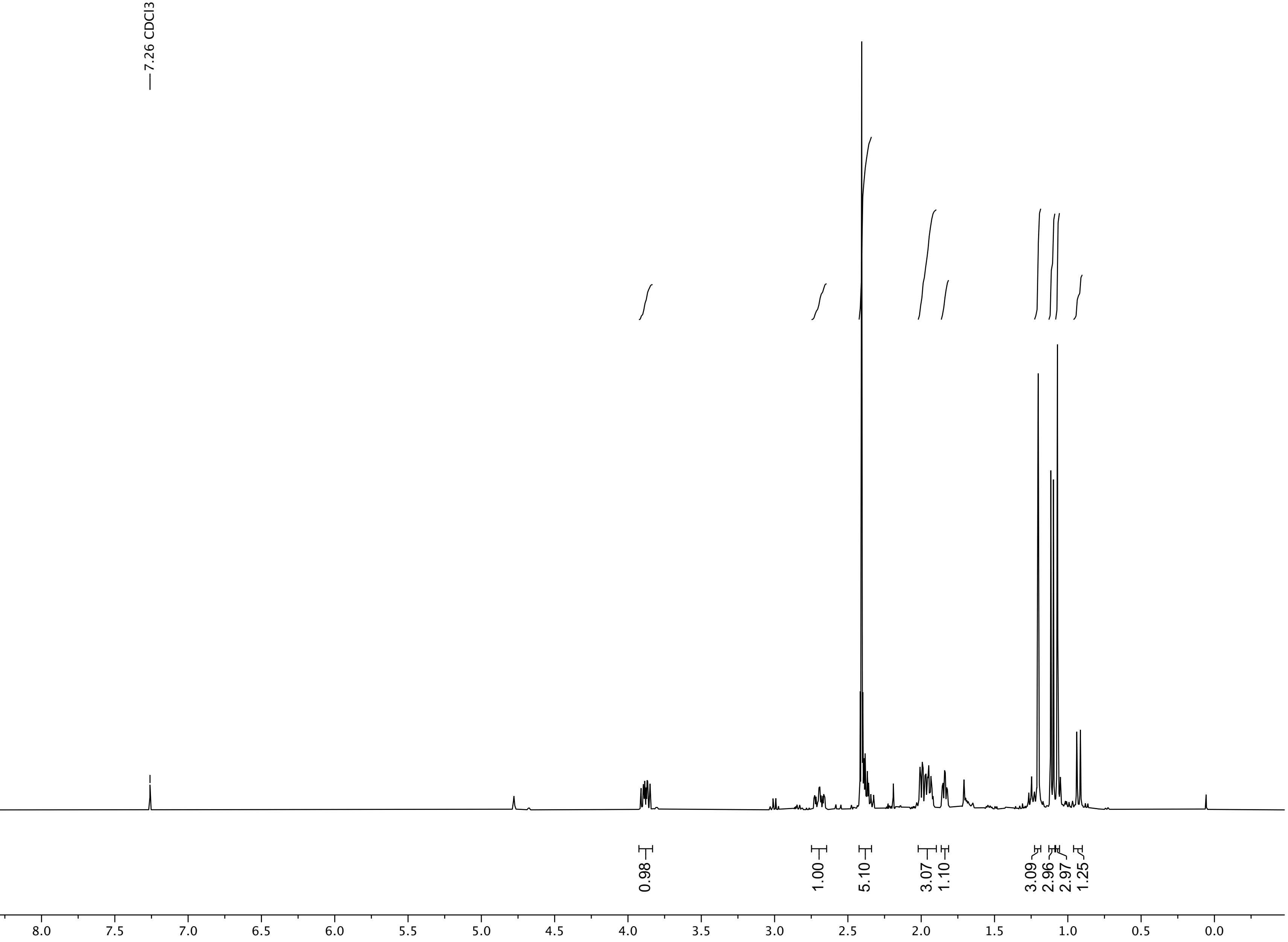

S-Methyl O-((1S,2S,3S,5R)-2,6,6-trimethylbicyclo[3.1.1]heptan-3-yl) carbonodithioate (57)

^{1}H-NMR (400 MHz, $CDCl_3$)

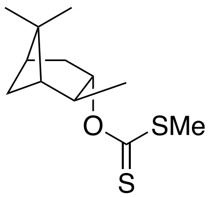

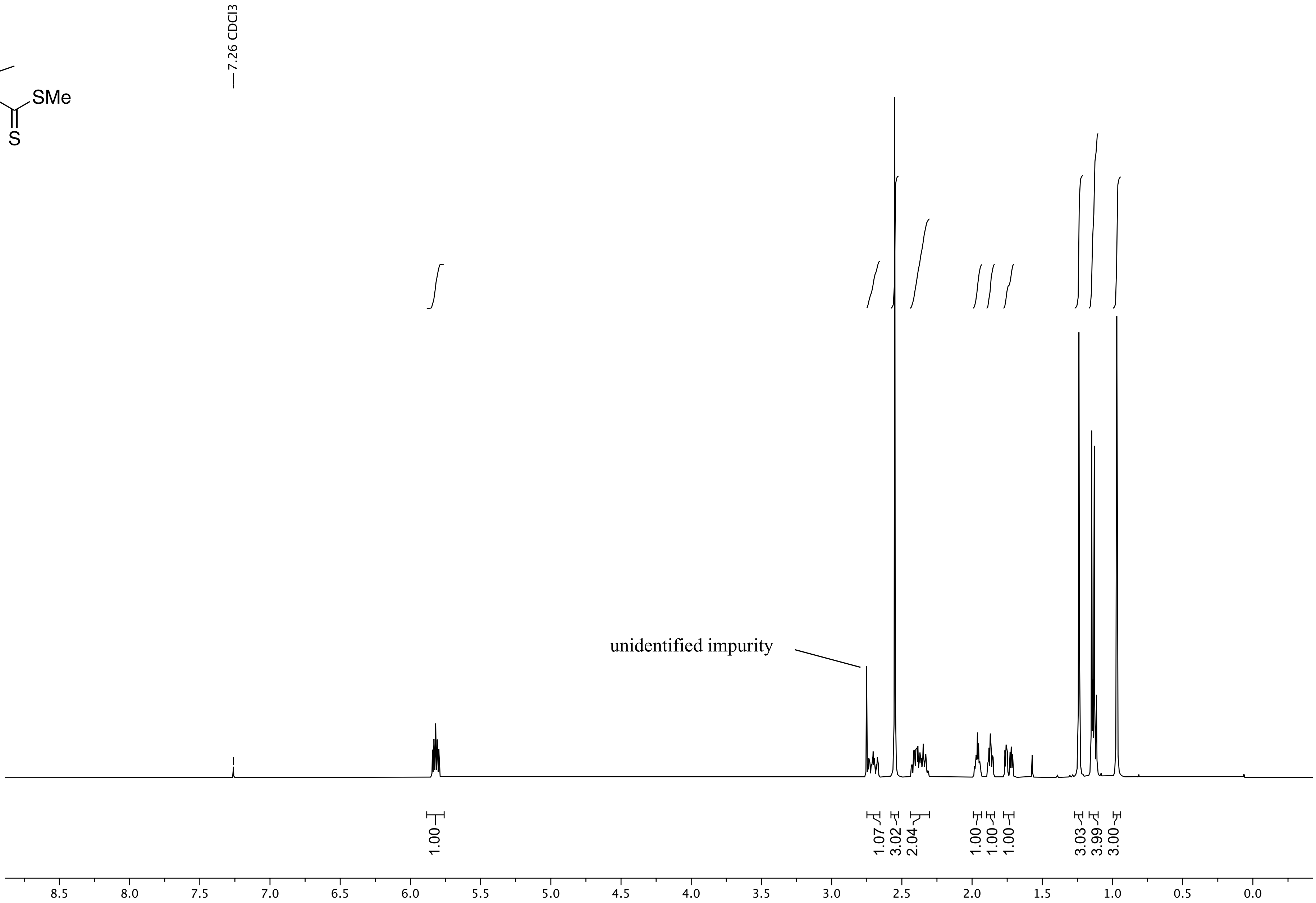

S-Methyl O-((1S,2S,3S,5R)-2,6,6-trimethylbicyclo[3.1.1]heptan-3-yl) carbonodithioate (57)

^{1}H-NMR (400 MHz, $CDCl_3$)

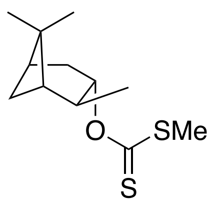

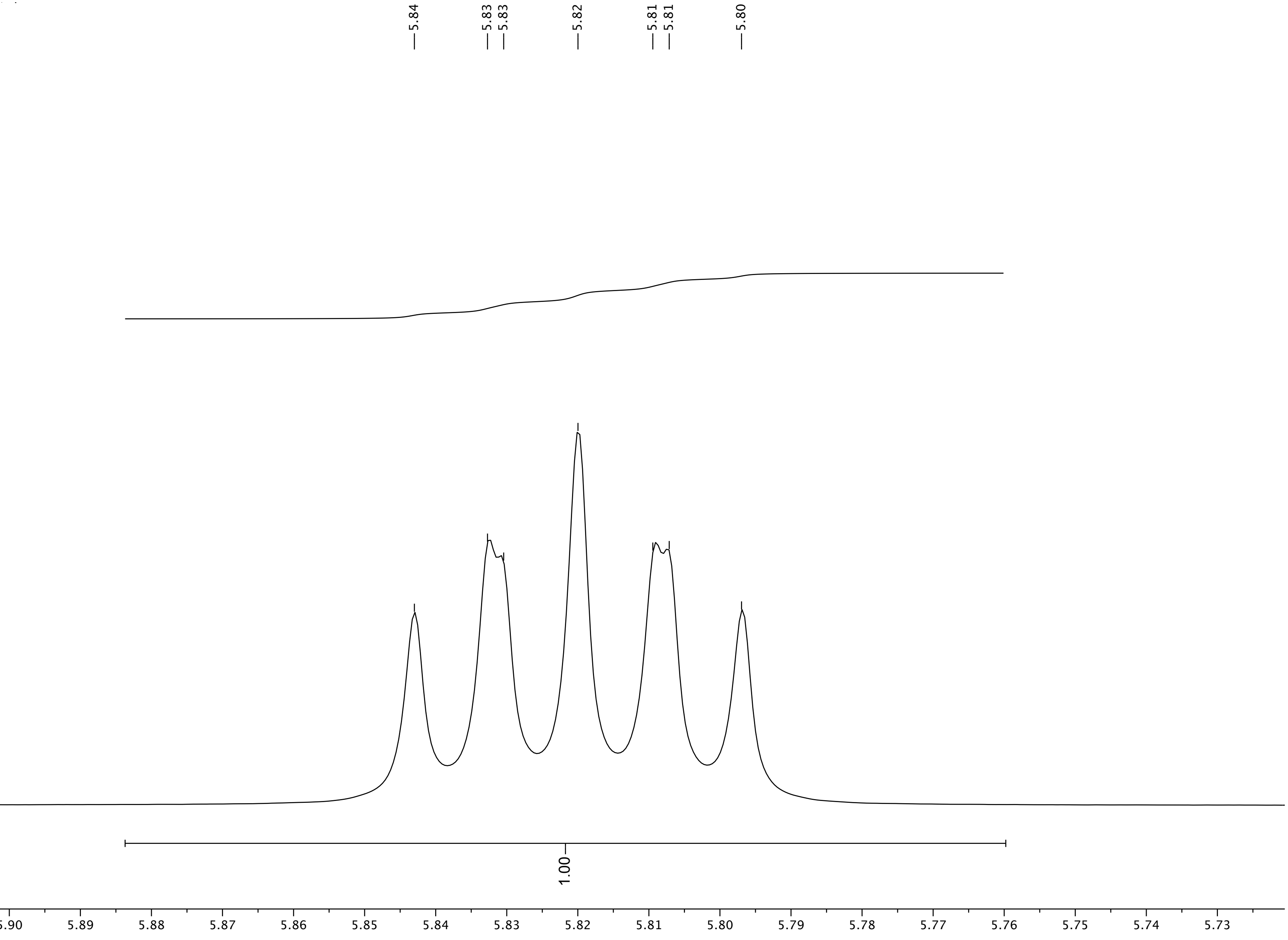

_S_-Methyl _O_-((1_S_,2_S_,3_S_,5_R_)-2,6,6-trimethylbicyclo[3.1.1]heptan-3-yl) carbonodithioate (57)

^{1}H-NMR (400 MHz, $CDCl_3$)

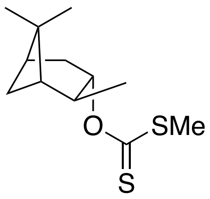

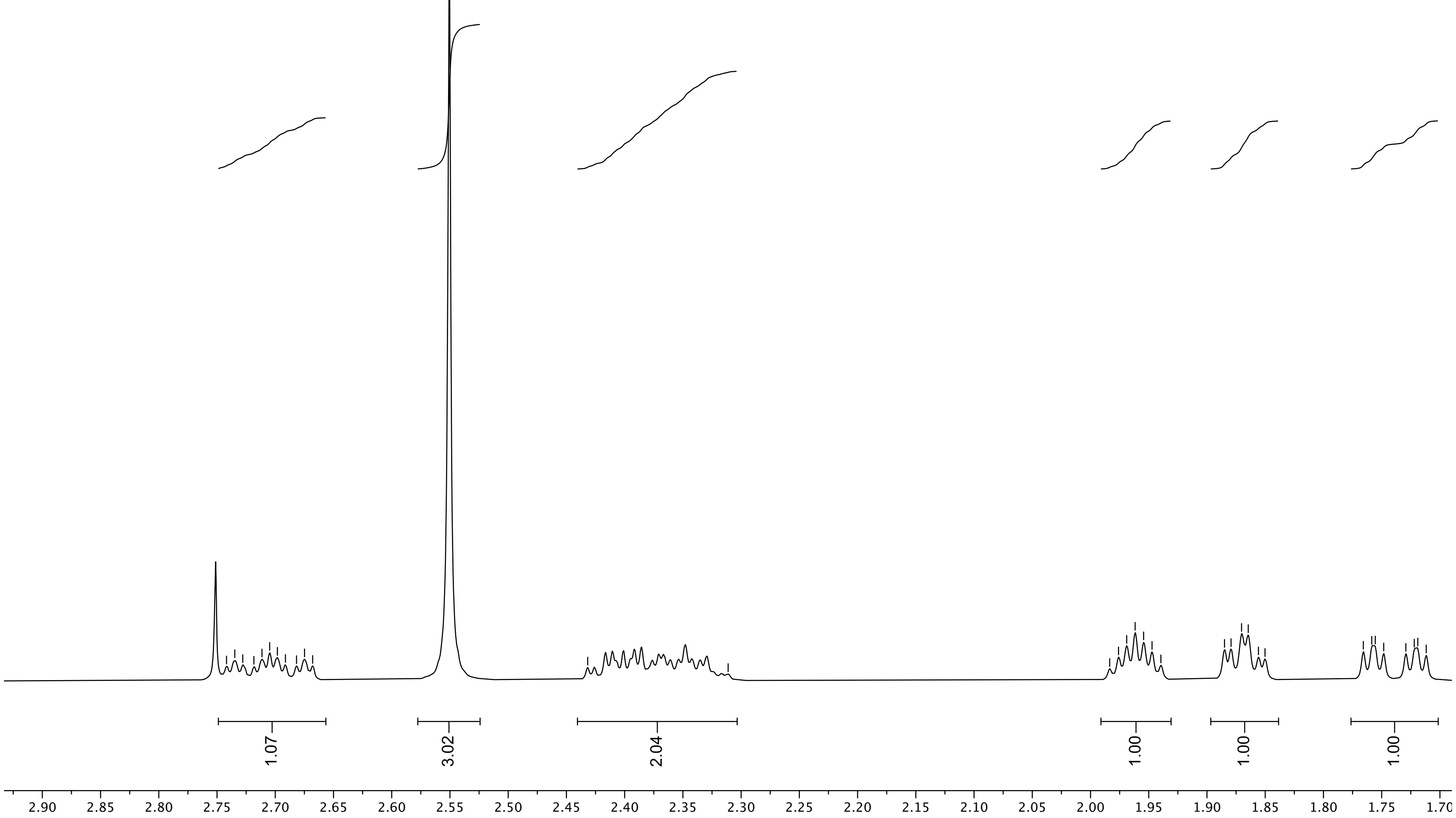

S-Methyl O-((1S,2S,3S,5R)-2,6,6-trimethylbicyclo[3.1.1]heptan-3-yl) carbonodithioate (57)

^{1}H-NMR (400 MHz, $CDCl_3$)

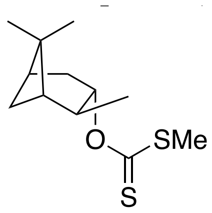

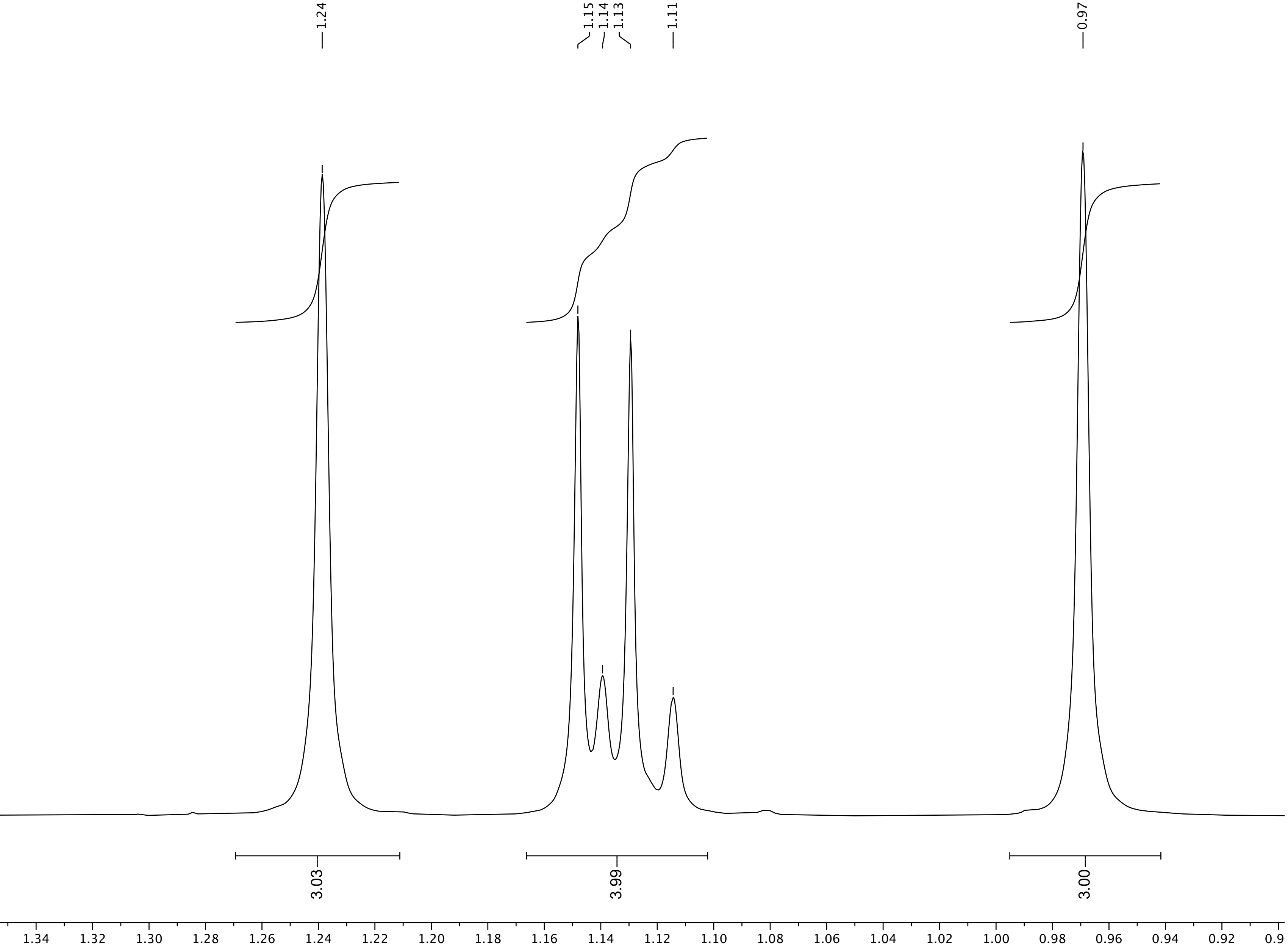

S-Methyl O-((1S,2S,3S,5R)-2,6,6-trimethylbicyclo[3.1.1]heptan-3-yl) carbonodithioate (57)

^{13}C-NMR (101 MHz, $CDCl_3$)

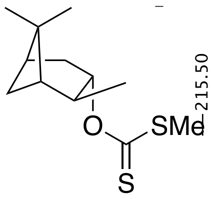

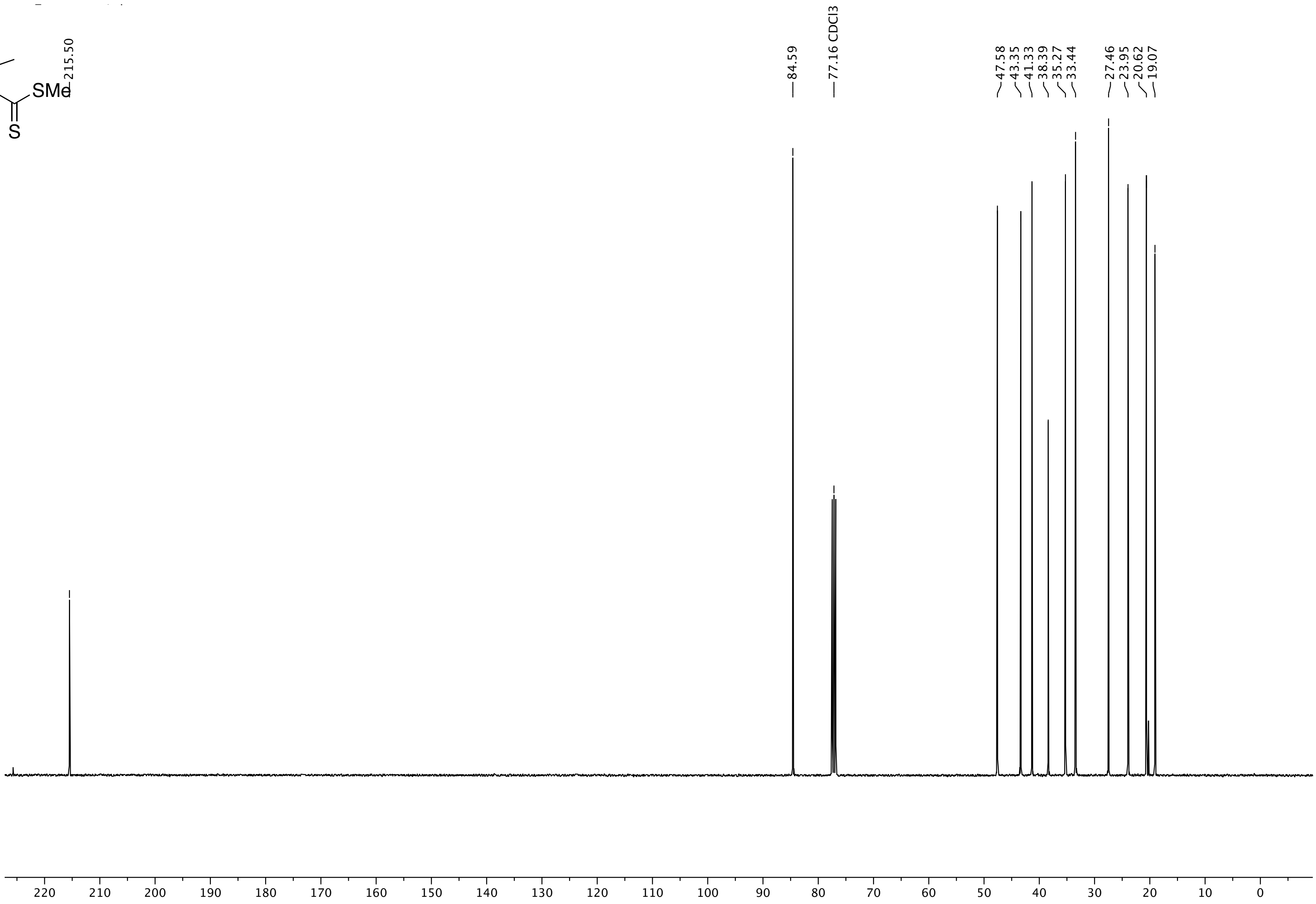

S-Methyl O-((1S,2S,3S,5R)-2,6,6-trimethylbicyclo[3.1.1]heptan-3-yl) carbonodithioate (57)

^{13}C-NMR (101 MHz, $CDCl_3$)

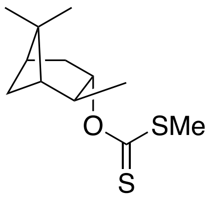

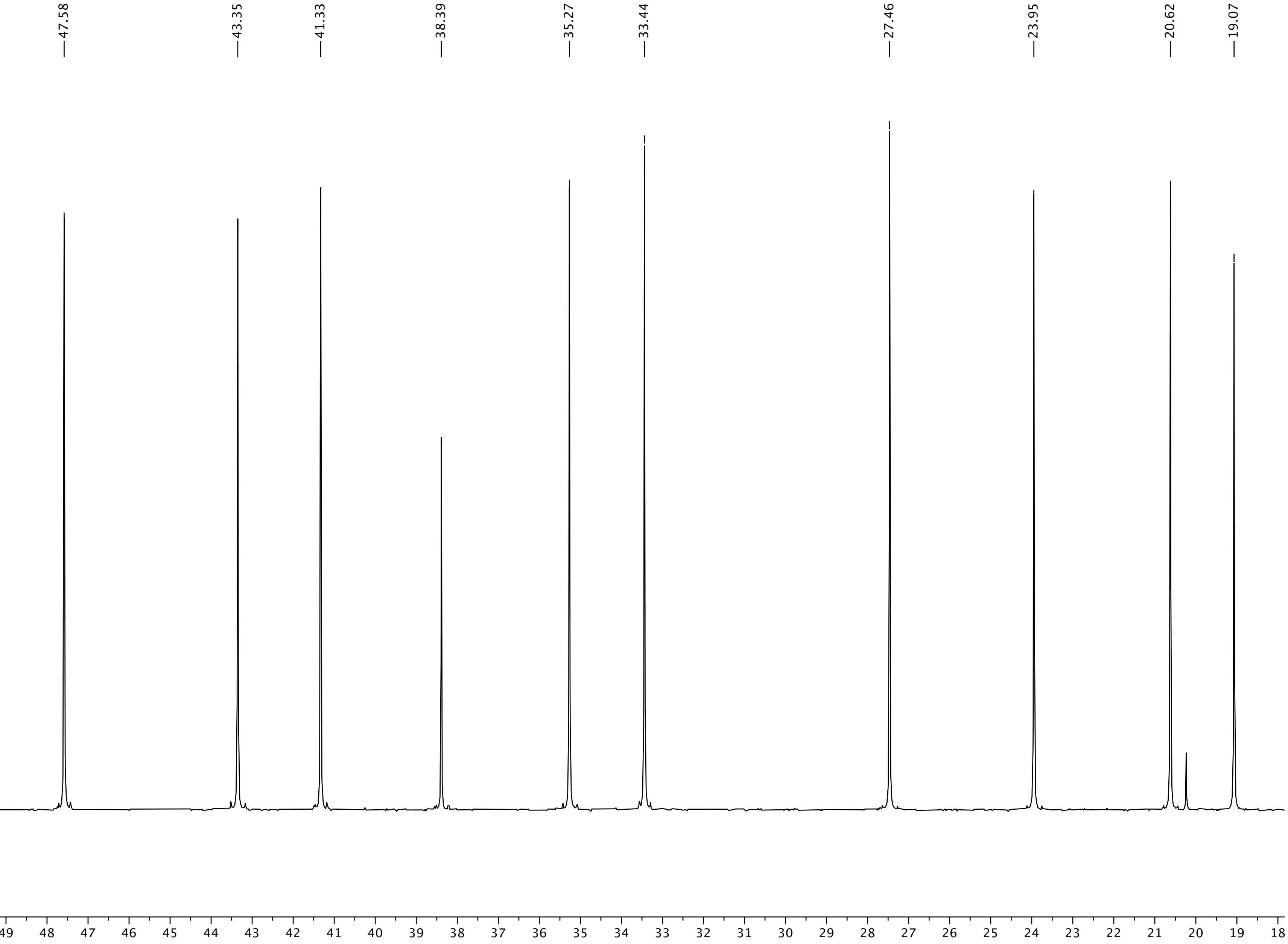

S-Methyl O-((1S,2S,3S,5R)-2,6,6-trimethylbicyclo[3.1.1]heptan-3-yl) carbonodithioate (57)

Dept-135 (101 MHz, $CDCl_3$)

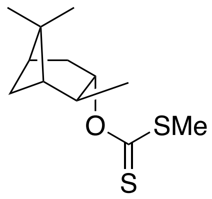

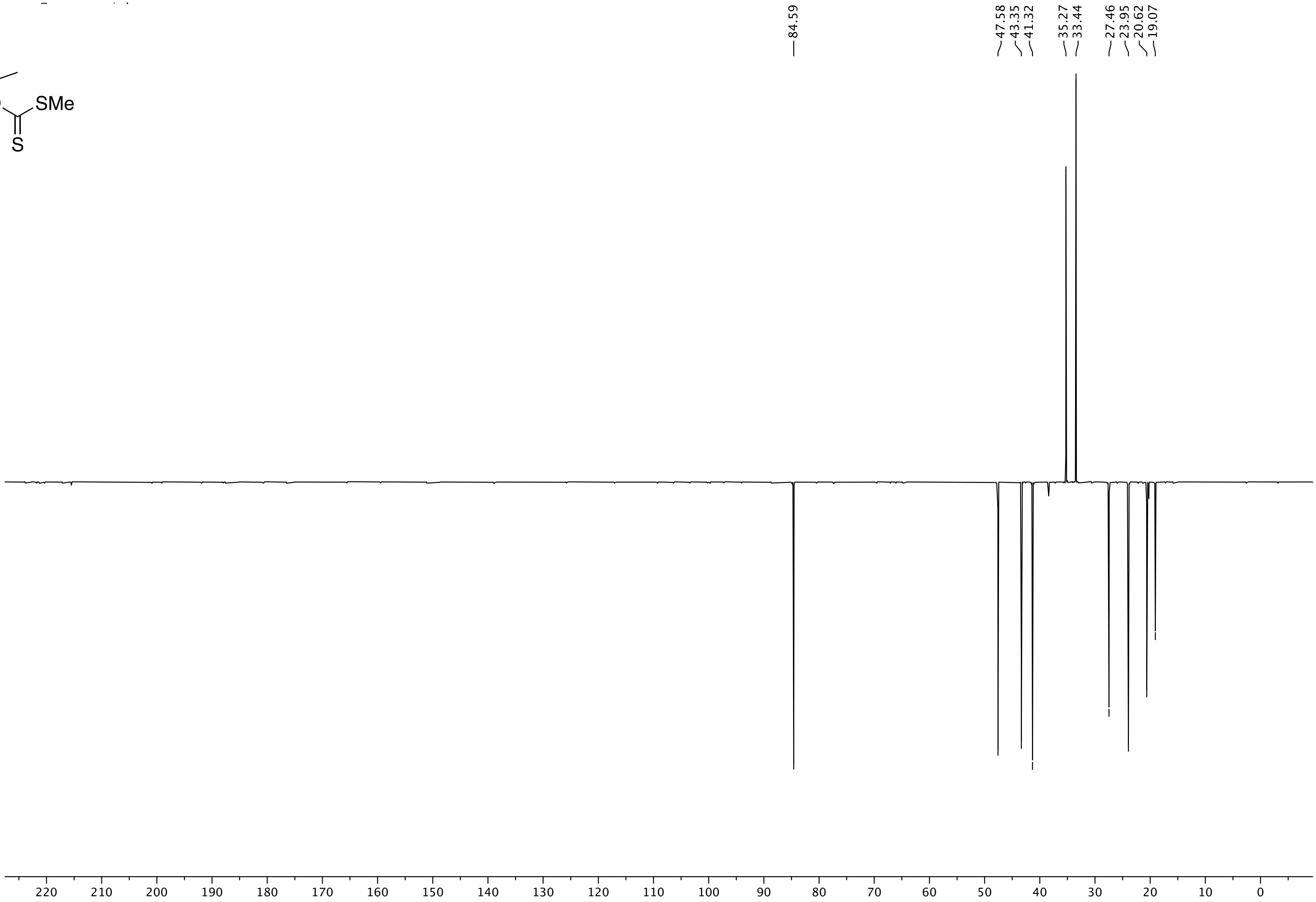

S-Methyl O-((1S,2S,3S,5R)-2,6,6-trimethylbicyclo[3.1.1]heptan-3-yl) carbonodithioate (57)

^{1}H-^{1}H COSY(400 MHz, $CDCl_3$)

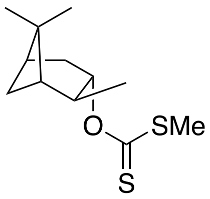

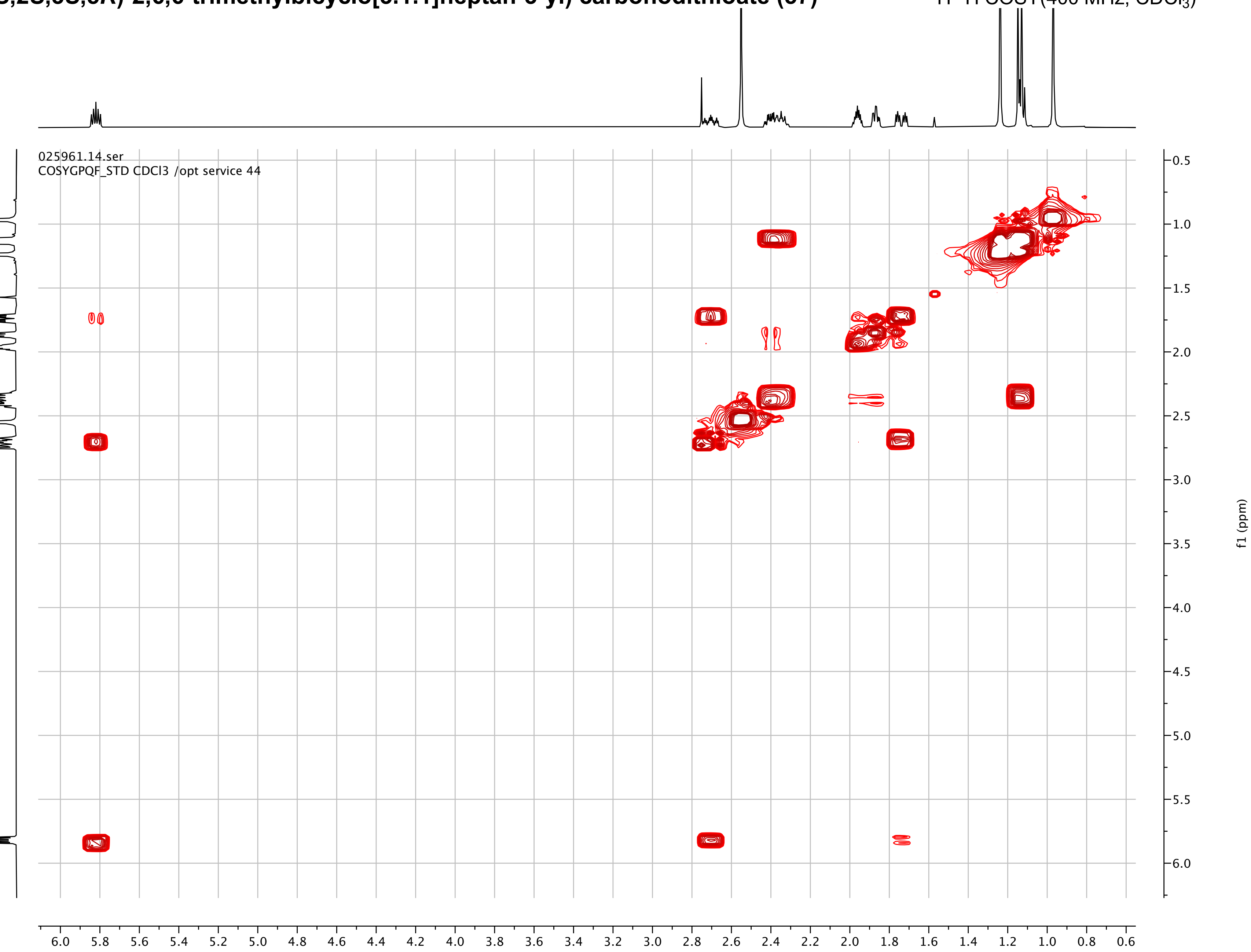

S-Methyl O-((1S,2S,3S,5R)-2,6,6-trimethylbicyclo[3.1.1]heptan-3-yl) carbonodithioate (57)

HSQC (400 MHz, $CDCl_3$)

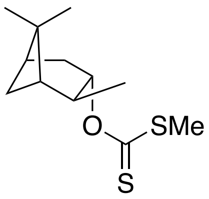

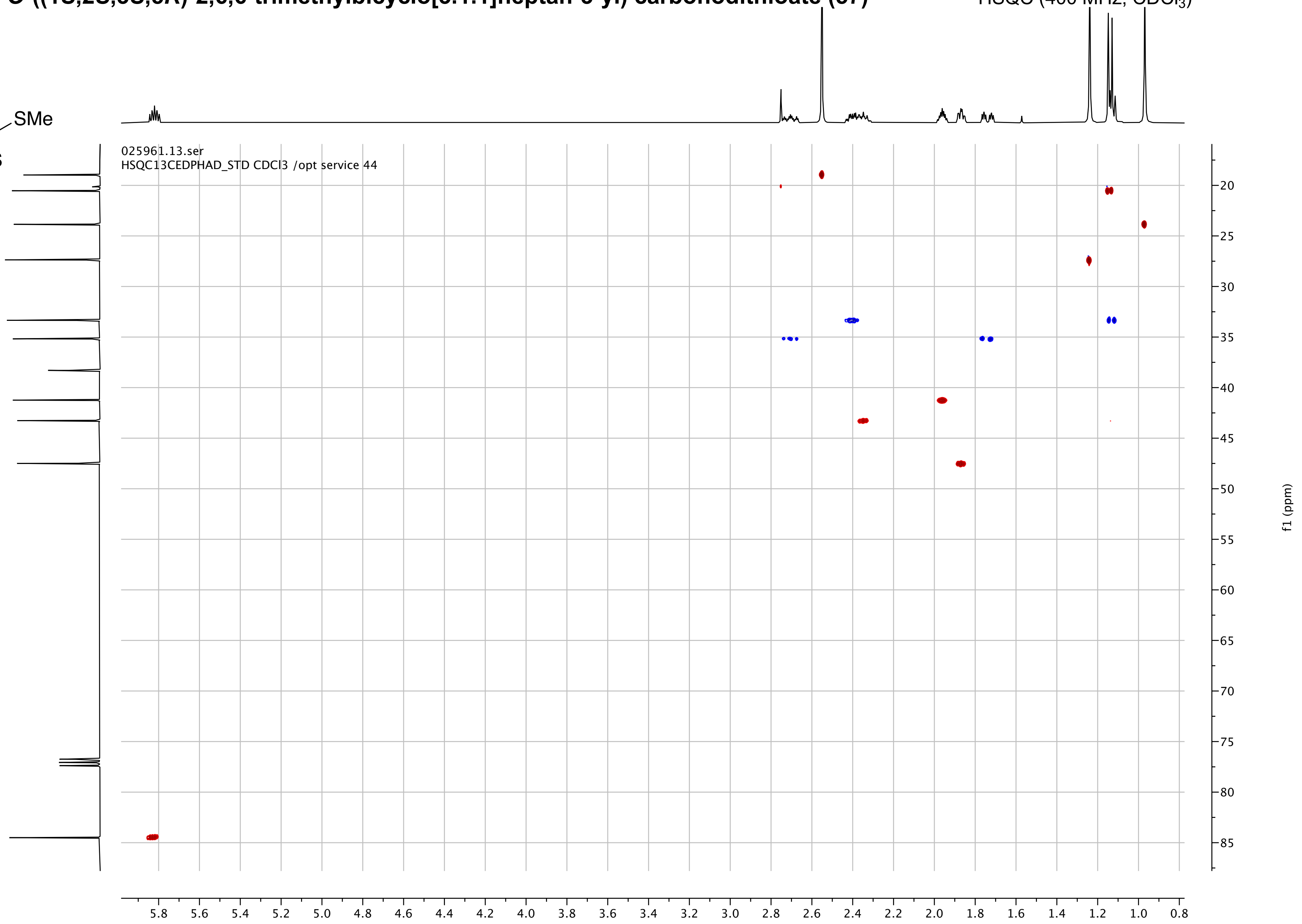

S-Methyl O-((1S,2S,3S,5R)-2,6,6-trimethylbicyclo[3.1.1]heptan-3-yl) carbonodithioate (57)

HSQC (400 MHz, $CDCl_3$)

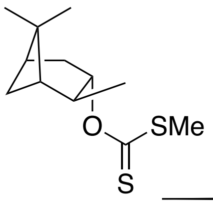

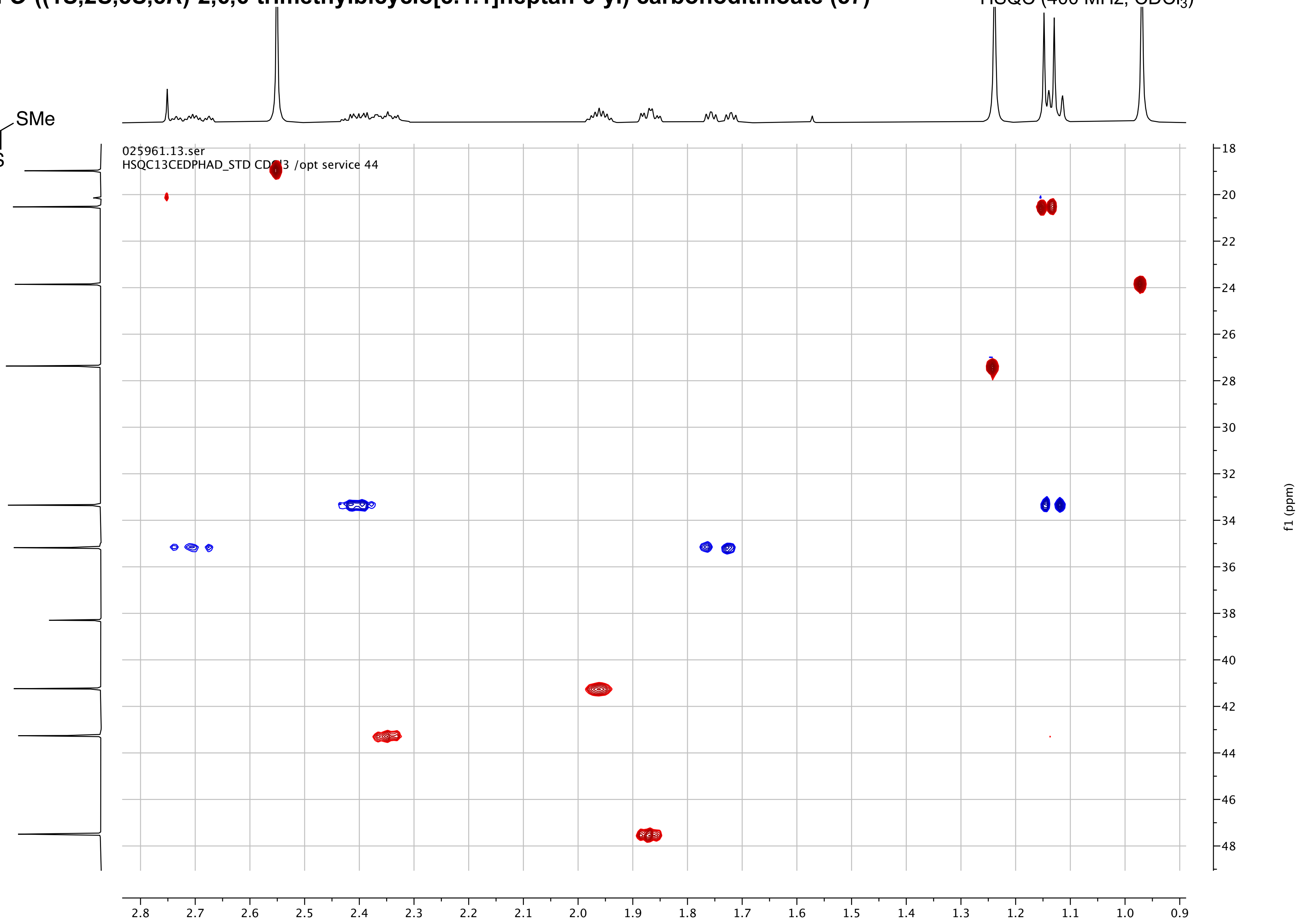

_S_-Methyl _S_-((1_S_,2_S_,3_S_,5_R_)-2,6,6-trimethylbicyclo[3.1.1]heptan-3-yl) carbonodithioate (58)

^{1}H-NMR (400 MHz, $CDCl_3$)

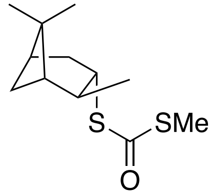

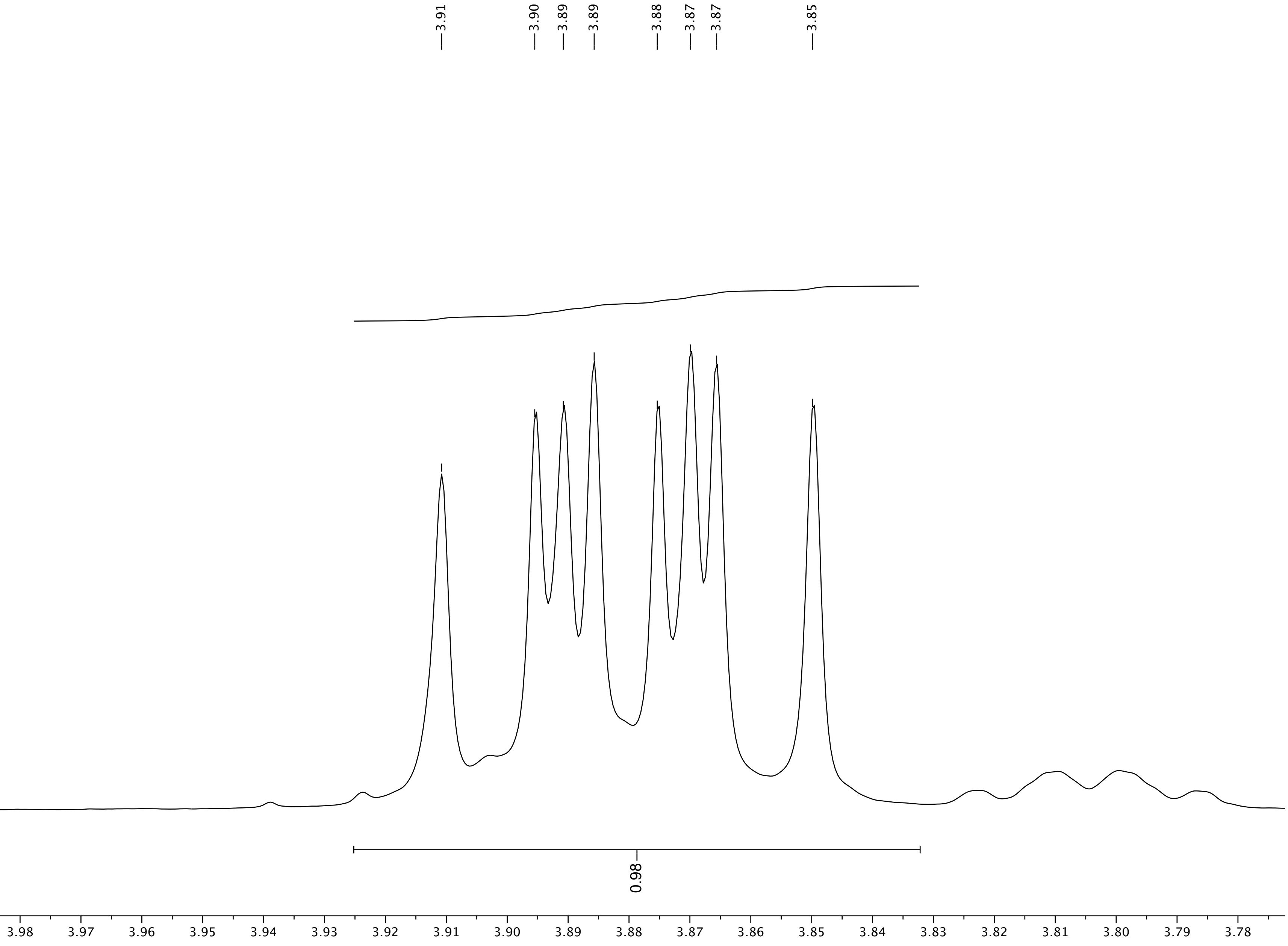

S-Methyl S-((1S,2S,3S,5R)-2,6,6-trimethylbicyclo[3.1.1]heptan-3-yl) carbonodithioate (58)

^{1}H-NMR (400 MHz, $CDCl_3$)

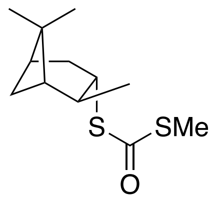

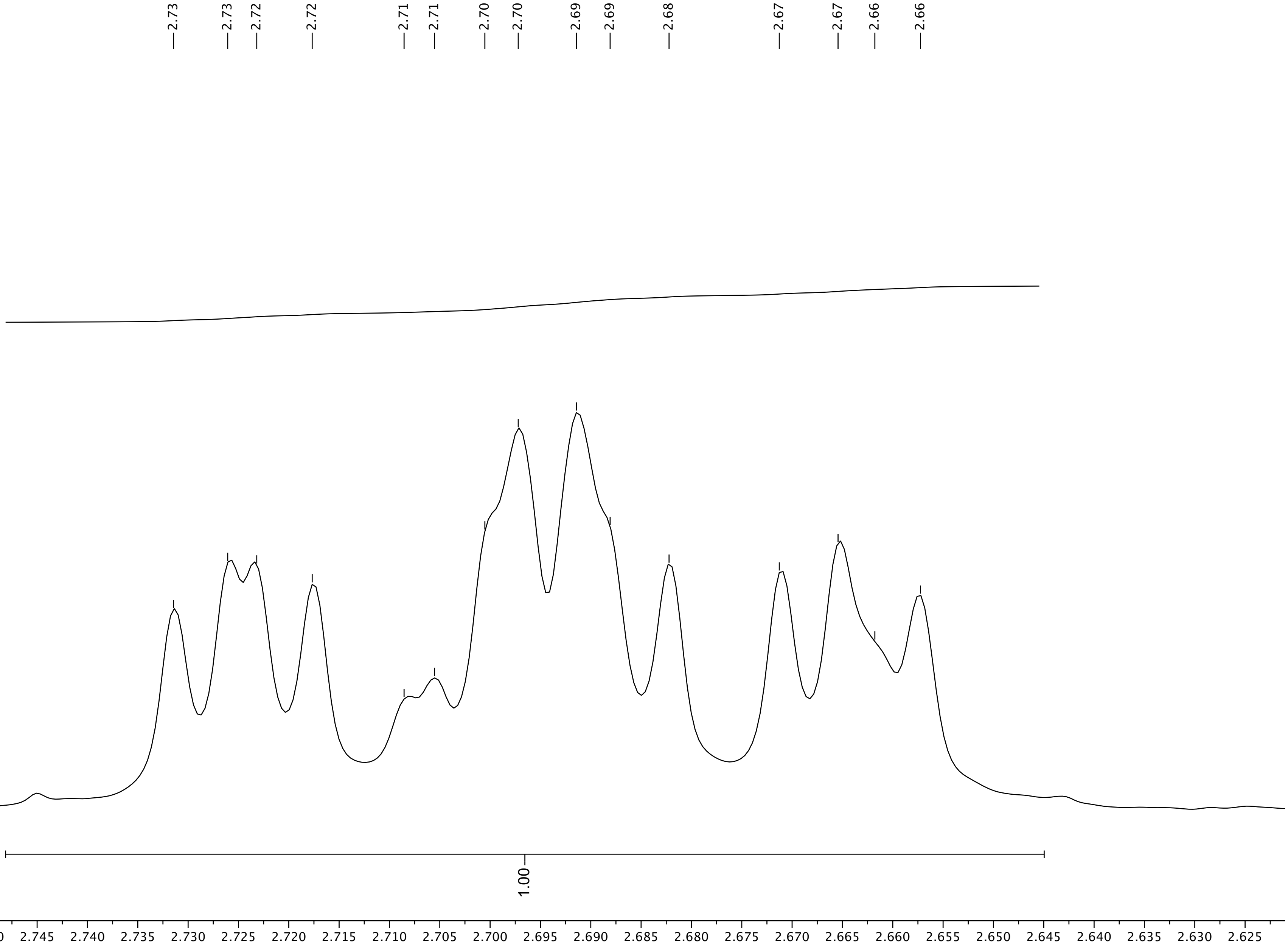

S-Methyl S-((1S,2S,3S,5R)-2,6,6-trimethylbicyclo[3.1.1]heptan-3-yl) carbonodithioate (58)

^{1}H-NMR (400 MHz, $CDCl_3$)

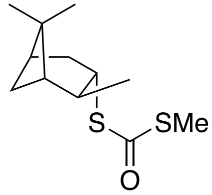

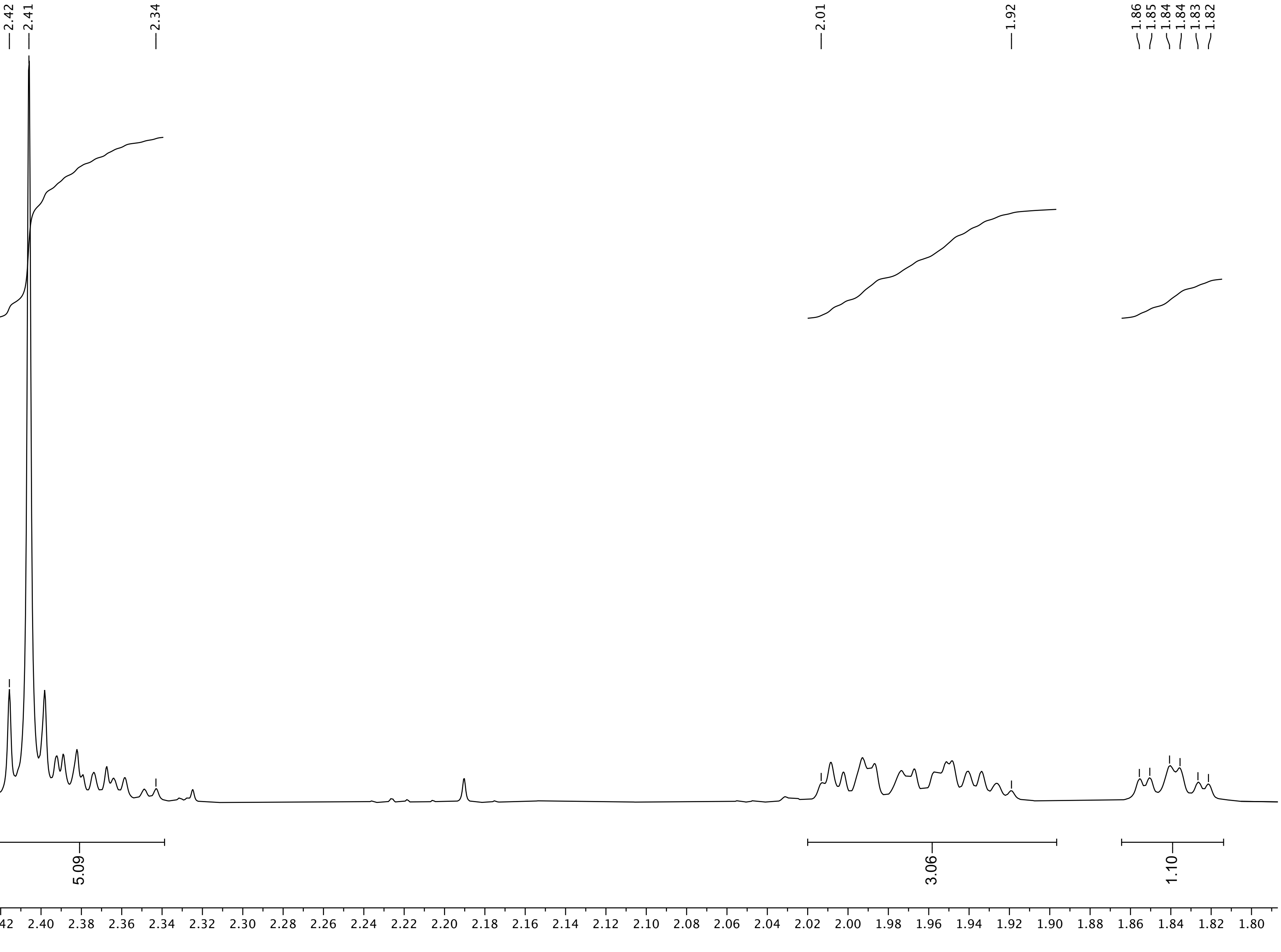

S-Methyl S-((1S,2S,3S,5R)-2,6,6-trimethylbicyclo[3.1.1]heptan-3-yl) carbonodithioate (58)

^{1}H-NMR (400 MHz, $CDCl_3$)

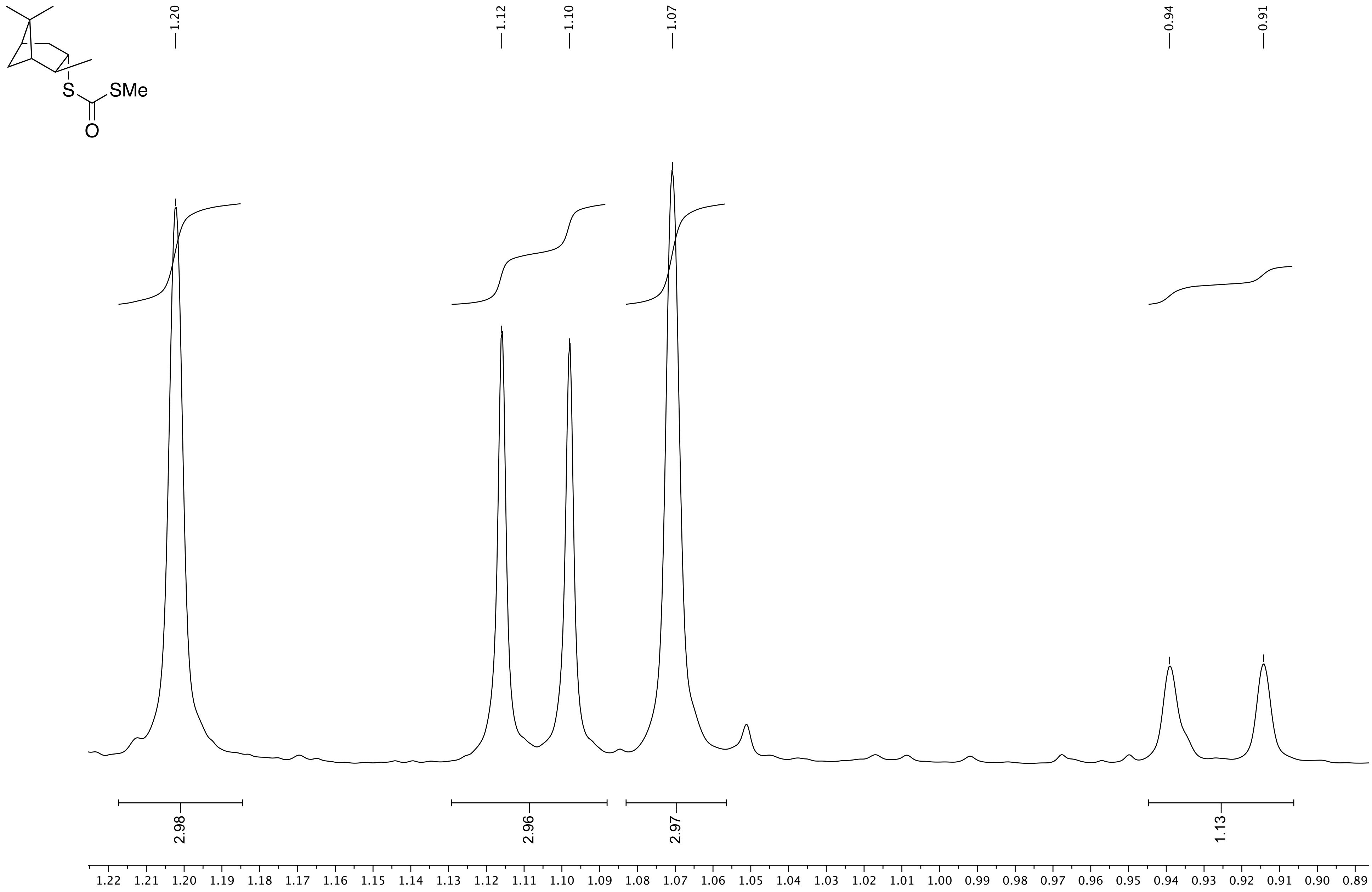

S-Methyl S-((1S,2S,3S,5R)-2,6,6-trimethylbicyclo[3.1.1]heptan-3-yl) carbonodithioate (58)

^{13}C-NMR (101 MHz, $CDCl_3$)

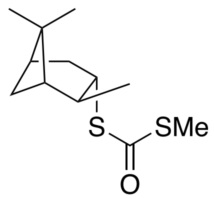

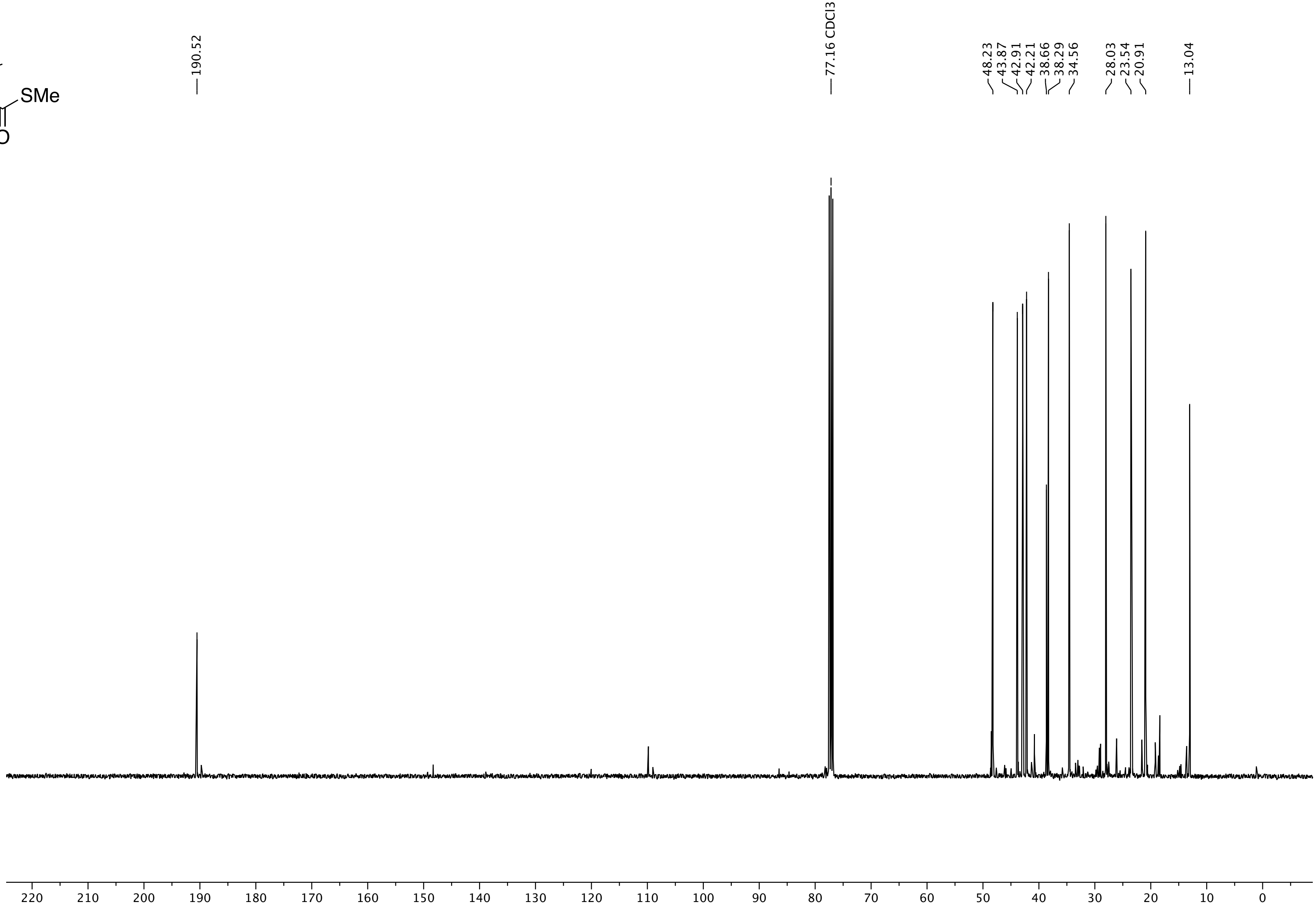

S-Methyl S-((1S,2S,3S,5R)-2,6,6-trimethylbicyclo[3.1.1]heptan-3-yl) carbonodithioate (58)

^{13}C-NMR (101 MHz, $CDCl_3$)

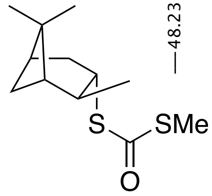

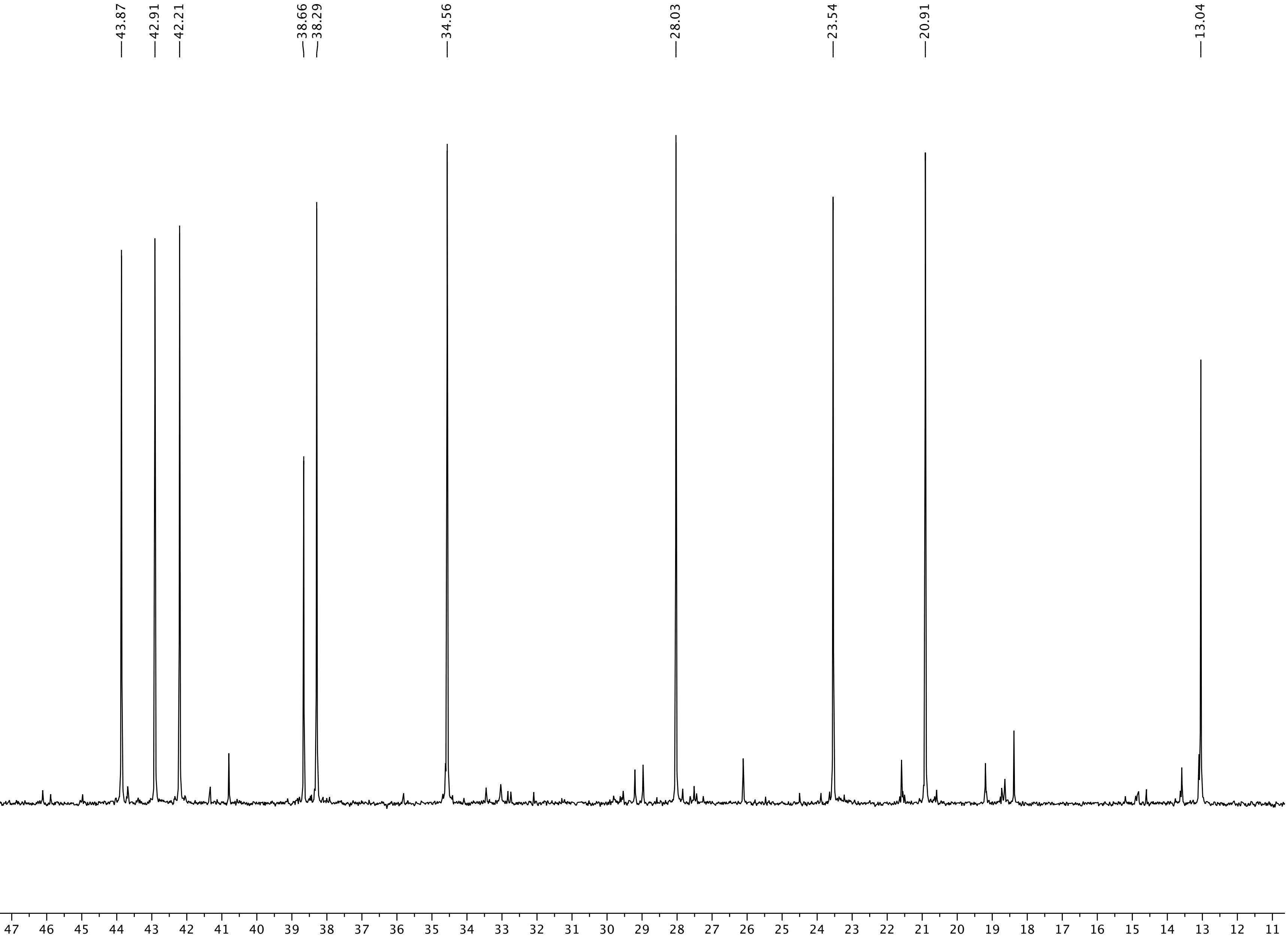

S-Methyl S-((1S,2S,3S,5R)-2,6,6-trimethylbicyclo[3.1.1]heptan-3-yl) carbonodithioate (58)

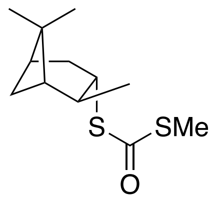

Dept-135 (101 MHz, $CDCl_3$)

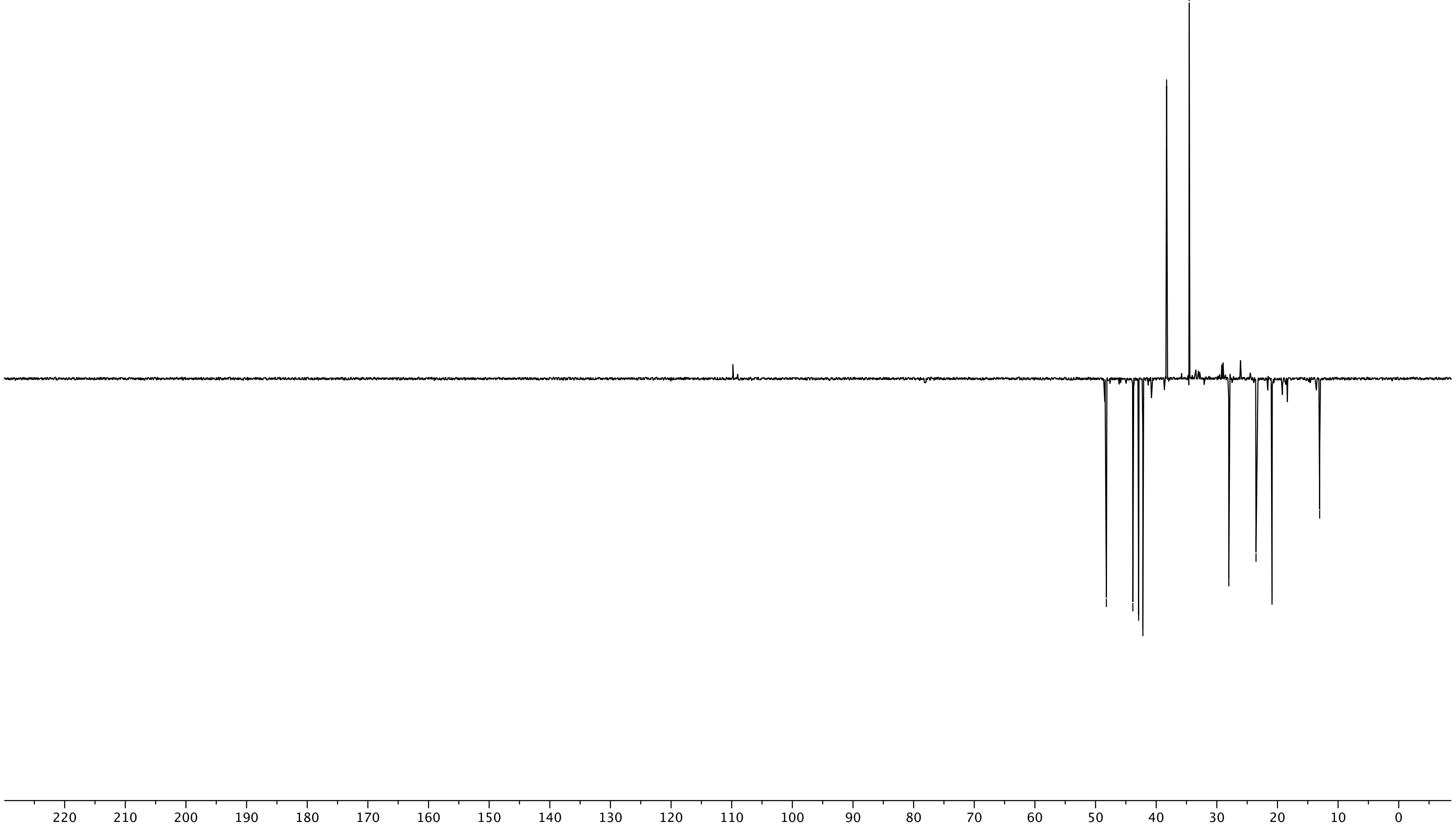

_S_-Methyl _S_-((1_S_,2_S_,3_S_,5_R_)-2,6,6-trimethylbicyclo[3.1.1]heptan-3-yl) carbonodithioate (58)

Dept-135 (101 MHz, $CDCl_3$)

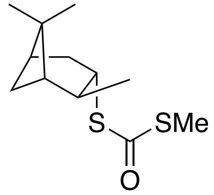

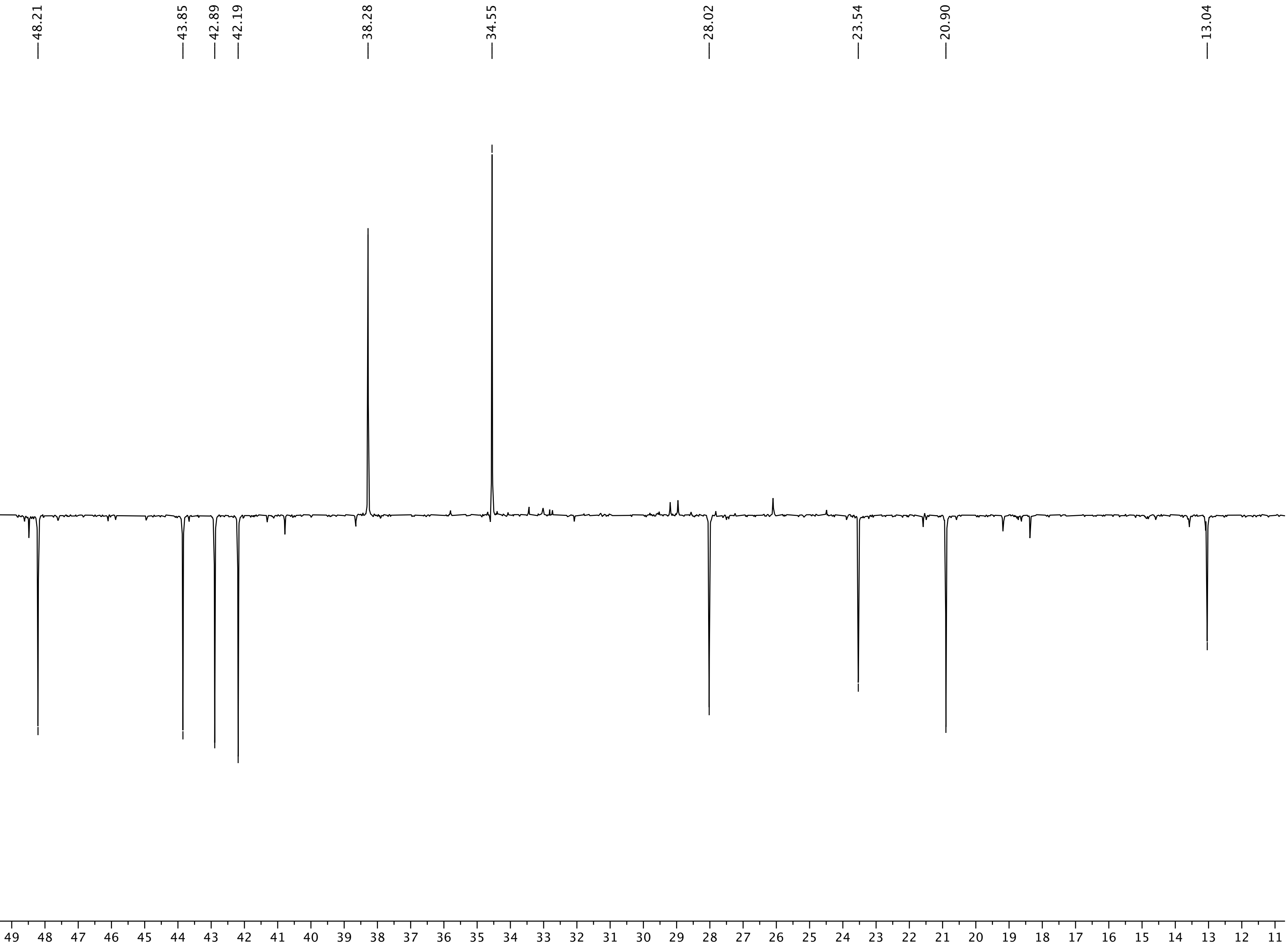

S-Methyl S-((1S,2S,3S,5R)-2,6,6-trimethylbicyclo[3.1.1]heptan-3-yl) carbonodithioate (58)

^{1}H-^{1}H COSY(400 MHz, $CDCl_3$)

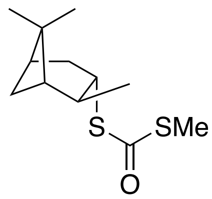

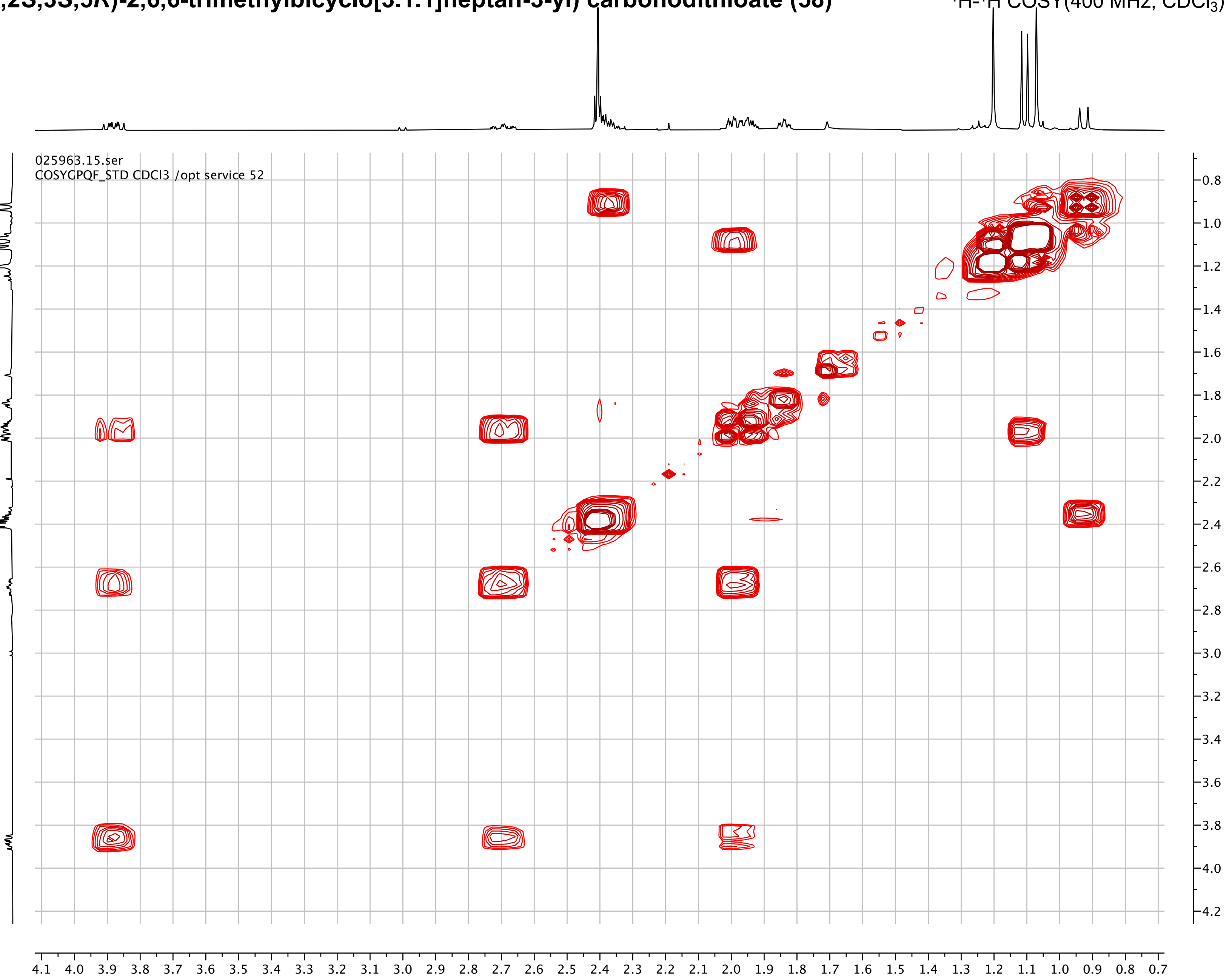

_S_-Methyl _S_-((1_S_,2_S_,3_S_,5_R_)-2,6,6-trimethylbicyclo[3.1.1]heptan-3-yl) carbonodithioate (58)

HSQC (400 MHz, $CDCl_3$)

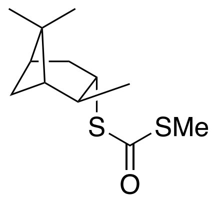

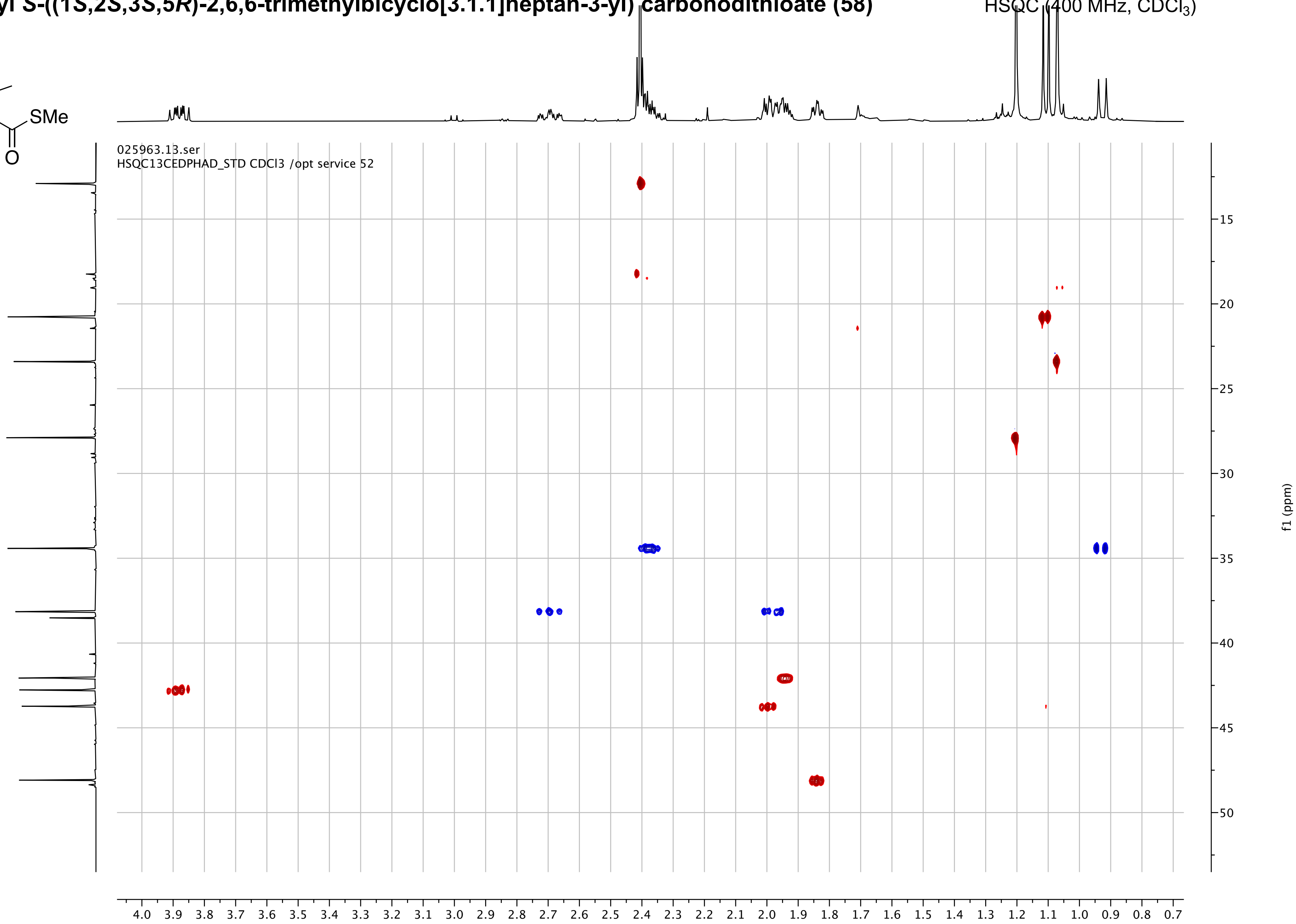

S-Methyl S-((1S,2S,3S,5R)-2,6,6-trimethylbicyclo[3.1.1]heptan-3-yl) carbonodithioate (58)

HMBC (400 MHz, $CDCl_3$)

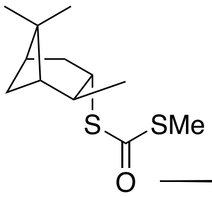

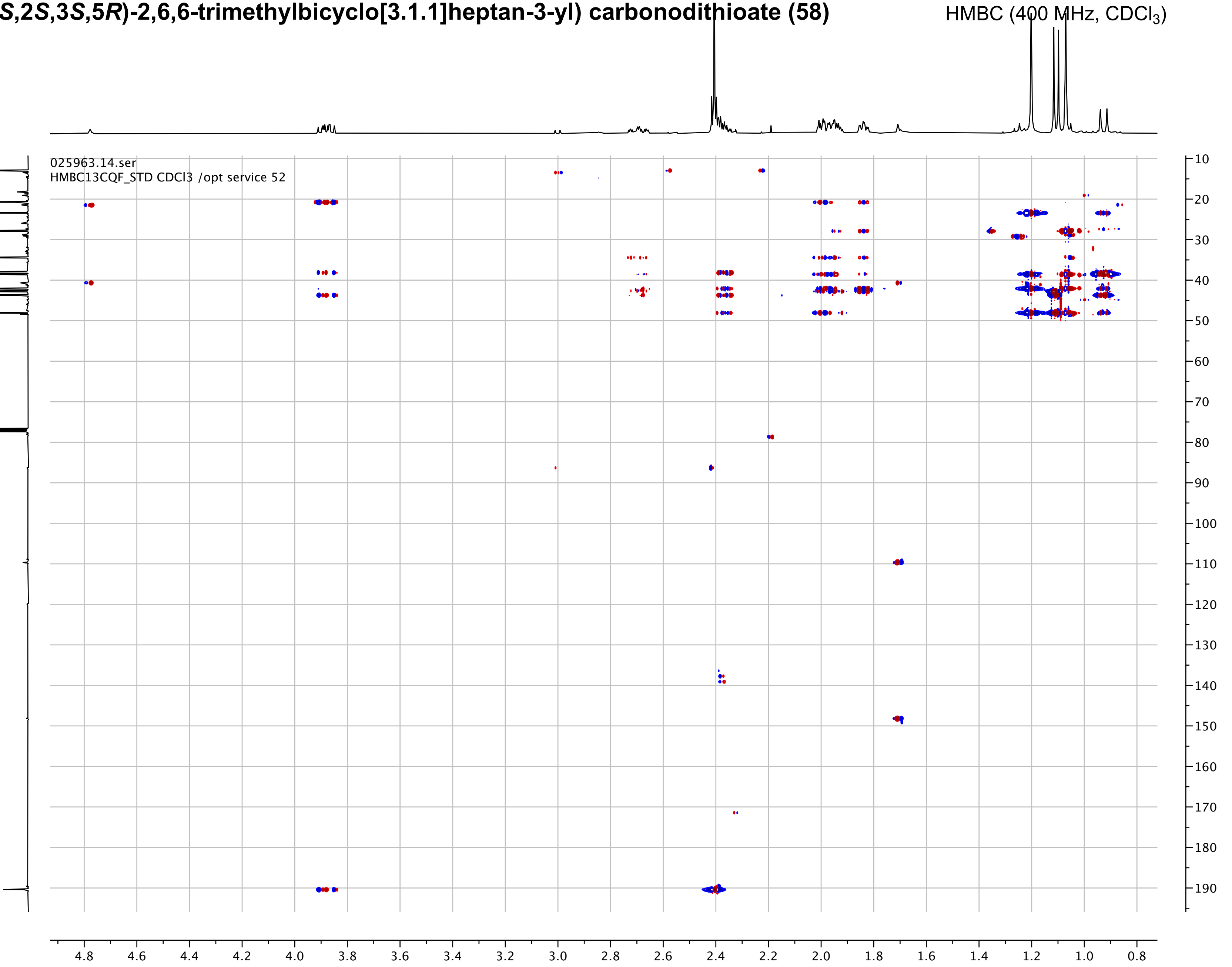

S-Methyl S-((1S,2S,3S,5R)-2,6,6-trimethylbicyclo[3.1.1]heptan-3-yl) carbonodithioate (58)

HMBC (400 MHz, $CDCl_3$)

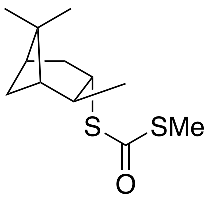

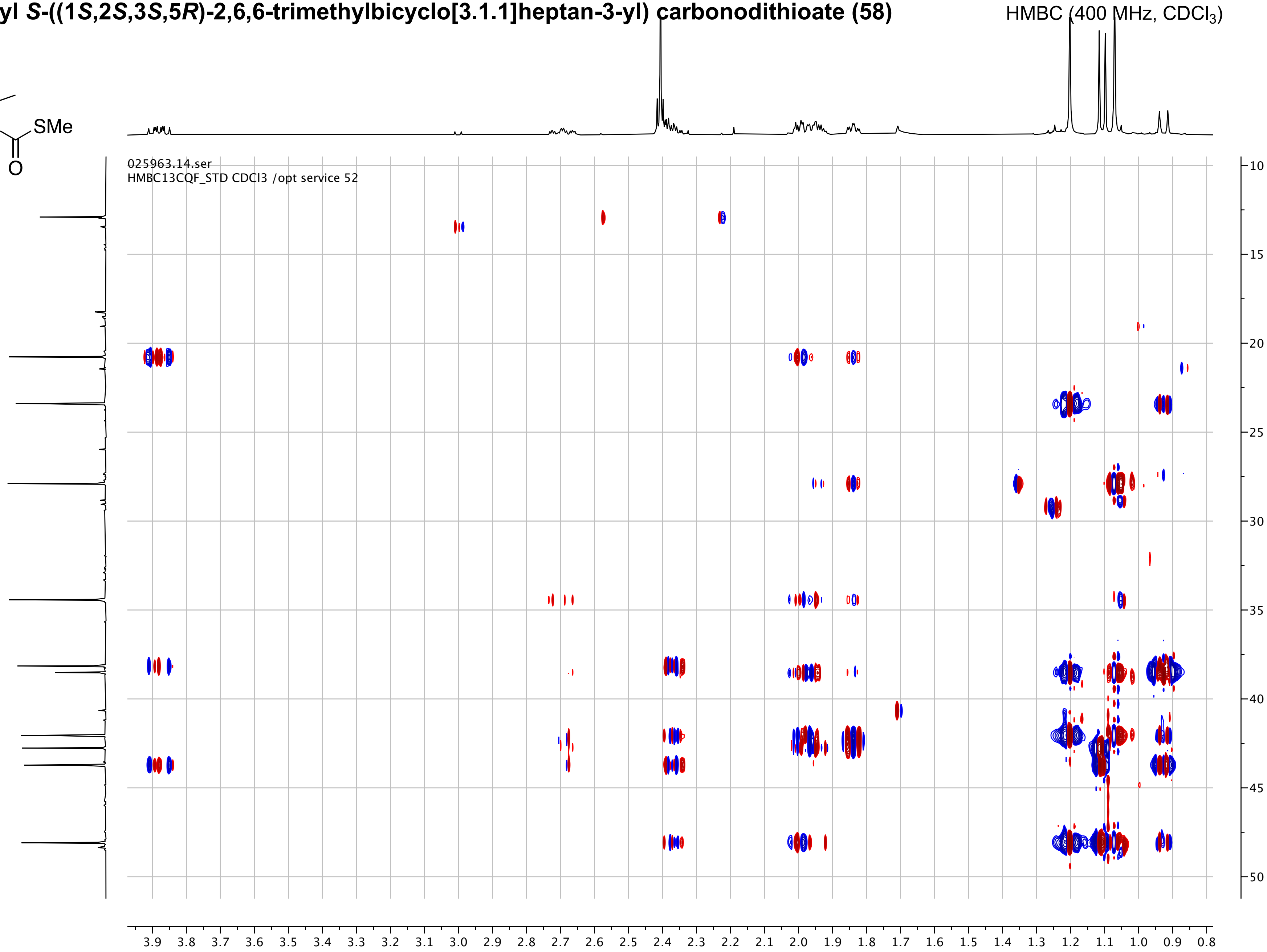

Ethyl 4-(4-methylquinolin-2-yl)undecanoate (59)

^{1}H-NMR, 300 MHz, $CDCl_3$

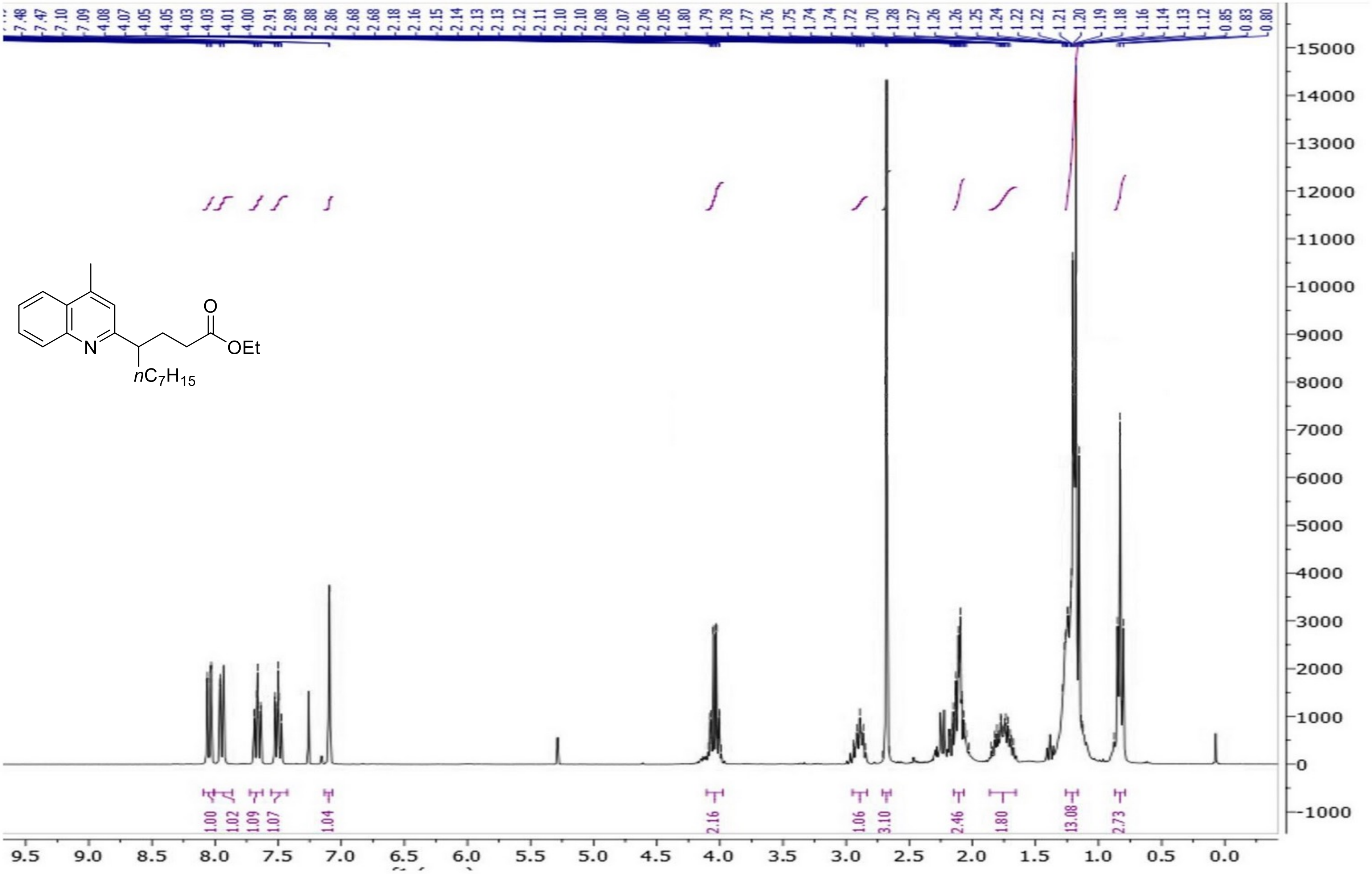

Ethyl 4-(4-methylquinolin-2-yl)undecanoate (59)

^{13}C-NMR, 75 MHz, $CDCl_3$

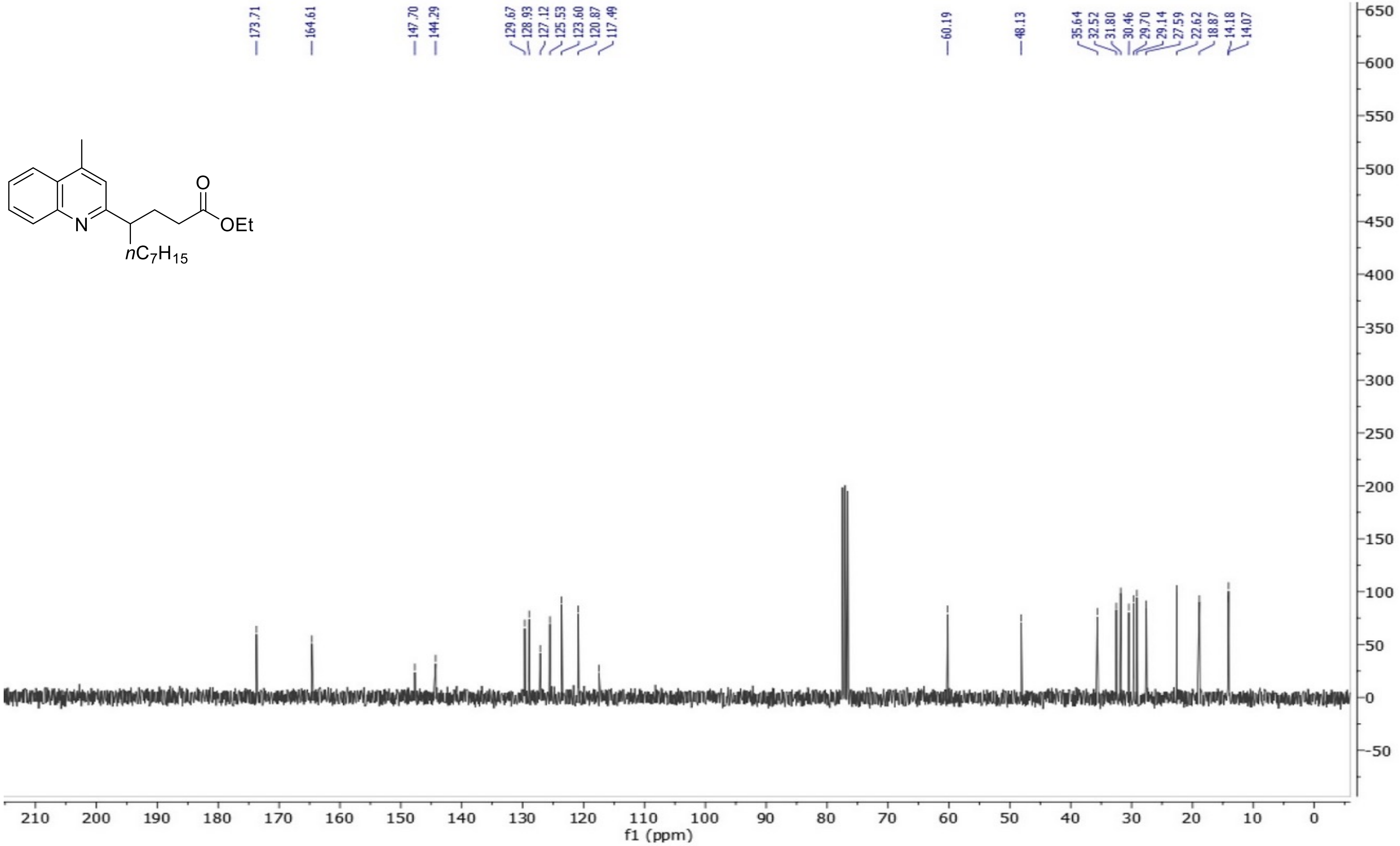

Ethyl 4-(4-chloropyridin-2-yl)undecanoate (60)

^{1}H-NMR, 300 MHz, $CDCl_3$

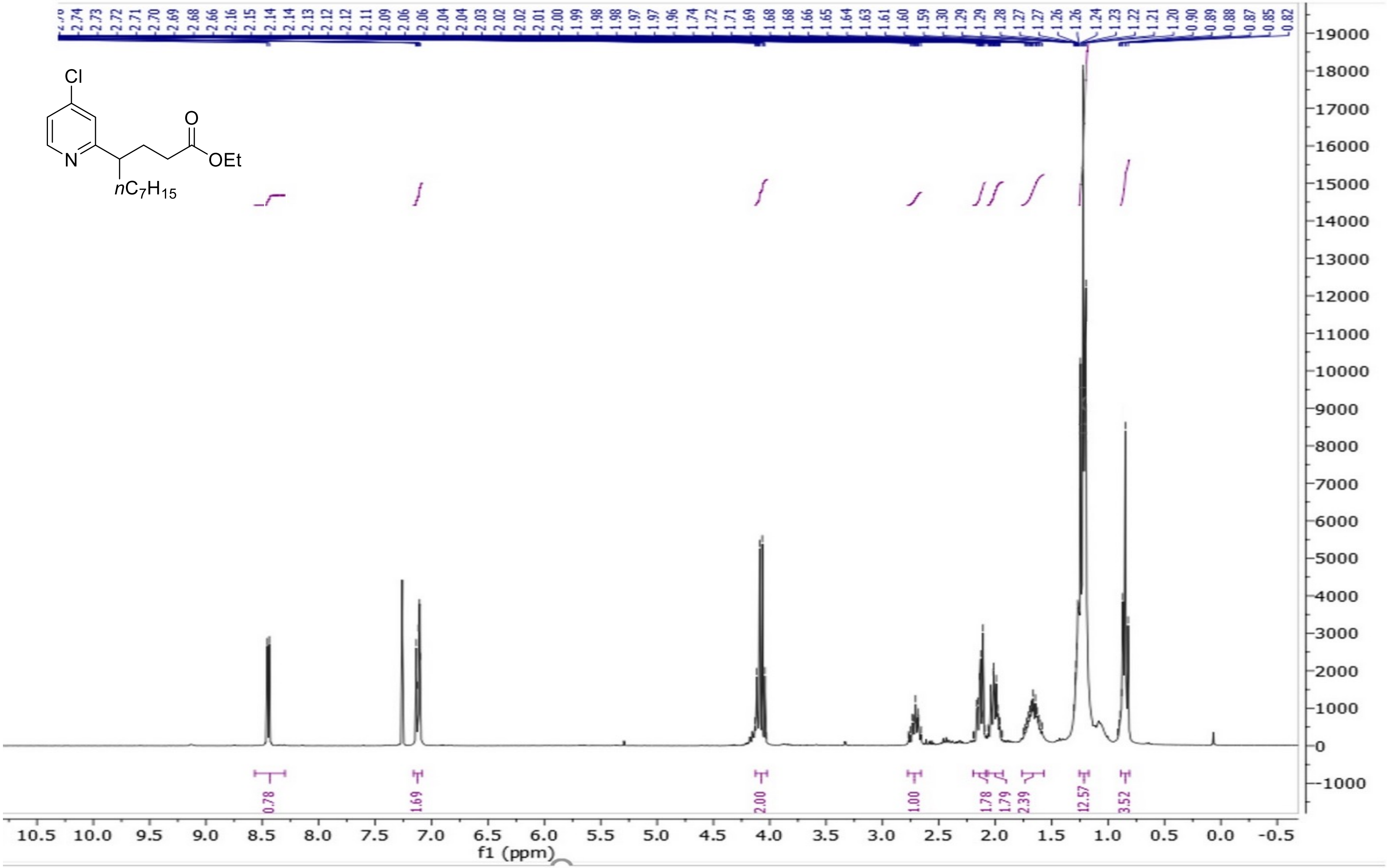

Ethyl 4-(4-chloropyridin-2-yl)undecanoate (60)

^{13}C-NMR, 75 MHz, $CDCl_3$

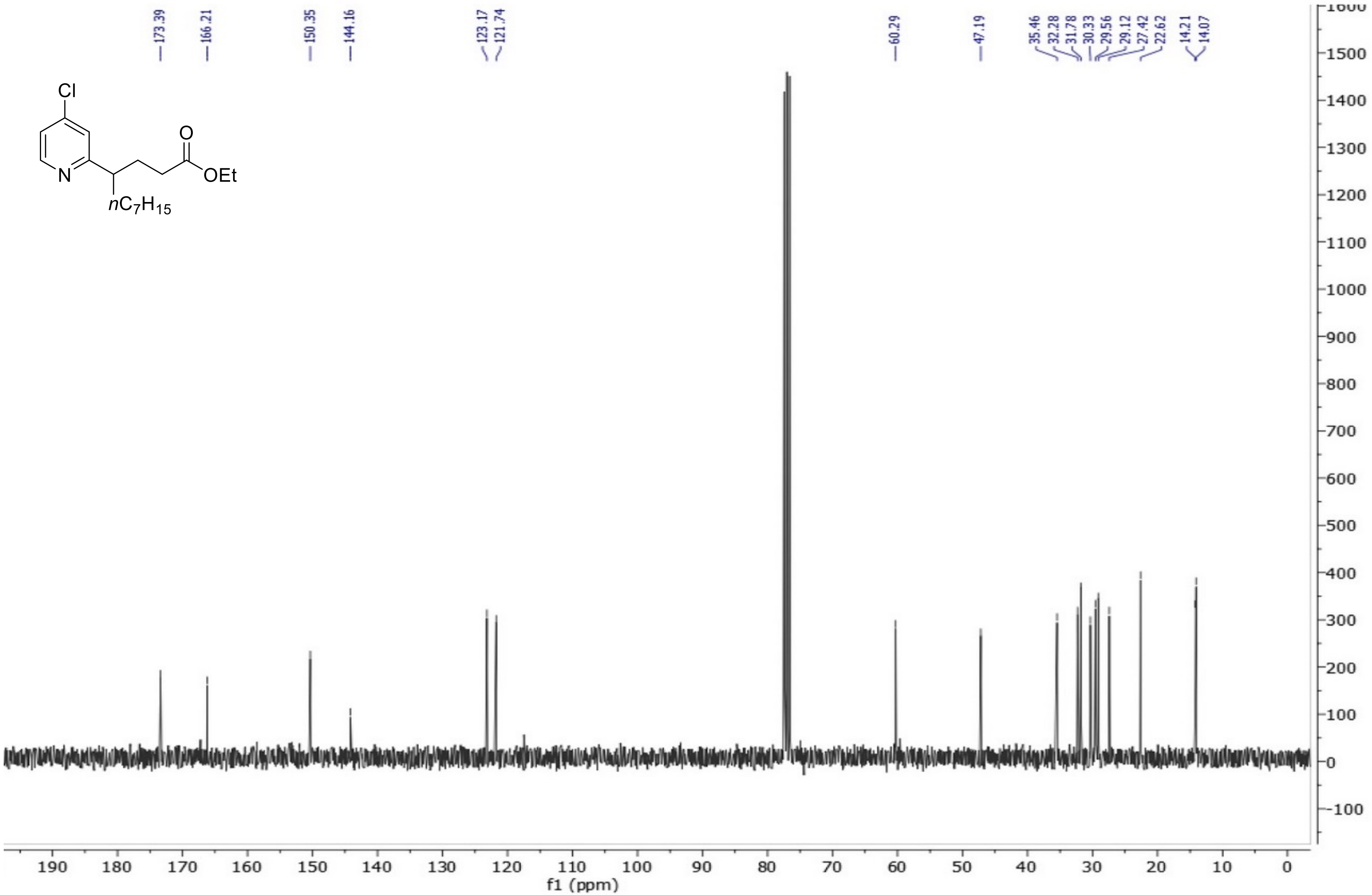

Ethyl 4-(phenanthridin-6-yl)undecanoate (61)

^{1}H-NMR, 300 MHz, $CDCl_3$

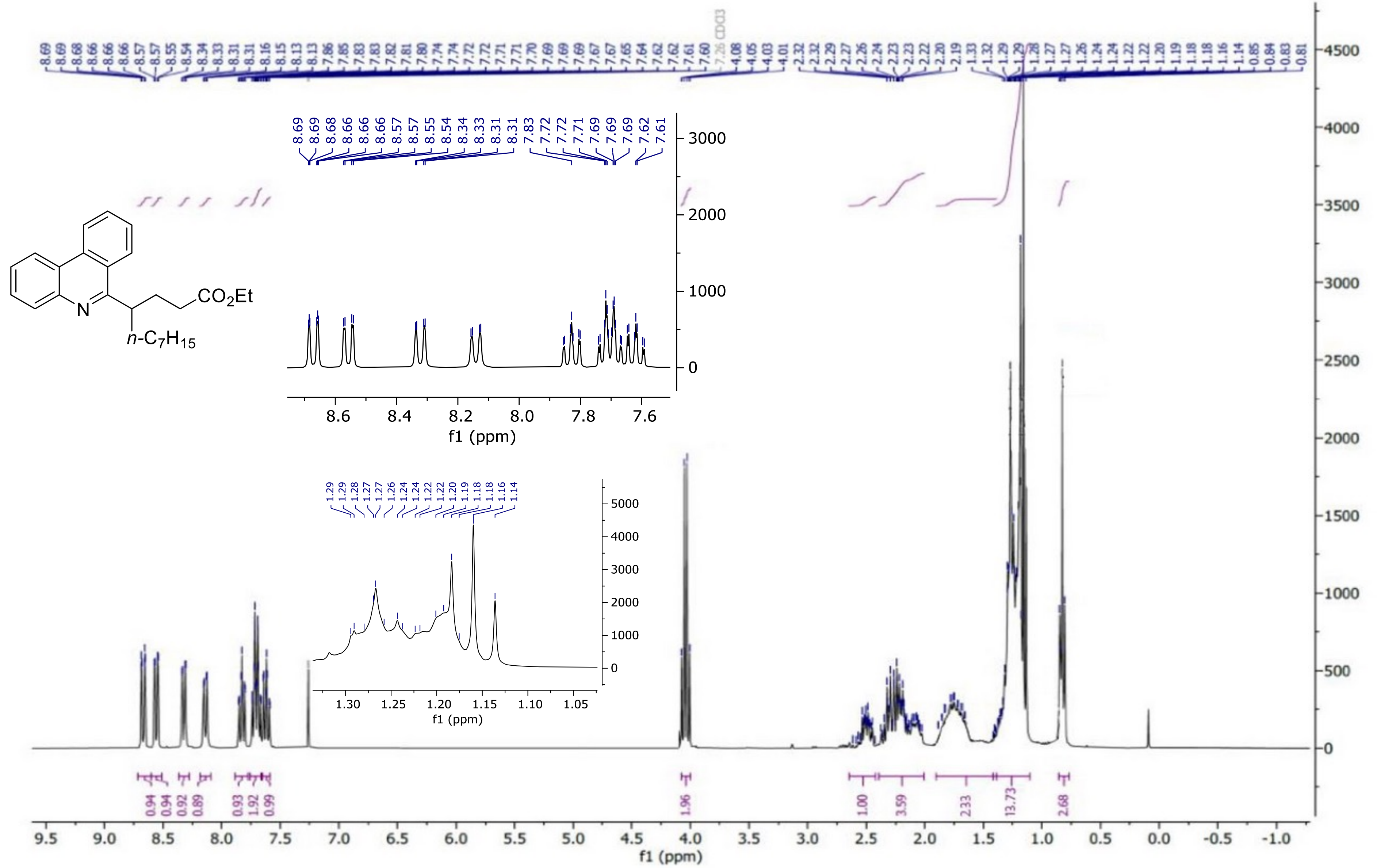

Ethyl 4-(phenanthridin-6-yl)undecanoate (61)

^{13}C-NMR, 75 MHz, $CDCl_3$

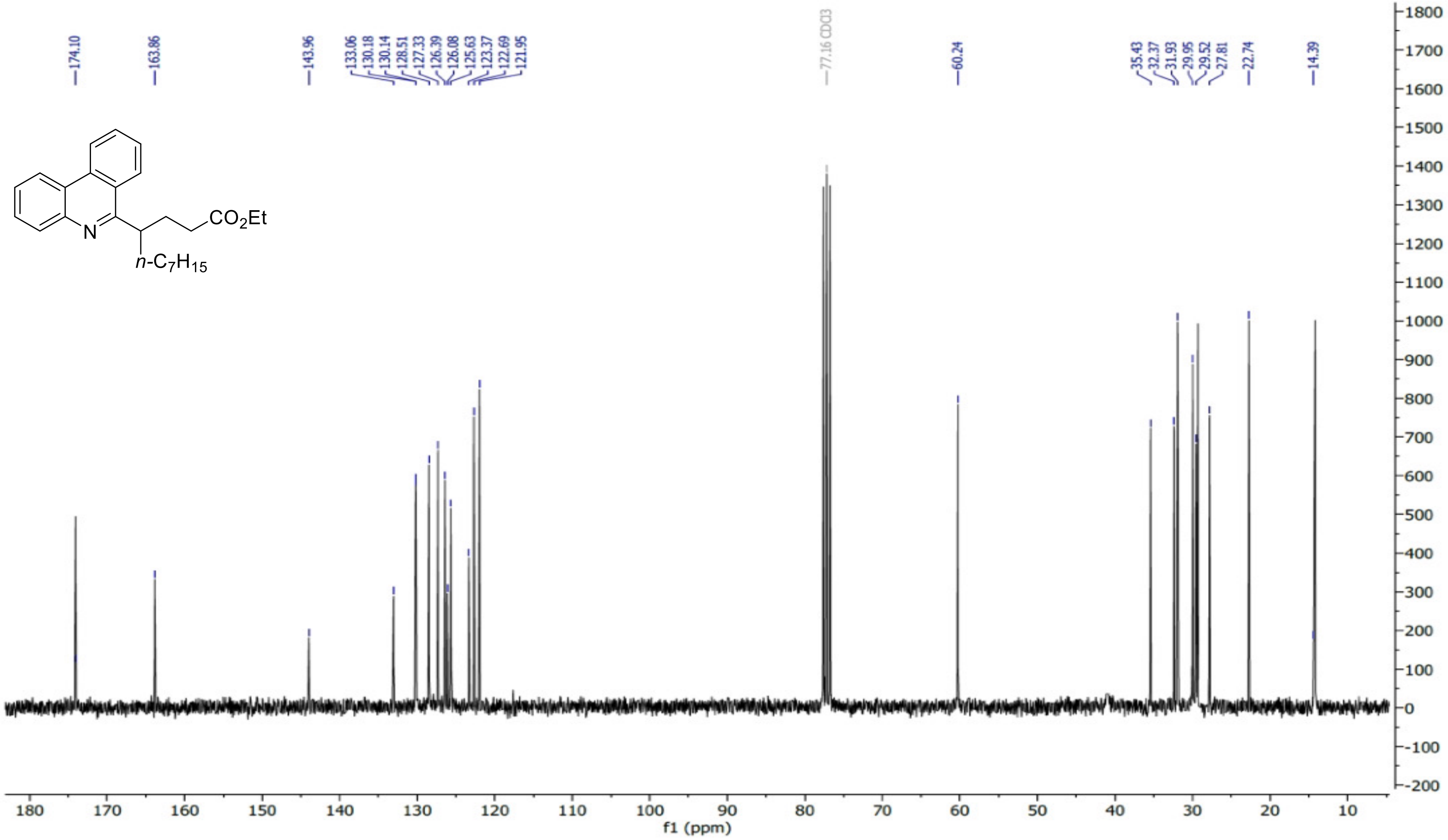

Ethyl 4-(4-chloropyridin-2-yl)-5-hydroxypentanoate (62)

^{1}H-NMR, 300 MHz, $CDCl_3$

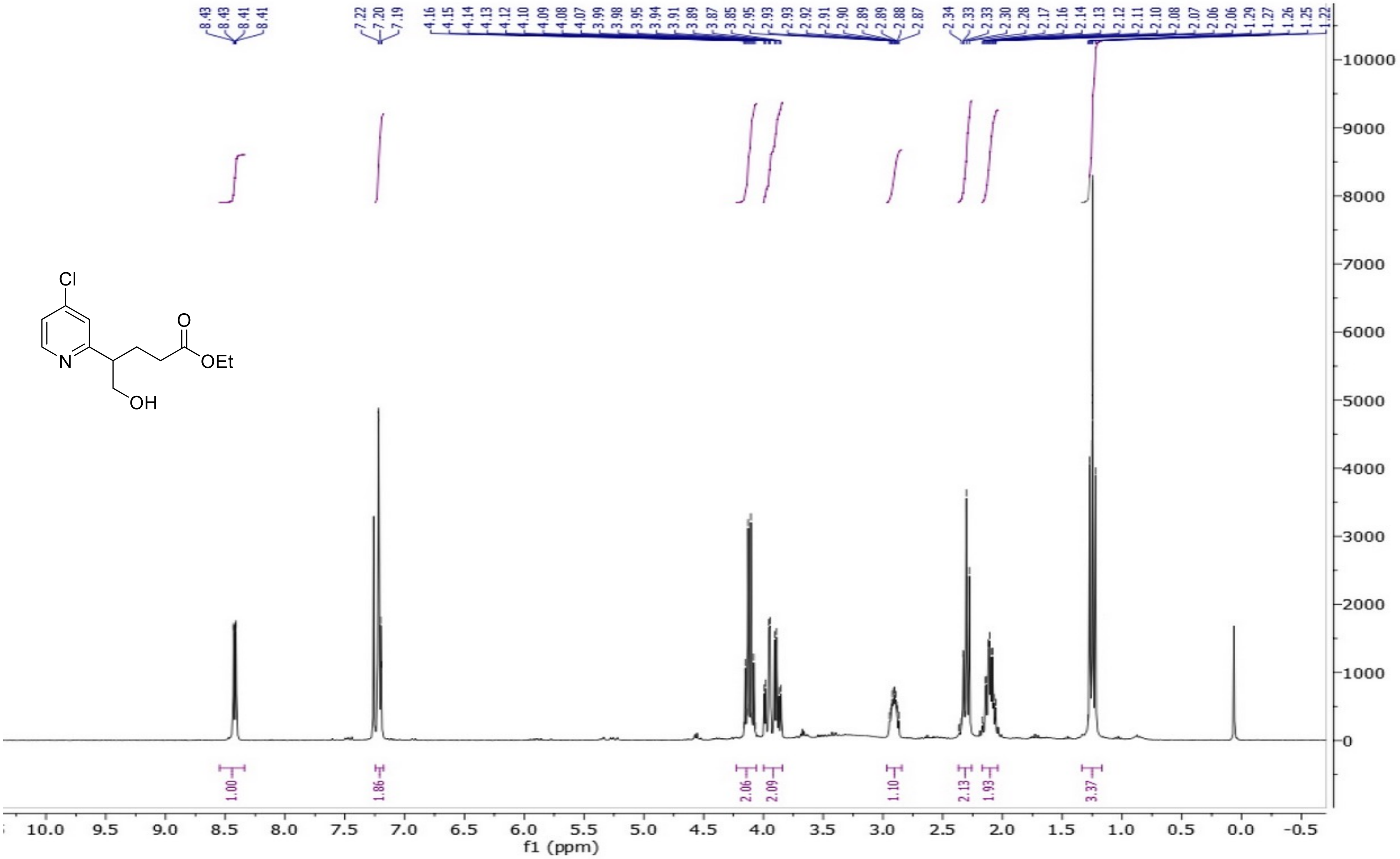

Ethyl 4-(4-chloropyridin-2-yl)-5-hydroxypentanoate (62)

^{13}C-NMR, 75 MHz, $CDCl_3$

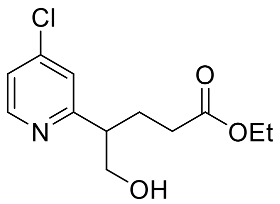

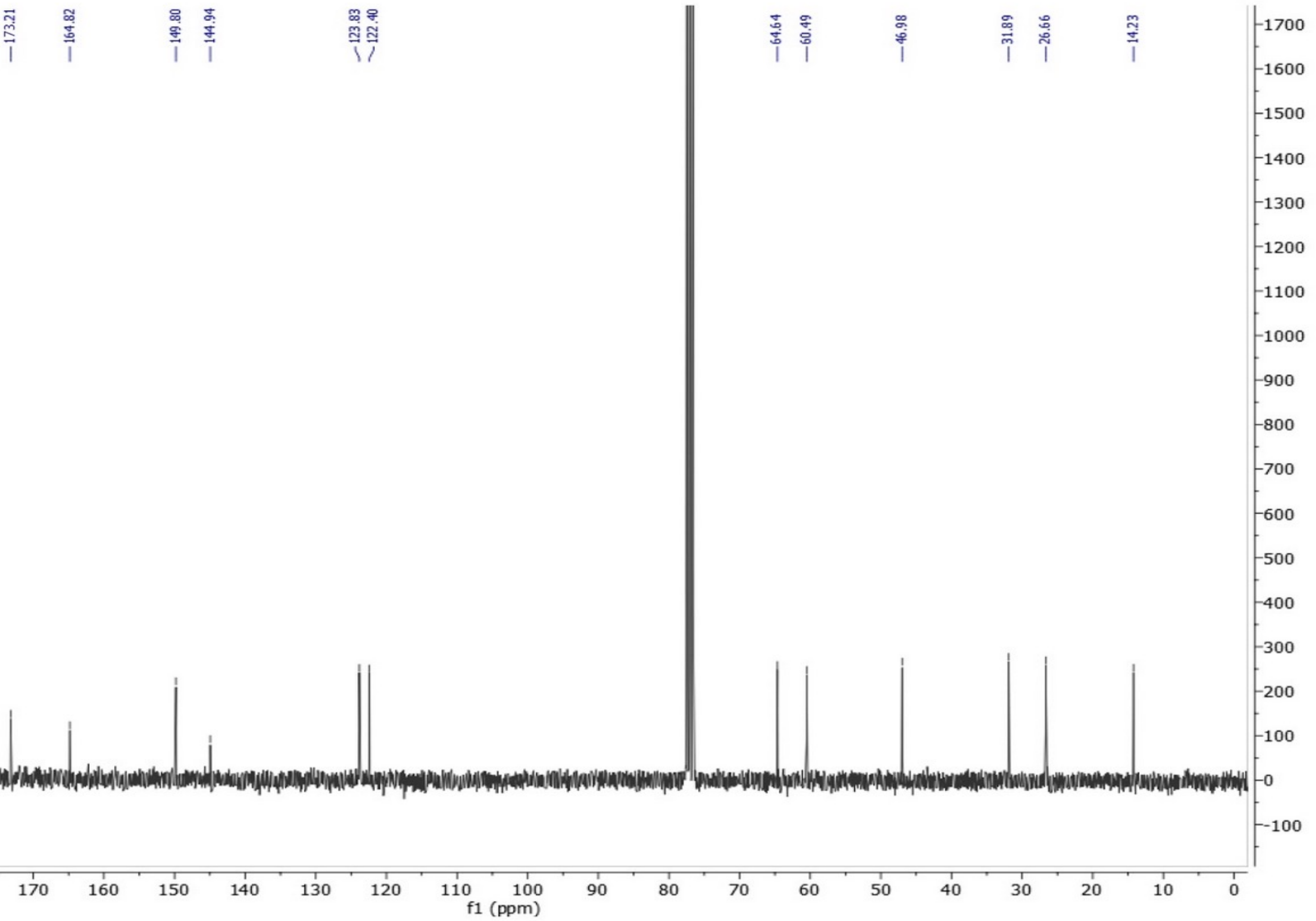

Ethyl 3-(1-(2,6-dichloropyridin-4-yl)-4-phenylcyclohexyl) propanoate (63)

^{1}H-NMR, 300 MHz, $CDCl_3$

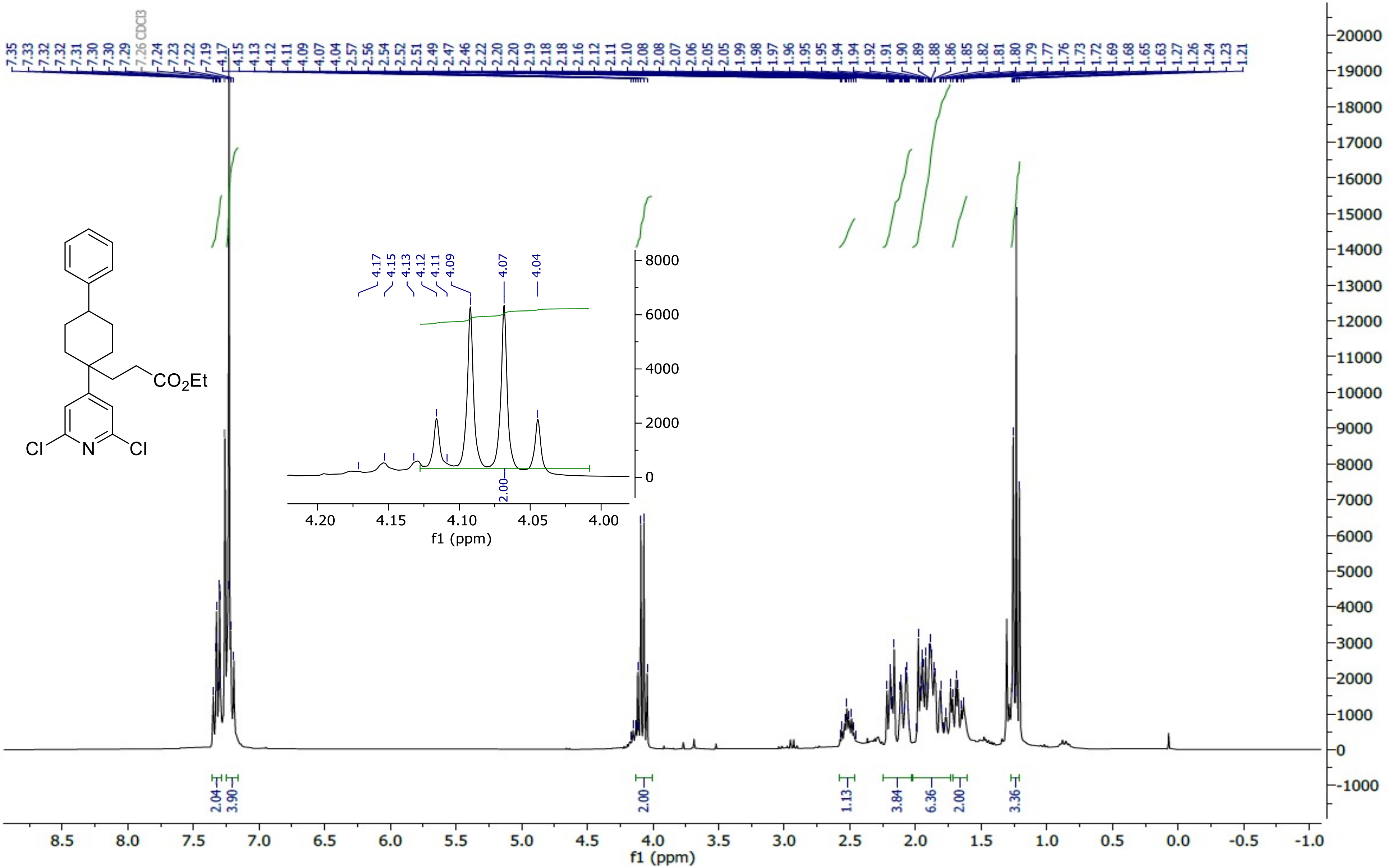

Ethyl 3-(1-(2,6-dichloropyridin-4-yl)-4-phenylcyclohexyl) propanoate (63)

^{13}C-NMR, 75 MHz, $CDCl_3$

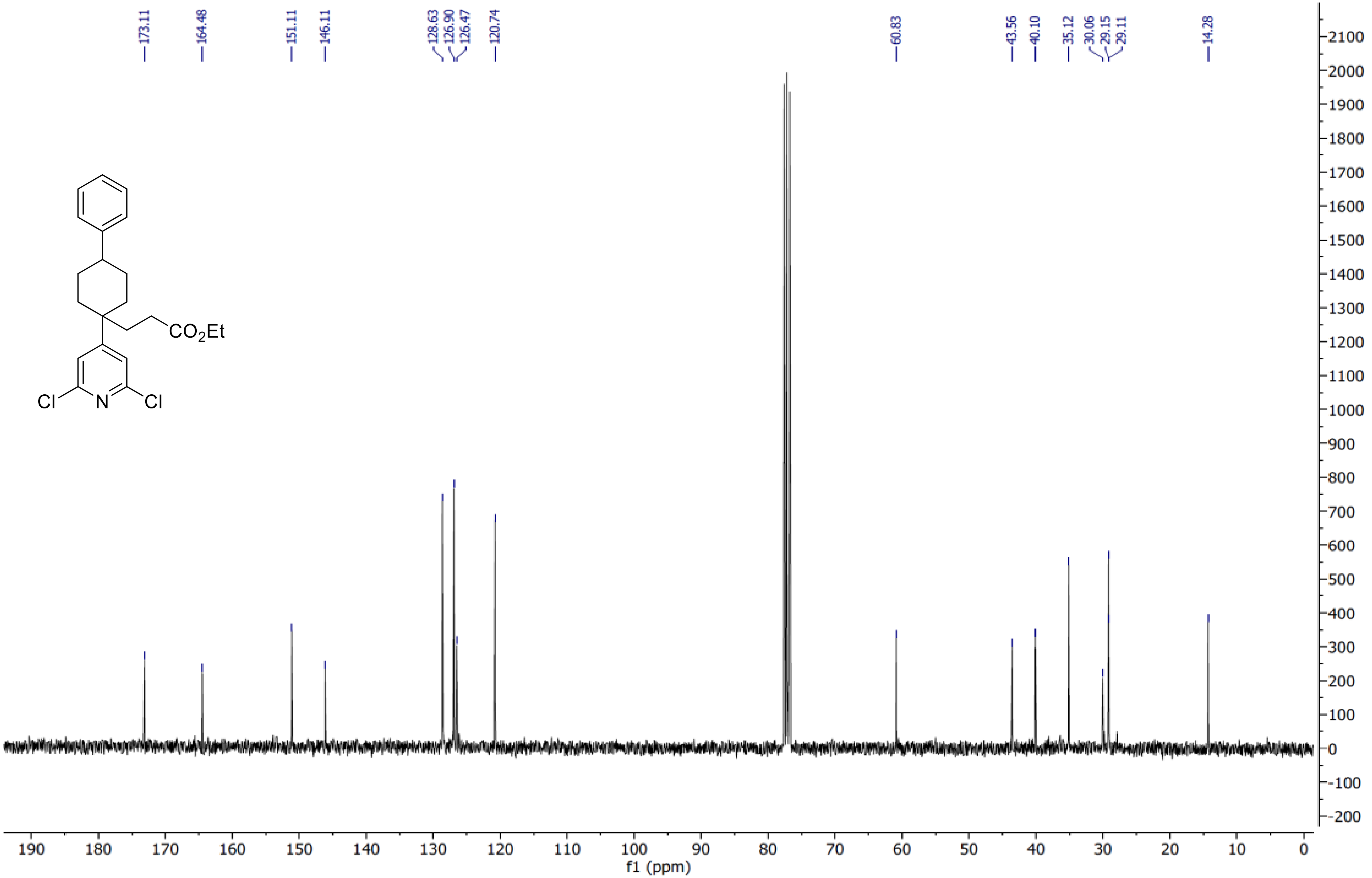

Ethyl 4-(4-chloropyridin-2-yl)-4-(2-oxopyrrolidin-1-yl) butanoate (64)

^{1}H-NMR, 300 MHz, $CDCl_3$

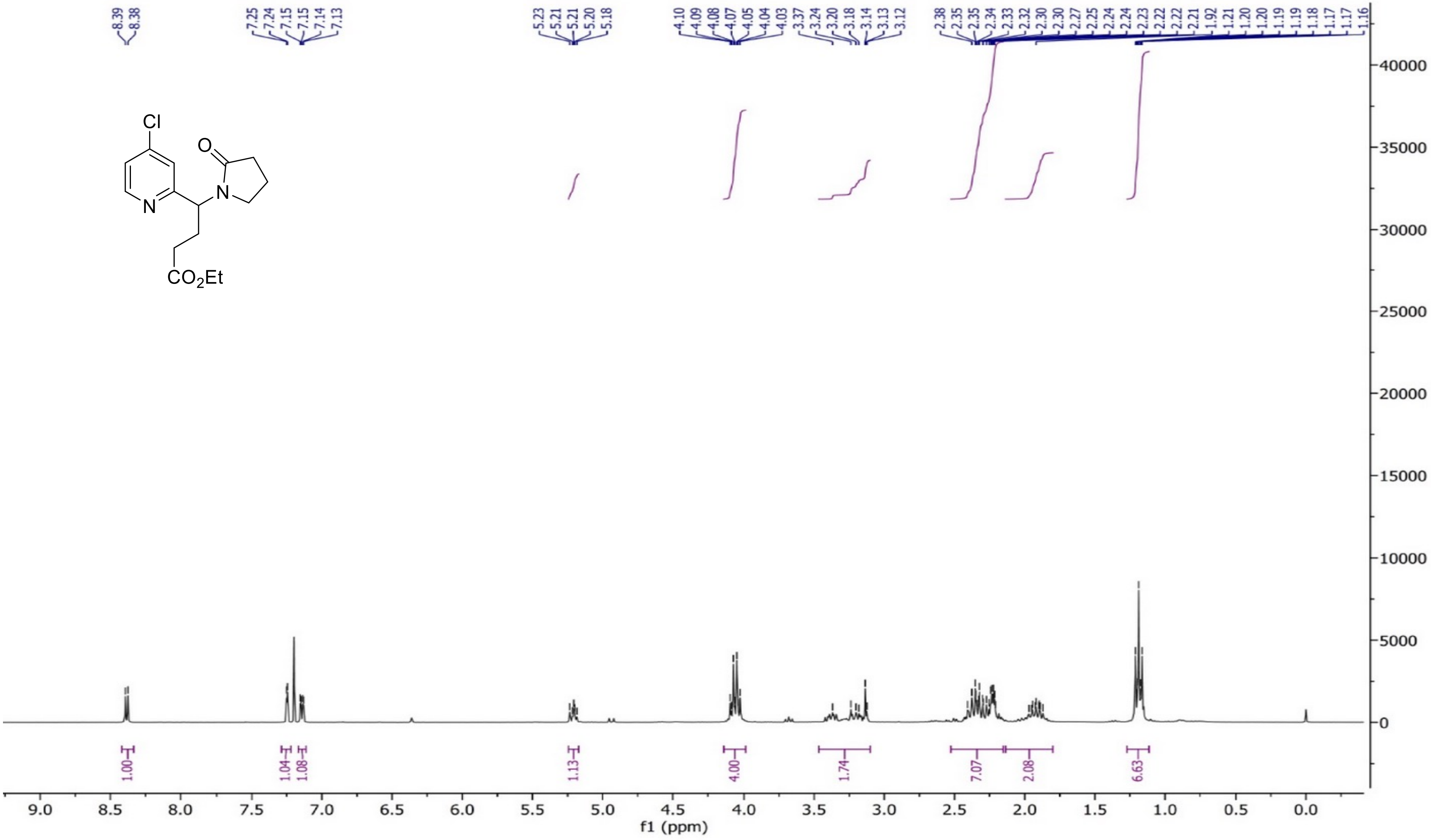

Ethyl 4-(4-chloropyridin-2-yl)-4-(2-oxopyrrolidin-1-yl) butanoate (64)

^{13}C-NMR, 75 MHz, $CDCl_3$

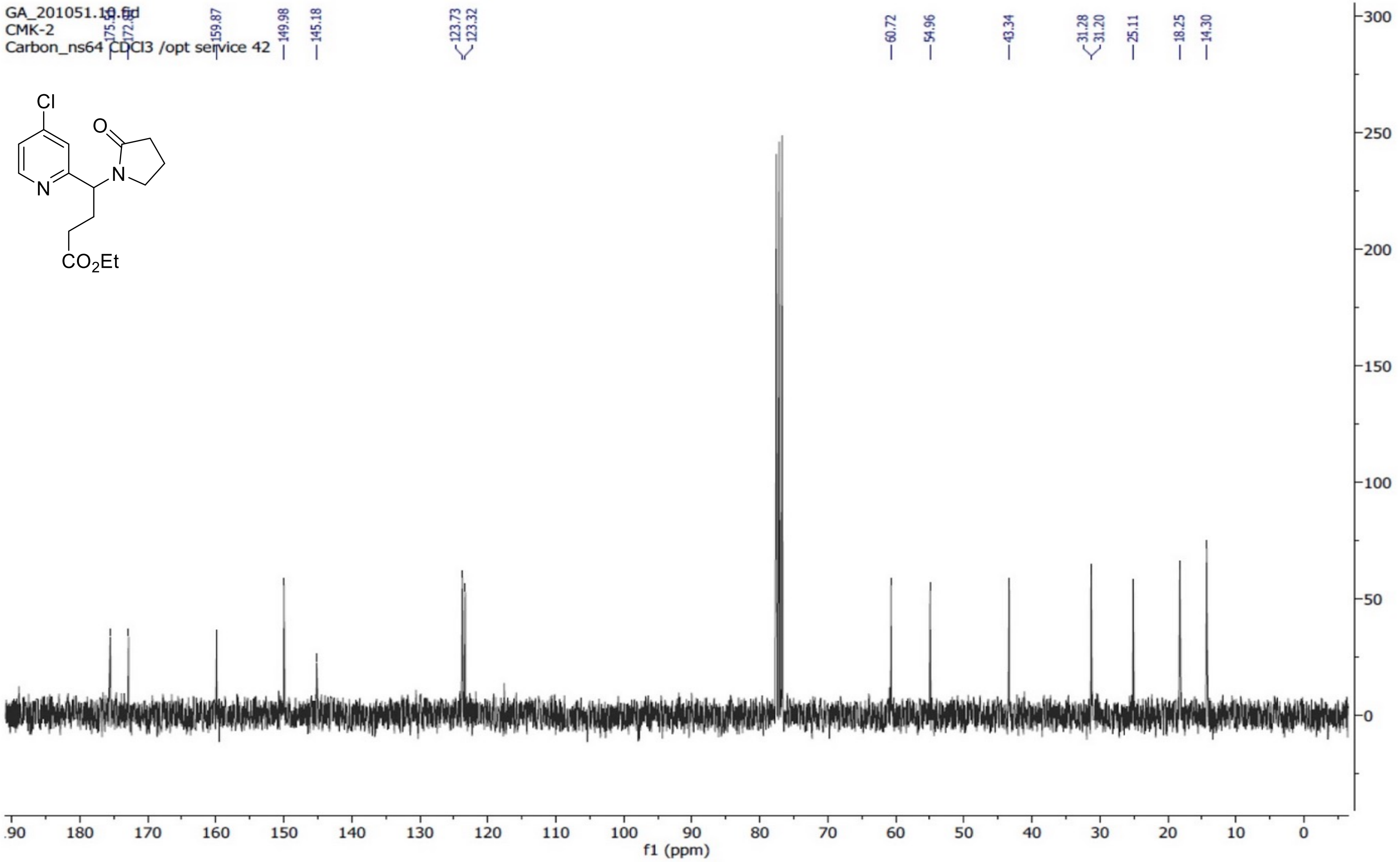

Ethyl 4-acetoxy-4-(4-methylquinolin-2-yl)pentanoate (65)

^{1}H-NMR, 300 MHz, $CDCl_3$

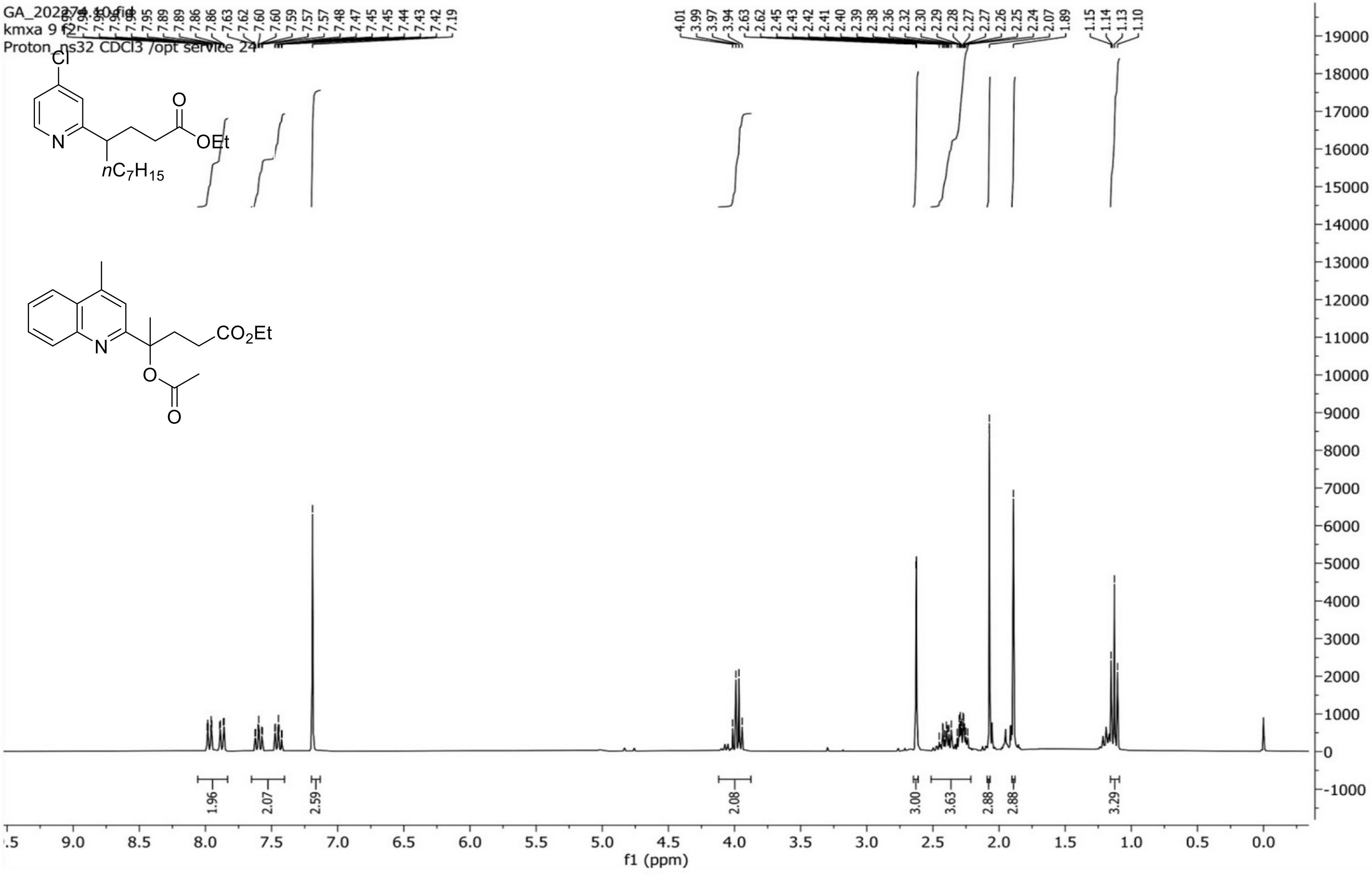

Ethyl 4-acetoxy-4-(4-methylquinolin-2-yl)pentanoate (65)

^{1}H-NMR, 100 MHz, $CDCl_3$

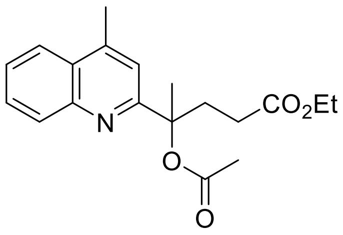

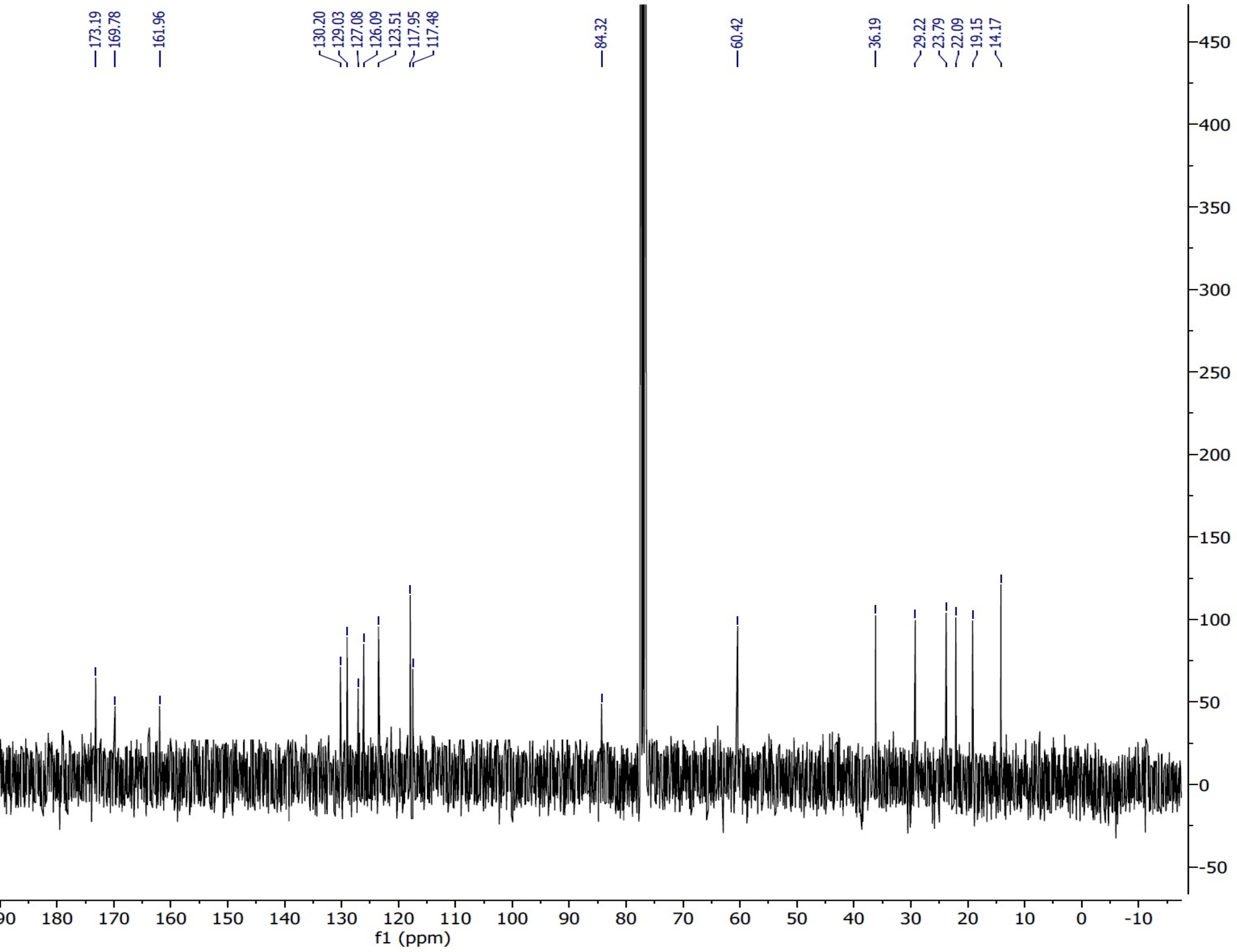

Ethyl 4-acetoxy-4-(4-chloropyridin-2-yl)pentanoate (66)

^{1}H-NMR, 300 MHz, $CDCl_3$

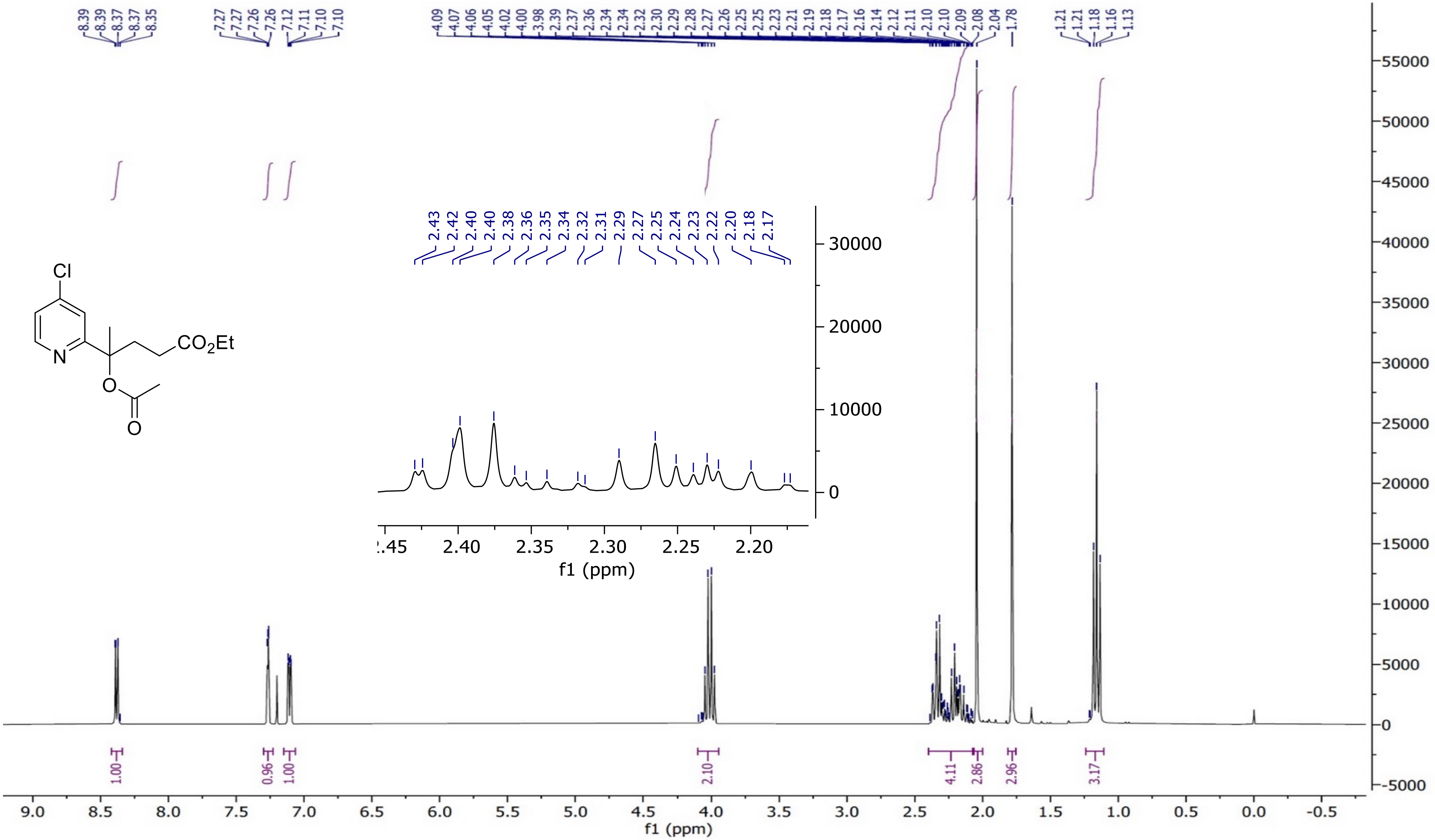

Ethyl 4-acetoxy-4-(4-chloropyridin-2-yl)pentanoate (66)

^{13}C-NMR, 75 MHz, $CDCl_3$

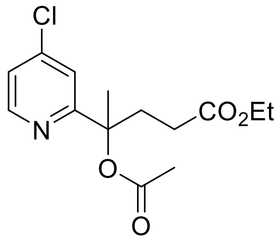

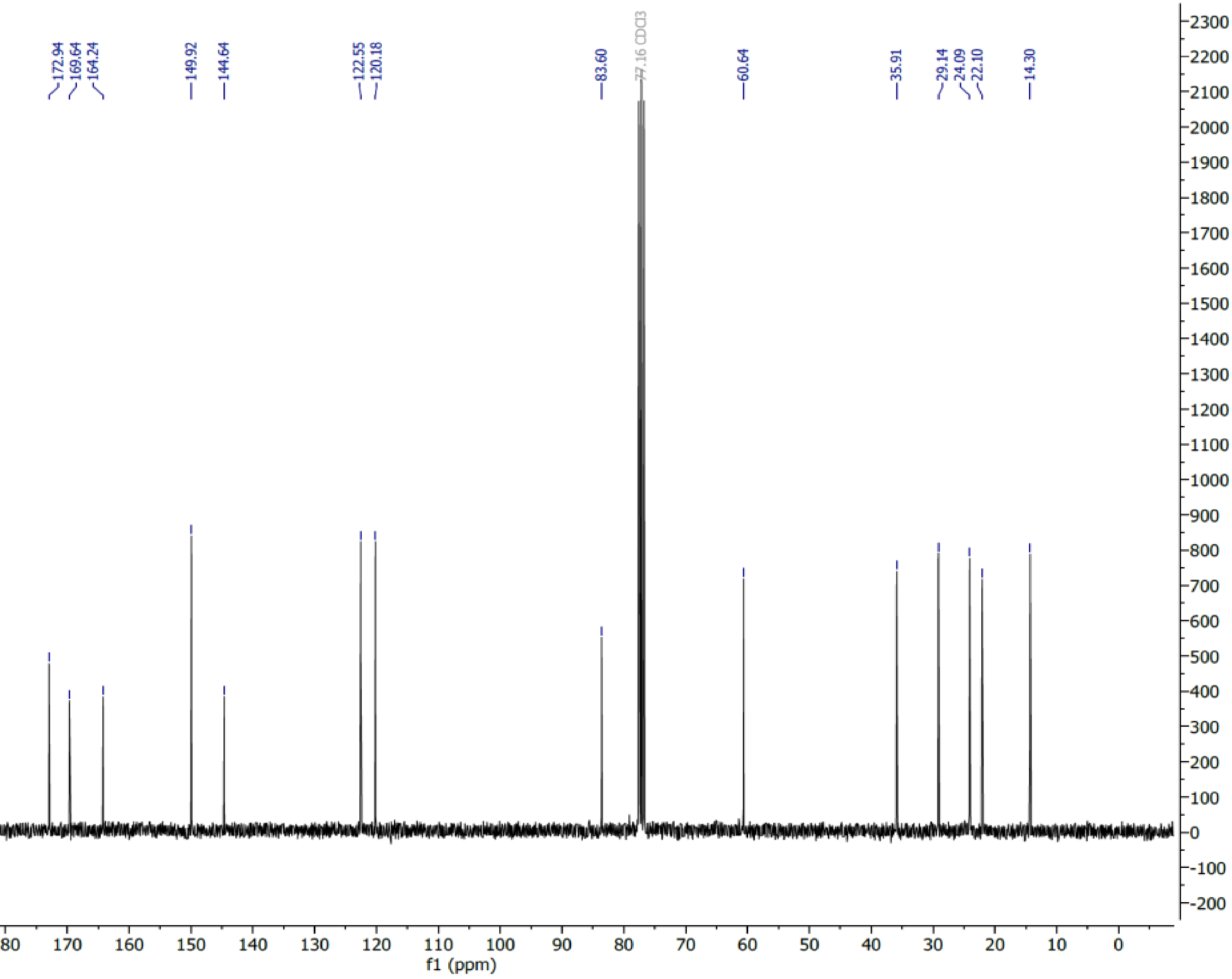

2-Chloro-4-isopropylpyridine (67)

^{1}H-NMR, 300 MHz, $CDCl_3$

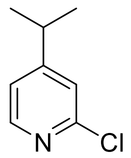

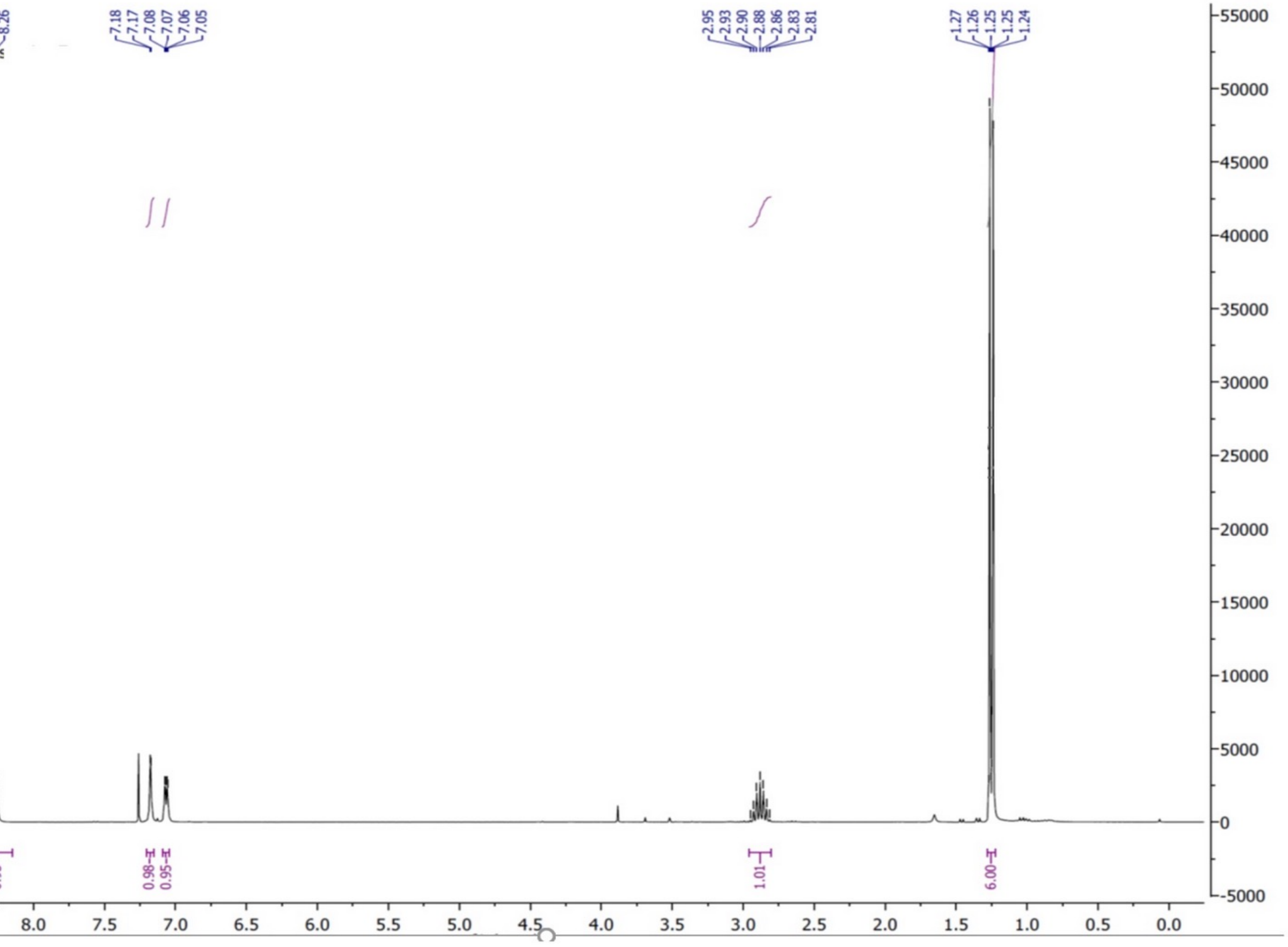

2-Chloro-4-isopropylpyridine (67)

^{13}C-NMR, 75 MHz, $CDCl_3$

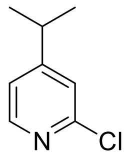

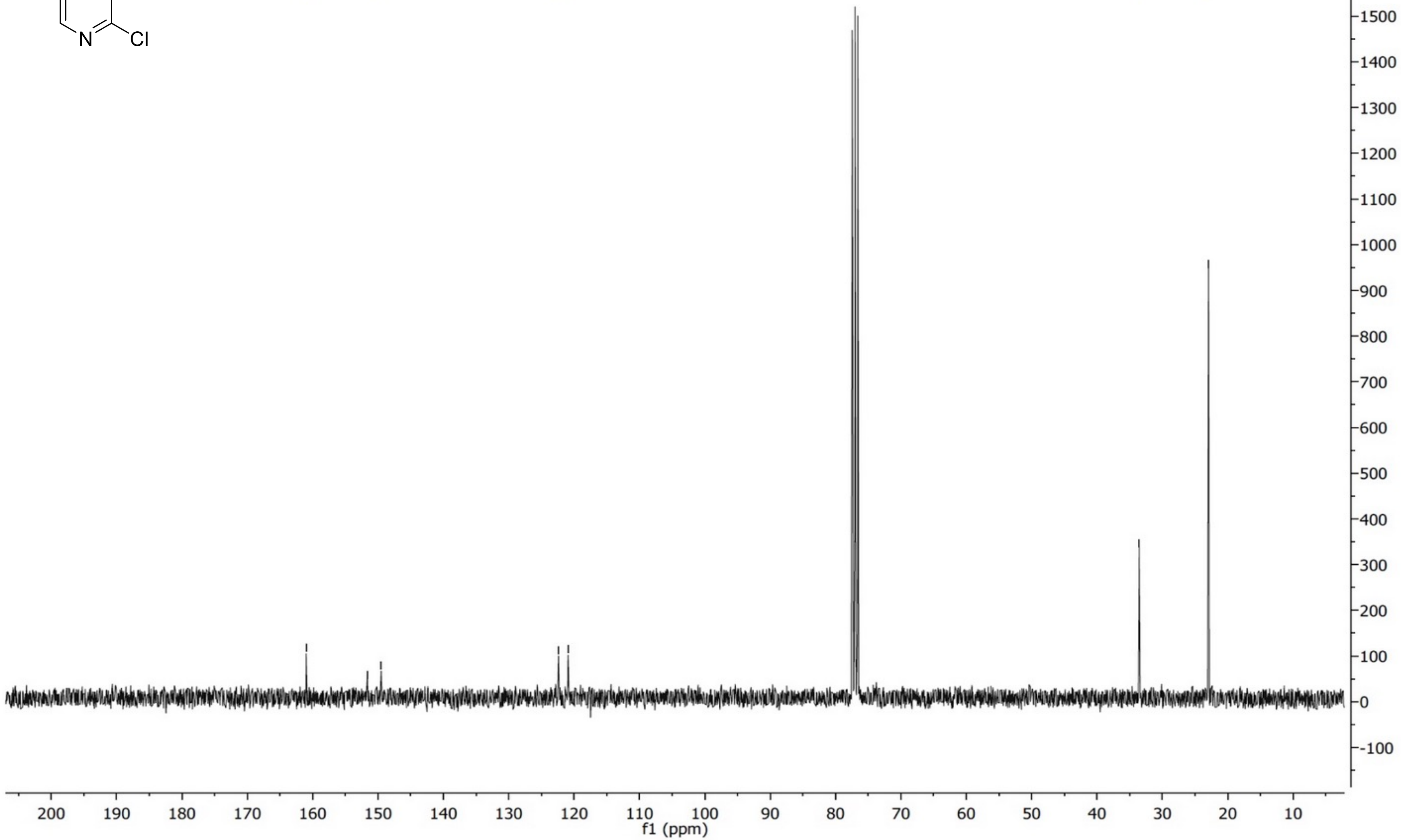

4-(1-Adamantyl)-2-chloro-pyridine (68)

^{1}H-NMR, 300 MHz, $CDCl_3$

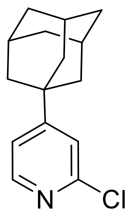

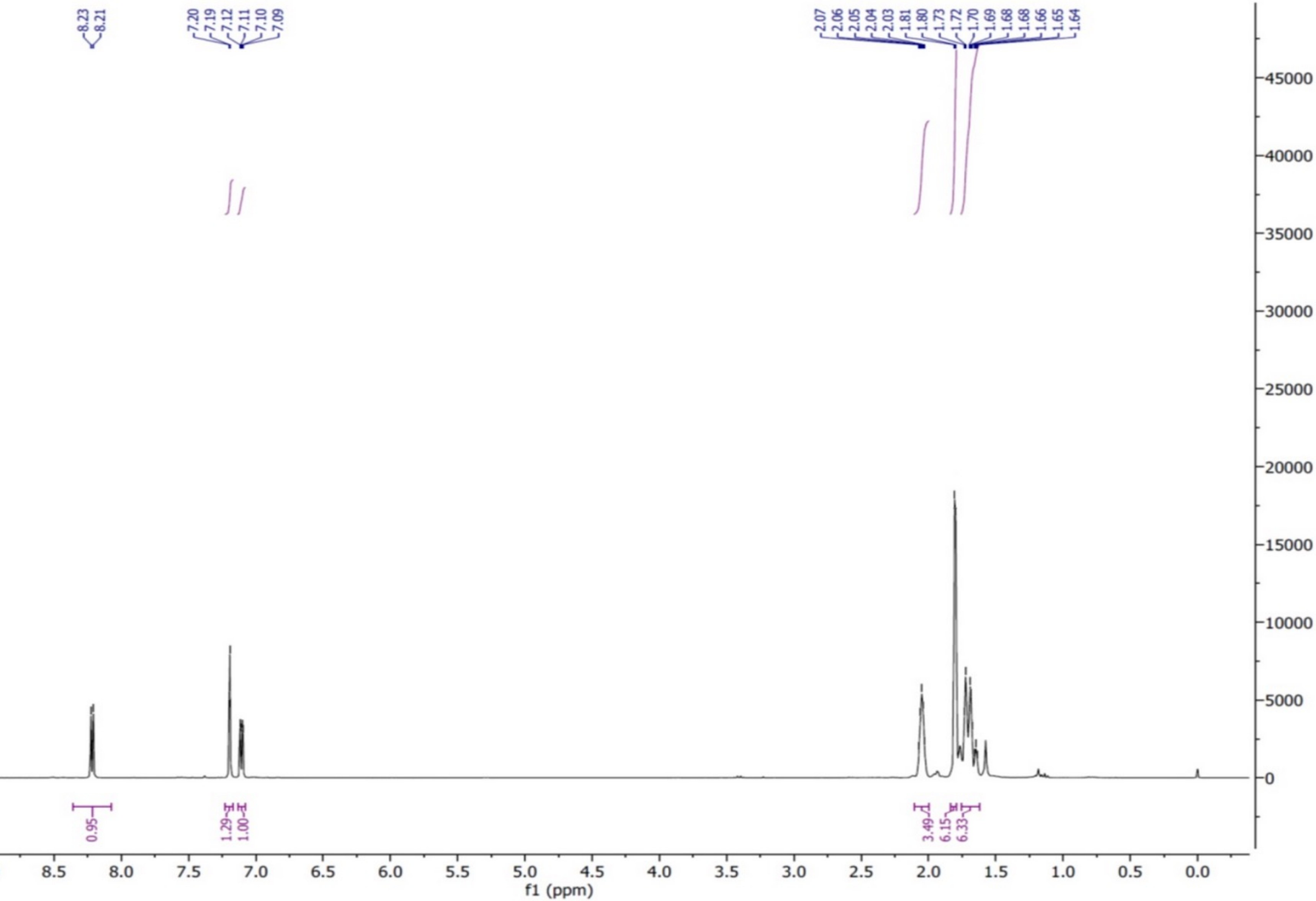

4-(1-Adamantyl)-2-chloro-pyridine (68)

^{13}C-NMR, 75 MHz, $CDCl_3$

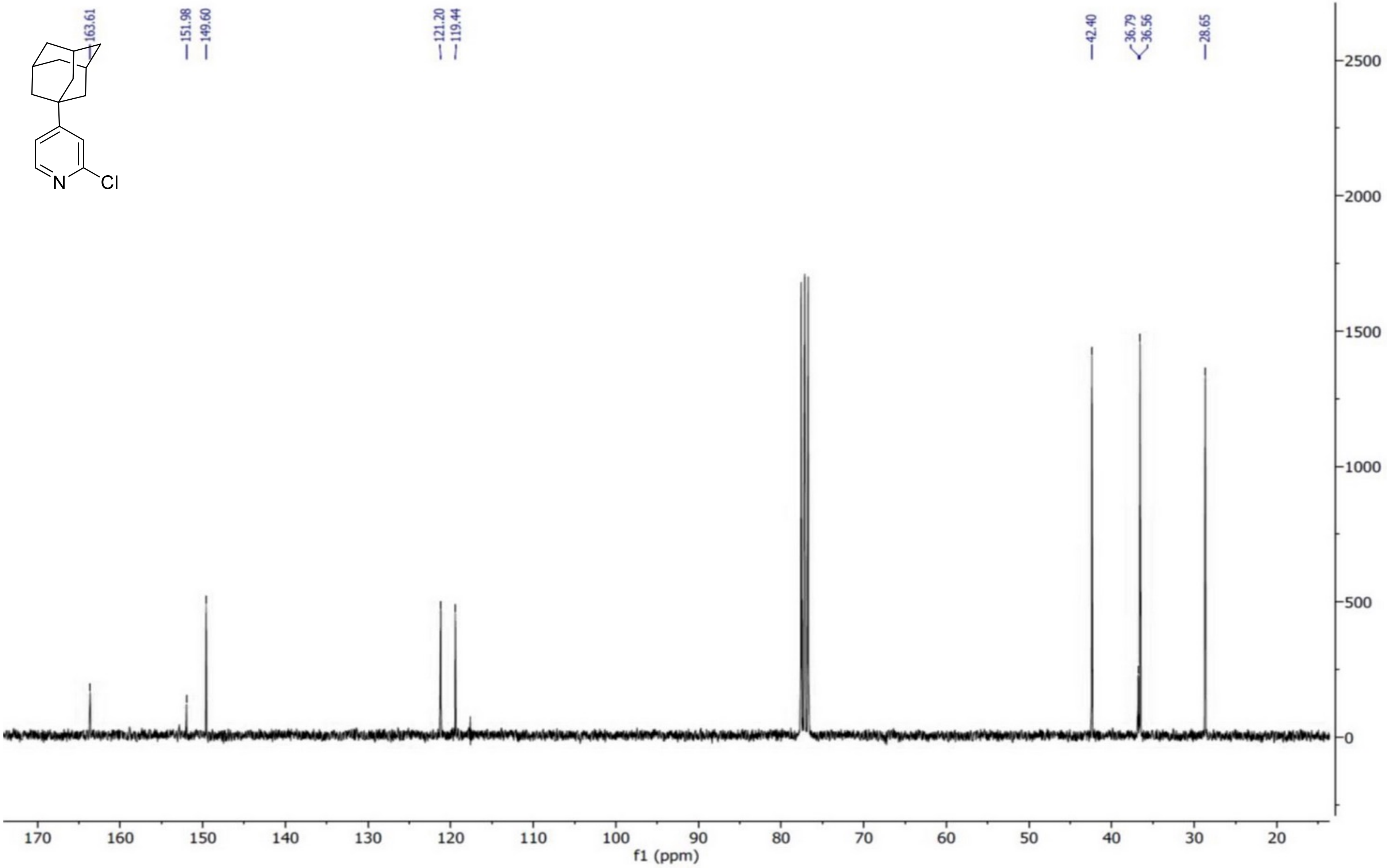

Supplement: SC-012-D1SC02748D-s002 [file SC-012-D1SC02748D-s002.pdf]
